# Development of a Fully Automated, Web-Based, Tailored Intervention Promoting Regular Physical Activity Among Insufficiently Active Adults With Type 2 Diabetes: Integrating the I-Change Model, Self-Determination Theory, and Motivational Interviewing Components

**DOI:** 10.2196/resprot.4099

**Published:** 2015-02-17

**Authors:** Michel Moreau, Marie-Pierre Gagnon, François Boudreau

**Affiliations:** ^1^Interdisciplinary Group of Health Applied ResearchNursing DepartmentUniversité du Québec à Trois-RivièresTrois-Rivières, QCCanada; ^2^Canada Research Chair in Technologies and Practices in HealthFaculty of NursingUniversité LavalQuébec, QCCanada

**Keywords:** physical activity, tailoring, computer-tailoring, type 2 diabetes mellitus, eHealth, World Wide Web, Internet, motivational interviewing, self determination, I-Change Model, behavior modification

## Abstract

**Background:**

Type 2 diabetes is a major challenge for Canadian public health authorities, and regular physical activity is a key factor in the management of this disease. Given that fewer than half of people with type 2 diabetes in Canada are sufficiently active to meet the recommendations, effective programs targeting the adoption of regular physical activity (PA) are in demand for this population. Many researchers argue that Web-based, tailored interventions targeting PA are a promising and effective avenue for sedentary populations like Canadians with type 2 diabetes, but few have described the detailed development of this kind of intervention.

**Objective:**

This paper aims to describe the systematic development of the Web-based, tailored intervention, Diabète en Forme, promoting regular aerobic PA among adult Canadian francophones with type 2 diabetes. This paper can be used as a reference for health professionals interested in developing similar interventions. We also explored the integration of theoretical components derived from the I-Change Model, Self-Determination Theory, and Motivational Interviewing, which is a potential path for enhancing the effectiveness of tailored interventions on PA adoption and maintenance.

**Methods:**

The intervention development was based on the program-planning model for tailored interventions of Kreuter et al. An additional step was added to the model to evaluate the intervention’s usability prior to the implementation phase. An 8-week intervention was developed. The key components of the intervention include a self-monitoring tool for PA behavior, a weekly action planning tool, and eight tailored motivational sessions based on attitude, self-efficacy, intention, type of motivation, PA behavior, and other constructs and techniques. Usability evaluation, a step added to the program-planning model, helped to make several improvements to the intervention prior to the implementation phase.

**Results:**

The intervention development cost was about CDN $59,700 and took approximately 54 full-time weeks. The intervention officially started on September 29, 2014. Out of 2300 potential participants targeted for the tailored intervention, approximately 530 people visited the website, 170 people completed the registration process, and 83 corresponded to the selection criteria and were enrolled in the intervention.

**Conclusions:**

Usability evaluation is an essential step in the development of a Web-based tailored intervention in order to make pre-implementation improvements. The effectiveness and relevance of the theoretical framework used for the intervention will be analyzed following the process and impact evaluation. Implications for future research are discussed.

##  Introduction

Type 2 diabetes is a chronic metabolic disease characterized by poor regulation of blood glucose, which can lead to many health complications. It is by far the most common type of diabetes and the most widespread worldwide [1,2]. In 2009, 2.4 million Canadians were living with type 2 diabetes, and this number is expected to grow to 3.7 million by 2019 [3]. Diabetes is also the leading cause of various health problems in Canadian adults (eg, blindness, end stage renal disease, and non-traumatic amputation) and increases the risk of hospitalization for different causes compared to individuals not having diabetes [3]. Accordingly, the Public Health Agency of Canada urges the development of new population approaches targeting the delay and reduction of complications associated with type 2 diabetes [3].

In addition to pharmacological treatment, lifestyle modifications are a major component of programs aiming to reduce cardiovascular risk factors associated with diabetes, which mainly include the promotion of regular physical activity (PA) and the control of energy intake [4,5]. Scientific evidence increasingly shows the importance of regular PA as a means to reduce the risk of complications associated with type 2 diabetes [5-8]. More specifically, practicing regular PA may help to improve glycemic control [5-7], reduce visceral adiposity [7], lower plasma triglycerides [7], and reduce all-cause mortality risk [8]. Despite this encouraging knowledge, most Canadians with type 2 diabetes do not adequately engage in regular PA in order to obtain the benefits associated with this behavior. In the province of Quebec, only 30% of people with type 2 diabetes have been considered sufficiently active in comparison to the recommendations [9], and this trend is similar elsewhere in Canada [3,10]. Wide-reaching and effective PA promotion programs are therefore needed and recommended [3,4,11].

The use of information technologies like the Internet is seen as a promising avenue for the development of large scale interventions with behavior change purposes [12-16]. Tailoring, the generation of personalized feedback by a computer program based on prior individual assessment(s) [17], accounts for a large part of research using information technologies in the context of health behavior change. This approach has demonstrated its potential to be more effective than more general types of health behavior change interventions, including those targeting PA [12,18,19]. It has also been demonstrated that tailoring can be effective on various communication channels including print-based [20,21], automated phone [22], and Internet, mobile, or offline computer [12,19,22-24]. However, although evidence suggests this has not yet become an established reality across all countries, education levels, and age categories [12,25], Internet and mobile communication channels could have a greater dissemination potential [13,26]. The evolution of Canadian Internet use further suggests that public health organizations should take advantage of this technology to develop and deliver their services [27-29], including services targeting populations with chronic diseases [30].

As mentioned in previous literature, scientific reporting regarding how health behavior change interventions work is currently lacking when compared to publications examining the effectiveness of these interventions, as incomplete information is provided on their theoretical basis, behavior change techniques employed, and other elements forming the core components of interventions [21,31,32]. This claim has also been made specifically in the tailoring field where inconsistencies remain when reporting information related to intervention content and development [33].

Using recent reporting standards in the fields of health behavior change and tailoring [33,34], this paper will therefore try to build on previous research by demonstrating the in-depth development of the Diabète en Forme (DEF) Web-based, tailored intervention targeting adoption and maintenance of regular aerobic PA among Canadian francophone adults with type 2 diabetes. Given that Short et al recently published a paper on the development of an evidence-based, tailored print intervention using essentially the same standards and program-planning model as us [35], our paper will add to their work by repeating the procedure for a fully automated, Web-based, tailored intervention.

We will also explore the use of concepts derived from Self-Determination Theory (SDT), a theory focusing on types rather than just amount of motivation [36], and Motivational Interviewing (MI), a counseling approach using a collaborative style of communication for strengthening a person’s own motivation and commitment to change [37], as elements of the intervention core components [38-43]. The integration and applications of SDT and MI have been extensively discussed and encouraged in the PA domain in recent years [44-48]. Research on PA adoption and maintenance increasingly shows the added value of autonomous motivation, an SDT and MI-related construct, to help individuals adopt and, more importantly, maintain regular PA [49-51]. The use of SDT and MI becomes even more relevant in the context of this study knowing that it remains unclear whether actual tailored and online interventions targeting PA behavior can promote long-lasting change on their target populations [18,52]. Therefore we will describe more details about SDT, MI, and components of the DEF tailored intervention derived from them.

## Methods

### Overview

An adaptation of the program-planning model developed by Kreuter et al [53] was conceptualized to adjust the model to a Web-based intervention. Kreuter’s model was initially designed for print-based communication and includes the following nine steps: (1) analyzing the health problem, (2) developing a program framework, (3) developing the tailoring assessments, (4) designing feedback, (5) writing tailored messages, (6) creating tailored algorithms, (7) automating the tailoring process, (8) implementing the program, and (9) evaluating the program.

We felt it necessary to adapt the model to the needs of our Web-based intervention because it appeared more logical for our team to follow the original steps in a different order (see Table 1). First, we created the algorithms in Step 4 because we deemed it more logical to decide on which circumstances participants should receive which messages prior to any message writing activities. Making this step prior to writing the message booklet allowed us to have a concrete overview of how many messages should be written, which message participants should receive, and under which circumstances they should receive it. By creating the algorithms during Step 4, we were also able to assign a coding variable to each message, which later facilitated the automation of the tailoring process (Step 7). Finally, the original Step 4, designing feedbacks, became Step 6 because our team agreed on the difficulty of envisioning the appearance of the feedbacks without knowing what their content would be. Since messages are at the core of the intervention, it once again seemed more intuitive for our team to write the messages before designing their appearance to adapt the design of the Web platform to the messages’ length and style.

In addition to changing the order of the steps, we divided Step 9 (Kreuter’s), evaluation of the program, into two different steps: (1) usability evaluation, which comes prior to implementing the intervention, and (2) process, impact, and outcome evaluation, which is subsequent to the implementation of the intervention. Usability evaluation, a new step added to the model, will be described and discussed later in this paper. The adapted model is presented in Table 1.

**Table 1:** Program-planning model for the DEF Web-based tailored intervention.

Steps	Step description
1. Analyzing the health problem	Learn as much as possible about the population and the health outcome.
2. Developing a program framework	An outline is created to describe all parts of the Web-based tailored intervention.
3. Developing tailoring assessment	Assessment questionnaires that will be used to collect information are created.
4. Creating tailored algorithms (order changed)	Logic statements and decision rules are created. These specify which messages should be given to which participants under which circumstances.
5. Writing tailored messages	All intervention messages are created based on logic statements and decision rules specified in Step 4.
6. Designing feedback (order changed)	Decisions are made about how the tailored messages will look and how they will be presented in the intervention. This step includes designing all templates for the DEF website.
7. Automating the tailoring process and creating the website	The algorithms and messages are translated into a computer program that automatically allows participants to receive appropriate feedback in regard to their individual profile. The tailored intervention website is created.
8. Usability evaluation (step added)	The intervention is tested with a small group of participants and final improvements are made to the intervention prior to its implementation.
9. Implementing the program	The intervention is put in use and becomes available to the official and larger group of participants recruited for the DEF intervention.
10. Evaluating the process, impact, and outcome	The intervention is analyzed and evaluated on these three aspects.

### Step 1: Analyzing the Health Problem

#### Overview

According to Kreuter et al, three main strategies can serve to gain knowledge and understanding of aerobic PA behavior within our target population: (1) reviewing applicable theories and models, (2) reviewing previous research, and (3) collecting original data. The first strategy aims to identify which model or theory explaining health-related behavior would best suit our main objective: the adoption and maintenance of regular aerobic PA among Canadian francophone adults with type 2 diabetes. The second strategy serves to gain additional knowledge by reviewing the work others have done addressing the same topic or health problem, which can help refine the model to be used to build the intervention and gain extra knowledge on other related topics. Finally, in combination with the first two strategies, collecting original data will help fill the remaining knowledge gap to have an optimal overview of the health problem.

#### Reviewing Applicable Theories and Models

A wide range of theories and models have been used in behavior change interventions. Among them, Social Cognitive Theory [54], Transtheoretical Model [55], Theory of Planned Behavior [56], and Health Belief Model [57] are the most widely used theories and models in tailored interventions targeting PA uptake and maintenance [12,18,19,21,22]. Another interesting model used in this field is the I-Change Model [58], which can be interpreted as a coherent integration of the ideas of the theories and models mentioned above, combined with other goal-setting theories [59]. In recent years, the use of the I-Change Model in tailored interventions has resulted in long-term positive impacts on PA behavior among adults of various age groups [60,61].

Although it is well accepted that tailoring is an effective population approach to the promotion of regular PA, it has been mentioned that future research should focus on establishing larger effect sizes and sustained effects on PA behavior [18]. In that sense, SDT, which accounts for a significant body of research on PA behavior motivation (ie, over a hundred papers) [51], may represent a great potential theory for enhancing the performance of more standard tailored interventions [23,51,62]. SDT can be defined as a comprehensive and evolving macro-theory of human personality and motivated behavior, fundamentally centered on the fulfillment of needs (ie, competence, autonomy, and relatedness) that leads to self-actualization and the realization of one’s potential [63]. One key component of this theory is the distinction between autonomous forms of motivation (ie, identified, integrated, and intrinsic motivation) and controlled forms of motivation (ie, amotivation, extrinsic, and introjected motivation). Within SDT, PA behavior is autonomously motivated when it is practiced for personal value and utility (eg, personally wanting to maintain good health or being a role model). On the other hand, PA behavior is motivated in a controlled way when it is practiced for external reasons such as avoiding punishment contingencies or seeking external rewards (eg, social approval/disapproval, material gains/losses, avoiding feelings of guilt). As controlled forms of motivation are expected to sometimes motivate short-term PA behavior but not to sustain its maintenance [51], autonomous motivation is more strongly correlated to PA behavior adoption and maintenance over a long period of time [51,64]. Therefore, the main reason why SDT can add value to standard tailoring is because it can provide the theoretical background needed to help individuals develop a more autonomous motivation toward regular PA.

In order to develop a more autonomous motivation toward PA in an individual, SDT also explains that the social environment surrounding the individual in a PA context must support three basic psychological needs: (1) autonomy, (2) competence, and (3) relatedness (see Table 2 for a definition of these needs). But although SDT can provide a few guidelines in the creation of such an environment, this theory has received some criticism regarding its practical use for doing so when compared to MI [46-48,65]. More precisely, SDT represents a coherent framework for understanding the process underpinning the development of autonomous motivation, but it does not provide a concrete and evidence-based approach that can be used by health professionals to create SDT-based interventions. And it is mainly for this reason that MI can complement SDT [47,48], while conversely, this model provides a great practical framework based on more than 20 years of research that could provide an SDT needs-supportive environment necessary for autonomous motivation development [37,47,48]. MI, a counseling approach using a collaborative conversation style for strengthening a person’s own motivation and commitment to change [37], is composed of concepts such as spirit, processes, strategies, and interviewing skills that can facilitate the development of autonomous motivation in individuals toward the adoption of regular PA [37]. Broadly speaking, within MI, all concepts previously mentioned serve to elicit change-talk, which is statements by the client revealing consideration, motivation, or commitment to change, responsible for the development of autonomous motivation [66]. An in-depth description of MI concepts within a tailoring context are presented in the work of Friederichs et al [39,40]. Concepts derived from SDT and MI used for our intervention are presented in Table 2.

**Table 2:** Overview of MI-SDT concept application to the DEF intervention: SDT needs and related need-supportive strategies.

Strategies			Integration to the DEF intervention
**Autonomy: being the perceived origin or source of one’s own behavior [67]**			
	**MI-related strategies**		
		Let the client make decisions about what and how to change [47]	The weekly action plan tool to which participants have access allows them to select their objectives and activities by themselves. The program explicitly recognizes that the participant is the best person to make decisions about what and how to change.
		Roll with resistance [47]	The program has specific feedback for participants with low scores on intention, self-efficacy, and attitude. Reflections of content are provided when participants adopt resistant behavior (eg, do not elaborate on their beliefs, choose to do no PA for a certain week).
		Explore options [47]	The program asks participants open and multiple choice questions during motivational sessions. [39]
		Encourage Change-Talk [47]	Motivational sessions use MI interviewing skills (OARS; [37]) and other techniques oriented in favor of change.
	**SDT-related strategies**		
		Provide a menu of effective options for change [48]	The action planning tool proposed preferred physical activities and solutions to common barriers of people with type 2 diabetes.
		Provide a rationale for information given [48]	The rationale of each motivational session and tool is explained either through a video or short sentence on the first page of each intervention component.
		Supporting patients’ choices and initiatives [48]	Positive feedback is provided to participants when they make even the smallest commitment toward change. The program does not judge participants who provide answers that are not in keeping with change.
**Competence: feeling effective in one’s ongoing interactions with the social environment and experiencing opportunities to exercise and express one’s capacities [63]**			
	**MI-related strategies**		
		Present clear and neutral information about behavior and outcomes [47]	Participants can receive information about the risks associated with physical inactivity and the benefits of regular physical activity during the first two motivational sessions. Participants are told explicitly that they are the only experts about what and how to change.
		Help the client develop appropriate goals [47]	The action plan tool encourages participants to set realistic weekly behavioral goals for themselves.
		Provide positive feedback [47]	Participants are frequently valorised for their participation in the motivational sessions and efforts toward change. Participants are also valorised for their strengths and values in motivational sessions 3 and 5.
		Support self-efficacy [47]	The program affirms the strengths of participants and unconditionally recognizes their capacity and ability to change.
	**SDT-related strategies**		
		Help skills building and problem solving [48]	The program gives participants information and tools on how to calculate and self-monitor their PA level. It also helps participant identify effective solutions to their barriers and provides information on how to practice PA safely.
**Relatedness: feeling connected to others, to caring for and being cared for by those others, to having sense of belongingness both with other individuals and with one’s community [68]**			
	**MI-related strategies**		
		Express empathy [47]	Messages are written in a way that shows participants that their opinions matter.
		Explore client’s concern [47]	The first motivational session explores worries participants may have toward their insufficient PA level in an empathic way. A tab is dedicated to participants who want to share their opinion on the website or express concerns about it.
		Demonstrate understanding of the client’s position [47]	The reflections and summary are used throughout the motivational sessions to try to understand what participants think or feel.
		Avoid judgment or blame [47]	Messages to participants are written in a neutral or rewarding style.
	**SDT-related strategies**		
		Provide unconditional positive regard [48]	Messages to participants are never judgmental. The program unconditionally recognizes that participants are able to change their PA behavior.
		Provide a consistently warm interpersonal environment [48]	The introduction videos at the start of each motivational session use an enthusiastic but calm tone. We have tried to make the website and tools inviting and warm for participants.

#### Reviewing Previous Research

##### Overview

In the context of this intervention, four distinct research fields were reviewed to complement the knowledge acquired with the first research strategy: (1) determinants of aerobic PA adoption and maintenance among people with type 2 diabetes, (2) factors contributing to the effectiveness of tailored interventions targeting PA behavior, (3) factors contributing to the effectiveness of Web-based interventions targeting PA behavior, and (4) effective behavior change techniques used in interventions targeting PA behavior.

For each of the fields, our team searched for articles published before December 29, 2013, in PsycINFO, Medline (PubMed), and Embase databases. Search strings used for each field are described in Table 3.

**Table 3:** Research strings used for reviewing articles of all four research fields related to the DEF intervention.

Topic	Search strings
Determinants of PA adoption and maintenance among people with type 2 diabetes	Determinants: (correlate OR determinant OR mediator OR moderator OR predictor)
	Physical activity: (“physical activity” OR exercise)
	Type 2 diabetes: diabetes AND (“type 2” OR “type II”)
Factors contributing to the effectiveness of tailored interventions targeting PA behaviora	Tailoring: tailor* AND (feedback* OR intervention* OR individualized OR program*)
	Physical activity: (“physical activity” OR exercise)
Factors contributing to the effectiveness of Web-based interventions targeting PAa	Internet: (Internet OR web OR “e-health” OR eHealth OR online) AND (intervention* OR individualized OR program OR platform OR service)
	Physical activity: (“physical activity” OR exercise)
Effective behavior change techniques used in interventions targeting PA behaviora	Behavior change techniques: (“behaviour change” OR “behavior change”) AND techni*
	Physical activity: (“physical activity” OR exercise)

^a^Only systematic reviews and meta-analysis papers have been selected for those topics.

##### 1. Determinants of Physical Activity Adoption in People With Type 2 Diabetes

Nine articles identifying potential determinants of aerobic PA in adults with type 2 diabetes were found in our qualitative review [9,69-76]. The determinants of aerobic PA in this population identified were (1) self-efficacy, (2) moral norm, (3) attitude, (4) barriers, and (5) intention. Other correlates found were gender, perceived disability, past PA behavior, and various sociodemographic factors, but we did not include these in the targeted constructs because of their hardly modifiable nature in the context of the intervention. Moral norm was also excluded from the intervention. However, the intervention will include values exploration exercises that may have a positive impact on this construct. Furthermore, we identified other constructs related to the I-Change Model (ie, awareness, knowledge, action planning, and social influence) and SDT (ie, autonomous motivation) used in an effective intervention targeting PA in older adults [60].

**Figure 1:**
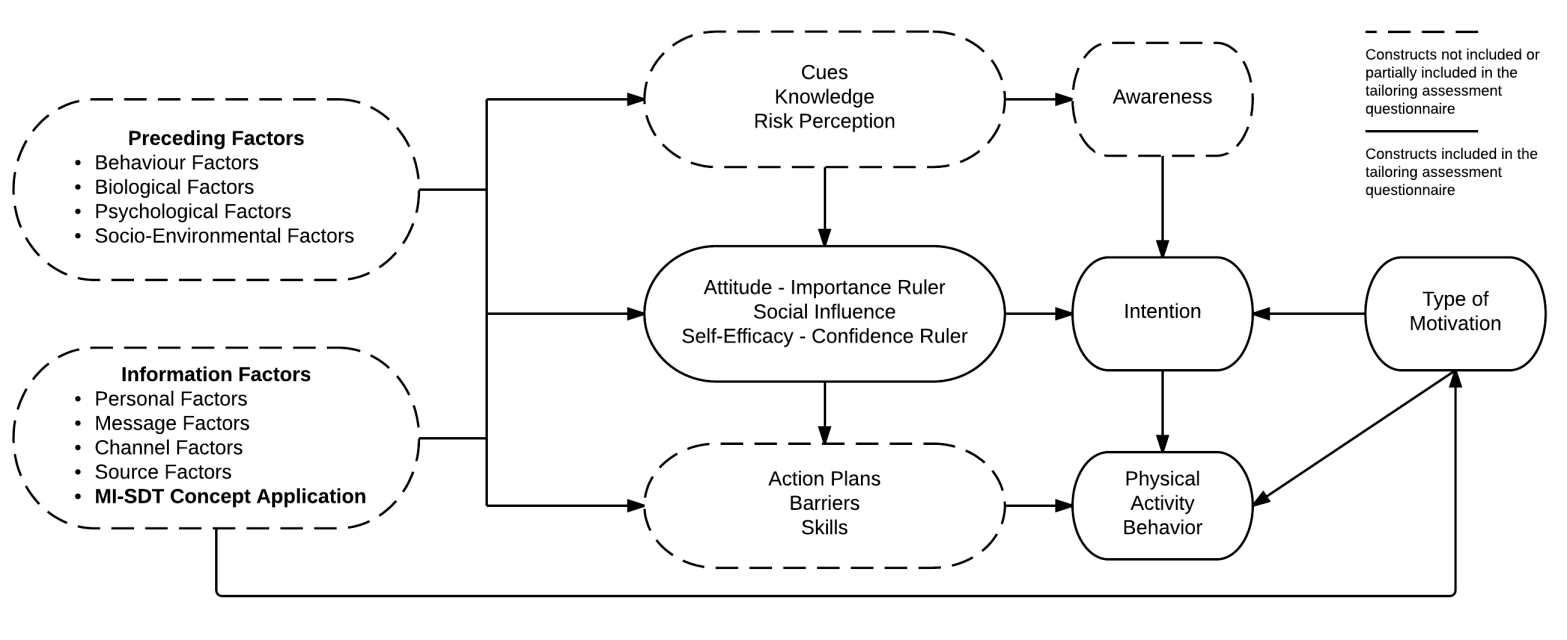
Theoretical framework of the DEF tailored intervention.

At this stage, we believed we had all the necessary information to choose the intervention model. That is, in order to take into account all the knowledge gained from our research, our team decided to develop its own model adapted from MI, SDT, and the I-Change model, on which the DEF tailored intervention would be based (see Figure 1). There are several arguments for how we decided to develop our model: (1) the model integrates more possible sociocognitive determinants related to PA behavior among Canadian adults with type 2 diabetes than any other single model [58], (2) the I-Change Model integrates in a coherent manner a majority of ideas from the most popular theories and models used for tailoring [58,77], (3) the I-Change Model is associated with tailored interventions inducing long-term effects on PA behavior in older adults [60,61], (4) the integration of SDT and MI concepts to the I-Change Model could help induce PA maintenance among participants through autonomous motivation [47,51,64], (5) there is variability in Canadian adults with type 2 diabetes scores on constructs related to the I-Change Model [9], and (6) the theoretical framework chosen offers many possibilities in terms of behavior change techniques and principles on which we can develop our intervention [34,37,47,48].

Like the I-Change Model original framework, our model assumes there are three phases in the PA behavior change process: (1) to increase a person’s awareness about the importance of making a PA behavior change, (2) to motivate the person in favor of the adoption of regular PA, and (3) to transfer this motivation toward the adoption of regular PA [77]. As demonstrated in Figure 1, we also kept all of the I-Change Model constructs without using all of them for the tailored content of the intervention. However, constructs that did not serve for the tailored content were used in a more general fashion in the intervention (see Table 4).

New constructs added to the I-Change Model are (1) Type of Motivation (SDT related), (2) Importance ruler (MI related), (3) Confidence ruler (MI related), and (4) MI-SDT concept application. First, Type of Motivation will help to identify which type of motivation (autonomous vs controlled) regulates the participants’ practice of PA, to give evaluative feedback on participants’ type of motivation, and to analyze whether the intervention has succeeded at developing autonomous motivation toward PA in participants. Second, Importance ruler and Confidence ruler, which are constructs closely related to Attitude and Self-efficacy [37], were used mainly to provide feedback to participants in an MI-style of communication (further details are provided in Step 3). Last, the remaining added construct, MI-SDT concept application, aimed to provide messages to participants within a needs-supportive social environment.

Although our model resembles previous attempts that integrated more than one model for the prediction of PA behavior change [83,85], it is important to mention that we do not position our model as a new predictive model for behavior change and that it is not an attempt to “reinvent the wheel”. Instead, we position our model in an intervention-based context where its goal is mainly to encompass the most potential determinants of change to build an effective intervention.

**Table 4:** Constructs of the DEF intervention model and related evidence-based or theory-based behavior change techniques.

Construct & behavior change techniques			DEF examples
**Constructs included in the tailoring assessment**			
	**Self-efficacy**		
		Reduce negative outcomes [78]	The program provides tailored evaluative feedback^a^on self-efficacy that reassures participants with low self-efficacy scores.
		Self-talk [78]	At the end of an action-planning activity, the program encourages participants to persevere no matter what the final outcomes of the week’s plan are.
		Social support (unspecified)^b^[5,78]	An MI-like session is provided after feedback on self-efficacy, allowing participants to select strengths that could help them change. An elaboration exercise follows where participants conduct a more in-depth evaluation of how those strengths could help them change concretely.
		Vicarious experiences [79]	Video testimonies of 2 regularly active people with type 2 diabetes are provided during the program’s 6th tailored motivational session.
		Imaginary reward [37]	During the 6th tailored motivational session, participants are invited to explore what their ideal PA practice would be like 1 year after the program. They are also asked what they would gain from this accomplishment and how proud they would feel if they succeed at being regularly active within a year after the program.
		Social reward [78]	During motivational sessions 4 and 8, participants are valorized by the program if they took part in more PA during the program than when they started. The self-monitoring tool praises participants with evaluative feedback^a^every time they participate in a new PA session.
		Self-reward [78]	If the participant’s current PA level is higher than their initial PA level during motivational sessions 4 and 8 (when PA level is reassessed), an evaluative feedback^a^is provided advising participants to be proud of themselves.
	**Confidence ruler**		
		Social support (unspecified) [5,78]	Tailored comparative progress feedback^a^is provided during motivational session 8; it compares the initial confidence ruler score with the current one. The feedback is accompanied by an MI question eliciting change-talk tailored to their current confidence ruler score.
	**Attitude**		
		Information about health consequences [5,78,80]	Participants are informed on the consequences of an insufficient PA level during motivational session 1 and on the benefits of an optimal PA level during motivational session 2.
		Reduce negative outcomes [78]	The program provides tailored evaluative feedback^a^based on the overall attitude score that reassures participants when the score is low, and congratulates them when the score is high.
		Social support (unspecified) [5,78]	Two MI-like sessions are provided aiming at changing attitude of participants. Motivational session 1 allows participants to select two health-risks associated with type 2 diabetes that they want to avoid, which they later elaborate on to analyze what the concrete impacts of those risks in their lives would be. Similar activities are repeated during motivational session 2. This time, participants identify two PA benefits that are important to them instead of two health risks.
	**Importance ruler**		
		Social support (unspecified) [5,78]	Tailored comparative progress feedback^a^is provided during motivational session 4. This feedback compares the initial importance ruler score with the current one and is accompanied by an MI-based question eliciting change-talk tailored to their current importance ruler score.
	**Intention**		
		Social support (unspecified) [5,78]	Tailored comparative progress feedback^a^is provided during motivational sessions 4 and 8. It compares the initial intention score with the current one and is accompanied by an MI-based question eliciting change-talk.
		Reduce negative outcomes [78]	The program provides tailored evaluative feedback^a^on the intention score; it reassures participants with a low score and congratulates participants with a high one.
	**PA behavior**		
		Feedback on behavior [78,81]	During motivational session 1, tailored comparative-normative feedback^a^comparing the participants’ current PA level with the PA recommendations of the Canadian Diabetes Association is provided to participants.
		Review behavioral goal(s) [5,78,81]	During their action plan activities, participants are encouraged to choose a higher PA level than their current one for their weekly goals; however, we recommend setting a higher goal only if they feel confident enough to reach it.
		Social comparison [78]	During motivational session 1, participants are informed about the percentage of Canadians with type 2 diabetes meeting PA recommendations versus those not meeting them.
		Instruction on how to perform the behavior [78,80]	Participants are advised about what PA intensity to aim for. In addition, a tab is dedicated to safety advice for people with type 2 diabetes practicing PA.
		Commitment [78]	At the end of the action plan tool, participants can click on a commitment check box that reads “I, (name of participant), commit to executing my action plan for the week”.
		Self-monitoring of behavior [18,78,81,82]	A specific tool on the website is dedicated to participants who wish to monitor their behavior.
	**Type of motivation**		
		Social support (unspecified) [5,78]	During motivational session 3, the program provides descriptive feedback^a^on type of motivation whether participants possess more controlled or autonomous forms of motivation. During the same motivational session, participants are asked to identify their most important values to see how regular PA would help them be more “congruent” with these values in their daily lives.
**Constructs not included in the tailoring assessment**			
	**Awareness: Cues, knowledge, and risk perception**		
		Feedback on behavior [78,81]	See Feedback on behavior for PA behavior.
		Information about health consequences [5,78,80]	See Information about health consequences for Attitude.
		Prompt/cues [78]	When participants complete an action plan, they receive an email advising them that it is now time to execute it. In their action plan, participants also select the days that they will practice their physical activities.
	**Social influence**		
		Social support (unspecified) [5,78]	Social support is provided through our MI-SDT concept application construct throughout the program activities (see Table 2).
		Social comparison [83]	See Social comparison for PA behavior.
	**Action plans—barriers**		
		Goal setting (behavior) [18,78,81,82]	Participants are asked to set a behavioral goal (ie, a PA level they want to reach for a specific week) each time they build an action plan.
		Problem-solving [5,78]	During their action-planning activities, participants can select which barrier is more likely to prevent them from being active and then select a solution to overcome this barrier accordingly.
		Action planning [80,83]	A specific tool on the website serves to build action plan for each week of the program.
	**Personal factors**		
		Content is personalized for each participant [60]	All emails and messages of the motivational sessions are adapted by gender (Contextualization^a^) and use the participants’ names (Identification^a^). Participants are also explicitly advised that the program will give them feedback based on their answers to the tailoring assessment questionnaire (Raising expectations of customization^a^).
	**Message factors**		
		Tailored messages adapted to participants’ scores of the DEF model constructs [60]	Tailored feedback is provided during each motivational session for constructs included in the tailoring assessment questionnaire.
	**Channel factors**		
		Use of the Internet and emails [12]	Only website pages and emails are used to deliver messages to participants.
	**Source factors**		
		Credible source [84]	The team responsible for the DEF intervention is composed of exercise and diabetes experts. Each motivational session starts with a video featuring an exercise specialist who explains the purpose of each session.

^a^The terminology used for the types of tailored feedback and other tailored components is based on the Harrington & Noar reporting standards for tailored interventions [33].

^b^Motivational Interviewing techniques used as part of the DEF intervention have been coded as *social support (unspecified)* because the behavior change techniques taxonomy used for this paper labels Motivational Interviewing as such [34]. Details about all motivational sessions and intervention overview are provided in Steps 2 and 4.

##### 2. Factors Contributing to the Effectiveness of Tailored Interventions Targeting Physical Activity Behavior

Although many reviews have been published in recent years in the field of tailoring, few aimed to describe factors contributing to the effectiveness of such interventions. Noar et al discovered that tailored interventions targeting health behavior change are more effective when (1) there are multiple contacts with participants, (2) they include theoretical, behavioral, and demographic constructs, (3) they use more than three constructs for tailored content, and (4) they target constructs such as self-efficacy, stages of change, social support, and processes of change [20]. Krebs et al noted that tailored interventions are more effective when (1) there are multiple contacts with participants and (2) they use dynamic tailoring (ie, assessing intervention variables prior to each feedback) instead of static tailoring (ie, providing one baseline assessment on which to base all successive feedback) [22]. These factors were all implemented in the intervention, except for the inclusion of two constructs, stages of change and processes of change, which do not fit with the DEF theoretical framework.

##### 3. Factors Contributing to the Effectiveness of Web-Based Interventions Targeting Physical Activity

Regarding Web-based interventions targeting PA, many reviews mentioned factors associated with the effectiveness of these interventions. The contributing factors are (1) intervention targeting only one behavior [19], (2) extensive use of the theoretical framework [19], (3) inclusion of multiple behavior change techniques [19], (4) use of additional methods for interacting with participants (mainly text messages and emails) [19], (5) inclusion of educational components [12,86,87], (6) longer intervention durations [12], (7) use of Social Cognitive Theory [12] or Theory of Planned Behavior [19], (8) regularly updating the content of the intervention [12], (9) inclusion of a self-monitoring tool [87], (10) inclusion of tailored information [87], (11) allowing communication with the health care provider [87], (12) linking the intervention with an entity already known by participants [88], (13) inclusion of an exercise program [87], and (14) allowing communication with other participants [87]. These factors have all been considered for the development of the DEF intervention except for the last two (ie, inclusion of an exercise program and allowing communication with other participants) since we wanted to limit our intervention to a fully automated, tailored perspective without any peer-support strategies (further details are provided in Step 10) and to let participants choose their own PA goals and activities. Finally, it can be inferred that the use of Social Cognitive Theory and Theory of Planned Behavior is considered for the intervention in that the I-Change Model integrates ideas from both of these theories.

##### 4. Effective Behavior Change Techniques Used in Interventions Targeting Physical Activity Behavior

In recent years, many reviewers also focused on effective behavior change techniques targeting physical activity [5,12,18,78-82,84]. Most evidence-based behavior change techniques found in our literature review are used for the intervention and can be seen in Tables 2 and 4. However, one evidence-based behavior change technique, focus on past success [5], has not been retained. Our main concern about this technique was related to the actual context of the study. Since participants recruited were people not meeting Canadian PA recommendations, we suspected that this strategy could have undermined the basic need of competence for those who never experienced any past PA successes prior to the intervention. Given this suspicion, our team chose not to implement this technique because we were unsure how it could harmlessly fit into our intervention.

#### Collecting Original Data

Prior to the development of the intervention, a research team member (FB) conducted a study using an extended version of the Theory of Planned Behavior as the theoretical framework to examine the key determinants predicting the practice of regular PA among individuals with type 2 diabetes [89]. The study results offered a good starting point for the selection of the constructs used for the intervention.

To conclude Step 1, it is also important to note that our literature review included three other papers not mentioned so far, which provided insights into common beliefs related to aerobic PA among people with type 2 diabetes [9,90] and aerobic PA preferences among Canadians with type 2 diabetes [84].

### Step 2: Developing the Program Framework

#### Defining the Program Objectives

Program objectives were based on the 2013 Canadian Diabetes Association guidelines related to PA for adults between 18 and 65 years old [4]. The DEF intervention limited its focus strictly on Canadian Diabetes Association guidelines specific to aerobic PA since it is one of the two most important types of PA among people with type 2 diabetes [4] and is less complex to manage than resistance PA in a fully automated online intervention perspective. The main objectives of the intervention are to (1) increase aerobic PA level of people with type 2 diabetes to 150 minutes of moderate to vigorous aerobic PA per week and (2) maintain or increase the total number of minutes of moderate to vigorous aerobic PA gained per week, at 1 month and 6 months after the end of the DEF intervention.

Inclusion criteria to be eligible to participate in the intervention are having type 2 diabetes, not meeting the Canadian Diabetes Association guidelines related to aerobic PA, being able to understand French, being able to navigate on the Internet, being between 18 and 65 years of age, and not having medical indications limiting the practice of PA.

#### Defining Program Constraints

The program development was limited to CDN $60,000 from a $232,000 project budget and a 3-year study timeline. The tailored content of the website was developed by the research team with a software offering advanced possibilities called TailorBuilder [91], which is dedicated to research that uses personalized online questionnaires and feedback. Flowchart algorithms and website design templates were also developed by the research team with LucidChart [92], which is an easy-to-use computer-based and Web-based platform used for diagramming tasks.

Other parts of the intervention were developed with external human resources and expertise. First, along with the MI workshop participation of 2 researchers on the team (MM, FB), we benefited from consulting with an MI expert who provided guidance on how to best implement MI strategies into our fully automated, Web-based intervention. Second, website development and implementation of the tailored content into the website were performed entirely by AlphaZero, an Internet solution company [93]. Third, all intervention videos (n=11) were created by a media solution company called Point Bleu Productions [94] and were then uploaded onto the Internet with an upgraded license of Vimeo [95], permitting privacy settings necessary for our intervention. Fourth, Step 8, usability evaluation, was executed jointly with a specialized researcher who developed an interview guide for this evaluation and advised the research team on ways to achieve this step properly. Last, we used free software, BB FlashBack Express [96], which allows computer screen, video, and sound recording, for the usability evaluation.

#### Describing the General Program Framework and Its Components

Based on the evidence identified in our literature review, program constraints, and the research team’s expertise, a general framework was developed. A detailed graphic overview of the DEF intervention timeline and components is presented in Multimedia Appendix 1.

An 8-week Web-based, tailored intervention was conceived with the information gathered. The intervention contained a self-monitoring tool, an action planning tool, a motivational session for each week of the program, multiple uses of email, and extra tabs providing safety tips, answers to frequently asked questions, and research team technical support.

The tailored motivational sessions constitute the core of the intervention. The order in which each motivational session is positioned was established in such a way as to correspond to the theoretical framework developed in Step 1. First, motivational session 1 aims to raise awareness among participants on how their insufficient PA level can impact their health. This corresponds to the first phase of the I-Change Model: increasing the importance of change in participants. Second, all other sessions aim to motivate participants toward increasing their PA level over the course of the intervention. This corresponds to the second phase of the I-Change Model, which is to develop motivation toward making the desired change among participants. Third, all motivational sessions are built to end by asking participants if they wish to build an action plan for the week, which aims to help them take action and thus increase their PA level. This last step is the final phase of the I-Change Model where motivation is transferred into the adoption of the desired behavior.

Regarding the integration of SDT and MI concepts, the research team attempted to implement the strategies outlined in Table 2 throughout each intervention component to provide a social environment that supports the basic needs of competence, autonomy, and relatedness. In addition, in order to develop an autonomous motivation in participants, all motivational sessions were geared so that participants could develop their own reasons to practice more physical activities, using various techniques associated with MI and its basic interview skills: (1) asking open questions, (2) affirming, (3) reflecting, (4) summarizing, and (5) informing and advising [37]. MI techniques and strategies are used throughout the sessions to elicit as much change-talk as possible from participants, as this is the main objective for MI practitioners because more change-talk is likely to better induce change in participants [66]. Finally, it should be mentioned that the development of the tailored motivational sessions used the latest research by Friederichs et al on MI-based tailoring [39]. More specifically, the sessions vary between the use of multiple choice and open-ended questions throughout the intervention to provide the most effective and enjoyable experience for participants.

Multimedia Appendix 1 provides more details on each session and intervention components.

#### Ethics

Ethics approval was obtained from the Ethics Committee of Research with Humans from the Université du Québec à Trois-Rivières on June 30, 2011.

#### Consent

Written informed consent was obtained from participants who participated in the usability evaluation and online informed consent was obtained from participants in the DEF intervention for publication of this report.

### Step 3: Developing a Tailoring Assessment Questionnaire

The questionnaire was developed using the constructs selected for the tailored content of the intervention. In addition to these constructs, sociodemographic and anthropometric data (self-reported) were collected including height, weight, age, occupation, origins, and marital status. Items related to each tailored construct are presented in Table 5.

**Table 5:** Description of the variables assessed for the tailored content of the intervention.

Construct	Items, n	Questions and possible answers
PA behavior	6	During a typical 7-day week, how many times do you practice the following activities for at least 10 consecutive minutes? (adapted version of GLTEQ) [97,98]: items are number of times/week and minutes/time for vigorous, moderate, and low PA
Intention	3	I intend to be regularly active in the next month [9]: totally disagree (-3) to totally agree (+3)
		My plans are to practice physical activities regularly in the next month [9]: totally disagree (-3) to totally agree (+3)
		I estimate that my chances of practicing physical activities over the next month are [9]: extremely weak (-3) to extremely good (+3)
Attitude	4	I think that practicing physical activities regularly in the next month would be [9]: very bad (-3) to very good (+3); very useless (-3) to very useful (+3); very unenjoyable (-3) to very enjoyable (+3); very unpleasant (-3) to very pleasant (+3)
Self-efficacy	3	I feel capable of practicing physical activities regularly in the next month [9]: totally disagree (-3) to totally agree (+3)
		For me, practicing physical activities regularly in the next month would be [9]: very difficult (-3) to very easy (+3)
		How much control do you feel you have over the fact of practicing physical activities regularly [9]: no control at all (-3) to complete control (+3)
Type of motivation	23	I will practice physical activities in the next month because… [99,100] (BREQ-2 combined with four more items measuring integrated motivation)
Importance ruler	1	How important is it for you to practice physical activities regularly [37]: absolutely not important (0) to absolutely important (10)
Confidence ruler	1	How confident are you that you can practice physical activities regularly [37]: absolutely not confident (0) to absolutely confident (10)
Gender	1	Select your gender (n/a): male or female
First name	1	What is your first name? (n/a) (open question)

This tailoring assessment questionnaire will be incorporated into the website, within the registration process, which participants will have to complete to officially be part of the intervention. This registration process can be completed during the 2-week enrollment period and includes five main steps: (1) creating a website account, (2) completing the selection criteria phase, (3) reading the detailed information about the project, (4) giving informed consent, and (5) completing the tailoring assessment questionnaire.

Moreover, some constructs were reassessed during motivational session 4 and motivational session 8 to provide a dynamic tailoring experience and comparative-progress feedback to participants. The constructs that were reassessed during motivational session 4 are PA behavior, intention, and importance ruler. During motivational session 8, the reassessed constructs were PA behavior, intention, and confidence ruler. There are two major reasons that confidence ruler and importance ruler were used instead of self-efficacy and attitude to provide comparative-progress feedbacks. The first reason is that fewer items are necessary to measure MI-constructs compared to those of the I-Change Model, which eases the task of answering questions for participants and may make the motivational sessions more enjoyable. The other reason is that MI constructs were developed specifically for intervention purposes. In other words, scores obtained on importance ruler and confidence ruler allow the program to provide an appropriate tailored feedback aimed at eliciting change-talk and therefore better foster autonomous motivation toward change in participants.

### Step 4: Creating Tailoring Algorithms

Tailoring algorithms are the logic statements or decision rules that specify which messages should be given to which participants under which circumstances [53]. These algorithms were created for the following intervention components: (1) enrollment process, (2) all motivational sessions, and (3) the action planning tool. In order to have a clear view of the algorithms required, we used LucidChart to create flowchart representations of the algorithms, giving an overview of all the processes and decisions to be made for each component. All flowcharts can be examined in detail in Multimedia Appendix 2.

A flowchart can be viewed as a type of diagram that uses symbols to graphically represent the processes, decisions, operations, and other concepts of a program [101]. Although there is a standardized use of specific symbols when drawing flowcharts [101], it is important to mention that we did not fully adopt the correct use of these symbols due to the fact that this methodology comes from a computer science and engineering background, which is not our primary expertise. However, all flowcharts created for the intervention are detailed and designed in a way that health professionals who are not familiar with the flowchart method will still be able to understand the algorithms of each component.

With the flowchart representations completed, the next phase in officially creating the algorithms is to transform the representations into real computation formulas. This phase was executed in Step 7, automating the tailoring process, with the TailorBuilder software.

### Step 5: Writing Tailored Messages

A message booklet was developed for the tailored and non-tailored components of the intervention. The parts of the booklet related to the tailored components were developed based on the flowcharts created in Step 4. For these tailored components, a variable name was assigned to each possible message block to facilitate the later automation of the tailoring process (Step 7). For the non-tailored components, messages were written for the website home page, main menu (when participants are logged in to the website), all emails that participants will receive, the self-monitoring tool, and Safety Tips, FAQs, and Contact tabs. Because of many updates made during the development of the intervention, no official message booklet can be offered as a reference in this paper. However, a sample of the message booklet is provided in Multimedia Appendix 3.

Considerable efforts were also made to write every intervention message in a needs-supportive style of communication to correctly apply the SDT and MI concepts outlined in Table 2. By considerable efforts, we mean that messages were written with careful attention by MM and FB to support basic needs, based on knowledge gained from our literature review on MI and SDT, MI workshops attendance, and guidance from our MI expert collaborator. However, once written, the message booklet was reviewed only by the research team members. Neither our MI expert collaborator nor an SDT expert reviewed the message booklet. To verify if the messages adequately support the basic needs of competence, autonomy, and relatedness, participants will be asked to answer an adapted version of the 6-item modified Health Care Climate Questionnaire [102] 1 month after the intervention (first follow-up).

### Step 6: Developing Design Templates

The development of the intervention components design was executed in two parts. First, non-tailored components were designed by a member of the research group (MM) with LucidChart. Web pages corresponding to each of these components were drawn for a first time by MM and then discussed and revised with the Web solution company and FB to refine them. All mock-up templates created with the software can be seen in Multimedia Appendix 4. Second, tailored components were designed with TailorBuilder via an integrated hypertext markup language (HTML) editor, combined with external cascading style sheets (CSS) used to design certain parts of the messages. The intervention logo was designed by the communications service at the Université du Québec à Trois-Rivières.

It is also important to mention that in addition to the discussions held with the Web solution company, efforts were made to respect some of the general design principles described in the program-planning of Kreuter et al [53]: (1) preferably have one objective per Web page, (2) choose a single feel for the tailored feedback provided (eg, authoritative, empathetic), (3) do not overfill pages with content and leave empty space, (4) be consistent with illustrations or photographic style, (5) limit the design to a few font styles, and (6) use bold effects and color sparingly. Examples of the application of these principles are presented in Table 6.

**Table 6:** Examples of the application of the general design principles proposed by Kreuter et al.

Design principles	Example of application
Preferably have one objective per Web page	All intervention components are separated into different tabs on the website. Each page of the tailored motivational sessions and action-plan tool usually only has one specific goal, maximum two.
Choose a single feel for the tailored feedback provided	Efforts were made to ensure that the intervention content is written in a needs-supportive and MI style of communication.
Don’t overfill pages with content and leave empty space	Participants can view the content of a page involving tailored components without having to scroll. Messages are separated in short paragraphs.
Be consistent with illustration or photographic style	Because our population varies between 18-65 years of age, we chose not to integrate any images in the website in order to be as inclusive as possible. The only images on the website appear in the introduction videos of each tailored motivational session. The age of the people in these images varies extensively.
Limit the design to a few font styles	All content is written in Arial, with a font size of 16 points or more.
Use bold effects and color sparingly	Generally speaking, only logo colors are used on the website. Bold effects are used for the title of the Web pages and occasionally to emphasize an important message.

### Step 7: Automating the Tailoring Process and Creating the Website

The Web solution company created most of the non-tailored intervention components using the previously designed mock-up templates of Step 6. These non-tailored components were thus transformed into functional Web pages and include the following: (1) website home page, (2) account creation page, (3) a more info page, (4) a forgotten password page, (5) safety tips tab, (6) FAQs tab, (7) contact tab, and (8) a self-monitoring tool.

All other components were generated into functional Web pages using TailorBuilder and include the following: (1) tailored motivational sessions, (2) action-planning tool, (3) main menu when participants are logged in, (4) follow-up questionnaires, (5) pre-programmed emails, and (6) an enrollment session including selection criteria, detailed information about the project, consent of participants, and the tailoring assessment questionnaire. These components were generated in two major steps. First, messages and questions related to each component were linked with their appropriate coding variable and integrated into their HTML/CSS format. Second, by using the variable codes assigned to the entire set of messages and questions, all the algorithms were translated into their computer format, which is mainly composed of decision rule statements (eg, IF…ELSE…THAN…) implemented with a simplified programming language integrated in TailorBuilder. An overview of all the programming code written with TailorBuilder is presented in Multimedia Appendix 5.

Once the two parties involved in this step finished creating their Web pages, the Web solution company merged all the Web pages into one website, thus creating the complete DEF Web platform ready for Step 8, evaluating usability.

### Step 8: Evaluating Usability

Our usability evaluation not only assessed the users’ ability to perform tasks successfully on the DEF website but also examined the users’ broader interaction with the website comprising their thoughts, feelings, and perceptions [103]. The method used to execute this step was a first experience for the research team and was based on regular practice in the field of usability evaluation [103,104]. Detailed activities of this step are described below.

In order to receive guidance on the best way to evaluate our website, we first recruited a field expert who assisted the team in the development of an evaluation framework, but not for the realization of the evaluation itself. Concretely, by examining the project’s needs and constraints for the project, this expert provided an interview guide, concrete guidelines to follow when running this kind of evaluation, and advice on tools we can use to facilitate the evaluation. Since we also recruited an external interviewer for the evaluation, some guidelines were discussed extensively with this professional to standardize the interview procedures.

Participants for the usability evaluation were recruited from a diabetes education center located in Trois-Rivières. A small sample of the study population (n=11) was recruited. Some characteristics of the participants were (1) 6 females, (2) average age of 53.5 years, (3) all but one not meeting PA recommendations, with a group average of 50 minutes per week of moderate aerobic PA, (4) average years with type 2 diabetes diagnosis was 8.11, (5) all but one using the Internet at least daily, for an average of 1 hour per session, (6) 5 participants with elementary school as their highest education level, 2 participants with high school, and 4 with a university degree or higher, and (7) all participants followed by at least one health professional. Although small, this sample seems representative of the population targeted and the larger population of Canadian adults with type 2 diabetes [105,106].

Participants were invited to the facilities of the Université du Québec à Trois-Rivières, where one-on-one interviews were conducted in a research lab by the external interviewer (a clinical nurse) using a think-aloud protocol and a software allowing for sound, video, and computer screen recording. During the interview, participants were asked to execute some predefined tasks on the website, while all of their facial expressions, comments and actions on the website were recorded. While participants executed the tasks, the interviewer used the interview guide to make sure that the participants voiced their thoughts and feelings to be able to measure their comprehension and enjoyment of specific parts of the website. The data collected during the recorded interviews was used for analysis and usability evaluation on various metrics including time on task, number, and type of errors made, bugs, and relevant issues [103]. All metrics were useful in making website improvements.

**Figure 2:**
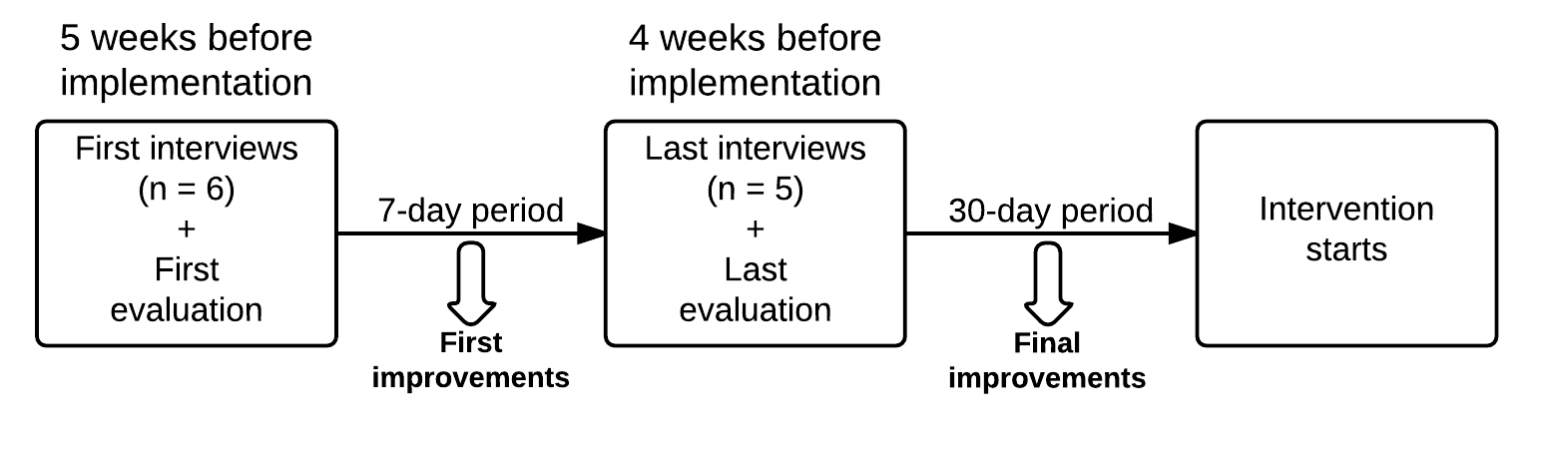
Iterations of the usability evaluation of the DEF intervention.

It is also important to mention that the evaluation was conducted iteratively. That is, a first evaluation period took place about 5 weeks before the intervention was implemented. During this first period, six interviews were conducted over a 2-day period after which a first set of improvements was made to the intervention. Four weeks prior to the intervention implementation, a second evaluation period took place during which five interviews were again conducted over a 2-day period. After this final evaluation period, a month was granted for final improvements before implementing the intervention. Due to the project’s time restrictions and because we felt quite confident about launching the intervention with the data collected during the two evaluation periods, no other usability testing was conducted. See Figure 2 for iterations of the usability evaluation of the DEF intervention.

Following this step, numerous improvements (ie, more than 40) were made, ranging from minor (eg, changing a word that was hard to understand on a page to an easier one) to major (eg, redesigning a whole page). Here are some of the major improvements made to the website: (1) reduction of the number of pages that must be viewed to complete the enrollment process (ie, 5 pages fewer, from 20 to 15 pages), (2) replacement of the neutral background of videos to backgrounds inspiring PA behavior, (3) redesign and reorganization of the information on the self-monitoring tool page, (4) reduction of the length of the information messages and summary messages by about 50% for almost every tailored motivational session, (5) significant reduction in the number of choices for MI-based multiple choice questions in tailored motivational sessions 3 and 5, (6) redesign of the emails sent to recruit participants, and (7) reduction in the number of times participants have to scroll to almost zero.

### Step 9: Implementing the Program

In the Web-based context of the DEF intervention, data from participants are handled automatically through interactions between the website and TailorBuilder to provide appropriate feedback. This step therefore involves only establishing (1) how participants will be recruited and (2) how the data will be collected and exported to data-analysis software.

For the recruitment phase, the DEF tailored intervention was implemented in partnership with Diabète Québec, a recognized organization for Canadians with type 2 diabetes (particularly in the province of Quebec). Consequently, following a joint agreement between both parties, Diabète Québec sent two invitation emails over 2 weeks to nearly 2300 Canadian francophone adults with type 2 diabetes, who were members of its newsletter.

Sociodemographics and data related to each construct of our theoretical model were collected automatically by TailorBuilder. All data collection questionnaires related to the intervention were automated, so all answers given to each question of the questionnaires were automatically saved under their appropriate variable code and assigned to the appropriate participant. Data exportation was executed easily with TailorBuilder, which allows for the creation of Statistical Package for the Social Sciences (SPSS) files that merge all the data collected in a ready-for-analysis format.

### Step 10: Evaluating the Process, Impact, and Outcome

According to Kreuter’s program-planning model, this step serves to determine how effective the program was in meeting its objectives and how the program could be modified for greater performance in the future. Kreuter et al propose their own questions to properly evaluate a program. Our team decided to keep only some of these questions and to add others to better suit the context of our intervention. Questions that the research team will seek to answer through this step are (1) through which processes did behavior change occur in participants? (2) how effective was the program in meeting its behavioral objectives? (3) how effective was the program compared to another type of Web-based intervention using a more general approach (ie, non-tailored) combined with peer-support strategies? (4) was the intervention content well received by the participants? and (5) how can the intervention be improved in the future regarding every dimensions of the Reach Effectiveness Adoption Implementation Maintenance (RE-AIM) framework [107]?

In order to address these questions, it is important to note that this intervention is part of a larger project led by one of the authors (FB). This research project will specifically seek to compare the effectiveness of two types of Web-based interventions having the same behavior change purpose: (1) the intervention described in this paper and (2) a Web-based intervention offering similar but non-tailored content combined with peer-support strategies (ie, a Facebook discussion group).

## Results

### Overview

Readers can access all videos of the intervention in Multimedia Appendix 6. A demo version of the intervention website is also available in Multimedia Appendix 7.

### Development Cost

The DEF intervention development cost was about CDN $59,700 and took approximately 54 full-time weeks to develop. Details on the costs and duration of each step are provided in Table 7.

**Table 7:** Cost per step and total cost for the DEF intervention development.

Component		Cost, $CAD
**Step 1 duration: 11 weeks**		
	PsyMontreal Inc – guidance on MI applications for the DEF intervention	$230
	Three MI workshop participations	$1000
**Step 2 duration: 4 weeks**		$0
**Step 3 duration: 3 weeks**		$0
**Step 4 duration: 3 weeks**		
	LucidChart software license	$100
**Step 5 duration: 12 weeks**		$0
**Step 6 duration: 4 weeks**		$0
**Step 7 duration: 11 weeks**		
	AlphaZero – website development	$4500
	Point Bleu – media productions	$8500
	TailorBuilder software license	$5000
	Vimeo Plus membership	$70
**Step 8 duration: 6 weeks**		
	Usability evaluation expert guidance	$1000
	Usability evaluation interviews	$1300
**Step 9 duration: n/a**		
	Financial compensation paid to Diabète Québec	$3000
Researcher salary (MM) for the coordination of the intervention development (54 full-time weeks)		$35,000
Total cost for intervention development including Steps 1-9		$59,700

### Enrollment Period and Sample Size Calculation for the DEF Tailored Intervention

The enrollment period started on September 15, 2014, while the intervention officially started on September 29, 2014. Out of 2300 potential participants targeted for the DEF tailored intervention, approximately 530 people visited the website, 170 people completed the registration process, and 83 corresponded to the selection criteria and were enrolled in the intervention. Four weeks after the intervention started, no dropouts were reported. Similar enrollment results have been obtained for the two other groups of the study (ie, peer-support group and control group). Prior to recruiting participants, a statistical power analysis to determine the required sample size was conducted based on using repeated-measure analysis of variance to detect change in PA. Thus, according to a mixed plan 3 (ie, Web-based tailored intervention group, Web-based peer-support intervention group and control group) x 3 (ie, baseline, 1- and 6-month follow-up), assuming a statistical power of 0.80 and alpha=.05, a total sample size of 204 participants is necessary to detect a conservative effect size of *f*=.10 [20,22]. This sample size was calculated with the software G*Power 3.1.6.

## Discussion

### Principal Considerations

Through the experience of developing the DEF Web-based tailored intervention, the research team learned several lessons that will help guide future health professionals interested in developing such an intervention.

First, we confirm that the development of a tailored intervention can be quite a complex and time-consuming process, as has been highlighted by previous tailored intervention developers [35,108]. However, in our case, once the development and implementation parts were executed, we found that minimal effort was then required by the research team during the intervention itself. Considering the reach and effectiveness that this kind of intervention can have on health behaviors, our beliefs that this promising approach (ie, Web-based tailoring) can have a significant positive impact on a population level at a decent cost are reinforced. These beliefs are also increasingly supported by evidence [109-111].

Further adding to the complexity of the development, the use of multiple behavior change theories can also require additional efforts, as researchers must be sure to understand all theories and models underlying the intervention and ensure that the integration of these provides a coherent framework for later development steps. We chose to integrate the I-Change Model, SDT, and MI to further explore potential factors that will add to the current effectiveness of tailored interventions targeting PA behavior, as it has been suggested as an area for future research [18]. Similarly, it is also important to note that some evidence suggests that using multiple theories generally results in less effective interventions compared to the use of a single theory [112], but this trend does not seem to be well supported and other evidence suggests that the use of constructs derived from different theories can result in the long-term effectiveness of interventions [60].

Regarding the development method used for the intervention, the program-planning model developed by Kreuter et al was very useful for building an evidence-based tailored intervention. As we mentioned, very small changes were made to the initial framework in order to make it fit with our logic, context, and needs. However, one interesting area for future research could focus on developing new strategies to accelerate the process through which evidence-based tailored interventions are developed, given that this is generally a time-consuming task. For example, in our case, all steps were executed in a sequential manner (eg, once Step 1 was completed, Step 2 was executed), but future developers could examine the feasibility of executing certain steps simultaneously to reduce development time. Or, researchers could also try to determine which systems are faster for automating the tailoring process of interventions, whether it be TailorBuilder [91], other similar tailoring systems [113,114], the use of direct computer programming human resources, or other means.

For the automation of the tailoring process and the creation of the website, TailorBuilder has been a very important tool for Web pages including tailored components. This software allowed us to make these tailored Web pages a reality, even if the research team had minimal computer programming background. However, it is important to note that one member possessed some basic skills with the client-side programming languages CSS, HTML, and JavaScript, which we highly recommend acquiring before developing a Web-based tailored intervention, unless one plans on using specialized human resources for the entire website construction.

As noted earlier, the step added to the program-planning model of Kreuter et al, usability evaluation, was found to be very useful during the development of the intervention, as it allowed for several important website improvements. However, more iterations during the usability evaluation phase could have led to more insights on how to improve the website. Also, we might have gained more insights within the same amount of time by using a slightly different method such as the Rapid Iterative Testing and Evaluation method (RITE) [115] in which, under certain conditions, changes to the website can be made as soon as a problem is identified even after observing only 1 participant. In our case, the difference is that we waited until the end of each testing period, including 5 or 6 participants per period, to make any improvement. Plus, the method used did not allow us to test the final improvements on a sample of our target population before starting the intervention. In other words, only the research team was able to pre-evaluate these final improvements.

Additionally, our literature review covered a wide range of fields and provided a strong corpus of evidence on which to base the intervention. However, it is important to note that other fields of research that are less explored in the tailoring field could have further contributed to our evidence basis, such as fields related to message design (eg, the “look and feel” of messages) and message structure (eg, emotional appeals, narrative vs statistical, gain vs loss framing) [23]. In order to further optimize their interventions, future developers may want to consider these fields or others in their literature review and thus broaden their corpus of evidence.

Finally, to our knowledge, this paper presents one of the most exhaustive development descriptions of a Web-based, tailored intervention published to date. However, although we believe this work can help health professionals adopt a deeper view of what the task of developing such an intervention truly represents, we are also of the opinion that greater efforts could be made in future papers to be even more educational, contributory, and reproducible on many levels (eg, clarity of the programming code presented, completeness of message booklet and demo website, use of open source software to facilitate reproducibility). We encourage future developers to embrace this challenge.

### Limitations

Two main limitations related to the development of the intervention are worth mentioning at this stage of the project. First, the assessment of development costs was not performed according to a standardized method [116,117] and should be interpreted with caution. This assessment should be interpreted as including all costs associated with the development of the intervention (Steps 1 to 9) and paid from the grant awarded to FB by the Quebec Health Research Fund. All other potential development costs that could be associated with the project and not paid from the grant were not considered in the assessment, such as the salaries of 2 researchers, MPG and FB. Costs during the intervention and costs associated with Step 10 of our development model (eg, salaries for assistant researchers during these periods and financial compensation for follow-up completions) were also not included because we did not view them as strictly associated with the development phase of the intervention. Although not following a standardized method, and along with the abovementioned details, the cost assessment presented in this paper could guide future investigators on the tangible development costs they will have to pay directly from their awarded research grant, if following a similar development methodology.

Another limitation is that behavior maintenance may currently be minimally addressed by the intervention (ie, as a reference, interventions promoting better maintenance normally last more than 24 weeks [118]), while we clearly stated that it was one of our main goals along with PA behavior adoption. To explain our reasoning, we wanted to determine whether behavior maintenance could be promoted primarily by the use of intervention strategies intended to develop autonomous motivation in participants, thus leading them to keep practicing PA after the intervention for personally relevant reasons and enjoyment of the behavior. Since this is the major reason why we issued behavior maintenance as an intervention objective, we acknowledge that the intervention does not support all the scientific evidence related to PA maintenance available to date.

### Conclusions

This paper aimed to fill a gap in the field of Web-based intervention research, which is in need of more in-depth descriptions of the development process of such interventions. It provides a detailed example of a fully automated, Web-based, tailored intervention development for researchers interested in developing similar interventions. Also, as it represents a potential avenue to enhance the effectiveness of tailored interventions, this paper discussed the integration of the I-Change Model, Self-Determination Theory, and Motivational Interviewing as the theoretical framework of the intervention.

Finally, we reemphasize that usability evaluation, a step added to the program-planning model of Kreuter et al, is strongly encouraged prior to the implementation phase in order to better optimize an intervention before disseminating it on a larger scale.

## Multimedia Appendix 1

DEF program framework.

## Multimedia Appendix 2

DEF algorithms.

## Multimedia Appendix 3

DEF message booklet.

## Multimedia Appendix 4

DEF design.

## Multimedia Appendix 5

DEF TailorBuilder code.

## Multimedia Appendix 6

DEF videos.

## Multimedia Appendix 7

DEF demo website.

## Abbreviations

DEF: Diabète en Forme
MI: Motivational Interviewing
PA: physical activity
SDT: Self-Determination Theory
