# Supplementary material for: Development of a Fully Automated, Web-Based, Tailored Intervention Promoting Regular Physical Activity Among Insufficiently Active Adults With Type 2 Diabetes: Integrating the I-Change Model, Self-Determination Theory, and Motivational Interviewing Components
Source: JMIR Res Protoc. 2015 Feb 17;4(1):e25. doi: 10.2196/resprot.4099 (PMC4376153; doi:10.2196/resprot.4099)
Supplement: Supplementary file 1 [file resprot_v4i1e25_app1.pdf]

- ***Use "Ctrl" and "+" to zoom in and "Ctrl" and "-" to zoom out***

# Overview of the DEF intervention: Timeline and description of intervention components

Timeline

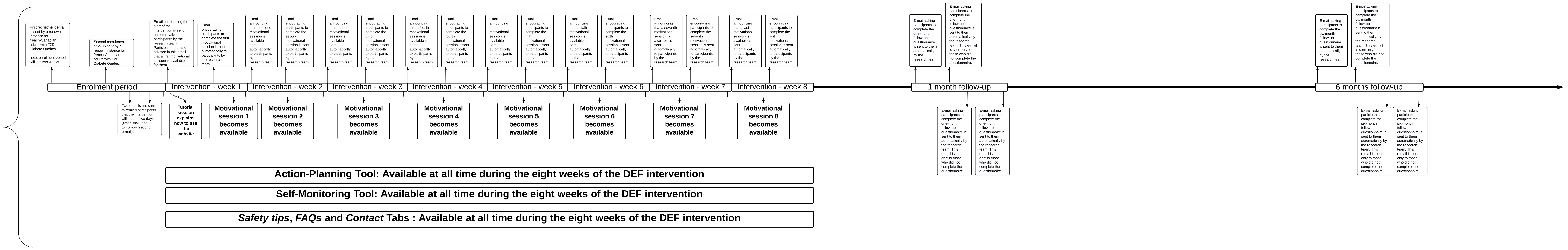


Intervention components

**Motivational session 1**

- First session starts with a video in which an exercise specialist explains the purpose of the session.
- First session follows with a comparative-normative feedback comparing baseline PA level of participants with the 2013 CDA recommendations for aerobic PA
- Since every participants involved in the DEF intervention are insufficiently active, participants are then invited to do an exercise in which they will explore which health risks related to their PA level and T2D are worrisome for them. Participants will be invited to elaborate on the impact of these risks in their lives. Reflections and affirmations on participants' answers are provided by the program.
- Participants are asked if they want to receive information on how practicing regular PA could help them avoid the risks they selected.
- Summary that is oriented toward change-talk of participants is provided at the end of the session.
- Participants are then invited, if they agree, to create an action plan for the current or following week.

**Motivational session 2**

- Second session starts with a video in which an exercise specialist explains the purpose of the session.
- Second session follows with an evaluative feedback on the attitude score of participants.
- Participants are then invited to do an exercise in which they will explore which benefits associated with regular PA would be the most important for them. After, participants are invited to elaborate on what could be the impact of these benefits in their lives. Reflections and affirmations on participants' answers are provided by the program.
- Participants are asked if they want to receive information on how practicing regular PA could help them obtain the benefits they considered important.
- Summary that is oriented toward change-talk of participants is provided at the end of the session.
- Participants are then invited, if they agree, to create an action plan for the current or following week.

**Motivational session 3**

- Third session starts with a video in which an exercise specialist explains the purpose of the session.
- Third session follows with an evaluative feedback on the type of motivation of participants.
- Participants are then invited to do an exercise in which they will select the values important to them. After, participants are invited to elaborate on how regular PA could help them be more congruent with their values. Reflections and affirmations on answers of participants are provided by the program.
- Participants are asked if they want to receive information about the topic of the session. If they choose to, participants are then explained how an autonomous motivation could help them do more PA and have more pleasure doing it.
- Summary that is oriented toward change-talk of participants is provided at the end of the session.
- Participants are then invited, if they want to, to create an action plan for the current or following week.

**Motivational session 4**

- Fourth session starts with a video in which an exercise specialist explains the purpose of the session.
- Fourth session follows with a new assessment on importance-ruler construct.
- A comparative-progressive feedback on importance-ruler follows directly after the assessment.
- The session continues with a new assessment on intention construct.
- A comparative-progressive feedback on intention follows directly after the assessment.
- The session continues with a new assessment on PA behaviour construct.
- A comparative-progressive feedback on PA behaviour follows directly after the assessment.
- Participants are then invited, if they want to, to create an action plan for the current or following week.

**Motivational session 5**

- Fifth session starts with a video in which an exercise specialist explains the purpose of the session.
- Fifth session follows with an evaluative feedback on self-efficacy score of participants.
- Participants are then invited to do an exercise in which they will select their most important strenghts. After, participants are invited to elaborate on how those strengths could help them be more active. Reflections and affirmations on answers of participants are provided by the program.
- Participants are asked if they want to receive information about what can influence their sens of self-efficacy. If they choose to, participants are then explained which factors can influence self-efficacy and how they could manage these factors.
- Summary that is oriented toward change-talk of participants is provided at the end of the session.
- Participants are then invited, if they want to, to create an action plan for the current or following week.

**Motivational session 6**

- Sixth session starts with a video in which an exercise specialist explains the purpose of the session.
- Sixth session follows with an exercise that allows participants to picture their ideal PA routine within 1 year time.
- Participants are then asked to choose the number of minutes of PA per week they would do, which physical activities would they practice, which solutions would they use to overcome barriers and how proud would they be to have succeeded at being regularly active within 1 year time.
- Summary that is oriented toward change-talk of participants is provided at the end of the session.
- Participants are then invited, if they want to, to create an action plan for the current or following week.

**Motivational session 7**

- Seventh session starts with a video in which an exercise specialist explains the purpose of the session.
- Seventh session follows with a descriptive feedback on change-talk evoked by participants during session 1 and 2.
- Session continues with a descriptive feedback on change-talk evoked by participants during session 3 and 5.
- Session continues with a descriptive feedback on change-talk evoked during session 6.
- Participants are congratulated for their progress and their work toward a higher PA level thus far.
- Participants are then invited, if they want to, to create an action plan for the current or following week.

**Motivational session 8**

- Eighth session starts with a video in which an exercise specialist explains the purpose of the session.
- Eighth session follows with a new assessment on confidence-ruler construct.
- A comparative-progressive feedback on confidence-ruler follows directly after the assessment.
- The session continues with a new assessment on intention construct.
- A comparative-progressive feedback on intention follows directly after the assessment.
- The session continues with a new assessment on PA behaviour construct.
- A comparative-progressive feedback on PA behaviour follows directly after the assessment.
- Participant are congratulated for their progress during the program and are then invited, if they want to, to create an action plan for the last week of the program.
- A conclusion message is provided to participants.

**Self-Monitoring tool**

- Participants can enter each physical activity session they have done during the intervention time-period.
- Participants can view their total of minutes of moderate to rigorous physical activity per week. A total of minutes is given for each week of the program.
- Each time they enter a new moderate to rigorous physical activity session, participants are given an evaluative feedback which intends to valorise their efforts toward a regularly active lifestyle.
- This tool is available at all time during the time of the intervention.

**Action-Planning tool**

- Participants can make an action plan for each week of the intervention.
- Action plans include solutions to overcome barriers, goal setting (behaviour), selection of physical activities (ex. fast walking, cycling, swimming, etc.), selection of week days on which physical activities will be executed, selection of most important motivations to complete the action plan and participant commitment to execute the action plan.
- Summary of action plans can be either printed (print button directly available on the summary page) or viewed directly from the web platform.
- Action planning tool can be either accessed after a motivational session or directly on the main page of the program.
- This tool is available at all time during the intervention.

**Emails**

- Recruitment: two emails are sent to a member's list of a renown instance for french-Canadians with T2D (Diabète Québec), one for each week of the enrolment period.
- Emails are sent automatically to enrolled participants to remind them of the start of the intervention and to announce new content on the website.
- An email is automatically sent to a participant who finishes a motivational session. The email congratulates the participant and encourages him to use other tools on the website.
- An email is automatically sent to a participant who finishes an action plan. The email congratulates the participant and gives him verbal emotional social support.
- 4 emails are sent to participants for each of the follow-up questionnaire.

**Other tabs**

- A tab, Safety tips, is designed to give participants some advices on how to practice moderate physical activity in a secure manner. Advices are given for various conditions associated to people with T2D .
- A tab, *FAQs,* is designed to answer some questions about PA (ex. what is the CDA recommandations?, what constitutes a moderate to vigorous physical activity?, etc.) or about the DEF intervention (ex. what is the purpose of the DEF program? what can I get from this program? etc.).
- A tab, *Contact,* is designed to encourage participants to contact the research team when needed and to give their appreciations and suggestions about the DEF intervention.
