# Supplementary material for: Development of a Fully Automated, Web-Based, Tailored Intervention Promoting Regular Physical Activity Among Insufficiently Active Adults With Type 2 Diabetes: Integrating the I-Change Model, Self-Determination Theory, and Motivational Interviewing Components
Source: JMIR Res Protoc. 2015 Feb 17;4(1):e25. doi: 10.2196/resprot.4099 (PMC4376153; doi:10.2196/resprot.4099)
Supplement: Supplementary file 2 [file resprot_v4i1e25_app2.pdf]

Read before browsing

- ***Scroll through the pages below to see the flowcharts for all tailored components***
- ***Use "Ctrl" and "+" to zoom in and "Ctrl" and "-" to zoom out***
- ***Gender is not included as a tailored construct in the flowcharts due to unnecessary duplications of the drawings. One can consider that all messages are adapted to the gender of participants.***

Signification of the symbols used in flowcharts:

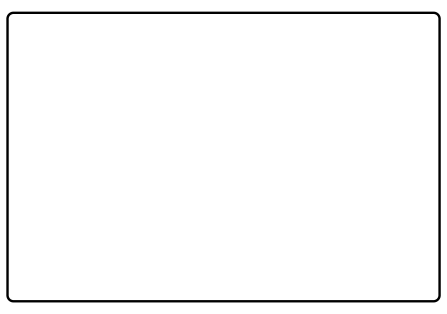

Indicates a description of a message given to the participant or a process followed by the participant.

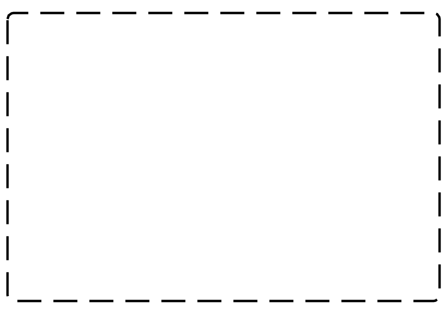

Indicates a description of the path taken by the participant in order to access a specific component.

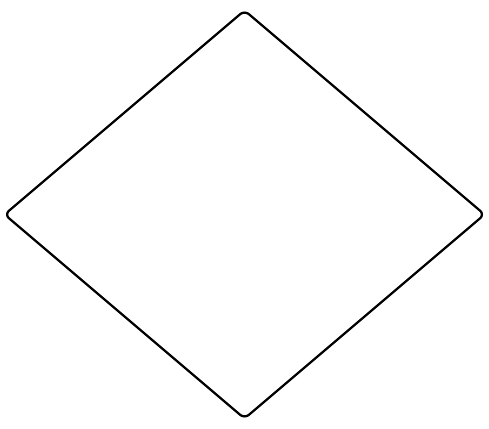

Indicates a question to be answered by the participant or by the program.

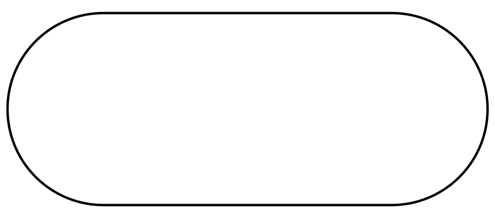

Indicates the start and the end of each component.

Indicates a default direction to which the participant is redirected.

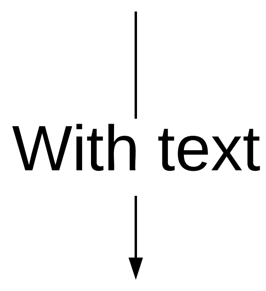

Indicates a decision made in order to redirect the participant appropriately based on previously obtained information.

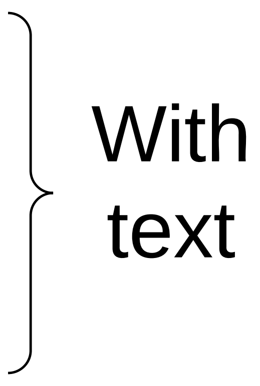

Provides additional notes about a specific part of a component.

Enrolment process

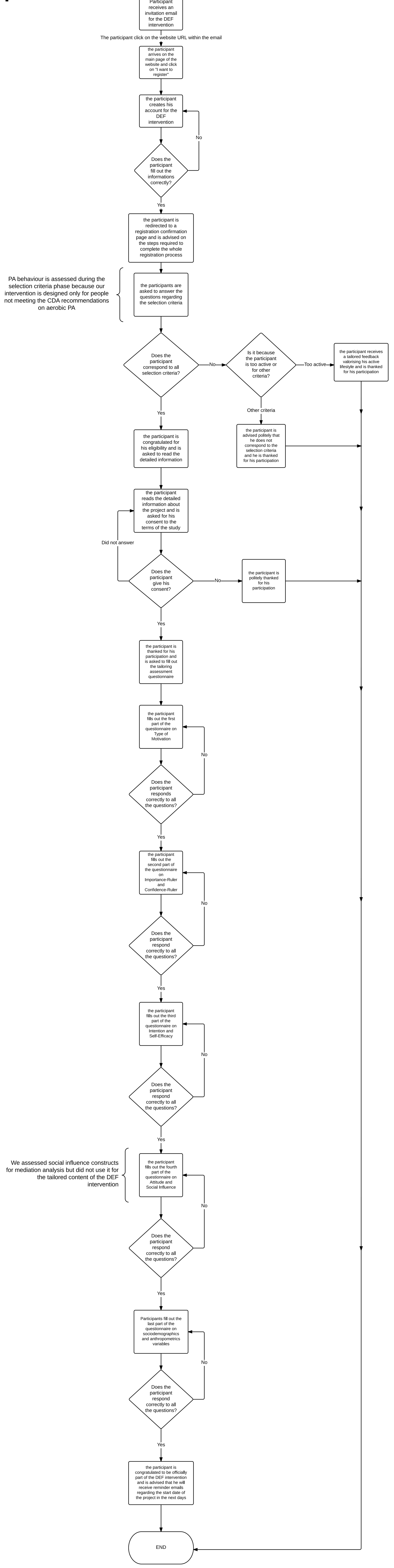

Tailored motivational session 1

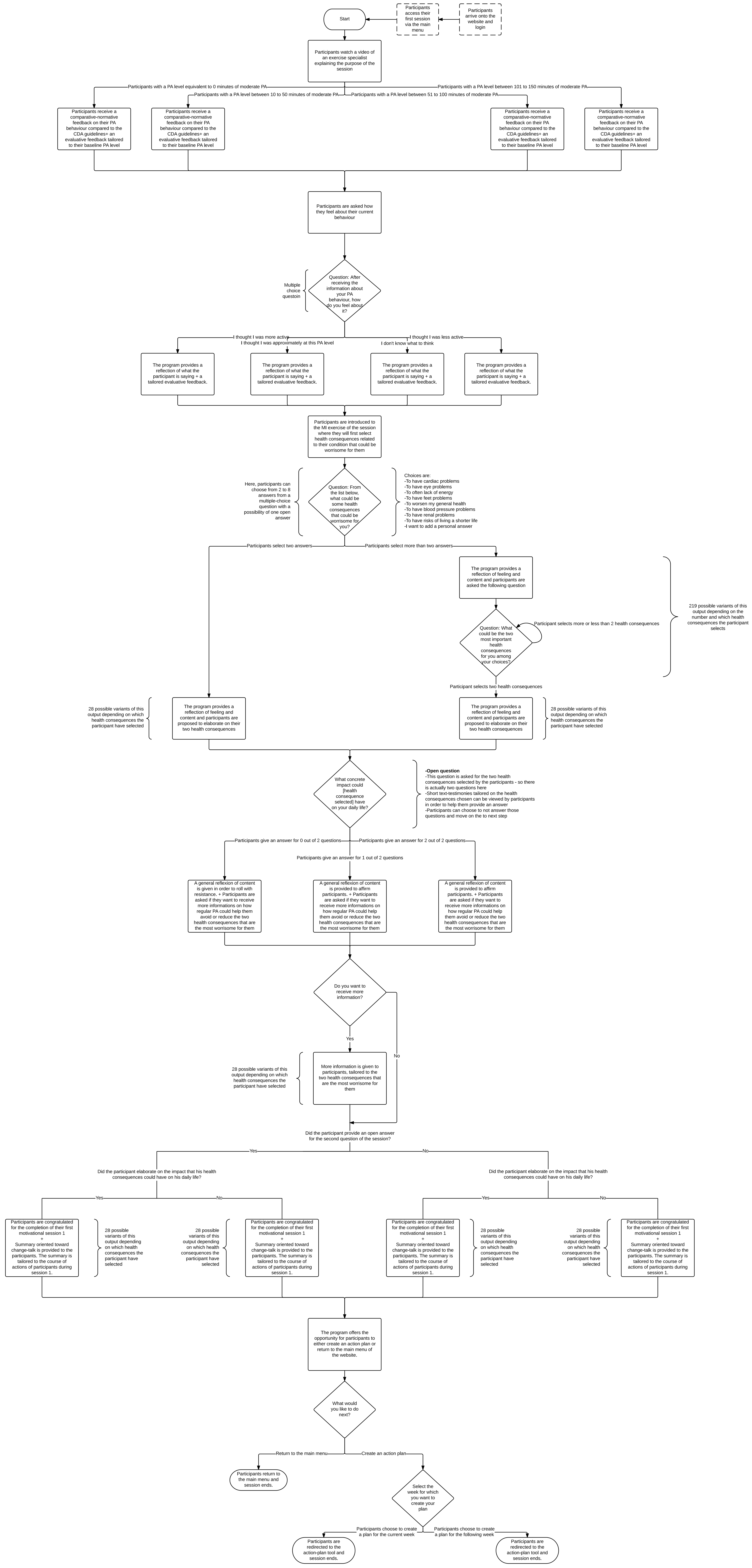

Total number of possible variations of the session: 142272

Total number of possible variations of the session when gender is included (approximation): 284544

Tailored motivational session 2

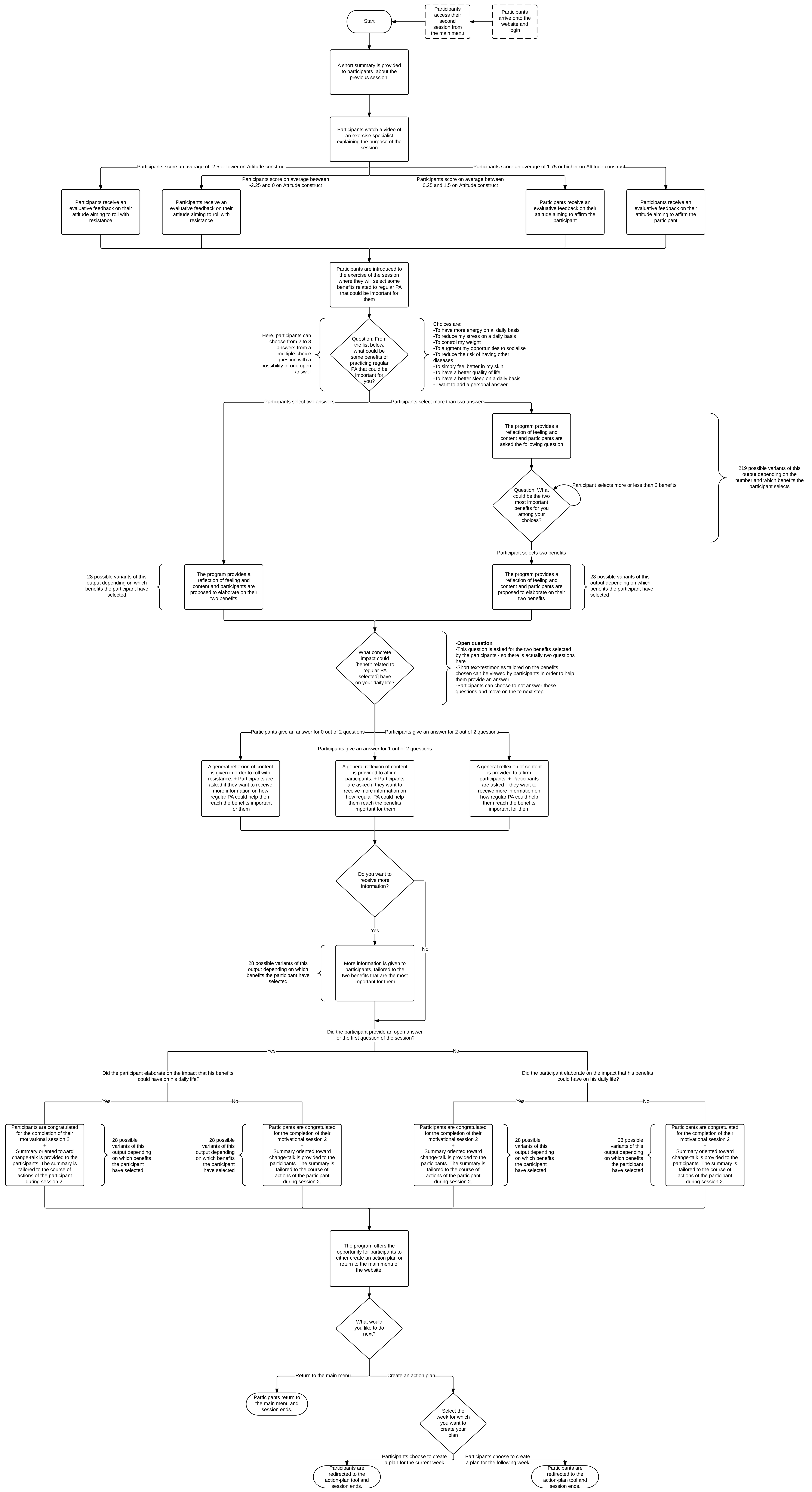

Total number of possible variations of the session: 106704
Total number of possible variations of the session when gender is included (approximation): 213408

Tailored motivational session 3

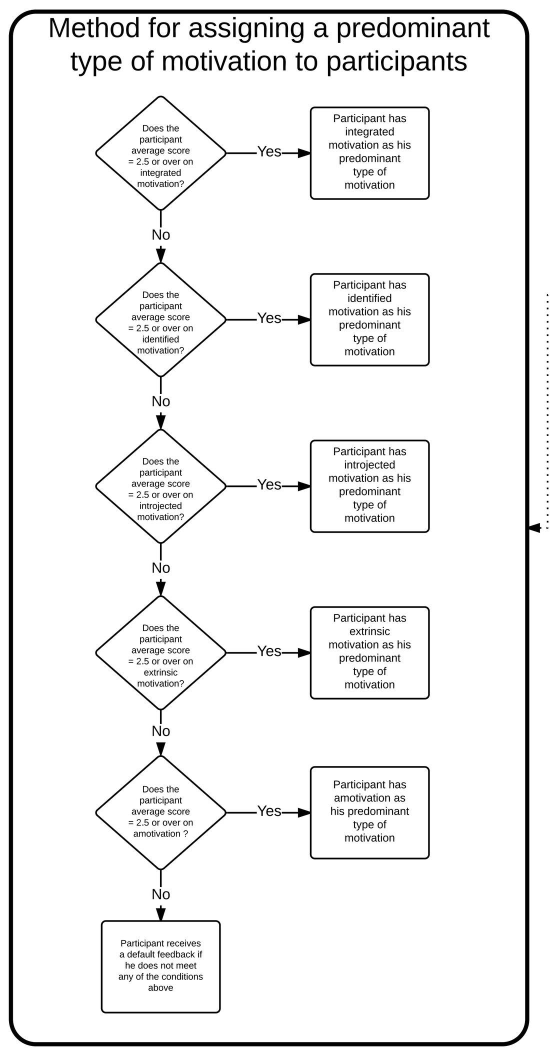

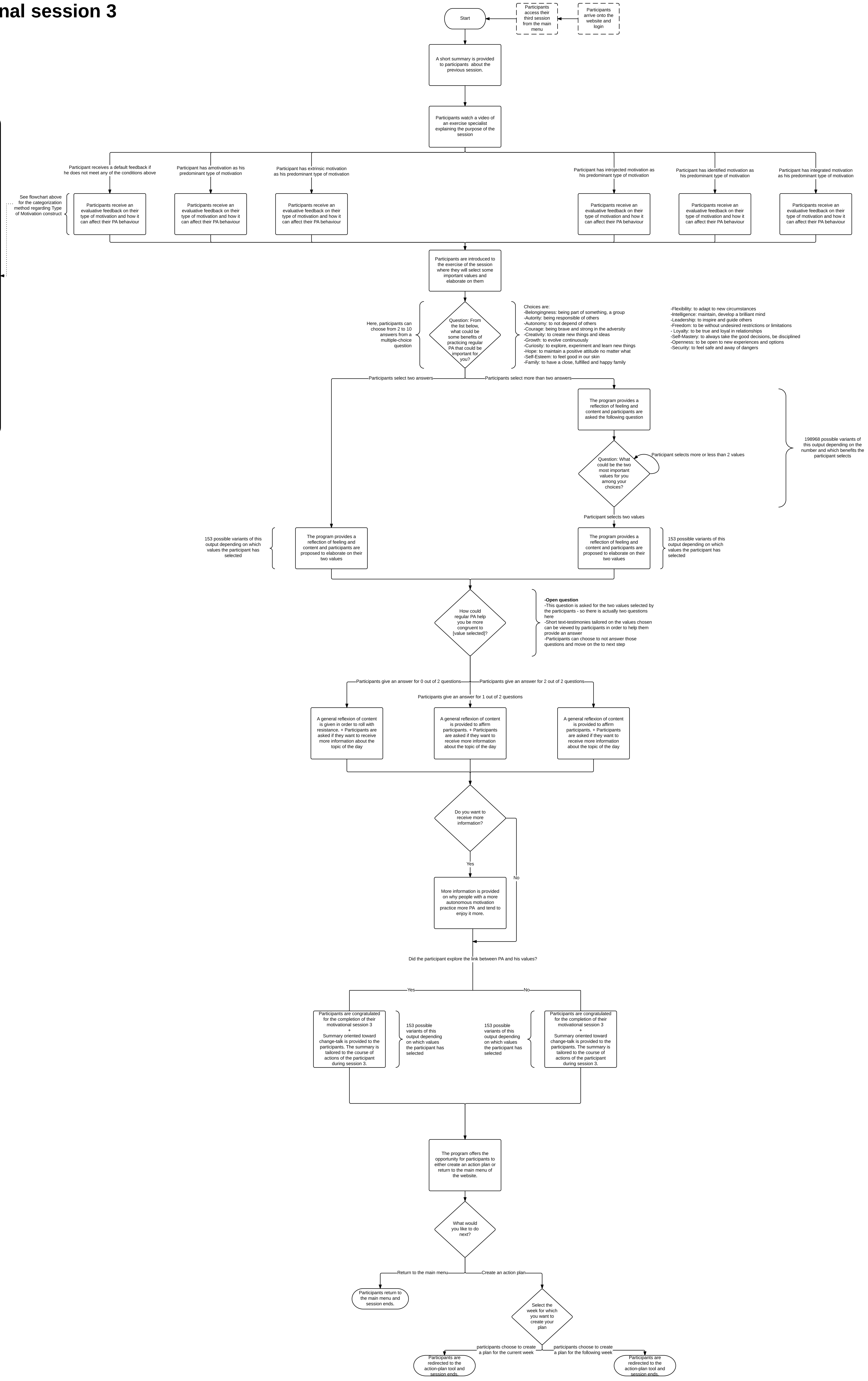

Total number of possible variations of the session: 14336712
Total number of possible variations of the session when gender is considered (approximation): 28673424

Tailored motivational session 4

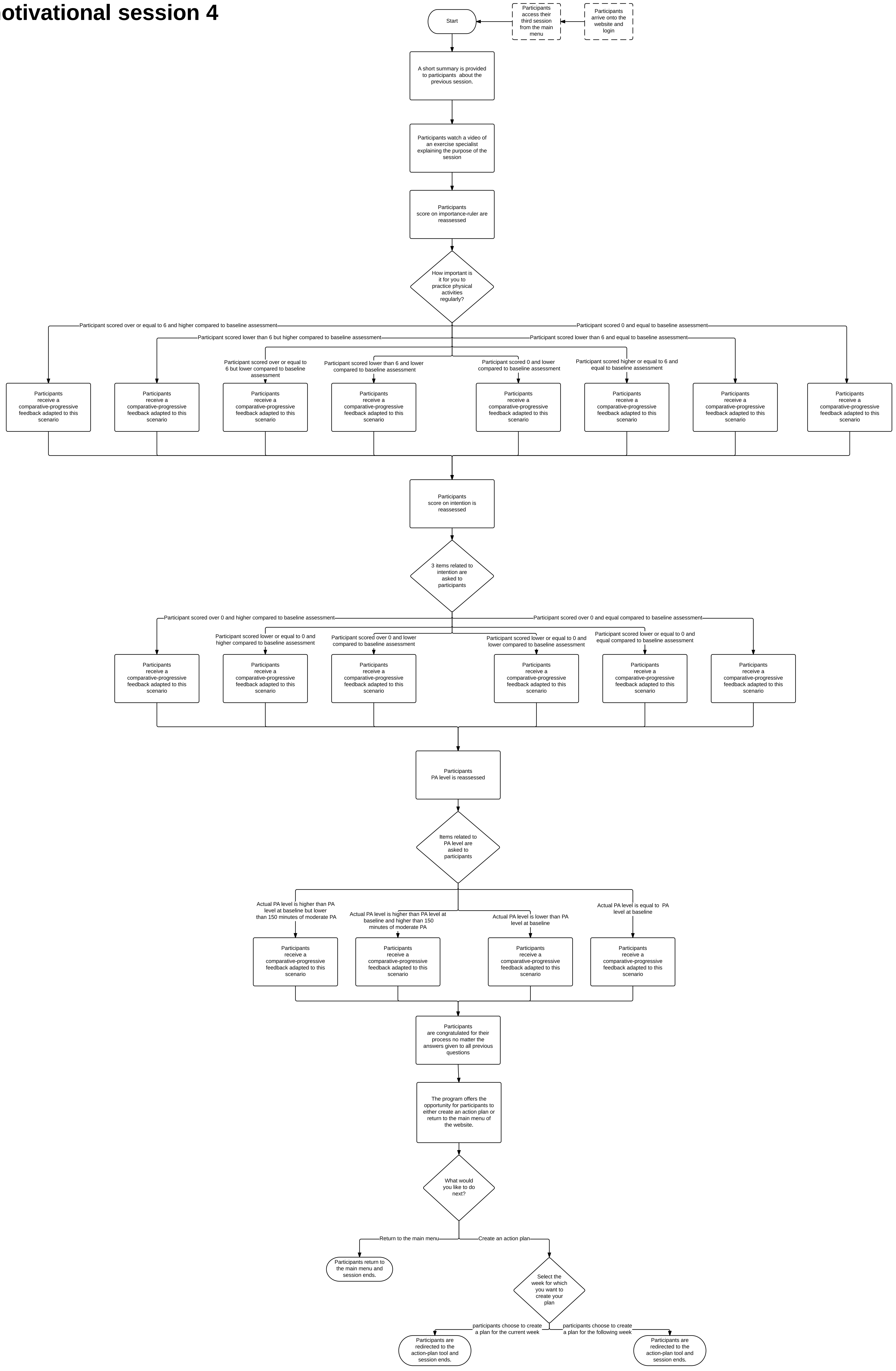

Total number of possible variations of the session: 576
Total number of possible variations of the session when gender is included (approximation): 1152

Tailored motivational session 5

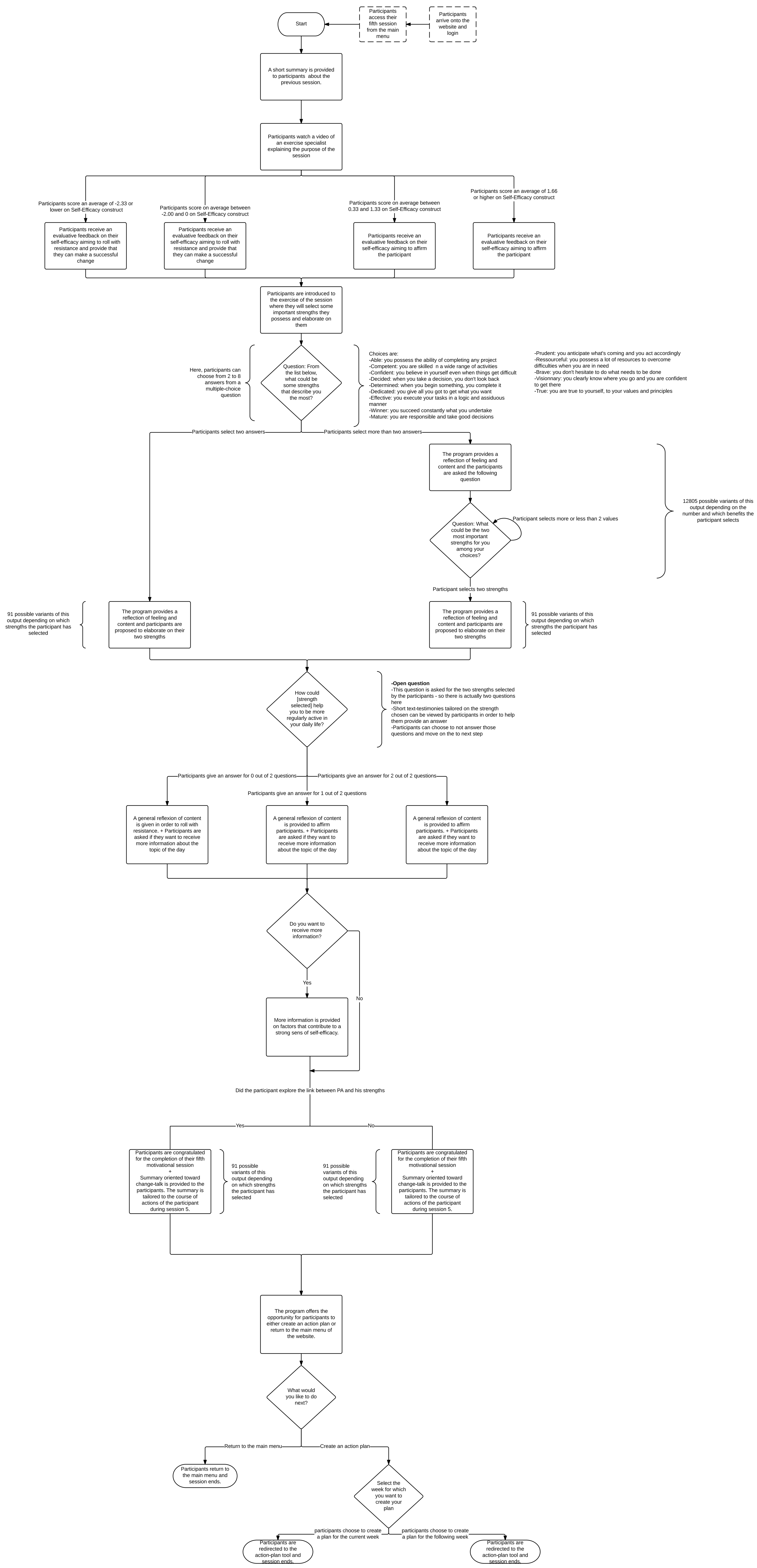

Total number of possible variations of the session: 619008
Total number of possible variations of the session when gender is considered (approximation): 1238016

Tailored motivational session 6

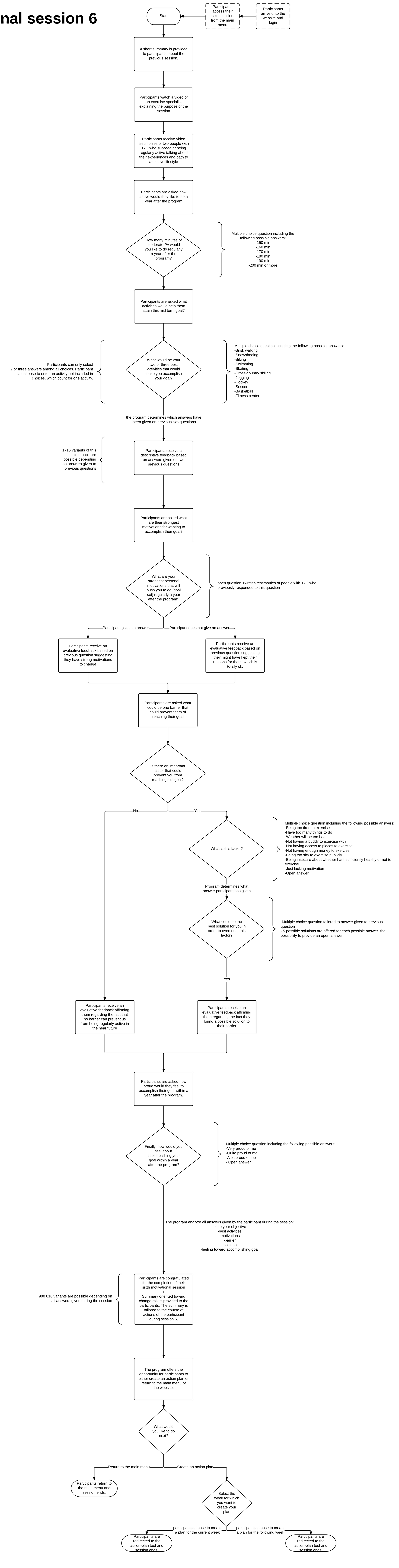

Total number of possible variations of the session: 2965248
Total number of possible variations of the session when gender is considered (approximation): 5930496

Tailored motivational session 7

Note: this session uses all previous sessions in order to provide a summary of the intervention to the participant.

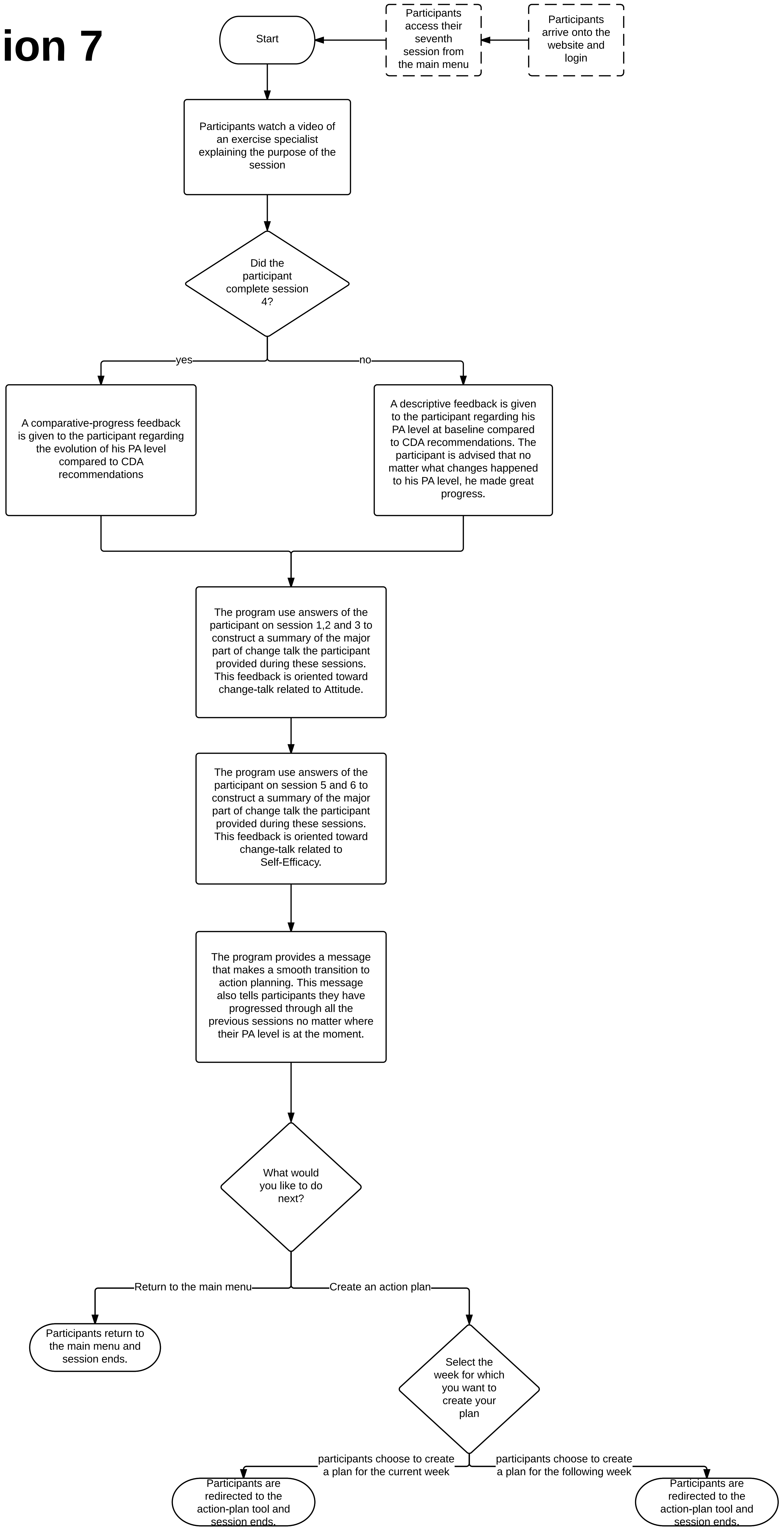

Total number of possible variations of the session: 1571851008

Total number of possible variations of the session when gender is included (approximation): 3143702016

Tailored motivational session 8

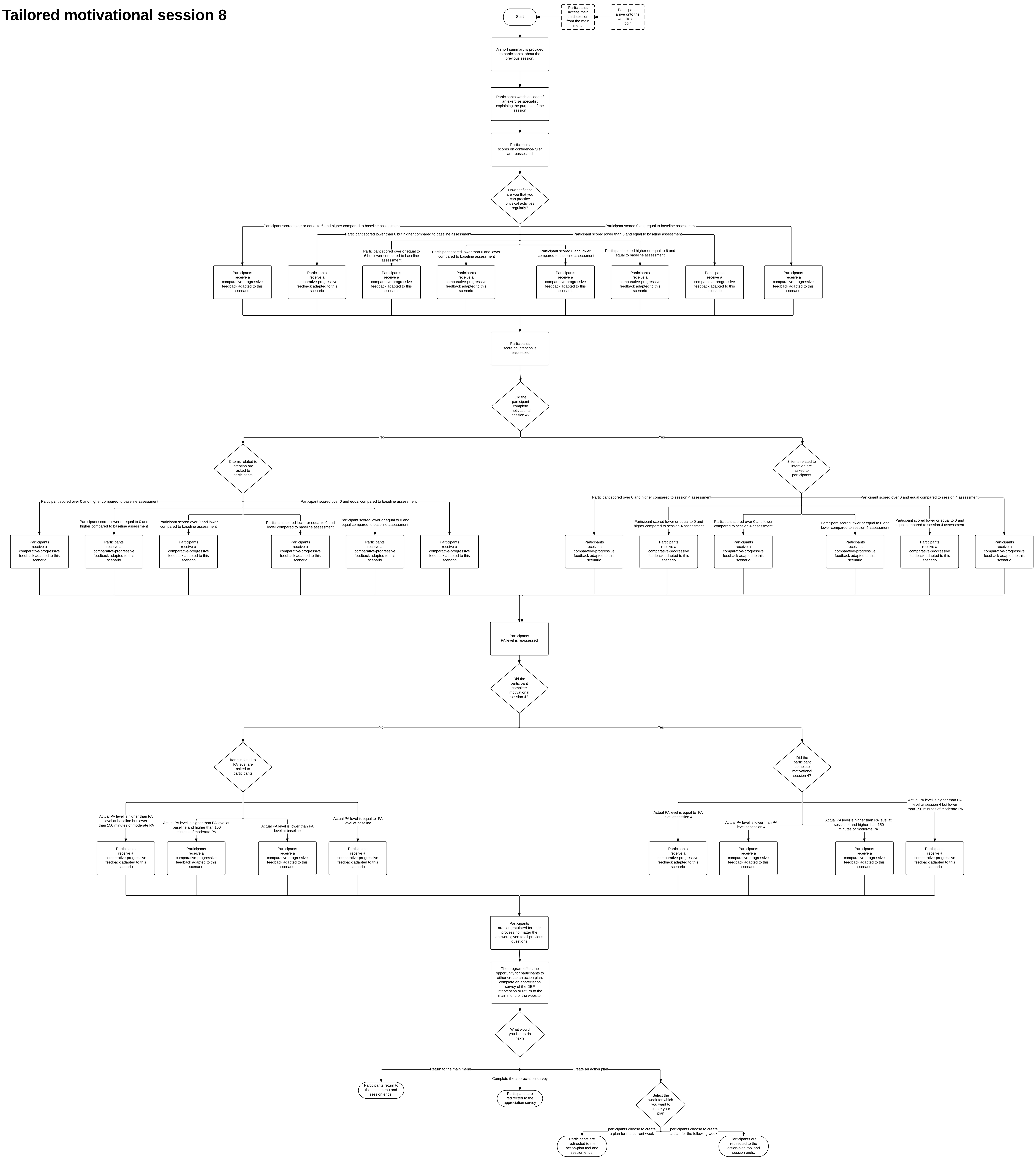

Total number of possible variations of the session: 1536
Total number of possible variations of the session when gender is included (approximation): 3072

Action plan session

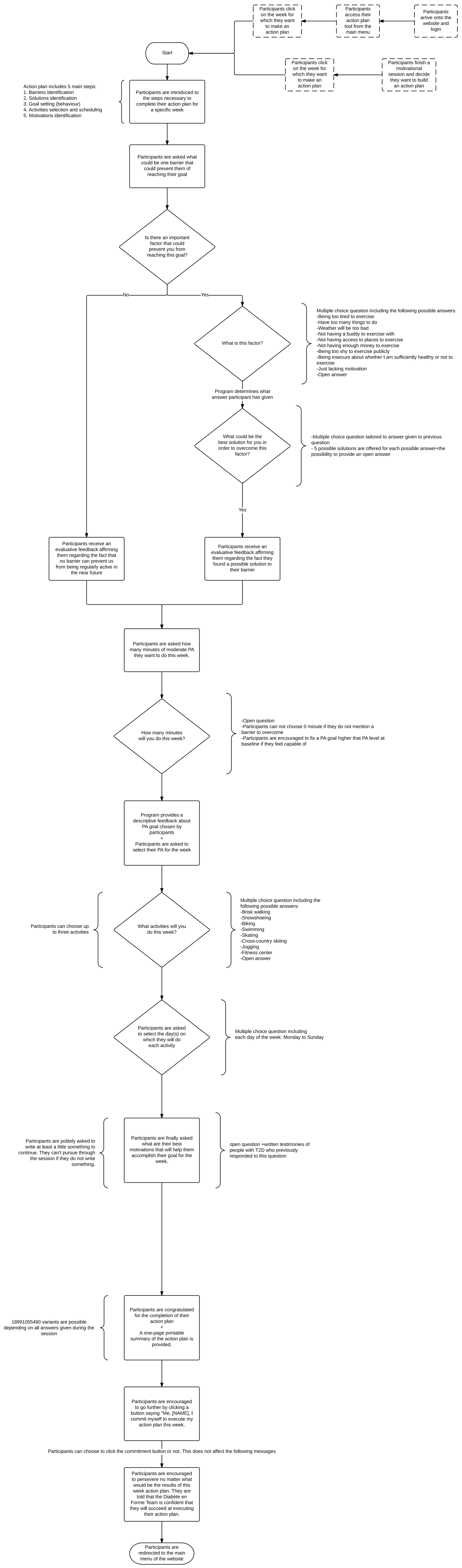

Total number of possible variations of the session: 18991055490
Total number of possible variations of the session when gender is considered (approximation): 37982110980
