# Supplementary material for: Development of a Fully Automated, Web-Based, Tailored Intervention Promoting Regular Physical Activity Among Insufficiently Active Adults With Type 2 Diabetes: Integrating the I-Change Model, Self-Determination Theory, and Motivational Interviewing Components
Source: JMIR Res Protoc. 2015 Feb 17;4(1):e25. doi: 10.2196/resprot.4099 (PMC4376153; doi:10.2196/resprot.4099)
Supplement: Supplementary file 3 [file resprot_v4i1e25_app3.pdf]

SAMPLE OF THE MESSAGE BOOKLET FOR THE *DIABÈTE EN FORME* WEB-BASED TAILORING INTERVENTION

8th of April 2014

Non-official document

Messages have not been translated from French to English.

It is not for readers to see what the official content of the intervention is.

The purpose of this document is to give an overview of the shape and length of the message booklet.

For questions:

michel.moreau-lapointe.1@ulaval.ca

SECTIONS

First motivational session 4

Second motivational session 18

Third motivational session 36

Fifth motivational session 54

Sixth motivation session 70

Seventh motivational session 86

Action plan tool 97

Emails 109

Messages for introduction videos 121

Messages implemented by AlphaZero 132

First motivational session

Page 1 - Intro		
**Definition**	**Coding variables of each message**	**Content of the message**
Introduction	S1.INTRO	Bienvenue à votre première séance interactive [PRENOM]! Le début d'une belle aventure!<br><br>Message de la vidéo : <u>à écrire</u>
Objectifs de la séance	S1.OBJ	OBJECTIFS DE LA SÉANCE :<br><br>Prendre conscience de votre niveau d'activité physique actuel par rapport aux recommandations de l'Association Canadienne du Diabète<br>Explorer les conséquences de santé possibles de votre niveau d'activité physique actuel<br>Recevoir de l'information personnalisée sur les éléments 1 et 2<br>Commencer à planifier votre pratique d'activités physiques (optionel)
Durée de la séance	S1.LEN	DURÉE DE LA SÉANCE :<br>10 à 15 minutes maximum
Transition	S1.P1TRANS	Lorsque vous le voudrez [PRENOM], cliquez sur continuer pour commencer votre première séance!
Page 2 – Niveau d'activité physique actuel		
Introduction au niveau d'activité physique du participant	S1.PA_INTRO	**Votre niveau d'activité physique actuel**<br><br>Le graphique ci-dessous [PRENOM] représente votre niveau d'activité physique. Votre niveau est comparé avec les recommandations de l'Association Canadienne du Diabète en matière d'activités physiqueS.<br><br>Petit rappel<br>Selon l'Association Canadienne du Diabète, **pratiquer de l'activité physique régulièrement** signifie cumuler au moins **150 minutes d'activités physiques par semaine à intensité modérée.** Pour s'assurer qu'une activité compte comme une activité physique à intensité modérée, trois critères peuvent servir de repère :<br><br>1. Votre fréquence cardiaque augmente légèrement<br>2. Vous ne pouvez pas prononcer un phrase complète facilement, car vous sentez que vous êtes essoufflé(e)<br>3. L'activité physique doit durer au moins 10 minutes consécutiveS.<br><br>La marche rapide, le vélo, la nage à un rythme modérée, le tennis, le badminton sont tous des exemples d'activités dans lesquelles il est possible d'atteindre une intensité modérée.<br><br><br><br>Voyons maintenant où se situe votre niveau actuel d'activités

		physiques [PRENOM] :<br><br>(Graphique comparant le niveau du participant avec les recommandations) - [APREC]
Si le participant fait 0 minute HOMME	S1.PA_ZEROM	Présentement, il semble que vous ne pratiquez aucune activité physique [PRENOM]. Soyez assuré que vous n'êtes pas le seul à ne pas atteindre les recommandations canadiennes en matière d'activités physiqueS.Au Québec, près de 7 personnes atteintes du diabète sur 10 ne pratiquent pas suffisamment d'activités physiqueS.D'un autre côté par contre, 300 000 personnes réussissent à pratiquer des activités physiques régulièrement et atteignent les recommandations de l'Association Canadienne du Diabète.<br><br>Maintenant, plusieurs raisons possibles pourraient expliquer le fait que vous ne fassiez aucune activité physique pour l'instant. Nous explorerons ensemble plusieurs d'entre elles au cours des prochaines semaineS.D'abord, une d'entre elles pourrait être que vous soyez peu informé sur les conséquences potentielles d'un point de vue « santé » reliées à une pratique d'activité physique insuffisante face aux recommandationS.Avoir une idée claire de ces conséquences pourrait, [PRENOM], agir comme une source de motivation et vous aider à atteindre le niveau recommandé d'activités physiqueS.Si vous désirez approfondir ce sujet, il nous ferait plaisir d'en discuter avec vous dans les 10 prochaines minuteS5.<br><br>Encore une fois, vous restez votre propre expert [PRENOM]. Même après toutes les discussions que nous aurons ensemble, nous tenons à vous mentionner que vous êtes toujours libre de faire les choix que vous considérez bons pour vouS.Nous tenons à vous supporter le plus positivement possible et nous n'irons jamais à l'encontre de vos choix.
Si le participant fait 0 minute FEMME	S1.PA_ZEROF	Présentement, il semble que vous ne pratiquez aucune activité physique [PRENOM]. Soyez assurée que vous n'êtes pas la seule à ne pas atteindre les recommandations canadiennes en matière d'activités physiqueS.Au Québec, près de 7 personnes atteintes du diabète sur 10 ne pratiquent pas suffisamment d'activités physiqueS.D'un autre côté par contre, 300 000 personnes de tout âge atteintes du diabète réussissent à pratiquer des activités physiques régulièrement et atteignent les recommandations de l'Association Canadienne du Diabète.<br><br>Maintenant, plusieurs raisons possibles pourraient expliquer le fait que vous ne fassiez aucune activité physique pour l'instant. Nous explorerons ensemble plusieurs d'entre elles au cours des

		prochaines semaineS.D’abord, une d'entre elles pourrait être que vous soyez peu informé sur les conséquences potentielles d’un point de vue « santé » reliées à une pratique d'activité physique insuffisante face aux recommandationS.Avoir une idée claire de ces conséquences pourrait, [PRENOM], agir comme une source de motivation et vous aider à atteindre le niveau recommandé d’activités physiqueS.Si vous désirez approfondir ce sujet, il nous ferait plaisir d'en discuter avec vous dans les 10 prochaines minuteS5.<br><br>Encore une fois, vous restez votre propre experte [PRENOM]. Même après toutes les discussions que nous aurons ensemble, nous tenons à vous mentionner que vous êtes toujours libre de faire les choix que vous considérez bons pour vouS.Nous tenons à vous supporter le plus positivement possible et nous n'irons jamais à l'encontre de vos choix.
Si le participant fait moins de 50 minutes FEMME	S1.PA_50F	Présentement, il semble que vous pratiquez déjà un peu d'activités physiques [PRENOM]. [F.APTOT] minutes, c'est un pas dans la bonne directioS6.Soyez assuré que vous n’êtes pas la seule à ne pas atteindre les recommandations canadiennes en matière d'activités physiqueS.Au Québec, près de 7 personnes atteintes du diabète sur 10 ne pratiquent pas suffisamment d'activités physiqueS.D'un autre côté par contre, 300 000 personnes réussissent à pratiquer des activités physiques régulièrement et atteignent les recommandations de l'Association Canadienne du Diabète.<br><br>Maintenant, plusieurs raisons possibles pourraient expliquer le fait que vous fassiez moins d'activités physiques que les recommandationS.Nous explorerons ensemble plusieurs d’entre elles au cours des prochaines semaineS.D’abord, une d'entre elles pourrait être que vous soyez peu informé sur les conséquences potentielles d’un point de vue « santé » reliées à une pratique d'activité physique insuffisante face aux recommandationS.Avoir une idée claire de ces conséquences pourrait, [PRENOM], agir comme une source de motivation et vous aider à atteindre le niveau recommandé d’activités physiqueS.Si vous désirez approfondir ce sujet, il nous ferait plaisir d'en discuter avec vous dans les 10 prochaines minuteS5.<br><br>Encore une fois, vous restez votre propre experte [PRENOM]. Même après toutes les discussions que nous aurons ensemble, nous tenons à vous mentionner que vous êtes toujours libre de faire les choix que vous considérez bons pour vouS.Nous tenons à vous supporter le plus positivement possible et nous n'irons jamais à l'encontre de vos choix.
Si le participant fait moins de 50 minutes HOMME	S1.PA_50M	Présentement, il semble que vous pratiquez déjà un peu d'activités physiques [PRENOM]. [F.APTOT] minutes, c'est un pas dans la bonne direction Soyez assuré(e) que vous n’êtes pas le seul à ne pas atteindre les recommandations canadiennes en matière d'activités physiqueS.Au Québec, près de 7 personnes atteintes du

		diabète sur 10 ne pratiquent pas suffisamment d'activités physiqueS.D'un autre côté par contre, 300 000 personnes réussissent à pratiquer des activités physiques régulièrement et atteignent les recommandations de l'Association Canadienne du Diabète.<br><br>Maintenant, plusieurs raisons possibles pourraient expliquer le fait que vous fassiez moins d'activités physiques que les recommandationS.Nous explorerons ensemble plusieurs d'entre elles au cours des prochaines semaineS.D'abord, une d'entre elles pourrait être que vous soyez peu informé sur les conséquences potentielles d'un point de vue « santé » reliées à une pratique d'activité physique insuffisante face aux recommandationS.Avoir une idée claire de ces conséquences pourrait, [PRENOM], agir comme une source de motivation et vous aider à atteindre le niveau recommandé d'activités physiqueS.Si vous désirez approfondir ce sujet, il nous ferait plaisir d'en discuter avec vous dans les 10 prochaines minuteS5.<br><br>Encore une fois, vous restez votre propre expert [PRENOM]. Même après toutes les discussions que nous aurons ensemble, nous tenons à vous mentionner que vous êtes toujours libre de faire les choix que vous considérez bons pour vouS.Nous tenons à vous supporter le plus positivement possible et nous n'irons jamais à l'encontre de vos choix.
Si le participant fait moins de 100 minutes HOMME	S1.PA_100M	Présentement, il semble que vous pratiquez déjà un peu d'activités physiques [PRENOM]. [F.APTOT] minutes, voilà déjà une bonne habitude. Soyez assuré que vous n'êtes pas le seul à ne pas atteindre les recommandations canadiennes en matière d'activités physiqueS.Au Québec, près de 7 personnes atteintes du diabète sur 10 ne pratiquent pas suffisamment d'activités physiqueS.D'un autre côté par contre, 300 000 personnes réussissent à pratiquer des activités physiques régulièrement et atteignent les recommandations de l'Association Canadienne du Diabète. Si vous voulez être actif régulièrement, vous êtes sur le bon chemin!<br><br>Maintenant, plusieurs raisons possibles pourraient expliquer le fait que vous fassiez moins d'activités physiques que les recommandationS.Nous explorerons ensemble plusieurs d'entre elles au cours des prochaines semaineS.D'abord, une d'entre elles pourrait être que vous soyez peu informé sur les conséquences potentielles d'un point de vue « santé » reliées à une pratique d'activité physique insuffisante face aux recommandationS.Avoir une idée claire de ces conséquences pourrait, [PRENOM], agir comme une source de motivation et vous aider à atteindre le niveau recommandé d'activités physiqueS.Si vous désirez approfondir ce sujet, il nous ferait plaisir d'en discuter avec vous dans les 10 prochaines minuteS5.<br><br>Encore une fois, vous restez votre propre expert [PRENOM]. Même après toutes les discussions que nous aurons ensemble, nous

		tenons à vous mentionner que vous êtes toujours libre de faire les choix que vous considérez bons pour vouS.Nous tenons à vous supporter le plus positivement possible et nous n'irons jamais à l'encontre de vos choix.
Si le participant fait moins de 100 minutes FEMME	S1.PA_100F	Présentement, il semble que vous pratiquez déjà un peu d'activités physiques [PRENOM]. [F.APTOT] minutes, voilà déjà une bonne habitude. Soyez assurée que vous n'êtes pas la seule à ne pas atteindre les recommandations canadiennes en matière d'activités physiqueS.Au Québec, près de 7 personnes atteintes du diabète sur 10 ne pratiquent pas suffisamment d'activités physiqueS.D'un autre côté par contre, 300 000 personnes réussissent à pratiquer des activités physiques régulièrement et atteignent les recommandations de l'Association Canadienne du Diabète. Si vous voulez être active régulièrement, vous êtes sur le bon chemin!<br><br>Maintenant, plusieurs raisons possibles pourraient expliquer le fait que vous fassiez moins d'activités physiques que les recommandationS.Nous explorerons ensemble plusieurs d'entre elles au cours des prochaines semaineS.D'abord, une d'entre elles pourrait être que vous soyez peu informé sur les conséquences potentielles d'un point de vue « santé » reliées à une pratique d'activité physique insuffisante face aux recommandationS.Avoir une idée claire de ces conséquences pourrait, [PRENOM], agir comme une source de motivation et vous aider à atteindre le niveau recommandé d'activités physiqueS.Si vous désirez approfondir ce sujet, il nous ferait plaisir d'en discuter avec vous dans les 10 prochaines minuteS5.<br><br>Encore une fois, vous restez votre propre experte [PRENOM]. Même après toutes les discussions que nous aurons ensemble, nous tenons à vous mentionner que vous êtes toujours libre de faire les choix que vous considérez bons pour vouS.Nous tenons à vous supporter le plus positivement possible et nous n'irons jamais à l'encontre de vos choix.
Si le participant fait plus de 100 minutes HOMME	S1.PA_100PM	Présentement, il semble que vous pratiquez déjà souvent des activités physiques [PRENOM]. [F.APTOT] minutes, vous y êtes presque! Soyez assuré que vous n'êtes pas le seul à ne pas atteindre les recommandations canadiennes en matière d'activités physiqueS.Au Québec, près de 7 personnes atteintes du diabète sur 10 ne pratiquent pas suffisamment d'activités physiqueS.D'un autre côté par contre, 300 000 personnes réussissent à pratiquer des activités physiques régulièrement et atteignent les recommandations de l'Association Canadienne du Diabète. Si vous voulez être actif régulièrement, vous êtes définitivement sur le bon chemin!<br><br>Maintenant, plusieurs raisons possibles pourraient expliquer le fait que vous fassiez un peu moins d'activités physiques que les recommandationS.Nous explorerons ensemble plusieurs d'entre elles au cours des prochaines semaineS.D'abord, une d'entre elles pourrait être que vous soyez peu informé sur les conséquences

		potentielles d'un point de vue « santé » reliées à une pratique d'activité physique insuffisante face aux recommandationS.Avoir une idée claire de ces conséquences pourrait, [PRENOM], agir comme une source de motivation et vous aider à atteindre le niveau recommandé d'activités physiqueS.Si vous désirez approfondir ce sujet, il nous ferait plaisir d'en discuter avec vous dans les 10 prochaines minuteS5.<br><br>Encore une fois, vous restez votre propre expert [PRENOM]. Même après toutes les discussions que nous aurons ensemble, nous tenons à vous mentionner que vous êtes toujours libre de faire les choix que vous considérez bons pour vouS.Nous tenons à vous supporter le plus positivement possible et nous n'irons jamais à l'encontre de vos choix.
Si le participant fait plus de 100 minutes FEMME	S1.PA_100PF	Présentement, il semble que vous pratiquez déjà souvent des activités physiques [PRENOM]. [F.APTOT] minutes, vous y êtes presque! C'est excellent! Soyez assurée que vous n'êtes pas la seule à ne pas atteindre les recommandations canadiennes en matière d'activités physiqueS.Au Québec, près de 7 personnes atteintes du diabète sur 10 ne pratiquent pas suffisamment d'activités physiqueS.D'un autre côté par contre, 300 000 personnes réussissent à pratiquer des activités physiques régulièrement et atteignent les recommandations de l'Association Canadienne du Diabète.Si vous voulez être active régulièrement, vous êtes définitivement sur le bon chemin!<br><br>Maintenant, plusieurs raisons possibles pourraient expliquer le fait que vous fassiez un peu moins d'activités physiques que les recommandationS.Nous explorerons ensemble plusieurs d'entre elles au cours des prochaines semaineS.D'abord, une d'entre elles pourrait être que vous soyez peu informé sur les conséquences potentielles d'un point de vue « santé » reliées à une pratique d'activité physique insuffisante face aux recommandationS.Avoir une idée claire de ces conséquences pourrait, [PRENOM], agir comme une source de motivation et vous aider à atteindre le niveau recommandé d'activités physiqueS.Si vous désirez approfondir ce sujet, il nous ferait plaisir d'en discuter avec vous dans les 10 prochaines minuteS5.<br>Encore une fois, vous restez votre propre expert [PRENOM]. Même après toutes les discussions que nous aurons ensemble, nous tenons à vous mentionner que vous êtes toujours libre de faire les choix que vous considérez bons pour vouS.Nous tenons à vous supporter le plus positivement possible et nous n'irons jamais à l'encontre de vos choix.
Message transition	S1.P2TRANS	Cliquez sur continuer pour la suite [PRENOM]!
Page 3 – Question suite à l'information fournie (demander-fournir-demander)		
Question	S1.Q1	Avant d'aller plus loin<br><br>[PRENOM], nous venons tout juste de voir votre niveau actuel

		d'activité physique<br>Que pensez-vous de cette information?<br><br>Je pensais que mon niveau était plus élevé que ce que j'ai vu<br>Je pensais que mon niveau était moins élevé que ce que j'ai vu<br>Je pensais que j'étais à peu près à ce niveau<br>Je ne sais pas trop quoi penser<br><br>(graphique remontrant le niveau actuel du participant par rapport aux recommandations)
Page 4 – Reflet sur l'état du participant		
Le participant pensait qu'il était plus actif	S1.FQ1_1	D'accord, vous pensiez faire plus d'activités physiques [PRENOM]. Rappelez-vous qu'il s'agit d'une estimation, alors cela pourrait ne pas correspondre exactement à votre niveau actuel. D'un autre côté, cette estimation est assez juste la plupart du tempS5.
Le participant pensait qu'il était moins actif	S1.FQ1_2	D'accord, vous pensiez faire moins d'activités physiques [PRENOM]. Bonne nouvelle si vous en faites plus!
LE participant pensait qu'il était à peu près à ce niveau	S1.FQ1_3	Vous estimez bien votre niveau d'activités physiques [PRENOM]. Félicitations!
Le participant ne sait pas trop	S1.FQ1_4	Vous n'aviez pas vraiment d'idée quant au niveau d'activité physique auquel vous êtes actuellement. Vous pouvez maintenant en avoir une meilleure idée [PRENOM]. Nous sommes heureux de vous en avoir fait part.
Transition	S1.P4TRANS	Maintenant, si vous êtes d'accord, discutons ensemble des conséquences possibles d'un point de vue « santé » de votre niveau actuel d'activités physiqueS5.
Page 5 – Question évocatrices sur les conséquences possibles du niveau actuel d'activité physique du participant		
Introduction et question évocatrice	S1.Q2	[PRENOM], nous venons de voir ensemble que votre niveau actuel d'activités physiques n'atteint pas les recommandationS5.<br><br>Les personnes atteintes du diabète de type 2 qui ne sont pas suffisamment actives s'exposent à certaines conséquences pour leur santé. Les études scientifiques sont claires là-dessuS.Il est possible que ces conséquences puissent vous créer de l'inquiétude.<br><br>Parmi la liste ci-dessous, y a-t-il des conséquences qui, à vos yeux, pourraient vous inquiéter [PRENOM] ?<br>(vous pouvez sélectionner de 0 à 8 réponses)<br><br>1- Avoir des problèmes cardiaques<br>2- Avoir des problèmes aux yeux<br>3- Avoir moins d'énergie au quotidien<br>4- Avoir des problèmes aux pieds<br>5- Avoir une moins bonne santé en général<br>6- Avoir des problèmes de tension artérielle<br>7- Avoir des problèmes aux reins

		8- Avoir des risques de vivre moins longtemps
Question évocatrice ouverte<br><br>Et transition	S1.O_Q2	**Pourrait-il y avoir d'autre(s) conséquence(s) qui pourraient vous inquiéter [PRENOM]?**<br>Si tel est le cas, nous vous encourageons à inscrire celle(s)-ci dans l'espace libre qui suit.<br><br>(espace de réponse)<br><br>Ce que d'autres personnes atteintes du diabète ont répondu:<br><br>"***Prendre plus de poids, ça pourrait être quelque chose qui m'inquiète.***"<br><br>Lorsque vous le voudrez, allez à la page suivante.
	Page 6 – Reflet sur les conséquences qui sont importantes pour le participant	
Si 2 conséquences sélectionnées – FEMME	S1.FF2_Q2	D'accord [PRENOM], parmi les choix proposés, [F.FBRISK] semblent être les conséquences qui vous inquiètent davantage.<br><br>Si vous le voulez, explorons ensemble comment, pour vous, ces conséquences pourraient avoir un impact dans votre quotidieS6.<br><br>Continuez pour la suite [PRENOM].
Si 2 conséquences sélectionnées – HOMME	S1.MF2_Q2	D'accord [PRENOM], parmi les choix proposés, [F.FBRISK] semblent être les conséquences qui vous inquiètent davantage.<br><br>Si vous le voulez, explorons ensemble comment, pour vous, ces conséquences pourraient avoir un impact concret dans votre quotidieS6.<br><br>Continuez pour la suite [PRENOM].
Si 3 conséquences sélectionnées – FEMME	S1.FF3_Q2	D'accord [PRENOM], parmi les choix proposés, vous voyez beaucoup de conséquences qui vous inquiètent. Tentons de voir ensemble si une ou deux conséquences parmi celles sélectionnées pourraient être plus importantes que les autreS.Après quoi, nous discuterons plus en profondeur de celles-là.
Si 3 conséquences sélectionnées – HOMME	S1. MF3_Q2	D'accord [PRENOM], parmi les choix proposés, vous voyez beaucoup de conséquences qui sont importantes pour vouS.Tentons de voir ensemble si une ou deux conséquences parmi celles sélectionnées pourraient être plus importantes que les autreS.Après quoi, nous discuterons plus en profondeur de celles-là.
Question si le participant sélectionne 3 conséquences ou plus	S1.Q3	Quelles seraient donc <u>une</u> ou <u>deux</u> conséquences encore plus importantes que les autres pour vous [PRENOM]?<br>(sélectionnez 1 ou 2 réponses seulement)
	Page 7 – Reflet sur les conséquences les plus importantes chez le participant ayant sélectionné 3 conséquences et plus au départ	
	Note : cette page est sautée pour les participants ayant moins de 3 conséquences sélectionnées	

Reflet pour le participant qui a choisi 2 conséquences HOMME	S1.MF2_Q3	D'accord [PRENOM]. Vous voyez plusieurs conséquences qui vous inquiètent, et [F.FB3PRISK] semblent être les conséquences qui vous inquiètent plus que toutes les autreS.Si vous le voulez, explorons maintenant ensemble comment, pour vous, ces conséquences pourraient avoir un impact dans votre quotidieS6.<br><br>Continuez pour la suite [PRENOM].
Reflet pour le participant qui a choisi 2 conséquences FEMME	S1.FF2_Q3	D'accord [PRENOM]. Vous voyez plusieurs conséquences qui vous inquiètent, et [F.FB3PRISK] semblent être les conséquences qui vous inquiètent plus que toutes les autreS.Si vous le voulez, explorons maintenant ensemble comment, pour vous, ces conséquences pourraient avoir un impact dans votre quotidieS6.<br><br>Continuez pour la suite [PRENOM].
	Page 8 – Questions d'élaborations sur les conséquences choisies	
	Note : cette page est sautée pour les participants ayant sélectionné 0 conséquence	
Introduction pour 2 conséquences sélectionnées	S1.Q4_I	[PRENOM], vous venez de mentionner qu' [F.FBRISK] étaient des conséquences importantes pour vouS5.<br><br>Maintenant, avoir une idée claire de l'impact que ces conséquences pourraient avoir dans votre vie pourrait vous permettre de prendre une décision plus éclairée par rapport à votre pratique d'activités physiques future. Aussi, si tel est votre désir, cela pourrait vous permettre de vous bâtir une motivation encore plus solide en vue de faire plus d'activités physiqueS5.
Questions conséquentes avec le nombre de conséquences choisis	S1.Q4	1. En une phrase [PRENOM], comment le fait d'[F.ELAB1] pourrait influencer votre quotidien?<br>(espace de réponse)<br><br>Ce que d'autres personnes atteintes du diabète ont répondu :<br><br>*" **[EXELAB1]** "*<br><br>2. En une phrase [PRENOM], comment le fait d'[F.ELAB2] pourrait influencer votre quotidien?<br>(espace de réponse)<br><br>Ce que d'autres personnes atteintes du diabète ont répondu :<br><br>*" **[EXELAB2]** "*<br><br>1. En une phrase [PRENOM], comment le fait d'[F.ELAB3P1] pourrait influencer votre quotidien?<br>(espace de réponse)<br><br>Ce que d'autres personnes atteintes du diabète ont répondu :<br><br>*" **[EXELAB3P1]** "*

		2. En une phrase [PRENOM], comment le fait d'[F.ELAB3P2] pourrait influencer quotidien?<br>(espace de réponse)<br><br>Ce que d'autres personnes atteintes du diabète ont répondu :<br><br>" ***[EXELAB3P2]*** "
Message de transition	S1.P8TRANS	Lorsque vous le voudrez, cliquez sur continuer pour la suite.
Page 9 – Reflet sur l'élaboration du participant/Question afin de demander au participant s'il veut recevoir de l'information		
Note : si le participant a sélectionné 0 conséquence, cette page est sautée		
Reflet élaboration 0/2 HOMME	S1.Q4_02M	Bien! Vous avez peut-être seulement réfléchi à quelque chose [PRENOM]. Cela dit, vous semblez convaincu qu'[F.FBRISK] sont des aspects qui vous préoccupent.
Reflet élaboration 0/2 FEMME	S1. Q4_02F	Bien! Vous avez peut-être seulement réfléchi à quelque chose [PRENOM]. Cela dit, vous semblez convaincue qu'[F.FBRISK] sont des aspects qui vous préoccupent.
Reflet élaboration 1/2	S1. Q4_12	Merci de partager [PRENOM]. Vous avez fourni une réponse pour une des conséquences importantes pour vouS.Il semble que vous voyez concrètement comment le fait rester à votre niveau actuel d'activités physiques pourrait avoir un impact dans votre quotidieS6.
Reflet élaboration 2/2	S1. Q4_22M	Merci de partager [PRENOM]. Vous avez fourni une réponse pour les deux conséquences qui sont les plus importantes pour vouS.Il semble que vous voyez concrètement comment le fait d'[F.FBRISK] pourrait avoir un impact dans votre quotidieS6.
Question pour demander de l'information pour les participants voyant deux conséquences plus importantes	S1.Q5	Avant de conclure et de résumer la séance [PRENOM], nous pensons avoir de l'information qui pourrait vous intéresser à propos des conséquences qui vous inquiètent. Aussi, si cela vous intéresse, nous aimerions vous partager de l'information sur les conséquences liées à un manque d'activités physiques de manière générale.<br><br>Aimeriez-vous recevoir ces informations [PRENOM]?<br>(oui/non)
Page 10 – Information fournie au participant		
Note : cette page est sautée si le participant ne veut pas d'information		
Message d'introduction et d'information générale s'adressant aux participants ayant	S1.INFO_INT	Excellent [PRENOM]! Voici de l'information spécialement construite pour vouS5.<br><br>D'abord, une chose sûre est qu'un diabète mal contrôlé peut générer plusieurs complications à moyen et long terme. Tous les problèmes et complications qui pourraient survenir en raison de votre diabète sont liés à la gestion de votre glycémie, c'est-à-dire du

sélectionné au-delà de 0 conséquence		taux de sucre dans votre sang. Ainsi, si votre diabète et votre glycémie sont mal contrôlés, les conséquences dont nous avons discuté dans cette séance pourraient potentiellement survenir.
Message s'adressant aux participants ayant sélectionné la conséquence 1<br>1- Avoir des problèmes cardiaques	S1.INFO_1	[F.ELAB1] est une conséquence importante pour vous, et il s'agit d'une possibilité bien réelle lorsqu'une personne est atteinte du diabète. En ce moment même, de nombreuses personnes atteintes du diabète de type 2 possèdent ce type de problème. Selon une étude scientifique, les personnes atteintes du diabète de type 2 qui ne pratiquent pas suffisamment d'activités physiques ont davantage de chances de voir survenir cette complication comparativement à ceux qui pratiquent régulièrement des activités physiqueS. Précisément, pratiquer régulièrement des activités physiques peut permettre de retarder significativement ce problème de santé, ou alors de l'éviter complètement.
Message s'adressant aux participants ayant sélectionné la conséquence 2<br>2- Avoir des problèmes aux yeux	S1.INFO_INFO2	[F.ELAB1] est une conséquence importante pour vous, et il s'agit d'une des conséquences les plus connues pour les personnes atteintes du diabète. Des maladies des yeux comme la cataracte, le glaucome ou simplement une perte progressive de la vue sont des problèmes fréquents chez les personnes atteintes du diabète de type 2. Il est cependant possible de réduire vos chances d'avoir ce problème en prenant soin de bien contrôler votre diabète et votre glycémie. Comme mentionné plus tôt, pratiquer régulièrement des activités physiques fait partie d'un des moyens de contrôler votre glycémie et votre diabète. Ainsi, vous pourriez éviter ce problème, ou alors empêcher qu'il s'aggrave en augmentant votre pratique d'activités physiqueS5.
Message s'adressant aux participants ayant sélectionné la conséquence 3<br>3- Avoir moins d'énergie au quotidien	S1.INFO_3	[F.ELAB1] est une conséquence importante pour vous, et il s'agit d'un problème commun pour les personnes atteintes du diabète. En effet, une mauvaise gestion de votre diabète peut engendrer une mauvaise circulation sanguine entraînant ainsi une baisse d'énergie constante. Pour la plupart des personnes qui le vivent, cela est très dérangeant que ne pas avoir suffisamment d'énergie pour entreprendre des activités, qui normalement, paraissent simples à réaliser. Pratiquer régulièrement des activités physiques pourrait être un des meilleurs moyens, sinon le meilleur, d'augmenter son niveau d'énergie au quotidieS6.
Message s'adressant aux participants ayant sélectionné la conséquence 4<br>4- Avoir des problèmes aux pieds	S1.INFO_4	[F.ELAB1] est une conséquence importante pour vous, et il s'agit d'un problème commun pour les personnes atteintes du diabète. En effet, une mauvaise gestion de votre diabète peut engendrer une mauvaise circulation sanguine au niveau des piedS.Cela peut vous rendre ainsi plus susceptible face aux infections et à la perte de sensibilité au niveau des piedS. Encore une fois, pratiquer régulièrement des activités physiques aide à contrôler efficacement votre diabète et votre glycémie. Par le fait même, vous pourriez ainsi diminuer significativement vos chances ou alors éviter vos problèmes aux piedS.
Message s'adressant aux participants ayant	S1.INFO_5	[F.ELAB1] est quelque chose qui vous préoccupe [PRENOM]. Étant donné que vous avez choisi cette conséquence, il est possible que vous vous préoccupiez de l'ensemble des conséquences qui vous ont été proposéeS.En fait, toutes les conséquences mentionnées

sélectionné la conséquence 5<br>5- Avoir une moins bonne santé en général		précédemment proviennent de la même cause : une mauvaise gestion de votre diabète et de votre glycémie. En décidant de pratiquer des activités physiques régulièrement, il vous serait ainsi possible de réduire fortement vos chances d'avoir ces nombreux problèmeS.L'activité physique est un des meilleurs médicaments pour maintenir une bonne santé globale.
Message s'adressant aux participants ayant sélectionné la conséquence 6<br>6- Avoir des problèmes de tension artérielle	S1.INFO_6	[F.ELAB1] est une conséquence importante pour vouS.Ce phénomène est commun pour les personnes atteintes du diabète et il s'apparente souvent aux problèmes cardiaqueS.Lorsque le diabète et la glycémie sont mal contrôlés, ce type de problème peut mener à des maladies importantes telles que les maladies coronariennes et les accidents vasculaires cérébraux. Les études démontrent que les personnes atteintes du diabète de type 2 qui pratiquent régulièrement des activités physiques sont moins susceptibles d'avoir ces types de maladies comparativement aux personnes atteintes faisant moins d'activités physiques [PRENOM].
Message s'adressant aux participants ayant sélectionné la conséquence 7<br>7- Avoir des problèmes aux reins	S1.INFO_7	[F.ELAB1] est une conséquence importante pour vous, et il s'agit d'une possibilité bien réelle pour les personnes atteintes du diabète. Communément appelé la néphropathie diabétique, ce problème aux reins peut générer des conséquences graves comprenant une diminution importante de votre espérance de vie lorsque votre diabète et votre glycémie sont mal contrôléS.Afin d'éviter ce problème ou de minimiser ses complications, prendre en charge votre diabète en pratiquant régulièrement des activités physiques pourrait être une excellente solutioS6.
Message s'adressant aux participants ayant sélectionné la conséquence 8<br>8- Avoir des risques de vivre moins longtemps	S1.INFO_8	[F.ELAB1] est une conséquence importante pour vous, et il s'agit d'une possibilité bien réelle pour les personnes atteintes du diabète. D'un autre côté, cela se produit surtout lorsque le diabète et la glycémie sont mal contrôléS.En effet, lorsque le diabète est bien contrôlé, les risques de vivre moins longtemps à cause du diabète diminuent fortement. Dans cette optique [PRENOM], une étude récente sur le diabète a démontré que les personnes qui pratiquent régulièrement des activités physiques ont plus de chance de vivre plus longtemps et en bonne santé comparativement aux personnes moins activeS.Intéressant non?
Message suivant l'information sur les conséquences précises	S1.INFO_SDT	Nous savons que vous êtes la personne la mieux placée pour juger de ce qui est bon pour vous [PRENOM]. Cela dit, le message que nous aimerions vous transmettre est simple : Pratiquer régulièrement des activités physiques pourrait être une des meilleures façons, pour vous, de contrôler votre diabète et éviter des problèmes de santé qui vous seraient importantS5.
Message de conclusion à la page d'information	S1.INFO_TRANS	Nous espérons que ces informations vous seront utileS.Nous sommes allés à l'essentiel et nous ne voulons pas vous bombarder d'information [PRENOM]. Avec ces informations, nous pensons que vous pourrez déjà prendre une décision plus éclairée concernant votre pratique d'activités physiques future.<br><br>La prochaine page indique la fin de la séance. En fonction des

		réponses que vous avez fournies, elle résumera votre séance d'aujourd'hui, et peut-être saura-t-elle encore une fois vous éclairer davantage.<br><br>Vous aurez également un aperçu de ce qui s'en vient dans la prochaine séance et les autres à venir!<br><br>Cliquez sur continuer pour avancer [PRENOM].
Page 11 – Résumé de la séance		
Remerciements	**S1.RES_INT**	**UN RÉSUMÉ, POUR VOUS**<br><br>Merci pour la discussion d'aujourd'hui [PRENOM]. Si vous nous le permettez, voici ce que nous comprenons de l'échange que nous avons eu avec vouS5.
Résumé niveau d'activité physique	S1.RES_AP	Votre niveau actuel d'activités physiques semble correspondre présentement à [F.APTOT] d'activités physiques modérées par semaine [PRENOM]. L'Association Canadienne du Diabète, de son côté, recommande 150 minutes d'activités physiques d'intensité modérée par semaine.
Reflet pour les personnes ayant sélectionné 2 conséquences FEMME	S1.RES_2	Cela dit, parmi les conséquences liées au fait d'être atteinte du diabète et insuffisamment active, il semble qu'[F.FBRISK] soient les conséquences qui vous inquiètent le plus [PRENOM].
Reflet pour les personnes ayant sélectionné 2 conséquences HOMME	S1.RES_2	Cela dit, parmi les conséquences liées au fait d'être atteint du diabète et insuffisamment actif, il semble qu'[F.FBRISK] soient les conséquences qui vous inquiètent le plus [PRENOM].
Reflet élaboration pour les participants ayant élaboré sur 0/2 conséquence	S1.RES_02	Vous avez ensuite eu la possibilité d'explorer en profondeur ces conséquences en vous demandant quel impact concret le fait d'[F.FBRISK] pourrait avoir dans votre quotidieS6.Bien que cela puisse aider à voir plus clairement ce que vous voudriez éviter, vous n'avez rien écrit à ce sujet. Sachez que nous respectons entièrement votre choix [PRENOM].
Reflet élaboration pour les participants ayant élaboré sur 1/2 conséquence	S1.RES_12	Vous avez ensuite eu la possibilité d'explorer en profondeur ces conséquences en vous demandant quel impact concret le fait d'[F.FBRISK] pourrait avoir dans votre quotidieS6.Vous avez approfondi votre réflexion pour une de vos conséquenceS.Voici donc, pour vous, comment le fait d' [NRISKCHOICE] pourrait avoir un impact concret : "[NELABCHOICE]". Connaître cet impact concret peut réellement vous aider à bâtir votre motivation à pratiquer davantage d'activités physiques [PRENOM].
Reflet élaboration pour les participants ayant élaboré sur 2/2 conséquence	S1.RES_22	Vous avez ensuite eu la possibilité d'explorer en profondeur ces conséquences en vous demandant quel impact concret le fait d'[F.FBRISK] pourrait avoir dans votre quotidieS6.Pour vous, le fait d'[F.ELAB1] pourrait avoir un impact concret de cette façon : "[Q.ELAB1]". De plus, le fait d'avoir [F.ELAB2] pourrait quant à lui avoir un impact concret de cette façon: "[Q.ELAB2]". Connaître ces

		impacts peut réellement vous aider à bâtir votre motivation à pratiquer davantage d'activités physiques [PRENOM].
Message supplémentaire si le participant a évoqué une conséquence ouverte importante pour lui	S1.RES_OP	Pour compléter votre réflexion, vous avez également mentionné une préoccupation personnelle qui peut sans aucun doute peser dans la balance et contribuer à votre motivatioS6.Voici votre préoccupation personnelle liée au fait que vous ne pratiquiez pas suffisamment d'activités physiques pour le moment: "[RISKOPEN]".
Message pour le participant n'ayant pas voulu recevoir de l'information	S1.RES_INFO0	Nous vous avons également proposé de l'information, mais vous en aviez assez pour aujourd'hui. Nous aimerons simplement vous dire une chose [PRENOM] : Pratiquer régulièrement des activités physiques pourrait être une des meilleures façons, pour vous, de contrôler votre diabète et éviter bien des conséquences qui pourraient peut-être s'avérer importantes pour vouS5.
Message pour le participant ayant voulu recevoir de l'information et a sélectionné 1 ou 2 conséquences et plus HOMME	S1.RES_INFO1M	Nous vous avons finalement proposé de l'information [PRENOM] et vous avez été ouverte à la recevoir. Vous savez maintenant le message important que nous voulions vous transmettre : Pratiquer régulièrement des activités physiques pourrait être une des meilleures façons, pour vous, de contrôler votre diabète et éviter les problèmes qui vous seraient les plus importants, c'est-à-dire [F.FBRISK].
Message pour le participant ayant voulu recevoir de l'information et a sélectionné 1 ou 2 conséquences et plus FEMME	S1.RES_INFO1F	Nous vous avons finalement proposé de l'information [PRENOM] et vous avez été ouvert à la recevoir. Vous savez maintenant le message important que nous voulions vous transmettre : Pratiquer régulièrement des activités physiques pourrait être une des meilleures façons, pour vous, de contrôler votre diabète et éviter les problèmes qui vous seraient les plus importants, c'est-à-dire [F.FBRISK].
Message complétant le résumé	S1.RES_TRANS	Voilà [PRENOM], ce dont nous avons discuté ensemble aujourd'hui. Vous avez progressé dans votre réflexion à savoir si vous pratiquerez davantage d'activités physiques dans les prochaines semaineS.Bravo.<br><br>Merci encore pour cet échange [PRENOM].<br><br>Cliquez sur continuer pour voir ce qui s'en vient pour vous dans les prochains jours!
Page 12 – **Prochaine séance et plan d'action**		
Message indiquant le dernier choix à faire du	S1.FINALM	Vers où tout cela vous mène?<br><br>Nous avons bien discuté aujourd'hui [PRENOM]. Avec ce que nous vivons ensemble, il pourrait être possible que votre motivation puisse changer positivement en vue de faire plus d'activités

participant HOMME		physiqueS.Si tel est le cas, **planifier votre pratique d'activités physiques** pour les prochains jours pourrait vous aider à passer à l'action!<br><br>Plusieurs choix s'offrent maintenant à vous [PRENOM]. Sentez-vous absolument libre de choisir ce qui vous convient. Vous êtes l'expert.
Message indiquant le dernier choix à faire du participant FEMME	S1.FINALF	Vers où tout cela vous mène?<br><br>Nous avons bien discuté aujourd'hui [PRENOM]. Avec ce que nous vivons ensemble, il pourrait être possible que votre motivation puisse changer positivement en vue de faire plus d'activités physiqueS.Si tel est le cas, **planifier votre pratique d'activités physiques** pour les prochains jours pourrait vous aider à passer à l'action!<br><br>Plusieurs choix s'offrent maintenant à vous [PRENOM]. Sentez-vous absolument libre de choisir ce qui vous convient. Vous êtes l'experte.
Question finale	S1.Q_FINAL	Alors, qu'aimeriez-vous faire maintenant?*<br><br>1- Je veux planifier mes activités physiques pour les prochains jours<br><br>2- Je veux retourner sur la page principale de Diabète en Forme

Second motivational session

Bouton d'entrée		
Nom	code	message
Les bénéfices de l'activité physique	-	Objectifs :<br>Explorer votre attitude par rapport à l'activité physique<br>Identifier les bénéfices reliés à l'activité physique que vous jugerez importants<br>Recevoir de l'information sur les bénéfices reliés à l'activité physique
Page 1 – retour sur la séance précédente		
Définition	Code	message
Retour sur la séance 1	S2.RETOURS1	AVANT DE COMMENCER…<br>PETIT RETOUR SUR LA DERNIÈRE SÉANCE<br><br>[PRENOM], à la dernière séance, vous avez remarqué que votre pratique d'activités physiques n'atteignait pas les recommandations canadienneS.En effet, vous pratiquiez [F.APTOT] minutes d'activités physiques au début du programme.

		Petit rappel : l'Association Canadienne du Diabète considère que pratiquer régulièrement des activités physiques correspond à 150 minutes d'activités physiques d'intensité modérée par semaine.<br><br>Vous avez ensuite eu la chance d'explorer les conséquences possibles liées à votre niveau actuel d'activité physique. Ainsi, cela vous a permis de réfléchir aux impacts que ces conséquences pourraient avoir dans votre quotidieS6.<br><br>Afin que vous puissiez prendre une décision éclairée concernant votre pratique d'activités physiques [PRENOM], nous tenions finalement à s'assurer que vous possédiez l'information suivante : Pratiquer des activités physiques régulièrement pourrait être une des meilleures façons de contrôler votre diabète et de réduire ses risques de complicationS.<br><br>Maintenant [PRENOM], les séances qui suivent vous aideront à explorer votre pratique d'activités physiques de manière plus positive qu'à la dernière séance.<br><br>Sans plus tarder, vous pouvez avancer pour la suite!<br>Bonne séance [PRENOM]!
Page 2 – vidéo de bienvenue et détails de la séance		
Mot de bienvenue - Vidéo de présentation	S2.B.VIDEO	Bienvenue à votre deuxième séance interactive [PRENOM],<br><br>Message du vidéo : Bonjour! Merci d'être là aujourd'hui! Je m'appelle Michel et je suis un des responsables du projet Diabète en Forme auquel vous prenez activement part depuis déjà au moins une semaine! J'espère vraiment que vous aimez votre expérience jusqu'à maintenant.<br><br>Vous le savez sans doute déjà, Si vous effectuez la séance aujourd'hui, nous allons explorer ensemble votre attitude et les bénéfices liés à la pratique d'activités physiqueS. La séance d'aujourd'hui pourrait donc vous permettre de répondre à certaines questions comme : 1) Actuellement, à quel point c'est important pour vous de pratiquer régulièrement de l'activité physique 2) Quels sont les bénéfices reliés à la pratique d'activités physiques qui pourraient être importants pour vouS.Aujourd'hui, nous discuterons ensemble, nous échangerons de l'information, mais au final, sachez que nous restons convaincus que vous êtes la personne la mieux placée pour prendre vos décisionS.<br><br>Finalement, que vous soyez très motivé pour l'instant, ou alors pas du tout motivé, ou alors un peu des deux, nous vous encourageons sincèrement à participer à cette séance. Nous pensons que ça ne peut qu'être avantageux pour vouS.Que ce soit pour vous motiver davantage, ou simplement pour en savoir plus sur vous et l'activité physique. Allez-y, participez à cette séance et voyez ce que vous pourrez en retirer.

		Voilà c'est tout pour moi! J'espère vraiment que vous aimerez votre séance et encore merci d'être là!
Détails techniques de la séance	S2.B.DÉTAILS	RAPPEL DES OBJECTIFS DE LA SÉANCE :<br><br>Discuter de votre attitude par rapport à l'activité physique<br><br>Identifier et approfondir les bénéfices qui seraient importants pour vous en lien avec la pratique régulière d'activités physiques<br><br>Planifier votre pratique d'activités physiques pour cette semaine ou pour les semaines qui suivent (optionel)<br><br>DURÉE DE LA SÉANCE<br><br>10 à 15 minutes
Valorisation du participant avant de commencer la séance	S2.B.VALORI	Félicitations pour vos avancements à travers Diabète en Forme [PRENOM].<br><br>Sans plus tarder, cliquez sur commencer pour débuter la séance!
Page 3 – Reflet sur l'attitude du participant à l'inscription		
Reflet pour une personne possédant une attitude très faible	S2.ATTI0	MON ATTITUDE ET LA PRATIQUE RÉGULIÈRE D'ACTIVITÉS PHYSIQUES<br><br>D'accord [PRENOM]! Commençons sans plus tarder!<br><br>En fonction de vos réponses à l'inscription, il semble que votre attitude face à l'activité physique soit assez défavorable. Pour vous, pratiquer régulièrement des activités physiques ne semble ni plaisant ni important pour l'instant. Nous aimerions donc d'abord vous dire ceci : vous avez vos propres raisons, et votre façon de penser est tout à fait acceptable [PRENOM].<br><br>La séance d'aujourd'hui sert à explorer les bénéfices associés à la pratique régulière d'activités physiques qui pourraient être importants pour vouS.Nous sommes conscients que vous n'avez pas une attitude favorable face à l'activité physique pour l'instant [PRENOM]. D'un autre côté, pourquoi ne pas essayer ensemble de trouver des aspects positifs pour vous à pratiquer des activités physiques? Après quoi, vous serez toujours l'unique maître de décider si vous voulez être acti[FVE] régulièrement ou non!<br><br>Si vous êtes prêt[E] à aller de l'avant, cliquez sur continuer [PRENOM].
Reflet pour une personne possédant une	S2.ATTILOW	MON ATTITUDE ET LA PRATIQUE RÉGULIÈRE D'ACTIVITÉS PHYSIQUES<br><br>D'accord [PRENOM]! Commençons sans plus tarder!

attitude faible		En fonction de vos réponses à l'inscription, il semble que votre attitude face à l'activité physique soit un peu négative. Pour vous, pratiquer régulièrement des activités physiques ne semble pas très plaisant, ou alors ce n'est pas très important pour vouS.Si tel est toujours le cas, nous aimerions donc d'abord vous dire ceci : vous avez vos propres raisons, et votre façon de penser est tout à fait acceptable [PRENOM].<br><br>La séance d'aujourd'hui sert à explorer les bénéfices associés à la pratique régulière d'activités physiques qui pourraient être importants pour vouS.Nous sommes conscients que vous n'avez pas une attitude favorable face à l'activité physique pour l'instant [PRENOM]. D'un autre côté, pourquoi ne pas essayer ensemble de trouver des aspects positifs pour vous à pratiquer des activités physiques?<br><br>Cette séance pourrait simplement vous aider à avoir une idée plus claire de l'importance de l'activité physique dans votre vie [PRENOM]. Après quoi, vous serez toujours l'unique maître de décider si vous voulez être acti[FVE] régulièrement ou non!<br><br>Si vous êtes prêt[E] à aller de l'avant, cliquez sur continuer [PRENOM]!
Reflet pour une personne possédant une attitude moyenne	S2.ATTIMID	MON ATTITUDE ET LA PRATIQUE RÉGULIÈRE D'ACTIVITÉS PHYSIQUES<br><br>D'accord [PRENOM]! Commençons sans plus tarder!<br><br>En fonction de vos réponses à l'inscription, il semble que votre attitude face à l'activité physique soit plus positive que négative. C'est une excellente une nouvelle! Voyons cela de façon plus détaillée. D'un côté, seuls quelques éléments peuvent vous incommoder dans le fait de pratiquer régulièrement des activités physiqueS.De l'autre côté cependant, vous voyez plusieurs éléments positifs à pratiquer des activités physiques régulièrement. Sachez que la plupart des personnes atteintes du diabète de type 2 se trouvent dans cette position [PRENOM], et c'est normal. Dans cette situation, il peut s'avérer difficile de décider de devenir plus acti[FVE], car on y perçoit à la fois des inconvénients et des bénéficeS5.<br><br>La séance d'aujourd'hui vous servira à explorer d'un autre œil les bénéfices associés à la pratique régulière d'activités physiques qui pourraient être importants pour vouS.Suite à cette séance, il se peut donc que vous soyez mieux en mesure de prendre des décisions convaincues concernant votre pratique d'activités physiqueS.<br><br>Si vous êtes prêt[E] à aller de l'avant [PRENOM], cliquez sur continuer!
Reflet pour une personne possédant une attitude	S2.ATTIHIGH	MON ATTITUDE ET LA PRATIQUE RÉGULIÈRE D'ACTIVITÉS PHYSIQUES<br><br>D'accord [PRENOM]! Commençons sans plus tarder!

positive		En fonction de vos réponses à l'inscription, il semble que votre attitude soit très positive face au fait de pratiquer régulièrement des activités physiqueS.C'est excellent! Vous voyez un certain plaisir au fait d'être acti[FVE] et vous considérez que c'est important pour vous [PRENOM]. Wow!<br><br>Pour continuer dans cette lancée, la séance d'aujourd'hui vous servira donc à explorer les bénéfices associés à la pratique régulière d'activités physiques qui pourraient être importants pour vouS.En effectuant cette séance, vous pourrez renforcir l'importance que vous attachez déjà au fait de pratiquer régulièrement des activités physiqueS.Intéressant non?<br><br>Si vous êtes prêt[E] à aller de l'avant [PRENOM], cliquez sur continuer!
Page 4 – Début de l'exploration des bénéfices associés à l'activité physique		
Introduction aux bénéfices de la pratique régulière de l'activité physique	S2.INTROBEN	DES BÉNÉFICES QUI IMPORTENT<br><br>D'accord [PRENOM]!<br><br>Vous savez sans doute déjà que pratiquer régulièrement des activités physiques pourrait engendrer de nombreux bénéfices sur votre santé. En effet, en plus de vous aider à mieux contrôler votre diabète et de réduire les risques associés à cette maladie, être acti[FVE] régulièrement peut amener bien d'autres bénéfices dans votre quotidieS6.<br><br>Les bénéfices qui vous seront présentés ci-dessous sont tous appuyés par des études scientifiques [PRENOM]. Autrement dit, ce sont de réels effets positifs que vous pouvez ressentir en pratiquant régulièrement des activités physiqueS.<br><br>Si vous le voulez, voyons maintenant si certains de ces bénéfices pourraient être importants pour vouS5.
Question sur les bénéfices	S2.BEN1	Parmi cette liste, quels seraient donc les bénéfices qui seraient importants pour vous si vous pratiquiez régulièrement des activités physiques [PRENOM]? (Sélectionnez de 0 à 8 bénéfices)<br><br>Avoir plus d'énergie au quotidien
Choix de réponses	S2.BEN2	Diminuer votre stress au quotidien
	S2.BEN3	Contrôler votre poids
	S2.BEN4	Avoir plus d'interactions sociales
	S2.BEN5	Réduire vos risques des problèmes de santé (ostéoporose, certains cancers, arthrite, etc.)
	S2.BEN6	Vous sentir mieux dans votre peau
	S2.BEN7	Avoir une meilleure qualité de vie
	S2.BEN8	Avoir un meilleur sommeil

Question ouverte	S2.BENOUV	Pourrait-il y avoir un autre bénéfice plus personnel qui serait important pour vous [PRENOM]?<br>Nous vous encourageons à l'inscrire dans l'espace qui suit.<br>(espace libre)
Exemple de réponse	S2.EXBEN	Exemple de réponse d'une personne atteinte du diabète de type 2 :<br>« Je crois qu'être acti[FVE] régulièrement pourrait m'aider à avoir de meilleures relations avec mes procheS.»
Transition vers la prochaine page	S2.BES6.TRANSI	Si vous pensez à un bénéfice personnel [PRENOM], l'inscrire dans l'espace pourrait vous aider à mieux peser les pour et les contres de devenir acti[FVE] régulièrement. Cela dit, vous êtes tout à fait libre d'écrire quelque chose ou non!<br><br>Cliquez sur continuer pour avancer lorsque vous vous sentez prêt[E] [PRENOM]!
Page 5 – Reflet sur les bénéfices importants de la personne		
Reflet pour la sélection de 0 bénéfice	S2.REFBEN0	D'accord [PRENOM], si vous avez inscrit quelque chose dans l'espace libre, nous y reviendrons plus tard.<br><br>Pour le moment, il semble que vous ne voyez pas de bénéfice vraiment important pour vous parmi ceux qui vous étaient proposéS. C'est tout à fait possible!<br><br>Si vous êtes d'accord [PRENOM], nous aimerions vous partager un peu plus d'informationS.Ces informations pourraient vous aider à bien comprendre les impacts positifs que représentent les bénéfices de l'activité physique sur votre santé.
Question demande d'information	S2.Q.INFOBEN0	Aimeriez-vous recevoir ces informations [PRENOM]?
Reflet pour la sélection d'un bénéfice	S2.REFBEN1	Excellent [PRENOM]! Il semble que vous voyez au moins un bénéfice qui pourrait être important pour vouS.En fait, si vous décidiez de devenir acti[FVE] régulièrement, vous apprécierez le fait [DE] [REFBEN].<br><br>Maintenant, comme à la première séance [PRENOM], tentons de voir maintenant quels impacts concrets pourraient avoir ce bénéfice dans votre vie.<br><br>Si vous avez inscrit quelque chose dans l'espace libre, nous y reviendrons plus tard!<br><br>Cliquez sur continuer pour avancer.
Reflet pour la sélection de deux bénéfices	S2.REFBEN2	Excellent [PRENOM]! Il semble que vous voyez deux bénéfices qui pourraient être importants pour vouS.En fait, si vous décidiez de devenir acti[FVE] régulièrement, vous apprécierez le fait [DE] [REFBEN].<br><br>Maintenant, comme à la première séance [PRENOM], tentons de voir quels impacts concrets pourraient avoir ces bénéfices dans votre vie.<br><br>Si vous avez inscrit quelque chose dans l'espace libre, nous y reviendrons plus tard!

		Cliquez sur continuer pour avancer.
Reflet pour la sélection de trois bénéfices et plus	S2.REFBEN3P	Wow [PRENOM]! Vous voyez beaucoup de bénéfices au fait de pratiquer régulièrement des activités physiqueS.Si vous décidiez d'être acti[FVE] régulièrement, voici tous les bénéfices que vous apprécieriez [PRENOM] :<br><br>[BEN1]<br>[BEN2]<br>[BEN3]<br>[BEN4]<br>[BEN5]<br>[BEN6]<br>[BEN7]<br>[BEN8]<br><br>Si vous avez inscrit quelque chose dans l'espace libre, nous y reviendrons plus tard.
Question – choix de deux bénéfices encore plus importants	S2.Q.3PBEN	Maintenant, pourriez-vous sélectionner seulement qu'un ou deux bénéfices qui seraient encore plus importants que tous les autres pour vous [PRENOM]?<br><br>(les mêmes choix de réponses qu'à la première question)
Page 6- Reflet sur la route du 3 bénéfices et plus/ Question d'élaboration sur les bénéfices		
NOTE : SI LA PERSONNE SÉLECTIONNE 0 BÉNÉFICE, CETTE PAGE EST SAUTÉE		
Deuxième reflet pour le bénéfice le plus importants des participants voyant plus de deux bénéfices importants pour eux	S2.REF2BEN3P1	Excellent [PRENOM]! Vous avez trouvé quelque chose d'encore plus important pour vous parmi les bénéfices que vous avez choisiS.Il semble que vous apprécieriez plus que tout autre bénéfice le fait [DE] [REF3PBEN]<br><br>Comme à la première séance [PRENOM], tentons de voir maintenant quels impacts concrets pourraient avoir ce bénéfice dans votre vie. Ci-dessous, un espace vous sera dédié.
Deuxième reflet pour les bénéfices les plus importants des participants voyant plus de deux bénéfices importants pour eux	S2.REF2BEN3P2	Excellent [PRENOM]! Vous avez trouvé quelque chose d'encore plus important pour vous parmi les bénéfices que vous avez choisiS.Il semble que vous apprécieriez plus que tout autre bénéfice le fait [DE] [REF3PBEN]<br><br>Comme à la première séance [PRENOM], tentons de voir maintenant quels impacts concrets pourraient avoir ces bénéfices dans votre vie. Ci-dessous, deux espaces vous seront dédiéS5.
Introduction à la question d'élaboration	S2.INTRO1BEN	[PRENOM], nous venons de mentionner que vous apprécieriez le fait [DE] [REFBEN] si vous étiez acti[FVE] régulièrement.<br><br>Maintenant [PRENOM], pensez aux impacts positifs concrets que ce bénéfice pourrait vous apporter dans votre vie. Ci-dessous, un espace vous est dédié pour inscrire ces impactS5.

Introduction aux questions d'élaboration	S2.INTRO2BEN	[PRENOM], nous venons de mentionner que vous apprécieriez le fait [DE] [REFBEN] si vous étiez acti[FVE] régulièrement.<br><br>Maintenant [PRENOM], pensez aux impacts positifs concrets que ces bénéfices pourraient vous apporter dans votre vie. Ci-dessous, deux espaces vous sont dédiés pour inscrire ces impacts:
Question d'élaboration sur un premier bénéfice important pour la personne	S2.Q.ELAB1	Pour vous, en une phrase [PRENOM], comment le fait [DE2] [B.ELAB1] pourrait avoir des impacts positifs dans votre [VIE] ?
Question d'élaboration supplémentaire si la personne voit deux bénéfices importants	S2.Q.ELAB2	Pour vous, en une phrase [PRENOM], comment le fait [DE2] [B.ELAB2] pourrait avoir des impacts positifs dans votre [VIE] ?
Microtailoring servant d'exemple d'élaboration d'une personne atteinte du diabète de type 2 pour la question 1	S2.B.EXEL1	Ben1 – Je me sentirais vraiment mieux avec plus d´énergie. Je pourrais faire tout ce que je veux vraiment faire dans une journée, sans être limité[E].<br><br>Ben2 – Je me sentirais tellement plus libre en étant moins stressé[E] tout le tempS.J'angoisserais moins et ça serait vraiment mieux pour moi.<br><br>Ben3 – Je sais que faire régulièrement des activités physiques m'aiderait à gérer mon poids et je serais vraiment content[E] de moi de réussir ça. J'éprouverais du bonheur.<br><br>Ben4 – Ça me ferait vraiment du bien de voir plus de gens, comme si je m'inscrivais à des cours en groupe. Ça m'aiderait aussi à être plus en confiance avec les autres et ça j'aimerais ça.<br><br>Ben5 – Je me sentirais bien, en santé. Je me sentirais fier[E] de prendre soin de moi et de savoir que je fais la bonne chose.<br><br>Ben6 – Je me sentirais plus à l'aise avec moi-même, et avec les autreS.Ça m'aiderait à être encore plus heureu[XSE]!<br><br>Ben7 – J'éprouverais un sentiment de bien-être constant qui me manque pour l'instant. Je serais juste mieux, et ça me ferait vraiment du bien!<br><br>Ben8 – Je me sentirais beaucoup mieux et beaucoup plus énergique dans la vie de tous les jourS.Ça m'aiderait à offrir le meilleur de moi aux autres, et ça j'aimerais ça.

Microtailoring servant d'exemple d'élaboration d'une personne atteinte du diabète de type 2 pour la question 2	S2.B.EXEL2	Ben2- Je me sentirais tellement plus libre en étant moins stressé[E] tout le tempS.J'angoisserais moins et ça serait vraiment mieux pour moi.<br>Ben3- Ça me ferait vraiment du bien de voir plus de gens, comme si je m'inscrivais à des cours en groupe. Ça m'aiderait aussi à être plus en confiance avec les autres et ça j'aimerais ça.<br>Ben4- Ça me ferait vraiment du bien de voir plus de gens, comme si je m'inscrivais à des cours en groupe. Ça m'aiderait aussi à être plus en confiance avec les autres et ça j'aimerais ça.<br>Ben5- Je me sentirais bien, en santé. Je me sentirais fier[E] de prendre soin de moi et de savoir que je fais la bonne chose.<br>Ben6- Je me sentirais plus à l'aise avec moi-même, et avec les autreS.Ça m'aiderait à être encore plus heureu[XSE]!<br>Ben7- J'éprouverais un sentiment de bien-être constant qui me manque pour l'instant. Je serais juste mieux, et ça me ferait vraiment du bien!<br>Ben8- Je me sentirais beaucoup mieux et beaucoup plus énergique dans la vie de tous les jourS.Ça m'aiderait à offrir le meilleur de moi aux autres, et ça j'aimerais ça.
Transition	S2.B.TRANSEL	Encore une fois [PRENOM], vous êtes libre d'écrire quelque chose ou non! Cet exercice peut simplement vous aider à y voir plus clair dans vos décisions concernant votre pratique d'activités physiqueS5.<br><br>Lorsque vous avez terminé, cliquez sur continuer pour la suite!
Page 7 – Reflet sur l'élaboration		
NOTE : CETTE PAGE EST SAUTÉE SI 0 BÉNÉFICE		
Reflet sur l'élaboration si le participant n'a rien inscrit dans l'espace et ne voyait qu'un bénéfice	S2.B.REFEL01	Très bien [PRENOM]! Il semble que vous ayez peut-être seulement réfléchi aux impacts positifs que le fait [DE] [REFBEN] pourrait apporter à votre vie. C'est tout à fait acceptable!
Reflet sur l'élaboration si le participant a écrit dans l'espace et ne voyait qu'un bénéfice	S2.B.REFEL11	Très bien [PRENOM]! Il semble que vous ayez trouvé des impacts positifs concrets liés au bénéfice important pour vous, c'est-à-dire « [REFBEN] ». C'est excellent!
Reflet sur l'élaboration si le participant n'a rien	S2.B.REFEL02	Très bien [PRENOM]! Il semble que vous ayez peut-être seulement réfléchi aux impacts positifs que les bénéfices importants pour vous pourraient apporter à votre vie. C'est tout à fait acceptable!

inscrit dans l'espace et voyait deux bénéfice		
Reflet sur l'élaboration si le participant a écrit dans un espace sur 2 et voyait 2 bénéfices	S2.B.REFEL12	Très bien [PRENOM]! Il semble que vous ayez trouvé des impacts positifs concrets liés à un bénéfice important pour vous, c'est-à-dire « [REFBENCHOIX] ». C'est excellent! Aussi, peut-être avez-vous seulement réfléchi aux impacts positifs liés à votre second bénéfice.
Reflet sur l'élaboration si le participant a écrit dans les deux espaces et voyait 2 bénéfices	S2.B.REFEL22	Très bien [PRENOM]! Il semble que vous ayez trouvé des impacts positifs concrets liés aux deux bénéfices importants pour vouS.C'est excellent!<br>about
Question pour demander si le participant veut recevoir de l'information	S2.Q.INFOBEN	Avant de vous fournir un résumé de votre séance d'aujourd'hui [PRENOM], nous aimerions vous transmettre des informations qui pourraient vous intéresser concernant les bénéfices qui sont importants pour vouS.Aussi, nous aimerions vous donner de l'information sur les bénéfices associés au fait de pratiquer régulièrement des activités physiques de manière générale.<br><br>Aimeriez-vous recevoir ces informations [PRENOM]?<br><br><br>Choix : oui/non
Page 8 – page d'informations		
NOTE : cette page est sautée si le participant ne veut pas plus d'informations		
Introduction à l'information fournie au participant	S2.B.INTROINFO	DES INFORMATIONS, POUR VOUS (titre)<br><br>Merci [PRENOM], nous admirons votre curiosité. Voici donc les informations que nous aimerions vous partager.
Information sur le bénéfice 1	S2.B.INFO1	À propos de : Avoir plus d'énergie au quotidien (titre)<br><br>Tant chez les personnes atteintes du diabète de type 2 que dans la population en général, pratiquer régulièrement des activités physiques peut vraiment faire une différence dans votre niveau d'énergie [PRENOM]. En fait, la plupart des gens qui commencent à pratiquer régulièrement des activités physiques en ressentent généralement très vite des effets constants sur leur niveau d'énergie. Souvent, ces effets arrivent en quelques semaines seulement, et des fois, bien plus rapidement. Règle simple : plus vous tendez vers les recommandations de l'Association Canadienne du Diabète en terme d'activités physiques, plus votre niveau d'énergie pourrait s'améliorer positivement. BONUS : les études démontrent également que pratiquer régulièrement des activités

		physiques est très efficace afin d'avoir une meilleure concentration et une meilleure vigilance au quotidieS6.
Information sur le bénéfice 2	S2.B.INFO2	À propos de : Diminuer votre stress au quotidien<br><br>La majorité des adultes d'aujourd'hui éprouvent du stress ou de l'anxiété chaque jour. Vous n'êtes pas seul[E] [PRENOM]. Les gens utilisent généralement plusieurs façons pour tenter de diminuer ce stress quotidien : discuter avec des proches, dormir, se détendre devant la télévision, manger, écouter de la musique et pratiquer des activités physiqueS.Cela dit, pratiquer des activités physiques pourrait cependant être la meilleure façon de gérer votre stresS.C'est simple, il s'agit de la façon qui produit le plus d'effets, à la fois sur votre santé physique et votre santé mentale. Les études démontrent qu'une seule séance d'activités physiques de 10 minutes est suffisante pour observer des effets antistress [PRENOM]. Évidemment, plus on en pratique régulièrement, plus on obtient des résultatS.Voici des exemples concrets d'effets importants que pratiquer régulièrement des activités physiques pourrait vous apporter [PRENOM] : diminuer votre niveau de tension, améliorer votre humeur, améliorer votre sommeil et améliorer votre estime.
Information sur le bénéfice 3	S2.B.INFO3	À propos de : Contrôler votre poids<br><br>[PRENOM], vous avez sûrement déjà entendu dire que l'atteinte du poids santé peut vous aider à prévenir plusieurs complications liées à votre diabète, ainsi que plusieurs autres problèmes de santé. Vous savez sans doute également que l'activité physique et l'alimentation sont les deux grands éléments à considérer lorsque nous voulons contrôler notre poids de façon saine [PRENOM]. L'un ne va pas sans l'autre. La mission de Diabète en Forme n'est cependant pas de vous parler d'alimentation, mais de vous aider à trouver vos propres motivations à pratiquer des activités physiqueS.Cela dit, si contrôler votre poids est important pour vous [PRENOM], pratiquer régulièrement des activités physiques se doit d'être un élément essentiel de votre démarche. Bien honnêtement, pratiquer des activités physiques au moins 150 minutes par semaine à une intensité modérée constitue un minimum pour un bon contrôle du poids, de manière générale. Si c'est ce que vous désirez [PRENOM], nous sommes sincèrement convaincus que vous pouvez y arriver.
Information sur le bénéfice 4	S2.B.INFO4	À propos de : Avoir plus d'interactions sociales<br><br>[PRENOM], vous aurez compris que pratiquer régulièrement des activités physiques n'engendre pas automatiquement plus d'interactions sociales

		avec les autreS.Cependant, certains types et certaines façons de pratiquer des activités physiques amènent à être en contact avec les genS.Voici certaines façons de favoriser vos interactions sociales en pratiquant des activités physiques [PRENOM] :<br>Planifier des séances hebdomadaires de marche, de vélo ou de randonnée avec un(e) ou des ami (e)S5.<br>Planifier des séances hebdomadaires d'activités physiques en famille : aller monter une montagne, marcher jusqu'au parc du quartier.<br>S'inscrire à un cours en groupe d'une activité physique qui vous plaît<br>Pratiquer des activités physiques dans des endroits publics : dans un parc ou dans un centre d'entraînement.<br>Avoir un entraîneur avec qui vous développez une bonne relatioS6.<br>Pour avoir de bonnes interactions sociales, il vous faut simplement choisir les activités qui vous conviennent [PRENOM]. Si c'est ce que vous désirez, vous en êtes définitivement capable.
Information sur le bénéfice 5	S2.B.INFO5	À propos de : Réduire vos risques d'avoir d'autres problèmes de santé<br><br>[PRENOM], plusieurs personnes atteintes du diabète pratiquent également des activités physiques pour se protéger d'autres problèmes de santé qu'elles n'aimeraient pas avoir dans leur vie. Vous avez sûrement déjà entendu que pratiquer des activités physiques est un excellent moyen de prévenir plusieurs maladieS.C'est tout à fait le cas [PRENOM]. En pratiquant régulièrement des activités physiques, vous réduisez vos risques d'avoir plusieurs problèmes de santé, ou alors de les aggraver. Voici plusieurs problèmes de santé que l'activité physique peut prévenir de manière importante : l'ostéoporose, l'arthrite, les AVC, les Infarctus, les maladies cardiovasculaires en général, le cancer de la prostate, le cancer du sein, la dépression, l'insomnie, le rhume, la grippe et d'autreS.L'activité physique peut également soulager certains de ces problèmes de santé si vous en êtes déjà atteint[E].<br><br>Il n'y a pas à dire, pratiquer régulièrement des activités physiques est un des meilleurs traitements qu'on peut s'offrir pour rester en santé longtemps [PRENOM].
Information sur le bénéfice 6	S2.B.INFO6	À propos de : Vous sentir mieux dans votre peau<br><br>Ceux qui pratiquent régulièrement des activités physiques le savent [PRENOM], le fait de bouger pendant une période continue d'au moins 10 minutes crée un sentiment de bien-être bien réel. En fait, plusieurs chercheurs attribuent ce phénomène à des hormones appelées « endorphines » qui sont libérées dans notre corps lorsque nous faisons un effort physique. Plus on stimule souvent ce mécanisme [PRENOM], plus il devient alors facile d'éprouver du plaisir pour les petites choses de la vie.

		De manière générale, les gens qui pratiquent régulièrement des activités physiques ont donc plus de facilité à éprouver du bonheur facilement. Ces mêmes personnes éprouvent généralement aussi moins de stress, ce qui contribue à se sentir bieS6.Pratiquer régulièrement des activités physiques pourrait ainsi réellement vous faire sentir mieux dans votre peau [PRENOM].
Information sur le bénéfice 7	B.INFO7	À propos de : Avoir une meilleure qualité de vie<br><br>Lorsqu'on parle de la qualité de vie [PRENOM], on parle un peu de tout. Votre santé physique, mentale, émotionnelle et vos relations avec vos proches sont toutes des éléments qui sont englobés par ce qu'on appelle la « qualité de vie ». Plus ces quatre niveaux vont bien, plus notre vie se porte bien n'est-ce pas? C'est un peu la beauté de pratiquer régulièrement des activités physiques [PRENOM]. En étant acti[FVE] régulièrement, les études démontrent que vous pouvez retirer des bénéfices importants dans les quatre niveaux. L'activité physique peut vous aider à prévenir bien des maladies, à être plus concentré[E] au quotidien, à avoir une meilleure humeur avec vos proches et vous-même, sans compter bien d'autres bénéfices [PRENOM].
Information sur le bénéfice 8	S2.B.INFO8	À propos de : Avoir un meilleur sommeil<br><br>Tout le monde sera d'accord qu'on se sent toujours mieux lorsqu'on a bien dormi [PRENOM]. Quand vous dormez bien, vous êtes sans doute plus concentré[E] et plus éveillé[E]. Votre mémoire fonctionne également mieux et vous avez plus de facilité à avoir une humeur positive avec vous-même et avec les autreS.Ce qui est intéressant [PRENOM], c'est qu'en pratiquant des activités physiques régulièrement, vous pourriez effectivement vous offrir un sommeil plus récupérateur. Petit rappel : pratiquer régulièrement des activités physiques correspond à 150 minutes d'activités physiques par semaine à intensité modérée. Cela dit, des études ont démontré que l'activité physique faite sur une base régulière a un lien positif avec une hormone appelée « sérotonine ». Cette hormone est très importante afin d'avoir un sommeil de qualité. Sans aucun doute, pratiquer des activités physiques régulièrement pourrait vous aider à mieux dormir [PRENOM], et ainsi vous pourriez bénéficier d'une bonne humeur constante et d'une bonne concentration au quotidieS6.
Information « Take home message »	S2.B.INFOGEN	D'autres informations, pour vous<br><br>Voilà, nous sommes encore une fois allés à l'essentiel. Au final, le message que nous voulons vous transmettre est simple [PRENOM] : pratiquer des activités physiques régulièrement pourrait réellement vous offrir l'opportunité d'obtenir des bénéfices qui sont importants pour vouS5.<br><br>À la page suivante, nous tenterons de vous faire un résumé de ce que vous nous avez communiqué aujourd'hui. Nous espérons sincèrement que cela aura du sens pour vous [PRENOM]. Après ce résumé, il vous sera aussi

		possible de planifier des activités physiques pour les prochains jours si vous le désirez.
Information pour la personne voyant 0 bénéfices	S2.B.INFO0	À propos de : L'activité physique, ses bénéfices et la qualité de vie<br><br>Comme vous avez vu [PRENOM], l'activité physique peut apporter de nombreux bénéfices sur la santé, mais ceux-ci ne semblent pas très importants pour vous pour l'instant. C'est tout à fait acceptable [PRENOM] et nous ne sommes pas ici pour vous juger. Si vous permettez, nous aimerions simplement vous parler du concept de qualité de vie quelques instantS5.<br><br>Lorsqu'on parle de la qualité de vie [PRENOM], on parle un peu de tout. Votre santé physique, mentale, émotionnelle et vos relations avec vos proches sont toutes des éléments qui sont englobés par ce qu'on appelle la « qualité de vie ». Plus ces quatre niveaux vont bien, plus notre vie se porte bien n'est-ce pas? C'est un peu la beauté de pratiquer régulièrement des activités physiques [PRENOM].<br><br>En étant acti[FVE] régulièrement, les études démontrent que vous pouvez retirer des bénéfices importants dans les quatre niveaux. L'activité physique peut ainsi vous aider à prévenir bien des maladies, à être plus concentré[E] au quotidien, à avoir une meilleure humeur avec vos proches et vous-même, sans compter bien d'autres bénéfices qui contribuent à une meilleure qualité de vie.<br><br>Ainsi, comment le fait d'avoir une meilleure qualité de vie pourrait avoir des impacts positifs dans votre vie [PRENOM]?
Info général et transition pour 0 bénéfice	S2.B.INFOGEN0	Voilà, nous sommes encore une fois allés à l'essentiel [PRENOM].<br><br>À la page suivante, nous tenterons de vous faire un résumé de ce que vous nous avez communiqué aujourd'hui. Nous espérons sincèrement que cela aura du sens pour vous [PRENOM]. Après ce résumé, il vous sera aussi possible de planifier des activités physiques pour les prochains jours <u>si vous le désirez</u>.

Question d'appréciation de l'information	S2.Q.B.AIME	Avant d'aller plus loin, que pensez-vous des informations précédentes [PRENOM]?<br><br>Choix :<br>1 – J'ai aimé<br>2 – Je n'ai pas vraiment aimé<br>3 – Je ne sais pas trop
Page 9 – Résumé de la séance		
Reflet sur sentiment par rapport à l'information - choix 1	S2.B.REFAIME1	Merci de le partager [PRENOM].<br><br>Il semble que vous ayez apprécié les informations précédentes! Nous sommes heureux de l'entendre.<br><br>Voici maintenant votre résumé de la séance d'aujourd'hui [PRENOM].<br><br>Merci encore et félicitations de vous être rendu[E] jusqu'ici déjà.
Reflet sur sentiment par rapport à l'information - choix 2	S2.B.REFAIME2	Mmmmm…<br><br>Les informations précédentes n'ont pas satisfait vos attentes [PRENOM]. Nous espérons vraiment pouvoir mieux saisir ce dont vous aurez besoin à travers les prochaines activités de Diabète en Forme.<br><br>Voici quand même un résumé de votre séance d'aujourd'hui [PRENOM].<br><br>Merci encore et félicitations de vous être rendu[E] jusqu'ici déjà.
Reflet sur sentiment par rapport à l'information - choix 3	S2.B.REFAIME3	Mmmmm…<br><br>Vous ne savez pas trop quoi penser des informations précédentes [PRENOM]. Nous espérons qu'elles puissent malgré tout vous aider à prendre des décisions plus éclairéeS5.<br><br>Pour le moment, voici quand même un résumé de votre séance d'aujourd'hui [PRENOM].<br><br>Merci encore et félicitations de vous être rendu[E] jusqu'ici déjà.
Remerciements	S2.B.MERCI	--------intégrés au reflet par rapport à l'information------
Retour sur attitude zéro	S2.B.RES5.ATT0	Résumé de la séance, pour vous<br><br>D'accord [PRENOM], voici ce que nous retenonS.Nous avons commencé la séance d'aujourd'hui en discutant de votre attitude par rapport au fait de pratiquer régulièrement des activités physiqueS. Au moment de l'inscription, il semble que votre attitude était négative. Vous ne voyiez ni vraiment de plaisir à être acti[FVE] régulièrement ni d'importance. Ensemble, nous avons malgré tout tenté de voir quels bénéfices reliés à

		l'activité physique pourraient être importants pour vouS.Bravo pour votre démarche.
Retour sur attitude faible	S2.B.RES5.ATTLOW	Résumé de la séance, pour vous<br><br>D'accord [PRENOM], voici ce que nous retenonS.Nous avons commencé la séance d'aujourd'hui en discutant de votre attitude par rapport au fait de pratiquer régulièrement des activités physiqueS. Au moment de l'inscription, il semble que votre attitude était un peu négative. Vous voyiez peu de plaisir à être acti[FVE] régulièrement et peu d'importance. Ensemble, nous avons malgré tout tenté de voir quels bénéfices reliés à l'activité physique pourraient être importants pour vouS.Bravo pour votre démarche.
Retour sur attitude moyen	S2.B.RES5.ATTMID	Résumé de la séance, pour vous<br><br>D'accord [PRENOM], voici ce que nous retenonS.Nous avons commencé la séance d'aujourd'hui en discutant de votre attitude par rapport au fait de pratiquer régulièrement des activités physiqueS. Au moment de l'inscription, il semble que votre attitude était plutôt positive. Vous y verriez un peu de plaisir à être acti[FVE] régulièrement et un peu d'importance. C'est un bon départ! Ensemble, nous avons donc tenté de voir quels bénéfices reliés à l'activité physique pourraient être importants pour vouS.Bravo pour votre démarche.
Retour sur attitude forte	S2.B.RES5.ATTHIGH	Résumé de la séance, pour vous<br><br>D'accord [PRENOM], voici ce que nous retenonS.Nous avons commencé la séance d'aujourd'hui en discutant de votre attitude par rapport au fait de pratiquer régulièrement des activités physiqueS. Au moment de l'inscription, il semble que votre attitude était très positive! Vous y verriez beaucoup de plaisir au fait d'être acti[FVE] régulièrement et beaucoup d'importance. C'est excellent! Ensemble, nous avons donc tenté de voir quels bénéfices reliés à l'activité physique sont importants pour vous afin de rendre votre attitude encore plus solide. Bravo pour votre démarche.
Retour sur les bénéfices importants – 0 ben	S2.B.RES5.BEN0	Des raisons de vouloir devenir plus acti[FVE]<br><br>Maintenant, parmi les bénéfices que pratiquer régulièrement des activités physiques pourrait vous apporter [PRENOM], vous n'en avez trouvé aucun qui soit important pour vous pour le moment. C'est tout à fait acceptable et vous êtes l'expert[E].
Retour sur les bénéfices importants – 1 bénéfice	S2.B.RES5.BEN1	Des raisons de vouloir devenir plus acti[FVE]<br><br>Maintenant, parmi les bénéfices que pratiquer régulièrement des activités physiques pourrait vous apporter [PRENOM], le fait [DE] [REFBEN] représenterait le bénéfice le plus important pour vouS.
Retour sur les	S2.B.RES5.BEN2	Des raisons de vouloir devenir plus acti[FVE]

bénéfices importants – 2 bénéfices		Maintenant, parmi les bénéfices que pratiquer régulièrement des activités physiques pourrait vous apporter [PRENOM], le fait [DE] [REFBEN] représenterait le bénéfice le plus important pour vouS5.
Retour sur les bénéfices importants – 3 bénéfices et plus avec 1 bénéfice plus important	S2.B.RES5.BEN31	Des raisons de vouloir devenir plus acti[FVE]<br><br>Il semble que beaucoup de bénéfices seraient importants pour vous dans le fait de pratiquer régulièrement des activités physiques [PRENOM]. Les voici :<br>[BEN1]<br>[BEN2]<br>[BEN3]<br>[BEN4]<br>[BEN5]<br>[BEN6]<br>[BEN7]<br>[BEN8]<br>Parmi ceux-ci, le fait [DE] [REF3PBEN] semble être un bénéfice encore plus important pour vous [PRENOM].
Retour sur les bénéfices importants – 3 bénéfices et plus avec 1 bénéfice plus important	S2.B.RES5.BEN32	Des raisons de vouloir devenir plus acti[FVE]<br><br>Il semble que beaucoup de bénéfices seraient importants pour vous dans le fait de pratiquer régulièrement des activités physiques [PRENOM]. Les voici :<br>[BEN1]<br>[BEN2]<br>[BEN3]<br>[BEN4]<br>[BEN5]<br>[BEN6]<br>[BEN7]<br>[BEN8]<br>Parmi ceux-ci, le fait [DE] [REF3PBEN] semblent être deux bénéfices encore plus importants pour vous [PRENOM].
Retour sur élaboration du premier bénéfice si le participant a choisi 1 bénéfice seulement, et qu'il a élaboré sur celui-ci	S2.B.RES5.ELAB11	Selon vos réponses, les impacts positifs que ce bénéfice pourrait amener dans votre vie sont les suivants: « [Q.B.ELAB1] ».
Retour sur élaboration du premier bénéfice si le participant a choisi 1 bénéfice seulement, et qu'il n'a pas élaboré	S2.B.RES5.ELABO1	En lien avec celui-ci, vous avez eu un moment pour réfléchir aux impacts positifs qu'il pourrait apportés dans votre vie. Quels étaient ces impacts [PRENOM]?

sur celui-ci.		
Retour sur élaboration du deuxième bénéfice si le participant a choisi 2 bénéfices mais n'a pas élaboré sur aucuS6.	S2.B.RES5.ELABO2	En lien avec ceux-ci, vous avez eu un moment pour réfléchir aux impacts positifs qu'ils pourraient apportés dans votre vie. Quels étaient ces impacts [PRENOM]?
Retour sur élaboration du deuxième bénéfice si le participant a choisi 2 bénéfices mais a élaboré sur 1	S2.B.RES5.ELAB12	En lien avec ces bénéfices, vous avez réfléchi aux impacts positifs qu'ils pourraient apportés dans votre vie [PRENOM]. Voici donc les impacts que le fait [DE] [REFBENCHOIX] pourrait avoir dans votre vie: «[Q.B.ELABCHOIX] ».
Retour sur élaboration du deuxième bénéfice si le participant a choisi 2 bénéfices et a élaboré sur 2	S2.B.RES5.ELAB22	En lien avec ces bénéfices, vous avez réfléchi aux impacts positifs qu'ils pourraient apportés dans votre vie [PRENOM]. Voici donc les impacts que le fait [DE] [B.ELAB1] pourrait avoir dans votre vie: «[Q.B.ELAB1] ». Et voici les impacts que le fait [DE] [B.ELAB2] pourrait avoir dans votre vie : « Q.B.ELAB2 ».
retour sur bénéfice personnel	S2.B.RES5.BENOUV	[COTEDEPLUS], il semble que vous verriez un bénéfice plus personnel au fait de pratiquer régulièrement des activités physiques [PRENOM] : « Q.BENOUV ».
Retour sur information	S2.B.RES5.INFO	Nous vous avons finalement transmis quelques informations que nous jugions susceptibles de vous intéresser. Ces informations vous ont également été transmises par courriel pour qu'ainsi vous puissiez les revoir si vous le voulez.<br><br>Merci [PRENOM]
Retour sur information si elle a été refusée	S2.B.RES5.INFO0	Vous avez finalement décidé de passer les informations que nous avions pour vous [PRENOM]. Il est possible que vous en sachiez déjà bien assez sur les bénéfices de l'activité physique. Comme nous disons depuis le début, nous croyons que vous êtes votre propre expert[E]. Pour finir ce résumé, nous aimerions seulement vous dire ceci : si jamais vous vouliez devenir plus acti[FVE], nous avons sincèrement confiance que vous pouvez y arriver. Et nous serons là pour vous soutenir.<br><br>Merci [PRENOM]
Ce qui vient dans la prochaine séance	S2.B.RES5.VENIR	À venir dans la prochaine séance<br><br>Dans la prochaine séance, nous discuterons de votre motivation [PRENOM]. Ensuite, nous discuterons (si vous le voulez!) des choses qui sont importantes pour vous dans la vie et nous tenterons de voir si le fait de pratiquer régulièrement des activités physiques pourrait vous aider à mieux profiter de ces choseS.<br><br>Au plaisir d'avoir une autre conversation avec vous [PRENOM]!<br><br>Merci énormément

Conclusion/transition vers plan d'action	S2.B.RES5.TRANS	Lorsque vous êtes prêt[E], vous pouvez aller à la page finale!
Page 10 – Plan d'action (dernière page)		
Message pour attitude 0	S2.B.PLAN0	Vers où tout cela vous mène?<br><br>Nous avons bien discuté durant les dernières semaines [PRENOM]. Avec ce que nous vivons ensemble, il pourrait être possible que votre attitude, votre confiance et votre motivation aient alors changé positivement en vue de faire plus d'activités physiqueS.<br><br>Peu importe comment vous vous sentez, plusieurs choix s'offrent maintenant à vous [PRENOM]. Sentez-vous absolument libre de choisir ce qui vous convient (vous êtes l'unique expert[E] de vous-même!).
Question sur ce que le participant veut faire maintenant	S2.Q.B.PLAN	Alors, qu'aimeriez-vous faire maintenant?*<br><br>1- Je veux planifier mes activités physiques pour les prochains jours<br><br>2- Je vois des choses qui m'empêchent de faire plus d'activités physiques, j'aimerais en discuter<br><br>3- Je veux tout simplement quitter pour l'instant (si tel est le cas, au plaisir de vous revoir bientôt!)<br><br>*Vous pourrez toujours revenir à ces options lorsque vous accédez au site de Diabète en Forme [PRENOM]. N'hésitez pas à les consulter à tout moment opportun, ce pourrait réellement être de bons outils à utiliser.

Third motivational session

Bouton d'entrée		
Ce qui pourrait apparaître en dessous	-	Approfondir votre motivation par rapport à l'activité physique<br>Explorer ce qui est important pour vous et voir comment cela pourrait être lié à l'activité physique<br>Planifier votre pratique d'activités physiques (optionel)
Page 1 – Retour séance 2		
Message	S3.BACK	Avant de commencer<br>Retour sur votre dernière séance<br><br>Bonjour [PRENOM]! C'est un plaisir de vous revoir!<br><br>Avant de commencer, voici ce dont nous avons discuté principalement à la dernière séance :<br><br>Nous avons d'abord discuté de votre attitude par rapport au fait de pratiquer des activités physiques régulièrement, et ensemble, nous avons ensuite tenté de voir comment il serait possible de la rendre plus positive.<br><br>Pour ce faire, nous avons donc exploré les bénéfices qui pourraient être reliés à une pratique régulière d'activités physiqueS.Vous avez possiblement identifié ceux qui étaient plus importants pour vous et vous avez pu prendre un moment pour réfléchir sur les impacts concrets que ces bénéfices pourraient avoir dans votre vie.<br><br>Nous espérons que cette séance vous a aidé à voir plus clair et peut-être voyez-vous maintenant la pratique d'activités physiques plus positivement qu'avant [PRENOM]!<br><br>Cliquez sur continuer pour accéder à la 3ième séance lorsque vous le voudrez!
Page 2 – Introduction à la séance 3		
	S3.INTRO_VID	Bienvenue à cette séance [PRENOM]!<br><br>VIDÉO D'INTRODUCTION : « Bonjour monsieur/madame, bienvenue à cette troisième séance d'informationS.C'est Michel de Diabète en Forme qui vous parle.<br><br>Encore une fois, merci énormément d'être là. J'espère que vous avez toujours du plaisir à utiliser Diabète en Forme et que ça s'avère utile pour vouS.Je l'espère sincèrement.<br><br>Le sujet d'aujourd'hui va être un peu spécial (pause). Comme vous allez voir, on va discuter ensemble de votre motivation, mais sous un angle différent (pause).

		Concrètement, vous savez qu'il y a plusieurs raisons possibles pour lesquelles on peut pratiquer des activités physiques n'est-ce pas? Et bien, les études démontrent que certaines raisons sont plus efficaces que d'autres pour nous motiver. Prennons un exemple simple, imaginons deux personnes Linda et Martine<br><br>Parlons de Linda d'abord, Linda pratique des activités physiques de temps en temps, elle marche un peu et va au gym. Mais à chaque fois, elle a de la difficulté à se motiver, elle le fait surtout parce que les autres (son médecin, sa famille, ses amis) lui disent que c'est bon pour elle. Malgré tous les bienfaits qu'elle entend à propos de l'activité physique autour d'elle, Linda doute beaucoup que c'est vraiment bon pour elle. Elle n'éprouve pas vraiment de sentiment positif à bouger, mais en plus, elle se sent coupable lorsqu'elle n'y va paS.Rien de bon quoi!<br><br>Parlons de Martine maintenant. Martine possède ses propres raisons de pratiquer des activités physiqueS.Ce que les gens disent autour d'elle importe peu, elle est personnellement convaincue que cela apporte du positif dans sa vie, que ce soit pour sa santé ou pour d'autres raisons personnelleS.Martine a rarement de la difficulté à se motiver et elle éprouve une grande satisfaction à pratiquer des activités physiques régulièrement.<br><br>Vous voyez sans doute la différence, les raisons qui poussent Linda à faire des activités physiques proviennent de l'extérieur, alors que Martine possède des raisons en lesquelles elle croit personnellement.<br><br>Depuis le début de l'aventure, vous aurez compris que nous travaillons ensemble afin que vous puissiez développer une motivation comme celle de Martine. Que vous possédiez vos propres raisons de bouger.<br><br>Et bien la séance d'aujourd'hui viendra boucler la boucle! Précisément, nous discuterons aujourd'hui des raisons pour lesquelles vous pratiquiez des activités physiques au moment de l'inscription et on vous proposera un exercice en lien avec ceci! On va tenter de voir ce qui est important pour vous dans la vie, et on essayera de faire des liens positifs avec le fait de pratiquer des activités physiqueS.Tout ça, seulement si vous le voulez bien sûr!<br><br>J'espère que vous aimerez cette expérience. C'est tout pour moi, je vous souhaite une excellente séance et à bientôt!

	S3.OBJ_DUR	OBJECTIFS DE LA SÉANCE<br><br>Discuter de votre motivation sous un nouvel angle<br>Identifier ce qui est important pour vous dans la vie et faire des liens positifs avec la pratique d'activités physiques<br>Planifier votre pratique d'activités physiques pour les prochains jours (optionel)<br><br>DURÉE<br><br>10 à 15 minutes<br><br>POURQUOI CETTE SÉANCE?<br><br>Cette séance pourrait vous aider à développer une motivation forte, constante et positive à pratiquer des activités physiqueS5.<br><br>Nous serons déjà à mi-chemin du programme suite à cette séance [PRENOM]. Félicitations pour vos avancements encore une foiS5.<br><br>Lorsque vous le voudrez, cliquez sur continuer pour débuter la séance!
Page 3 –Reflet sur le type de motivation et discussion sur ce qui s'en vient		
	S3.INTRODU_REG	Bonjour [PRENOM],<br><br>À la première séance nous avions discuté de la force de votre motivatioS6.Vous vous souvenez? Maintenant, nous allons regarder de quoi était composée cette motivation au moment de votre inscriptioS6.Voyons voir.
	S3.REG_AMOT	Vous connaissez sans doute des bénéfices associés à la pratique d'activités physiques [PRENOM]. D'un autre côté, vous ne voyiez pas pourquoi, vous, vous devriez faire de l'activité physique au moment de l'inscriptioS6.Cela dit, peut-être avez-vous pu identifier certaines raisons personnelles de vouloir pratiquer des activités lors des premières séances et que votre motivation a changé depuiS.<br><br>Aujourd'hui [PRENOM], il s'agira de la dernière séance où nous discuterons d'aspects pouvant vous amener vers une motivation plus forte, plus constante et plus positive.<br><br>Si vous êtes partant[E], nous discuterons de vos valeurs personnelleS.Autrement dit, ce qui est important pour vous dans la vie. Ensuite, nous verrons comment pratiquer des

		activités physiques pourrait vous aider à atteindre les valeurs qui sont importantes pour vouS.<br><br>Cette activité que nous ferons ensemble peut s'avérer très utile pour amener votre motivation à un niveau supérieur, qui vous fera éprouver une plus grande satisfaction à pratiquer des activités physiqueS.<br><br>Si vous le désirez [PRENOM], cliquez sur continuer pour aller de l'avant.
	S3.REG_EXT	En fonction de vos réponses à l'inscription, il semble que vous pratiquiez des activités physiques surtout en raison de gens qui vous entourent [PRENOM]. Ce qui vous pousse à bouger, c'est le fait que les gens autour de vous vous disent que vous devriez le faire. Il est vrai que cette raison peut vous aider à pratiquer des activités physiqueS.D'un autre côté, ce type de raison n'est pas l'idéal. En fait, les gens qui pratiquent des activités physiques pour cette raison en viennent souvent à perdre leur motivation, ou alors à éprouver peu de satisfaction personnelle à en faire.<br><br>Vous avez peut-être déjà identifié des raisons plus personnelles qui vous motivent positivement lors des premières séances [PRENOM]. Cela dit, si vous êtes partant[E], nous discuterons de vos valeurs personnelles dans les prochaines minuteS.Autrement dit, de ce qui est important pour vous dans la vie. Nous tenterons de voir par la suite comment la pratique d'activités physiques pourrait vous aider à atteindre les valeurs qui sont importantes pour vouS.<br><br>Cette activité que nous ferons ensemble peut s'avérer très utile pour amener votre motivation à un niveau supérieur, qui vous fera éprouver une plus grande satisfaction à pratiquer des activités physiqueS.<br><br>Si vous le désirez [PRENOM], cliquez sur continuer pour aller de l'avant.
	S3.REG_INTRO	En fonction de vos réponses à l'inscription, il semble que vous êtes votre plus grande critique concernant votre pratique d'activités physiques [PRENOM]. Vous nous avez mentionné que vous vous sentiez coupable ou honteu[XSE] lorsque vous ne faites pas d'activités physiqueS.Il est vrai que ce type de raison peut vous aider à pratiquer des activités physiqueS.D'un autre côté, ce type de raison n'est pas l'idéal. En fait, les gens qui pratiquent des activités physiques pour cette raison en viennent souvent à perdre leur motivation, ou alors à éprouver peu de satisfaction personnelle à en faire.<br><br>Vous avez peut-être déjà identifié des raisons plus

		personnelles qui vous motivent positivement lors des premières séances [PRENOM]. Cela dit, si vous êtes partant[E], nous discuterons de vos valeurs personnelles dans les prochaines minuteS.Autrement dit, de ce qui est important pour vous dans la vie. Nous tenterons de voir par la suite comment la pratique d'activités physiques pourrait vous aider à atteindre les valeurs qui sont importantes pour vouS.<br><br>Cette activité que nous ferons ensemble peut s'avérer très utile pour amener votre motivation à un niveau supérieur, qui vous fera éprouver une plus grande satisfaction à pratiquer des activités physiqueS.<br><br>Si vous le désirez [PRENOM], cliquez sur continuer pour aller de l'avant.
	S3.REG_IDEN	En fonction de vos réponses à l'inscription [PRENOM], il semble que vous possédiez déjà des raisons personnellement importantes pour vous de pratiquer des activités physiqueS.C'est excellent. Les personnes qui ont leurs propres raisons personnelles de bouger possèdent généralement une motivation plus constante et positive en vue de pratiquer des activités physiqueS.Elles éprouvent une grande satisfaction à être activeS.<br><br>Vous avez peut-être déjà identifié d'autres raisons importantes pour vous lors des premières séances [PRENOM]. Cela dit, la séance d'aujourd'hui pourrait vous aider à en identifier davantage.<br><br>Cela dit, si vous êtes partant[E], nous discuterons de vos valeurs personnelles dans les prochaines minuteS.Autrement dit, de ce qui est important pour vous dans la vie. Nous tenterons de voir par la suite comment la pratique d'activités physiques pourrait vous aider à atteindre les valeurs qui sont importantes pour vouS.<br><br>Cette activité que nous ferons ensemble peut s'avérer très utile pour amener votre motivation à un niveau supérieur, qui vous fera éprouver une encore plus grande satisfaction à pratiquer des activités physiqueS.<br><br>Si vous le désirez [PRENOM], cliquez sur continuer pour aller de l'avant.
	S3.REG_INTEG	En fonction de vos réponses à l'inscription, il semble que vous possédiez déjà des raisons personnelles importantes pour vous de pratiquer des activités physiqueS.En fait, il semble que pratiquer des activités physiques fasse partie de vous [PRENOM], de qui vous êteS.C'est excellent. Les personnes qui ont ce type de motivation en eux possèdent généralement une motivation plus constante et positive en

		vue de pratiquer des activités physiqueS.Elles éprouvent une grande satisfaction à être activeS.<br><br>Vous avez peut-être déjà identifié d'autres raisons importantes pour vous lors des premières séances [PRENOM]. Cela dit, la séance d'aujourd'hui pourrait vous aider à en identifier davantage.<br><br>Cela dit, si vous êtes partant[E], nous discuterons de vos valeurs personnelles dans les prochaines minuteS.Autrement dit, de ce qui est important pour vous dans la vie. Nous tenterons de voir par la suite comment la pratique d'activités physiques pourrait vous aider à atteindre les valeurs qui sont importantes pour vouS.<br><br>Cette activité que nous ferons ensemble peut s'avérer très utile pour amener votre motivation à un niveau supérieur, qui vous fera éprouver une encore plus grande satisfaction à pratiquer des activités physiqueS.<br><br>Si vous le désirez [PRENOM], cliquez sur continuer pour aller de l'avant.
Page 4 – Questions sur les valeurs		
	S3.INTRO_Q.VAL	Excellent [PRENOM].<br><br>Maintenant, vous savez ce qui s'en vient et vous savez pourquoi il est pertinent de faire cet exercice.<br><br>Ci-dessous, vous trouverez une liste de valeurs diverseS.Prenez le temps que vous voulez pour examiner tous les choix [PRENOM].
	S3.Q_VAL	Parmi les valeurs ci-dessous, lesquelles sont les plus importantes pour vous [PRENOM]?<br>(sélectionnez de 2 à 10 réponses)<br><br>Choix de réponses :<br><br>Amitié – avoir des amis proches avec qui il y a un support mutuel<br>Amour – aimer, s'aimer soi-même et être aimé(e) par ceux qui m'entourent<br>Appartenance – faire partie de quelque chose, d'un groupe<br>Autorité – être responsable des autres<br>Autonomie – ne pas dépendre des autres, être indépendant(e)<br>Beauté – apprécier sa propre beauté et la beauté autour de soi<br>Compassion – être sensible aux autres et agir pour leur bien

		Compromis – être en mesure de prendre et de donner trouver un terrain d'entente<br>Confort – avoir une vie plaisante et confortable<br>Courage – être brave et fort(e) dans l'adversité<br>Créativité – créer de nouvelles choses ou de nouvelles idées<br>Croissance – évoluer continuellement<br>Curiosité – explorer, expérimenter et apprendre de nouvelles choses<br>Espoir- maintenir une attitude positive et optimiste peu importe ce qu'il advient<br>Estime de soi – se sentir bien dans sa peau<br>Famille – avoir une famille heureuse, soudée, comblée<br>Fiabilité – être fiable, être quelqu'un de confiance<br>Flexibilité – s'ajuster aux nouvelles circonstances facilement<br>Générosité – donner sans attendre de recevoir en retour<br>Intelligence – garder son mental vif et actif<br>Leadership – inspirer et guider les autres<br>Liberté – être sans restrictions, sans limitations non-désirées<br>Loyauté – être loyal(e) et vrai(e) dans ses relations<br>Maîtrise de soi – prendre les bonnes décisions, être discipliné(e)<br>Ouverture d'esprit – être ouvert(e) aux nouvelles expériences, idées, et options<br>Plaisir – avoir une vie excitante et stimulante<br>Responsabilité – prendre des décisions responsables et les maintenir<br>Sécurité – se sentir en sécurité, à l'abri des dangers<br>Service – être aidant(e) et aux services des autres
	S3.Q.OPEN_VAL	Y a-t-il une autre valeur qui vous représente que vous aimeriez partager [PRENOM]?<br>Si tel est le cas, nous vous encourageons sincèrement à l'inscrire ci-dessouS5.<br><br>(espace de réponse)<br><br>Ce que d'autres personnes atteintes du diabète ont dit :<br>« L'intégrité – vivre sa vie de manière cohérente avec mes principes et mes valeurs »<br>« La santé – être physiquement et mentalement bien »
	S3.TRANS_P4P5	Lorsque vous avez terminé, cliquez sur continuer pour avancer [PRENOM].
Page 5 – Reflet sur valeurs		

	S3.REF_VAL2	Wow [PRENOM],<br><br>Vous savez précisément ce qui est important pour vous :<br><br>[VAL1]<br>[VAL2]<br>[VAL3]<br>[VAL4]<br>[VAL5]<br>[VAL6]<br>[VAL7]<br>[VAL8]<br>[VAL9]<br>[VAL10]<br>[VAL11]<br>[VAL12]<br>[VAL13]<br>[VAL14]<br>[VAL15]<br>[VAL16]<br>[VAL17]<br>[VAL18]<br>[VAL19]<br>[VAL20]<br>[VAL21]<br>[VAL22]<br>[VAL23]<br>[VAL24]<br>[VAL25]<br>[VAL26]<br>[VAL27]<br>[VAL28]<br>[VAL29]<br>[VAL30]<br><br>Ces valeurs semblent vous tenir à cœur et faire partie intégrante de votre vie [PRENOM]. Si vous êtes d'accord, allons explorer ensemble le lien qu'elles pourraient avoir avec la pratique d'activités physiqueS.<br><br>Cliquez sur continuer pour la suite.<br>.
	S3.REF_VAL3_10	Excellent.<br><br>Il semble que plusieurs des valeurs proposées fassent partie de votre vie [PRENOM] :<br><br>[VAL1]<br>[VAL2]<br>[VAL3]<br>[VAL4]

		[VAL5]<br>[VAL6]<br>[VAL7]<br>[VAL8]<br>[VAL9]<br>[VAL10]<br>[VAL11]<br>[VAL12]<br>[VAL13]<br>[VAL14]<br>[VAL15]<br>[VAL16]<br>[VAL17]<br>[VAL18]<br>[VAL19]<br>[VAL20]<br>[VAL21]<br>[VAL22]<br>[VAL23]<br>[VAL24]<br>[VAL25]<br>[VAL26]<br>[VAL27]<br>[VAL28]<br>[VAL29]<br>[VAL30]<br><br>Ces valeurs sont honorableS.Vous pouvez en être fi(è)r(e) [PRENOM].
	S3.Q. 3P_VAL	Parmi celles-ci maintenant, pourriez-vous en choisir deux qui sont vraiment les plus importantes pour vous?<br>S6.B. Il est important de sélectionner seulement DEUX valeurs<br><br>(choix de réponses)<br><br>Cliquez sur continuer pour la suite [PRENOM].
Page 6 – Question faisant le lien entre l'activité physique et les valeurs		
	S3.VAL3P_INTRO	D'accord [PRENOM], vous avez identifié les deux valeurs les plus importantes pour vouS.[VAL_1] et [VAL_2] semblent être des valeurs qui font partie intégrante de votre vie.<br><br>Comme mentionné plus tôt, explorer le lien entre la pratique d'activités physiques et les valeurs importantes pour vous pourrait vous aider à amener votre motivation à un niveau

		supérieur. Autrement dit, cela pourrait vous aider à saisir le sens profond que pratiquer des activités physiques pourrait avoir pour vouS5.<br><br>Si vous le voulez, tentons de faire cet exercice ensemble [PRENOM]. Vous pourrez prendre le temps que vous voulez pour y réfléchir.
	S3.VAL2_INTRO	D'accord [PRENOM], nous venons de constater que [VAL_1] et [VAL_2] semblent être des valeurs importantes pour vous dans la vie.<br><br>Comme mentionné plus tôt, explorer le lien entre la pratique d'activités physiques et ces valeurs pourrait vous aider à amener votre motivation à un niveau supérieur. Autrement dit, cela pourrait vous aider à saisir le sens profond que pratiquer des activités physiques pourrait avoir pour vouS5.<br><br>L'exercice qui suit peut vous aider [PRENOM], mais garder en tête qu'il est optionnel. Nous ne voulons pas fous forcer à écrire quoi que ce soit. Vous êtes tout à fait libre.<br><br>Si vous le voulez, vous avez simplement à répondre aux questions qui suivent. N'hésitez pas à prendre le temps que vous voulez pour y réfléchir.
	S3.Q.ELAB_VAL1	[VAL_1]<br>En une phrase ou deux [PRENOM], comment, selon vous, pratiquer des activités physiques pourrait vous aider à [S3.Q.EX1]?<br><br>(espace de réponse)<br><br>Ce que d'autres personnes possédant la même valeur ont dit :<br><br>« [S3.TEM1] »
	S3.Q.ELAB_VAL2	[VAL_2]<br>En une phrase ou deux [PRENOM], comment, selon vous, pratiquer des activités physiques pourrait vous aider à [S3.Q.EX2]?<br><br>(espace de réponse)<br><br>Ce que d'autres personnes possédant la même valeur ont dit :

		« [S3.TEM2] »
	S3.Q.EX1/S3.Q.EX2<br>**MICROTAILORING**	Amitié = entretenir vos amitiés<br>Amour = aimer et être aimé(e) par ceux qui vous entourent<br>Appartenance = faire partie de quelquechose, d´un groupe<br>Autorité = vous occuper des autres<br>Autonomie = être indépendant(e) et autonome<br>Beauté = apprécier la beauté en vous et autour de vous<br>Compassion = être sensible aux autres et agir pour leur bien<br>Compromis = être en mesure de faire des compromis<br>Confort = avoir une vie plaisante et confortable<br>Courage = être brave dans l´adversité<br>Créativité = avoir des idées nouvelles, à créer<br>Croissance = évoluer continuellement<br>Curiosité = explorer et apprendre de nouvelles choses<br>Espoir = maintenir une attitude positive et optimiste peu importe ce qu´il advient<br>Estime de soi = vous sentir bien dans votre peau<br>Famille = avoir une famille comblée et heureuse<br>Fiabilité = être fiable, être quelqu´un de confiance<br>Flexibilité = vous ajuster aux nouvelles circonstances qui se présentent<br>Générosité = donner sans attendre de recevoir en retour<br>Intelligence = être une personne intelligente et à garder un mental actif<br>Leadership = inspirer et guider les autres<br>Liberté = être libre et sans limitations non-désirées<br>Loyauté = être authentique envers vous-même et dans vos relations<br>Maîtrise de soi = être à votre affaire et prendre les bonnes décisions<br>Ouverture d'esprit = être une personne ouverte d'esprit<br>Plaisir = avoir une vie excitante et stimulante<br>Responsabilité = à prendre des décisions responsables et les maintenir<br>Sécurité = vous sentir en sécurité et à l´abri de dangers<br>Service = être une personne aidante et aux services des autres
	[S3.TEM1]/<br>[S3.TEM2]<br>**MICROTAILORING**	Amitié = C´est sûr que je serais plus en forme pour être là pour mes amiS.Je serais là plus longtemps et en santé pour eux aussi.<br>Amour = Je serais vraiment content de moi. Je serais bien dans ma peau et les autres le sentiraient probablement aussi.<br>Appartenance = Je ferais partie des personnes qui ont la force de prendre leur santé en maiS6.Je montrerais l'exemple à ma famille.<br>Autorité = Je serais plus vif. J´aurais encore plus

		d´assurance pour m´occuper et être responsable des autreS5.<br>Autonomie = C´est sûr que je resterais en santé plus longtempS.Je serais moins à risque de dépendre des autres à cause qu´il m´arrive quelque chose.<br>Beauté = Je me sentirais mieux dans ma peau donc j´apprécierais encore plus tout ce qui m´entourent<br>Compassion = Je me préoccuperais moins de ma santé et j´aurais l´esprit libre pour m´occuper des autres<br>Compromis = C´est pas si excitant pour moi de bouger. D´un autre côté, ça m´aiderait c´est sûr. Ça pourrait démontrer ma capacité à faire preuve de compromis dans la vie.<br>Confort = J´aurais la conscience beaucoup plus tranquille et je me sentirais certainement mieux dans la vie de tous les jourS5.<br>Courage = J'ai le diabète oui, mais je peux me prendre en mains malgré tout. Je ferais preuve de courage en pratiquant régulièrement des activités physiques c'est sûr.<br>Créativité = Je sais que l'activité physique aide à garder un esprit vigoureux. Ça pourrait sûrement m'aider à rester inspiré constamment.<br>Croissance = Je m'assurerais de garder une bonne santé et de me tenir en forme. Juste ça serait une évolution pour moi.<br>Curiosité = J'aimerais pratiquer des activités physiques régulièrement pour voir ce qui m'arriverait, comment je me sentiraiS.Ça serait intéressant.<br>Espoir = Faire des activités physiques aide à avoir un bon mental. Ça m'aiderait à voir les choses positivement même dans des moments difficileS5.<br>Estime de soi = Je ressentirais de la fierté si je faisais des activités physiques régulièrement. C'est certaiS6.<br>Famille = Je serais fier de montrer l'exemple à ma famille. Ça pourrait aussi nous permettre de faire des activités différentes ensemble.<br>Fiabilité = J'aurais une humeur plus stable. Je serais plus discipliné pour les tâches que je dois accomplir<br>Flexibilité = Ça améliorerait mon humeur. Ça m'aiderait à réagir moins fortement à certaines choses qui me dérangent pluS5.<br>Générosité = je serais mieux dans ma peau. Le fait d'être bien m'aiderait à être une personne encore plus généreuse.<br>Intelligence = Ça m´aiderait à avoir une meilleure concentration et une meilleure attentioS6.J'aurais l'esprit plus libre.<br>Leadership = Je serais un exemple pour les personnes qui ont de la misère à pratiquer régulièrement des activités physiqueS.Je veux être un modèle de réussite.<br>Liberté = J'éviterais bien des problèmes de santé. Je ne serais pas encombré par des limitations que je pourrais

		éviter.<br>Loyauté = Je veux offrir le meilleur de moi-même aux autreS.Ça m'aiderait à rester en santé, à être encore plus positif dans la vie, et avec ceux que j'aime.<br>Maîtrise de soi = Je sais que c'est la bonne chose à faire. Faire des activités physiques m'aiderait à être plus en cohérence avec la personne que je veux vraiment être.<br>Ouverture d'esprit = Bien que l'activité physique ne m'excite pas beaucoup. Je pourrais essayer quand même et trouver des moyens d'aimer ça. Je suis sûr que ça serait bon pour moi en pluS5.<br>Plaisir = Je sais qu'on doit se sentir mieux quand on est actif. Ça m'aiderait à éprouver du plaisir plus facilement pour les petites choses de la vie.<br>Responsabilité = Je sais que c´est la bonne chose à faire pour moi. Je veux le faire pour ma santé, pour mon avenir.<br>Sécurité = Je protégerais ma santé si je faisais des activités physiques régulièrement. J'augmente fortement mes chances de vivre une vie plus en santé.<br>Service = En m´occupant de moi avant tout, je pourrai m´occuper du bien des autres beaucoup mieux et beaucoup plus longtempS5.
	S3.TRANS_P5P6	Lorsque vous avez terminé, cliquez sur continuer pour la suite!
Page 7 – Reflet sur les liens valeur-activité physique		
	S3.REF_ELA0	D'accord [PRENOM], vous avez peut-être seulement réfléchi au lien que la pratique d'activités physiques pourraient avoir avec les valeurs qui sont importantes pour vouS5.<br><br>Avant de résumer la séance d'aujourd'hui, nous aimerions une fois de plus vous partager un peu d'informationS.Celles-ci seront en lien avec le sujet d'aujourd'hui et ce sera plutôt bref.
	S3.REF_ELA1	Bien [PRENOM]. Vous semblez faire des liens positifs entre la pratique d'activités physiques et au moins une de vos valeurs qui vous représentent bieS6.<br><br>Avant de résumer la séance d'aujourd'hui, nous aimerions une fois de plus vous partager un peu d'informationS.Celles-ci seront en lien avec le sujet d'aujourd'hui et ce sera plutôt bref.
	S3.REF_ELA2	Wow [PRENOM], vous semblez faire des liens positifs entre la pratique d'activités physiques et les valeurs qui sont importantes pour vouS5.

		Avant de résumer la séance d'aujourd'hui, nous aimerions une fois de plus vous partager un peu d'informationS.Celles-ci seront en lien avec le sujet d'aujourd'hui et ce sera plutôt bref.
	S3.Q.INFO	Aimeriez-vous recevoir ces informations?
Page 8 -Informations		
	S3.INFOF	Quelques informations, pour vous<br><br>L'exercice que vous venez de faire est excellent pour développer une motivation positive et durable en vue de pratiquer des activités physiques<br><br>La majorité des femmes atteintes du diabète de type 2 qui sont régulièrement actives éprouvent une grande satisfaction à le faire. Ce n'est cependant pas pour aucune raisoS6.En fait, comme vous, elles se sont attardées à faire des liens positifs entre le fait d'être actives régulièrement et les choses qui leur sont réellement importantes dans la vie.<br><br>En faisant cette réflexion, elles ont donc personnellement réalisé que pratiquer des activités physiques est un facteur important pour être intègre à leurs valeurs et à elles-mêmeS.La plupart vous diraient qu'elles en sont fièreS5.<br><br>L'exercice que vous venez de faire peut s'avérer être une étape essentielle en vue de devenir active pour la vie et être heureuse de l'être [PRENOM].<br><br>Une fois que vous verrez les liens entre le fait de pratiquer régulièrement des activités physiques et les choses qui sont importantes pour vous dans la vie, il ne vous suffira que de trouver des activités physiques qui vous plaisent et dans lesquelles vous vous sentez confiante.<br><br>À propos des activités physiques qui vous plaisent et votre confiance face au fait d'effectuer régulièrement des activités physiques, assurez-vous que nous discuterons de ces aspects dans les prochaines séanceS.L'outil « Plan d'action » peut également vous aider à ces niveaux.<br><br>Cliquez sur continuer pour accéder au résumé de la séance d'aujourd'hui [PRENOM].
	S3.INFOH	Quelques informations, pour vous

		L'exercice que vous venez de faire est excellent pour développer une motivation positive et durable en vue de pratiquer des activités physiqueS.Les études le démontrent.<br><br>La majorité des hommes atteints du diabète de type 2 qui sont régulièrement actifs éprouvent une grande satisfaction à le faire. Ce n'est cependant pas pour aucune raisoS6.En fait, comme vous, ils se sont attardés à faire des liens positifs entre le fait d'être actifs régulièrement et les choses qui leur sont réellement importantes dans la vie.<br><br>En faisant cette réflexion, ils ont donc personnellement réalisé que pratiquer des activités physiques est un facteur important pour être intègre à leurs valeurs et à elles-mêmeS.La plupart vous diraient qu'elles en sont fièreS5.<br><br>L'exercice que vous venez de faire peut s'avérer être une étape essentielle en vue de devenir actif pour la vie et être heureux de l'être [PRENOM].<br><br>Une fois que vous verrez les liens entre le fait de pratiquer régulièrement des activités physiques et les choses qui sont importantes pour vous dans la vie, il ne vous suffira que de trouver des activités physiques qui vous plaisent et dans lesquelles vous vous sentez confiante.<br><br>À propos des activités physiques qui vous plaisent et votre confiance face au fait d'effectuer régulièrement des activités physiques, assurez-vous que nous discuterons de ces aspects dans les prochaines séanceS.L'outil « Plan d'action » peut également vous aider à ces niveaux.<br><br>Voilà [PRENOM], nous espérons avoir éclairci certains aspects en vous faisant part de ces informationS5.<br><br>Cela dit, avant de continuer, comment avez-vous trouvé ces informations?<br><br>Choix :<br>1 – J'ai aimé<br>2 – Je n'ai pas vraiment aimé<br>3 – Je ne sais pas trop<br><br>Cliquez ensuite sur continuer pour accéder au résumé de la séance d'aujourd'hui [PRENOM].

Page 9 – Résumé de la séance		
	S3.REF_INFO1	Merci de le partager [PRENOM].<br><br>Il semble que vous ayez apprécié les informations précédentes! Nous sommes heureux de l'entendre.<br><br>Voici maintenant votre résumé de la séance d'aujourd'hui.<br><br>Merci encore et félicitations pour avoir franchi une autre étape du programme
	S3.REF_INFO2	Mmmm…<br><br>Il semble que les informations précédentes n'ont pas satisfait vos attentes [PRENOM]. Merci de le partager. Nous espérons pouvoir vous fournir les informations dont vous avez besoin à travers les prochaines activités de Diabète en Forme.<br><br>Voici malgré tout un résumé de votre séance d'aujourd'hui.<br><br>Merci encore et félicitations pour avoir franchi une autre étape du programme.
	S3.REF_INFO3	Mmmmm…<br><br>Vous ne savez pas trop quoi penser des informations précédentes [PRENOM]. Nous espérons qu'elles puissent malgré tout vous aider à prendre des décisions plus éclairéeS5.<br><br>Pour le moment, voici quand même un résumé de votre séance d'aujourd'hui [PRENOM].<br><br>Merci encore et félicitations pour avoir franchi une autre étape du programme.
	S3.RES_REG.AM	RÉSUMÉ DE LA SÉANCE, POUR VOUS<br><br>Voici ce que nous retenons de la séance d'aujourd'hui [PRENOM]. Nous avons d'abord tenté d'explorer ce qui expliquait votre motivation au moment de l'inscriptioS6.En fait, à ce moment, vous ne sembliez pas vraiment voir pourquoi vous devriez pratiquer des activités physiqueS.Ainsi, pour complémenter les premières séances, nous vous avons donc proposé un exercice qui possède le potentiel de vous amener vers une motivation

		plus forte et plus positive.
	S3.RES_REG.EXT	RÉSUMÉ DE LA SÉANCE, POUR VOUS<br><br>Voici ce que nous retenons de la séance d'aujourd'hui [PRENOM]. Nous avons d'abord tenté d'explorer ce qui expliquait votre motivation au moment de l'inscriptioS6.À ce moment, il semble que vous pratiquiez des activités physiques surtout parce que les gens autour de vous vous disent de le faire. Ainsi, pour complémenter les premières séances, nous vous avons donc proposé un exercice qui possède le potentiel de vous amener vers une motivation plus personnelle, plus forte et plus positive.
	S3.RES_REG.INTRO	RÉSUMÉ DE LA SÉANCE, POUR VOUS<br><br>Voici ce que nous retenons de la séance d'aujourd'hui [PRENOM]. Nous avons d'abord tenté d'explorer ce qui expliquait votre motivation au moment de l'inscriptioS6.À ce moment, il semble que vous pratiquiez des activités physiques surtout parce que vous vous sentez coupable de ne pas en faire. Ainsi, pour complémenter les premières séances, nous vous avons donc proposé un exercice qui possède le potentiel de vous amener vers une motivation plus personnelle, plus forte et plus positive.
	S3.RES_REG.IDEN	RÉSUMÉ DE LA SÉANCE, POUR VOUS<br><br>Voici ce que nous retenons de la séance d'aujourd'hui [PRENOM]. Nous avons d'abord tenté d'explorer ce qui expliquait votre motivation au moment de l'inscriptioS6.À ce moment, il semble que vous aviez des raisons personnelles de faire des activités physiqueS.Lorsque les raisons de pratiquer des activités physiques viennent de vous, il s'agit là d'une excellente source de motivatioS6.Ainsi, pour complémenter les premières séances, nous vous avons donc proposé un exercice qui possède le potentiel de vous amener vers une motivation encore plus forte et plus positive. Possedez
	S3.RES_REG.INTEGR	RÉSUMÉ DE LA SÉANCE, POUR VOUS<br><br>Voici ce que nous retenons de la séance d'aujourd'hui [PRENOM]. Nous avons d'abord tenté d'explorer ce qui expliquait votre motivation au moment de l'inscriptioS6.À ce moment, il semble que vous pratiquiez des activités physiques parce que cela fait partie de vous, de qui vous êteS.C'est excellent. Les personnes qui possèdent ce type de motivation restent généralement motivées au fil du temps et éprouvent beaucoup de satisfactions à être

		activeS.Ainsi, pour complémenter les premières séances, nous vous avons donc proposé un exercice qui possède le potentiel de vous amener vers une motivation encore plus forte et plus positive.
	S3.RES_VAL	À travers l'exercice, vous avez identifié des valeurs qui sont importantes pour vous dans la vie. Voici les deux plus importantes :<br><br>[VAL_1]<br>[VAL_2]<br><br>Vous avez ensuite eu la possibilité de voir comment la pratique d'activités physiques pourrait vous aider à mieux atteindre ces valeurS.
	S3.RES_APVAL0	Vous n'avez cependant rien mentionné à ce sujet. Comment le fait de pratiquer des activités physiques pourrait vous aider à [S3.Q.EX1]? Comment le fait de pratiquer des activités physiques pourrait vous aider à [S3.Q.EX2]? Vous y avez peut-être déjà réfléchi [PRENOM].
	S3.RES_APVAL1	Voici comment, pour vous, pratiquer des activités physiques pourrait vous aider à [S3.Q.EXCHOICE1] : « [S3.Q.CHOICE] ». Comment le fait de pratiquer des activités physiques pourrait vous aider à [S3.Q.EXCHOICE2]?
	S3.RES_APVAL2	Voici comment, pour vous, pratiquer des activités physiques pourrait vous aider à [S3.Q.EX1] : « [S3.Q.ELA1_VAL] ». Et voici comment, pour vous, pratiquer des activités physiques pourrait vous aider à [S3.Q.EX2] : « [S3.Q.ELA2] ».
	S3.RES5.QOPEN	De plus, vous avez partagé une autre valeur qui était importante pour vous [PRENOM] : « S3.Q.OPENVAL ». Encore une fois, comment la pratique d'activités physiques pourrait vous aider à mieux atteindre cette valeur?
	S3.RES5.MERCI	Voilà ce que nous retenons de la séance d'aujourd'hui. Nous espérons que cet exercice vous a servi [PRENOM] .<br><br>Merci pour cette discussion
	S3.RES5.AVENIR	Encore bravo [PRENOM] . Dans la prochaine séance, nous discuterons de votre niveau de confiance concernant votre capacité à pratiquer régulièrement des activités physiqueS.<br><br>À travers cette séance, nous tenterons d'identifier vos plus grandes forces et nous verrons comment elles pourront vous aider dans votre démarche entourant la pratique d'activités physiqueS.Tout ça, si vous le voulez bien sûr.

		Cliquez maintenant sur continuer pour accéder à la dernière page [PRENOM] !
Page 10 – Plan d'action/redirection		
	S3.INTRO_PLAN	Vers où tout cela vous mène?<br><br>Comme aux séances précédentes, plusieurs choix s'offrent maintenant à vous [PRENOM]. Sentez-vous absolument libre de choisir ce qui vous convient. Vous le savez mieux que nouS5.
	S3.Q.PLAN	Qu'aimeriez-vous faire maintenant?*<br><br>1- Je veux planifier mes activités physiques pour les prochains jours<br><br>2- Je vois des choses qui m'empêchent de faire plus d'activités physiques, j'aimerais en discuter<br><br>3- Je veux tout simplement quitter pour l'instant (si tel est le cas, au plaisir de vous revoir bientôt!)<br><br>*Vous pourrez toujours revenir à ces options lorsque vous accédez au site de Diabète en Forme [PRENOM]. N'hésitez pas à les consulter à tout moment opportun, ce pourrait réellement être de bons outils à utiliser.

Fifth motivational session

Page 1 – Retour sur la séance 3		
	S5.RETOUR	AVANT DE COMMENCER<br>RETOUR SUR LA DERNI ÈRE SÉANCE<br><br>Bonjour [PRENOM], c'est un plaisir de vous revoir!<br><br>À la dernière séance, nous avons convenu que certains types de motivation pourrait être mieux que les autres afin d'éprouver une plus grande satisfaction à pratiquer des activités physiques régulièrement.<br><br>Pour favoriser une motivation positive en vous, nous avons donc tenté d'examiner comment le fait de pratiquer des activités physiques pourrait vous aider à mieux atteindre les valeurs qui sont importantes pour vous dans la vie. En effet, les personnes qui sont en mesure de faire le pont entre la pratique d'activités physiques et ce qui leur est important dans la vie éprouvent une satisfaction profonde à être activeS.<br><br>Nous espérons que cette dernière séance vous a été utile

		[PRENOM] !<br><br>Ce sera un plaisir de discuter à nouveau avec vous aujourd'hui au travers de la séance qui suit.<br><br>Lorsque vous êtes prêt[E], cliquez sur continuer pour avancer!<br><br>L'équipe de Diabète en Forme
Page 2 – Introduction à la séance 4		
	S5.INTRO_VIDEO	Bienvenue à votre 4ième séance [PRENOM]!<br><br>Message vidéo : Bonjour monsieur/madame, c'est encore Michel de Diabète en Forme qui vous parle. Vous êtes déjà rendu à votre 4ième séance d'informations aujourd'hui. Le temps passe vite! J'espère que vous aimez toujours votre expérience sur le programme de Diabète en Forme.<br><br>Vous serez peut-être d'accord avec le fait qu'il est difficile de maintenir de bonnes habitudes en matière d'activités physiqueS.En effet, plusieurs personnes atteintes du diabète de type 2 abandonnent parfois très rapidement leurs bonnes habitudes pour différentes raisonS.Une d'entre elles peut être que la pratique d'activités physiques demande un effort constant, faisant en sorte que certaines personnes ne sentent pas posséder la confiance ou la force nécessaire afin de réussir à maintenir leur mode de vie régulièrement actif.<br><br>Il est vrai que de devenir actif ou active régulièrement demande un effort constant, presque à chaque jour, tout au long de votre vie. D'un autre côté, il existe des solutions pour trouver la force de réussir ce changement dans votre vie. Si vous le voulez, nous tenterons une d'entre elles ensemble aujourd'hui! Nous explorerons ensemble vos forces et vos qualités, et nous tenterons de voir comment celles-ci pourraient vous faire voir qu'il est possible pour vous de pratiquer des activités physiques régulièrement.<br><br>Vous verrez, c'est une activité très positive. Si je le pouvais, je ferais faire cet exercice à toute la planète entière!<br><br>C'est déjà tout pour moi! J'espère que vous aimerez, vraiment.<br><br>À bientôt et merci encore!
	S5.OBJECTIFS_DETAILS	OBJECTIFS DE LA SÉANCE<br><br>Discuter de votre niveau de confiance en votre capacité de pratiquer régulièrement des activités physiques<br><br>Explorer les forces et les qualités qui vous correspondent

		Planifier vos activités physiques pour les prochains jours et identifier des solutions aux barrières qui pourraient vous empêcher d'être plus acti[FVE] (optionel)<br><br>DURÉE DE LA SÉANCE<br><br>10 à 15 minutes<br><br>Ce que vous pouvez retirer de cette séance?<br>Une plus grande confiance en votre capacité de réussir à pratiquer des activités physiques régulièrement.<br><br>Lorsque vous êtes prêt[E] [PRENOM], cliquez sur continuer pour aller de l'avant.
Page 3 – Reflet sur Efficacité Personnelle		
	S5.EFF_LOW	Votre niveau de confiance<br><br>Selon vos réponses à l'inscription [PRENOM], il semble que vous n'étiez pas vraiment confiant[E] en votre capacité de pratiquer des activités physiques régulièrement au cours du prochain moiS.Si tel est toujours le cas, sachez que vous n'êtes pas la seule personne. Quels sont les éléments qui pourraient vous aider à croire davantage en votre capacité de pratiquer régulièrement des activités physiques [PRENOM]?<br><br>En fait, bien des personnes atteintes du diabète de type 2 se trouvent dans cette situatioS6.Ce qui est bien, c'est que plusieurs personnes ont également su devenir plus confiantes en leur capacité en effectuant le même type d'exercice que vous ferez aujourd'hui. Vous en êtes donc assurément capable vous aussi [PRENOM]!<br><br>Si vous le voulez, nous tenterons aujourd'hui d'identifier ensemble les différentes forces qui vous correspondent en tant que personne, et qui seront en mesure de vous faire progresser dans votre pratique d'activités physiques au quotidieS6.<br><br>Cliquez sur continuer pour avancer [PRENOM]!
	S5.EFF_MIDLOW	Votre niveau de confiance<br><br>Selon vos réponses à l'inscription, il semble que vous aviez peu confiance en votre capacité de pratiquer régulièrement des activités physiques au cours du prochain moiS.Si tel est toujours le cas [PRENOM], sachez que vous n'êtes pas la seule personne. D'un autre côté, vos réponses indiquent également que vous n'êtes pas complètement inconfiant[E]. Quels sont les éléments qui alimentent ce brin de confiance en votre capacité de réussir [PRENOM]?

		En fait, bien des personnes atteintes du diabète de type 2 se trouvent dans cette situatioS6.Ce qui est bien, c’est que plusieurs personnes ont également su devenir plus confiantes en leur capacité en effectuant le même type d’exercice que vous ferez aujourd’hui. Vous en êtes donc assurément capable vous aussi [PRENOM]!<br><br>Si vous le voulez, nous tenterons aujourd’hui d’identifier ensemble les différentes forces qui vous correspondent en tant que personne, et qui pourraient s’avérer être utiles afin de vous faire progresser dans votre pratique d’activités physiques au quotidieS6.<br><br>Cliquez sur continuer pour avancer [PRENOM]!
	S5.EFF_MID HIGH	Votre niveau de confiance<br><br>Selon vos réponses à l’inscription, voici ce que nous comprenons [PRENOM]. Bien que certains facteurs vous retiennent d’être plus confiance, vous semblez avoir une bonne confiance en votre capacité de pratiquer des activités physiques régulièrement au cours du prochain moiS.C’est excellent! Quels sont les éléments qui alimentent votre confiance [PRENOM]?<br><br>Si vous le voulez, nous tenterons aujourd’hui d’identifier ensemble les différentes forces qui vous correspondent en tant que personne, et qui pourraient s’avérer être utiles afin de vous faire progresser dans votre pratique d’activités physiques au quotidieS6.<br><br>Cliquez sur continuer pour avancer [PRENOM]!
	S5.EFF_HIG H	Votre niveau de confiance<br><br>Selon vos réponses à l’inscription, vous semblez avoir une forte confiance en votre capacité de pratiquer des activités physiques régulièrement [PRENOM]. C’est excellent! La plupart des personnes atteintes du diabète de type 2 ne possèdent pas une aussi forte confiance que vouS.Quels sont donc les éléments qui alimentent cette confiance [PRENOM]?<br><br>Si vous le voulez, nous tenterons aujourd’hui d’identifier ensemble les différentes forces qui vous correspondent en tant que personne, et qui pourraient s’avérer être utiles afin de vous faire progresser dans votre pratique d’activités physiques au quotidieS6.<br><br>Cliquez sur continuer pour avancer [PRENOM]!
Page 4 – Questions sur les forces de la personne		
	S5.INTRO_Q 1	D’accord [PRENOM], voici la première partie de l’exercice d’aujourd’hui!

		Les études démontrent que cet exercice peut être efficace pour gagner de la confiance en votre capacité de pratiquer des activités physiques régulièrement.<br><br>L'objectif est d'identifier les forces et les qualités qui vous correspondent d'abord. Par la suite, vous tenterons de voir comment les qualités qui vous correspondent pourraient vous aider à réussir à pratiquer davantage d'activités physiqueS5.<br><br>Cela dit, vous trouverez ci-dessous une liste de forces et de qualités déjà mentionnées par d'autres personnes atteintes du diabète de type 2 [PRENOM].
	S5.Q1	Parmi cette liste, lesquelles de ces forces ou qualités vous représentent le mieux?<br>(sélectionnez de 2 à 8 réponses)<br><br>-Aimant(e)– vous démontrez beaucoup d'amour pour les autres<br>-Attentif/Attentive– vous êtes conscient(e) de vos besoins et ceux des autres<br>-À l'écoute – vous prenez le temps d'écouter, de bien comprendre les gens<br>-Capable – vous possédez une confiance en votre capacité à réussir ce que vous entreprenez généralement<br>-Compétent(e) – vous êtes doué(e) dans la plupart des activités que vous effectuez<br>-Confiant(e) – vous croyez en vous, même dans des circonstances difficiles<br>-Décidé(e) – lorsque vous prenez des décisions, vous ne revenez pas en arrière<br>-Déterminé(e) – lorsque vous commencez un projet, vous en venez à bout<br>-Dévoué(e) – vous vous donnez corps et âme pour obtenir ce que vous voulez<br>-Efficace – vous effectuez vos activités de façon logique et assidue<br>-Énergique – vous avez beaucoup d'énergie à offrir<br>-Enjoué(e) – vous avez souvent le sourire aux lèvres<br>-Gagnant(e) – vous réussissez constamment ce que vous entreprenez<br>-Mature – vous êtes responsable et prenez de bonnes décisions pour vous et les autres<br>-Organisé(e) – vous planifiez et vous effectuez les choses dans l'ordre<br>-Oseux/Oseuse – vous foncez et vous explorez souvent de nouvelles choses<br>-Persévérant(e) – vous n'abandonnez pas lorsque c'est difficile<br>-Prévoyant(e) – vous anticipez ce qui s'en vient et vous agissez en conséquence<br>-Reconnaissant(e) – vous reconnaissez la chance que vous avez d'être là où vous êtes aujourd'hui<br>-Raisonnable – vous appréciez les bonnes choses de façon

		modérée<br>- Rempli(e) de ressources – vous possédez beaucoup de ressources pour surmonter des difficultés en cas de besoin<br>- Sage – vous prenez le temps de bien peser le pour et le contre de chaque situation et prenez la bonne décision<br>- Vaillant(e) – vous n'hésitez pas à faire ce qui doit être fait, et ce, malgré un manque de motivatioS6.<br>- Visionnaire – vous savez clairement où vous allez et vous êtes confiant(e) d'y arriver<br>- Vrai(e) – vous êtes fidèle à vous-même, à vos valeurs, à vos principes
	S5.Q2_OPEN	Y a-t-il d'autres forces ou qualités qui vous décrivent bien [PRENOM]?<br>Si vous avez un élément en tête, nous vous encourageons à l'inscrire ci-dessouS5.<br><br>(espace de réponses)<br><br>Ce que d'autres personnes atteintes du diabète de type 2 ont dit :<br>Je suis courageux/courageuse : je n'ai pas peur de réaliser ce qui est difficile<br><br>Cliquez sur continuer lorsque vous avez fait vos choix [PRENOM]!
Page 5 – Reflet sur les forces sélectionnées		
	S5.REF_STR2<br><br>*S5.STR signifie que les forces que la personne a sélectionnée seront présentées tel que dans la question S5.Q1	Excellent [PRENOM], vous avez trouvé des qualités bien précises qui vous correspondent davantage. Les voici :<br>[S5.STR1]*<br>[S5.STR2]<br>[S5.STR3]<br>[S5.STR4]<br>[S5.STR5]<br>[S5.STR6]<br>[S5.STR7]<br>[S5.STR8]<br>[S5.STR9]<br>[S5.STR10]<br>[S5.STR11]<br>[S5.STR12]<br>[S5.STR13]<br>[S5.STR14]<br>[S5.STR15]<br>[S5.STR16]<br>[S5.STR17]<br>[S5.STR18]<br>[S5.STR19]<br>[S5.STR20]<br>[S5.STR21]<br>[S5.STR22]<br>[S5.STR23]

		[S5.STR24]<br>[S5.STR25]<br><br>Vous semblez [S5.STR_VALORI1] et [S5.STR_VALORI2]. C'est inspirant [PRENOM], bravo pour ces qualités qui vous habitent.
	[S5.STR_VALORI1]<br>Et<br>[S5.STR_VALORI2]<br>**microtailoring**	Aimant(e) = être une personne avec beaucoup d'amour à offrir<br>Attentif/Attentive = être une personne prête à aider les autres<br>À l'écoute = être une bonne oreille pour les autres<br>Capable = être une personne qui croyez en votre capacité d'accomplir toutes choses<br>Compétent(e) = être une personne qui soit douée dans plusieurs domaines<br>Confiant(e)= être une personne qui possède une forte confiance en vous<br>Décidé(e)= être une personne qui regarde droit devant<br>Déterminé(e)= être une personne qui vient à bout de ce qu'elle entreprend<br>Dévoué(e)= être une personne qui donne tout ce qu'elle a pour réussir<br>Efficace = être une personne assidue qui accompli toute activité avec briau<br>Énergique=être une personne vivante qui a beaucoup d'énergie<br>Enjoué(e)= être une personne avec une bonne humeur contagieuse<br>Gagnant(e)= être une personne qui finit toujours par réussir<br>Mature = être une personne qui prend des décisions responsables<br>Organisé(e)= être une personne qui planifie efficacement ses activités<br>Oseux/Oseuse= être une personne qui n'hésite pas à vivre de nouvelles expériences<br>Persévérant(e) = être une personne qui fonce malgré les difficultés<br>Prévoyant(e) = être une personne qui agit en pensant à long terme<br>Reconnaissant(e)=être une personne qui apprécie sa situation présente<br>Raisonnable= être une personne qui sait se modérer<br>Rempli(e) de ressources= être une personne qui possède beaucoup de moyens pour contrer des situations indésirables<br>Sage= être une personne qui réfléchit bien avant d'agir<br>Vaillant(e)= être une personne qui fait ce qui doit être fait sans hésitation<br>Visionnaire= être une personne qui sait clairement vers où elle se dirige dans la vie<br>Vrai(e)=être une personne intègre, fidèle à vos principes

	S5.REF_STROPEN	Vous avez également écrit quelque chose dans l'espace libre nous y reviendrons plus tard.
	S5.REF_TRANSITIONP5P6	Si vous êtes partant[E], tentons de voir ensemble maintenant comment vos qualités pourraient vous aider à pratiquer davantage d'activités physiques [PRENOM].<br><br>Cliquez sur continuer pour la suite.
	S5.REF_STR310	Excellent [PRENOM]! Vous avez trouvé plusieurs forces et qualités qui vous correspondent bieS6.Si vous avez écrit quelque chose dans l'espace libre, nous y reviendrons plus tard! Pour le moment, voici les forces que vous avez sélectionnées :<br><br>[S5.STR1]*<br>[S5.STR2]<br>[S5.STR3]<br>[S5.STR4]<br>[S5.STR5]<br>[S5.STR6]<br>[S5.STR7]<br>[S5.STR8]<br>[S5.STR9]<br>[S5.STR10]<br>[S5.STR11]<br>[S5.STR12]<br>[S5.STR13]<br>[S5.STR14]<br>[S5.STR15]<br>[S5.STR16]<br>[S5.STR17]<br>[S5.STR18]<br>[S5.STR19]<br>[S5.STR20]<br>[S5.STR21]<br>[S5.STR22]<br>[S5.STR23]<br>[S5.STR24]<br>[S5.STR25]<br><br>Vous êtes une personne remplie de qualités [PRENOM]. Avant d'aller plus loin, tentons de voir quelles de vos qualités sont celles qui vous correspondent plus que toutes les autreS5.
	S5.Q_STR310	Parmi celles que vous avez choisies, quelles seraient donc les <u>deux plus importantes forces </u>chez vous?<br>S6.B. Il est important de sélectionner SEULEMENT DEUX forces [PRENOM]. Autrement, le programme sera déréglé et nous ne pourrons continuer.

		Choix de réponse « tailored » en fonction des réponses à S5.Q1<br><br>Cliquez sur continuer pour la suite [PRENOM].
Page 6 – Reflet pour ceux ayant choisi 3 forces et plus		
	S5.REF310<br><br>*Similaire à [S5.STR_VALORI1] et [S5.STR_VALORI2]	D'accord! Vous avez trouvé deux forces plus importantes pour vous [PRENOM]. Vous semblez [S5.STR310_VALORI1]* et [S5.STR310_VALORI2]. C'est inspirant [PRENOM], bravo pour ces qualités qui vous habitent.<br><br>Si vous êtes partant[E], tentons de voir ensemble maintenant comment ces qualités pourraient vous aider à faire plus d'activités physiques [PRENOM].<br><br>Si vous le voulez, cliquez sur continuer pour la suite.
	S5.ERREUR_SUP	OUPS! [PRENOM], vous avez sélectionné plus de deux forces à la question précédente. Il vous faut sélectionner exactement DEUX de vos forceS5.<br><br>Cliquez sur précédent pour revenir en arrière et ajustez vos réponseS5.<br><br>Autrement, le programme sera déréglé et vous ne pourrez pas finir la séance normalement!
	S5.ERREUR_INF	OUPS! [PRENOM], vous avez sélectionné moins de deux de vos forces à la question précédente. Il vous faut sélectionner exactement DEUX de vos forceS.<br><br>Cliquez sur précédent pour revenir en arrière et ajustez vos réponses [PRENOM].<br><br>Autrement, le programme sera déréglé et vous ne pourrez pas finir la séance normalement!
Page 7 – Élaboration sur les forces		
	S5.ELAB_INTRO	Bien! Nous sommes heureux de faire l'exercice qui suit avec vous [PRENOM].<br><br>Le saviez-vous? Les études démontrent que d'affirmer nos propres forces et de créer des liens entre celles-ci et la pratique régulière d'activités physiques peut aider à améliorer grandement votre confiance en votre capacité d'y arriver. Si vous le voulez, tentons donc l'exercice ensemble.<br><br>Vous connaissez sans doute déjà la formule après les dernières séanceS.Répondez simplement aux questions qui suivent [PRENOM]. Prenez le temps que vous voulez pour réfléchir à vos réponseS5.

	S5.Q4	En une phrase ou deux, comment le fait d'[S5.STR _VALORI1] pourrait vous donner la force de pratiquer régulièrement des activités physiques [PRENOM]?<br><br>(espace de réponse)<br><br>Ce que d'autres personnes possédant la même force que vous ont dit :<br>« [S5.EX_STR1] »
	S5.Q5	En une phrase ou deux, comment le fait d'[S5.STR _VALORI2] pourrait vous donner la force de pratiquer régulièrement des activités physiques [PRENOM]?<br><br>(espace de réponse)<br><br>Ce que d'autres personnes possédant la même force que vous ont dit :<br>« [S5.EX_STR2] »
	[S5.EX_STR1]<br>Et<br>[S5.EX_STR2]<br>**microtailoring**	Aimant(e) = Je veux donner de l'amour le plus longtemps possible aux autreS.Je dois rester en santé.<br>Attentif/Attentive = Je dois être attentif à moi-même d'abord si je veux aider les autreS.<br>À l'écoute = Je dois être à l'écoute des autres, mais aussi de moi-même. Je dois m'écouter et faire ce qui est bon pour moi.<br>Capable = Je sais que je suis capable d'y arriver. J'ai réussi des choses aussi dures ou sinon plus que ça.<br>Compétent(e) = Je suis douée dans plusieurs choseS.Je ne vois pas pourquoi pratiquer des activités physiques devrait être différent.<br>Confiant(e)= J'ai confiance en moi. Je n'ai qu'à prendre une décision ferme que je vais devenir actif régulièrement et je vais y arriver.<br>Décidé(e)= Je reviens rarement en arrière lorsque je décide de faire un projet. Si je décide de devenir active régulièrement, je vais y arriver et maintenir l'habitude.<br>Déterminé(e)= Je vais toujours au bout de mes projetS.Ce devrait être la même chose avec la pratique d'activités physiqueS5.<br>Dévoué(e)= Je fais tout ce qu'il faut pour réussir ce que j'entreprendS.Je dois faire la même chose avec l'activité physique. Je vais le faire.<br>Efficace = Je pourrais organiser mon horaire pour intégrer des activités physiqueS.Je suis certainement assez efficace pour faire ça.<br>Énergique= Je pourrais dépenser une partie de mon énergie dans ma santé. C'est sûr que j'ai assez d'énergie pour pratiquer des activités physiqueS.Je suis capable.<br>Enjoué(e)= Je peux maintenir mon humeur positive lorsque vient le temps de pratiquer des activités physiqueS.L'activité physique c'est positif et ça me ferait plaisir d'en faire.<br>Gagnant(e)= Je performe dans tout ce que j'entreprendS.Devenir actif régulièrement ne sera pas une exceptioS6.

		Mature = Si j’use de mon jugement, je sais quelle est la bonne chose à faire. Je veux devenir active régulièrement<br>Organisé(e)= Intégrer des activités physiques à mon horaire ça serait facile pour moi. Je vais le faire, il faut juste que je pratique des activités simples comme la marche ou la natatioS6.<br>Oseux/Oseuse= Ce n’est pas mon genre de ne pas essayer. Pratiquer des activités physiques régulièrement est un beau défi à relever. Le relever serait une expérience vraiment excitante.<br>Persévérant(e) = Malgré ce qui pourrait m’empêcher de faire des activités physiques, cela ne devrait pas m’arrêter. C’est vrai que je suis persévérant, je vais y arriver, je vais devenir actif.<br>Prévoyant(e) = Je connais les bénéfices de pratiquer des activités physiques régulièrement et les conséquences de ne pas le faire. Tout penche pour que j’en fasse. Ça me correspond de devenir une personne active.<br>Reconnaissant(e)= Je suis chanceuse de pouvoir prendre soin de moi de façon autonome. Il faut que ça continue comme ça. Je veux devenir plus active<br>Raisonnable= J’aime les bonnes choses de la vie, mais je suis capable de faire la part des choseS.Je veux devenir actif, c’est bon pour moi. Je vais y arriver.<br>Rempli(e) de ressources= J’ai tout ce qu’il faut pour devenir une personne active. J’ai de l’argent, une famille et des amis qui me supporteraient et m’encourageraient. Je peux y arriver.<br>Sage=Si je pèse les pour et les contres, c’est beaucoup plus avantageux pour moi de devenir actif régulièrement. C’est la plus sage décision que je pourrais prendre dans ma vie.<br>Vaillant(e)= Je ne recule pas devant un défi. Devenir actif régulièrement est seulement un autre défi à relever. Je suis capable de le faire.<br>Visionnaire=Je sais où je veux aller, et devenir actif régulièrement doit faire partie du chemiS6.Je vais trouver une façon d’y arriver je n’abandonnerai jamaiS5.<br>Vrai(e) =J’ai toujours été honnête envers moi et mes besoinS.Je ne nie pas que de devenir actif régulièrement serait très bon pour moi. Je veux le devenir. Je suis capable.
	S5.TRANSITIONP7P8	Cliquez sur continuer lorsque vous avez terminé [PRENOM].
Page 8 – Reflet sur élaboration		
	S5.REF_ELABO2	D’accord [PRENOM], vous n’avez rien inscrit aux questions précédenteS.Vous avez peut-être seulement réfléchi à comment vos qualités pourraient vous être utile afin de pratiquer des activités physiques régulièrement.<br><br>Fidèles à nos habitudes, avant de résumer la séance, nous pensons posséder quelques informations supplémentaires qui pourraient vous intéresser [PRENOM].
	S5.REF_ELAB12	Merci de partager ces informations avec nous [PRENOM]. Il semble que vous voyez comment au moins une de vos grandes qualités pourrait vous aider à pratiquer des activités physiques

		plus régulièrement. Voilà une bonne nouvelle!<br><br>Fidèles à nos habitudes, avant de résumer la séance, nous pensons posséder quelques informations supplémentaires qui pourraient vous intéresser [PRENOM].
		Merci de partager ces informations avec nous [PRENOM]. Il semble que vous voyez bien comment vos deux grandes qualités peuvent vous aider à pratiquer des activités physiques régulièrement. Voilà de bonnes nouvelles!<br><br>Fidèles à nos habitudes, avant de résumer la séance, nous pensons posséder quelques informations supplémentaires qui pourraient vous intéresser [PRENOM].
	S5.Q6	Aimeriez-vous recevoir ces informations [PRENOM]?<br><br>Oui, j'aimerais bien les recevoir<br>Non, pas aujourd'hui<br><br>Cliquez sur continuer pour avancer.
Page 9 – Informations efficacité personnelle		
Cette page est sautée si le participant ne veut pas d'info		
	S5.INFO	Quelques informations pour vous<br><br>Vous le savez sans doute déjà [PRENOM], plusieurs facteurs peuvent faire en sorte qu'on se sente capable ou incapable de pratiquer régulièrement des activités physiques régulièrement. Le but des informations qui suivent est de faire un retour sur les facteurs principaux qui pourraient affecter votre confiance positivement. Sans plus tarder, allons-y!<br><br>1. Des expériences personnelles positives<br>Lorqu'on ne se sent pas habile dans une activité, il est souvent normal de ne pas vouloir la répéter. À l'inverse, lorsqu'on se sent bon, on a souvent tendance à éprouver du plaisir à faire cette activité et à vouloir la répéter. Pour nous aider à maintenant une bonne confiance, un élément important est donc de trouver des activités physiques dans lesquelles on se sent bon et à l'aise. La bicyclette, la marche d'un pas vif et la natation sont des exemples de bonnes activités à effectuer pour favoriser notre confiance lorsque nous sommes peu familiers avec l'activité physique. En fait, toute activité physique qui vous attire naturellement pourrait s'avérer être un bon choix pour vous [PRENOM].<br><br>2. Un entourage qui vous fait sentir compétent[E]<br>Avez-vous déjà rencontré quelqu'un qui vous faisait sentir coupable de ne pas pratiquer des activités physiques? Votre médecin? Votre infirmière? Vos proches? Dans tous les cas, ce n'est pas très agréable n'est-ce pas? Pour vous aider à bâtir une forte confiance en vous, il est préférable de discuter de votre pratique d'activités physiques avec des gens qui possèdent le

		profil suivant :<br>Ils tentent de vous comprendre, mais ils ne vous jugent pas<br>Ils valorisent vos efforts en vue de devenir acti[FVE] régulièrement<br>Ils vous encouragent à persévérer et à donner le meilleur de vous-même<br>Ils vous font sentir que vous pouvez réussir en toute circonstance, à persévérer, malgré les échecs que vous pouvez vivre.<br>Pouvez-vous penser à une personne de ce genre dans votre entourage [PRENOM]? Sinon, n'hésitez pas à encourager vos proches à agir en ce sens!<br><br>3. Des expériences positives de personnes qui vous ressemblent<br>En effet, il peut être excellent de connaître des personnes comme vous qui ont pu réussir à devenir actif régulièrement et à maintenir cette habitude. Cela peut vous faire voir que l'objectif est réellement possible pour vous également. Dans cette optique, nous vous présenterons des témoignages de personnes atteintes du diabète de type 2 qui sont maintenant régulièrement activeS.Ces témoignages sauront peut-être vous donner la force dont vous avez besoin [PRENOM].<br><br>4. Percevoir vos échecs comme des tentatives<br>Vous serez d'accord [PRENOM], réussir à pratiquer des activités physiques régulièrement ne se fait pas du jour au lendemaiS6.Cela demande un certain effort pour prendre cette bonne habitude. Cela dit, si par moment vous n'atteignez pas votre objectif, ne baissez pas les braS.Les tentatives qui échouent sont tout à fait normales dans le développement de cette bonne habitude [PRENOM]. C'est en continuant toujours d'essayer de nouvelles méthodes pour arriver à votre objectif que vous trouverez la bonne. Si vous voulez devenir une personne active physiquement, n'arrêtez jamais d'essayer [PRENOM]!<br><br>Voilà donc ce que nous avions pour vous aujourd'hui.
	S5.Q7	Comment avez-vous trouvez ces informations [PRENOM]?<br>1- J'ai aimé<br>2- Je n'ai pas aimé<br>3- Je ne sais pas trop<br><br>Cliquez maintenant sur continuer pour accéder au résumé de la séance d'aujourd'hui!
Page 10 – Résumé de la séance		
	S5.REF_INFO1	Merci de le partager [PRENOM].<br><br>Il semble que vous ayez apprécié les informations précédentes! Nous sommes heureux de l'entendre.<br><br>Voici maintenant votre résumé de la séance d'aujourd'hui.

		Merci encore et félicitations pour avoir franchi une autre étape du programme
	S5.REF_INFO2	Mmmm…<br><br>Il semble que les informations précédentes n'ont pas satisfait vos attentes [PRENOM]. Merci de le partager. Nous espérons pouvoir vous fournir les informations dont vous avez besoin à travers les prochaines activités de Diabète en Formeé<br><br>Voici malgré tout un résumé de votre séance d'aujourd'hui.<br><br>Merci encore et félicitations pour avoir franchi une autre étape du programme.
	S5.REF_INFO3	Mmmmm…<br><br>Vous ne savez pas trop quoi penser des informations précédentes [PRENOM]. Nous espérons qu'elles puissent malgré tout vous aider à prendre des décisions plus éclairéeS5.<br><br>Pour le moment, voici quand même un résumé de votre séance d'aujourd'hui [PRENOM].<br><br>Merci encore et félicitations pour avoir franchi une autre étape du programme.
	S5.RES_EFFLOW	Résumé de la séance 4, pour vous<br><br>Encore une fois, merci pour cet échange [PRENOM], voici, pour vous, ce que nous retenons d'aujourd'hui.<br><br>Au moment, de l'inscription, vous aviez peu confiance en votre capacité à devenir acti[FVE] physiquement au cours du prochain moiS.Cela dit, nous avons ainsi tenté de voir ensemble ce qui pourrait vous rendre plus confiant[E].
	S5.RES_EFFMIDLOW	Résumé de la séance 4, pour vous<br><br>Encore une fois, merci pour cet échange [PRENOM], voici, pour vous, ce que nous retenons d'aujourd'hui.<br><br>Au moment, de l'inscription, vous aviez peu confiance en votre capacité à devenir acti[FVE] physiquement au cours du prochain moiS.D'un autre côté, vous sembliez quand même avoir un brin de confiance en votre capacité de réussir. Ensemble, nous avons ainsi tenté de continuer en ce sens et de voir ensemble ce qui

		pourrait vous rendre plus confiant[E].
	S5.RES_EFFMIDHIGH	Résumé de la séance 4, pour vous<br><br>Encore une fois, merci pour cet échange [PRENOM], voici, pour vous, ce que nous retenons d'aujourd'hui.<br><br>Au moment de l'inscription, vous possédiez déjà une certaine confiance en votre capacité à pratiquer des activités physiques régulièrement [PRENOM]. C'est excellent! Pour continuer dans cette direction, nous avons ainsi tenté de voir ensemble ce qui pourrait vous rendre encore plus confiant[E] en votre capacité de réussir.
	S5.RES_EFFHIGH	Résumé de la séance 4, pour vous<br><br>Encore une fois, merci pour cet échange [PRENOM], voici, pour vous, ce que nous retenons d'aujourd'hui.<br><br>Au moment de l'inscription, vous possédiez déjà une grande confiance en votre capacité de pratiquer régulièrement des activités physiqueS.C'est excellent! Pour continuer dans cette direction, nous avons ainsi tenté de voir ensemble ce qui pourrait vous rendre encore plus confiant[E] en votre capacité de réussir.
	S5.RES_STR2	Vos forces et l'activité physique<br><br>À travers cette séance, vous avez pu identifier des forces qui vous correspondent [PRENOM] et tenter de faire des liens avec la pratique d'activités physiqueS.Les deux forces qui vous correspondent le plus semblent être celles-ci :<br><br>[S5.STR1] à [S5.STR2]<br><br>Vous semblez [S5.STR_VALORI1] et [S5.STR_VALORI2]. Ces forces sont en vous [PRENOM], bravo.
	S5.RES_STR310	À travers cette séance, vous avez pu identifier des forces qui vous correspondent [PRENOM] et tenter de faire des liens avec la pratique d'activités physiqueS5.<br><br>Vous possédez beaucoup de forces en vous [PRENOM], voici celles qui vous correspondent :<br><br>[S5.STR1] à [S5.STR25]<br><br>De plus, vous avez identifié deux forces qui vous correspondaient davantage. Vous semblez [S5.STR_VALORI1] et [S5.STR_VALORI2]. Ces forces sont en vous [PRENOM], bravo.
	S5.RES_ELAB0	Comme cela peut contribuer à votre confiance, vous avez ensuite

		été amené à trouver comment vos forces pourraient vous aider à devenir acti[FVE] régulièrement. Comment le fait d'[S5.STR_VALORI1] pourrait donc vous aider [PRENOM]? Comment le fait d'[S5.STR_VALORI2] pourrait donc vous aider [PRENOM]? Ce sont là d'excellentes qualités que vous possédez.
	S5.RES_ELAB12	Comme cela peut contribuer à votre confiance, vous avez ensuite été amené à trouver comment vos forces pourraient vous aider à devenir acti[FVE] régulièrement. Voici comment, pour vous, le fait d'[S5.STR_VALORICHOICE] pourrait vous aider à pratiquer davantage d'activités physiques : « [S5.QCHOICE] ». Comment le fait d'[S5.STR_VALORI1] pourrait donc vous aider [PRENOM]? Ce sont là d'excellentes qualités que vous possédez [PRENOM].
	S5.RES_ELAB22	Comme cela peut contribuer à votre confiance, vous avez ensuite été amené à trouver comment vos forces pourraient vous aider à devenir acti[FVE] régulièrement. Voici comment, pour vous, le fait d'[S5.STR_VALORI1] pourrait vous aider à pratiquer davantage d'activités physiques : « [S5.Q4] ». Voici comment, pour vous, le fait d'[S5.STR_VALORI2] pourrait vous aider à pratiquer davantage d'activités physiques : « [S5.Q5] ».
	S5.RES_STROPEN	Une autre force plus personnelle semble aussi vous habiter [PRENOM] : [S5.Q2_OPEN]. Cette force pourrait aussi vous aider à pratiquer davantage d'activités physiques [PRENOM].
	S5.RES_INFO0	Vous avez choisi de ne pas recevoir d'informations aujourd'hui. Celles-ci portaient sur les différentes façons dont nous pouvons favoriser une forte confiance en notre capacité de réussir à être acti[FVE] régulièrement. Si vous voulez finalement les recevoir par courriel, cliquez la case ci-dessous simplement.<br>O Oui, je veux recevoir les informations par courriel
	S5.RES_INFO1	Voici un rappel des trois façons de favoriser une forte confiance en vos capacités de devenir acti[FVE] régulièrement [PRENOM] :<br>Trouver des activités qui vous font sentir compétent[E] et dans lesquelles vous sentez que vous avez du plaisir<br>Discuter de votre pratique d'activités physiques avec des personnes qui vous supportent positivement<br>Connaître des expériences positives de personnes qui vous ressemblent
	S5.RES_CONCLU	Nous espérons sincèrement que cette séance vous aidera à prendre confiance [PRENOM], peu importe à quel point vous étiez confiant[E] au départ. Ce n'est jamais trop!<br><br>Voilà, ce que nous avons retenu de la séance d'aujourd'hui. Qu'en pensez-vous [PRENOM]?
	S5.AVENIR	À VENIR<br>Wow [PRENOM], déjà quatre séances réaliséeS.Bravo.<br><br>Dans la prochaine séance, nous tenterons de voir, pour vous, quelle serait la façon idéale d'intégrer la pratique d'activités physiques régulière dans votre quotidieS6.De plus, vous aurez accès à des témoignages de personnes atteintes du diabète de type 2 ayant réussi à devenir actif et active régulièrement.

		Nous espérons que cela vous plaira!<br><br>Cliquez sur continuer pour accéder à la dernière page.
Page 11 – Plan d'action		
		Vers où tout cela vous mène?<br><br>Nous avons bien discuté durant les dernières semaines [PRENOM]. Avec ce que nous vivons ensemble, il pourrait être possible que votre attitude, votre confiance et votre motivation aient alors changé positivement en vue de faire plus d'activités physiqueS.<br><br>Si vous n'avez pas essayé de planifier vos activités physiques avec l'aide de Diabète en Forme encore, nous vous encourageons à le faire, car cela peut s'avérer très bénéfique!<br><br>Peu importe comment vous vous sentez, plusieurs choix s'offrent maintenant à vous [PRENOM]. Sentez-vous absolument libre de choisir ce qui vous convient (vous êtes l'unique expert[E] de vous-même!).
		Alors, qu'aimeriez-vous faire maintenant?*<br><br>1- Je veux planifier mes activités physiques pour les prochains jours<br><br>2- Je vois des choses qui m'empêchent de faire plus d'activités physiques, j'aimerais en discuter<br><br>3- Je veux tout simplement quitter pour l'instant (si tel est le cas, au plaisir de vous revoir bientôt!)<br><br>*Vous pourrez toujours revenir à ces options lorsque vous accédez au site de Diabète en Forme [PRENOM]. N'hésitez pas à les consulter à tout moment opportun, ce pourrait réellement être de bons outils à utiliser.<br><br>Merci pour tout<br><br>L'équipe de Diabète en Forme
**Sixth motivation session**		
Page 1 – Retour sur la séance 4		
	S6.RETOUR	AVANT DE COMMENCER<br>RETOUR SUR LA DERNIÈRE SÉANCE

<table>
<tr><td></td><td></td><td>Bonjour [PRENOM]. C’est bon de vous revoir.

À la dernière séance, nous avons discuté de votre confiance en votre capacité de pratiquer des activités physiques régulièrement. Afin de tenter de rendre cette confiance plus solide, nous avons exploré ensemble les forces que vous possédez afin de créer des liens entre celles-ci et votre pratique d’activités physiqueS5.

Vous vous souvenez? Nous avions mentionné que les études démontrent que cet exercice est excellent pour gagner de la confiance en vous et ainsi réussir éventuellement à pratiquer des activités physiques régulièrement.

Nous espérons que votre confiance se porte pour le mieux depuis cette séance! Si vous voulez devenir une personne régulièrement active [PRENOM], nous sommes encore une fois profondément convaincus que vous en êtes capable. Vous êtes remplis de qualités et de forces qui peuvent vous aider.

La séance d’aujourd’hui pourrait également vous aider à renforcir davantage votre confiance en votre capacité de réussir cet objectif de devenir une personne régulièrement active.

Nous espérons encore une fois que vous aimerez!

Bonne séance [PRENOM].

L’équipe de Diabète en Forme

→Cliquez sur continuer pour avancer</td></tr>
<tr><td colspan="3">Page 2 - Introduction à la 5ième séance</td></tr>
<tr><td></td><td>S6.INTRO_VIDEO</td><td>Bienvenue à votre 5ième séance [PRENOM]!

Message vidéo : Bonjour monsieur/madame, c’est Michel de Diabète en Forme. Je suis très content que vous soyez toujours avec nous sur Diabète en Forme.

Vous avez déjà traversé la majorité du programme en participant aux quatre premières séances d’informations précédenteS.Je vous félicite sincèrement. J’espère que cela vous a aidé à trouver la motivation réussir à pratiquer régulièrement des activités physiques et que vous sentez que vous êtes capable de réussir ce beau défi.

Maintenant, si vous le voulez, la séance d’aujourd’hui vous servira à visualiser votre pratique d’activités physiques future. Concrètement, la séance vous guidera à travers une série de questions simples à répondre, qui vous aideront à percevoir quelle serait, pour vous, la manière idéale et réaliste de pratiquer des activités physiques régulièrement au quotidieS6.</td></tr>
</table>

		Cette activité est très efficace pour se visualiser en situation de réussite et s’imaginer à quel point on pourrait se sentir heureux ou heureuse de ce changement, soit de pratiquer régulièrement des activités physiqueS5.<br><br>En effectuant cet exercice, cela pourrait donc vous aider à la fois pour votre motivation et votre confiance en votre capacité de réussir ce changement.<br><br>Sans plus tarder, je vous encourage à foncer!<br><br>J’espère que vous aimerez cette séance!<br><br>Bon succès et à bientôt!
	S6.OBJECTIFS _DUREE	OBJECTIFS DE LA SÉANCE<br><br>Visualiser votre pratique d’activités physiques<br><br>Identifier les solutions aux barrières que vous auriez à surmonter dans votre parcours<br><br>Planifier votre pratique d’activités physiques pour les prochains jours (optionel)<br><br>DURÉE<br>10 à 15 minutes<br><br>Pourquoi cette séance?<br>Cette séance pourrait vous permettre de gagner de la motivation et de la confiance en vous en visualisant votre pratique d’activités physiques régulière future.<br><br>Lorsque vous serez prêt[E], cliquez sur continuer pour commencer [PRENOM]!
Page 3 – Visualiser votre pratique d’activités physiques - Intro		
	S6.Intro_Q1_3	Visualiser votre pratique d’activités physiques peut vous aider à gagner motivation et confiance<br><br>[PRENOM], pour cette séance, supposons que nous sommes maintenant un an plus tard, et que vous avez réussi à devenir acti[FVE] régulièrement. Vous n’avez plus l’intention de revenir en arrière. Quelles activités physiques faites-vous? Où les faites-vous? Combien de minutes d’activités physiques faites-vous par semaine? Pour quelles raisons personnelles avez-vous décidé d’effectuer ce changement?<br><br>Voilà quelques questions auxquelles nous tenterons de répondre ensemble aujourd’hui si vous êtes partant[E].<br><br>Avant de commencer, nous aimerions vous partager deux

		témoignages de personnes atteintes du diabète de type 2 ayant réussi, malgré plusieurs embûches, à devenir des personnes actives régulièrement. Aujourd'hui, elles ne pourraient pas s'en passer.<br><br>Connaître leurs histoires pourrait vous aider à voir comment, à votre tour, vous pourriez intégrer des activités physiques régulièrement à votre quotidieS6.<br><br>Cliquez sur continuer pour accéder à ces deux témoignages vidéo [PRENOM]. Si vous ne voulez pas les regarder, vous pouvez également continuer directement à la page suivante.
Page 4 - Témoignages		
	S6.TEM1	Julie<br>Message du vidéo :<br>Bonjour je m'appelle Julie, j'ai 53 ans<br><br>J'ai appris que j'avais le Diabète il y a environ 3 anS.L'activité physique a jamais vraiment fait partie de ma vie sauf depuis 1 an et demi environ je diraiS5.<br><br>Au début, quand mon médecin me disait qu'il fallait que je fasse des activités physiques régulièrement, je VOULAIS RIEN savoir. Je voyais aucun moyen de prendre 150 minutes de mon temps pour aller bouger, en plus de mon travail, de ma famille et mes autres activitéS.Sans dire que je ne pensais pas que c'était vraiment important pour moi.<br><br>Un peu comme vous faites sur Diabète en Forme, c'est à force de discuter avec mon médecin et de m'informer que j'ai pris conscience des raisons qui me pousseraient à changer. L'activité physique, c'est essentiel vraiment. Je veux vivre longtemps, c'est la principale raison pourquoi je le faiS5.<br><br>C'est dans ma nature aussi d'être forte et de relever les défis qui se présentent à moi, je veux être là pour ceux que j'aime et être capable de les aider s'ils en ont besoiS6.<br><br>Ça a été ça pour moi les éléments déclencheurs, que je comprenne que l'activité physique, c'est un facteur clé si je veux vivre longtemps et être là pour mes procheS5.<br><br>Je fais au moins 150 minutes d'activités physiques par semaine depuis bientôt 1 aS6.Je vais marcher, je fais du vélo et je nage. Ça vraiment pas été facile de prendre l'habitude au début, mais pour moi c'était claire, il fallait que je me prenne en main, c'est ça que je voulais être, que je voulais devenir. Et aujourd'hui, si vous

		saviez à quel point je pourrais pas m'en passer, je me suis jamais senti aussi bien, aussi énergique et aussi motivé à faire tout ce que j'aime de toute ma vie!<br><br>J'avais toutes sorte de raisons de me faire croire que je pouvais pas y arriver, j'aurais pu en sortir des milliers…jusqu'à temps que je me dise : là, c'est assez julie!. Si moi j'y arrive, tout le monde peut y arriver. C'est cliché mais c'est ça! Je le pense vraiment.<br><br>Question : Comment tu te sens maintenant?<br>C'est…c'est…. Pour vrai, à voir aujourd'hui l'effet que ça a sur moi, je suis tellement fière, oui, j'suis vraiment fière de moi.
	S6.TEM2	Bernard<br>Je m'appelle Bernard, j'ai 45 ans<br><br>Je sais que j'ai le diabète depuis 10 anS.J'ai joué longtemps au tennis 1 fois par semaine avec un collègue mais à part ça je faisais pas grand-chose comme activité physique.<br><br>Ça a vraiment été dure de trouver la motivation, mais je m'étais jamais informé sérieusement sur l'activité physique. J'entendais tout le monde dire que c'est dont bon pour la santé faire des activités physiques, mais c'est comme si je me disais toujours que le message s'adressait pas à moi. J'avais peut-être pas confiance que j'étais capable de devenir actif et c'était pour ça que j'écoutais pas le message. Mon médecin, mon frère, ma mère, tout le monde me disait de bouger pluS.Je le faisais paS5.<br><br>Depuis 2 ans, je pratique des activités physiques régulièrement, je marche 30 minutes à 1 heure à chaque jour et des fois je vais nager quand j'ai quelqu'un avec qui y aller, mais j'ai pas souvent de partenaire alors j'fais plus marcher hahaha. J'ai fait un peu de spinning(vélo stationnaire) en groupe c'était le fun aussi, c'est simple pi tu vas à ton rythme.<br><br>Question : Ce qui t'as fait changé?<br>Ma santé se détériorait, j'avais des infections aux pieds…ça vraiment été là où je me suis dis, c'est assez là je me prends en maiS6.En plus, j'avais pas d'énergie, je manquais de motivation pour des choses que normalement j'aime. Il fallait que je fasse quelque chose pour redevenir moi-même, être intègre à moi-même la tsé!<br><br>Question : Comment tu te sens aujourd'hui?<br>Sérieusement, je ne peux pas remercier assez la vie de m'avoir donné le courage d'entreprendre ce changement-là. Je me sens tellement bien, je suis énergique, j'ai le goût de faire plein de choses, et je les fais! Les semaines où je fais moins d'activités physiques, je le sens tout suite, je me sens tellement mieux quand je bouge, c'est moi de bouger.

		Je peux pas me sentir mieux, de où je suis parti, et de voir où je suis rendu, je suis extrêmement fier de moi.<br><br>Si je peux donner un conseil à tout le monde qui nous écoute. Ça va être difficile au début peut-être, peut-être même pendant un bout. Mais si je peux vous dire une chose C'est ça : Arrêter jamais de chercher des solutions, des embûches il va y en avoir, mais n'arrêtez jamais de chercher des solutions, persévérez, le succès va venir. Des semaines où vous êtes moins motivé ou où vous êtes moins actif y va y en avoir, mais n'abandonnez pas à cause de ça, c'est normal d'être moins motivé des foiS.Si moi je suis capable, tout le monde est capable ça c'est sur!
	S6.TRANS_P45	Nous espérons que ces témoignages sauront vous inspirer [PRENOM]! Maintenant, à votre tour de visualiser votre propre pratique.<br><br>Cliquez sur continuer pour la suite!
Page 5 – Visualiser sa pratique d'activités physiques – exercice partie 1		
	S6.Intro_Vis	Excellent [PRENOM]!<br><br>Avant les témoignages que vous venez de voir, vous avez pris connaissance de l'objectif de l'exercice d'aujourd'hui. Étape par étape, tentons de visualiser ensemble votre pratique d'activités physiques future.<br><br>D'abord, tentons d'imaginer combien de minutes d'activités physiques que vous feriez, quelles activités physiques vous aimeriez faire et pour quelles raisons vous les feriez. Pour ce faire, répondez simplement aux trois questions qui suivent [PRENOM]!
	S6.Q1	Au moment de l'inscription, vous faisiez [F.APTOT] minutes d'activités physiques [PRENOM]. Combien de minutes d'activités physiques aimeriez-vous faire d'ici 1 an?<br><br>150 minutes (équivaut aux recommandations canadiennes)<br>170 minutes<br>190 minutes<br>200 minutes et plus
	S6.Q2	Quelles activités aimeriez-vous effectuer pour atteindre cet objectif?<br><br>1=Marche<br>10=Golf (sans voiturette)<br>100= la bicyclette<br>1000= le patinage<br>10000= la natation/nage<br>100000= le tennis<br>1000000= la danse<br>10000000= le jardinage<br>100000000= le jogging<br>1000000000= le vélo stationnaire

<table>
<tr><td></td><td></td><td>10000000000=le tapis roulant
100000000000= l'elliptique
1000000000000=hockey
10000000000000=basketball
100000000000000=soccer
1000000000000000=centre de conditionnement
10000000000000000= réponse ouverte</td></tr>
<tr><td></td><td>S6.Q3</td><td>Quelles seraient vos raisons principales pour lesquelles vous êtes devenu(e) une personne régulièrement active [PRENOM]?

Espace de réponse

Exemple : avoir plus d'énergie et être en santé longtemps

Cliquez sur continuer pour la suite [PRENOM]</td></tr>
<tr><td colspan="3">Page 6 – Reflet et Identification des barrières</td></tr>
<tr><td></td><td>S6.Intro_Vis2</td><td>D'accord [PRENOM], d'ici un an, vous réussirai à prendre l'habitude de pratiquer [S6.Q1] minutes par semaine. De plus, [S6.Q2] sont les activités physiques qui vous aideront à atteindre cet objectif. Finalement, vous devrez une personne régulièrement active pour les raisons suivantes principalement:</td></tr>
<tr><td></td><td>S6.Q4</td><td>Maintenant, y a-t-il quelque chose d'important qui pourrait vous empêcher de faire [S6.Q1] d'activités physiques d'ici un an [PRENOM]?

Oui/non</td></tr>
<tr><td></td><td>S6.Q5
S'affiche seulement si répond oui à S6.Q4</td><td>D'accord [PRENOM], quelles seraient donc ces barrières qui pourraient vous empêcher de réussir? (sélectionnez 1 ou 2 options)

Être trop fatigué pour faire de l'activité physique
Avoir beaucoup de choses à faire mis à part l'activité physique
La température sera trop mauvaise pour faire de l'activité physique (hiver/été)
Ne pas avoir personne avec qui faire de l'activité physique
Ne pas avoir accès à des emplacements pour faire de l'activité physique
Ne pas avoir d'argent pour faire de l'activité physique
Être trop gênée/manquer de confiance pour aller faire de l'activité physique
Avoir peur de ne pas être assez en santé pour faire de l'activité physique
Manquer de motivation</td></tr>
<tr><td></td><td>S6.QOPEN1</td><td>Y a-t-il autres choses qui pourrait nuire à vos chances de réussir [PRENOM]?</td></tr>
<tr><td></td><td>S6.TRANSITIONP5P6</td><td>Cliquez sur continuer lorsque vous aurez fait vos choix [PRENOM]!</td></tr>
<tr><td colspan="3">Page 7– Reflets barrière – trouver des solutions</td></tr>
<tr><td></td><td>S6.BAR0</td><td>Excellent [PRENOM], il semble que vous ne voyez aucune barrière qui vous empêcherait d'atteindre votre objectif de faire [S6.Q1] d'activités physiques par semaine d'ici 1 aS6.Très bonne nouvelle! Il vous suffira de garder une bonne habitude de planifier vos activités physiques de semaine en semaine, et ainsi foncez! Vous en êtes capable!

Maintenant, passons ensemble à une autre étape.</td></tr>
</table>

		Cliquez sur continuer pour avancer [PRENOM].
	S6.Bar1	D'accord [PRENOM], il semble que vous voyez une barrière pouvant éventuellement menacer l'atteinte de votre objectif de faire [S6.Q1] d'activités physiques par semaine d'ici 1 aS6.Voici cette barrière :<br>[S6.REF_BAR]<br><br>Si vous avez également mentionné une barrière plus personnelle, nous y reviendrons plus tard sans faute.
	S6.BAR2	D'accord [PRENOM], il semble que vous voyez deux barrières pouvant éventuellement menacer l'atteinte de votre objectif de faire [S6.Q1] d'activités physiques par semaine d'ici 1 aS6.Voici ces barrières :<br>[S6.2REF_BAR1]<br>[S6.2REF_BAR2]<br><br>Si vous avez également mentionné une barrière plus personnelle, nous y reviendrons plus tard sans faute.
	**Microtailoring**<br>S6.REF_BAR	1 = Être trop fatigué pour faire de l'activité physique<br>2= Avoir beaucoup de choses à faire mis à part l'activité physique<br>3 =La température sera trop mauvaise pour faire de l'activité physique (hiver/été)<br>4 =Ne pas avoir personne avec qui faire de l'activité physique<br>5 =Ne pas avoir accès à des emplacements pour faire de l'activité physique<br>6 =Ne pas avoir d'argent pour faire de l'activité physique<br>7 =Être trop gênée/manquer de confiance pour aller faire de l'activité physique<br>8 =Avoir peur de ne pas être assez en santé pour faire de l'activité physique<br>9 = Manquer de motivation
	**Microtailoring**<br>S6.2REF_BAR1	1 = Être trop fatigué pour faire de l'activité physique<br>2= Avoir beaucoup de choses à faire mis à part l'activité physique<br>3 =La température sera trop mauvaise pour faire de l'activité physique (hiver/été)<br>4 =Ne pas avoir personne avec qui faire de l'activité physique<br>5 =Ne pas avoir accès à des emplacements pour faire de l'activité physique<br>6 =Ne pas avoir d'argent pour faire de l'activité physique<br>7 =Être trop gênée/manquer de confiance pour aller faire de l'activité physique<br>8 =Avoir peur de ne pas être assez en santé pour faire de l'activité physique<br>9 = Manquer de motivation
	**Microtailoring**<br>S6.2REF_BAR2	2= Avoir beaucoup de choses à faire mis à part l'activité physique<br>3 =La température sera trop mauvaise pour faire de l'activité physique (hiver/été)<br>4 =Ne pas avoir personne avec qui faire de l'activité physique<br>5 =Ne pas avoir accès à des emplacements pour faire de l'activité physique<br>6 =Ne pas avoir d'argent pour faire de l'activité physique<br>7 =Être trop gênée/manquer de confiance pour aller faire de l'activité physique<br>8 =Avoir peur de ne pas être assez en santé pour faire de l'activité physique<br>9 = Manquer de motivation
	S6.Q6	Comme à toute embûche, il y a toujours une solution possible. Ci-dessous, vous verrez une liste de solutions aux barrières qui pourraient vous empêcher de pratiquer autant d'activités physiques que vous le désiriez dans le future. Ces pistes de solutions ont toutes été tentées par d'autres personnes atteintes du diabète de type 2 auparavant et elles se sont avérées souvent efficaceS5.

		Cela dit, plaçons-nous dans l'éventualité où les barrières que vous avez sélectionnées pourraient survenir [PRENOM].<br><br>Quelle solution serait la plus réaliste et la plus puissante à utiliser pour vous de façon à vaincre cette barrière : [S6.QREF_BAR1] ?<br>IF S6.Q5 = 1<br>1= Je vais faire de l'activité tôt dans la journée, le matin ou le midi.<br>2= J'essaie de garder en tête que si je fais de l'activité physique, j'aurai de plus en plus d'énergie dans la vie.<br>3= Je vais planifier des activités avec d'autres personnes qui me motiveront<br>4=Je prends une collation pour me donner de l'énergie et j'y vais quand même<br>5= réponse personnelle<br><br>IF S6.Q5 = 2<br>1= Faire de l'activité physique par bloc de 10 minutes les journées où je n'ai pas le tempS.Ex : 10 minutes le mation, 10 minutes l'après midi et 10 minutes le soir<br>2= Je vais remplacer des activités où je suis assis (télé, ordinateur) par des activités physiques<br>3=Je vais me procurer un agenda et inscrire dans mon horaire les moments où je vais faire de l'activité physique<br>4= Je vais prendre des pauses de travail active et faire des 10 minutes de marche.<br>5= Je vais me fixer des rendez-vous avec d'autres personnes qui me plaisent pour aller faire de l'activité physique<br><br>IF S6.Q5 = 3<br>1= je vais me procurer l'équipement nécessaire pour en faire même quand il pleut, quand il neige ou quand il fait trop chaud.<br>2= Je vais faire de l'activité physique chez nous ou à l'intérieur dans un endroit que j'aurai choisi quand il fait moins beau<br>3= Je vais me préparer une activité physique alternative au cas où il ne fait pas assez beau pour aller dehors<br>4=je vais me dresser une liste des activités que je serais prêt à faire si il ne fait pas beau<br>IF S6.Q5 = 4<br>1=Je vais me joindre à un groupe qui pratique l'activité physique que j'aime. Ex : la marche, l'aquaforme ou la natation<br>2= Je vais inviter mes amis, ma famille ou mes collègues à faire de l'activité physique avec moi<br>3= Je vais amener mon chien marcher avec moi<br>4= Pour moins me sentir seule, je vais aussi participer dans des discussions en ligne sur des pages facebook : Diabète Québec et autreS5.<br><br>IF S6.Q5 = 5<br>1= Je vais faire de la marche ou du vélo dehors, c'est simple et gratuit.<br>2= Je vais faire de l'activité physique dans ma maison (yoga, programme d'AP sur DVD, vélo stationnaire, tapis roulant, élliptique).<br>3= Je vais aller voir au centre communautaire de mon quartier pour me donner des options<br>4= je vais discuter avec mon médecin, avec un ami ou un spécialiste de l'activité physique pour avoir des conseils<br><br>IF S6.Q5 = 6<br>1= je vais trouver des activités physiques qui ne coûte rien : marcher dehors, faire du vélo, nager à la piscine communautaire.<br>2= Je vais aller voir au centre communautaire de mon quartier pour me donner des options<br>3=Je vais économiser quelques sous pour m'acheter un DVD d'activité physique ou pour une autre activité physique que j'aimerais faire<br>4= Je vais aller marcher avec ma famille, des amis ou des collègues<br><br>IF S6.Q5 = 7<br>1= Je vais choisir une activité physique simple comme la marche ou le vélo.<br>2= Je vais aller faire de l'activité physique avec des gens qui m'acceptent comme je suis, qui me juge paS5.<br>3= Au départ, je vais faire de l'activité physique dans des endroits où je suis seul pour prendre confiance.<br>4=Je vais éviter des endroits qui me gêne le plus comme les centres de conditionnement.

		IF S6.Q5 = 8<br>1= Je vais consulter mon médecin pour mettre au clair si je peux faire de l'activité physique<br>2= Je vais consulter un spécialiste de l'activité physique pour qu'il me rassure et me conseille sur ce qui est sécuritaire pour moi<br>3= Je vais commencer à petite dose. 10 à 20 minutes maximum les jours où j'en fais et voir comment ça va.<br>4= Je vais faire de l'activité physique accompagnée d'un spécialiste de l'activité physique<br>5= Je vais faire de l'activité physique dans un groupe où un spécialiste de l'AP est présent<br><br>IF S6.Q5 = 9<br>1= je signe un contrat papier avec moi-même comme quoi je m'engage à être actif<br>2= je signe un contrat papier avec mes proches comme quoi je m'engage à être actif<br>3=Je vais essayer de nouvelles activités physiques pour trouver celles qui me plaisent vraiment<br>4= À chaque fois que je dois faire de l'activité physique, je prends un temps pour me rappeler ce que cela va m'apporter de positif dans ma vie, à court et long terme.<br>5= Je vais faire de l'activité physique avec des gens que j'aime pour me motiver
	S6.Q7	Quelle solution serait la plus réaliste et la plus puissante à utiliser pour vous de façon à vaincre cette barrière : [S6.QREF_BAR1] ?<br><br>1= Je vais faire de l'activité tôt dans la journée, le matin ou le midi.<br>2= J'essaie de garder en tête que si je fais de l'activité physique, j'aurai de plus en plus d'énergie dans la vie.<br>3= Je vais planifier des activités avec d'autres personnes qui me motiveront<br>4=Je prends une collation pour me donner de l'énergie et j'y vais quand même<br>5= réponse personnelle<br><br>IF S6.Q5 = 2<br>1= Faire de l'activité physique par bloc de 10 minutes les journées où je n'ai pas le tempS.Ex : 10 minutes le mation, 10 minutes l'après midi et 10 minutes le soir<br>2= Je vais remplacer des activités où je suis assis (télé, ordinateur) par des activités physiques<br>3=Je vais me procurer un agenda et inscrire dans mon horaire les moments où je vais faire de l'activité physique<br>4= Je vais prendre des pauses de travail active et faire des 10 minutes de marche.<br>5= Je vais me fixer des rendez-vous avec d'autres personnes qui me plaisent pour aller faire de l'activité physique<br><br>IF S6.Q5 = 3<br>1= je vais me procurer l'équipement nécessaire pour en faire même quand il pleut, quand il neige ou quand il fait trop chaud.<br>2= Je vais faire de l'activité physique chez nous ou à l'intérieur dans un endroit que j'aurai choisi quand il fait moins beau<br>3= Je vais me préparer une activité physique alternative au cas où il ne fait pas assez beau pour aller dehors<br>4=je vais me dresser une liste des activités que je serais prêt à faire si il ne fait pas beau<br>IF S6.Q5 = 4<br>1=Je vais me joindre à un groupe qui pratique l'activité physique que j'aime. Ex : la marche, l'aquaforme ou la natation<br>2= Je vais inviter mes amis, ma famille ou mes collègues à faire de l'activité physique avec moi<br>3= Je vais amener mon chien marcher avec moi<br>4= Pour moins me sentir seule, je vais aussi participer dans des discussions en ligne sur des pages facebook : Diabète Québec et autreS5.<br><br>IF S6.Q5 = 5<br>1= Je vais faire de la marche ou du vélo dehors, c'est simple et gratuit.<br>2= Je vais faire de l'activité physique dans ma maison (yoga, programme d'AP sur DVD, vélo stationnaire, tapis roulant, élliptique).<br>3= Je vais aller voir au centre communautaire de mon quartier pour me donner des options<br>4= je vais discuter avec mon médecin, avec un ami ou un spécialiste de l'activité physique pour avoir des conseils<br><br>IF S6.Q5 = 6

		1= je vais trouver des activités physiques qui ne coûte rien : marcher dehors, faire du vélo, nager à la piscine communautaire.<br>2= Je vais aller voir au centre communautaire de mon quartier pour me donner des options<br>3=Je vais économiser quelques sous pour m'acheter un DVD d'activité physique ou pour une autre activité physique que j'aimerais faire<br>4= Je vais aller marcher avec ma famille, des amis ou des collègues<br><br>IF S6.Q5 = 7<br>1= Je vais choisir une activité physique simple comme la marche ou le vélo.<br>2= Je vais aller faire de l'activité physique avec des gens qui m'acceptent comme je suis, qui me juge paS5.<br>3= Au départ, je vais faire de l'activité physique dans des endroits où je suis seul pour prendre confiance.<br>4=Je vais éviter des endroits qui me gêne le plus comme les centres de conditionnement.<br><br>IF S6.Q5 = 8<br>1= Je vais consulter mon médecin pour mettre au clair si je peux faire de l'activité physique<br>2= Je vais consulter un spécialiste de l'activité physique pour qu'il me rassure et me conseille sur ce qui est sécuritaire pour moi<br>3= Je vais commencer à petite dose. 10 à 20 minutes maximum les jours où j'en fais et voir comment ça va.<br>4= Je vais faire de l'activité physique accompagnée d'un spécialiste de l'activité physique<br>5= Je vais faire de l'activité physique dans un groupe où un spécialiste de l'AP est présent<br><br>IF S6.Q5 = 9<br>1= je signe un contrat papier avec moi-même comme quoi je m'engage à être actif<br>2= je signe un contrat papier avec mes proches comme quoi je m'engage à être actif<br>3=Je vais essayer de nouvelles activités physiques pour trouver celles qui me plaisent vraiment<br>4= À chaque fois que je dois faire de l'activité physique, je prends un temps pour me rappeler ce que cela va m'apporter de positif dans ma vie, à court et long terme.<br>5= Je vais faire de l'activité physique avec des gens que j'aime pour me motiver
	S6.TRANSITIONP6P7	Voyez-vous d'autres solutions possibles pour vaincre vos barrières [PRENOM]? Quelles sont-elles? Nous vous encourageons à tenter tout ce qui sera susceptible de vous faire atteindre votre objectif.<br><br>Cliquez sur continuer pour avancer.
Page 8 – Reflet solution/ Support social		
	S6.REF_SOL11	Excellent [PRENOM], il semble que vous ayez trouvé une solution qui pourrait vous aider à surmonter votre barrière si jamais elle survient. Nous y reviendrons!
	S6.REF_SOL22	Excellent [PRENOM], il semble que vous ayez trouvé des solutions qui pourraient vous aider à surmonter vos barrières si jamais elles surviennent. Nous y reviendrons!
	S6.INTROLOWQ8	Maintenant, vous avez mentionné au moment de l'inscription que votre entourage proche ne vous supportait pas vraiment dans votre pratique d'activités physiques [PRENOM]. D'un autre côté, il est possible qu'une personne en particulier proche de vous puisse vous supporter positivement et personnellement. En effet, les études démontrent que cela pourrait être utile pour contribuer à améliorer et maintenir votre motivatioS6.Si vous trouvez quelqu'un qui vous accompagnera dans votre pratique d'activités

<table>
<tr><td></td><td></td><td>physiques en plus de vous supporter positivement, c'est encore mieux!</td></tr>
<tr><td></td><td>S6.INTROMIDQ8</td><td>Maintenant, vous avez mentionné au moment de l'inscription que votre entourage proche vous supportait moyennement dans votre pratique d'activités physiques [PRENOM]. D'un autre côté, il est possible qu'une personne en particulier proche de vous puisse vous supporter plus positivement et personnellement. En effet, les études démontrent que cela pourrait être utile pour contribuer à améliorer et maintenir votre motivatioS6.De plus, si vous trouvez quelqu'un qui vous accompagnera dans votre pratique d'activités physiques en plus de vous supporter positivement, c'est encore mieux!</td></tr>
<tr><td></td><td>S6.INTROHIGHQ8</td><td>Maintenant, vous avez mentionné au moment de l'inscription que votre entourage proche vous supportait positivement dans votre pratique d'activités physiques [PRENOM]. C'est une excellente nouvelle. En effet, les études démontrent que cela pourrait être utile pour contribuer à améliorer et maintenir votre motivatioS6.De plus, si vous trouvez quelqu'un qui vous accompagnera dans votre pratique d'activités physiques en plus de vous supporter positivement, c'est encore mieux!</td></tr>
<tr><td></td><td></td><td>Voici une liste de personnes potentielles qui pourraient remplir ce rôle [PRENOM].

Y aurait-il quelqu'un en particulier qui joue ou qui pourrait jouer ce rôle dans cette liste?

1=conjoint/conjointe
2=mon ou mes enfants
3=mon père
4=ma mère
5=un ami ou une amie
6=mon médecin
7=mon infirmière
8=Mon spécialiste de l'activité physique (entraîneur)
9=Seulement moi-même</td></tr>
<tr><td></td><td>S6.TRANSITIONP7P8</td><td>Pourrait-il y avoir quelqu'un d'autre qui n'est pas dans ces choix [PRENOM]? Si oui, gardez cette personne en tête dans vos moments difficiles et encouragez celle-ci à vous supporter positivement en vue d'atteindre votre objectif.

Cliquez sur continuer pour la suite.</td></tr>
<tr><td colspan="3">Page 9 – Imaginer son sentiment</td></tr>
<tr><td></td><td>S6.SENTIMENT</td><td>Comment vous sentiriez-vous?
Vous commencez à avoir une idée claire de ce à quoi ressemblerait votre pratique d'activités physiques régulière [PRENOM]. Bravo! Avant de terminer la séance d'aujourd'hui, faisons ce dernier petit exercice ensemble si vous le voulez bieS6.</td></tr>
</table>

		Supposons que vous avez réussi ce changement et que vous faites maintenant [S6.Q1] minutes d'activités physiques par semaine.
	S6.Q9F	Comment vous sentiriez-vous d'avoir réussi ce beau défi [PRENOM]?<br><br>-très fière de moi<br>- assez fière de moi<br>-Un peu fière de moi<br>- Je ressentirais autre chose
	S6.Q9M	Comment vous sentiriez-vous d'avoir réussi ce beau défi [PRENOM]?<br><br>-très fier de moi<br>- assez fier de moi<br>-Un peu fier de moi<br>- Je ressentirais autrement
	S6.QOPEN2	D'accord [PRENOM], que ressentiriez-vous exactement? Tentez d'inscrire un sentiment positif!<br>(espace libre)
	S6.TRANSITIO NP8P9	Lorsque vous aurez terminé, cliquez sur continuer pour accéder à à votre résumé de la séance.
Page 10 – Résumé de la séance		
	S6.RES_INTRO 1	Votre pratique d'activités physiques future<br><br>Il semble que vous ayez maintenant une bonne idée de ce à quoi ressemblerait votre pratique d'activités physiques régulière idéale d'ici 1 an [PRENOM]. Voilà encore une fois une bonne nouvelle!<br><br>Voici donc, pour vous, à quoi ressemblerait votre objectif.<br><br>D'ici un an, vous avez mentionné que pratiquer [S6.Q1] minutes d'activités physiques serait une bonne cible pour vouS.Pour vous amener à atteindre cette cible dans un climat plaisant, [S6.REF_ACTIVITÉS] pourraient être vos meilleures activités à pratiquer régulièrement!<br><br>Suite aux séances que nous avons eues ensemble, il semble que vos principales raisons de vouloir atteindre cet objectif soient les suivantes : [S6.Q3]. Si vous avez pris le temps d'y réfléchir, ce sont sans aucun doute des raisons honorables qui ont du sens pour vous [PRENOM].
	S6.RES_INTRO 1	Votre pratique d'activités physiques future

		Il semble que vous ayez maintenant une bonne idée de ce à quoi ressemblerait votre pratique d’activités physiques régulière idéale d’ici 1 an [PRENOM]. Voilà encore une fois une bonne nouvelle!<br><br>Voici donc, pour vous, à quoi ressemblerait votre objectif.<br><br>D’ici un an, vous avez mentionné que pratiquer [S6.Q1] minutes d’activités physiques serait une bonne cible pour vouS.Pour vous amener à atteindre cette cible dans un climat plaisant, [S6.REF_ACTIVITÉS] pourrait être votre meilleure activité à pratiquer régulièrement.<br><br>Suite aux séances que nous avons eues ensemble, il semble que vos principales raisons de vouloir atteindre cet objectif soient les suivantes : [S6.Q3]. Si vous avez pris le temps d’y réfléchir, ce sont sans aucun doute des raisons honorables qui ont du sens pour vous [PRENOM].
	Microtailoring S6.REF_ACTIVITÉS	1=faire de la marche à un pas rapide<br>10=jouer au golf à la marche<br>100= faire de la bicyclette<br>1000= faire du patinage<br>10000= faire de la natation<br>100000= jouer au tennis<br>1000000= faire de la danse<br>10000000= faire du jardinage<br>100000000= faire du jogging<br>1000000000= faire du vélo stationnaire<br>10000000000=faire du tapis roulant<br>100000000000= faire de l’elliptique<br>1000000000000=faire du hockey<br>10000000000000=faire du basketball<br>100000000000000=faire du soccer<br>1000000000000000=faire de l´exercice dans un centre d´entraînement
	S6.RES_BAR0	Il semble que vous ne voyez aucune barrière qui vous empêcherait de réussir cet objectif [PRENOM]. C’est excellent pour vous, n’hésitez surtout pas à foncer! Vous êtes capable de réussir cet objectif.
	S6.RES_BAR2	Ensuite vous avez mentionné que certaines barrières pourraient vous rendre moins confiant(e) d’atteindre cet objectif [PRENOM]. D’un autre côté, vous avez également identifié des solutions pouvant vous aider à vaincre celles-ci! Voici votre plan pour vaincre les barrières éventuelles qui pourraient nuire à l’atteinte de votre objectif.<br><br>[S6.REF_BAR1] -----------------------------→ [S6.REF_SOL1]<br>[S6.REF_BAR2]-----------------------------→ [S6.REF_SOL2]<br><br>Vous voyez d’autres solutions [PRENOM]? Nous vous encourageons à tenter tout ce que vous pouvez en vue d’atteindre votre objectif, tout en demeurant sécuritaire bien sûr!
	S6.RES_BAR1	Ensuite vous avez mentionné que certaines barrières pourraient vous rendre moins confiant(e) d’atteindre cet objectif [PRENOM].

		D'un autre côté, vous avez également identifié des solutions pouvant vous aider à vaincre celles-ci! Voici votre plan pour vaincre les barrières éventuelles qui pourraient nuire à l'atteinte de votre objectif.<br><br>[S6.REF_BAR1] -----------------------------→ [S6.REF_SOL1]<br><br>Vous voyez d'autres solutions [PRENOM]? Nous vous encourageons à tenter tout ce que vous pouvez en vue d'atteindre votre objectif, tout en demeurant sécuritaire bien sûr!
	S6.RES_FEEL1	Si vous réussiriez à devenir une personne qui pratique des activités physiques régulièrement [PRENOM], il semble que vous vous sentiriez plutôt [FIER] de vouS.Vous pouvez définitivement l'être, car il s'agit d'un beau défi à relever.
	S6.RES_FEEL2	Si vous réussiriez à devenir une personne qui pratique des activités physiques régulièrement [PRENOM], il semble que vous vous sentiriez assez [FIER] de vouS.Vous pouvez définitivement l'être, car il s'agit d'un beau défi à relever.
	S6.RES_FEEL3	Si vous réussiriez à devenir une personne qui pratique des activités physiques régulièrement [PRENOM], il semble que vous vous sentiriez énormément [FIER] de vouS5.
	S6.RES_FEELOPEN	Si vous réussiriez à devenir une personne qui pratique des activités physiques régulièrement [PRENOM], il semble que vous vous sentiriez ainsi : [S6.QOPEN2]. Quoi qu'il en soit, devenir une personne active régulièrement est un beau défi à relever, et vous pourrez en être [FIER].
	S6.RES_TABLEAU	EN BREF<br><br>Votre objectif d'ici 1 an :<br>[S6.Q1] d'activités physiques par semaine<br>Vos activités physiques que vous aimez pratiquer :<br>[S6.REF_ACTIVITÉS]<br>Vos solutions aux barrières potentielles :<br>[S6.REF_BAR1]-------------------→ [S6.REF_SOL1]<br>[S6.REF_BAR2]-------------------→ [S6.REF_SOL2]<br><br>Vos raisons les plus importantes qui vous mèneraient vers la réussite de cet objectif :<br>[S6.QOPEN1]<br><br>Comment vous vous sentiriez de réussir cet objectif<br>[S6.Q9M] OR [S6.Q9F] [S6.QOPEN2]
	S6.RES_CONCLU	Vous avez maintenant une belle image de l'objectif que vous voulez atteindre et des moyens d'y arriver [PRENOM]. C'est excellent. Pour vous aider à bien passer à l'action et prendre l'habitude de pratiquer des activités physiques, n'hésitez pas à utiliser à planifier vos activités physiques des prochains jours suite à cette séance. Diabète en Forme peut vous accompagner dans cette démarche!

		Encore une fois, merci pour cet échange [PRENOM] et au plaisir de discuter à nouveau avec vous très bientôt!<br><br>L'équipe de Diabète en Forme
	S6.AVENIR	Nous serons déjà à la dernière séance du programme dans quelques jours [PRENOM]. Le temps passe vite!<br><br>Dans cette dernière séance, nous tenterons de résumer tous les échanges que nous avons eu ensemble au cours du programme. Cela pourra vous aider à bien voir toutes les raisons pouvant aider votre motivation et votre confiance en votre capacité de pratiquer des activités physiqueS5.<br><br>Vous recevrez par courriel un rappel pour vous annoncer quand la séance sera disponible pour vous!<br><br>Nous espérons que vous aimerez.<br><br>À très bientôt [PRENOM]<br><br>Cliquez sur continuer pour accéder à la toute dernière page!
Page 11 – Plan d'action		
	S6.Plan	Passez à l'action<br><br>Vous avez une idée claire d'où vous voulez vous rendre maintenant [PRENOM]. Vous êtes capable d'y arriver sans aucun doute. À vous de choisir la suite!<br><br>Alors, qu'aimeriez-vous faire maintenant?*<br><br>1- Je veux planifier mes activités physiques pour les prochains jours<br><br>2- Je vois des choses qui m'empêchent de faire plus d'activités physiques, j'aimerais en discuter<br><br>3- Je veux tout simplement quitter pour l'instant (si tel est le cas, au plaisir de vous revoir bientôt!)<br><br>*Vous pourrez toujours revenir à ces options lorsque vous accédez au site de Diabète en Forme [PRENOM]. N'hésitez pas à les consulter à tout moment opportun, ce pourrait réellement être de bons outils à utiliser.<br><br>Merci pour tout

		L’équipe de Diabète en Forme
**Seventh motivational session**		
Page 1 – Avant-propos		
	S7.AVANTPROPOSF	FÉLICITATIONS<br>Bonjour [PRENOM], nous voilà déjà à votre dernière séance! Bravo pour votre parcours sur Diabète en Forme.<br><br>Aujourd’hui, nous reviendrons sur chacune des séances que vous avez effectuées à travers le programme. Nous reviendrons donc sur votre dernière séance, en plus des autres, à travers la séance d’aujourd’hui.<br><br>Lorsque vous vous sentez prête, cliquez sur continuer pour avancer [PRENOM].
		FÉLICITATIONS<br>Bonjour [PRENOM], nous voilà déjà à votre dernière séance! Bravo pour votre parcours sur Diabète en Forme.<br><br>Aujourd’hui, nous reviendrons sur chacune des séances que vous avez effectuées à travers le programme. Nous reviendrons donc sur votre dernière séance, en plus des autres, à travers la séance d’aujourd’hui.<br><br>Lorsque vous vous sentez prêt, cliquez sur continuer pour avancer [PRENOM].
Page 2 - Introduction		
	S7.INTRO	Bienvenue à votre toute dernière séance [PRENOM]!<br><br>Message du vidéo : Bonjour monsieur/madame. C’est un plaisir de vous avoir pour une dernière fois, officiellement, avec nous aujourd’hui.<br><br>Comme vous le savez déjà sans doute, la séance d’aujourd’hui fera un topo de toutes les séances que nous avons effectué ensemble. Cela vous permettra ainsi de vous apercevoir de l’ensemble des facteurs positifs que vous avez évoqué au cours des dernières semaineS.Ces facteurs qui pourraient vous aider à trouver toute la motivation, la force et la confiance nécessaire afin de devenir une personne active.<br><br>J’espère que cette séance vous permettra de bien voir tout le chemin que vous avez parcouru.<br><br>Je ne vous retiens pas plus longtemps! Bonne séance et bonne continuité!<br><br>À votre santé!
	S7.OBJECTIFS	OBJECTIFS DE LA SÉANCE

	_DUREE	Résumer l'ensemble de vos séances d'information<br>Faire le bilan de ces informations<br>Juger vous-même ce que vous voudrez par la suite<br><br>DURÉE<br>10 à 15 minutes<br><br>Ce que vous pouvez retirer de cette séance<br>Retrouvez tous les éléments que vous avez mentionné pour vous aider à développer votre motivation et votre confiance en votre capacité de pratiquer des activités physiques régulièrement.<br><br>Cliquez sur continuer pour la suite!
Page 3 – Valorisation et présentation des parties		
	S7.P3INTRO	[PRENOM], vous avez franchi énormément d'étapes à travers Diabète en Forme. Encore une fois, félicitations!<br><br>Voici comment nous vous présenterons l'ensemble de votre parcours<br><br>PARTIE 1 – Votre niveau d'activités physiques au tout début du programme<br>Nous reviendrons simplement sur votre niveau d'activités physiques au début de l'aventure.<br>PARTIE 2 – Votre motivation<br>Nous reviendrons sur les séances 1, 2 et 3 où vous avez identifié plusieurs raisons personnelles vous incitant à pratiquer des activités physiques régulièrement.<br>PARTIE 3 – Votre confiance et votre pratique d'activités physiques d'ici 1 an<br>Nous reviendrons sur la séance 4 où vous avez su identifier des forces qui pourraient vous aider. De plus, nous aborderons également la séance 5 où vous avez pu bien visualiser votre pratique d'activités physiques pour le futur.<br><br>Nous terminerons le tout en examinant où vous en êtes au niveau de votre motivation et de votre confiance en votre capacité de réussir à devenir une personne active régulièrement [PRENOM].<br><br>Si vous êtes partant[E], cliquez sur continuer pour démarrer sans plus tarder!
Page 4 – Niveau d'activités physiques du participant au début du programme		
	S7.NIVEAU	Partie 1 – Votre niveau d'activités physiques<br>[PRENOM], voici le niveau d'activités physiques que vous aviez au début de l'aventure, accompagner d'où vous en étiez à la moitié du programme :<br><br>GRAPHIQUE avec trois mesures incluant les recommandations canadiennes d'activités physiques

		Peu importe où vous en êtes maintenant [PRENOM], nous vous encourageons à continuer dans la bonne directioS6.Vous espérez que vous verrez autant que nous, que vous possédez toute la motivation et la force nécessaire pour réussir à devenir une personne active régulièrement.<br><br>Cliquez sur continuer pour la suite
Page 5 – Votre motivation		
	S7.MOTLOW_INTRO<br><br>Microtailoring [S7.F.ELABCHOICE1] [S7.F.ELABCHOICE2]<br><br>Variable choisissant entre f.elab1 ou f.3pelab1 ou<br><br>Variable choisissant entre f.elab2 ou f.3pelab2<br><br><br>S7.F.Q.ELAB1<br>S7.F.Q.ELAB2<br><br><br>Variable choisissant entre q.elab1 ou q.elab3p1 ou « pourquoi voulez-vous éviter cette conséquence? »<br><br>Variable choisissant entre q.elab2 ou q.elab3p2 ou « pourquoi voulez-vous éviter cette conséquence? »	Partie 2 –Votre Motivation<br>[PRENOM], bien que vous étiez peu motivé[E] au début du programme, vous avez su identifier de nombreux éléments qui pourraient vous mener à pratiquer des activités physiques régulièrement. Voici en résumé, les éléments que vous avez mentionné :<br><br>Les conséquences que vous voulez éviter en pratiquant des activités physiques<br><br>[RISK1]<br>[RISK2]<br>[RISK3]<br>[RISK4]<br>[RISK5]<br>[RISK6]<br>[RISK7]<br>[RISK8]<br>[RISKOPEN]<br><br>Les raisons pour lesquelles vous voulez éviter certaines de ces conséquences<br><br>[S7.F.ELABCHOICE1] : S7.F.Q.ELAB1<br><br>[S7.F.ELABCHOICE2]: S7.F.Q.ELAB2<br><br>[RISKOPEN] : Pourquoi voulez-vous éviter cette conséquence [PRENOM]?<br><br>Les bénéfices reliés à la pratique d'activités physiques régulière qui vous importent :<br>[BEN1]<br>[BEN2]<br>[BEN3]<br>[BEN4]<br>[BEN5]<br>[BEN6]<br>[BEN7]<br>[BEN8]

	S7.B.QELABCH<br>OICE1<br><br>S7.B.QELABCH<br>OICE2	Les raisons pour lesquelles vous voulez obtenir certains de ces bénéfices<br>[BEN_1] : [S7.B.QELABCHOICE1]<br><br>[BEN_2] : [S7.B.QELABCHOICE2]<br><br>[BENOUV] : Pourquoi ce bénéfice est important pour vous [PRENOM]?<br><br>Deux valeurs importantes pour vous dans la vie : [VAL_1] et [VAL_2]<br><br>Voici comment le fait de pratiquer des activités physiques pourrait vous aider à [S3.QELA1_VAL] : [S3.Q.EX1]<br><br>Et voici comment le fait de pratiquer des activités physiques pourrait vous aider à [S3.QELA2_VAL] : [S3.Q.EX2]
	S7.MOTMIDLO<br>W_INTRO3PR	Partie 2 –Votre Motivation<br><br>[PRENOM], vous aviez peu l'intention de pratiquer des activités physiques au début du programme. D'un autre côté, vous ne sembliez cependant pas complètement démotivé(e). Pour continuer en ce sens, vous avez su identifier de nombreux éléments qui pourraient vous mener à pratiquer des activités physiques régulièrement. Voici en résumé, les éléments que vous avez mentionné :<br><br>Les conséquences que vous voulez éviter en pratiquant des activités physiques<br><br>[RISK1]<br>[RISK2]<br>[RISK3]<br>[RISK4]<br>[RISK5]<br>[RISK6]<br>[RISK7]<br>[RISK8]<br>[RISKOPEN]<br><br>Les raisons pour lesquelles vous voulez éviter certaines de ces conséquences<br><br>[S7.F.ELABCHOICE1] : S7.F.Q.ELAB1<br><br>[S7.F.ELABCHOICE2]: S7.F.Q.ELAB2<br><br>[RISKOPEN] : Pourquoi voulez-vous éviter cette conséquence [PRENOM]?

		Les bénéfices reliés à la pratique d'activités physiques régulière qui vous importent :<br>[BEN1]<br>[BEN2]<br>[BEN3]<br>[BEN4]<br>[BEN5]<br>[BEN6]<br>[BEN7]<br>[BEN8]<br><br>Les raisons pour lesquelles vous voulez obtenir certains de ces bénéfices<br>[BEN_1] : [S7.B.QELABCHOICE1]<br><br>[BEN_2] : [S7.B.QELABCHOICE2]<br><br>[BENOUV] : Pourquoi ce bénéfice est important pour vous [PRENOM]?<br><br>Deux valeurs importantes pour vous dans la vie : [VAL_1] et [VAL_2]<br><br>Voici comment le fait de pratiquer des activités physiques pourrait vous aider à [S3.QELA1_VAL] : [S3.Q.EX1]<br><br>Et voici comment le fait de pratiquer des activités physiques pourrait vous aider à [S3.QELA2_VAL] : [S3.Q.EX2]
	S7.MOTMIDHIGH_INTRO	Partie 2 –Votre Motivation<br><br>[PRENOM], vous aviez déjà une légère intention de pratiquer des activités physiques au début du programme. C'est excellent. Pour continuer en ce sens, vous avez su identifier de nombreux éléments qui pourraient vous mener à pratiquer des activités physiques régulièrement. Voici en résumé, les éléments que vous avez mentionné :<br><br>Les conséquences que vous voulez éviter en pratiquant des activités physiques<br><br>[RISK1]<br>[RISK2]<br>[RISK3]<br>[RISK4]<br>[RISK5]<br>[RISK6]<br>[RISK7]

		[RISK8]<br>[RISKOPEN]<br><br>Les raisons pour lesquelles vous voulez éviter certaines de ces conséquences<br><br>[S7.F.ELABCHOICE1] : S7.F.Q.ELAB1<br><br>[S7.F.ELABCHOICE2]: S7.F.Q.ELAB2<br><br>[RISKOPEN] : Pourquoi voulez-vous éviter cette conséquence [PRENOM]?<br><br>Les bénéfices reliés à la pratique d'activités physiques régulière qui vous importent :<br>[BEN1]<br>[BEN2]<br>[BEN3]<br>[BEN4]<br>[BEN5]<br>[BEN6]<br>[BEN7]<br>[BEN8]<br><br>Les raisons pour lesquelles vous voulez obtenir certains de ces bénéfices<br>[BEN_1] : [S7.B.QELABCHOICE1]<br><br>[BEN_2] : [S7.B.QELABCHOICE2]<br><br>[BENOUV] : Pourquoi ce bénéfice est important pour vous [PRENOM]?<br><br>Deux valeurs importantes pour vous dans la vie : [VAL_1] et [VAL_2]<br><br>Voici comment le fait de pratiquer des activités physiques pourrait vous aider à [S3.QELA1_VAL] : [S3.Q.EX1]<br><br>Et voici comment le fait de pratiquer des activités physiques pourrait vous aider à [S3.QELA2_VAL] : [S3.Q.EX2]
	S7.MOTHIGH_INTRO	Partie 2 –Votre Motivation<br><br>[PRENOM], vous aviez déjà une forte intention de pratiquer des activités physiques au début du programme. C'est excellent. Pour continuer en ce sens, vous avez su identifier de nombreux éléments qui pourraient vous mener à pratiquer des activités physiques régulièrement. Voici en résumé, les éléments que vous

		avez mentionné :<br><br>Les conséquences que vous voulez éviter en pratiquant des activités physiques<br><br>[RISK1]<br>[RISK2]<br>[RISK3]<br>[RISK4]<br>[RISK5]<br>[RISK6]<br>[RISK7]<br>[RISK8]<br>[RISKOPEN]<br><br>Les raisons pour lesquelles vous voulez éviter certaines de ces conséquences<br><br>[S7.F.ELABCHOICE1] : S7.F.Q.ELAB1<br><br>[S7.F.ELABCHOICE2]: S7.F.Q.ELAB2<br><br>[RISKOPEN] : Pourquoi voulez-vous éviter cette conséquence [PRENOM]?<br><br>Les bénéfices reliés à la pratique d'activités physiques régulière qui vous importent :<br>[BEN1]<br>[BEN2]<br>[BEN3]<br>[BEN4]<br>[BEN5]<br>[BEN6]<br>[BEN7]<br>[BEN8]<br><br>Les raisons pour lesquelles vous voulez obtenir certains de ces bénéfices<br>[BEN_1] : [S7.B.QELABCHOICE1]<br><br>[BEN_2] : [S7.B.QELABCHOICE2]<br><br>[BENOUV] : Pourquoi ce bénéfice est important pour vous [PRENOM]?<br><br>Deux valeurs importantes pour vous dans la vie : [VAL_1] et [VAL_2]<br><br>Voici comment le fait de pratiquer des activités physiques pourrait vous aider à [S3.QELA1_VAL] : [S3.Q.EX1]

		Et voici comment le fait de pratiquer des activités physiques pourrait vous aider à [S3.QELA2_VAL] : [S3.Q.EX2]
	S7.TRANSI.MOT	Nous espérons que cela vous aide à bien voir tous les éléments susceptibles de vous motiver [PRENOM]. Nous pensons sincèrement que vous avez évolué à travers ces séanceS.Ce fut du temps bien dépensé, que vous offrez à votre santé. Bravo [PRENOM].<br><br>Suite à ces séances, quels sont d'autres facteurs qui pourraient vous motiver encore plus?<br><br>Cliquez sur continuer pour la suite.
Page 6 – Vos forces, votre confiance et votre plan à long terme		
	S7.INTROEFFLOW	Vos forces, votre confiance et votre plan à long terme<br><br>Maintenant [PRENOM], parlons un peu de votre niveau de confiance en votre capacité de réussir à pratiquer des activités physiqueS.Afin de gagner plus de confiance, vous avez su identifier vos forces et vos solutions aux barrières pouvant vous empêcher de pratiques des activités physiqueS.
	S7.INTROEFFMIDLOW	Vos forces, votre confiance et votre plan à long terme<br><br>Maintenant [PRENOM], parlons un peu de votre niveau de confiance en votre capacité de réussir à pratiquer des activités physiqueS.Afin de gagner plus de confiance, vous avez su identifier vos forces et vos solutions aux barrières pouvant vous empêcher de pratiques des activités physiqueS5.
	S7.INTROEFFMIDHIGH	Vos forces, votre confiance et votre plan à long terme<br><br>Au début du programme, vous possédiez déjà une plutôt bonne confiance en votre capacité de réussir à pratiquer des activités physiques régulièrement [PRENOM]. Afin de gagner encore plus confiance vous avez su identifier vos forces et vos solutions aux barrières pouvant vous empêcher de pratiques des activités physiqueS5.
	S7.INTROEFFHIGH	Vos forces, votre confiance et votre plan à long terme<br><br>Au début du programme, vous possédiez déjà une excellente confiance en votre capacité de réussir à pratiquer des activités physiques régulièrement [PRENOM]. Afin de gagner encore plus confiance vous avez su identifier vos forces et vos solutions aux barrières pouvant vous empêcher de pratiques des activités physiqueS5.

	S7.STR_TABLEAU	Voici tous les éléments que vous avez évoqués démontrant votre force et votre capacité de réussir le défi de devenir une personne active régulièrement :<br><br>Les forces qui vous habitent :<br>[STR1]<br>[STR2]<br>[STR3]<br>[STR4]<br>[STR5]<br>[STR6]<br>[STR7]<br>[STR8]<br>[STR9]<br>[STR10]<br>[STR11]<br>[STR12]<br>[STR13]<br>[STR14]<br>[STR15]<br>[STR16]<br>[STR17]<br>[STR18]<br>[STR19]<br>[STR20]<br>[STR21]<br>[STR22]<br>[STR23]<br>[STR24]<br>[STR25]<br><br>Vos forces principales et comment elles pourraient vous aider à pratiquer régulièrement des activités physiques<br><br>[STR_1]<br>[STR_VALORI1] pourrait vous aider à pratiquer davantage d'activités physiques de cette façon : [S5.Q4]<br><br><br>[STR_2]<br>[STR_VALORI2] pourrait vous aider à pratiquer davantage d'activités physiques de cette façon : [S5.Q5]<br><br>Vos solutions aux barrières potentielles<br><br>[S6.REF_BAR] ----------------------→ [S6.REF_SOL1]

		Voici tous les éléments que vous avez évoqués démontrant votre force et votre capacité de réussir le défi de devenir une personne active régulièrement :<br><br>Les forces qui vous habitent :<br>[STR1]<br>[STR2]<br>[STR3]<br>[STR4]<br>[STR5]<br>[STR6]<br>[STR7]<br>[STR8]<br>[STR9]<br>[STR10]<br>[STR11]<br>[STR12]<br>[STR13]<br>[STR14]<br>[STR15]<br>[STR16]<br>[STR17]<br>[STR18]<br>[STR19]<br>[STR20]<br>[STR21]<br>[STR22]<br>[STR23]<br>[STR24]<br>[STR25]<br><br>Vos forces principales et comment elles pourraient vous aider à pratiquer régulièrement des activités physiques<br><br>[STR_1]<br>[STR_VALORI1] pourrait vous aider à pratiquer davantage d'activités physiques de cette façon : [S5.Q4]<br><br>[STR_2]<br>[STR_VALORI2] pourrait vous aider à pratiquer davantage d'activités physiques de cette façon : [S5.Q5]<br><br>Vos solutions aux barrières potentielles<br><br>[S6.2REF_BAR1] -----→ [S6.REF_SOL1]<br>[S6.2REF_BAR2] -----→ [S6.REF_SOL2]<br><br>Quelles solutions vous assureraient de réussir à pratiquer des activités physiques régulièrement [PRENOM]? Pensez à des

		solutions réalistes et n'arrêtez jamais d'essayer, et ce, jusqu'à l'atteinte de votre objectif. Vous en êtes capable et vous possédez la force nécessaire [PRENOM]. Vous pouvez définitivement croire en vos capacités de réussir, peu importe ce qu'il advient.
	S7.TRANSI_EFF	Où se situe votre confiance maintenant [PRENOM]? Comment pourrait-elle être encore plus forte?<br><br>Nous sommes convaincus que vous possédez tout ce qu'il faut pour pratiquer des activités physiques régulièrement. Tout le monde peut marcher ou alors faire du vélo. Ce sont d'excellentes activitéS5.<br><br>Cliquez sur continuer pour la suite.
Page 7 – Wrap-up		
	S7.WRAP	Finalement, vers où tout cela vous mène [PRENOM]?<br><br>À travers ce programme, vous avez identifié plusieurs raisons vous aidant à développer une attitude positive et une forte motivation à pratiquer des activités physiques régulièrement.<br><br>Vous avez identifié des éléments pouvant vous donner confiance en votre capacité de réussir un beau défi.<br><br>Vous aimeriez réussir à pratiquer régulièrement [S6.Q1] minutes d'activités physiques par semaine d'ici un aS6.Les activités physiques que vous aimez pratiquer sont [S6.Q2]. Les raisons principales pour lesquelles vous voulez réussir ce défi sont les suivantes : [S6.Q3].<br><br>De plus, il semble que vous vous sentiriez [S6.FEEL] de réussir cet objectif [PRENOM].<br><br>Vous avez progressé énormément à travers ce programme [PRENOM]. Vous avez plusieurs éléments en votre possession pour vous motiver et vous donner confiance, quels sont donc les éléments qui pourraient vous faire continuer à aller dans cette direction? Vous avez tout pour réussir ce défi [PRENOM] et beaucoup de positif vous attend.<br><br>Comme nous l'avons évoqué plus tôt dans le programme, planifier sa pratique d'activités physiques à court terme peut s'avérer essentiel pour passer à l'action et prendre goût à la pratique d'activités physiques régulière.<br><br>La prochaine page vous proposera pour une dernière fois de passer à l'action et planifier vos activités physiques [PRENOM]. N'hésitez surtout pas à l'essayer si vous ne l'avez pas fait encore! Pour les plus habitués, n'hésitez pas à foncer également!

		Cliquez sur continuer pour avancer [PRENOM]!
Page 8 – Plan d'action		
	S7.PLAN	Voilà l'occasion de planifier votre pratique une dernière fois avec Diabète en Forme.<br><br>Pour continuer de vous accompagner même après le programme [PRENOM], nous vous enverrons par courriel une version imprimable du plan d'action que vous pourrez utiliser à tout moment de votre vie. Ainsi, vous pourrez conserver l'habitude de bouger régulièrement en planifiant vos activités!<br><br>Vous recevrez également un courriel à la dernière semaine du programme vous invitant à remplir le dernier questionnaire dans le cadre du projet de recherche. Nous apprécierions énormément avoir vos réponses à ce questionnaire. En fait, vos réponses sont essentielles afin de préserver la plus haute qualité de notre étude et améliorer les services qui vous seront offerts à l'avenir.<br><br>Maintenant que tout cela est dit, aimeriez-vous planifier votre pratique d'activités physiques pour les prochains jours [PRENOM]?<br><br>Oui, je veux me faire un plan d'action<br>Non, je ne veux pas me faire un plan d'action
Page 9 – Réponse à la personne ayant dit non		
	S7.NOPLAN	D'accord [PRENOM], merci énormément d'avoir complété les 6 séances d'information du programme.<br><br>Nous espérons que tout ce cheminement vous a aidé à en connaître davantage sur votre pratique d'activités physiques et à développer votre motivatioS6.Nous espérons aussi que ce programme vous a permis de prendre confiance en vous au point de devenir une personne active régulièrement pour la vie<br><br>Vous aurez des nouvelles de nous par courriel très bientôt!<br><br>Merci pour tout [PRENOM] et encore une fois, bravo pour votre parcours avec nous!<br><br>L'équipe de Diabète en Forme
**Action plan tool**		
Page 1 - Introduction		
Introduction si la	PLAN.INTRO1	[PRENOM], vous voyez actuellement un ou plusieurs facteurs qui pourraient vous empêcher de faire plus d'activités physiques

personne mentionne qu'elle voit des barrières qui l'empêcherait de faire davantage d'activités physiques		qu'actuellement.<br><br>Ensemble, notre défi sera donc d'identifier ces facteurs et d'y trouver des solutions efficaces pour que vous soyez en mesure de devenir une personne plus active.<br><br>Allons-y sans plus tarder [PRENOM].<br><br>Cliquez sur continuer
Introduction si la personne veut simplement se fixer un plan d'action	PLAN.INTRO2	Bienvenue dans l'outil « plan d'action » de Diabète en Forme [PRENOM]!<br><br>S'il s'agit de votre première expérience avec cet outil, vous pouvez lire la courte introduction ci-dessous qui vous explique l'utilité de vous construire un plan d'actioS6.Autrement, vous pouvez continuer directement à la page suivante!<br><br>**Un plan d'action par semaine = prendre une bonne habitude**<br><br>En effet [PRENOM], un plan d'action est très utile lorsque nous voulons changer nos habitudes, et ainsi faire plus d'activités physiqueS.Pour chacune des huit semaines du programme, l'outil « Plan d'action » pourra vous permettre de :<br><br>1. Vous fixer un objectif hebdomadaire réaliste en nombre de minutes d'activités physique.<br>2. Établir un plan simple et concret pour réussir cet objectif<br>3. Vous engager à exécuter ce PLAN.<br><br>En utilisant cet outil, il vous sera ainsi plus facile de passer à l'action!<br><br>Cliquez sur continuer pour commencer votre plan [PRENOM]!
Page 2 – Question sur les barrières		
	PLAN.BARR	QUELLES SONT VOS BARRIÈRES [PRENOM]?<br><br>La première étape est de tenter d'identifier clairement les facteurs qui vous empêcheraient de faire plus d'activités physiqueS5.<br><br>Ci-dessous, vous trouverez une liste de barrières avec lesquelles d'autres personnes atteintes du diabète de type 2 ont eu des difficultés [PRENOM].<br><br>Parmi ces barrières, quelles sont celles qui vous empêcheraient le plus de faire davantage d'activités physiques?<br>(sélectionnez 1 ou 2 réponses)<br><br>Être trop fatigué pour faire de l'activité physique

		Avoir beaucoup de choses à faire mis à part l'activité physique<br>Avoir à faire de l'activité physique quand la température est mauvaise (hiver/été)<br>Ne pas avoir de partenaire avec qui faire de l'activité physique<br>Ne pas avoir accès à des emplacements pour faire de l'activité physique<br>Ne pas avoir d'argent pour faire de l'activité physique<br>Être trop gênée/manquer de confiance pour aller faire de l'activité physique<br>Avoir peur de ne pas être assez en santé pour faire de l'activité physique<br>Manquer de motivation<br>Je veux inscrire une autre réponse
	PLAN.BARROPEN	D'accord [PRENOM], quelle serait votre barrière plus personnelle?<br>(espace de réponse)
	PLAN.TRANSIP2P3	Cliquez sur continuer lorsque vos choix seront faitS5.
Page 3 –Solutions aux barrières		
	PLAN.FBBARR1	mmmm….<br><br>Vous avez peur que votre fatigue vous empêche de faire plus d'activités physiqueS.Si vous le voulez, tentons ensemble d'y trouver une solutioS6.
	PLAN.FBBARR2	mmmm….<br>Vous avez peur d'avoir un horaire trop chargé pour faire plus d'activités physiqueS.Si vous le voulez, tentons ensemble d'y trouver une solutioS6.
	PLAN.FBBARR3	mmmm….<br>Vous pensez que le fait d'avoir à faire des activités physiques lorsque la température est mauvaise vous empêcherait de faire plus d'activités physiqueS.Si vous le voulez, tentons ensemble d'y trouver une solutioS6.
	PLAN.FBBARR4	mmmm….<br>Vous pensez que le fait de ne pas avoir de partenaire pourrait vous empêcher de faire plus d'activités physiqueS.Si vous le voulez, tentons ensemble d'y trouver une solutioS6.
	PLAN.FBBARR5	mmmm…<br>Vous pensez que le manque d'emplacements pourrait vous empêcher de faire plus d'activités physiqueS.Si vous le voulez, tentons ensemble d'y trouver une solutioS6.
	PLAN.FBBARR6	mmmm….<br>Vous pensez ne pas avoir suffisamment d'argent pour faire plus d'activités physiqueS.Si vous le voulez, tentons ensemble d'y trouver une solutioS6.
	PLAN.FBBARR7	mmmm….<br>Vous pensez que votre gêne pourrait vous empêcher de faire plus d'activités physiqueS.Si vous le voulez, tentons ensemble d'y trouver une solutioS6.
	PLAN.FBBARR8	mmmm….<br>Vous avez peur que votre santé vous empêche de faire plus d'activités physiqueS.Si vous le voulez, tentons ensemble d'y trouver une solutioS6.
	PLAN.FBBARR9	mmmm….

		Vous pensez que votre manque de motivation pourrait vous empêcher de faire plus d'activités physiqueS.Si vous le voulez, tentons ensemble d'y trouver une solutioS6.
	PLAN.FBBARROPEN	mmmm….<br>Vous avez identifié une barrière plus personnelle [PRENOM]. La voici : [PLAN.BARROPEN].
	PLAN.SOLINTRO	Ci-dessous, vous trouverez des solutions que d'autres personnes atteintes du diabète de type 2 ont utilisées pour vaincre la ou les mêmes barrières que vouS5.
	PLAN.SOL1	Quelle pourrait être une solution pour vaincre votre fatigue ?<br><br>1= Je vais faire de l'activité tôt dans la journée, le matin ou le midi.<br>2= J'essaie de garder en tête que si je fais de l'activité physique, j'aurai de plus en plus d'énergie dans la vie.<br>3= Je vais planifier des activités avec d'autres personnes qui me motiveront<br>4=Je prends une collation pour me donner de l'énergie et j'y vais quand même<br>5= réponse personnelle
	PLAN.SOL2	Quelle pourrait être une solution pour composer avec votre horaire chargé?<br>1= Faire de l'activité physique par bloc de 10 minutes les journées où je n'ai pas le tempS.Ex : 10 minutes le matin, 10 minutes l'après midi et 10 minutes le soir<br>2= Je vais remplacer des activités où je suis assis (télé, ordinateur) par des activités physiques<br>3=Je vais me procurer un agenda et inscrire dans mon horaire les moments où je vais faire de l'activité physique<br>4= Je vais prendre des pauses de travail actives et faire des 10 minutes de marche.<br>5= Je vais me fixer des rendez-vous avec d'autres personnes qui me plaisent pour aller faire de l'activité physique
	PLAN.SOL3	Quelle pourrait être une solution pour vaincre la mauvaise température?<br>1= je vais me procurer l'équipement nécessaire pour en faire même quand il pleut, quand il neige ou quand il fait trop chaud.<br>2= Je vais faire de l'activité physique chez nous ou à l'intérieur dans un endroit que j'aurai choisi quand il fait moins beau<br>3= Je vais me préparer une activité physique alternative au cas où il ne fait pas assez beau pour aller dehors
	PLAN.SOL4	Quelle solution pourrait vous aider à pallier avec le fait que vous n'avez pas de partenaire?<br>1=Je vais me joindre à un groupe qui pratique l'activité physique que j'aime. Ex : la marche, l'aquaforme ou la natation<br>2= Je vais inviter mes amis, ma famille ou mes collègues à faire de l'activité physique avec moi<br>3= Je vais amener mon chien marcher avec moi<br>4= Pour moins me sentir seule, je vais aussi participer dans des discussions en ligne sur des pages facebook : Diabète Québec et autreS5.
	PLAN.SOL5	Quelle solution pourrait vous aider à identifier un emplacement pour faires des activités physiques?<br>1= Je vais faire de la marche ou du vélo dehors, c'est simple et gratuit.<br>2= Je vais faire de l'activité physique dans ma maison (yoga, programme d'AP sur DVD, vélo stationnaire, tapis roulant, élliptique).<br>3= Je vais aller voir au centre communautaire de mon quartier pour me donner des options<br>4= je vais discuter avec mon médecin, avec un ami ou un spécialiste de l'activité physique pour avoir des conseils
	PLAN.SOL6	Quelle solution pourrait vous aider à faire plus d'activités

		physiques malgré votre manque d'argent?<br>1= je vais trouver des activités physiques qui ne coûte rien : marcher dehors, faire du vélo, nager à la piscine communautaire.<br>2= Je vais aller voir au centre communautaire de mon quartier pour me donner des options<br>3=Je vais économiser quelques sous pour m'acheter un DVD d'activité physique ou pour une autre activité physique que j'aimerais faire<br>4= Je vais aller marcher avec ma famille, des amis ou des collègues
	PLAN.SOL7	Quelle solution pourrait vous aider à faire plus d'activités physiques malgré votre gêne?<br>1= Je vais choisir une activité physique simple comme la marche ou le vélo.<br>2= Je vais aller faire de l'activité physique avec des gens qui m'acceptent comme je suis, qui me juge paS5.<br>3= Au départ, je vais faire de l'activité physique dans des endroits où je suis seul pour prendre confiance.<br>4=Je vais éviter des endroits qui me gêne le plus comme les centres de conditionnement.
	PLAN.SOL8	Quelle solution pourrait vous aider à faire plus d'activités physiques malgré votre peur pour votre santé?<br>1= Je vais consulter mon médecin pour mettre au clair si je peux faire de l'activité physique<br>2= Je vais consulter un spécialiste de l'activité physique pour qu'il me rassure et me conseille sur ce qui est sécuritaire pour moi<br>3= Je vais commencer à petite dose. 10 à 20 minutes maximum les jours où j'en fais et voir comment ça va.<br>4= Je vais faire de l'activité physique accompagnée d'un spécialiste de l'activité physique<br>5= Je vais faire de l'activité physique dans un groupe où un spécialiste de l'AP est présent
	PLAN.SOL9	Quelle solution pourrait vous aider à faire plus d'activités physiques malgré votre manque de motivation?<br>1= je signe un contrat papier avec moi-même comme quoi je m'engage à être actif<br>2= je signe un contrat papier avec mes proches comme quoi je m'engage à être actif<br>3=Je vais essayer de nouvelles activités physiques pour trouver celles qui me plaisent vraiment<br>4= À chaque fois que je dois faire de l'activité physique, je prends un temps pour me rappeler ce que cela va m'apporter de positif dans ma vie, à court et long terme.<br>5= Je vais faire de l'activité physique avec des gens que j'aime pour me motiver
	PLAN.SOLOPEN	Selon vous, quelle solution pourrait vous aider à vaincre cette barrière?<br>(OPEN)
	PLAN.TRANSIP3 P4	Prenez le temps de bien réfléchir à vos options et cliquez sur continuer lorsque vous avez terminé.
Page 4 –Feedback solution		
	PLAN.FBSOLINT RO	Excellent [PRENOM], il semble que vous ayez trouvé des pistes de solutions pour vaincre la ou les barrières qui vous empêcheraient de faire plus d'activités physiqueS.<br>Voilà un pas de plus vers une vie plus active!<br><br>Rappelez-vous, ce n'est pas grave si la solution que vous avez choisie ne fonctionne paS.Il vous sera toujours possible d'essayer d'autres solutionS.L'important est donc de ne jamais abandonner. Vous êtes capable d'entreprendre ce changement [PRENOM], il suffit de trouver la solution qui fonctionne pour vous et vous l'avez peut-être déjà trouvé!<br><br>Voici donc votre plan pour vaincre votre, ou alors vos barrières cette semaine :

	PLAN.FBSOL1	(SOUS FORME DE TABLEAU)<br><br>SEMAINE DU --------<br><br>Votre barrière<br>[PLAN.BARRMICRO1]<br><br>Votre solution pour y remédier :<br>[PLAN.SOLMICRO1]<br><br>Questions supplémentaires pour raffiner votre plan :<br>Quand dans la semaine appliquerez-vous ces solutions? Quel jour de la semaine? À quel moment de la journée? Après quelle activité de votre journée appliquerez-vous cette solution?
	PLAN.FBSOL2	(SOUS FORME DE TABLEAU)<br><br>SEMAINE DU --------<br><br>Votre première barrière<br>[PLAN.BARRMICRO1]<br><br>Votre solution pour y remédier :<br>[PLAN.SOLMICRO1]<br><br>Votre deuxième barrière<br>[PLAN.BARRMICRO2]<br><br>Votre solution pour y remédier :<br>[PLAN.SOLMICRO2]<br><br>Questions supplémentaires pour raffiner votre plan :<br>Quand dans la semaine appliquerez-vous ces solutions? Quel jour de la semaine? À quel moment de la journée? Après quelle activité de votre journée appliquerez-vous cette solution?
	PLAN.TRANSIP4<br>P5	Vos solutions vous seront transmises par courriel [PRENOM]. Vous pourrez donc l'imprimer ou alors y avoir accès en tout tempS.Nous avons confiance que vous pouvez vaincre vos barrièreS5.<br><br>Maintenant, aimeriez-vous également planifier vos activités physiques de cette semaine [PRENOM]?<br><br>Oui, je veux planifier mes activités de cette semaine<br>Non merci, je veux maintenant quitter
Page 5 – Choisir la semaine		

<table>
<tr><td></td><td>PLAN.SEM</td><td>Commençons dès maintenant [PRENOM]!

Pouvez-vous d'abord indiquer pour quelle semaine du programme vous voulez construire un plan d'action aujourd'hui?

Semaine du 1er septembre
Semaine du 8 septembre
Semaine du 15 septembre
Semaine du 22 septembre
Semaine du 29 septembre
Semaine du 6 octobre
Semaine du 13 octobre
Semaine du 20 octobre
Cliquez sur continuer par la suite.</td></tr>
<tr><td colspan="3">Page 6 – Fixation d'objectif pour la semaine</td></tr>
<tr><td></td><td>PLAN.OBJECTIF</td><td>Votre niveau d'activités physiques au début du programme : [F.APTOT] minutes d'activités physiques
Votre objectif pour la semaine du [PLAN.DATE] : ??

Excellent [PRENOM],

Avant d'inscrire votre objectif, nous tenons à vous encourager à fixer un objectif supérieur au niveau que vous aviez au début du programme. Lancez-vous un défi à relever [PRENOM], vous en êtes capable!

Maintenant, combien voulez-vous faire de minutes d'activités physiques à intensité modérée pour la semaine choisie?

(open) minutes d'activités physiques modérées

Il est également important que vous choisissiez un objectif que vous pensez être capable d'atteindre [PRENOM]. Nous pensons que si vous confiance se situe entre 6 à 10 sur une échelle de 10, vous aurez de bonnes chances d'y arriver. N'ayez donc pas peur de réviser votre objectif en conséquence!

À quel niveau se situe votre confiance en votre capacité de réussir cet objectif [PRENOM]?

6 – Cela représente un défi, mais je peux réussir cet objectif
7
8— J'ai une très bonne confiance de réussir mon objectif
9
10 – Je suis convaincu(e) hors de tout doute que je réussirai mon objectif</td></tr>
<tr><td></td><td></td><td>Cliquez sur continuer lorsque vous avez complété cette étape.</td></tr>
<tr><td colspan="3">Page 7 – Les activités</td></tr>
<tr><td></td><td>PLAN.FBOBJECTIF67</td><td>Bravo! C'est un beau défi que vous avez devant vous [PRENOM]. Nous sommes convaincus que vous trouverez la force nécessaire</td></tr>
</table>

		pour réussir votre objectif.
	PLAN.FBOBJECTIF89	Vous semblez possédez une bonne confiance en votre capacité de réussir cet objectif [PRENOM]. Excellente nouvelle! Nous sommes également convaincus que vous trouverez la force nécessaire pour réussir votre objectif.
	PLAN.FBOBJECTIF10	Vous semblez possédez une excellente confiance en votre capacité de réussir cet objectif [PRENOM]. C'est une très bonne nouvelle! Nous sommes également convaincus que vous trouverez la force nécessaire pour réussir votre objectif.
	PLAN.ACTIVITÉS	Les activités que vous ferez pour votre semaine<br><br>Maintenant, choisissez les activités qui vous mèneront à atteindre votre objectif [PRENOM].<br>(Sélectionnez 3 activités maximum)<br><br>1=Marche<br>10=Golf (sans voiturette)<br>100= la bicyclette<br>1000= le patinage<br>10000= la natation/nage<br>100000= le tennis<br>1000000= la danse<br>10000000= le jardinage<br>100000000= le jogging<br>1000000000= le vélo stationnaire<br>10000000000=le tapis roulant<br>100000000000= l'elliptique<br>1000000000000=hockey<br>10000000000000=basketball<br>100000000000000=soccer<br>1000000000000000=centre de conditionnement<br>10000000000000000= le ski alpin<br>100000000000000000= le ski de fond<br>1000000000000000000= la raquette d'hiver<br>10000000000000000000= Je veux inscrire une autre activité<br><br>Cliquez sur continuer par la suite.
	PLAN.ACTIVITEOPEN	Quelle est cette activité [PRENOM]?
Page 8 – jours de la semaine		
	PLAN.JOURINTRO	Très bien! Maintenant, choisissez les jours de la semaine où vous ferez chacune de vos activités [PRENOM].
	PLAN.JOUR01	Faire de la marche<br>Lundi<br>Mardi<br>Mercredi<br>Jeudi<br>Vendredi<br>Samedi<br>Dimanche
	PLAN.JOUR02	Jouer au golf<br>Lundi<br>Mardi<br>Mercredi

		Jeudi<br>Vendredi<br>Samedi<br>Dimanche
	PLAN.JOUR03	La bicyclette<br>Lundi<br>Mardi<br>Mercredi<br>Jeudi<br>Vendredi<br>Samedi<br>Dimanche
	PLAN.JOUR04	Le patinage<br>Lundi<br>Mardi<br>Mercredi<br>Jeudi<br>Vendredi<br>Samedi<br>Dimanche
	PLAN.JOUR05	La natation<br>Lundi<br>Mardi<br>Mercredi<br>Jeudi<br>Vendredi<br>Samedi<br>Dimanche
	PLAN.JOUR06	Le tennis<br>Lundi<br>Mardi<br>Mercredi<br>Jeudi<br>Vendredi<br>Samedi<br>Dimanche
	PLAN.JOUR07	La danse<br>Lundi<br>Mardi<br>Mercredi<br>Jeudi<br>Vendredi<br>Samedi<br>Dimanche
	PLAN.JOUR08	Le jardinage<br>Lundi<br>Mardi<br>Mercredi<br>Jeudi<br>Vendredi

		Samedi<br>Dimanche
	PLAN.JOUR09	Le jogging<br>Lundi<br>Mardi<br>Mercredi<br>Jeudi<br>Vendredi<br>Samedi<br>Dimanche
	PLAN.JOUR10	Le vélo stationnaire<br>Lundi<br>Mardi<br>Mercredi<br>Jeudi<br>Vendredi<br>Samedi<br>Dimanche
	PLAN.JOUR11	Le tapis roulant<br>Lundi<br>Mardi<br>Mercredi<br>Jeudi<br>Vendredi<br>Samedi<br>Dimanche
	PLAN.JOUR12	L'elliptique<br>Lundi<br>Mardi<br>Mercredi<br>Jeudi<br>Vendredi<br>Samedi<br>Dimanche
	PLAN.JOUR13	Le hockey<br>Lundi<br>Mardi<br>Mercredi<br>Jeudi<br>Vendredi<br>Samedi<br>Dimanche
	PLAN.JOUR14	Le basketball<br>Lundi<br>Mardi<br>Mercredi<br>Jeudi<br>Vendredi<br>Samedi<br>Dimanche

	PLAN.JOUR15	Le soccer<br>Lundi<br>Mardi<br>Mercredi<br>Jeudi<br>Vendredi<br>Samedi<br>Dimanche
	PLAN.JOUR16	Aller au centre de conditionnement (gym)<br>Lundi<br>Mardi<br>Mercredi<br>Jeudi<br>Vendredi<br>Samedi<br>Dimanche
	PLAN.JOUR17	Le ski alpin<br>Lundi<br>Mardi<br>Mercredi<br>Jeudi<br>Vendredi<br>Samedi<br>Dimanche
	PLAN.JOUR18	Le ski de fond<br>Lundi<br>Mardi<br>Mercredi<br>Jeudi<br>Vendredi<br>Samedi<br>Dimanche
	PLAN.JOUR19	La raquette d'hiver<br>Lundi<br>Mardi<br>Mercredi<br>Jeudi<br>Vendredi<br>Samedi<br>Dimanche
	PLAN.JOUR20	PLAN.ACTIVITEOPEN<br>Lundi<br>Mardi<br>Mercredi<br>Jeudi<br>Vendredi<br>Samedi<br>Dimanche
		Cliquez sur continuer lorsque vous aurez fait vos choix [PRENOM].

Page 9 – Raisons d'effectuer ce changement		
	PLAN.RAISONS	Très bien [PRENOM], il ne reste plus qu'une étape. De plus, en fonction de vos réponses, vous devriez faire en moyenne [PLAN.APMOYENNE] minutes par activité physique que vous ferez dans votre semaine.<br><br>Voici une dernière question pour compléter votre plan d'action [PRENOM] :<br><br>Pour quelles raisons principales voulez-vous réaliser l'objectif que vous vous êtes fixez?<br><br>(open)<br><br>Exemple : Je veux me prendre en maiS6.Je veux pouvoir avoir plus d'énergie et faire plein d'activités que j'aime sans baisse d'énergie.<br><br>Votre plan est maintenant fin prêt! Cliquez sur continuer pour y accéder [PRENOM].
Page 10 – Présentation du plan d'action		
	PLAN.PLAN	PLAN D'ACTION COMPLÉTÉ!<br><br>C'est fait [PRENOM], il ne vous reste qu'à mettre votre plan à exécution! Nous sommes convaincus que vous en êtes capable. Une copie de votre plan vous sera envoyée par courriel!<br><br>Votre plan pour la semaine du [PLAN.DATE]<br><br>Cette semaine vous ferez : [PLAN.OBJECTIF]<br>Votre niveau de confiance : [PLAN.CONFIANCE]<br><br>Vos activités tout au long de la semaine :<br>Lundi Mardi Mercredi Jeudi Vendredi Samedi Dimanche<br>21 variables : [PLAN.AP1LUNDI] à [PLAN.AP1DIMANCHE]<br>[PLAN.AP2LUNDI] à [PLAN.AP2DIMANCHE]<br>[PLAN.AP3LUNDI] à [PLAN.AP3DIMANCHE]
		Nombre de minutes par activité recommandée : [PLAN.APMOYENNE]<br><br>Les raisons principales qui vous poussent à réussir cet objectif : [PLAN.RAISON]<br><br>Questions complémentaires :<br>À quelle heure ferez-vous chacune de vos activités?<br>Après quelle activité de la journée irez-vous faire votre activité? (Exemple : après le déjeuner, après m'être brossé les dents le matin, etc.)

		Avez-vous besoin de prévoir d'autres éléments pour vous assurer d'effectuer vos activités physiques?<br><br>Nous vous recontacterons par courriel très bientôt [PRENOM]!<br><br>À votre santé,<br><br>L'équipe de Diabète en Forme<br><br># Je veux maintenant retourner à l'accueil

# Emails

Email de rappel invitant les gens à compléter leur inscription

Vous voulez toujours participer au projet Diabète en Forme?

Bonjour [PRENOM],

Merci énormément de vous intéresser au projet Diabète en Forme. Grâce à votre participation, nous sommes convaincus que nous sortirons ensemble gagnants de cette expérience.

Comme vous le savez sans doute déjà, Diabète en Forme peut vous accompagner dans votre démarche liée à votre pratique d'activités physiques en vous fournissant plusieurs informations personnaliséeS.

Pour participer au propramme/projet, il vous suffit de compléter votre inscription en cliquant sur le lien suivant : www……com

Le programme débutera le 1er septembre, il faut reste donc [RECRU_JOURS] pour vous inscrire!

Au plaisir d'évoluer avec vous sur Diabète en Forme [PRENOM],

Michel Moreau Lapointe, responsable du projet de recherche

2ième e-mail de rappel invitant les gens à compléter leur inscription

Il est encore temps de participer au projet Diabète en Forme!

Bonjour [PRENOM],

Vous le savez déjà, Diabète en Forme peut vous accompagner dans votre démarche liée à votre pratique d’activités physiques en vous fournissant plusieurs informations personnaliséeS.

Pour participer au propramme/projet, il vous suffit de compléter votre inscription en cliquant sur le lien suivant : www……com

Le programme débutera le 1er septembre, il faut reste donc [RECRU_JOURS] pour vous inscrire! Il faut faire vite!

Merci énormément de vous intéresser au projet Diabète en Forme. Grâce à votre participation, nous sommes convaincus que nous sortirons ensemble gagnants de cette expérience.

Au plaisir d’évoluer avec vous sur Diabète en Forme [PRENOM],

Michel Moreau Lapointe, responsable du projet de recherche

E-mail annonçant la 1ière séance aux gens

UNE PREMIÈRE SÉANCE D’INFORMATIONS EST DISPONIBLE POUR VOUS

Bonjour [PRENOM]!

Félicitations d’avoir complété votre séance d’inscription sur Diabète en Forme. Une belle expérience vous attend!

Bonne nouvelle, la première séance intitulée « Votre niveau actuel d’activités physiques » est déjà disponible pour vouS.Nous vous encourageons à aller réaliser celle-ci dans les meilleurs délais afin de progresser à travers le programme de Diabète en Forme.

Pour accéder à votre séance, cliquez sur le lien suivant : www….com

Au plaisir de vous retrouver sur le site [PRENOM],

Michel Moreau Lapointe, responsable du projet recherche

E-mail rappelant la 1ière séance aux gens

COMPLÉTER SA PREMIÈRE SÉANCE D’INFORMATIONS

Bonjour [PRENOM]!

Nous serons déjà à la fin de la première semaine du programme Diabète en Forme.

Nous voulions simplement vous encourager à compléter votre toute première séance intitulée « Votre niveau actuel d'activités physiques » qui est disponible pour vous sur le site internet. Nous vous encourageons à aller réaliser celle-ci afin d'avoir accès à votre deuxième séance la semaine prochaine!

Pour accéder à votre séance, cliquez sur le lien suivant : www….com

Ce sera encore une fois un plaisir de vous retrouver sur le site [PRENOM],

Michel Moreau Lapointe, responsable du projet recherche

E-mail annonçant la 2ième séance aux gens

VOTRE DEUXIÈME SÉANCE D'INFORMATIONS EST MAINTENANT DISPONIBLE

Bonjour [PRENOM]!

Nous voilà déjà à la deuxième semaine du programme. Félicitations!

Bonne nouvelle, si vous avez complété votre première séance d'informations, votre deuxième séance intitulée « Votre motivation et les bénéfices de l'activité physique » est maintenant disponible pour vouS.Nous vous encourageons à aller réaliser celle-ci dans les meilleurs délais afin de toujours continuer à progresser à travers le programme de Diabète en Forme.

Pour accéder à votre deuxième séance, cliquez sur le lien suivant : www….com

Pour compléter votre première séance, cliquez sur le lien suivant : www…com

J'espère que vous appréciez votre expérience sur Diabète en Forme jusqu'à présent!

Michel Moreau Lapointe, responsable du projet recherche

E-mail rappelant la 2ième séance aux gens

COMPLÉTER VOTRE DEUXIÈME SÉANCE D'INFORMATIONS

Bonjour [PRENOM]!

Nous serons déjà à la fin de la deuxième semaine du programme Diabète en Forme.

Nous voulions simplement vous encourager à compléter votre deuxième séance intitulée « Votre motivation et les bénéfices de l'activité physique » qui est disponible pour vous sur le site internet. Nous vous encourageons à aller réaliser celle-ci afin d'avoir accès à votre troisième séance la semaine prochaine!

Si vous n'avez toujours pas réalisé votre première séance, faites vite avant que le retard devienne irrattrapable!

Pour accéder à votre séance, cliquez sur le lien suivant : www….com

Pour compléter votre première séance, cliquez sur le lien suivant : www…com

Ce sera toujours un plaisir [PRENOM],

Michel Moreau Lapointe, responsable du projet recherche

E-mail annonçant la 3ième séance aux gens

BIENTÔT À MI-PARCOURS : VOTRE TROISIÈME SÉANCE D'INFORMATIONS EST MAINTENANT DISPONIBLE

Bonjour [PRENOM]!

Nous voilà déjà à la deuxième semaine du programme. Félicitations!

Bonne nouvelle, si vous avez complété votre deuxième séance d'informations, votre troisième séance intitulée « Vos valeurs et l'activité physique » est maintenant disponible pour vouS.Comme à l'habitude, nous vous encourageons à aller réaliser celle-ci dans les meilleurs délais afin de toujours continuer à progresser à travers le programme de Diabète en Forme [PRENOM].

Pour accéder à votre troisième séance, cliquez sur le lien suivant : www….com

Pour compléter votre deuxième séance, cliquez sur le lien suivant : www…com

À votre santé!

Michel Moreau Lapointe, responsable du projet recherche

E-mail rappelant la 3ième séance aux gens

COMPLÉTER VOTRE TROISIÈME SÉANCE D'INFORMATIONS

Bonjour [PRENOM]!

Nous serons déjà à la fin de la troisième semaine du programme Diabète en Forme.

Nous voulons simplement vous amener à compléter votre troisième séance intitulée « Vos valeurs et l'activité physique » qui est présentement disponible pour vouS.Nous vous encourageons à aller réaliser celle-ci afin d'avancer et d'avoir accès à votre quatrième séance très bientôt!

Si vous n'avez toujours pas réalisé votre deuxième séance, faites vite avant que le retard devienne irrattrapable!

Pour accéder à votre séance, cliquez sur le lien suivant : www….com

Pour compléter votre deuxième séance, cliquez sur le lien suivant : www…com

Ce sera toujours un plaisir [PRENOM],

Michel Moreau Lapointe, responsable du projet recherche

E-mail annonçant la mid-check up séance aux gens

PETITE MISE À JOUR

Bonjour [PRENOM]!

J'espère que vous allez bien et que vous appréciez votre expérience maintenant que vous êtes déjà à la mi-chemin du programme. Bravo!

Suite à vos premières séances d'informations sur Diabète en Forme, nous sommes intéressés à connaître vos niveaux de motivation et d'activités physiques actuelS. En cliquant sur le lien un peu plus bas, vous pourrez répondre à environ une dizaine de questions rapides qui nous permettront de savoir où vous en êtes [PRENOM], et ainsi vous encourager efficacement par la suite!

Voici le lien vers les questions rapides : www…..com

Au plaisir d'échanger avec vous!

Michel Moreau Lapointe, responsable du projet Diabète en Forme

---

E-mail rappelant la mid-check up séance aux gens

OÙ SE SITUE VOTRE MOTIVATION PRÉSENTEMENT?

Bonjour [PRENOM]!

Nous effectuons un simple petit rappel avec vous, car l'outil que nous voulons vous offrir présentement ne sera disponible que cette semaine!

Suite à vos premières séances d'informations sur Diabète en Forme, nous sommes intéressés à connaître vos niveaux de motivation et d'activités physiques actuelS. En

cliquant sur le lien un peu plus bas, vous pourrez répondre à environ une dizaine de questions rapides qui nous permettront de savoir où vous en êtes [PRENOM], et ainsi vous encourager efficacement par la suite!

Voici le lien vers les questions rapides : www…..com

Au plaisir d'échanger avec vous!

Michel Moreau Lapointe, responsable du projet Diabète en Forme

---

E-mail annonçant la 4ième séance aux gens

VOTRE QUATRIÈME SÉANCE D'INFORMATIONS EST MAINTENANT DISPONIBLE

Bonjour [PRENOM]!

Bon début de semaine! J'espère que vous vous portez toujours bieS6.

Bonne nouvelle, si vous avez complété votre troisième séance d'informations, votre quatrième séance intitulée « Vos Forces et l'activité physique» est maintenant disponible pour vouS.Comme toujours, nous vous encourageons à réaliser celle-ci dans les meilleurs délais afin de progresser dans votre démarche reliée à la pratique d'activités physiqueS.

Pour accéder à votre deuxième séance, cliquez sur le lien suivant : www….com

Pour compléter votre troisième avant de passer à celle-ci, cliquez sur le lien suivant : www…com

La séance de cette semaine est une de mes séances préférées [PRENOM]! Amusez-vous!

Michel Moreau Lapointe, responsable du projet recherche

E-mail rappelant la 4ième séance aux gens

QUATRIÈME SÉANCE D'INFORMATIONS : VOS FORCES ET L'ACTIVITÉ PHYSIQUE

Bonjour [PRENOM]!

J'espère que vous allez bien! Nous serons déjà à la fin de la cinquième semaine du programme.

Nous voulions simplement vous encourager à compléter votre quatrième séance intitulée « Votre motivation et les bénéfices de l'activité physique » qui est

disponible pour vous sur le site internet. Nous vous encourageons à aller réaliser celle-ci afin d'avoir accès à votre cinquième séance la semaine prochaine!

Si vous n'avez toujours pas réalisé votre troisième séance, vous pouvez le faire en cliquant sur le lien un peu plus bas!

Pour accéder à votre séance, cliquez sur le lien suivant : www….com

Pour compléter votre première séance, cliquez sur le lien suivant : www…com

Ce sera toujours un plaisir [PRENOM],

Michel Moreau Lapointe, responsable du projet recherche

E-mail annonçant la 5ième séance aux gens

VOTRE CINQUIÈME SÉANCE D'INFORMATIONS EST MAINTENANT DISPONIBLE

Bonjour [PRENOM]!

Déjà bientôt la fin du programme. Plus que deux séances d'informations!

Bonne nouvelle, si vous avez complété votre quatrième séance d'informations, votre cinquième séance intitulée « Anticiper votre pratique d'activités physiques future » est maintenant disponible pour vouS.Comme à l'habitude, nous vous encourageons à aller réaliser celle-ci dans les meilleurs délais afin de progresser dans votre démarche [PRENOM]. Allez-y sans hésiter!

Pour accéder à votre cinquième séance, cliquez sur le lien suivant : www….com

Pour compléter votre quatrième séance, cliquez sur le lien suivant : www…com

Je vous souhaite une excellente séance.

Michel Moreau Lapointe, responsable du projet Diabète en Forme

---

E-mail rappelant la 5ième séance aux gens

CINQUIÈME SÉANCE D'INFORMATIONS : ANTICIPER VOTRE PRATIQUE D'ACTIVITÉS PHYSIQUES FUTURE

Bonjour [PRENOM]!

Nous serons déjà à la fin de la sixième semaine du programme Diabète en Forme.

Nous voulons simplement vous amener à compléter votre cinquième séance intitulée « Anticiper votre pratique d'activités physiques future » qui est présentement disponible pour vouS.Nous vous encourageons à aller réaliser celle-ci afin d'avancer et d'avoir accès à votre toute dernière séance la semaine prochaine!

Si vous n'avez toujours pas réalisé votre quatrième séance, vous pouvez le faire en cliquant sur le lien un peu plus bas!

Pour accéder à votre séance de cette semaine, cliquez sur le lien suivant : www….com

Pour compléter votre quatrième séance, cliquez sur le lien suivant : www…com

Nous vous encourageons à persévérer [PRENOM]!

Michel Moreau Lapointe, responsable du projet recherche

E-mail annonçant la 6ième séance aux gens

VOTRE SÉANCE FINALE EST MAINTENANT DISPONIBLE!

Bonjour [PRENOM]!

Bon début de semaine! Toutes mes sincères félicitations de vous être rendu(E) jusqu'ici aujourd'hui. Quel parcours!

Si vous avez complété toutes vos séances d'informations jusqu'à présent, votre toute dernière séance intitulée « Votre cheminement vers la réussite» est maintenant disponible pour vouS.Différente des autres, cette séance pourra vous permettre de bien mettre en évidence tout le chemin que vous avez parcouru durant les dernières semaines avec nous [PRENOM]. Cela dit, récompensez-vous en réalisant celle-ci dans les meilleurs délais!

Pour accéder à votre séance finale, cliquez sur le lien suivant : www….com

Pour finaliser vos séances préalables, cliquez sur le lien suivant : www…com

Nous espérons vraiment que vous constaterez tout le progrès que vous avez réalisé à travers cette expérience!

À votre santé [PRENOM],

Michel Moreau Lapointe, responsable du projet recherche

E-mail rappelant la 6ième séance aux gens

VOTRE SÉANCE FINALE EST MAINTENANT DISPONIBLE!

Bonjour [PRENOM]!

Bon début de semaine! Toutes mes sincères félicitations de vous être rendu(E) jusqu'ici aujourd'hui. Quel parcours!

Si vous avez complété toutes vos séances d'informations jusqu'à présent, votre toute dernière séance intitulée « Votre cheminement vers la réussite» est maintenant disponible pour vouS.Différente des autres, cette séance pourra vous permettre de bien mettre en évidence tout le chemin que vous avez parcouru durant les dernières semaines avec nous [PRENOM]. Cela dit, récompensez-vous en réalisant celle-ci dans les meilleurs délais!

Pour accéder à votre séance finale, cliquez sur le lien suivant : www….com

Pour finaliser vos séances préalables, cliquez sur le lien suivant : www…com

Nous espérons vraiment que vous constaterez tout le progrès que vous avez réalisé à travers cette expérience!

À votre santé [PRENOM],

Michel Moreau Lapointe, responsable du projet recherche

E-mail annonçant le final-check up séance aux gens

DERNIÈRE MISE À JOUR : OÙ SE SITUE MAINTENANT VOTRE NIVEAU D'ACTIVITÉS PHYSIQUES?

Bonjour [PRENOM]!

J'espère que vous allez bieS6.Voici un dernier petit outil pour vous en cette dernière semaine du programme!

Où se situent vos niveaux de motivation et d'activités physiques maintenant [PRENOM] ? En cliquant sur le lien un peu plus bas, vous pourrez répondre à environ une dizaine de questions rapides qui nous permettront de savoir où vous en êtes [PRENOM] comparativement au début du programme. Nous pourrons également vous encouragez efficacement pour une dernière fois par la suite!

Voici le lien vers les questions rapides : www…..com

Au plaisir d'échanger une dernière fois avec vous!

Michel Moreau Lapointe, responsable du projet Diabète en Forme

---

E-mail annonce follow-up

IMPORTANT : QUESTIONNAIRE DE FIN DE PROJET DIABÈTE EN FORME

Bonjour [PRENOM]!

J'espère que vous êtes sorti(e) grandi de l'expérience que vous avez vécue avec nouS.

Vous vous souvenez? Dans le cadre de l'étude Diabète en Forme, nous vous avions prévenu qu'un questionnaire serait à remplir suite au programme que vous avez suivi. Nous y voilà!

Nous apprécierions énormément votre participation à ce questionnaire, car la qualité de notre étude dépend grandement de vos réponses [PRENOM]. Le questionnaire ne prendra qu'une dizaine de minutes de votre tempS5.

Pour accéder et répondre à ce questionnaire, cliquez sur le lien suivant: www…..com

Merci énormément de prendre le temps de répondre, et ainsi améliorer les services futurs offerts aux personnes atteintes du diabète de type 2

Je vous souhaite une excellente continuité [PRENOM] ,

Michel Moreau Lapointe, responsable du projet Diabète en Forme

---

E-mail rappel follow-up

S'IL VOUS PLAÎT RÉPONDRE AU QUESTIONNAIRE DE FIN DE PROJET DIABÈTE EN FORME

Bonjour [PRENOM]!

J'espère que vous êtes sorti(e) grandi de l'expérience que vous avez vécu avec nouS.

Vous vous souvenez? Dans le cadre de l'étude Diabète en Forme, nous vous avions prévenu qu'un questionnaire serait à remplir suite au programme que vous avez suivi. Nous y voilà!

Nous apprécierions énormément votre participation à ce questionnaire, car la qualité de notre étude dépend grandement de vos réponses [PRENOM]. Le questionnaire ne prendra qu'une dizaine de minutes de votre tempS5.

Pour accéder et répondre à ce questionnaire, cliquez sur le lien suivant: www…..com

Merci énormément de prendre le temps de répondre, et ainsi améliorer les services futurs offerts aux personnes atteintes du diabète de type 2

Je vous souhaite une excellente continuité,

Michel Moreau Lapointe, responsable du projet Diabète en Forme

---

E-mail rappel 3 follow-up

IL EST IMPORTANT DE RÉPONDRE AU QUESTIONNAIRE DE FIN DE PROJET DIABÈTE EN FORME

Bonjour [PRENOM]!

J'espère que vous êtes sorti(e) grandi de l'expérience que vous avez vécu avec nouS.

Vous vous souvenez? Dans le cadre de l'étude Diabète en Forme, nous vous avions prévenu qu'un questionnaire serait à remplir suite au programme que vous avez suivi. Nous y voilà!

Nous apprécierions énormément votre participation à ce questionnaire, car la qualité de notre étude dépend grandement de vos réponses [PRENOM]. Le questionnaire ne prendra qu'une dizaine de minutes de votre tempS5.

Pour accéder et répondre à ce questionnaire, cliquez sur le lien suivant: www…..com

Merci énormément de prendre le temps de répondre, et ainsi améliorer les services futurs offerts aux personnes atteintes du diabète de type 2

Je vous souhaite une excellente continuité,

Michel Moreau Lapointe, responsable du projet Diabète en Forme

---

Email for recruitment

DIABÈTEENFORME **en collaboration avec** Diabète Québec

Monsieur/Madame,

Faire de l'activité physique régulièrement est maintenant fortement reconnu comme ayant de nombreux bénéfices sur la santé. C'est pourquoi, dans le cadre du programme Diabète en Forme, **nous voulons vous offrir l'opportunité de participer à une étude vous offrant un programme motivationnel personnalisé sur l'activité physique,** qui prendra en considération vos goûts et vos opinions afin de mieux interagir avec vouS.Ce programme personnalisé est disponible sur Internet. Vous pouvez donc participer à cette étude depuis votre résidence!

Nous pensons que ce programme Internet pourrait vous aider à intégrer la pratique d'activités physiques régulière dans votre vie de tous les jourS.**Cette étude débutera au mois de septembre et les personnes atteintes du diabète de type 2 sont invitées à y prendre part.**

Votre participation à cette étude est sur une base essentiellement volontaire. Cette étude, subventionnée par Diabète Québec et le Fonds de Recherche du Québec en Santé, est sous la direction du Dr François Boudreau de l'Université du Québec à Trois-RivièreS.Les résultats permettront d'obtenir des informations pertinentes et d'améliorer les programmes Internet faisant la promotion de l'activité physique auprès des adulteS.

Pour vous rendre sur le site Internet Diabète en Forme, cliquez sur le lien ci-dessous:

www.diabeteenforme.ca

Dans le cas où vous rencontriez des difficultés, nous vous invitons à communiquer avec le coordonnateur de l'étude à l'adresse suivante:michel.moreau.lapointe@uqtr.ca

Au nom de notre équipe Diabète en Forme, nous vous remercions à l'avance de votre précieuse collaboratioS6.

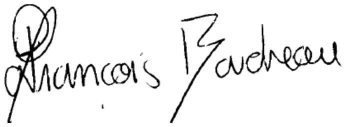

François Boudreau, PhD
Professeur
Université du Québec à Trois-Rivières

Messages for introduction videos

Animation - Diabète en Forme

Bonjour, je m'appelle Michel Moreau, je suis kinésiologue, spécialiste de l'activité physique et membre du groupe interdisciplinaire de recherche appliquée en santé de l'Université du Québec à Trois-RivièreS.Je suis également un des co-créateurs du projet Diabète en Forme et je suis ici pour vous le présenter aujourd'hui.

Animation - Diabète en Forme, un projet de recherche, un nouveau service

Diabète en Forme c'est à la fois un projet de recherche, mais aussi le début d'un nouveau service offert aux personnes atteintes du diabète pouvant être décrit comme un programme motivationnel internet de huit semaines qui peut vous accompagner de A à Z dans votre démarche relié à la pratique d'activités physiqueS.Le programme vous accompagnera de façon à travailler sur -votre motivation, votre confiance et la construction de plans d'action simples pour passer à l'action et ainsi devenir une personne active régulièrement, à votre façoS6.

Par-dessus tout, en fonction de vos caractéristiques personnelles, la plateforme vous donnera des rétroactions personnalisées sur les sujets les plus pertinents reliés à l'activité physique.

Animation - Pourquoi Diabète en Forme

Plusieurs bons services sont offerts actuellement aux personnes atteintes du diabète au Québec et ce qu'on veut faire avec Diabète en Forme c'est d'offrir un service de qualité qui va venir complémenter les services déjà en place. C'est donc pourquoi nous avons décidé de développer ce projet de recherche qui vous permettra d'expérimenter le service Diabète en Forme, en plus de nous aider à rendre ce service encore meilleur.

Au final, notre mission est donc de vous donner la capacité et la motivation d'atteindre un niveau d'activité physique optimal, et ainsi, bien gérer votre diabète et améliorer votre santé.

Animation – L'activité physique, vivre plus librement avec son diabète

Je n'pense pas faire de surprise à personne en disant que l'activité physique est très importante pour ce qui est de la prise en charge de son diabète. Ceux et celles qui pratiquent régulièrement des activités physiques sont très avantagés niveau santé et contrôlent beaucoup mieux leur diabète, et ce que je souhaiterais que les gens retiennent en regardant cette vidéo c'est simple. Que vous soyez motivé à faire plus d'activité physique ou alors pas du tout motivé pour le

moment. Soyez curieux et curieuses, lancez-vous dans l'aventure et venez sentir ce que Diabète en Forme peut accomplir avec vous, en équipe.

Alors allez-y, commencer votre inscription en cliquant sur « je veux m'inscrire » sous cette vidéo (en pointant le bouton) et au plaisir de vous voir participer au programme Diabète en Forme très bientôt!

Séance 1 :

Bonjour, je m'appelle Michel, kinésiologue spécialiste de l'activité physique, je vous souhaite personnellement la bienvenue sur votre plateforme Diabète en Forme que vous pourrez utiliser pour les 8 prochaines semaineS.J'aimerais aussi vous dire félicitations de vous être rendu jusqu'ici.

Maintenant, vous avez probablement déjà une bonne idée du contenu de la plateforme et de ses outilS.En naviguant quelque peu, vous trouverez facilement les nombreuses options qui s'offrent à vous, notamment vos séances d'informations personnalisées, vos plans d'action, l'outil vous permettant d'enregistrer vos activités physiques, ainsi que d'autres onglets vous permettant d'en savoir plus sur la pratique d'activités physiques et le projet Diabète en Forme.

Vous êtes maintenant sur le point de commencer votre première séance d'informations, bravo! Cette séance sera utile pour vous aider à visualiser où se situe votre niveau d'activité physique actuel en comparaison avec les recommandations de l'Association Canadienne du Diabète. Cette séance servira également à amorcer une discussion axée sur votre motivation face à votre pratique d'activités physiqueS.De plus, si vous vous sentez prêt, il vous sera aussi possible de planifier vos activités physiques pour les prochains jourS5.

Sans plus tarder, je vous souhaite une excellente séance et au plaisir de vous revoir bientôt!

Séance 2

Bonjour! C’est Michel de Diabète en Forme! Bienvenue à votre deuxième séance d’informations personnaliséeS.J’espère vraiment que vous aimez votre expérience sur la plateforme jusqu’à maintenant et que le programme vous aide positivement dans votre réflexion face à votre pratique d’activités physiqueS5.

J’espère également que vous sentez que notre approche vous aide à prendre vos propres décisions de manière plus confiante à l’égard de la pratique d’activités physiqueS. Nous croyons que vous êtes la personne la mieux placée pour prendre les décisions qui vous concernent et nous tenons à vous respecter, peu importe vos choix.

Maintenant, la séance d’aujourd’hui nous permettra de discuter ensemble à propos de votre attitude actuelle face à l’activité physique. Votre attitude est un des éléments les plus importants en ce qui a trait à votre motivation, c’est pourquoi il est important d’en discuter et de s’assurer qu’elle soit favorable face à l’activité physique. Ainsi, il vous sera plus facile de rester motivé par la suite!

Pour ce faire, nous tenterons d’approfondir quels sont les bénéfices santé reliés au fait d’être actif qui pourrait être importants pour vouS.Cette activité pourrait ainsi contribuer à vous donner un regard plus positif face à l’activité physique.

Sans plus tarder, allons voir où se situait votre attitude vis-à-vis l’activité physique à l’inscription! J’espère que vous aimerez votre séance, merci de nous faire confiance et d’évoluer avec nous!

À très bientôt!

Séance 3

Bonjour, c'est Michel de Diabète en Forme- ! Nous voilà déjà à votre troisième séance d'informations personnalisées! J'espère que vous allez bien et que vous avez toujours du plaisir à utiliser Diabète en Forme. Peut-être sentez-vous également une progression dans votre motivation et vos actions suite aux deux premières séances d'informationS.

Aujourd'hui, la séance servira à discuter ensemble de votre motivation face à l'activité physique. Concrètement, nous examinerons pour quelles raisons principales vous étiez motivé à pratiquer des activités physiques au moment de l'inscriptioS6.C'est essentiel de connaître précisément ce qui vous motive à bouger.

En fait, les études démontrent que les personnes qui éprouvent la plus grande satisfaction et la plus grande motivation à pratiquer des activités physiques sont celles qui sont également capables de faire des liens positifs entre le fait d'être actives et les choses qui sont importantes dans leur vie, comme leur santé ou le fait de vouloir vivre longtemps pour être présent pour leur famille. C'est donc pourquoi la séance d'aujourd'hui vous permettra de faire un court exercice en ce sens!

J'espère sincèrement que vous aimerez cette expérience. Alors sans plus tarder, je vous souhaite encore une fois une excellente séance!

Au plaisir de se revoir très bientôt!

Séance 4

Bonjour! C'est Michel de Diabète en Forme! Nous voilà déjà à la moitié de votre programme personnalisé! J'aimerais d'abord prendre le temps de vous féliciter personnellement pour vos avancementS.Bravo!

La séance d'aujourd'hui sera très simple, elle servira simplement à effectuer une mise à jour à propos de votre attitude à l'égard de l'activité physique, de votre motivation et de votre niveau d'activités physiques des dernières semaineS.Les réponses que vous fournirez dans cette séance seront comparées à vos réponses à l'inscriptioS6.Nous pourrons donc regarder ensemble votre évolution et nous serons ainsi en mesure de vous donner des rétroactions positives sur votre cheminement depuis le début de votre programme. Peu importe où vous en êtes maintenant, cette séance de mise au point peut être bénéfique pour vouS5.

Que votre attitude, votre motivation ou votre niveau d'activité physique ait changé ou non, nous vous encourageons à participer à cette séance et y répondre le plus sincèrement possible. Ainsi, il sera possible de regarder ensemble quelles seront les options possibles pour votre démarche future en ce qui concerne votre pratique d'activités physiqueS5.

Sans plus tarder, lancez-vous et encore une fois au plaisir de vous revoir très bientôt!

Séance 5

Bonjour, c'est Michel de Diabète en Forme! Vous êtes maintenant rendu à votre 5ième séance d'informations personnaliséeS.Comme toujours, j'espère que vous aimez votre expérience sur le programme Diabète en Forme.

Vous serez peut-être d'accord avec le fait qu'il est difficile de maintenir de bonnes habitudes en matière d'activités physiqueS.En effet, la pratique d'activités physiques demande un effort constant, faisant en sorte que certaines personnes ne sentent pas posséder la confiance nécessaire afin de réussir à maintenir un mode de vie régulièrement actif.

Il est vrai que bouger régulièrement demande un effort constant,- semaine après semaine. D'un autre côté, il existe des solutions pour trouver la force de réussir cet objectif. Si vous le voulez, nous tenterons une de ces solutions aujourd'hui! Nous explorerons ensemble vos forces et vos qualités, et nous tenterons de voir comment celles-ci pourraient vous faire voir qu'il est possible pour vous de pratiquer des activités physiques régulièrement. Il n'y a aucun doute pour nous que vous possédez tout ce qu'il faut, toutes les qualités nécessaires, pour devenir une personne régulièrement active.

Avant tout, voyons d'abord à quel point vous vous sentiez capable de pratiquer régulièrement des activités physiques au moment de l'inscriptioS6.

Lorsque vous le voulez, cliquer sur continuer pour commencer la séance!

À très bientôt!

Séance 6

Bonjour! C'est toujours Michel de Diabète en Forme. Je suis très content que vous soyez toujours avec nouS5.

Vous avez déjà traversé la majorité du programme en participant aux 5 premières séances d'informations précédenteS.Je vous félicite sincèrement. J'espère que cela vous a aidé à trouver la motivation de réussir à pratiquer régulièrement des activités physiques et que vous vous sentez capable de réussir ce beau défi. Nous croyons fortement que vous l'êteS5.

Maintenant, si vous le voulez, la séance d'aujourd'hui vous servira à visualiser votre pratique d'activités physiques future. Concrètement, la séance vous guidera à travers une série de questions simples à répondre, qui vous aideront à percevoir quelle serait, pour vous, la manière idéale et réaliste de pratiquer des activités physiques régulièrement dans votre quotidieS6.

Cette activité peut être très efficace pour vous visualiser en situation de réussite et vous imaginer comment vous vous sentiriez d'être régulièrement actif ou active au quotidieS6.

En effectuant cet exercice, cela pourrait donc encore une fois contribuer positivement à votre motivation, ainsi qu'à votre confiance et vous aider à voir plus clairement à quoi ressemblerait votre quotidien si vous étiez une personne active régulièrement.

J'espère que l'exercice vous plaira!

Bon succès et à très bientôt!

Séance 7

Bonjour, c’est toujours Michel de Diabète en Forme! c’est un plaisir de vous avoir pour une avant dernière fois avec nous aujourd’hui.

Comme vous le savez déjà sans doute, la séance d’aujourd’hui fera un résumé de toutes les séances que nous avons effectué ensemble. Cela vous permettra ainsi de prendre conscience de l’ensemble des facteurs positifs que vous avez évoqué au cours des dernières semaineS.Des facteurs qui pourraient vous aider à trouver toute la motivation, la force et la confiance nécessaire afin de devenir une personne active.

J’espère que cette séance vous permettra de bien voir tout le chemin que vous avez parcouru.

Et au plaisir de se revoir la semaine prochaine pour une dernière mise à jour.

Séance 8

Bonjour! C'est Michel de Diabète en Forme pour une dernière fois avec vous!

La séance d'aujourd'hui sera très simple, elle servira simplement à effectuer une dernière mise à jour à propos de votre confiance, de votre motivation et de votre niveau d'activités physiques des quatre dernières semaineS. Nous pourrons donc regarder ensemble votre évolution depuis le début du programme. Nous serons ainsi en mesure de vous donner des rétroactions positives sur votre cheminement complet peu importe où vous en êtes maintenant.

À la toute fin de cette séance, nous vous poserons également quelques courtes questions afin de connaître votre appréciation globale du programme, ce qui nous aidera grandement à améliorer cette plateforme.

Que votre confiance, votre motivation ou votre niveau d'activité physique ait changé ou non, nous vous encourageons à participer à cette séance et y répondre le plus sincèrement possible. Ainsi, il sera possible de regarder ensemble quelles seront les options possibles pour votre démarche future en ce qui concerne votre pratique d'activités physiqueS5.

J'aimerais aussi vous remercier personnellement d'avoir participer activement au programme, et je vous félicite sincèrement pour tout votre parcours sur Diabète en Forme.

Bonne dernière séance et bonne continuité!

TEXTES DES TÉMOIGNAGES DE LA SÉANCE 6

Témoignage 1

Bonjour je m'appelle Céline, j'ai 55 ans

J'ai appris que j'avais le Diabète il y a environ 3 anS.L'activité physique a jamais vraiment fait partie de ma vie sauf depuis 1 an et demi enviroS6.

Au début, quand mon médecin me disait qu'il fallait que je fasse des activités physiques régulièrement, j'étais vraiment pas intéressée. . –Je voyais aucun moyen de prendre 150 minutes de mon temps pour aller bouger, en plus de mon travail, de ma famille et mes autres activitéS.Sans dire que je ne pensais pas que c'était vraiment important pour moi.

Animation-transition (comment en es-tu arrivé à devenir active régulièrement)

Un peu comme vous faites sur Diabète en Forme, c'est à force de discuter avec mon médecin et de m'informer moi-même que j'ai pris conscience des raisons qui me pousseraient à changer. L'activité physique, c'est essentiel si je veux vivre le plus longtemps possible, en santé.

C'est dans ma nature aussi d'être forte et de relever les défis qui se présentent à moi, je veux être un exemple pour ceux que j'aime et je veux surtout pouvoir être là quand ils seront dans le besoiS6.

Ça a donc été ça pour moi les éléments déclencheurs, que je comprenne que l'activité physique, c'est un facteur clé si je veux vivre longtemps, et si je veux être là pour mes procheS.Pu personne n'a besoin de me tordre le bras pour que j'aille bouger et que je fasse des activités physiques-. les efforts se font naturellement parce que je sais pourquoi, profondément, il faut que je sois active.

Animation – transition (qu'est-ce que tu fais comme activité physique maintenant)

Je fais au moins 150 minutes d'activités physiques par semaine depuis bientôt 1 aS6.Je vais marcher, je fais du vélo et je nage. Ça vraiment pas été facile de prendre l'habitude au début, mais comme je disais pour moi c'était claire, il fallait que je me prenne en main, c'est ça que je voulais devenir. Et aujourd'hui, si vous saviez à quel point je ne pourrais pas m'en passer, je me suis jamais senti aussi bien, aussi énergique et aussi motivé dans tout ce que je faiS.Ah et je contrôle mieux mon poids aussi ça c'est sûr.

Et j'avais toutes sorte de raisons de me faire croire que je pouvais pas y arriver, j'aurais pu en sortir des milliers…jusqu'à temps que je me dise : là, c'est assez julie!. Et si moi j'y suis arrivé, tout le monde peut y arriver, vraiment. C'est cliché mais c'est ça! Je le pense sincèrement.

Animation – transition Question : Comment tu te sens maintenant?

Pour vrai, à voir aujourd'hui l'effet que ça a sur moi, la seule chose que je peux dire, c'est que je suis fière de moi, et heureuse! (termine avec un sourire sincère)

Témoignage 2

Je m'appelle Charles, j'ai 45 ans

Je sais que j'ai le diabète depuis 10 anS.J'ai joué longtemps au tennis 1 fois par semaine avec un collègue, j'ai fait du sport jeune, mais à part ça j'ai pas fait grand-chose comme activité physique par le passé.

Ça a vraiment été dur de trouver la motivation, mais je ne m'étais jamais informé sérieusement sur l'activité physique. J'entendais tout le monde dire que c'est dont bon pour la santé faire des activités physiques, mais c'est comme si je me disais toujours que le message s'adressait pas à moi. J'avais peut-être pas confiance que j'étais capable de devenir actif et c'était pour ça que j'écoutais pas le message. Mon médecin, mon frère, ma mère, tout le monde me disait de bouger pluS.Je le faisais paS5.

Animation-transition Question : Ce qui t'as fait changé?

Ma santé se détériorait, j'avais des infections aux pieds…ça vraiment été là où je me suis dis, c'est assez là je me prends en maiS6.En plus, je n'avais pas d'énergie, je manquais de motivation pour des choses que normalement j'aime. Il fallait que je fasse quelque chose pour redevenir moi-même, être la personne que je veux! C'était le temps que je fasse de quoi pour ma santé, c'était vraiment le tempS5.

Animation-transition qu'est-ce que tu fais aujourd'hui

Depuis 2 ans, je pratique des activités physiques régulièrement, je marche 30 minutes à 1 heure à chaque jour et des fois je vais nager quand j'ai quelqu'un avec qui y aller, mais j'ai pas souvent de partenaire alors j'fais plus marcher et je cours un peu dans mes marches (rire). J'ai fait un peu de spinning (vélo stationnaire) en groupe c'était le fun aussi, c'est simple pi tu vas à ton rythme.

Animation-transition Question : Comment tu te sens aujourd'hui?

Sérieusement, je ne peux pas remercier assez la vie de m'avoir donné le courage d'entreprendre ce changement-là. Je me sens tellement bien, je suis énergique, j'ai le goût de faire plein de choses, et je les fais! Les semaines où je fais moins d'activités physiques, je le sens tout suite, je me sens tellement mieux quand je bouge, c'est rendu moi de bouger.

Je peux pas me sentir mieux, de où je suis parti, et de voir où je suis rendu, je suis extrêmement fier de moi.

Messages implemented by AlphaZero

Page de login – période d'inscription: 11 août au 29 août

Page de login – première semaine du programme: 30 août au 6 septembre

Page de login – reste du programme: 7 septembre et plus

PAGE DE LOGIN – INSCRIPTION (11 août au 29 août inclusivement)
À gauche de la page

Diabète en Forme est un programme qui vous aide à intégrer la pratique régulière d'activités physiques dans votre vie, avec vouS.

Voir où votre niveau d'activités physiques actuel se situe

Développer votre confiance et votre motivation au quotidien

Planifier vos activités physiques et devenir une personne régulièrement active, à votre façoS6.

Le programme débute dans (Nombre de jours restants jusqu’au 31 août)

À droite de la page
vidéo et bouton d’inscription

PAGE DE LOGIN –DÉBUT PROGRAMME (30 août au 6 septembre)

À gauche de la page

Diabète en Forme est un programme qui vous aide à intégrer la pratique régulière d'activités physiques dans votre vie, avec vouS.

Voir où votre niveau d'activités physiques actuel se situe

Développer votre confiance et votre motivation au quotidien

Planifier vos activités physiques et devenir une personne régulièrement active, à votre façoS6.

Le programme est disponible dès maintenant

À droite de la page dans un encadré

La période d'inscription est maintenant terminée! Si vous avez manqué de temps pour vous inscrire avant le 1er septembre, vous ne pourrez pas participer à Diabète en Forme.

Nous vous remercions sincèrement de votre intérêt à l'égard de notre projet! Pour toute question, vous pouvez communiquer à l'adresse suivante: michel.moreau.lapointe@uqtr.ca
L'équipe de Diabète en Forme

PAGE DE LOGIN –DÉBUT PROGRAMME (7 septembre et plus)

À gauche de la page

Diabète en Forme est un programme qui vous aide à intégrer la pratique régulière d'activités physiques dans votre vie, avec vouS.

Voir où votre niveau d'activités physiques actuel se situe

Développer votre confiance et votre motivation au quotidien

Planifier vos activités physiques et devenir une personne régulièrement active, à votre façoS6.

À droite de la page

" Pratiquer des activités physiques régulièrement signifie pratiquer un minimum de 150 minutes d'activités physiques d'intensité modérée par semaine "   - L'Association Canadienne du Diabète

PAGE DE LOGIN – DÉCONNEXION

À gauche de la page

Diabète en Forme est un programme qui vous aide à intégrer la pratique régulière d'activités physiques dans votre vie, avec vouS.

Voir où votre niveau d'activités physiques actuel se situe

Développer votre confiance et votre motivation au quotidien

Planifier vos activités physiques et devenir une personne régulièrement active, à votre façoS6.

À droite de la page

Au plaisir de vous revoir.

- L'équipe de Diabète en Forme

ONGLET “Je veux m’inscrire” - CRÉATION IDENTIFIANT ET MOT DE PASSE

Titre: Créez vous un compte facilement

Afin que nous puissions vous reconnaître tout au long du projet, créez vous rapidement un compte en remplissant les informations demandées ci-dessouS.En créant votre compte, vous démarrerez ainsi le processus d'inscriptioS6.

(INPUTS: courriel, mot de passe)

Vous recevrez un courriel de confirmation une fois votre compte créé.

ONGLET - EN SAVOIR PLUS

TITRE : Diabète en Forme: Un projet de recherche qui veut rendre service aux personnes atteintes du diabète de type 2, avec vouS5.

Diabète en Forme est avant tout un programme de huit semaines qui peut vous accompagner dans votre démarche en vue de pratiquer des activités physiques plus régulièrement. Trouver la motivation et les moyens de pratiquer des activités physiques régulièrement n'est pas toujours facile, et c'est pourquoi Diabète en Forme veut vous aider en vous offrant:

Des séances d'informations personnalisées et interactives visant plusieurs sujets qui pourront vous aider à bâtir votre motivatioS6.

Un outil qui vous accompagne dans la planification de vos activités physiques

Un outil qui vous permettra d'enregistrer les activités physiques que vous effectuez afin de visualiser où vous en êteS5.

D'autres éléments d'informations qui pourraient répondre à vos inquiétudes vis-à-vis la pratique d'activités physiqueS5.

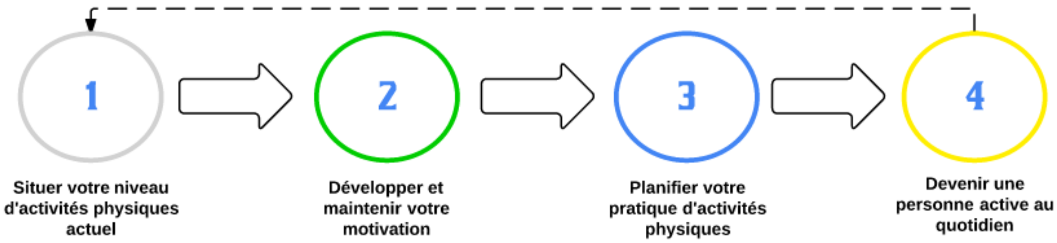

Diabète en Forme se veut aussi être un projet de recherche dirigé par le professeur-chercheur François Boudreau, Ph.D, de l'Université du Québec à Trois-RivièreS.L'objectif de ce projet est d'évaluer l'efficacité de programmes Internet visant la promotion de la pratique d'activités physiques chez les personnes atteintes du diabète de type 2. Avec votre participation, vous pourriez, en plus de recevoir un service vous aidant dans votre démarche vis-à-vis la pratique d'activités physiques, contribuer à l'amélioration des services offerts à l'ensemble des personnes atteintes de cette maladie dans la province du Québec.

Pour en connaître encore davantage sur le projet, cliquez sur le bouton "Je veux m'inscrire" et ainsi amorcer votre inscriptioS6.Cela ne vous engage à rien et vous pourrez toujours décider de ne plus participer.

ONGLET – MOT DE PASSE OUBLIÉ

Titre: Vous avez oublié votre mot de passe? Aucun souci.

Entrez simplement votre adresse courriel ci-dessous et nous vous renverrons votre mot de passe à cette adresse.

ONGLET – NOUS CONTACTER

TITRE: Nous voulons connaître votre avis

Vous avez des questionnements ou des commentaires à propos du projet Diabète en Forme? Nous vous encourageons à nous les partager en écrivant votre message ci-dessous ou à l'adresse suivante : michel.moreau.lapointe@uqtr.ca . En nous partageant vos questionnements ou vos commentaires, vous nous aider grandement à améliorer la plateforme afin de la rendre la plus optimale possible. Merci d’avance de nous aider.

ONGLET – NOUS CONTACTER (MESSAGE ENVOYÉ)

MERCI!

Nous vous répondrons dans les meilleurs délais et nous vous remercions pour votre message.

ONGLET - FOIRE AUX QUESTIONS

Titre: À propos de la pratique d'activités physiques

Voici des questions fréquemment demandées par des personnes atteintes du diabète. Cliquez simplement sur un sujet qui vous intéresse pour en savoir davantage.

1. Quelles sont les recommandations de l'Association Canadienne du Diabète en terme d'activités physiques?

L'Association Canadienne du Diabète recommande de pratiquer au moins 150 minutes d'activités physiques d'intensité modérée par semaine. Une activité physique effectuée à une intensité modérée signifie que votre fréquence cardiaque s'est élevée lors de l'effort et que vous vous êtes senti(e) essouflé(e). De plus, pour qu'une activité physique compte dans votre cumul de minutes, elle doit être effectuée sur une période d'au moins 10 minutes consécutives .

Il est également recommandé d'effectuer vos 150 minutes d'activités physiques d'intensité modérée sur au moins trois jours, comme le lundi (50 minutes), mercredi (60 minutes) et vendredi (40 minutes) par exemple.

2. Quels sont les signes que je pratique une activité physique d'intensité modérée?

Une activité physique effectuée à une intensité modérée signifie que votre fréquence cardiaque s'est élevée lors de l'effort et que vous vous êtes senti(e) essouflé(e). De plus, pour qu'une activité physique compte dans votre cumul de minutes par semaine, elle doit être effectuée sur une période d'au moins 10 minutes consécutives . Lors d'une telle activité, vous pouvez parler, mais pas chanter. Des activités physiques populaires d'une intensité modérée sont la marche rapide, le jogging lent, la natation, la randonnée, la raquette, le ski de fond et le vélo.

3. Est-ce recommandé que je pratique des activités physiques d'intensité élevée?

Une activité physique d'intensité élevée représente une activité où vous sentez un essouflement très important et une hausse importante de votre fréquence cardiaque. Si vous n'avez jamais

vraiment fait d'activités physiques dans le passé, ou alors vous n'avez pas effectué d'activités physiques depuis un certain temps, nous vous proposons de viser des activités physiques d'intensité modérée. Avant de débuter des activités physiques d'intensité élevée, nous vous recommandons fortement d'entreprendre une discussion à ce sujet avec votre professionnel de la santé afin qu'il vous conseille et vous donne le feu vert.

4. Est-ce que mes activités physiques d'intensité légère contribuent à atteindre les 150 minutes d'activités physiques d'intensité modérée recommandées par l'Association Canadienne du Diabète?

Une activité physique d'intensité légère correspond à une activité où vous ne sentez pas vraiment d'essoufflement ou d'élévation de votre fréquence cardiaque, mais où vous êtes quand même en mouvement. Les activités physiques qui apportent les bénéfices optimaux pour votre santé sont les activités physiques d'intensité modérée ou de plus haute intensité. Malheureusement, nous ne tenons donc pas compte du nombre de minutes d'activités physiques légères dans votre cumul de minutes par semaine. Par contre, certaines activités physiques légères, comme la marche à un rythme normal, peuvent être transformées en activités physiques d'intensité modérée en augmentant tout simplement l'effort d'un brin, soit en effectuant une marche à un rythme plus soutenu par exemple.

5. Suis-je trop âgé(e) pour commencer à pratiquer des activités physiques régulièrement?

Il n'y a pas d'âge pour commencer à pratiquer des activités physiques plus régulièrement. Plusieurs personnes décident de devenir actives dans la cinquantaine ou la soixantaine. Les bénéfices que vous retirerez grâce à un mode de vie actif sont de loin plus grands que les risques possibleS.De plus, nul besoin de viser 150 minutes d'activités physiques à intensité modérée par semaine au départ. Allez-y progressivement et tentez d'augmenter votre nombre de minutes par semaine lorsque vous vous sentez en confiance. Pour connaître les détails qui vous concernent plus personnellement avant de commencer à bouger, vous pouvez discuter avec votre professionnel de la santé qui saura vous guider, vous mettre en confiance et vous donner le feu vert.

6. D'autres questionnements?

Vous avez d'autres questionnements concernant la pratique d'activités physiques? Nous vous encourageons à nous contacter en cliquant ici: Nous contacter.

TITRE 2: À propos du projet Diabète en Forme

Voici des questions fréquemment demandées concernant le projet Diabète

en Forme. Cliquez simplement sur un sujet qui vous intéresse pour en savoir davantage.

1- Qu'est-ce que le site Internet Diabète en Forme ?

Le site Internet Diabète en Forme a pour objectif de faire la promotion de l'activité physique auprès des adultes atteints du diabète de type 2, en vous aidant à développer votre motivation et planifier votre pratique d'activités physiqueS.Il s'agit donc principalement d'un programme motivationnel et de planification, qui vous aide dans votre démarche vis-à-vis la pratique d'activités physiques en vous offrant plusieurs outils utiles en vue de pratiquer des activités physiques plus régulièrement.

2- Qui a développé le programme PURE-Cybersanté-Activité Physique ?

Ce programme a été développé par :

Chercheur responsable du projet :

François Boudreau, Ph.D., Professeur, Université du Québec à Trois-RivièreS5.

Co-chercheurs :

Dr Paul Poirier, Cardiologue, Institut universitaire de cardiologie et de pneumologie de

Québec;

Dre José Côté, PhD, Professeure, Université de Montréal.

3- Est-ce que ce programme est sous la responsabilité d'un organisme subventionnaire ?

Oui, ce programme a reçu un financement des Fonds de Recherche du Québec en Santé.

4- De quelle façon mes données personnelles seront protégées :

Durant votre participation à ce projet, le chercheur responsable ainsi que son personnel recueilleront et consigneront dans un dossier de recherche les renseignements vous concernant. Seuls les renseignements nécessaires pour répondre aux objectifs scientifiques de ce projet seront recueilliS5.

Comme vous l'avez déjà remarqué, de façon à vous transmettre des informations personnalisées portant sur l'activité physique, nous vous demandons de répondre à certaines questions depuis votre ordinateur; par exemple, votre prénom, votre âge, votre sexe, votre statut civil, votre niveau de scolarité et votre origine ethnique. En

participant à cette étude, vous acceptez que vos renseignements personnels soient transmis « de votre ordinateur à un serveur sécurisé ». Le serveur sécurisé utilise un protocole HTTPS, c'est un peu comme un fourgon blindé: il vous assure la sécurité du transport (vos renseignements personnels). De plus, vos renseignements personnels recueillis sur le serveur seront stockés séparément de votre « nom d'utilisateur ». De cette manière, l'anonymat est garanti, vos renseignements ne pourront jamais être associés à un élément permettant de vous identifier.

5. Ai-je le droit de quitter le projet de recherche ?

Votre participation à ce projet de recherche est volontaire. Vous êtes donc libre de refuser d'y participer. Vous pouvez également vous retirer de ce projet à n'importe quel moment, sans avoir à donner de raisons, en faisant connaître votre décision au coordonnateur du projet à l'adresse suivante: michel.moreau.lapointe@uqtr.ca .

Votre décision de ne pas participer à ce projet de recherche ou de vous en retirer n'aura aucune conséquence sur la qualité des soins et des services auxquels vous avez droit ou sur votre relation avec le chercheur responsable du projet et les autres intervenantS5.

6. D'autres questionnements?

Vous avez d'autres questionnements concernant le projet Diabète en Forme? Nous vous encourageons à nous contacter en cliquant ici: Nous contacter.

ONGLET – OUTIL DE MESURE D'ACTIVITÉS PHYSIQUES

TITRE: Enregistrez chaque activité physique que vous effectuez

Enregistrer vos activités physiques au fil des semaines peut vous aider à mieux connaître où se situe votre niveau d'activités physiques ainsi qu'à constater votre progression depuis le début du programme. La bonne habitude d'enregister vos activités peut donc contribuer positivement à votre motivation et vous donner un repère afin de vous assurer d'atteindre vos objectifS5.

REPÈRES

Activités physiques légères

Indice: Effort Minimal

Exemples: Marche, Quilles et Curling

Activités physiques modérées

Indice: Votre fréquence cardiaque s'élève et vous sentez un essoufflement

Exemples: Marche rapide, Jogging lent, Vélo, Natation, Tennis, Badminton et autres

Activités physiques intenses

Indice: Votre fréquence cardiaque est très élevée et vous sentez un essoufflement important

Exemples: Course à pied, Vélo intensif, Natation intensive, Ski de fond ou autres

ONGLET - CONSEILS SÉCURITÉ

TITRE: Quelques conseils pour pratiquer des activités physiques en toute sécurité

Vous commencez à pratiquer des activités physiques un peu plus régulièrement? Voici quelques sujets qui pourraient vous intéresser afin de pratiquer vos activités physiques en toute sécurité. Comme chaque situation est unique, sachez également que le meilleur moyen de vous assurer d'une pratique sécuritaire est d'avoir une discussion avec votre professionnel de la santé à ce sujet.

Cliquez simplement sur un sujet qui vous intéresse pour en savoir davantage

1. Conseils de sécurité généraux pour la pratique d'activités physiques

Il peut être bon de toujours garder les éléments suivants à l’esprit afin d’éviter des complications inutileS.Ces quelques conseils généraux font éviter généralement la majorité des problèmes et blessures lorsqu’ils sont réalisés à chaque activité :

1. Faites toujours vos exercices de réchauffement et de récupératioS6.À chaque activité physique d’intensité modérée ou plus élevée que vous pratiquez, penser à commencer graduellement et à terminer graduellement. Au début de votre activité, augmenter graduellement votre intensité afin de passer d’une intensité faible à modérée. À la fin de votre activité, vous pouvez faire l’inverse et passer ainsi d’une intensité modérée à une intensité faible. Voyez le graphique ci-dessous pour vous aider à visualiser le tout.

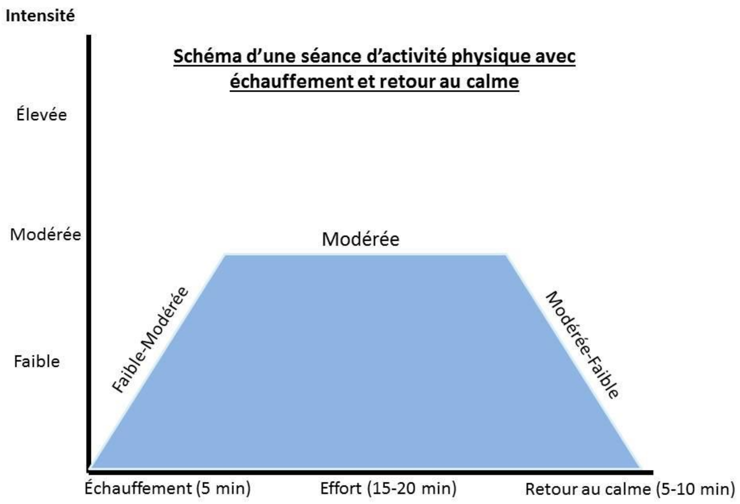

2. Écoutez votre corps et prenez des pauses au besoiS6. Il faut savoir contrôler son orgueil lorsqu'on pratique des activités physiqueS.Si vous sentez des symptômes inhabituels survenir lors de vos activités, n'attendez pas plus longtemps et prenez une pause ou alors arrêtez complètement votre activité pour la journée. Voici quelques symptômes inhabituels que vous devriez surveiller : les étourdissements, les maux de tête, la confusion ou la nausée.

Si vous sentez simplement que vous êtes essouflé(e), que votre fréquence cardiaque s'élève ou alors que vos muscles travaillent, vous êtes sur la bonne voie et vous pouvez continuer!

3. Buvez de l'eau avant, pendant et après l'activité physique. La déshydratation peut être cause de plusieurs blessures ou de malaise lors d'activités physiqueS.Pendant l'entraînement, visez 300 ml d'eau par 30 minutes d'effort. Si votre urine semble jaunâtre avant ou après l'entraînement, pensez à vous hydrater en visant jusqu'à un litre d'eau dépendamment de la couleur de votre urine.

4. Portez votre bracelet d'identification MedicAlert. MedicAlert vous connecte à l'identification médicale d'urgence et au réseau d'information médicale les plus fiableS.Bien que cela ne devrait pas arriver, la ligne d'urgence 24 heures de MedicAlert assure que votre information médicale critique soit accessible aux ambulanciers en 5 secondes seulement.

2. Vous prenez de l'insuline ou des médicaments antidiabétiques

Prenez-vous de l'insuline ou des médicaments antidiabétiques ? Il peut être important de surveiller votre taux de glycémie lorsque vous pratiquez une activité physique. Voici quelques points à avoir en tête. Ces points sont d'autant plus importants si vous commencez à faire de Conse

Le tableau vous propose des balises sur la glycémie (taux de sucre dans le sang) et des conseils de sécurité à suivre quand vous faites de l'activité physique. Note : Il pourrait être important de parler de ce tableau avec votre professionnel de la santé qui s'occupe de votre diabète pour vous assurer qu'il correspond bien à votre situatioS6.

**Avant l'activité physique**	**Si votre niveau de glycémie est en bas de <u>5,6 mmol/L</u>:**<br><br>**Il est peut-être trop bas pour faire de l'activité physique en ce moment. Avant de commencer votre activité, visez donc une collation contenant 15 grammes de glucides avec un fruit ou une tranche de pain par exemple.**
**Pendant l'activité physique**	Vérifiez votre glycémie toutes les 30 minuteS5.<br><br>Surveillez les signes de baisse de glycémie, comme les étourdissements, les maux de tête ou la confusioS6.<br><br>**Si votre glycémie est basse (moins de 4,0 mmol/L) :**<br><br>Arrêtez et reposez-vous<br>Prenez une collation (15 grammes de glucides à action rapide, comme un comprimé de glucose ou un bonbon dur contenant du sucre)<br>Vérifiez ensuite votre glycémie 15 minutes plus tard

	**Vous pourrez alors continuer votre activité quand votre glycémie a retrouvé un niveau sécuritaire**
**Après l'activité physique**	Vérifiez votre glycémie dès l'arrêt de votre activité.<br><br>Il est également bon de vérifier de nouveau 2-3 fois pendant les deux heures qui suivent.<br><br>**Si votre glycémie est basse (en bas de 4.0 mmol/L) :**<br><br>Prenez une collation (15 grammes de glucides à action rapide, comme un comprimé de glucose ou un bonbon dur contenant du sucre)<br>Vérifiez ensuite votre glycémie 15 minutes plus tard

3. Conseils pour tous à l'égard de votre glycémie (taux de sucre dans le sang) lors de la pratique d'activités physiques

Ces conseils généraux concernant votre glycémie (taux de sucre dans le sang) lorsque vous prévoyez faire des activités physiques pourraient s'avérer très bénéfiques afin d'éviter des événements désagréableS.Voici certains éléments à considérer.

Faire votre activité 1-2 heures après avoir mangé est préférable. Suite à un repas, votre glycémie s'élève et vous rend plus vulnérable à certains malaises lors d'activités physiqueS.Attendre 1 à 2 heures suite à votre repas permet à votre glycémie de se stabiliser.

Il est mieux d'éviter de faire des activités physiques immédiatement après avoir pris de l'insuline.

Il est également mieux d'éviter de faire des activités trop tard le soir, pour empêcher votre glycémie de baisser pendant votre sommeil.

Posséder toujours sur vous une forme de glucide à action rapide. Par exemple, apportez des comprimés de glucose ou des petits bonbons durs contenant du sucre.

Parler avec votre professionnel de la santé des façons de prévenir l'hypoglycémie avec votre insuline et votre diète.

4. Vos pieds et la pratique d'activités physiques

Il peut être important de prendre soin de vos pieds si vous êtes atteint(e) du diabète. Nous vous recommandons d'avoir une conversation avec votre professionnel de la santé si vos pieds courent un risque ou s'ils pourraient vous causer des problèmes quand vous faites de l'activité physique. Vous préférerez peut-être faire des activités physiques qui ne vous forcent pas à rester debout trop longtempS.Ces activités pourront vous aider à réduire la pression sur vos piedS.En voici des exemples :

activités aquatiques : aquagym, aquajogging, natation

bicyclette (à l'intérieur ou à l'extérieur) ou vélo pour bras

rame

exercices assis

De plus, voici quelques conseils additionnels pour garder vos pieds en forme :

Ne portez que des souliers qui vous vont parfaitement.

Vérifiez chaque jour si vos pieds ont des plaies ou des blessureS.S’il faut les laisser guérir, reportez vos activités physiques au besoin ou essayez certaines des activités dans la liste ci-dessuS5.

Faites toujours examiner immédiatement toute blessure aux piedS5.

Après une certaine période d’activité, assurez-vous de prendre une pause pour vérifier vos piedS5.

5. Les médicaments pour pression artérielle et la pratique d'activités physiques

Prenez-vous des médicaments pour la pression artérielle? Vous pouvez vérifier auprès de votre professionnel de la santé pour en être certain(e). Vous pouvez tout de même faire de l’activité physique avec ces médicaments, mais vous devez prendre quelques précautions pour assurer votre sécurité. Deux éléments principaux sont à surveiller:

Assurez-vous de vous rafraîchir. Avec vos médicaments, votre corps pourrait avoir plus de difficulté à se refroidir quand vous êtes actif ou active. Gardez votre corps plus frais en :

Buvant beaucoup d’eau pendant votre activité

Faisant vos activités le matin ou le soir, soit quand le temps est plus frais

Diminuant l’intensité de votre activité ou sa durée quand la température ou l’endroit où vous faites votre activité est chaud ou humide.

Vos médicaments peuvent affecter votre glycémie et vous pourriez vous sentir un peu faible. Si vous vous sentez faible ou étourdi :

Arrêtez l'activité ou ralentissez

Prenez un comprimé de glucose ou mangez un bonbon dur

Attendez de bien vous sentir avant de recommencer votre activité.

6. D'autres questionnements?

Vous avez d'autres questionnements concernant la sécurité lors de la pratique d'activités physiques? Nous vous encourageons à nous contacter en cliquant ici: Nous contacter.
