# Supplementary material for: Development of a Fully Automated, Web-Based, Tailored Intervention Promoting Regular Physical Activity Among Insufficiently Active Adults With Type 2 Diabetes: Integrating the I-Change Model, Self-Determination Theory, and Motivational Interviewing Components
Source: JMIR Res Protoc. 2015 Feb 17;4(1):e25. doi: 10.2196/resprot.4099 (PMC4376153; doi:10.2196/resprot.4099)
Supplement: Supplementary file 5 [file resprot_v4i1e25_app5.pdf]

TailorBuilder code and CSS style sheets of the *Diabète en Forme* web-based tailored intervention

8th of April 2014

Non-official document

It is not for readers to understand the code written for the intervention.

The purpose of this document is only to give an overview of all the code to be written with the software TailorBuilder to properly build an intervention similar to the DEF tailored intervention.

For questions:

michel.moreau-lapointe.1@ulaval.ca

UNIVERSITÉ LAVAL

UQTR Université du Québec à Trois-Rivières

SECTIONS

MAIN MENU – END FORMULAS 4
MAIN MENU – ADVICE FORMULAS 228
MENU OF THE MOTIVATIONAL SESSIONS 232
MENU OF THE ACTION PLAN TOOL 236
REGISTRATION SESSION – END FORMULAS 237
REGISTRATION SESSION– ADVICE AND ROUTING FORMULAS 245
TAILORED MOTIVATIONAL SESSION 1 – ADVICE FORMULAS 249
TAILORED MOTIVATIONAL SESSION 2– ADVICE FORMULAS 256
TAILORED MOTIVATIONAL SESSION 3– ADVICE FORMULAS 260
TAILORED MOTIVATIONAL SESSION 4– ADVICE FORMULAS 262
TAILORED MOTIVATIONAL SESSION 5– ADVICE FORMULAS 264
TAILORED MOTIVATIONAL SESSION 6–ADVICE FORMULAS 265
TAILORED MOTIVATIONAL SESSION 7– ADVICE FORMULAS 266
TAILORED MOTIVATIONAL SESSION 8– ADVICE FORMULAS 267
ACTION PLAN WEEK 1 – ADVICE FORMULAS 269
ACTION PLAN WEEK 2–ADVICE FORMULAS 271
ACTION PLAN WEEK 3–ADVICE FORMULAS 273
ACTION PLAN WEEK 4–ADVICE FORMULAS 274
ACTION PLAN WEEK 5–ADVICE FORMULAS 276
ACTION PLAN WEEK 6–ADVICE FORMULAS 277
ACTION PLAN WEEK 7–ADVICE FORMULAS 278
ACTION PLAN WEEK 8–ADVICE FORMULAS 280
STYLESHEET - MENU OF THE ACTION PLAN TOOL 282
STYLE SHEET - MOTIVATIONAL SESSIONS, TUTORIAL and APPRECIATION 288
STYLESHEET - ACTION PLANS AND REGISTRATION SESSION 330
STYLESHEET - MOTIVATIONAL SESSIONS MENU 363
STYLESHEET - MAIN MENU 370

MAIN MENU – END FORMULAS

[TBVAR_DATEFIRSTVISIT = '10-09-2014'];
[ACCOUNT:EMAIL = TB_ACCOUNT];
[ACCOUNT:NAME = PRENOM];
[DATE_DEBUT = '10-09-2014'];
[DATE_OFF = TBVAR_DATENOW - DATE_DEBUT];
**codes for creating a variable-text creating 'monday' to 'saturday' **
[DATE_REFPOINT = '04-05-2014'];
[DATE_DIFFERENCE = TBVAR_DATENOW - DATE_REFPOINT];
IF (((TBVAR_TIMENOW GE 600) AND ((DATE_DIFFERENCE EQ 120) OR (DATE_DIFFERENCE EQ 127) OR (DATE_DIFFERENCE EQ 134) OR (DATE_DIFFERENCE EQ 141) OR (DATE_DIFFERENCE EQ 148) OR (DATE_DIFFERENCE EQ 155) OR (DATE_DIFFERENCE EQ 162) OR (DATE_DIFFERENCE EQ 169) OR (DATE_DIFFERENCE EQ 176) OR (DATE_DIFFERENCE EQ 183) OR (DATE_DIFFERENCE EQ 190) OR (DATE_DIFFERENCE EQ 197) OR (DATE_DIFFERENCE EQ 204) OR (DATE_DIFFERENCE EQ 211) OR (DATE_DIFFERENCE EQ 218) OR (DATE_DIFFERENCE EQ 225) OR (DATE_DIFFERENCE EQ 232) OR (DATE_DIFFERENCE EQ 239) OR (DATE_DIFFERENCE EQ 246) OR (DATE_DIFFERENCE EQ 253) OR (DATE_DIFFERENCE EQ 260) OR (DATE_DIFFERENCE EQ 267) OR (DATE_DIFFERENCE EQ 274) OR (DATE_DIFFERENCE EQ 281) OR (DATE_DIFFERENCE EQ 288) OR (DATE_DIFFERENCE EQ 295) OR (DATE_DIFFERENCE EQ 302) OR (DATE_DIFFERENCE EQ 309) OR (DATE_DIFFERENCE EQ 316) OR (DATE_DIFFERENCE EQ 323) OR (DATE_DIFFERENCE EQ 330) OR (DATE_DIFFERENCE EQ 337) OR (DATE_DIFFERENCE EQ 344) OR (DATE_DIFFERENCE EQ 351) OR (DATE_DIFFERENCE EQ 358) OR (DATE_DIFFERENCE EQ 365) OR (DATE_DIFFERENCE EQ 372) OR (DATE_DIFFERENCE EQ 379) OR (DATE_DIFFERENCE EQ 386) OR (DATE_DIFFERENCE EQ 393) OR (DATE_DIFFERENCE EQ 400) OR (DATE_DIFFERENCE EQ 407) OR (DATE_DIFFERENCE EQ 414) OR (DATE_DIFFERENCE EQ 421))) OR ((TBVAR_TIMENOW LT 600) AND ((DATE_DIFFERENCE EQ 121) OR (DATE_DIFFERENCE EQ 128) OR (DATE_DIFFERENCE EQ 135) OR (DATE_DIFFERENCE EQ 142) OR (DATE_DIFFERENCE EQ 149) OR (DATE_DIFFERENCE EQ 156) OR (DATE_DIFFERENCE EQ 163) OR (DATE_DIFFERENCE EQ 170) OR (DATE_DIFFERENCE EQ 177) OR (DATE_DIFFERENCE EQ 184) OR (DATE_DIFFERENCE EQ 191) OR (DATE_DIFFERENCE EQ 198) OR (DATE_DIFFERENCE EQ 205) OR (DATE_DIFFERENCE EQ 212) OR (DATE_DIFFERENCE EQ 219) OR (DATE_DIFFERENCE EQ 226) OR (DATE_DIFFERENCE EQ 233) OR (DATE_DIFFERENCE EQ 240) OR (DATE_DIFFERENCE EQ 247) OR (DATE_DIFFERENCE EQ 254) OR (DATE_DIFFERENCE EQ 261) OR (DATE_DIFFERENCE EQ 268) OR (DATE_DIFFERENCE EQ 275) OR (DATE_DIFFERENCE EQ 282) OR (DATE_DIFFERENCE EQ 289) OR (DATE_DIFFERENCE EQ 296) OR (DATE_DIFFERENCE EQ 303) OR (DATE_DIFFERENCE EQ 310) OR (DATE_DIFFERENCE EQ 317) OR (DATE_DIFFERENCE EQ 324) OR (DATE_DIFFERENCE EQ 331) OR (DATE_DIFFERENCE EQ 338) OR (DATE_DIFFERENCE EQ 345) OR (DATE_DIFFERENCE EQ 352) OR (DATE_DIFFERENCE EQ 359) OR (DATE_DIFFERENCE EQ 366) OR (DATE_DIFFERENCE EQ 373) OR (DATE_DIFFERENCE EQ 380) OR (DATE_DIFFERENCE EQ 387) OR (DATE_DIFFERENCE EQ 394) OR (DATE_DIFFERENCE EQ 401) OR (DATE_DIFFERENCE EQ 408) OR (DATE_DIFFERENCE EQ 415) OR (DATE_DIFFERENCE EQ 422)))) [DATE_TEXT = 'Lundi'];

IF (((TBVAR_TIMENOW GE 600) AND ((DATE_DIFFERENCE EQ 121) OR (DATE_DIFFERENCE EQ 128) OR (DATE_DIFFERENCE EQ 135) OR (DATE_DIFFERENCE EQ 142) OR (DATE_DIFFERENCE EQ 149) OR (DATE_DIFFERENCE EQ 156) OR (DATE_DIFFERENCE EQ 163) OR (DATE_DIFFERENCE EQ 170) OR (DATE_DIFFERENCE EQ 177) OR (DATE_DIFFERENCE EQ 184) OR (DATE_DIFFERENCE EQ 191) OR (DATE_DIFFERENCE EQ 198) OR (DATE_DIFFERENCE EQ 205) OR (DATE_DIFFERENCE EQ 212) OR (DATE_DIFFERENCE EQ 219) OR (DATE_DIFFERENCE EQ 226) OR (DATE_DIFFERENCE EQ 233) OR (DATE_DIFFERENCE EQ 240) OR (DATE_DIFFERENCE EQ 247) OR (DATE_DIFFERENCE EQ 254) OR (DATE_DIFFERENCE EQ 261) OR (DATE_DIFFERENCE EQ 268) OR (DATE_DIFFERENCE EQ 275) OR (DATE_DIFFERENCE EQ 282) OR (DATE_DIFFERENCE EQ 289) OR (DATE_DIFFERENCE EQ 296) OR (DATE_DIFFERENCE EQ 303) OR (DATE_DIFFERENCE EQ 310) OR (DATE_DIFFERENCE EQ 317) OR (DATE_DIFFERENCE EQ 324) OR (DATE_DIFFERENCE EQ 331) OR (DATE_DIFFERENCE EQ 338) OR (DATE_DIFFERENCE EQ 345) OR (DATE_DIFFERENCE EQ 352) OR (DATE_DIFFERENCE EQ 359) OR (DATE_DIFFERENCE EQ 366) OR (DATE_DIFFERENCE EQ 373) OR (DATE_DIFFERENCE EQ 380) OR (DATE_DIFFERENCE EQ 387) OR (DATE_DIFFERENCE EQ 394) OR (DATE_DIFFERENCE EQ 401) OR (DATE_DIFFERENCE EQ 408) OR (DATE_DIFFERENCE EQ 415) OR (DATE_DIFFERENCE EQ 422))) OR ((TBVAR_TIMENOW LT 600) AND ((DATE_DIFFERENCE EQ 122) OR (DATE_DIFFERENCE EQ 129) OR (DATE_DIFFERENCE EQ 136) OR (DATE_DIFFERENCE EQ 143) OR (DATE_DIFFERENCE EQ 150) OR (DATE_DIFFERENCE EQ 157) OR (DATE_DIFFERENCE EQ 164) OR (DATE_DIFFERENCE EQ 171) OR (DATE_DIFFERENCE EQ 178) OR (DATE_DIFFERENCE EQ 185) OR (DATE_DIFFERENCE EQ 192) OR (DATE_DIFFERENCE EQ 199) OR (DATE_DIFFERENCE EQ 206) OR (DATE_DIFFERENCE EQ 213) OR (DATE_DIFFERENCE EQ 220) OR (DATE_DIFFERENCE EQ 227) OR (DATE_DIFFERENCE EQ 234) OR (DATE_DIFFERENCE EQ 241) OR (DATE_DIFFERENCE EQ 248) OR (DATE_DIFFERENCE EQ 255) OR (DATE_DIFFERENCE EQ 262) OR (DATE_DIFFERENCE EQ 269) OR (DATE_DIFFERENCE EQ 276) OR (DATE_DIFFERENCE EQ 283) OR (DATE_DIFFERENCE EQ 290) OR (DATE_DIFFERENCE EQ 297) OR (DATE_DIFFERENCE EQ 304) OR (DATE_DIFFERENCE EQ 311) OR (DATE_DIFFERENCE EQ 318) OR (DATE_DIFFERENCE EQ 325) OR (DATE_DIFFERENCE EQ 332) OR (DATE_DIFFERENCE EQ 339) OR (DATE_DIFFERENCE EQ 346) OR (DATE_DIFFERENCE EQ 353) OR (DATE_DIFFERENCE EQ 360) OR (DATE_DIFFERENCE EQ 367) OR (DATE_DIFFERENCE EQ 374) OR (DATE_DIFFERENCE EQ 381) OR (DATE_DIFFERENCE EQ 388) OR (DATE_DIFFERENCE EQ 395) OR (DATE_DIFFERENCE EQ 402) OR (DATE_DIFFERENCE EQ 409) OR (DATE_DIFFERENCE EQ 416) OR (DATE_DIFFERENCE EQ 423)))) [DATE_TEXT = 'Mardi'];
IF (((TBVAR_TIMENOW GE 600) AND ((DATE_DIFFERENCE EQ 122) OR (DATE_DIFFERENCE EQ 129) OR (DATE_DIFFERENCE EQ 136) OR (DATE_DIFFERENCE EQ 143) OR (DATE_DIFFERENCE EQ 150) OR (DATE_DIFFERENCE EQ 157) OR (DATE_DIFFERENCE EQ 164) OR (DATE_DIFFERENCE EQ 171) OR (DATE_DIFFERENCE EQ 178) OR (DATE_DIFFERENCE EQ 185) OR (DATE_DIFFERENCE EQ 192) OR (DATE_DIFFERENCE EQ 199) OR (DATE_DIFFERENCE EQ 206) OR (DATE_DIFFERENCE EQ 213) OR (DATE_DIFFERENCE EQ 220) OR (DATE_DIFFERENCE EQ 227) OR (DATE_DIFFERENCE EQ 234) OR (DATE_DIFFERENCE EQ 241) OR (DATE_DIFFERENCE EQ 248) OR (DATE_DIFFERENCE EQ 255) OR (DATE_DIFFERENCE EQ 262) OR (DATE_DIFFERENCE EQ 269) OR (DATE_DIFFERENCE EQ 276) OR (DATE_DIFFERENCE EQ 283) OR (DATE_DIFFERENCE EQ 290) OR (DATE_DIFFERENCE EQ 297) OR (DATE_DIFFERENCE EQ 304) OR (DATE_DIFFERENCE EQ 311) OR (DATE_DIFFERENCE EQ 318) OR (DATE_DIFFERENCE EQ 325) OR (DATE_DIFFERENCE EQ 332) OR (DATE_DIFFERENCE EQ 339) OR

(DATE_DIFFERENCE EQ 346) OR (DATE_DIFFERENCE EQ 353) OR (DATE_DIFFERENCE EQ 360) OR
(DATE_DIFFERENCE EQ 367) OR (DATE_DIFFERENCE EQ 374) OR (DATE_DIFFERENCE EQ 381) OR
(DATE_DIFFERENCE EQ 388) OR (DATE_DIFFERENCE EQ 395) OR (DATE_DIFFERENCE EQ 402) OR
(DATE_DIFFERENCE EQ 409) OR (DATE_DIFFERENCE EQ 416) OR (DATE_DIFFERENCE EQ 423))) OR
((TBVAR_TIMENOW LT 600) AND ((DATE_DIFFERENCE EQ 123) OR (DATE_DIFFERENCE EQ 130) OR
(DATE_DIFFERENCE EQ 137) OR (DATE_DIFFERENCE EQ 144) OR (DATE_DIFFERENCE EQ 151) OR
(DATE_DIFFERENCE EQ 158) OR (DATE_DIFFERENCE EQ 165) OR (DATE_DIFFERENCE EQ 172) OR
(DATE_DIFFERENCE EQ 179) OR (DATE_DIFFERENCE EQ 186) OR (DATE_DIFFERENCE EQ 193) OR
(DATE_DIFFERENCE EQ 200) OR (DATE_DIFFERENCE EQ 207) OR (DATE_DIFFERENCE EQ 214) OR
(DATE_DIFFERENCE EQ 221) OR (DATE_DIFFERENCE EQ 228) OR (DATE_DIFFERENCE EQ 235) OR
(DATE_DIFFERENCE EQ 242) OR (DATE_DIFFERENCE EQ 249) OR (DATE_DIFFERENCE EQ 256) OR
(DATE_DIFFERENCE EQ 263) OR (DATE_DIFFERENCE EQ 270) OR (DATE_DIFFERENCE EQ 277) OR
(DATE_DIFFERENCE EQ 284) OR (DATE_DIFFERENCE EQ 291) OR (DATE_DIFFERENCE EQ 298) OR
(DATE_DIFFERENCE EQ 305) OR (DATE_DIFFERENCE EQ 312) OR (DATE_DIFFERENCE EQ 319) OR
(DATE_DIFFERENCE EQ 326) OR (DATE_DIFFERENCE EQ 333) OR (DATE_DIFFERENCE EQ 340) OR
(DATE_DIFFERENCE EQ 347) OR (DATE_DIFFERENCE EQ 354) OR (DATE_DIFFERENCE EQ 361) OR
(DATE_DIFFERENCE EQ 368) OR (DATE_DIFFERENCE EQ 375) OR (DATE_DIFFERENCE EQ 382) OR
(DATE_DIFFERENCE EQ 389) OR (DATE_DIFFERENCE EQ 396) OR (DATE_DIFFERENCE EQ 403) OR
(DATE_DIFFERENCE EQ 410) OR (DATE_DIFFERENCE EQ 417) OR (DATE_DIFFERENCE EQ 424)))) [DATE_TEXT
= 'Mercredi'];
IF (((TBVAR_TIMENOW GE 600) AND ((DATE_DIFFERENCE EQ 123) OR (DATE_DIFFERENCE EQ 130) OR
(DATE_DIFFERENCE EQ 137) OR (DATE_DIFFERENCE EQ 144) OR (DATE_DIFFERENCE EQ 151) OR
(DATE_DIFFERENCE EQ 158) OR (DATE_DIFFERENCE EQ 165) OR (DATE_DIFFERENCE EQ 172) OR
(DATE_DIFFERENCE EQ 179) OR (DATE_DIFFERENCE EQ 186) OR (DATE_DIFFERENCE EQ 193) OR
(DATE_DIFFERENCE EQ 200) OR (DATE_DIFFERENCE EQ 207) OR (DATE_DIFFERENCE EQ 214) OR
(DATE_DIFFERENCE EQ 221) OR (DATE_DIFFERENCE EQ 228) OR (DATE_DIFFERENCE EQ 235) OR
(DATE_DIFFERENCE EQ 242) OR (DATE_DIFFERENCE EQ 249) OR (DATE_DIFFERENCE EQ 256) OR
(DATE_DIFFERENCE EQ 263) OR (DATE_DIFFERENCE EQ 270) OR (DATE_DIFFERENCE EQ 277) OR
(DATE_DIFFERENCE EQ 284) OR (DATE_DIFFERENCE EQ 291) OR (DATE_DIFFERENCE EQ 298) OR
(DATE_DIFFERENCE EQ 305) OR (DATE_DIFFERENCE EQ 312) OR (DATE_DIFFERENCE EQ 319) OR
(DATE_DIFFERENCE EQ 326) OR (DATE_DIFFERENCE EQ 333) OR (DATE_DIFFERENCE EQ 340) OR
(DATE_DIFFERENCE EQ 347) OR (DATE_DIFFERENCE EQ 354) OR (DATE_DIFFERENCE EQ 361) OR
(DATE_DIFFERENCE EQ 368) OR (DATE_DIFFERENCE EQ 375) OR (DATE_DIFFERENCE EQ 382) OR
(DATE_DIFFERENCE EQ 389) OR (DATE_DIFFERENCE EQ 396) OR (DATE_DIFFERENCE EQ 403) OR
(DATE_DIFFERENCE EQ 410) OR (DATE_DIFFERENCE EQ 417) OR (DATE_DIFFERENCE EQ 424))) OR
((TBVAR_TIMENOW LT 600) AND ((DATE_DIFFERENCE EQ 124) OR (DATE_DIFFERENCE EQ 131) OR
(DATE_DIFFERENCE EQ 138) OR (DATE_DIFFERENCE EQ 145) OR (DATE_DIFFERENCE EQ 152) OR
(DATE_DIFFERENCE EQ 159) OR (DATE_DIFFERENCE EQ 166) OR (DATE_DIFFERENCE EQ 173) OR
(DATE_DIFFERENCE EQ 180) OR (DATE_DIFFERENCE EQ 187) OR (DATE_DIFFERENCE EQ 194) OR
(DATE_DIFFERENCE EQ 201) OR (DATE_DIFFERENCE EQ 208) OR (DATE_DIFFERENCE EQ 215) OR
(DATE_DIFFERENCE EQ 222) OR (DATE_DIFFERENCE EQ 229) OR (DATE_DIFFERENCE EQ 236) OR
(DATE_DIFFERENCE EQ 243) OR (DATE_DIFFERENCE EQ 250) OR (DATE_DIFFERENCE EQ 257) OR

(DATE_DIFFERENCE EQ 264) OR (DATE_DIFFERENCE EQ 271) OR (DATE_DIFFERENCE EQ 278) OR
(DATE_DIFFERENCE EQ 285) OR (DATE_DIFFERENCE EQ 292) OR (DATE_DIFFERENCE EQ 299) OR
(DATE_DIFFERENCE EQ 306) OR (DATE_DIFFERENCE EQ 313) OR (DATE_DIFFERENCE EQ 320) OR
(DATE_DIFFERENCE EQ 327) OR (DATE_DIFFERENCE EQ 334) OR (DATE_DIFFERENCE EQ 341) OR
(DATE_DIFFERENCE EQ 348) OR (DATE_DIFFERENCE EQ 355) OR (DATE_DIFFERENCE EQ 362) OR
(DATE_DIFFERENCE EQ 369) OR (DATE_DIFFERENCE EQ 376) OR (DATE_DIFFERENCE EQ 383) OR
(DATE_DIFFERENCE EQ 390) OR (DATE_DIFFERENCE EQ 397) OR (DATE_DIFFERENCE EQ 404) OR
(DATE_DIFFERENCE EQ 411) OR (DATE_DIFFERENCE EQ 418) OR (DATE_DIFFERENCE EQ 425)))) [DATE_TEXT = 'Jeudi'];
IF (((TBVAR_TIMENOW GE 600) AND ((DATE_DIFFERENCE EQ 124) OR (DATE_DIFFERENCE EQ 131) OR
(DATE_DIFFERENCE EQ 138) OR (DATE_DIFFERENCE EQ 145) OR (DATE_DIFFERENCE EQ 152) OR
(DATE_DIFFERENCE EQ 159) OR (DATE_DIFFERENCE EQ 166) OR (DATE_DIFFERENCE EQ 173) OR
(DATE_DIFFERENCE EQ 180) OR (DATE_DIFFERENCE EQ 187) OR (DATE_DIFFERENCE EQ 194) OR
(DATE_DIFFERENCE EQ 201) OR (DATE_DIFFERENCE EQ 208) OR (DATE_DIFFERENCE EQ 215) OR
(DATE_DIFFERENCE EQ 222) OR (DATE_DIFFERENCE EQ 229) OR (DATE_DIFFERENCE EQ 236) OR
(DATE_DIFFERENCE EQ 243) OR (DATE_DIFFERENCE EQ 250) OR (DATE_DIFFERENCE EQ 257) OR
(DATE_DIFFERENCE EQ 264) OR (DATE_DIFFERENCE EQ 271) OR (DATE_DIFFERENCE EQ 278) OR
(DATE_DIFFERENCE EQ 285) OR (DATE_DIFFERENCE EQ 292) OR (DATE_DIFFERENCE EQ 299) OR
(DATE_DIFFERENCE EQ 306) OR (DATE_DIFFERENCE EQ 313) OR (DATE_DIFFERENCE EQ 320) OR
(DATE_DIFFERENCE EQ 327) OR (DATE_DIFFERENCE EQ 334) OR (DATE_DIFFERENCE EQ 341) OR
(DATE_DIFFERENCE EQ 348) OR (DATE_DIFFERENCE EQ 355) OR (DATE_DIFFERENCE EQ 362) OR
(DATE_DIFFERENCE EQ 369) OR (DATE_DIFFERENCE EQ 376) OR (DATE_DIFFERENCE EQ 383) OR
(DATE_DIFFERENCE EQ 390) OR (DATE_DIFFERENCE EQ 397) OR (DATE_DIFFERENCE EQ 404) OR
(DATE_DIFFERENCE EQ 411) OR (DATE_DIFFERENCE EQ 418) OR (DATE_DIFFERENCE EQ 425))) OR
((TBVAR_TIMENOW LT 600) AND ((DATE_DIFFERENCE EQ 125) OR (DATE_DIFFERENCE EQ 132) OR
(DATE_DIFFERENCE EQ 139) OR (DATE_DIFFERENCE EQ 146) OR (DATE_DIFFERENCE EQ 153) OR
(DATE_DIFFERENCE EQ 160) OR (DATE_DIFFERENCE EQ 167) OR (DATE_DIFFERENCE EQ 174) OR
(DATE_DIFFERENCE EQ 181) OR (DATE_DIFFERENCE EQ 188) OR (DATE_DIFFERENCE EQ 195) OR
(DATE_DIFFERENCE EQ 202) OR (DATE_DIFFERENCE EQ 209) OR (DATE_DIFFERENCE EQ 216) OR
(DATE_DIFFERENCE EQ 223) OR (DATE_DIFFERENCE EQ 230) OR (DATE_DIFFERENCE EQ 237) OR
(DATE_DIFFERENCE EQ 244) OR (DATE_DIFFERENCE EQ 251) OR (DATE_DIFFERENCE EQ 258) OR
(DATE_DIFFERENCE EQ 265) OR (DATE_DIFFERENCE EQ 272) OR (DATE_DIFFERENCE EQ 279) OR
(DATE_DIFFERENCE EQ 286) OR (DATE_DIFFERENCE EQ 293) OR (DATE_DIFFERENCE EQ 300) OR
(DATE_DIFFERENCE EQ 307) OR (DATE_DIFFERENCE EQ 314) OR (DATE_DIFFERENCE EQ 321) OR
(DATE_DIFFERENCE EQ 328) OR (DATE_DIFFERENCE EQ 335) OR (DATE_DIFFERENCE EQ 342) OR
(DATE_DIFFERENCE EQ 349) OR (DATE_DIFFERENCE EQ 356) OR (DATE_DIFFERENCE EQ 363) OR
(DATE_DIFFERENCE EQ 370) OR (DATE_DIFFERENCE EQ 377) OR (DATE_DIFFERENCE EQ 384) OR
(DATE_DIFFERENCE EQ 391) OR (DATE_DIFFERENCE EQ 398) OR (DATE_DIFFERENCE EQ 405) OR
(DATE_DIFFERENCE EQ 412) OR (DATE_DIFFERENCE EQ 419) OR (DATE_DIFFERENCE EQ 426)))) [DATE_TEXT = 'Vendredi'];
IF (((TBVAR_TIMENOW GE 600) AND ((DATE_DIFFERENCE EQ 125) OR (DATE_DIFFERENCE EQ 132) OR
(DATE_DIFFERENCE EQ 139) OR (DATE_DIFFERENCE EQ 146) OR (DATE_DIFFERENCE EQ 153) OR

(DATE_DIFFERENCE EQ 160) OR (DATE_DIFFERENCE EQ 167) OR (DATE_DIFFERENCE EQ 174) OR
(DATE_DIFFERENCE EQ 181) OR (DATE_DIFFERENCE EQ 188) OR (DATE_DIFFERENCE EQ 195) OR
(DATE_DIFFERENCE EQ 202) OR (DATE_DIFFERENCE EQ 209) OR (DATE_DIFFERENCE EQ 216) OR
(DATE_DIFFERENCE EQ 223) OR (DATE_DIFFERENCE EQ 230) OR (DATE_DIFFERENCE EQ 237) OR
(DATE_DIFFERENCE EQ 244) OR (DATE_DIFFERENCE EQ 251) OR (DATE_DIFFERENCE EQ 258) OR
(DATE_DIFFERENCE EQ 265) OR (DATE_DIFFERENCE EQ 272) OR (DATE_DIFFERENCE EQ 279) OR
(DATE_DIFFERENCE EQ 286) OR (DATE_DIFFERENCE EQ 293) OR (DATE_DIFFERENCE EQ 300) OR
(DATE_DIFFERENCE EQ 307) OR (DATE_DIFFERENCE EQ 314) OR (DATE_DIFFERENCE EQ 321) OR
(DATE_DIFFERENCE EQ 328) OR (DATE_DIFFERENCE EQ 335) OR (DATE_DIFFERENCE EQ 342) OR
(DATE_DIFFERENCE EQ 349) OR (DATE_DIFFERENCE EQ 356) OR (DATE_DIFFERENCE EQ 363) OR
(DATE_DIFFERENCE EQ 370) OR (DATE_DIFFERENCE EQ 377) OR (DATE_DIFFERENCE EQ 384) OR
(DATE_DIFFERENCE EQ 391) OR (DATE_DIFFERENCE EQ 398) OR (DATE_DIFFERENCE EQ 405) OR
(DATE_DIFFERENCE EQ 412) OR (DATE_DIFFERENCE EQ 419) OR (DATE_DIFFERENCE EQ 426))) OR
((TBVAR_TIMENOW LT 600) AND ((DATE_DIFFERENCE EQ 126) OR (DATE_DIFFERENCE EQ 133) OR
(DATE_DIFFERENCE EQ 140) OR (DATE_DIFFERENCE EQ 147) OR (DATE_DIFFERENCE EQ 154) OR
(DATE_DIFFERENCE EQ 161) OR (DATE_DIFFERENCE EQ 168) OR (DATE_DIFFERENCE EQ 175) OR
(DATE_DIFFERENCE EQ 182) OR (DATE_DIFFERENCE EQ 189) OR (DATE_DIFFERENCE EQ 196) OR
(DATE_DIFFERENCE EQ 203) OR (DATE_DIFFERENCE EQ 210) OR (DATE_DIFFERENCE EQ 217) OR
(DATE_DIFFERENCE EQ 224) OR (DATE_DIFFERENCE EQ 231) OR (DATE_DIFFERENCE EQ 238) OR
(DATE_DIFFERENCE EQ 245) OR (DATE_DIFFERENCE EQ 252) OR (DATE_DIFFERENCE EQ 259) OR
(DATE_DIFFERENCE EQ 266) OR (DATE_DIFFERENCE EQ 273) OR (DATE_DIFFERENCE EQ 280) OR
(DATE_DIFFERENCE EQ 287) OR (DATE_DIFFERENCE EQ 294) OR (DATE_DIFFERENCE EQ 301) OR
(DATE_DIFFERENCE EQ 308) OR (DATE_DIFFERENCE EQ 315) OR (DATE_DIFFERENCE EQ 322) OR
(DATE_DIFFERENCE EQ 329) OR (DATE_DIFFERENCE EQ 336) OR (DATE_DIFFERENCE EQ 343) OR
(DATE_DIFFERENCE EQ 350) OR (DATE_DIFFERENCE EQ 357) OR (DATE_DIFFERENCE EQ 364) OR
(DATE_DIFFERENCE EQ 371) OR (DATE_DIFFERENCE EQ 378) OR (DATE_DIFFERENCE EQ 385) OR
(DATE_DIFFERENCE EQ 392) OR (DATE_DIFFERENCE EQ 399) OR (DATE_DIFFERENCE EQ 406) OR
(DATE_DIFFERENCE EQ 413) OR (DATE_DIFFERENCE EQ 420) OR (DATE_DIFFERENCE EQ 427)))) [DATE_TEXT = 'Samedi'];
IF (((TBVAR_TIMENOW GE 600) AND ((DATE_DIFFERENCE EQ 126) OR (DATE_DIFFERENCE EQ 133) OR
(DATE_DIFFERENCE EQ 140) OR (DATE_DIFFERENCE EQ 147) OR (DATE_DIFFERENCE EQ 154) OR
(DATE_DIFFERENCE EQ 161) OR (DATE_DIFFERENCE EQ 168) OR (DATE_DIFFERENCE EQ 175) OR
(DATE_DIFFERENCE EQ 182) OR (DATE_DIFFERENCE EQ 189) OR (DATE_DIFFERENCE EQ 196) OR
(DATE_DIFFERENCE EQ 203) OR (DATE_DIFFERENCE EQ 210) OR (DATE_DIFFERENCE EQ 217) OR
(DATE_DIFFERENCE EQ 224) OR (DATE_DIFFERENCE EQ 231) OR (DATE_DIFFERENCE EQ 238) OR
(DATE_DIFFERENCE EQ 245) OR (DATE_DIFFERENCE EQ 252) OR (DATE_DIFFERENCE EQ 259) OR
(DATE_DIFFERENCE EQ 266) OR (DATE_DIFFERENCE EQ 273) OR (DATE_DIFFERENCE EQ 280) OR
(DATE_DIFFERENCE EQ 287) OR (DATE_DIFFERENCE EQ 294) OR (DATE_DIFFERENCE EQ 301) OR
(DATE_DIFFERENCE EQ 308) OR (DATE_DIFFERENCE EQ 315) OR (DATE_DIFFERENCE EQ 322) OR
(DATE_DIFFERENCE EQ 329) OR (DATE_DIFFERENCE EQ 336) OR (DATE_DIFFERENCE EQ 343) OR
(DATE_DIFFERENCE EQ 350) OR (DATE_DIFFERENCE EQ 357) OR (DATE_DIFFERENCE EQ 364) OR
(DATE_DIFFERENCE EQ 371) OR (DATE_DIFFERENCE EQ 378) OR (DATE_DIFFERENCE EQ 385) OR

(DATE_DIFFERENCE EQ 392) OR (DATE_DIFFERENCE EQ 399) OR (DATE_DIFFERENCE EQ 406) OR (DATE_DIFFERENCE EQ 413) OR (DATE_DIFFERENCE EQ 420) OR (DATE_DIFFERENCE EQ 427))) OR ((TBVAR_TIMENOW LT 600) AND ((DATE_DIFFERENCE EQ 120) OR (DATE_DIFFERENCE EQ 127) OR (DATE_DIFFERENCE EQ 134) OR (DATE_DIFFERENCE EQ 141) OR (DATE_DIFFERENCE EQ 148) OR (DATE_DIFFERENCE EQ 155) OR (DATE_DIFFERENCE EQ 162) OR (DATE_DIFFERENCE EQ 169) OR (DATE_DIFFERENCE EQ 176) OR (DATE_DIFFERENCE EQ 183) OR (DATE_DIFFERENCE EQ 190) OR (DATE_DIFFERENCE EQ 197) OR (DATE_DIFFERENCE EQ 204) OR (DATE_DIFFERENCE EQ 211) OR (DATE_DIFFERENCE EQ 218) OR (DATE_DIFFERENCE EQ 225) OR (DATE_DIFFERENCE EQ 232) OR (DATE_DIFFERENCE EQ 239) OR (DATE_DIFFERENCE EQ 246) OR (DATE_DIFFERENCE EQ 253) OR (DATE_DIFFERENCE EQ 260) OR (DATE_DIFFERENCE EQ 267) OR (DATE_DIFFERENCE EQ 274) OR (DATE_DIFFERENCE EQ 281) OR (DATE_DIFFERENCE EQ 288) OR (DATE_DIFFERENCE EQ 295) OR (DATE_DIFFERENCE EQ 302) OR (DATE_DIFFERENCE EQ 309) OR (DATE_DIFFERENCE EQ 316) OR (DATE_DIFFERENCE EQ 323) OR (DATE_DIFFERENCE EQ 330) OR (DATE_DIFFERENCE EQ 337) OR (DATE_DIFFERENCE EQ 344) OR (DATE_DIFFERENCE EQ 351) OR (DATE_DIFFERENCE EQ 358) OR (DATE_DIFFERENCE EQ 365) OR (DATE_DIFFERENCE EQ 372) OR (DATE_DIFFERENCE EQ 379) OR (DATE_DIFFERENCE EQ 386) OR (DATE_DIFFERENCE EQ 393) OR (DATE_DIFFERENCE EQ 400) OR (DATE_DIFFERENCE EQ 407) OR (DATE_DIFFERENCE EQ 414) OR (DATE_DIFFERENCE EQ 421)))) [DATE_TEXT = 'Dimanche'];
Codes for indicating number of days left before the program
[DATE_STARTPROG = '28-09-2014'];
[DAYS_DIFFERENCE = DATE_STARTPROG - TBVAR_DATENOW];
IF (TBVAR_TIMENOW LE 600) [DAYS_DIFFERENCE = DAYS_DIFFERENCE + 1];
variables for gender differences
IF (SEXE EQ 2) [LETTRE_E = 'e'];
IF (SEXE EQ 1) [ACTIF_VE = 'actif'];
IF (SEXE EQ 2) [ACTIF_VE = 'active'];
VARIABLE POUR LA SÉANCE 2 sur l'attitude et les bénéfices de l'activité physique
IF (S2.QELAB1 NE 0) [S2.PRESUPRAELAB1 = 'l´impact qu´aurait ce bénéfice dans votre vie: '];
IF (S2.QELAB2 NE 0) [S2.PRESUPRAELAB2 = 'l´impact qu´aurait ce bénéfice dans votre vie: '];
IF (SI_MI_ATTITUDE EQ 0) [S2.ATT.RES = 'Votre attitude face à l´activité physique était assez défavorable au moment de l´inscription. Bravo pour avoir persévéré malgré tout et pour avoir effectué la séance d´aujourd´hui.'];
IF ((SI_MI_ATTITUDE GE 1) AND (SI_MI_ATTITUDE LE 5)) [S2.ATT.RES = 'Il semble que votre attitude face à l´activité physique était un peu défavorable au moment de l´inscription. Bravo pour avoir persévéré malgré tout et pour avoir effectué la séance d´aujourd´hui.'];
IF ((SI_MI_ATTITUDE GE 6) AND (SI_MI_ATTITUDE LE 8)) [S2.ATT.RES = 'En fonction de vos réponses à l´inscription, il semble que votre attitude face à l´activité physique soit plutôt favorable. Voilà une excellente nouvelle!'];
IF (SI_MI_ATTITUDE GE 9) [S2.ATT.RES = 'En fonction de vos réponses à l´inscription, il semble que votre attitude soit très positive face au fait de pratiquer régulièrement des activités physiques. C´est une excellent nouvelle!'];
IF (S2.QELAB1 EQ 0) [S2.SUPRAELAB1 = 'Quel impact aurait ce bénéfice dans votre vie?'];

IF (S2.QELAB1 NE 0) [S2.SUPRAELAB1 = S2.QELAB1];
IF (S2.QELAB2 EQ 0) [S2.SUPRAELAB2 = 'Quel impact aurait ce bénéfice dans votre vie?'];
IF (S2.QELAB2 NE 0) [S2.SUPRAELAB2 = S2.QELAB2];
IF (S2.INFOFEEL EQ 0) [S2.INFORESINTRO = 'Voici un court résumé de votre séance d´aujourd´hui. Bravo pour avoir trouvé vos propres raisons pouvant vous motiver à bouger davantage.'];
IF (S2.INFOFEEL EQ 1) [S2.INFORESINTRO = 'Merci de partager votre appréciation des informations. Il semble que vous ayez aimé les informations précédentes! Voici maintenant un court résumé de votre séance. Bravo d´avoir trouvé vos propres raisons de bouger davantage.'];
IF (S2.INFOFEEL EQ 2) [S2.INFORESINTRO = 'Mmmm...Il semble que vous n´ayez pas aimé les informations précédentes. Nous espérons sincèrement pouvoir faire mieux à l´avenir. Voici maintenant, si vous voulez, un court résumé de votre séance.'];
IF (S2.INFOFEEL EQ 3) [S2.INFORESINTRO = 'Mmmm...Il semble que vous ignorez quoi penser des informations précédentes. Nous espérons que cela pourra quand même vous servir. Voici maintenant, si vous voulez, un court résumé de votre séance.'];
[BENSUM = QBEN1+QBEN2+QBEN3+QBEN4+QBEN5+QBEN6+QBEN7+QBEN8];
[SUM.3PBEN = Q.3PBEN1+Q.3PBEN2+Q.3PBEN3+Q.3PBEN4+Q.3PBEN5+Q.3PBEN6+Q.3PBEN7+Q.3PBEN8];
IF ((BENSUM EQ 101) OR (SUM.3PBEN EQ 101)) [S2.REFBENTXT = 'augmenter votre niveau d´énergie au quotidien'];
IF ((BENSUM EQ 102) OR (SUM.3PBEN EQ 102)) [S2.REFBENTXT = 'diminuer votre niveau de stress au quotidien'];
IF ((BENSUM EQ 103) OR (SUM.3PBEN EQ 103)) [S2.REFBENTXT = 'contrôler votre poids'];
IF ((BENSUM EQ 104) OR (SUM.3PBEN EQ 104)) [S2.REFBENTXT = 'favoriser les interactions avec les autres'];
IF ((BENSUM EQ 105) OR (SUM.3PBEN EQ 105)) [S2.REFBENTXT = 'réduire vos risques de maladies'];
IF ((BENSUM EQ 106) OR (SUM.3PBEN EQ 106)) [S2.REFBENTXT = 'vous sentir mieux dans votre peau'];
IF ((BENSUM EQ 107) OR (SUM.3PBEN EQ 107)) [S2.REFBENTXT = 'avoir une meilleure qualité de vie'];
IF ((BENSUM EQ 108) OR (SUM.3PBEN EQ 108)) [S2.REFBENTXT = 'avoir un meilleur sommeil'];
IF (((BENSUM GE 200) AND (BENSUM LT 300) AND (QBEN1 EQ 101) AND (QBEN2 EQ 102)) OR ((Q.3PBEN1 EQ 101) AND (Q.3PBEN2 EQ 102))) [S2.REFBENTXT = 'augmenter votre niveau d´énergie et de diminuer votre niveau de stress'];
IF (((BENSUM GE 200) AND (BENSUM LT 300) AND (QBEN1 EQ 101) AND (QBEN3 EQ 103)) OR ((Q.3PBEN1 EQ 101) AND (Q.3PBEN3 EQ 103))) [S2.REFBENTXT = 'augmenter votre niveau d´énergie et de contrôler votre poids'];
IF (((BENSUM GE 200) AND (BENSUM LT 300) AND (QBEN1 EQ 101) AND (QBEN4 EQ 104)) OR ((Q.3PBEN1 EQ 101) AND (Q.3PBEN4 EQ 104))) [S2.REFBENTXT = 'augmenter votre niveau d´énergie et de favoriser vos interactions sociales'];
IF (((BENSUM GE 200) AND (BENSUM LT 300) AND (QBEN1 EQ 101) AND (QBEN5 EQ 105)) OR ((Q.3PBEN1 EQ 101) AND (Q.3PBEN5 EQ 105))) [S2.REFBENTXT = 'augmenter votre niveau d´énergie et de réduire vos risques de maladies'];
IF (((BENSUM GE 200) AND (BENSUM LT 300) AND (QBEN1 EQ 101) AND (QBEN6 EQ 106)) OR ((Q.3PBEN1 EQ 101) AND (Q.3PBEN6 EQ 106))) [S2.REFBENTXT = 'augmenter votre niveau d´énergie et de vous sentir mieux dans votre peau'];

IF (((BENSUM GE 200) AND (BENSUM LT 300) AND (QBEN1 EQ 101) AND (QBEN7 EQ 107)) OR ((Q.3PBEN1 EQ 101) AND (Q.3PBEN7 EQ 107))) [S2.REFBENTXT = 'augmenter votre niveau d´énergie et d´avoir une meilleure qualité de vie'];
IF (((BENSUM GE 200) AND (BENSUM LT 300) AND (QBEN1 EQ 101) AND (QBEN8 EQ 108)) OR ((Q.3PBEN1 EQ 101) AND (Q.3PBEN8 EQ 108))) [S2.REFBENTXT = 'augmenter votre niveau d´énergie et d´avoir un meilleur sommeil'];
IF (((BENSUM GE 200) AND (BENSUM LT 300) AND (QBEN2 EQ 102) AND (QBEN3 EQ 103)) OR ((Q.3PBEN2 EQ 102) AND (Q.3PBEN3 EQ 103))) [S2.REFBENTXT = 'diminuer votre niveau de stress et de contrôler votre poids'];
IF (((BENSUM GE 200) AND (BENSUM LT 300) AND (QBEN2 EQ 102) AND (QBEN4 EQ 104)) OR ((Q.3PBEN2 EQ 102) AND (Q.3PBEN4 EQ 104))) [S2.REFBENTXT = 'diminuer votre niveau de stress et de favoriser vos interactions sociales'];
IF (((BENSUM GE 200) AND (BENSUM LT 300) AND (QBEN2 EQ 102) AND (QBEN5 EQ 105)) OR ((Q.3PBEN2 EQ 102) AND (Q.3PBEN5 EQ 105))) [S2.REFBENTXT = 'diminuer votre niveau de stress et de réduire vos risques de maladies'];
IF (((BENSUM GE 200) AND (BENSUM LT 300) AND (QBEN2 EQ 102) AND (QBEN6 EQ 106)) OR ((Q.3PBEN2 EQ 102) AND (Q.3PBEN6 EQ 106))) [S2.REFBENTXT = 'diminuer votre niveau de stress et de vous sentir mieux dans votre peau'];
IF (((BENSUM GE 200) AND (BENSUM LT 300) AND (QBEN2 EQ 102) AND (QBEN7 EQ 107)) OR ((Q.3PBEN2 EQ 102) AND (Q.3PBEN7 EQ 107))) [S2.REFBENTXT = 'diminuer votre niveau de stress et d´avoir une meilleure qualité de vie'];
IF (((BENSUM GE 200) AND (BENSUM LT 300) AND (QBEN2 EQ 102) AND (QBEN8 EQ 108)) OR ((Q.3PBEN2 EQ 102) AND (Q.3PBEN8 EQ 108))) [S2.REFBENTXT = 'diminuer votre niveau de stress et d´avoir un meilleur sommeil'];
IF (((BENSUM GE 200) AND (BENSUM LT 300) AND (QBEN3 EQ 103) AND (QBEN4 EQ 104)) OR ((Q.3PBEN3 EQ 103) AND (Q.3PBEN4 EQ 104))) [S2.REFBENTXT = 'contrôler votre poids et de favoriser vos interactions sociales'];
IF (((BENSUM GE 200) AND (BENSUM LT 300) AND (QBEN3 EQ 103) AND (QBEN5 EQ 105)) OR ((Q.3PBEN3 EQ 103) AND (Q.3PBEN5 EQ 105))) [S2.REFBENTXT = 'contrôler votre poids et de réduire vos risques de maladies'];
IF (((BENSUM GE 200) AND (BENSUM LT 300) AND (QBEN3 EQ 103) AND (QBEN6 EQ 106)) OR ((Q.3PBEN3 EQ 103) AND (Q.3PBEN6 EQ 106))) [S2.REFBENTXT = 'contrôler votre poids et de vous sentir mieux dans votre peau'];
IF (((BENSUM GE 200) AND (BENSUM LT 300) AND (QBEN3 EQ 103) AND (QBEN7 EQ 107)) OR ((Q.3PBEN3 EQ 103) AND (Q.3PBEN7 EQ 107))) [S2.REFBENTXT = 'contrôler votre poids et d´avoir une meilleure qualité de vie'];
IF (((BENSUM GE 200) AND (BENSUM LT 300) AND (QBEN3 EQ 103) AND (QBEN8 EQ 108)) OR ((Q.3PBEN3 EQ 103) AND (Q.3PBEN8 EQ 108))) [S2.REFBENTXT = 'contrôler votre poids et d´avoir un meilleur sommeil'];
IF (((BENSUM GE 200) AND (BENSUM LT 300) AND (QBEN4 EQ 104) AND (QBEN5 EQ 105)) OR ((Q.3PBEN4 EQ 104) AND (Q.3PBEN5 EQ 105))) [S2.REFBENTXT = 'favoriser vos interactions sociales et de réduire vos risques de maladies'];

IF (((BENSUM GE 200) AND (BENSUM LT 300) AND (QBEN4 EQ 104) AND (QBEN6 EQ 106)) OR ((Q.3PBEN4 EQ 104) AND (Q.3PBEN6 EQ 106))) [S2.REFBENTXT = 'favoriser vos interactions sociales et de vous sentir mieux dans votre peau'];
IF (((BENSUM GE 200) AND (BENSUM LT 300) AND (QBEN4 EQ 104) AND (QBEN7 EQ 107)) OR ((Q.3PBEN4 EQ 104) AND (Q.3PBEN7 EQ 107))) [S2.REFBENTXT = 'favoriser vos interactions sociales et d´avoir une meilleure qualité de vie'];
IF (((BENSUM GE 200) AND (BENSUM LT 300) AND (QBEN4 EQ 104) AND (QBEN8 EQ 108)) OR ((Q.3PBEN4 EQ 104) AND (Q.3PBEN8 EQ 108))) [S2.REFBENTXT = 'favoriser vos interactions sociales et d´avoir un meilleur sommeil'];
IF (((BENSUM GE 200) AND (BENSUM LT 300) AND (QBEN5 EQ 105) AND (QBEN6 EQ 106)) OR ((Q.3PBEN5 EQ 105) AND (Q.3PBEN6 EQ 106))) [S2.REFBENTXT = 'réduire vos risques de maladies et de vous sentir mieux dans votre peau'];
IF (((BENSUM GE 200) AND (BENSUM LT 300) AND (QBEN5 EQ 105) AND (QBEN7 EQ 107)) OR ((Q.3PBEN5 EQ 105) AND (Q.3PBEN7 EQ 107))) [S2.REFBENTXT = 'réduire vos risques de maladies et d´avoir une meilleure qualité de vie'];
IF (((BENSUM GE 200) AND (BENSUM LT 300) AND (QBEN5 EQ 105) AND (QBEN8 EQ 108)) OR ((Q.3PBEN5 EQ 105) AND (Q.3PBEN8 EQ 108))) [S2.REFBENTXT = 'réduire vos risques de maladies et d´avoir un meilleur sommeil'];
IF (((BENSUM GE 200) AND (BENSUM LT 300) AND (QBEN6 EQ 106) AND (QBEN7 EQ 107)) OR ((Q.3PBEN6 EQ 106) AND (Q.3PBEN7 EQ 107))) [S2.REFBENTXT = 'vous sentir mieux dans votre peau et d´avoir une meilleure qualité de vie'];
IF (((BENSUM GE 200) AND (BENSUM LT 300) AND (QBEN6 EQ 106) AND (QBEN8 EQ 108)) OR ((Q.3PBEN6 EQ 106) AND (Q.3PBEN8 EQ 108))) [S2.REFBENTXT = 'vous sentir mieux dans votre peau et d´avoir un meilleur sommeil'];
IF (((BENSUM GE 200) AND (BENSUM LT 300) AND (QBEN7 EQ 107) AND (QBEN8 EQ 108)) OR ((Q.3PBEN7 EQ 107) AND (Q.3PBEN8 EQ 108))) [S2.REFBENTXT = 'avoir une meilleure qualité de vie et un meilleur sommeil'];
IF (((BENSUM GE 100) AND (BENSUM LT 300) AND (QBEN1 EQ 101)) OR ((BENSUM GE 100) AND (BENSUM LT 300) AND (QBEN7 EQ 107) AND (QBEN2 EQ 0) AND (QBEN3 EQ 0) AND (QBEN4 EQ 0) AND (QBEN5 EQ 0) AND (QBEN6 EQ 0)) OR ((BENSUM GE 100) AND (BENSUM LT 300) AND (QBEN8 EQ 108) AND (QBEN2 EQ 0) AND (QBEN3 EQ 0) AND (QBEN4 EQ 0) AND (QBEN5 EQ 0) AND (QBEN6 EQ 0)) OR ((BENSUM GE 300) AND (Q.3PBEN1 EQ 101)) OR ((BENSUM GE 300) AND (Q.3PBEN7 EQ 107) AND (Q.3PBEN2 EQ 0) AND (Q.3PBEN3 EQ 0) AND (Q.3PBEN4 EQ 0) AND (Q.3PBEN5 EQ 0) AND (Q.3PBEN6 EQ 0)) OR ((BENSUM GE 300) AND (Q.3PBEN8 EQ 108) AND (Q.3PBEN2 EQ 0) AND (Q.3PBEN3 EQ 0) AND (Q.3PBEN4 EQ 0) AND (Q.3PBEN5 EQ 0) AND (Q.3PBEN6 EQ 0))) [DE = 'd´'];
IF (((BENSUM GE 100) AND (BENSUM LT 300) AND (QBEN1 EQ 0) AND ((QBEN2 EQ 102) OR (QBEN3 EQ 103) OR (QBEN4 EQ 104) OR (QBEN5 EQ 105) OR (QBEN6 EQ 106))) OR ((BENSUM GE 300) AND (Q.3PBEN1 EQ 0) AND ((Q.3PBEN2 EQ 102) OR (Q.3PBEN3 EQ 103) OR (Q.3PBEN4 EQ 104) OR (Q.3PBEN5 EQ 105) OR (Q.3PBEN6 EQ 106)))) [DE = 'de '];
IF (QBEN1 EQ 101) [BEN1TXT = 'Augmenter votre niveau d´énergie au quotidien'];
IF (QBEN2 EQ 102) [BEN2TXT = 'Diminuer votre niveau de stress au quotidien'];
IF (QBEN3 EQ 103) [BEN3TXT = 'Contrôler votre poids'];

IF (QBEN4 EQ 104) [BEN4TXT = 'Favoriser vos interactions avec les autres'];
IF (QBEN5 EQ 105) [BEN5TXT = 'Réduire vos risques de maladies'];
IF (QBEN6 EQ 106) [BEN6TXT = 'Vous sentir mieux dans votre peau'];
IF (QBEN7 EQ 107) [BEN7TXT = 'Avoir une meilleure qualité de vie'];
IF (QBEN8 EQ 108) [BEN8TXT = 'Avoir un meilleur sommeil'];
IF (((BENSUM GE 100) AND (BENSUM LT 300) AND (QBEN1 EQ 101)) OR ((BENSUM GE 300) AND (Q.3PBEN1 EQ 101))) [S2.ELABTXT1 = 'augmenter votre niveau d´énergie au quotidien'];
IF (((BENSUM GE 100) AND (BENSUM LT 300) AND (QBEN1 EQ 0) AND (QBEN2 EQ 102)) OR ((BENSUM GE 300) AND (Q.3PBEN1 EQ 0) AND (Q.3PBEN2 EQ 102))) [S2.ELABTXT1 = 'diminuer votre niveau de stress au quotidien'];
IF (((BENSUM GE 100) AND (BENSUM LT 300) AND (QBEN1 EQ 0) AND (QBEN2 EQ 0) AND (QBEN3 EQ 103)) OR ((BENSUM GE 300) AND (Q.3PBEN1 EQ 0) AND (Q.3PBEN2 EQ 0) AND (Q.3PBEN3 EQ 103))) [S2.ELABTXT1 = 'contrôler votre poids'];
IF (((BENSUM GE 100) AND (BENSUM LT 300) AND (QBEN1 EQ 0) AND (QBEN2 EQ 0) AND (QBEN3 EQ 0) AND (QBEN4 EQ 104)) OR ((BENSUM GE 300) AND (Q.3PBEN1 EQ 0) AND (Q.3PBEN2 EQ 0) AND (Q.3PBEN3 EQ 0) AND (Q.3PBEN4 EQ 104))) [S2.ELABTXT1 = 'favoriser vos interactions avec les autres'];
IF (((BENSUM GE 100) AND (BENSUM LT 300) AND (QBEN1 EQ 0) AND (QBEN2 EQ 0) AND (QBEN3 EQ 0) AND (QBEN4 EQ 0) AND (QBEN5 EQ 105)) OR ((BENSUM GE 300) AND (Q.3PBEN1 EQ 0) AND (Q.3PBEN2 EQ 0) AND (Q.3PBEN3 EQ 0) AND (Q.3PBEN4 EQ 0) AND (Q.3PBEN5 EQ 105))) [S2.ELABTXT1 = 'réduire vos risques de maladies'];
IF (((BENSUM GE 100) AND (BENSUM LT 300) AND (QBEN1 EQ 0) AND (QBEN2 EQ 0) AND (QBEN3 EQ 0) AND (QBEN4 EQ 0) AND (QBEN5 EQ 0) AND (QBEN6 EQ 106)) OR ((BENSUM GE 300) AND (Q.3PBEN1 EQ 0) AND (Q.3PBEN2 EQ 0) AND (Q.3PBEN3 EQ 0) AND (Q.3PBEN4 EQ 0) AND (Q.3PBEN5 EQ 0) AND (Q.3PBEN6 EQ 106))) [S2.ELABTXT1 = 'vous sentir mieux dans votre peau'];
IF (((BENSUM GE 100) AND (BENSUM LT 300) AND (QBEN1 EQ 0) AND (QBEN2 EQ 0) AND (QBEN3 EQ 0) AND (QBEN4 EQ 0) AND (QBEN5 EQ 0) AND (QBEN6 EQ 0) AND (QBEN7 EQ 107)) OR ((BENSUM GE 300) AND (Q.3PBEN1 EQ 0) AND (Q.3PBEN2 EQ 0) AND (Q.3PBEN3 EQ 0) AND (Q.3PBEN4 EQ 0) AND (Q.3PBEN5 EQ 0) AND (Q.3PBEN6 EQ 0) AND (Q.3PBEN7 EQ 107))) [S2.ELABTXT1 = 'avoir une meilleure qualité de vie'];
IF (((BENSUM GE 100) AND (BENSUM LT 300) AND (QBEN1 EQ 0) AND (QBEN2 EQ 0) AND (QBEN3 EQ 0) AND (QBEN4 EQ 0) AND (QBEN5 EQ 0) AND (QBEN6 EQ 0) AND (QBEN7 EQ 0) AND (QBEN8 EQ 108)) OR ((BENSUM GE 300) AND (Q.3PBEN1 EQ 0) AND (Q.3PBEN2 EQ 0) AND (Q.3PBEN3 EQ 0) AND (Q.3PBEN4 EQ 0) AND (Q.3PBEN5 EQ 0) AND (Q.3PBEN6 EQ 0) AND (Q.3PBEN7 EQ 0) AND (Q.3PBEN8 EQ 108))) [S2.ELABTXT1 = 'avoir une meilleure qualité de vie'];
IF (((BENSUM GE 100) AND (BENSUM LT 300) AND (QBEN1 EQ 101) AND (QBEN2 EQ 102)) OR ((BENSUM GE 300) AND (Q.3PBEN1 EQ 101) AND (Q.3PBEN2 EQ 102))) [S2.ELABTXT2 = 'diminuer votre niveau de stress au quotidien'];
IF (((BENSUM GE 100) AND (BENSUM LT 300) AND ((QBEN1 EQ 101) OR (QBEN2 EQ 102)) AND (QBEN3 EQ 103)) OR ((BENSUM GE 300) AND ((Q.3PBEN1 EQ 101) OR (Q.3PBEN2 EQ 102)) AND (Q.3PBEN3 EQ 103))) [S2.ELABTXT2 = 'contrôler votre poids'];
IF (((BENSUM GE 100) AND (BENSUM LT 300) AND ((QBEN1 EQ 101) OR (QBEN2 EQ 102) OR (QBEN3 EQ 103)) AND (QBEN4 EQ 104)) OR ((BENSUM GE 300) AND ((Q.3PBEN1 EQ 101) OR (Q.3PBEN2 EQ 102) OR (Q.3PBEN3 EQ 103)) AND (Q.3PBEN4 EQ 104))) [S2.ELABTXT2 = 'favoriser vos interactions avec les autres'];

IF (((BENSUM GE 100) AND (BENSUM LT 300) AND ((QBEN1 EQ 101) OR (QBEN2 EQ 102) OR (QBEN3 EQ 103) OR (QBEN4 EQ 104)) AND (QBEN5 EQ 105)) OR ((BENSUM GE 300) AND ((Q.3PBEN1 EQ 101) OR (Q.3PBEN2 EQ 102) OR (Q.3PBEN3 EQ 103) OR (Q.3PBEN4 EQ 104)) AND (Q.3PBEN5 EQ 105))) [S2.ELABTXT2 = 'réduire vos risques de maladies'];
IF (((BENSUM GE 100) AND (BENSUM LT 300) AND ((QBEN1 EQ 101) OR (QBEN2 EQ 102) OR (QBEN3 EQ 103) OR (QBEN4 EQ 104) OR (QBEN5 EQ 105)) AND (QBEN6 EQ 106)) OR ((BENSUM GE 300) AND ((Q.3PBEN1 EQ 101) OR (Q.3PBEN2 EQ 102) OR (Q.3PBEN3 EQ 103) OR (Q.3PBEN4 EQ 104) OR (Q.3PBEN5 EQ 105)) AND (Q.3PBEN6 EQ 106))) [S2.ELABTXT2 = 'vous sentir mieux dans votre peau'];
IF (((BENSUM GE 100) AND (BENSUM LT 300) AND ((QBEN1 EQ 101) OR (QBEN2 EQ 102) OR (QBEN3 EQ 103) OR (QBEN4 EQ 104) OR (QBEN5 EQ 105) OR (QBEN6 EQ 106)) AND (QBEN7 EQ 107)) OR ((BENSUM GE 300) AND ((Q.3PBEN1 EQ 101) OR (Q.3PBEN2 EQ 102) OR (Q.3PBEN3 EQ 103) OR (Q.3PBEN4 EQ 104) OR (Q.3PBEN5 EQ 105) OR (Q.3PBEN6 EQ 106)) AND (Q.3PBEN7 EQ 107))) [S2.ELABTXT2 = 'avoir une meilleure qualité de vie'];
IF (((BENSUM GE 100) AND (BENSUM LT 300) AND ((QBEN1 EQ 101) OR (QBEN2 EQ 102) OR (QBEN3 EQ 103) OR (QBEN4 EQ 104) OR (QBEN5 EQ 105) OR (QBEN6 EQ 106) OR (QBEN7 EQ 107)) AND (QBEN8 EQ 108)) OR ((BENSUM GE 300) AND ((Q.3PBEN1 EQ 101) AND (Q.3PBEN2 EQ 102) AND (Q.3PBEN3 EQ 103) AND (Q.3PBEN4 EQ 104) AND (Q.3PBEN5 EQ 105) AND (Q.3PBEN6 EQ 106) AND (Q.3PBEN7 EQ 107)) AND (Q.3PBEN8 EQ 108))) [S2.ELABTXT2 = 'avoir une meilleure qualité de vie'];
IF (((BENSUM GE 100) AND (BENSUM LT 300) AND (QBEN1 EQ 101)) OR ((BENSUM GE 300) AND (Q.3PBEN1 EQ 101))) [S2.TEMTXT1 = 'Je me sentirais vraiment mieux avec plus d´énergie. Je pourrais faire tout ce que je veux vraiment faire dans une journée, sans être limité.'];
IF (((BENSUM GE 100) AND (BENSUM LT 300) AND (QBEN1 EQ 0) AND (QBEN2 EQ 102)) OR ((BENSUM GE 300) AND (Q.3PBEN1 EQ 0) AND (Q.3PBEN2 EQ 102))) [S2.TEMTXT1 = 'Je me sentirais tellement plus libre en étant moins stressée à toute occasion. J´angoisserais moins et ça serait vraiment mieux pour moi.'];
IF (((BENSUM GE 100) AND (BENSUM LT 300) AND (QBEN1 EQ 0) AND (QBEN2 EQ 0) AND (QBEN3 EQ 103)) OR ((BENSUM GE 300) AND (Q.3PBEN1 EQ 0) AND (Q.3PBEN2 EQ 0) AND (Q.3PBEN3 EQ 103))) [S2.TEMTXT1 = 'Je sais que faire régulièrement des activités physiques m´aiderait à gérer mon poids et je serais vraiment contente de moi de réussir ça.'];
IF (((BENSUM GE 100) AND (BENSUM LT 300) AND (QBEN1 EQ 0) AND (QBEN2 EQ 0) AND (QBEN3 EQ 0) AND (QBEN4 EQ 104)) OR ((BENSUM GE 300) AND (Q.3PBEN1 EQ 0) AND (Q.3PBEN2 EQ 0) AND (Q.3PBEN3 EQ 0) AND (Q.3PBEN4 EQ 104))) [S2.TEMTXT1 = 'Ça me ferait vraiment du bien de voir plus de gens, comme si je m´inscrivais à des cours en groupe. Ça m´aiderait aussi à être plus en confiance avec les autres et ça j´aimerais ça aussi.'];
IF (((BENSUM GE 100) AND (BENSUM LT 300) AND (QBEN1 EQ 0) AND (QBEN2 EQ 0) AND (QBEN3 EQ 0) AND (QBEN4 EQ 0) AND (QBEN5 EQ 105)) OR ((BENSUM GE 300) AND (Q.3PBEN1 EQ 0) AND (Q.3PBEN2 EQ 0) AND (Q.3PBEN3 EQ 0) AND (Q.3PBEN4 EQ 0) AND (Q.3PBEN5 EQ 105))) [S2.TEMTXT1 = 'Je me sentirais bien, en santé. Je me sentirais fier de prendre soin de moi et de savoir que je fais la bonne chose.'];
IF (((BENSUM GE 100) AND (BENSUM LT 300) AND (QBEN1 EQ 0) AND (QBEN2 EQ 0) AND (QBEN3 EQ 0) AND (QBEN4 EQ 0) AND (QBEN5 EQ 0) AND (QBEN6 EQ 106)) OR ((BENSUM GE 300) AND (Q.3PBEN1 EQ 0) AND (Q.3PBEN2 EQ 0) AND (Q.3PBEN3 EQ 0) AND (Q.3PBEN4 EQ 0) AND (Q.3PBEN5 EQ 0) AND (Q.3PBEN6 EQ 106))) [S2.TEMTXT1 = 'Je me sentirais plus à l´aise avec moi-même, et avec les autres. Ça m´aiderait à être encore plus heureuse.'];

IF (((BENSUM GE 100) AND (BENSUM LT 300) AND (QBEN1 EQ 0) AND (QBEN2 EQ 0) AND (QBEN3 EQ 0) AND (QBEN4 EQ 0) AND (QBEN5 EQ 0) AND (QBEN6 EQ 0) AND (QBEN7 EQ 107)) OR ((BENSUM GE 300) AND (Q.3PBEN1 EQ 0) AND (Q.3PBEN2 EQ 0) AND (Q.3PBEN3 EQ 0) AND (Q.3PBEN4 EQ 0) AND (Q.3PBEN5 EQ 0) AND (Q.3PBEN6 EQ 0) AND (Q.3PBEN7 EQ 107))) [S2.TEMTXT1 = 'J´éprouverais un sentiment de bien-être constant qui me manque pour l´instant. Je serais juste mieux, et ça me ferait vraiment du bien!'];
IF (((BENSUM GE 100) AND (BENSUM LT 300) AND (QBEN1 EQ 0) AND (QBEN2 EQ 0) AND (QBEN3 EQ 0) AND (QBEN4 EQ 0) AND (QBEN5 EQ 0) AND (QBEN6 EQ 0) AND (QBEN7 EQ 0) AND (QBEN8 EQ 108)) OR ((BENSUM GE 300) AND (Q.3PBEN1 EQ 0) AND (Q.3PBEN2 EQ 0) AND (Q.3PBEN3 EQ 0) AND (Q.3PBEN4 EQ 0) AND (Q.3PBEN5 EQ 0) AND (Q.3PBEN6 EQ 0) AND (Q.3PBEN7 EQ 0) AND (Q.3PBEN8 EQ 108))) [S2.TEMTXT1 = 'Je me sentirais beaucoup mieux et beaucoup plus énergique dans la vie de tous les jours. Ça m´aiderait à offrir la meilleure de moi aux autres, et ça j´aimerais ça.'];
IF (((BENSUM GE 100) AND (BENSUM LT 300) AND (QBEN1 EQ 101) AND (QBEN2 EQ 102)) OR ((BENSUM GE 300) AND (Q.3PBEN1 EQ 101) AND (Q.3PBEN2 EQ 102))) [S2.TEMTXT2 = 'Je me sentirais tellement plus libre en étant moins stressée en tout temps. J´angoisserais moins et ça serait vraiment mieux pour moi.'];
IF (((BENSUM GE 100) AND (BENSUM LT 300) AND ((QBEN1 EQ 101) OR (QBEN2 EQ 102)) AND (QBEN3 EQ 103)) OR ((BENSUM GE 300) AND ((Q.3PBEN1 EQ 101) OR (Q.3PBEN2 EQ 102)) AND (Q.3PBEN3 EQ 103))) [S2.TEMTXT2 = 'Je sais que faire régulièrement des activités physiques m´aiderait à gérer mon poids et je serais vraiment contente de moi de réussir ça.'];
IF (((BENSUM GE 100) AND (BENSUM LT 300) AND ((QBEN1 EQ 101) OR (QBEN2 EQ 102) OR (QBEN3 EQ 103)) AND (QBEN4 EQ 104)) OR ((BENSUM GE 300) AND ((Q.3PBEN1 EQ 101) OR (Q.3PBEN2 EQ 102) OR (Q.3PBEN3 EQ 103)) AND (Q.3PBEN4 EQ 104))) [S2.TEMTXT2 = 'Ça me ferait vraiment du bien de voir plus de gens, comme si je m´inscrivais à des cours en groupe. Ça m´aiderait aussi à être plus en confiance avec les autres et ça j´aimerais ça aussi.'];
IF (((BENSUM GE 100) AND (BENSUM LT 300) AND ((QBEN1 EQ 101) OR (QBEN2 EQ 102) OR (QBEN3 EQ 103) OR (QBEN4 EQ 104)) AND (QBEN5 EQ 105)) OR ((BENSUM GE 300) AND ((Q.3PBEN1 EQ 101) OR (Q.3PBEN2 EQ 102) OR (Q.3PBEN3 EQ 103) OR (Q.3PBEN4 EQ 104)) AND (Q.3PBEN5 EQ 105))) [S2.TEMTXT2 = 'Je me sentirais bien, en santé. Je me sentirais fier de prendre soin de moi et de savoir que je fais la bonne chose.'];
IF (((BENSUM GE 100) AND (BENSUM LT 300) AND ((QBEN1 EQ 101) OR (QBEN2 EQ 102) OR (QBEN3 EQ 103) OR (QBEN4 EQ 104) OR (QBEN5 EQ 105)) AND (QBEN6 EQ 106)) OR ((BENSUM GE 300) AND ((Q.3PBEN1 EQ 101) OR (Q.3PBEN2 EQ 102) OR (Q.3PBEN3 EQ 103) OR (Q.3PBEN4 EQ 104) OR (Q.3PBEN5 EQ 105)) AND (Q.3PBEN6 EQ 106))) [S2.TEMTXT2 = 'Je me sentirais plus à l´aise avec moi-même, et avec les autres. Ça m´aiderait à être encore plus heureuse.'];
IF (((BENSUM GE 100) AND (BENSUM LT 300) AND ((QBEN1 EQ 101) OR (QBEN2 EQ 102) OR (QBEN3 EQ 103) OR (QBEN4 EQ 104) OR (QBEN5 EQ 105) OR (QBEN6 EQ 106)) AND (QBEN7 EQ 107)) OR ((BENSUM GE 300) AND ((Q.3PBEN1 EQ 101) OR (Q.3PBEN2 EQ 102) OR (Q.3PBEN3 EQ 103) OR (Q.3PBEN4 EQ 104) OR (Q.3PBEN5 EQ 105) OR (Q.3PBEN6 EQ 106)) AND (Q.3PBEN7 EQ 107))) [S2.TEMTXT2 = 'J´éprouverais un sentiment de bien-être constant qui me manque pour l´instant. Je serais juste mieux, et ça me ferait vraiment du bien!'];
IF (((BENSUM GE 100) AND (BENSUM LT 300) AND ((QBEN1 EQ 101) OR (QBEN2 EQ 102) OR (QBEN3 EQ 103) OR (QBEN4 EQ 104) OR (QBEN5 EQ 105) OR (QBEN6 EQ 106) OR (QBEN7 EQ 107)) AND (QBEN8 EQ 108)) OR ((BENSUM GE 300) AND ((Q.3PBEN1 EQ 101) AND (Q.3PBEN2 EQ 102) AND (Q.3PBEN3 EQ 103)

AND (Q.3PBEN4 EQ 104) AND (Q.3PBEN5 EQ 105) AND (Q.3PBEN6 EQ 106) AND (Q.3PBEN7 EQ 107)) AND (Q.3PBEN8 EQ 108))) [S2.TEMTXT2 = 'Je me sentirais beaucoup mieux et beaucoup plus énergique dans la vie de tous les jours. Ça m´aiderait à offrir la meilleur de moi aux autres, et ça j´aimerais ça.'];
IF (((BENSUM GE 200) AND (BENSUM LT 300) AND (S2.QELAB1 NE 0) AND (S2.QELAB2 EQ 0)) OR ((SUM.3PBEN GE 200) AND (S2.QELAB1 NE 0) AND (S2.QELAB2 EQ 0))) [S2.BENCHOICETXT = S2.ELABTXT1];
IF (((BENSUM GE 200) AND (BENSUM LT 300) AND (S2.QELAB1 EQ 0) AND (S2.QELAB2 NE 0)) OR ((SUM.3PBEN GE 200) AND (S2.QELAB1 EQ 0) AND (S2.QELAB2 NE 0))) [S2.BENCHOICETXT = S2.ELABTXT2];
IF (((BENSUM GE 200) AND (BENSUM LT 300) AND (S2.QELAB1 NE 0) AND (S2.QELAB2 EQ 0)) OR ((SUM.3PBEN GE 200) AND (S2.QELAB1 NE 0) AND (S2.QELAB2 EQ 0))) [S2.BENELABCHOICETXT = S2.QELAB1];
IF (((BENSUM GE 200) AND (BENSUM LT 300) AND (S2.QELAB1 EQ 0) AND (S2.QELAB2 NE 0)) OR ((SUM.3PBEN GE 200) AND (S2.QELAB1 EQ 0) AND (S2.QELAB2 NE 0))) [S2.BENELABCHOICETXT = S2.QELAB2];
IF (BENSUM EQ 0) [COTEDEPLUS = 'D´un autre côté, '];
IF (BENSUM GE 101) [COTEDEPLUS = 'Par-dessus tout, '];
PROGRAMMATION DE LA SÉANCE 4 COMMENCE ICI
IF ((F.AMOTIV GE 2.5) AND (F.MOTEXT LT 2.5) AND (F.MOTINTRO LT 2.5) AND (F.MOTIDEN LT 2.5) AND (F.MOTINTEG LT 2.5)) [S3.SUPRATYPE = 'Au moment de l´inscription, vous ignoriez peut-être pourquoi vous devriez pratiquer des activités physiques. Heureusement, vous avez été en mesure d´identifier des raisons personnelles de vouloir pratiquer des activités lors des premières séances.'];
IF ((F.MOTEXT GE 2.5) AND (F.MOTINTRO LT 2.5) AND (F.MOTIDEN LT 2.5) AND (F.MOTINTEG LT 2.5)) [S3.SUPRATYPE = 'Au moment de l´inscription, vous pratiquiez des activités physiques surtout en raison des gens qui vous entourent. Heureusement, vous avez été en mesure d´identifier des raisons personnelles de vouloir pratiquer des activités lors des premières séances.'];
IF ((F.MOTINTRO GE 2.5) AND (F.MOTIDEN LT 2.5) AND (F.MOTINTEG LT 2.5)) [S3.SUPRATYPE = 'Au moment de l´inscription, vous pratiquiez des activités physiques surtout pour ne pas vous sentir coupable. Heureusement, vous avez été en mesure d´identifier des raisons personnelles de pratiquer des activités physiques qui vous feront bouger plus naturellement, sans vous sentir coupable. '];
IF ((F.MOTIDEN GE 2.5) AND (F.MOTINTEG LT 2.5)) [S3.SUPRATYPE = 'Au moment de l´inscription, vous pratiquiez déjà des activités physiques pour des raisons personnellement importantes pour vous. C´est excellent!'];
IF (F.MOTINTEG GE 2.5) [S3.SUPRATYPE = 'Au moment de l´inscription, vous pratiquiez déjà des activités physiques, car cela fait partie de qui vous êtes comme personne. C´est excellent!'];
IF ((F.AMOTIV LT 2.5) AND (F.MOTEXT LT 2.5) AND (F.MOTINTRO LT 2.5) AND (F.MOTIDEN LT 2.5) AND (F.MOTINTEG LT 2.5)) [S3.SUPRATYPE = 'Au moment de l´inscription, nous n´arrivions pas à identifier pour quelles raisons vous faisiez des activités physiques. Heureusement, vous avez été en mesure d´identifier des raisons personnelles de vouloir pratiquer des activités lors des premières séances.'];
IF (S3_P_11QINFOFEEL EQ 0) [S3.INFORESINTRO = 'Voici un court résumé de votre séance d´aujourd´hui. Bravo d´avoir trouver vos propres raisons pouvant vous motiver à bouger davantage.'];
IF (S3_P_11QINFOFEEL EQ 1) [S3.INFORESINTRO = 'Merci de partager votre appréciation des informations. Il semble que vous ayez aimé les informations précédentes! Voici maintenant un court résumé de votre séance. Bravo d´avoir trouvé vos propres raisons de bouger davantage.'];

IF (S3_P_11QINFOFEEL EQ 2) [S3.INFORESINTRO = 'Mmmm...Il semble que vous n´ayez pas aimé les informations précédentes. Nous espérons sincèrement pouvoir faire mieux à l´avenir. Voici maintenant, si vous voulez bien, un court résumé de votre séance.'];
IF (S3_P_11QINFOFEEL EQ 3) [S3.INFORESINTRO = 'Mmmm...Il semble que vous ignorez quoi penser des informations précédentes. Nous espérons que cela pourra quand même vous servir. Voici maintenant, si vous voulez bien, un court résumé de votre séance.'];
IF (S3.P9QELAB1 NE 0) [S3.SUPRAELAB1.1 = 'L´activité physique régulière vous aiderait à '];
IF (S3.P9QELAB1 EQ 0) [S3.SUPRAELAB1.1 = 'Comment l´activité physique régulière vous aiderait à '];
IF (S3.P9QELAB1 NE 0) [S3.SUPRAELAB1.2 = ' de cette façon: '];
IF (S3.P9QELAB1 EQ 0) [S3.SUPRAELAB1.2 = ' ?'];
IF (S3.P9QELAB2 NE 0) [S3.SUPRAELAB2.1 = 'L´activité physique régulière vous aiderait à '];
IF (S3.P9QELAB2 EQ 0) [S3.SUPRAELAB2.1 = 'Comment l´activité physique régulière vous aiderait à '];
IF (S3.P9QELAB2 NE 0) [S3.SUPRAELAB2.2 = ' de cette façon: '];
IF (S3.P9QELAB2 EQ 0) [S3.SUPRAELAB2.2 = ' ?'];
IF (S3.P9QELAB1 NE 0) [S3.SUPRACHOICE1 = S3.P9QELAB1];
IF (S3.P9QELAB2 NE 0) [S3.SUPRACHOICE2 = S3.P9QELAB2];
IF (SEXE EQ 1) [XSETXT = 'x'];
IF (SEXE EQ 2) [XSETXT = 'se'];
[S3.SUMVAL =
S3.QVAL1+S3.QVAL2+S3.QVAL3+S3.QVAL4+S3.QVAL5+S3.QVAL6+S3.QVAL7+S3.QVAL8+S3.QVAL9+S3.QVAL_10+S3.QVAL_11+S3.QVAL_12+S3.QVAL_13+S3.QVAL_14+S3.QVAL_15+S3.QVAL_16+S3.QVAL_17+S3.QVAL_18+S3.QVAL_19+S3.QVAL_20+S3.QVAL_21+S3.QVAL_22+S3.QVAL_23+S3.QVAL_24+S3.QVAL_25+S3.QVAL_26+S3.QVAL_27+S3.QVAL_28+S3.QVAL_29];
[S3.2PSUMVAL =
S3.2PQVAL1+S3.2PQVAL2+S3.2PQVAL3+S3.2PQVAL4+S3.2PQVAL5+S3.2PQVAL6+S3.2PQVAL7+S3.2PQVAL8+S3.2PQVAL9+S3.2PQVAL_10+S3.2PQVAL_11+S3.2PQVAL_12+S3.2PQVAL_13+S3.2PQVAL_14+S3.2PQVAL_15+S3.2PQVAL_16+S3.2PQVAL_17+S3.2PQVAL_18+S3.2PQVAL_19+S3.2PQVAL_20+S3.2PQVAL_21+S3.2PQVAL_22+S3.2PQVAL_23+S3.2PQVAL_24+S3.2PQVAL_25+S3.2PQVAL_26+S3.2PQVAL_27+S3.2PQVAL_28+S3.2PQVAL_29];
IF (S3.QVAL1 EQ 101) [VAL1TXT = 'L´Amitié'];
IF (S3.QVAL2 EQ 102) [VAL2TXT = 'L´Amour'];
IF (S3.QVAL3 EQ 103) [VAL3TXT = 'L´Appartenance'];
IF (S3.QVAL4 EQ 104) [VAL4TXT = 'L´Autorité'];
IF (S3.QVAL5 EQ 105) [VAL5TXT = 'L´Autonomie'];
IF (S3.QVAL6 EQ 106) [VAL6TXT = 'La Beauté'];
IF (S3.QVAL7 EQ 107) [VAL7TXT = 'La Compassion'];
IF (S3.QVAL8 EQ 108) [VAL8TXT = 'Le Compromis'];
IF (S3.QVAL9 EQ 109) [VAL9TXT = 'Le Confort'];
IF (S3.QVAL_10 EQ 110) [VAL10TXT = 'Le Courage'];
IF (S3.QVAL_11 EQ 111) [VAL11TXT = 'La Créativité'];
IF (S3.QVAL_12 EQ 112) [VAL12TXT = 'La Croissance'];
IF (S3.QVAL_13 EQ 113) [VAL13TXT = 'La Curiosité'];

```
IF (S3.QVAL_14 EQ 114) [VAL14TXT = 'L´Espoir'];
IF (S3.QVAL_15 EQ 115) [VAL15TXT = 'L´Estime de soi'];
IF (S3.QVAL_16 EQ 116) [VAL16TXT = 'La Famille'];
IF (S3.QVAL_17 EQ 117) [VAL17TXT = 'La Fiabilité'];
IF (S3.QVAL_18 EQ 118) [VAL18TXT = 'La Flexibilité'];
IF (S3.QVAL_19 EQ 119) [VAL19TXT = 'La Générosité'];
IF (S3.QVAL_20 EQ 120) [VAL20TXT = 'L´Intelligence'];
IF (S3.QVAL_21 EQ 121) [VAL21TXT = 'Le Leadership'];
IF (S3.QVAL_22 EQ 122) [VAL22TXT = 'La Liberté'];
IF (S3.QVAL_23 EQ 123) [VAL23TXT = 'La Loyauté'];
IF (S3.QVAL_24 EQ 124) [VAL24TXT = 'La Maîtrise de soi'];
IF (S3.QVAL_25 EQ 125) [VAL25TXT = 'L´Ouverture d´esprit'];
IF (S3.QVAL_26 EQ 126) [VAL26TXT = 'Le Plaisir'];
IF (S3.QVAL_27 EQ 127) [VAL27TXT = 'La Responsabilité'];
IF (S3.QVAL_28 EQ 128) [VAL28TXT = 'La Sécurité'];
IF (S3.QVAL_29 EQ 129) [VAL29TXT = 'Le Service'];
IF (((S3.SUMVAL GE 200) AND (S3.SUMVAL LT 300) AND (S3.QVAL1 EQ 101)) OR (S3.2PQVAL1 EQ 101))
[S3.VAL_1 = 'l´Amitié'];
IF (((S3.SUMVAL GE 200) AND (S3.SUMVAL LT 300) AND (S3.QVAL1 EQ 0) AND (S3.QVAL2 EQ 102)) OR
((S3.2PQVAL1 EQ 0) AND (S3.2PQVAL2 EQ 102))) [S3.VAL_1 = 'l´Amour'];
IF (((S3.SUMVAL GE 200) AND (S3.SUMVAL LT 300) AND (S3.QVAL1 EQ 0) AND (S3.QVAL2 EQ 0) AND
(S3.QVAL3 EQ 103)) OR ((S3.2PQVAL1 EQ 0) AND (S3.2PQVAL2 EQ 0) AND (S3.2PQVAL3 EQ 103))) [S3.VAL_1
= 'l´Appartenance'];
IF (((S3.SUMVAL GE 200) AND (S3.SUMVAL LT 300) AND (S3.QVAL1 EQ 0) AND (S3.QVAL2 EQ 0) AND
(S3.QVAL3 EQ 0) AND (S3.QVAL4 EQ 104)) OR ((S3.2PQVAL1 EQ 0) AND (S3.2PQVAL2 EQ 0) AND
(S3.2PQVAL3 EQ 0) AND (S3.2PQVAL4 EQ 104))) [S3.VAL_1 = 'l´Autorité'];
IF (((S3.SUMVAL GE 200) AND (S3.SUMVAL LT 300) AND (S3.QVAL1 EQ 0) AND (S3.QVAL2 EQ 0) AND
(S3.QVAL3 EQ 0) AND (S3.QVAL4 EQ 0) AND (S3.QVAL5 EQ 105)) OR ((S3.2PQVAL1 EQ 0) AND (S3.2PQVAL2
EQ 0) AND (S3.2PQVAL3 EQ 0) AND (S3.2PQVAL4 EQ 0) AND (S3.2PQVAL5 EQ 105))) [S3.VAL_1 =
'l´Autonomie'];
IF (((S3.SUMVAL GE 200) AND (S3.SUMVAL LT 300) AND (S3.QVAL1 EQ 0) AND (S3.QVAL2 EQ 0) AND
(S3.QVAL3 EQ 0) AND (S3.QVAL4 EQ 0) AND (S3.QVAL5 EQ 0) AND (S3.QVAL6 EQ 106)) OR ((S3.2PQVAL1 EQ
0) AND (S3.2PQVAL2 EQ 0) AND (S3.2PQVAL3 EQ 0) AND (S3.2PQVAL4 EQ 0) AND (S3.2PQVAL5 EQ 0) AND
(S3.2PQVAL6 EQ 106))) [S3.VAL_1 = 'la Beauté'];
IF (((S3.SUMVAL GE 200) AND (S3.SUMVAL LT 300) AND (S3.QVAL1 EQ 0) AND (S3.QVAL2 EQ 0) AND
(S3.QVAL3 EQ 0) AND (S3.QVAL4 EQ 0) AND (S3.QVAL5 EQ 0) AND (S3.QVAL6 EQ 0) AND (S3.QVAL7 EQ
107)) OR ((S3.2PQVAL1 EQ 0) AND (S3.2PQVAL2 EQ 0) AND (S3.2PQVAL3 EQ 0) AND (S3.2PQVAL4 EQ 0)
AND (S3.2PQVAL5 EQ 0) AND (S3.2PQVAL6 EQ 0) AND (S3.2PQVAL7 EQ 107))) [S3.VAL_1 = 'la Compassion'];
IF (((S3.SUMVAL GE 200) AND (S3.SUMVAL LT 300) AND (S3.QVAL1 EQ 0) AND (S3.QVAL2 EQ 0) AND
(S3.QVAL3 EQ 0) AND (S3.QVAL4 EQ 0) AND (S3.QVAL5 EQ 0) AND (S3.QVAL6 EQ 0) AND (S3.QVAL7 EQ 0)
AND (S3.QVAL8 EQ 108)) OR ((S3.2PQVAL1 EQ 0) AND (S3.2PQVAL2 EQ 0) AND (S3.2PQVAL3 EQ 0) AND
```

(S3.2PQVAL4 EQ 0) AND (S3.2PQVAL5 EQ 0) AND (S3.2PQVAL6 EQ 0) AND (S3.2PQVAL7 EQ 0) AND (S3.2PQVAL8 EQ 108))) [S3.VAL_1 = 'le Compromis'];
IF (((S3.SUMVAL GE 200) AND (S3.SUMVAL LT 300) AND (S3.QVAL1 EQ 0) AND (S3.QVAL2 EQ 0) AND (S3.QVAL3 EQ 0) AND (S3.QVAL4 EQ 0) AND (S3.QVAL5 EQ 0) AND (S3.QVAL6 EQ 0) AND (S3.QVAL7 EQ 0) AND (S3.QVAL8 EQ 0) AND (S3.QVAL9 EQ 109)) OR ((S3.2PQVAL1 EQ 0) AND (S3.2PQVAL2 EQ 0) AND (S3.2PQVAL3 EQ 0) AND (S3.2PQVAL4 EQ 0) AND (S3.2PQVAL5 EQ 0) AND (S3.2PQVAL6 EQ 0) AND (S3.2PQVAL7 EQ 0) AND (S3.2PQVAL8 EQ 0) AND (S3.2PQVAL9 EQ 109))) [S3.VAL_1 = 'le Confort'];
IF (((S3.SUMVAL GE 200) AND (S3.SUMVAL LT 300) AND (S3.QVAL1 EQ 0) AND (S3.QVAL2 EQ 0) AND (S3.QVAL3 EQ 0) AND (S3.QVAL4 EQ 0) AND (S3.QVAL5 EQ 0) AND (S3.QVAL6 EQ 0) AND (S3.QVAL7 EQ 0) AND (S3.QVAL8 EQ 0) AND (S3.QVAL9 EQ 0) AND (S3.QVAL_10 EQ 101.1)) OR ((S3.2PQVAL1 EQ 0) AND (S3.2PQVAL2 EQ 0) AND (S3.2PQVAL3 EQ 0) AND (S3.2PQVAL4 EQ 0) AND (S3.2PQVAL5 EQ 0) AND (S3.2PQVAL6 EQ 0) AND (S3.2PQVAL7 EQ 0) AND (S3.2PQVAL8 EQ 0) AND (S3.2PQVAL9 EQ 0) AND (S3.2PQVAL_10 EQ 110))) [S3.VAL_1 = 'le Courage'];
IF (((S3.SUMVAL GE 200) AND (S3.SUMVAL LT 300) AND (S3.QVAL1 EQ 0) AND (S3.QVAL2 EQ 0) AND (S3.QVAL3 EQ 0) AND (S3.QVAL4 EQ 0) AND (S3.QVAL5 EQ 0) AND (S3.QVAL6 EQ 0) AND (S3.QVAL7 EQ 0) AND (S3.QVAL8 EQ 0) AND (S3.QVAL9 EQ 0) AND (S3.QVAL_10 EQ 0) AND (S3.QVAL_11 EQ 101.2)) OR ((S3.2PQVAL1 EQ 0) AND (S3.2PQVAL2 EQ 0) AND (S3.2PQVAL3 EQ 0) AND (S3.2PQVAL4 EQ 0) AND (S3.2PQVAL5 EQ 0) AND (S3.2PQVAL6 EQ 0) AND (S3.2PQVAL7 EQ 0) AND (S3.2PQVAL8 EQ 0) AND (S3.2PQVAL9 EQ 0) AND (S3.2PQVAL_10 EQ 0) AND (S3.2PQVAL_11 EQ 111))) [S3.VAL_1 = 'la Créativité'];
IF (((S3.SUMVAL GE 200) AND (S3.SUMVAL LT 300) AND (S3.QVAL1 EQ 0) AND (S3.QVAL2 EQ 0) AND (S3.QVAL3 EQ 0) AND (S3.QVAL4 EQ 0) AND (S3.QVAL5 EQ 0) AND (S3.QVAL6 EQ 0) AND (S3.QVAL7 EQ 0) AND (S3.QVAL8 EQ 0) AND (S3.QVAL9 EQ 0) AND (S3.QVAL_10 EQ 0) AND (S3.QVAL_11 EQ 0) AND (S3.QVAL_12 EQ 101.3)) OR ((S3.2PQVAL1 EQ 0) AND (S3.2PQVAL2 EQ 0) AND (S3.2PQVAL3 EQ 0) AND (S3.2PQVAL4 EQ 0) AND (S3.2PQVAL5 EQ 0) AND (S3.2PQVAL6 EQ 0) AND (S3.2PQVAL7 EQ 0) AND (S3.2PQVAL8 EQ 0) AND (S3.2PQVAL9 EQ 0) AND (S3.2PQVAL_10 EQ 0) AND (S3.2PQVAL_11 EQ 0) AND (S3.2PQVAL_12 EQ 112))) [S3.VAL_1 = 'la Croissance'];
IF (((S3.SUMVAL GE 200) AND (S3.SUMVAL LT 300) AND (S3.QVAL1 EQ 0) AND (S3.QVAL2 EQ 0) AND (S3.QVAL3 EQ 0) AND (S3.QVAL4 EQ 0) AND (S3.QVAL5 EQ 0) AND (S3.QVAL6 EQ 0) AND (S3.QVAL7 EQ 0) AND (S3.QVAL8 EQ 0) AND (S3.QVAL9 EQ 0) AND (S3.QVAL_10 EQ 0) AND (S3.QVAL_11 EQ 0) AND (S3.QVAL_12 EQ 0) AND (S3.QVAL_13 EQ 101.4)) OR ((S3.2PQVAL1 EQ 0) AND (S3.2PQVAL2 EQ 0) AND (S3.2PQVAL3 EQ 0) AND (S3.2PQVAL4 EQ 0) AND (S3.2PQVAL5 EQ 0) AND (S3.2PQVAL6 EQ 0) AND (S3.2PQVAL7 EQ 0) AND (S3.2PQVAL8 EQ 0) AND (S3.2PQVAL9 EQ 0) AND (S3.2PQVAL_10 EQ 0) AND (S3.2PQVAL_11 EQ 0) AND (S3.2PQVAL_12 EQ 0) AND (S3.2PQVAL_13 EQ 113))) [S3.VAL_1 = 'la Curiosité'];
IF (((S3.SUMVAL GE 200) AND (S3.SUMVAL LT 300) AND (S3.QVAL1 EQ 0) AND (S3.QVAL2 EQ 0) AND (S3.QVAL3 EQ 0) AND (S3.QVAL4 EQ 0) AND (S3.QVAL5 EQ 0) AND (S3.QVAL6 EQ 0) AND (S3.QVAL7 EQ 0) AND (S3.QVAL8 EQ 0) AND (S3.QVAL9 EQ 0) AND (S3.QVAL_10 EQ 0) AND (S3.QVAL_11 EQ 0) AND (S3.QVAL_12 EQ 0) AND (S3.QVAL_13 EQ 0) AND (S3.QVAL_14 EQ 101.5)) OR ((S3.2PQVAL1 EQ 0) AND (S3.2PQVAL2 EQ 0) AND (S3.2PQVAL3 EQ 0) AND (S3.2PQVAL4 EQ 0) AND (S3.2PQVAL5 EQ 0) AND (S3.2PQVAL6 EQ 0) AND (S3.2PQVAL7 EQ 0) AND (S3.2PQVAL8 EQ 0) AND (S3.2PQVAL9 EQ 0) AND (S3.2PQVAL_10 EQ 0) AND (S3.2PQVAL_11 EQ 0) AND (S3.2PQVAL_12 EQ 0) AND (S3.2PQVAL_13 EQ 0) AND (S3.2PQVAL_14 EQ 114))) [S3.VAL_1 = 'l´Espoir'];

IF (((S3.SUMVAL GE 200) AND (S3.SUMVAL LT 300) AND (S3.QVAL1 EQ 0) AND (S3.QVAL2 EQ 0) AND (S3.QVAL3 EQ 0) AND (S3.QVAL4 EQ 0) AND (S3.QVAL5 EQ 0) AND (S3.QVAL6 EQ 0) AND (S3.QVAL7 EQ 0) AND (S3.QVAL8 EQ 0) AND (S3.QVAL9 EQ 0) AND (S3.QVAL_10 EQ 0) AND (S3.QVAL_11 EQ 0) AND (S3.QVAL_12 EQ 0) AND (S3.QVAL_13 EQ 0) AND (S3.QVAL_14 EQ 0) AND (S3.QVAL_15 EQ 101.6)) OR ((S3.2PQVAL1 EQ 0) AND (S3.2PQVAL2 EQ 0) AND (S3.2PQVAL3 EQ 0) AND (S3.2PQVAL4 EQ 0) AND (S3.2PQVAL5 EQ 0) AND (S3.2PQVAL6 EQ 0) AND (S3.2PQVAL7 EQ 0) AND (S3.2PQVAL8 EQ 0) AND (S3.2PQVAL9 EQ 0) AND (S3.2PQVAL_10 EQ 0) AND (S3.2PQVAL_11 EQ 0) AND (S3.2PQVAL_12 EQ 0) AND (S3.2PQVAL_13 EQ 0) AND (S3.2PQVAL_14 EQ 0) AND (S3.2PQVAL_15 EQ 115))) [S3.VAL_1 = 'l´Estime de soi'];
IF (((S3.SUMVAL GE 200) AND (S3.SUMVAL LT 300) AND (S3.QVAL1 EQ 0) AND (S3.QVAL2 EQ 0) AND (S3.QVAL3 EQ 0) AND (S3.QVAL4 EQ 0) AND (S3.QVAL5 EQ 0) AND (S3.QVAL6 EQ 0) AND (S3.QVAL7 EQ 0) AND (S3.QVAL8 EQ 0) AND (S3.QVAL9 EQ 0) AND (S3.QVAL_10 EQ 0) AND (S3.QVAL_11 EQ 0) AND (S3.QVAL_12 EQ 0) AND (S3.QVAL_13 EQ 0) AND (S3.QVAL_14 EQ 0) AND (S3.QVAL_15 EQ 0) AND (S3.QVAL_16 EQ 101.7)) OR ((S3.2PQVAL1 EQ 0) AND (S3.2PQVAL2 EQ 0) AND (S3.2PQVAL3 EQ 0) AND (S3.2PQVAL4 EQ 0) AND (S3.2PQVAL5 EQ 0) AND (S3.2PQVAL6 EQ 0) AND (S3.2PQVAL7 EQ 0) AND (S3.2PQVAL8 EQ 0) AND (S3.2PQVAL9 EQ 0) AND (S3.2PQVAL_10 EQ 0) AND (S3.2PQVAL_11 EQ 0) AND (S3.2PQVAL_12 EQ 0) AND (S3.2PQVAL_13 EQ 0) AND (S3.2PQVAL_14 EQ 0) AND (S3.2PQVAL_15 EQ 0) AND (S3.2PQVAL_16 EQ 116))) [S3.VAL_1 = 'la Famille'];
IF (((S3.SUMVAL GE 200) AND (S3.SUMVAL LT 300) AND (S3.QVAL1 EQ 0) AND (S3.QVAL2 EQ 0) AND (S3.QVAL3 EQ 0) AND (S3.QVAL4 EQ 0) AND (S3.QVAL5 EQ 0) AND (S3.QVAL6 EQ 0) AND (S3.QVAL7 EQ 0) AND (S3.QVAL8 EQ 0) AND (S3.QVAL9 EQ 0) AND (S3.QVAL_10 EQ 0) AND (S3.QVAL_11 EQ 0) AND (S3.QVAL_12 EQ 0) AND (S3.QVAL_13 EQ 0) AND (S3.QVAL_14 EQ 0) AND (S3.QVAL_15 EQ 0) AND (S3.QVAL_16 EQ 0) AND (S3.QVAL_17 EQ 101.8)) OR ((S3.2PQVAL1 EQ 0) AND (S3.2PQVAL2 EQ 0) AND (S3.2PQVAL3 EQ 0) AND (S3.2PQVAL4 EQ 0) AND (S3.2PQVAL5 EQ 0) AND (S3.2PQVAL6 EQ 0) AND (S3.2PQVAL7 EQ 0) AND (S3.2PQVAL8 EQ 0) AND (S3.2PQVAL9 EQ 0) AND (S3.2PQVAL_10 EQ 0) AND (S3.2PQVAL_11 EQ 0) AND (S3.2PQVAL_12 EQ 0) AND (S3.2PQVAL_13 EQ 0) AND (S3.2PQVAL_14 EQ 0) AND (S3.2PQVAL_15 EQ 0) AND (S3.2PQVAL_16 EQ 0) AND (S3.2PQVAL_17 EQ 117))) [S3.VAL_1 = 'la Fiabilité'];
IF (((S3.SUMVAL GE 200) AND (S3.SUMVAL LT 300) AND (S3.QVAL1 EQ 0) AND (S3.QVAL2 EQ 0) AND (S3.QVAL3 EQ 0) AND (S3.QVAL4 EQ 0) AND (S3.QVAL5 EQ 0) AND (S3.QVAL6 EQ 0) AND (S3.QVAL7 EQ 0) AND (S3.QVAL8 EQ 0) AND (S3.QVAL9 EQ 0) AND (S3.QVAL_10 EQ 0) AND (S3.QVAL_11 EQ 0) AND (S3.QVAL_12 EQ 0) AND (S3.QVAL_13 EQ 0) AND (S3.QVAL_14 EQ 0) AND (S3.QVAL_15 EQ 0) AND (S3.QVAL_16 EQ 0) AND (S3.QVAL_17 EQ 0) AND (S3.QVAL_18 EQ 101.9)) OR ((S3.2PQVAL1 EQ 0) AND (S3.2PQVAL2 EQ 0) AND (S3.2PQVAL3 EQ 0) AND (S3.2PQVAL4 EQ 0) AND (S3.2PQVAL5 EQ 0) AND (S3.2PQVAL6 EQ 0) AND (S3.2PQVAL7 EQ 0) AND (S3.2PQVAL8 EQ 0) AND (S3.2PQVAL9 EQ 0) AND (S3.2PQVAL_10 EQ 0) AND (S3.2PQVAL_11 EQ 0) AND (S3.2PQVAL_12 EQ 0) AND (S3.2PQVAL_13 EQ 0) AND (S3.2PQVAL_14 EQ 0) AND (S3.2PQVAL_15 EQ 0) AND (S3.2PQVAL_16 EQ 0) AND (S3.2PQVAL_17 EQ 0) AND (S3.2PQVAL_18 EQ 118))) [S3.VAL_1 = 'la Flexibilité'];
IF (((S3.SUMVAL GE 200) AND (S3.SUMVAL LT 300) AND (S3.QVAL1 EQ 0) AND (S3.QVAL2 EQ 0) AND (S3.QVAL3 EQ 0) AND (S3.QVAL4 EQ 0) AND (S3.QVAL5 EQ 0) AND (S3.QVAL6 EQ 0) AND (S3.QVAL7 EQ 0) AND (S3.QVAL8 EQ 0) AND (S3.QVAL9 EQ 0) AND (S3.QVAL_10 EQ 0) AND (S3.QVAL_11 EQ 0) AND (S3.QVAL_12 EQ 0) AND (S3.QVAL_13 EQ 0) AND (S3.QVAL_14 EQ 0) AND (S3.QVAL_15 EQ 0) AND

(S3.QVAL_16 EQ 0) AND (S3.QVAL_17 EQ 0) AND (S3.QVAL_18 EQ 0) AND (S3.QVAL_19 EQ 101.19)) OR ((S3.2PQVAL1 EQ 0) AND (S3.2PQVAL2 EQ 0) AND (S3.2PQVAL3 EQ 0) AND (S3.2PQVAL4 EQ 0) AND (S3.2PQVAL5 EQ 0) AND (S3.2PQVAL6 EQ 0) AND (S3.2PQVAL7 EQ 0) AND (S3.2PQVAL8 EQ 0) AND (S3.2PQVAL9 EQ 0) AND (S3.2PQVAL_10 EQ 0) AND (S3.2PQVAL_11 EQ 0) AND (S3.2PQVAL_12 EQ 0) AND (S3.2PQVAL_13 EQ 0) AND (S3.2PQVAL_14 EQ 0) AND (S3.2PQVAL_15 EQ 0) AND (S3.2PQVAL_16 EQ 0) AND (S3.2PQVAL_17 EQ 0) AND (S3.2PQVAL_18 EQ 0) AND (S3.2PQVAL_19 EQ 119))) [S3.VAL_1 = 'la Générosité'];
IF (((S3.SUMVAL GE 200) AND (S3.SUMVAL LT 300) AND (S3.QVAL1 EQ 0) AND (S3.QVAL2 EQ 0) AND (S3.QVAL3 EQ 0) AND (S3.QVAL4 EQ 0) AND (S3.QVAL5 EQ 0) AND (S3.QVAL6 EQ 0) AND (S3.QVAL7 EQ 0) AND (S3.QVAL8 EQ 0) AND (S3.QVAL9 EQ 0) AND (S3.QVAL_10 EQ 0) AND (S3.QVAL_11 EQ 0) AND (S3.QVAL_12 EQ 0) AND (S3.QVAL_13 EQ 0) AND (S3.QVAL_14 EQ 0) AND (S3.QVAL_15 EQ 0) AND (S3.QVAL_16 EQ 0) AND (S3.QVAL_17 EQ 0) AND (S3.QVAL_18 EQ 0) AND (S3.QVAL_19 EQ 0) AND (S3.QVAL_20 EQ 102.1)) OR ((S3.2PQVAL1 EQ 0) AND (S3.2PQVAL2 EQ 0) AND (S3.2PQVAL3 EQ 0) AND (S3.2PQVAL4 EQ 0) AND (S3.2PQVAL5 EQ 0) AND (S3.2PQVAL6 EQ 0) AND (S3.2PQVAL7 EQ 0) AND (S3.2PQVAL8 EQ 0) AND (S3.2PQVAL9 EQ 0) AND (S3.2PQVAL_10 EQ 0) AND (S3.2PQVAL_11 EQ 0) AND (S3.2PQVAL_12 EQ 0) AND (S3.2PQVAL_13 EQ 0) AND (S3.2PQVAL_14 EQ 0) AND (S3.2PQVAL_15 EQ 0) AND (S3.2PQVAL_16 EQ 0) AND (S3.2PQVAL_17 EQ 0) AND (S3.2PQVAL_18 EQ 0) AND (S3.2PQVAL_19 EQ 0) AND (S3.2PQVAL_20 EQ 120))) [S3.VAL_1 = 'l´Intelligence'];
IF (((S3.SUMVAL GE 200) AND (S3.SUMVAL LT 300) AND (S3.QVAL1 EQ 0) AND (S3.QVAL2 EQ 0) AND (S3.QVAL3 EQ 0) AND (S3.QVAL4 EQ 0) AND (S3.QVAL5 EQ 0) AND (S3.QVAL6 EQ 0) AND (S3.QVAL7 EQ 0) AND (S3.QVAL8 EQ 0) AND (S3.QVAL9 EQ 0) AND (S3.QVAL_10 EQ 0) AND (S3.QVAL_11 EQ 0) AND (S3.QVAL_12 EQ 0) AND (S3.QVAL_13 EQ 0) AND (S3.QVAL_14 EQ 0) AND (S3.QVAL_15 EQ 0) AND (S3.QVAL_16 EQ 0) AND (S3.QVAL_17 EQ 0) AND (S3.QVAL_18 EQ 0) AND (S3.QVAL_19 EQ 0) AND (S3.QVAL_20 EQ 0) AND (S3.QVAL_21 EQ 102.2)) OR ((S3.2PQVAL1 EQ 0) AND (S3.2PQVAL2 EQ 0) AND (S3.2PQVAL3 EQ 0) AND (S3.2PQVAL4 EQ 0) AND (S3.2PQVAL5 EQ 0) AND (S3.2PQVAL6 EQ 0) AND (S3.2PQVAL7 EQ 0) AND (S3.2PQVAL8 EQ 0) AND (S3.2PQVAL9 EQ 0) AND (S3.2PQVAL_10 EQ 0) AND (S3.2PQVAL_11 EQ 0) AND (S3.2PQVAL_12 EQ 0) AND (S3.2PQVAL_13 EQ 0) AND (S3.2PQVAL_14 EQ 0) AND (S3.2PQVAL_15 EQ 0) AND (S3.2PQVAL_16 EQ 0) AND (S3.2PQVAL_17 EQ 0) AND (S3.2PQVAL_18 EQ 0) AND (S3.2PQVAL_19 EQ 0) AND (S3.2PQVAL_20 EQ 0) AND (S3.2PQVAL_21 EQ 121))) [S3.VAL_1 = 'le Leadership'];
IF (((S3.SUMVAL GE 200) AND (S3.SUMVAL LT 300) AND (S3.QVAL1 EQ 0) AND (S3.QVAL2 EQ 0) AND (S3.QVAL3 EQ 0) AND (S3.QVAL4 EQ 0) AND (S3.QVAL5 EQ 0) AND (S3.QVAL6 EQ 0) AND (S3.QVAL7 EQ 0) AND (S3.QVAL8 EQ 0) AND (S3.QVAL9 EQ 0) AND (S3.QVAL_10 EQ 0) AND (S3.QVAL_11 EQ 0) AND (S3.QVAL_12 EQ 0) AND (S3.QVAL_13 EQ 0) AND (S3.QVAL_14 EQ 0) AND (S3.QVAL_15 EQ 0) AND (S3.QVAL_16 EQ 0) AND (S3.QVAL_17 EQ 0) AND (S3.QVAL_18 EQ 0) AND (S3.QVAL_19 EQ 0) AND (S3.QVAL_20 EQ 0) AND (S3.QVAL_21 EQ 0) AND (S3.QVAL_22 EQ 102.3)) OR ((S3.2PQVAL1 EQ 0) AND (S3.2PQVAL2 EQ 0) AND (S3.2PQVAL3 EQ 0) AND (S3.2PQVAL4 EQ 0) AND (S3.2PQVAL5 EQ 0) AND (S3.2PQVAL6 EQ 0) AND (S3.2PQVAL7 EQ 0) AND (S3.2PQVAL8 EQ 0) AND (S3.2PQVAL9 EQ 0) AND (S3.2PQVAL_10 EQ 0) AND (S3.2PQVAL_11 EQ 0) AND (S3.2PQVAL_12 EQ 0) AND (S3.2PQVAL_13 EQ 0) AND (S3.2PQVAL_14 EQ 0) AND (S3.2PQVAL_15 EQ 0) AND (S3.2PQVAL_16 EQ 0) AND (S3.2PQVAL_17 EQ 0) AND (S3.2PQVAL_18 EQ 0) AND (S3.2PQVAL_19 EQ 0) AND (S3.2PQVAL_20 EQ 0) AND (S3.2PQVAL_21 EQ 0) AND (S3.2PQVAL_22 EQ 122))) [S3.VAL_1 = 'la Liberté'];

IF (((S3.SUMVAL GE 200) AND (S3.SUMVAL LT 300) AND (S3.QVAL1 EQ 0) AND (S3.QVAL2 EQ 0) AND (S3.QVAL3 EQ 0) AND (S3.QVAL4 EQ 0) AND (S3.QVAL5 EQ 0) AND (S3.QVAL6 EQ 0) AND (S3.QVAL7 EQ 0) AND (S3.QVAL8 EQ 0) AND (S3.QVAL9 EQ 0) AND (S3.QVAL_10 EQ 0) AND (S3.QVAL_11 EQ 0) AND (S3.QVAL_12 EQ 0) AND (S3.QVAL_13 EQ 0) AND (S3.QVAL_14 EQ 0) AND (S3.QVAL_15 EQ 0) AND (S3.QVAL_16 EQ 0) AND (S3.QVAL_17 EQ 0) AND (S3.QVAL_18 EQ 0) AND (S3.QVAL_19 EQ 0) AND (S3.QVAL_20 EQ 0) AND (S3.QVAL_21 EQ 0) AND (S3.QVAL_22 EQ 0) AND (S3.QVAL_23 EQ 102.4)) OR ((S3.2PQVAL1 EQ 0) AND (S3.2PQVAL2 EQ 0) AND (S3.2PQVAL3 EQ 0) AND (S3.2PQVAL4 EQ 0) AND (S3.2PQVAL5 EQ 0) AND (S3.2PQVAL6 EQ 0) AND (S3.2PQVAL7 EQ 0) AND (S3.2PQVAL8 EQ 0) AND (S3.2PQVAL9 EQ 0) AND (S3.2PQVAL_10 EQ 0) AND (S3.2PQVAL_11 EQ 0) AND (S3.2PQVAL_12 EQ 0) AND (S3.2PQVAL_13 EQ 0) AND (S3.2PQVAL_14 EQ 0) AND (S3.2PQVAL_15 EQ 0) AND (S3.2PQVAL_16 EQ 0) AND (S3.2PQVAL_17 EQ 0) AND (S3.2PQVAL_18 EQ 0) AND (S3.2PQVAL_19 EQ 0) AND (S3.2PQVAL_20 EQ 0) AND (S3.2PQVAL_21 EQ 0) AND (S3.2PQVAL_22 EQ 0) AND (S3.2PQVAL_23 EQ 123))) [S3.VAL_1 = 'la Loyauté'];
IF (((S3.SUMVAL GE 200) AND (S3.SUMVAL LT 300) AND (S3.QVAL1 EQ 0) AND (S3.QVAL2 EQ 0) AND (S3.QVAL3 EQ 0) AND (S3.QVAL4 EQ 0) AND (S3.QVAL5 EQ 0) AND (S3.QVAL6 EQ 0) AND (S3.QVAL7 EQ 0) AND (S3.QVAL8 EQ 0) AND (S3.QVAL9 EQ 0) AND (S3.QVAL_10 EQ 0) AND (S3.QVAL_11 EQ 0) AND (S3.QVAL_12 EQ 0) AND (S3.QVAL_13 EQ 0) AND (S3.QVAL_14 EQ 0) AND (S3.QVAL_15 EQ 0) AND (S3.QVAL_16 EQ 0) AND (S3.QVAL_17 EQ 0) AND (S3.QVAL_18 EQ 0) AND (S3.QVAL_19 EQ 0) AND (S3.QVAL_20 EQ 0) AND (S3.QVAL_21 EQ 0) AND (S3.QVAL_22 EQ 0) AND (S3.QVAL_23 EQ 0) AND (S3.QVAL_24 EQ 102.5)) OR ((S3.2PQVAL1 EQ 0) AND (S3.2PQVAL2 EQ 0) AND (S3.2PQVAL3 EQ 0) AND (S3.2PQVAL4 EQ 0) AND (S3.2PQVAL5 EQ 0) AND (S3.2PQVAL6 EQ 0) AND (S3.2PQVAL7 EQ 0) AND (S3.2PQVAL8 EQ 0) AND (S3.2PQVAL9 EQ 0) AND (S3.2PQVAL_10 EQ 0) AND (S3.2PQVAL_11 EQ 0) AND (S3.2PQVAL_12 EQ 0) AND (S3.2PQVAL_13 EQ 0) AND (S3.2PQVAL_14 EQ 0) AND (S3.2PQVAL_15 EQ 0) AND (S3.2PQVAL_16 EQ 0) AND (S3.2PQVAL_17 EQ 0) AND (S3.2PQVAL_18 EQ 0) AND (S3.2PQVAL_19 EQ 0) AND (S3.2PQVAL_20 EQ 0) AND (S3.2PQVAL_21 EQ 0) AND (S3.2PQVAL_22 EQ 0) AND (S3.2PQVAL_23 EQ 0) AND (S3.2PQVAL_24 EQ 124))) [S3.VAL_1 = 'la Maîtrise de soi'];
IF (((S3.SUMVAL GE 200) AND (S3.SUMVAL LT 300) AND (S3.QVAL1 EQ 0) AND (S3.QVAL2 EQ 0) AND (S3.QVAL3 EQ 0) AND (S3.QVAL4 EQ 0) AND (S3.QVAL5 EQ 0) AND (S3.QVAL6 EQ 0) AND (S3.QVAL7 EQ 0) AND (S3.QVAL8 EQ 0) AND (S3.QVAL9 EQ 0) AND (S3.QVAL_10 EQ 0) AND (S3.QVAL_11 EQ 0) AND (S3.QVAL_12 EQ 0) AND (S3.QVAL_13 EQ 0) AND (S3.QVAL_14 EQ 0) AND (S3.QVAL_15 EQ 0) AND (S3.QVAL_16 EQ 0) AND (S3.QVAL_17 EQ 0) AND (S3.QVAL_18 EQ 0) AND (S3.QVAL_19 EQ 0) AND (S3.QVAL_20 EQ 0) AND (S3.QVAL_21 EQ 0) AND (S3.QVAL_22 EQ 0) AND (S3.QVAL_23 EQ 0) AND (S3.QVAL_24 EQ 0) AND (S3.QVAL_25 EQ 102.6)) OR ((S3.2PQVAL1 EQ 0) AND (S3.2PQVAL2 EQ 0) AND (S3.2PQVAL3 EQ 0) AND (S3.2PQVAL4 EQ 0) AND (S3.2PQVAL5 EQ 0) AND (S3.2PQVAL6 EQ 0) AND (S3.2PQVAL7 EQ 0) AND (S3.2PQVAL8 EQ 0) AND (S3.2PQVAL9 EQ 0) AND (S3.2PQVAL_10 EQ 0) AND (S3.2PQVAL_11 EQ 0) AND (S3.2PQVAL_12 EQ 0) AND (S3.2PQVAL_13 EQ 0) AND (S3.2PQVAL_14 EQ 0) AND (S3.2PQVAL_15 EQ 0) AND (S3.2PQVAL_16 EQ 0) AND (S3.2PQVAL_17 EQ 0) AND (S3.2PQVAL_18 EQ 0) AND (S3.2PQVAL_19 EQ 0) AND (S3.2PQVAL_20 EQ 0) AND (S3.2PQVAL_21 EQ 0) AND (S3.2PQVAL_22 EQ 0) AND (S3.2PQVAL_23 EQ 0) AND (S3.2PQVAL_24 EQ 0) AND (S3.2PQVAL_25 EQ 125))) [S3.VAL_1 = 'l´Ouverture d´esprit'];
IF (((S3.SUMVAL GE 200) AND (S3.SUMVAL LT 300) AND (S3.QVAL1 EQ 0) AND (S3.QVAL2 EQ 0) AND (S3.QVAL3 EQ 0) AND (S3.QVAL4 EQ 0) AND (S3.QVAL5 EQ 0) AND (S3.QVAL6 EQ 0) AND (S3.QVAL7 EQ 0)

AND (S3.QVAL8 EQ 0) AND (S3.QVAL9 EQ 0) AND (S3.QVAL_10 EQ 0) AND (S3.QVAL_11 EQ 0) AND (S3.QVAL_12 EQ 0) AND (S3.QVAL_13 EQ 0) AND (S3.QVAL_14 EQ 0) AND (S3.QVAL_15 EQ 0) AND (S3.QVAL_16 EQ 0) AND (S3.QVAL_17 EQ 0) AND (S3.QVAL_18 EQ 0) AND (S3.QVAL_19 EQ 0) AND (S3.QVAL_20 EQ 0) AND (S3.QVAL_21 EQ 0) AND (S3.QVAL_22 EQ 0) AND (S3.QVAL_23 EQ 0) AND (S3.QVAL_24 EQ 0) AND (S3.QVAL_25 EQ 0) AND (S3.QVAL_26 EQ 102.7)) OR ((S3.2PQVAL1 EQ 0) AND (S3.2PQVAL2 EQ 0) AND (S3.2PQVAL3 EQ 0) AND (S3.2PQVAL4 EQ 0) AND (S3.2PQVAL5 EQ 0) AND (S3.2PQVAL6 EQ 0) AND (S3.2PQVAL7 EQ 0) AND (S3.2PQVAL8 EQ 0) AND (S3.2PQVAL9 EQ 0) AND (S3.2PQVAL_10 EQ 0) AND (S3.2PQVAL_11 EQ 0) AND (S3.2PQVAL_12 EQ 0) AND (S3.2PQVAL_13 EQ 0) AND (S3.2PQVAL_14 EQ 0) AND (S3.2PQVAL_15 EQ 0) AND (S3.2PQVAL_16 EQ 0) AND (S3.2PQVAL_17 EQ 0) AND (S3.2PQVAL_18 EQ 0) AND (S3.2PQVAL_19 EQ 0) AND (S3.2PQVAL_20 EQ 0) AND (S3.2PQVAL_21 EQ 0) AND (S3.2PQVAL_22 EQ 0) AND (S3.2PQVAL_23 EQ 0) AND (S3.2PQVAL_24 EQ 0) AND (S3.2PQVAL_25 EQ 0) AND (S3.2PQVAL_26 EQ 126))) [S3.VAL_1 = 'le Plaisir'];
IF (((S3.SUMVAL GE 200) AND (S3.SUMVAL LT 300) AND (S3.QVAL1 EQ 0) AND (S3.QVAL2 EQ 0) AND (S3.QVAL3 EQ 0) AND (S3.QVAL4 EQ 0) AND (S3.QVAL5 EQ 0) AND (S3.QVAL6 EQ 0) AND (S3.QVAL7 EQ 0) AND (S3.QVAL8 EQ 0) AND (S3.QVAL9 EQ 0) AND (S3.QVAL_10 EQ 0) AND (S3.QVAL_11 EQ 0) AND (S3.QVAL_12 EQ 0) AND (S3.QVAL_13 EQ 0) AND (S3.QVAL_14 EQ 0) AND (S3.QVAL_15 EQ 0) AND (S3.QVAL_16 EQ 0) AND (S3.QVAL_17 EQ 0) AND (S3.QVAL_18 EQ 0) AND (S3.QVAL_19 EQ 0) AND (S3.QVAL_20 EQ 0) AND (S3.QVAL_21 EQ 0) AND (S3.QVAL_22 EQ 0) AND (S3.QVAL_23 EQ 0) AND (S3.QVAL_24 EQ 0) AND (S3.QVAL_25 EQ 0) AND (S3.QVAL_26 EQ 0) AND (S3.QVAL_27 EQ 102.8)) OR ((S3.2PQVAL1 EQ 0) AND (S3.2PQVAL2 EQ 0) AND (S3.2PQVAL3 EQ 0) AND (S3.2PQVAL4 EQ 0) AND (S3.2PQVAL5 EQ 0) AND (S3.2PQVAL6 EQ 0) AND (S3.2PQVAL7 EQ 0) AND (S3.2PQVAL8 EQ 0) AND (S3.2PQVAL9 EQ 0) AND (S3.2PQVAL_10 EQ 0) AND (S3.2PQVAL_11 EQ 0) AND (S3.2PQVAL_12 EQ 0) AND (S3.2PQVAL_13 EQ 0) AND (S3.2PQVAL_14 EQ 0) AND (S3.2PQVAL_15 EQ 0) AND (S3.2PQVAL_16 EQ 0) AND (S3.2PQVAL_17 EQ 0) AND (S3.2PQVAL_18 EQ 0) AND (S3.2PQVAL_19 EQ 0) AND (S3.2PQVAL_20 EQ 0) AND (S3.2PQVAL_21 EQ 0) AND (S3.2PQVAL_22 EQ 0) AND (S3.2PQVAL_23 EQ 0) AND (S3.2PQVAL_24 EQ 0) AND (S3.2PQVAL_25 EQ 0) AND (S3.2PQVAL_26 EQ 0) AND (S3.2PQVAL_27 EQ 127))) [S3.VAL_1 = 'la Responsabilité'];
IF (((S3.SUMVAL GE 200) AND (S3.SUMVAL LT 300) AND (S3.QVAL1 EQ 0) AND (S3.QVAL2 EQ 0) AND (S3.QVAL3 EQ 0) AND (S3.QVAL4 EQ 0) AND (S3.QVAL5 EQ 0) AND (S3.QVAL6 EQ 0) AND (S3.QVAL7 EQ 0) AND (S3.QVAL8 EQ 0) AND (S3.QVAL9 EQ 0) AND (S3.QVAL_10 EQ 0) AND (S3.QVAL_11 EQ 0) AND (S3.QVAL_12 EQ 0) AND (S3.QVAL_13 EQ 0) AND (S3.QVAL_14 EQ 0) AND (S3.QVAL_15 EQ 0) AND (S3.QVAL_16 EQ 0) AND (S3.QVAL_17 EQ 0) AND (S3.QVAL_18 EQ 0) AND (S3.QVAL_19 EQ 0) AND (S3.QVAL_20 EQ 0) AND (S3.QVAL_21 EQ 0) AND (S3.QVAL_22 EQ 0) AND (S3.QVAL_23 EQ 0) AND (S3.QVAL_24 EQ 0) AND (S3.QVAL_25 EQ 0) AND (S3.QVAL_26 EQ 0) AND (S3.QVAL_27 EQ 0) AND (S3.QVAL_28 EQ 102.9)) OR ((S3.2PQVAL1 EQ 0) AND (S3.2PQVAL2 EQ 0) AND (S3.2PQVAL3 EQ 0) AND (S3.2PQVAL4 EQ 0) AND (S3.2PQVAL5 EQ 0) AND (S3.2PQVAL6 EQ 0) AND (S3.2PQVAL7 EQ 0) AND (S3.2PQVAL8 EQ 0) AND (S3.2PQVAL9 EQ 0) AND (S3.2PQVAL_10 EQ 0) AND (S3.2PQVAL_11 EQ 0) AND (S3.2PQVAL_12 EQ 0) AND (S3.2PQVAL_13 EQ 0) AND (S3.2PQVAL_14 EQ 0) AND (S3.2PQVAL_15 EQ 0) AND (S3.2PQVAL_16 EQ 0) AND (S3.2PQVAL_17 EQ 0) AND (S3.2PQVAL_18 EQ 0) AND (S3.2PQVAL_19 EQ 0) AND (S3.2PQVAL_20 EQ 0) AND (S3.2PQVAL_21 EQ 0) AND (S3.2PQVAL_22 EQ 0) AND (S3.2PQVAL_23 EQ 0) AND (S3.2PQVAL_24 EQ 0) AND (S3.2PQVAL_25 EQ 0) AND (S3.2PQVAL_26 EQ 0) AND (S3.2PQVAL_27 EQ 0) AND (S3.2PQVAL_28 EQ 128))) [S3.VAL_1 = 'la Sécurité'];

IF (((S3.SUMVAL GE 200) AND (S3.SUMVAL LT 300) AND (S3.QVAL1 EQ 0) AND (S3.QVAL2 EQ 0) AND (S3.QVAL3 EQ 0) AND (S3.QVAL4 EQ 0) AND (S3.QVAL5 EQ 0) AND (S3.QVAL6 EQ 0) AND (S3.QVAL7 EQ 0) AND (S3.QVAL8 EQ 0) AND (S3.QVAL9 EQ 0) AND (S3.QVAL_10 EQ 0) AND (S3.QVAL_11 EQ 0) AND (S3.QVAL_12 EQ 0) AND (S3.QVAL_13 EQ 0) AND (S3.QVAL_14 EQ 0) AND (S3.QVAL_15 EQ 0) AND (S3.QVAL_16 EQ 0) AND (S3.QVAL_17 EQ 0) AND (S3.QVAL_18 EQ 0) AND (S3.QVAL_19 EQ 0) AND (S3.QVAL_20 EQ 0) AND (S3.QVAL_21 EQ 0) AND (S3.QVAL_22 EQ 0) AND (S3.QVAL_23 EQ 0) AND (S3.QVAL_24 EQ 0) AND (S3.QVAL_25 EQ 0) AND (S3.QVAL_26 EQ 0) AND (S3.QVAL_27 EQ 102.8) AND (S3.QVAL_28 EQ 0) AND (S3.QVAL_29 EQ 102.29)) OR ((S3.2PQVAL1 EQ 0) AND (S3.2PQVAL2 EQ 0) AND (S3.2PQVAL3 EQ 0) AND (S3.2PQVAL4 EQ 0) AND (S3.2PQVAL5 EQ 0) AND (S3.2PQVAL6 EQ 0) AND (S3.2PQVAL7 EQ 0) AND (S3.2PQVAL8 EQ 0) AND (S3.2PQVAL9 EQ 0) AND (S3.2PQVAL_10 EQ 0) AND (S3.2PQVAL_11 EQ 0) AND (S3.2PQVAL_12 EQ 0) AND (S3.2PQVAL_13 EQ 0) AND (S3.2PQVAL_14 EQ 0) AND (S3.2PQVAL_15 EQ 0) AND (S3.2PQVAL_16 EQ 0) AND (S3.2PQVAL_17 EQ 0) AND (S3.2PQVAL_18 EQ 0) AND (S3.2PQVAL_19 EQ 0) AND (S3.2PQVAL_20 EQ 0) AND (S3.2PQVAL_21 EQ 0) AND (S3.2PQVAL_22 EQ 0) AND (S3.2PQVAL_23 EQ 0) AND (S3.2PQVAL_24 EQ 0) AND (S3.2PQVAL_25 EQ 0) AND (S3.2PQVAL_26 EQ 0) AND (S3.2PQVAL_27 EQ 0) AND (S3.2PQVAL_28 EQ 0) AND (S3.2PQVAL_29 EQ 129))) [S3.VAL_1 = 'le Service'];
IF (((S3.SUMVAL GE 200) AND (S3.SUMVAL LT 300) AND (S3.QVAL1 EQ 101) AND (S3.QVAL2 EQ 102)) OR ((S3.2PQVAL1 EQ 101) AND (S3.2PQVAL2 EQ 102))) [S3.VAL_2 = 'l´Amour'];
IF (((S3.SUMVAL GE 200) AND (S3.SUMVAL LT 300) AND ((S3.QVAL1 EQ 101) OR (S3.QVAL2 EQ 102)) AND (S3.QVAL3 EQ 103)) OR (((S3.2PQVAL1 EQ 101) OR (S3.2PQVAL2 EQ 102)) AND (S3.2PQVAL3 EQ 103))) [S3.VAL_2 = 'l´Appartenance'];
IF (((S3.SUMVAL GE 200) AND (S3.SUMVAL LT 300) AND ((S3.QVAL1 EQ 101) OR (S3.QVAL2 EQ 102) OR (S3.QVAL3 EQ 103)) AND (S3.QVAL4 EQ 104)) OR (((S3.2PQVAL1 EQ 101) OR (S3.2PQVAL2 EQ 102) OR (S3.2PQVAL3 EQ 103)) AND (S3.2PQVAL4 EQ 104))) [S3.VAL_2 = 'l´Autorité'];
IF (((S3.SUMVAL GE 200) AND (S3.SUMVAL LT 300) AND ((S3.QVAL1 EQ 101) OR (S3.QVAL2 EQ 102) OR (S3.QVAL3 EQ 103) OR (S3.QVAL4 EQ 104)) AND (S3.QVAL5 EQ 105)) OR (((S3.2PQVAL1 EQ 101) OR (S3.2PQVAL2 EQ 102) OR (S3.2PQVAL3 EQ 103) OR (S3.2PQVAL4 EQ 104)) AND (S3.2PQVAL5 EQ 105))) [S3.VAL_2 = 'l´Autonomie'];
IF (((S3.SUMVAL GE 200) AND (S3.SUMVAL LT 300) AND ((S3.QVAL1 EQ 101) OR (S3.QVAL2 EQ 102) OR (S3.QVAL3 EQ 103) OR (S3.QVAL4 EQ 104) OR (S3.QVAL5 EQ 105)) AND (S3.QVAL6 EQ 106)) OR (((S3.2PQVAL1 EQ 101) OR (S3.2PQVAL2 EQ 102) OR (S3.2PQVAL3 EQ 103) OR (S3.2PQVAL4 EQ 104) OR (S3.2PQVAL5 EQ 105)) AND (S3.2PQVAL6 EQ 106))) [S3.VAL_2 = 'la Beauté'];
IF (((S3.SUMVAL GE 200) AND (S3.SUMVAL LT 300) AND ((S3.QVAL1 EQ 101) OR (S3.QVAL2 EQ 102) OR (S3.QVAL3 EQ 103) OR (S3.QVAL4 EQ 104) OR (S3.QVAL5 EQ 105) OR (S3.QVAL6 EQ 106)) AND (S3.QVAL7 EQ 107)) OR (((S3.2PQVAL1 EQ 101) OR (S3.2PQVAL2 EQ 102) OR (S3.2PQVAL3 EQ 103) OR (S3.2PQVAL4 EQ 104) OR (S3.2PQVAL5 EQ 105) OR (S3.2PQVAL6 EQ 106)) AND (S3.2PQVAL7 EQ 107))) [S3.VAL_2 = 'la Compassion'];
IF (((S3.SUMVAL GE 200) AND (S3.SUMVAL LT 300) AND ((S3.QVAL1 EQ 101) OR (S3.QVAL2 EQ 102) OR (S3.QVAL3 EQ 103) OR (S3.QVAL4 EQ 104) OR (S3.QVAL5 EQ 105) OR (S3.QVAL6 EQ 106) OR (S3.QVAL7 EQ 107)) AND (S3.QVAL8 EQ 108)) OR (((S3.2PQVAL1 EQ 101) OR (S3.2PQVAL2 EQ 102) OR (S3.2PQVAL3 EQ 103) OR (S3.2PQVAL4 EQ 104) OR (S3.2PQVAL5 EQ 105) OR (S3.2PQVAL6 EQ 106) OR (S3.2PQVAL7 EQ 107)) AND (S3.2PQVAL8 EQ 108))) [S3.VAL_2 = 'le Compromis'];

IF (((S3.SUMVAL GE 200) AND (S3.SUMVAL LT 300) AND ((S3.QVAL1 EQ 101) OR (S3.QVAL2 EQ 102) OR (S3.QVAL3 EQ 103) OR (S3.QVAL4 EQ 104) OR (S3.QVAL5 EQ 105) OR (S3.QVAL6 EQ 106) OR (S3.QVAL7 EQ 107) OR (S3.QVAL8 EQ 108)) AND (S3.QVAL9 EQ 109)) OR (((S3.2PQVAL1 EQ 101) OR (S3.2PQVAL2 EQ 102) OR (S3.2PQVAL3 EQ 103) OR (S3.2PQVAL4 EQ 104) OR (S3.2PQVAL5 EQ 105) OR (S3.2PQVAL6 EQ 106) OR (S3.2PQVAL7 EQ 107) OR (S3.2PQVAL8 EQ 108)) AND (S3.2PQVAL9 EQ 109))) [S3.VAL_2 = 'le Confort'];
IF (((S3.SUMVAL GE 200) AND (S3.SUMVAL LT 300) AND ((S3.QVAL1 EQ 101) OR (S3.QVAL2 EQ 102) OR (S3.QVAL3 EQ 103) OR (S3.QVAL4 EQ 104) OR (S3.QVAL5 EQ 105) OR (S3.QVAL6 EQ 106) OR (S3.QVAL7 EQ 107) OR (S3.QVAL8 EQ 108) OR (S3.QVAL9 EQ 109)) AND (S3.QVAL_10 EQ 101.1)) OR (((S3.2PQVAL1 EQ 101) OR (S3.2PQVAL2 EQ 102) OR (S3.2PQVAL3 EQ 103) OR (S3.2PQVAL4 EQ 104) OR (S3.2PQVAL5 EQ 105) OR (S3.2PQVAL6 EQ 106) OR (S3.2PQVAL7 EQ 107) OR (S3.2PQVAL8 EQ 108) OR (S3.2PQVAL9 EQ 109)) AND (S3.2PQVAL_10 EQ 110))) [S3.VAL_2 = 'le Courage'];
IF (((S3.SUMVAL GE 200) AND (S3.SUMVAL LT 300) AND ((S3.QVAL1 EQ 101) OR (S3.QVAL2 EQ 102) OR (S3.QVAL3 EQ 103) OR (S3.QVAL4 EQ 104) OR (S3.QVAL5 EQ 105) OR (S3.QVAL6 EQ 106) OR (S3.QVAL7 EQ 107) OR (S3.QVAL8 EQ 108) OR (S3.QVAL9 EQ 109) OR (S3.QVAL_10 EQ 101.1)) AND (S3.QVAL_11 EQ 101.2)) OR (((S3.2PQVAL1 EQ 101) OR (S3.2PQVAL2 EQ 102) OR (S3.2PQVAL3 EQ 103) OR (S3.2PQVAL4 EQ 104) OR (S3.2PQVAL5 EQ 105) OR (S3.2PQVAL6 EQ 106) OR (S3.2PQVAL7 EQ 107) OR (S3.2PQVAL8 EQ 108) OR (S3.2PQVAL9 EQ 109) OR (S3.2PQVAL_10 EQ 110)) AND (S3.2PQVAL_11 EQ 111))) [S3.VAL_2 = 'la Créativité'];
IF (((S3.SUMVAL GE 200) AND (S3.SUMVAL LT 300) AND ((S3.QVAL1 EQ 101) OR (S3.QVAL2 EQ 102) OR (S3.QVAL3 EQ 103) OR (S3.QVAL4 EQ 104) OR (S3.QVAL5 EQ 105) OR (S3.QVAL6 EQ 106) OR (S3.QVAL7 EQ 107) OR (S3.QVAL8 EQ 108) OR (S3.QVAL9 EQ 109) OR (S3.QVAL_10 EQ 101.1) OR (S3.QVAL_11 EQ 101.2)) AND (S3.QVAL_12 EQ 101.3)) OR (((S3.2PQVAL1 EQ 101) OR (S3.2PQVAL2 EQ 102) OR (S3.2PQVAL3 EQ 103) OR (S3.2PQVAL4 EQ 104) OR (S3.2PQVAL5 EQ 105) OR (S3.2PQVAL6 EQ 106) OR (S3.2PQVAL7 EQ 107) OR (S3.2PQVAL8 EQ 108) OR (S3.2PQVAL9 EQ 109) OR (S3.2PQVAL_10 EQ 110) OR (S3.2PQVAL_11 EQ 111)) AND (S3.2PQVAL_12 EQ 112))) [S3.VAL_2 = 'la Croissance'];
IF (((S3.SUMVAL GE 200) AND (S3.SUMVAL LT 300) AND ((S3.QVAL1 EQ 101) OR (S3.QVAL2 EQ 102) OR (S3.QVAL3 EQ 103) OR (S3.QVAL4 EQ 104) OR (S3.QVAL5 EQ 105) OR (S3.QVAL6 EQ 106) OR (S3.QVAL7 EQ 107) OR (S3.QVAL8 EQ 108) OR (S3.QVAL9 EQ 109) OR (S3.QVAL_10 EQ 101.1) OR (S3.QVAL_11 EQ 101.2) OR (S3.QVAL_12 EQ 101.3)) AND (S3.QVAL_13 EQ 101.4)) OR (((S3.2PQVAL1 EQ 101) OR (S3.2PQVAL2 EQ 102) OR (S3.2PQVAL3 EQ 103) OR (S3.2PQVAL4 EQ 104) OR (S3.2PQVAL5 EQ 105) OR (S3.2PQVAL6 EQ 106) OR (S3.2PQVAL7 EQ 107) OR (S3.2PQVAL8 EQ 108) OR (S3.2PQVAL9 EQ 109) OR (S3.2PQVAL_10 EQ 110) OR (S3.2PQVAL_11 EQ 111) OR (S3.2PQVAL_12 EQ 112)) AND (S3.2PQVAL_13 EQ 113))) [S3.VAL_2 = 'la Curiosité'];
IF (((S3.SUMVAL GE 200) AND (S3.SUMVAL LT 300) AND ((S3.QVAL1 EQ 101) OR (S3.QVAL2 EQ 102) OR (S3.QVAL3 EQ 103) OR (S3.QVAL4 EQ 104) OR (S3.QVAL5 EQ 105) OR (S3.QVAL6 EQ 106) OR (S3.QVAL7 EQ 107) OR (S3.QVAL8 EQ 108) OR (S3.QVAL9 EQ 109) OR (S3.QVAL_10 EQ 101.1) OR (S3.QVAL_11 EQ 101.2) OR (S3.QVAL_12 EQ 101.3) OR (S3.QVAL_13 EQ 101.4)) AND (S3.QVAL_14 EQ 101.5)) OR (((S3.2PQVAL1 EQ 101) OR (S3.2PQVAL2 EQ 102) OR (S3.2PQVAL3 EQ 103) OR (S3.2PQVAL4 EQ 104) OR (S3.2PQVAL5 EQ 105) OR (S3.2PQVAL6 EQ 106) OR (S3.2PQVAL7 EQ 107) OR (S3.2PQVAL8 EQ 108) OR (S3.2PQVAL9 EQ 109) OR (S3.2PQVAL_10 EQ 110) OR (S3.2PQVAL_11 EQ 111) OR (S3.2PQVAL_12 EQ 112) OR (S3.2PQVAL_13 EQ 113)) AND (S3.2PQVAL_14 EQ 114))) [S3.VAL_2 = 'l´Espoir'];

IF (((S3.SUMVAL GE 200) AND (S3.SUMVAL LT 300) AND ((S3.QVAL1 EQ 101) OR (S3.QVAL2 EQ 102) OR (S3.QVAL3 EQ 103) OR (S3.QVAL4 EQ 104) OR (S3.QVAL5 EQ 105) OR (S3.QVAL6 EQ 106) OR (S3.QVAL7 EQ 107) OR (S3.QVAL8 EQ 108) OR (S3.QVAL9 EQ 109) OR (S3.QVAL_10 EQ 101.1) OR (S3.QVAL_11 EQ 101.2) OR (S3.QVAL_12 EQ 101.3) OR (S3.QVAL_13 EQ 101.4) OR (S3.QVAL_14 EQ 101.5)) AND (S3.QVAL_15 EQ 101.6)) OR (((S3.2PQVAL1 EQ 101) OR (S3.2PQVAL2 EQ 102) OR (S3.2PQVAL3 EQ 103) OR (S3.2PQVAL4 EQ 104) OR (S3.2PQVAL5 EQ 105) OR (S3.2PQVAL6 EQ 106) OR (S3.2PQVAL7 EQ 107) OR (S3.2PQVAL8 EQ 108) OR (S3.2PQVAL9 EQ 109) OR (S3.2PQVAL_10 EQ 110) OR (S3.2PQVAL_11 EQ 111) OR (S3.2PQVAL_12 EQ 112) OR (S3.2PQVAL_13 EQ 113) OR (S3.2PQVAL_14 EQ 114)) AND (S3.2PQVAL_15 EQ 115))) [S3.VAL_2 = 'l´Estime de soi'];
IF (((S3.SUMVAL GE 200) AND (S3.SUMVAL LT 300) AND ((S3.QVAL1 EQ 101) OR (S3.QVAL2 EQ 102) OR (S3.QVAL3 EQ 103) OR (S3.QVAL4 EQ 104) OR (S3.QVAL5 EQ 105) OR (S3.QVAL6 EQ 106) OR (S3.QVAL7 EQ 107) OR (S3.QVAL8 EQ 108) OR (S3.QVAL9 EQ 109) OR (S3.QVAL_10 EQ 101.1) OR (S3.QVAL_11 EQ 101.2) OR (S3.QVAL_12 EQ 101.3) OR (S3.QVAL_13 EQ 101.4) OR (S3.QVAL_14 EQ 101.5) OR (S3.QVAL_15 EQ 101.6)) AND (S3.QVAL_16 EQ 101.7)) OR (((S3.2PQVAL1 EQ 101) OR (S3.2PQVAL2 EQ 102) OR (S3.2PQVAL3 EQ 103) OR (S3.2PQVAL4 EQ 104) OR (S3.2PQVAL5 EQ 105) OR (S3.2PQVAL6 EQ 106) OR (S3.2PQVAL7 EQ 107) OR (S3.2PQVAL8 EQ 108) OR (S3.2PQVAL9 EQ 109) OR (S3.2PQVAL_10 EQ 110) OR (S3.2PQVAL_11 EQ 111) OR (S3.2PQVAL_12 EQ 112) OR (S3.2PQVAL_13 EQ 113) OR (S3.2PQVAL_14 EQ 114) OR (S3.2PQVAL_15 EQ 115)) AND (S3.2PQVAL_16 EQ 116))) [S3.VAL_2 = 'la Famille'];
IF (((S3.SUMVAL GE 200) AND (S3.SUMVAL LT 300) AND ((S3.QVAL1 EQ 101) OR (S3.QVAL2 EQ 102) OR (S3.QVAL3 EQ 103) OR (S3.QVAL4 EQ 104) OR (S3.QVAL5 EQ 105) OR (S3.QVAL6 EQ 106) OR (S3.QVAL7 EQ 107) OR (S3.QVAL8 EQ 108) OR (S3.QVAL9 EQ 109) OR (S3.QVAL_10 EQ 101.1) OR (S3.QVAL_11 EQ 101.2) OR (S3.QVAL_12 EQ 101.3) OR (S3.QVAL_13 EQ 101.4) OR (S3.QVAL_14 EQ 101.5) OR (S3.QVAL_15 EQ 101.6) OR (S3.QVAL_16 EQ 101.7)) AND (S3.QVAL_17 EQ 101.8)) OR (((S3.2PQVAL1 EQ 101) OR (S3.2PQVAL2 EQ 102) OR (S3.2PQVAL3 EQ 103) OR (S3.2PQVAL4 EQ 104) OR (S3.2PQVAL5 EQ 105) OR (S3.2PQVAL6 EQ 106) OR (S3.2PQVAL7 EQ 107) OR (S3.2PQVAL8 EQ 108) OR (S3.2PQVAL9 EQ 109) OR (S3.2PQVAL_10 EQ 110) OR (S3.2PQVAL_11 EQ 111) OR (S3.2PQVAL_12 EQ 112) OR (S3.2PQVAL_13 EQ 113) OR (S3.2PQVAL_14 EQ 114) OR (S3.2PQVAL_15 EQ 115) OR (S3.2PQVAL_16 EQ 116)) AND (S3.2PQVAL_17 EQ 117))) [S3.VAL_2 = 'la Fiabilité'];
IF (((S3.SUMVAL GE 200) AND (S3.SUMVAL LT 300) AND ((S3.QVAL1 EQ 101) OR (S3.QVAL2 EQ 102) OR (S3.QVAL3 EQ 103) OR (S3.QVAL4 EQ 104) OR (S3.QVAL5 EQ 105) OR (S3.QVAL6 EQ 106) OR (S3.QVAL7 EQ 107) OR (S3.QVAL8 EQ 108) OR (S3.QVAL9 EQ 109) OR (S3.QVAL_10 EQ 101.1) OR (S3.QVAL_11 EQ 101.2) OR (S3.QVAL_12 EQ 101.3) OR (S3.QVAL_13 EQ 101.4) OR (S3.QVAL_14 EQ 101.5) OR (S3.QVAL_15 EQ 101.6) OR (S3.QVAL_16 EQ 101.7) OR (S3.QVAL_17 EQ 101.8)) AND (S3.QVAL_18 EQ 101.9)) OR (((S3.2PQVAL1 EQ 101) OR (S3.2PQVAL2 EQ 102) OR (S3.2PQVAL3 EQ 103) OR (S3.2PQVAL4 EQ 104) OR (S3.2PQVAL5 EQ 105) OR (S3.2PQVAL6 EQ 106) OR (S3.2PQVAL7 EQ 107) OR (S3.2PQVAL8 EQ 108) OR (S3.2PQVAL9 EQ 109) OR (S3.2PQVAL_10 EQ 110) OR (S3.2PQVAL_11 EQ 111) OR (S3.2PQVAL_12 EQ 112) OR (S3.2PQVAL_13 EQ 113) OR (S3.2PQVAL_14 EQ 114) OR (S3.2PQVAL_15 EQ 115) OR (S3.2PQVAL_16 EQ 116) OR (S3.2PQVAL_17 EQ 117)) AND (S3.2PQVAL_18 EQ 118))) [S3.VAL_2 = 'la Flexibilité'];
IF (((S3.SUMVAL GE 200) AND (S3.SUMVAL LT 300) AND ((S3.QVAL1 EQ 101) OR (S3.QVAL2 EQ 102) OR (S3.QVAL3 EQ 103) OR (S3.QVAL4 EQ 104) OR (S3.QVAL5 EQ 105) OR (S3.QVAL6 EQ 106) OR (S3.QVAL7 EQ 107) OR (S3.QVAL8 EQ 108) OR (S3.QVAL9 EQ 109) OR (S3.QVAL_10 EQ 101.1) OR (S3.QVAL_11 EQ 101.2) OR (S3.QVAL_12 EQ 101.3) OR (S3.QVAL_13 EQ 101.4) OR (S3.QVAL_14 EQ 101.5) OR (S3.QVAL_15 EQ

101.6) OR (S3.QVAL_16 EQ 101.7) OR (S3.QVAL_17 EQ 101.8) OR (S3.QVAL_18 EQ 101.9)) AND (S3.QVAL_19 EQ 101.19))OR (((S3.2PQVAL1 EQ 101) OR (S3.2PQVAL2 EQ 102) OR (S3.2PQVAL3 EQ 103) OR (S3.2PQVAL4 EQ 104) OR (S3.2PQVAL5 EQ 105) OR (S3.2PQVAL6 EQ 106) OR (S3.2PQVAL7 EQ 107) OR (S3.2PQVAL8 EQ 108) OR (S3.2PQVAL9 EQ 109) OR (S3.2PQVAL_10 EQ 110) OR (S3.2PQVAL_11 EQ 111) OR (S3.2PQVAL_12 EQ 112) OR (S3.2PQVAL_13 EQ 113) OR (S3.2PQVAL_14 EQ 114) OR (S3.2PQVAL_15 EQ 115) OR (S3.2PQVAL_16 EQ 116) OR (S3.2PQVAL_17 EQ 117) OR (S3.2PQVAL_18 EQ 118)) AND (S3.2PQVAL_19 EQ 119))) [S3.VAL_2 = 'la Générosité'];
IF (((S3.SUMVAL GE 200) AND (S3.SUMVAL LT 300) AND ((S3.QVAL1 EQ 101) OR (S3.QVAL2 EQ 102) OR (S3.QVAL3 EQ 103) OR (S3.QVAL4 EQ 104) OR (S3.QVAL5 EQ 105) OR (S3.QVAL6 EQ 106) OR (S3.QVAL7 EQ 107) OR (S3.QVAL8 EQ 108) OR (S3.QVAL9 EQ 109) OR (S3.QVAL_10 EQ 101.1) OR (S3.QVAL_11 EQ 101.2) OR (S3.QVAL_12 EQ 101.3) OR (S3.QVAL_13 EQ 101.4) OR (S3.QVAL_14 EQ 101.5) OR (S3.QVAL_15 EQ 101.6) OR (S3.QVAL_16 EQ 101.7) OR (S3.QVAL_17 EQ 101.8) OR (S3.QVAL_18 EQ 101.9) OR (S3.QVAL_19 EQ 101.19)) AND (S3.QVAL_20 EQ 102.1)) OR (((S3.2PQVAL1 EQ 101) OR (S3.2PQVAL2 EQ 102) OR (S3.2PQVAL3 EQ 103) OR (S3.2PQVAL4 EQ 104) OR (S3.2PQVAL5 EQ 105) OR (S3.2PQVAL6 EQ 106) OR (S3.2PQVAL7 EQ 107) OR (S3.2PQVAL8 EQ 108) OR (S3.2PQVAL9 EQ 109) OR (S3.2PQVAL_10 EQ 110) OR (S3.2PQVAL_11 EQ 111) OR (S3.2PQVAL_12 EQ 112) OR (S3.2PQVAL_13 EQ 113) OR (S3.2PQVAL_14 EQ 114) OR (S3.2PQVAL_15 EQ 115) OR (S3.2PQVAL_16 EQ 116) OR (S3.2PQVAL_17 EQ 117) OR (S3.2PQVAL_18 EQ 118) OR (S3.2PQVAL_19 EQ 119)) AND (S3.2PQVAL_20 EQ 120))) [S3.VAL_2 = 'l´Intelligence'];
IF (((S3.SUMVAL GE 200) AND (S3.SUMVAL LT 300) AND ((S3.QVAL1 EQ 101) OR (S3.QVAL2 EQ 102) OR (S3.QVAL3 EQ 103) OR (S3.QVAL4 EQ 104) OR (S3.QVAL5 EQ 105) OR (S3.QVAL6 EQ 106) OR (S3.QVAL7 EQ 107) OR (S3.QVAL8 EQ 108) OR (S3.QVAL9 EQ 109) OR (S3.QVAL_10 EQ 101.1) OR (S3.QVAL_11 EQ 101.2) OR (S3.QVAL_12 EQ 101.3) OR (S3.QVAL_13 EQ 101.4) OR (S3.QVAL_14 EQ 101.5) OR (S3.QVAL_15 EQ 101.6) OR (S3.QVAL_16 EQ 101.7) OR (S3.QVAL_17 EQ 101.8) OR (S3.QVAL_18 EQ 101.9) OR (S3.QVAL_19 EQ 101.19) OR (S3.QVAL_20 EQ 102.1)) AND (S3.QVAL_21 EQ 102.2)) OR (((S3.2PQVAL1 EQ 101) OR (S3.2PQVAL2 EQ 102) OR (S3.2PQVAL3 EQ 103) OR (S3.2PQVAL4 EQ 104) OR (S3.2PQVAL5 EQ 105) OR (S3.2PQVAL6 EQ 106) OR (S3.2PQVAL7 EQ 107) OR (S3.2PQVAL8 EQ 108) OR (S3.2PQVAL9 EQ 109) OR (S3.2PQVAL_10 EQ 110) OR (S3.2PQVAL_11 EQ 111) OR (S3.2PQVAL_12 EQ 112) OR (S3.2PQVAL_13 EQ 113) OR (S3.2PQVAL_14 EQ 114) OR (S3.2PQVAL_15 EQ 115) OR (S3.2PQVAL_16 EQ 116) OR (S3.2PQVAL_17 EQ 117) OR (S3.2PQVAL_18 EQ 118) OR (S3.2PQVAL_19 EQ 119) OR (S3.2PQVAL_20 EQ 120)) AND (S3.2PQVAL_21 EQ 121))) [S3.VAL_2 = 'le Leadership'];
IF (((S3.SUMVAL GE 200) AND (S3.SUMVAL LT 300) AND ((S3.QVAL1 EQ 101) OR (S3.QVAL2 EQ 102) OR (S3.QVAL3 EQ 103) OR (S3.QVAL4 EQ 104) OR (S3.QVAL5 EQ 105) OR (S3.QVAL6 EQ 106) OR (S3.QVAL7 EQ 107) OR (S3.QVAL8 EQ 108) OR (S3.QVAL9 EQ 109) OR (S3.QVAL_10 EQ 101.1) OR (S3.QVAL_11 EQ 101.2) OR (S3.QVAL_12 EQ 101.3) OR (S3.QVAL_13 EQ 101.4) OR (S3.QVAL_14 EQ 101.5) OR (S3.QVAL_15 EQ 101.6) OR (S3.QVAL_16 EQ 101.7) OR (S3.QVAL_17 EQ 101.8) OR (S3.QVAL_18 EQ 101.9) OR (S3.QVAL_19 EQ 101.19) OR (S3.QVAL_20 EQ 102.1) OR (S3.QVAL_21 EQ 102.2)) AND (S3.QVAL_22 EQ 102.3)) OR (((S3.2PQVAL1 EQ 101) OR (S3.2PQVAL2 EQ 102) OR (S3.2PQVAL3 EQ 103) OR (S3.2PQVAL4 EQ 104) OR (S3.2PQVAL5 EQ 105) OR (S3.2PQVAL6 EQ 106) OR (S3.2PQVAL7 EQ 107) OR (S3.2PQVAL8 EQ 108) OR (S3.2PQVAL9 EQ 109) OR (S3.2PQVAL_10 EQ 110) OR (S3.2PQVAL_11 EQ 111) OR (S3.2PQVAL_12 EQ 112) OR (S3.2PQVAL_13 EQ 113) OR (S3.2PQVAL_14 EQ 114) OR (S3.2PQVAL_15 EQ 115) OR (S3.2PQVAL_16 EQ 116) OR (S3.2PQVAL_17 EQ 117) OR (S3.2PQVAL_18 EQ 118) OR (S3.2PQVAL_19 EQ 119) OR

(S3.2PQVAL_20 EQ 120) OR (S3.2PQVAL_21 EQ 121)) AND (S3.2PQVAL_22 EQ 122))) [S3.VAL_2 = 'la Liberté'];
IF (((S3.SUMVAL GE 200) AND (S3.SUMVAL LT 300) AND ((S3.QVAL1 EQ 101) OR (S3.QVAL2 EQ 102) OR (S3.QVAL3 EQ 103) OR (S3.QVAL4 EQ 104) OR (S3.QVAL5 EQ 105) OR (S3.QVAL6 EQ 106) OR (S3.QVAL7 EQ 107) OR (S3.QVAL8 EQ 108) OR (S3.QVAL9 EQ 109) OR (S3.QVAL_10 EQ 101.1) OR (S3.QVAL_11 EQ 101.2) OR (S3.QVAL_12 EQ 101.3) OR (S3.QVAL_13 EQ 101.4) OR (S3.QVAL_14 EQ 101.5) OR (S3.QVAL_15 EQ 101.6) OR (S3.QVAL_16 EQ 101.7) OR (S3.QVAL_17 EQ 101.8) OR (S3.QVAL_18 EQ 101.9) OR (S3.QVAL_19 EQ 101.19) OR (S3.QVAL_20 EQ 102.1) OR (S3.QVAL_21 EQ 102.2) OR (S3.QVAL_22 EQ 102.3)) AND (S3.QVAL_23 EQ 102.4)) OR (((S3.2PQVAL1 EQ 101) OR (S3.2PQVAL2 EQ 102) OR (S3.2PQVAL3 EQ 103) OR (S3.2PQVAL4 EQ 104) OR (S3.2PQVAL5 EQ 105) OR (S3.2PQVAL6 EQ 106) OR (S3.2PQVAL7 EQ 107) OR (S3.2PQVAL8 EQ 108) OR (S3.2PQVAL9 EQ 109) OR (S3.2PQVAL_10 EQ 110) OR (S3.2PQVAL_11 EQ 111) OR (S3.2PQVAL_12 EQ 112) OR (S3.2PQVAL_13 EQ 113) OR (S3.2PQVAL_14 EQ 114) OR (S3.2PQVAL_15 EQ 115) OR (S3.2PQVAL_16 EQ 116) OR (S3.2PQVAL_17 EQ 117) OR (S3.2PQVAL_18 EQ 118) OR (S3.2PQVAL_19 EQ 119) OR (S3.2PQVAL_20 EQ 120) OR (S3.2PQVAL_21 EQ 121) OR (S3.2PQVAL_22 EQ 122)) AND (S3.2PQVAL_23 EQ 123))) [S3.VAL_2 = 'la Loyauté'];
IF (((S3.SUMVAL GE 200) AND (S3.SUMVAL LT 300) AND ((S3.QVAL1 EQ 101) OR (S3.QVAL2 EQ 102) OR (S3.QVAL3 EQ 103) OR (S3.QVAL4 EQ 104) OR (S3.QVAL5 EQ 105) OR (S3.QVAL6 EQ 106) OR (S3.QVAL7 EQ 107) OR (S3.QVAL8 EQ 108) OR (S3.QVAL9 EQ 109) OR (S3.QVAL_10 EQ 101.1) OR (S3.QVAL_11 EQ 101.2) OR (S3.QVAL_12 EQ 101.3) OR (S3.QVAL_13 EQ 101.4) OR (S3.QVAL_14 EQ 101.5) OR (S3.QVAL_15 EQ 101.6) OR (S3.QVAL_16 EQ 101.7) OR (S3.QVAL_17 EQ 101.8) OR (S3.QVAL_18 EQ 101.9) OR (S3.QVAL_19 EQ 101.19) OR (S3.QVAL_20 EQ 102.1) OR (S3.QVAL_21 EQ 102.2) OR (S3.QVAL_22 EQ 102.3) OR (S3.QVAL_23 EQ 102.4)) AND (S3.QVAL_24 EQ 102.5)) OR (((S3.2PQVAL1 EQ 101) OR (S3.2PQVAL2 EQ 102) OR (S3.2PQVAL3 EQ 103) OR (S3.2PQVAL4 EQ 104) OR (S3.2PQVAL5 EQ 105) OR (S3.2PQVAL6 EQ 106) OR (S3.2PQVAL7 EQ 107) OR (S3.2PQVAL8 EQ 108) OR (S3.2PQVAL9 EQ 109) OR (S3.2PQVAL_10 EQ 110) OR (S3.2PQVAL_11 EQ 111) OR (S3.2PQVAL_12 EQ 112) OR (S3.2PQVAL_13 EQ 113) OR (S3.2PQVAL_14 EQ 114) OR (S3.2PQVAL_15 EQ 115) OR (S3.2PQVAL_16 EQ 116) OR (S3.2PQVAL_17 EQ 117) OR (S3.2PQVAL_18 EQ 118) OR (S3.2PQVAL_19 EQ 119) OR (S3.2PQVAL_20 EQ 120) OR (S3.2PQVAL_21 EQ 121) OR (S3.2PQVAL_22 EQ 122) OR (S3.2PQVAL_23 EQ 123)) AND (S3.2PQVAL_24 EQ 124))) [S3.VAL_2 = 'la Maîtrise de soi'];
IF (((S3.SUMVAL GE 200) AND (S3.SUMVAL LT 300) AND ((S3.QVAL1 EQ 101) OR (S3.QVAL2 EQ 102) OR (S3.QVAL3 EQ 103) OR (S3.QVAL4 EQ 104) OR (S3.QVAL5 EQ 105) OR (S3.QVAL6 EQ 106) OR (S3.QVAL7 EQ 107) OR (S3.QVAL8 EQ 108) OR (S3.QVAL9 EQ 109) OR (S3.QVAL_10 EQ 101.1) OR (S3.QVAL_11 EQ 101.2) OR (S3.QVAL_12 EQ 101.3) OR (S3.QVAL_13 EQ 101.4) OR (S3.QVAL_14 EQ 101.5) OR (S3.QVAL_15 EQ 101.6) OR (S3.QVAL_16 EQ 101.7) OR (S3.QVAL_17 EQ 101.8) OR (S3.QVAL_18 EQ 101.9) OR (S3.QVAL_19 EQ 101.19) OR (S3.QVAL_20 EQ 102.1) OR (S3.QVAL_21 EQ 102.2) OR (S3.QVAL_22 EQ 102.3) OR (S3.QVAL_23 EQ 102.4) OR (S3.QVAL_24 EQ 102.5)) AND (S3.QVAL_25 EQ 102.6)) OR (((S3.2PQVAL1 EQ 101) OR (S3.2PQVAL2 EQ 102) OR (S3.2PQVAL3 EQ 103) OR (S3.2PQVAL4 EQ 104) OR (S3.2PQVAL5 EQ 105) OR (S3.2PQVAL6 EQ 106) OR (S3.2PQVAL7 EQ 107) OR (S3.2PQVAL8 EQ 108) OR (S3.2PQVAL9 EQ 109) OR (S3.2PQVAL_10 EQ 110) OR (S3.2PQVAL_11 EQ 111) OR (S3.2PQVAL_12 EQ 112) OR (S3.2PQVAL_13 EQ 113) OR (S3.2PQVAL_14 EQ 114) OR (S3.2PQVAL_15 EQ 115) OR (S3.2PQVAL_16 EQ 116) OR (S3.2PQVAL_17 EQ 117) OR (S3.2PQVAL_18 EQ 118) OR (S3.2PQVAL_19 EQ 119) OR (S3.2PQVAL_20 EQ

120) OR (S3.2PQVAL_21 EQ 121) OR (S3.2PQVAL_22 EQ 122) OR (S3.2PQVAL_23 EQ 123) OR (S3.2PQVAL_24 EQ 124)) AND (S3.2PQVAL_25 EQ 125))) [S3.VAL_2 = 'l´Ouverture d´esprit'];
IF (((S3.SUMVAL GE 200) AND (S3.SUMVAL LT 300) AND ((S3.QVAL1 EQ 101) OR (S3.QVAL2 EQ 102) OR (S3.QVAL3 EQ 103) OR (S3.QVAL4 EQ 104) OR (S3.QVAL5 EQ 105) OR (S3.QVAL6 EQ 106) OR (S3.QVAL7 EQ 107) OR (S3.QVAL8 EQ 108) OR (S3.QVAL9 EQ 109) OR (S3.QVAL_10 EQ 101.1) OR (S3.QVAL_11 EQ 101.2) OR (S3.QVAL_12 EQ 101.3) OR (S3.QVAL_13 EQ 101.4) OR (S3.QVAL_14 EQ 101.5) OR (S3.QVAL_15 EQ 101.6) OR (S3.QVAL_16 EQ 101.7) OR (S3.QVAL_17 EQ 101.8) OR (S3.QVAL_18 EQ 101.9) OR (S3.QVAL_19 EQ 101.19) OR (S3.QVAL_20 EQ 102.1) OR (S3.QVAL_21 EQ 102.2) OR (S3.QVAL_22 EQ 102.3) OR (S3.QVAL_23 EQ 102.4) OR (S3.QVAL_24 EQ 102.5) OR (S3.QVAL_25 EQ 102.6)) AND (S3.QVAL_26 EQ 102.7)) OR (((S3.2PQVAL1 EQ 101) OR (S3.2PQVAL2 EQ 102) OR (S3.2PQVAL3 EQ 103) OR (S3.2PQVAL4 EQ 104) OR (S3.2PQVAL5 EQ 105) OR (S3.2PQVAL6 EQ 106) OR (S3.2PQVAL7 EQ 107) OR (S3.2PQVAL8 EQ 108) OR (S3.2PQVAL9 EQ 109) OR (S3.2PQVAL_10 EQ 110) OR (S3.2PQVAL_11 EQ 111) OR (S3.2PQVAL_12 EQ 112) OR (S3.2PQVAL_13 EQ 113) OR (S3.2PQVAL_14 EQ 114) OR (S3.2PQVAL_15 EQ 115) OR (S3.2PQVAL_16 EQ 116) OR (S3.2PQVAL_17 EQ 117) OR (S3.2PQVAL_18 EQ 118) OR (S3.2PQVAL_19 EQ 119) OR (S3.2PQVAL_20 EQ 120) OR (S3.2PQVAL_21 EQ 121) OR (S3.2PQVAL_22 EQ 122) OR (S3.2PQVAL_23 EQ 123) OR (S3.2PQVAL_24 EQ 124) OR (S3.2PQVAL_25 EQ 125)) AND (S3.2PQVAL_26 EQ 126))) [S3.VAL_2 = 'le Plaisir'];
IF (((S3.SUMVAL GE 200) AND (S3.SUMVAL LT 300) AND ((S3.QVAL1 EQ 101) OR (S3.QVAL2 EQ 102) OR (S3.QVAL3 EQ 103) OR (S3.QVAL4 EQ 104) OR (S3.QVAL5 EQ 105) OR (S3.QVAL6 EQ 106) OR (S3.QVAL7 EQ 107) OR (S3.QVAL8 EQ 108) OR (S3.QVAL9 EQ 109) OR (S3.QVAL_10 EQ 101.1) OR (S3.QVAL_11 EQ 101.2) OR (S3.QVAL_12 EQ 101.3) OR (S3.QVAL_13 EQ 101.4) OR (S3.QVAL_14 EQ 101.5) OR (S3.QVAL_15 EQ 101.6) OR (S3.QVAL_16 EQ 101.7) OR (S3.QVAL_17 EQ 101.8) OR (S3.QVAL_18 EQ 101.9) OR (S3.QVAL_19 EQ 101.19) OR (S3.QVAL_20 EQ 102.1) OR (S3.QVAL_21 EQ 102.2) OR (S3.QVAL_22 EQ 102.3) OR (S3.QVAL_23 EQ 102.4) OR (S3.QVAL_24 EQ 102.5) OR (S3.QVAL_25 EQ 102.6) OR (S3.QVAL_26 EQ 102.7)) AND (S3.QVAL_27 EQ 102.8)) OR (((S3.2PQVAL1 EQ 101) OR (S3.2PQVAL2 EQ 102) OR (S3.2PQVAL3 EQ 103) OR (S3.2PQVAL4 EQ 104) OR (S3.2PQVAL5 EQ 105) OR (S3.2PQVAL6 EQ 106) OR (S3.2PQVAL7 EQ 107) OR (S3.2PQVAL8 EQ 108) OR (S3.2PQVAL9 EQ 109) OR (S3.2PQVAL_10 EQ 110) OR (S3.2PQVAL_11 EQ 111) OR (S3.2PQVAL_12 EQ 112) OR (S3.2PQVAL_13 EQ 113) OR (S3.2PQVAL_14 EQ 114) OR (S3.2PQVAL_15 EQ 115) OR (S3.2PQVAL_16 EQ 116) OR (S3.2PQVAL_17 EQ 117) OR (S3.2PQVAL_18 EQ 118) OR (S3.2PQVAL_19 EQ 119) OR (S3.2PQVAL_20 EQ 120) OR (S3.2PQVAL_21 EQ 121) OR (S3.2PQVAL_22 EQ 122) OR (S3.2PQVAL_23 EQ 123) OR (S3.2PQVAL_24 EQ 124) OR (S3.2PQVAL_25 EQ 125) OR (S3.2PQVAL_26 EQ 126)) AND (S3.2PQVAL_27 EQ 127))) [S3.VAL_2 = 'la Responsabilité'];
IF (((S3.SUMVAL GE 200) AND (S3.SUMVAL LT 300) AND ((S3.QVAL1 EQ 101) OR (S3.QVAL2 EQ 102) OR (S3.QVAL3 EQ 103) OR (S3.QVAL4 EQ 104) OR (S3.QVAL5 EQ 105) OR (S3.QVAL6 EQ 106) OR (S3.QVAL7 EQ 107) OR (S3.QVAL8 EQ 108) OR (S3.QVAL9 EQ 109) OR (S3.QVAL_10 EQ 101.1) OR (S3.QVAL_11 EQ 101.2) OR (S3.QVAL_12 EQ 101.3) OR (S3.QVAL_13 EQ 101.4) OR (S3.QVAL_14 EQ 101.5) OR (S3.QVAL_15 EQ 101.6) OR (S3.QVAL_16 EQ 101.7) OR (S3.QVAL_17 EQ 101.8) OR (S3.QVAL_18 EQ 101.9) OR (S3.QVAL_19 EQ 101.19) OR (S3.QVAL_20 EQ 102.1) OR (S3.QVAL_21 EQ 102.2) OR (S3.QVAL_22 EQ 102.3) OR (S3.QVAL_23 EQ 102.4) OR (S3.QVAL_24 EQ 102.5) OR (S3.QVAL_25 EQ 102.6) OR (S3.QVAL_26 EQ 102.7) OR (S3.QVAL_27 EQ 102.8)) AND (S3.QVAL_28 EQ 102.9)) OR (((S3.2PQVAL1 EQ 101) OR (S3.2PQVAL2 EQ 102) OR (S3.2PQVAL3 EQ 103) OR (S3.2PQVAL4 EQ 104) OR (S3.2PQVAL5 EQ 105) OR (S3.2PQVAL6 EQ 106) OR (S3.2PQVAL7 EQ 107) OR (S3.2PQVAL8 EQ 108) OR (S3.2PQVAL9 EQ 109) OR (S3.2PQVAL_10 EQ 110) OR

(S3.2PQVAL_11 EQ 111) OR (S3.2PQVAL_12 EQ 112) OR (S3.2PQVAL_13 EQ 113) OR (S3.2PQVAL_14 EQ 114) OR (S3.2PQVAL_15 EQ 115) OR (S3.2PQVAL_16 EQ 116) OR (S3.2PQVAL_17 EQ 117) OR (S3.2PQVAL_18 EQ 118) OR (S3.2PQVAL_19 EQ 119) OR (S3.2PQVAL_20 EQ 120) OR (S3.2PQVAL_21 EQ 121) OR (S3.2PQVAL_22 EQ 122) OR (S3.2PQVAL_23 EQ 123) OR (S3.2PQVAL_24 EQ 124) OR (S3.2PQVAL_25 EQ 125) OR (S3.2PQVAL_26 EQ 126) OR (S3.2PQVAL_27 EQ 127)) AND (S3.2PQVAL_28 EQ 128))) [S3.VAL_2 = 'la Sécurité'];
IF (((S3.SUMVAL GE 200) AND (S3.SUMVAL LT 300) AND ((S3.QVAL1 EQ 101) OR (S3.QVAL2 EQ 102) OR (S3.QVAL3 EQ 103) OR (S3.QVAL4 EQ 104) OR (S3.QVAL5 EQ 105) OR (S3.QVAL6 EQ 106) OR (S3.QVAL7 EQ 107) OR (S3.QVAL8 EQ 108) OR (S3.QVAL9 EQ 109) OR (S3.QVAL_10 EQ 101.1) OR (S3.QVAL_11 EQ 101.2) OR (S3.QVAL_12 EQ 101.3) OR (S3.QVAL_13 EQ 101.4) OR (S3.QVAL_14 EQ 101.5) OR (S3.QVAL_15 EQ 101.6) OR (S3.QVAL_16 EQ 101.7) OR (S3.QVAL_17 EQ 101.8) OR (S3.QVAL_18 EQ 101.9) OR (S3.QVAL_19 EQ 101.19) OR (S3.QVAL_20 EQ 102.1) OR (S3.QVAL_21 EQ 102.2) OR (S3.QVAL_22 EQ 102.3) OR (S3.QVAL_23 EQ 102.4) OR (S3.QVAL_24 EQ 102.5) OR (S3.QVAL_25 EQ 102.6) OR (S3.QVAL_26 EQ 102.7) OR (S3.QVAL_27 EQ 102.8) OR (S3.QVAL_28 EQ 102.9)) AND (S3.QVAL_29 EQ 102.29)) OR (((S3.2PQVAL1 EQ 101) OR (S3.2PQVAL2 EQ 102) OR (S3.2PQVAL3 EQ 103) OR (S3.2PQVAL4 EQ 104) OR (S3.2PQVAL5 EQ 105) OR (S3.2PQVAL6 EQ 106) OR (S3.2PQVAL7 EQ 107) OR (S3.2PQVAL8 EQ 108) OR (S3.2PQVAL9 EQ 109) OR (S3.2PQVAL_10 EQ 110) OR (S3.2PQVAL_11 EQ 111) OR (S3.2PQVAL_12 EQ 112) OR (S3.2PQVAL_13 EQ 113) OR (S3.2PQVAL_14 EQ 114) OR (S3.2PQVAL_15 EQ 115) OR (S3.2PQVAL_16 EQ 116) OR (S3.2PQVAL_17 EQ 117) OR (S3.2PQVAL_18 EQ 118) OR (S3.2PQVAL_19 EQ 119) OR (S3.2PQVAL_20 EQ 120) OR (S3.2PQVAL_21 EQ 121) OR (S3.2PQVAL_22 EQ 122) OR (S3.2PQVAL_23 EQ 123) OR (S3.2PQVAL_24 EQ 124) OR (S3.2PQVAL_25 EQ 125) OR (S3.2PQVAL_26 EQ 126) OR (S3.2PQVAL_27 EQ 127) OR (S3.2PQVAL_28 EQ 128)) AND (S3.2PQVAL_29 EQ 129))) [S3.VAL_2 = 'le Service'];
IF (((S3.SUMVAL GE 200) AND (S3.SUMVAL LT 300) AND (S3.QVAL1 EQ 101)) OR (S3.2PQVAL1 EQ 101)) [S3.P9MICROQ1 = 'entretenir vos amitiés'];
IF (((S3.SUMVAL GE 200) AND (S3.SUMVAL LT 300) AND (S3.QVAL1 EQ 0) AND (S3.QVAL2 EQ 102)) OR ((S3.2PQVAL1 EQ 0) AND (S3.2PQVAL2 EQ 102))) [S3.P9MICROQ1 = 'aimer et être aiméE par ceux qui vous entourent'];
IF (((S3.SUMVAL GE 200) AND (S3.SUMVAL LT 300) AND (S3.QVAL1 EQ 0) AND (S3.QVAL2 EQ 0) AND (S3.QVAL3 EQ 103)) OR ((S3.2PQVAL1 EQ 0) AND (S3.2PQVAL2 EQ 0) AND (S3.2PQVAL3 EQ 103))) [S3.P9MICROQ1 = 'faire partie de quelquechose, d´un groupe'];
IF (((S3.SUMVAL GE 200) AND (S3.SUMVAL LT 300) AND (S3.QVAL1 EQ 0) AND (S3.QVAL2 EQ 0) AND (S3.QVAL3 EQ 0) AND (S3.QVAL4 EQ 104)) OR ((S3.2PQVAL1 EQ 0) AND (S3.2PQVAL2 EQ 0) AND (S3.2PQVAL3 EQ 0) AND (S3.2PQVAL4 EQ 104))) [S3.P9MICROQ1 = 'vous occuper des autres'];
IF (((S3.SUMVAL GE 200) AND (S3.SUMVAL LT 300) AND (S3.QVAL1 EQ 0) AND (S3.QVAL2 EQ 0) AND (S3.QVAL3 EQ 0) AND (S3.QVAL4 EQ 0) AND (S3.QVAL5 EQ 105)) OR ((S3.2PQVAL1 EQ 0) AND (S3.2PQVAL2 EQ 0) AND (S3.2PQVAL3 EQ 0) AND (S3.2PQVAL4 EQ 0) AND (S3.2PQVAL5 EQ 105))) [S3.P9MICROQ1 = 'être indépendant{e} et autonome'];
IF (((S3.SUMVAL GE 200) AND (S3.SUMVAL LT 300) AND (S3.QVAL1 EQ 0) AND (S3.QVAL2 EQ 0) AND (S3.QVAL3 EQ 0) AND (S3.QVAL4 EQ 0) AND (S3.QVAL5 EQ 0) AND (S3.QVAL6 EQ 106)) OR ((S3.2PQVAL1 EQ 0) AND (S3.2PQVAL2 EQ 0) AND (S3.2PQVAL3 EQ 0) AND (S3.2PQVAL4 EQ 0) AND (S3.2PQVAL5 EQ 0) AND (S3.2PQVAL6 EQ 106))) [S3.P9MICROQ1 = 'apprécier la beauté en vous et autour de vous'];

IF (((S3.SUMVAL GE 200) AND (S3.SUMVAL LT 300) AND (S3.QVAL1 EQ 0) AND (S3.QVAL2 EQ 0) AND (S3.QVAL3 EQ 0) AND (S3.QVAL4 EQ 0) AND (S3.QVAL5 EQ 0) AND (S3.QVAL6 EQ 0) AND (S3.QVAL7 EQ 107)) OR ((S3.2PQVAL1 EQ 0) AND (S3.2PQVAL2 EQ 0) AND (S3.2PQVAL3 EQ 0) AND (S3.2PQVAL4 EQ 0) AND (S3.2PQVAL5 EQ 0) AND (S3.2PQVAL6 EQ 0) AND (S3.2PQVAL7 EQ 107))) [S3.P9MICROQ1 = 'être sensible aux autres et agir pour leur bien'];
IF (((S3.SUMVAL GE 200) AND (S3.SUMVAL LT 300) AND (S3.QVAL1 EQ 0) AND (S3.QVAL2 EQ 0) AND (S3.QVAL3 EQ 0) AND (S3.QVAL4 EQ 0) AND (S3.QVAL5 EQ 0) AND (S3.QVAL6 EQ 0) AND (S3.QVAL7 EQ 0) AND (S3.QVAL8 EQ 108)) OR ((S3.2PQVAL1 EQ 0) AND (S3.2PQVAL2 EQ 0) AND (S3.2PQVAL3 EQ 0) AND (S3.2PQVAL4 EQ 0) AND (S3.2PQVAL5 EQ 0) AND (S3.2PQVAL6 EQ 0) AND (S3.2PQVAL7 EQ 0) AND (S3.2PQVAL8 EQ 108))) [S3.P9MICROQ1 = 'être en mesure de faire des compromis'];
IF (((S3.SUMVAL GE 200) AND (S3.SUMVAL LT 300) AND (S3.QVAL1 EQ 0) AND (S3.QVAL2 EQ 0) AND (S3.QVAL3 EQ 0) AND (S3.QVAL4 EQ 0) AND (S3.QVAL5 EQ 0) AND (S3.QVAL6 EQ 0) AND (S3.QVAL7 EQ 0) AND (S3.QVAL8 EQ 0) AND (S3.QVAL9 EQ 109)) OR ((S3.2PQVAL1 EQ 0) AND (S3.2PQVAL2 EQ 0) AND (S3.2PQVAL3 EQ 0) AND (S3.2PQVAL4 EQ 0) AND (S3.2PQVAL5 EQ 0) AND (S3.2PQVAL6 EQ 0) AND (S3.2PQVAL7 EQ 0) AND (S3.2PQVAL8 EQ 0) AND (S3.2PQVAL9 EQ 109))) [S3.P9MICROQ1 = 'avoir une vie plaisante et confortable'];
IF (((S3.SUMVAL GE 200) AND (S3.SUMVAL LT 300) AND (S3.QVAL1 EQ 0) AND (S3.QVAL2 EQ 0) AND (S3.QVAL3 EQ 0) AND (S3.QVAL4 EQ 0) AND (S3.QVAL5 EQ 0) AND (S3.QVAL6 EQ 0) AND (S3.QVAL7 EQ 0) AND (S3.QVAL8 EQ 0) AND (S3.QVAL9 EQ 0) AND (S3.QVAL_10 EQ 101.1)) OR ((S3.2PQVAL1 EQ 0) AND (S3.2PQVAL2 EQ 0) AND (S3.2PQVAL3 EQ 0) AND (S3.2PQVAL4 EQ 0) AND (S3.2PQVAL5 EQ 0) AND (S3.2PQVAL6 EQ 0) AND (S3.2PQVAL7 EQ 0) AND (S3.2PQVAL8 EQ 0) AND (S3.2PQVAL9 EQ 0) AND (S3.2PQVAL_10 EQ 110))) [S3.P9MICROQ1 = 'être brave dans l´adversité'];
IF (((S3.SUMVAL GE 200) AND (S3.SUMVAL LT 300) AND (S3.QVAL1 EQ 0) AND (S3.QVAL2 EQ 0) AND (S3.QVAL3 EQ 0) AND (S3.QVAL4 EQ 0) AND (S3.QVAL5 EQ 0) AND (S3.QVAL6 EQ 0) AND (S3.QVAL7 EQ 0) AND (S3.QVAL8 EQ 0) AND (S3.QVAL9 EQ 0) AND (S3.QVAL_10 EQ 0) AND (S3.QVAL_11 EQ 101.2)) OR ((S3.2PQVAL1 EQ 0) AND (S3.2PQVAL2 EQ 0) AND (S3.2PQVAL3 EQ 0) AND (S3.2PQVAL4 EQ 0) AND (S3.2PQVAL5 EQ 0) AND (S3.2PQVAL6 EQ 0) AND (S3.2PQVAL7 EQ 0) AND (S3.2PQVAL8 EQ 0) AND (S3.2PQVAL9 EQ 0) AND (S3.2PQVAL_10 EQ 0) AND (S3.2PQVAL_11 EQ 111))) [S3.P9MICROQ1 = 'avoir des idées nouvelles, à créer'];
IF (((S3.SUMVAL GE 200) AND (S3.SUMVAL LT 300) AND (S3.QVAL1 EQ 0) AND (S3.QVAL2 EQ 0) AND (S3.QVAL3 EQ 0) AND (S3.QVAL4 EQ 0) AND (S3.QVAL5 EQ 0) AND (S3.QVAL6 EQ 0) AND (S3.QVAL7 EQ 0) AND (S3.QVAL8 EQ 0) AND (S3.QVAL9 EQ 0) AND (S3.QVAL_10 EQ 0) AND (S3.QVAL_11 EQ 0) AND (S3.QVAL_12 EQ 101.3)) OR ((S3.2PQVAL1 EQ 0) AND (S3.2PQVAL2 EQ 0) AND (S3.2PQVAL3 EQ 0) AND (S3.2PQVAL4 EQ 0) AND (S3.2PQVAL5 EQ 0) AND (S3.2PQVAL6 EQ 0) AND (S3.2PQVAL7 EQ 0) AND (S3.2PQVAL8 EQ 0) AND (S3.2PQVAL9 EQ 0) AND (S3.2PQVAL_10 EQ 0) AND (S3.2PQVAL_11 EQ 0) AND (S3.2PQVAL_12 EQ 112))) [S3.P9MICROQ1 = 'évoluer continuellement'];
IF (((S3.SUMVAL GE 200) AND (S3.SUMVAL LT 300) AND (S3.QVAL1 EQ 0) AND (S3.QVAL2 EQ 0) AND (S3.QVAL3 EQ 0) AND (S3.QVAL4 EQ 0) AND (S3.QVAL5 EQ 0) AND (S3.QVAL6 EQ 0) AND (S3.QVAL7 EQ 0) AND (S3.QVAL8 EQ 0) AND (S3.QVAL9 EQ 0) AND (S3.QVAL_10 EQ 0) AND (S3.QVAL_11 EQ 0) AND (S3.QVAL_12 EQ 0) AND (S3.QVAL_13 EQ 101.4)) OR ((S3.2PQVAL1 EQ 0) AND (S3.2PQVAL2 EQ 0) AND (S3.2PQVAL3 EQ 0) AND (S3.2PQVAL4 EQ 0) AND (S3.2PQVAL5 EQ 0) AND (S3.2PQVAL6 EQ 0) AND (S3.2PQVAL7 EQ 0) AND (S3.2PQVAL8 EQ 0) AND (S3.2PQVAL9 EQ 0) AND (S3.2PQVAL_10 EQ 0) AND

(S3.2PQVAL_11 EQ 0) AND (S3.2PQVAL_12 EQ 0) AND (S3.2PQVAL_13 EQ 113))) [S3.P9MICROQ1 = 'explorer et apprendre de nouvelles choses'];
IF (((S3.SUMVAL GE 200) AND (S3.SUMVAL LT 300) AND (S3.QVAL1 EQ 0) AND (S3.QVAL2 EQ 0) AND (S3.QVAL3 EQ 0) AND (S3.QVAL4 EQ 0) AND (S3.QVAL5 EQ 0) AND (S3.QVAL6 EQ 0) AND (S3.QVAL7 EQ 0) AND (S3.QVAL8 EQ 0) AND (S3.QVAL9 EQ 0) AND (S3.QVAL_10 EQ 0) AND (S3.QVAL_11 EQ 0) AND (S3.QVAL_12 EQ 0) AND (S3.QVAL_13 EQ 0) AND (S3.QVAL_14 EQ 101.5)) OR ((S3.2PQVAL1 EQ 0) AND (S3.2PQVAL2 EQ 0) AND (S3.2PQVAL3 EQ 0) AND (S3.2PQVAL4 EQ 0) AND (S3.2PQVAL5 EQ 0) AND (S3.2PQVAL6 EQ 0) AND (S3.2PQVAL7 EQ 0) AND (S3.2PQVAL8 EQ 0) AND (S3.2PQVAL9 EQ 0) AND (S3.2PQVAL_10 EQ 0) AND (S3.2PQVAL_11 EQ 0) AND (S3.2PQVAL_12 EQ 0) AND (S3.2PQVAL_13 EQ 0) AND (S3.2PQVAL_14 EQ 114))) [S3.P9MICROQ1 = 'maintenir une attitude positive et optimiste peu importe ce qu´il advient'];
IF (((S3.SUMVAL GE 200) AND (S3.SUMVAL LT 300) AND (S3.QVAL1 EQ 0) AND (S3.QVAL2 EQ 0) AND (S3.QVAL3 EQ 0) AND (S3.QVAL4 EQ 0) AND (S3.QVAL5 EQ 0) AND (S3.QVAL6 EQ 0) AND (S3.QVAL7 EQ 0) AND (S3.QVAL8 EQ 0) AND (S3.QVAL9 EQ 0) AND (S3.QVAL_10 EQ 0) AND (S3.QVAL_11 EQ 0) AND (S3.QVAL_12 EQ 0) AND (S3.QVAL_13 EQ 0) AND (S3.QVAL_14 EQ 0) AND (S3.QVAL_15 EQ 101.6)) OR ((S3.2PQVAL1 EQ 0) AND (S3.2PQVAL2 EQ 0) AND (S3.2PQVAL3 EQ 0) AND (S3.2PQVAL4 EQ 0) AND (S3.2PQVAL5 EQ 0) AND (S3.2PQVAL6 EQ 0) AND (S3.2PQVAL7 EQ 0) AND (S3.2PQVAL8 EQ 0) AND (S3.2PQVAL9 EQ 0) AND (S3.2PQVAL_10 EQ 0) AND (S3.2PQVAL_11 EQ 0) AND (S3.2PQVAL_12 EQ 0) AND (S3.2PQVAL_13 EQ 0) AND (S3.2PQVAL_14 EQ 0) AND (S3.2PQVAL_15 EQ 115))) [S3.P9MICROQ1 = 'vous sentir bien dans votre peau'];
IF (((S3.SUMVAL GE 200) AND (S3.SUMVAL LT 300) AND (S3.QVAL1 EQ 0) AND (S3.QVAL2 EQ 0) AND (S3.QVAL3 EQ 0) AND (S3.QVAL4 EQ 0) AND (S3.QVAL5 EQ 0) AND (S3.QVAL6 EQ 0) AND (S3.QVAL7 EQ 0) AND (S3.QVAL8 EQ 0) AND (S3.QVAL9 EQ 0) AND (S3.QVAL_10 EQ 0) AND (S3.QVAL_11 EQ 0) AND (S3.QVAL_12 EQ 0) AND (S3.QVAL_13 EQ 0) AND (S3.QVAL_14 EQ 0) AND (S3.QVAL_15 EQ 0) AND (S3.QVAL_16 EQ 101.7)) OR ((S3.2PQVAL1 EQ 0) AND (S3.2PQVAL2 EQ 0) AND (S3.2PQVAL3 EQ 0) AND (S3.2PQVAL4 EQ 0) AND (S3.2PQVAL5 EQ 0) AND (S3.2PQVAL6 EQ 0) AND (S3.2PQVAL7 EQ 0) AND (S3.2PQVAL8 EQ 0) AND (S3.2PQVAL9 EQ 0) AND (S3.2PQVAL_10 EQ 0) AND (S3.2PQVAL_11 EQ 0) AND (S3.2PQVAL_12 EQ 0) AND (S3.2PQVAL_13 EQ 0) AND (S3.2PQVAL_14 EQ 0) AND (S3.2PQVAL_15 EQ 0) AND (S3.2PQVAL_16 EQ 116))) [S3.P9MICROQ1 = 'avoir une famille comblée et heureuse'];
IF (((S3.SUMVAL GE 200) AND (S3.SUMVAL LT 300) AND (S3.QVAL1 EQ 0) AND (S3.QVAL2 EQ 0) AND (S3.QVAL3 EQ 0) AND (S3.QVAL4 EQ 0) AND (S3.QVAL5 EQ 0) AND (S3.QVAL6 EQ 0) AND (S3.QVAL7 EQ 0) AND (S3.QVAL8 EQ 0) AND (S3.QVAL9 EQ 0) AND (S3.QVAL_10 EQ 0) AND (S3.QVAL_11 EQ 0) AND (S3.QVAL_12 EQ 0) AND (S3.QVAL_13 EQ 0) AND (S3.QVAL_14 EQ 0) AND (S3.QVAL_15 EQ 0) AND (S3.QVAL_16 EQ 0) AND (S3.QVAL_17 EQ 101.8)) OR ((S3.2PQVAL1 EQ 0) AND (S3.2PQVAL2 EQ 0) AND (S3.2PQVAL3 EQ 0) AND (S3.2PQVAL4 EQ 0) AND (S3.2PQVAL5 EQ 0) AND (S3.2PQVAL6 EQ 0) AND (S3.2PQVAL7 EQ 0) AND (S3.2PQVAL8 EQ 0) AND (S3.2PQVAL9 EQ 0) AND (S3.2PQVAL_10 EQ 0) AND (S3.2PQVAL_11 EQ 0) AND (S3.2PQVAL_12 EQ 0) AND (S3.2PQVAL_13 EQ 0) AND (S3.2PQVAL_14 EQ 0) AND (S3.2PQVAL_15 EQ 0) AND (S3.2PQVAL_16 EQ 0) AND (S3.2PQVAL_17 EQ 117))) [S3.P9MICROQ1 = 'être fiable, être quelqu´un de confiance'];
IF (((S3.SUMVAL GE 200) AND (S3.SUMVAL LT 300) AND (S3.QVAL1 EQ 0) AND (S3.QVAL2 EQ 0) AND (S3.QVAL3 EQ 0) AND (S3.QVAL4 EQ 0) AND (S3.QVAL5 EQ 0) AND (S3.QVAL6 EQ 0) AND (S3.QVAL7 EQ 0) AND (S3.QVAL8 EQ 0) AND (S3.QVAL9 EQ 0) AND (S3.QVAL_10 EQ 0) AND (S3.QVAL_11 EQ 0) AND

(S3.QVAL_12 EQ 0) AND (S3.QVAL_13 EQ 0) AND (S3.QVAL_14 EQ 0) AND (S3.QVAL_15 EQ 0) AND (S3.QVAL_16 EQ 0) AND (S3.QVAL_17 EQ 0) AND (S3.QVAL_18 EQ 101.9)) OR ((S3.2PQVAL1 EQ 0) AND (S3.2PQVAL2 EQ 0) AND (S3.2PQVAL3 EQ 0) AND (S3.2PQVAL4 EQ 0) AND (S3.2PQVAL5 EQ 0) AND (S3.2PQVAL6 EQ 0) AND (S3.2PQVAL7 EQ 0) AND (S3.2PQVAL8 EQ 0) AND (S3.2PQVAL9 EQ 0) AND (S3.2PQVAL_10 EQ 0) AND (S3.2PQVAL_11 EQ 0) AND (S3.2PQVAL_12 EQ 0) AND (S3.2PQVAL_13 EQ 0) AND (S3.2PQVAL_14 EQ 0) AND (S3.2PQVAL_15 EQ 0) AND (S3.2PQVAL_16 EQ 0) AND (S3.2PQVAL_17 EQ 0) AND (S3.2PQVAL_18 EQ 118))) [S3.P9MICROQ1 = 'vous ajuster aux nouvelles circonstances qui se présentent'];
IF (((S3.SUMVAL GE 200) AND (S3.SUMVAL LT 300) AND (S3.QVAL1 EQ 0) AND (S3.QVAL2 EQ 0) AND (S3.QVAL3 EQ 0) AND (S3.QVAL4 EQ 0) AND (S3.QVAL5 EQ 0) AND (S3.QVAL6 EQ 0) AND (S3.QVAL7 EQ 0) AND (S3.QVAL8 EQ 0) AND (S3.QVAL9 EQ 0) AND (S3.QVAL_10 EQ 0) AND (S3.QVAL_11 EQ 0) AND (S3.QVAL_12 EQ 0) AND (S3.QVAL_13 EQ 0) AND (S3.QVAL_14 EQ 0) AND (S3.QVAL_15 EQ 0) AND (S3.QVAL_16 EQ 0) AND (S3.QVAL_17 EQ 0) AND (S3.QVAL_18 EQ 0) AND (S3.QVAL_19 EQ 101.19)) OR ((S3.2PQVAL1 EQ 0) AND (S3.2PQVAL2 EQ 0) AND (S3.2PQVAL3 EQ 0) AND (S3.2PQVAL4 EQ 0) AND (S3.2PQVAL5 EQ 0) AND (S3.2PQVAL6 EQ 0) AND (S3.2PQVAL7 EQ 0) AND (S3.2PQVAL8 EQ 0) AND (S3.2PQVAL9 EQ 0) AND (S3.2PQVAL_10 EQ 0) AND (S3.2PQVAL_11 EQ 0) AND (S3.2PQVAL_12 EQ 0) AND (S3.2PQVAL_13 EQ 0) AND (S3.2PQVAL_14 EQ 0) AND (S3.2PQVAL_15 EQ 0) AND (S3.2PQVAL_16 EQ 0) AND (S3.2PQVAL_17 EQ 0) AND (S3.2PQVAL_18 EQ 0) AND (S3.2PQVAL_19 EQ 119))) [S3.P9MICROQ1 = 'donner sans attendre de recevoir en retour'];
IF (((S3.SUMVAL GE 200) AND (S3.SUMVAL LT 300) AND (S3.QVAL1 EQ 0) AND (S3.QVAL2 EQ 0) AND (S3.QVAL3 EQ 0) AND (S3.QVAL4 EQ 0) AND (S3.QVAL5 EQ 0) AND (S3.QVAL6 EQ 0) AND (S3.QVAL7 EQ 0) AND (S3.QVAL8 EQ 0) AND (S3.QVAL9 EQ 0) AND (S3.QVAL_10 EQ 0) AND (S3.QVAL_11 EQ 0) AND (S3.QVAL_12 EQ 0) AND (S3.QVAL_13 EQ 0) AND (S3.QVAL_14 EQ 0) AND (S3.QVAL_15 EQ 0) AND (S3.QVAL_16 EQ 0) AND (S3.QVAL_17 EQ 0) AND (S3.QVAL_18 EQ 0) AND (S3.QVAL_19 EQ 0) AND (S3.QVAL_20 EQ 102.1)) OR ((S3.2PQVAL1 EQ 0) AND (S3.2PQVAL2 EQ 0) AND (S3.2PQVAL3 EQ 0) AND (S3.2PQVAL4 EQ 0) AND (S3.2PQVAL5 EQ 0) AND (S3.2PQVAL6 EQ 0) AND (S3.2PQVAL7 EQ 0) AND (S3.2PQVAL8 EQ 0) AND (S3.2PQVAL9 EQ 0) AND (S3.2PQVAL_10 EQ 0) AND (S3.2PQVAL_11 EQ 0) AND (S3.2PQVAL_12 EQ 0) AND (S3.2PQVAL_13 EQ 0) AND (S3.2PQVAL_14 EQ 0) AND (S3.2PQVAL_15 EQ 0) AND (S3.2PQVAL_16 EQ 0) AND (S3.2PQVAL_17 EQ 0) AND (S3.2PQVAL_18 EQ 0) AND (S3.2PQVAL_19 EQ 0) AND (S3.2PQVAL_20 EQ 120))) [S3.P9MICROQ1 = 'être une personne intelligente et à garder un mental actif'];
IF (((S3.SUMVAL GE 200) AND (S3.SUMVAL LT 300) AND (S3.QVAL1 EQ 0) AND (S3.QVAL2 EQ 0) AND (S3.QVAL3 EQ 0) AND (S3.QVAL4 EQ 0) AND (S3.QVAL5 EQ 0) AND (S3.QVAL6 EQ 0) AND (S3.QVAL7 EQ 0) AND (S3.QVAL8 EQ 0) AND (S3.QVAL9 EQ 0) AND (S3.QVAL_10 EQ 0) AND (S3.QVAL_11 EQ 0) AND (S3.QVAL_12 EQ 0) AND (S3.QVAL_13 EQ 0) AND (S3.QVAL_14 EQ 0) AND (S3.QVAL_15 EQ 0) AND (S3.QVAL_16 EQ 0) AND (S3.QVAL_17 EQ 0) AND (S3.QVAL_18 EQ 0) AND (S3.QVAL_19 EQ 0) AND (S3.QVAL_20 EQ 0) AND (S3.QVAL_21 EQ 102.2)) OR ((S3.2PQVAL1 EQ 0) AND (S3.2PQVAL2 EQ 0) AND (S3.2PQVAL3 EQ 0) AND (S3.2PQVAL4 EQ 0) AND (S3.2PQVAL5 EQ 0) AND (S3.2PQVAL6 EQ 0) AND (S3.2PQVAL7 EQ 0) AND (S3.2PQVAL8 EQ 0) AND (S3.2PQVAL9 EQ 0) AND (S3.2PQVAL_10 EQ 0) AND (S3.2PQVAL_11 EQ 0) AND (S3.2PQVAL_12 EQ 0) AND (S3.2PQVAL_13 EQ 0) AND (S3.2PQVAL_14 EQ 0) AND (S3.2PQVAL_15 EQ 0) AND (S3.2PQVAL_16 EQ 0) AND (S3.2PQVAL_17 EQ 0) AND (S3.2PQVAL_18 EQ

0) AND (S3.2PQVAL_19 EQ 0) AND (S3.2PQVAL_20 EQ 0) AND (S3.2PQVAL_21 EQ 121))) [S3.P9MICROQ1 = 'inspirer et guider les autres'];
IF (((S3.SUMVAL GE 200) AND (S3.SUMVAL LT 300) AND (S3.QVAL1 EQ 0) AND (S3.QVAL2 EQ 0) AND (S3.QVAL3 EQ 0) AND (S3.QVAL4 EQ 0) AND (S3.QVAL5 EQ 0) AND (S3.QVAL6 EQ 0) AND (S3.QVAL7 EQ 0) AND (S3.QVAL8 EQ 0) AND (S3.QVAL9 EQ 0) AND (S3.QVAL_10 EQ 0) AND (S3.QVAL_11 EQ 0) AND (S3.QVAL_12 EQ 0) AND (S3.QVAL_13 EQ 0) AND (S3.QVAL_14 EQ 0) AND (S3.QVAL_15 EQ 0) AND (S3.QVAL_16 EQ 0) AND (S3.QVAL_17 EQ 0) AND (S3.QVAL_18 EQ 0) AND (S3.QVAL_19 EQ 0) AND (S3.QVAL_20 EQ 0) AND (S3.QVAL_21 EQ 0) AND (S3.QVAL_22 EQ 102.3)) OR ((S3.2PQVAL1 EQ 0) AND (S3.2PQVAL2 EQ 0) AND (S3.2PQVAL3 EQ 0) AND (S3.2PQVAL4 EQ 0) AND (S3.2PQVAL5 EQ 0) AND (S3.2PQVAL6 EQ 0) AND (S3.2PQVAL7 EQ 0) AND (S3.2PQVAL8 EQ 0) AND (S3.2PQVAL9 EQ 0) AND (S3.2PQVAL_10 EQ 0) AND (S3.2PQVAL_11 EQ 0) AND (S3.2PQVAL_12 EQ 0) AND (S3.2PQVAL_13 EQ 0) AND (S3.2PQVAL_14 EQ 0) AND (S3.2PQVAL_15 EQ 0) AND (S3.2PQVAL_16 EQ 0) AND (S3.2PQVAL_17 EQ 0) AND (S3.2PQVAL_18 EQ 0) AND (S3.2PQVAL_19 EQ 0) AND (S3.2PQVAL_20 EQ 0) AND (S3.2PQVAL_21 EQ 0) AND (S3.2PQVAL_22 EQ 122))) [S3.P9MICROQ1 = 'être libre et sans limitations non-désirées'];
IF (((S3.SUMVAL GE 200) AND (S3.SUMVAL LT 300) AND (S3.QVAL1 EQ 0) AND (S3.QVAL2 EQ 0) AND (S3.QVAL3 EQ 0) AND (S3.QVAL4 EQ 0) AND (S3.QVAL5 EQ 0) AND (S3.QVAL6 EQ 0) AND (S3.QVAL7 EQ 0) AND (S3.QVAL8 EQ 0) AND (S3.QVAL9 EQ 0) AND (S3.QVAL_10 EQ 0) AND (S3.QVAL_11 EQ 0) AND (S3.QVAL_12 EQ 0) AND (S3.QVAL_13 EQ 0) AND (S3.QVAL_14 EQ 0) AND (S3.QVAL_15 EQ 0) AND (S3.QVAL_16 EQ 0) AND (S3.QVAL_17 EQ 0) AND (S3.QVAL_18 EQ 0) AND (S3.QVAL_19 EQ 0) AND (S3.QVAL_20 EQ 0) AND (S3.QVAL_21 EQ 0) AND (S3.QVAL_22 EQ 0) AND (S3.QVAL_23 EQ 102.4)) OR ((S3.2PQVAL1 EQ 0) AND (S3.2PQVAL2 EQ 0) AND (S3.2PQVAL3 EQ 0) AND (S3.2PQVAL4 EQ 0) AND (S3.2PQVAL5 EQ 0) AND (S3.2PQVAL6 EQ 0) AND (S3.2PQVAL7 EQ 0) AND (S3.2PQVAL8 EQ 0) AND (S3.2PQVAL9 EQ 0) AND (S3.2PQVAL_10 EQ 0) AND (S3.2PQVAL_11 EQ 0) AND (S3.2PQVAL_12 EQ 0) AND (S3.2PQVAL_13 EQ 0) AND (S3.2PQVAL_14 EQ 0) AND (S3.2PQVAL_15 EQ 0) AND (S3.2PQVAL_16 EQ 0) AND (S3.2PQVAL_17 EQ 0) AND (S3.2PQVAL_18 EQ 0) AND (S3.2PQVAL_19 EQ 0) AND (S3.2PQVAL_20 EQ 0) AND (S3.2PQVAL_21 EQ 0) AND (S3.2PQVAL_22 EQ 0) AND (S3.2PQVAL_23 EQ 123))) [S3.P9MICROQ1 = 'être authentique envers vous-même et dans vos relations'];
IF (((S3.SUMVAL GE 200) AND (S3.SUMVAL LT 300) AND (S3.QVAL1 EQ 0) AND (S3.QVAL2 EQ 0) AND (S3.QVAL3 EQ 0) AND (S3.QVAL4 EQ 0) AND (S3.QVAL5 EQ 0) AND (S3.QVAL6 EQ 0) AND (S3.QVAL7 EQ 0) AND (S3.QVAL8 EQ 0) AND (S3.QVAL9 EQ 0) AND (S3.QVAL_10 EQ 0) AND (S3.QVAL_11 EQ 0) AND (S3.QVAL_12 EQ 0) AND (S3.QVAL_13 EQ 0) AND (S3.QVAL_14 EQ 0) AND (S3.QVAL_15 EQ 0) AND (S3.QVAL_16 EQ 0) AND (S3.QVAL_17 EQ 0) AND (S3.QVAL_18 EQ 0) AND (S3.QVAL_19 EQ 0) AND (S3.QVAL_20 EQ 0) AND (S3.QVAL_21 EQ 0) AND (S3.QVAL_22 EQ 0) AND (S3.QVAL_23 EQ 0) AND (S3.QVAL_24 EQ 102.5)) OR ((S3.2PQVAL1 EQ 0) AND (S3.2PQVAL2 EQ 0) AND (S3.2PQVAL3 EQ 0) AND (S3.2PQVAL4 EQ 0) AND (S3.2PQVAL5 EQ 0) AND (S3.2PQVAL6 EQ 0) AND (S3.2PQVAL7 EQ 0) AND (S3.2PQVAL8 EQ 0) AND (S3.2PQVAL9 EQ 0) AND (S3.2PQVAL_10 EQ 0) AND (S3.2PQVAL_11 EQ 0) AND (S3.2PQVAL_12 EQ 0) AND (S3.2PQVAL_13 EQ 0) AND (S3.2PQVAL_14 EQ 0) AND (S3.2PQVAL_15 EQ 0) AND (S3.2PQVAL_16 EQ 0) AND (S3.2PQVAL_17 EQ 0) AND (S3.2PQVAL_18 EQ 0) AND (S3.2PQVAL_19 EQ 0) AND (S3.2PQVAL_20 EQ 0) AND (S3.2PQVAL_21 EQ 0) AND (S3.2PQVAL_22 EQ 0) AND (S3.2PQVAL_23 EQ 0) AND (S3.2PQVAL_24 EQ 124))) [S3.P9MICROQ1 = 'être à votre affaire et prendre les bonnes décisions'];

IF (((S3.SUMVAL GE 200) AND (S3.SUMVAL LT 300) AND (S3.QVAL1 EQ 0) AND (S3.QVAL2 EQ 0) AND (S3.QVAL3 EQ 0) AND (S3.QVAL4 EQ 0) AND (S3.QVAL5 EQ 0) AND (S3.QVAL6 EQ 0) AND (S3.QVAL7 EQ 0) AND (S3.QVAL8 EQ 0) AND (S3.QVAL9 EQ 0) AND (S3.QVAL_10 EQ 0) AND (S3.QVAL_11 EQ 0) AND (S3.QVAL_12 EQ 0) AND (S3.QVAL_13 EQ 0) AND (S3.QVAL_14 EQ 0) AND (S3.QVAL_15 EQ 0) AND (S3.QVAL_16 EQ 0) AND (S3.QVAL_17 EQ 0) AND (S3.QVAL_18 EQ 0) AND (S3.QVAL_19 EQ 0) AND (S3.QVAL_20 EQ 0) AND (S3.QVAL_21 EQ 0) AND (S3.QVAL_22 EQ 0) AND (S3.QVAL_23 EQ 0) AND (S3.QVAL_24 EQ 0) AND (S3.QVAL_25 EQ 102.6)) OR ((S3.2PQVAL1 EQ 0) AND (S3.2PQVAL2 EQ 0) AND (S3.2PQVAL3 EQ 0) AND (S3.2PQVAL4 EQ 0) AND (S3.2PQVAL5 EQ 0) AND (S3.2PQVAL6 EQ 0) AND (S3.2PQVAL7 EQ 0) AND (S3.2PQVAL8 EQ 0) AND (S3.2PQVAL9 EQ 0) AND (S3.2PQVAL_10 EQ 0) AND (S3.2PQVAL_11 EQ 0) AND (S3.2PQVAL_12 EQ 0) AND (S3.2PQVAL_13 EQ 0) AND (S3.2PQVAL_14 EQ 0) AND (S3.2PQVAL_15 EQ 0) AND (S3.2PQVAL_16 EQ 0) AND (S3.2PQVAL_17 EQ 0) AND (S3.2PQVAL_18 EQ 0) AND (S3.2PQVAL_19 EQ 0) AND (S3.2PQVAL_20 EQ 0) AND (S3.2PQVAL_21 EQ 0) AND (S3.2PQVAL_22 EQ 0) AND (S3.2PQVAL_23 EQ 0) AND (S3.2PQVAL_24 EQ 0) AND (S3.2PQVAL_25 EQ 125))) [S3.P9MICROQ1 = 'être une personne ouverte d´esprit'];
IF (((S3.SUMVAL GE 200) AND (S3.SUMVAL LT 300) AND (S3.QVAL1 EQ 0) AND (S3.QVAL2 EQ 0) AND (S3.QVAL3 EQ 0) AND (S3.QVAL4 EQ 0) AND (S3.QVAL5 EQ 0) AND (S3.QVAL6 EQ 0) AND (S3.QVAL7 EQ 0) AND (S3.QVAL8 EQ 0) AND (S3.QVAL9 EQ 0) AND (S3.QVAL_10 EQ 0) AND (S3.QVAL_11 EQ 0) AND (S3.QVAL_12 EQ 0) AND (S3.QVAL_13 EQ 0) AND (S3.QVAL_14 EQ 0) AND (S3.QVAL_15 EQ 0) AND (S3.QVAL_16 EQ 0) AND (S3.QVAL_17 EQ 0) AND (S3.QVAL_18 EQ 0) AND (S3.QVAL_19 EQ 0) AND (S3.QVAL_20 EQ 0) AND (S3.QVAL_21 EQ 0) AND (S3.QVAL_22 EQ 0) AND (S3.QVAL_23 EQ 0) AND (S3.QVAL_24 EQ 0) AND (S3.QVAL_25 EQ 0) AND (S3.QVAL_26 EQ 102.7)) OR ((S3.2PQVAL1 EQ 0) AND (S3.2PQVAL2 EQ 0) AND (S3.2PQVAL3 EQ 0) AND (S3.2PQVAL4 EQ 0) AND (S3.2PQVAL5 EQ 0) AND (S3.2PQVAL6 EQ 0) AND (S3.2PQVAL7 EQ 0) AND (S3.2PQVAL8 EQ 0) AND (S3.2PQVAL9 EQ 0) AND (S3.2PQVAL_10 EQ 0) AND (S3.2PQVAL_11 EQ 0) AND (S3.2PQVAL_12 EQ 0) AND (S3.2PQVAL_13 EQ 0) AND (S3.2PQVAL_14 EQ 0) AND (S3.2PQVAL_15 EQ 0) AND (S3.2PQVAL_16 EQ 0) AND (S3.2PQVAL_17 EQ 0) AND (S3.2PQVAL_18 EQ 0) AND (S3.2PQVAL_19 EQ 0) AND (S3.2PQVAL_20 EQ 0) AND (S3.2PQVAL_21 EQ 0) AND (S3.2PQVAL_22 EQ 0) AND (S3.2PQVAL_23 EQ 0) AND (S3.2PQVAL_24 EQ 0) AND (S3.2PQVAL_25 EQ 0) AND (S3.2PQVAL_26 EQ 126))) [S3.P9MICROQ1 = 'avoir une vie excitante et stimulante'];
IF (((S3.SUMVAL GE 200) AND (S3.SUMVAL LT 300) AND (S3.QVAL1 EQ 0) AND (S3.QVAL2 EQ 0) AND (S3.QVAL3 EQ 0) AND (S3.QVAL4 EQ 0) AND (S3.QVAL5 EQ 0) AND (S3.QVAL6 EQ 0) AND (S3.QVAL7 EQ 0) AND (S3.QVAL8 EQ 0) AND (S3.QVAL9 EQ 0) AND (S3.QVAL_10 EQ 0) AND (S3.QVAL_11 EQ 0) AND (S3.QVAL_12 EQ 0) AND (S3.QVAL_13 EQ 0) AND (S3.QVAL_14 EQ 0) AND (S3.QVAL_15 EQ 0) AND (S3.QVAL_16 EQ 0) AND (S3.QVAL_17 EQ 0) AND (S3.QVAL_18 EQ 0) AND (S3.QVAL_19 EQ 0) AND (S3.QVAL_20 EQ 0) AND (S3.QVAL_21 EQ 0) AND (S3.QVAL_22 EQ 0) AND (S3.QVAL_23 EQ 0) AND (S3.QVAL_24 EQ 0) AND (S3.QVAL_25 EQ 0) AND (S3.QVAL_26 EQ 0) AND (S3.QVAL_27 EQ 102.8)) OR ((S3.2PQVAL1 EQ 0) AND (S3.2PQVAL2 EQ 0) AND (S3.2PQVAL3 EQ 0) AND (S3.2PQVAL4 EQ 0) AND (S3.2PQVAL5 EQ 0) AND (S3.2PQVAL6 EQ 0) AND (S3.2PQVAL7 EQ 0) AND (S3.2PQVAL8 EQ 0) AND (S3.2PQVAL9 EQ 0) AND (S3.2PQVAL_10 EQ 0) AND (S3.2PQVAL_11 EQ 0) AND (S3.2PQVAL_12 EQ 0) AND (S3.2PQVAL_13 EQ 0) AND (S3.2PQVAL_14 EQ 0) AND (S3.2PQVAL_15 EQ 0) AND (S3.2PQVAL_16 EQ 0) AND (S3.2PQVAL_17 EQ 0) AND (S3.2PQVAL_18 EQ 0) AND (S3.2PQVAL_19 EQ 0) AND (S3.2PQVAL_20 EQ 0) AND (S3.2PQVAL_21 EQ 0) AND (S3.2PQVAL_22 EQ 0) AND (S3.2PQVAL_23 EQ 0) AND (S3.2PQVAL_24

EQ 0) AND (S3.2PQVAL_25 EQ 0) AND (S3.2PQVAL_26 EQ 0) AND (S3.2PQVAL_27 EQ 127))) [S3.P9MICROQ1 = 'à prendre des décisions responsables et les maintenir'];
IF (((S3.SUMVAL GE 200) AND (S3.SUMVAL LT 300) AND (S3.QVAL1 EQ 0) AND (S3.QVAL2 EQ 0) AND (S3.QVAL3 EQ 0) AND (S3.QVAL4 EQ 0) AND (S3.QVAL5 EQ 0) AND (S3.QVAL6 EQ 0) AND (S3.QVAL7 EQ 0) AND (S3.QVAL8 EQ 0) AND (S3.QVAL9 EQ 0) AND (S3.QVAL_10 EQ 0) AND (S3.QVAL_11 EQ 0) AND (S3.QVAL_12 EQ 0) AND (S3.QVAL_13 EQ 0) AND (S3.QVAL_14 EQ 0) AND (S3.QVAL_15 EQ 0) AND (S3.QVAL_16 EQ 0) AND (S3.QVAL_17 EQ 0) AND (S3.QVAL_18 EQ 0) AND (S3.QVAL_19 EQ 0) AND (S3.QVAL_20 EQ 0) AND (S3.QVAL_21 EQ 0) AND (S3.QVAL_22 EQ 0) AND (S3.QVAL_23 EQ 0) AND (S3.QVAL_24 EQ 0) AND (S3.QVAL_25 EQ 0) AND (S3.QVAL_26 EQ 0) AND (S3.QVAL_27 EQ 0) AND (S3.QVAL_28 EQ 102.9)) OR ((S3.2PQVAL1 EQ 0) AND (S3.2PQVAL2 EQ 0) AND (S3.2PQVAL3 EQ 0) AND (S3.2PQVAL4 EQ 0) AND (S3.2PQVAL5 EQ 0) AND (S3.2PQVAL6 EQ 0) AND (S3.2PQVAL7 EQ 0) AND (S3.2PQVAL8 EQ 0) AND (S3.2PQVAL9 EQ 0) AND (S3.2PQVAL_10 EQ 0) AND (S3.2PQVAL_11 EQ 0) AND (S3.2PQVAL_12 EQ 0) AND (S3.2PQVAL_13 EQ 0) AND (S3.2PQVAL_14 EQ 0) AND (S3.2PQVAL_15 EQ 0) AND (S3.2PQVAL_16 EQ 0) AND (S3.2PQVAL_17 EQ 0) AND (S3.2PQVAL_18 EQ 0) AND (S3.2PQVAL_19 EQ 0) AND (S3.2PQVAL_20 EQ 0) AND (S3.2PQVAL_21 EQ 0) AND (S3.2PQVAL_22 EQ 0) AND (S3.2PQVAL_23 EQ 0) AND (S3.2PQVAL_24 EQ 0) AND (S3.2PQVAL_25 EQ 0) AND (S3.2PQVAL_26 EQ 0) AND (S3.2PQVAL_27 EQ 0) AND (S3.2PQVAL_28 EQ 128))) [S3.P9MICROQ1 = 'vous sentir en sécurité et à l´abri de dangers'];
IF (((S3.SUMVAL GE 200) AND (S3.SUMVAL LT 300) AND (S3.QVAL1 EQ 0) AND (S3.QVAL2 EQ 0) AND (S3.QVAL3 EQ 0) AND (S3.QVAL4 EQ 0) AND (S3.QVAL5 EQ 0) AND (S3.QVAL6 EQ 0) AND (S3.QVAL7 EQ 0) AND (S3.QVAL8 EQ 0) AND (S3.QVAL9 EQ 0) AND (S3.QVAL_10 EQ 0) AND (S3.QVAL_11 EQ 0) AND (S3.QVAL_12 EQ 0) AND (S3.QVAL_13 EQ 0) AND (S3.QVAL_14 EQ 0) AND (S3.QVAL_15 EQ 0) AND (S3.QVAL_16 EQ 0) AND (S3.QVAL_17 EQ 0) AND (S3.QVAL_18 EQ 0) AND (S3.QVAL_19 EQ 0) AND (S3.QVAL_20 EQ 0) AND (S3.QVAL_21 EQ 0) AND (S3.QVAL_22 EQ 0) AND (S3.QVAL_23 EQ 0) AND (S3.QVAL_24 EQ 0) AND (S3.QVAL_25 EQ 0) AND (S3.QVAL_26 EQ 0) AND (S3.QVAL_27 EQ 102.8) AND (S3.QVAL_28 EQ 0) AND (S3.QVAL_29 EQ 102.29)) OR ((S3.2PQVAL1 EQ 0) AND (S3.2PQVAL2 EQ 0) AND (S3.2PQVAL3 EQ 0) AND (S3.2PQVAL4 EQ 0) AND (S3.2PQVAL5 EQ 0) AND (S3.2PQVAL6 EQ 0) AND (S3.2PQVAL7 EQ 0) AND (S3.2PQVAL8 EQ 0) AND (S3.2PQVAL9 EQ 0) AND (S3.2PQVAL_10 EQ 0) AND (S3.2PQVAL_11 EQ 0) AND (S3.2PQVAL_12 EQ 0) AND (S3.2PQVAL_13 EQ 0) AND (S3.2PQVAL_14 EQ 0) AND (S3.2PQVAL_15 EQ 0) AND (S3.2PQVAL_16 EQ 0) AND (S3.2PQVAL_17 EQ 0) AND (S3.2PQVAL_18 EQ 0) AND (S3.2PQVAL_19 EQ 0) AND (S3.2PQVAL_20 EQ 0) AND (S3.2PQVAL_21 EQ 0) AND (S3.2PQVAL_22 EQ 0) AND (S3.2PQVAL_23 EQ 0) AND (S3.2PQVAL_24 EQ 0) AND (S3.2PQVAL_25 EQ 0) AND (S3.2PQVAL_26 EQ 0) AND (S3.2PQVAL_27 EQ 0) AND (S3.2PQVAL_28 EQ 0) AND (S3.2PQVAL_29 EQ 129))) [S3.P9MICROQ1 = 'être une personne aidante et aux services des autres'];
IF (((S3.SUMVAL GE 200) AND (S3.SUMVAL LT 300) AND (S3.QVAL1 EQ 101) AND (S3.QVAL2 EQ 102)) OR ((S3.2PQVAL1 EQ 101) AND (S3.2PQVAL2 EQ 102))) [S3.P9MICROQ2 = 'aimer et être aimé[LETTRE_E] par ceux qui vous entourent'];
IF (((S3.SUMVAL GE 200) AND (S3.SUMVAL LT 300) AND ((S3.QVAL1 EQ 101) OR (S3.QVAL2 EQ 102)) AND (S3.QVAL3 EQ 103)) OR (((S3.2PQVAL1 EQ 101) OR (S3.2PQVAL2 EQ 102)) AND (S3.2PQVAL3 EQ 103))) [S3.P9MICROQ2 = 'faire partie de quelquechose, d´un groupe'];

IF (((S3.SUMVAL GE 200) AND (S3.SUMVAL LT 300) AND ((S3.QVAL1 EQ 101) OR (S3.QVAL2 EQ 102) OR (S3.QVAL3 EQ 103)) AND (S3.QVAL4 EQ 104)) OR (((S3.2PQVAL1 EQ 101) OR (S3.2PQVAL2 EQ 102) OR (S3.2PQVAL3 EQ 103)) AND (S3.2PQVAL4 EQ 104))) [S3.P9MICROQ2 = 'vous occuper des autres'];
IF (((S3.SUMVAL GE 200) AND (S3.SUMVAL LT 300) AND ((S3.QVAL1 EQ 101) OR (S3.QVAL2 EQ 102) OR (S3.QVAL3 EQ 103) OR (S3.QVAL4 EQ 104)) AND (S3.QVAL5 EQ 105)) OR (((S3.2PQVAL1 EQ 101) OR (S3.2PQVAL2 EQ 102) OR (S3.2PQVAL3 EQ 103) OR (S3.2PQVAL4 EQ 104)) AND (S3.2PQVAL5 EQ 105))) [S3.P9MICROQ2 = 'être indépendant{e} et autonome'];
IF (((S3.SUMVAL GE 200) AND (S3.SUMVAL LT 300) AND ((S3.QVAL1 EQ 101) OR (S3.QVAL2 EQ 102) OR (S3.QVAL3 EQ 103) OR (S3.QVAL4 EQ 104) OR (S3.QVAL5 EQ 105)) AND (S3.QVAL6 EQ 106)) OR (((S3.2PQVAL1 EQ 101) OR (S3.2PQVAL2 EQ 102) OR (S3.2PQVAL3 EQ 103) OR (S3.2PQVAL4 EQ 104) OR (S3.2PQVAL5 EQ 105)) AND (S3.2PQVAL6 EQ 106))) [S3.P9MICROQ2 = 'apprécier la beauté en vous et autour de vous'];
IF (((S3.SUMVAL GE 200) AND (S3.SUMVAL LT 300) AND ((S3.QVAL1 EQ 101) OR (S3.QVAL2 EQ 102) OR (S3.QVAL3 EQ 103) OR (S3.QVAL4 EQ 104) OR (S3.QVAL5 EQ 105) OR (S3.QVAL6 EQ 106)) AND (S3.QVAL7 EQ 107)) OR (((S3.2PQVAL1 EQ 101) OR (S3.2PQVAL2 EQ 102) OR (S3.2PQVAL3 EQ 103) OR (S3.2PQVAL4 EQ 104) OR (S3.2PQVAL5 EQ 105) OR (S3.2PQVAL6 EQ 106)) AND (S3.2PQVAL7 EQ 107))) [S3.P9MICROQ2 = 'être sensible aux autres et agir pour leur bien'];
IF (((S3.SUMVAL GE 200) AND (S3.SUMVAL LT 300) AND ((S3.QVAL1 EQ 101) OR (S3.QVAL2 EQ 102) OR (S3.QVAL3 EQ 103) OR (S3.QVAL4 EQ 104) OR (S3.QVAL5 EQ 105) OR (S3.QVAL6 EQ 106) OR (S3.QVAL7 EQ 107)) AND (S3.QVAL8 EQ 108)) OR (((S3.2PQVAL1 EQ 101) OR (S3.2PQVAL2 EQ 102) OR (S3.2PQVAL3 EQ 103) OR (S3.2PQVAL4 EQ 104) OR (S3.2PQVAL5 EQ 105) OR (S3.2PQVAL6 EQ 106) OR (S3.2PQVAL7 EQ 107)) AND (S3.2PQVAL8 EQ 108))) [S3.P9MICROQ2 = 'être en mesure de faire des compromis'];
IF (((S3.SUMVAL GE 200) AND (S3.SUMVAL LT 300) AND ((S3.QVAL1 EQ 101) OR (S3.QVAL2 EQ 102) OR (S3.QVAL3 EQ 103) OR (S3.QVAL4 EQ 104) OR (S3.QVAL5 EQ 105) OR (S3.QVAL6 EQ 106) OR (S3.QVAL7 EQ 107) OR (S3.QVAL8 EQ 108)) AND (S3.QVAL9 EQ 109)) OR (((S3.2PQVAL1 EQ 101) OR (S3.2PQVAL2 EQ 102) OR (S3.2PQVAL3 EQ 103) OR (S3.2PQVAL4 EQ 104) OR (S3.2PQVAL5 EQ 105) OR (S3.2PQVAL6 EQ 106) OR (S3.2PQVAL7 EQ 107) OR (S3.2PQVAL8 EQ 108)) AND (S3.2PQVAL9 EQ 109))) [S3.P9MICROQ2 = 'avoir une vie plaisante et confortable'];
IF (((S3.SUMVAL GE 200) AND (S3.SUMVAL LT 300) AND ((S3.QVAL1 EQ 101) OR (S3.QVAL2 EQ 102) OR (S3.QVAL3 EQ 103) OR (S3.QVAL4 EQ 104) OR (S3.QVAL5 EQ 105) OR (S3.QVAL6 EQ 106) OR (S3.QVAL7 EQ 107) OR (S3.QVAL8 EQ 108) OR (S3.QVAL9 EQ 109)) AND (S3.QVAL_10 EQ 101.1)) OR (((S3.2PQVAL1 EQ 101) OR (S3.2PQVAL2 EQ 102) OR (S3.2PQVAL3 EQ 103) OR (S3.2PQVAL4 EQ 104) OR (S3.2PQVAL5 EQ 105) OR (S3.2PQVAL6 EQ 106) OR (S3.2PQVAL7 EQ 107) OR (S3.2PQVAL8 EQ 108) OR (S3.2PQVAL9 EQ 109)) AND (S3.2PQVAL_10 EQ 110))) [S3.P9MICROQ2 = 'être brave dans l´adversité'];
IF (((S3.SUMVAL GE 200) AND (S3.SUMVAL LT 300) AND ((S3.QVAL1 EQ 101) OR (S3.QVAL2 EQ 102) OR (S3.QVAL3 EQ 103) OR (S3.QVAL4 EQ 104) OR (S3.QVAL5 EQ 105) OR (S3.QVAL6 EQ 106) OR (S3.QVAL7 EQ 107) OR (S3.QVAL8 EQ 108) OR (S3.QVAL9 EQ 109) OR (S3.QVAL_10 EQ 101.1)) AND (S3.QVAL_11 EQ 101.2)) OR (((S3.2PQVAL1 EQ 101) OR (S3.2PQVAL2 EQ 102) OR (S3.2PQVAL3 EQ 103) OR (S3.2PQVAL4 EQ 104) OR (S3.2PQVAL5 EQ 105) OR (S3.2PQVAL6 EQ 106) OR (S3.2PQVAL7 EQ 107) OR (S3.2PQVAL8 EQ 108) OR (S3.2PQVAL9 EQ 109) OR (S3.2PQVAL_10 EQ 110)) AND (S3.2PQVAL_11 EQ 111))) [S3.P9MICROQ2 = 'avoir des idées nouvelles, à créer'];

IF (((S3.SUMVAL GE 200) AND (S3.SUMVAL LT 300) AND ((S3.QVAL1 EQ 101) OR (S3.QVAL2 EQ 102) OR (S3.QVAL3 EQ 103) OR (S3.QVAL4 EQ 104) OR (S3.QVAL5 EQ 105) OR (S3.QVAL6 EQ 106) OR (S3.QVAL7 EQ 107) OR (S3.QVAL8 EQ 108) OR (S3.QVAL9 EQ 109) OR (S3.QVAL_10 EQ 101.1) OR (S3.QVAL_11 EQ 101.2)) AND (S3.QVAL_12 EQ 101.3)) OR (((S3.2PQVAL1 EQ 101) OR (S3.2PQVAL2 EQ 102) OR (S3.2PQVAL3 EQ 103) OR (S3.2PQVAL4 EQ 104) OR (S3.2PQVAL5 EQ 105) OR (S3.2PQVAL6 EQ 106) OR (S3.2PQVAL7 EQ 107) OR (S3.2PQVAL8 EQ 108) OR (S3.2PQVAL9 EQ 109) OR (S3.2PQVAL_10 EQ 110) OR (S3.2PQVAL_11 EQ 111)) AND (S3.2PQVAL_12 EQ 112))) [S3.P9MICROQ2 = 'évoluer continuellement'];
IF (((S3.SUMVAL GE 200) AND (S3.SUMVAL LT 300) AND ((S3.QVAL1 EQ 101) OR (S3.QVAL2 EQ 102) OR (S3.QVAL3 EQ 103) OR (S3.QVAL4 EQ 104) OR (S3.QVAL5 EQ 105) OR (S3.QVAL6 EQ 106) OR (S3.QVAL7 EQ 107) OR (S3.QVAL8 EQ 108) OR (S3.QVAL9 EQ 109) OR (S3.QVAL_10 EQ 101.1) OR (S3.QVAL_11 EQ 101.2) OR (S3.QVAL_12 EQ 101.3)) AND (S3.QVAL_13 EQ 101.4)) OR (((S3.2PQVAL1 EQ 101) OR (S3.2PQVAL2 EQ 102) OR (S3.2PQVAL3 EQ 103) OR (S3.2PQVAL4 EQ 104) OR (S3.2PQVAL5 EQ 105) OR (S3.2PQVAL6 EQ 106) OR (S3.2PQVAL7 EQ 107) OR (S3.2PQVAL8 EQ 108) OR (S3.2PQVAL9 EQ 109) OR (S3.2PQVAL_10 EQ 110) OR (S3.2PQVAL_11 EQ 111) OR (S3.2PQVAL_12 EQ 112)) AND (S3.2PQVAL_13 EQ 113))) [S3.P9MICROQ2 = 'explorer et apprendre de nouvelles choses'];
IF (((S3.SUMVAL GE 200) AND (S3.SUMVAL LT 300) AND ((S3.QVAL1 EQ 101) OR (S3.QVAL2 EQ 102) OR (S3.QVAL3 EQ 103) OR (S3.QVAL4 EQ 104) OR (S3.QVAL5 EQ 105) OR (S3.QVAL6 EQ 106) OR (S3.QVAL7 EQ 107) OR (S3.QVAL8 EQ 108) OR (S3.QVAL9 EQ 109) OR (S3.QVAL_10 EQ 101.1) OR (S3.QVAL_11 EQ 101.2) OR (S3.QVAL_12 EQ 101.3) OR (S3.QVAL_13 EQ 101.4)) AND (S3.QVAL_14 EQ 101.5)) OR (((S3.2PQVAL1 EQ 101) OR (S3.2PQVAL2 EQ 102) OR (S3.2PQVAL3 EQ 103) OR (S3.2PQVAL4 EQ 104) OR (S3.2PQVAL5 EQ 105) OR (S3.2PQVAL6 EQ 106) OR (S3.2PQVAL7 EQ 107) OR (S3.2PQVAL8 EQ 108) OR (S3.2PQVAL9 EQ 109) OR (S3.2PQVAL_10 EQ 110) OR (S3.2PQVAL_11 EQ 111) OR (S3.2PQVAL_12 EQ 112) OR (S3.2PQVAL_13 EQ 113)) AND (S3.2PQVAL_14 EQ 114))) [S3.P9MICROQ2 = 'maintenir une attitude positive et optimiste peu importe ce qu´il advient'];
IF (((S3.SUMVAL GE 200) AND (S3.SUMVAL LT 300) AND ((S3.QVAL1 EQ 101) OR (S3.QVAL2 EQ 102) OR (S3.QVAL3 EQ 103) OR (S3.QVAL4 EQ 104) OR (S3.QVAL5 EQ 105) OR (S3.QVAL6 EQ 106) OR (S3.QVAL7 EQ 107) OR (S3.QVAL8 EQ 108) OR (S3.QVAL9 EQ 109) OR (S3.QVAL_10 EQ 101.1) OR (S3.QVAL_11 EQ 101.2) OR (S3.QVAL_12 EQ 101.3) OR (S3.QVAL_13 EQ 101.4) OR (S3.QVAL_14 EQ 101.5)) AND (S3.QVAL_15 EQ 101.6)) OR (((S3.2PQVAL1 EQ 101) OR (S3.2PQVAL2 EQ 102) OR (S3.2PQVAL3 EQ 103) OR (S3.2PQVAL4 EQ 104) OR (S3.2PQVAL5 EQ 105) OR (S3.2PQVAL6 EQ 106) OR (S3.2PQVAL7 EQ 107) OR (S3.2PQVAL8 EQ 108) OR (S3.2PQVAL9 EQ 109) OR (S3.2PQVAL_10 EQ 110) OR (S3.2PQVAL_11 EQ 111) OR (S3.2PQVAL_12 EQ 112) OR (S3.2PQVAL_13 EQ 113) OR (S3.2PQVAL_14 EQ 114)) AND (S3.2PQVAL_15 EQ 115))) [S3.P9MICROQ2 = 'vous sentir bien dans votre peau'];
IF (((S3.SUMVAL GE 200) AND (S3.SUMVAL LT 300) AND ((S3.QVAL1 EQ 101) OR (S3.QVAL2 EQ 102) OR (S3.QVAL3 EQ 103) OR (S3.QVAL4 EQ 104) OR (S3.QVAL5 EQ 105) OR (S3.QVAL6 EQ 106) OR (S3.QVAL7 EQ 107) OR (S3.QVAL8 EQ 108) OR (S3.QVAL9 EQ 109) OR (S3.QVAL_10 EQ 101.1) OR (S3.QVAL_11 EQ 101.2) OR (S3.QVAL_12 EQ 101.3) OR (S3.QVAL_13 EQ 101.4) OR (S3.QVAL_14 EQ 101.5) OR (S3.QVAL_15 EQ 101.6)) AND (S3.QVAL_16 EQ 101.7)) OR (((S3.2PQVAL1 EQ 101) OR (S3.2PQVAL2 EQ 102) OR (S3.2PQVAL3 EQ 103) OR (S3.2PQVAL4 EQ 104) OR (S3.2PQVAL5 EQ 105) OR (S3.2PQVAL6 EQ 106) OR (S3.2PQVAL7 EQ 107) OR (S3.2PQVAL8 EQ 108) OR (S3.2PQVAL9 EQ 109) OR (S3.2PQVAL_10 EQ 110) OR (S3.2PQVAL_11 EQ 111) OR (S3.2PQVAL_12 EQ 112) OR (S3.2PQVAL_13 EQ 113) OR (S3.2PQVAL_14 EQ 114) OR

(S3.2PQVAL_15 EQ 115)) AND (S3.2PQVAL_16 EQ 116))) [S3.P9MICROQ2 = 'avoir une famille comblée et heureuse'];
IF (((S3.SUMVAL GE 200) AND (S3.SUMVAL LT 300) AND ((S3.QVAL1 EQ 101) OR (S3.QVAL2 EQ 102) OR (S3.QVAL3 EQ 103) OR (S3.QVAL4 EQ 104) OR (S3.QVAL5 EQ 105) OR (S3.QVAL6 EQ 106) OR (S3.QVAL7 EQ 107) OR (S3.QVAL8 EQ 108) OR (S3.QVAL9 EQ 109) OR (S3.QVAL_10 EQ 101.1) OR (S3.QVAL_11 EQ 101.2) OR (S3.QVAL_12 EQ 101.3) OR (S3.QVAL_13 EQ 101.4) OR (S3.QVAL_14 EQ 101.5) OR (S3.QVAL_15 EQ 101.6) OR (S3.QVAL_16 EQ 101.7)) AND (S3.QVAL_17 EQ 101.8)) OR (((S3.2PQVAL1 EQ 101) OR (S3.2PQVAL2 EQ 102) OR (S3.2PQVAL3 EQ 103) OR (S3.2PQVAL4 EQ 104) OR (S3.2PQVAL5 EQ 105) OR (S3.2PQVAL6 EQ 106) OR (S3.2PQVAL7 EQ 107) OR (S3.2PQVAL8 EQ 108) OR (S3.2PQVAL9 EQ 109) OR (S3.2PQVAL_10 EQ 110) OR (S3.2PQVAL_11 EQ 111) OR (S3.2PQVAL_12 EQ 112) OR (S3.2PQVAL_13 EQ 113) OR (S3.2PQVAL_14 EQ 114) OR (S3.2PQVAL_15 EQ 115) OR (S3.2PQVAL_16 EQ 116)) AND (S3.2PQVAL_17 EQ 117))) [S3.P9MICROQ2 = 'être fiable, être quelqu´un de confiance'];
IF (((S3.SUMVAL GE 200) AND (S3.SUMVAL LT 300) AND ((S3.QVAL1 EQ 101) OR (S3.QVAL2 EQ 102) OR (S3.QVAL3 EQ 103) OR (S3.QVAL4 EQ 104) OR (S3.QVAL5 EQ 105) OR (S3.QVAL6 EQ 106) OR (S3.QVAL7 EQ 107) OR (S3.QVAL8 EQ 108) OR (S3.QVAL9 EQ 109) OR (S3.QVAL_10 EQ 101.1) OR (S3.QVAL_11 EQ 101.2) OR (S3.QVAL_12 EQ 101.3) OR (S3.QVAL_13 EQ 101.4) OR (S3.QVAL_14 EQ 101.5) OR (S3.QVAL_15 EQ 101.6) OR (S3.QVAL_16 EQ 101.7) OR (S3.QVAL_17 EQ 101.8)) AND (S3.QVAL_18 EQ 101.9)) OR (((S3.2PQVAL1 EQ 101) OR (S3.2PQVAL2 EQ 102) OR (S3.2PQVAL3 EQ 103) OR (S3.2PQVAL4 EQ 104) OR (S3.2PQVAL5 EQ 105) OR (S3.2PQVAL6 EQ 106) OR (S3.2PQVAL7 EQ 107) OR (S3.2PQVAL8 EQ 108) OR (S3.2PQVAL9 EQ 109) OR (S3.2PQVAL_10 EQ 110) OR (S3.2PQVAL_11 EQ 111) OR (S3.2PQVAL_12 EQ 112) OR (S3.2PQVAL_13 EQ 113) OR (S3.2PQVAL_14 EQ 114) OR (S3.2PQVAL_15 EQ 115) OR (S3.2PQVAL_16 EQ 116) OR (S3.2PQVAL_17 EQ 117)) AND (S3.2PQVAL_18 EQ 118))) [S3.P9MICROQ2 = 'vous ajuster aux nouvelles circonstances qui se présentent'];
IF (((S3.SUMVAL GE 200) AND (S3.SUMVAL LT 300) AND ((S3.QVAL1 EQ 101) OR (S3.QVAL2 EQ 102) OR (S3.QVAL3 EQ 103) OR (S3.QVAL4 EQ 104) OR (S3.QVAL5 EQ 105) OR (S3.QVAL6 EQ 106) OR (S3.QVAL7 EQ 107) OR (S3.QVAL8 EQ 108) OR (S3.QVAL9 EQ 109) OR (S3.QVAL_10 EQ 101.1) OR (S3.QVAL_11 EQ 101.2) OR (S3.QVAL_12 EQ 101.3) OR (S3.QVAL_13 EQ 101.4) OR (S3.QVAL_14 EQ 101.5) OR (S3.QVAL_15 EQ 101.6) OR (S3.QVAL_16 EQ 101.7) OR (S3.QVAL_17 EQ 101.8) OR (S3.QVAL_18 EQ 101.9)) AND (S3.QVAL_19 EQ 101.19))OR (((S3.2PQVAL1 EQ 101) OR (S3.2PQVAL2 EQ 102) OR (S3.2PQVAL3 EQ 103) OR (S3.2PQVAL4 EQ 104) OR (S3.2PQVAL5 EQ 105) OR (S3.2PQVAL6 EQ 106) OR (S3.2PQVAL7 EQ 107) OR (S3.2PQVAL8 EQ 108) OR (S3.2PQVAL9 EQ 109) OR (S3.2PQVAL_10 EQ 110) OR (S3.2PQVAL_11 EQ 111) OR (S3.2PQVAL_12 EQ 112) OR (S3.2PQVAL_13 EQ 113) OR (S3.2PQVAL_14 EQ 114) OR (S3.2PQVAL_15 EQ 115) OR (S3.2PQVAL_16 EQ 116) OR (S3.2PQVAL_17 EQ 117) OR (S3.2PQVAL_18 EQ 118)) AND (S3.2PQVAL_19 EQ 119))) [S3.P9MICROQ2 = 'donner sans attendre de recevoir en retour'];
IF (((S3.SUMVAL GE 200) AND (S3.SUMVAL LT 300) AND ((S3.QVAL1 EQ 101) OR (S3.QVAL2 EQ 102) OR (S3.QVAL3 EQ 103) OR (S3.QVAL4 EQ 104) OR (S3.QVAL5 EQ 105) OR (S3.QVAL6 EQ 106) OR (S3.QVAL7 EQ 107) OR (S3.QVAL8 EQ 108) OR (S3.QVAL9 EQ 109) OR (S3.QVAL_10 EQ 101.1) OR (S3.QVAL_11 EQ 101.2) OR (S3.QVAL_12 EQ 101.3) OR (S3.QVAL_13 EQ 101.4) OR (S3.QVAL_14 EQ 101.5) OR (S3.QVAL_15 EQ 101.6) OR (S3.QVAL_16 EQ 101.7) OR (S3.QVAL_17 EQ 101.8) OR (S3.QVAL_18 EQ 101.9) OR (S3.QVAL_19 EQ 101.19)) AND (S3.QVAL_20 EQ 102.1)) OR (((S3.2PQVAL1 EQ 101) OR (S3.2PQVAL2 EQ 102) OR (S3.2PQVAL3 EQ 103) OR (S3.2PQVAL4 EQ 104) OR (S3.2PQVAL5 EQ 105) OR (S3.2PQVAL6 EQ 106) OR (S3.2PQVAL7 EQ 107) OR (S3.2PQVAL8 EQ 108) OR (S3.2PQVAL9 EQ 109) OR (S3.2PQVAL_10 EQ 110) OR

(S3.2PQVAL_11 EQ 111) OR (S3.2PQVAL_12 EQ 112) OR (S3.2PQVAL_13 EQ 113) OR (S3.2PQVAL_14 EQ 114) OR (S3.2PQVAL_15 EQ 115) OR (S3.2PQVAL_16 EQ 116) OR (S3.2PQVAL_17 EQ 117) OR (S3.2PQVAL_18 EQ 118) OR (S3.2PQVAL_19 EQ 119)) AND (S3.2PQVAL_20 EQ 120))) [S3.P9MICROQ2 = 'être une personne intelligente et à garder un mental actif'];
IF (((S3.SUMVAL GE 200) AND (S3.SUMVAL LT 300) AND ((S3.QVAL1 EQ 101) OR (S3.QVAL2 EQ 102) OR (S3.QVAL3 EQ 103) OR (S3.QVAL4 EQ 104) OR (S3.QVAL5 EQ 105) OR (S3.QVAL6 EQ 106) OR (S3.QVAL7 EQ 107) OR (S3.QVAL8 EQ 108) OR (S3.QVAL9 EQ 109) OR (S3.QVAL_10 EQ 101.1) OR (S3.QVAL_11 EQ 101.2) OR (S3.QVAL_12 EQ 101.3) OR (S3.QVAL_13 EQ 101.4) OR (S3.QVAL_14 EQ 101.5) OR (S3.QVAL_15 EQ 101.6) OR (S3.QVAL_16 EQ 101.7) OR (S3.QVAL_17 EQ 101.8) OR (S3.QVAL_18 EQ 101.9) OR (S3.QVAL_19 EQ 101.19) OR (S3.QVAL_20 EQ 102.1)) AND (S3.QVAL_21 EQ 102.2)) OR (((S3.2PQVAL1 EQ 101) OR (S3.2PQVAL2 EQ 102) OR (S3.2PQVAL3 EQ 103) OR (S3.2PQVAL4 EQ 104) OR (S3.2PQVAL5 EQ 105) OR (S3.2PQVAL6 EQ 106) OR (S3.2PQVAL7 EQ 107) OR (S3.2PQVAL8 EQ 108) OR (S3.2PQVAL9 EQ 109) OR (S3.2PQVAL_10 EQ 110) OR (S3.2PQVAL_11 EQ 111) OR (S3.2PQVAL_12 EQ 112) OR (S3.2PQVAL_13 EQ 113) OR (S3.2PQVAL_14 EQ 114) OR (S3.2PQVAL_15 EQ 115) OR (S3.2PQVAL_16 EQ 116) OR (S3.2PQVAL_17 EQ 117) OR (S3.2PQVAL_18 EQ 118) OR (S3.2PQVAL_19 EQ 119) OR (S3.2PQVAL_20 EQ 120)) AND (S3.2PQVAL_21 EQ 121))) [S3.P9MICROQ2 = 'inspirer et guider les autres'];
IF (((S3.SUMVAL GE 200) AND (S3.SUMVAL LT 300) AND ((S3.QVAL1 EQ 101) OR (S3.QVAL2 EQ 102) OR (S3.QVAL3 EQ 103) OR (S3.QVAL4 EQ 104) OR (S3.QVAL5 EQ 105) OR (S3.QVAL6 EQ 106) OR (S3.QVAL7 EQ 107) OR (S3.QVAL8 EQ 108) OR (S3.QVAL9 EQ 109) OR (S3.QVAL_10 EQ 101.1) OR (S3.QVAL_11 EQ 101.2) OR (S3.QVAL_12 EQ 101.3) OR (S3.QVAL_13 EQ 101.4) OR (S3.QVAL_14 EQ 101.5) OR (S3.QVAL_15 EQ 101.6) OR (S3.QVAL_16 EQ 101.7) OR (S3.QVAL_17 EQ 101.8) OR (S3.QVAL_18 EQ 101.9) OR (S3.QVAL_19 EQ 101.19) OR (S3.QVAL_20 EQ 102.1) OR (S3.QVAL_21 EQ 102.2)) AND (S3.QVAL_22 EQ 102.3)) OR (((S3.2PQVAL1 EQ 101) OR (S3.2PQVAL2 EQ 102) OR (S3.2PQVAL3 EQ 103) OR (S3.2PQVAL4 EQ 104) OR (S3.2PQVAL5 EQ 105) OR (S3.2PQVAL6 EQ 106) OR (S3.2PQVAL7 EQ 107) OR (S3.2PQVAL8 EQ 108) OR (S3.2PQVAL9 EQ 109) OR (S3.2PQVAL_10 EQ 110) OR (S3.2PQVAL_11 EQ 111) OR (S3.2PQVAL_12 EQ 112) OR (S3.2PQVAL_13 EQ 113) OR (S3.2PQVAL_14 EQ 114) OR (S3.2PQVAL_15 EQ 115) OR (S3.2PQVAL_16 EQ 116) OR (S3.2PQVAL_17 EQ 117) OR (S3.2PQVAL_18 EQ 118) OR (S3.2PQVAL_19 EQ 119) OR (S3.2PQVAL_20 EQ 120) OR (S3.2PQVAL_21 EQ 121)) AND (S3.2PQVAL_22 EQ 122))) [S3.P9MICROQ2 = 'être libre et sans limitations non-désirées'];
IF (((S3.SUMVAL GE 200) AND (S3.SUMVAL LT 300) AND ((S3.QVAL1 EQ 101) OR (S3.QVAL2 EQ 102) OR (S3.QVAL3 EQ 103) OR (S3.QVAL4 EQ 104) OR (S3.QVAL5 EQ 105) OR (S3.QVAL6 EQ 106) OR (S3.QVAL7 EQ 107) OR (S3.QVAL8 EQ 108) OR (S3.QVAL9 EQ 109) OR (S3.QVAL_10 EQ 101.1) OR (S3.QVAL_11 EQ 101.2) OR (S3.QVAL_12 EQ 101.3) OR (S3.QVAL_13 EQ 101.4) OR (S3.QVAL_14 EQ 101.5) OR (S3.QVAL_15 EQ 101.6) OR (S3.QVAL_16 EQ 101.7) OR (S3.QVAL_17 EQ 101.8) OR (S3.QVAL_18 EQ 101.9) OR (S3.QVAL_19 EQ 101.19) OR (S3.QVAL_20 EQ 102.1) OR (S3.QVAL_21 EQ 102.2) OR (S3.QVAL_22 EQ 102.3)) AND (S3.QVAL_23 EQ 102.4)) OR (((S3.2PQVAL1 EQ 101) OR (S3.2PQVAL2 EQ 102) OR (S3.2PQVAL3 EQ 103) OR (S3.2PQVAL4 EQ 104) OR (S3.2PQVAL5 EQ 105) OR (S3.2PQVAL6 EQ 106) OR (S3.2PQVAL7 EQ 107) OR (S3.2PQVAL8 EQ 108) OR (S3.2PQVAL9 EQ 109) OR (S3.2PQVAL_10 EQ 110) OR (S3.2PQVAL_11 EQ 111) OR (S3.2PQVAL_12 EQ 112) OR (S3.2PQVAL_13 EQ 113) OR (S3.2PQVAL_14 EQ 114) OR (S3.2PQVAL_15 EQ 115) OR (S3.2PQVAL_16 EQ 116) OR (S3.2PQVAL_17 EQ 117) OR (S3.2PQVAL_18 EQ 118) OR (S3.2PQVAL_19 EQ 119) OR (S3.2PQVAL_20 EQ 120) OR (S3.2PQVAL_21 EQ 121) OR (S3.2PQVAL_22 EQ

122)) AND (S3.2PQVAL_23 EQ 123))) [S3.P9MICROQ2 = 'être authentique envers vous-même et dans vos relations'];
IF (((S3.SUMVAL GE 200) AND (S3.SUMVAL LT 300) AND ((S3.QVAL1 EQ 101) OR (S3.QVAL2 EQ 102) OR (S3.QVAL3 EQ 103) OR (S3.QVAL4 EQ 104) OR (S3.QVAL5 EQ 105) OR (S3.QVAL6 EQ 106) OR (S3.QVAL7 EQ 107) OR (S3.QVAL8 EQ 108) OR (S3.QVAL9 EQ 109) OR (S3.QVAL_10 EQ 101.1) OR (S3.QVAL_11 EQ 101.2) OR (S3.QVAL_12 EQ 101.3) OR (S3.QVAL_13 EQ 101.4) OR (S3.QVAL_14 EQ 101.5) OR (S3.QVAL_15 EQ 101.6) OR (S3.QVAL_16 EQ 101.7) OR (S3.QVAL_17 EQ 101.8) OR (S3.QVAL_18 EQ 101.9) OR (S3.QVAL_19 EQ 101.19) OR (S3.QVAL_20 EQ 102.1) OR (S3.QVAL_21 EQ 102.2) OR (S3.QVAL_22 EQ 102.3) OR (S3.QVAL_23 EQ 102.4)) AND (S3.QVAL_24 EQ 102.5)) OR (((S3.2PQVAL1 EQ 101) OR (S3.2PQVAL2 EQ 102) OR (S3.2PQVAL3 EQ 103) OR (S3.2PQVAL4 EQ 104) OR (S3.2PQVAL5 EQ 105) OR (S3.2PQVAL6 EQ 106) OR (S3.2PQVAL7 EQ 107) OR (S3.2PQVAL8 EQ 108) OR (S3.2PQVAL9 EQ 109) OR (S3.2PQVAL_10 EQ 110) OR (S3.2PQVAL_11 EQ 111) OR (S3.2PQVAL_12 EQ 112) OR (S3.2PQVAL_13 EQ 113) OR (S3.2PQVAL_14 EQ 114) OR (S3.2PQVAL_15 EQ 115) OR (S3.2PQVAL_16 EQ 116) OR (S3.2PQVAL_17 EQ 117) OR (S3.2PQVAL_18 EQ 118) OR (S3.2PQVAL_19 EQ 119) OR (S3.2PQVAL_20 EQ 120) OR (S3.2PQVAL_21 EQ 121) OR (S3.2PQVAL_22 EQ 122) OR (S3.2PQVAL_23 EQ 123)) AND (S3.2PQVAL_24 EQ 124))) [S3.P9MICROQ2 = 'être à votre affaire et prendre les bonnes décisions'];
IF (((S3.SUMVAL GE 200) AND (S3.SUMVAL LT 300) AND ((S3.QVAL1 EQ 101) OR (S3.QVAL2 EQ 102) OR (S3.QVAL3 EQ 103) OR (S3.QVAL4 EQ 104) OR (S3.QVAL5 EQ 105) OR (S3.QVAL6 EQ 106) OR (S3.QVAL7 EQ 107) OR (S3.QVAL8 EQ 108) OR (S3.QVAL9 EQ 109) OR (S3.QVAL_10 EQ 101.1) OR (S3.QVAL_11 EQ 101.2) OR (S3.QVAL_12 EQ 101.3) OR (S3.QVAL_13 EQ 101.4) OR (S3.QVAL_14 EQ 101.5) OR (S3.QVAL_15 EQ 101.6) OR (S3.QVAL_16 EQ 101.7) OR (S3.QVAL_17 EQ 101.8) OR (S3.QVAL_18 EQ 101.9) OR (S3.QVAL_19 EQ 101.19) OR (S3.QVAL_20 EQ 102.1) OR (S3.QVAL_21 EQ 102.2) OR (S3.QVAL_22 EQ 102.3) OR (S3.QVAL_23 EQ 102.4) OR (S3.QVAL_24 EQ 102.5)) AND (S3.QVAL_25 EQ 102.6)) OR (((S3.2PQVAL1 EQ 101) OR (S3.2PQVAL2 EQ 102) OR (S3.2PQVAL3 EQ 103) OR (S3.2PQVAL4 EQ 104) OR (S3.2PQVAL5 EQ 105) OR (S3.2PQVAL6 EQ 106) OR (S3.2PQVAL7 EQ 107) OR (S3.2PQVAL8 EQ 108) OR (S3.2PQVAL9 EQ 109) OR (S3.2PQVAL_10 EQ 110) OR (S3.2PQVAL_11 EQ 111) OR (S3.2PQVAL_12 EQ 112) OR (S3.2PQVAL_13 EQ 113) OR (S3.2PQVAL_14 EQ 114) OR (S3.2PQVAL_15 EQ 115) OR (S3.2PQVAL_16 EQ 116) OR (S3.2PQVAL_17 EQ 117) OR (S3.2PQVAL_18 EQ 118) OR (S3.2PQVAL_19 EQ 119) OR (S3.2PQVAL_20 EQ 120) OR (S3.2PQVAL_21 EQ 121) OR (S3.2PQVAL_22 EQ 122) OR (S3.2PQVAL_23 EQ 123) OR (S3.2PQVAL_24 EQ 124)) AND (S3.2PQVAL_25 EQ 125))) [S3.P9MICROQ2 = 'être une personne ouverte d´esprit'];
IF (((S3.SUMVAL GE 200) AND (S3.SUMVAL LT 300) AND ((S3.QVAL1 EQ 101) OR (S3.QVAL2 EQ 102) OR (S3.QVAL3 EQ 103) OR (S3.QVAL4 EQ 104) OR (S3.QVAL5 EQ 105) OR (S3.QVAL6 EQ 106) OR (S3.QVAL7 EQ 107) OR (S3.QVAL8 EQ 108) OR (S3.QVAL9 EQ 109) OR (S3.QVAL_10 EQ 101.1) OR (S3.QVAL_11 EQ 101.2) OR (S3.QVAL_12 EQ 101.3) OR (S3.QVAL_13 EQ 101.4) OR (S3.QVAL_14 EQ 101.5) OR (S3.QVAL_15 EQ 101.6) OR (S3.QVAL_16 EQ 101.7) OR (S3.QVAL_17 EQ 101.8) OR (S3.QVAL_18 EQ 101.9) OR (S3.QVAL_19 EQ 101.19) OR (S3.QVAL_20 EQ 102.1) OR (S3.QVAL_21 EQ 102.2) OR (S3.QVAL_22 EQ 102.3) OR (S3.QVAL_23 EQ 102.4) OR (S3.QVAL_24 EQ 102.5) OR (S3.QVAL_25 EQ 102.6)) AND (S3.QVAL_26 EQ 102.7)) OR (((S3.2PQVAL1 EQ 101) OR (S3.2PQVAL2 EQ 102) OR (S3.2PQVAL3 EQ 103) OR (S3.2PQVAL4 EQ 104) OR (S3.2PQVAL5 EQ 105) OR (S3.2PQVAL6 EQ 106) OR (S3.2PQVAL7 EQ 107) OR (S3.2PQVAL8 EQ 108) OR (S3.2PQVAL9 EQ 109) OR (S3.2PQVAL_10 EQ 110) OR (S3.2PQVAL_11 EQ 111) OR (S3.2PQVAL_12 EQ 112) OR (S3.2PQVAL_13 EQ 113) OR (S3.2PQVAL_14 EQ 114) OR (S3.2PQVAL_15 EQ 115) OR

(S3.2PQVAL_16 EQ 116) OR (S3.2PQVAL_17 EQ 117) OR (S3.2PQVAL_18 EQ 118) OR (S3.2PQVAL_19 EQ 119) OR (S3.2PQVAL_20 EQ 120) OR (S3.2PQVAL_21 EQ 121) OR (S3.2PQVAL_22 EQ 122) OR (S3.2PQVAL_23 EQ 123) OR (S3.2PQVAL_24 EQ 124) OR (S3.2PQVAL_25 EQ 125)) AND (S3.2PQVAL_26 EQ 126))) [S3.P9MICROQ2 = 'avoir une vie excitante et stimulante'];
IF (((S3.SUMVAL GE 200) AND (S3.SUMVAL LT 300) AND ((S3.QVAL1 EQ 101) OR (S3.QVAL2 EQ 102) OR (S3.QVAL3 EQ 103) OR (S3.QVAL4 EQ 104) OR (S3.QVAL5 EQ 105) OR (S3.QVAL6 EQ 106) OR (S3.QVAL7 EQ 107) OR (S3.QVAL8 EQ 108) OR (S3.QVAL9 EQ 109) OR (S3.QVAL_10 EQ 101.1) OR (S3.QVAL_11 EQ 101.2) OR (S3.QVAL_12 EQ 101.3) OR (S3.QVAL_13 EQ 101.4) OR (S3.QVAL_14 EQ 101.5) OR (S3.QVAL_15 EQ 101.6) OR (S3.QVAL_16 EQ 101.7) OR (S3.QVAL_17 EQ 101.8) OR (S3.QVAL_18 EQ 101.9) OR (S3.QVAL_19 EQ 101.19) OR (S3.QVAL_20 EQ 102.1) OR (S3.QVAL_21 EQ 102.2) OR (S3.QVAL_22 EQ 102.3) OR (S3.QVAL_23 EQ 102.4) OR (S3.QVAL_24 EQ 102.5) OR (S3.QVAL_25 EQ 102.6) OR (S3.QVAL_26 EQ 102.7)) AND (S3.QVAL_27 EQ 102.8)) OR (((S3.2PQVAL1 EQ 101) OR (S3.2PQVAL2 EQ 102) OR (S3.2PQVAL3 EQ 103) OR (S3.2PQVAL4 EQ 104) OR (S3.2PQVAL5 EQ 105) OR (S3.2PQVAL6 EQ 106) OR (S3.2PQVAL7 EQ 107) OR (S3.2PQVAL8 EQ 108) OR (S3.2PQVAL9 EQ 109) OR (S3.2PQVAL_10 EQ 110) OR (S3.2PQVAL_11 EQ 111) OR (S3.2PQVAL_12 EQ 112) OR (S3.2PQVAL_13 EQ 113) OR (S3.2PQVAL_14 EQ 114) OR (S3.2PQVAL_15 EQ 115) OR (S3.2PQVAL_16 EQ 116) OR (S3.2PQVAL_17 EQ 117) OR (S3.2PQVAL_18 EQ 118) OR (S3.2PQVAL_19 EQ 119) OR (S3.2PQVAL_20 EQ 120) OR (S3.2PQVAL_21 EQ 121) OR (S3.2PQVAL_22 EQ 122) OR (S3.2PQVAL_23 EQ 123) OR (S3.2PQVAL_24 EQ 124) OR (S3.2PQVAL_25 EQ 125) OR (S3.2PQVAL_26 EQ 126)) AND (S3.2PQVAL_27 EQ 127))) [S3.P9MICROQ2 = 'à prendre des décisions responsables et les maintenir'];
IF (((S3.SUMVAL GE 200) AND (S3.SUMVAL LT 300) AND ((S3.QVAL1 EQ 101) OR (S3.QVAL2 EQ 102) OR (S3.QVAL3 EQ 103) OR (S3.QVAL4 EQ 104) OR (S3.QVAL5 EQ 105) OR (S3.QVAL6 EQ 106) OR (S3.QVAL7 EQ 107) OR (S3.QVAL8 EQ 108) OR (S3.QVAL9 EQ 109) OR (S3.QVAL_10 EQ 101.1) OR (S3.QVAL_11 EQ 101.2) OR (S3.QVAL_12 EQ 101.3) OR (S3.QVAL_13 EQ 101.4) OR (S3.QVAL_14 EQ 101.5) OR (S3.QVAL_15 EQ 101.6) OR (S3.QVAL_16 EQ 101.7) OR (S3.QVAL_17 EQ 101.8) OR (S3.QVAL_18 EQ 101.9) OR (S3.QVAL_19 EQ 101.19) OR (S3.QVAL_20 EQ 102.1) OR (S3.QVAL_21 EQ 102.2) OR (S3.QVAL_22 EQ 102.3) OR (S3.QVAL_23 EQ 102.4) OR (S3.QVAL_24 EQ 102.5) OR (S3.QVAL_25 EQ 102.6) OR (S3.QVAL_26 EQ 102.7) OR (S3.QVAL_27 EQ 102.8)) AND (S3.QVAL_28 EQ 102.9)) OR (((S3.2PQVAL1 EQ 101) OR (S3.2PQVAL2 EQ 102) OR (S3.2PQVAL3 EQ 103) OR (S3.2PQVAL4 EQ 104) OR (S3.2PQVAL5 EQ 105) OR (S3.2PQVAL6 EQ 106) OR (S3.2PQVAL7 EQ 107) OR (S3.2PQVAL8 EQ 108) OR (S3.2PQVAL9 EQ 109) OR (S3.2PQVAL_10 EQ 110) OR (S3.2PQVAL_11 EQ 111) OR (S3.2PQVAL_12 EQ 112) OR (S3.2PQVAL_13 EQ 113) OR (S3.2PQVAL_14 EQ 114) OR (S3.2PQVAL_15 EQ 115) OR (S3.2PQVAL_16 EQ 116) OR (S3.2PQVAL_17 EQ 117) OR (S3.2PQVAL_18 EQ 118) OR (S3.2PQVAL_19 EQ 119) OR (S3.2PQVAL_20 EQ 120) OR (S3.2PQVAL_21 EQ 121) OR (S3.2PQVAL_22 EQ 122) OR (S3.2PQVAL_23 EQ 123) OR (S3.2PQVAL_24 EQ 124) OR (S3.2PQVAL_25 EQ 125) OR (S3.2PQVAL_26 EQ 126) OR (S3.2PQVAL_27 EQ 127)) AND (S3.2PQVAL_28 EQ 128))) [S3.P9MICROQ2 = 'vous sentir en sécurité et à l´abri de dangers'];
IF (((S3.SUMVAL GE 200) AND (S3.SUMVAL LT 300) AND ((S3.QVAL1 EQ 101) OR (S3.QVAL2 EQ 102) OR (S3.QVAL3 EQ 103) OR (S3.QVAL4 EQ 104) OR (S3.QVAL5 EQ 105) OR (S3.QVAL6 EQ 106) OR (S3.QVAL7 EQ 107) OR (S3.QVAL8 EQ 108) OR (S3.QVAL9 EQ 109) OR (S3.QVAL_10 EQ 101.1) OR (S3.QVAL_11 EQ 101.2) OR (S3.QVAL_12 EQ 101.3) OR (S3.QVAL_13 EQ 101.4) OR (S3.QVAL_14 EQ 101.5) OR (S3.QVAL_15 EQ 101.6) OR (S3.QVAL_16 EQ 101.7) OR (S3.QVAL_17 EQ 101.8) OR (S3.QVAL_18 EQ 101.9) OR (S3.QVAL_19 EQ 101.19) OR (S3.QVAL_20 EQ 102.1) OR (S3.QVAL_21 EQ 102.2) OR (S3.QVAL_22 EQ 102.3) OR

(S3.QVAL_23 EQ 102.4) OR (S3.QVAL_24 EQ 102.5) OR (S3.QVAL_25 EQ 102.6) OR (S3.QVAL_26 EQ 102.7) OR (S3.QVAL_27 EQ 102.8) OR (S3.QVAL_28 EQ 102.9)) AND (S3.QVAL_29 EQ 102.29)) OR (((S3.2PQVAL1 EQ 101) OR (S3.2PQVAL2 EQ 102) OR (S3.2PQVAL3 EQ 103) OR (S3.2PQVAL4 EQ 104) OR (S3.2PQVAL5 EQ 105) OR (S3.2PQVAL6 EQ 106) OR (S3.2PQVAL7 EQ 107) OR (S3.2PQVAL8 EQ 108) OR (S3.2PQVAL9 EQ 109) OR (S3.2PQVAL_10 EQ 110) OR (S3.2PQVAL_11 EQ 111) OR (S3.2PQVAL_12 EQ 112) OR (S3.2PQVAL_13 EQ 113) OR (S3.2PQVAL_14 EQ 114) OR (S3.2PQVAL_15 EQ 115) OR (S3.2PQVAL_16 EQ 116) OR (S3.2PQVAL_17 EQ 117) OR (S3.2PQVAL_18 EQ 118) OR (S3.2PQVAL_19 EQ 119) OR (S3.2PQVAL_20 EQ 120) OR (S3.2PQVAL_21 EQ 121) OR (S3.2PQVAL_22 EQ 122) OR (S3.2PQVAL_23 EQ 123) OR (S3.2PQVAL_24 EQ 124) OR (S3.2PQVAL_25 EQ 125) OR (S3.2PQVAL_26 EQ 126) OR (S3.2PQVAL_27 EQ 127) OR (S3.2PQVAL_28 EQ 128)) AND (S3.2PQVAL_29 EQ 129))) [S3.P9MICROQ2 = 'être une personne aidante et aux services des autres'];
IF (((S3.SUMVAL GE 200) AND (S3.SUMVAL LT 300) AND (S3.QVAL1 EQ 101)) OR (S3.2PQVAL1 EQ 101)) [S3.P9TEMQ1 = 'C´est sûr que je serais plus en forme pour être là pour mes amis. Je serais là plus longtemps et en santé pour eux aussi.'];
IF (((S3.SUMVAL GE 200) AND (S3.SUMVAL LT 300) AND (S3.QVAL1 EQ 0) AND (S3.QVAL2 EQ 102)) OR ((S3.2PQVAL1 EQ 0) AND (S3.2PQVAL2 EQ 102))) [S3.P9TEMQ1 = 'Je serais vraiment content de moi. Je serais bien dans ma peau et les autres aimeraient probablement aussi.'];
IF (((S3.SUMVAL GE 200) AND (S3.SUMVAL LT 300) AND (S3.QVAL1 EQ 0) AND (S3.QVAL2 EQ 0) AND (S3.QVAL3 EQ 103)) OR ((S3.2PQVAL1 EQ 0) AND (S3.2PQVAL2 EQ 0) AND (S3.2PQVAL3 EQ 103))) [S3.P9TEMQ1 = 'Je ferais partie des personnes qui ont la force de prendre leur santé en main. Je montrerais l'exemple à ma famille.'];
IF (((S3.SUMVAL GE 200) AND (S3.SUMVAL LT 300) AND (S3.QVAL1 EQ 0) AND (S3.QVAL2 EQ 0) AND (S3.QVAL3 EQ 0) AND (S3.QVAL4 EQ 104)) OR ((S3.2PQVAL1 EQ 0) AND (S3.2PQVAL2 EQ 0) AND (S3.2PQVAL3 EQ 0) AND (S3.2PQVAL4 EQ 104))) [S3.P9TEMQ1 = 'Je serais plus vif. J´aurais encore plus d´assurance pour m´occuper et être responsable des autres.'];
IF (((S3.SUMVAL GE 200) AND (S3.SUMVAL LT 300) AND (S3.QVAL1 EQ 0) AND (S3.QVAL2 EQ 0) AND (S3.QVAL3 EQ 0) AND (S3.QVAL4 EQ 0) AND (S3.QVAL5 EQ 105)) OR ((S3.2PQVAL1 EQ 0) AND (S3.2PQVAL2 EQ 0) AND (S3.2PQVAL3 EQ 0) AND (S3.2PQVAL4 EQ 0) AND (S3.2PQVAL5 EQ 105))) [S3.P9TEMQ1 = 'C´est sûr que je resterais en santé plus longtemps. Je serais moins à risque de dépendre des autres à cause qu´il m´arrive quelque chose.'];
IF (((S3.SUMVAL GE 200) AND (S3.SUMVAL LT 300) AND (S3.QVAL1 EQ 0) AND (S3.QVAL2 EQ 0) AND (S3.QVAL3 EQ 0) AND (S3.QVAL4 EQ 0) AND (S3.QVAL5 EQ 0) AND (S3.QVAL6 EQ 106)) OR ((S3.2PQVAL1 EQ 0) AND (S3.2PQVAL2 EQ 0) AND (S3.2PQVAL3 EQ 0) AND (S3.2PQVAL4 EQ 0) AND (S3.2PQVAL5 EQ 0) AND (S3.2PQVAL6 EQ 106))) [S3.P9TEMQ1 = 'Je me sentirais mieux dans ma peau donc j´apprécierais encore plus tout ce qui m´entourent.'];
IF (((S3.SUMVAL GE 200) AND (S3.SUMVAL LT 300) AND (S3.QVAL1 EQ 0) AND (S3.QVAL2 EQ 0) AND (S3.QVAL3 EQ 0) AND (S3.QVAL4 EQ 0) AND (S3.QVAL5 EQ 0) AND (S3.QVAL6 EQ 0) AND (S3.QVAL7 EQ 107)) OR ((S3.2PQVAL1 EQ 0) AND (S3.2PQVAL2 EQ 0) AND (S3.2PQVAL3 EQ 0) AND (S3.2PQVAL4 EQ 0) AND (S3.2PQVAL5 EQ 0) AND (S3.2PQVAL6 EQ 0) AND (S3.2PQVAL7 EQ 107))) [S3.P9TEMQ1 = 'Je me préoccuperais moins de ma santé et j´aurais l´esprit libre pour m´occuper des autres.'];
IF (((S3.SUMVAL GE 200) AND (S3.SUMVAL LT 300) AND (S3.QVAL1 EQ 0) AND (S3.QVAL2 EQ 0) AND (S3.QVAL3 EQ 0) AND (S3.QVAL4 EQ 0) AND (S3.QVAL5 EQ 0) AND (S3.QVAL6 EQ 0) AND (S3.QVAL7 EQ 0)

AND (S3.QVAL8 EQ 108)) OR ((S3.2PQVAL1 EQ 0) AND (S3.2PQVAL2 EQ 0) AND (S3.2PQVAL3 EQ 0) AND (S3.2PQVAL4 EQ 0) AND (S3.2PQVAL5 EQ 0) AND (S3.2PQVAL6 EQ 0) AND (S3.2PQVAL7 EQ 0) AND (S3.2PQVAL8 EQ 108))) [S3.P9TEMQ1 = 'C´est pas si excitant pour moi de bouger. D´un autre côté, ça m´aiderait c´est sûr. Ça pourrait démontrer ma capacité à faire preuve de compromis dans la vie.'];
IF (((S3.SUMVAL GE 200) AND (S3.SUMVAL LT 300) AND (S3.QVAL1 EQ 0) AND (S3.QVAL2 EQ 0) AND (S3.QVAL3 EQ 0) AND (S3.QVAL4 EQ 0) AND (S3.QVAL5 EQ 0) AND (S3.QVAL6 EQ 0) AND (S3.QVAL7 EQ 0) AND (S3.QVAL8 EQ 0) AND (S3.QVAL9 EQ 109)) OR ((S3.2PQVAL1 EQ 0) AND (S3.2PQVAL2 EQ 0) AND (S3.2PQVAL3 EQ 0) AND (S3.2PQVAL4 EQ 0) AND (S3.2PQVAL5 EQ 0) AND (S3.2PQVAL6 EQ 0) AND (S3.2PQVAL7 EQ 0) AND (S3.2PQVAL8 EQ 0) AND (S3.2PQVAL9 EQ 109))) [S3.P9TEMQ1 = 'J´aurais la conscience beaucoup plus tranquille et je me sentirais certainement mieux dans la vie de tous les jours.'];
IF (((S3.SUMVAL GE 200) AND (S3.SUMVAL LT 300) AND (S3.QVAL1 EQ 0) AND (S3.QVAL2 EQ 0) AND (S3.QVAL3 EQ 0) AND (S3.QVAL4 EQ 0) AND (S3.QVAL5 EQ 0) AND (S3.QVAL6 EQ 0) AND (S3.QVAL7 EQ 0) AND (S3.QVAL8 EQ 0) AND (S3.QVAL9 EQ 0) AND (S3.QVAL_10 EQ 101.1)) OR ((S3.2PQVAL1 EQ 0) AND (S3.2PQVAL2 EQ 0) AND (S3.2PQVAL3 EQ 0) AND (S3.2PQVAL4 EQ 0) AND (S3.2PQVAL5 EQ 0) AND (S3.2PQVAL6 EQ 0) AND (S3.2PQVAL7 EQ 0) AND (S3.2PQVAL8 EQ 0) AND (S3.2PQVAL9 EQ 0) AND (S3.2PQVAL_10 EQ 110))) [S3.P9TEMQ1 = 'J'ai un diabète oui, mais je peux me prendre en mains malgré tout. Je ferais preuve de courage en pratiquant régulièrement des activités physiques c'est sûr.'];
IF (((S3.SUMVAL GE 200) AND (S3.SUMVAL LT 300) AND (S3.QVAL1 EQ 0) AND (S3.QVAL2 EQ 0) AND (S3.QVAL3 EQ 0) AND (S3.QVAL4 EQ 0) AND (S3.QVAL5 EQ 0) AND (S3.QVAL6 EQ 0) AND (S3.QVAL7 EQ 0) AND (S3.QVAL8 EQ 0) AND (S3.QVAL9 EQ 0) AND (S3.QVAL_10 EQ 0) AND (S3.QVAL_11 EQ 101.2)) OR ((S3.2PQVAL1 EQ 0) AND (S3.2PQVAL2 EQ 0) AND (S3.2PQVAL3 EQ 0) AND (S3.2PQVAL4 EQ 0) AND (S3.2PQVAL5 EQ 0) AND (S3.2PQVAL6 EQ 0) AND (S3.2PQVAL7 EQ 0) AND (S3.2PQVAL8 EQ 0) AND (S3.2PQVAL9 EQ 0) AND (S3.2PQVAL_10 EQ 0) AND (S3.2PQVAL_11 EQ 111))) [S3.P9TEMQ1 = 'Je sais que l'activité physique aide à garder un esprit vigoureux. Ça pourrait sûrement m'aider à rester inspiré constamment.'];
IF (((S3.SUMVAL GE 200) AND (S3.SUMVAL LT 300) AND (S3.QVAL1 EQ 0) AND (S3.QVAL2 EQ 0) AND (S3.QVAL3 EQ 0) AND (S3.QVAL4 EQ 0) AND (S3.QVAL5 EQ 0) AND (S3.QVAL6 EQ 0) AND (S3.QVAL7 EQ 0) AND (S3.QVAL8 EQ 0) AND (S3.QVAL9 EQ 0) AND (S3.QVAL_10 EQ 0) AND (S3.QVAL_11 EQ 0) AND (S3.QVAL_12 EQ 101.3)) OR ((S3.2PQVAL1 EQ 0) AND (S3.2PQVAL2 EQ 0) AND (S3.2PQVAL3 EQ 0) AND (S3.2PQVAL4 EQ 0) AND (S3.2PQVAL5 EQ 0) AND (S3.2PQVAL6 EQ 0) AND (S3.2PQVAL7 EQ 0) AND (S3.2PQVAL8 EQ 0) AND (S3.2PQVAL9 EQ 0) AND (S3.2PQVAL_10 EQ 0) AND (S3.2PQVAL_11 EQ 0) AND (S3.2PQVAL_12 EQ 112))) [S3.P9TEMQ1 = 'Je m'assurerais de garder une bonne santé et de me tenir en forme. Juste ça serait une évolution pour moi.'];
IF (((S3.SUMVAL GE 200) AND (S3.SUMVAL LT 300) AND (S3.QVAL1 EQ 0) AND (S3.QVAL2 EQ 0) AND (S3.QVAL3 EQ 0) AND (S3.QVAL4 EQ 0) AND (S3.QVAL5 EQ 0) AND (S3.QVAL6 EQ 0) AND (S3.QVAL7 EQ 0) AND (S3.QVAL8 EQ 0) AND (S3.QVAL9 EQ 0) AND (S3.QVAL_10 EQ 0) AND (S3.QVAL_11 EQ 0) AND (S3.QVAL_12 EQ 0) AND (S3.QVAL_13 EQ 101.4)) OR ((S3.2PQVAL1 EQ 0) AND (S3.2PQVAL2 EQ 0) AND (S3.2PQVAL3 EQ 0) AND (S3.2PQVAL4 EQ 0) AND (S3.2PQVAL5 EQ 0) AND (S3.2PQVAL6 EQ 0) AND (S3.2PQVAL7 EQ 0) AND (S3.2PQVAL8 EQ 0) AND (S3.2PQVAL9 EQ 0) AND (S3.2PQVAL_10 EQ 0) AND (S3.2PQVAL_11 EQ 0) AND (S3.2PQVAL_12 EQ 0) AND (S3.2PQVAL_13 EQ 113))) [S3.P9TEMQ1 = 'J'aimerais pratiquer des activités physiques régulièrement pour voir ce qui m'arriverait, comment je me sentirais. Ça serait intéressant.'];

IF (((S3.SUMVAL GE 200) AND (S3.SUMVAL LT 300) AND (S3.QVAL1 EQ 0) AND (S3.QVAL2 EQ 0) AND (S3.QVAL3 EQ 0) AND (S3.QVAL4 EQ 0) AND (S3.QVAL5 EQ 0) AND (S3.QVAL6 EQ 0) AND (S3.QVAL7 EQ 0) AND (S3.QVAL8 EQ 0) AND (S3.QVAL9 EQ 0) AND (S3.QVAL_10 EQ 0) AND (S3.QVAL_11 EQ 0) AND (S3.QVAL_12 EQ 0) AND (S3.QVAL_13 EQ 0) AND (S3.QVAL_14 EQ 101.5)) OR ((S3.2PQVAL1 EQ 0) AND (S3.2PQVAL2 EQ 0) AND (S3.2PQVAL3 EQ 0) AND (S3.2PQVAL4 EQ 0) AND (S3.2PQVAL5 EQ 0) AND (S3.2PQVAL6 EQ 0) AND (S3.2PQVAL7 EQ 0) AND (S3.2PQVAL8 EQ 0) AND (S3.2PQVAL9 EQ 0) AND (S3.2PQVAL_10 EQ 0) AND (S3.2PQVAL_11 EQ 0) AND (S3.2PQVAL_12 EQ 0) AND (S3.2PQVAL_13 EQ 0) AND (S3.2PQVAL_14 EQ 114))) [S3.P9TEMQ1 = 'Faire des activités physiques aide à avoir un bon mental. Ça m'aiderait à voir les choses positivement même dans des moments difficiles.'];
IF (((S3.SUMVAL GE 200) AND (S3.SUMVAL LT 300) AND (S3.QVAL1 EQ 0) AND (S3.QVAL2 EQ 0) AND (S3.QVAL3 EQ 0) AND (S3.QVAL4 EQ 0) AND (S3.QVAL5 EQ 0) AND (S3.QVAL6 EQ 0) AND (S3.QVAL7 EQ 0) AND (S3.QVAL8 EQ 0) AND (S3.QVAL9 EQ 0) AND (S3.QVAL_10 EQ 0) AND (S3.QVAL_11 EQ 0) AND (S3.QVAL_12 EQ 0) AND (S3.QVAL_13 EQ 0) AND (S3.QVAL_14 EQ 0) AND (S3.QVAL_15 EQ 101.6)) OR ((S3.2PQVAL1 EQ 0) AND (S3.2PQVAL2 EQ 0) AND (S3.2PQVAL3 EQ 0) AND (S3.2PQVAL4 EQ 0) AND (S3.2PQVAL5 EQ 0) AND (S3.2PQVAL6 EQ 0) AND (S3.2PQVAL7 EQ 0) AND (S3.2PQVAL8 EQ 0) AND (S3.2PQVAL9 EQ 0) AND (S3.2PQVAL_10 EQ 0) AND (S3.2PQVAL_11 EQ 0) AND (S3.2PQVAL_12 EQ 0) AND (S3.2PQVAL_13 EQ 0) AND (S3.2PQVAL_14 EQ 0) AND (S3.2PQVAL_15 EQ 115))) [S3.P9TEMQ1 = 'Je ressentirais de la fierté si je faisais des activités physiques régulièrement. C´est certain.'];
IF (((S3.SUMVAL GE 200) AND (S3.SUMVAL LT 300) AND (S3.QVAL1 EQ 0) AND (S3.QVAL2 EQ 0) AND (S3.QVAL3 EQ 0) AND (S3.QVAL4 EQ 0) AND (S3.QVAL5 EQ 0) AND (S3.QVAL6 EQ 0) AND (S3.QVAL7 EQ 0) AND (S3.QVAL8 EQ 0) AND (S3.QVAL9 EQ 0) AND (S3.QVAL_10 EQ 0) AND (S3.QVAL_11 EQ 0) AND (S3.QVAL_12 EQ 0) AND (S3.QVAL_13 EQ 0) AND (S3.QVAL_14 EQ 0) AND (S3.QVAL_15 EQ 0) AND (S3.QVAL_16 EQ 101.7)) OR ((S3.2PQVAL1 EQ 0) AND (S3.2PQVAL2 EQ 0) AND (S3.2PQVAL3 EQ 0) AND (S3.2PQVAL4 EQ 0) AND (S3.2PQVAL5 EQ 0) AND (S3.2PQVAL6 EQ 0) AND (S3.2PQVAL7 EQ 0) AND (S3.2PQVAL8 EQ 0) AND (S3.2PQVAL9 EQ 0) AND (S3.2PQVAL_10 EQ 0) AND (S3.2PQVAL_11 EQ 0) AND (S3.2PQVAL_12 EQ 0) AND (S3.2PQVAL_13 EQ 0) AND (S3.2PQVAL_14 EQ 0) AND (S3.2PQVAL_15 EQ 0) AND (S3.2PQVAL_16 EQ 116))) [S3.P9TEMQ1 = 'Je serais fier de montrer l'exemple à ma famille. Ça pourrait aussi nous permettre de faire des activités différentes ensemble.'];
IF (((S3.SUMVAL GE 200) AND (S3.SUMVAL LT 300) AND (S3.QVAL1 EQ 0) AND (S3.QVAL2 EQ 0) AND (S3.QVAL3 EQ 0) AND (S3.QVAL4 EQ 0) AND (S3.QVAL5 EQ 0) AND (S3.QVAL6 EQ 0) AND (S3.QVAL7 EQ 0) AND (S3.QVAL8 EQ 0) AND (S3.QVAL9 EQ 0) AND (S3.QVAL_10 EQ 0) AND (S3.QVAL_11 EQ 0) AND (S3.QVAL_12 EQ 0) AND (S3.QVAL_13 EQ 0) AND (S3.QVAL_14 EQ 0) AND (S3.QVAL_15 EQ 0) AND (S3.QVAL_16 EQ 0) AND (S3.QVAL_17 EQ 101.8)) OR ((S3.2PQVAL1 EQ 0) AND (S3.2PQVAL2 EQ 0) AND (S3.2PQVAL3 EQ 0) AND (S3.2PQVAL4 EQ 0) AND (S3.2PQVAL5 EQ 0) AND (S3.2PQVAL6 EQ 0) AND (S3.2PQVAL7 EQ 0) AND (S3.2PQVAL8 EQ 0) AND (S3.2PQVAL9 EQ 0) AND (S3.2PQVAL_10 EQ 0) AND (S3.2PQVAL_11 EQ 0) AND (S3.2PQVAL_12 EQ 0) AND (S3.2PQVAL_13 EQ 0) AND (S3.2PQVAL_14 EQ 0) AND (S3.2PQVAL_15 EQ 0) AND (S3.2PQVAL_16 EQ 0) AND (S3.2PQVAL_17 EQ 117))) [S3.P9TEMQ1 = 'J'aurais une humeur plus stable. Je serais plus discipliné pour les tâches que je dois accomplir.'];
IF (((S3.SUMVAL GE 200) AND (S3.SUMVAL LT 300) AND (S3.QVAL1 EQ 0) AND (S3.QVAL2 EQ 0) AND (S3.QVAL3 EQ 0) AND (S3.QVAL4 EQ 0) AND (S3.QVAL5 EQ 0) AND (S3.QVAL6 EQ 0) AND (S3.QVAL7 EQ 0) AND (S3.QVAL8 EQ 0) AND (S3.QVAL9 EQ 0) AND (S3.QVAL_10 EQ 0) AND (S3.QVAL_11 EQ 0) AND (S3.QVAL_12 EQ 0) AND (S3.QVAL_13 EQ 0) AND (S3.QVAL_14 EQ 0) AND (S3.QVAL_15 EQ 0) AND

(S3.QVAL_16 EQ 0) AND (S3.QVAL_17 EQ 0) AND (S3.QVAL_18 EQ 101.9)) OR ((S3.2PQVAL1 EQ 0) AND (S3.2PQVAL2 EQ 0) AND (S3.2PQVAL3 EQ 0) AND (S3.2PQVAL4 EQ 0) AND (S3.2PQVAL5 EQ 0) AND (S3.2PQVAL6 EQ 0) AND (S3.2PQVAL7 EQ 0) AND (S3.2PQVAL8 EQ 0) AND (S3.2PQVAL9 EQ 0) AND (S3.2PQVAL_10 EQ 0) AND (S3.2PQVAL_11 EQ 0) AND (S3.2PQVAL_12 EQ 0) AND (S3.2PQVAL_13 EQ 0) AND (S3.2PQVAL_14 EQ 0) AND (S3.2PQVAL_15 EQ 0) AND (S3.2PQVAL_16 EQ 0) AND (S3.2PQVAL_17 EQ 0) AND (S3.2PQVAL_18 EQ 118))) [S3.P9TEMQ1 = 'Ça améliorerait mon humeur. Ça m'aiderait à réagir moins fortement à certaines choses qui me dérangent plus.'];
IF (((S3.SUMVAL GE 200) AND (S3.SUMVAL LT 300) AND (S3.QVAL1 EQ 0) AND (S3.QVAL2 EQ 0) AND (S3.QVAL3 EQ 0) AND (S3.QVAL4 EQ 0) AND (S3.QVAL5 EQ 0) AND (S3.QVAL6 EQ 0) AND (S3.QVAL7 EQ 0) AND (S3.QVAL8 EQ 0) AND (S3.QVAL9 EQ 0) AND (S3.QVAL_10 EQ 0) AND (S3.QVAL_11 EQ 0) AND (S3.QVAL_12 EQ 0) AND (S3.QVAL_13 EQ 0) AND (S3.QVAL_14 EQ 0) AND (S3.QVAL_15 EQ 0) AND (S3.QVAL_16 EQ 0) AND (S3.QVAL_17 EQ 0) AND (S3.QVAL_18 EQ 0) AND (S3.QVAL_19 EQ 101.19)) OR ((S3.2PQVAL1 EQ 0) AND (S3.2PQVAL2 EQ 0) AND (S3.2PQVAL3 EQ 0) AND (S3.2PQVAL4 EQ 0) AND (S3.2PQVAL5 EQ 0) AND (S3.2PQVAL6 EQ 0) AND (S3.2PQVAL7 EQ 0) AND (S3.2PQVAL8 EQ 0) AND (S3.2PQVAL9 EQ 0) AND (S3.2PQVAL_10 EQ 0) AND (S3.2PQVAL_11 EQ 0) AND (S3.2PQVAL_12 EQ 0) AND (S3.2PQVAL_13 EQ 0) AND (S3.2PQVAL_14 EQ 0) AND (S3.2PQVAL_15 EQ 0) AND (S3.2PQVAL_16 EQ 0) AND (S3.2PQVAL_17 EQ 0) AND (S3.2PQVAL_18 EQ 0) AND (S3.2PQVAL_19 EQ 119))) [S3.P9TEMQ1 = 'Je serais mieux dans ma peau. Être bien m'aiderait à être une personne encore plus généreuse.'];
IF (((S3.SUMVAL GE 200) AND (S3.SUMVAL LT 300) AND (S3.QVAL1 EQ 0) AND (S3.QVAL2 EQ 0) AND (S3.QVAL3 EQ 0) AND (S3.QVAL4 EQ 0) AND (S3.QVAL5 EQ 0) AND (S3.QVAL6 EQ 0) AND (S3.QVAL7 EQ 0) AND (S3.QVAL8 EQ 0) AND (S3.QVAL9 EQ 0) AND (S3.QVAL_10 EQ 0) AND (S3.QVAL_11 EQ 0) AND (S3.QVAL_12 EQ 0) AND (S3.QVAL_13 EQ 0) AND (S3.QVAL_14 EQ 0) AND (S3.QVAL_15 EQ 0) AND (S3.QVAL_16 EQ 0) AND (S3.QVAL_17 EQ 0) AND (S3.QVAL_18 EQ 0) AND (S3.QVAL_19 EQ 0) AND (S3.QVAL_20 EQ 102.1)) OR ((S3.2PQVAL1 EQ 0) AND (S3.2PQVAL2 EQ 0) AND (S3.2PQVAL3 EQ 0) AND (S3.2PQVAL4 EQ 0) AND (S3.2PQVAL5 EQ 0) AND (S3.2PQVAL6 EQ 0) AND (S3.2PQVAL7 EQ 0) AND (S3.2PQVAL8 EQ 0) AND (S3.2PQVAL9 EQ 0) AND (S3.2PQVAL_10 EQ 0) AND (S3.2PQVAL_11 EQ 0) AND (S3.2PQVAL_12 EQ 0) AND (S3.2PQVAL_13 EQ 0) AND (S3.2PQVAL_14 EQ 0) AND (S3.2PQVAL_15 EQ 0) AND (S3.2PQVAL_16 EQ 0) AND (S3.2PQVAL_17 EQ 0) AND (S3.2PQVAL_18 EQ 0) AND (S3.2PQVAL_19 EQ 0) AND (S3.2PQVAL_20 EQ 120))) [S3.P9TEMQ1 = 'Ça m´aiderait à avoir une meilleure concentration et une meilleure attention. J'aurais l'esprit plus libre.'];
IF (((S3.SUMVAL GE 200) AND (S3.SUMVAL LT 300) AND (S3.QVAL1 EQ 0) AND (S3.QVAL2 EQ 0) AND (S3.QVAL3 EQ 0) AND (S3.QVAL4 EQ 0) AND (S3.QVAL5 EQ 0) AND (S3.QVAL6 EQ 0) AND (S3.QVAL7 EQ 0) AND (S3.QVAL8 EQ 0) AND (S3.QVAL9 EQ 0) AND (S3.QVAL_10 EQ 0) AND (S3.QVAL_11 EQ 0) AND (S3.QVAL_12 EQ 0) AND (S3.QVAL_13 EQ 0) AND (S3.QVAL_14 EQ 0) AND (S3.QVAL_15 EQ 0) AND (S3.QVAL_16 EQ 0) AND (S3.QVAL_17 EQ 0) AND (S3.QVAL_18 EQ 0) AND (S3.QVAL_19 EQ 0) AND (S3.QVAL_20 EQ 0) AND (S3.QVAL_21 EQ 102.2)) OR ((S3.2PQVAL1 EQ 0) AND (S3.2PQVAL2 EQ 0) AND (S3.2PQVAL3 EQ 0) AND (S3.2PQVAL4 EQ 0) AND (S3.2PQVAL5 EQ 0) AND (S3.2PQVAL6 EQ 0) AND (S3.2PQVAL7 EQ 0) AND (S3.2PQVAL8 EQ 0) AND (S3.2PQVAL9 EQ 0) AND (S3.2PQVAL_10 EQ 0) AND (S3.2PQVAL_11 EQ 0) AND (S3.2PQVAL_12 EQ 0) AND (S3.2PQVAL_13 EQ 0) AND (S3.2PQVAL_14 EQ 0) AND (S3.2PQVAL_15 EQ 0) AND (S3.2PQVAL_16 EQ 0) AND (S3.2PQVAL_17 EQ 0) AND (S3.2PQVAL_18 EQ 0) AND (S3.2PQVAL_19 EQ 0) AND (S3.2PQVAL_20 EQ 0) AND (S3.2PQVAL_21 EQ 121))) [S3.P9TEMQ1 = 'Je

serais un exemple pour les personnes qui ont de la misère à pratiquer régulièrement des activités physiques. Je veux être un modèle de réussite.'];
IF (((S3.SUMVAL GE 200) AND (S3.SUMVAL LT 300) AND (S3.QVAL1 EQ 0) AND (S3.QVAL2 EQ 0) AND (S3.QVAL3 EQ 0) AND (S3.QVAL4 EQ 0) AND (S3.QVAL5 EQ 0) AND (S3.QVAL6 EQ 0) AND (S3.QVAL7 EQ 0) AND (S3.QVAL8 EQ 0) AND (S3.QVAL9 EQ 0) AND (S3.QVAL_10 EQ 0) AND (S3.QVAL_11 EQ 0) AND (S3.QVAL_12 EQ 0) AND (S3.QVAL_13 EQ 0) AND (S3.QVAL_14 EQ 0) AND (S3.QVAL_15 EQ 0) AND (S3.QVAL_16 EQ 0) AND (S3.QVAL_17 EQ 0) AND (S3.QVAL_18 EQ 0) AND (S3.QVAL_19 EQ 0) AND (S3.QVAL_20 EQ 0) AND (S3.QVAL_21 EQ 0) AND (S3.QVAL_22 EQ 102.3)) OR ((S3.2PQVAL1 EQ 0) AND (S3.2PQVAL2 EQ 0) AND (S3.2PQVAL3 EQ 0) AND (S3.2PQVAL4 EQ 0) AND (S3.2PQVAL5 EQ 0) AND (S3.2PQVAL6 EQ 0) AND (S3.2PQVAL7 EQ 0) AND (S3.2PQVAL8 EQ 0) AND (S3.2PQVAL9 EQ 0) AND (S3.2PQVAL_10 EQ 0) AND (S3.2PQVAL_11 EQ 0) AND (S3.2PQVAL_12 EQ 0) AND (S3.2PQVAL_13 EQ 0) AND (S3.2PQVAL_14 EQ 0) AND (S3.2PQVAL_15 EQ 0) AND (S3.2PQVAL_16 EQ 0) AND (S3.2PQVAL_17 EQ 0) AND (S3.2PQVAL_18 EQ 0) AND (S3.2PQVAL_19 EQ 0) AND (S3.2PQVAL_20 EQ 0) AND (S3.2PQVAL_21 EQ 0) AND (S3.2PQVAL_22 EQ 122))) [S3.P9TEMQ1 = 'J'éviterais bien des problèmes de santé. Je ne serais pas encombré par des limitations que je pourrais éviter.'];
IF (((S3.SUMVAL GE 200) AND (S3.SUMVAL LT 300) AND (S3.QVAL1 EQ 0) AND (S3.QVAL2 EQ 0) AND (S3.QVAL3 EQ 0) AND (S3.QVAL4 EQ 0) AND (S3.QVAL5 EQ 0) AND (S3.QVAL6 EQ 0) AND (S3.QVAL7 EQ 0) AND (S3.QVAL8 EQ 0) AND (S3.QVAL9 EQ 0) AND (S3.QVAL_10 EQ 0) AND (S3.QVAL_11 EQ 0) AND (S3.QVAL_12 EQ 0) AND (S3.QVAL_13 EQ 0) AND (S3.QVAL_14 EQ 0) AND (S3.QVAL_15 EQ 0) AND (S3.QVAL_16 EQ 0) AND (S3.QVAL_17 EQ 0) AND (S3.QVAL_18 EQ 0) AND (S3.QVAL_19 EQ 0) AND (S3.QVAL_20 EQ 0) AND (S3.QVAL_21 EQ 0) AND (S3.QVAL_22 EQ 0) AND (S3.QVAL_23 EQ 102.4)) OR ((S3.2PQVAL1 EQ 0) AND (S3.2PQVAL2 EQ 0) AND (S3.2PQVAL3 EQ 0) AND (S3.2PQVAL4 EQ 0) AND (S3.2PQVAL5 EQ 0) AND (S3.2PQVAL6 EQ 0) AND (S3.2PQVAL7 EQ 0) AND (S3.2PQVAL8 EQ 0) AND (S3.2PQVAL9 EQ 0) AND (S3.2PQVAL_10 EQ 0) AND (S3.2PQVAL_11 EQ 0) AND (S3.2PQVAL_12 EQ 0) AND (S3.2PQVAL_13 EQ 0) AND (S3.2PQVAL_14 EQ 0) AND (S3.2PQVAL_15 EQ 0) AND (S3.2PQVAL_16 EQ 0) AND (S3.2PQVAL_17 EQ 0) AND (S3.2PQVAL_18 EQ 0) AND (S3.2PQVAL_19 EQ 0) AND (S3.2PQVAL_20 EQ 0) AND (S3.2PQVAL_21 EQ 0) AND (S3.2PQVAL_22 EQ 0) AND (S3.2PQVAL_23 EQ 123))) [S3.P9TEMQ1 = 'Je veux offrir mon meilleur aux autres. Ça m'aiderait à rester en santé, à être encore plus optimiste dans la vie, et avec ceux que j'aime.'];
IF (((S3.SUMVAL GE 200) AND (S3.SUMVAL LT 300) AND (S3.QVAL1 EQ 0) AND (S3.QVAL2 EQ 0) AND (S3.QVAL3 EQ 0) AND (S3.QVAL4 EQ 0) AND (S3.QVAL5 EQ 0) AND (S3.QVAL6 EQ 0) AND (S3.QVAL7 EQ 0) AND (S3.QVAL8 EQ 0) AND (S3.QVAL9 EQ 0) AND (S3.QVAL_10 EQ 0) AND (S3.QVAL_11 EQ 0) AND (S3.QVAL_12 EQ 0) AND (S3.QVAL_13 EQ 0) AND (S3.QVAL_14 EQ 0) AND (S3.QVAL_15 EQ 0) AND (S3.QVAL_16 EQ 0) AND (S3.QVAL_17 EQ 0) AND (S3.QVAL_18 EQ 0) AND (S3.QVAL_19 EQ 0) AND (S3.QVAL_20 EQ 0) AND (S3.QVAL_21 EQ 0) AND (S3.QVAL_22 EQ 0) AND (S3.QVAL_23 EQ 0) AND (S3.QVAL_24 EQ 102.5)) OR ((S3.2PQVAL1 EQ 0) AND (S3.2PQVAL2 EQ 0) AND (S3.2PQVAL3 EQ 0) AND (S3.2PQVAL4 EQ 0) AND (S3.2PQVAL5 EQ 0) AND (S3.2PQVAL6 EQ 0) AND (S3.2PQVAL7 EQ 0) AND (S3.2PQVAL8 EQ 0) AND (S3.2PQVAL9 EQ 0) AND (S3.2PQVAL_10 EQ 0) AND (S3.2PQVAL_11 EQ 0) AND (S3.2PQVAL_12 EQ 0) AND (S3.2PQVAL_13 EQ 0) AND (S3.2PQVAL_14 EQ 0) AND (S3.2PQVAL_15 EQ 0) AND (S3.2PQVAL_16 EQ 0) AND (S3.2PQVAL_17 EQ 0) AND (S3.2PQVAL_18 EQ 0) AND (S3.2PQVAL_19 EQ 0) AND (S3.2PQVAL_20 EQ 0) AND (S3.2PQVAL_21 EQ 0) AND (S3.2PQVAL_22 EQ 0) AND (S3.2PQVAL_23

EQ 0) AND (S3.2PQVAL_24 EQ 124))) [S3.P9TEMQ1 = 'Je sais que c'est la bonne chose à faire. Faire des activités physiques m'aiderait à être plus en cohérence avec la personne que je veux vraiment être.'];
IF (((S3.SUMVAL GE 200) AND (S3.SUMVAL LT 300) AND (S3.QVAL1 EQ 0) AND (S3.QVAL2 EQ 0) AND (S3.QVAL3 EQ 0) AND (S3.QVAL4 EQ 0) AND (S3.QVAL5 EQ 0) AND (S3.QVAL6 EQ 0) AND (S3.QVAL7 EQ 0) AND (S3.QVAL8 EQ 0) AND (S3.QVAL9 EQ 0) AND (S3.QVAL_10 EQ 0) AND (S3.QVAL_11 EQ 0) AND (S3.QVAL_12 EQ 0) AND (S3.QVAL_13 EQ 0) AND (S3.QVAL_14 EQ 0) AND (S3.QVAL_15 EQ 0) AND (S3.QVAL_16 EQ 0) AND (S3.QVAL_17 EQ 0) AND (S3.QVAL_18 EQ 0) AND (S3.QVAL_19 EQ 0) AND (S3.QVAL_20 EQ 0) AND (S3.QVAL_21 EQ 0) AND (S3.QVAL_22 EQ 0) AND (S3.QVAL_23 EQ 0) AND (S3.QVAL_24 EQ 0) AND (S3.QVAL_25 EQ 102.6)) OR ((S3.2PQVAL1 EQ 0) AND (S3.2PQVAL2 EQ 0) AND (S3.2PQVAL3 EQ 0) AND (S3.2PQVAL4 EQ 0) AND (S3.2PQVAL5 EQ 0) AND (S3.2PQVAL6 EQ 0) AND (S3.2PQVAL7 EQ 0) AND (S3.2PQVAL8 EQ 0) AND (S3.2PQVAL9 EQ 0) AND (S3.2PQVAL_10 EQ 0) AND (S3.2PQVAL_11 EQ 0) AND (S3.2PQVAL_12 EQ 0) AND (S3.2PQVAL_13 EQ 0) AND (S3.2PQVAL_14 EQ 0) AND (S3.2PQVAL_15 EQ 0) AND (S3.2PQVAL_16 EQ 0) AND (S3.2PQVAL_17 EQ 0) AND (S3.2PQVAL_18 EQ 0) AND (S3.2PQVAL_19 EQ 0) AND (S3.2PQVAL_20 EQ 0) AND (S3.2PQVAL_21 EQ 0) AND (S3.2PQVAL_22 EQ 0) AND (S3.2PQVAL_23 EQ 0) AND (S3.2PQVAL_24 EQ 0) AND (S3.2PQVAL_25 EQ 125))) [S3.P9TEMQ1 = 'Bien que l'activité physique ne m'excite pas beaucoup. Je pourrais essayer quand même et trouver des moyens d'aimer ça. Je suis sûr que ça serait bon pour moi en plus.'];
IF (((S3.SUMVAL GE 200) AND (S3.SUMVAL LT 300) AND (S3.QVAL1 EQ 0) AND (S3.QVAL2 EQ 0) AND (S3.QVAL3 EQ 0) AND (S3.QVAL4 EQ 0) AND (S3.QVAL5 EQ 0) AND (S3.QVAL6 EQ 0) AND (S3.QVAL7 EQ 0) AND (S3.QVAL8 EQ 0) AND (S3.QVAL9 EQ 0) AND (S3.QVAL_10 EQ 0) AND (S3.QVAL_11 EQ 0) AND (S3.QVAL_12 EQ 0) AND (S3.QVAL_13 EQ 0) AND (S3.QVAL_14 EQ 0) AND (S3.QVAL_15 EQ 0) AND (S3.QVAL_16 EQ 0) AND (S3.QVAL_17 EQ 0) AND (S3.QVAL_18 EQ 0) AND (S3.QVAL_19 EQ 0) AND (S3.QVAL_20 EQ 0) AND (S3.QVAL_21 EQ 0) AND (S3.QVAL_22 EQ 0) AND (S3.QVAL_23 EQ 0) AND (S3.QVAL_24 EQ 0) AND (S3.QVAL_25 EQ 0) AND (S3.QVAL_26 EQ 102.7)) OR ((S3.2PQVAL1 EQ 0) AND (S3.2PQVAL2 EQ 0) AND (S3.2PQVAL3 EQ 0) AND (S3.2PQVAL4 EQ 0) AND (S3.2PQVAL5 EQ 0) AND (S3.2PQVAL6 EQ 0) AND (S3.2PQVAL7 EQ 0) AND (S3.2PQVAL8 EQ 0) AND (S3.2PQVAL9 EQ 0) AND (S3.2PQVAL_10 EQ 0) AND (S3.2PQVAL_11 EQ 0) AND (S3.2PQVAL_12 EQ 0) AND (S3.2PQVAL_13 EQ 0) AND (S3.2PQVAL_14 EQ 0) AND (S3.2PQVAL_15 EQ 0) AND (S3.2PQVAL_16 EQ 0) AND (S3.2PQVAL_17 EQ 0) AND (S3.2PQVAL_18 EQ 0) AND (S3.2PQVAL_19 EQ 0) AND (S3.2PQVAL_20 EQ 0) AND (S3.2PQVAL_21 EQ 0) AND (S3.2PQVAL_22 EQ 0) AND (S3.2PQVAL_23 EQ 0) AND (S3.2PQVAL_24 EQ 0) AND (S3.2PQVAL_25 EQ 0) AND (S3.2PQVAL_26 EQ 126))) [S3.P9TEMQ1 = 'Je sais qu'on doit se sentir mieux quand on est actif. Ça m'aiderait à éprouver du plaisir plus facilement pour les petites choses de la vie.'];
IF (((S3.SUMVAL GE 200) AND (S3.SUMVAL LT 300) AND (S3.QVAL1 EQ 0) AND (S3.QVAL2 EQ 0) AND (S3.QVAL3 EQ 0) AND (S3.QVAL4 EQ 0) AND (S3.QVAL5 EQ 0) AND (S3.QVAL6 EQ 0) AND (S3.QVAL7 EQ 0) AND (S3.QVAL8 EQ 0) AND (S3.QVAL9 EQ 0) AND (S3.QVAL_10 EQ 0) AND (S3.QVAL_11 EQ 0) AND (S3.QVAL_12 EQ 0) AND (S3.QVAL_13 EQ 0) AND (S3.QVAL_14 EQ 0) AND (S3.QVAL_15 EQ 0) AND (S3.QVAL_16 EQ 0) AND (S3.QVAL_17 EQ 0) AND (S3.QVAL_18 EQ 0) AND (S3.QVAL_19 EQ 0) AND (S3.QVAL_20 EQ 0) AND (S3.QVAL_21 EQ 0) AND (S3.QVAL_22 EQ 0) AND (S3.QVAL_23 EQ 0) AND (S3.QVAL_24 EQ 0) AND (S3.QVAL_25 EQ 0) AND (S3.QVAL_26 EQ 0) AND (S3.QVAL_27 EQ 102.8)) OR ((S3.2PQVAL1 EQ 0) AND (S3.2PQVAL2 EQ 0) AND (S3.2PQVAL3 EQ 0) AND (S3.2PQVAL4 EQ 0) AND (S3.2PQVAL5 EQ 0) AND (S3.2PQVAL6 EQ 0) AND (S3.2PQVAL7 EQ 0) AND (S3.2PQVAL8 EQ 0) AND (S3.2PQVAL9 EQ 0) AND (S3.2PQVAL_10 EQ 0) AND (S3.2PQVAL_11 EQ 0) AND (S3.2PQVAL_12 EQ 0) AND

(S3.2PQVAL_13 EQ 0) AND (S3.2PQVAL_14 EQ 0) AND (S3.2PQVAL_15 EQ 0) AND (S3.2PQVAL_16 EQ 0) AND (S3.2PQVAL_17 EQ 0) AND (S3.2PQVAL_18 EQ 0) AND (S3.2PQVAL_19 EQ 0) AND (S3.2PQVAL_20 EQ 0) AND (S3.2PQVAL_21 EQ 0) AND (S3.2PQVAL_22 EQ 0) AND (S3.2PQVAL_23 EQ 0) AND (S3.2PQVAL_24 EQ 0) AND (S3.2PQVAL_25 EQ 0) AND (S3.2PQVAL_26 EQ 0) AND (S3.2PQVAL_27 EQ 127))) [S3.P9TEMQ1 = 'Je sais que c´est la bonne chose à faire pour moi. Je veux faire ça pour ma santé, pour mon avenir.'];
IF (((S3.SUMVAL GE 200) AND (S3.SUMVAL LT 300) AND (S3.QVAL1 EQ 0) AND (S3.QVAL2 EQ 0) AND (S3.QVAL3 EQ 0) AND (S3.QVAL4 EQ 0) AND (S3.QVAL5 EQ 0) AND (S3.QVAL6 EQ 0) AND (S3.QVAL7 EQ 0) AND (S3.QVAL8 EQ 0) AND (S3.QVAL9 EQ 0) AND (S3.QVAL_10 EQ 0) AND (S3.QVAL_11 EQ 0) AND (S3.QVAL_12 EQ 0) AND (S3.QVAL_13 EQ 0) AND (S3.QVAL_14 EQ 0) AND (S3.QVAL_15 EQ 0) AND (S3.QVAL_16 EQ 0) AND (S3.QVAL_17 EQ 0) AND (S3.QVAL_18 EQ 0) AND (S3.QVAL_19 EQ 0) AND (S3.QVAL_20 EQ 0) AND (S3.QVAL_21 EQ 0) AND (S3.QVAL_22 EQ 0) AND (S3.QVAL_23 EQ 0) AND (S3.QVAL_24 EQ 0) AND (S3.QVAL_25 EQ 0) AND (S3.QVAL_26 EQ 0) AND (S3.QVAL_27 EQ 0) AND (S3.QVAL_28 EQ 102.9)) OR ((S3.2PQVAL1 EQ 0) AND (S3.2PQVAL2 EQ 0) AND (S3.2PQVAL3 EQ 0) AND (S3.2PQVAL4 EQ 0) AND (S3.2PQVAL5 EQ 0) AND (S3.2PQVAL6 EQ 0) AND (S3.2PQVAL7 EQ 0) AND (S3.2PQVAL8 EQ 0) AND (S3.2PQVAL9 EQ 0) AND (S3.2PQVAL_10 EQ 0) AND (S3.2PQVAL_11 EQ 0) AND (S3.2PQVAL_12 EQ 0) AND (S3.2PQVAL_13 EQ 0) AND (S3.2PQVAL_14 EQ 0) AND (S3.2PQVAL_15 EQ 0) AND (S3.2PQVAL_16 EQ 0) AND (S3.2PQVAL_17 EQ 0) AND (S3.2PQVAL_18 EQ 0) AND (S3.2PQVAL_19 EQ 0) AND (S3.2PQVAL_20 EQ 0) AND (S3.2PQVAL_21 EQ 0) AND (S3.2PQVAL_22 EQ 0) AND (S3.2PQVAL_23 EQ 0) AND (S3.2PQVAL_24 EQ 0) AND (S3.2PQVAL_25 EQ 0) AND (S3.2PQVAL_26 EQ 0) AND (S3.2PQVAL_27 EQ 0) AND (S3.2PQVAL_28 EQ 128))) [S3.P9TEMQ1 = 'Je protégerais ma santé si je faisais des activités physiques régulièrement. J'augmente fortement mes chances de vivre une vie plus en santé.'];
IF (((S3.SUMVAL GE 200) AND (S3.SUMVAL LT 300) AND (S3.QVAL1 EQ 0) AND (S3.QVAL2 EQ 0) AND (S3.QVAL3 EQ 0) AND (S3.QVAL4 EQ 0) AND (S3.QVAL5 EQ 0) AND (S3.QVAL6 EQ 0) AND (S3.QVAL7 EQ 0) AND (S3.QVAL8 EQ 0) AND (S3.QVAL9 EQ 0) AND (S3.QVAL_10 EQ 0) AND (S3.QVAL_11 EQ 0) AND (S3.QVAL_12 EQ 0) AND (S3.QVAL_13 EQ 0) AND (S3.QVAL_14 EQ 0) AND (S3.QVAL_15 EQ 0) AND (S3.QVAL_16 EQ 0) AND (S3.QVAL_17 EQ 0) AND (S3.QVAL_18 EQ 0) AND (S3.QVAL_19 EQ 0) AND (S3.QVAL_20 EQ 0) AND (S3.QVAL_21 EQ 0) AND (S3.QVAL_22 EQ 0) AND (S3.QVAL_23 EQ 0) AND (S3.QVAL_24 EQ 0) AND (S3.QVAL_25 EQ 0) AND (S3.QVAL_26 EQ 0) AND (S3.QVAL_27 EQ 102.8) AND (S3.QVAL_28 EQ 0) AND (S3.QVAL_29 EQ 102.29)) OR ((S3.2PQVAL1 EQ 0) AND (S3.2PQVAL2 EQ 0) AND (S3.2PQVAL3 EQ 0) AND (S3.2PQVAL4 EQ 0) AND (S3.2PQVAL5 EQ 0) AND (S3.2PQVAL6 EQ 0) AND (S3.2PQVAL7 EQ 0) AND (S3.2PQVAL8 EQ 0) AND (S3.2PQVAL9 EQ 0) AND (S3.2PQVAL_10 EQ 0) AND (S3.2PQVAL_11 EQ 0) AND (S3.2PQVAL_12 EQ 0) AND (S3.2PQVAL_13 EQ 0) AND (S3.2PQVAL_14 EQ 0) AND (S3.2PQVAL_15 EQ 0) AND (S3.2PQVAL_16 EQ 0) AND (S3.2PQVAL_17 EQ 0) AND (S3.2PQVAL_18 EQ 0) AND (S3.2PQVAL_19 EQ 0) AND (S3.2PQVAL_20 EQ 0) AND (S3.2PQVAL_21 EQ 0) AND (S3.2PQVAL_22 EQ 0) AND (S3.2PQVAL_23 EQ 0) AND (S3.2PQVAL_24 EQ 0) AND (S3.2PQVAL_25 EQ 0) AND (S3.2PQVAL_26 EQ 0) AND (S3.2PQVAL_27 EQ 0) AND (S3.2PQVAL_28 EQ 0) AND (S3.2PQVAL_29 EQ 129))) [S3.P9TEMQ1 = 'En m´occupant de moi avant tout, je pourrai m´occuper du bien des autres beaucoup mieux et beaucoup plus longtemps.'];
IF (((S3.SUMVAL GE 200) AND (S3.SUMVAL LT 300) AND (S3.QVAL1 EQ 101) AND (S3.QVAL2 EQ 102)) OR ((S3.2PQVAL1 EQ 101) AND (S3.2PQVAL2 EQ 102))) [S3.P9TEMQ2 = 'Je serais vraiment content de moi. Je serais bien dans ma peau et les autres aimeraient probablement ça aussi.'];

IF (((S3.SUMVAL GE 200) AND (S3.SUMVAL LT 300) AND ((S3.QVAL1 EQ 101) OR (S3.QVAL2 EQ 102)) AND (S3.QVAL3 EQ 103)) OR (((S3.2PQVAL1 EQ 101) OR (S3.2PQVAL2 EQ 102)) AND (S3.2PQVAL3 EQ 103))) [S3.P9TEMQ2 = 'Je ferais partie des personnes qui ont la force de prendre leur santé en main. Je montrerais l'exemple à ma famille.'];
IF (((S3.SUMVAL GE 200) AND (S3.SUMVAL LT 300) AND ((S3.QVAL1 EQ 101) OR (S3.QVAL2 EQ 102) OR (S3.QVAL3 EQ 103)) AND (S3.QVAL4 EQ 104)) OR (((S3.2PQVAL1 EQ 101) OR (S3.2PQVAL2 EQ 102) OR (S3.2PQVAL3 EQ 103)) AND (S3.2PQVAL4 EQ 104))) [S3.P9TEMQ2 = 'Je serais plus vif. J´aurais encore plus d´assurance pour m´occuper et être responsable des autres.'];
IF (((S3.SUMVAL GE 200) AND (S3.SUMVAL LT 300) AND ((S3.QVAL1 EQ 101) OR (S3.QVAL2 EQ 102) OR (S3.QVAL3 EQ 103) OR (S3.QVAL4 EQ 104)) AND (S3.QVAL5 EQ 105)) OR (((S3.2PQVAL1 EQ 101) OR (S3.2PQVAL2 EQ 102) OR (S3.2PQVAL3 EQ 103) OR (S3.2PQVAL4 EQ 104)) AND (S3.2PQVAL5 EQ 105))) [S3.P9TEMQ2 = 'C´est sûr que je resterais en santé plus longtemps. Je serais moins à risque de dépendre des autres à cause qu´il m´arrive quelque chose.'];
IF (((S3.SUMVAL GE 200) AND (S3.SUMVAL LT 300) AND ((S3.QVAL1 EQ 101) OR (S3.QVAL2 EQ 102) OR (S3.QVAL3 EQ 103) OR (S3.QVAL4 EQ 104) OR (S3.QVAL5 EQ 105)) AND (S3.QVAL6 EQ 106)) OR (((S3.2PQVAL1 EQ 101) OR (S3.2PQVAL2 EQ 102) OR (S3.2PQVAL3 EQ 103) OR (S3.2PQVAL4 EQ 104) OR (S3.2PQVAL5 EQ 105)) AND (S3.2PQVAL6 EQ 106))) [S3.P9TEMQ2 = 'Je me sentirais mieux dans ma peau donc j´apprécierais encore plus tout ce qui m´entourent.'];
IF (((S3.SUMVAL GE 200) AND (S3.SUMVAL LT 300) AND ((S3.QVAL1 EQ 101) OR (S3.QVAL2 EQ 102) OR (S3.QVAL3 EQ 103) OR (S3.QVAL4 EQ 104) OR (S3.QVAL5 EQ 105) OR (S3.QVAL6 EQ 106)) AND (S3.QVAL7 EQ 107)) OR (((S3.2PQVAL1 EQ 101) OR (S3.2PQVAL2 EQ 102) OR (S3.2PQVAL3 EQ 103) OR (S3.2PQVAL4 EQ 104) OR (S3.2PQVAL5 EQ 105) OR (S3.2PQVAL6 EQ 106)) AND (S3.2PQVAL7 EQ 107))) [S3.P9TEMQ2 = 'Je me préoccuperais moins de ma santé et j´aurais l´esprit libre pour m´occuper des autres.'];
IF (((S3.SUMVAL GE 200) AND (S3.SUMVAL LT 300) AND ((S3.QVAL1 EQ 101) OR (S3.QVAL2 EQ 102) OR (S3.QVAL3 EQ 103) OR (S3.QVAL4 EQ 104) OR (S3.QVAL5 EQ 105) OR (S3.QVAL6 EQ 106) OR (S3.QVAL7 EQ 107)) AND (S3.QVAL8 EQ 108)) OR (((S3.2PQVAL1 EQ 101) OR (S3.2PQVAL2 EQ 102) OR (S3.2PQVAL3 EQ 103) OR (S3.2PQVAL4 EQ 104) OR (S3.2PQVAL5 EQ 105) OR (S3.2PQVAL6 EQ 106) OR (S3.2PQVAL7 EQ 107)) AND (S3.2PQVAL8 EQ 108))) [S3.P9TEMQ2 = 'C´est pas si excitant pour moi de bouger. D´un autre côté, ça m´aiderait c´est sûr. Ça pourrait démontrer ma capacité à faire preuve de compromis dans la vie.'];
IF (((S3.SUMVAL GE 200) AND (S3.SUMVAL LT 300) AND ((S3.QVAL1 EQ 101) OR (S3.QVAL2 EQ 102) OR (S3.QVAL3 EQ 103) OR (S3.QVAL4 EQ 104) OR (S3.QVAL5 EQ 105) OR (S3.QVAL6 EQ 106) OR (S3.QVAL7 EQ 107) OR (S3.QVAL8 EQ 108)) AND (S3.QVAL9 EQ 109)) OR (((S3.2PQVAL1 EQ 101) OR (S3.2PQVAL2 EQ 102) OR (S3.2PQVAL3 EQ 103) OR (S3.2PQVAL4 EQ 104) OR (S3.2PQVAL5 EQ 105) OR (S3.2PQVAL6 EQ 106) OR (S3.2PQVAL7 EQ 107) OR (S3.2PQVAL8 EQ 108)) AND (S3.2PQVAL9 EQ 109))) [S3.P9TEMQ2 = 'J´aurais la conscience beaucoup plus tranquille et je me sentirais certainement mieux dans la vie de tous les jours.'];
IF (((S3.SUMVAL GE 200) AND (S3.SUMVAL LT 300) AND ((S3.QVAL1 EQ 101) OR (S3.QVAL2 EQ 102) OR (S3.QVAL3 EQ 103) OR (S3.QVAL4 EQ 104) OR (S3.QVAL5 EQ 105) OR (S3.QVAL6 EQ 106) OR (S3.QVAL7 EQ 107) OR (S3.QVAL8 EQ 108) OR (S3.QVAL9 EQ 109)) AND (S3.QVAL_10 EQ 101.1)) OR (((S3.2PQVAL1 EQ 101) OR (S3.2PQVAL2 EQ 102) OR (S3.2PQVAL3 EQ 103) OR (S3.2PQVAL4 EQ 104) OR (S3.2PQVAL5 EQ 105) OR (S3.2PQVAL6 EQ 106) OR (S3.2PQVAL7 EQ 107) OR (S3.2PQVAL8 EQ 108) OR (S3.2PQVAL9 EQ 109)) AND (S3.2PQVAL_10 EQ 110))) [S3.P9TEMQ2 = 'J'ai un diabète oui, mais je peux me prendre en mains malgré tout. Je ferais preuve de courage en pratiquant régulièrement des activités physiques c'est sûr.'];

IF (((S3.SUMVAL GE 200) AND (S3.SUMVAL LT 300) AND ((S3.QVAL1 EQ 101) OR (S3.QVAL2 EQ 102) OR (S3.QVAL3 EQ 103) OR (S3.QVAL4 EQ 104) OR (S3.QVAL5 EQ 105) OR (S3.QVAL6 EQ 106) OR (S3.QVAL7 EQ 107) OR (S3.QVAL8 EQ 108) OR (S3.QVAL9 EQ 109) OR (S3.QVAL_10 EQ 101.1)) AND (S3.QVAL_11 EQ 101.2)) OR (((S3.2PQVAL1 EQ 101) OR (S3.2PQVAL2 EQ 102) OR (S3.2PQVAL3 EQ 103) OR (S3.2PQVAL4 EQ 104) OR (S3.2PQVAL5 EQ 105) OR (S3.2PQVAL6 EQ 106) OR (S3.2PQVAL7 EQ 107) OR (S3.2PQVAL8 EQ 108) OR (S3.2PQVAL9 EQ 109) OR (S3.2PQVAL_10 EQ 110)) AND (S3.2PQVAL_11 EQ 111))) [S3.P9TEMQ2 = 'Je sais que l'activité physique aide à garder un esprit vigoureux. Ça pourrait sûrement m'aider à rester inspiré constamment.'];
IF (((S3.SUMVAL GE 200) AND (S3.SUMVAL LT 300) AND ((S3.QVAL1 EQ 101) OR (S3.QVAL2 EQ 102) OR (S3.QVAL3 EQ 103) OR (S3.QVAL4 EQ 104) OR (S3.QVAL5 EQ 105) OR (S3.QVAL6 EQ 106) OR (S3.QVAL7 EQ 107) OR (S3.QVAL8 EQ 108) OR (S3.QVAL9 EQ 109) OR (S3.QVAL_10 EQ 101.1) OR (S3.QVAL_11 EQ 101.2)) AND (S3.QVAL_12 EQ 101.3)) OR (((S3.2PQVAL1 EQ 101) OR (S3.2PQVAL2 EQ 102) OR (S3.2PQVAL3 EQ 103) OR (S3.2PQVAL4 EQ 104) OR (S3.2PQVAL5 EQ 105) OR (S3.2PQVAL6 EQ 106) OR (S3.2PQVAL7 EQ 107) OR (S3.2PQVAL8 EQ 108) OR (S3.2PQVAL9 EQ 109) OR (S3.2PQVAL_10 EQ 110) OR (S3.2PQVAL_11 EQ 111)) AND (S3.2PQVAL_12 EQ 112))) [S3.P9TEMQ2 = 'Je m'assurerais de garder une bonne santé et de me tenir en forme. Juste ça serait une évolution pour moi.'];
IF (((S3.SUMVAL GE 200) AND (S3.SUMVAL LT 300) AND ((S3.QVAL1 EQ 101) OR (S3.QVAL2 EQ 102) OR (S3.QVAL3 EQ 103) OR (S3.QVAL4 EQ 104) OR (S3.QVAL5 EQ 105) OR (S3.QVAL6 EQ 106) OR (S3.QVAL7 EQ 107) OR (S3.QVAL8 EQ 108) OR (S3.QVAL9 EQ 109) OR (S3.QVAL_10 EQ 101.1) OR (S3.QVAL_11 EQ 101.2) OR (S3.QVAL_12 EQ 101.3)) AND (S3.QVAL_13 EQ 101.4)) OR (((S3.2PQVAL1 EQ 101) OR (S3.2PQVAL2 EQ 102) OR (S3.2PQVAL3 EQ 103) OR (S3.2PQVAL4 EQ 104) OR (S3.2PQVAL5 EQ 105) OR (S3.2PQVAL6 EQ 106) OR (S3.2PQVAL7 EQ 107) OR (S3.2PQVAL8 EQ 108) OR (S3.2PQVAL9 EQ 109) OR (S3.2PQVAL_10 EQ 110) OR (S3.2PQVAL_11 EQ 111) OR (S3.2PQVAL_12 EQ 112)) AND (S3.2PQVAL_13 EQ 113))) [S3.P9TEMQ2 = 'J'aimerais pratiquer des activités physiques régulièrement pour voir ce qui m'arriverait, comment je me sentirais. Ça serait intéressant.'];
IF (((S3.SUMVAL GE 200) AND (S3.SUMVAL LT 300) AND ((S3.QVAL1 EQ 101) OR (S3.QVAL2 EQ 102) OR (S3.QVAL3 EQ 103) OR (S3.QVAL4 EQ 104) OR (S3.QVAL5 EQ 105) OR (S3.QVAL6 EQ 106) OR (S3.QVAL7 EQ 107) OR (S3.QVAL8 EQ 108) OR (S3.QVAL9 EQ 109) OR (S3.QVAL_10 EQ 101.1) OR (S3.QVAL_11 EQ 101.2) OR (S3.QVAL_12 EQ 101.3) OR (S3.QVAL_13 EQ 101.4)) AND (S3.QVAL_14 EQ 101.5)) OR (((S3.2PQVAL1 EQ 101) OR (S3.2PQVAL2 EQ 102) OR (S3.2PQVAL3 EQ 103) OR (S3.2PQVAL4 EQ 104) OR (S3.2PQVAL5 EQ 105) OR (S3.2PQVAL6 EQ 106) OR (S3.2PQVAL7 EQ 107) OR (S3.2PQVAL8 EQ 108) OR (S3.2PQVAL9 EQ 109) OR (S3.2PQVAL_10 EQ 110) OR (S3.2PQVAL_11 EQ 111) OR (S3.2PQVAL_12 EQ 112) OR (S3.2PQVAL_13 EQ 113)) AND (S3.2PQVAL_14 EQ 114))) [S3.P9TEMQ2 = 'Faire des activités physiques aide à avoir un bon mental. Ça m'aiderait à voir les choses positivement même dans des moments difficiles.'];
IF (((S3.SUMVAL GE 200) AND (S3.SUMVAL LT 300) AND ((S3.QVAL1 EQ 101) OR (S3.QVAL2 EQ 102) OR (S3.QVAL3 EQ 103) OR (S3.QVAL4 EQ 104) OR (S3.QVAL5 EQ 105) OR (S3.QVAL6 EQ 106) OR (S3.QVAL7 EQ 107) OR (S3.QVAL8 EQ 108) OR (S3.QVAL9 EQ 109) OR (S3.QVAL_10 EQ 101.1) OR (S3.QVAL_11 EQ 101.2) OR (S3.QVAL_12 EQ 101.3) OR (S3.QVAL_13 EQ 101.4) OR (S3.QVAL_14 EQ 101.5)) AND (S3.QVAL_15 EQ 101.6)) OR (((S3.2PQVAL1 EQ 101) OR (S3.2PQVAL2 EQ 102) OR (S3.2PQVAL3 EQ 103) OR (S3.2PQVAL4 EQ 104) OR (S3.2PQVAL5 EQ 105) OR (S3.2PQVAL6 EQ 106) OR (S3.2PQVAL7 EQ 107) OR (S3.2PQVAL8 EQ 108) OR (S3.2PQVAL9 EQ 109) OR (S3.2PQVAL_10 EQ 110) OR (S3.2PQVAL_11 EQ 111) OR (S3.2PQVAL_12 EQ

112) OR (S3.2PQVAL_13 EQ 113) OR (S3.2PQVAL_14 EQ 114)) AND (S3.2PQVAL_15 EQ 115))) [S3.P9TEMQ2 = 'Je ressentirais de la fierté si je faisais des activités physiques régulièrement. C'est certain.'];
IF (((S3.SUMVAL GE 200) AND (S3.SUMVAL LT 300) AND ((S3.QVAL1 EQ 101) OR (S3.QVAL2 EQ 102) OR (S3.QVAL3 EQ 103) OR (S3.QVAL4 EQ 104) OR (S3.QVAL5 EQ 105) OR (S3.QVAL6 EQ 106) OR (S3.QVAL7 EQ 107) OR (S3.QVAL8 EQ 108) OR (S3.QVAL9 EQ 109) OR (S3.QVAL_10 EQ 101.1) OR (S3.QVAL_11 EQ 101.2) OR (S3.QVAL_12 EQ 101.3) OR (S3.QVAL_13 EQ 101.4) OR (S3.QVAL_14 EQ 101.5) OR (S3.QVAL_15 EQ 101.6)) AND (S3.QVAL_16 EQ 101.7)) OR (((S3.2PQVAL1 EQ 101) OR (S3.2PQVAL2 EQ 102) OR (S3.2PQVAL3 EQ 103) OR (S3.2PQVAL4 EQ 104) OR (S3.2PQVAL5 EQ 105) OR (S3.2PQVAL6 EQ 106) OR (S3.2PQVAL7 EQ 107) OR (S3.2PQVAL8 EQ 108) OR (S3.2PQVAL9 EQ 109) OR (S3.2PQVAL_10 EQ 110) OR (S3.2PQVAL_11 EQ 111) OR (S3.2PQVAL_12 EQ 112) OR (S3.2PQVAL_13 EQ 113) OR (S3.2PQVAL_14 EQ 114) OR (S3.2PQVAL_15 EQ 115)) AND (S3.2PQVAL_16 EQ 116))) [S3.P9TEMQ2 = 'Je serais fier de montrer l'exemple à ma famille. Ça pourrait aussi nous permettre de faire des activités différentes ensemble.'];
IF (((S3.SUMVAL GE 200) AND (S3.SUMVAL LT 300) AND ((S3.QVAL1 EQ 101) OR (S3.QVAL2 EQ 102) OR (S3.QVAL3 EQ 103) OR (S3.QVAL4 EQ 104) OR (S3.QVAL5 EQ 105) OR (S3.QVAL6 EQ 106) OR (S3.QVAL7 EQ 107) OR (S3.QVAL8 EQ 108) OR (S3.QVAL9 EQ 109) OR (S3.QVAL_10 EQ 101.1) OR (S3.QVAL_11 EQ 101.2) OR (S3.QVAL_12 EQ 101.3) OR (S3.QVAL_13 EQ 101.4) OR (S3.QVAL_14 EQ 101.5) OR (S3.QVAL_15 EQ 101.6) OR (S3.QVAL_16 EQ 101.7)) AND (S3.QVAL_17 EQ 101.8)) OR (((S3.2PQVAL1 EQ 101) OR (S3.2PQVAL2 EQ 102) OR (S3.2PQVAL3 EQ 103) OR (S3.2PQVAL4 EQ 104) OR (S3.2PQVAL5 EQ 105) OR (S3.2PQVAL6 EQ 106) OR (S3.2PQVAL7 EQ 107) OR (S3.2PQVAL8 EQ 108) OR (S3.2PQVAL9 EQ 109) OR (S3.2PQVAL_10 EQ 110) OR (S3.2PQVAL_11 EQ 111) OR (S3.2PQVAL_12 EQ 112) OR (S3.2PQVAL_13 EQ 113) OR (S3.2PQVAL_14 EQ 114) OR (S3.2PQVAL_15 EQ 115) OR (S3.2PQVAL_16 EQ 116)) AND (S3.2PQVAL_17 EQ 117))) [S3.P9TEMQ2 = 'J'aurais une humeur plus stable. Je serais plus discipliné pour les tâches que je dois accomplir.'];
IF (((S3.SUMVAL GE 200) AND (S3.SUMVAL LT 300) AND ((S3.QVAL1 EQ 101) OR (S3.QVAL2 EQ 102) OR (S3.QVAL3 EQ 103) OR (S3.QVAL4 EQ 104) OR (S3.QVAL5 EQ 105) OR (S3.QVAL6 EQ 106) OR (S3.QVAL7 EQ 107) OR (S3.QVAL8 EQ 108) OR (S3.QVAL9 EQ 109) OR (S3.QVAL_10 EQ 101.1) OR (S3.QVAL_11 EQ 101.2) OR (S3.QVAL_12 EQ 101.3) OR (S3.QVAL_13 EQ 101.4) OR (S3.QVAL_14 EQ 101.5) OR (S3.QVAL_15 EQ 101.6) OR (S3.QVAL_16 EQ 101.7) OR (S3.QVAL_17 EQ 101.8)) AND (S3.QVAL_18 EQ 101.9)) OR (((S3.2PQVAL1 EQ 101) OR (S3.2PQVAL2 EQ 102) OR (S3.2PQVAL3 EQ 103) OR (S3.2PQVAL4 EQ 104) OR (S3.2PQVAL5 EQ 105) OR (S3.2PQVAL6 EQ 106) OR (S3.2PQVAL7 EQ 107) OR (S3.2PQVAL8 EQ 108) OR (S3.2PQVAL9 EQ 109) OR (S3.2PQVAL_10 EQ 110) OR (S3.2PQVAL_11 EQ 111) OR (S3.2PQVAL_12 EQ 112) OR (S3.2PQVAL_13 EQ 113) OR (S3.2PQVAL_14 EQ 114) OR (S3.2PQVAL_15 EQ 115) OR (S3.2PQVAL_16 EQ 116) OR (S3.2PQVAL_17 EQ 117)) AND (S3.2PQVAL_18 EQ 118))) [S3.P9TEMQ2 = 'Ça améliorerait mon humeur. Ça m'aiderait à réagir moins fortement à certaines choses qui me dérangent plus.'];
IF (((S3.SUMVAL GE 200) AND (S3.SUMVAL LT 300) AND ((S3.QVAL1 EQ 101) OR (S3.QVAL2 EQ 102) OR (S3.QVAL3 EQ 103) OR (S3.QVAL4 EQ 104) OR (S3.QVAL5 EQ 105) OR (S3.QVAL6 EQ 106) OR (S3.QVAL7 EQ 107) OR (S3.QVAL8 EQ 108) OR (S3.QVAL9 EQ 109) OR (S3.QVAL_10 EQ 101.1) OR (S3.QVAL_11 EQ 101.2) OR (S3.QVAL_12 EQ 101.3) OR (S3.QVAL_13 EQ 101.4) OR (S3.QVAL_14 EQ 101.5) OR (S3.QVAL_15 EQ 101.6) OR (S3.QVAL_16 EQ 101.7) OR (S3.QVAL_17 EQ 101.8) OR (S3.QVAL_18 EQ 101.9)) AND (S3.QVAL_19 EQ 101.19))OR (((S3.2PQVAL1 EQ 101) OR (S3.2PQVAL2 EQ 102) OR (S3.2PQVAL3 EQ 103) OR (S3.2PQVAL4 EQ 104) OR (S3.2PQVAL5 EQ 105) OR (S3.2PQVAL6 EQ 106) OR (S3.2PQVAL7 EQ 107) OR (S3.2PQVAL8 EQ 108) OR (S3.2PQVAL9 EQ 109) OR (S3.2PQVAL_10 EQ 110) OR (S3.2PQVAL_11 EQ 111) OR

(S3.2PQVAL_12 EQ 112) OR (S3.2PQVAL_13 EQ 113) OR (S3.2PQVAL_14 EQ 114) OR (S3.2PQVAL_15 EQ 115) OR (S3.2PQVAL_16 EQ 116) OR (S3.2PQVAL_17 EQ 117) OR (S3.2PQVAL_18 EQ 118)) AND (S3.2PQVAL_19 EQ 119))) [S3.P9TEMQ2 = 'Je serais mieux dans ma peau. Être bien m'aiderait à être une personne encore plus généreuse.'];
IF (((S3.SUMVAL GE 200) AND (S3.SUMVAL LT 300) AND ((S3.QVAL1 EQ 101) OR (S3.QVAL2 EQ 102) OR (S3.QVAL3 EQ 103) OR (S3.QVAL4 EQ 104) OR (S3.QVAL5 EQ 105) OR (S3.QVAL6 EQ 106) OR (S3.QVAL7 EQ 107) OR (S3.QVAL8 EQ 108) OR (S3.QVAL9 EQ 109) OR (S3.QVAL_10 EQ 101.1) OR (S3.QVAL_11 EQ 101.2) OR (S3.QVAL_12 EQ 101.3) OR (S3.QVAL_13 EQ 101.4) OR (S3.QVAL_14 EQ 101.5) OR (S3.QVAL_15 EQ 101.6) OR (S3.QVAL_16 EQ 101.7) OR (S3.QVAL_17 EQ 101.8) OR (S3.QVAL_18 EQ 101.9) OR (S3.QVAL_19 EQ 101.19)) AND (S3.QVAL_20 EQ 102.1)) OR (((S3.2PQVAL1 EQ 101) OR (S3.2PQVAL2 EQ 102) OR (S3.2PQVAL3 EQ 103) OR (S3.2PQVAL4 EQ 104) OR (S3.2PQVAL5 EQ 105) OR (S3.2PQVAL6 EQ 106) OR (S3.2PQVAL7 EQ 107) OR (S3.2PQVAL8 EQ 108) OR (S3.2PQVAL9 EQ 109) OR (S3.2PQVAL_10 EQ 110) OR (S3.2PQVAL_11 EQ 111) OR (S3.2PQVAL_12 EQ 112) OR (S3.2PQVAL_13 EQ 113) OR (S3.2PQVAL_14 EQ 114) OR (S3.2PQVAL_15 EQ 115) OR (S3.2PQVAL_16 EQ 116) OR (S3.2PQVAL_17 EQ 117) OR (S3.2PQVAL_18 EQ 118) OR (S3.2PQVAL_19 EQ 119)) AND (S3.2PQVAL_20 EQ 120))) [S3.P9TEMQ2 = 'Ça m´aiderait à avoir une meilleure concentration et une meilleure attention. J'aurais l'esprit plus libre.'];
IF (((S3.SUMVAL GE 200) AND (S3.SUMVAL LT 300) AND ((S3.QVAL1 EQ 101) OR (S3.QVAL2 EQ 102) OR (S3.QVAL3 EQ 103) OR (S3.QVAL4 EQ 104) OR (S3.QVAL5 EQ 105) OR (S3.QVAL6 EQ 106) OR (S3.QVAL7 EQ 107) OR (S3.QVAL8 EQ 108) OR (S3.QVAL9 EQ 109) OR (S3.QVAL_10 EQ 101.1) OR (S3.QVAL_11 EQ 101.2) OR (S3.QVAL_12 EQ 101.3) OR (S3.QVAL_13 EQ 101.4) OR (S3.QVAL_14 EQ 101.5) OR (S3.QVAL_15 EQ 101.6) OR (S3.QVAL_16 EQ 101.7) OR (S3.QVAL_17 EQ 101.8) OR (S3.QVAL_18 EQ 101.9) OR (S3.QVAL_19 EQ 101.19) OR (S3.QVAL_20 EQ 102.1)) AND (S3.QVAL_21 EQ 102.2)) OR (((S3.2PQVAL1 EQ 101) OR (S3.2PQVAL2 EQ 102) OR (S3.2PQVAL3 EQ 103) OR (S3.2PQVAL4 EQ 104) OR (S3.2PQVAL5 EQ 105) OR (S3.2PQVAL6 EQ 106) OR (S3.2PQVAL7 EQ 107) OR (S3.2PQVAL8 EQ 108) OR (S3.2PQVAL9 EQ 109) OR (S3.2PQVAL_10 EQ 110) OR (S3.2PQVAL_11 EQ 111) OR (S3.2PQVAL_12 EQ 112) OR (S3.2PQVAL_13 EQ 113) OR (S3.2PQVAL_14 EQ 114) OR (S3.2PQVAL_15 EQ 115) OR (S3.2PQVAL_16 EQ 116) OR (S3.2PQVAL_17 EQ 117) OR (S3.2PQVAL_18 EQ 118) OR (S3.2PQVAL_19 EQ 119) OR (S3.2PQVAL_20 EQ 120)) AND (S3.2PQVAL_21 EQ 121))) [S3.P9TEMQ2 = 'Je serais un exemple pour les personnes qui ont de la misère à pratiquer régulièrement des activités physiques. Je veux être un modèle de réussite.'];
IF (((S3.SUMVAL GE 200) AND (S3.SUMVAL LT 300) AND ((S3.QVAL1 EQ 101) OR (S3.QVAL2 EQ 102) OR (S3.QVAL3 EQ 103) OR (S3.QVAL4 EQ 104) OR (S3.QVAL5 EQ 105) OR (S3.QVAL6 EQ 106) OR (S3.QVAL7 EQ 107) OR (S3.QVAL8 EQ 108) OR (S3.QVAL9 EQ 109) OR (S3.QVAL_10 EQ 101.1) OR (S3.QVAL_11 EQ 101.2) OR (S3.QVAL_12 EQ 101.3) OR (S3.QVAL_13 EQ 101.4) OR (S3.QVAL_14 EQ 101.5) OR (S3.QVAL_15 EQ 101.6) OR (S3.QVAL_16 EQ 101.7) OR (S3.QVAL_17 EQ 101.8) OR (S3.QVAL_18 EQ 101.9) OR (S3.QVAL_19 EQ 101.19) OR (S3.QVAL_20 EQ 102.1) OR (S3.QVAL_21 EQ 102.2)) AND (S3.QVAL_22 EQ 102.3)) OR (((S3.2PQVAL1 EQ 101) OR (S3.2PQVAL2 EQ 102) OR (S3.2PQVAL3 EQ 103) OR (S3.2PQVAL4 EQ 104) OR (S3.2PQVAL5 EQ 105) OR (S3.2PQVAL6 EQ 106) OR (S3.2PQVAL7 EQ 107) OR (S3.2PQVAL8 EQ 108) OR (S3.2PQVAL9 EQ 109) OR (S3.2PQVAL_10 EQ 110) OR (S3.2PQVAL_11 EQ 111) OR (S3.2PQVAL_12 EQ 112) OR (S3.2PQVAL_13 EQ 113) OR (S3.2PQVAL_14 EQ 114) OR (S3.2PQVAL_15 EQ 115) OR (S3.2PQVAL_16 EQ 116) OR (S3.2PQVAL_17 EQ 117) OR (S3.2PQVAL_18 EQ 118) OR (S3.2PQVAL_19 EQ 119) OR (S3.2PQVAL_20 EQ 120) OR (S3.2PQVAL_21 EQ 121)) AND (S3.2PQVAL_22 EQ 122))) [S3.P9TEMQ2 =

'J'éviterais bien des problèmes de santé. Je ne serais pas encombré par des limitations que je pourrais éviter.'];
IF (((S3.SUMVAL GE 200) AND (S3.SUMVAL LT 300) AND ((S3.QVAL1 EQ 101) OR (S3.QVAL2 EQ 102) OR (S3.QVAL3 EQ 103) OR (S3.QVAL4 EQ 104) OR (S3.QVAL5 EQ 105) OR (S3.QVAL6 EQ 106) OR (S3.QVAL7 EQ 107) OR (S3.QVAL8 EQ 108) OR (S3.QVAL9 EQ 109) OR (S3.QVAL_10 EQ 101.1) OR (S3.QVAL_11 EQ 101.2) OR (S3.QVAL_12 EQ 101.3) OR (S3.QVAL_13 EQ 101.4) OR (S3.QVAL_14 EQ 101.5) OR (S3.QVAL_15 EQ 101.6) OR (S3.QVAL_16 EQ 101.7) OR (S3.QVAL_17 EQ 101.8) OR (S3.QVAL_18 EQ 101.9) OR (S3.QVAL_19 EQ 101.19) OR (S3.QVAL_20 EQ 102.1) OR (S3.QVAL_21 EQ 102.2) OR (S3.QVAL_22 EQ 102.3)) AND (S3.QVAL_23 EQ 102.4)) OR (((S3.2PQVAL1 EQ 101) OR (S3.2PQVAL2 EQ 102) OR (S3.2PQVAL3 EQ 103) OR (S3.2PQVAL4 EQ 104) OR (S3.2PQVAL5 EQ 105) OR (S3.2PQVAL6 EQ 106) OR (S3.2PQVAL7 EQ 107) OR (S3.2PQVAL8 EQ 108) OR (S3.2PQVAL9 EQ 109) OR (S3.2PQVAL_10 EQ 110) OR (S3.2PQVAL_11 EQ 111) OR (S3.2PQVAL_12 EQ 112) OR (S3.2PQVAL_13 EQ 113) OR (S3.2PQVAL_14 EQ 114) OR (S3.2PQVAL_15 EQ 115) OR (S3.2PQVAL_16 EQ 116) OR (S3.2PQVAL_17 EQ 117) OR (S3.2PQVAL_18 EQ 118) OR (S3.2PQVAL_19 EQ 119) OR (S3.2PQVAL_20 EQ 120) OR (S3.2PQVAL_21 EQ 121) OR (S3.2PQVAL_22 EQ 122)) AND (S3.2PQVAL_23 EQ 123))) [S3.P9TEMQ2 = 'Je veux offrir mon meilleur aux autres. Ça m'aiderait à rester en santé, à être encore plus optimiste dans la vie, et avec ceux que j'aime.'];
IF (((S3.SUMVAL GE 200) AND (S3.SUMVAL LT 300) AND ((S3.QVAL1 EQ 101) OR (S3.QVAL2 EQ 102) OR (S3.QVAL3 EQ 103) OR (S3.QVAL4 EQ 104) OR (S3.QVAL5 EQ 105) OR (S3.QVAL6 EQ 106) OR (S3.QVAL7 EQ 107) OR (S3.QVAL8 EQ 108) OR (S3.QVAL9 EQ 109) OR (S3.QVAL_10 EQ 101.1) OR (S3.QVAL_11 EQ 101.2) OR (S3.QVAL_12 EQ 101.3) OR (S3.QVAL_13 EQ 101.4) OR (S3.QVAL_14 EQ 101.5) OR (S3.QVAL_15 EQ 101.6) OR (S3.QVAL_16 EQ 101.7) OR (S3.QVAL_17 EQ 101.8) OR (S3.QVAL_18 EQ 101.9) OR (S3.QVAL_19 EQ 101.19) OR (S3.QVAL_20 EQ 102.1) OR (S3.QVAL_21 EQ 102.2) OR (S3.QVAL_22 EQ 102.3) OR (S3.QVAL_23 EQ 102.4)) AND (S3.QVAL_24 EQ 102.5)) OR (((S3.2PQVAL1 EQ 101) OR (S3.2PQVAL2 EQ 102) OR (S3.2PQVAL3 EQ 103) OR (S3.2PQVAL4 EQ 104) OR (S3.2PQVAL5 EQ 105) OR (S3.2PQVAL6 EQ 106) OR (S3.2PQVAL7 EQ 107) OR (S3.2PQVAL8 EQ 108) OR (S3.2PQVAL9 EQ 109) OR (S3.2PQVAL_10 EQ 110) OR (S3.2PQVAL_11 EQ 111) OR (S3.2PQVAL_12 EQ 112) OR (S3.2PQVAL_13 EQ 113) OR (S3.2PQVAL_14 EQ 114) OR (S3.2PQVAL_15 EQ 115) OR (S3.2PQVAL_16 EQ 116) OR (S3.2PQVAL_17 EQ 117) OR (S3.2PQVAL_18 EQ 118) OR (S3.2PQVAL_19 EQ 119) OR (S3.2PQVAL_20 EQ 120) OR (S3.2PQVAL_21 EQ 121) OR (S3.2PQVAL_22 EQ 122) OR (S3.2PQVAL_23 EQ 123)) AND (S3.2PQVAL_24 EQ 124))) [S3.P9TEMQ2 = 'Je sais que c'est la bonne chose à faire. Faire des activités physiques m'aiderait à être plus en cohérence avec la personne que je veux vraiment être.'];
IF (((S3.SUMVAL GE 200) AND (S3.SUMVAL LT 300) AND ((S3.QVAL1 EQ 101) OR (S3.QVAL2 EQ 102) OR (S3.QVAL3 EQ 103) OR (S3.QVAL4 EQ 104) OR (S3.QVAL5 EQ 105) OR (S3.QVAL6 EQ 106) OR (S3.QVAL7 EQ 107) OR (S3.QVAL8 EQ 108) OR (S3.QVAL9 EQ 109) OR (S3.QVAL_10 EQ 101.1) OR (S3.QVAL_11 EQ 101.2) OR (S3.QVAL_12 EQ 101.3) OR (S3.QVAL_13 EQ 101.4) OR (S3.QVAL_14 EQ 101.5) OR (S3.QVAL_15 EQ 101.6) OR (S3.QVAL_16 EQ 101.7) OR (S3.QVAL_17 EQ 101.8) OR (S3.QVAL_18 EQ 101.9) OR (S3.QVAL_19 EQ 101.19) OR (S3.QVAL_20 EQ 102.1) OR (S3.QVAL_21 EQ 102.2) OR (S3.QVAL_22 EQ 102.3) OR (S3.QVAL_23 EQ 102.4) OR (S3.QVAL_24 EQ 102.5)) AND (S3.QVAL_25 EQ 102.6)) OR (((S3.2PQVAL1 EQ 101) OR (S3.2PQVAL2 EQ 102) OR (S3.2PQVAL3 EQ 103) OR (S3.2PQVAL4 EQ 104) OR (S3.2PQVAL5 EQ 105) OR (S3.2PQVAL6 EQ 106) OR (S3.2PQVAL7 EQ 107) OR (S3.2PQVAL8 EQ 108) OR (S3.2PQVAL9 EQ 109) OR (S3.2PQVAL_10 EQ 110) OR (S3.2PQVAL_11 EQ 111) OR (S3.2PQVAL_12 EQ 112) OR (S3.2PQVAL_13 EQ 113) OR (S3.2PQVAL_14 EQ 114) OR (S3.2PQVAL_15 EQ 115) OR (S3.2PQVAL_16 EQ 116) OR

(S3.2PQVAL_17 EQ 117) OR (S3.2PQVAL_18 EQ 118) OR (S3.2PQVAL_19 EQ 119) OR (S3.2PQVAL_20 EQ 120) OR (S3.2PQVAL_21 EQ 121) OR (S3.2PQVAL_22 EQ 122) OR (S3.2PQVAL_23 EQ 123) OR (S3.2PQVAL_24 EQ 124)) AND (S3.2PQVAL_25 EQ 125))) [S3.P9TEMQ2 = 'Bien que l'activité physique ne m'excite pas beaucoup. Je pourrais essayer quand même et trouver des moyens d'aimer ça. Je suis sûr que ça serait bon pour moi en plus.'];
IF (((S3.SUMVAL GE 200) AND (S3.SUMVAL LT 300) AND ((S3.QVAL1 EQ 101) OR (S3.QVAL2 EQ 102) OR (S3.QVAL3 EQ 103) OR (S3.QVAL4 EQ 104) OR (S3.QVAL5 EQ 105) OR (S3.QVAL6 EQ 106) OR (S3.QVAL7 EQ 107) OR (S3.QVAL8 EQ 108) OR (S3.QVAL9 EQ 109) OR (S3.QVAL_10 EQ 101.1) OR (S3.QVAL_11 EQ 101.2) OR (S3.QVAL_12 EQ 101.3) OR (S3.QVAL_13 EQ 101.4) OR (S3.QVAL_14 EQ 101.5) OR (S3.QVAL_15 EQ 101.6) OR (S3.QVAL_16 EQ 101.7) OR (S3.QVAL_17 EQ 101.8) OR (S3.QVAL_18 EQ 101.9) OR (S3.QVAL_19 EQ 101.19) OR (S3.QVAL_20 EQ 102.1) OR (S3.QVAL_21 EQ 102.2) OR (S3.QVAL_22 EQ 102.3) OR (S3.QVAL_23 EQ 102.4) OR (S3.QVAL_24 EQ 102.5) OR (S3.QVAL_25 EQ 102.6)) AND (S3.QVAL_26 EQ 102.7)) OR (((S3.2PQVAL1 EQ 101) OR (S3.2PQVAL2 EQ 102) OR (S3.2PQVAL3 EQ 103) OR (S3.2PQVAL4 EQ 104) OR (S3.2PQVAL5 EQ 105) OR (S3.2PQVAL6 EQ 106) OR (S3.2PQVAL7 EQ 107) OR (S3.2PQVAL8 EQ 108) OR (S3.2PQVAL9 EQ 109) OR (S3.2PQVAL_10 EQ 110) OR (S3.2PQVAL_11 EQ 111) OR (S3.2PQVAL_12 EQ 112) OR (S3.2PQVAL_13 EQ 113) OR (S3.2PQVAL_14 EQ 114) OR (S3.2PQVAL_15 EQ 115) OR (S3.2PQVAL_16 EQ 116) OR (S3.2PQVAL_17 EQ 117) OR (S3.2PQVAL_18 EQ 118) OR (S3.2PQVAL_19 EQ 119) OR (S3.2PQVAL_20 EQ 120) OR (S3.2PQVAL_21 EQ 121) OR (S3.2PQVAL_22 EQ 122) OR (S3.2PQVAL_23 EQ 123) OR (S3.2PQVAL_24 EQ 124) OR (S3.2PQVAL_25 EQ 125)) AND (S3.2PQVAL_26 EQ 126))) [S3.P9TEMQ2 = 'Je sais qu'on doit se sentir mieux quand on est actif. Ça m'aiderait à éprouver du plaisir plus facilement pour les petites choses de la vie.'];
IF (((S3.SUMVAL GE 200) AND (S3.SUMVAL LT 300) AND ((S3.QVAL1 EQ 101) OR (S3.QVAL2 EQ 102) OR (S3.QVAL3 EQ 103) OR (S3.QVAL4 EQ 104) OR (S3.QVAL5 EQ 105) OR (S3.QVAL6 EQ 106) OR (S3.QVAL7 EQ 107) OR (S3.QVAL8 EQ 108) OR (S3.QVAL9 EQ 109) OR (S3.QVAL_10 EQ 101.1) OR (S3.QVAL_11 EQ 101.2) OR (S3.QVAL_12 EQ 101.3) OR (S3.QVAL_13 EQ 101.4) OR (S3.QVAL_14 EQ 101.5) OR (S3.QVAL_15 EQ 101.6) OR (S3.QVAL_16 EQ 101.7) OR (S3.QVAL_17 EQ 101.8) OR (S3.QVAL_18 EQ 101.9) OR (S3.QVAL_19 EQ 101.19) OR (S3.QVAL_20 EQ 102.1) OR (S3.QVAL_21 EQ 102.2) OR (S3.QVAL_22 EQ 102.3) OR (S3.QVAL_23 EQ 102.4) OR (S3.QVAL_24 EQ 102.5) OR (S3.QVAL_25 EQ 102.6) OR (S3.QVAL_26 EQ 102.7)) AND (S3.QVAL_27 EQ 102.8)) OR (((S3.2PQVAL1 EQ 101) OR (S3.2PQVAL2 EQ 102) OR (S3.2PQVAL3 EQ 103) OR (S3.2PQVAL4 EQ 104) OR (S3.2PQVAL5 EQ 105) OR (S3.2PQVAL6 EQ 106) OR (S3.2PQVAL7 EQ 107) OR (S3.2PQVAL8 EQ 108) OR (S3.2PQVAL9 EQ 109) OR (S3.2PQVAL_10 EQ 110) OR (S3.2PQVAL_11 EQ 111) OR (S3.2PQVAL_12 EQ 112) OR (S3.2PQVAL_13 EQ 113) OR (S3.2PQVAL_14 EQ 114) OR (S3.2PQVAL_15 EQ 115) OR (S3.2PQVAL_16 EQ 116) OR (S3.2PQVAL_17 EQ 117) OR (S3.2PQVAL_18 EQ 118) OR (S3.2PQVAL_19 EQ 119) OR (S3.2PQVAL_20 EQ 120) OR (S3.2PQVAL_21 EQ 121) OR (S3.2PQVAL_22 EQ 122) OR (S3.2PQVAL_23 EQ 123) OR (S3.2PQVAL_24 EQ 124) OR (S3.2PQVAL_25 EQ 125) OR (S3.2PQVAL_26 EQ 126)) AND (S3.2PQVAL_27 EQ 127))) [S3.P9TEMQ2 = 'Je sais que c´est la bonne chose à faire pour moi. Je veux faire ça pour ma santé, pour mon avenir.'];
IF (((S3.SUMVAL GE 200) AND (S3.SUMVAL LT 300) AND ((S3.QVAL1 EQ 101) OR (S3.QVAL2 EQ 102) OR (S3.QVAL3 EQ 103) OR (S3.QVAL4 EQ 104) OR (S3.QVAL5 EQ 105) OR (S3.QVAL6 EQ 106) OR (S3.QVAL7 EQ 107) OR (S3.QVAL8 EQ 108) OR (S3.QVAL9 EQ 109) OR (S3.QVAL_10 EQ 101.1) OR (S3.QVAL_11 EQ 101.2) OR (S3.QVAL_12 EQ 101.3) OR (S3.QVAL_13 EQ 101.4) OR (S3.QVAL_14 EQ 101.5) OR (S3.QVAL_15 EQ 101.6) OR (S3.QVAL_16 EQ 101.7) OR (S3.QVAL_17 EQ 101.8) OR (S3.QVAL_18 EQ 101.9) OR (S3.QVAL_19

EQ 101.19) OR (S3.QVAL_20 EQ 102.1) OR (S3.QVAL_21 EQ 102.2) OR (S3.QVAL_22 EQ 102.3) OR (S3.QVAL_23 EQ 102.4) OR (S3.QVAL_24 EQ 102.5) OR (S3.QVAL_25 EQ 102.6) OR (S3.QVAL_26 EQ 102.7) OR (S3.QVAL_27 EQ 102.8)) AND (S3.QVAL_28 EQ 102.9)) OR (((S3.2PQVAL1 EQ 101) OR (S3.2PQVAL2 EQ 102) OR (S3.2PQVAL3 EQ 103) OR (S3.2PQVAL4 EQ 104) OR (S3.2PQVAL5 EQ 105) OR (S3.2PQVAL6 EQ 106) OR (S3.2PQVAL7 EQ 107) OR (S3.2PQVAL8 EQ 108) OR (S3.2PQVAL9 EQ 109) OR (S3.2PQVAL_10 EQ 110) OR (S3.2PQVAL_11 EQ 111) OR (S3.2PQVAL_12 EQ 112) OR (S3.2PQVAL_13 EQ 113) OR (S3.2PQVAL_14 EQ 114) OR (S3.2PQVAL_15 EQ 115) OR (S3.2PQVAL_16 EQ 116) OR (S3.2PQVAL_17 EQ 117) OR (S3.2PQVAL_18 EQ 118) OR (S3.2PQVAL_19 EQ 119) OR (S3.2PQVAL_20 EQ 120) OR (S3.2PQVAL_21 EQ 121) OR (S3.2PQVAL_22 EQ 122) OR (S3.2PQVAL_23 EQ 123) OR (S3.2PQVAL_24 EQ 124) OR (S3.2PQVAL_25 EQ 125) OR (S3.2PQVAL_26 EQ 126) OR (S3.2PQVAL_27 EQ 127)) AND (S3.2PQVAL_28 EQ 128))) [S3.P9TEMQ2 = 'Je protégerais ma santé si je faisais des activités physiques régulièrement. J’augmente fortement mes chances de vivre une vie plus en santé.'];
IF (((S3.SUMVAL GE 200) AND (S3.SUMVAL LT 300) AND ((S3.QVAL1 EQ 101) OR (S3.QVAL2 EQ 102) OR (S3.QVAL3 EQ 103) OR (S3.QVAL4 EQ 104) OR (S3.QVAL5 EQ 105) OR (S3.QVAL6 EQ 106) OR (S3.QVAL7 EQ 107) OR (S3.QVAL8 EQ 108) OR (S3.QVAL9 EQ 109) OR (S3.QVAL_10 EQ 101.1) OR (S3.QVAL_11 EQ 101.2) OR (S3.QVAL_12 EQ 101.3) OR (S3.QVAL_13 EQ 101.4) OR (S3.QVAL_14 EQ 101.5) OR (S3.QVAL_15 EQ 101.6) OR (S3.QVAL_16 EQ 101.7) OR (S3.QVAL_17 EQ 101.8) OR (S3.QVAL_18 EQ 101.9) OR (S3.QVAL_19 EQ 101.19) OR (S3.QVAL_20 EQ 102.1) OR (S3.QVAL_21 EQ 102.2) OR (S3.QVAL_22 EQ 102.3) OR (S3.QVAL_23 EQ 102.4) OR (S3.QVAL_24 EQ 102.5) OR (S3.QVAL_25 EQ 102.6) OR (S3.QVAL_26 EQ 102.7) OR (S3.QVAL_27 EQ 102.8) OR (S3.QVAL_28 EQ 102.9)) AND (S3.QVAL_29 EQ 102.29)) OR (((S3.2PQVAL1 EQ 101) OR (S3.2PQVAL2 EQ 102) OR (S3.2PQVAL3 EQ 103) OR (S3.2PQVAL4 EQ 104) OR (S3.2PQVAL5 EQ 105) OR (S3.2PQVAL6 EQ 106) OR (S3.2PQVAL7 EQ 107) OR (S3.2PQVAL8 EQ 108) OR (S3.2PQVAL9 EQ 109) OR (S3.2PQVAL_10 EQ 110) OR (S3.2PQVAL_11 EQ 111) OR (S3.2PQVAL_12 EQ 112) OR (S3.2PQVAL_13 EQ 113) OR (S3.2PQVAL_14 EQ 114) OR (S3.2PQVAL_15 EQ 115) OR (S3.2PQVAL_16 EQ 116) OR (S3.2PQVAL_17 EQ 117) OR (S3.2PQVAL_18 EQ 118) OR (S3.2PQVAL_19 EQ 119) OR (S3.2PQVAL_20 EQ 120) OR (S3.2PQVAL_21 EQ 121) OR (S3.2PQVAL_22 EQ 122) OR (S3.2PQVAL_23 EQ 123) OR (S3.2PQVAL_24 EQ 124) OR (S3.2PQVAL_25 EQ 125) OR (S3.2PQVAL_26 EQ 126) OR (S3.2PQVAL_27 EQ 127) OR (S3.2PQVAL_28 EQ 128)) AND (S3.2PQVAL_29 EQ 129))) [S3.P9TEMQ2 = 'En m´occupant de moi avant tout, je pourrai m´occuper du bien des autres beaucoup mieux et beaucoup plus longtemps.'];
SÉANCE 5 - équations
IF (S5_P9QFEELINFO EQ 0) [S5.INFORESINTRO = 'Vous esquivez les informations supplémentaires pour aujourd´hui. Aucun souci! Voici donc votre résumé de la séance.'];
IF (S5_P9QFEELINFO EQ 1) [S5.INFORESINTRO = 'Il semble que vous ayez apprécié les informations précédentes! Merci de nous en faire part! Nous sommes heureux de l´entendre. Voici maintenant votre résumé de la séance d´aujourd´hui.'];
IF (S5_P9QFEELINFO EQ 2) [S5.INFORESINTRO = 'Mmmm...Il semble que vous n´ayez pas aimé les informations précédentes. Nous espérons sincèrement pouvoir faire mieux à l´avenir. Voici maintenant, si vous voulez, un court résumé de votre séance.'];
IF (S5_P9QFEELINFO EQ 3) [S5.INFORESINTRO = 'Mmmm...Il semble que vous ignorez quoi penser des informations précédentes. Nous espérons que cela pourra quand même vous servir. Voici maintenant, si vous voulez, un court résumé de votre séance.'];

IF (SI_MI_EFFIC LT 2) [S5.SUPRAEFF ='Au moment, de l´inscription, vous aviez peu confiance en votre capacité à bouger davantage au cours du prochain mois. D´un autre côté, nous avons trouvé ensemble des forces qui pourraient vous donner davantage de confiance.'];
IF ((SI_MI_EFFIC GE 3) AND (SI_MI_EFFIC LT 6)) [S5.SUPRAEFF ='Au moment de l´inscription, vous aviez peu confiance en votre capacité à devenir [ACTIF_VE] physiquement au cours du prochain mois. D´un autre côté, vous sembliez malgré tout avoir un brin de confiance en votre capacité de réussir.'];
IF ((SI_MI_EFFIC GT 5) AND (SI_MI_EFFIC LT 8)) [S5.SUPRAEFF ='Au moment de l´inscription, vous possédiez déjà une certaine confiance positive en votre capacité à pratiquer des activités physiques régulièrement. C´est excellent!'];
IF ((SI_MI_EFFIC GT 7) AND (SI_MI_EFFIC LT 11)) [S5.SUPRAEFF ='Au moment de l´inscription, vous possédiez déjà une grande confiance en votre capacité de pratiquer régulièrement des activités physiques. C´est excellent!'];
IF (S5_P7ELAB1 NE 0) [S5.SUPRAELAB1.2 = ' pourrait vous servir à pratiquer des activités physiques régulièrement de cette façon : '];
IF (S5_P7ELAB1 EQ 0) [S5.SUPRAELAB1.1 = 'Comment '];
IF (S5_P7ELAB1 EQ 0) [S5.SUPRAELAB1.2 = ' pourrait vous servir à pratiquer des activités physiques régulièrement? Cette force peut vous aider.'];
IF (S5_P7ELAB2 NE 0) [S5.SUPRAELAB2.2 = ' pourrait vous servir à pratiquer des activités physiques régulièrement de cette façon : '];
IF (S5_P7ELAB2 EQ 0) [S5.SUPRAELAB2.1 = 'Comment '];
IF (S5_P7ELAB2 EQ 0) [S5.SUPRAELAB2.2 = ' pourrait vous servir à pratiquer des activités physiques régulièrement? Cette force peut vous aider.'];
[S5.SUMVAL = S5.QVAL1+S5.QVAL2+S5.QVAL3+S5.QVAL4+S5.QVAL5+S5.QVAL6+S5.QVAL7+S5.QVAL8+S5.QVAL9+S5.QVAL_10+S5.QVAL_11+S5.QVAL_12+S5.QVAL_13+S5.QVAL_14+S5.QVAL_15+S5.QVAL_16+S5.QVAL_17+S5.QVAL_18+S5.QVAL_19+S5.QVAL_20+S5.QVAL_21+S5.QVAL_22+S5.QVAL_23+S5.QVAL_24+S5.QVAL_25];
[S5.2PSUMVAL = S5.2PQVAL1+S5.2PQVAL2+S5.2PQVAL3+S5.2PQVAL4+S5.2PQVAL5+S5.2PQVAL6+S5.2PQVAL7+S5.2PQVAL8+S5.2PQVAL9+S5.2PQVAL_10+S5.2PQVAL_11+S5.2PQVAL_12+S5.2PQVAL_13+S5.2PQVAL_14+S5.2PQVAL_15+S5.2PQVAL_16+S5.2PQVAL_17+S5.2PQVAL_18+S5.2PQVAL_19+S5.2PQVAL_20+S5.2PQVAL_21+S5.2PQVAL_22+S5.2PQVAL_23+S5.2PQVAL_24+S5.2PQVAL_25];
IF (((S5.SUMVAL GE 200) AND (S5.SUMVAL LT 300) AND (S5.QVAL1 EQ 101)) OR (S5.2PQVAL1 EQ 101)) [S5.VAL_1 = 'être une personne aimante'];
IF (((S5.SUMVAL GE 200) AND (S5.SUMVAL LT 300) AND (S5.QVAL1 EQ 0) AND (S5.QVAL2 EQ 102)) OR ((S5.2PQVAL1 EQ 0) AND (S5.2PQVAL2 EQ 102))) [S5.VAL_1 = 'être une personne prête à aider les autres'];
IF (((S5.SUMVAL GE 200) AND (S5.SUMVAL LT 300) AND (S5.QVAL1 EQ 0) AND (S5.QVAL2 EQ 0) AND (S5.QVAL3 EQ 103)) OR ((S5.2PQVAL1 EQ 0) AND (S5.2PQVAL2 EQ 0) AND (S5.2PQVAL3 EQ 103))) [S5.VAL_1 = 'être une bonne oreille pour les autres'];
IF (((S5.SUMVAL GE 200) AND (S5.SUMVAL LT 300) AND (S5.QVAL1 EQ 0) AND (S5.QVAL2 EQ 0) AND (S5.QVAL3 EQ 0) AND (S5.QVAL4 EQ 104)) OR ((S5.2PQVAL1 EQ 0) AND (S5.2PQVAL2 EQ 0) AND (S5.2PQVAL3 EQ 0) AND (S5.2PQVAL4 EQ 104))) [S5.VAL_1 = 'être une personne qui croyez en votre capacité d´accomplir toutes choses'];

IF (((S5.SUMVAL GE 200) AND (S5.SUMVAL LT 300) AND (S5.QVAL1 EQ 0) AND (S5.QVAL2 EQ 0) AND (S5.QVAL3 EQ 0) AND (S5.QVAL4 EQ 0) AND (S5.QVAL5 EQ 105)) OR ((S5.2PQVAL1 EQ 0) AND (S5.2PQVAL2 EQ 0) AND (S5.2PQVAL3 EQ 0) AND (S5.2PQVAL4 EQ 0) AND (S5.2PQVAL5 EQ 105))) [S5.VAL_1 = 'être une personne douée dans plusieurs domaines'];
IF (((S5.SUMVAL GE 200) AND (S5.SUMVAL LT 300) AND (S5.QVAL1 EQ 0) AND (S5.QVAL2 EQ 0) AND (S5.QVAL3 EQ 0) AND (S5.QVAL4 EQ 0) AND (S5.QVAL5 EQ 0) AND (S5.QVAL6 EQ 106)) OR ((S5.2PQVAL1 EQ 0) AND (S5.2PQVAL2 EQ 0) AND (S5.2PQVAL3 EQ 0) AND (S5.2PQVAL4 EQ 0) AND (S5.2PQVAL5 EQ 0) AND (S5.2PQVAL6 EQ 106))) [S5.VAL_1 = 'être une personne qui possède une forte confiance en vous'];
IF (((S5.SUMVAL GE 200) AND (S5.SUMVAL LT 300) AND (S5.QVAL1 EQ 0) AND (S5.QVAL2 EQ 0) AND (S5.QVAL3 EQ 0) AND (S5.QVAL4 EQ 0) AND (S5.QVAL5 EQ 0) AND (S5.QVAL6 EQ 0) AND (S5.QVAL7 EQ 107)) OR ((S5.2PQVAL1 EQ 0) AND (S5.2PQVAL2 EQ 0) AND (S5.2PQVAL3 EQ 0) AND (S5.2PQVAL4 EQ 0) AND (S5.2PQVAL5 EQ 0) AND (S5.2PQVAL6 EQ 0) AND (S5.2PQVAL7 EQ 107))) [S5.VAL_1 = 'être une personne généralement décidée'];
IF (((S5.SUMVAL GE 200) AND (S5.SUMVAL LT 300) AND (S5.QVAL1 EQ 0) AND (S5.QVAL2 EQ 0) AND (S5.QVAL3 EQ 0) AND (S5.QVAL4 EQ 0) AND (S5.QVAL5 EQ 0) AND (S5.QVAL6 EQ 0) AND (S5.QVAL7 EQ 0) AND (S5.QVAL8 EQ 108)) OR ((S5.2PQVAL1 EQ 0) AND (S5.2PQVAL2 EQ 0) AND (S5.2PQVAL3 EQ 0) AND (S5.2PQVAL4 EQ 0) AND (S5.2PQVAL5 EQ 0) AND (S5.2PQVAL6 EQ 0) AND (S5.2PQVAL7 EQ 0) AND (S5.2PQVAL8 EQ 108))) [S5.VAL_1 = 'être une personne qui vient à bout de ce qu´elle entreprend'];
IF (((S5.SUMVAL GE 200) AND (S5.SUMVAL LT 300) AND (S5.QVAL1 EQ 0) AND (S5.QVAL2 EQ 0) AND (S5.QVAL3 EQ 0) AND (S5.QVAL4 EQ 0) AND (S5.QVAL5 EQ 0) AND (S5.QVAL6 EQ 0) AND (S5.QVAL7 EQ 0) AND (S5.QVAL8 EQ 0) AND (S5.QVAL9 EQ 109)) OR ((S5.2PQVAL1 EQ 0) AND (S5.2PQVAL2 EQ 0) AND (S5.2PQVAL3 EQ 0) AND (S5.2PQVAL4 EQ 0) AND (S5.2PQVAL5 EQ 0) AND (S5.2PQVAL6 EQ 0) AND (S5.2PQVAL7 EQ 0) AND (S5.2PQVAL8 EQ 0) AND (S5.2PQVAL9 EQ 109))) [S5.VAL_1 = 'être une personne qui donne tout ce qu´elle a pour réussir'];
IF (((S5.SUMVAL GE 200) AND (S5.SUMVAL LT 300) AND (S5.QVAL1 EQ 0) AND (S5.QVAL2 EQ 0) AND (S5.QVAL3 EQ 0) AND (S5.QVAL4 EQ 0) AND (S5.QVAL5 EQ 0) AND (S5.QVAL6 EQ 0) AND (S5.QVAL7 EQ 0) AND (S5.QVAL8 EQ 0) AND (S5.QVAL9 EQ 0) AND (S5.QVAL_10 EQ 101.1)) OR ((S5.2PQVAL1 EQ 0) AND (S5.2PQVAL2 EQ 0) AND (S5.2PQVAL3 EQ 0) AND (S5.2PQVAL4 EQ 0) AND (S5.2PQVAL5 EQ 0) AND (S5.2PQVAL6 EQ 0) AND (S5.2PQVAL7 EQ 0) AND (S5.2PQVAL8 EQ 0) AND (S5.2PQVAL9 EQ 0) AND (S5.2PQVAL_10 EQ 110))) [S5.VAL_1 = 'être une personne assidue qui accompli toute chose avec succès'];
IF (((S5.SUMVAL GE 200) AND (S5.SUMVAL LT 300) AND (S5.QVAL1 EQ 0) AND (S5.QVAL2 EQ 0) AND (S5.QVAL3 EQ 0) AND (S5.QVAL4 EQ 0) AND (S5.QVAL5 EQ 0) AND (S5.QVAL6 EQ 0) AND (S5.QVAL7 EQ 0) AND (S5.QVAL8 EQ 0) AND (S5.QVAL9 EQ 0) AND (S5.QVAL_10 EQ 0) AND (S5.QVAL_11 EQ 101.2)) OR ((S5.2PQVAL1 EQ 0) AND (S5.2PQVAL2 EQ 0) AND (S5.2PQVAL3 EQ 0) AND (S5.2PQVAL4 EQ 0) AND (S5.2PQVAL5 EQ 0) AND (S5.2PQVAL6 EQ 0) AND (S5.2PQVAL7 EQ 0) AND (S5.2PQVAL8 EQ 0) AND (S5.2PQVAL9 EQ 0) AND (S5.2PQVAL_10 EQ 0) AND (S5.2PQVAL_11 EQ 111))) [S5.VAL_1 = 'être une personne vivante qui a beaucoup d´énergie à donner'];
IF (((S5.SUMVAL GE 200) AND (S5.SUMVAL LT 300) AND (S5.QVAL1 EQ 0) AND (S5.QVAL2 EQ 0) AND (S5.QVAL3 EQ 0) AND (S5.QVAL4 EQ 0) AND (S5.QVAL5 EQ 0) AND (S5.QVAL6 EQ 0) AND (S5.QVAL7 EQ 0) AND (S5.QVAL8 EQ 0) AND (S5.QVAL9 EQ 0) AND (S5.QVAL_10 EQ 0) AND (S5.QVAL_11 EQ 0) AND (S5.QVAL_12 EQ 101.3)) OR ((S5.2PQVAL1 EQ 0) AND (S5.2PQVAL2 EQ 0) AND (S5.2PQVAL3 EQ 0) AND (S5.2PQVAL4 EQ 0) AND (S5.2PQVAL5 EQ 0) AND (S5.2PQVAL6 EQ 0) AND (S5.2PQVAL7 EQ 0) AND

(S5.2PQVAL8 EQ 0) AND (S5.2PQVAL9 EQ 0) AND (S5.2PQVAL_10 EQ 0) AND (S5.2PQVAL_11 EQ 0) AND (S5.2PQVAL_12 EQ 112))) [S5.VAL_1 = 'être une personne avec une bonne humeur contagieuse'];
IF (((S5.SUMVAL GE 200) AND (S5.SUMVAL LT 300) AND (S5.QVAL1 EQ 0) AND (S5.QVAL2 EQ 0) AND (S5.QVAL3 EQ 0) AND (S5.QVAL4 EQ 0) AND (S5.QVAL5 EQ 0) AND (S5.QVAL6 EQ 0) AND (S5.QVAL7 EQ 0) AND (S5.QVAL8 EQ 0) AND (S5.QVAL9 EQ 0) AND (S5.QVAL_10 EQ 0) AND (S5.QVAL_11 EQ 0) AND (S5.QVAL_12 EQ 0) AND (S5.QVAL_13 EQ 101.4)) OR ((S5.2PQVAL1 EQ 0) AND (S5.2PQVAL2 EQ 0) AND (S5.2PQVAL3 EQ 0) AND (S5.2PQVAL4 EQ 0) AND (S5.2PQVAL5 EQ 0) AND (S5.2PQVAL6 EQ 0) AND (S5.2PQVAL7 EQ 0) AND (S5.2PQVAL8 EQ 0) AND (S5.2PQVAL9 EQ 0) AND (S5.2PQVAL_10 EQ 0) AND (S5.2PQVAL_11 EQ 0) AND (S5.2PQVAL_12 EQ 0) AND (S5.2PQVAL_13 EQ 113))) [S5.VAL_1 = 'être une personne qui finit toujours par réussir'];
IF (((S5.SUMVAL GE 200) AND (S5.SUMVAL LT 300) AND (S5.QVAL1 EQ 0) AND (S5.QVAL2 EQ 0) AND (S5.QVAL3 EQ 0) AND (S5.QVAL4 EQ 0) AND (S5.QVAL5 EQ 0) AND (S5.QVAL6 EQ 0) AND (S5.QVAL7 EQ 0) AND (S5.QVAL8 EQ 0) AND (S5.QVAL9 EQ 0) AND (S5.QVAL_10 EQ 0) AND (S5.QVAL_11 EQ 0) AND (S5.QVAL_12 EQ 0) AND (S5.QVAL_13 EQ 0) AND (S5.QVAL_14 EQ 101.5)) OR ((S5.2PQVAL1 EQ 0) AND (S5.2PQVAL2 EQ 0) AND (S5.2PQVAL3 EQ 0) AND (S5.2PQVAL4 EQ 0) AND (S5.2PQVAL5 EQ 0) AND (S5.2PQVAL6 EQ 0) AND (S5.2PQVAL7 EQ 0) AND (S5.2PQVAL8 EQ 0) AND (S5.2PQVAL9 EQ 0) AND (S5.2PQVAL_10 EQ 0) AND (S5.2PQVAL_11 EQ 0) AND (S5.2PQVAL_12 EQ 0) AND (S5.2PQVAL_13 EQ 0) AND (S5.2PQVAL_14 EQ 114))) [S5.VAL_1 = 'être une personne qui prend des décisions responsables'];
IF (((S5.SUMVAL GE 200) AND (S5.SUMVAL LT 300) AND (S5.QVAL1 EQ 0) AND (S5.QVAL2 EQ 0) AND (S5.QVAL3 EQ 0) AND (S5.QVAL4 EQ 0) AND (S5.QVAL5 EQ 0) AND (S5.QVAL6 EQ 0) AND (S5.QVAL7 EQ 0) AND (S5.QVAL8 EQ 0) AND (S5.QVAL9 EQ 0) AND (S5.QVAL_10 EQ 0) AND (S5.QVAL_11 EQ 0) AND (S5.QVAL_12 EQ 0) AND (S5.QVAL_13 EQ 0) AND (S5.QVAL_14 EQ 0) AND (S5.QVAL_15 EQ 101.6)) OR ((S5.2PQVAL1 EQ 0) AND (S5.2PQVAL2 EQ 0) AND (S5.2PQVAL3 EQ 0) AND (S5.2PQVAL4 EQ 0) AND (S5.2PQVAL5 EQ 0) AND (S5.2PQVAL6 EQ 0) AND (S5.2PQVAL7 EQ 0) AND (S5.2PQVAL8 EQ 0) AND (S5.2PQVAL9 EQ 0) AND (S5.2PQVAL_10 EQ 0) AND (S5.2PQVAL_11 EQ 0) AND (S5.2PQVAL_12 EQ 0) AND (S5.2PQVAL_13 EQ 0) AND (S5.2PQVAL_14 EQ 0) AND (S5.2PQVAL_15 EQ 115))) [S5.VAL_1 = 'être une personne qui planifie efficacement ses activités'];
IF (((S5.SUMVAL GE 200) AND (S5.SUMVAL LT 300) AND (S5.QVAL1 EQ 0) AND (S5.QVAL2 EQ 0) AND (S5.QVAL3 EQ 0) AND (S5.QVAL4 EQ 0) AND (S5.QVAL5 EQ 0) AND (S5.QVAL6 EQ 0) AND (S5.QVAL7 EQ 0) AND (S5.QVAL8 EQ 0) AND (S5.QVAL9 EQ 0) AND (S5.QVAL_10 EQ 0) AND (S5.QVAL_11 EQ 0) AND (S5.QVAL_12 EQ 0) AND (S5.QVAL_13 EQ 0) AND (S5.QVAL_14 EQ 0) AND (S5.QVAL_15 EQ 0) AND (S5.QVAL_16 EQ 101.7)) OR ((S5.2PQVAL1 EQ 0) AND (S5.2PQVAL2 EQ 0) AND (S5.2PQVAL3 EQ 0) AND (S5.2PQVAL4 EQ 0) AND (S5.2PQVAL5 EQ 0) AND (S5.2PQVAL6 EQ 0) AND (S5.2PQVAL7 EQ 0) AND (S5.2PQVAL8 EQ 0) AND (S5.2PQVAL9 EQ 0) AND (S5.2PQVAL_10 EQ 0) AND (S5.2PQVAL_11 EQ 0) AND (S5.2PQVAL_12 EQ 0) AND (S5.2PQVAL_13 EQ 0) AND (S5.2PQVAL_14 EQ 0) AND (S5.2PQVAL_15 EQ 0) AND (S5.2PQVAL_16 EQ 116))) [S5.VAL_1 = 'être une personne qui n´hésite pas à vivre de nouvelles expériences'];
IF (((S5.SUMVAL GE 200) AND (S5.SUMVAL LT 300) AND (S5.QVAL1 EQ 0) AND (S5.QVAL2 EQ 0) AND (S5.QVAL3 EQ 0) AND (S5.QVAL4 EQ 0) AND (S5.QVAL5 EQ 0) AND (S5.QVAL6 EQ 0) AND (S5.QVAL7 EQ 0) AND (S5.QVAL8 EQ 0) AND (S5.QVAL9 EQ 0) AND (S5.QVAL_10 EQ 0) AND (S5.QVAL_11 EQ 0) AND (S5.QVAL_12 EQ 0) AND (S5.QVAL_13 EQ 0) AND (S5.QVAL_14 EQ 0) AND (S5.QVAL_15 EQ 0) AND (S5.QVAL_16 EQ 0) AND (S5.QVAL_17 EQ 101.8)) OR ((S5.2PQVAL1 EQ 0) AND (S5.2PQVAL2 EQ 0) AND

(S5.2PQVAL3 EQ 0) AND (S5.2PQVAL4 EQ 0) AND (S5.2PQVAL5 EQ 0) AND (S5.2PQVAL6 EQ 0) AND (S5.2PQVAL7 EQ 0) AND (S5.2PQVAL8 EQ 0) AND (S5.2PQVAL9 EQ 0) AND (S5.2PQVAL_10 EQ 0) AND (S5.2PQVAL_11 EQ 0) AND (S5.2PQVAL_12 EQ 0) AND (S5.2PQVAL_13 EQ 0) AND (S5.2PQVAL_14 EQ 0) AND (S5.2PQVAL_15 EQ 0) AND (S5.2PQVAL_16 EQ 0) AND (S5.2PQVAL_17 EQ 117))) [S5.VAL_1 = 'être une personne qui fonce malgré les difficultés'];
IF (((S5.SUMVAL GE 200) AND (S5.SUMVAL LT 300) AND (S5.QVAL1 EQ 0) AND (S5.QVAL2 EQ 0) AND (S5.QVAL3 EQ 0) AND (S5.QVAL4 EQ 0) AND (S5.QVAL5 EQ 0) AND (S5.QVAL6 EQ 0) AND (S5.QVAL7 EQ 0) AND (S5.QVAL8 EQ 0) AND (S5.QVAL9 EQ 0) AND (S5.QVAL_10 EQ 0) AND (S5.QVAL_11 EQ 0) AND (S5.QVAL_12 EQ 0) AND (S5.QVAL_13 EQ 0) AND (S5.QVAL_14 EQ 0) AND (S5.QVAL_15 EQ 0) AND (S5.QVAL_16 EQ 0) AND (S5.QVAL_17 EQ 0) AND (S5.QVAL_18 EQ 101.9)) OR ((S5.2PQVAL1 EQ 0) AND (S5.2PQVAL2 EQ 0) AND (S5.2PQVAL3 EQ 0) AND (S5.2PQVAL4 EQ 0) AND (S5.2PQVAL5 EQ 0) AND (S5.2PQVAL6 EQ 0) AND (S5.2PQVAL7 EQ 0) AND (S5.2PQVAL8 EQ 0) AND (S5.2PQVAL9 EQ 0) AND (S5.2PQVAL_10 EQ 0) AND (S5.2PQVAL_11 EQ 0) AND (S5.2PQVAL_12 EQ 0) AND (S5.2PQVAL_13 EQ 0) AND (S5.2PQVAL_14 EQ 0) AND (S5.2PQVAL_15 EQ 0) AND (S5.2PQVAL_16 EQ 0) AND (S5.2PQVAL_17 EQ 0) AND (S5.2PQVAL_18 EQ 118))) [S5.VAL_1 = 'être une personne qui agit en pensant à long terme'];
IF (((S5.SUMVAL GE 200) AND (S5.SUMVAL LT 300) AND (S5.QVAL1 EQ 0) AND (S5.QVAL2 EQ 0) AND (S5.QVAL3 EQ 0) AND (S5.QVAL4 EQ 0) AND (S5.QVAL5 EQ 0) AND (S5.QVAL6 EQ 0) AND (S5.QVAL7 EQ 0) AND (S5.QVAL8 EQ 0) AND (S5.QVAL9 EQ 0) AND (S5.QVAL_10 EQ 0) AND (S5.QVAL_11 EQ 0) AND (S5.QVAL_12 EQ 0) AND (S5.QVAL_13 EQ 0) AND (S5.QVAL_14 EQ 0) AND (S5.QVAL_15 EQ 0) AND (S5.QVAL_16 EQ 0) AND (S5.QVAL_17 EQ 0) AND (S5.QVAL_18 EQ 0) AND (S5.QVAL_19 EQ 101.19)) OR ((S5.2PQVAL1 EQ 0) AND (S5.2PQVAL2 EQ 0) AND (S5.2PQVAL3 EQ 0) AND (S5.2PQVAL4 EQ 0) AND (S5.2PQVAL5 EQ 0) AND (S5.2PQVAL6 EQ 0) AND (S5.2PQVAL7 EQ 0) AND (S5.2PQVAL8 EQ 0) AND (S5.2PQVAL9 EQ 0) AND (S5.2PQVAL_10 EQ 0) AND (S5.2PQVAL_11 EQ 0) AND (S5.2PQVAL_12 EQ 0) AND (S5.2PQVAL_13 EQ 0) AND (S5.2PQVAL_14 EQ 0) AND (S5.2PQVAL_15 EQ 0) AND (S5.2PQVAL_16 EQ 0) AND (S5.2PQVAL_17 EQ 0) AND (S5.2PQVAL_18 EQ 0) AND (S5.2PQVAL_19 EQ 119))) [S5.VAL_1 = 'être une personne qui apprécie sa situation présente'];
IF (((S5.SUMVAL GE 200) AND (S5.SUMVAL LT 300) AND (S5.QVAL1 EQ 0) AND (S5.QVAL2 EQ 0) AND (S5.QVAL3 EQ 0) AND (S5.QVAL4 EQ 0) AND (S5.QVAL5 EQ 0) AND (S5.QVAL6 EQ 0) AND (S5.QVAL7 EQ 0) AND (S5.QVAL8 EQ 0) AND (S5.QVAL9 EQ 0) AND (S5.QVAL_10 EQ 0) AND (S5.QVAL_11 EQ 0) AND (S5.QVAL_12 EQ 0) AND (S5.QVAL_13 EQ 0) AND (S5.QVAL_14 EQ 0) AND (S5.QVAL_15 EQ 0) AND (S5.QVAL_16 EQ 0) AND (S5.QVAL_17 EQ 0) AND (S5.QVAL_18 EQ 0) AND (S5.QVAL_19 EQ 0) AND (S5.QVAL_20 EQ 102.1)) OR ((S5.2PQVAL1 EQ 0) AND (S5.2PQVAL2 EQ 0) AND (S5.2PQVAL3 EQ 0) AND (S5.2PQVAL4 EQ 0) AND (S5.2PQVAL5 EQ 0) AND (S5.2PQVAL6 EQ 0) AND (S5.2PQVAL7 EQ 0) AND (S5.2PQVAL8 EQ 0) AND (S5.2PQVAL9 EQ 0) AND (S5.2PQVAL_10 EQ 0) AND (S5.2PQVAL_11 EQ 0) AND (S5.2PQVAL_12 EQ 0) AND (S5.2PQVAL_13 EQ 0) AND (S5.2PQVAL_14 EQ 0) AND (S5.2PQVAL_15 EQ 0) AND (S5.2PQVAL_16 EQ 0) AND (S5.2PQVAL_17 EQ 0) AND (S5.2PQVAL_18 EQ 0) AND (S5.2PQVAL_19 EQ 0) AND (S5.2PQVAL_20 EQ 120))) [S5.VAL_1 = 'être une personne qui sait se modérer'];
IF (((S5.SUMVAL GE 200) AND (S5.SUMVAL LT 300) AND (S5.QVAL1 EQ 0) AND (S5.QVAL2 EQ 0) AND (S5.QVAL3 EQ 0) AND (S5.QVAL4 EQ 0) AND (S5.QVAL5 EQ 0) AND (S5.QVAL6 EQ 0) AND (S5.QVAL7 EQ 0) AND (S5.QVAL8 EQ 0) AND (S5.QVAL9 EQ 0) AND (S5.QVAL_10 EQ 0) AND (S5.QVAL_11 EQ 0) AND (S5.QVAL_12 EQ 0) AND (S5.QVAL_13 EQ 0) AND (S5.QVAL_14 EQ 0) AND (S5.QVAL_15 EQ 0) AND (S5.QVAL_16 EQ 0) AND (S5.QVAL_17 EQ 0) AND (S5.QVAL_18 EQ 0) AND (S5.QVAL_19 EQ 0) AND

(S5.QVAL_20 EQ 0) AND (S5.QVAL_21 EQ 102.2)) OR ((S5.2PQVAL1 EQ 0) AND (S5.2PQVAL2 EQ 0) AND (S5.2PQVAL3 EQ 0) AND (S5.2PQVAL4 EQ 0) AND (S5.2PQVAL5 EQ 0) AND (S5.2PQVAL6 EQ 0) AND (S5.2PQVAL7 EQ 0) AND (S5.2PQVAL8 EQ 0) AND (S5.2PQVAL9 EQ 0) AND (S5.2PQVAL_10 EQ 0) AND (S5.2PQVAL_11 EQ 0) AND (S5.2PQVAL_12 EQ 0) AND (S5.2PQVAL_13 EQ 0) AND (S5.2PQVAL_14 EQ 0) AND (S5.2PQVAL_15 EQ 0) AND (S5.2PQVAL_16 EQ 0) AND (S5.2PQVAL_17 EQ 0) AND (S5.2PQVAL_18 EQ 0) AND (S5.2PQVAL_19 EQ 0) AND (S5.2PQVAL_20 EQ 0) AND (S5.2PQVAL_21 EQ 121))) [S5.VAL_1 = 'être une personne possédant beaucoup de moyens pour contrer des situations indésirables'];
IF (((S5.SUMVAL GE 200) AND (S5.SUMVAL LT 300) AND (S5.QVAL1 EQ 0) AND (S5.QVAL2 EQ 0) AND (S5.QVAL3 EQ 0) AND (S5.QVAL4 EQ 0) AND (S5.QVAL5 EQ 0) AND (S5.QVAL6 EQ 0) AND (S5.QVAL7 EQ 0) AND (S5.QVAL8 EQ 0) AND (S5.QVAL9 EQ 0) AND (S5.QVAL_10 EQ 0) AND (S5.QVAL_11 EQ 0) AND (S5.QVAL_12 EQ 0) AND (S5.QVAL_13 EQ 0) AND (S5.QVAL_14 EQ 0) AND (S5.QVAL_15 EQ 0) AND (S5.QVAL_16 EQ 0) AND (S5.QVAL_17 EQ 0) AND (S5.QVAL_18 EQ 0) AND (S5.QVAL_19 EQ 0) AND (S5.QVAL_20 EQ 0) AND (S5.QVAL_21 EQ 0) AND (S5.QVAL_22 EQ 102.3)) OR ((S5.2PQVAL1 EQ 0) AND (S5.2PQVAL2 EQ 0) AND (S5.2PQVAL3 EQ 0) AND (S5.2PQVAL4 EQ 0) AND (S5.2PQVAL5 EQ 0) AND (S5.2PQVAL6 EQ 0) AND (S5.2PQVAL7 EQ 0) AND (S5.2PQVAL8 EQ 0) AND (S5.2PQVAL9 EQ 0) AND (S5.2PQVAL_10 EQ 0) AND (S5.2PQVAL_11 EQ 0) AND (S5.2PQVAL_12 EQ 0) AND (S5.2PQVAL_13 EQ 0) AND (S5.2PQVAL_14 EQ 0) AND (S5.2PQVAL_15 EQ 0) AND (S5.2PQVAL_16 EQ 0) AND (S5.2PQVAL_17 EQ 0) AND (S5.2PQVAL_18 EQ 0) AND (S5.2PQVAL_19 EQ 0) AND (S5.2PQVAL_20 EQ 0) AND (S5.2PQVAL_22 EQ 122))) [S5.VAL_1 = 'être une personne qui réfléchit avant d´agir'];
IF (((S5.SUMVAL GE 200) AND (S5.SUMVAL LT 300) AND (S5.QVAL1 EQ 0) AND (S5.QVAL2 EQ 0) AND (S5.QVAL3 EQ 0) AND (S5.QVAL4 EQ 0) AND (S5.QVAL5 EQ 0) AND (S5.QVAL6 EQ 0) AND (S5.QVAL7 EQ 0) AND (S5.QVAL8 EQ 0) AND (S5.QVAL9 EQ 0) AND (S5.QVAL_10 EQ 0) AND (S5.QVAL_11 EQ 0) AND (S5.QVAL_12 EQ 0) AND (S5.QVAL_13 EQ 0) AND (S5.QVAL_14 EQ 0) AND (S5.QVAL_15 EQ 0) AND (S5.QVAL_16 EQ 0) AND (S5.QVAL_17 EQ 0) AND (S5.QVAL_18 EQ 0) AND (S5.QVAL_19 EQ 0) AND (S5.QVAL_20 EQ 0) AND (S5.QVAL_21 EQ 0) AND (S5.QVAL_22 EQ 0) AND (S5.QVAL_23 EQ 102.4)) OR ((S5.2PQVAL1 EQ 0) AND (S5.2PQVAL2 EQ 0) AND (S5.2PQVAL3 EQ 0) AND (S5.2PQVAL4 EQ 0) AND (S5.2PQVAL5 EQ 0) AND (S5.2PQVAL6 EQ 0) AND (S5.2PQVAL7 EQ 0) AND (S5.2PQVAL8 EQ 0) AND (S5.2PQVAL9 EQ 0) AND (S5.2PQVAL_10 EQ 0) AND (S5.2PQVAL_11 EQ 0) AND (S5.2PQVAL_12 EQ 0) AND (S5.2PQVAL_13 EQ 0) AND (S5.2PQVAL_14 EQ 0) AND (S5.2PQVAL_15 EQ 0) AND (S5.2PQVAL_16 EQ 0) AND (S5.2PQVAL_17 EQ 0) AND (S5.2PQVAL_18 EQ 0) AND (S5.2PQVAL_19 EQ 0) AND (S5.2PQVAL_20 EQ 0) AND (S5.2PQVAL_22 EQ 0) AND (S5.2PQVAL_23 EQ 123))) [S5.VAL_1 = 'être une personne qui fait ce qui doit être fait sans hésitation'];
IF (((S5.SUMVAL GE 200) AND (S5.SUMVAL LT 300) AND (S5.QVAL1 EQ 0) AND (S5.QVAL2 EQ 0) AND (S5.QVAL3 EQ 0) AND (S5.QVAL4 EQ 0) AND (S5.QVAL5 EQ 0) AND (S5.QVAL6 EQ 0) AND (S5.QVAL7 EQ 0) AND (S5.QVAL8 EQ 0) AND (S5.QVAL9 EQ 0) AND (S5.QVAL_10 EQ 0) AND (S5.QVAL_11 EQ 0) AND (S5.QVAL_12 EQ 0) AND (S5.QVAL_13 EQ 0) AND (S5.QVAL_14 EQ 0) AND (S5.QVAL_15 EQ 0) AND (S5.QVAL_16 EQ 0) AND (S5.QVAL_17 EQ 0) AND (S5.QVAL_18 EQ 0) AND (S5.QVAL_19 EQ 0) AND (S5.QVAL_20 EQ 0) AND (S5.QVAL_21 EQ 0) AND (S5.QVAL_22 EQ 0) AND (S5.QVAL_23 EQ 0) AND (S5.QVAL_24 EQ 102.5)) OR ((S5.2PQVAL1 EQ 0) AND (S5.2PQVAL2 EQ 0) AND (S5.2PQVAL3 EQ 0) AND (S5.2PQVAL4 EQ 0) AND (S5.2PQVAL5 EQ 0) AND (S5.2PQVAL6 EQ 0) AND (S5.2PQVAL7 EQ 0) AND (S5.2PQVAL8 EQ 0) AND (S5.2PQVAL9 EQ 0) AND (S5.2PQVAL_10 EQ 0) AND (S5.2PQVAL_11 EQ 0) AND (S5.2PQVAL_12 EQ 0) AND (S5.2PQVAL_13 EQ 0) AND (S5.2PQVAL_14 EQ 0) AND (S5.2PQVAL_15 EQ 0)

AND (S5.2PQVAL_16 EQ 0) AND (S5.2PQVAL_17 EQ 0) AND (S5.2PQVAL_18 EQ 0) AND (S5.2PQVAL_19 EQ 0) AND (S5.2PQVAL_20 EQ 0) AND (S5.2PQVAL_22 EQ 0) AND (S5.2PQVAL_23 EQ 0) AND (S5.2PQVAL_24 EQ 124))) [S5.VAL_1 = 'être une personne qui sait clairement vers où elle se dirige dans la vie'];
IF (((S5.SUMVAL GE 200) AND (S5.SUMVAL LT 300) AND (S5.QVAL1 EQ 0) AND (S5.QVAL2 EQ 0) AND (S5.QVAL3 EQ 0) AND (S5.QVAL4 EQ 0) AND (S5.QVAL5 EQ 0) AND (S5.QVAL6 EQ 0) AND (S5.QVAL7 EQ 0) AND (S5.QVAL8 EQ 0) AND (S5.QVAL9 EQ 0) AND (S5.QVAL_10 EQ 0) AND (S5.QVAL_11 EQ 0) AND (S5.QVAL_12 EQ 0) AND (S5.QVAL_13 EQ 0) AND (S5.QVAL_14 EQ 0) AND (S5.QVAL_15 EQ 0) AND (S5.QVAL_16 EQ 0) AND (S5.QVAL_17 EQ 0) AND (S5.QVAL_18 EQ 0) AND (S5.QVAL_19 EQ 0) AND (S5.QVAL_20 EQ 0) AND (S5.QVAL_21 EQ 0) AND (S5.QVAL_22 EQ 0) AND (S5.QVAL_23 EQ 0) AND (S5.QVAL_24 EQ 0) AND (S5.QVAL_25 EQ 102.6)) OR ((S5.2PQVAL1 EQ 0) AND (S5.2PQVAL2 EQ 0) AND (S5.2PQVAL3 EQ 0) AND (S5.2PQVAL4 EQ 0) AND (S5.2PQVAL5 EQ 0) AND (S5.2PQVAL6 EQ 0) AND (S5.2PQVAL7 EQ 0) AND (S5.2PQVAL8 EQ 0) AND (S5.2PQVAL9 EQ 0) AND (S5.2PQVAL_10 EQ 0) AND (S5.2PQVAL_11 EQ 0) AND (S5.2PQVAL_12 EQ 0) AND (S5.2PQVAL_13 EQ 0) AND (S5.2PQVAL_14 EQ 0) AND (S5.2PQVAL_15 EQ 0) AND (S5.2PQVAL_16 EQ 0) AND (S5.2PQVAL_17 EQ 0) AND (S5.2PQVAL_18 EQ 0) AND (S5.2PQVAL_19 EQ 0) AND (S5.2PQVAL_20 EQ 0) AND (S5.2PQVAL_22 EQ 0) AND (S5.2PQVAL_23 EQ 0) AND (S5.2PQVAL_24 EQ 0) AND (S5.2PQVAL_25 EQ 125))) [S5.VAL_1 = 'être une personne intègre, fidèle à vos principes'];
IF (((S5.SUMVAL GE 200) AND (S5.SUMVAL LT 300) AND (S5.QVAL1 EQ 101) AND (S5.QVAL2 EQ 102)) OR ((S5.2PQVAL1 EQ 101) AND (S5.2PQVAL2 EQ 102))) [S5.VAL_2 = 'être une personne prête à aider les autres'];
IF (((S5.SUMVAL GE 200) AND (S5.SUMVAL LT 300) AND ((S5.QVAL1 EQ 101) OR (S5.QVAL2 EQ 102)) AND (S5.QVAL3 EQ 103)) OR (((S5.2PQVAL1 EQ 101) OR (S5.2PQVAL2 EQ 102)) AND (S5.2PQVAL3 EQ 103))) [S5.VAL_2 = 'être une bonne oreille pour les autres'];
IF (((S5.SUMVAL GE 200) AND (S5.SUMVAL LT 300) AND ((S5.QVAL1 EQ 101) OR (S5.QVAL2 EQ 102) OR (S5.QVAL3 EQ 103)) AND (S5.QVAL4 EQ 104)) OR (((S5.2PQVAL1 EQ 101) OR (S5.2PQVAL2 EQ 102) OR (S5.2PQVAL3 EQ 103)) AND (S5.2PQVAL4 EQ 104))) [S5.VAL_2 = 'être une personne qui croyez en votre capacité d´accomplir toutes choses'];
IF (((S5.SUMVAL GE 200) AND (S5.SUMVAL LT 300) AND ((S5.QVAL1 EQ 101) OR (S5.QVAL2 EQ 102) OR (S5.QVAL3 EQ 103) OR (S5.QVAL4 EQ 104)) AND (S5.QVAL5 EQ 105)) OR (((S5.2PQVAL1 EQ 101) OR (S5.2PQVAL2 EQ 102) OR (S5.2PQVAL3 EQ 103) OR (S5.2PQVAL4 EQ 104)) AND (S5.2PQVAL5 EQ 105))) [S5.VAL_2 = 'être une personne douée dans plusieurs domaines'];
IF (((S5.SUMVAL GE 200) AND (S5.SUMVAL LT 300) AND ((S5.QVAL1 EQ 101) OR (S5.QVAL2 EQ 102) OR (S5.QVAL3 EQ 103) OR (S5.QVAL4 EQ 104) OR (S5.QVAL5 EQ 105)) AND (S5.QVAL6 EQ 106)) OR (((S5.2PQVAL1 EQ 101) OR (S5.2PQVAL2 EQ 102) OR (S5.2PQVAL3 EQ 103) OR (S5.2PQVAL4 EQ 104) OR (S5.2PQVAL5 EQ 105)) AND (S5.2PQVAL6 EQ 106))) [S5.VAL_2 = 'être une personne qui possède une forte confiance en vous'];
IF (((S5.SUMVAL GE 200) AND (S5.SUMVAL LT 300) AND ((S5.QVAL1 EQ 101) OR (S5.QVAL2 EQ 102) OR (S5.QVAL3 EQ 103) OR (S5.QVAL4 EQ 104) OR (S5.QVAL5 EQ 105) OR (S5.QVAL6 EQ 106)) AND (S5.QVAL7 EQ 107)) OR (((S5.2PQVAL1 EQ 101) OR (S5.2PQVAL2 EQ 102) OR (S5.2PQVAL3 EQ 103) OR (S5.2PQVAL4 EQ 104) OR (S5.2PQVAL5 EQ 105) OR (S5.2PQVAL6 EQ 106)) AND (S5.2PQVAL7 EQ 107))) [S5.VAL_2 = 'être une personne généralement décidée'];

IF (((S5.SUMVAL GE 200) AND (S5.SUMVAL LT 300) AND ((S5.QVAL1 EQ 101) OR (S5.QVAL2 EQ 102) OR (S5.QVAL3 EQ 103) OR (S5.QVAL4 EQ 104) OR (S5.QVAL5 EQ 105) OR (S5.QVAL6 EQ 106) OR (S5.QVAL7 EQ 107)) AND (S5.QVAL8 EQ 108)) OR (((S5.2PQVAL1 EQ 101) OR (S5.2PQVAL2 EQ 102) OR (S5.2PQVAL3 EQ 103) OR (S5.2PQVAL4 EQ 104) OR (S5.2PQVAL5 EQ 105) OR (S5.2PQVAL6 EQ 106) OR (S5.2PQVAL7 EQ 107)) AND (S5.2PQVAL8 EQ 108))) [S5.VAL_2 = 'être une personne qui vient à bout de ce qu´elle entreprend'];
IF (((S5.SUMVAL GE 200) AND (S5.SUMVAL LT 300) AND ((S5.QVAL1 EQ 101) OR (S5.QVAL2 EQ 102) OR (S5.QVAL3 EQ 103) OR (S5.QVAL4 EQ 104) OR (S5.QVAL5 EQ 105) OR (S5.QVAL6 EQ 106) OR (S5.QVAL7 EQ 107) OR (S5.QVAL8 EQ 108)) AND (S5.QVAL9 EQ 109)) OR (((S5.2PQVAL1 EQ 101) OR (S5.2PQVAL2 EQ 102) OR (S5.2PQVAL3 EQ 103) OR (S5.2PQVAL4 EQ 104) OR (S5.2PQVAL5 EQ 105) OR (S5.2PQVAL6 EQ 106) OR (S5.2PQVAL7 EQ 107) OR (S5.2PQVAL8 EQ 108)) AND (S5.2PQVAL9 EQ 109))) [S5.VAL_2 = 'être une personne qui donne tout ce qu´elle a pour réussir'];
IF (((S5.SUMVAL GE 200) AND (S5.SUMVAL LT 300) AND ((S5.QVAL1 EQ 101) OR (S5.QVAL2 EQ 102) OR (S5.QVAL3 EQ 103) OR (S5.QVAL4 EQ 104) OR (S5.QVAL5 EQ 105) OR (S5.QVAL6 EQ 106) OR (S5.QVAL7 EQ 107) OR (S5.QVAL8 EQ 108) OR (S5.QVAL9 EQ 109)) AND (S5.QVAL_10 EQ 101.1)) OR (((S5.2PQVAL1 EQ 101) OR (S5.2PQVAL2 EQ 102) OR (S5.2PQVAL3 EQ 103) OR (S5.2PQVAL4 EQ 104) OR (S5.2PQVAL5 EQ 105) OR (S5.2PQVAL6 EQ 106) OR (S5.2PQVAL7 EQ 107) OR (S5.2PQVAL8 EQ 108) OR (S5.2PQVAL9 EQ 109)) AND (S5.2PQVAL_10 EQ 110))) [S5.VAL_2 = 'être une personne assidue qui accompli toute chose avec succès'];
IF (((S5.SUMVAL GE 200) AND (S5.SUMVAL LT 300) AND ((S5.QVAL1 EQ 101) OR (S5.QVAL2 EQ 102) OR (S5.QVAL3 EQ 103) OR (S5.QVAL4 EQ 104) OR (S5.QVAL5 EQ 105) OR (S5.QVAL6 EQ 106) OR (S5.QVAL7 EQ 107) OR (S5.QVAL8 EQ 108) OR (S5.QVAL9 EQ 109) OR (S5.QVAL_10 EQ 101.1)) AND (S5.QVAL_11 EQ 101.2)) OR (((S5.2PQVAL1 EQ 101) OR (S5.2PQVAL2 EQ 102) OR (S5.2PQVAL3 EQ 103) OR (S5.2PQVAL4 EQ 104) OR (S5.2PQVAL5 EQ 105) OR (S5.2PQVAL6 EQ 106) OR (S5.2PQVAL7 EQ 107) OR (S5.2PQVAL8 EQ 108) OR (S5.2PQVAL9 EQ 109) OR (S5.2PQVAL_10 EQ 110)) AND (S5.2PQVAL_11 EQ 111))) [S5.VAL_2 = 'être une personne vivante qui a beaucoup d´énergie à donner'];
IF (((S5.SUMVAL GE 200) AND (S5.SUMVAL LT 300) AND ((S5.QVAL1 EQ 101) OR (S5.QVAL2 EQ 102) OR (S5.QVAL3 EQ 103) OR (S5.QVAL4 EQ 104) OR (S5.QVAL5 EQ 105) OR (S5.QVAL6 EQ 106) OR (S5.QVAL7 EQ 107) OR (S5.QVAL8 EQ 108) OR (S5.QVAL9 EQ 109) OR (S5.QVAL_10 EQ 101.1) OR (S5.QVAL_11 EQ 101.2)) AND (S5.QVAL_12 EQ 101.3)) OR (((S5.2PQVAL1 EQ 101) OR (S5.2PQVAL2 EQ 102) OR (S5.2PQVAL3 EQ 103) OR (S5.2PQVAL4 EQ 104) OR (S5.2PQVAL5 EQ 105) OR (S5.2PQVAL6 EQ 106) OR (S5.2PQVAL7 EQ 107) OR (S5.2PQVAL8 EQ 108) OR (S5.2PQVAL9 EQ 109) OR (S5.2PQVAL_10 EQ 110) OR (S5.2PQVAL_11 EQ 111)) AND (S5.2PQVAL_12 EQ 112))) [S5.VAL_2 = 'être une personne avec une bonne humeur contagieuse'];
IF (((S5.SUMVAL GE 200) AND (S5.SUMVAL LT 300) AND ((S5.QVAL1 EQ 101) OR (S5.QVAL2 EQ 102) OR (S5.QVAL3 EQ 103) OR (S5.QVAL4 EQ 104) OR (S5.QVAL5 EQ 105) OR (S5.QVAL6 EQ 106) OR (S5.QVAL7 EQ 107) OR (S5.QVAL8 EQ 108) OR (S5.QVAL9 EQ 109) OR (S5.QVAL_10 EQ 101.1) OR (S5.QVAL_11 EQ 101.2) OR (S5.QVAL_12 EQ 101.3)) AND (S5.QVAL_13 EQ 101.4)) OR (((S5.2PQVAL1 EQ 101) OR (S5.2PQVAL2 EQ 102) OR (S5.2PQVAL3 EQ 103) OR (S5.2PQVAL4 EQ 104) OR (S5.2PQVAL5 EQ 105) OR (S5.2PQVAL6 EQ 106) OR (S5.2PQVAL7 EQ 107) OR (S5.2PQVAL8 EQ 108) OR (S5.2PQVAL9 EQ 109) OR (S5.2PQVAL_10 EQ 110) OR (S5.2PQVAL_11 EQ 111) OR (S5.2PQVAL_12 EQ 112)) AND (S5.2PQVAL_13 EQ 113))) [S5.VAL_2 = 'être une personne qui finit toujours par réussir'];
IF (((S5.SUMVAL GE 200) AND (S5.SUMVAL LT 300) AND ((S5.QVAL1 EQ 101) OR (S5.QVAL2 EQ 102) OR (S5.QVAL3 EQ 103) OR (S5.QVAL4 EQ 104) OR (S5.QVAL5 EQ 105) OR (S5.QVAL6 EQ 106) OR (S5.QVAL7 EQ 107) OR (S5.QVAL8 EQ 108) OR (S5.QVAL9 EQ 109) OR (S5.QVAL_10 EQ 101.1) OR (S5.QVAL_11 EQ 101.2)

OR (S5.QVAL_12 EQ 101.3) OR (S5.QVAL_13 EQ 101.4)) AND (S5.QVAL_14 EQ 101.5)) OR (((S5.2PQVAL1 EQ 101) OR (S5.2PQVAL2 EQ 102) OR (S5.2PQVAL3 EQ 103) OR (S5.2PQVAL4 EQ 104) OR (S5.2PQVAL5 EQ 105) OR (S5.2PQVAL6 EQ 106) OR (S5.2PQVAL7 EQ 107) OR (S5.2PQVAL8 EQ 108) OR (S5.2PQVAL9 EQ 109) OR (S5.2PQVAL_10 EQ 110) OR (S5.2PQVAL_11 EQ 111) OR (S5.2PQVAL_12 EQ 112) OR (S5.2PQVAL_13 EQ 113)) AND (S5.2PQVAL_14 EQ 114))) [S5.VAL_2 = 'être une personne qui prend des décisions responsables'];
IF (((S5.SUMVAL GE 200) AND (S5.SUMVAL LT 300) AND ((S5.QVAL1 EQ 101) OR (S5.QVAL2 EQ 102) OR (S5.QVAL3 EQ 103) OR (S5.QVAL4 EQ 104) OR (S5.QVAL5 EQ 105) OR (S5.QVAL6 EQ 106) OR (S5.QVAL7 EQ 107) OR (S5.QVAL8 EQ 108) OR (S5.QVAL9 EQ 109) OR (S5.QVAL_10 EQ 101.1) OR (S5.QVAL_11 EQ 101.2) OR (S5.QVAL_12 EQ 101.3) OR (S5.QVAL_13 EQ 101.4) OR (S5.QVAL_14 EQ 101.5)) AND (S5.QVAL_15 EQ 101.6)) OR (((S5.2PQVAL1 EQ 101) OR (S5.2PQVAL2 EQ 102) OR (S5.2PQVAL3 EQ 103) OR (S5.2PQVAL4 EQ 104) OR (S5.2PQVAL5 EQ 105) OR (S5.2PQVAL6 EQ 106) OR (S5.2PQVAL7 EQ 107) OR (S5.2PQVAL8 EQ 108) OR (S5.2PQVAL9 EQ 109) OR (S5.2PQVAL_10 EQ 110) OR (S5.2PQVAL_11 EQ 111) OR (S5.2PQVAL_12 EQ 112) OR (S5.2PQVAL_13 EQ 113) OR (S5.2PQVAL_14 EQ 114)) AND (S5.2PQVAL_15 EQ 115))) [S5.VAL_2 = 'être une personne qui planifie efficacement ses activités'];
IF (((S5.SUMVAL GE 200) AND (S5.SUMVAL LT 300) AND ((S5.QVAL1 EQ 101) OR (S5.QVAL2 EQ 102) OR (S5.QVAL3 EQ 103) OR (S5.QVAL4 EQ 104) OR (S5.QVAL5 EQ 105) OR (S5.QVAL6 EQ 106) OR (S5.QVAL7 EQ 107) OR (S5.QVAL8 EQ 108) OR (S5.QVAL9 EQ 109) OR (S5.QVAL_10 EQ 101.1) OR (S5.QVAL_11 EQ 101.2) OR (S5.QVAL_12 EQ 101.3) OR (S5.QVAL_13 EQ 101.4) OR (S5.QVAL_14 EQ 101.5) OR (S5.QVAL_15 EQ 101.6)) AND (S5.QVAL_16 EQ 101.7)) OR (((S5.2PQVAL1 EQ 101) OR (S5.2PQVAL2 EQ 102) OR (S5.2PQVAL3 EQ 103) OR (S5.2PQVAL4 EQ 104) OR (S5.2PQVAL5 EQ 105) OR (S5.2PQVAL6 EQ 106) OR (S5.2PQVAL7 EQ 107) OR (S5.2PQVAL8 EQ 108) OR (S5.2PQVAL9 EQ 109) OR (S5.2PQVAL_10 EQ 110) OR (S5.2PQVAL_11 EQ 111) OR (S5.2PQVAL_12 EQ 112) OR (S5.2PQVAL_13 EQ 113) OR (S5.2PQVAL_14 EQ 114) OR (S5.2PQVAL_15 EQ 115)) AND (S5.2PQVAL_16 EQ 116))) [S5.VAL_2 = 'être une personne qui n´hésite pas à vivre de nouvelles expériences'];
IF (((S5.SUMVAL GE 200) AND (S5.SUMVAL LT 300) AND ((S5.QVAL1 EQ 101) OR (S5.QVAL2 EQ 102) OR (S5.QVAL3 EQ 103) OR (S5.QVAL4 EQ 104) OR (S5.QVAL5 EQ 105) OR (S5.QVAL6 EQ 106) OR (S5.QVAL7 EQ 107) OR (S5.QVAL8 EQ 108) OR (S5.QVAL9 EQ 109) OR (S5.QVAL_10 EQ 101.1) OR (S5.QVAL_11 EQ 101.2) OR (S5.QVAL_12 EQ 101.3) OR (S5.QVAL_13 EQ 101.4) OR (S5.QVAL_14 EQ 101.5) OR (S5.QVAL_15 EQ 101.6) OR (S5.QVAL_16 EQ 101.7)) AND (S5.QVAL_17 EQ 101.8)) OR (((S5.2PQVAL1 EQ 101) OR (S5.2PQVAL2 EQ 102) OR (S5.2PQVAL3 EQ 103) OR (S5.2PQVAL4 EQ 104) OR (S5.2PQVAL5 EQ 105) OR (S5.2PQVAL6 EQ 106) OR (S5.2PQVAL7 EQ 107) OR (S5.2PQVAL8 EQ 108) OR (S5.2PQVAL9 EQ 109) OR (S5.2PQVAL_10 EQ 110) OR (S5.2PQVAL_11 EQ 111) OR (S5.2PQVAL_12 EQ 112) OR (S5.2PQVAL_13 EQ 113) OR (S5.2PQVAL_14 EQ 114) OR (S5.2PQVAL_15 EQ 115) OR (S5.2PQVAL_16 EQ 116)) AND (S5.2PQVAL_17 EQ 117))) [S5.VAL_2 = 'être une personne qui fonce malgré les difficultés'];
IF (((S5.SUMVAL GE 200) AND (S5.SUMVAL LT 300) AND ((S5.QVAL1 EQ 101) OR (S5.QVAL2 EQ 102) OR (S5.QVAL3 EQ 103) OR (S5.QVAL4 EQ 104) OR (S5.QVAL5 EQ 105) OR (S5.QVAL6 EQ 106) OR (S5.QVAL7 EQ 107) OR (S5.QVAL8 EQ 108) OR (S5.QVAL9 EQ 109) OR (S5.QVAL_10 EQ 101.1) OR (S5.QVAL_11 EQ 101.2) OR (S5.QVAL_12 EQ 101.3) OR (S5.QVAL_13 EQ 101.4) OR (S5.QVAL_14 EQ 101.5) OR (S5.QVAL_15 EQ 101.6) OR (S5.QVAL_16 EQ 101.7) OR (S5.QVAL_17 EQ 101.8)) AND (S5.QVAL_18 EQ 101.9)) OR (((S5.2PQVAL1 EQ 101) OR (S5.2PQVAL2 EQ 102) OR (S5.2PQVAL3 EQ 103) OR (S5.2PQVAL4 EQ 104) OR (S5.2PQVAL5 EQ 105) OR (S5.2PQVAL6 EQ 106) OR (S5.2PQVAL7 EQ 107) OR (S5.2PQVAL8 EQ 108) OR

(S5.2PQVAL9 EQ 109) OR (S5.2PQVAL_10 EQ 110) OR (S5.2PQVAL_11 EQ 111) OR (S5.2PQVAL_12 EQ 112) OR (S5.2PQVAL_13 EQ 113) OR (S5.2PQVAL_14 EQ 114) OR (S5.2PQVAL_15 EQ 115) OR (S5.2PQVAL_16 EQ 116) OR (S5.2PQVAL_17 EQ 117)) AND (S5.2PQVAL_18 EQ 118))) [S5.VAL_2 = 'être une personne qui agit en pensant à long terme'];
IF (((S5.SUMVAL GE 200) AND (S5.SUMVAL LT 300) AND ((S5.QVAL1 EQ 101) OR (S5.QVAL2 EQ 102) OR (S5.QVAL3 EQ 103) OR (S5.QVAL4 EQ 104) OR (S5.QVAL5 EQ 105) OR (S5.QVAL6 EQ 106) OR (S5.QVAL7 EQ 107) OR (S5.QVAL8 EQ 108) OR (S5.QVAL9 EQ 109) OR (S5.QVAL_10 EQ 101.1) OR (S5.QVAL_11 EQ 101.2) OR (S5.QVAL_12 EQ 101.3) OR (S5.QVAL_13 EQ 101.4) OR (S5.QVAL_14 EQ 101.5) OR (S5.QVAL_15 EQ 101.6) OR (S5.QVAL_16 EQ 101.7) OR (S5.QVAL_17 EQ 101.8) OR (S5.QVAL_18 EQ 101.9)) AND (S5.QVAL_19 EQ 101.19))OR (((S5.2PQVAL1 EQ 101) OR (S5.2PQVAL2 EQ 102) OR (S5.2PQVAL3 EQ 103) OR (S5.2PQVAL4 EQ 104) OR (S5.2PQVAL5 EQ 105) OR (S5.2PQVAL6 EQ 106) OR (S5.2PQVAL7 EQ 107) OR (S5.2PQVAL8 EQ 108) OR (S5.2PQVAL9 EQ 109) OR (S5.2PQVAL_10 EQ 110) OR (S5.2PQVAL_11 EQ 111) OR (S5.2PQVAL_12 EQ 112) OR (S5.2PQVAL_13 EQ 113) OR (S5.2PQVAL_14 EQ 114) OR (S5.2PQVAL_15 EQ 115) OR (S5.2PQVAL_16 EQ 116) OR (S5.2PQVAL_17 EQ 117) OR (S5.2PQVAL_18 EQ 118)) AND (S5.2PQVAL_19 EQ 119))) [S5.VAL_2 = 'être une personne qui apprécie sa situation présente'];
IF (((S5.SUMVAL GE 200) AND (S5.SUMVAL LT 300) AND ((S5.QVAL1 EQ 101) OR (S5.QVAL2 EQ 102) OR (S5.QVAL3 EQ 103) OR (S5.QVAL4 EQ 104) OR (S5.QVAL5 EQ 105) OR (S5.QVAL6 EQ 106) OR (S5.QVAL7 EQ 107) OR (S5.QVAL8 EQ 108) OR (S5.QVAL9 EQ 109) OR (S5.QVAL_10 EQ 101.1) OR (S5.QVAL_11 EQ 101.2) OR (S5.QVAL_12 EQ 101.3) OR (S5.QVAL_13 EQ 101.4) OR (S5.QVAL_14 EQ 101.5) OR (S5.QVAL_15 EQ 101.6) OR (S5.QVAL_16 EQ 101.7) OR (S5.QVAL_17 EQ 101.8) OR (S5.QVAL_18 EQ 101.9) OR (S5.QVAL_19 EQ 101.19)) AND (S5.QVAL_20 EQ 102.1)) OR (((S5.2PQVAL1 EQ 101) OR (S5.2PQVAL2 EQ 102) OR (S5.2PQVAL3 EQ 103) OR (S5.2PQVAL4 EQ 104) OR (S5.2PQVAL5 EQ 105) OR (S5.2PQVAL6 EQ 106) OR (S5.2PQVAL7 EQ 107) OR (S5.2PQVAL8 EQ 108) OR (S5.2PQVAL9 EQ 109) OR (S5.2PQVAL_10 EQ 110) OR (S5.2PQVAL_11 EQ 111) OR (S5.2PQVAL_12 EQ 112) OR (S5.2PQVAL_13 EQ 113) OR (S5.2PQVAL_14 EQ 114) OR (S5.2PQVAL_15 EQ 115) OR (S5.2PQVAL_16 EQ 116) OR (S5.2PQVAL_17 EQ 117) OR (S5.2PQVAL_18 EQ 118) OR (S5.2PQVAL_19 EQ 119)) AND (S5.2PQVAL_20 EQ 120))) [S5.VAL_2 = 'être une personne qui sait se modérer'];
IF (((S5.SUMVAL GE 200) AND (S5.SUMVAL LT 300) AND ((S5.QVAL1 EQ 101) OR (S5.QVAL2 EQ 102) OR (S5.QVAL3 EQ 103) OR (S5.QVAL4 EQ 104) OR (S5.QVAL5 EQ 105) OR (S5.QVAL6 EQ 106) OR (S5.QVAL7 EQ 107) OR (S5.QVAL8 EQ 108) OR (S5.QVAL9 EQ 109) OR (S5.QVAL_10 EQ 101.1) OR (S5.QVAL_11 EQ 101.2) OR (S5.QVAL_12 EQ 101.3) OR (S5.QVAL_13 EQ 101.4) OR (S5.QVAL_14 EQ 101.5) OR (S5.QVAL_15 EQ 101.6) OR (S5.QVAL_16 EQ 101.7) OR (S5.QVAL_17 EQ 101.8) OR (S5.QVAL_18 EQ 101.9) OR (S5.QVAL_19 EQ 101.19) OR (S5.QVAL_20 EQ 102.1)) AND (S5.QVAL_21 EQ 102.2)) OR (((S5.2PQVAL1 EQ 101) OR (S5.2PQVAL2 EQ 102) OR (S5.2PQVAL3 EQ 103) OR (S5.2PQVAL4 EQ 104) OR (S5.2PQVAL5 EQ 105) OR (S5.2PQVAL6 EQ 106) OR (S5.2PQVAL7 EQ 107) OR (S5.2PQVAL8 EQ 108) OR (S5.2PQVAL9 EQ 109) OR (S5.2PQVAL_10 EQ 110) OR (S5.2PQVAL_11 EQ 111) OR (S5.2PQVAL_12 EQ 112) OR (S5.2PQVAL_13 EQ 113) OR (S5.2PQVAL_14 EQ 114) OR (S5.2PQVAL_15 EQ 115) OR (S5.2PQVAL_16 EQ 116) OR (S5.2PQVAL_17 EQ 117) OR (S5.2PQVAL_18 EQ 118) OR (S5.2PQVAL_19 EQ 119) OR (S5.2PQVAL_20 EQ 120)) AND (S5.2PQVAL_21 EQ 121))) [S5.VAL_2 = 'être une personne possédant beaucoup de moyens pour contrer des situations indésirables'];
IF (((S5.SUMVAL GE 200) AND (S5.SUMVAL LT 300) AND ((S5.QVAL1 EQ 101) OR (S5.QVAL2 EQ 102) OR (S5.QVAL3 EQ 103) OR (S5.QVAL4 EQ 104) OR (S5.QVAL5 EQ 105) OR (S5.QVAL6 EQ 106) OR (S5.QVAL7 EQ

107) OR (S5.QVAL8 EQ 108) OR (S5.QVAL9 EQ 109) OR (S5.QVAL_10 EQ 101.1) OR (S5.QVAL_11 EQ 101.2) OR (S5.QVAL_12 EQ 101.3) OR (S5.QVAL_13 EQ 101.4) OR (S5.QVAL_14 EQ 101.5) OR (S5.QVAL_15 EQ 101.6) OR (S5.QVAL_16 EQ 101.7) OR (S5.QVAL_17 EQ 101.8) OR (S5.QVAL_18 EQ 101.9) OR (S5.QVAL_19 EQ 101.19) OR (S5.QVAL_20 EQ 102.1) OR (S5.QVAL_21 EQ 102.2)) AND (S5.QVAL_22 EQ 102.3)) OR (((S5.2PQVAL1 EQ 101) OR (S5.2PQVAL2 EQ 102) OR (S5.2PQVAL3 EQ 103) OR (S5.2PQVAL4 EQ 104) OR (S5.2PQVAL5 EQ 105) OR (S5.2PQVAL6 EQ 106) OR (S5.2PQVAL7 EQ 107) OR (S5.2PQVAL8 EQ 108) OR (S5.2PQVAL9 EQ 109) OR (S5.2PQVAL_10 EQ 110) OR (S5.2PQVAL_11 EQ 111) OR (S5.2PQVAL_12 EQ 112) OR (S5.2PQVAL_13 EQ 113) OR (S5.2PQVAL_14 EQ 114) OR (S5.2PQVAL_15 EQ 115) OR (S5.2PQVAL_16 EQ 116) OR (S5.2PQVAL_17 EQ 117) OR (S5.2PQVAL_18 EQ 118) OR (S5.2PQVAL_19 EQ 119) OR (S5.2PQVAL_20 EQ 120) OR (S5.2PQVAL_21 EQ 121)) AND (S5.2PQVAL_22 EQ 122))) [S5.VAL_2 = 'être une personne qui réfléchit avant d´agir'];
IF (((S5.SUMVAL GE 200) AND (S5.SUMVAL LT 300) AND ((S5.QVAL1 EQ 101) OR (S5.QVAL2 EQ 102) OR (S5.QVAL3 EQ 103) OR (S5.QVAL4 EQ 104) OR (S5.QVAL5 EQ 105) OR (S5.QVAL6 EQ 106) OR (S5.QVAL7 EQ 107) OR (S5.QVAL8 EQ 108) OR (S5.QVAL9 EQ 109) OR (S5.QVAL_10 EQ 101.1) OR (S5.QVAL_11 EQ 101.2) OR (S5.QVAL_12 EQ 101.3) OR (S5.QVAL_13 EQ 101.4) OR (S5.QVAL_14 EQ 101.5) OR (S5.QVAL_15 EQ 101.6) OR (S5.QVAL_16 EQ 101.7) OR (S5.QVAL_17 EQ 101.8) OR (S5.QVAL_18 EQ 101.9) OR (S5.QVAL_19 EQ 101.19) OR (S5.QVAL_20 EQ 102.1) OR (S5.QVAL_21 EQ 102.2) OR (S5.QVAL_22 EQ 102.3)) AND (S5.QVAL_23 EQ 102.4)) OR (((S5.2PQVAL1 EQ 101) OR (S5.2PQVAL2 EQ 102) OR (S5.2PQVAL3 EQ 103) OR (S5.2PQVAL4 EQ 104) OR (S5.2PQVAL5 EQ 105) OR (S5.2PQVAL6 EQ 106) OR (S5.2PQVAL7 EQ 107) OR (S5.2PQVAL8 EQ 108) OR (S5.2PQVAL9 EQ 109) OR (S5.2PQVAL_10 EQ 110) OR (S5.2PQVAL_11 EQ 111) OR (S5.2PQVAL_12 EQ 112) OR (S5.2PQVAL_13 EQ 113) OR (S5.2PQVAL_14 EQ 114) OR (S5.2PQVAL_15 EQ 115) OR (S5.2PQVAL_16 EQ 116) OR (S5.2PQVAL_17 EQ 117) OR (S5.2PQVAL_18 EQ 118) OR (S5.2PQVAL_19 EQ 119) OR (S5.2PQVAL_20 EQ 120) OR (S5.2PQVAL_21 EQ 121) OR (S5.2PQVAL_22 EQ 122)) AND (S5.2PQVAL_23 EQ 123))) [S5.VAL_2 = 'être une personne qui fait ce qui doit être fait sans hésitation'];
IF (((S5.SUMVAL GE 200) AND (S5.SUMVAL LT 300) AND ((S5.QVAL1 EQ 101) OR (S5.QVAL2 EQ 102) OR (S5.QVAL3 EQ 103) OR (S5.QVAL4 EQ 104) OR (S5.QVAL5 EQ 105) OR (S5.QVAL6 EQ 106) OR (S5.QVAL7 EQ 107) OR (S5.QVAL8 EQ 108) OR (S5.QVAL9 EQ 109) OR (S5.QVAL_10 EQ 101.1) OR (S5.QVAL_11 EQ 101.2) OR (S5.QVAL_12 EQ 101.3) OR (S5.QVAL_13 EQ 101.4) OR (S5.QVAL_14 EQ 101.5) OR (S5.QVAL_15 EQ 101.6) OR (S5.QVAL_16 EQ 101.7) OR (S5.QVAL_17 EQ 101.8) OR (S5.QVAL_18 EQ 101.9) OR (S5.QVAL_19 EQ 101.19) OR (S5.QVAL_20 EQ 102.1) OR (S5.QVAL_21 EQ 102.2) OR (S5.QVAL_22 EQ 102.3) OR (S5.QVAL_23 EQ 102.4)) AND (S5.QVAL_24 EQ 102.5)) OR (((S5.2PQVAL1 EQ 101) OR (S5.2PQVAL2 EQ 102) OR (S5.2PQVAL3 EQ 103) OR (S5.2PQVAL4 EQ 104) OR (S5.2PQVAL5 EQ 105) OR (S5.2PQVAL6 EQ 106) OR (S5.2PQVAL7 EQ 107) OR (S5.2PQVAL8 EQ 108) OR (S5.2PQVAL9 EQ 109) OR (S5.2PQVAL_10 EQ 110) OR (S5.2PQVAL_11 EQ 111) OR (S5.2PQVAL_12 EQ 112) OR (S5.2PQVAL_13 EQ 113) OR (S5.2PQVAL_14 EQ 114) OR (S5.2PQVAL_15 EQ 115) OR (S5.2PQVAL_16 EQ 116) OR (S5.2PQVAL_17 EQ 117) OR (S5.2PQVAL_18 EQ 118) OR (S5.2PQVAL_19 EQ 119) OR (S5.2PQVAL_20 EQ 120) OR (S5.2PQVAL_21 EQ 121) OR (S5.2PQVAL_22 EQ 122) OR (S5.2PQVAL_23 EQ 123)) AND (S5.2PQVAL_24 EQ 124))) [S5.VAL_2 = 'être une personne qui sait clairement vers où elle se dirige dans la vie'];
IF (((S5.SUMVAL GE 200) AND (S5.SUMVAL LT 300) AND ((S5.QVAL1 EQ 101) OR (S5.QVAL2 EQ 102) OR (S5.QVAL3 EQ 103) OR (S5.QVAL4 EQ 104) OR (S5.QVAL5 EQ 105) OR (S5.QVAL6 EQ 106) OR (S5.QVAL7 EQ 107) OR (S5.QVAL8 EQ 108) OR (S5.QVAL9 EQ 109) OR (S5.QVAL_10 EQ 101.1) OR (S5.QVAL_11 EQ 101.2)

OR (S5.QVAL_12 EQ 101.3) OR (S5.QVAL_13 EQ 101.4) OR (S5.QVAL_14 EQ 101.5) OR (S5.QVAL_15 EQ 101.6) OR (S5.QVAL_16 EQ 101.7) OR (S5.QVAL_17 EQ 101.8) OR (S5.QVAL_18 EQ 101.9) OR (S5.QVAL_19 EQ 101.19) OR (S5.QVAL_20 EQ 102.1) OR (S5.QVAL_21 EQ 102.2) OR (S5.QVAL_22 EQ 102.3) OR (S5.QVAL_23 EQ 102.4) OR (S5.QVAL_24 EQ 102.5)) AND (S5.QVAL_25 EQ 102.6)) OR (((S5.2PQVAL1 EQ 101) OR (S5.2PQVAL2 EQ 102) OR (S5.2PQVAL3 EQ 103) OR (S5.2PQVAL4 EQ 104) OR (S5.2PQVAL5 EQ 105) OR (S5.2PQVAL6 EQ 106) OR (S5.2PQVAL7 EQ 107) OR (S5.2PQVAL8 EQ 108) OR (S5.2PQVAL9 EQ 109) OR (S5.2PQVAL_10 EQ 110) OR (S5.2PQVAL_11 EQ 111) OR (S5.2PQVAL_12 EQ 112) OR (S5.2PQVAL_13 EQ 113) OR (S5.2PQVAL_14 EQ 114) OR (S5.2PQVAL_15 EQ 115) OR (S5.2PQVAL_16 EQ 116) OR (S5.2PQVAL_17 EQ 117) OR (S5.2PQVAL_18 EQ 118) OR (S5.2PQVAL_19 EQ 119) OR (S5.2PQVAL_20 EQ 120) OR (S5.2PQVAL_21 EQ 121) OR (S5.2PQVAL_22 EQ 122) OR (S5.2PQVAL_23 EQ 123) OR (S5.2PQVAL_24 EQ 124)) AND (S5.2PQVAL_25 EQ 125))) [S5.VAL_2 = 'être une personne intègre, fidèle à vos principes'];
IF (((S5.SUMVAL GE 200) AND (S5.SUMVAL LT 300) AND (S5.QVAL1 EQ 101)) OR (S5.2PQVAL1 EQ 101)) [S5.MICROQ1 = 'Je veux donner de l´amour longtemps aux autres. Je dois rester en santé.'];
IF (((S5.SUMVAL GE 200) AND (S5.SUMVAL LT 300) AND (S5.QVAL1 EQ 0) AND (S5.QVAL2 EQ 102)) OR ((S5.2PQVAL1 EQ 0) AND (S5.2PQVAL2 EQ 102))) [S5.MICROQ1 = 'Je dois être à l´écoute de moi-même d´abord si je veux aider les autres. '];
IF (((S5.SUMVAL GE 200) AND (S5.SUMVAL LT 300) AND (S5.QVAL1 EQ 0) AND (S5.QVAL2 EQ 0) AND (S5.QVAL3 EQ 103)) OR ((S5.2PQVAL1 EQ 0) AND (S5.2PQVAL2 EQ 0) AND (S5.2PQVAL3 EQ 103))) [S5.MICROQ1 = 'Je dois être à l´écoute des autres, mais aussi de moi-même. Je dois m´écouter et faire ce qui est bon pour moi.'];
IF (((S5.SUMVAL GE 200) AND (S5.SUMVAL LT 300) AND (S5.QVAL1 EQ 0) AND (S5.QVAL2 EQ 0) AND (S5.QVAL3 EQ 0) AND (S5.QVAL4 EQ 104)) OR ((S5.2PQVAL1 EQ 0) AND (S5.2PQVAL2 EQ 0) AND (S5.2PQVAL3 EQ 0) AND (S5.2PQVAL4 EQ 104))) [S5.MICROQ1 = 'Je sais que je suis capable d´y arriver. J´ai réussi des choses aussi dures ou sinon plus que ça.'];
IF (((S5.SUMVAL GE 200) AND (S5.SUMVAL LT 300) AND (S5.QVAL1 EQ 0) AND (S5.QVAL2 EQ 0) AND (S5.QVAL3 EQ 0) AND (S5.QVAL4 EQ 0) AND (S5.QVAL5 EQ 105)) OR ((S5.2PQVAL1 EQ 0) AND (S5.2PQVAL2 EQ 0) AND (S5.2PQVAL3 EQ 0) AND (S5.2PQVAL4 EQ 0) AND (S5.2PQVAL5 EQ 105))) [S5.MICROQ1 = 'Je suis douée dans plusieurs choses. J´ignore pourquoi pratiquer des activités physiques devrait être différent.'];
IF (((S5.SUMVAL GE 200) AND (S5.SUMVAL LT 300) AND (S5.QVAL1 EQ 0) AND (S5.QVAL2 EQ 0) AND (S5.QVAL3 EQ 0) AND (S5.QVAL4 EQ 0) AND (S5.QVAL5 EQ 0) AND (S5.QVAL6 EQ 106)) OR ((S5.2PQVAL1 EQ 0) AND (S5.2PQVAL2 EQ 0) AND (S5.2PQVAL3 EQ 0) AND (S5.2PQVAL4 EQ 0) AND (S5.2PQVAL5 EQ 0) AND (S5.2PQVAL6 EQ 106))) [S5.MICROQ1 = 'J´ai confiance en moi. Je n´ai qu´à prendre une décision ferme que je vais bouger régulièrement et je vais y arriver.'];
IF (((S5.SUMVAL GE 200) AND (S5.SUMVAL LT 300) AND (S5.QVAL1 EQ 0) AND (S5.QVAL2 EQ 0) AND (S5.QVAL3 EQ 0) AND (S5.QVAL4 EQ 0) AND (S5.QVAL5 EQ 0) AND (S5.QVAL6 EQ 0) AND (S5.QVAL7 EQ 107)) OR ((S5.2PQVAL1 EQ 0) AND (S5.2PQVAL2 EQ 0) AND (S5.2PQVAL3 EQ 0) AND (S5.2PQVAL4 EQ 0) AND (S5.2PQVAL5 EQ 0) AND (S5.2PQVAL6 EQ 0) AND (S5.2PQVAL7 EQ 107))) [S5.MICROQ1 = 'Je reviens rarement en arrière lorsque je décide de faire un projet. Si je décide de devenir active régulièrement, je vais y arriver et maintenir l´habitude.'];
IF (((S5.SUMVAL GE 200) AND (S5.SUMVAL LT 300) AND (S5.QVAL1 EQ 0) AND (S5.QVAL2 EQ 0) AND (S5.QVAL3 EQ 0) AND (S5.QVAL4 EQ 0) AND (S5.QVAL5 EQ 0) AND (S5.QVAL6 EQ 0) AND (S5.QVAL7 EQ 0)

AND (S5.QVAL8 EQ 108)) OR ((S5.2PQVAL1 EQ 0) AND (S5.2PQVAL2 EQ 0) AND (S5.2PQVAL3 EQ 0) AND (S5.2PQVAL4 EQ 0) AND (S5.2PQVAL5 EQ 0) AND (S5.2PQVAL6 EQ 0) AND (S5.2PQVAL7 EQ 0) AND (S5.2PQVAL8 EQ 108))) [S5.MICROQ1 = 'Je vais toujours au bout de mes projets. Ce devrait être la même chose avec la pratique d´activités physiques.'];
IF (((S5.SUMVAL GE 200) AND (S5.SUMVAL LT 300) AND (S5.QVAL1 EQ 0) AND (S5.QVAL2 EQ 0) AND (S5.QVAL3 EQ 0) AND (S5.QVAL4 EQ 0) AND (S5.QVAL5 EQ 0) AND (S5.QVAL6 EQ 0) AND (S5.QVAL7 EQ 0) AND (S5.QVAL8 EQ 0) AND (S5.QVAL9 EQ 109)) OR ((S5.2PQVAL1 EQ 0) AND (S5.2PQVAL2 EQ 0) AND (S5.2PQVAL3 EQ 0) AND (S5.2PQVAL4 EQ 0) AND (S5.2PQVAL5 EQ 0) AND (S5.2PQVAL6 EQ 0) AND (S5.2PQVAL7 EQ 0) AND (S5.2PQVAL8 EQ 0) AND (S5.2PQVAL9 EQ 109))) [S5.MICROQ1 = 'Je fais tout ce qu´il faut pour réussir ce que j´entreprends. Je dois faire la même chose avec l´activité physique. Je vais réussir.'];
IF (((S5.SUMVAL GE 200) AND (S5.SUMVAL LT 300) AND (S5.QVAL1 EQ 0) AND (S5.QVAL2 EQ 0) AND (S5.QVAL3 EQ 0) AND (S5.QVAL4 EQ 0) AND (S5.QVAL5 EQ 0) AND (S5.QVAL6 EQ 0) AND (S5.QVAL7 EQ 0) AND (S5.QVAL8 EQ 0) AND (S5.QVAL9 EQ 0) AND (S5.QVAL_10 EQ 101.1)) OR ((S5.2PQVAL1 EQ 0) AND (S5.2PQVAL2 EQ 0) AND (S5.2PQVAL3 EQ 0) AND (S5.2PQVAL4 EQ 0) AND (S5.2PQVAL5 EQ 0) AND (S5.2PQVAL6 EQ 0) AND (S5.2PQVAL7 EQ 0) AND (S5.2PQVAL8 EQ 0) AND (S5.2PQVAL9 EQ 0) AND (S5.2PQVAL_10 EQ 110))) [S5.MICROQ1 = 'Je pourrais organiser mon horaire pour intégrer des activités physiques. Je suis certainement assez efficace pour faire ça.'];
IF (((S5.SUMVAL GE 200) AND (S5.SUMVAL LT 300) AND (S5.QVAL1 EQ 0) AND (S5.QVAL2 EQ 0) AND (S5.QVAL3 EQ 0) AND (S5.QVAL4 EQ 0) AND (S5.QVAL5 EQ 0) AND (S5.QVAL6 EQ 0) AND (S5.QVAL7 EQ 0) AND (S5.QVAL8 EQ 0) AND (S5.QVAL9 EQ 0) AND (S5.QVAL_10 EQ 0) AND (S5.QVAL_11 EQ 101.2)) OR ((S5.2PQVAL1 EQ 0) AND (S5.2PQVAL2 EQ 0) AND (S5.2PQVAL3 EQ 0) AND (S5.2PQVAL4 EQ 0) AND (S5.2PQVAL5 EQ 0) AND (S5.2PQVAL6 EQ 0) AND (S5.2PQVAL7 EQ 0) AND (S5.2PQVAL8 EQ 0) AND (S5.2PQVAL9 EQ 0) AND (S5.2PQVAL_10 EQ 0) AND (S5.2PQVAL_11 EQ 111))) [S5.MICROQ1 = 'Je pourrais dépenser une partie de mon énergie dans ma santé. C´est sûr que j´ai assez d´énergie pour pratiquer des activités physiques. Je suis capable.'];
IF (((S5.SUMVAL GE 200) AND (S5.SUMVAL LT 300) AND (S5.QVAL1 EQ 0) AND (S5.QVAL2 EQ 0) AND (S5.QVAL3 EQ 0) AND (S5.QVAL4 EQ 0) AND (S5.QVAL5 EQ 0) AND (S5.QVAL6 EQ 0) AND (S5.QVAL7 EQ 0) AND (S5.QVAL8 EQ 0) AND (S5.QVAL9 EQ 0) AND (S5.QVAL_10 EQ 0) AND (S5.QVAL_11 EQ 0) AND (S5.QVAL_12 EQ 101.3)) OR ((S5.2PQVAL1 EQ 0) AND (S5.2PQVAL2 EQ 0) AND (S5.2PQVAL3 EQ 0) AND (S5.2PQVAL4 EQ 0) AND (S5.2PQVAL5 EQ 0) AND (S5.2PQVAL6 EQ 0) AND (S5.2PQVAL7 EQ 0) AND (S5.2PQVAL8 EQ 0) AND (S5.2PQVAL9 EQ 0) AND (S5.2PQVAL_10 EQ 0) AND (S5.2PQVAL_11 EQ 0) AND (S5.2PQVAL_12 EQ 112))) [S5.MICROQ1 = 'Je peux maintenir mon humeur positive lorsqu´il est temps de pratiquer des activités physiques. L´activité physique c´est bon et ça me ferait plaisir d´en faire. '];
IF (((S5.SUMVAL GE 200) AND (S5.SUMVAL LT 300) AND (S5.QVAL1 EQ 0) AND (S5.QVAL2 EQ 0) AND (S5.QVAL3 EQ 0) AND (S5.QVAL4 EQ 0) AND (S5.QVAL5 EQ 0) AND (S5.QVAL6 EQ 0) AND (S5.QVAL7 EQ 0) AND (S5.QVAL8 EQ 0) AND (S5.QVAL9 EQ 0) AND (S5.QVAL_10 EQ 0) AND (S5.QVAL_11 EQ 0) AND (S5.QVAL_12 EQ 0) AND (S5.QVAL_13 EQ 101.4)) OR ((S5.2PQVAL1 EQ 0) AND (S5.2PQVAL2 EQ 0) AND (S5.2PQVAL3 EQ 0) AND (S5.2PQVAL4 EQ 0) AND (S5.2PQVAL5 EQ 0) AND (S5.2PQVAL6 EQ 0) AND (S5.2PQVAL7 EQ 0) AND (S5.2PQVAL8 EQ 0) AND (S5.2PQVAL9 EQ 0) AND (S5.2PQVAL_10 EQ 0) AND (S5.2PQVAL_11 EQ 0) AND (S5.2PQVAL_12 EQ 0) AND (S5.2PQVAL_13 EQ 113))) [S5.MICROQ1 = 'Je performe dans tout ce que j´entreprends. Bouger régulièrement n´est pas une exception, je suis capable. '];

IF (((S5.SUMVAL GE 200) AND (S5.SUMVAL LT 300) AND (S5.QVAL1 EQ 0) AND (S5.QVAL2 EQ 0) AND (S5.QVAL3 EQ 0) AND (S5.QVAL4 EQ 0) AND (S5.QVAL5 EQ 0) AND (S5.QVAL6 EQ 0) AND (S5.QVAL7 EQ 0) AND (S5.QVAL8 EQ 0) AND (S5.QVAL9 EQ 0) AND (S5.QVAL_10 EQ 0) AND (S5.QVAL_11 EQ 0) AND (S5.QVAL_12 EQ 0) AND (S5.QVAL_13 EQ 0) AND (S5.QVAL_14 EQ 101.5)) OR ((S5.2PQVAL1 EQ 0) AND (S5.2PQVAL2 EQ 0) AND (S5.2PQVAL3 EQ 0) AND (S5.2PQVAL4 EQ 0) AND (S5.2PQVAL5 EQ 0) AND (S5.2PQVAL6 EQ 0) AND (S5.2PQVAL7 EQ 0) AND (S5.2PQVAL8 EQ 0) AND (S5.2PQVAL9 EQ 0) AND (S5.2PQVAL_10 EQ 0) AND (S5.2PQVAL_11 EQ 0) AND (S5.2PQVAL_12 EQ 0) AND (S5.2PQVAL_13 EQ 0) AND (S5.2PQVAL_14 EQ 114))) [S5.MICROQ1 = 'Si j´use de mon jugement, je sais quelle est la bonne chose à faire. Je veux devenir active régulièrement.'];
IF (((S5.SUMVAL GE 200) AND (S5.SUMVAL LT 300) AND (S5.QVAL1 EQ 0) AND (S5.QVAL2 EQ 0) AND (S5.QVAL3 EQ 0) AND (S5.QVAL4 EQ 0) AND (S5.QVAL5 EQ 0) AND (S5.QVAL6 EQ 0) AND (S5.QVAL7 EQ 0) AND (S5.QVAL8 EQ 0) AND (S5.QVAL9 EQ 0) AND (S5.QVAL_10 EQ 0) AND (S5.QVAL_11 EQ 0) AND (S5.QVAL_12 EQ 0) AND (S5.QVAL_13 EQ 0) AND (S5.QVAL_14 EQ 0) AND (S5.QVAL_15 EQ 101.6)) OR ((S5.2PQVAL1 EQ 0) AND (S5.2PQVAL2 EQ 0) AND (S5.2PQVAL3 EQ 0) AND (S5.2PQVAL4 EQ 0) AND (S5.2PQVAL5 EQ 0) AND (S5.2PQVAL6 EQ 0) AND (S5.2PQVAL7 EQ 0) AND (S5.2PQVAL8 EQ 0) AND (S5.2PQVAL9 EQ 0) AND (S5.2PQVAL_10 EQ 0) AND (S5.2PQVAL_11 EQ 0) AND (S5.2PQVAL_12 EQ 0) AND (S5.2PQVAL_13 EQ 0) AND (S5.2PQVAL_14 EQ 0) AND (S5.2PQVAL_15 EQ 115))) [S5.MICROQ1 = 'Intégrer des activités physiques à mon horaire ça serait facile pour moi. Je vais faire ça, il faut juste que je pratique des activités simples comme la marche ou la natation. '];
IF (((S5.SUMVAL GE 200) AND (S5.SUMVAL LT 300) AND (S5.QVAL1 EQ 0) AND (S5.QVAL2 EQ 0) AND (S5.QVAL3 EQ 0) AND (S5.QVAL4 EQ 0) AND (S5.QVAL5 EQ 0) AND (S5.QVAL6 EQ 0) AND (S5.QVAL7 EQ 0) AND (S5.QVAL8 EQ 0) AND (S5.QVAL9 EQ 0) AND (S5.QVAL_10 EQ 0) AND (S5.QVAL_11 EQ 0) AND (S5.QVAL_12 EQ 0) AND (S5.QVAL_13 EQ 0) AND (S5.QVAL_14 EQ 0) AND (S5.QVAL_15 EQ 0) AND (S5.QVAL_16 EQ 101.7)) OR ((S5.2PQVAL1 EQ 0) AND (S5.2PQVAL2 EQ 0) AND (S5.2PQVAL3 EQ 0) AND (S5.2PQVAL4 EQ 0) AND (S5.2PQVAL5 EQ 0) AND (S5.2PQVAL6 EQ 0) AND (S5.2PQVAL7 EQ 0) AND (S5.2PQVAL8 EQ 0) AND (S5.2PQVAL9 EQ 0) AND (S5.2PQVAL_10 EQ 0) AND (S5.2PQVAL_11 EQ 0) AND (S5.2PQVAL_12 EQ 0) AND (S5.2PQVAL_13 EQ 0) AND (S5.2PQVAL_14 EQ 0) AND (S5.2PQVAL_15 EQ 0) AND (S5.2PQVAL_16 EQ 116))) [S5.MICROQ1 = 'Ce n´est pas mon genre d´éviter d´essayer. Pratiquer des activités physiques régulièrement est un beau défi à relever. Relever ce défi serait une expérience vraiment excitante.'];
IF (((S5.SUMVAL GE 200) AND (S5.SUMVAL LT 300) AND (S5.QVAL1 EQ 0) AND (S5.QVAL2 EQ 0) AND (S5.QVAL3 EQ 0) AND (S5.QVAL4 EQ 0) AND (S5.QVAL5 EQ 0) AND (S5.QVAL6 EQ 0) AND (S5.QVAL7 EQ 0) AND (S5.QVAL8 EQ 0) AND (S5.QVAL9 EQ 0) AND (S5.QVAL_10 EQ 0) AND (S5.QVAL_11 EQ 0) AND (S5.QVAL_12 EQ 0) AND (S5.QVAL_13 EQ 0) AND (S5.QVAL_14 EQ 0) AND (S5.QVAL_15 EQ 0) AND (S5.QVAL_16 EQ 0) AND (S5.QVAL_17 EQ 101.8)) OR ((S5.2PQVAL1 EQ 0) AND (S5.2PQVAL2 EQ 0) AND (S5.2PQVAL3 EQ 0) AND (S5.2PQVAL4 EQ 0) AND (S5.2PQVAL5 EQ 0) AND (S5.2PQVAL6 EQ 0) AND (S5.2PQVAL7 EQ 0) AND (S5.2PQVAL8 EQ 0) AND (S5.2PQVAL9 EQ 0) AND (S5.2PQVAL_10 EQ 0) AND (S5.2PQVAL_11 EQ 0) AND (S5.2PQVAL_12 EQ 0) AND (S5.2PQVAL_13 EQ 0) AND (S5.2PQVAL_14 EQ 0) AND (S5.2PQVAL_15 EQ 0) AND (S5.2PQVAL_16 EQ 0) AND (S5.2PQVAL_17 EQ 117))) [S5.MICROQ1 = 'Malgré tout ce qui pourrait m´empêcher de faire des activités physiques, je dois éviter que cela m´arrête. C´est vrai que je suis persévérant, je vais y arriver, je vais devenir actif.'];

IF (((S5.SUMVAL GE 200) AND (S5.SUMVAL LT 300) AND (S5.QVAL1 EQ 0) AND (S5.QVAL2 EQ 0) AND (S5.QVAL3 EQ 0) AND (S5.QVAL4 EQ 0) AND (S5.QVAL5 EQ 0) AND (S5.QVAL6 EQ 0) AND (S5.QVAL7 EQ 0) AND (S5.QVAL8 EQ 0) AND (S5.QVAL9 EQ 0) AND (S5.QVAL_10 EQ 0) AND (S5.QVAL_11 EQ 0) AND (S5.QVAL_12 EQ 0) AND (S5.QVAL_13 EQ 0) AND (S5.QVAL_14 EQ 0) AND (S5.QVAL_15 EQ 0) AND (S5.QVAL_16 EQ 0) AND (S5.QVAL_17 EQ 0) AND (S5.QVAL_18 EQ 101.9)) OR ((S5.2PQVAL1 EQ 0) AND (S5.2PQVAL2 EQ 0) AND (S5.2PQVAL3 EQ 0) AND (S5.2PQVAL4 EQ 0) AND (S5.2PQVAL5 EQ 0) AND (S5.2PQVAL6 EQ 0) AND (S5.2PQVAL7 EQ 0) AND (S5.2PQVAL8 EQ 0) AND (S5.2PQVAL9 EQ 0) AND (S5.2PQVAL_10 EQ 0) AND (S5.2PQVAL_11 EQ 0) AND (S5.2PQVAL_12 EQ 0) AND (S5.2PQVAL_13 EQ 0) AND (S5.2PQVAL_14 EQ 0) AND (S5.2PQVAL_15 EQ 0) AND (S5.2PQVAL_16 EQ 0) AND (S5.2PQVAL_17 EQ 0) AND (S5.2PQVAL_18 EQ 118))) [S5.MICROQ1 = 'Je connais les bénéfices de pratiquer des activités physiques régulièrement et les conséquences si je n´en pratique pas. Tout penche pour que j´en fasse. Ça me correspond de devenir une personne active.'];
IF (((S5.SUMVAL GE 200) AND (S5.SUMVAL LT 300) AND (S5.QVAL1 EQ 0) AND (S5.QVAL2 EQ 0) AND (S5.QVAL3 EQ 0) AND (S5.QVAL4 EQ 0) AND (S5.QVAL5 EQ 0) AND (S5.QVAL6 EQ 0) AND (S5.QVAL7 EQ 0) AND (S5.QVAL8 EQ 0) AND (S5.QVAL9 EQ 0) AND (S5.QVAL_10 EQ 0) AND (S5.QVAL_11 EQ 0) AND (S5.QVAL_12 EQ 0) AND (S5.QVAL_13 EQ 0) AND (S5.QVAL_14 EQ 0) AND (S5.QVAL_15 EQ 0) AND (S5.QVAL_16 EQ 0) AND (S5.QVAL_17 EQ 0) AND (S5.QVAL_18 EQ 0) AND (S5.QVAL_19 EQ 101.19)) OR ((S5.2PQVAL1 EQ 0) AND (S5.2PQVAL2 EQ 0) AND (S5.2PQVAL3 EQ 0) AND (S5.2PQVAL4 EQ 0) AND (S5.2PQVAL5 EQ 0) AND (S5.2PQVAL6 EQ 0) AND (S5.2PQVAL7 EQ 0) AND (S5.2PQVAL8 EQ 0) AND (S5.2PQVAL9 EQ 0) AND (S5.2PQVAL_10 EQ 0) AND (S5.2PQVAL_11 EQ 0) AND (S5.2PQVAL_12 EQ 0) AND (S5.2PQVAL_13 EQ 0) AND (S5.2PQVAL_14 EQ 0) AND (S5.2PQVAL_15 EQ 0) AND (S5.2PQVAL_16 EQ 0) AND (S5.2PQVAL_17 EQ 0) AND (S5.2PQVAL_18 EQ 0) AND (S5.2PQVAL_19 EQ 119))) [S5.MICROQ1 = 'Je suis chanceuse de pouvoir prendre soin de moi de façon autonome. Il faut que ça continue comme ça. Je veux devenir plus active.'];
IF (((S5.SUMVAL GE 200) AND (S5.SUMVAL LT 300) AND (S5.QVAL1 EQ 0) AND (S5.QVAL2 EQ 0) AND (S5.QVAL3 EQ 0) AND (S5.QVAL4 EQ 0) AND (S5.QVAL5 EQ 0) AND (S5.QVAL6 EQ 0) AND (S5.QVAL7 EQ 0) AND (S5.QVAL8 EQ 0) AND (S5.QVAL9 EQ 0) AND (S5.QVAL_10 EQ 0) AND (S5.QVAL_11 EQ 0) AND (S5.QVAL_12 EQ 0) AND (S5.QVAL_13 EQ 0) AND (S5.QVAL_14 EQ 0) AND (S5.QVAL_15 EQ 0) AND (S5.QVAL_16 EQ 0) AND (S5.QVAL_17 EQ 0) AND (S5.QVAL_18 EQ 0) AND (S5.QVAL_19 EQ 0) AND (S5.QVAL_20 EQ 102.1)) OR ((S5.2PQVAL1 EQ 0) AND (S5.2PQVAL2 EQ 0) AND (S5.2PQVAL3 EQ 0) AND (S5.2PQVAL4 EQ 0) AND (S5.2PQVAL5 EQ 0) AND (S5.2PQVAL6 EQ 0) AND (S5.2PQVAL7 EQ 0) AND (S5.2PQVAL8 EQ 0) AND (S5.2PQVAL9 EQ 0) AND (S5.2PQVAL_10 EQ 0) AND (S5.2PQVAL_11 EQ 0) AND (S5.2PQVAL_12 EQ 0) AND (S5.2PQVAL_13 EQ 0) AND (S5.2PQVAL_14 EQ 0) AND (S5.2PQVAL_15 EQ 0) AND (S5.2PQVAL_16 EQ 0) AND (S5.2PQVAL_17 EQ 0) AND (S5.2PQVAL_18 EQ 0) AND (S5.2PQVAL_19 EQ 0) AND (S5.2PQVAL_20 EQ 120))) [S5.MICROQ1 = 'J´aime les bonnes choses de la vie, mais je suis capable de faire la part des choses. Je veux devenir actif, c´est bon pour moi. Je vais y arriver.'];
IF (((S5.SUMVAL GE 200) AND (S5.SUMVAL LT 300) AND (S5.QVAL1 EQ 0) AND (S5.QVAL2 EQ 0) AND (S5.QVAL3 EQ 0) AND (S5.QVAL4 EQ 0) AND (S5.QVAL5 EQ 0) AND (S5.QVAL6 EQ 0) AND (S5.QVAL7 EQ 0) AND (S5.QVAL8 EQ 0) AND (S5.QVAL9 EQ 0) AND (S5.QVAL_10 EQ 0) AND (S5.QVAL_11 EQ 0) AND (S5.QVAL_12 EQ 0) AND (S5.QVAL_13 EQ 0) AND (S5.QVAL_14 EQ 0) AND (S5.QVAL_15 EQ 0) AND (S5.QVAL_16 EQ 0) AND (S5.QVAL_17 EQ 0) AND (S5.QVAL_18 EQ 0) AND (S5.QVAL_19 EQ 0) AND (S5.QVAL_20 EQ 0) AND (S5.QVAL_21 EQ 102.2)) OR ((S5.2PQVAL1 EQ 0) AND (S5.2PQVAL2 EQ 0) AND

(S5.2PQVAL3 EQ 0) AND (S5.2PQVAL4 EQ 0) AND (S5.2PQVAL5 EQ 0) AND (S5.2PQVAL6 EQ 0) AND (S5.2PQVAL7 EQ 0) AND (S5.2PQVAL8 EQ 0) AND (S5.2PQVAL9 EQ 0) AND (S5.2PQVAL_10 EQ 0) AND (S5.2PQVAL_11 EQ 0) AND (S5.2PQVAL_12 EQ 0) AND (S5.2PQVAL_13 EQ 0) AND (S5.2PQVAL_14 EQ 0) AND (S5.2PQVAL_15 EQ 0) AND (S5.2PQVAL_16 EQ 0) AND (S5.2PQVAL_17 EQ 0) AND (S5.2PQVAL_18 EQ 0) AND (S5.2PQVAL_19 EQ 0) AND (S5.2PQVAL_20 EQ 0) AND (S5.2PQVAL_21 EQ 121))) [S5.MICROQ1 = 'J´ai tout ce qu´il faut pour devenir une personne active. J´ai de l´argent, une famille et des amis qui me supporteraient et m´encourageraient. Je peux y arriver.'];
IF (((S5.SUMVAL GE 200) AND (S5.SUMVAL LT 300) AND (S5.QVAL1 EQ 0) AND (S5.QVAL2 EQ 0) AND (S5.QVAL3 EQ 0) AND (S5.QVAL4 EQ 0) AND (S5.QVAL5 EQ 0) AND (S5.QVAL6 EQ 0) AND (S5.QVAL7 EQ 0) AND (S5.QVAL8 EQ 0) AND (S5.QVAL9 EQ 0) AND (S5.QVAL_10 EQ 0) AND (S5.QVAL_11 EQ 0) AND (S5.QVAL_12 EQ 0) AND (S5.QVAL_13 EQ 0) AND (S5.QVAL_14 EQ 0) AND (S5.QVAL_15 EQ 0) AND (S5.QVAL_16 EQ 0) AND (S5.QVAL_17 EQ 0) AND (S5.QVAL_18 EQ 0) AND (S5.QVAL_19 EQ 0) AND (S5.QVAL_20 EQ 0) AND (S5.QVAL_21 EQ 0) AND (S5.QVAL_22 EQ 102.3)) OR ((S5.2PQVAL1 EQ 0) AND (S5.2PQVAL2 EQ 0) AND (S5.2PQVAL3 EQ 0) AND (S5.2PQVAL4 EQ 0) AND (S5.2PQVAL5 EQ 0) AND (S5.2PQVAL6 EQ 0) AND (S5.2PQVAL7 EQ 0) AND (S5.2PQVAL8 EQ 0) AND (S5.2PQVAL9 EQ 0) AND (S5.2PQVAL_10 EQ 0) AND (S5.2PQVAL_11 EQ 0) AND (S5.2PQVAL_12 EQ 0) AND (S5.2PQVAL_13 EQ 0) AND (S5.2PQVAL_14 EQ 0) AND (S5.2PQVAL_15 EQ 0) AND (S5.2PQVAL_16 EQ 0) AND (S5.2PQVAL_17 EQ 0) AND (S5.2PQVAL_18 EQ 0) AND (S5.2PQVAL_19 EQ 0) AND (S5.2PQVAL_20 EQ 0) AND (S5.2PQVAL_22 EQ 122))) [S5.MICROQ1 = 'Si je pèse les pour et les contres, c´est beaucoup plus avantageux pour moi de bouger régulièrement. C´est la plus sage décision que je pourrais prendre dans ma vie.'];
IF (((S5.SUMVAL GE 200) AND (S5.SUMVAL LT 300) AND (S5.QVAL1 EQ 0) AND (S5.QVAL2 EQ 0) AND (S5.QVAL3 EQ 0) AND (S5.QVAL4 EQ 0) AND (S5.QVAL5 EQ 0) AND (S5.QVAL6 EQ 0) AND (S5.QVAL7 EQ 0) AND (S5.QVAL8 EQ 0) AND (S5.QVAL9 EQ 0) AND (S5.QVAL_10 EQ 0) AND (S5.QVAL_11 EQ 0) AND (S5.QVAL_12 EQ 0) AND (S5.QVAL_13 EQ 0) AND (S5.QVAL_14 EQ 0) AND (S5.QVAL_15 EQ 0) AND (S5.QVAL_16 EQ 0) AND (S5.QVAL_17 EQ 0) AND (S5.QVAL_18 EQ 0) AND (S5.QVAL_19 EQ 0) AND (S5.QVAL_20 EQ 0) AND (S5.QVAL_21 EQ 0) AND (S5.QVAL_22 EQ 0) AND (S5.QVAL_23 EQ 102.4)) OR ((S5.2PQVAL1 EQ 0) AND (S5.2PQVAL2 EQ 0) AND (S5.2PQVAL3 EQ 0) AND (S5.2PQVAL4 EQ 0) AND (S5.2PQVAL5 EQ 0) AND (S5.2PQVAL6 EQ 0) AND (S5.2PQVAL7 EQ 0) AND (S5.2PQVAL8 EQ 0) AND (S5.2PQVAL9 EQ 0) AND (S5.2PQVAL_10 EQ 0) AND (S5.2PQVAL_11 EQ 0) AND (S5.2PQVAL_12 EQ 0) AND (S5.2PQVAL_13 EQ 0) AND (S5.2PQVAL_14 EQ 0) AND (S5.2PQVAL_15 EQ 0) AND (S5.2PQVAL_16 EQ 0) AND (S5.2PQVAL_17 EQ 0) AND (S5.2PQVAL_18 EQ 0) AND (S5.2PQVAL_19 EQ 0) AND (S5.2PQVAL_20 EQ 0) AND (S5.2PQVAL_22 EQ 0) AND (S5.2PQVAL_23 EQ 123))) [S5.MICROQ1 = 'J´évite toujours de reculer devant un défi. Bouger régulièrement est seulement un autre défi à relever. Je suis capable de faire ça.'];
IF (((S5.SUMVAL GE 200) AND (S5.SUMVAL LT 300) AND (S5.QVAL1 EQ 0) AND (S5.QVAL2 EQ 0) AND (S5.QVAL3 EQ 0) AND (S5.QVAL4 EQ 0) AND (S5.QVAL5 EQ 0) AND (S5.QVAL6 EQ 0) AND (S5.QVAL7 EQ 0) AND (S5.QVAL8 EQ 0) AND (S5.QVAL9 EQ 0) AND (S5.QVAL_10 EQ 0) AND (S5.QVAL_11 EQ 0) AND (S5.QVAL_12 EQ 0) AND (S5.QVAL_13 EQ 0) AND (S5.QVAL_14 EQ 0) AND (S5.QVAL_15 EQ 0) AND (S5.QVAL_16 EQ 0) AND (S5.QVAL_17 EQ 0) AND (S5.QVAL_18 EQ 0) AND (S5.QVAL_19 EQ 0) AND (S5.QVAL_20 EQ 0) AND (S5.QVAL_21 EQ 0) AND (S5.QVAL_22 EQ 0) AND (S5.QVAL_23 EQ 0) AND (S5.QVAL_24 EQ 102.5)) OR ((S5.2PQVAL1 EQ 0) AND (S5.2PQVAL2 EQ 0) AND (S5.2PQVAL3 EQ 0) AND (S5.2PQVAL4 EQ 0) AND (S5.2PQVAL5 EQ 0) AND (S5.2PQVAL6 EQ 0) AND (S5.2PQVAL7 EQ 0) AND (S5.2PQVAL8 EQ 0) AND (S5.2PQVAL9 EQ 0) AND (S5.2PQVAL_10 EQ 0) AND (S5.2PQVAL_11 EQ 0) AND

(S5.2PQVAL_12 EQ 0) AND (S5.2PQVAL_13 EQ 0) AND (S5.2PQVAL_14 EQ 0) AND (S5.2PQVAL_15 EQ 0) AND (S5.2PQVAL_16 EQ 0) AND (S5.2PQVAL_17 EQ 0) AND (S5.2PQVAL_18 EQ 0) AND (S5.2PQVAL_19 EQ 0) AND (S5.2PQVAL_20 EQ 0) AND (S5.2PQVAL_22 EQ 0) AND (S5.2PQVAL_23 EQ 0) AND (S5.2PQVAL_24 EQ 124))) [S5.MICROQ1 = 'Je sais où je veux aller, et bouger régulièrement doit faire partie du chemin. Je vais trouver une façon d´y arriver je n´abandonnerai jamais.'];
IF (((S5.SUMVAL GE 200) AND (S5.SUMVAL LT 300) AND (S5.QVAL1 EQ 0) AND (S5.QVAL2 EQ 0) AND (S5.QVAL3 EQ 0) AND (S5.QVAL4 EQ 0) AND (S5.QVAL5 EQ 0) AND (S5.QVAL6 EQ 0) AND (S5.QVAL7 EQ 0) AND (S5.QVAL8 EQ 0) AND (S5.QVAL9 EQ 0) AND (S5.QVAL_10 EQ 0) AND (S5.QVAL_11 EQ 0) AND (S5.QVAL_12 EQ 0) AND (S5.QVAL_13 EQ 0) AND (S5.QVAL_14 EQ 0) AND (S5.QVAL_15 EQ 0) AND (S5.QVAL_16 EQ 0) AND (S5.QVAL_17 EQ 0) AND (S5.QVAL_18 EQ 0) AND (S5.QVAL_19 EQ 0) AND (S5.QVAL_20 EQ 0) AND (S5.QVAL_21 EQ 0) AND (S5.QVAL_22 EQ 0) AND (S5.QVAL_23 EQ 0) AND (S5.QVAL_24 EQ 0) AND (S5.QVAL_25 EQ 102.6)) OR ((S5.2PQVAL1 EQ 0) AND (S5.2PQVAL2 EQ 0) AND (S5.2PQVAL3 EQ 0) AND (S5.2PQVAL4 EQ 0) AND (S5.2PQVAL5 EQ 0) AND (S5.2PQVAL6 EQ 0) AND (S5.2PQVAL7 EQ 0) AND (S5.2PQVAL8 EQ 0) AND (S5.2PQVAL9 EQ 0) AND (S5.2PQVAL_10 EQ 0) AND (S5.2PQVAL_11 EQ 0) AND (S5.2PQVAL_12 EQ 0) AND (S5.2PQVAL_13 EQ 0) AND (S5.2PQVAL_14 EQ 0) AND (S5.2PQVAL_15 EQ 0) AND (S5.2PQVAL_16 EQ 0) AND (S5.2PQVAL_17 EQ 0) AND (S5.2PQVAL_18 EQ 0) AND (S5.2PQVAL_19 EQ 0) AND (S5.2PQVAL_20 EQ 0) AND (S5.2PQVAL_22 EQ 0) AND (S5.2PQVAL_23 EQ 0) AND (S5.2PQVAL_24 EQ 0) AND (S5.2PQVAL_25 EQ 125))) [S5.MICROQ1 = 'J´ai toujours été honnête envers moi et mes besoins. Bouger régulièrement serait très bon pour moi, c´est sûr. Je veux et suis capable.'];
IF (((S5.SUMVAL GE 200) AND (S5.SUMVAL LT 300) AND (S5.QVAL1 EQ 101) AND (S5.QVAL2 EQ 102)) OR ((S5.2PQVAL1 EQ 101) AND (S5.2PQVAL2 EQ 102))) [S5.MICROQ2 = 'Je dois être à l´écoute de moi-même d´abord si je veux aider les autres.'];
IF (((S5.SUMVAL GE 200) AND (S5.SUMVAL LT 300) AND ((S5.QVAL1 EQ 101) OR (S5.QVAL2 EQ 102)) AND (S5.QVAL3 EQ 103)) OR (((S5.2PQVAL1 EQ 101) OR (S5.2PQVAL2 EQ 102)) AND (S5.2PQVAL3 EQ 103))) [S5.MICROQ2 = 'Je dois être à l´écoute des autres, mais aussi de moi-même. Je dois m´écouter et faire ce qui est bon pour moi.'];
IF (((S5.SUMVAL GE 200) AND (S5.SUMVAL LT 300) AND ((S5.QVAL1 EQ 101) OR (S5.QVAL2 EQ 102) OR (S5.QVAL3 EQ 103)) AND (S5.QVAL4 EQ 104)) OR (((S5.2PQVAL1 EQ 101) OR (S5.2PQVAL2 EQ 102) OR (S5.2PQVAL3 EQ 103)) AND (S5.2PQVAL4 EQ 104))) [S5.MICROQ2 = 'Je sais que je suis capable d´y arriver. J´ai réussi des choses aussi dures ou sinon plus que ça.'];
IF (((S5.SUMVAL GE 200) AND (S5.SUMVAL LT 300) AND ((S5.QVAL1 EQ 101) OR (S5.QVAL2 EQ 102) OR (S5.QVAL3 EQ 103) OR (S5.QVAL4 EQ 104)) AND (S5.QVAL5 EQ 105)) OR (((S5.2PQVAL1 EQ 101) OR (S5.2PQVAL2 EQ 102) OR (S5.2PQVAL3 EQ 103) OR (S5.2PQVAL4 EQ 104)) AND (S5.2PQVAL5 EQ 105))) [S5.MICROQ2 = 'Je suis douée dans plusieurs choses. J´ignore pourquoi pratiquer des activités physiques devrait être différent.'];
IF (((S5.SUMVAL GE 200) AND (S5.SUMVAL LT 300) AND ((S5.QVAL1 EQ 101) OR (S5.QVAL2 EQ 102) OR (S5.QVAL3 EQ 103) OR (S5.QVAL4 EQ 104) OR (S5.QVAL5 EQ 105)) AND (S5.QVAL6 EQ 106)) OR (((S5.2PQVAL1 EQ 101) OR (S5.2PQVAL2 EQ 102) OR (S5.2PQVAL3 EQ 103) OR (S5.2PQVAL4 EQ 104) OR (S5.2PQVAL5 EQ 105)) AND (S5.2PQVAL6 EQ 106))) [S5.MICROQ2 = 'J´ai confiance en moi. Je n´ai qu´à prendre une décision ferme que je vais bouger régulièrement et je vais y arriver.'];

IF (((S5.SUMVAL GE 200) AND (S5.SUMVAL LT 300) AND ((S5.QVAL1 EQ 101) OR (S5.QVAL2 EQ 102) OR (S5.QVAL3 EQ 103) OR (S5.QVAL4 EQ 104) OR (S5.QVAL5 EQ 105) OR (S5.QVAL6 EQ 106)) AND (S5.QVAL7 EQ 107)) OR (((S5.2PQVAL1 EQ 101) OR (S5.2PQVAL2 EQ 102) OR (S5.2PQVAL3 EQ 103) OR (S5.2PQVAL4 EQ 104) OR (S5.2PQVAL5 EQ 105) OR (S5.2PQVAL6 EQ 106)) AND (S5.2PQVAL7 EQ 107))) [S5.MICROQ2 = 'Je reviens rarement en arrière lorsque je décide de faire un projet. Si je décide de devenir active régulièrement, je vais y arriver et maintenir l´habitude.'];
IF (((S5.SUMVAL GE 200) AND (S5.SUMVAL LT 300) AND ((S5.QVAL1 EQ 101) OR (S5.QVAL2 EQ 102) OR (S5.QVAL3 EQ 103) OR (S5.QVAL4 EQ 104) OR (S5.QVAL5 EQ 105) OR (S5.QVAL6 EQ 106) OR (S5.QVAL7 EQ 107)) AND (S5.QVAL8 EQ 108)) OR (((S5.2PQVAL1 EQ 101) OR (S5.2PQVAL2 EQ 102) OR (S5.2PQVAL3 EQ 103) OR (S5.2PQVAL4 EQ 104) OR (S5.2PQVAL5 EQ 105) OR (S5.2PQVAL6 EQ 106) OR (S5.2PQVAL7 EQ 107)) AND (S5.2PQVAL8 EQ 108))) [S5.MICROQ2 = 'Je vais toujours au bout de mes projets. Ce devrait être la même chose avec la pratique d´activités physiques.'];
IF (((S5.SUMVAL GE 200) AND (S5.SUMVAL LT 300) AND ((S5.QVAL1 EQ 101) OR (S5.QVAL2 EQ 102) OR (S5.QVAL3 EQ 103) OR (S5.QVAL4 EQ 104) OR (S5.QVAL5 EQ 105) OR (S5.QVAL6 EQ 106) OR (S5.QVAL7 EQ 107) OR (S5.QVAL8 EQ 108)) AND (S5.QVAL9 EQ 109)) OR (((S5.2PQVAL1 EQ 101) OR (S5.2PQVAL2 EQ 102) OR (S5.2PQVAL3 EQ 103) OR (S5.2PQVAL4 EQ 104) OR (S5.2PQVAL5 EQ 105) OR (S5.2PQVAL6 EQ 106) OR (S5.2PQVAL7 EQ 107) OR (S5.2PQVAL8 EQ 108)) AND (S5.2PQVAL9 EQ 109))) [S5.MICROQ2 = 'Je fais tout ce qu´il faut pour réussir ce que j´entreprends. Je dois faire la même chose avec l´activité physique. Je vais faire ça.'];
IF (((S5.SUMVAL GE 200) AND (S5.SUMVAL LT 300) AND ((S5.QVAL1 EQ 101) OR (S5.QVAL2 EQ 102) OR (S5.QVAL3 EQ 103) OR (S5.QVAL4 EQ 104) OR (S5.QVAL5 EQ 105) OR (S5.QVAL6 EQ 106) OR (S5.QVAL7 EQ 107) OR (S5.QVAL8 EQ 108) OR (S5.QVAL9 EQ 109)) AND (S5.QVAL_10 EQ 101.1)) OR (((S5.2PQVAL1 EQ 101) OR (S5.2PQVAL2 EQ 102) OR (S5.2PQVAL3 EQ 103) OR (S5.2PQVAL4 EQ 104) OR (S5.2PQVAL5 EQ 105) OR (S5.2PQVAL6 EQ 106) OR (S5.2PQVAL7 EQ 107) OR (S5.2PQVAL8 EQ 108) OR (S5.2PQVAL9 EQ 109)) AND (S5.2PQVAL_10 EQ 110))) [S5.MICROQ2 = 'Je pourrais organiser mon horaire pour intégrer des activités physiques. Je suis certainement assez efficace pour faire ça.'];
IF (((S5.SUMVAL GE 200) AND (S5.SUMVAL LT 300) AND ((S5.QVAL1 EQ 101) OR (S5.QVAL2 EQ 102) OR (S5.QVAL3 EQ 103) OR (S5.QVAL4 EQ 104) OR (S5.QVAL5 EQ 105) OR (S5.QVAL6 EQ 106) OR (S5.QVAL7 EQ 107) OR (S5.QVAL8 EQ 108) OR (S5.QVAL9 EQ 109) OR (S5.QVAL_10 EQ 101.1)) AND (S5.QVAL_11 EQ 101.2)) OR (((S5.2PQVAL1 EQ 101) OR (S5.2PQVAL2 EQ 102) OR (S5.2PQVAL3 EQ 103) OR (S5.2PQVAL4 EQ 104) OR (S5.2PQVAL5 EQ 105) OR (S5.2PQVAL6 EQ 106) OR (S5.2PQVAL7 EQ 107) OR (S5.2PQVAL8 EQ 108) OR (S5.2PQVAL9 EQ 109) OR (S5.2PQVAL_10 EQ 110)) AND (S5.2PQVAL_11 EQ 111))) [S5.MICROQ2 = 'Je pourrais dépenser une partie de mon énergie dans ma santé. C´est sûr que j´ai assez d´énergie pour pratiquer des activités physiques. Je suis capable.'];
IF (((S5.SUMVAL GE 200) AND (S5.SUMVAL LT 300) AND ((S5.QVAL1 EQ 101) OR (S5.QVAL2 EQ 102) OR (S5.QVAL3 EQ 103) OR (S5.QVAL4 EQ 104) OR (S5.QVAL5 EQ 105) OR (S5.QVAL6 EQ 106) OR (S5.QVAL7 EQ 107) OR (S5.QVAL8 EQ 108) OR (S5.QVAL9 EQ 109) OR (S5.QVAL_10 EQ 101.1) OR (S5.QVAL_11 EQ 101.2)) AND (S5.QVAL_12 EQ 101.3)) OR (((S5.2PQVAL1 EQ 101) OR (S5.2PQVAL2 EQ 102) OR (S5.2PQVAL3 EQ 103) OR (S5.2PQVAL4 EQ 104) OR (S5.2PQVAL5 EQ 105) OR (S5.2PQVAL6 EQ 106) OR (S5.2PQVAL7 EQ 107) OR (S5.2PQVAL8 EQ 108) OR (S5.2PQVAL9 EQ 109) OR (S5.2PQVAL_10 EQ 110) OR (S5.2PQVAL_11 EQ 111)) AND (S5.2PQVAL_12 EQ 112))) [S5.MICROQ2 = 'Je peux maintenir mon humeur positive lorsqu´il est temps de pratiquer des activités physiques. L´activité physique c´est bon et ça me ferait plaisir d´en faire.'];

IF (((S5.SUMVAL GE 200) AND (S5.SUMVAL LT 300) AND ((S5.QVAL1 EQ 101) OR (S5.QVAL2 EQ 102) OR (S5.QVAL3 EQ 103) OR (S5.QVAL4 EQ 104) OR (S5.QVAL5 EQ 105) OR (S5.QVAL6 EQ 106) OR (S5.QVAL7 EQ 107) OR (S5.QVAL8 EQ 108) OR (S5.QVAL9 EQ 109) OR (S5.QVAL_10 EQ 101.1) OR (S5.QVAL_11 EQ 101.2) OR (S5.QVAL_12 EQ 101.3)) AND (S5.QVAL_13 EQ 101.4)) OR (((S5.2PQVAL1 EQ 101) OR (S5.2PQVAL2 EQ 102) OR (S5.2PQVAL3 EQ 103) OR (S5.2PQVAL4 EQ 104) OR (S5.2PQVAL5 EQ 105) OR (S5.2PQVAL6 EQ 106) OR (S5.2PQVAL7 EQ 107) OR (S5.2PQVAL8 EQ 108) OR (S5.2PQVAL9 EQ 109) OR (S5.2PQVAL_10 EQ 110) OR (S5.2PQVAL_11 EQ 111) OR (S5.2PQVAL_12 EQ 112)) AND (S5.2PQVAL_13 EQ 113))) [S5.MICROQ2 = 'Je performe dans tout ce que j´entreprends. Devenir active régulièrement n´est pas une exception à la règle.'];
IF (((S5.SUMVAL GE 200) AND (S5.SUMVAL LT 300) AND ((S5.QVAL1 EQ 101) OR (S5.QVAL2 EQ 102) OR (S5.QVAL3 EQ 103) OR (S5.QVAL4 EQ 104) OR (S5.QVAL5 EQ 105) OR (S5.QVAL6 EQ 106) OR (S5.QVAL7 EQ 107) OR (S5.QVAL8 EQ 108) OR (S5.QVAL9 EQ 109) OR (S5.QVAL_10 EQ 101.1) OR (S5.QVAL_11 EQ 101.2) OR (S5.QVAL_12 EQ 101.3)) AND (S5.QVAL_13 EQ 101.4)) OR (((S5.2PQVAL1 EQ 101) OR (S5.2PQVAL2 EQ 102) OR (S5.2PQVAL3 EQ 103) OR (S5.2PQVAL4 EQ 104) OR (S5.2PQVAL5 EQ 105) OR (S5.2PQVAL6 EQ 106) OR (S5.2PQVAL7 EQ 107) OR (S5.2PQVAL8 EQ 108) OR (S5.2PQVAL9 EQ 109) OR (S5.2PQVAL_10 EQ 110) OR (S5.2PQVAL_11 EQ 111) OR (S5.2PQVAL_12 EQ 112) OR (S5.2PQVAL_13 EQ 113)) AND (S5.2PQVAL_14 EQ 114))) [S5.MICROQ2 = 'Si j´use de mon jugement, je sais quelle est la bonne chose à faire. Je veux devenir active régulièrement'];
IF (((S5.SUMVAL GE 200) AND (S5.SUMVAL LT 300) AND ((S5.QVAL1 EQ 101) OR (S5.QVAL2 EQ 102) OR (S5.QVAL3 EQ 103) OR (S5.QVAL4 EQ 104) OR (S5.QVAL5 EQ 105) OR (S5.QVAL6 EQ 106) OR (S5.QVAL7 EQ 107) OR (S5.QVAL8 EQ 108) OR (S5.QVAL9 EQ 109) OR (S5.QVAL_10 EQ 101.1) OR (S5.QVAL_11 EQ 101.2) OR (S5.QVAL_12 EQ 101.3) OR (S5.QVAL_13 EQ 101.4) OR (S5.QVAL_14 EQ 101.5)) AND (S5.QVAL_15 EQ 101.6)) OR (((S5.2PQVAL1 EQ 101) OR (S5.2PQVAL2 EQ 102) OR (S5.2PQVAL3 EQ 103) OR (S5.2PQVAL4 EQ 104) OR (S5.2PQVAL5 EQ 105) OR (S5.2PQVAL6 EQ 106) OR (S5.2PQVAL7 EQ 107) OR (S5.2PQVAL8 EQ 108) OR (S5.2PQVAL9 EQ 109) OR (S5.2PQVAL_10 EQ 110) OR (S5.2PQVAL_11 EQ 111) OR (S5.2PQVAL_12 EQ 112) OR (S5.2PQVAL_13 EQ 113) OR (S5.2PQVAL_14 EQ 114)) AND (S5.2PQVAL_15 EQ 115))) [S5.MICROQ2 = 'Intégrer des activités physiques à mon horaire ça serait facile pour moi. Il faut juste que je pratique des activités simples comme la marche ou la natation.'];
IF (((S5.SUMVAL GE 200) AND (S5.SUMVAL LT 300) AND ((S5.QVAL1 EQ 101) OR (S5.QVAL2 EQ 102) OR (S5.QVAL3 EQ 103) OR (S5.QVAL4 EQ 104) OR (S5.QVAL5 EQ 105) OR (S5.QVAL6 EQ 106) OR (S5.QVAL7 EQ 107) OR (S5.QVAL8 EQ 108) OR (S5.QVAL9 EQ 109) OR (S5.QVAL_10 EQ 101.1) OR (S5.QVAL_11 EQ 101.2) OR (S5.QVAL_12 EQ 101.3) OR (S5.QVAL_13 EQ 101.4) OR (S5.QVAL_14 EQ 101.5) OR (S5.QVAL_15 EQ 101.6)) AND (S5.QVAL_16 EQ 101.7)) OR (((S5.2PQVAL1 EQ 101) OR (S5.2PQVAL2 EQ 102) OR (S5.2PQVAL3 EQ 103) OR (S5.2PQVAL4 EQ 104) OR (S5.2PQVAL5 EQ 105) OR (S5.2PQVAL6 EQ 106) OR (S5.2PQVAL7 EQ 107) OR (S5.2PQVAL8 EQ 108) OR (S5.2PQVAL9 EQ 109) OR (S5.2PQVAL_10 EQ 110) OR (S5.2PQVAL_11 EQ 111) OR (S5.2PQVAL_12 EQ 112) OR (S5.2PQVAL_13 EQ 113) OR (S5.2PQVAL_14 EQ 114) OR (S5.2PQVAL_15 EQ 115)) AND (S5.2PQVAL_16 EQ 116))) [S5.MICROQ2 = 'Ce n´est pas mon genre d´éviter d´essayer. Pratiquer des activités physiques régulièrement est un beau défi à relever. Le relever serait une expérience vraiment excitante.'];
IF (((S5.SUMVAL GE 200) AND (S5.SUMVAL LT 300) AND ((S5.QVAL1 EQ 101) OR (S5.QVAL2 EQ 102) OR (S5.QVAL3 EQ 103) OR (S5.QVAL4 EQ 104) OR (S5.QVAL5 EQ 105) OR (S5.QVAL6 EQ 106) OR (S5.QVAL7 EQ 107) OR (S5.QVAL8 EQ 108) OR (S5.QVAL9 EQ 109) OR (S5.QVAL_10 EQ 101.1) OR (S5.QVAL_11 EQ 101.2) OR (S5.QVAL_12 EQ 101.3) OR (S5.QVAL_13 EQ 101.4) OR (S5.QVAL_14 EQ 101.5) OR (S5.QVAL_15 EQ

101.6) OR (S5.QVAL_16 EQ 101.7)) AND (S5.QVAL_17 EQ 101.8)) OR (((S5.2PQVAL1 EQ 101) OR (S5.2PQVAL2 EQ 102) OR (S5.2PQVAL3 EQ 103) OR (S5.2PQVAL4 EQ 104) OR (S5.2PQVAL5 EQ 105) OR (S5.2PQVAL6 EQ 106) OR (S5.2PQVAL7 EQ 107) OR (S5.2PQVAL8 EQ 108) OR (S5.2PQVAL9 EQ 109) OR (S5.2PQVAL_10 EQ 110) OR (S5.2PQVAL_11 EQ 111) OR (S5.2PQVAL_12 EQ 112) OR (S5.2PQVAL_13 EQ 113) OR (S5.2PQVAL_14 EQ 114) OR (S5.2PQVAL_15 EQ 115) OR (S5.2PQVAL_16 EQ 116)) AND (S5.2PQVAL_17 EQ 117))) [S5.MICROQ2 = 'Malgré tout ce qui pourrait m´empêcher de faire des activités physiques, je dois éviter que cela m´arrête. C´est vrai que je suis persévérant, je vais y arriver, je vais devenir actif.'];
IF (((S5.SUMVAL GE 200) AND (S5.SUMVAL LT 300) AND ((S5.QVAL1 EQ 101) OR (S5.QVAL2 EQ 102) OR (S5.QVAL3 EQ 103) OR (S5.QVAL4 EQ 104) OR (S5.QVAL5 EQ 105) OR (S5.QVAL6 EQ 106) OR (S5.QVAL7 EQ 107) OR (S5.QVAL8 EQ 108) OR (S5.QVAL9 EQ 109) OR (S5.QVAL_10 EQ 101.1) OR (S5.QVAL_11 EQ 101.2) OR (S5.QVAL_12 EQ 101.3) OR (S5.QVAL_13 EQ 101.4) OR (S5.QVAL_14 EQ 101.5) OR (S5.QVAL_15 EQ 101.6) OR (S5.QVAL_16 EQ 101.7) OR (S5.QVAL_17 EQ 101.8)) AND (S5.QVAL_18 EQ 101.9)) OR (((S5.2PQVAL1 EQ 101) OR (S5.2PQVAL2 EQ 102) OR (S5.2PQVAL3 EQ 103) OR (S5.2PQVAL4 EQ 104) OR (S5.2PQVAL5 EQ 105) OR (S5.2PQVAL6 EQ 106) OR (S5.2PQVAL7 EQ 107) OR (S5.2PQVAL8 EQ 108) OR (S5.2PQVAL9 EQ 109) OR (S5.2PQVAL_10 EQ 110) OR (S5.2PQVAL_11 EQ 111) OR (S5.2PQVAL_12 EQ 112) OR (S5.2PQVAL_13 EQ 113) OR (S5.2PQVAL_14 EQ 114) OR (S5.2PQVAL_15 EQ 115) OR (S5.2PQVAL_16 EQ 116) OR (S5.2PQVAL_17 EQ 117)) AND (S5.2PQVAL_18 EQ 118))) [S5.MICROQ2 = 'Je connais les bénéfices de pratiquer des activités physiques régulièrement et les conséquences du contraire. Tout penche pour que j´en fasse. Ça me correspond de devenir une personne active.'];
IF (((S5.SUMVAL GE 200) AND (S5.SUMVAL LT 300) AND ((S5.QVAL1 EQ 101) OR (S5.QVAL2 EQ 102) OR (S5.QVAL3 EQ 103) OR (S5.QVAL4 EQ 104) OR (S5.QVAL5 EQ 105) OR (S5.QVAL6 EQ 106) OR (S5.QVAL7 EQ 107) OR (S5.QVAL8 EQ 108) OR (S5.QVAL9 EQ 109) OR (S5.QVAL_10 EQ 101.1) OR (S5.QVAL_11 EQ 101.2) OR (S5.QVAL_12 EQ 101.3) OR (S5.QVAL_13 EQ 101.4) OR (S5.QVAL_14 EQ 101.5) OR (S5.QVAL_15 EQ 101.6) OR (S5.QVAL_16 EQ 101.7) OR (S5.QVAL_17 EQ 101.8) OR (S5.QVAL_18 EQ 101.9)) AND (S5.QVAL_19 EQ 101.19))OR (((S5.2PQVAL1 EQ 101) OR (S5.2PQVAL2 EQ 102) OR (S5.2PQVAL3 EQ 103) OR (S5.2PQVAL4 EQ 104) OR (S5.2PQVAL5 EQ 105) OR (S5.2PQVAL6 EQ 106) OR (S5.2PQVAL7 EQ 107) OR (S5.2PQVAL8 EQ 108) OR (S5.2PQVAL9 EQ 109) OR (S5.2PQVAL_10 EQ 110) OR (S5.2PQVAL_11 EQ 111) OR (S5.2PQVAL_12 EQ 112) OR (S5.2PQVAL_13 EQ 113) OR (S5.2PQVAL_14 EQ 114) OR (S5.2PQVAL_15 EQ 115) OR (S5.2PQVAL_16 EQ 116) OR (S5.2PQVAL_17 EQ 117) OR (S5.2PQVAL_18 EQ 118)) AND (S5.2PQVAL_19 EQ 119))) [S5.MICROQ2 = 'Je suis chanceuse de pouvoir prendre soin de moi de façon autonome. Il faut que ça continue comme ça. Je veux devenir plus active.'];
IF (((S5.SUMVAL GE 200) AND (S5.SUMVAL LT 300) AND ((S5.QVAL1 EQ 101) OR (S5.QVAL2 EQ 102) OR (S5.QVAL3 EQ 103) OR (S5.QVAL4 EQ 104) OR (S5.QVAL5 EQ 105) OR (S5.QVAL6 EQ 106) OR (S5.QVAL7 EQ 107) OR (S5.QVAL8 EQ 108) OR (S5.QVAL9 EQ 109) OR (S5.QVAL_10 EQ 101.1) OR (S5.QVAL_11 EQ 101.2) OR (S5.QVAL_12 EQ 101.3) OR (S5.QVAL_13 EQ 101.4) OR (S5.QVAL_14 EQ 101.5) OR (S5.QVAL_15 EQ 101.6) OR (S5.QVAL_16 EQ 101.7) OR (S5.QVAL_17 EQ 101.8) OR (S5.QVAL_18 EQ 101.9) OR (S5.QVAL_19 EQ 101.19)) AND (S5.QVAL_20 EQ 102.1)) OR (((S5.2PQVAL1 EQ 101) OR (S5.2PQVAL2 EQ 102) OR (S5.2PQVAL3 EQ 103) OR (S5.2PQVAL4 EQ 104) OR (S5.2PQVAL5 EQ 105) OR (S5.2PQVAL6 EQ 106) OR (S5.2PQVAL7 EQ 107) OR (S5.2PQVAL8 EQ 108) OR (S5.2PQVAL9 EQ 109) OR (S5.2PQVAL_10 EQ 110) OR (S5.2PQVAL_11 EQ 111) OR (S5.2PQVAL_12 EQ 112) OR (S5.2PQVAL_13 EQ 113) OR (S5.2PQVAL_14 EQ 114) OR (S5.2PQVAL_15 EQ 115) OR (S5.2PQVAL_16 EQ 116) OR (S5.2PQVAL_17 EQ 117) OR

(S5.2PQVAL_18 EQ 118) OR (S5.2PQVAL_19 EQ 119)) AND (S5.2PQVAL_20 EQ 120))) [S5.MICROQ2 = 'J´aime les bonnes choses de la vie, mais je suis capable de faire la part des choses. Je veux devenir actif, c´est bon pour moi. Je vais y arriver.'];
IF (((S5.SUMVAL GE 200) AND (S5.SUMVAL LT 300) AND ((S5.QVAL1 EQ 101) OR (S5.QVAL2 EQ 102) OR (S5.QVAL3 EQ 103) OR (S5.QVAL4 EQ 104) OR (S5.QVAL5 EQ 105) OR (S5.QVAL6 EQ 106) OR (S5.QVAL7 EQ 107) OR (S5.QVAL8 EQ 108) OR (S5.QVAL9 EQ 109) OR (S5.QVAL_10 EQ 101.1) OR (S5.QVAL_11 EQ 101.2) OR (S5.QVAL_12 EQ 101.3) OR (S5.QVAL_13 EQ 101.4) OR (S5.QVAL_14 EQ 101.5) OR (S5.QVAL_15 EQ 101.6) OR (S5.QVAL_16 EQ 101.7) OR (S5.QVAL_17 EQ 101.8) OR (S5.QVAL_18 EQ 101.9) OR (S5.QVAL_19 EQ 101.19) OR (S5.QVAL_20 EQ 102.1)) AND (S5.QVAL_21 EQ 102.2)) OR (((S5.2PQVAL1 EQ 101) OR (S5.2PQVAL2 EQ 102) OR (S5.2PQVAL3 EQ 103) OR (S5.2PQVAL4 EQ 104) OR (S5.2PQVAL5 EQ 105) OR (S5.2PQVAL6 EQ 106) OR (S5.2PQVAL7 EQ 107) OR (S5.2PQVAL8 EQ 108) OR (S5.2PQVAL9 EQ 109) OR (S5.2PQVAL_10 EQ 110) OR (S5.2PQVAL_11 EQ 111) OR (S5.2PQVAL_12 EQ 112) OR (S5.2PQVAL_13 EQ 113) OR (S5.2PQVAL_14 EQ 114) OR (S5.2PQVAL_15 EQ 115) OR (S5.2PQVAL_16 EQ 116) OR (S5.2PQVAL_17 EQ 117) OR (S5.2PQVAL_18 EQ 118) OR (S5.2PQVAL_19 EQ 119) OR (S5.2PQVAL_20 EQ 120)) AND (S5.2PQVAL_21 EQ 121))) [S5.MICROQ2 = 'J´ai tout ce qu´il faut pour devenir une personne active. J´ai de l´argent, une famille et des amis qui me supporteraient et m´encourageraient. Je peux y arriver.'];
IF (((S5.SUMVAL GE 200) AND (S5.SUMVAL LT 300) AND ((S5.QVAL1 EQ 101) OR (S5.QVAL2 EQ 102) OR (S5.QVAL3 EQ 103) OR (S5.QVAL4 EQ 104) OR (S5.QVAL5 EQ 105) OR (S5.QVAL6 EQ 106) OR (S5.QVAL7 EQ 107) OR (S5.QVAL8 EQ 108) OR (S5.QVAL9 EQ 109) OR (S5.QVAL_10 EQ 101.1) OR (S5.QVAL_11 EQ 101.2) OR (S5.QVAL_12 EQ 101.3) OR (S5.QVAL_13 EQ 101.4) OR (S5.QVAL_14 EQ 101.5) OR (S5.QVAL_15 EQ 101.6) OR (S5.QVAL_16 EQ 101.7) OR (S5.QVAL_17 EQ 101.8) OR (S5.QVAL_18 EQ 101.9) OR (S5.QVAL_19 EQ 101.19) OR (S5.QVAL_20 EQ 102.1) OR (S5.QVAL_21 EQ 102.2)) AND (S5.QVAL_22 EQ 102.3)) OR (((S5.2PQVAL1 EQ 101) OR (S5.2PQVAL2 EQ 102) OR (S5.2PQVAL3 EQ 103) OR (S5.2PQVAL4 EQ 104) OR (S5.2PQVAL5 EQ 105) OR (S5.2PQVAL6 EQ 106) OR (S5.2PQVAL7 EQ 107) OR (S5.2PQVAL8 EQ 108) OR (S5.2PQVAL9 EQ 109) OR (S5.2PQVAL_10 EQ 110) OR (S5.2PQVAL_11 EQ 111) OR (S5.2PQVAL_12 EQ 112) OR (S5.2PQVAL_13 EQ 113) OR (S5.2PQVAL_14 EQ 114) OR (S5.2PQVAL_15 EQ 115) OR (S5.2PQVAL_16 EQ 116) OR (S5.2PQVAL_17 EQ 117) OR (S5.2PQVAL_18 EQ 118) OR (S5.2PQVAL_19 EQ 119) OR (S5.2PQVAL_20 EQ 120) OR (S5.2PQVAL_21 EQ 121)) AND (S5.2PQVAL_22 EQ 122))) [S5.MICROQ2 = 'Si je pèse les pour et les contres, c´est beaucoup plus avantageux pour moi de bouger régulièrement. C´est la plus sage décision que je pourrais prendre dans ma vie.'];
IF (((S5.SUMVAL GE 200) AND (S5.SUMVAL LT 300) AND ((S5.QVAL1 EQ 101) OR (S5.QVAL2 EQ 102) OR (S5.QVAL3 EQ 103) OR (S5.QVAL4 EQ 104) OR (S5.QVAL5 EQ 105) OR (S5.QVAL6 EQ 106) OR (S5.QVAL7 EQ 107) OR (S5.QVAL8 EQ 108) OR (S5.QVAL9 EQ 109) OR (S5.QVAL_10 EQ 101.1) OR (S5.QVAL_11 EQ 101.2) OR (S5.QVAL_12 EQ 101.3) OR (S5.QVAL_13 EQ 101.4) OR (S5.QVAL_14 EQ 101.5) OR (S5.QVAL_15 EQ 101.6) OR (S5.QVAL_16 EQ 101.7) OR (S5.QVAL_17 EQ 101.8) OR (S5.QVAL_18 EQ 101.9) OR (S5.QVAL_19 EQ 101.19) OR (S5.QVAL_20 EQ 102.1) OR (S5.QVAL_21 EQ 102.2) OR (S5.QVAL_22 EQ 102.3)) AND (S5.QVAL_23 EQ 102.4)) OR (((S5.2PQVAL1 EQ 101) OR (S5.2PQVAL2 EQ 102) OR (S5.2PQVAL3 EQ 103) OR (S5.2PQVAL4 EQ 104) OR (S5.2PQVAL5 EQ 105) OR (S5.2PQVAL6 EQ 106) OR (S5.2PQVAL7 EQ 107) OR (S5.2PQVAL8 EQ 108) OR (S5.2PQVAL9 EQ 109) OR (S5.2PQVAL_10 EQ 110) OR (S5.2PQVAL_11 EQ 111) OR (S5.2PQVAL_12 EQ 112) OR (S5.2PQVAL_13 EQ 113) OR (S5.2PQVAL_14 EQ 114) OR (S5.2PQVAL_15 EQ 115) OR (S5.2PQVAL_16 EQ 116) OR (S5.2PQVAL_17 EQ 117) OR (S5.2PQVAL_18 EQ 118) OR

(S5.2PQVAL_19 EQ 119) OR (S5.2PQVAL_20 EQ 120) OR (S5.2PQVAL_21 EQ 121) OR (S5.2PQVAL_22 EQ 122)) AND (S5.2PQVAL_23 EQ 123))) [S5.MICROQ2 = 'J´évite toujours de reculer devant un défi. Bouger régulièrement est seulement un autre défi à relever. Je suis capable de faire ça.'];
IF (((S5.SUMVAL GE 200) AND (S5.SUMVAL LT 300) AND ((S5.QVAL1 EQ 101) OR (S5.QVAL2 EQ 102) OR (S5.QVAL3 EQ 103) OR (S5.QVAL4 EQ 104) OR (S5.QVAL5 EQ 105) OR (S5.QVAL6 EQ 106) OR (S5.QVAL7 EQ 107) OR (S5.QVAL8 EQ 108) OR (S5.QVAL9 EQ 109) OR (S5.QVAL_10 EQ 101.1) OR (S5.QVAL_11 EQ 101.2) OR (S5.QVAL_12 EQ 101.3) OR (S5.QVAL_13 EQ 101.4) OR (S5.QVAL_14 EQ 101.5) OR (S5.QVAL_15 EQ 101.6) OR (S5.QVAL_16 EQ 101.7) OR (S5.QVAL_17 EQ 101.8) OR (S5.QVAL_18 EQ 101.9) OR (S5.QVAL_19 EQ 101.19) OR (S5.QVAL_20 EQ 102.1) OR (S5.QVAL_21 EQ 102.2) OR (S5.QVAL_22 EQ 102.3) OR (S5.QVAL_23 EQ 102.4)) AND (S5.QVAL_24 EQ 102.5)) OR (((S5.2PQVAL1 EQ 101) OR (S5.2PQVAL2 EQ 102) OR (S5.2PQVAL3 EQ 103) OR (S5.2PQVAL4 EQ 104) OR (S5.2PQVAL5 EQ 105) OR (S5.2PQVAL6 EQ 106) OR (S5.2PQVAL7 EQ 107) OR (S5.2PQVAL8 EQ 108) OR (S5.2PQVAL9 EQ 109) OR (S5.2PQVAL_10 EQ 110) OR (S5.2PQVAL_11 EQ 111) OR (S5.2PQVAL_12 EQ 112) OR (S5.2PQVAL_13 EQ 113) OR (S5.2PQVAL_14 EQ 114) OR (S5.2PQVAL_15 EQ 115) OR (S5.2PQVAL_16 EQ 116) OR (S5.2PQVAL_17 EQ 117) OR (S5.2PQVAL_18 EQ 118) OR (S5.2PQVAL_19 EQ 119) OR (S5.2PQVAL_20 EQ 120) OR (S5.2PQVAL_21 EQ 121) OR (S5.2PQVAL_22 EQ 122) OR (S5.2PQVAL_23 EQ 123)) AND (S5.2PQVAL_24 EQ 124))) [S5.MICROQ2 = 'Je sais où je veux aller, et bouger régulièrement doit faire partie du chemin. Je vais trouver une façon d´y arriver je n´abandonnerai jamais.'];
IF (((S5.SUMVAL GE 200) AND (S5.SUMVAL LT 300) AND ((S5.QVAL1 EQ 101) OR (S5.QVAL2 EQ 102) OR (S5.QVAL3 EQ 103) OR (S5.QVAL4 EQ 104) OR (S5.QVAL5 EQ 105) OR (S5.QVAL6 EQ 106) OR (S5.QVAL7 EQ 107) OR (S5.QVAL8 EQ 108) OR (S5.QVAL9 EQ 109) OR (S5.QVAL_10 EQ 101.1) OR (S5.QVAL_11 EQ 101.2) OR (S5.QVAL_12 EQ 101.3) OR (S5.QVAL_13 EQ 101.4) OR (S5.QVAL_14 EQ 101.5) OR (S5.QVAL_15 EQ 101.6) OR (S5.QVAL_16 EQ 101.7) OR (S5.QVAL_17 EQ 101.8) OR (S5.QVAL_18 EQ 101.9) OR (S5.QVAL_19 EQ 101.19) OR (S5.QVAL_20 EQ 102.1) OR (S5.QVAL_21 EQ 102.2) OR (S5.QVAL_22 EQ 102.3) OR (S5.QVAL_23 EQ 102.4) OR (S5.QVAL_24 EQ 102.5)) AND (S5.QVAL_25 EQ 102.6)) OR (((S5.2PQVAL1 EQ 101) OR (S5.2PQVAL2 EQ 102) OR (S5.2PQVAL3 EQ 103) OR (S5.2PQVAL4 EQ 104) OR (S5.2PQVAL5 EQ 105) OR (S5.2PQVAL6 EQ 106) OR (S5.2PQVAL7 EQ 107) OR (S5.2PQVAL8 EQ 108) OR (S5.2PQVAL9 EQ 109) OR (S5.2PQVAL_10 EQ 110) OR (S5.2PQVAL_11 EQ 111) OR (S5.2PQVAL_12 EQ 112) OR (S5.2PQVAL_13 EQ 113) OR (S5.2PQVAL_14 EQ 114) OR (S5.2PQVAL_15 EQ 115) OR (S5.2PQVAL_16 EQ 116) OR (S5.2PQVAL_17 EQ 117) OR (S5.2PQVAL_18 EQ 118) OR (S5.2PQVAL_19 EQ 119) OR (S5.2PQVAL_20 EQ 120) OR (S5.2PQVAL_21 EQ 121) OR (S5.2PQVAL_22 EQ 122) OR (S5.2PQVAL_23 EQ 123) OR (S5.2PQVAL_24 EQ 124)) AND (S5.2PQVAL_25 EQ 125))) [S5.MICROQ2 = 'J´ai toujours été honnête envers moi et mes besoins. Bouger régulièrement serait très bon pour moi, c´est sûr. Je suis capable.'];
Codage pour la première séance de mise à jour (séance 4)
[S4.TOT_INTEN = S4.QINT1+S4.QINT2+S4.QINT3];
[S4.INTEN = S4.TOT_INTEN/3];
[S4.APINTENSE = S4.APINTENSEMIN*S4.APINTENSEX*2];
[S4.APMODEREE = S4.APMODEREEMIN*S4.APMODEREEX];
[S4.APLEGMETS = S4.APLEGEREMIN*S4.APLEGEREX*3.3];
[S4.APMODMETS = S4.APMODEREEMIN*S4.APMODEREEX*4];
[S4.APINTMETS = S4.APINTENSEMIN*S4.APINTENSEX*8];
[S4.APTOT = S4.APMODEREE + S4.APINTENSE];

[S4.METS = S4.APLEGMETS+S4.APINTMETS+S4.APINTMETS];
seance 8
AP formulas
[S8.APINTENSE = S8.APINTENSEMIN*S8.APINTENSEX*2];
[S8.APMODEREE = S8.APMODEREEMIN*S8.APMODEREEX];
[S8.APLEGMETS = S8.APLEGEREMIN*S8.APLEGEREX*3.3];
[S8.APMODMETS = S8.APMODEREEMIN*S8.APMODEREEX*3.3];
[S8.APINTMETS = S8.APINTENSEMIN*S8.APINTENSEX*8];
[S8.APTOT = S8.APMODEREE + S8.APINTENSE];
[S8.METS = S8.APLEGMETS+S8.APINTMETS+S8.APINTMETS];
INTENTION non complete s4
[S8.TOT_INTEN = S8.0QINT1+S8.0QINT2+S8.0QINT3];
[S8.INTEN = S8.TOT_INTEN/3];
INTENTION complete s4
[S8.1TOT_INTEN = S8.QINT1+S8.QINT2+S8.QINT3];
[S8.1INTEN = S8.1TOT_INTEN/3];
FORMULES S6
[S6.SUMAP =
S6.QACTIVITE1+S6.QACTIVITE2+S6.QACTIVITE3+S6.QACTIVITE4+S6.QACTIVITE5+S6.QACTIVITE6+S6.QACTIVITE7+S6.QACTIVITE8+S6.QACTIVITE9+S6.QACTIVITE_10+S6.QACTIVITE_11];
[S6.SUMBAR = S6.1BAR+S6.2BAR+S6.3BAR+S6.4BAR+S6.5BAR+S6.6BAR+S6.7BAR+S6.8BAR+S6.9BAR];
[S6.SUMSOL =
S6.P8QSOL1+S6.P8QSOL2+S6.P8QSOL3+S6.P8QSOL4+S6.P8QSOL5+S6.P8QSOL6+S6.P8QSOL7+S6.P8QSOL8+S6.P8QSOL9];
IF (S6.SUMAP GE 200) [ET = ' et '];
IF (S6.QAOPEN NE 0) [S6.TXTAPOP = 'votre [S6.QAOPEN]'];
IF (S6.QAOPEN NE 0) [S6.2TXTAPOP = ' et votre [S6.QAOPEN]'];
IF (S6.QACTIVITE1 EQ 101) [S6.AP1 = 'la marche rapide'];
IF ((S6.QACTIVITE1 NE 101) AND (S6.QACTIVITE2 EQ 102)) [S6.AP1 = 'la raquette à neige'];
IF ((S6.QACTIVITE1 NE 101) AND (S6.QACTIVITE2 NE 102) AND (S6.QACTIVITE3 EQ 103)) [S6.AP1 = 'le vélo'];
IF ((S6.QACTIVITE1 NE 101) AND (S6.QACTIVITE2 NE 102) AND (S6.QACTIVITE3 NE 103) AND (S6.QACTIVITE4 EQ 104)) [S6.AP1 = 'la natation'];
IF ((S6.QACTIVITE1 NE 101) AND (S6.QACTIVITE2 NE 102) AND (S6.QACTIVITE3 NE 103) AND (S6.QACTIVITE4 NE 104) AND (S6.QACTIVITE5 EQ 105)) [S6.AP1 = 'le patinage'];
IF ((S6.QACTIVITE1 NE 101) AND (S6.QACTIVITE2 NE 102) AND (S6.QACTIVITE3 NE 103) AND (S6.QACTIVITE4 NE 104) AND (S6.QACTIVITE5 NE 105) AND (S6.QACTIVITE6 EQ 106)) [S6.AP1 = 'le ski de fond'];
IF ((S6.QACTIVITE1 NE 101) AND (S6.QACTIVITE2 NE 102) AND (S6.QACTIVITE3 NE 103) AND (S6.QACTIVITE4 NE 104) AND (S6.QACTIVITE5 NE 105) AND (S6.QACTIVITE6 NE 106) AND (S6.QACTIVITE7 EQ 107)) [S6.AP1 = 'la course à pieds'];
IF ((S6.QACTIVITE1 NE 101) AND (S6.QACTIVITE2 NE 102) AND (S6.QACTIVITE3 NE 103) AND (S6.QACTIVITE4 NE 104) AND (S6.QACTIVITE5 NE 105) AND (S6.QACTIVITE6 NE 106) AND (S6.QACTIVITE7 NE 107) AND (S6.QACTIVITE8 EQ 108)) [S6.AP1 = 'le hockey'];

IF ((S6.QACTIVITE1 NE 101) AND (S6.QACTIVITE2 NE 102) AND (S6.QACTIVITE3 NE 103) AND (S6.QACTIVITE4 NE 104) AND (S6.QACTIVITE5 NE 105) AND (S6.QACTIVITE6 NE 106) AND (S6.QACTIVITE7 NE 107) AND (S6.QACTIVITE8 NE 108) AND (S6.QACTIVITE9 EQ 109)) [S6.AP1 = 'le basketball'];
IF ((S6.QACTIVITE1 NE 101) AND (S6.QACTIVITE2 NE 102) AND (S6.QACTIVITE3 NE 103) AND (S6.QACTIVITE4 NE 104) AND (S6.QACTIVITE5 NE 105) AND (S6.QACTIVITE6 NE 106) AND (S6.QACTIVITE7 NE 107) AND (S6.QACTIVITE8 NE 108) AND (S6.QACTIVITE9 NE 109) AND (S6.QACTIVITE_10 EQ 110)) [S6.AP1 = 'le soccer'];
IF ((S6.QACTIVITE1 NE 101) AND (S6.QACTIVITE2 NE 102) AND (S6.QACTIVITE3 NE 103) AND (S6.QACTIVITE4 NE 104) AND (S6.QACTIVITE5 NE 105) AND (S6.QACTIVITE6 NE 106) AND (S6.QACTIVITE7 NE 107) AND (S6.QACTIVITE8 NE 108) AND (S6.QACTIVITE9 NE 109) AND (S6.QACTIVITE_10 NE 110) AND (S6.QACTIVITE_11 EQ 111)) [S6.AP1 = 'le centre de conditionnement'];
IF ((S6.QACTIVITE1 NE 101) AND (S6.QACTIVITE2 NE 102) AND (S6.QACTIVITE3 NE 103) AND (S6.QACTIVITE4 NE 104) AND (S6.QACTIVITE5 NE 105) AND (S6.QACTIVITE6 NE 106) AND (S6.QACTIVITE7 NE 107) AND (S6.QACTIVITE8 NE 108) AND (S6.QACTIVITE9 NE 109) AND (S6.QACTIVITE_10 NE 110) AND (S6.QACTIVITE_11 NE 111) AND (S6.QACTIVITE_12 EQ 112)) [S6.AP1 = S6.QAOPEN];
IF ((S6.QACTIVITE1 EQ 101) AND (S6.QACTIVITE2 EQ 102)) [S6.AP2 = 'la raquette à neige'];
IF (((S6.QACTIVITE1 EQ 101) OR (S6.QACTIVITE2 EQ 102)) AND (S6.QACTIVITE3 EQ 103)) [S6.AP2 = 'le vélo'];
IF (((S6.QACTIVITE1 EQ 101) OR (S6.QACTIVITE2 EQ 102) OR (S6.QACTIVITE3 EQ 103)) AND (S6.QACTIVITE4 EQ 104)) [S6.AP2 = 'la natation'];
IF (((S6.QACTIVITE1 EQ 101) OR (S6.QACTIVITE2 EQ 102) OR (S6.QACTIVITE3 EQ 103) OR (S6.QACTIVITE4 EQ 104)) AND (S6.QACTIVITE5 EQ 105)) [S6.AP2 = 'le patinage'];
IF (((S6.QACTIVITE1 EQ 101) OR (S6.QACTIVITE2 EQ 102) OR (S6.QACTIVITE3 EQ 103) OR (S6.QACTIVITE4 EQ 104) OR (S6.QACTIVITE5 EQ 105)) AND (S6.QACTIVITE6 EQ 106)) [S6.AP2 = 'lle ski de fond'];
IF (((S6.QACTIVITE1 EQ 101) OR (S6.QACTIVITE2 EQ 102) OR (S6.QACTIVITE3 EQ 103) OR (S6.QACTIVITE4 EQ 104) OR (S6.QACTIVITE5 EQ 105) OR (S6.QACTIVITE6 EQ 106)) AND (S6.QACTIVITE7 EQ 107)) [S6.AP2 = 'la course à pieds'];
IF (((S6.QACTIVITE1 EQ 101) OR (S6.QACTIVITE2 EQ 102) OR (S6.QACTIVITE3 EQ 103) OR (S6.QACTIVITE4 EQ 104) OR (S6.QACTIVITE5 EQ 105) OR (S6.QACTIVITE6 EQ 106) OR (S6.QACTIVITE7 EQ 107)) AND (S6.QACTIVITE8 EQ 108)) [S6.AP2 = 'le hockey'];
IF (((S6.QACTIVITE1 EQ 101) OR (S6.QACTIVITE2 EQ 102) OR (S6.QACTIVITE3 EQ 103) OR (S6.QACTIVITE4 EQ 104) OR (S6.QACTIVITE5 EQ 105) OR (S6.QACTIVITE6 EQ 106) OR (S6.QACTIVITE7 EQ 107) OR (S6.QACTIVITE8 EQ 108)) AND (S6.QACTIVITE9 EQ 109)) [S6.AP2 = 'le basketball'];
IF (((S6.QACTIVITE1 EQ 101) OR (S6.QACTIVITE2 EQ 102) OR (S6.QACTIVITE3 EQ 103) OR (S6.QACTIVITE4 EQ 104) OR (S6.QACTIVITE5 EQ 105) OR (S6.QACTIVITE6 EQ 106) OR (S6.QACTIVITE7 EQ 107) OR (S6.QACTIVITE8 EQ 108) OR (S6.QACTIVITE9 EQ 109)) AND (S6.QACTIVITE_10 EQ 110)) [S6.AP2 = 'le soccer'];
IF (((S6.QACTIVITE1 EQ 101) OR (S6.QACTIVITE2 EQ 102) OR (S6.QACTIVITE3 EQ 103) OR (S6.QACTIVITE4 EQ 104) OR (S6.QACTIVITE5 EQ 105) OR (S6.QACTIVITE6 EQ 106) OR (S6.QACTIVITE7 EQ 107) OR (S6.QACTIVITE8 EQ 108) OR (S6.QACTIVITE9 EQ 109) OR (S6.QACTIVITE_10 EQ 110)) AND (S6.QACTIVITE_11 EQ 111)) [S6.AP2 = 'le centre de conditionnement'];
IF (S6.P7BARCHOICE EQ 101) [S6.BAR = 'Être trop fatigué ou fatiguée pour faire de l´activité physique'];
IF (S6.P7BARCHOICE EQ 102) [S6.BAR = 'Avoir beaucoup de choses à faire mis à part l´activité physique'];

IF (S6.P7BARCHOICE EQ 103) [S6.BAR = 'La température sera trop mauvaise pour faire de l´activité physique'];
IF (S6.P7BARCHOICE EQ 104) [S6.BAR = 'Ne pas avoir personne avec qui faire de l´activité physique'];
IF (S6.P7BARCHOICE EQ 105) [S6.BAR = 'Ne pas avoir accès à des emplacements pour faire de l´activité physique'];
IF (S6.P7BARCHOICE EQ 106) [S6.BAR = 'Ne pas avoir d´argent pour faire de l´activité physique'];
IF (S6.P7BARCHOICE EQ 107) [S6.BAR = 'Être trop gêné ou gênée, manquer de confiance pour aller faire de l´activité physique'];
IF (S6.P7BARCHOICE EQ 108) [S6.BAR = 'Avoir peur d´être insuffisamment en santé pour faire de l´activité physique'];
IF (S6.P7BARCHOICE EQ 109) [S6.BAR = 'Manquer de motivation'];
IF ((S6.P7BARCHOICE EQ 110) AND (S6.Q_BARANSWER NE 0)) [S6.BAR = S6.Q_BARANSWER];
IF (S6.SUMSOL EQ 101) [S6.SOL = 'Je vais faire de l´activité tôt dans la journée, en matinée ou à l´heure du midi.'];
IF (S6.SUMSOL EQ 102) [S6.SOL = 'J´essaie de garder en tête que si je fais de l´activité physique, j´aurai de plus en plus d´énergie dans la vie.'];
IF (S6.SUMSOL EQ 103) [S6.SOL = 'Je vais planifier des activités avec d´autres personnes qui me motiveront.'];
IF (S6.SUMSOL EQ 104) [S6.SOL = 'Je prends une collation pour me donner de l´énergie et j´y vais quand même.'];
IF (S6.SUMSOL EQ 201) [S6.SOL = 'Faire de l´activité physique par bloc de 10 minutes les journées où je n´ai pas de temps.'];
IF (S6.SUMSOL EQ 202) [S6.SOL = 'Je vais remplacer des activités où je suis assisE, télé, ordinateur ou autres, par des activités physiques.'];
IF (S6.SUMSOL EQ 203) [S6.SOL = 'Je vais me procurer un agenda et inscrire dans mon horaire les moments où je vais faire de l´activité physique.'];
IF (S6.SUMSOL EQ 204) [S6.SOL = 'Je vais prendre des pauses de travail active et faire des 10 minutes de marche.'];
IF (S6.SUMSOL EQ 205) [S6.SOL = 'Je vais me fixer des moments avec d´autres personnes qui me plaisent pour aller faire de l´activité physique.'];
IF (S6.SUMSOL EQ 301) [S6.SOL = 'Je vais me procurer l´équipement nécessaire pour en faire même quand il pleut, quand il neige ou quand il fait trop chaud.'];
IF (S6.SUMSOL EQ 302) [S6.SOL = 'Je vais faire de l'activité physique chez nous ou à l´intérieur dans un endroit que j´aurai choisi quand il fait moins beau.'];
IF (S6.SUMSOL EQ 303) [S6.SOL = 'Je vais me préparer une activité physique alternative au cas où il serait désagréable d´aller dehors.'];
IF (S6.SUMSOL EQ 304) [S6.SOL = 'Je vais me dresser une liste des activités que je serais prêtE à faire si la température est mauvaise.'];
IF (S6.SUMSOL EQ 401) [S6.SOL = 'Je vais me joindre à un groupe qui pratique l´activité physique que j´aime. Ex : la marche, l´aquaforme ou la natation.'];
IF (S6.SUMSOL EQ 402) [S6.SOL = 'Je vais inviter mes amis, ma famille ou mes collègues à faire de l´activité physique avec moi.'];

IF (S6.SUMSOL EQ 403) [S6.SOL = 'Je vais amener mon chien marcher avec moi.'];
IF (S6.SUMSOL EQ 404) [S6.SOL = 'Je vais participer dans des discussions en ligne sur des pages facebook : Diabète Québec et autres.'];
IF (S6.SUMSOL EQ 501) [S6.SOL = 'Je vais faire de la marche ou du vélo dehors, c´est simple et gratuit.'];
IF (S6.SUMSOL EQ 502) [S6.SOL = 'Je vais faire de l´activité physique dans ma maison comme du yoga, un programme sur DVD, vélo stationnaire, tapis roulant, etc.'];
IF (S6.SUMSOL EQ 503) [S6.SOL = 'Je vais aller voir au centre communautaire de mon quartier pour me donner des options.'];
IF (S6.SUMSOL EQ 504) [S6.SOL = 'Je vais discuter avec mon médecin, avec un ami ou un spécialiste de l´activité physique pour avoir des conseils.'];
IF (S6.SUMSOL EQ 601) [S6.SOL = 'Je vais trouver des activités physiques abordables et simples : marcher dehors, faire du vélo, nager à la piscine communautaire.'];
IF (S6.SUMSOL EQ 602) [S6.SOL = 'Je vais aller voir au centre communautaire de mon quartier pour me donner des options.'];
IF (S6.SUMSOL EQ 603) [S6.SOL = 'Je vais économiser pour m´acheter un DVD d´activité physique ou pour une autre activité physique que j´aimerais faire.'];
IF (S6.SUMSOL EQ 604) [S6.SOL = 'Je vais aller marcher avec ma famille, des amis ou des collègues.'];
IF (S6.SUMSOL EQ 701) [S6.SOL = 'Je vais choisir une activité physique simple comme la marche ou la bicyclette.'];
IF (S6.SUMSOL EQ 702) [S6.SOL = 'Je vais aller faire de l´activité physique avec des gens qui m´acceptent comme je suis, qui évitent de me juger.'];
IF (S6.SUMSOL EQ 703) [S6.SOL = 'Au départ, je vais faire de l´activité physique dans des endroits où je suis seul[LETTRE_E] pour prendre confiance.'];
IF (S6.SUMSOL EQ 704) [S6.SOL = 'Je vais éviter des endroits qui me gêne davantage comme les centres de conditionnement.'];
IF (S6.SUMSOL EQ 801) [S6.SOL = 'Je vais consulter mon médecin pour mettre au clair si je peux faire de l´activité physique.'];
IF (S6.SUMSOL EQ 802) [S6.SOL = 'Je vais consulter un spécialiste de l´activité physique pour qu´il me rassure et me conseille sur ce qui est sécuritaire pour moi.'];
IF (S6.SUMSOL EQ 803) [S6.SOL = 'Je vais commencer à petite dose. 10 à 20 minutes maximum d´activités physiques les jours où je suis actif, active.'];
IF (S6.SUMSOL EQ 804) [S6.SOL = 'Je vais faire de l´activité physique accompagnéE d´un spécialiste de l´activité physique.'];
IF (S6.SUMSOL EQ 805) [S6.SOL = 'Je vais faire de l´activité physique dans un groupe où un spécialiste de l´activité physique est présent.'];
IF (S6.SUMSOL EQ 901) [S6.SOL = 'Je vais signer un contrat papier personnel comme quoi je m´engage à être actif, active.'];
IF (S6.SUMSOL EQ 902) [S6.SOL = 'Je vais signer un contrat papier avec mes proches comme quoi je m´engage à être actif, active.'];
IF (S6.SUMSOL EQ 903) [S6.SOL = 'Je vais essayer de nouvelles activités physiques pour trouver celles qui me plaisent vraiment.'];

IF (S6.SUMSOL EQ 904) [S6.SOL = 'À chaque activité physique que je fais, je prends un temps pour me rappeler ce que cela va m´apporter de mieux dans ma vie.'];
IF (S6.SUMSOL EQ 905) [S6.SOL = 'Je vais faire de l´activité physique avec des gens que j´aime pour me motiver.'];
IF ((S6.SUMSOL EQ 999) AND (S6.P8QSOLOPEN NE 0)) [S6.SOL = S6.P8QSOLOPEN];
IF ((S6.P9QFEELH EQ 101) AND (SEXE EQ 1)) [S6.SENTIMENT = 'très fier'];
IF ((S6.P9QFEELH EQ 102) AND (SEXE EQ 1)) [S6.SENTIMENT = 'assez fier'];
IF ((S6.P9QFEELH EQ 103) AND (SEXE EQ 1)) [S6.SENTIMENT = 'un peu fier'];
IF ((S6.P9QFEELF EQ 101) AND (SEXE EQ 2)) [S6.SENTIMENT = 'très fière'];
IF ((S6.P9QFEELF EQ 102) AND (SEXE EQ 2)) [S6.SENTIMENT = 'assez fière'];
IF ((S6.P9QFEELF EQ 103) AND (SEXE EQ 2)) [S6.SENTIMENT = 'un peu fière'];
IF ((S6.P9QFEELF EQ 104) OR (S6.P9QFEELH EQ 104)) [S6.SENTIMENT = S6.P9QFEELOPEN];
PLAN DE LA SEMAINE 1
FORMULES P1
IF ((P1.QACTI12 EQ 112) AND (P1.QAOPEN NE 0)) [P1.QACTIVITE_12 = 112];
[P1.2SUMAP2 = P1.QACTIVITE1];
[P1.3SUMAP2 = P1.QACTIVITE1+P1.QACTIVITE2];
[P1.4SUMAP2 = P1.QACTIVITE1+P1.QACTIVITE2+P1.QACTIVITE3];
[P1.5SUMAP2 = P1.QACTIVITE1+P1.QACTIVITE2+P1.QACTIVITE3+P1.QACTIVITE4];
[P1.6SUMAP2 = P1.QACTIVITE1+P1.QACTIVITE2+P1.QACTIVITE3+P1.QACTIVITE4+P1.QACTIVITE5];
[P1.7SUMAP2 =
P1.QACTIVITE1+P1.QACTIVITE2+P1.QACTIVITE3+P1.QACTIVITE4+P1.QACTIVITE5+P1.QACTIVITE6];
[P1.8SUMAP2 =
P1.QACTIVITE1+P1.QACTIVITE2+P1.QACTIVITE3+P1.QACTIVITE4+P1.QACTIVITE5+P1.QACTIVITE6+P1.QACTIVITE7];
[P1.9SUMAP2 =
P1.QACTIVITE1+P1.QACTIVITE2+P1.QACTIVITE3+P1.QACTIVITE4+P1.QACTIVITE5+P1.QACTIVITE6+P1.QACTIVITE7+P1.QACTIVITE8];
[P1.10SUMAP2 =
P1.QACTIVITE1+P1.QACTIVITE2+P1.QACTIVITE3+P1.QACTIVITE4+P1.QACTIVITE5+P1.QACTIVITE6+P1.QACTIVITE7+P1.QACTIVITE8+P1.QACTIVITE9];
[P1.11SUMAP2 =
P1.QACTIVITE1+P1.QACTIVITE2+P1.QACTIVITE3+P1.QACTIVITE4+P1.QACTIVITE5+P1.QACTIVITE6+P1.QACTIVITE7+P1.QACTIVITE8+P1.QACTIVITE9+P1.QACTIVITE_10];
[P1.12SUMAP2 =
P1.QACTIVITE1+P1.QACTIVITE2+P1.QACTIVITE3+P1.QACTIVITE4+P1.QACTIVITE5+P1.QACTIVITE6+P1.QACTIVITE7+P1.QACTIVITE8+P1.QACTIVITE9+P1.QACTIVITE_10+P1.QACTIVITE_11];
[P1.3SUMAP3 = P1.QACTIVITE1+P1.QACTIVITE2];
[P1.4SUMAP3 = P1.QACTIVITE1+P1.QACTIVITE2+P1.QACTIVITE3];
[P1.5SUMAP3 = P1.QACTIVITE1+P1.QACTIVITE2+P1.QACTIVITE3+P1.QACTIVITE4];
[P1.6SUMAP3 = P1.QACTIVITE1+P1.QACTIVITE2+P1.QACTIVITE3+P1.QACTIVITE4+P1.QACTIVITE5];

```
[P1.7SUMAP3 =
P1.QACTIVITE1+P1.QACTIVITE2+P1.QACTIVITE3+P1.QACTIVITE4+P1.QACTIVITE5+P1.QACTIVITE6];
[P1.8SUMAP3 =
P1.QACTIVITE1+P1.QACTIVITE2+P1.QACTIVITE3+P1.QACTIVITE4+P1.QACTIVITE5+P1.QACTIVITE6+P1.QACTI
VITE7];
[P1.9SUMAP3 =
P1.QACTIVITE1+P1.QACTIVITE2+P1.QACTIVITE3+P1.QACTIVITE4+P1.QACTIVITE5+P1.QACTIVITE6+P1.QACTI
VITE7+P1.QACTIVITE8];
[P1.10SUMAP3 =
P1.QACTIVITE1+P1.QACTIVITE2+P1.QACTIVITE3+P1.QACTIVITE4+P1.QACTIVITE5+P1.QACTIVITE6+P1.QACTI
VITE7+P1.QACTIVITE8+P1.QACTIVITE9];
[P1.11SUMAP3 =
P1.QACTIVITE1+P1.QACTIVITE2+P1.QACTIVITE3+P1.QACTIVITE4+P1.QACTIVITE5+P1.QACTIVITE6+P1.QACTI
VITE7+P1.QACTIVITE8+P1.QACTIVITE9+P1.QACTIVITE_10];
[P1.12SUMAP3 =
P1.QACTIVITE1+P1.QACTIVITE2+P1.QACTIVITE3+P1.QACTIVITE4+P1.QACTIVITE5+P1.QACTIVITE6+P1.QACTI
VITE7+P1.QACTIVITE8+P1.QACTIVITE9+P1.QACTIVITE_10+P1.QACTIVITE_11];
[P1.SUMAP =
P1.QACTIVITE1+P1.QACTIVITE2+P1.QACTIVITE3+P1.QACTIVITE4+P1.QACTIVITE5+P1.QACTIVITE6+P1.QACTI
VITE7+P1.QACTIVITE8+P1.QACTIVITE9+P1.QACTIVITE_10+P1.QACTIVITE_11+P1.QACTIVITE_12];
[P1.SUMSOL =
P1.P8QSOL1+P1.P8QSOL2+P1.P8QSOL3+P1.P8QSOL4+P1.P8QSOL5+P1.P8QSOL6+P1.P8QSOL7+P1.P8QSOL8
+P1.P8QSOL9];
IF (P1.QACTIVITE1 EQ 101) [P1.AP1 = 'marche rapide'];
IF ((P1.QACTIVITE1 NE 101) AND (P1.QACTIVITE2 EQ 102)) [P1.AP1 = 'raquettes à neige'];
IF ((P1.QACTIVITE1 NE 101) AND (P1.QACTIVITE2 NE 102) AND (P1.QACTIVITE3 EQ 103)) [P1.AP1 = 'vélo'];
IF ((P1.QACTIVITE1 NE 101) AND (P1.QACTIVITE2 NE 102) AND (P1.QACTIVITE3 NE 103) AND
(P1.QACTIVITE4 EQ 104)) [P1.AP1 = 'natation'];
IF ((P1.QACTIVITE1 NE 101) AND (P1.QACTIVITE2 NE 102) AND (P1.QACTIVITE3 NE 103) AND
(P1.QACTIVITE4 NE 104) AND (P1.QACTIVITE5 EQ 105)) [P1.AP1 = 'ski de fond'];
IF ((P1.QACTIVITE1 NE 101) AND (P1.QACTIVITE2 NE 102) AND (P1.QACTIVITE3 NE 103) AND
(P1.QACTIVITE4 NE 104) AND (P1.QACTIVITE5 NE 105) AND (P1.QACTIVITE6 EQ 106)) [P1.AP1 = 'tennis'];
IF ((P1.QACTIVITE1 NE 101) AND (P1.QACTIVITE2 NE 102) AND (P1.QACTIVITE3 NE 103) AND
(P1.QACTIVITE4 NE 104) AND (P1.QACTIVITE5 NE 105) AND (P1.QACTIVITE6 NE 106) AND (P1.QACTIVITE7
EQ 107)) [P1.AP1 = 'course à pieds'];
IF ((P1.QACTIVITE1 NE 101) AND (P1.QACTIVITE2 NE 102) AND (P1.QACTIVITE3 NE 103) AND
(P1.QACTIVITE4 NE 104) AND (P1.QACTIVITE5 NE 105) AND (P1.QACTIVITE6 NE 106) AND (P1.QACTIVITE7
NE 107) AND (P1.QACTIVITE8 EQ 108)) [P1.AP1 = 'hockey'];
IF ((P1.QACTIVITE1 NE 101) AND (P1.QACTIVITE2 NE 102) AND (P1.QACTIVITE3 NE 103) AND
(P1.QACTIVITE4 NE 104) AND (P1.QACTIVITE5 NE 105) AND (P1.QACTIVITE6 NE 106) AND (P1.QACTIVITE7
NE 107) AND (P1.QACTIVITE8 NE 108) AND (P1.QACTIVITE9 EQ 109)) [P1.AP1 = 'basketball'];
```

IF ((P1.QACTIVITE1 NE 101) AND (P1.QACTIVITE2 NE 102) AND (P1.QACTIVITE3 NE 103) AND (P1.QACTIVITE4 NE 104) AND (P1.QACTIVITE5 NE 105) AND (P1.QACTIVITE6 NE 106) AND (P1.QACTIVITE7 NE 107) AND (P1.QACTIVITE8 NE 108) AND (P1.QACTIVITE9 NE 109) AND (P1.QACTIVITE_10 EQ 110)) [P1.AP1 = 'soccer'];
IF ((P1.QACTIVITE1 NE 101) AND (P1.QACTIVITE2 NE 102) AND (P1.QACTIVITE3 NE 103) AND (P1.QACTIVITE4 NE 104) AND (P1.QACTIVITE5 NE 105) AND (P1.QACTIVITE6 NE 106) AND (P1.QACTIVITE7 NE 107) AND (P1.QACTIVITE8 NE 108) AND (P1.QACTIVITE9 NE 109) AND (P1.QACTIVITE_10 NE 110) AND (P1.QACTIVITE_11 EQ 111)) [P1.AP1 = 'centre de conditionnement'];
IF ((P1.QACTIVITE1 NE 101) AND (P1.QACTIVITE2 NE 102) AND (P1.QACTIVITE3 NE 103) AND (P1.QACTIVITE4 NE 104) AND (P1.QACTIVITE5 NE 105) AND (P1.QACTIVITE6 NE 106) AND (P1.QACTIVITE7 NE 107) AND (P1.QACTIVITE8 NE 108) AND (P1.QACTIVITE9 NE 109) AND (P1.QACTIVITE_10 NE 110) AND (P1.QACTIVITE_11 NE 111) AND (P1.QACTIVITE_12 EQ 112)) [P1.AP1 = P1.QAOPEN];
IF ((P1.2SUMAP2 GE 100) AND (P1.2SUMAP2 LT 200) AND (P1.QACTIVITE2 EQ 102)) [P1.AP2 = 'raquettes à neige'];
IF ((P1.3SUMAP2 GE 100) AND (P1.3SUMAP2 LT 200) AND (P1.QACTIVITE3 EQ 103)) [P1.AP2 = 'vélo'];
IF ((P1.4SUMAP2 GE 100) AND (P1.4SUMAP2 LT 200) AND (P1.QACTIVITE4 EQ 104)) [P1.AP2 = 'natation'];
IF ((P1.5SUMAP2 GE 100) AND (P1.5SUMAP2 LT 200) AND (P1.QACTIVITE5 EQ 105)) [P1.AP2 = 'ski de fond'];
IF ((P1.6SUMAP2 GE 100) AND (P1.6SUMAP2 LT 200) AND (P1.QACTIVITE6 EQ 106)) [P1.AP2 = 'tennis'];
IF ((P1.7SUMAP2 GE 100) AND (P1.7SUMAP2 LT 200) AND (P1.QACTIVITE7 EQ 107)) [P1.AP2 = 'course à pieds'];
IF ((P1.8SUMAP2 GE 100) AND (P1.8SUMAP2 LT 200) AND (P1.QACTIVITE8 EQ 108)) [P1.AP2 = 'hockey'];
IF ((P1.9SUMAP2 GE 100) AND (P1.9SUMAP2 LT 200) AND (P1.QACTIVITE9 EQ 109)) [P1.AP2 = 'basketball'];
IF ((P1.10SUMAP2 GE 100) AND (P1.10SUMAP2 LT 200) AND (P1.QACTIVITE_10 EQ 110)) [P1.AP2 = 'soccer'];
IF ((P1.11SUMAP2 GE 100) AND (P1.11SUMAP2 LT 200) AND (P1.QACTIVITE_11 EQ 111)) [P1.AP2 = 'centre de conditionnement'];
IF ((P1.12SUMAP2 GE 100) AND (P1.12SUMAP2 LT 200) AND (P1.QACTIVITE_12 EQ 112)) [P1.AP2 = P1.QAOPEN];
IF ((P1.3SUMAP3 GE 200) AND (P1.3SUMAP3 LT 300) AND (P1.QACTIVITE3 EQ 103)) [P1.AP3 = 'vélo'];
IF ((P1.4SUMAP3 GE 200) AND (P1.4SUMAP3 LT 300) AND (P1.QACTIVITE4 EQ 104)) [P1.AP3 = 'natation'];
IF ((P1.5SUMAP3 GE 200) AND (P1.5SUMAP3 LT 300) AND (P1.QACTIVITE5 EQ 105)) [P1.AP3 = 'ski de fond'];
IF ((P1.6SUMAP3 GE 200) AND (P1.6SUMAP3 LT 300) AND (P1.QACTIVITE6 EQ 106)) [P1.AP3 = 'tennis'];
IF ((P1.7SUMAP3 GE 200) AND (P1.7SUMAP3 LT 300) AND (P1.QACTIVITE7 EQ 107)) [P1.AP3 = 'course à pieds'];
IF ((P1.8SUMAP3 GE 200) AND (P1.8SUMAP3 LT 300) AND (P1.QACTIVITE8 EQ 108)) [P1.AP3 = 'hockey'];
IF ((P1.9SUMAP3 GE 200) AND (P1.9SUMAP3 LT 300) AND (P1.QACTIVITE9 EQ 109)) [P1.AP3 = 'basketball'];
IF ((P1.10SUMAP3 GE 200) AND (P1.10SUMAP3 LT 300) AND (P1.QACTIVITE_10 EQ 110)) [P1.AP3 = 'soccer'];
IF ((P1.11SUMAP3 GE 200) AND (P1.11SUMAP3 LT 300) AND (P1.QACTIVITE_11 EQ 111)) [P1.AP3 = 'centre de conditionnement'];
IF ((P1.12SUMAP3 GE 200) AND (P1.12SUMAP3 LT 300) AND (P1.QACTIVITE_12 EQ 112)) [P1.AP3 = P1.QAOPEN];

ACTIVITÉ 1 DANS LE PLAN POUR CHAQUE JOUR DE LA SEMAINE
IF ((P1.QACTIVITE1 EQ 101) AND (P1.AP1JOUR EQ 1)) [P1.LUNDIAP1 = P1.AP1];
IF ((P1.QACTIVITE1 NE 101) AND (P1.QACTIVITE2 EQ 102) AND (P1.AP2JOUR EQ 1)) [P1.LUNDIAP1 = P1.AP1];
IF ((P1.QACTIVITE1 NE 101) AND (P1.QACTIVITE2 NE 102) AND (P1.QACTIVITE3 EQ 103) AND (P1.AP3JOUR EQ 1)) [P1.LUNDIAP1 = P1.AP1];
IF ((P1.QACTIVITE1 NE 101) AND (P1.QACTIVITE2 NE 102) AND (P1.QACTIVITE3 NE 103) AND (P1.QACTIVITE4 EQ 104) AND (P1.AP4JOUR EQ 1)) [P1.LUNDIAP1 = P1.AP1];
IF ((P1.QACTIVITE1 NE 101) AND (P1.QACTIVITE2 NE 102) AND (P1.QACTIVITE3 NE 103) AND (P1.QACTIVITE4 NE 104) AND (P1.QACTIVITE5 EQ 105) AND (P1.AP5JOUR EQ 1)) [P1.LUNDIAP1 = P1.AP1];
IF ((P1.QACTIVITE1 NE 101) AND (P1.QACTIVITE2 NE 102) AND (P1.QACTIVITE3 NE 103) AND (P1.QACTIVITE4 NE 104) AND (P1.QACTIVITE5 NE 105) AND (P1.QACTIVITE6 EQ 106) AND (P1.AP6JOUR EQ 1)) [P1.LUNDIAP1 = P1.AP1];
IF ((P1.QACTIVITE1 NE 101) AND (P1.QACTIVITE2 NE 102) AND (P1.QACTIVITE3 NE 103) AND (P1.QACTIVITE4 NE 104) AND (P1.QACTIVITE5 NE 105) AND (P1.QACTIVITE6 NE 106) AND (P1.QACTIVITE7 EQ 107) AND (P1.AP7JOUR EQ 1)) [P1.LUNDIAP1 = P1.AP1];
IF ((P1.QACTIVITE1 NE 101) AND (P1.QACTIVITE2 NE 102) AND (P1.QACTIVITE3 NE 103) AND (P1.QACTIVITE4 NE 104) AND (P1.QACTIVITE5 NE 105) AND (P1.QACTIVITE6 NE 106) AND (P1.QACTIVITE7 NE 107) AND (P1.QACTIVITE8 EQ 108) AND (P1.AP8JOUR EQ 1)) [P1.LUNDIAP1 = P1.AP1];
IF ((P1.QACTIVITE1 NE 101) AND (P1.QACTIVITE2 NE 102) AND (P1.QACTIVITE3 NE 103) AND (P1.QACTIVITE4 NE 104) AND (P1.QACTIVITE5 NE 105) AND (P1.QACTIVITE6 NE 106) AND (P1.QACTIVITE7 NE 107) AND (P1.QACTIVITE8 NE 108) AND (P1.QACTIVITE9 EQ 109) AND (P1.AP9JOUR EQ 1)) [P1.LUNDIAP1 = P1.AP1];
IF ((P1.QACTIVITE1 NE 101) AND (P1.QACTIVITE2 NE 102) AND (P1.QACTIVITE3 NE 103) AND (P1.QACTIVITE4 NE 104) AND (P1.QACTIVITE5 NE 105) AND (P1.QACTIVITE6 NE 106) AND (P1.QACTIVITE7 NE 107) AND (P1.QACTIVITE8 NE 108) AND (P1.QACTIVITE9 NE 109) AND (P1.QACTIVITE_10 EQ 110) AND (P1.AP10JOUR EQ 1)) [P1.LUNDIAP1 = P1.AP1];
IF ((P1.QACTIVITE1 NE 101) AND (P1.QACTIVITE2 NE 102) AND (P1.QACTIVITE3 NE 103) AND (P1.QACTIVITE4 NE 104) AND (P1.QACTIVITE5 NE 105) AND (P1.QACTIVITE6 NE 106) AND (P1.QACTIVITE7 NE 107) AND (P1.QACTIVITE8 NE 108) AND (P1.QACTIVITE9 NE 109) AND (P1.QACTIVITE_10 NE 110) AND (P1.QACTIVITE_11 EQ 111) AND (P1.AP11JOUR EQ 1)) [P1.LUNDIAP1 = P1.AP1];
IF ((P1.QACTIVITE1 NE 101) AND (P1.QACTIVITE2 NE 102) AND (P1.QACTIVITE3 NE 103) AND (P1.QACTIVITE4 NE 104) AND (P1.QACTIVITE5 NE 105) AND (P1.QACTIVITE6 NE 106) AND (P1.QACTIVITE7 NE 107) AND (P1.QACTIVITE8 NE 108) AND (P1.QACTIVITE9 NE 109) AND (P1.QACTIVITE_10 NE 110) AND (P1.QACTIVITE_11 NE 111) AND (P1.QACTIVITE_12 EQ 112) AND (P1.AP12JOUR EQ 1)) [P1.LUNDIAP1 = P1.AP1];
IF ((P1.QACTIVITE1 EQ 101) AND (P1.AP1JOUR EQ 2)) [P1.MARDIAP1 = P1.AP1];
IF ((P1.QACTIVITE1 NE 101) AND (P1.QACTIVITE2 EQ 102) AND (P1.AP2JOUR EQ 2)) [P1.MARDIAP1 = P1.AP1];
IF ((P1.QACTIVITE1 NE 101) AND (P1.QACTIVITE2 NE 102) AND (P1.QACTIVITE3 EQ 103) AND (P1.AP3JOUR EQ 2)) [P1.MARDIAP1 = P1.AP1];

IF ((P1.QACTIVITE1 NE 101) AND (P1.QACTIVITE2 NE 102) AND (P1.QACTIVITE3 NE 103) AND (P1.QACTIVITE4 EQ 104) AND (P1.AP4JOUR EQ 2)) [P1.MARDIAP1 = P1.AP1];
IF ((P1.QACTIVITE1 NE 101) AND (P1.QACTIVITE2 NE 102) AND (P1.QACTIVITE3 NE 103) AND (P1.QACTIVITE4 NE 104) AND (P1.QACTIVITE5 EQ 105) AND (P1.AP5JOUR EQ 2)) [P1.MARDIAP1 = P1.AP1];
IF ((P1.QACTIVITE1 NE 101) AND (P1.QACTIVITE2 NE 102) AND (P1.QACTIVITE3 NE 103) AND (P1.QACTIVITE4 NE 104) AND (P1.QACTIVITE5 NE 105) AND (P1.QACTIVITE6 EQ 106) AND (P1.AP6JOUR EQ 2)) [P1.MARDIAP1 = P1.AP1];
IF ((P1.QACTIVITE1 NE 101) AND (P1.QACTIVITE2 NE 102) AND (P1.QACTIVITE3 NE 103) AND (P1.QACTIVITE4 NE 104) AND (P1.QACTIVITE5 NE 105) AND (P1.QACTIVITE6 NE 106) AND (P1.QACTIVITE7 EQ 107) AND (P1.AP7JOUR EQ 2)) [P1.MARDIAP1 = P1.AP1];
IF ((P1.QACTIVITE1 NE 101) AND (P1.QACTIVITE2 NE 102) AND (P1.QACTIVITE3 NE 103) AND (P1.QACTIVITE4 NE 104) AND (P1.QACTIVITE5 NE 105) AND (P1.QACTIVITE6 NE 106) AND (P1.QACTIVITE7 NE 107) AND (P1.QACTIVITE8 EQ 108) AND (P1.AP8JOUR EQ 2)) [P1.MARDIAP1 = P1.AP1];
IF ((P1.QACTIVITE1 NE 101) AND (P1.QACTIVITE2 NE 102) AND (P1.QACTIVITE3 NE 103) AND (P1.QACTIVITE4 NE 104) AND (P1.QACTIVITE5 NE 105) AND (P1.QACTIVITE6 NE 106) AND (P1.QACTIVITE7 NE 107) AND (P1.QACTIVITE8 NE 108) AND (P1.QACTIVITE9 EQ 109) AND (P1.AP9JOUR EQ 2)) [P1.MARDIAP1 = P1.AP1];
IF ((P1.QACTIVITE1 NE 101) AND (P1.QACTIVITE2 NE 102) AND (P1.QACTIVITE3 NE 103) AND (P1.QACTIVITE4 NE 104) AND (P1.QACTIVITE5 NE 105) AND (P1.QACTIVITE6 NE 106) AND (P1.QACTIVITE7 NE 107) AND (P1.QACTIVITE8 NE 108) AND (P1.QACTIVITE9 NE 109) AND (P1.QACTIVITE_10 EQ 110) AND (P1.AP10JOUR EQ 2)) [P1.MARDIAP1 = P1.AP1];
IF ((P1.QACTIVITE1 NE 101) AND (P1.QACTIVITE2 NE 102) AND (P1.QACTIVITE3 NE 103) AND (P1.QACTIVITE4 NE 104) AND (P1.QACTIVITE5 NE 105) AND (P1.QACTIVITE6 NE 106) AND (P1.QACTIVITE7 NE 107) AND (P1.QACTIVITE8 NE 108) AND (P1.QACTIVITE9 NE 109) AND (P1.QACTIVITE_10 NE 110) AND (P1.QACTIVITE_11 EQ 111) AND (P1.AP11JOUR EQ 2)) [P1.MARDIAP1 = P1.AP1];
IF ((P1.QACTIVITE1 NE 101) AND (P1.QACTIVITE2 NE 102) AND (P1.QACTIVITE3 NE 103) AND (P1.QACTIVITE4 NE 104) AND (P1.QACTIVITE5 NE 105) AND (P1.QACTIVITE6 NE 106) AND (P1.QACTIVITE7 NE 107) AND (P1.QACTIVITE8 NE 108) AND (P1.QACTIVITE9 NE 109) AND (P1.QACTIVITE_10 NE 110) AND (P1.QACTIVITE_11 NE 111) AND (P1.QACTIVITE_12 EQ 112) AND (P1.AP12JOUR EQ 2)) [P1.MARDIAP1 = P1.AP1];
IF ((P1.QACTIVITE1 EQ 101) AND (P1.AP1JOUR EQ 3)) [P1.MERCREDIAP1 = P1.AP1];
IF ((P1.QACTIVITE1 NE 101) AND (P1.QACTIVITE2 EQ 102) AND (P1.AP2JOUR EQ 3)) [P1.MERCREDIAP1 = P1.AP1];
IF ((P1.QACTIVITE1 NE 101) AND (P1.QACTIVITE2 NE 102) AND (P1.QACTIVITE3 EQ 103) AND (P1.AP3JOUR EQ 3)) [P1.MERCREDIAP1 = P1.AP1];
IF ((P1.QACTIVITE1 NE 101) AND (P1.QACTIVITE2 NE 102) AND (P1.QACTIVITE3 NE 103) AND (P1.QACTIVITE4 EQ 104) AND (P1.AP4JOUR EQ 3)) [P1.MERCREDIAP1 = P1.AP1];
IF ((P1.QACTIVITE1 NE 101) AND (P1.QACTIVITE2 NE 102) AND (P1.QACTIVITE3 NE 103) AND (P1.QACTIVITE4 NE 104) AND (P1.QACTIVITE5 EQ 105) AND (P1.AP5JOUR EQ 3)) [P1.MERCREDIAP1 = P1.AP1];

IF ((P1.QACTIVITE1 NE 101) AND (P1.QACTIVITE2 NE 102) AND (P1.QACTIVITE3 NE 103) AND (P1.QACTIVITE4 NE 104) AND (P1.QACTIVITE5 NE 105) AND (P1.QACTIVITE6 EQ 106) AND (P1.AP6JOUR EQ 3)) [P1.MERCREDIAP1 = P1.AP1];
IF ((P1.QACTIVITE1 NE 101) AND (P1.QACTIVITE2 NE 102) AND (P1.QACTIVITE3 NE 103) AND (P1.QACTIVITE4 NE 104) AND (P1.QACTIVITE5 NE 105) AND (P1.QACTIVITE6 NE 106) AND (P1.QACTIVITE7 EQ 107) AND (P1.AP7JOUR EQ 3)) [P1.MERCREDIAP1 = P1.AP1];
IF ((P1.QACTIVITE1 NE 101) AND (P1.QACTIVITE2 NE 102) AND (P1.QACTIVITE3 NE 103) AND (P1.QACTIVITE4 NE 104) AND (P1.QACTIVITE5 NE 105) AND (P1.QACTIVITE6 NE 106) AND (P1.QACTIVITE7 NE 107) AND (P1.QACTIVITE8 EQ 108) AND (P1.AP8JOUR EQ 3)) [P1.MERCREDIAP1 = P1.AP1];
IF ((P1.QACTIVITE1 NE 101) AND (P1.QACTIVITE2 NE 102) AND (P1.QACTIVITE3 NE 103) AND (P1.QACTIVITE4 NE 104) AND (P1.QACTIVITE5 NE 105) AND (P1.QACTIVITE6 NE 106) AND (P1.QACTIVITE7 NE 107) AND (P1.QACTIVITE8 NE 108) AND (P1.QACTIVITE9 EQ 109) AND (P1.AP9JOUR EQ 3)) [P1.MERCREDIAP1 = P1.AP1];
IF ((P1.QACTIVITE1 NE 101) AND (P1.QACTIVITE2 NE 102) AND (P1.QACTIVITE3 NE 103) AND (P1.QACTIVITE4 NE 104) AND (P1.QACTIVITE5 NE 105) AND (P1.QACTIVITE6 NE 106) AND (P1.QACTIVITE7 NE 107) AND (P1.QACTIVITE8 NE 108) AND (P1.QACTIVITE9 NE 109) AND (P1.QACTIVITE_10 EQ 110) AND (P1.AP10JOUR EQ 3)) [P1.MERCREDIAP1 = P1.AP1];
IF ((P1.QACTIVITE1 NE 101) AND (P1.QACTIVITE2 NE 102) AND (P1.QACTIVITE3 NE 103) AND (P1.QACTIVITE4 NE 104) AND (P1.QACTIVITE5 NE 105) AND (P1.QACTIVITE6 NE 106) AND (P1.QACTIVITE7 NE 107) AND (P1.QACTIVITE8 NE 108) AND (P1.QACTIVITE9 NE 109) AND (P1.QACTIVITE_10 NE 110) AND (P1.QACTIVITE_11 EQ 111) AND (P1.AP11JOUR EQ 3)) [P1.MERCREDIAP1 = P1.AP1];
IF ((P1.QACTIVITE1 NE 101) AND (P1.QACTIVITE2 NE 102) AND (P1.QACTIVITE3 NE 103) AND (P1.QACTIVITE4 NE 104) AND (P1.QACTIVITE5 NE 105) AND (P1.QACTIVITE6 NE 106) AND (P1.QACTIVITE7 NE 107) AND (P1.QACTIVITE8 NE 108) AND (P1.QACTIVITE9 NE 109) AND (P1.QACTIVITE_10 NE 110) AND (P1.QACTIVITE_11 NE 111) AND (P1.QACTIVITE_12 EQ 112) AND (P1.AP12JOUR EQ 3)) [P1.MERCREDIAP1 = P1.AP1];
IF ((P1.QACTIVITE1 EQ 101) AND (P1.AP1JOUR EQ 4)) [P1.JEUDIAP1 = P1.AP1];
IF ((P1.QACTIVITE1 NE 101) AND (P1.QACTIVITE2 EQ 102) AND (P1.AP2JOUR EQ 4)) [P1.JEUDIAP1 = P1.AP1];
IF ((P1.QACTIVITE1 NE 101) AND (P1.QACTIVITE2 NE 102) AND (P1.QACTIVITE3 EQ 103) AND (P1.AP3JOUR EQ 4)) [P1.JEUDIAP1 = P1.AP1];
IF ((P1.QACTIVITE1 NE 101) AND (P1.QACTIVITE2 NE 102) AND (P1.QACTIVITE3 NE 103) AND (P1.QACTIVITE4 EQ 104) AND (P1.AP4JOUR EQ 4)) [P1.JEUDIAP1 = P1.AP1];
IF ((P1.QACTIVITE1 NE 101) AND (P1.QACTIVITE2 NE 102) AND (P1.QACTIVITE3 NE 103) AND (P1.QACTIVITE4 NE 104) AND (P1.QACTIVITE5 EQ 105) AND (P1.AP5JOUR EQ 4)) [P1.JEUDIAP1 = P1.AP1];
IF ((P1.QACTIVITE1 NE 101) AND (P1.QACTIVITE2 NE 102) AND (P1.QACTIVITE3 NE 103) AND (P1.QACTIVITE4 NE 104) AND (P1.QACTIVITE5 NE 105) AND (P1.QACTIVITE6 EQ 106) AND (P1.AP6JOUR EQ 4)) [P1.JEUDIAP1 = P1.AP1];
IF ((P1.QACTIVITE1 NE 101) AND (P1.QACTIVITE2 NE 102) AND (P1.QACTIVITE3 NE 103) AND (P1.QACTIVITE4 NE 104) AND (P1.QACTIVITE5 NE 105) AND (P1.QACTIVITE6 NE 106) AND (P1.QACTIVITE7 EQ 107) AND (P1.AP7JOUR EQ 4)) [P1.JEUDIAP1 = P1.AP1];

IF ((P1.QACTIVITE1 NE 101) AND (P1.QACTIVITE2 NE 102) AND (P1.QACTIVITE3 NE 103) AND (P1.QACTIVITE4 NE 104) AND (P1.QACTIVITE5 NE 105) AND (P1.QACTIVITE6 NE 106) AND (P1.QACTIVITE7 NE 107) AND (P1.QACTIVITE8 EQ 108) AND (P1.AP8JOUR EQ 4)) [P1.JEUDIAP1 = P1.AP1];
IF ((P1.QACTIVITE1 NE 101) AND (P1.QACTIVITE2 NE 102) AND (P1.QACTIVITE3 NE 103) AND (P1.QACTIVITE4 NE 104) AND (P1.QACTIVITE5 NE 105) AND (P1.QACTIVITE6 NE 106) AND (P1.QACTIVITE7 NE 107) AND (P1.QACTIVITE8 NE 108) AND (P1.QACTIVITE9 EQ 109) AND (P1.AP9JOUR EQ 4)) [P1.JEUDIAP1 = P1.AP1];
IF ((P1.QACTIVITE1 NE 101) AND (P1.QACTIVITE2 NE 102) AND (P1.QACTIVITE3 NE 103) AND (P1.QACTIVITE4 NE 104) AND (P1.QACTIVITE5 NE 105) AND (P1.QACTIVITE6 NE 106) AND (P1.QACTIVITE7 NE 107) AND (P1.QACTIVITE8 NE 108) AND (P1.QACTIVITE9 NE 109) AND (P1.QACTIVITE_10 EQ 110) AND (P1.AP10JOUR EQ 4)) [P1.JEUDIAP1 = P1.AP1];
IF ((P1.QACTIVITE1 NE 101) AND (P1.QACTIVITE2 NE 102) AND (P1.QACTIVITE3 NE 103) AND (P1.QACTIVITE4 NE 104) AND (P1.QACTIVITE5 NE 105) AND (P1.QACTIVITE6 NE 106) AND (P1.QACTIVITE7 NE 107) AND (P1.QACTIVITE8 NE 108) AND (P1.QACTIVITE9 NE 109) AND (P1.QACTIVITE_10 NE 110) AND (P1.QACTIVITE_11 EQ 111) AND (P1.AP11JOUR EQ 4)) [P1.JEUDIAP1 = P1.AP1];
IF ((P1.QACTIVITE1 NE 101) AND (P1.QACTIVITE2 NE 102) AND (P1.QACTIVITE3 NE 103) AND (P1.QACTIVITE4 NE 104) AND (P1.QACTIVITE5 NE 105) AND (P1.QACTIVITE6 NE 106) AND (P1.QACTIVITE7 NE 107) AND (P1.QACTIVITE8 NE 108) AND (P1.QACTIVITE9 NE 109) AND (P1.QACTIVITE_10 NE 110) AND (P1.QACTIVITE_11 NE 111) AND (P1.QACTIVITE_12 EQ 112) AND (P1.AP12JOUR EQ 4)) [P1.JEUDIAP1 = P1.AP1];
IF ((P1.QACTIVITE1 EQ 101) AND (P1.AP1JOUR EQ 5)) [P1.VENDREDIAP1 = P1.AP1];
IF ((P1.QACTIVITE1 NE 101) AND (P1.QACTIVITE2 EQ 102) AND (P1.AP2JOUR EQ 5)) [P1.VENDREDIAP1 = P1.AP1];
IF ((P1.QACTIVITE1 NE 101) AND (P1.QACTIVITE2 NE 102) AND (P1.QACTIVITE3 EQ 103) AND (P1.AP3JOUR EQ 5)) [P1.VENDREDIAP1 = P1.AP1];
IF ((P1.QACTIVITE1 NE 101) AND (P1.QACTIVITE2 NE 102) AND (P1.QACTIVITE3 NE 103) AND (P1.QACTIVITE4 EQ 104) AND (P1.AP4JOUR EQ 5)) [P1.VENDREDIAP1 = P1.AP1];
IF ((P1.QACTIVITE1 NE 101) AND (P1.QACTIVITE2 NE 102) AND (P1.QACTIVITE3 NE 103) AND (P1.QACTIVITE4 NE 104) AND (P1.QACTIVITE5 EQ 105) AND (P1.AP5JOUR EQ 5)) [P1.VENDREDIAP1 = P1.AP1];
IF ((P1.QACTIVITE1 NE 101) AND (P1.QACTIVITE2 NE 102) AND (P1.QACTIVITE3 NE 103) AND (P1.QACTIVITE4 NE 104) AND (P1.QACTIVITE5 NE 105) AND (P1.QACTIVITE6 EQ 106) AND (P1.AP6JOUR EQ 5)) [P1.VENDREDIAP1 = P1.AP1];
IF ((P1.QACTIVITE1 NE 101) AND (P1.QACTIVITE2 NE 102) AND (P1.QACTIVITE3 NE 103) AND (P1.QACTIVITE4 NE 104) AND (P1.QACTIVITE5 NE 105) AND (P1.QACTIVITE6 NE 106) AND (P1.QACTIVITE7 EQ 107) AND (P1.AP7JOUR EQ 5)) [P1.VENDREDIAP1 = P1.AP1];
IF ((P1.QACTIVITE1 NE 101) AND (P1.QACTIVITE2 NE 102) AND (P1.QACTIVITE3 NE 103) AND (P1.QACTIVITE4 NE 104) AND (P1.QACTIVITE5 NE 105) AND (P1.QACTIVITE6 NE 106) AND (P1.QACTIVITE7 NE 107) AND (P1.QACTIVITE8 EQ 108) AND (P1.AP8JOUR EQ 5)) [P1.VENDREDIAP1 = P1.AP1];
IF ((P1.QACTIVITE1 NE 101) AND (P1.QACTIVITE2 NE 102) AND (P1.QACTIVITE3 NE 103) AND (P1.QACTIVITE4 NE 104) AND (P1.QACTIVITE5 NE 105) AND (P1.QACTIVITE6 NE 106) AND (P1.QACTIVITE7

NE 107) AND (P1.QACTIVITE8 NE 108) AND (P1.QACTIVITE9 EQ 109) AND (P1.AP9JOUR EQ 5)) [P1.VENDREDIAP1 = P1.AP1];
IF ((P1.QACTIVITE1 NE 101) AND (P1.QACTIVITE2 NE 102) AND (P1.QACTIVITE3 NE 103) AND (P1.QACTIVITE4 NE 104) AND (P1.QACTIVITE5 NE 105) AND (P1.QACTIVITE6 NE 106) AND (P1.QACTIVITE7 NE 107) AND (P1.QACTIVITE8 NE 108) AND (P1.QACTIVITE9 NE 109) AND (P1.QACTIVITE_10 EQ 110) AND (P1.AP10JOUR EQ 5)) [P1.VENDREDIAP1 = P1.AP1];
IF ((P1.QACTIVITE1 NE 101) AND (P1.QACTIVITE2 NE 102) AND (P1.QACTIVITE3 NE 103) AND (P1.QACTIVITE4 NE 104) AND (P1.QACTIVITE5 NE 105) AND (P1.QACTIVITE6 NE 106) AND (P1.QACTIVITE7 NE 107) AND (P1.QACTIVITE8 NE 108) AND (P1.QACTIVITE9 NE 109) AND (P1.QACTIVITE_10 NE 110) AND (P1.QACTIVITE_11 EQ 111) AND (P1.AP11JOUR EQ 5)) [P1.VENDREDIAP1 = P1.AP1];
IF ((P1.QACTIVITE1 NE 101) AND (P1.QACTIVITE2 NE 102) AND (P1.QACTIVITE3 NE 103) AND (P1.QACTIVITE4 NE 104) AND (P1.QACTIVITE5 NE 105) AND (P1.QACTIVITE6 NE 106) AND (P1.QACTIVITE7 NE 107) AND (P1.QACTIVITE8 NE 108) AND (P1.QACTIVITE9 NE 109) AND (P1.QACTIVITE_10 NE 110) AND (P1.QACTIVITE_11 NE 111) AND (P1.QACTIVITE_12 EQ 112) AND (P1.AP12JOUR EQ 5)) [P1.VENDREDIAP1 = P1.AP1];
IF ((P1.QACTIVITE1 EQ 101) AND (P1.AP1JOUR EQ 6)) [P1.SAMEDIAP1 = P1.AP1];
IF ((P1.QACTIVITE1 NE 101) AND (P1.QACTIVITE2 EQ 102) AND (P1.AP2JOUR EQ 6)) [P1.SAMEDIAP1 = P1.AP1];
IF ((P1.QACTIVITE1 NE 101) AND (P1.QACTIVITE2 NE 102) AND (P1.QACTIVITE3 EQ 103) AND (P1.AP3JOUR EQ 6)) [P1.SAMEDIAP1 = P1.AP1];
IF ((P1.QACTIVITE1 NE 101) AND (P1.QACTIVITE2 NE 102) AND (P1.QACTIVITE3 NE 103) AND (P1.QACTIVITE4 EQ 104) AND (P1.AP4JOUR EQ 6)) [P1.SAMEDIAP1 = P1.AP1];
IF ((P1.QACTIVITE1 NE 101) AND (P1.QACTIVITE2 NE 102) AND (P1.QACTIVITE3 NE 103) AND (P1.QACTIVITE4 NE 104) AND (P1.QACTIVITE5 EQ 105) AND (P1.AP5JOUR EQ 6)) [P1.SAMEDIAP1 = P1.AP1];
IF ((P1.QACTIVITE1 NE 101) AND (P1.QACTIVITE2 NE 102) AND (P1.QACTIVITE3 NE 103) AND (P1.QACTIVITE4 NE 104) AND (P1.QACTIVITE5 NE 105) AND (P1.QACTIVITE6 EQ 106) AND (P1.AP6JOUR EQ 6)) [P1.SAMEDIAP1 = P1.AP1];
IF ((P1.QACTIVITE1 NE 101) AND (P1.QACTIVITE2 NE 102) AND (P1.QACTIVITE3 NE 103) AND (P1.QACTIVITE4 NE 104) AND (P1.QACTIVITE5 NE 105) AND (P1.QACTIVITE6 NE 106) AND (P1.QACTIVITE7 EQ 107) AND (P1.AP7JOUR EQ 6)) [P1.SAMEDIAP1 = P1.AP1];
IF ((P1.QACTIVITE1 NE 101) AND (P1.QACTIVITE2 NE 102) AND (P1.QACTIVITE3 NE 103) AND (P1.QACTIVITE4 NE 104) AND (P1.QACTIVITE5 NE 105) AND (P1.QACTIVITE6 NE 106) AND (P1.QACTIVITE7 NE 107) AND (P1.QACTIVITE8 EQ 108) AND (P1.AP8JOUR EQ 6)) [P1.SAMEDIAP1 = P1.AP1];
IF ((P1.QACTIVITE1 NE 101) AND (P1.QACTIVITE2 NE 102) AND (P1.QACTIVITE3 NE 103) AND (P1.QACTIVITE4 NE 104) AND (P1.QACTIVITE5 NE 105) AND (P1.QACTIVITE6 NE 106) AND (P1.QACTIVITE7 NE 107) AND (P1.QACTIVITE8 NE 108) AND (P1.QACTIVITE9 EQ 109) AND (P1.AP9JOUR EQ 6)) [P1.SAMEDIAP1 = P1.AP1];
IF ((P1.QACTIVITE1 NE 101) AND (P1.QACTIVITE2 NE 102) AND (P1.QACTIVITE3 NE 103) AND (P1.QACTIVITE4 NE 104) AND (P1.QACTIVITE5 NE 105) AND (P1.QACTIVITE6 NE 106) AND (P1.QACTIVITE7 NE 107) AND (P1.QACTIVITE8 NE 108) AND (P1.QACTIVITE9 NE 109) AND (P1.QACTIVITE_10 EQ 110) AND (P1.AP10JOUR EQ 6)) [P1.SAMEDIAP1 = P1.AP1];

IF ((P1.QACTIVITE1 NE 101) AND (P1.QACTIVITE2 NE 102) AND (P1.QACTIVITE3 NE 103) AND (P1.QACTIVITE4 NE 104) AND (P1.QACTIVITE5 NE 105) AND (P1.QACTIVITE6 NE 106) AND (P1.QACTIVITE7 NE 107) AND (P1.QACTIVITE8 NE 108) AND (P1.QACTIVITE9 NE 109) AND (P1.QACTIVITE_10 NE 110) AND (P1.QACTIVITE_11 EQ 111) AND (P1.AP11JOUR EQ 6)) [P1.SAMEDIAP1 = P1.AP1];
IF ((P1.QACTIVITE1 NE 101) AND (P1.QACTIVITE2 NE 102) AND (P1.QACTIVITE3 NE 103) AND (P1.QACTIVITE4 NE 104) AND (P1.QACTIVITE5 NE 105) AND (P1.QACTIVITE6 NE 106) AND (P1.QACTIVITE7 NE 107) AND (P1.QACTIVITE8 NE 108) AND (P1.QACTIVITE9 NE 109) AND (P1.QACTIVITE_10 NE 110) AND (P1.QACTIVITE_11 NE 111) AND (P1.QACTIVITE_12 EQ 112) AND (P1.AP12JOUR EQ 6)) [P1.SAMEDIAP1 = P1.AP1];
IF ((P1.QACTIVITE1 EQ 101) AND (P1.AP1JOUR EQ 7)) [P1.DIMANCHEAP1 = P1.AP1];
IF ((P1.QACTIVITE1 NE 101) AND (P1.QACTIVITE2 EQ 102) AND (P1.AP2JOUR EQ 7)) [P1.DIMANCHEAP1 = P1.AP1];
IF ((P1.QACTIVITE1 NE 101) AND (P1.QACTIVITE2 NE 102) AND (P1.QACTIVITE3 EQ 103) AND (P1.AP3JOUR EQ 7)) [P1.DIMANCHEAP1 = P1.AP1];
IF ((P1.QACTIVITE1 NE 101) AND (P1.QACTIVITE2 NE 102) AND (P1.QACTIVITE3 NE 103) AND (P1.QACTIVITE4 EQ 104) AND (P1.AP4JOUR EQ 7)) [P1.DIMANCHEAP1 = P1.AP1];
IF ((P1.QACTIVITE1 NE 101) AND (P1.QACTIVITE2 NE 102) AND (P1.QACTIVITE3 NE 103) AND (P1.QACTIVITE4 NE 104) AND (P1.QACTIVITE5 EQ 105) AND (P1.AP5JOUR EQ 7)) [P1.DIMANCHEAP1 = P1.AP1];
IF ((P1.QACTIVITE1 NE 101) AND (P1.QACTIVITE2 NE 102) AND (P1.QACTIVITE3 NE 103) AND (P1.QACTIVITE4 NE 104) AND (P1.QACTIVITE5 NE 105) AND (P1.QACTIVITE6 EQ 106) AND (P1.AP6JOUR EQ 7)) [P1.DIMANCHEAP1 = P1.AP1];
IF ((P1.QACTIVITE1 NE 101) AND (P1.QACTIVITE2 NE 102) AND (P1.QACTIVITE3 NE 103) AND (P1.QACTIVITE4 NE 104) AND (P1.QACTIVITE5 NE 105) AND (P1.QACTIVITE6 NE 106) AND (P1.QACTIVITE7 EQ 107) AND (P1.AP7JOUR EQ 7)) [P1.DIMANCHEAP1 = P1.AP1];
IF ((P1.QACTIVITE1 NE 101) AND (P1.QACTIVITE2 NE 102) AND (P1.QACTIVITE3 NE 103) AND (P1.QACTIVITE4 NE 104) AND (P1.QACTIVITE5 NE 105) AND (P1.QACTIVITE6 NE 106) AND (P1.QACTIVITE7 NE 107) AND (P1.QACTIVITE8 EQ 108) AND (P1.AP8JOUR EQ 7)) [P1.DIMANCHEAP1 = P1.AP1];
IF ((P1.QACTIVITE1 NE 101) AND (P1.QACTIVITE2 NE 102) AND (P1.QACTIVITE3 NE 103) AND (P1.QACTIVITE4 NE 104) AND (P1.QACTIVITE5 NE 105) AND (P1.QACTIVITE6 NE 106) AND (P1.QACTIVITE7 NE 107) AND (P1.QACTIVITE8 NE 108) AND (P1.QACTIVITE9 EQ 109) AND (P1.AP9JOUR EQ 7)) [P1.DIMANCHEAP1 = P1.AP1];
IF ((P1.QACTIVITE1 NE 101) AND (P1.QACTIVITE2 NE 102) AND (P1.QACTIVITE3 NE 103) AND (P1.QACTIVITE4 NE 104) AND (P1.QACTIVITE5 NE 105) AND (P1.QACTIVITE6 NE 106) AND (P1.QACTIVITE7 NE 107) AND (P1.QACTIVITE8 NE 108) AND (P1.QACTIVITE9 NE 109) AND (P1.QACTIVITE_10 EQ 110) AND (P1.AP10JOUR EQ 7)) [P1.DIMANCHEAP1 = P1.AP1];
IF ((P1.QACTIVITE1 NE 101) AND (P1.QACTIVITE2 NE 102) AND (P1.QACTIVITE3 NE 103) AND (P1.QACTIVITE4 NE 104) AND (P1.QACTIVITE5 NE 105) AND (P1.QACTIVITE6 NE 106) AND (P1.QACTIVITE7 NE 107) AND (P1.QACTIVITE8 NE 108) AND (P1.QACTIVITE9 NE 109) AND (P1.QACTIVITE_10 NE 110) AND (P1.QACTIVITE_11 EQ 111) AND (P1.AP11JOUR EQ 7)) [P1.DIMANCHEAP1 = P1.AP1];
IF ((P1.QACTIVITE1 NE 101) AND (P1.QACTIVITE2 NE 102) AND (P1.QACTIVITE3 NE 103) AND (P1.QACTIVITE4 NE 104) AND (P1.QACTIVITE5 NE 105) AND (P1.QACTIVITE6 NE 106) AND (P1.QACTIVITE7

NE 107) AND (P1.QACTIVITE8 NE 108) AND (P1.QACTIVITE9 NE 109) AND (P1.QACTIVITE_10 NE 110) AND (P1.QACTIVITE_11 NE 111) AND (P1.QACTIVITE_12 EQ 112) AND (P1.AP12JOUR EQ 7)) [P1.DIMANCHEAP1 = P1.AP1];
ACTIVITÉ 2 DANS LE PLAN POUR CHAQUE JOUR DE LA SEMAINE
IF ((P1.2SUMAP2 GE 100) AND (P1.2SUMAP2 LT 200) AND (P1.QACTIVITE2 EQ 102) AND (P1.AP2JOUR EQ 1)) [P1.LUNDIAP2 = P1.AP2];
IF ((P1.3SUMAP2 GE 100) AND (P1.3SUMAP2 LT 200) AND (P1.QACTIVITE3 EQ 103) AND (P1.AP3JOUR EQ 1)) [P1.LUNDIAP2 = P1.AP2];
IF ((P1.4SUMAP2 GE 100) AND (P1.4SUMAP2 LT 200) AND (P1.QACTIVITE4 EQ 104) AND (P1.AP4JOUR EQ 1)) [P1.LUNDIAP2 = P1.AP2];
IF ((P1.5SUMAP2 GE 100) AND (P1.5SUMAP2 LT 200) AND (P1.QACTIVITE5 EQ 105) AND (P1.AP5JOUR EQ 1)) [P1.LUNDIAP2 = P1.AP2];
IF ((P1.6SUMAP2 GE 100) AND (P1.6SUMAP2 LT 200) AND (P1.QACTIVITE6 EQ 106) AND (P1.AP6JOUR EQ 1)) [P1.LUNDIAP2 = P1.AP2];
IF ((P1.7SUMAP2 GE 100) AND (P1.7SUMAP2 LT 200) AND (P1.QACTIVITE7 EQ 107) AND (P1.AP7JOUR EQ 1)) [P1.LUNDIAP2 = P1.AP2];
IF ((P1.8SUMAP2 GE 100) AND (P1.8SUMAP2 LT 200) AND (P1.QACTIVITE8 EQ 108) AND (P1.AP8JOUR EQ 1)) [P1.LUNDIAP2 = P1.AP2];
IF ((P1.9SUMAP2 GE 100) AND (P1.9SUMAP2 LT 200) AND (P1.QACTIVITE9 EQ 109) AND (P1.AP9JOUR EQ 1)) [P1.LUNDIAP2 = P1.AP2];
IF ((P1.10SUMAP2 GE 100) AND (P1.10SUMAP2 LT 200) AND (P1.QACTIVITE_10 EQ 110) AND (P1.AP10JOUR EQ 1)) [P1.LUNDIAP2 = P1.AP2];
IF ((P1.11SUMAP2 GE 100) AND (P1.11SUMAP2 LT 200) AND (P1.QACTIVITE_11 EQ 111) AND (P1.AP11JOUR EQ 1)) [P1.LUNDIAP2 = P1.AP2];
IF ((P1.12SUMAP2 GE 100) AND (P1.12SUMAP2 LT 200) AND (P1.QACTIVITE_12 EQ 112) AND (P1.AP12JOUR EQ 1)) [P1.LUNDIAP2 = P1.AP2];
IF ((P1.2SUMAP2 GE 100) AND (P1.2SUMAP2 LT 200) AND (P1.QACTIVITE2 EQ 102) AND (P1.AP2JOUR EQ 2)) [P1.MARDIAP2 = P1.AP2];
IF ((P1.3SUMAP2 GE 100) AND (P1.3SUMAP2 LT 200) AND (P1.QACTIVITE3 EQ 103) AND (P1.AP3JOUR EQ 2)) [P1.MARDIAP2 = P1.AP2];
IF ((P1.4SUMAP2 GE 100) AND (P1.4SUMAP2 LT 200) AND (P1.QACTIVITE4 EQ 104) AND (P1.AP4JOUR EQ 2)) [P1.MARDIAP2 = P1.AP2];
IF ((P1.5SUMAP2 GE 100) AND (P1.5SUMAP2 LT 200) AND (P1.QACTIVITE5 EQ 105) AND (P1.AP5JOUR EQ 2)) [P1.MARDIAP2 = P1.AP2];
IF ((P1.6SUMAP2 GE 100) AND (P1.6SUMAP2 LT 200) AND (P1.QACTIVITE6 EQ 106) AND (P1.AP6JOUR EQ 2)) [P1.MARDIAP2 = P1.AP2];
IF ((P1.7SUMAP2 GE 100) AND (P1.7SUMAP2 LT 200) AND (P1.QACTIVITE7 EQ 107) AND (P1.AP7JOUR EQ 2)) [P1.MARDIAP2 = P1.AP2];
IF ((P1.8SUMAP2 GE 100) AND (P1.8SUMAP2 LT 200) AND (P1.QACTIVITE8 EQ 108) AND (P1.AP8JOUR EQ 2)) [P1.MARDIAP2 = P1.AP2];
IF ((P1.9SUMAP2 GE 100) AND (P1.9SUMAP2 LT 200) AND (P1.QACTIVITE9 EQ 109) AND (P1.AP9JOUR EQ 2)) [P1.MARDIAP2 = P1.AP2];

IF ((P1.10SUMAP2 GE 100) AND (P1.10SUMAP2 LT 200) AND (P1.QACTIVITE_10 EQ 110) AND (P1.AP10JOUR EQ 2)) [P1.MARDIAP2 = P1.AP2];
IF ((P1.11SUMAP2 GE 100) AND (P1.11SUMAP2 LT 200) AND (P1.QACTIVITE_11 EQ 111) AND (P1.AP11JOUR EQ 2)) [P1.MARDIAP2 = P1.AP2];
IF ((P1.12SUMAP2 GE 100) AND (P1.12SUMAP2 LT 200) AND (P1.QACTIVITE_12 EQ 112) AND (P1.AP12JOUR EQ 2)) [P1.MARDIAP2 = P1.AP2];
IF ((P1.2SUMAP2 GE 100) AND (P1.2SUMAP2 LT 200) AND (P1.QACTIVITE2 EQ 102) AND (P1.AP2JOUR EQ 3)) [P1.MERCREDIAP2 = P1.AP2];
IF ((P1.3SUMAP2 GE 100) AND (P1.3SUMAP2 LT 200) AND (P1.QACTIVITE3 EQ 103) AND (P1.AP3JOUR EQ 3)) [P1.MERCREDIAP2 = P1.AP2];
IF ((P1.4SUMAP2 GE 100) AND (P1.4SUMAP2 LT 200) AND (P1.QACTIVITE4 EQ 104) AND (P1.AP4JOUR EQ 3)) [P1.MERCREDIAP2 = P1.AP2];
IF ((P1.5SUMAP2 GE 100) AND (P1.5SUMAP2 LT 200) AND (P1.QACTIVITE5 EQ 105) AND (P1.AP5JOUR EQ 3)) [P1.MERCREDIAP2 = P1.AP2];
IF ((P1.6SUMAP2 GE 100) AND (P1.6SUMAP2 LT 200) AND (P1.QACTIVITE6 EQ 106) AND (P1.AP6JOUR EQ 3)) [P1.MERCREDIAP2 = P1.AP2];
IF ((P1.7SUMAP2 GE 100) AND (P1.7SUMAP2 LT 200) AND (P1.QACTIVITE7 EQ 107) AND (P1.AP7JOUR EQ 3)) [P1.MERCREDIAP2 = P1.AP2];
IF ((P1.8SUMAP2 GE 100) AND (P1.8SUMAP2 LT 200) AND (P1.QACTIVITE8 EQ 108) AND (P1.AP8JOUR EQ 3)) [P1.MERCREDIAP2 = P1.AP2];
IF ((P1.9SUMAP2 GE 100) AND (P1.9SUMAP2 LT 200) AND (P1.QACTIVITE9 EQ 109) AND (P1.AP9JOUR EQ 3)) [P1.MERCREDIAP2 = P1.AP2];
IF ((P1.10SUMAP2 GE 100) AND (P1.10SUMAP2 LT 200) AND (P1.QACTIVITE_10 EQ 110) AND (P1.AP10JOUR EQ 3)) [P1.MERCREDIAP2 = P1.AP2];
IF ((P1.11SUMAP2 GE 100) AND (P1.11SUMAP2 LT 200) AND (P1.QACTIVITE_11 EQ 111) AND (P1.AP11JOUR EQ 3)) [P1.MERCREDIAP2 = P1.AP2];
IF ((P1.12SUMAP2 GE 100) AND (P1.12SUMAP2 LT 200) AND (P1.QACTIVITE_12 EQ 112) AND (P1.AP12JOUR EQ 3)) [P1.MERCREDIAP2 = P1.AP2];
IF ((P1.2SUMAP2 GE 100) AND (P1.2SUMAP2 LT 200) AND (P1.QACTIVITE2 EQ 102) AND (P1.AP2JOUR EQ 4)) [P1.JEUDIAP2 = P1.AP2];
IF ((P1.3SUMAP2 GE 100) AND (P1.3SUMAP2 LT 200) AND (P1.QACTIVITE3 EQ 103) AND (P1.AP3JOUR EQ 4)) [P1.JEUDIAP2 = P1.AP2];
IF ((P1.4SUMAP2 GE 100) AND (P1.4SUMAP2 LT 200) AND (P1.QACTIVITE4 EQ 104) AND (P1.AP4JOUR EQ 4)) [P1.JEUDIAP2 = P1.AP2];
IF ((P1.5SUMAP2 GE 100) AND (P1.5SUMAP2 LT 200) AND (P1.QACTIVITE5 EQ 105) AND (P1.AP5JOUR EQ 4)) [P1.JEUDIAP2 = P1.AP2];
IF ((P1.6SUMAP2 GE 100) AND (P1.6SUMAP2 LT 200) AND (P1.QACTIVITE6 EQ 106) AND (P1.AP6JOUR EQ 4)) [P1.JEUDIAP2 = P1.AP2];
IF ((P1.7SUMAP2 GE 100) AND (P1.7SUMAP2 LT 200) AND (P1.QACTIVITE7 EQ 107) AND (P1.AP7JOUR EQ 4)) [P1.JEUDIAP2 = P1.AP2];
IF ((P1.8SUMAP2 GE 100) AND (P1.8SUMAP2 LT 200) AND (P1.QACTIVITE8 EQ 108) AND (P1.AP8JOUR EQ 4)) [P1.JEUDIAP2 = P1.AP2];

IF ((P1.9SUMAP2 GE 100) AND (P1.9SUMAP2 LT 200) AND (P1.QACTIVITE9 EQ 109) AND (P1.AP9JOUR EQ 4)) [P1.JEUDIAP2 = P1.AP2];
IF ((P1.10SUMAP2 GE 100) AND (P1.10SUMAP2 LT 200) AND (P1.QACTIVITE_10 EQ 110) AND (P1.AP10JOUR EQ 4)) [P1.JEUDIAP2 = P1.AP2];
IF ((P1.11SUMAP2 GE 100) AND (P1.11SUMAP2 LT 200) AND (P1.QACTIVITE_11 EQ 111) AND (P1.AP11JOUR EQ 4)) [P1.JEUDIAP2 = P1.AP2];
IF ((P1.12SUMAP2 GE 100) AND (P1.12SUMAP2 LT 200) AND (P1.QACTIVITE_12 EQ 112) AND (P1.AP12JOUR EQ 4)) [P1.JEUDIAP2 = P1.AP2];
IF ((P1.2SUMAP2 GE 100) AND (P1.2SUMAP2 LT 200) AND (P1.QACTIVITE2 EQ 102) AND (P1.AP2JOUR EQ 5)) [P1.VENDREDIAP2 = P1.AP2];
IF ((P1.3SUMAP2 GE 100) AND (P1.3SUMAP2 LT 200) AND (P1.QACTIVITE3 EQ 103) AND (P1.AP3JOUR EQ 5)) [P1.VENDREDIAP2 = P1.AP2];
IF ((P1.4SUMAP2 GE 100) AND (P1.4SUMAP2 LT 200) AND (P1.QACTIVITE4 EQ 104) AND (P1.AP4JOUR EQ 5)) [P1.VENDREDIAP2 = P1.AP2];
IF ((P1.5SUMAP2 GE 100) AND (P1.5SUMAP2 LT 200) AND (P1.QACTIVITE5 EQ 105) AND (P1.AP5JOUR EQ 5)) [P1.VENDREDIAP2 = P1.AP2];
IF ((P1.6SUMAP2 GE 100) AND (P1.6SUMAP2 LT 200) AND (P1.QACTIVITE6 EQ 106) AND (P1.AP6JOUR EQ 5)) [P1.VENDREDIAP2 = P1.AP2];
IF ((P1.7SUMAP2 GE 100) AND (P1.7SUMAP2 LT 200) AND (P1.QACTIVITE7 EQ 107) AND (P1.AP7JOUR EQ 5)) [P1.VENDREDIAP2 = P1.AP2];
IF ((P1.8SUMAP2 GE 100) AND (P1.8SUMAP2 LT 200) AND (P1.QACTIVITE8 EQ 108) AND (P1.AP8JOUR EQ 5)) [P1.VENDREDIAP2 = P1.AP2];
IF ((P1.9SUMAP2 GE 100) AND (P1.9SUMAP2 LT 200) AND (P1.QACTIVITE9 EQ 109) AND (P1.AP9JOUR EQ 5)) [P1.VENDREDIAP2 = P1.AP2];
IF ((P1.10SUMAP2 GE 100) AND (P1.10SUMAP2 LT 200) AND (P1.QACTIVITE_10 EQ 110) AND (P1.AP10JOUR EQ 5)) [P1.VENDREDIAP2 = P1.AP2];
IF ((P1.11SUMAP2 GE 100) AND (P1.11SUMAP2 LT 200) AND (P1.QACTIVITE_11 EQ 111) AND (P1.AP11JOUR EQ 5)) [P1.VENDREDIAP2 = P1.AP2];
IF ((P1.12SUMAP2 GE 100) AND (P1.12SUMAP2 LT 200) AND (P1.QACTIVITE_12 EQ 112) AND (P1.AP12JOUR EQ 5)) [P1.VENDREDIAP2 = P1.AP2];
IF ((P1.2SUMAP2 GE 100) AND (P1.2SUMAP2 LT 200) AND (P1.QACTIVITE2 EQ 102) AND (P1.AP2JOUR EQ 6)) [P1.SAMEDIAP2 = P1.AP2];
IF ((P1.3SUMAP2 GE 100) AND (P1.3SUMAP2 LT 200) AND (P1.QACTIVITE3 EQ 103) AND (P1.AP3JOUR EQ 6)) [P1.SAMEDIAP2 = P1.AP2];
IF ((P1.4SUMAP2 GE 100) AND (P1.4SUMAP2 LT 200) AND (P1.QACTIVITE4 EQ 104) AND (P1.AP4JOUR EQ 6)) [P1.SAMEDIAP2 = P1.AP2];
IF ((P1.5SUMAP2 GE 100) AND (P1.5SUMAP2 LT 200) AND (P1.QACTIVITE5 EQ 105) AND (P1.AP5JOUR EQ 6)) [P1.SAMEDIAP2 = P1.AP2];
IF ((P1.6SUMAP2 GE 100) AND (P1.6SUMAP2 LT 200) AND (P1.QACTIVITE6 EQ 106) AND (P1.AP6JOUR EQ 6)) [P1.SAMEDIAP2 = P1.AP2];
IF ((P1.7SUMAP2 GE 100) AND (P1.7SUMAP2 LT 200) AND (P1.QACTIVITE7 EQ 107) AND (P1.AP7JOUR EQ 6)) [P1.SAMEDIAP2 = P1.AP2];

IF ((P1.8SUMAP2 GE 100) AND (P1.8SUMAP2 LT 200) AND (P1.QACTIVITE8 EQ 108) AND (P1.AP8JOUR EQ 6)) [P1.SAMEDIAP2 = P1.AP2];
IF ((P1.9SUMAP2 GE 100) AND (P1.9SUMAP2 LT 200) AND (P1.QACTIVITE9 EQ 109) AND (P1.AP9JOUR EQ 6)) [P1.SAMEDIAP2 = P1.AP2];
IF ((P1.10SUMAP2 GE 100) AND (P1.10SUMAP2 LT 200) AND (P1.QACTIVITE_10 EQ 110) AND (P1.AP10JOUR EQ 6)) [P1.SAMEDIAP2 = P1.AP2];
IF ((P1.11SUMAP2 GE 100) AND (P1.11SUMAP2 LT 200) AND (P1.QACTIVITE_11 EQ 111) AND (P1.AP11JOUR EQ 6)) [P1.SAMEDIAP2 = P1.AP2];
IF ((P1.12SUMAP2 GE 100) AND (P1.12SUMAP2 LT 200) AND (P1.QACTIVITE_12 EQ 112) AND (P1.AP12JOUR EQ 6)) [P1.SAMEDIAP2 = P1.AP2];
IF ((P1.2SUMAP2 GE 100) AND (P1.2SUMAP2 LT 200) AND (P1.QACTIVITE2 EQ 102) AND (P1.AP2JOUR EQ 7)) [P1.DIMANCHEAP2 = P1.AP2];
IF ((P1.3SUMAP2 GE 100) AND (P1.3SUMAP2 LT 200) AND (P1.QACTIVITE3 EQ 103) AND (P1.AP3JOUR EQ 7)) [P1.DIMANCHEAP2 = P1.AP2];
IF ((P1.4SUMAP2 GE 100) AND (P1.4SUMAP2 LT 200) AND (P1.QACTIVITE4 EQ 104) AND (P1.AP4JOUR EQ 7)) [P1.DIMANCHEAP2 = P1.AP2];
IF ((P1.5SUMAP2 GE 100) AND (P1.5SUMAP2 LT 200) AND (P1.QACTIVITE5 EQ 105) AND (P1.AP5JOUR EQ 7)) [P1.DIMANCHEAP2 = P1.AP2];
IF ((P1.6SUMAP2 GE 100) AND (P1.6SUMAP2 LT 200) AND (P1.QACTIVITE6 EQ 106) AND (P1.AP6JOUR EQ 7)) [P1.DIMANCHEAP2 = P1.AP2];
IF ((P1.7SUMAP2 GE 100) AND (P1.7SUMAP2 LT 200) AND (P1.QACTIVITE7 EQ 107) AND (P1.AP7JOUR EQ 7)) [P1.DIMANCHEAP2 = P1.AP2];
IF ((P1.8SUMAP2 GE 100) AND (P1.8SUMAP2 LT 200) AND (P1.QACTIVITE8 EQ 108) AND (P1.AP8JOUR EQ 7)) [P1.DIMANCHEAP2 = P1.AP2];
IF ((P1.9SUMAP2 GE 100) AND (P1.9SUMAP2 LT 200) AND (P1.QACTIVITE9 EQ 109) AND (P1.AP9JOUR EQ 7)) [P1.DIMANCHEAP2 = P1.AP2];
IF ((P1.10SUMAP2 GE 100) AND (P1.10SUMAP2 LT 200) AND (P1.QACTIVITE_10 EQ 110) AND (P1.AP10JOUR EQ 7)) [P1.DIMANCHEAP2 = P1.AP2];
IF ((P1.11SUMAP2 GE 100) AND (P1.11SUMAP2 LT 200) AND (P1.QACTIVITE_11 EQ 111) AND (P1.AP11JOUR EQ 7)) [P1.DIMANCHEAP2 = P1.AP2];
IF ((P1.12SUMAP2 GE 100) AND (P1.12SUMAP2 LT 200) AND (P1.QACTIVITE_12 EQ 112) AND (P1.AP12JOUR EQ 7)) [P1.DIMANCHEAP2 = P1.AP2];
ACTIVITÉ 3 DANS LE PLAN POUR CHAQUE JOUR DE LA SEMAINE
IF ((P1.3SUMAP3 GE 200) AND (P1.3SUMAP3 LT 300) AND (P1.QACTIVITE3 EQ 103) AND (P1.AP3JOUR EQ 1)) [P1.LUNDIAP3 = P1.AP3];
IF ((P1.4SUMAP3 GE 200) AND (P1.4SUMAP3 LT 300) AND (P1.QACTIVITE4 EQ 104) AND (P1.AP4JOUR EQ 1)) [P1.LUNDIAP3 = P1.AP3];
IF ((P1.5SUMAP3 GE 200) AND (P1.5SUMAP3 LT 300) AND (P1.QACTIVITE5 EQ 105) AND (P1.AP5JOUR EQ 1)) [P1.LUNDIAP3 = P1.AP3];
IF ((P1.6SUMAP3 GE 200) AND (P1.6SUMAP3 LT 300) AND (P1.QACTIVITE6 EQ 106) AND (P1.AP6JOUR EQ 1)) [P1.LUNDIAP3 = P1.AP3];

IF ((P1.7SUMAP3 GE 200) AND (P1.7SUMAP3 LT 300) AND (P1.QACTIVITE7 EQ 107) AND (P1.AP7JOUR EQ 1)) [P1.LUNDIAP3 = P1.AP3];
IF ((P1.8SUMAP3 GE 200) AND (P1.8SUMAP3 LT 300) AND (P1.QACTIVITE8 EQ 108) AND (P1.AP8JOUR EQ 1)) [P1.LUNDIAP3 = P1.AP3];
IF ((P1.9SUMAP3 GE 200) AND (P1.9SUMAP3 LT 300) AND (P1.QACTIVITE9 EQ 109) AND (P1.AP9JOUR EQ 1)) [P1.LUNDIAP3 = P1.AP3];
IF ((P1.10SUMAP3 GE 200) AND (P1.10SUMAP3 LT 300) AND (P1.QACTIVITE_10 EQ 110) AND (P1.AP10JOUR EQ 1)) [P1.LUNDIAP3 = P1.AP3];
IF ((P1.11SUMAP3 GE 200) AND (P1.11SUMAP3 LT 300) AND (P1.QACTIVITE_11 EQ 111) AND (P1.AP11JOUR EQ 1)) [P1.LUNDIAP3 = P1.AP3];
IF ((P1.12SUMAP3 GE 200) AND (P1.12SUMAP3 LT 300) AND (P1.QACTIVITE_12 EQ 112) AND (P1.AP12JOUR EQ 1)) [P1.LUNDIAP3 = P1.AP3];
IF ((P1.3SUMAP3 GE 200) AND (P1.3SUMAP3 LT 300) AND (P1.QACTIVITE3 EQ 103) AND (P1.AP3JOUR EQ 2)) [P1.MARDIAP3 = P1.AP3];
IF ((P1.4SUMAP3 GE 200) AND (P1.4SUMAP3 LT 300) AND (P1.QACTIVITE4 EQ 104) AND (P1.AP4JOUR EQ 2)) [P1.MARDIAP3 = P1.AP3];
IF ((P1.5SUMAP3 GE 200) AND (P1.5SUMAP3 LT 300) AND (P1.QACTIVITE5 EQ 105) AND (P1.AP5JOUR EQ 2)) [P1.MARDIAP3 = P1.AP3];
IF ((P1.6SUMAP3 GE 200) AND (P1.6SUMAP3 LT 300) AND (P1.QACTIVITE6 EQ 106) AND (P1.AP6JOUR EQ 2)) [P1.MARDIAP3 = P1.AP3];
IF ((P1.7SUMAP3 GE 200) AND (P1.7SUMAP3 LT 300) AND (P1.QACTIVITE7 EQ 107) AND (P1.AP7JOUR EQ 2)) [P1.MARDIAP3 = P1.AP3];
IF ((P1.8SUMAP3 GE 200) AND (P1.8SUMAP3 LT 300) AND (P1.QACTIVITE8 EQ 108) AND (P1.AP8JOUR EQ 2)) [P1.MARDIAP3 = P1.AP3];
IF ((P1.9SUMAP3 GE 200) AND (P1.9SUMAP3 LT 300) AND (P1.QACTIVITE9 EQ 109) AND (P1.AP9JOUR EQ 2)) [P1.MARDIAP3 = P1.AP3];
IF ((P1.10SUMAP3 GE 200) AND (P1.10SUMAP3 LT 300) AND (P1.QACTIVITE_10 EQ 110) AND (P1.AP10JOUR EQ 2)) [P1.MARDIAP3 = P1.AP3];
IF ((P1.11SUMAP3 GE 200) AND (P1.11SUMAP3 LT 300) AND (P1.QACTIVITE_11 EQ 111) AND (P1.AP11JOUR EQ 2)) [P1.MARDIAP3 = P1.AP3];
IF ((P1.12SUMAP3 GE 200) AND (P1.12SUMAP3 LT 300) AND (P1.QACTIVITE_12 EQ 112) AND (P1.AP12JOUR EQ 2)) [P1.MARDIAP3 = P1.AP3];
IF ((P1.3SUMAP3 GE 200) AND (P1.3SUMAP3 LT 300) AND (P1.QACTIVITE3 EQ 103) AND (P1.AP3JOUR EQ 3)) [P1.MERCREDIAP3 = P1.AP3];
IF ((P1.4SUMAP3 GE 200) AND (P1.4SUMAP3 LT 300) AND (P1.QACTIVITE4 EQ 104) AND (P1.AP4JOUR EQ 3)) [P1.MERCREDIAP3 = P1.AP3];
IF ((P1.5SUMAP3 GE 200) AND (P1.5SUMAP3 LT 300) AND (P1.QACTIVITE5 EQ 105) AND (P1.AP5JOUR EQ 3)) [P1.MERCREDIAP3 = P1.AP3];
IF ((P1.6SUMAP3 GE 200) AND (P1.6SUMAP3 LT 300) AND (P1.QACTIVITE6 EQ 106) AND (P1.AP6JOUR EQ 3)) [P1.MERCREDIAP3 = P1.AP3];
IF ((P1.7SUMAP3 GE 200) AND (P1.7SUMAP3 LT 300) AND (P1.QACTIVITE7 EQ 107) AND (P1.AP7JOUR EQ 3)) [P1.MERCREDIAP3 = P1.AP3];

IF ((P1.8SUMAP3 GE 200) AND (P1.8SUMAP3 LT 300) AND (P1.QACTIVITE8 EQ 108) AND (P1.AP8JOUR EQ 3)) [P1.MERCREDIAP3 = P1.AP3];
IF ((P1.9SUMAP3 GE 200) AND (P1.9SUMAP3 LT 300) AND (P1.QACTIVITE9 EQ 109) AND (P1.AP9JOUR EQ 3)) [P1.MERCREDIAP3 = P1.AP3];
IF ((P1.10SUMAP3 GE 200) AND (P1.10SUMAP3 LT 300) AND (P1.QACTIVITE_10 EQ 110) AND (P1.AP10JOUR EQ 3)) [P1.MERCREDIAP3 = P1.AP3];
IF ((P1.11SUMAP3 GE 200) AND (P1.11SUMAP3 LT 300) AND (P1.QACTIVITE_11 EQ 111) AND (P1.AP11JOUR EQ 3)) [P1.MERCREDIAP3 = P1.AP3];
IF ((P1.12SUMAP3 GE 200) AND (P1.12SUMAP3 LT 300) AND (P1.QACTIVITE_12 EQ 112) AND (P1.AP12JOUR EQ 3)) [P1.MERCREDIAP3 = P1.AP3];
IF ((P1.3SUMAP3 GE 200) AND (P1.3SUMAP3 LT 300) AND (P1.QACTIVITE3 EQ 103) AND (P1.AP3JOUR EQ 4)) [P1.JEUDIAP3 = P1.AP3];
IF ((P1.4SUMAP3 GE 200) AND (P1.4SUMAP3 LT 300) AND (P1.QACTIVITE4 EQ 104) AND (P1.AP4JOUR EQ 4)) [P1.JEUDIAP3 = P1.AP3];
IF ((P1.5SUMAP3 GE 200) AND (P1.5SUMAP3 LT 300) AND (P1.QACTIVITE5 EQ 105) AND (P1.AP5JOUR EQ 4)) [P1.JEUDIAP3 = P1.AP3];
IF ((P1.6SUMAP3 GE 200) AND (P1.6SUMAP3 LT 300) AND (P1.QACTIVITE6 EQ 106) AND (P1.AP6JOUR EQ 4)) [P1.JEUDIAP3 = P1.AP3];
IF ((P1.7SUMAP3 GE 200) AND (P1.7SUMAP3 LT 300) AND (P1.QACTIVITE7 EQ 107) AND (P1.AP7JOUR EQ 4)) [P1.JEUDIAP3 = P1.AP3];
IF ((P1.8SUMAP3 GE 200) AND (P1.8SUMAP3 LT 300) AND (P1.QACTIVITE8 EQ 108) AND (P1.AP8JOUR EQ 4)) [P1.JEUDIAP3 = P1.AP3];
IF ((P1.9SUMAP3 GE 200) AND (P1.9SUMAP3 LT 300) AND (P1.QACTIVITE9 EQ 109) AND (P1.AP9JOUR EQ 4)) [P1.JEUDIAP3 = P1.AP3];
IF ((P1.10SUMAP3 GE 200) AND (P1.10SUMAP3 LT 300) AND (P1.QACTIVITE_10 EQ 110) AND (P1.AP10JOUR EQ 4)) [P1.JEUDIAP3 = P1.AP3];
IF ((P1.11SUMAP3 GE 200) AND (P1.11SUMAP3 LT 300) AND (P1.QACTIVITE_11 EQ 111) AND (P1.AP11JOUR EQ 4)) [P1.JEUDIAP3 = P1.AP3];
IF ((P1.12SUMAP3 GE 200) AND (P1.12SUMAP3 LT 300) AND (P1.QACTIVITE_12 EQ 112) AND (P1.AP12JOUR EQ 4)) [P1.JEUDIAP3 = P1.AP3];
IF ((P1.3SUMAP3 GE 200) AND (P1.3SUMAP3 LT 300) AND (P1.QACTIVITE3 EQ 103) AND (P1.AP3JOUR EQ 5)) [P1.VENDREDIAP3 = P1.AP3];
IF ((P1.4SUMAP3 GE 200) AND (P1.4SUMAP3 LT 300) AND (P1.QACTIVITE4 EQ 104) AND (P1.AP4JOUR EQ 5)) [P1.VENDREDIAP3 = P1.AP3];
IF ((P1.5SUMAP3 GE 200) AND (P1.5SUMAP3 LT 300) AND (P1.QACTIVITE5 EQ 105) AND (P1.AP5JOUR EQ 5)) [P1.VENDREDIAP3 = P1.AP3];
IF ((P1.6SUMAP3 GE 200) AND (P1.6SUMAP3 LT 300) AND (P1.QACTIVITE6 EQ 106) AND (P1.AP6JOUR EQ 5)) [P1.VENDREDIAP3 = P1.AP3];
IF ((P1.7SUMAP3 GE 200) AND (P1.7SUMAP3 LT 300) AND (P1.QACTIVITE7 EQ 107) AND (P1.AP7JOUR EQ 5)) [P1.VENDREDIAP3 = P1.AP3];
IF ((P1.8SUMAP3 GE 200) AND (P1.8SUMAP3 LT 300) AND (P1.QACTIVITE8 EQ 108) AND (P1.AP8JOUR EQ 5)) [P1.VENDREDIAP3 = P1.AP3];

IF ((P1.9SUMAP3 GE 200) AND (P1.9SUMAP3 LT 300) AND (P1.QACTIVITE9 EQ 109) AND (P1.AP9JOUR EQ 5)) [P1.VENDREDIAP3 = P1.AP3];
IF ((P1.10SUMAP3 GE 200) AND (P1.10SUMAP3 LT 300) AND (P1.QACTIVITE_10 EQ 110) AND (P1.AP10JOUR EQ 5)) [P1.VENDREDIAP3 = P1.AP3];
IF ((P1.11SUMAP3 GE 200) AND (P1.11SUMAP3 LT 300) AND (P1.QACTIVITE_11 EQ 111) AND (P1.AP11JOUR EQ 5)) [P1.VENDREDIAP3 = P1.AP3];
IF ((P1.12SUMAP3 GE 200) AND (P1.12SUMAP3 LT 300) AND (P1.QACTIVITE_12 EQ 112) AND (P1.AP12JOUR EQ 5)) [P1.VENDREDIAP3 = P1.AP3];
IF ((P1.3SUMAP3 GE 200) AND (P1.3SUMAP3 LT 300) AND (P1.QACTIVITE3 EQ 103) AND (P1.AP3JOUR EQ 6)) [P1.SAMEDIAP3 = P1.AP3];
IF ((P1.4SUMAP3 GE 200) AND (P1.4SUMAP3 LT 300) AND (P1.QACTIVITE4 EQ 104) AND (P1.AP4JOUR EQ 6)) [P1.SAMEDIAP3 = P1.AP3];
IF ((P1.5SUMAP3 GE 200) AND (P1.5SUMAP3 LT 300) AND (P1.QACTIVITE5 EQ 105) AND (P1.AP5JOUR EQ 6)) [P1.SAMEDIAP3 = P1.AP3];
IF ((P1.6SUMAP3 GE 200) AND (P1.6SUMAP3 LT 300) AND (P1.QACTIVITE6 EQ 106) AND (P1.AP6JOUR EQ 6)) [P1.SAMEDIAP3 = P1.AP3];
IF ((P1.7SUMAP3 GE 200) AND (P1.7SUMAP3 LT 300) AND (P1.QACTIVITE7 EQ 107) AND (P1.AP7JOUR EQ 6)) [P1.SAMEDIAP3 = P1.AP3];
IF ((P1.8SUMAP3 GE 200) AND (P1.8SUMAP3 LT 300) AND (P1.QACTIVITE8 EQ 108) AND (P1.AP8JOUR EQ 6)) [P1.SAMEDIAP3 = P1.AP3];
IF ((P1.9SUMAP3 GE 200) AND (P1.9SUMAP3 LT 300) AND (P1.QACTIVITE9 EQ 109) AND (P1.AP9JOUR EQ 6)) [P1.SAMEDIAP3 = P1.AP3];
IF ((P1.10SUMAP3 GE 200) AND (P1.10SUMAP3 LT 300) AND (P1.QACTIVITE_10 EQ 110) AND (P1.AP10JOUR EQ 6)) [P1.SAMEDIAP3 = P1.AP3];
IF ((P1.11SUMAP3 GE 200) AND (P1.11SUMAP3 LT 300) AND (P1.QACTIVITE_11 EQ 111) AND (P1.AP11JOUR EQ 6)) [P1.SAMEDIAP3 = P1.AP3];
IF ((P1.12SUMAP3 GE 200) AND (P1.12SUMAP3 LT 300) AND (P1.QACTIVITE_12 EQ 112) AND (P1.AP12JOUR EQ 6)) [P1.SAMEDIAP3 = P1.AP3];
IF ((P1.3SUMAP3 GE 200) AND (P1.3SUMAP3 LT 300) AND (P1.QACTIVITE3 EQ 103) AND (P1.AP3JOUR EQ 7)) [P1.DIMANCHEAP3 = P1.AP3];
IF ((P1.4SUMAP3 GE 200) AND (P1.4SUMAP3 LT 300) AND (P1.QACTIVITE4 EQ 104) AND (P1.AP4JOUR EQ 7)) [P1.DIMANCHEAP3 = P1.AP3];
IF ((P1.5SUMAP3 GE 200) AND (P1.5SUMAP3 LT 300) AND (P1.QACTIVITE5 EQ 105) AND (P1.AP5JOUR EQ 7)) [P1.DIMANCHEAP3 = P1.AP3];
IF ((P1.6SUMAP3 GE 200) AND (P1.6SUMAP3 LT 300) AND (P1.QACTIVITE6 EQ 106) AND (P1.AP6JOUR EQ 7)) [P1.DIMANCHEAP3 = P1.AP3];
IF ((P1.7SUMAP3 GE 200) AND (P1.7SUMAP3 LT 300) AND (P1.QACTIVITE7 EQ 107) AND (P1.AP7JOUR EQ 7)) [P1.DIMANCHEAP3 = P1.AP3];
IF ((P1.8SUMAP3 GE 200) AND (P1.8SUMAP3 LT 300) AND (P1.QACTIVITE8 EQ 108) AND (P1.AP8JOUR EQ 7)) [P1.DIMANCHEAP3 = P1.AP3];
IF ((P1.9SUMAP3 GE 200) AND (P1.9SUMAP3 LT 300) AND (P1.QACTIVITE9 EQ 109) AND (P1.AP9JOUR EQ 7)) [P1.DIMANCHEAP3 = P1.AP3];

IF ((P1.10SUMAP3 GE 200) AND (P1.10SUMAP3 LT 300) AND (P1.QACTIVITE_10 EQ 110) AND (P1.AP10JOUR EQ 7)) [P1.DIMANCHEAP3 = P1.AP3];
IF ((P1.11SUMAP3 GE 200) AND (P1.11SUMAP3 LT 300) AND (P1.QACTIVITE_11 EQ 111) AND (P1.AP11JOUR EQ 7)) [P1.DIMANCHEAP3 = P1.AP3];
IF ((P1.12SUMAP3 GE 200) AND (P1.12SUMAP3 LT 300) AND (P1.QACTIVITE_12 EQ 112) AND (P1.AP12JOUR EQ 7)) [P1.DIMANCHEAP3 = P1.AP3];
POURSUITE DU CODE
IF (P1.P7BARCHOICE EQ 101) [P1.BAR = 'Être trop fatigué ou fatiguée pour faire de l´activité physique'];
IF (P1.P7BARCHOICE EQ 102) [P1.BAR = 'Avoir beaucoup de choses à faire mis à part l´activité physique'];
IF (P1.P7BARCHOICE EQ 103) [P1.BAR = 'température sera trop mauvaise pour faire de l´activité physique'];
IF (P1.P7BARCHOICE EQ 104) [P1.BAR = 'Ne pas avoir personne avec qui faire de l´activité physique'];
IF (P1.P7BARCHOICE EQ 105) [P1.BAR = 'Ne pas avoir accès à des emplacements pour faire de l´activité physique'];
IF (P1.P7BARCHOICE EQ 106) [P1.BAR = 'Ne pas avoir d´argent pour faire de l´activité physique'];
IF (P1.P7BARCHOICE EQ 107) [P1.BAR = 'Être trop gêné ou gênée, manquer de confiance pour aller faire de l´activité physique'];
IF (P1.P7BARCHOICE EQ 108) [P1.BAR = 'Avoir peur d´être insuffisamment en santé pour faire de l´activité physique'];
IF (P1.P7BARCHOICE EQ 109) [P1.BAR = 'Manquer de motivation'];
IF ((P1.P7BARCHOICE EQ 110) AND (P1.Q_BARANSWER NE 0)) [P1.BAR = P1.Q_BARANSWER];
IF (P1.SUMSOL EQ 101) [P1.SOL = 'Je vais faire de l´activité tôt dans la journée, en matinée ou à l´heure du midi.'];
IF (P1.SUMSOL EQ 102) [P1.SOL = 'J´essaie de garder en tête que si je fais de l´activité physique, j´aurai de plus en plus d´énergie dans la vie.'];
IF (P1.SUMSOL EQ 103) [P1.SOL = 'Je vais planifier des activités avec d´autres personnes qui me motiveront.'];
IF (P1.SUMSOL EQ 104) [P1.SOL = 'Je prends une collation pour me donner de l´énergie et j´y vais quand même.'];
IF (P1.SUMSOL EQ 201) [P1.SOL = 'Faire de l´activité physique par bloc de 10 minutes les journées où je n´ai pas de temps.'];
IF (P1.SUMSOL EQ 202) [P1.SOL = 'Je vais remplacer des activités où je suis assis ou assise, télé, ordinateur ou autres, par des activités physiques.'];
IF (P1.SUMSOL EQ 203) [P1.SOL = 'Je vais me procurer un agenda et inscrire dans mon horaire les moments où je vais faire de l´activité physique.'];
IF (P1.SUMSOL EQ 204) [P1.SOL = 'Je vais prendre des pauses de travail active et faire des 10 minutes de marche.'];
IF (P1.SUMSOL EQ 205) [P1.SOL = 'Je vais me fixer des moments avec d´autres personnes qui me plaisent pour aller faire de l´activité physique.'];
IF (P1.SUMSOL EQ 301) [P1.SOL = 'Je vais me procurer l´équipement nécessaire pour en faire même quand il pleut, quand il neige ou quand il fait trop chaud.'];
IF (P1.SUMSOL EQ 302) [P1.SOL = 'Je vais faire de l’activité physique chez nous ou à l´intérieur dans un endroit que j´aurai choisi quand il fait moins beau.'];

IF (P1.SUMSOL EQ 303) [P1.SOL = 'Je vais me préparer une activité physique alternative au cas où il serait désagréable d´aller dehors.'];
IF (P1.SUMSOL EQ 304) [P1.SOL = 'Je vais me dresser une liste des activités que je serais prêtE à faire si la température est mauvaise.'];
IF (P1.SUMSOL EQ 401) [P1.SOL = 'Je vais me joindre à un groupe qui pratique l´activité physique que j´aime. Ex : la marche, l´aquaforme ou la natation.'];
IF (P1.SUMSOL EQ 402) [P1.SOL = 'Je vais inviter mes amis, ma famille ou mes collègues à faire de l´activité physique avec moi.'];
IF (P1.SUMSOL EQ 403) [P1.SOL = 'Je vais amener mon chien marcher avec moi.'];
IF (P1.SUMSOL EQ 404) [P1.SOL = 'Je vais participer dans des discussions en ligne sur des pages facebook : Diabète Québec et autres.'];
IF (P1.SUMSOL EQ 501) [P1.SOL = 'Je vais faire de la marche ou du vélo dehors, c´est simple et gratuit.'];
IF (P1.SUMSOL EQ 502) [P1.SOL = 'Je vais faire de l´activité physique dans ma maison comme du yoga, un programme sur DVD, vélo stationnaire, tapis roulant, etc.'];
IF (P1.SUMSOL EQ 503) [P1.SOL = 'Je vais aller voir au centre communautaire de mon quartier pour me donner des options.'];
IF (P1.SUMSOL EQ 504) [P1.SOL = 'Je vais discuter avec mon médecin, avec un ami ou un spécialiste de l´activité physique pour avoir des conseils.'];
IF (P1.SUMSOL EQ 601) [P1.SOL = 'Je vais trouver des activités physiques abordables et simples : marcher dehors, faire du vélo, nager à la piscine communautaire.'];
IF (P1.SUMSOL EQ 602) [P1.SOL = 'Je vais aller voir au centre communautaire de mon quartier pour me donner des options.'];
IF (P1.SUMSOL EQ 603) [P1.SOL = 'Je vais économiser pour m´acheter un DVD d´activité physique ou pour une autre activité physique que j´aimerais faire.'];
IF (P1.SUMSOL EQ 604) [P1.SOL = 'Je vais aller marcher avec ma famille, des amis ou des collègues.'];
IF (P1.SUMSOL EQ 701) [P1.SOL = 'Je vais choisir une activité physique simple comme la marche ou la bicyclette.'];
IF (P1.SUMSOL EQ 702) [P1.SOL = 'Je vais aller faire de l´activité physique avec des gens qui m´acceptent comme je suis, qui évitent de me juger.'];
IF (P1.SUMSOL EQ 703) [P1.SOL = 'Au départ, je vais faire de l´activité physique dans des endroits où je suis seul[LETTRE_E] pour prendre confiance.'];
IF (P1.SUMSOL EQ 704) [P1.SOL = 'Je vais éviter des endroits qui me gêne davantage comme les centres de conditionnement.'];
IF (P1.SUMSOL EQ 801) [P1.SOL = 'Je vais consulter mon médecin pour mettre au clair si je peux faire de l´activité physique.'];
IF (P1.SUMSOL EQ 802) [P1.SOL = 'Je vais consulter un spécialiste de l´activité physique pour qu´il me rassure et me conseille sur ce qui est sécuritaire pour moi.'];
IF (P1.SUMSOL EQ 803) [P1.SOL = 'Je vais commencer à petite dose. 10 à 20 minutes maximum d´activités physiques les jours où je suis actif, active.'];
IF (P1.SUMSOL EQ 804) [P1.SOL = 'Je vais faire de l´activité physique accompagnéE d´un spécialiste de l´activité physique.'];

IF (P1.SUMSOL EQ 805) [P1.SOL = 'Je vais faire de l´activité physique dans un groupe où un spécialiste de l´activité physique est présent.'];
IF (P1.SUMSOL EQ 901) [P1.SOL = 'Je vais signer un contrat papier personnel comme quoi je m´engage à être actif, active.'];
IF (P1.SUMSOL EQ 902) [P1.SOL = 'Je vais signer un contrat papier avec mes proches comme quoi je m´engage à être actif, active.'];
IF (P1.SUMSOL EQ 903) [P1.SOL = 'Je vais essayer de nouvelles activités physiques pour trouver celles qui me plaisent vraiment.'];
IF (P1.SUMSOL EQ 904) [P1.SOL = 'À chaque activité physique que je fais, je prends un temps pour me rappeler ce que cela va m´apporter de mieux dans ma vie.'];
IF (P1.SUMSOL EQ 905) [P1.SOL = 'Je vais faire de l´activité physique avec des gens que j´aime pour me motiver.'];
IF ((P1.SUMSOL EQ 999) AND (P1.P8QSOLOPEN NE 0)) [P1.SOL = P1.P8QSOLOPEN];
IF (P1.SUMSOL EQ 0) [P1.SOL = P1.P8QSOL_10];
PLAN DE LA semaine 2
formules p2
IF ((P2.QACTI12 EQ 112) AND (P2.QAOPEN NE 0)) [P2.QACTIVITE_12 = 112];
[P2.2SUMAP2 = P2.QACTIVITE1];
[P2.3SUMAP2 = P2.QACTIVITE1+P2.QACTIVITE2];
[P2.4SUMAP2 = P2.QACTIVITE1+P2.QACTIVITE2+P2.QACTIVITE3];
[P2.5SUMAP2 = P2.QACTIVITE1+P2.QACTIVITE2+P2.QACTIVITE3+P2.QACTIVITE4];
[P2.6SUMAP2 = P2.QACTIVITE1+P2.QACTIVITE2+P2.QACTIVITE3+P2.QACTIVITE4+P2.QACTIVITE5];
[P2.7SUMAP2 =
P2.QACTIVITE1+P2.QACTIVITE2+P2.QACTIVITE3+P2.QACTIVITE4+P2.QACTIVITE5+P2.QACTIVITE6];
[P2.8SUMAP2 =
P2.QACTIVITE1+P2.QACTIVITE2+P2.QACTIVITE3+P2.QACTIVITE4+P2.QACTIVITE5+P2.QACTIVITE6+P2.QACTIVITE7];
[P2.9SUMAP2 =
P2.QACTIVITE1+P2.QACTIVITE2+P2.QACTIVITE3+P2.QACTIVITE4+P2.QACTIVITE5+P2.QACTIVITE6+P2.QACTIVITE7+P2.QACTIVITE8];
[P2.10SUMAP2 =
P2.QACTIVITE1+P2.QACTIVITE2+P2.QACTIVITE3+P2.QACTIVITE4+P2.QACTIVITE5+P2.QACTIVITE6+P2.QACTIVITE7+P2.QACTIVITE8+P2.QACTIVITE9];
[P2.11SUMAP2 =
P2.QACTIVITE1+P2.QACTIVITE2+P2.QACTIVITE3+P2.QACTIVITE4+P2.QACTIVITE5+P2.QACTIVITE6+P2.QACTIVITE7+P2.QACTIVITE8+P2.QACTIVITE9+P2.QACTIVITE_10];
[P2.12SUMAP2 =
P2.QACTIVITE1+P2.QACTIVITE2+P2.QACTIVITE3+P2.QACTIVITE4+P2.QACTIVITE5+P2.QACTIVITE6+P2.QACTIVITE7+P2.QACTIVITE8+P2.QACTIVITE9+P2.QACTIVITE_10+P2.QACTIVITE_11];
[P2.3SUMAP3 = P2.QACTIVITE1+P2.QACTIVITE2];
[P2.4SUMAP3 = P2.QACTIVITE1+P2.QACTIVITE2+P2.QACTIVITE3];
[P2.5SUMAP3 = P2.QACTIVITE1+P2.QACTIVITE2+P2.QACTIVITE3+P2.QACTIVITE4];

```
[P2.6SUMAP3 = P2.QACTIVITE1+P2.QACTIVITE2+P2.QACTIVITE3+P2.QACTIVITE4+P2.QACTIVITE5];
[P2.7SUMAP3 =
P2.QACTIVITE1+P2.QACTIVITE2+P2.QACTIVITE3+P2.QACTIVITE4+P2.QACTIVITE5+P2.QACTIVITE6];
[P2.8SUMAP3 =
P2.QACTIVITE1+P2.QACTIVITE2+P2.QACTIVITE3+P2.QACTIVITE4+P2.QACTIVITE5+P2.QACTIVITE6+P2.QACTI
VITE7];
[P2.9SUMAP3 =
P2.QACTIVITE1+P2.QACTIVITE2+P2.QACTIVITE3+P2.QACTIVITE4+P2.QACTIVITE5+P2.QACTIVITE6+P2.QACTI
VITE7+P2.QACTIVITE8];
[P2.10SUMAP3 =
P2.QACTIVITE1+P2.QACTIVITE2+P2.QACTIVITE3+P2.QACTIVITE4+P2.QACTIVITE5+P2.QACTIVITE6+P2.QACTI
VITE7+P2.QACTIVITE8+P2.QACTIVITE9];
[P2.11SUMAP3 =
P2.QACTIVITE1+P2.QACTIVITE2+P2.QACTIVITE3+P2.QACTIVITE4+P2.QACTIVITE5+P2.QACTIVITE6+P2.QACTI
VITE7+P2.QACTIVITE8+P2.QACTIVITE9+P2.QACTIVITE_10];
[P2.12SUMAP3 =
P2.QACTIVITE1+P2.QACTIVITE2+P2.QACTIVITE3+P2.QACTIVITE4+P2.QACTIVITE5+P2.QACTIVITE6+P2.QACTI
VITE7+P2.QACTIVITE8+P2.QACTIVITE9+P2.QACTIVITE_10+P2.QACTIVITE_11];
[P2.SUMAP =
P2.QACTIVITE1+P2.QACTIVITE2+P2.QACTIVITE3+P2.QACTIVITE4+P2.QACTIVITE5+P2.QACTIVITE6+P2.QACTI
VITE7+P2.QACTIVITE8+P2.QACTIVITE9+P2.QACTIVITE_10+P2.QACTIVITE_11+P2.QACTIVITE_12];
[P2.SUMSOL =
P2.P8QSOL1+P2.P8QSOL2+P2.P8QSOL3+P2.P8QSOL4+P2.P8QSOL5+P2.P8QSOL6+P2.P8QSOL7+P2.P8QSOL8
+P2.P8QSOL9];
IF (P2.QACTIVITE1 EQ 101) [P2.AP1 = 'marche rapide'];
IF ((P2.QACTIVITE1 NE 101) AND (P2.QACTIVITE2 EQ 102)) [P2.AP1 = 'raquettes à neige'];
IF ((P2.QACTIVITE1 NE 101) AND (P2.QACTIVITE2 NE 102) AND (P2.QACTIVITE3 EQ 103)) [P2.AP1 = 'vélo'];
IF ((P2.QACTIVITE1 NE 101) AND (P2.QACTIVITE2 NE 102) AND (P2.QACTIVITE3 NE 103) AND
(P2.QACTIVITE4 EQ 104)) [P2.AP1 = 'natation'];
IF ((P2.QACTIVITE1 NE 101) AND (P2.QACTIVITE2 NE 102) AND (P2.QACTIVITE3 NE 103) AND
(P2.QACTIVITE4 NE 104) AND (P2.QACTIVITE5 EQ 105)) [P2.AP1 = 'ski de fond'];
IF ((P2.QACTIVITE1 NE 101) AND (P2.QACTIVITE2 NE 102) AND (P2.QACTIVITE3 NE 103) AND
(P2.QACTIVITE4 NE 104) AND (P2.QACTIVITE5 NE 105) AND (P2.QACTIVITE6 EQ 106)) [P2.AP1 = 'tennis'];
IF ((P2.QACTIVITE1 NE 101) AND (P2.QACTIVITE2 NE 102) AND (P2.QACTIVITE3 NE 103) AND
(P2.QACTIVITE4 NE 104) AND (P2.QACTIVITE5 NE 105) AND (P2.QACTIVITE6 NE 106) AND (P2.QACTIVITE7
EQ 107)) [P2.AP1 = 'course à pieds'];
IF ((P2.QACTIVITE1 NE 101) AND (P2.QACTIVITE2 NE 102) AND (P2.QACTIVITE3 NE 103) AND
(P2.QACTIVITE4 NE 104) AND (P2.QACTIVITE5 NE 105) AND (P2.QACTIVITE6 NE 106) AND (P2.QACTIVITE7
NE 107) AND (P2.QACTIVITE8 EQ 108)) [P2.AP1 = 'hockey'];
IF ((P2.QACTIVITE1 NE 101) AND (P2.QACTIVITE2 NE 102) AND (P2.QACTIVITE3 NE 103) AND
(P2.QACTIVITE4 NE 104) AND (P2.QACTIVITE5 NE 105) AND (P2.QACTIVITE6 NE 106) AND (P2.QACTIVITE7
NE 107) AND (P2.QACTIVITE8 NE 108) AND (P2.QACTIVITE9 EQ 109)) [P2.AP1 = 'basketball'];
```

IF ((P2.QACTIVITE1 NE 101) AND (P2.QACTIVITE2 NE 102) AND (P2.QACTIVITE3 NE 103) AND (P2.QACTIVITE4 NE 104) AND (P2.QACTIVITE5 NE 105) AND (P2.QACTIVITE6 NE 106) AND (P2.QACTIVITE7 NE 107) AND (P2.QACTIVITE8 NE 108) AND (P2.QACTIVITE9 NE 109) AND (P2.QACTIVITE_10 EQ 110)) [P2.AP1 = 'soccer'];
IF ((P2.QACTIVITE1 NE 101) AND (P2.QACTIVITE2 NE 102) AND (P2.QACTIVITE3 NE 103) AND (P2.QACTIVITE4 NE 104) AND (P2.QACTIVITE5 NE 105) AND (P2.QACTIVITE6 NE 106) AND (P2.QACTIVITE7 NE 107) AND (P2.QACTIVITE8 NE 108) AND (P2.QACTIVITE9 NE 109) AND (P2.QACTIVITE_10 NE 110) AND (P2.QACTIVITE_11 EQ 111)) [P2.AP1 = 'centre de conditionnement'];
IF ((P2.QACTIVITE1 NE 101) AND (P2.QACTIVITE2 NE 102) AND (P2.QACTIVITE3 NE 103) AND (P2.QACTIVITE4 NE 104) AND (P2.QACTIVITE5 NE 105) AND (P2.QACTIVITE6 NE 106) AND (P2.QACTIVITE7 NE 107) AND (P2.QACTIVITE8 NE 108) AND (P2.QACTIVITE9 NE 109) AND (P2.QACTIVITE_10 NE 110) AND (P2.QACTIVITE_11 NE 111) AND (P2.QACTIVITE_12 EQ 112)) [P2.AP1 = P2.QAOPEN];
IF ((P2.2SUMAP2 GE 100) AND (P2.2SUMAP2 LT 200) AND (P2.QACTIVITE2 EQ 102)) [P2.AP2 = 'raquettes à neige'];
IF ((P2.3SUMAP2 GE 100) AND (P2.3SUMAP2 LT 200) AND (P2.QACTIVITE3 EQ 103)) [P2.AP2 = 'vélo'];
IF ((P2.4SUMAP2 GE 100) AND (P2.4SUMAP2 LT 200) AND (P2.QACTIVITE4 EQ 104)) [P2.AP2 = 'natation'];
IF ((P2.5SUMAP2 GE 100) AND (P2.5SUMAP2 LT 200) AND (P2.QACTIVITE5 EQ 105)) [P2.AP2 = 'ski de fond'];
IF ((P2.6SUMAP2 GE 100) AND (P2.6SUMAP2 LT 200) AND (P2.QACTIVITE6 EQ 106)) [P2.AP2 = 'tennis'];
IF ((P2.7SUMAP2 GE 100) AND (P2.7SUMAP2 LT 200) AND (P2.QACTIVITE7 EQ 107)) [P2.AP2 = 'course à pieds'];
IF ((P2.8SUMAP2 GE 100) AND (P2.8SUMAP2 LT 200) AND (P2.QACTIVITE8 EQ 108)) [P2.AP2 = 'hockey'];
IF ((P2.9SUMAP2 GE 100) AND (P2.9SUMAP2 LT 200) AND (P2.QACTIVITE9 EQ 109)) [P2.AP2 = 'basketball'];
IF ((P2.10SUMAP2 GE 100) AND (P2.10SUMAP2 LT 200) AND (P2.QACTIVITE_10 EQ 110)) [P2.AP2 = 'soccer'];
IF ((P2.11SUMAP2 GE 100) AND (P2.11SUMAP2 LT 200) AND (P2.QACTIVITE_11 EQ 111)) [P2.AP2 = 'centre de conditionnement'];
IF ((P2.12SUMAP2 GE 100) AND (P2.12SUMAP2 LT 200) AND (P2.QACTIVITE_12 EQ 112)) [P2.AP2 = P2.QAOPEN];
IF ((P2.3SUMAP3 GE 200) AND (P2.3SUMAP3 LT 300) AND (P2.QACTIVITE3 EQ 103)) [P2.AP3 = 'vélo'];
IF ((P2.4SUMAP3 GE 200) AND (P2.4SUMAP3 LT 300) AND (P2.QACTIVITE4 EQ 104)) [P2.AP3 = 'natation'];
IF ((P2.5SUMAP3 GE 200) AND (P2.5SUMAP3 LT 300) AND (P2.QACTIVITE5 EQ 105)) [P2.AP3 = 'ski de fond'];
IF ((P2.6SUMAP3 GE 200) AND (P2.6SUMAP3 LT 300) AND (P2.QACTIVITE6 EQ 106)) [P2.AP3 = 'tennis'];
IF ((P2.7SUMAP3 GE 200) AND (P2.7SUMAP3 LT 300) AND (P2.QACTIVITE7 EQ 107)) [P2.AP3 = 'course à pieds'];
IF ((P2.8SUMAP3 GE 200) AND (P2.8SUMAP3 LT 300) AND (P2.QACTIVITE8 EQ 108)) [P2.AP3 = 'hockey'];
IF ((P2.9SUMAP3 GE 200) AND (P2.9SUMAP3 LT 300) AND (P2.QACTIVITE9 EQ 109)) [P2.AP3 = 'basketball'];
IF ((P2.10SUMAP3 GE 200) AND (P2.10SUMAP3 LT 300) AND (P2.QACTIVITE_10 EQ 110)) [P2.AP3 = 'soccer'];
IF ((P2.11SUMAP3 GE 200) AND (P2.11SUMAP3 LT 300) AND (P2.QACTIVITE_11 EQ 111)) [P2.AP3 = 'centre de conditionnement'];
IF ((P2.12SUMAP3 GE 200) AND (P2.12SUMAP3 LT 300) AND (P2.QACTIVITE_12 EQ 112)) [P2.AP3 = P2.QAOPEN];

ACTIVITÉ 1 DANS LE PLAN POUR CHAQUE JOUR DE LA SEMAINE
IF ((P2.QACTIVITE1 EQ 101) AND (P2.AP1JOUR EQ 1)) [P2.LUNDIAP1 = P2.AP1];
IF ((P2.QACTIVITE1 NE 101) AND (P2.QACTIVITE2 EQ 102) AND (P2.AP2JOUR EQ 1)) [P2.LUNDIAP1 = P2.AP1];
IF ((P2.QACTIVITE1 NE 101) AND (P2.QACTIVITE2 NE 102) AND (P2.QACTIVITE3 EQ 103) AND (P2.AP3JOUR EQ 1)) [P2.LUNDIAP1 = P2.AP1];
IF ((P2.QACTIVITE1 NE 101) AND (P2.QACTIVITE2 NE 102) AND (P2.QACTIVITE3 NE 103) AND (P2.QACTIVITE4 EQ 104) AND (P2.AP4JOUR EQ 1)) [P2.LUNDIAP1 = P2.AP1];
IF ((P2.QACTIVITE1 NE 101) AND (P2.QACTIVITE2 NE 102) AND (P2.QACTIVITE3 NE 103) AND (P2.QACTIVITE4 NE 104) AND (P2.QACTIVITE5 EQ 105) AND (P2.AP5JOUR EQ 1)) [P2.LUNDIAP1 = P2.AP1];
IF ((P2.QACTIVITE1 NE 101) AND (P2.QACTIVITE2 NE 102) AND (P2.QACTIVITE3 NE 103) AND (P2.QACTIVITE4 NE 104) AND (P2.QACTIVITE5 NE 105) AND (P2.QACTIVITE6 EQ 106) AND (P2.AP6JOUR EQ 1)) [P2.LUNDIAP1 = P2.AP1];
IF ((P2.QACTIVITE1 NE 101) AND (P2.QACTIVITE2 NE 102) AND (P2.QACTIVITE3 NE 103) AND (P2.QACTIVITE4 NE 104) AND (P2.QACTIVITE5 NE 105) AND (P2.QACTIVITE6 NE 106) AND (P2.QACTIVITE7 EQ 107) AND (P2.AP7JOUR EQ 1)) [P2.LUNDIAP1 = P2.AP1];
IF ((P2.QACTIVITE1 NE 101) AND (P2.QACTIVITE2 NE 102) AND (P2.QACTIVITE3 NE 103) AND (P2.QACTIVITE4 NE 104) AND (P2.QACTIVITE5 NE 105) AND (P2.QACTIVITE6 NE 106) AND (P2.QACTIVITE7 NE 107) AND (P2.QACTIVITE8 EQ 108) AND (P2.AP8JOUR EQ 1)) [P2.LUNDIAP1 = P2.AP1];
IF ((P2.QACTIVITE1 NE 101) AND (P2.QACTIVITE2 NE 102) AND (P2.QACTIVITE3 NE 103) AND (P2.QACTIVITE4 NE 104) AND (P2.QACTIVITE5 NE 105) AND (P2.QACTIVITE6 NE 106) AND (P2.QACTIVITE7 NE 107) AND (P2.QACTIVITE8 NE 108) AND (P2.QACTIVITE9 EQ 109) AND (P2.AP9JOUR EQ 1)) [P2.LUNDIAP1 = P2.AP1];
IF ((P2.QACTIVITE1 NE 101) AND (P2.QACTIVITE2 NE 102) AND (P2.QACTIVITE3 NE 103) AND (P2.QACTIVITE4 NE 104) AND (P2.QACTIVITE5 NE 105) AND (P2.QACTIVITE6 NE 106) AND (P2.QACTIVITE7 NE 107) AND (P2.QACTIVITE8 NE 108) AND (P2.QACTIVITE9 NE 109) AND (P2.QACTIVITE_10 EQ 110) AND (P2.AP10JOUR EQ 1)) [P2.LUNDIAP1 = P2.AP1];
IF ((P2.QACTIVITE1 NE 101) AND (P2.QACTIVITE2 NE 102) AND (P2.QACTIVITE3 NE 103) AND (P2.QACTIVITE4 NE 104) AND (P2.QACTIVITE5 NE 105) AND (P2.QACTIVITE6 NE 106) AND (P2.QACTIVITE7 NE 107) AND (P2.QACTIVITE8 NE 108) AND (P2.QACTIVITE9 NE 109) AND (P2.QACTIVITE_10 NE 110) AND (P2.QACTIVITE_11 EQ 111) AND (P2.AP11JOUR EQ 1)) [P2.LUNDIAP1 = P2.AP1];
IF ((P2.QACTIVITE1 NE 101) AND (P2.QACTIVITE2 NE 102) AND (P2.QACTIVITE3 NE 103) AND (P2.QACTIVITE4 NE 104) AND (P2.QACTIVITE5 NE 105) AND (P2.QACTIVITE6 NE 106) AND (P2.QACTIVITE7 NE 107) AND (P2.QACTIVITE8 NE 108) AND (P2.QACTIVITE9 NE 109) AND (P2.QACTIVITE_10 NE 110) AND (P2.QACTIVITE_11 NE 111) AND (P2.QACTIVITE_12 EQ 112) AND (P2.AP12JOUR EQ 1)) [P2.LUNDIAP1 = P2.AP1];
IF ((P2.QACTIVITE1 EQ 101) AND (P2.AP1JOUR EQ 2)) [P2.MARDIAP1 = P2.AP1];
IF ((P2.QACTIVITE1 NE 101) AND (P2.QACTIVITE2 EQ 102) AND (P2.AP2JOUR EQ 2)) [P2.MARDIAP1 = P2.AP1];
IF ((P2.QACTIVITE1 NE 101) AND (P2.QACTIVITE2 NE 102) AND (P2.QACTIVITE3 EQ 103) AND (P2.AP3JOUR EQ 2)) [P2.MARDIAP1 = P2.AP1];

IF ((P2.QACTIVITE1 NE 101) AND (P2.QACTIVITE2 NE 102) AND (P2.QACTIVITE3 NE 103) AND (P2.QACTIVITE4 EQ 104) AND (P2.AP4JOUR EQ 2)) [P2.MARDIAP1 = P2.AP1];
IF ((P2.QACTIVITE1 NE 101) AND (P2.QACTIVITE2 NE 102) AND (P2.QACTIVITE3 NE 103) AND (P2.QACTIVITE4 NE 104) AND (P2.QACTIVITE5 EQ 105) AND (P2.AP5JOUR EQ 2)) [P2.MARDIAP1 = P2.AP1];
IF ((P2.QACTIVITE1 NE 101) AND (P2.QACTIVITE2 NE 102) AND (P2.QACTIVITE3 NE 103) AND (P2.QACTIVITE4 NE 104) AND (P2.QACTIVITE5 NE 105) AND (P2.QACTIVITE6 EQ 106) AND (P2.AP6JOUR EQ 2)) [P2.MARDIAP1 = P2.AP1];
IF ((P2.QACTIVITE1 NE 101) AND (P2.QACTIVITE2 NE 102) AND (P2.QACTIVITE3 NE 103) AND (P2.QACTIVITE4 NE 104) AND (P2.QACTIVITE5 NE 105) AND (P2.QACTIVITE6 NE 106) AND (P2.QACTIVITE7 EQ 107) AND (P2.AP7JOUR EQ 2)) [P2.MARDIAP1 = P2.AP1];
IF ((P2.QACTIVITE1 NE 101) AND (P2.QACTIVITE2 NE 102) AND (P2.QACTIVITE3 NE 103) AND (P2.QACTIVITE4 NE 104) AND (P2.QACTIVITE5 NE 105) AND (P2.QACTIVITE6 NE 106) AND (P2.QACTIVITE7 NE 107) AND (P2.QACTIVITE8 EQ 108) AND (P2.AP8JOUR EQ 2)) [P2.MARDIAP1 = P2.AP1];
IF ((P2.QACTIVITE1 NE 101) AND (P2.QACTIVITE2 NE 102) AND (P2.QACTIVITE3 NE 103) AND (P2.QACTIVITE4 NE 104) AND (P2.QACTIVITE5 NE 105) AND (P2.QACTIVITE6 NE 106) AND (P2.QACTIVITE7 NE 107) AND (P2.QACTIVITE8 NE 108) AND (P2.QACTIVITE9 EQ 109) AND (P2.AP9JOUR EQ 2)) [P2.MARDIAP1 = P2.AP1];
IF ((P2.QACTIVITE1 NE 101) AND (P2.QACTIVITE2 NE 102) AND (P2.QACTIVITE3 NE 103) AND (P2.QACTIVITE4 NE 104) AND (P2.QACTIVITE5 NE 105) AND (P2.QACTIVITE6 NE 106) AND (P2.QACTIVITE7 NE 107) AND (P2.QACTIVITE8 NE 108) AND (P2.QACTIVITE9 NE 109) AND (P2.QACTIVITE_10 EQ 110) AND (P2.AP10JOUR EQ 2)) [P2.MARDIAP1 = P2.AP1];
IF ((P2.QACTIVITE1 NE 101) AND (P2.QACTIVITE2 NE 102) AND (P2.QACTIVITE3 NE 103) AND (P2.QACTIVITE4 NE 104) AND (P2.QACTIVITE5 NE 105) AND (P2.QACTIVITE6 NE 106) AND (P2.QACTIVITE7 NE 107) AND (P2.QACTIVITE8 NE 108) AND (P2.QACTIVITE9 NE 109) AND (P2.QACTIVITE_10 NE 110) AND (P2.QACTIVITE_11 EQ 111) AND (P2.AP11JOUR EQ 2)) [P2.MARDIAP1 = P2.AP1];
IF ((P2.QACTIVITE1 NE 101) AND (P2.QACTIVITE2 NE 102) AND (P2.QACTIVITE3 NE 103) AND (P2.QACTIVITE4 NE 104) AND (P2.QACTIVITE5 NE 105) AND (P2.QACTIVITE6 NE 106) AND (P2.QACTIVITE7 NE 107) AND (P2.QACTIVITE8 NE 108) AND (P2.QACTIVITE9 NE 109) AND (P2.QACTIVITE_10 NE 110) AND (P2.QACTIVITE_11 NE 111) AND (P2.QACTIVITE_12 EQ 112) AND (P2.AP12JOUR EQ 2)) [P2.MARDIAP1 = P2.AP1];
IF ((P2.QACTIVITE1 EQ 101) AND (P2.AP1JOUR EQ 3)) [P2.MERCREDIAP1 = P2.AP1];
IF ((P2.QACTIVITE1 NE 101) AND (P2.QACTIVITE2 EQ 102) AND (P2.AP2JOUR EQ 3)) [P2.MERCREDIAP1 = P2.AP1];
IF ((P2.QACTIVITE1 NE 101) AND (P2.QACTIVITE2 NE 102) AND (P2.QACTIVITE3 EQ 103) AND (P2.AP3JOUR EQ 3)) [P2.MERCREDIAP1 = P2.AP1];
IF ((P2.QACTIVITE1 NE 101) AND (P2.QACTIVITE2 NE 102) AND (P2.QACTIVITE3 NE 103) AND (P2.QACTIVITE4 EQ 104) AND (P2.AP4JOUR EQ 3)) [P2.MERCREDIAP1 = P2.AP1];
IF ((P2.QACTIVITE1 NE 101) AND (P2.QACTIVITE2 NE 102) AND (P2.QACTIVITE3 NE 103) AND (P2.QACTIVITE4 NE 104) AND (P2.QACTIVITE5 EQ 105) AND (P2.AP5JOUR EQ 3)) [P2.MERCREDIAP1 = P2.AP1];

IF ((P2.QACTIVITE1 NE 101) AND (P2.QACTIVITE2 NE 102) AND (P2.QACTIVITE3 NE 103) AND (P2.QACTIVITE4 NE 104) AND (P2.QACTIVITES5 NE 105) AND (P2.QACTIVITE6 EQ 106) AND (P2.AP6JOUR EQ 3)) [P2.MERCREDIAP1 = P2.AP1];
IF ((P2.QACTIVITE1 NE 101) AND (P2.QACTIVITE2 NE 102) AND (P2.QACTIVITE3 NE 103) AND (P2.QACTIVITE4 NE 104) AND (P2.QACTIVITES5 NE 105) AND (P2.QACTIVITE6 NE 106) AND (P2.QACTIVITE7 EQ 107) AND (P2.AP7JOUR EQ 3)) [P2.MERCREDIAP1 = P2.AP1];
IF ((P2.QACTIVITE1 NE 101) AND (P2.QACTIVITE2 NE 102) AND (P2.QACTIVITE3 NE 103) AND (P2.QACTIVITE4 NE 104) AND (P2.QACTIVITES5 NE 105) AND (P2.QACTIVITE6 NE 106) AND (P2.QACTIVITE7 NE 107) AND (P2.QACTIVITE8 EQ 108) AND (P2.AP8JOUR EQ 3)) [P2.MERCREDIAP1 = P2.AP1];
IF ((P2.QACTIVITE1 NE 101) AND (P2.QACTIVITE2 NE 102) AND (P2.QACTIVITE3 NE 103) AND (P2.QACTIVITE4 NE 104) AND (P2.QACTIVITE5 NE 105) AND (P2.QACTIVITE6 NE 106) AND (P2.QACTIVITE7 NE 107) AND (P2.QACTIVITE8 NE 108) AND (P2.QACTIVITE9 EQ 109) AND (P2.AP9JOUR EQ 3)) [P2.MERCREDIAP1 = P2.AP1];
IF ((P2.QACTIVITE1 NE 101) AND (P2.QACTIVITE2 NE 102) AND (P2.QACTIVITE3 NE 103) AND (P2.QACTIVITE4 NE 104) AND (P2.QACTIVITE5 NE 105) AND (P2.QACTIVITE6 NE 106) AND (P2.QACTIVITE7 NE 107) AND (P2.QACTIVITE8 NE 108) AND (P2.QACTIVITE9 NE 109) AND (P2.QACTIVITE_10 EQ 110) AND (P2.AP10JOUR EQ 3)) [P2.MERCREDIAP1 = P2.AP1];
IF ((P2.QACTIVITE1 NE 101) AND (P2.QACTIVITE2 NE 102) AND (P2.QACTIVITE3 NE 103) AND (P2.QACTIVITE4 NE 104) AND (P2.QACTIVITE5 NE 105) AND (P2.QACTIVITE6 NE 106) AND (P2.QACTIVITE7 NE 107) AND (P2.QACTIVITE8 NE 108) AND (P2.QACTIVITE9 NE 109) AND (P2.QACTIVITE_10 NE 110) AND (P2.QACTIVITE_11 EQ 111) AND (P2.AP11JOUR EQ 3)) [P2.MERCREDIAP1 = P2.AP1];
IF ((P2.QACTIVITE1 NE 101) AND (P2.QACTIVITE2 NE 102) AND (P2.QACTIVITE3 NE 103) AND (P2.QACTIVITE4 NE 104) AND (P2.QACTIVITE5 NE 105) AND (P2.QACTIVITE6 NE 106) AND (P2.QACTIVITE7 NE 107) AND (P2.QACTIVITE8 NE 108) AND (P2.QACTIVITE9 NE 109) AND (P2.QACTIVITE_10 NE 110) AND (P2.QACTIVITE_11 NE 111) AND (P2.QACTIVITE_12 EQ 112) AND (P2.AP12JOUR EQ 3)) [P2.MERCREDIAP1 = P2.AP1];
IF ((P2.QACTIVITE1 EQ 101) AND (P2.AP1JOUR EQ 4)) [P2.JEUDIAP1 = P2.AP1];
IF ((P2.QACTIVITE1 NE 101) AND (P2.QACTIVITE2 EQ 102) AND (P2.AP2JOUR EQ 4)) [P2.JEUDIAP1 = P2.AP1];
IF ((P2.QACTIVITE1 NE 101) AND (P2.QACTIVITE2 NE 102) AND (P2.QACTIVITE3 EQ 103) AND (P2.AP3JOUR EQ 4)) [P2.JEUDIAP1 = P2.AP1];
IF ((P2.QACTIVITE1 NE 101) AND (P2.QACTIVITE2 NE 102) AND (P2.QACTIVITE3 NE 103) AND (P2.QACTIVITE4 EQ 104) AND (P2.AP4JOUR EQ 4)) [P2.JEUDIAP1 = P2.AP1];
IF ((P2.QACTIVITE1 NE 101) AND (P2.QACTIVITE2 NE 102) AND (P2.QACTIVITE3 NE 103) AND (P2.QACTIVITE4 NE 104) AND (P2.QACTIVITES5 EQ 105) AND (P2.AP5JOUR EQ 4)) [P2.JEUDIAP1 = P2.AP1];
IF ((P2.QACTIVITE1 NE 101) AND (P2.QACTIVITE2 NE 102) AND (P2.QACTIVITE3 NE 103) AND (P2.QACTIVITE4 NE 104) AND (P2.QACTIVITES5 NE 105) AND (P2.QACTIVITE6 EQ 106) AND (P2.AP6JOUR EQ 4)) [P2.JEUDIAP1 = P2.AP1];
IF ((P2.QACTIVITE1 NE 101) AND (P2.QACTIVITE2 NE 102) AND (P2.QACTIVITE3 NE 103) AND (P2.QACTIVITE4 NE 104) AND (P2.QACTIVITES5 NE 105) AND (P2.QACTIVITE6 NE 106) AND (P2.QACTIVITE7 EQ 107) AND (P2.AP7JOUR EQ 4)) [P2.JEUDIAP1 = P2.AP1];

IF ((P2.QACTIVITE1 NE 101) AND (P2.QACTIVITE2 NE 102) AND (P2.QACTIVITE3 NE 103) AND (P2.QACTIVITE4 NE 104) AND (P2.QACTIVITE5 NE 105) AND (P2.QACTIVITE6 NE 106) AND (P2.QACTIVITE7 NE 107) AND (P2.QACTIVITE8 EQ 108) AND (P2.AP8JOUR EQ 4)) [P2.JEUDIAP1 = P2.AP1];
IF ((P2.QACTIVITE1 NE 101) AND (P2.QACTIVITE2 NE 102) AND (P2.QACTIVITE3 NE 103) AND (P2.QACTIVITE4 NE 104) AND (P2.QACTIVITE5 NE 105) AND (P2.QACTIVITE6 NE 106) AND (P2.QACTIVITE7 NE 107) AND (P2.QACTIVITE8 NE 108) AND (P2.QACTIVITE9 EQ 109) AND (P2.AP9JOUR EQ 4)) [P2.JEUDIAP1 = P2.AP1];
IF ((P2.QACTIVITE1 NE 101) AND (P2.QACTIVITE2 NE 102) AND (P2.QACTIVITE3 NE 103) AND (P2.QACTIVITE4 NE 104) AND (P2.QACTIVITE5 NE 105) AND (P2.QACTIVITE6 NE 106) AND (P2.QACTIVITE7 NE 107) AND (P2.QACTIVITE8 NE 108) AND (P2.QACTIVITE9 NE 109) AND (P2.QACTIVITE_10 EQ 110) AND (P2.AP10JOUR EQ 4)) [P2.JEUDIAP1 = P2.AP1];
IF ((P2.QACTIVITE1 NE 101) AND (P2.QACTIVITE2 NE 102) AND (P2.QACTIVITE3 NE 103) AND (P2.QACTIVITE4 NE 104) AND (P2.QACTIVITE5 NE 105) AND (P2.QACTIVITE6 NE 106) AND (P2.QACTIVITE7 NE 107) AND (P2.QACTIVITE8 NE 108) AND (P2.QACTIVITE9 NE 109) AND (P2.QACTIVITE_10 NE 110) AND (P2.QACTIVITE_11 EQ 111) AND (P2.AP11JOUR EQ 4)) [P2.JEUDIAP1 = P2.AP1];
IF ((P2.QACTIVITE1 NE 101) AND (P2.QACTIVITE2 NE 102) AND (P2.QACTIVITE3 NE 103) AND (P2.QACTIVITE4 NE 104) AND (P2.QACTIVITE5 NE 105) AND (P2.QACTIVITE6 NE 106) AND (P2.QACTIVITE7 NE 107) AND (P2.QACTIVITE8 NE 108) AND (P2.QACTIVITE9 NE 109) AND (P2.QACTIVITE_10 NE 110) AND (P2.QACTIVITE_11 NE 111) AND (P2.QACTIVITE_12 EQ 112) AND (P2.AP12JOUR EQ 4)) [P2.JEUDIAP1 = P2.AP1];
IF ((P2.QACTIVITE1 EQ 101) AND (P2.AP1JOUR EQ 5)) [P2.VENDREDIAP1 = P2.AP1];
IF ((P2.QACTIVITE1 NE 101) AND (P2.QACTIVITE2 EQ 102) AND (P2.AP2JOUR EQ 5)) [P2.VENDREDIAP1 = P2.AP1];
IF ((P2.QACTIVITE1 NE 101) AND (P2.QACTIVITE2 NE 102) AND (P2.QACTIVITE3 EQ 103) AND (P2.AP3JOUR EQ 5)) [P2.VENDREDIAP1 = P2.AP1];
IF ((P2.QACTIVITE1 NE 101) AND (P2.QACTIVITE2 NE 102) AND (P2.QACTIVITE3 NE 103) AND (P2.QACTIVITE4 EQ 104) AND (P2.AP4JOUR EQ 5)) [P2.VENDREDIAP1 = P2.AP1];
IF ((P2.QACTIVITE1 NE 101) AND (P2.QACTIVITE2 NE 102) AND (P2.QACTIVITE3 NE 103) AND (P2.QACTIVITE4 NE 104) AND (P2.QACTIVITE5 EQ 105) AND (P2.AP5JOUR EQ 5)) [P2.VENDREDIAP1 = P2.AP1];
IF ((P2.QACTIVITE1 NE 101) AND (P2.QACTIVITE2 NE 102) AND (P2.QACTIVITE3 NE 103) AND (P2.QACTIVITE4 NE 104) AND (P2.QACTIVITE5 NE 105) AND (P2.QACTIVITE6 EQ 106) AND (P2.AP6JOUR EQ 5)) [P2.VENDREDIAP1 = P2.AP1];
IF ((P2.QACTIVITE1 NE 101) AND (P2.QACTIVITE2 NE 102) AND (P2.QACTIVITE3 NE 103) AND (P2.QACTIVITE4 NE 104) AND (P2.QACTIVITE5 NE 105) AND (P2.QACTIVITE6 NE 106) AND (P2.QACTIVITE7 EQ 107) AND (P2.AP7JOUR EQ 5)) [P2.VENDREDIAP1 = P2.AP1];
IF ((P2.QACTIVITE1 NE 101) AND (P2.QACTIVITE2 NE 102) AND (P2.QACTIVITE3 NE 103) AND (P2.QACTIVITE4 NE 104) AND (P2.QACTIVITE5 NE 105) AND (P2.QACTIVITE6 NE 106) AND (P2.QACTIVITE7 NE 107) AND (P2.QACTIVITE8 EQ 108) AND (P2.AP8JOUR EQ 5)) [P2.VENDREDIAP1 = P2.AP1];
IF ((P2.QACTIVITE1 NE 101) AND (P2.QACTIVITE2 NE 102) AND (P2.QACTIVITE3 NE 103) AND (P2.QACTIVITE4 NE 104) AND (P2.QACTIVITE5 NE 105) AND (P2.QACTIVITE6 NE 106) AND (P2.QACTIVITE7

NE 107) AND (P2.QACTIVITE8 NE 108) AND (P2.QACTIVITE9 EQ 109) AND (P2.AP9JOUR EQ 5)) [P2.VENDREDIAP1 = P2.AP1];
IF ((P2.QACTIVITE1 NE 101) AND (P2.QACTIVITE2 NE 102) AND (P2.QACTIVITE3 NE 103) AND (P2.QACTIVITE4 NE 104) AND (P2.QACTIVITE5 NE 105) AND (P2.QACTIVITE6 NE 106) AND (P2.QACTIVITE7 NE 107) AND (P2.QACTIVITE8 NE 108) AND (P2.QACTIVITE9 NE 109) AND (P2.QACTIVITE_10 EQ 110) AND (P2.AP10JOUR EQ 5)) [P2.VENDREDIAP1 = P2.AP1];
IF ((P2.QACTIVITE1 NE 101) AND (P2.QACTIVITE2 NE 102) AND (P2.QACTIVITE3 NE 103) AND (P2.QACTIVITE4 NE 104) AND (P2.QACTIVITE5 NE 105) AND (P2.QACTIVITE6 NE 106) AND (P2.QACTIVITE7 NE 107) AND (P2.QACTIVITE8 NE 108) AND (P2.QACTIVITE9 NE 109) AND (P2.QACTIVITE_10 NE 110) AND (P2.QACTIVITE_11 EQ 111) AND (P2.AP11JOUR EQ 5)) [P2.VENDREDIAP1 = P2.AP1];
IF ((P2.QACTIVITE1 NE 101) AND (P2.QACTIVITE2 NE 102) AND (P2.QACTIVITE3 NE 103) AND (P2.QACTIVITE4 NE 104) AND (P2.QACTIVITE5 NE 105) AND (P2.QACTIVITE6 NE 106) AND (P2.QACTIVITE7 NE 107) AND (P2.QACTIVITE8 NE 108) AND (P2.QACTIVITE9 NE 109) AND (P2.QACTIVITE_10 NE 110) AND (P2.QACTIVITE_11 NE 111) AND (P2.QACTIVITE_12 EQ 112) AND (P2.AP12JOUR EQ 5)) [P2.VENDREDIAP1 = P2.AP1];
IF ((P2.QACTIVITE1 EQ 101) AND (P2.AP1JOUR EQ 6)) [P2.SAMEDIAP1 = P2.AP1];
IF ((P2.QACTIVITE1 NE 101) AND (P2.QACTIVITE2 EQ 102) AND (P2.AP2JOUR EQ 6)) [P2.SAMEDIAP1 = P2.AP1];
IF ((P2.QACTIVITE1 NE 101) AND (P2.QACTIVITE2 NE 102) AND (P2.QACTIVITE3 EQ 103) AND (P2.AP3JOUR EQ 6)) [P2.SAMEDIAP1 = P2.AP1];
IF ((P2.QACTIVITE1 NE 101) AND (P2.QACTIVITE2 NE 102) AND (P2.QACTIVITE3 NE 103) AND (P2.QACTIVITE4 EQ 104) AND (P2.AP4JOUR EQ 6)) [P2.SAMEDIAP1 = P2.AP1];
IF ((P2.QACTIVITE1 NE 101) AND (P2.QACTIVITE2 NE 102) AND (P2.QACTIVITE3 NE 103) AND (P2.QACTIVITE4 NE 104) AND (P2.QACTIVITE5 EQ 105) AND (P2.AP5JOUR EQ 6)) [P2.SAMEDIAP1 = P2.AP1];
IF ((P2.QACTIVITE1 NE 101) AND (P2.QACTIVITE2 NE 102) AND (P2.QACTIVITE3 NE 103) AND (P2.QACTIVITE4 NE 104) AND (P2.QACTIVITE5 NE 105) AND (P2.QACTIVITE6 EQ 106) AND (P2.AP6JOUR EQ 6)) [P2.SAMEDIAP1 = P2.AP1];
IF ((P2.QACTIVITE1 NE 101) AND (P2.QACTIVITE2 NE 102) AND (P2.QACTIVITE3 NE 103) AND (P2.QACTIVITE4 NE 104) AND (P2.QACTIVITE5 NE 105) AND (P2.QACTIVITE6 NE 106) AND (P2.QACTIVITE7 EQ 107) AND (P2.AP7JOUR EQ 6)) [P2.SAMEDIAP1 = P2.AP1];
IF ((P2.QACTIVITE1 NE 101) AND (P2.QACTIVITE2 NE 102) AND (P2.QACTIVITE3 NE 103) AND (P2.QACTIVITE4 NE 104) AND (P2.QACTIVITE5 NE 105) AND (P2.QACTIVITE6 NE 106) AND (P2.QACTIVITE7 NE 107) AND (P2.QACTIVITE8 EQ 108) AND (P2.AP8JOUR EQ 6)) [P2.SAMEDIAP1 = P2.AP1];
IF ((P2.QACTIVITE1 NE 101) AND (P2.QACTIVITE2 NE 102) AND (P2.QACTIVITE3 NE 103) AND (P2.QACTIVITE4 NE 104) AND (P2.QACTIVITE5 NE 105) AND (P2.QACTIVITE6 NE 106) AND (P2.QACTIVITE7 NE 107) AND (P2.QACTIVITE8 NE 108) AND (P2.QACTIVITE9 EQ 109) AND (P2.AP9JOUR EQ 6)) [P2.SAMEDIAP1 = P2.AP1];
IF ((P2.QACTIVITE1 NE 101) AND (P2.QACTIVITE2 NE 102) AND (P2.QACTIVITE3 NE 103) AND (P2.QACTIVITE4 NE 104) AND (P2.QACTIVITE5 NE 105) AND (P2.QACTIVITE6 NE 106) AND (P2.QACTIVITE7 NE 107) AND (P2.QACTIVITE8 NE 108) AND (P2.QACTIVITE9 NE 109) AND (P2.QACTIVITE_10 EQ 110) AND (P2.AP10JOUR EQ 6)) [P2.SAMEDIAP1 = P2.AP1];

IF ((P2.QACTIVITE1 NE 101) AND (P2.QACTIVITE2 NE 102) AND (P2.QACTIVITE3 NE 103) AND (P2.QACTIVITE4 NE 104) AND (P2.QACTIVITES5 NE 105) AND (P2.QACTIVITE6 NE 106) AND (P2.QACTIVITE7 NE 107) AND (P2.QACTIVITE8 NE 108) AND (P2.QACTIVITE9 NE 109) AND (P2.QACTIVITE_10 NE 110) AND (P2.QACTIVITE_11 EQ 111) AND (P2.AP11JOUR EQ 6)) [P2.SAMEDIAP1 = P2.AP1];
IF ((P2.QACTIVITE1 NE 101) AND (P2.QACTIVITE2 NE 102) AND (P2.QACTIVITE3 NE 103) AND (P2.QACTIVITE4 NE 104) AND (P2.QACTIVITES5 NE 105) AND (P2.QACTIVITE6 NE 106) AND (P2.QACTIVITE7 NE 107) AND (P2.QACTIVITE8 NE 108) AND (P2.QACTIVITE9 NE 109) AND (P2.QACTIVITE_10 NE 110) AND (P2.QACTIVITE_11 NE 111) AND (P2.QACTIVITE_12 EQ 112) AND (P2.AP12JOUR EQ 6)) [P2.SAMEDIAP1 = P2.AP1];
IF ((P2.QACTIVITE1 EQ 101) AND (P2.AP1JOUR EQ 7)) [P2.DIMANCHEAP1 = P2.AP1];
IF ((P2.QACTIVITE1 NE 101) AND (P2.QACTIVITE2 EQ 102) AND (P2.AP2JOUR EQ 7)) [P2.DIMANCHEAP1 = P2.AP1];
IF ((P2.QACTIVITE1 NE 101) AND (P2.QACTIVITE2 NE 102) AND (P2.QACTIVITE3 EQ 103) AND (P2.AP3JOUR EQ 7)) [P2.DIMANCHEAP1 = P2.AP1];
IF ((P2.QACTIVITE1 NE 101) AND (P2.QACTIVITE2 NE 102) AND (P2.QACTIVITE3 NE 103) AND (P2.QACTIVITE4 EQ 104) AND (P2.AP4JOUR EQ 7)) [P2.DIMANCHEAP1 = P2.AP1];
IF ((P2.QACTIVITE1 NE 101) AND (P2.QACTIVITE2 NE 102) AND (P2.QACTIVITE3 NE 103) AND (P2.QACTIVITE4 NE 104) AND (P2.QACTIVITES5 EQ 105) AND (P2.AP5JOUR EQ 7)) [P2.DIMANCHEAP1 = P2.AP1];
IF ((P2.QACTIVITE1 NE 101) AND (P2.QACTIVITE2 NE 102) AND (P2.QACTIVITE3 NE 103) AND (P2.QACTIVITE4 NE 104) AND (P2.QACTIVITES5 NE 105) AND (P2.QACTIVITE6 EQ 106) AND (P2.AP6JOUR EQ 7)) [P2.DIMANCHEAP1 = P2.AP1];
IF ((P2.QACTIVITE1 NE 101) AND (P2.QACTIVITE2 NE 102) AND (P2.QACTIVITE3 NE 103) AND (P2.QACTIVITE4 NE 104) AND (P2.QACTIVITES5 NE 105) AND (P2.QACTIVITE6 NE 106) AND (P2.QACTIVITE7 EQ 107) AND (P2.AP7JOUR EQ 7)) [P2.DIMANCHEAP1 = P2.AP1];
IF ((P2.QACTIVITE1 NE 101) AND (P2.QACTIVITE2 NE 102) AND (P2.QACTIVITE3 NE 103) AND (P2.QACTIVITE4 NE 104) AND (P2.QACTIVITES5 NE 105) AND (P2.QACTIVITE6 NE 106) AND (P2.QACTIVITE7 NE 107) AND (P2.QACTIVITE8 EQ 108) AND (P2.AP8JOUR EQ 7)) [P2.DIMANCHEAP1 = P2.AP1];
IF ((P2.QACTIVITE1 NE 101) AND (P2.QACTIVITE2 NE 102) AND (P2.QACTIVITE3 NE 103) AND (P2.QACTIVITE4 NE 104) AND (P2.QACTIVITES5 NE 105) AND (P2.QACTIVITE6 NE 106) AND (P2.QACTIVITE7 NE 107) AND (P2.QACTIVITE8 NE 108) AND (P2.QACTIVITE9 EQ 109) AND (P2.AP9JOUR EQ 7)) [P2.DIMANCHEAP1 = P2.AP1];
IF ((P2.QACTIVITE1 NE 101) AND (P2.QACTIVITE2 NE 102) AND (P2.QACTIVITE3 NE 103) AND (P2.QACTIVITE4 NE 104) AND (P2.QACTIVITES5 NE 105) AND (P2.QACTIVITE6 NE 106) AND (P2.QACTIVITE7 NE 107) AND (P2.QACTIVITE8 NE 108) AND (P2.QACTIVITE9 NE 109) AND (P2.QACTIVITE_10 EQ 110) AND (P2.AP10JOUR EQ 7)) [P2.DIMANCHEAP1 = P2.AP1];
IF ((P2.QACTIVITE1 NE 101) AND (P2.QACTIVITE2 NE 102) AND (P2.QACTIVITE3 NE 103) AND (P2.QACTIVITE4 NE 104) AND (P2.QACTIVITES5 NE 105) AND (P2.QACTIVITE6 NE 106) AND (P2.QACTIVITE7 NE 107) AND (P2.QACTIVITE8 NE 108) AND (P2.QACTIVITE9 NE 109) AND (P2.QACTIVITE_10 NE 110) AND (P2.QACTIVITE_11 EQ 111) AND (P2.AP11JOUR EQ 7)) [P2.DIMANCHEAP1 = P2.AP1];
IF ((P2.QACTIVITE1 NE 101) AND (P2.QACTIVITE2 NE 102) AND (P2.QACTIVITE3 NE 103) AND (P2.QACTIVITE4 NE 104) AND (P2.QACTIVITES5 NE 105) AND (P2.QACTIVITE6 NE 106) AND (P2.QACTIVITE7

NE 107) AND (P2.QACTIVITE8 NE 108) AND (P2.QACTIVITE9 NE 109) AND (P2.QACTIVITE_10 NE 110) AND (P2.QACTIVITE_11 NE 111) AND (P2.QACTIVITE_12 EQ 112) AND (P2.AP12JOUR EQ 7)) [P2.DIMANCHEAP1 = P2.AP1];
ACTIVITÉ 2 DANS LE PLAN POUR CHAQUE JOUR DE LA SEMAINE
IF ((P2.2SUMAP2 GE 100) AND (P2.2SUMAP2 LT 200) AND (P2.QACTIVITE2 EQ 102) AND (P2.AP2JOUR EQ 1)) [P2.LUNDIAP2 = P2.AP2];
IF ((P2.3SUMAP2 GE 100) AND (P2.3SUMAP2 LT 200) AND (P2.QACTIVITE3 EQ 103) AND (P2.AP3JOUR EQ 1)) [P2.LUNDIAP2 = P2.AP2];
IF ((P2.4SUMAP2 GE 100) AND (P2.4SUMAP2 LT 200) AND (P2.QACTIVITE4 EQ 104) AND (P2.AP4JOUR EQ 1)) [P2.LUNDIAP2 = P2.AP2];
IF ((P2.5SUMAP2 GE 100) AND (P2.5SUMAP2 LT 200) AND (P2.QACTIVITE5 EQ 105) AND (P2.AP5JOUR EQ 1)) [P2.LUNDIAP2 = P2.AP2];
IF ((P2.6SUMAP2 GE 100) AND (P2.6SUMAP2 LT 200) AND (P2.QACTIVITE6 EQ 106) AND (P2.AP6JOUR EQ 1)) [P2.LUNDIAP2 = P2.AP2];
IF ((P2.7SUMAP2 GE 100) AND (P2.7SUMAP2 LT 200) AND (P2.QACTIVITE7 EQ 107) AND (P2.AP7JOUR EQ 1)) [P2.LUNDIAP2 = P2.AP2];
IF ((P2.8SUMAP2 GE 100) AND (P2.8SUMAP2 LT 200) AND (P2.QACTIVITE8 EQ 108) AND (P2.AP8JOUR EQ 1)) [P2.LUNDIAP2 = P2.AP2];
IF ((P2.9SUMAP2 GE 100) AND (P2.9SUMAP2 LT 200) AND (P2.QACTIVITE9 EQ 109) AND (P2.AP9JOUR EQ 1)) [P2.LUNDIAP2 = P2.AP2];
IF ((P2.10SUMAP2 GE 100) AND (P2.10SUMAP2 LT 200) AND (P2.QACTIVITE_10 EQ 110) AND (P2.AP10JOUR EQ 1)) [P2.LUNDIAP2 = P2.AP2];
IF ((P2.11SUMAP2 GE 100) AND (P2.11SUMAP2 LT 200) AND (P2.QACTIVITE_11 EQ 111) AND (P2.AP11JOUR EQ 1)) [P2.LUNDIAP2 = P2.AP2];
IF ((P2.12SUMAP2 GE 100) AND (P2.12SUMAP2 LT 200) AND (P2.QACTIVITE_12 EQ 112) AND (P2.AP12JOUR EQ 1)) [P2.LUNDIAP2 = P2.AP2];
IF ((P2.2SUMAP2 GE 100) AND (P2.2SUMAP2 LT 200) AND (P2.QACTIVITE2 EQ 102) AND (P2.AP2JOUR EQ 2)) [P2.MARDIAP2 = P2.AP2];
IF ((P2.3SUMAP2 GE 100) AND (P2.3SUMAP2 LT 200) AND (P2.QACTIVITE3 EQ 103) AND (P2.AP3JOUR EQ 2)) [P2.MARDIAP2 = P2.AP2];
IF ((P2.4SUMAP2 GE 100) AND (P2.4SUMAP2 LT 200) AND (P2.QACTIVITE4 EQ 104) AND (P2.AP4JOUR EQ 2)) [P2.MARDIAP2 = P2.AP2];
IF ((P2.5SUMAP2 GE 100) AND (P2.5SUMAP2 LT 200) AND (P2.QACTIVITE5 EQ 105) AND (P2.AP5JOUR EQ 2)) [P2.MARDIAP2 = P2.AP2];
IF ((P2.6SUMAP2 GE 100) AND (P2.6SUMAP2 LT 200) AND (P2.QACTIVITE6 EQ 106) AND (P2.AP6JOUR EQ 2)) [P2.MARDIAP2 = P2.AP2];
IF ((P2.7SUMAP2 GE 100) AND (P2.7SUMAP2 LT 200) AND (P2.QACTIVITE7 EQ 107) AND (P2.AP7JOUR EQ 2)) [P2.MARDIAP2 = P2.AP2];
IF ((P2.8SUMAP2 GE 100) AND (P2.8SUMAP2 LT 200) AND (P2.QACTIVITE8 EQ 108) AND (P2.AP8JOUR EQ 2)) [P2.MARDIAP2 = P2.AP2];
IF ((P2.9SUMAP2 GE 100) AND (P2.9SUMAP2 LT 200) AND (P2.QACTIVITE9 EQ 109) AND (P2.AP9JOUR EQ 2)) [P2.MARDIAP2 = P2.AP2];

IF ((P2.10SUMAP2 GE 100) AND (P2.10SUMAP2 LT 200) AND (P2.QACTIVITE_10 EQ 110) AND (P2.AP10JOUR EQ 2)) [P2.MARDIAP2 = P2.AP2];
IF ((P2.11SUMAP2 GE 100) AND (P2.11SUMAP2 LT 200) AND (P2.QACTIVITE_11 EQ 111) AND (P2.AP11JOUR EQ 2)) [P2.MARDIAP2 = P2.AP2];
IF ((P2.12SUMAP2 GE 100) AND (P2.12SUMAP2 LT 200) AND (P2.QACTIVITE_12 EQ 112) AND (P2.AP12JOUR EQ 2)) [P2.MARDIAP2 = P2.AP2];
IF ((P2.2SUMAP2 GE 100) AND (P2.2SUMAP2 LT 200) AND (P2.QACTIVITE2 EQ 102) AND (P2.AP2JOUR EQ 3)) [P2.MERCREDIAP2 = P2.AP2];
IF ((P2.3SUMAP2 GE 100) AND (P2.3SUMAP2 LT 200) AND (P2.QACTIVITE3 EQ 103) AND (P2.AP3JOUR EQ 3)) [P2.MERCREDIAP2 = P2.AP2];
IF ((P2.4SUMAP2 GE 100) AND (P2.4SUMAP2 LT 200) AND (P2.QACTIVITE4 EQ 104) AND (P2.AP4JOUR EQ 3)) [P2.MERCREDIAP2 = P2.AP2];
IF ((P2.5SUMAP2 GE 100) AND (P2.5SUMAP2 LT 200) AND (P2.QACTIVITE5 EQ 105) AND (P2.AP5JOUR EQ 3)) [P2.MERCREDIAP2 = P2.AP2];
IF ((P2.6SUMAP2 GE 100) AND (P2.6SUMAP2 LT 200) AND (P2.QACTIVITE6 EQ 106) AND (P2.AP6JOUR EQ 3)) [P2.MERCREDIAP2 = P2.AP2];
IF ((P2.7SUMAP2 GE 100) AND (P2.7SUMAP2 LT 200) AND (P2.QACTIVITE7 EQ 107) AND (P2.AP7JOUR EQ 3)) [P2.MERCREDIAP2 = P2.AP2];
IF ((P2.8SUMAP2 GE 100) AND (P2.8SUMAP2 LT 200) AND (P2.QACTIVITE8 EQ 108) AND (P2.AP8JOUR EQ 3)) [P2.MERCREDIAP2 = P2.AP2];
IF ((P2.9SUMAP2 GE 100) AND (P2.9SUMAP2 LT 200) AND (P2.QACTIVITE9 EQ 109) AND (P2.AP9JOUR EQ 3)) [P2.MERCREDIAP2 = P2.AP2];
IF ((P2.10SUMAP2 GE 100) AND (P2.10SUMAP2 LT 200) AND (P2.QACTIVITE_10 EQ 110) AND (P2.AP10JOUR EQ 3)) [P2.MERCREDIAP2 = P2.AP2];
IF ((P2.11SUMAP2 GE 100) AND (P2.11SUMAP2 LT 200) AND (P2.QACTIVITE_11 EQ 111) AND (P2.AP11JOUR EQ 3)) [P2.MERCREDIAP2 = P2.AP2];
IF ((P2.12SUMAP2 GE 100) AND (P2.12SUMAP2 LT 200) AND (P2.QACTIVITE_12 EQ 112) AND (P2.AP12JOUR EQ 3)) [P2.MERCREDIAP2 = P2.AP2];
IF ((P2.2SUMAP2 GE 100) AND (P2.2SUMAP2 LT 200) AND (P2.QACTIVITE2 EQ 102) AND (P2.AP2JOUR EQ 4)) [P2.JEUDIAP2 = P2.AP2];
IF ((P2.3SUMAP2 GE 100) AND (P2.3SUMAP2 LT 200) AND (P2.QACTIVITE3 EQ 103) AND (P2.AP3JOUR EQ 4)) [P2.JEUDIAP2 = P2.AP2];
IF ((P2.4SUMAP2 GE 100) AND (P2.4SUMAP2 LT 200) AND (P2.QACTIVITE4 EQ 104) AND (P2.AP4JOUR EQ 4)) [P2.JEUDIAP2 = P2.AP2];
IF ((P2.5SUMAP2 GE 100) AND (P2.5SUMAP2 LT 200) AND (P2.QACTIVITE5 EQ 105) AND (P2.AP5JOUR EQ 4)) [P2.JEUDIAP2 = P2.AP2];
IF ((P2.6SUMAP2 GE 100) AND (P2.6SUMAP2 LT 200) AND (P2.QACTIVITE6 EQ 106) AND (P2.AP6JOUR EQ 4)) [P2.JEUDIAP2 = P2.AP2];
IF ((P2.7SUMAP2 GE 100) AND (P2.7SUMAP2 LT 200) AND (P2.QACTIVITE7 EQ 107) AND (P2.AP7JOUR EQ 4)) [P2.JEUDIAP2 = P2.AP2];
IF ((P2.8SUMAP2 GE 100) AND (P2.8SUMAP2 LT 200) AND (P2.QACTIVITE8 EQ 108) AND (P2.AP8JOUR EQ 4)) [P2.JEUDIAP2 = P2.AP2];

IF ((P2.9SUMAP2 GE 100) AND (P2.9SUMAP2 LT 200) AND (P2.QACTIVITE9 EQ 109) AND (P2.AP9JOUR EQ 4)) [P2.JEUDIAP2 = P2.AP2];
IF ((P2.10SUMAP2 GE 100) AND (P2.10SUMAP2 LT 200) AND (P2.QACTIVITE_10 EQ 110) AND (P2.AP10JOUR EQ 4)) [P2.JEUDIAP2 = P2.AP2];
IF ((P2.11SUMAP2 GE 100) AND (P2.11SUMAP2 LT 200) AND (P2.QACTIVITE_11 EQ 111) AND (P2.AP11JOUR EQ 4)) [P2.JEUDIAP2 = P2.AP2];
IF ((P2.12SUMAP2 GE 100) AND (P2.12SUMAP2 LT 200) AND (P2.QACTIVITE_12 EQ 112) AND (P2.AP12JOUR EQ 4)) [P2.JEUDIAP2 = P2.AP2];
IF ((P2.2SUMAP2 GE 100) AND (P2.2SUMAP2 LT 200) AND (P2.QACTIVITE2 EQ 102) AND (P2.AP2JOUR EQ 5)) [P2.VENDREDIAP2 = P2.AP2];
IF ((P2.3SUMAP2 GE 100) AND (P2.3SUMAP2 LT 200) AND (P2.QACTIVITE3 EQ 103) AND (P2.AP3JOUR EQ 5)) [P2.VENDREDIAP2 = P2.AP2];
IF ((P2.4SUMAP2 GE 100) AND (P2.4SUMAP2 LT 200) AND (P2.QACTIVITE4 EQ 104) AND (P2.AP4JOUR EQ 5)) [P2.VENDREDIAP2 = P2.AP2];
IF ((P2.5SUMAP2 GE 100) AND (P2.5SUMAP2 LT 200) AND (P2.QACTIVITE5 EQ 105) AND (P2.AP5JOUR EQ 5)) [P2.VENDREDIAP2 = P2.AP2];
IF ((P2.6SUMAP2 GE 100) AND (P2.6SUMAP2 LT 200) AND (P2.QACTIVITE6 EQ 106) AND (P2.AP6JOUR EQ 5)) [P2.VENDREDIAP2 = P2.AP2];
IF ((P2.7SUMAP2 GE 100) AND (P2.7SUMAP2 LT 200) AND (P2.QACTIVITE7 EQ 107) AND (P2.AP7JOUR EQ 5)) [P2.VENDREDIAP2 = P2.AP2];
IF ((P2.8SUMAP2 GE 100) AND (P2.8SUMAP2 LT 200) AND (P2.QACTIVITE8 EQ 108) AND (P2.AP8JOUR EQ 5)) [P2.VENDREDIAP2 = P2.AP2];
IF ((P2.9SUMAP2 GE 100) AND (P2.9SUMAP2 LT 200) AND (P2.QACTIVITE9 EQ 109) AND (P2.AP9JOUR EQ 5)) [P2.VENDREDIAP2 = P2.AP2];
IF ((P2.10SUMAP2 GE 100) AND (P2.10SUMAP2 LT 200) AND (P2.QACTIVITE_10 EQ 110) AND (P2.AP10JOUR EQ 5)) [P2.VENDREDIAP2 = P2.AP2];
IF ((P2.11SUMAP2 GE 100) AND (P2.11SUMAP2 LT 200) AND (P2.QACTIVITE_11 EQ 111) AND (P2.AP11JOUR EQ 5)) [P2.VENDREDIAP2 = P2.AP2];
IF ((P2.12SUMAP2 GE 100) AND (P2.12SUMAP2 LT 200) AND (P2.QACTIVITE_12 EQ 112) AND (P2.AP12JOUR EQ 5)) [P2.VENDREDIAP2 = P2.AP2];
IF ((P2.2SUMAP2 GE 100) AND (P2.2SUMAP2 LT 200) AND (P2.QACTIVITE2 EQ 102) AND (P2.AP2JOUR EQ 6)) [P2.SAMEDIAP2 = P2.AP2];
IF ((P2.3SUMAP2 GE 100) AND (P2.3SUMAP2 LT 200) AND (P2.QACTIVITE3 EQ 103) AND (P2.AP3JOUR EQ 6)) [P2.SAMEDIAP2 = P2.AP2];
IF ((P2.4SUMAP2 GE 100) AND (P2.4SUMAP2 LT 200) AND (P2.QACTIVITE4 EQ 104) AND (P2.AP4JOUR EQ 6)) [P2.SAMEDIAP2 = P2.AP2];
IF ((P2.5SUMAP2 GE 100) AND (P2.5SUMAP2 LT 200) AND (P2.QACTIVITE5 EQ 105) AND (P2.AP5JOUR EQ 6)) [P2.SAMEDIAP2 = P2.AP2];
IF ((P2.6SUMAP2 GE 100) AND (P2.6SUMAP2 LT 200) AND (P2.QACTIVITE6 EQ 106) AND (P2.AP6JOUR EQ 6)) [P2.SAMEDIAP2 = P2.AP2];
IF ((P2.7SUMAP2 GE 100) AND (P2.7SUMAP2 LT 200) AND (P2.QACTIVITE7 EQ 107) AND (P2.AP7JOUR EQ 6)) [P2.SAMEDIAP2 = P2.AP2];

IF ((P2.8SUMAP2 GE 100) AND (P2.8SUMAP2 LT 200) AND (P2.QACTIVITE8 EQ 108) AND (P2.AP8JOUR EQ 6)) [P2.SAMEDIAP2 = P2.AP2];
IF ((P2.9SUMAP2 GE 100) AND (P2.9SUMAP2 LT 200) AND (P2.QACTIVITE9 EQ 109) AND (P2.AP9JOUR EQ 6)) [P2.SAMEDIAP2 = P2.AP2];
IF ((P2.10SUMAP2 GE 100) AND (P2.10SUMAP2 LT 200) AND (P2.QACTIVITE_10 EQ 110) AND (P2.AP10JOUR EQ 6)) [P2.SAMEDIAP2 = P2.AP2];
IF ((P2.11SUMAP2 GE 100) AND (P2.11SUMAP2 LT 200) AND (P2.QACTIVITE_11 EQ 111) AND (P2.AP11JOUR EQ 6)) [P2.SAMEDIAP2 = P2.AP2];
IF ((P2.12SUMAP2 GE 100) AND (P2.12SUMAP2 LT 200) AND (P2.QACTIVITE_12 EQ 112) AND (P2.AP12JOUR EQ 6)) [P2.SAMEDIAP2 = P2.AP2];
IF ((P2.2SUMAP2 GE 100) AND (P2.2SUMAP2 LT 200) AND (P2.QACTIVITE2 EQ 102) AND (P2.AP2JOUR EQ 7)) [P2.DIMANCHEAP2 = P2.AP2];
IF ((P2.3SUMAP2 GE 100) AND (P2.3SUMAP2 LT 200) AND (P2.QACTIVITE3 EQ 103) AND (P2.AP3JOUR EQ 7)) [P2.DIMANCHEAP2 = P2.AP2];
IF ((P2.4SUMAP2 GE 100) AND (P2.4SUMAP2 LT 200) AND (P2.QACTIVITE4 EQ 104) AND (P2.AP4JOUR EQ 7)) [P2.DIMANCHEAP2 = P2.AP2];
IF ((P2.5SUMAP2 GE 100) AND (P2.5SUMAP2 LT 200) AND (P2.QACTIVITE5 EQ 105) AND (P2.AP5JOUR EQ 7)) [P2.DIMANCHEAP2 = P2.AP2];
IF ((P2.6SUMAP2 GE 100) AND (P2.6SUMAP2 LT 200) AND (P2.QACTIVITE6 EQ 106) AND (P2.AP6JOUR EQ 7)) [P2.DIMANCHEAP2 = P2.AP2];
IF ((P2.7SUMAP2 GE 100) AND (P2.7SUMAP2 LT 200) AND (P2.QACTIVITE7 EQ 107) AND (P2.AP7JOUR EQ 7)) [P2.DIMANCHEAP2 = P2.AP2];
IF ((P2.8SUMAP2 GE 100) AND (P2.8SUMAP2 LT 200) AND (P2.QACTIVITE8 EQ 108) AND (P2.AP8JOUR EQ 7)) [P2.DIMANCHEAP2 = P2.AP2];
IF ((P2.9SUMAP2 GE 100) AND (P2.9SUMAP2 LT 200) AND (P2.QACTIVITE9 EQ 109) AND (P2.AP9JOUR EQ 7)) [P2.DIMANCHEAP2 = P2.AP2];
IF ((P2.10SUMAP2 GE 100) AND (P2.10SUMAP2 LT 200) AND (P2.QACTIVITE_10 EQ 110) AND (P2.AP10JOUR EQ 7)) [P2.DIMANCHEAP2 = P2.AP2];
IF ((P2.11SUMAP2 GE 100) AND (P2.11SUMAP2 LT 200) AND (P2.QACTIVITE_11 EQ 111) AND (P2.AP11JOUR EQ 7)) [P2.DIMANCHEAP2 = P2.AP2];
IF ((P2.12SUMAP2 GE 100) AND (P2.12SUMAP2 LT 200) AND (P2.QACTIVITE_12 EQ 112) AND (P2.AP12JOUR EQ 7)) [P2.DIMANCHEAP2 = P2.AP2];
ACTIVITÉ 3 DANS LE PLAN POUR CHAQUE JOUR DE LA SEMAINE
IF ((P2.3SUMAP3 GE 200) AND (P2.3SUMAP3 LT 300) AND (P2.QACTIVITE3 EQ 103) AND (P2.AP3JOUR EQ 1)) [P2.LUNDIAP3 = P2.AP3];
IF ((P2.4SUMAP3 GE 200) AND (P2.4SUMAP3 LT 300) AND (P2.QACTIVITE4 EQ 104) AND (P2.AP4JOUR EQ 1)) [P2.LUNDIAP3 = P2.AP3];
IF ((P2.5SUMAP3 GE 200) AND (P2.5SUMAP3 LT 300) AND (P2.QACTIVITE5 EQ 105) AND (P2.AP5JOUR EQ 1)) [P2.LUNDIAP3 = P2.AP3];
IF ((P2.6SUMAP3 GE 200) AND (P2.6SUMAP3 LT 300) AND (P2.QACTIVITE6 EQ 106) AND (P2.AP6JOUR EQ 1)) [P2.LUNDIAP3 = P2.AP3];

IF ((P2.7SUMAP3 GE 200) AND (P2.7SUMAP3 LT 300) AND (P2.QACTIVITE7 EQ 107) AND (P2.AP7JOUR EQ 1)) [P2.LUNDIAP3 = P2.AP3];
IF ((P2.8SUMAP3 GE 200) AND (P2.8SUMAP3 LT 300) AND (P2.QACTIVITE8 EQ 108) AND (P2.AP8JOUR EQ 1)) [P2.LUNDIAP3 = P2.AP3];
IF ((P2.9SUMAP3 GE 200) AND (P2.9SUMAP3 LT 300) AND (P2.QACTIVITE9 EQ 109) AND (P2.AP9JOUR EQ 1)) [P2.LUNDIAP3 = P2.AP3];
IF ((P2.10SUMAP3 GE 200) AND (P2.10SUMAP3 LT 300) AND (P2.QACTIVITE_10 EQ 110) AND (P2.AP10JOUR EQ 1)) [P2.LUNDIAP3 = P2.AP3];
IF ((P2.11SUMAP3 GE 200) AND (P2.11SUMAP3 LT 300) AND (P2.QACTIVITE_11 EQ 111) AND (P2.AP11JOUR EQ 1)) [P2.LUNDIAP3 = P2.AP3];
IF ((P2.12SUMAP3 GE 200) AND (P2.12SUMAP3 LT 300) AND (P2.QACTIVITE_12 EQ 112) AND (P2.AP12JOUR EQ 1)) [P2.LUNDIAP3 = P2.AP3];
IF ((P2.3SUMAP3 GE 200) AND (P2.3SUMAP3 LT 300) AND (P2.QACTIVITE3 EQ 103) AND (P2.AP3JOUR EQ 2)) [P2.MARDIAP3 = P2.AP3];
IF ((P2.4SUMAP3 GE 200) AND (P2.4SUMAP3 LT 300) AND (P2.QACTIVITE4 EQ 104) AND (P2.AP4JOUR EQ 2)) [P2.MARDIAP3 = P2.AP3];
IF ((P2.5SUMAP3 GE 200) AND (P2.5SUMAP3 LT 300) AND (P2.QACTIVITE5 EQ 105) AND (P2.AP5JOUR EQ 2)) [P2.MARDIAP3 = P2.AP3];
IF ((P2.6SUMAP3 GE 200) AND (P2.6SUMAP3 LT 300) AND (P2.QACTIVITE6 EQ 106) AND (P2.AP6JOUR EQ 2)) [P2.MARDIAP3 = P2.AP3];
IF ((P2.7SUMAP3 GE 200) AND (P2.7SUMAP3 LT 300) AND (P2.QACTIVITE7 EQ 107) AND (P2.AP7JOUR EQ 2)) [P2.MARDIAP3 = P2.AP3];
IF ((P2.8SUMAP3 GE 200) AND (P2.8SUMAP3 LT 300) AND (P2.QACTIVITE8 EQ 108) AND (P2.AP8JOUR EQ 2)) [P2.MARDIAP3 = P2.AP3];
IF ((P2.9SUMAP3 GE 200) AND (P2.9SUMAP3 LT 300) AND (P2.QACTIVITE9 EQ 109) AND (P2.AP9JOUR EQ 2)) [P2.MARDIAP3 = P2.AP3];
IF ((P2.10SUMAP3 GE 200) AND (P2.10SUMAP3 LT 300) AND (P2.QACTIVITE_10 EQ 110) AND (P2.AP10JOUR EQ 2)) [P2.MARDIAP3 = P2.AP3];
IF ((P2.11SUMAP3 GE 200) AND (P2.11SUMAP3 LT 300) AND (P2.QACTIVITE_11 EQ 111) AND (P2.AP11JOUR EQ 2)) [P2.MARDIAP3 = P2.AP3];
IF ((P2.12SUMAP3 GE 200) AND (P2.12SUMAP3 LT 300) AND (P2.QACTIVITE_12 EQ 112) AND (P2.AP12JOUR EQ 2)) [P2.MARDIAP3 = P2.AP3];
IF ((P2.3SUMAP3 GE 200) AND (P2.3SUMAP3 LT 300) AND (P2.QACTIVITE3 EQ 103) AND (P2.AP3JOUR EQ 3)) [P2.MERCREDIAP3 = P2.AP3];
IF ((P2.4SUMAP3 GE 200) AND (P2.4SUMAP3 LT 300) AND (P2.QACTIVITE4 EQ 104) AND (P2.AP4JOUR EQ 3)) [P2.MERCREDIAP3 = P2.AP3];
IF ((P2.5SUMAP3 GE 200) AND (P2.5SUMAP3 LT 300) AND (P2.QACTIVITE5 EQ 105) AND (P2.AP5JOUR EQ 3)) [P2.MERCREDIAP3 = P2.AP3];
IF ((P2.6SUMAP3 GE 200) AND (P2.6SUMAP3 LT 300) AND (P2.QACTIVITE6 EQ 106) AND (P2.AP6JOUR EQ 3)) [P2.MERCREDIAP3 = P2.AP3];
IF ((P2.7SUMAP3 GE 200) AND (P2.7SUMAP3 LT 300) AND (P2.QACTIVITE7 EQ 107) AND (P2.AP7JOUR EQ 3)) [P2.MERCREDIAP3 = P2.AP3];

IF ((P2.8SUMAP3 GE 200) AND (P2.8SUMAP3 LT 300) AND (P2.QACTIVITE8 EQ 108) AND (P2.AP8JOUR EQ 3)) [P2.MERCREDIAP3 = P2.AP3];
IF ((P2.9SUMAP3 GE 200) AND (P2.9SUMAP3 LT 300) AND (P2.QACTIVITE9 EQ 109) AND (P2.AP9JOUR EQ 3)) [P2.MERCREDIAP3 = P2.AP3];
IF ((P2.10SUMAP3 GE 200) AND (P2.10SUMAP3 LT 300) AND (P2.QACTIVITE_10 EQ 110) AND (P2.AP10JOUR EQ 3)) [P2.MERCREDIAP3 = P2.AP3];
IF ((P2.11SUMAP3 GE 200) AND (P2.11SUMAP3 LT 300) AND (P2.QACTIVITE_11 EQ 111) AND (P2.AP11JOUR EQ 3)) [P2.MERCREDIAP3 = P2.AP3];
IF ((P2.12SUMAP3 GE 200) AND (P2.12SUMAP3 LT 300) AND (P2.QACTIVITE_12 EQ 112) AND (P2.AP12JOUR EQ 3)) [P2.MERCREDIAP3 = P2.AP3];
IF ((P2.3SUMAP3 GE 200) AND (P2.3SUMAP3 LT 300) AND (P2.QACTIVITE3 EQ 103) AND (P2.AP3JOUR EQ 4)) [P2.JEUDIAP3 = P2.AP3];
IF ((P2.4SUMAP3 GE 200) AND (P2.4SUMAP3 LT 300) AND (P2.QACTIVITE4 EQ 104) AND (P2.AP4JOUR EQ 4)) [P2.JEUDIAP3 = P2.AP3];
IF ((P2.5SUMAP3 GE 200) AND (P2.5SUMAP3 LT 300) AND (P2.QACTIVITE5 EQ 105) AND (P2.AP5JOUR EQ 4)) [P2.JEUDIAP3 = P2.AP3];
IF ((P2.6SUMAP3 GE 200) AND (P2.6SUMAP3 LT 300) AND (P2.QACTIVITE6 EQ 106) AND (P2.AP6JOUR EQ 4)) [P2.JEUDIAP3 = P2.AP3];
IF ((P2.7SUMAP3 GE 200) AND (P2.7SUMAP3 LT 300) AND (P2.QACTIVITE7 EQ 107) AND (P2.AP7JOUR EQ 4)) [P2.JEUDIAP3 = P2.AP3];
IF ((P2.8SUMAP3 GE 200) AND (P2.8SUMAP3 LT 300) AND (P2.QACTIVITE8 EQ 108) AND (P2.AP8JOUR EQ 4)) [P2.JEUDIAP3 = P2.AP3];
IF ((P2.9SUMAP3 GE 200) AND (P2.9SUMAP3 LT 300) AND (P2.QACTIVITE9 EQ 109) AND (P2.AP9JOUR EQ 4)) [P2.JEUDIAP3 = P2.AP3];
IF ((P2.10SUMAP3 GE 200) AND (P2.10SUMAP3 LT 300) AND (P2.QACTIVITE_10 EQ 110) AND (P2.AP10JOUR EQ 4)) [P2.JEUDIAP3 = P2.AP3];
IF ((P2.11SUMAP3 GE 200) AND (P2.11SUMAP3 LT 300) AND (P2.QACTIVITE_11 EQ 111) AND (P2.AP11JOUR EQ 4)) [P2.JEUDIAP3 = P2.AP3];
IF ((P2.12SUMAP3 GE 200) AND (P2.12SUMAP3 LT 300) AND (P2.QACTIVITE_12 EQ 112) AND (P2.AP12JOUR EQ 4)) [P2.JEUDIAP3 = P2.AP3];
IF ((P2.3SUMAP3 GE 200) AND (P2.3SUMAP3 LT 300) AND (P2.QACTIVITE3 EQ 103) AND (P2.AP3JOUR EQ 5)) [P2.VENDREDIAP3 = P2.AP3];
IF ((P2.4SUMAP3 GE 200) AND (P2.4SUMAP3 LT 300) AND (P2.QACTIVITE4 EQ 104) AND (P2.AP4JOUR EQ 5)) [P2.VENDREDIAP3 = P2.AP3];
IF ((P2.5SUMAP3 GE 200) AND (P2.5SUMAP3 LT 300) AND (P2.QACTIVITE5 EQ 105) AND (P2.AP5JOUR EQ 5)) [P2.VENDREDIAP3 = P2.AP3];
IF ((P2.6SUMAP3 GE 200) AND (P2.6SUMAP3 LT 300) AND (P2.QACTIVITE6 EQ 106) AND (P2.AP6JOUR EQ 5)) [P2.VENDREDIAP3 = P2.AP3];
IF ((P2.7SUMAP3 GE 200) AND (P2.7SUMAP3 LT 300) AND (P2.QACTIVITE7 EQ 107) AND (P2.AP7JOUR EQ 5)) [P2.VENDREDIAP3 = P2.AP3];
IF ((P2.8SUMAP3 GE 200) AND (P2.8SUMAP3 LT 300) AND (P2.QACTIVITE8 EQ 108) AND (P2.AP8JOUR EQ 5)) [P2.VENDREDIAP3 = P2.AP3];

IF ((P2.9SUMAP3 GE 200) AND (P2.9SUMAP3 LT 300) AND (P2.QACTIVITE9 EQ 109) AND (P2.AP9JOUR EQ 5)) [P2.VENDREDIAP3 = P2.AP3];
IF ((P2.10SUMAP3 GE 200) AND (P2.10SUMAP3 LT 300) AND (P2.QACTIVITE_10 EQ 110) AND (P2.AP10JOUR EQ 5)) [P2.VENDREDIAP3 = P2.AP3];
IF ((P2.11SUMAP3 GE 200) AND (P2.11SUMAP3 LT 300) AND (P2.QACTIVITE_11 EQ 111) AND (P2.AP11JOUR EQ 5)) [P2.VENDREDIAP3 = P2.AP3];
IF ((P2.12SUMAP3 GE 200) AND (P2.12SUMAP3 LT 300) AND (P2.QACTIVITE_12 EQ 112) AND (P2.AP12JOUR EQ 5)) [P2.VENDREDIAP3 = P2.AP3];
IF ((P2.3SUMAP3 GE 200) AND (P2.3SUMAP3 LT 300) AND (P2.QACTIVITE3 EQ 103) AND (P2.AP3JOUR EQ 6)) [P2.SAMEDIAP3 = P2.AP3];
IF ((P2.4SUMAP3 GE 200) AND (P2.4SUMAP3 LT 300) AND (P2.QACTIVITE4 EQ 104) AND (P2.AP4JOUR EQ 6)) [P2.SAMEDIAP3 = P2.AP3];
IF ((P2.5SUMAP3 GE 200) AND (P2.5SUMAP3 LT 300) AND (P2.QACTIVITE5 EQ 105) AND (P2.AP5JOUR EQ 6)) [P2.SAMEDIAP3 = P2.AP3];
IF ((P2.6SUMAP3 GE 200) AND (P2.6SUMAP3 LT 300) AND (P2.QACTIVITE6 EQ 106) AND (P2.AP6JOUR EQ 6)) [P2.SAMEDIAP3 = P2.AP3];
IF ((P2.7SUMAP3 GE 200) AND (P2.7SUMAP3 LT 300) AND (P2.QACTIVITE7 EQ 107) AND (P2.AP7JOUR EQ 6)) [P2.SAMEDIAP3 = P2.AP3];
IF ((P2.8SUMAP3 GE 200) AND (P2.8SUMAP3 LT 300) AND (P2.QACTIVITE8 EQ 108) AND (P2.AP8JOUR EQ 6)) [P2.SAMEDIAP3 = P2.AP3];
IF ((P2.9SUMAP3 GE 200) AND (P2.9SUMAP3 LT 300) AND (P2.QACTIVITE9 EQ 109) AND (P2.AP9JOUR EQ 6)) [P2.SAMEDIAP3 = P2.AP3];
IF ((P2.10SUMAP3 GE 200) AND (P2.10SUMAP3 LT 300) AND (P2.QACTIVITE_10 EQ 110) AND (P2.AP10JOUR EQ 6)) [P2.SAMEDIAP3 = P2.AP3];
IF ((P2.11SUMAP3 GE 200) AND (P2.11SUMAP3 LT 300) AND (P2.QACTIVITE_11 EQ 111) AND (P2.AP11JOUR EQ 6)) [P2.SAMEDIAP3 = P2.AP3];
IF ((P2.12SUMAP3 GE 200) AND (P2.12SUMAP3 LT 300) AND (P2.QACTIVITE_12 EQ 112) AND (P2.AP12JOUR EQ 6)) [P2.SAMEDIAP3 = P2.AP3];
IF ((P2.3SUMAP3 GE 200) AND (P2.3SUMAP3 LT 300) AND (P2.QACTIVITE3 EQ 103) AND (P2.AP3JOUR EQ 7)) [P2.DIMANCHEAP3 = P2.AP3];
IF ((P2.4SUMAP3 GE 200) AND (P2.4SUMAP3 LT 300) AND (P2.QACTIVITE4 EQ 104) AND (P2.AP4JOUR EQ 7)) [P2.DIMANCHEAP3 = P2.AP3];
IF ((P2.5SUMAP3 GE 200) AND (P2.5SUMAP3 LT 300) AND (P2.QACTIVITE5 EQ 105) AND (P2.AP5JOUR EQ 7)) [P2.DIMANCHEAP3 = P2.AP3];
IF ((P2.6SUMAP3 GE 200) AND (P2.6SUMAP3 LT 300) AND (P2.QACTIVITE6 EQ 106) AND (P2.AP6JOUR EQ 7)) [P2.DIMANCHEAP3 = P2.AP3];
IF ((P2.7SUMAP3 GE 200) AND (P2.7SUMAP3 LT 300) AND (P2.QACTIVITE7 EQ 107) AND (P2.AP7JOUR EQ 7)) [P2.DIMANCHEAP3 = P2.AP3];
IF ((P2.8SUMAP3 GE 200) AND (P2.8SUMAP3 LT 300) AND (P2.QACTIVITE8 EQ 108) AND (P2.AP8JOUR EQ 7)) [P2.DIMANCHEAP3 = P2.AP3];
IF ((P2.9SUMAP3 GE 200) AND (P2.9SUMAP3 LT 300) AND (P2.QACTIVITE9 EQ 109) AND (P2.AP9JOUR EQ 7)) [P2.DIMANCHEAP3 = P2.AP3];

IF ((P2.10SUMAP3 GE 200) AND (P2.10SUMAP3 LT 300) AND (P2.QACTIVITE_10 EQ 110) AND (P2.AP10JOUR EQ 7)) [P2.DIMANCHEAP3 = P2.AP3];
IF ((P2.11SUMAP3 GE 200) AND (P2.11SUMAP3 LT 300) AND (P2.QACTIVITE_11 EQ 111) AND (P2.AP11JOUR EQ 7)) [P2.DIMANCHEAP3 = P2.AP3];
IF ((P2.12SUMAP3 GE 200) AND (P2.12SUMAP3 LT 300) AND (P2.QACTIVITE_12 EQ 112) AND (P2.AP12JOUR EQ 7)) [P2.DIMANCHEAP3 = P2.AP3];
POURSUITE DU CODE
IF (P2.P7BARCHOICE EQ 101) [P2.BAR = 'Être trop fatigué ou fatiguée pour faire de l´activité physique'];
IF (P2.P7BARCHOICE EQ 102) [P2.BAR = 'Avoir beaucoup de choses à faire mis à part l´activité physique'];
IF (P2.P7BARCHOICE EQ 103) [P2.BAR = 'température sera trop mauvaise pour faire de l´activité physique'];
IF (P2.P7BARCHOICE EQ 104) [P2.BAR = 'Ne pas avoir personne avec qui faire de l´activité physique'];
IF (P2.P7BARCHOICE EQ 105) [P2.BAR = 'Ne pas avoir accès à des emplacements pour faire de l´activité physique'];
IF (P2.P7BARCHOICE EQ 106) [P2.BAR = 'Ne pas avoir d´argent pour faire de l´activité physique'];
IF (P2.P7BARCHOICE EQ 107) [P2.BAR = 'Être trop gêné ou gênée, manquer de confiance pour aller faire de l´activité physique'];
IF (P2.P7BARCHOICE EQ 108) [P2.BAR = 'Avoir peur d´être insuffisamment en santé pour faire de l´activité physique'];
IF (P2.P7BARCHOICE EQ 109) [P2.BAR = 'Manquer de motivation'];
IF ((P2.P7BARCHOICE EQ 110) AND (P2.Q_BARANSWER NE 0)) [P2.BAR = P2.Q_BARANSWER];
IF (P2.SUMSOL EQ 101) [P2.SOL = 'Je vais faire de l´activité tôt dans la journée, en matinée ou à l´heure du midi.'];
IF (P2.SUMSOL EQ 102) [P2.SOL = 'J´essaie de garder en tête que si je fais de l´activité physique, j´aurai de plus en plus d´énergie dans la vie.'];
IF (P2.SUMSOL EQ 103) [P2.SOL = 'Je vais planifier des activités avec d´autres personnes qui me motiveront.'];
IF (P2.SUMSOL EQ 104) [P2.SOL = 'Je prends une collation pour me donner de l´énergie et j´y vais quand même.'];
IF (P2.SUMSOL EQ 201) [P2.SOL = 'Faire de l´activité physique par bloc de 10 minutes les journées où je n´ai pas de temps.'];
IF (P2.SUMSOL EQ 202) [P2.SOL = 'Je vais remplacer des activités où je suis assis ou assise, télé, ordinateur ou autres, par des activités physiques.'];
IF (P2.SUMSOL EQ 203) [P2.SOL = 'Je vais me procurer un agenda et inscrire dans mon horaire les moments où je vais faire de l´activité physique.'];
IF (P2.SUMSOL EQ 204) [P2.SOL = 'Je vais prendre des pauses de travail active et faire des 10 minutes de marche.'];
IF (P2.SUMSOL EQ 205) [P2.SOL = 'Je vais me fixer des moments avec d´autres personnes qui me plaisent pour aller faire de l´activité physique.'];
IF (P2.SUMSOL EQ 301) [P2.SOL = 'Je vais me procurer l´équipement nécessaire pour en faire même quand il pleut, quand il neige ou quand il fait trop chaud.'];
IF (P2.SUMSOL EQ 302) [P2.SOL = 'Je vais faire de l'activité physique chez nous ou à l´intérieur dans un endroit que j´aurai choisi quand il fait moins beau.'];

IF (P2.SUMSOL EQ 303) [P2.SOL = 'Je vais me préparer une activité physique alternative au cas où il serait désagréable d´aller dehors.'];
IF (P2.SUMSOL EQ 304) [P2.SOL = 'Je vais me dresser une liste des activités que je serais prêtE à faire si la température est mauvaise.'];
IF (P2.SUMSOL EQ 401) [P2.SOL = 'Je vais me joindre à un groupe qui pratique l´activité physique que j´aime. Ex : la marche, l´aquaforme ou la natation.'];
IF (P2.SUMSOL EQ 402) [P2.SOL = 'Je vais inviter mes amis, ma famille ou mes collègues à faire de l´activité physique avec moi.'];
IF (P2.SUMSOL EQ 403) [P2.SOL = 'Je vais amener mon chien marcher avec moi.'];
IF (P2.SUMSOL EQ 404) [P2.SOL = 'Je vais participer dans des discussions en ligne sur des pages facebook : Diabète Québec et autres.'];
IF (P2.SUMSOL EQ 501) [P2.SOL = 'Je vais faire de la marche ou du vélo dehors, c´est simple et gratuit.'];
IF (P2.SUMSOL EQ 502) [P2.SOL = 'Je vais faire de l´activité physique dans ma maison comme du yoga, un programme sur DVD, vélo stationnaire, tapis roulant, etc.'];
IF (P2.SUMSOL EQ 503) [P2.SOL = 'Je vais aller voir au centre communautaire de mon quartier pour me donner des options.'];
IF (P2.SUMSOL EQ 504) [P2.SOL = 'Je vais discuter avec mon médecin, avec un ami ou un spécialiste de l´activité physique pour avoir des conseils.'];
IF (P2.SUMSOL EQ 601) [P2.SOL = 'Je vais trouver des activités physiques abordables et simples : marcher dehors, faire du vélo, nager à la piscine communautaire.'];
IF (P2.SUMSOL EQ 602) [P2.SOL = 'Je vais aller voir au centre communautaire de mon quartier pour me donner des options.'];
IF (P2.SUMSOL EQ 603) [P2.SOL = 'Je vais économiser pour m´acheter un DVD d´activité physique ou pour une autre activité physique que j´aimerais faire.'];
IF (P2.SUMSOL EQ 604) [P2.SOL = 'Je vais aller marcher avec ma famille, des amis ou des collègues.'];
IF (P2.SUMSOL EQ 701) [P2.SOL = 'Je vais choisir une activité physique simple comme la marche ou la bicyclette.'];
IF (P2.SUMSOL EQ 702) [P2.SOL = 'Je vais aller faire de l´activité physique avec des gens qui m´acceptent comme je suis, qui évitent de me juger.'];
IF (P2.SUMSOL EQ 703) [P2.SOL = 'Au départ, je vais faire de l´activité physique dans des endroits où je suis seul[LETTRE_E] pour prendre confiance.'];
IF (P2.SUMSOL EQ 704) [P2.SOL = 'Je vais éviter des endroits qui me gêne davantage comme les centres de conditionnement.'];
IF (P2.SUMSOL EQ 801) [P2.SOL = 'Je vais consulter mon médecin pour mettre au clair si je peux faire de l´activité physique.'];
IF (P2.SUMSOL EQ 802) [P2.SOL = 'Je vais consulter un spécialiste de l´activité physique pour qu´il me rassure et me conseille sur ce qui est sécuritaire pour moi.'];
IF (P2.SUMSOL EQ 803) [P2.SOL = 'Je vais commencer à petite dose. 10 à 20 minutes maximum d´activités physiques les jours où je suis actif, active.'];
IF (P2.SUMSOL EQ 804) [P2.SOL = 'Je vais faire de l´activité physique accompagnéE d´un spécialiste de l´activité physique.'];

IF (P2.SUMSOL EQ 805) [P2.SOL = 'Je vais faire de l´activité physique dans un groupe où un spécialiste de l´activité physique est présent.'];
IF (P2.SUMSOL EQ 901) [P2.SOL = 'Je vais signer un contrat papier personnel comme quoi je m´engage à être actif, active.'];
IF (P2.SUMSOL EQ 902) [P2.SOL = 'Je vais signer un contrat papier avec mes proches comme quoi je m´engage à être actif, active.'];
IF (P2.SUMSOL EQ 903) [P2.SOL = 'Je vais essayer de nouvelles activités physiques pour trouver celles qui me plaisent vraiment.'];
IF (P2.SUMSOL EQ 904) [P2.SOL = 'À chaque activité physique que je fais, je prends un temps pour me rappeler ce que cela va m´apporter de mieux dans ma vie.'];
IF (P2.SUMSOL EQ 905) [P2.SOL = 'Je vais faire de l´activité physique avec des gens que j´aime pour me motiver.'];
IF ((P2.SUMSOL EQ 999) AND (P2.P8QSOLOPEN NE 0)) [P2.SOL = P2.P8QSOLOPEN];
IF (P2.SUMSOL EQ 0) [P2.SOL = P2.P8QSOL_10];
PLAN DE LA semaine 3
formules p3
IF ((P3.QACTI12 EQ 112) AND (P3.QAOPEN NE 0)) [P3.QACTIVITE_12 = 112];
[P3.2SUMAP2 = P3.QACTIVITE1];
[P3.3SUMAP2 = P3.QACTIVITE1+P3.QACTIVITE2];
[P3.4SUMAP2 = P3.QACTIVITE1+P3.QACTIVITE2+P3.QACTIVITE3];
[P3.5SUMAP2 = P3.QACTIVITE1+P3.QACTIVITE2+P3.QACTIVITE3+P3.QACTIVITE4];
[P3.6SUMAP2 = P3.QACTIVITE1+P3.QACTIVITE2+P3.QACTIVITE3+P3.QACTIVITE4+P3.QACTIVITE5];
[P3.7SUMAP2 =
P3.QACTIVITE1+P3.QACTIVITE2+P3.QACTIVITE3+P3.QACTIVITE4+P3.QACTIVITE5+P3.QACTIVITE6];
[P3.8SUMAP2 =
P3.QACTIVITE1+P3.QACTIVITE2+P3.QACTIVITE3+P3.QACTIVITE4+P3.QACTIVITE5+P3.QACTIVITE6+P3.QACTIVITE7];
[P3.9SUMAP2 =
P3.QACTIVITE1+P3.QACTIVITE2+P3.QACTIVITE3+P3.QACTIVITE4+P3.QACTIVITE5+P3.QACTIVITE6+P3.QACTIVITE7+P3.QACTIVITE8];
[P3.10SUMAP2 =
P3.QACTIVITE1+P3.QACTIVITE2+P3.QACTIVITE3+P3.QACTIVITE4+P3.QACTIVITE5+P3.QACTIVITE6+P3.QACTIVITE7+P3.QACTIVITE8+P3.QACTIVITE9];
[P3.11SUMAP2 =
P3.QACTIVITE1+P3.QACTIVITE2+P3.QACTIVITE3+P3.QACTIVITE4+P3.QACTIVITE5+P3.QACTIVITE6+P3.QACTIVITE7+P3.QACTIVITE8+P3.QACTIVITE9+P3.QACTIVITE_10];
[P3.12SUMAP2 =
P3.QACTIVITE1+P3.QACTIVITE2+P3.QACTIVITE3+P3.QACTIVITE4+P3.QACTIVITE5+P3.QACTIVITE6+P3.QACTIVITE7+P3.QACTIVITE8+P3.QACTIVITE9+P3.QACTIVITE_10+P3.QACTIVITE_11];
[P3.3SUMAP3 = P3.QACTIVITE1+P3.QACTIVITE2];
[P3.4SUMAP3 = P3.QACTIVITE1+P3.QACTIVITE2+P3.QACTIVITE3];
[P3.5SUMAP3 = P3.QACTIVITE1+P3.QACTIVITE2+P3.QACTIVITE3+P3.QACTIVITE4];

[P3.6SUMAP3 = P3.QACTIVITE1+P3.QACTIVITE2+P3.QACTIVITE3+P3.QACTIVITE4+P3.QACTIVITE5];
[P3.7SUMAP3 =
P3.QACTIVITE1+P3.QACTIVITE2+P3.QACTIVITE3+P3.QACTIVITE4+P3.QACTIVITE5+P3.QACTIVITE6];
[P3.8SUMAP3 =
P3.QACTIVITE1+P3.QACTIVITE2+P3.QACTIVITE3+P3.QACTIVITE4+P3.QACTIVITE5+P3.QACTIVITE6+P3.QACTIVITE7];
[P3.9SUMAP3 =
P3.QACTIVITE1+P3.QACTIVITE2+P3.QACTIVITE3+P3.QACTIVITE4+P3.QACTIVITE5+P3.QACTIVITE6+P3.QACTIVITE7+P3.QACTIVITE8];
[P3.10SUMAP3 =
P3.QACTIVITE1+P3.QACTIVITE2+P3.QACTIVITE3+P3.QACTIVITE4+P3.QACTIVITE5+P3.QACTIVITE6+P3.QACTIVITE7+P3.QACTIVITE8+P3.QACTIVITE9];
[P3.11SUMAP3 =
P3.QACTIVITE1+P3.QACTIVITE2+P3.QACTIVITE3+P3.QACTIVITE4+P3.QACTIVITE5+P3.QACTIVITE6+P3.QACTIVITE7+P3.QACTIVITE8+P3.QACTIVITE9+P3.QACTIVITE_10];
[P3.12SUMAP3 =
P3.QACTIVITE1+P3.QACTIVITE2+P3.QACTIVITE3+P3.QACTIVITE4+P3.QACTIVITE5+P3.QACTIVITE6+P3.QACTIVITE7+P3.QACTIVITE8+P3.QACTIVITE9+P3.QACTIVITE_10+P3.QACTIVITE_11];
[P3.SUMAP =
P3.QACTIVITE1+P3.QACTIVITE2+P3.QACTIVITE3+P3.QACTIVITE4+P3.QACTIVITE5+P3.QACTIVITE6+P3.QACTIVITE7+P3.QACTIVITE8+P3.QACTIVITE9+P3.QACTIVITE_10+P3.QACTIVITE_11+P3.QACTIVITE_12];
[P3.SUMSOL =
P3.P8QSOL1+P3.P8QSOL2+P3.P8QSOL3+P3.P8QSOL4+P3.P8QSOL5+P3.P8QSOL6+P3.P8QSOL7+P3.P8QSOL8+P3.P8QSOL9];
IF (P3.QACTIVITE1 EQ 101) [P3.AP1 = 'marche rapide'];
IF ((P3.QACTIVITE1 NE 101) AND (P3.QACTIVITE2 EQ 102)) [P3.AP1 = 'raquettes à neige'];
IF ((P3.QACTIVITE1 NE 101) AND (P3.QACTIVITE2 NE 102) AND (P3.QACTIVITE3 EQ 103)) [P3.AP1 = 'vélo'];
IF ((P3.QACTIVITE1 NE 101) AND (P3.QACTIVITE2 NE 102) AND (P3.QACTIVITE3 NE 103) AND (P3.QACTIVITE4 EQ 104)) [P3.AP1 = 'natation'];
IF ((P3.QACTIVITE1 NE 101) AND (P3.QACTIVITE2 NE 102) AND (P3.QACTIVITE3 NE 103) AND (P3.QACTIVITE4 NE 104) AND (P3.QACTIVITE5 EQ 105)) [P3.AP1 = 'ski de fond'];
IF ((P3.QACTIVITE1 NE 101) AND (P3.QACTIVITE2 NE 102) AND (P3.QACTIVITE3 NE 103) AND (P3.QACTIVITE4 NE 104) AND (P3.QACTIVITE5 NE 105) AND (P3.QACTIVITE6 EQ 106)) [P3.AP1 = 'tennis'];
IF ((P3.QACTIVITE1 NE 101) AND (P3.QACTIVITE2 NE 102) AND (P3.QACTIVITE3 NE 103) AND (P3.QACTIVITE4 NE 104) AND (P3.QACTIVITE5 NE 105) AND (P3.QACTIVITE6 NE 106) AND (P3.QACTIVITE7 EQ 107)) [P3.AP1 = 'course à pieds'];
IF ((P3.QACTIVITE1 NE 101) AND (P3.QACTIVITE2 NE 102) AND (P3.QACTIVITE3 NE 103) AND (P3.QACTIVITE4 NE 104) AND (P3.QACTIVITE5 NE 105) AND (P3.QACTIVITE6 NE 106) AND (P3.QACTIVITE7 NE 107) AND (P3.QACTIVITE8 EQ 108)) [P3.AP1 = 'hockey'];
IF ((P3.QACTIVITE1 NE 101) AND (P3.QACTIVITE2 NE 102) AND (P3.QACTIVITE3 NE 103) AND (P3.QACTIVITE4 NE 104) AND (P3.QACTIVITE5 NE 105) AND (P3.QACTIVITE6 NE 106) AND (P3.QACTIVITE7 NE 107) AND (P3.QACTIVITE8 NE 108) AND (P3.QACTIVITE9 EQ 109)) [P3.AP1 = 'basketball'];

IF ((P3.QACTIVITE1 NE 101) AND (P3.QACTIVITE2 NE 102) AND (P3.QACTIVITE3 NE 103) AND (P3.QACTIVITE4 NE 104) AND (P3.QACTIVITE5 NE 105) AND (P3.QACTIVITE6 NE 106) AND (P3.QACTIVITE7 NE 107) AND (P3.QACTIVITE8 NE 108) AND (P3.QACTIVITE9 NE 109) AND (P3.QACTIVITE_10 EQ 110)) [P3.AP1 = 'soccer'];
IF ((P3.QACTIVITE1 NE 101) AND (P3.QACTIVITE2 NE 102) AND (P3.QACTIVITE3 NE 103) AND (P3.QACTIVITE4 NE 104) AND (P3.QACTIVITE5 NE 105) AND (P3.QACTIVITE6 NE 106) AND (P3.QACTIVITE7 NE 107) AND (P3.QACTIVITE8 NE 108) AND (P3.QACTIVITE9 NE 109) AND (P3.QACTIVITE_10 NE 110) AND (P3.QACTIVITE_11 EQ 111)) [P3.AP1 = 'centre de conditionnement'];
IF ((P3.QACTIVITE1 NE 101) AND (P3.QACTIVITE2 NE 102) AND (P3.QACTIVITE3 NE 103) AND (P3.QACTIVITE4 NE 104) AND (P3.QACTIVITE5 NE 105) AND (P3.QACTIVITE6 NE 106) AND (P3.QACTIVITE7 NE 107) AND (P3.QACTIVITE8 NE 108) AND (P3.QACTIVITE9 NE 109) AND (P3.QACTIVITE_10 NE 110) AND (P3.QACTIVITE_11 NE 111) AND (P3.QACTIVITE_12 EQ 112)) [P3.AP1 = P3.QAOPEN];
IF ((P3.2SUMAP2 GE 100) AND (P3.2SUMAP2 LT 200) AND (P3.QACTIVITE2 EQ 102)) [P3.AP2 = 'raquettes à neige'];
IF ((P3.3SUMAP2 GE 100) AND (P3.3SUMAP2 LT 200) AND (P3.QACTIVITE3 EQ 103)) [P3.AP2 = 'vélo'];
IF ((P3.4SUMAP2 GE 100) AND (P3.4SUMAP2 LT 200) AND (P3.QACTIVITE4 EQ 104)) [P3.AP2 = 'natation'];
IF ((P3.5SUMAP2 GE 100) AND (P3.5SUMAP2 LT 200) AND (P3.QACTIVITE5 EQ 105)) [P3.AP2 = 'ski de fond'];
IF ((P3.6SUMAP2 GE 100) AND (P3.6SUMAP2 LT 200) AND (P3.QACTIVITE6 EQ 106)) [P3.AP2 = 'tennis'];
IF ((P3.7SUMAP2 GE 100) AND (P3.7SUMAP2 LT 200) AND (P3.QACTIVITE7 EQ 107)) [P3.AP2 = 'course à pieds'];
IF ((P3.8SUMAP2 GE 100) AND (P3.8SUMAP2 LT 200) AND (P3.QACTIVITE8 EQ 108)) [P3.AP2 = 'hockey'];
IF ((P3.9SUMAP2 GE 100) AND (P3.9SUMAP2 LT 200) AND (P3.QACTIVITE9 EQ 109)) [P3.AP2 = 'basketball'];
IF ((P3.10SUMAP2 GE 100) AND (P3.10SUMAP2 LT 200) AND (P3.QACTIVITE_10 EQ 110)) [P3.AP2 = 'soccer'];
IF ((P3.11SUMAP2 GE 100) AND (P3.11SUMAP2 LT 200) AND (P3.QACTIVITE_11 EQ 111)) [P3.AP2 = 'centre de conditionnement'];
IF ((P3.12SUMAP2 GE 100) AND (P3.12SUMAP2 LT 200) AND (P3.QACTIVITE_12 EQ 112)) [P3.AP2 = P3.QAOPEN];
IF ((P3.3SUMAP3 GE 200) AND (P3.3SUMAP3 LT 300) AND (P3.QACTIVITE3 EQ 103)) [P3.AP3 = 'vélo'];
IF ((P3.4SUMAP3 GE 200) AND (P3.4SUMAP3 LT 300) AND (P3.QACTIVITE4 EQ 104)) [P3.AP3 = 'natation'];
IF ((P3.5SUMAP3 GE 200) AND (P3.5SUMAP3 LT 300) AND (P3.QACTIVITE5 EQ 105)) [P3.AP3 = 'ski de fond'];
IF ((P3.6SUMAP3 GE 200) AND (P3.6SUMAP3 LT 300) AND (P3.QACTIVITE6 EQ 106)) [P3.AP3 = 'tennis'];
IF ((P3.7SUMAP3 GE 200) AND (P3.7SUMAP3 LT 300) AND (P3.QACTIVITE7 EQ 107)) [P3.AP3 = 'course à pieds'];
IF ((P3.8SUMAP3 GE 200) AND (P3.8SUMAP3 LT 300) AND (P3.QACTIVITE8 EQ 108)) [P3.AP3 = 'hockey'];
IF ((P3.9SUMAP3 GE 200) AND (P3.9SUMAP3 LT 300) AND (P3.QACTIVITE9 EQ 109)) [P3.AP3 = 'basketball'];
IF ((P3.10SUMAP3 GE 200) AND (P3.10SUMAP3 LT 300) AND (P3.QACTIVITE_10 EQ 110)) [P3.AP3 = 'soccer'];
IF ((P3.11SUMAP3 GE 200) AND (P3.11SUMAP3 LT 300) AND (P3.QACTIVITE_11 EQ 111)) [P3.AP3 = 'centre de conditionnement'];
IF ((P3.12SUMAP3 GE 200) AND (P3.12SUMAP3 LT 300) AND (P3.QACTIVITE_12 EQ 112)) [P3.AP3 = P3.QAOPEN];

ACTIVITÉ 1 DANS LE PLAN POUR CHAQUE JOUR DE LA SEMAINE
IF ((P3.QACTIVITE1 EQ 101) AND (P3.AP1JOUR EQ 1)) [P3.LUNDIAP1 = P3.AP1];
IF ((P3.QACTIVITE1 NE 101) AND (P3.QACTIVITE2 EQ 102) AND (P3.AP2JOUR EQ 1)) [P3.LUNDIAP1 = P3.AP1];
IF ((P3.QACTIVITE1 NE 101) AND (P3.QACTIVITE2 NE 102) AND (P3.QACTIVITE3 EQ 103) AND (P3.AP3JOUR EQ 1)) [P3.LUNDIAP1 = P3.AP1];
IF ((P3.QACTIVITE1 NE 101) AND (P3.QACTIVITE2 NE 102) AND (P3.QACTIVITE3 NE 103) AND (P3.QACTIVITE4 EQ 104) AND (P3.AP4JOUR EQ 1)) [P3.LUNDIAP1 = P3.AP1];
IF ((P3.QACTIVITE1 NE 101) AND (P3.QACTIVITE2 NE 102) AND (P3.QACTIVITE3 NE 103) AND (P3.QACTIVITE4 NE 104) AND (P3.QACTIVITE5 EQ 105) AND (P3.AP5JOUR EQ 1)) [P3.LUNDIAP1 = P3.AP1];
IF ((P3.QACTIVITE1 NE 101) AND (P3.QACTIVITE2 NE 102) AND (P3.QACTIVITE3 NE 103) AND (P3.QACTIVITE4 NE 104) AND (P3.QACTIVITE5 NE 105) AND (P3.QACTIVITE6 EQ 106) AND (P3.AP6JOUR EQ 1)) [P3.LUNDIAP1 = P3.AP1];
IF ((P3.QACTIVITE1 NE 101) AND (P3.QACTIVITE2 NE 102) AND (P3.QACTIVITE3 NE 103) AND (P3.QACTIVITE4 NE 104) AND (P3.QACTIVITE5 NE 105) AND (P3.QACTIVITE6 NE 106) AND (P3.QACTIVITE7 EQ 107) AND (P3.AP7JOUR EQ 1)) [P3.LUNDIAP1 = P3.AP1];
IF ((P3.QACTIVITE1 NE 101) AND (P3.QACTIVITE2 NE 102) AND (P3.QACTIVITE3 NE 103) AND (P3.QACTIVITE4 NE 104) AND (P3.QACTIVITE5 NE 105) AND (P3.QACTIVITE6 NE 106) AND (P3.QACTIVITE7 NE 107) AND (P3.QACTIVITE8 EQ 108) AND (P3.AP8JOUR EQ 1)) [P3.LUNDIAP1 = P3.AP1];
IF ((P3.QACTIVITE1 NE 101) AND (P3.QACTIVITE2 NE 102) AND (P3.QACTIVITE3 NE 103) AND (P3.QACTIVITE4 NE 104) AND (P3.QACTIVITE5 NE 105) AND (P3.QACTIVITE6 NE 106) AND (P3.QACTIVITE7 NE 107) AND (P3.QACTIVITE8 NE 108) AND (P3.QACTIVITE9 EQ 109) AND (P3.AP9JOUR EQ 1)) [P3.LUNDIAP1 = P3.AP1];
IF ((P3.QACTIVITE1 NE 101) AND (P3.QACTIVITE2 NE 102) AND (P3.QACTIVITE3 NE 103) AND (P3.QACTIVITE4 NE 104) AND (P3.QACTIVITE5 NE 105) AND (P3.QACTIVITE6 NE 106) AND (P3.QACTIVITE7 NE 107) AND (P3.QACTIVITE8 NE 108) AND (P3.QACTIVITE9 NE 109) AND (P3.QACTIVITE_10 EQ 110) AND (P3.AP10JOUR EQ 1)) [P3.LUNDIAP1 = P3.AP1];
IF ((P3.QACTIVITE1 NE 101) AND (P3.QACTIVITE2 NE 102) AND (P3.QACTIVITE3 NE 103) AND (P3.QACTIVITE4 NE 104) AND (P3.QACTIVITE5 NE 105) AND (P3.QACTIVITE6 NE 106) AND (P3.QACTIVITE7 NE 107) AND (P3.QACTIVITE8 NE 108) AND (P3.QACTIVITE9 NE 109) AND (P3.QACTIVITE_10 NE 110) AND (P3.QACTIVITE_11 EQ 111) AND (P3.AP11JOUR EQ 1)) [P3.LUNDIAP1 = P3.AP1];
IF ((P3.QACTIVITE1 NE 101) AND (P3.QACTIVITE2 NE 102) AND (P3.QACTIVITE3 NE 103) AND (P3.QACTIVITE4 NE 104) AND (P3.QACTIVITE5 NE 105) AND (P3.QACTIVITE6 NE 106) AND (P3.QACTIVITE7 NE 107) AND (P3.QACTIVITE8 NE 108) AND (P3.QACTIVITE9 NE 109) AND (P3.QACTIVITE_10 NE 110) AND (P3.QACTIVITE_11 NE 111) AND (P3.QACTIVITE_12 EQ 112) AND (P3.AP12JOUR EQ 1)) [P3.LUNDIAP1 = P3.AP1];
IF ((P3.QACTIVITE1 EQ 101) AND (P3.AP1JOUR EQ 2)) [P3.MARDIAP1 = P3.AP1];
IF ((P3.QACTIVITE1 NE 101) AND (P3.QACTIVITE2 EQ 102) AND (P3.AP2JOUR EQ 2)) [P3.MARDIAP1 = P3.AP1];
IF ((P3.QACTIVITE1 NE 101) AND (P3.QACTIVITE2 NE 102) AND (P3.QACTIVITE3 EQ 103) AND (P3.AP3JOUR EQ 2)) [P3.MARDIAP1 = P3.AP1];

IF ((P3.QACTIVITE1 NE 101) AND (P3.QACTIVITE2 NE 102) AND (P3.QACTIVITE3 NE 103) AND (P3.QACTIVITE4 EQ 104) AND (P3.AP4JOUR EQ 2)) [P3.MARDIAP1 = P3.AP1];
IF ((P3.QACTIVITE1 NE 101) AND (P3.QACTIVITE2 NE 102) AND (P3.QACTIVITE3 NE 103) AND (P3.QACTIVITE4 NE 104) AND (P3.QACTIVITE5 EQ 105) AND (P3.AP5JOUR EQ 2)) [P3.MARDIAP1 = P3.AP1];
IF ((P3.QACTIVITE1 NE 101) AND (P3.QACTIVITE2 NE 102) AND (P3.QACTIVITE3 NE 103) AND (P3.QACTIVITE4 NE 104) AND (P3.QACTIVITE5 NE 105) AND (P3.QACTIVITE6 EQ 106) AND (P3.AP6JOUR EQ 2)) [P3.MARDIAP1 = P3.AP1];
IF ((P3.QACTIVITE1 NE 101) AND (P3.QACTIVITE2 NE 102) AND (P3.QACTIVITE3 NE 103) AND (P3.QACTIVITE4 NE 104) AND (P3.QACTIVITE5 NE 105) AND (P3.QACTIVITE6 NE 106) AND (P3.QACTIVITE7 EQ 107) AND (P3.AP7JOUR EQ 2)) [P3.MARDIAP1 = P3.AP1];
IF ((P3.QACTIVITE1 NE 101) AND (P3.QACTIVITE2 NE 102) AND (P3.QACTIVITE3 NE 103) AND (P3.QACTIVITE4 NE 104) AND (P3.QACTIVITE5 NE 105) AND (P3.QACTIVITE6 NE 106) AND (P3.QACTIVITE7 NE 107) AND (P3.QACTIVITE8 EQ 108) AND (P3.AP8JOUR EQ 2)) [P3.MARDIAP1 = P3.AP1];
IF ((P3.QACTIVITE1 NE 101) AND (P3.QACTIVITE2 NE 102) AND (P3.QACTIVITE3 NE 103) AND (P3.QACTIVITE4 NE 104) AND (P3.QACTIVITE5 NE 105) AND (P3.QACTIVITE6 NE 106) AND (P3.QACTIVITE7 NE 107) AND (P3.QACTIVITE8 NE 108) AND (P3.QACTIVITE9 EQ 109) AND (P3.AP9JOUR EQ 2)) [P3.MARDIAP1 = P3.AP1];
IF ((P3.QACTIVITE1 NE 101) AND (P3.QACTIVITE2 NE 102) AND (P3.QACTIVITE3 NE 103) AND (P3.QACTIVITE4 NE 104) AND (P3.QACTIVITE5 NE 105) AND (P3.QACTIVITE6 NE 106) AND (P3.QACTIVITE7 NE 107) AND (P3.QACTIVITE8 NE 108) AND (P3.QACTIVITE9 NE 109) AND (P3.QACTIVITE_10 EQ 110) AND (P3.AP10JOUR EQ 2)) [P3.MARDIAP1 = P3.AP1];
IF ((P3.QACTIVITE1 NE 101) AND (P3.QACTIVITE2 NE 102) AND (P3.QACTIVITE3 NE 103) AND (P3.QACTIVITE4 NE 104) AND (P3.QACTIVITE5 NE 105) AND (P3.QACTIVITE6 NE 106) AND (P3.QACTIVITE7 NE 107) AND (P3.QACTIVITE8 NE 108) AND (P3.QACTIVITE9 NE 109) AND (P3.QACTIVITE_10 NE 110) AND (P3.QACTIVITE_11 EQ 111) AND (P3.AP11JOUR EQ 2)) [P3.MARDIAP1 = P3.AP1];
IF ((P3.QACTIVITE1 NE 101) AND (P3.QACTIVITE2 NE 102) AND (P3.QACTIVITE3 NE 103) AND (P3.QACTIVITE4 NE 104) AND (P3.QACTIVITE5 NE 105) AND (P3.QACTIVITE6 NE 106) AND (P3.QACTIVITE7 NE 107) AND (P3.QACTIVITE8 NE 108) AND (P3.QACTIVITE9 NE 109) AND (P3.QACTIVITE_10 NE 110) AND (P3.QACTIVITE_11 NE 111) AND (P3.QACTIVITE_12 EQ 112) AND (P3.AP12JOUR EQ 2)) [P3.MARDIAP1 = P3.AP1];
IF ((P3.QACTIVITE1 EQ 101) AND (P3.AP1JOUR EQ 3)) [P3.MERCREDIAP1 = P3.AP1];
IF ((P3.QACTIVITE1 NE 101) AND (P3.QACTIVITE2 EQ 102) AND (P3.AP2JOUR EQ 3)) [P3.MERCREDIAP1 = P3.AP1];
IF ((P3.QACTIVITE1 NE 101) AND (P3.QACTIVITE2 NE 102) AND (P3.QACTIVITE3 EQ 103) AND (P3.AP3JOUR EQ 3)) [P3.MERCREDIAP1 = P3.AP1];
IF ((P3.QACTIVITE1 NE 101) AND (P3.QACTIVITE2 NE 102) AND (P3.QACTIVITE3 NE 103) AND (P3.QACTIVITE4 EQ 104) AND (P3.AP4JOUR EQ 3)) [P3.MERCREDIAP1 = P3.AP1];
IF ((P3.QACTIVITE1 NE 101) AND (P3.QACTIVITE2 NE 102) AND (P3.QACTIVITE3 NE 103) AND (P3.QACTIVITE4 NE 104) AND (P3.QACTIVITE5 EQ 105) AND (P3.AP5JOUR EQ 3)) [P3.MERCREDIAP1 = P3.AP1];

IF ((P3.QACTIVITE1 NE 101) AND (P3.QACTIVITE2 NE 102) AND (P3.QACTIVITE3 NE 103) AND (P3.QACTIVITE4 NE 104) AND (P3.QACTIVITES5 NE 105) AND (P3.QACTIVITE6 EQ 106) AND (P3.AP6JOUR EQ 3)) [P3.MERCREDIAP1 = P3.AP1];
IF ((P3.QACTIVITE1 NE 101) AND (P3.QACTIVITE2 NE 102) AND (P3.QACTIVITE3 NE 103) AND (P3.QACTIVITE4 NE 104) AND (P3.QACTIVITES5 NE 105) AND (P3.QACTIVITE6 NE 106) AND (P3.QACTIVITE7 EQ 107) AND (P3.AP7JOUR EQ 3)) [P3.MERCREDIAP1 = P3.AP1];
IF ((P3.QACTIVITE1 NE 101) AND (P3.QACTIVITE2 NE 102) AND (P3.QACTIVITE3 NE 103) AND (P3.QACTIVITE4 NE 104) AND (P3.QACTIVITES5 NE 105) AND (P3.QACTIVITE6 NE 106) AND (P3.QACTIVITE7 NE 107) AND (P3.QACTIVITE8 EQ 108) AND (P3.AP8JOUR EQ 3)) [P3.MERCREDIAP1 = P3.AP1];
IF ((P3.QACTIVITE1 NE 101) AND (P3.QACTIVITE2 NE 102) AND (P3.QACTIVITE3 NE 103) AND (P3.QACTIVITE4 NE 104) AND (P3.QACTIVITES5 NE 105) AND (P3.QACTIVITE6 NE 106) AND (P3.QACTIVITE7 NE 107) AND (P3.QACTIVITE8 NE 108) AND (P3.QACTIVITE9 EQ 109) AND (P3.AP9JOUR EQ 3)) [P3.MERCREDIAP1 = P3.AP1];
IF ((P3.QACTIVITE1 NE 101) AND (P3.QACTIVITE2 NE 102) AND (P3.QACTIVITE3 NE 103) AND (P3.QACTIVITE4 NE 104) AND (P3.QACTIVITES5 NE 105) AND (P3.QACTIVITE6 NE 106) AND (P3.QACTIVITE7 NE 107) AND (P3.QACTIVITE8 NE 108) AND (P3.QACTIVITE9 NE 109) AND (P3.QACTIVITE_10 EQ 110) AND (P3.AP10JOUR EQ 3)) [P3.MERCREDIAP1 = P3.AP1];
IF ((P3.QACTIVITE1 NE 101) AND (P3.QACTIVITE2 NE 102) AND (P3.QACTIVITE3 NE 103) AND (P3.QACTIVITE4 NE 104) AND (P3.QACTIVITES5 NE 105) AND (P3.QACTIVITE6 NE 106) AND (P3.QACTIVITE7 NE 107) AND (P3.QACTIVITE8 NE 108) AND (P3.QACTIVITE9 NE 109) AND (P3.QACTIVITE_10 NE 110) AND (P3.QACTIVITE_11 EQ 111) AND (P3.AP11JOUR EQ 3)) [P3.MERCREDIAP1 = P3.AP1];
IF ((P3.QACTIVITE1 NE 101) AND (P3.QACTIVITE2 NE 102) AND (P3.QACTIVITE3 NE 103) AND (P3.QACTIVITE4 NE 104) AND (P3.QACTIVITES5 NE 105) AND (P3.QACTIVITE6 NE 106) AND (P3.QACTIVITE7 NE 107) AND (P3.QACTIVITE8 NE 108) AND (P3.QACTIVITE9 NE 109) AND (P3.QACTIVITE_10 NE 110) AND (P3.QACTIVITE_11 NE 111) AND (P3.QACTIVITE_12 EQ 112) AND (P3.AP12JOUR EQ 3)) [P3.MERCREDIAP1 = P3.AP1];
IF ((P3.QACTIVITE1 EQ 101) AND (P3.AP1JOUR EQ 4)) [P3.JEUDIAP1 = P3.AP1];
IF ((P3.QACTIVITE1 NE 101) AND (P3.QACTIVITE2 EQ 102) AND (P3.AP2JOUR EQ 4)) [P3.JEUDIAP1 = P3.AP1];
IF ((P3.QACTIVITE1 NE 101) AND (P3.QACTIVITE2 NE 102) AND (P3.QACTIVITE3 EQ 103) AND (P3.AP3JOUR EQ 4)) [P3.JEUDIAP1 = P3.AP1];
IF ((P3.QACTIVITE1 NE 101) AND (P3.QACTIVITE2 NE 102) AND (P3.QACTIVITE3 NE 103) AND (P3.QACTIVITE4 EQ 104) AND (P3.AP4JOUR EQ 4)) [P3.JEUDIAP1 = P3.AP1];
IF ((P3.QACTIVITE1 NE 101) AND (P3.QACTIVITE2 NE 102) AND (P3.QACTIVITE3 NE 103) AND (P3.QACTIVITE4 NE 104) AND (P3.QACTIVITES5 EQ 105) AND (P3.AP5JOUR EQ 4)) [P3.JEUDIAP1 = P3.AP1];
IF ((P3.QACTIVITE1 NE 101) AND (P3.QACTIVITE2 NE 102) AND (P3.QACTIVITE3 NE 103) AND (P3.QACTIVITE4 NE 104) AND (P3.QACTIVITES5 NE 105) AND (P3.QACTIVITE6 EQ 106) AND (P3.AP6JOUR EQ 4)) [P3.JEUDIAP1 = P3.AP1];
IF ((P3.QACTIVITE1 NE 101) AND (P3.QACTIVITE2 NE 102) AND (P3.QACTIVITE3 NE 103) AND (P3.QACTIVITE4 NE 104) AND (P3.QACTIVITES5 NE 105) AND (P3.QACTIVITE6 NE 106) AND (P3.QACTIVITE7 EQ 107) AND (P3.AP7JOUR EQ 4)) [P3.JEUDIAP1 = P3.AP1];

IF ((P3.QACTIVITE1 NE 101) AND (P3.QACTIVITE2 NE 102) AND (P3.QACTIVITE3 NE 103) AND (P3.QACTIVITE4 NE 104) AND (P3.QACTIVITE5 NE 105) AND (P3.QACTIVITE6 NE 106) AND (P3.QACTIVITE7 NE 107) AND (P3.QACTIVITE8 EQ 108) AND (P3.AP8JOUR EQ 4)) [P3.JEUDIAP1 = P3.AP1];
IF ((P3.QACTIVITE1 NE 101) AND (P3.QACTIVITE2 NE 102) AND (P3.QACTIVITE3 NE 103) AND (P3.QACTIVITE4 NE 104) AND (P3.QACTIVITE5 NE 105) AND (P3.QACTIVITE6 NE 106) AND (P3.QACTIVITE7 NE 107) AND (P3.QACTIVITE8 NE 108) AND (P3.QACTIVITE9 EQ 109) AND (P3.AP9JOUR EQ 4)) [P3.JEUDIAP1 = P3.AP1];
IF ((P3.QACTIVITE1 NE 101) AND (P3.QACTIVITE2 NE 102) AND (P3.QACTIVITE3 NE 103) AND (P3.QACTIVITE4 NE 104) AND (P3.QACTIVITE5 NE 105) AND (P3.QACTIVITE6 NE 106) AND (P3.QACTIVITE7 NE 107) AND (P3.QACTIVITE8 NE 108) AND (P3.QACTIVITE9 NE 109) AND (P3.QACTIVITE_10 EQ 110) AND (P3.AP10JOUR EQ 4)) [P3.JEUDIAP1 = P3.AP1];
IF ((P3.QACTIVITE1 NE 101) AND (P3.QACTIVITE2 NE 102) AND (P3.QACTIVITE3 NE 103) AND (P3.QACTIVITE4 NE 104) AND (P3.QACTIVITE5 NE 105) AND (P3.QACTIVITE6 NE 106) AND (P3.QACTIVITE7 NE 107) AND (P3.QACTIVITE8 NE 108) AND (P3.QACTIVITE9 NE 109) AND (P3.QACTIVITE_10 NE 110) AND (P3.QACTIVITE_11 EQ 111) AND (P3.AP11JOUR EQ 4)) [P3.JEUDIAP1 = P3.AP1];
IF ((P3.QACTIVITE1 NE 101) AND (P3.QACTIVITE2 NE 102) AND (P3.QACTIVITE3 NE 103) AND (P3.QACTIVITE4 NE 104) AND (P3.QACTIVITE5 NE 105) AND (P3.QACTIVITE6 NE 106) AND (P3.QACTIVITE7 NE 107) AND (P3.QACTIVITE8 NE 108) AND (P3.QACTIVITE9 NE 109) AND (P3.QACTIVITE_10 NE 110) AND (P3.QACTIVITE_11 NE 111) AND (P3.QACTIVITE_12 EQ 112) AND (P3.AP12JOUR EQ 4)) [P3.JEUDIAP1 = P3.AP1];
IF ((P3.QACTIVITE1 EQ 101) AND (P3.AP1JOUR EQ 5)) [P3.VENDREDIAP1 = P3.AP1];
IF ((P3.QACTIVITE1 NE 101) AND (P3.QACTIVITE2 EQ 102) AND (P3.AP2JOUR EQ 5)) [P3.VENDREDIAP1 = P3.AP1];
IF ((P3.QACTIVITE1 NE 101) AND (P3.QACTIVITE2 NE 102) AND (P3.QACTIVITE3 EQ 103) AND (P3.AP3JOUR EQ 5)) [P3.VENDREDIAP1 = P3.AP1];
IF ((P3.QACTIVITE1 NE 101) AND (P3.QACTIVITE2 NE 102) AND (P3.QACTIVITE3 NE 103) AND (P3.QACTIVITE4 EQ 104) AND (P3.AP4JOUR EQ 5)) [P3.VENDREDIAP1 = P3.AP1];
IF ((P3.QACTIVITE1 NE 101) AND (P3.QACTIVITE2 NE 102) AND (P3.QACTIVITE3 NE 103) AND (P3.QACTIVITE4 NE 104) AND (P3.QACTIVITE5 EQ 105) AND (P3.AP5JOUR EQ 5)) [P3.VENDREDIAP1 = P3.AP1];
IF ((P3.QACTIVITE1 NE 101) AND (P3.QACTIVITE2 NE 102) AND (P3.QACTIVITE3 NE 103) AND (P3.QACTIVITE4 NE 104) AND (P3.QACTIVITE5 NE 105) AND (P3.QACTIVITE6 EQ 106) AND (P3.AP6JOUR EQ 5)) [P3.VENDREDIAP1 = P3.AP1];
IF ((P3.QACTIVITE1 NE 101) AND (P3.QACTIVITE2 NE 102) AND (P3.QACTIVITE3 NE 103) AND (P3.QACTIVITE4 NE 104) AND (P3.QACTIVITE5 NE 105) AND (P3.QACTIVITE6 NE 106) AND (P3.QACTIVITE7 EQ 107) AND (P3.AP7JOUR EQ 5)) [P3.VENDREDIAP1 = P3.AP1];
IF ((P3.QACTIVITE1 NE 101) AND (P3.QACTIVITE2 NE 102) AND (P3.QACTIVITE3 NE 103) AND (P3.QACTIVITE4 NE 104) AND (P3.QACTIVITE5 NE 105) AND (P3.QACTIVITE6 NE 106) AND (P3.QACTIVITE7 NE 107) AND (P3.QACTIVITE8 EQ 108) AND (P3.AP8JOUR EQ 5)) [P3.VENDREDIAP1 = P3.AP1];
IF ((P3.QACTIVITE1 NE 101) AND (P3.QACTIVITE2 NE 102) AND (P3.QACTIVITE3 NE 103) AND (P3.QACTIVITE4 NE 104) AND (P3.QACTIVITE5 NE 105) AND (P3.QACTIVITE6 NE 106) AND (P3.QACTIVITE7

NE 107) AND (P3.QACTIVITE8 NE 108) AND (P3.QACTIVITE9 EQ 109) AND (P3.AP9JOUR EQ 5)) [P3.VENDREDIAP1 = P3.AP1];
IF ((P3.QACTIVITE1 NE 101) AND (P3.QACTIVITE2 NE 102) AND (P3.QACTIVITE3 NE 103) AND (P3.QACTIVITE4 NE 104) AND (P3.QACTIVITES5 NE 105) AND (P3.QACTIVITE6 NE 106) AND (P3.QACTIVITE7 NE 107) AND (P3.QACTIVITE8 NE 108) AND (P3.QACTIVITE9 NE 109) AND (P3.QACTIVITE_10 EQ 110) AND (P3.AP10JOUR EQ 5)) [P3.VENDREDIAP1 = P3.AP1];
IF ((P3.QACTIVITE1 NE 101) AND (P3.QACTIVITE2 NE 102) AND (P3.QACTIVITE3 NE 103) AND (P3.QACTIVITE4 NE 104) AND (P3.QACTIVITES5 NE 105) AND (P3.QACTIVITE6 NE 106) AND (P3.QACTIVITE7 NE 107) AND (P3.QACTIVITE8 NE 108) AND (P3.QACTIVITE9 NE 109) AND (P3.QACTIVITE_10 NE 110) AND (P3.QACTIVITE_11 EQ 111) AND (P3.AP11JOUR EQ 5)) [P3.VENDREDIAP1 = P3.AP1];
IF ((P3.QACTIVITE1 NE 101) AND (P3.QACTIVITE2 NE 102) AND (P3.QACTIVITE3 NE 103) AND (P3.QACTIVITE4 NE 104) AND (P3.QACTIVITES5 NE 105) AND (P3.QACTIVITE6 NE 106) AND (P3.QACTIVITE7 NE 107) AND (P3.QACTIVITE8 NE 108) AND (P3.QACTIVITE9 NE 109) AND (P3.QACTIVITE_10 NE 110) AND (P3.QACTIVITE_11 NE 111) AND (P3.QACTIVITE_12 EQ 112) AND (P3.AP12JOUR EQ 5)) [P3.VENDREDIAP1 = P3.AP1];
IF ((P3.QACTIVITE1 EQ 101) AND (P3.AP1JOUR EQ 6)) [P3.SAMEDIAP1 = P3.AP1];
IF ((P3.QACTIVITE1 NE 101) AND (P3.QACTIVITE2 EQ 102) AND (P3.AP2JOUR EQ 6)) [P3.SAMEDIAP1 = P3.AP1];
IF ((P3.QACTIVITE1 NE 101) AND (P3.QACTIVITE2 NE 102) AND (P3.QACTIVITE3 EQ 103) AND (P3.AP3JOUR EQ 6)) [P3.SAMEDIAP1 = P3.AP1];
IF ((P3.QACTIVITE1 NE 101) AND (P3.QACTIVITE2 NE 102) AND (P3.QACTIVITE3 NE 103) AND (P3.QACTIVITE4 EQ 104) AND (P3.AP4JOUR EQ 6)) [P3.SAMEDIAP1 = P3.AP1];
IF ((P3.QACTIVITE1 NE 101) AND (P3.QACTIVITE2 NE 102) AND (P3.QACTIVITE3 NE 103) AND (P3.QACTIVITE4 NE 104) AND (P3.QACTIVITES5 EQ 105) AND (P3.AP5JOUR EQ 6)) [P3.SAMEDIAP1 = P3.AP1];
IF ((P3.QACTIVITE1 NE 101) AND (P3.QACTIVITE2 NE 102) AND (P3.QACTIVITE3 NE 103) AND (P3.QACTIVITE4 NE 104) AND (P3.QACTIVITES5 NE 105) AND (P3.QACTIVITE6 EQ 106) AND (P3.AP6JOUR EQ 6)) [P3.SAMEDIAP1 = P3.AP1];
IF ((P3.QACTIVITE1 NE 101) AND (P3.QACTIVITE2 NE 102) AND (P3.QACTIVITE3 NE 103) AND (P3.QACTIVITE4 NE 104) AND (P3.QACTIVITES5 NE 105) AND (P3.QACTIVITE6 NE 106) AND (P3.QACTIVITE7 EQ 107) AND (P3.AP7JOUR EQ 6)) [P3.SAMEDIAP1 = P3.AP1];
IF ((P3.QACTIVITE1 NE 101) AND (P3.QACTIVITE2 NE 102) AND (P3.QACTIVITE3 NE 103) AND (P3.QACTIVITE4 NE 104) AND (P3.QACTIVITES5 NE 105) AND (P3.QACTIVITE6 NE 106) AND (P3.QACTIVITE7 NE 107) AND (P3.QACTIVITE8 EQ 108) AND (P3.AP8JOUR EQ 6)) [P3.SAMEDIAP1 = P3.AP1];
IF ((P3.QACTIVITE1 NE 101) AND (P3.QACTIVITE2 NE 102) AND (P3.QACTIVITE3 NE 103) AND (P3.QACTIVITE4 NE 104) AND (P3.QACTIVITES5 NE 105) AND (P3.QACTIVITE6 NE 106) AND (P3.QACTIVITE7 NE 107) AND (P3.QACTIVITE8 NE 108) AND (P3.QACTIVITE9 EQ 109) AND (P3.AP9JOUR EQ 6)) [P3.SAMEDIAP1 = P3.AP1];
IF ((P3.QACTIVITE1 NE 101) AND (P3.QACTIVITE2 NE 102) AND (P3.QACTIVITE3 NE 103) AND (P3.QACTIVITE4 NE 104) AND (P3.QACTIVITES5 NE 105) AND (P3.QACTIVITE6 NE 106) AND (P3.QACTIVITE7 NE 107) AND (P3.QACTIVITE8 NE 108) AND (P3.QACTIVITE9 NE 109) AND (P3.QACTIVITE_10 EQ 110) AND (P3.AP10JOUR EQ 6)) [P3.SAMEDIAP1 = P3.AP1];

IF ((P3.QACTIVITE1 NE 101) AND (P3.QACTIVITE2 NE 102) AND (P3.QACTIVITE3 NE 103) AND (P3.QACTIVITE4 NE 104) AND (P3.QACTIVITES5 NE 105) AND (P3.QACTIVITE6 NE 106) AND (P3.QACTIVITE7 NE 107) AND (P3.QACTIVITE8 NE 108) AND (P3.QACTIVITE9 NE 109) AND (P3.QACTIVITE_10 NE 110) AND (P3.QACTIVITE_11 EQ 111) AND (P3.AP11JOUR EQ 6)) [P3.SAMEDIAP1 = P3.AP1];
IF ((P3.QACTIVITE1 NE 101) AND (P3.QACTIVITE2 NE 102) AND (P3.QACTIVITE3 NE 103) AND (P3.QACTIVITE4 NE 104) AND (P3.QACTIVITES5 NE 105) AND (P3.QACTIVITE6 NE 106) AND (P3.QACTIVITE7 NE 107) AND (P3.QACTIVITE8 NE 108) AND (P3.QACTIVITE9 NE 109) AND (P3.QACTIVITE_10 NE 110) AND (P3.QACTIVITE_11 NE 111) AND (P3.QACTIVITE_12 EQ 112) AND (P3.AP12JOUR EQ 6)) [P3.SAMEDIAP1 = P3.AP1];
IF ((P3.QACTIVITE1 EQ 101) AND (P3.AP1JOUR EQ 7)) [P3.DIMANCHEAP1 = P3.AP1];
IF ((P3.QACTIVITE1 NE 101) AND (P3.QACTIVITE2 EQ 102) AND (P3.AP2JOUR EQ 7)) [P3.DIMANCHEAP1 = P3.AP1];
IF ((P3.QACTIVITE1 NE 101) AND (P3.QACTIVITE2 NE 102) AND (P3.QACTIVITE3 EQ 103) AND (P3.AP3JOUR EQ 7)) [P3.DIMANCHEAP1 = P3.AP1];
IF ((P3.QACTIVITE1 NE 101) AND (P3.QACTIVITE2 NE 102) AND (P3.QACTIVITE3 NE 103) AND (P3.QACTIVITE4 EQ 104) AND (P3.AP4JOUR EQ 7)) [P3.DIMANCHEAP1 = P3.AP1];
IF ((P3.QACTIVITE1 NE 101) AND (P3.QACTIVITE2 NE 102) AND (P3.QACTIVITE3 NE 103) AND (P3.QACTIVITE4 NE 104) AND (P3.QACTIVITES5 EQ 105) AND (P3.AP5JOUR EQ 7)) [P3.DIMANCHEAP1 = P3.AP1];
IF ((P3.QACTIVITE1 NE 101) AND (P3.QACTIVITE2 NE 102) AND (P3.QACTIVITE3 NE 103) AND (P3.QACTIVITE4 NE 104) AND (P3.QACTIVITES5 NE 105) AND (P3.QACTIVITE6 EQ 106) AND (P3.AP6JOUR EQ 7)) [P3.DIMANCHEAP1 = P3.AP1];
IF ((P3.QACTIVITE1 NE 101) AND (P3.QACTIVITE2 NE 102) AND (P3.QACTIVITE3 NE 103) AND (P3.QACTIVITE4 NE 104) AND (P3.QACTIVITES5 NE 105) AND (P3.QACTIVITE6 NE 106) AND (P3.QACTIVITE7 EQ 107) AND (P3.AP7JOUR EQ 7)) [P3.DIMANCHEAP1 = P3.AP1];
IF ((P3.QACTIVITE1 NE 101) AND (P3.QACTIVITE2 NE 102) AND (P3.QACTIVITE3 NE 103) AND (P3.QACTIVITE4 NE 104) AND (P3.QACTIVITES5 NE 105) AND (P3.QACTIVITE6 NE 106) AND (P3.QACTIVITE7 NE 107) AND (P3.QACTIVITE8 EQ 108) AND (P3.AP8JOUR EQ 7)) [P3.DIMANCHEAP1 = P3.AP1];
IF ((P3.QACTIVITE1 NE 101) AND (P3.QACTIVITE2 NE 102) AND (P3.QACTIVITE3 NE 103) AND (P3.QACTIVITE4 NE 104) AND (P3.QACTIVITES5 NE 105) AND (P3.QACTIVITE6 NE 106) AND (P3.QACTIVITE7 NE 107) AND (P3.QACTIVITE8 NE 108) AND (P3.QACTIVITE9 EQ 109) AND (P3.AP9JOUR EQ 7)) [P3.DIMANCHEAP1 = P3.AP1];
IF ((P3.QACTIVITE1 NE 101) AND (P3.QACTIVITE2 NE 102) AND (P3.QACTIVITE3 NE 103) AND (P3.QACTIVITE4 NE 104) AND (P3.QACTIVITES5 NE 105) AND (P3.QACTIVITE6 NE 106) AND (P3.QACTIVITE7 NE 107) AND (P3.QACTIVITE8 NE 108) AND (P3.QACTIVITE9 NE 109) AND (P3.QACTIVITE_10 EQ 110) AND (P3.AP10JOUR EQ 7)) [P3.DIMANCHEAP1 = P3.AP1];
IF ((P3.QACTIVITE1 NE 101) AND (P3.QACTIVITE2 NE 102) AND (P3.QACTIVITE3 NE 103) AND (P3.QACTIVITE4 NE 104) AND (P3.QACTIVITES5 NE 105) AND (P3.QACTIVITE6 NE 106) AND (P3.QACTIVITE7 NE 107) AND (P3.QACTIVITE8 NE 108) AND (P3.QACTIVITE9 NE 109) AND (P3.QACTIVITE_10 NE 110) AND (P3.QACTIVITE_11 EQ 111) AND (P3.AP11JOUR EQ 7)) [P3.DIMANCHEAP1 = P3.AP1];
IF ((P3.QACTIVITE1 NE 101) AND (P3.QACTIVITE2 NE 102) AND (P3.QACTIVITE3 NE 103) AND (P3.QACTIVITE4 NE 104) AND (P3.QACTIVITES5 NE 105) AND (P3.QACTIVITE6 NE 106) AND (P3.QACTIVITE7

NE 107) AND (P3.QACTIVITE8 NE 108) AND (P3.QACTIVITE9 NE 109) AND (P3.QACTIVITE_10 NE 110) AND (P3.QACTIVITE_11 NE 111) AND (P3.QACTIVITE_12 EQ 112) AND (P3.AP12JOUR EQ 7)) [P3.DIMANCHEAP1 = P3.AP1];
ACTIVITÉ 2 DANS LE PLAN POUR CHAQUE JOUR DE LA SEMAINE
IF ((P3.2SUMAP2 GE 100) AND (P3.2SUMAP2 LT 200) AND (P3.QACTIVITE2 EQ 102) AND (P3.AP2JOUR EQ 1)) [P3.LUNDIAP2 = P3.AP2];
IF ((P3.3SUMAP2 GE 100) AND (P3.3SUMAP2 LT 200) AND (P3.QACTIVITE3 EQ 103) AND (P3.AP3JOUR EQ 1)) [P3.LUNDIAP2 = P3.AP2];
IF ((P3.4SUMAP2 GE 100) AND (P3.4SUMAP2 LT 200) AND (P3.QACTIVITE4 EQ 104) AND (P3.AP4JOUR EQ 1)) [P3.LUNDIAP2 = P3.AP2];
IF ((P3.5SUMAP2 GE 100) AND (P3.5SUMAP2 LT 200) AND (P3.QACTIVITE5 EQ 105) AND (P3.AP5JOUR EQ 1)) [P3.LUNDIAP2 = P3.AP2];
IF ((P3.6SUMAP2 GE 100) AND (P3.6SUMAP2 LT 200) AND (P3.QACTIVITE6 EQ 106) AND (P3.AP6JOUR EQ 1)) [P3.LUNDIAP2 = P3.AP2];
IF ((P3.7SUMAP2 GE 100) AND (P3.7SUMAP2 LT 200) AND (P3.QACTIVITE7 EQ 107) AND (P3.AP7JOUR EQ 1)) [P3.LUNDIAP2 = P3.AP2];
IF ((P3.8SUMAP2 GE 100) AND (P3.8SUMAP2 LT 200) AND (P3.QACTIVITE8 EQ 108) AND (P3.AP8JOUR EQ 1)) [P3.LUNDIAP2 = P3.AP2];
IF ((P3.9SUMAP2 GE 100) AND (P3.9SUMAP2 LT 200) AND (P3.QACTIVITE9 EQ 109) AND (P3.AP9JOUR EQ 1)) [P3.LUNDIAP2 = P3.AP2];
IF ((P3.10SUMAP2 GE 100) AND (P3.10SUMAP2 LT 200) AND (P3.QACTIVITE_10 EQ 110) AND (P3.AP10JOUR EQ 1)) [P3.LUNDIAP2 = P3.AP2];
IF ((P3.11SUMAP2 GE 100) AND (P3.11SUMAP2 LT 200) AND (P3.QACTIVITE_11 EQ 111) AND (P3.AP11JOUR EQ 1)) [P3.LUNDIAP2 = P3.AP2];
IF ((P3.12SUMAP2 GE 100) AND (P3.12SUMAP2 LT 200) AND (P3.QACTIVITE_12 EQ 112) AND (P3.AP12JOUR EQ 1)) [P3.LUNDIAP2 = P3.AP2];
IF ((P3.2SUMAP2 GE 100) AND (P3.2SUMAP2 LT 200) AND (P3.QACTIVITE2 EQ 102) AND (P3.AP2JOUR EQ 2)) [P3.MARDIAP2 = P3.AP2];
IF ((P3.3SUMAP2 GE 100) AND (P3.3SUMAP2 LT 200) AND (P3.QACTIVITE3 EQ 103) AND (P3.AP3JOUR EQ 2)) [P3.MARDIAP2 = P3.AP2];
IF ((P3.4SUMAP2 GE 100) AND (P3.4SUMAP2 LT 200) AND (P3.QACTIVITE4 EQ 104) AND (P3.AP4JOUR EQ 2)) [P3.MARDIAP2 = P3.AP2];
IF ((P3.5SUMAP2 GE 100) AND (P3.5SUMAP2 LT 200) AND (P3.QACTIVITE5 EQ 105) AND (P3.AP5JOUR EQ 2)) [P3.MARDIAP2 = P3.AP2];
IF ((P3.6SUMAP2 GE 100) AND (P3.6SUMAP2 LT 200) AND (P3.QACTIVITE6 EQ 106) AND (P3.AP6JOUR EQ 2)) [P3.MARDIAP2 = P3.AP2];
IF ((P3.7SUMAP2 GE 100) AND (P3.7SUMAP2 LT 200) AND (P3.QACTIVITE7 EQ 107) AND (P3.AP7JOUR EQ 2)) [P3.MARDIAP2 = P3.AP2];
IF ((P3.8SUMAP2 GE 100) AND (P3.8SUMAP2 LT 200) AND (P3.QACTIVITE8 EQ 108) AND (P3.AP8JOUR EQ 2)) [P3.MARDIAP2 = P3.AP2];
IF ((P3.9SUMAP2 GE 100) AND (P3.9SUMAP2 LT 200) AND (P3.QACTIVITE9 EQ 109) AND (P3.AP9JOUR EQ 2)) [P3.MARDIAP2 = P3.AP2];

IF ((P3.10SUMAP2 GE 100) AND (P3.10SUMAP2 LT 200) AND (P3.QACTIVITE_10 EQ 110) AND (P3.AP10JOUR EQ 2)) [P3.MARDIAP2 = P3.AP2];
IF ((P3.11SUMAP2 GE 100) AND (P3.11SUMAP2 LT 200) AND (P3.QACTIVITE_11 EQ 111) AND (P3.AP11JOUR EQ 2)) [P3.MARDIAP2 = P3.AP2];
IF ((P3.12SUMAP2 GE 100) AND (P3.12SUMAP2 LT 200) AND (P3.QACTIVITE_12 EQ 112) AND (P3.AP12JOUR EQ 2)) [P3.MARDIAP2 = P3.AP2];
IF ((P3.2SUMAP2 GE 100) AND (P3.2SUMAP2 LT 200) AND (P3.QACTIVITE2 EQ 102) AND (P3.AP2JOUR EQ 3)) [P3.MERCREDIAP2 = P3.AP2];
IF ((P3.3SUMAP2 GE 100) AND (P3.3SUMAP2 LT 200) AND (P3.QACTIVITE3 EQ 103) AND (P3.AP3JOUR EQ 3)) [P3.MERCREDIAP2 = P3.AP2];
IF ((P3.4SUMAP2 GE 100) AND (P3.4SUMAP2 LT 200) AND (P3.QACTIVITE4 EQ 104) AND (P3.AP4JOUR EQ 3)) [P3.MERCREDIAP2 = P3.AP2];
IF ((P3.5SUMAP2 GE 100) AND (P3.5SUMAP2 LT 200) AND (P3.QACTIVITE5 EQ 105) AND (P3.AP5JOUR EQ 3)) [P3.MERCREDIAP2 = P3.AP2];
IF ((P3.6SUMAP2 GE 100) AND (P3.6SUMAP2 LT 200) AND (P3.QACTIVITE6 EQ 106) AND (P3.AP6JOUR EQ 3)) [P3.MERCREDIAP2 = P3.AP2];
IF ((P3.7SUMAP2 GE 100) AND (P3.7SUMAP2 LT 200) AND (P3.QACTIVITE7 EQ 107) AND (P3.AP7JOUR EQ 3)) [P3.MERCREDIAP2 = P3.AP2];
IF ((P3.8SUMAP2 GE 100) AND (P3.8SUMAP2 LT 200) AND (P3.QACTIVITE8 EQ 108) AND (P3.AP8JOUR EQ 3)) [P3.MERCREDIAP2 = P3.AP2];
IF ((P3.9SUMAP2 GE 100) AND (P3.9SUMAP2 LT 200) AND (P3.QACTIVITE9 EQ 109) AND (P3.AP9JOUR EQ 3)) [P3.MERCREDIAP2 = P3.AP2];
IF ((P3.10SUMAP2 GE 100) AND (P3.10SUMAP2 LT 200) AND (P3.QACTIVITE_10 EQ 110) AND (P3.AP10JOUR EQ 3)) [P3.MERCREDIAP2 = P3.AP2];
IF ((P3.11SUMAP2 GE 100) AND (P3.11SUMAP2 LT 200) AND (P3.QACTIVITE_11 EQ 111) AND (P3.AP11JOUR EQ 3)) [P3.MERCREDIAP2 = P3.AP2];
IF ((P3.12SUMAP2 GE 100) AND (P3.12SUMAP2 LT 200) AND (P3.QACTIVITE_12 EQ 112) AND (P3.AP12JOUR EQ 3)) [P3.MERCREDIAP2 = P3.AP2];
IF ((P3.2SUMAP2 GE 100) AND (P3.2SUMAP2 LT 200) AND (P3.QACTIVITE2 EQ 102) AND (P3.AP2JOUR EQ 4)) [P3.JEUDIAP2 = P3.AP2];
IF ((P3.3SUMAP2 GE 100) AND (P3.3SUMAP2 LT 200) AND (P3.QACTIVITE3 EQ 103) AND (P3.AP3JOUR EQ 4)) [P3.JEUDIAP2 = P3.AP2];
IF ((P3.4SUMAP2 GE 100) AND (P3.4SUMAP2 LT 200) AND (P3.QACTIVITE4 EQ 104) AND (P3.AP4JOUR EQ 4)) [P3.JEUDIAP2 = P3.AP2];
IF ((P3.5SUMAP2 GE 100) AND (P3.5SUMAP2 LT 200) AND (P3.QACTIVITE5 EQ 105) AND (P3.AP5JOUR EQ 4)) [P3.JEUDIAP2 = P3.AP2];
IF ((P3.6SUMAP2 GE 100) AND (P3.6SUMAP2 LT 200) AND (P3.QACTIVITE6 EQ 106) AND (P3.AP6JOUR EQ 4)) [P3.JEUDIAP2 = P3.AP2];
IF ((P3.7SUMAP2 GE 100) AND (P3.7SUMAP2 LT 200) AND (P3.QACTIVITE7 EQ 107) AND (P3.AP7JOUR EQ 4)) [P3.JEUDIAP2 = P3.AP2];
IF ((P3.8SUMAP2 GE 100) AND (P3.8SUMAP2 LT 200) AND (P3.QACTIVITE8 EQ 108) AND (P3.AP8JOUR EQ 4)) [P3.JEUDIAP2 = P3.AP2];

IF ((P3.9SUMAP2 GE 100) AND (P3.9SUMAP2 LT 200) AND (P3.QACTIVITE9 EQ 109) AND (P3.AP9JOUR EQ 4)) [P3.JEUDIAP2 = P3.AP2];
IF ((P3.10SUMAP2 GE 100) AND (P3.10SUMAP2 LT 200) AND (P3.QACTIVITE_10 EQ 110) AND (P3.AP10JOUR EQ 4)) [P3.JEUDIAP2 = P3.AP2];
IF ((P3.11SUMAP2 GE 100) AND (P3.11SUMAP2 LT 200) AND (P3.QACTIVITE_11 EQ 111) AND (P3.AP11JOUR EQ 4)) [P3.JEUDIAP2 = P3.AP2];
IF ((P3.12SUMAP2 GE 100) AND (P3.12SUMAP2 LT 200) AND (P3.QACTIVITE_12 EQ 112) AND (P3.AP12JOUR EQ 4)) [P3.JEUDIAP2 = P3.AP2];
IF ((P3.2SUMAP2 GE 100) AND (P3.2SUMAP2 LT 200) AND (P3.QACTIVITE2 EQ 102) AND (P3.AP2JOUR EQ 5)) [P3.VENDREDIAP2 = P3.AP2];
IF ((P3.3SUMAP2 GE 100) AND (P3.3SUMAP2 LT 200) AND (P3.QACTIVITE3 EQ 103) AND (P3.AP3JOUR EQ 5)) [P3.VENDREDIAP2 = P3.AP2];
IF ((P3.4SUMAP2 GE 100) AND (P3.4SUMAP2 LT 200) AND (P3.QACTIVITE4 EQ 104) AND (P3.AP4JOUR EQ 5)) [P3.VENDREDIAP2 = P3.AP2];
IF ((P3.5SUMAP2 GE 100) AND (P3.5SUMAP2 LT 200) AND (P3.QACTIVITE5 EQ 105) AND (P3.AP5JOUR EQ 5)) [P3.VENDREDIAP2 = P3.AP2];
IF ((P3.6SUMAP2 GE 100) AND (P3.6SUMAP2 LT 200) AND (P3.QACTIVITE6 EQ 106) AND (P3.AP6JOUR EQ 5)) [P3.VENDREDIAP2 = P3.AP2];
IF ((P3.7SUMAP2 GE 100) AND (P3.7SUMAP2 LT 200) AND (P3.QACTIVITE7 EQ 107) AND (P3.AP7JOUR EQ 5)) [P3.VENDREDIAP2 = P3.AP2];
IF ((P3.8SUMAP2 GE 100) AND (P3.8SUMAP2 LT 200) AND (P3.QACTIVITE8 EQ 108) AND (P3.AP8JOUR EQ 5)) [P3.VENDREDIAP2 = P3.AP2];
IF ((P3.9SUMAP2 GE 100) AND (P3.9SUMAP2 LT 200) AND (P3.QACTIVITE9 EQ 109) AND (P3.AP9JOUR EQ 5)) [P3.VENDREDIAP2 = P3.AP2];
IF ((P3.10SUMAP2 GE 100) AND (P3.10SUMAP2 LT 200) AND (P3.QACTIVITE_10 EQ 110) AND (P3.AP10JOUR EQ 5)) [P3.VENDREDIAP2 = P3.AP2];
IF ((P3.11SUMAP2 GE 100) AND (P3.11SUMAP2 LT 200) AND (P3.QACTIVITE_11 EQ 111) AND (P3.AP11JOUR EQ 5)) [P3.VENDREDIAP2 = P3.AP2];
IF ((P3.12SUMAP2 GE 100) AND (P3.12SUMAP2 LT 200) AND (P3.QACTIVITE_12 EQ 112) AND (P3.AP12JOUR EQ 5)) [P3.VENDREDIAP2 = P3.AP2];
IF ((P3.2SUMAP2 GE 100) AND (P3.2SUMAP2 LT 200) AND (P3.QACTIVITE2 EQ 102) AND (P3.AP2JOUR EQ 6)) [P3.SAMEDIAP2 = P3.AP2];
IF ((P3.3SUMAP2 GE 100) AND (P3.3SUMAP2 LT 200) AND (P3.QACTIVITE3 EQ 103) AND (P3.AP3JOUR EQ 6)) [P3.SAMEDIAP2 = P3.AP2];
IF ((P3.4SUMAP2 GE 100) AND (P3.4SUMAP2 LT 200) AND (P3.QACTIVITE4 EQ 104) AND (P3.AP4JOUR EQ 6)) [P3.SAMEDIAP2 = P3.AP2];
IF ((P3.5SUMAP2 GE 100) AND (P3.5SUMAP2 LT 200) AND (P3.QACTIVITE5 EQ 105) AND (P3.AP5JOUR EQ 6)) [P3.SAMEDIAP2 = P3.AP2];
IF ((P3.6SUMAP2 GE 100) AND (P3.6SUMAP2 LT 200) AND (P3.QACTIVITE6 EQ 106) AND (P3.AP6JOUR EQ 6)) [P3.SAMEDIAP2 = P3.AP2];
IF ((P3.7SUMAP2 GE 100) AND (P3.7SUMAP2 LT 200) AND (P3.QACTIVITE7 EQ 107) AND (P3.AP7JOUR EQ 6)) [P3.SAMEDIAP2 = P3.AP2];

IF ((P3.8SUMAP2 GE 100) AND (P3.8SUMAP2 LT 200) AND (P3.QACTIVITE8 EQ 108) AND (P3.AP8JOUR EQ 6)) [P3.SAMEDIAP2 = P3.AP2];
IF ((P3.9SUMAP2 GE 100) AND (P3.9SUMAP2 LT 200) AND (P3.QACTIVITE9 EQ 109) AND (P3.AP9JOUR EQ 6)) [P3.SAMEDIAP2 = P3.AP2];
IF ((P3.10SUMAP2 GE 100) AND (P3.10SUMAP2 LT 200) AND (P3.QACTIVITE_10 EQ 110) AND (P3.AP10JOUR EQ 6)) [P3.SAMEDIAP2 = P3.AP2];
IF ((P3.11SUMAP2 GE 100) AND (P3.11SUMAP2 LT 200) AND (P3.QACTIVITE_11 EQ 111) AND (P3.AP11JOUR EQ 6)) [P3.SAMEDIAP2 = P3.AP2];
IF ((P3.12SUMAP2 GE 100) AND (P3.12SUMAP2 LT 200) AND (P3.QACTIVITE_12 EQ 112) AND (P3.AP12JOUR EQ 6)) [P3.SAMEDIAP2 = P3.AP2];
IF ((P3.2SUMAP2 GE 100) AND (P3.2SUMAP2 LT 200) AND (P3.QACTIVITE2 EQ 102) AND (P3.AP2JOUR EQ 7)) [P3.DIMANCHEAP2 = P3.AP2];
IF ((P3.3SUMAP2 GE 100) AND (P3.3SUMAP2 LT 200) AND (P3.QACTIVITE3 EQ 103) AND (P3.AP3JOUR EQ 7)) [P3.DIMANCHEAP2 = P3.AP2];
IF ((P3.4SUMAP2 GE 100) AND (P3.4SUMAP2 LT 200) AND (P3.QACTIVITE4 EQ 104) AND (P3.AP4JOUR EQ 7)) [P3.DIMANCHEAP2 = P3.AP2];
IF ((P3.5SUMAP2 GE 100) AND (P3.5SUMAP2 LT 200) AND (P3.QACTIVITE5 EQ 105) AND (P3.AP5JOUR EQ 7)) [P3.DIMANCHEAP2 = P3.AP2];
IF ((P3.6SUMAP2 GE 100) AND (P3.6SUMAP2 LT 200) AND (P3.QACTIVITE6 EQ 106) AND (P3.AP6JOUR EQ 7)) [P3.DIMANCHEAP2 = P3.AP2];
IF ((P3.7SUMAP2 GE 100) AND (P3.7SUMAP2 LT 200) AND (P3.QACTIVITE7 EQ 107) AND (P3.AP7JOUR EQ 7)) [P3.DIMANCHEAP2 = P3.AP2];
IF ((P3.8SUMAP2 GE 100) AND (P3.8SUMAP2 LT 200) AND (P3.QACTIVITE8 EQ 108) AND (P3.AP8JOUR EQ 7)) [P3.DIMANCHEAP2 = P3.AP2];
IF ((P3.9SUMAP2 GE 100) AND (P3.9SUMAP2 LT 200) AND (P3.QACTIVITE9 EQ 109) AND (P3.AP9JOUR EQ 7)) [P3.DIMANCHEAP2 = P3.AP2];
IF ((P3.10SUMAP2 GE 100) AND (P3.10SUMAP2 LT 200) AND (P3.QACTIVITE_10 EQ 110) AND (P3.AP10JOUR EQ 7)) [P3.DIMANCHEAP2 = P3.AP2];
IF ((P3.11SUMAP2 GE 100) AND (P3.11SUMAP2 LT 200) AND (P3.QACTIVITE_11 EQ 111) AND (P3.AP11JOUR EQ 7)) [P3.DIMANCHEAP2 = P3.AP2];
IF ((P3.12SUMAP2 GE 100) AND (P3.12SUMAP2 LT 200) AND (P3.QACTIVITE_12 EQ 112) AND (P3.AP12JOUR EQ 7)) [P3.DIMANCHEAP2 = P3.AP2];
ACTIVITÉ 3 DANS LE PLAN POUR CHAQUE JOUR DE LA SEMAINE
IF ((P3.3SUMAP3 GE 200) AND (P3.3SUMAP3 LT 300) AND (P3.QACTIVITE3 EQ 103) AND (P3.AP3JOUR EQ 1)) [P3.LUNDIAP3 = P3.AP3];
IF ((P3.4SUMAP3 GE 200) AND (P3.4SUMAP3 LT 300) AND (P3.QACTIVITE4 EQ 104) AND (P3.AP4JOUR EQ 1)) [P3.LUNDIAP3 = P3.AP3];
IF ((P3.5SUMAP3 GE 200) AND (P3.5SUMAP3 LT 300) AND (P3.QACTIVITE5 EQ 105) AND (P3.AP5JOUR EQ 1)) [P3.LUNDIAP3 = P3.AP3];
IF ((P3.6SUMAP3 GE 200) AND (P3.6SUMAP3 LT 300) AND (P3.QACTIVITE6 EQ 106) AND (P3.AP6JOUR EQ 1)) [P3.LUNDIAP3 = P3.AP3];

IF ((P3.7SUMAP3 GE 200) AND (P3.7SUMAP3 LT 300) AND (P3.QACTIVITE7 EQ 107) AND (P3.AP7JOUR EQ 1)) [P3.LUNDIAP3 = P3.AP3];
IF ((P3.8SUMAP3 GE 200) AND (P3.8SUMAP3 LT 300) AND (P3.QACTIVITE8 EQ 108) AND (P3.AP8JOUR EQ 1)) [P3.LUNDIAP3 = P3.AP3];
IF ((P3.9SUMAP3 GE 200) AND (P3.9SUMAP3 LT 300) AND (P3.QACTIVITE9 EQ 109) AND (P3.AP9JOUR EQ 1)) [P3.LUNDIAP3 = P3.AP3];
IF ((P3.10SUMAP3 GE 200) AND (P3.10SUMAP3 LT 300) AND (P3.QACTIVITE_10 EQ 110) AND (P3.AP10JOUR EQ 1)) [P3.LUNDIAP3 = P3.AP3];
IF ((P3.11SUMAP3 GE 200) AND (P3.11SUMAP3 LT 300) AND (P3.QACTIVITE_11 EQ 111) AND (P3.AP11JOUR EQ 1)) [P3.LUNDIAP3 = P3.AP3];
IF ((P3.12SUMAP3 GE 200) AND (P3.12SUMAP3 LT 300) AND (P3.QACTIVITE_12 EQ 112) AND (P3.AP12JOUR EQ 1)) [P3.LUNDIAP3 = P3.AP3];
IF ((P3.3SUMAP3 GE 200) AND (P3.3SUMAP3 LT 300) AND (P3.QACTIVITE3 EQ 103) AND (P3.AP3JOUR EQ 2)) [P3.MARDIAP3 = P3.AP3];
IF ((P3.4SUMAP3 GE 200) AND (P3.4SUMAP3 LT 300) AND (P3.QACTIVITE4 EQ 104) AND (P3.AP4JOUR EQ 2)) [P3.MARDIAP3 = P3.AP3];
IF ((P3.5SUMAP3 GE 200) AND (P3.5SUMAP3 LT 300) AND (P3.QACTIVITE5 EQ 105) AND (P3.AP5JOUR EQ 2)) [P3.MARDIAP3 = P3.AP3];
IF ((P3.6SUMAP3 GE 200) AND (P3.6SUMAP3 LT 300) AND (P3.QACTIVITE6 EQ 106) AND (P3.AP6JOUR EQ 2)) [P3.MARDIAP3 = P3.AP3];
IF ((P3.7SUMAP3 GE 200) AND (P3.7SUMAP3 LT 300) AND (P3.QACTIVITE7 EQ 107) AND (P3.AP7JOUR EQ 2)) [P3.MARDIAP3 = P3.AP3];
IF ((P3.8SUMAP3 GE 200) AND (P3.8SUMAP3 LT 300) AND (P3.QACTIVITE8 EQ 108) AND (P3.AP8JOUR EQ 2)) [P3.MARDIAP3 = P3.AP3];
IF ((P3.9SUMAP3 GE 200) AND (P3.9SUMAP3 LT 300) AND (P3.QACTIVITE9 EQ 109) AND (P3.AP9JOUR EQ 2)) [P3.MARDIAP3 = P3.AP3];
IF ((P3.10SUMAP3 GE 200) AND (P3.10SUMAP3 LT 300) AND (P3.QACTIVITE_10 EQ 110) AND (P3.AP10JOUR EQ 2)) [P3.MARDIAP3 = P3.AP3];
IF ((P3.11SUMAP3 GE 200) AND (P3.11SUMAP3 LT 300) AND (P3.QACTIVITE_11 EQ 111) AND (P3.AP11JOUR EQ 2)) [P3.MARDIAP3 = P3.AP3];
IF ((P3.12SUMAP3 GE 200) AND (P3.12SUMAP3 LT 300) AND (P3.QACTIVITE_12 EQ 112) AND (P3.AP12JOUR EQ 2)) [P3.MARDIAP3 = P3.AP3];
IF ((P3.3SUMAP3 GE 200) AND (P3.3SUMAP3 LT 300) AND (P3.QACTIVITE3 EQ 103) AND (P3.AP3JOUR EQ 3)) [P3.MERCREDIAP3 = P3.AP3];
IF ((P3.4SUMAP3 GE 200) AND (P3.4SUMAP3 LT 300) AND (P3.QACTIVITE4 EQ 104) AND (P3.AP4JOUR EQ 3)) [P3.MERCREDIAP3 = P3.AP3];
IF ((P3.5SUMAP3 GE 200) AND (P3.5SUMAP3 LT 300) AND (P3.QACTIVITE5 EQ 105) AND (P3.AP5JOUR EQ 3)) [P3.MERCREDIAP3 = P3.AP3];
IF ((P3.6SUMAP3 GE 200) AND (P3.6SUMAP3 LT 300) AND (P3.QACTIVITE6 EQ 106) AND (P3.AP6JOUR EQ 3)) [P3.MERCREDIAP3 = P3.AP3];
IF ((P3.7SUMAP3 GE 200) AND (P3.7SUMAP3 LT 300) AND (P3.QACTIVITE7 EQ 107) AND (P3.AP7JOUR EQ 3)) [P3.MERCREDIAP3 = P3.AP3];

IF ((P3.8SUMAP3 GE 200) AND (P3.8SUMAP3 LT 300) AND (P3.QACTIVITE8 EQ 108) AND (P3.AP8JOUR EQ 3)) [P3.MERCREDIAP3 = P3.AP3];
IF ((P3.9SUMAP3 GE 200) AND (P3.9SUMAP3 LT 300) AND (P3.QACTIVITE9 EQ 109) AND (P3.AP9JOUR EQ 3)) [P3.MERCREDIAP3 = P3.AP3];
IF ((P3.10SUMAP3 GE 200) AND (P3.10SUMAP3 LT 300) AND (P3.QACTIVITE_10 EQ 110) AND (P3.AP10JOUR EQ 3)) [P3.MERCREDIAP3 = P3.AP3];
IF ((P3.11SUMAP3 GE 200) AND (P3.11SUMAP3 LT 300) AND (P3.QACTIVITE_11 EQ 111) AND (P3.AP11JOUR EQ 3)) [P3.MERCREDIAP3 = P3.AP3];
IF ((P3.12SUMAP3 GE 200) AND (P3.12SUMAP3 LT 300) AND (P3.QACTIVITE_12 EQ 112) AND (P3.AP12JOUR EQ 3)) [P3.MERCREDIAP3 = P3.AP3];
IF ((P3.3SUMAP3 GE 200) AND (P3.3SUMAP3 LT 300) AND (P3.QACTIVITE3 EQ 103) AND (P3.AP3JOUR EQ 4)) [P3.JEUDIAP3 = P3.AP3];
IF ((P3.4SUMAP3 GE 200) AND (P3.4SUMAP3 LT 300) AND (P3.QACTIVITE4 EQ 104) AND (P3.AP4JOUR EQ 4)) [P3.JEUDIAP3 = P3.AP3];
IF ((P3.5SUMAP3 GE 200) AND (P3.5SUMAP3 LT 300) AND (P3.QACTIVITE5 EQ 105) AND (P3.AP5JOUR EQ 4)) [P3.JEUDIAP3 = P3.AP3];
IF ((P3.6SUMAP3 GE 200) AND (P3.6SUMAP3 LT 300) AND (P3.QACTIVITE6 EQ 106) AND (P3.AP6JOUR EQ 4)) [P3.JEUDIAP3 = P3.AP3];
IF ((P3.7SUMAP3 GE 200) AND (P3.7SUMAP3 LT 300) AND (P3.QACTIVITE7 EQ 107) AND (P3.AP7JOUR EQ 4)) [P3.JEUDIAP3 = P3.AP3];
IF ((P3.8SUMAP3 GE 200) AND (P3.8SUMAP3 LT 300) AND (P3.QACTIVITE8 EQ 108) AND (P3.AP8JOUR EQ 4)) [P3.JEUDIAP3 = P3.AP3];
IF ((P3.9SUMAP3 GE 200) AND (P3.9SUMAP3 LT 300) AND (P3.QACTIVITE9 EQ 109) AND (P3.AP9JOUR EQ 4)) [P3.JEUDIAP3 = P3.AP3];
IF ((P3.10SUMAP3 GE 200) AND (P3.10SUMAP3 LT 300) AND (P3.QACTIVITE_10 EQ 110) AND (P3.AP10JOUR EQ 4)) [P3.JEUDIAP3 = P3.AP3];
IF ((P3.11SUMAP3 GE 200) AND (P3.11SUMAP3 LT 300) AND (P3.QACTIVITE_11 EQ 111) AND (P3.AP11JOUR EQ 4)) [P3.JEUDIAP3 = P3.AP3];
IF ((P3.12SUMAP3 GE 200) AND (P3.12SUMAP3 LT 300) AND (P3.QACTIVITE_12 EQ 112) AND (P3.AP12JOUR EQ 4)) [P3.JEUDIAP3 = P3.AP3];
IF ((P3.3SUMAP3 GE 200) AND (P3.3SUMAP3 LT 300) AND (P3.QACTIVITE3 EQ 103) AND (P3.AP3JOUR EQ 5)) [P3.VENDREDIAP3 = P3.AP3];
IF ((P3.4SUMAP3 GE 200) AND (P3.4SUMAP3 LT 300) AND (P3.QACTIVITE4 EQ 104) AND (P3.AP4JOUR EQ 5)) [P3.VENDREDIAP3 = P3.AP3];
IF ((P3.5SUMAP3 GE 200) AND (P3.5SUMAP3 LT 300) AND (P3.QACTIVITE5 EQ 105) AND (P3.AP5JOUR EQ 5)) [P3.VENDREDIAP3 = P3.AP3];
IF ((P3.6SUMAP3 GE 200) AND (P3.6SUMAP3 LT 300) AND (P3.QACTIVITE6 EQ 106) AND (P3.AP6JOUR EQ 5)) [P3.VENDREDIAP3 = P3.AP3];
IF ((P3.7SUMAP3 GE 200) AND (P3.7SUMAP3 LT 300) AND (P3.QACTIVITE7 EQ 107) AND (P3.AP7JOUR EQ 5)) [P3.VENDREDIAP3 = P3.AP3];
IF ((P3.8SUMAP3 GE 200) AND (P3.8SUMAP3 LT 300) AND (P3.QACTIVITE8 EQ 108) AND (P3.AP8JOUR EQ 5)) [P3.VENDREDIAP3 = P3.AP3];

IF ((P3.9SUMAP3 GE 200) AND (P3.9SUMAP3 LT 300) AND (P3.QACTIVITE9 EQ 109) AND (P3.AP9JOUR EQ 5)) [P3.VENDREDIAP3 = P3.AP3];
IF ((P3.10SUMAP3 GE 200) AND (P3.10SUMAP3 LT 300) AND (P3.QACTIVITE_10 EQ 110) AND (P3.AP10JOUR EQ 5)) [P3.VENDREDIAP3 = P3.AP3];
IF ((P3.11SUMAP3 GE 200) AND (P3.11SUMAP3 LT 300) AND (P3.QACTIVITE_11 EQ 111) AND (P3.AP11JOUR EQ 5)) [P3.VENDREDIAP3 = P3.AP3];
IF ((P3.12SUMAP3 GE 200) AND (P3.12SUMAP3 LT 300) AND (P3.QACTIVITE_12 EQ 112) AND (P3.AP12JOUR EQ 5)) [P3.VENDREDIAP3 = P3.AP3];
IF ((P3.3SUMAP3 GE 200) AND (P3.3SUMAP3 LT 300) AND (P3.QACTIVITE3 EQ 103) AND (P3.AP3JOUR EQ 6)) [P3.SAMEDIAP3 = P3.AP3];
IF ((P3.4SUMAP3 GE 200) AND (P3.4SUMAP3 LT 300) AND (P3.QACTIVITE4 EQ 104) AND (P3.AP4JOUR EQ 6)) [P3.SAMEDIAP3 = P3.AP3];
IF ((P3.5SUMAP3 GE 200) AND (P3.5SUMAP3 LT 300) AND (P3.QACTIVITE5 EQ 105) AND (P3.AP5JOUR EQ 6)) [P3.SAMEDIAP3 = P3.AP3];
IF ((P3.6SUMAP3 GE 200) AND (P3.6SUMAP3 LT 300) AND (P3.QACTIVITE6 EQ 106) AND (P3.AP6JOUR EQ 6)) [P3.SAMEDIAP3 = P3.AP3];
IF ((P3.7SUMAP3 GE 200) AND (P3.7SUMAP3 LT 300) AND (P3.QACTIVITE7 EQ 107) AND (P3.AP7JOUR EQ 6)) [P3.SAMEDIAP3 = P3.AP3];
IF ((P3.8SUMAP3 GE 200) AND (P3.8SUMAP3 LT 300) AND (P3.QACTIVITE8 EQ 108) AND (P3.AP8JOUR EQ 6)) [P3.SAMEDIAP3 = P3.AP3];
IF ((P3.9SUMAP3 GE 200) AND (P3.9SUMAP3 LT 300) AND (P3.QACTIVITE9 EQ 109) AND (P3.AP9JOUR EQ 6)) [P3.SAMEDIAP3 = P3.AP3];
IF ((P3.10SUMAP3 GE 200) AND (P3.10SUMAP3 LT 300) AND (P3.QACTIVITE_10 EQ 110) AND (P3.AP10JOUR EQ 6)) [P3.SAMEDIAP3 = P3.AP3];
IF ((P3.11SUMAP3 GE 200) AND (P3.11SUMAP3 LT 300) AND (P3.QACTIVITE_11 EQ 111) AND (P3.AP11JOUR EQ 6)) [P3.SAMEDIAP3 = P3.AP3];
IF ((P3.12SUMAP3 GE 200) AND (P3.12SUMAP3 LT 300) AND (P3.QACTIVITE_12 EQ 112) AND (P3.AP12JOUR EQ 6)) [P3.SAMEDIAP3 = P3.AP3];
IF ((P3.3SUMAP3 GE 200) AND (P3.3SUMAP3 LT 300) AND (P3.QACTIVITE3 EQ 103) AND (P3.AP3JOUR EQ 7)) [P3.DIMANCHEAP3 = P3.AP3];
IF ((P3.4SUMAP3 GE 200) AND (P3.4SUMAP3 LT 300) AND (P3.QACTIVITE4 EQ 104) AND (P3.AP4JOUR EQ 7)) [P3.DIMANCHEAP3 = P3.AP3];
IF ((P3.5SUMAP3 GE 200) AND (P3.5SUMAP3 LT 300) AND (P3.QACTIVITE5 EQ 105) AND (P3.AP5JOUR EQ 7)) [P3.DIMANCHEAP3 = P3.AP3];
IF ((P3.6SUMAP3 GE 200) AND (P3.6SUMAP3 LT 300) AND (P3.QACTIVITE6 EQ 106) AND (P3.AP6JOUR EQ 7)) [P3.DIMANCHEAP3 = P3.AP3];
IF ((P3.7SUMAP3 GE 200) AND (P3.7SUMAP3 LT 300) AND (P3.QACTIVITE7 EQ 107) AND (P3.AP7JOUR EQ 7)) [P3.DIMANCHEAP3 = P3.AP3];
IF ((P3.8SUMAP3 GE 200) AND (P3.8SUMAP3 LT 300) AND (P3.QACTIVITE8 EQ 108) AND (P3.AP8JOUR EQ 7)) [P3.DIMANCHEAP3 = P3.AP3];
IF ((P3.9SUMAP3 GE 200) AND (P3.9SUMAP3 LT 300) AND (P3.QACTIVITE9 EQ 109) AND (P3.AP9JOUR EQ 7)) [P3.DIMANCHEAP3 = P3.AP3];

IF ((P3.10SUMAP3 GE 200) AND (P3.10SUMAP3 LT 300) AND (P3.QACTIVITE_10 EQ 110) AND (P3.AP10JOUR EQ 7)) [P3.DIMANCHEAP3 = P3.AP3];
IF ((P3.11SUMAP3 GE 200) AND (P3.11SUMAP3 LT 300) AND (P3.QACTIVITE_11 EQ 111) AND (P3.AP11JOUR EQ 7)) [P3.DIMANCHEAP3 = P3.AP3];
IF ((P3.12SUMAP3 GE 200) AND (P3.12SUMAP3 LT 300) AND (P3.QACTIVITE_12 EQ 112) AND (P3.AP12JOUR EQ 7)) [P3.DIMANCHEAP3 = P3.AP3];
POURSUITE DU CODE
IF (P3.P7BARCHOICE EQ 101) [P3.BAR = 'Être trop fatigué ou fatiguée pour faire de l´activité physique'];
IF (P3.P7BARCHOICE EQ 102) [P3.BAR = 'Avoir beaucoup de choses à faire mis à part l´activité physique'];
IF (P3.P7BARCHOICE EQ 103) [P3.BAR = 'température sera trop mauvaise pour faire de l´activité physique'];
IF (P3.P7BARCHOICE EQ 104) [P3.BAR = 'Ne pas avoir personne avec qui faire de l´activité physique'];
IF (P3.P7BARCHOICE EQ 105) [P3.BAR = 'Ne pas avoir accès à des emplacements pour faire de l´activité physique'];
IF (P3.P7BARCHOICE EQ 106) [P3.BAR = 'Ne pas avoir d´argent pour faire de l´activité physique'];
IF (P3.P7BARCHOICE EQ 107) [P3.BAR = 'Être trop gêné ou gênée, manquer de confiance pour aller faire de l´activité physique'];
IF (P3.P7BARCHOICE EQ 108) [P3.BAR = 'Avoir peur d´être insuffisamment en santé pour faire de l´activité physique'];
IF (P3.P7BARCHOICE EQ 109) [P3.BAR = 'Manquer de motivation'];
IF ((P3.P7BARCHOICE EQ 110) AND (P3.Q_BARANSWER NE 0)) [P3.BAR = P3.Q_BARANSWER];
IF (P3.SUMSOL EQ 101) [P3.SOL = 'Je vais faire de l´activité tôt dans la journée, en matinée ou à l´heure du midi.'];
IF (P3.SUMSOL EQ 102) [P3.SOL = 'J´essaie de garder en tête que si je fais de l´activité physique, j´aurai de plus en plus d´énergie dans la vie.'];
IF (P3.SUMSOL EQ 103) [P3.SOL = 'Je vais planifier des activités avec d´autres personnes qui me motiveront.'];
IF (P3.SUMSOL EQ 104) [P3.SOL = 'Je prends une collation pour me donner de l´énergie et j´y vais quand même.'];
IF (P3.SUMSOL EQ 201) [P3.SOL = 'Faire de l´activité physique par bloc de 10 minutes les journées où je n´ai pas de temps.'];
IF (P3.SUMSOL EQ 202) [P3.SOL = 'Je vais remplacer des activités où je suis assis ou assise, télé, ordinateur ou autres, par des activités physiques.'];
IF (P3.SUMSOL EQ 203) [P3.SOL = 'Je vais me procurer un agenda et inscrire dans mon horaire les moments où je vais faire de l´activité physique.'];
IF (P3.SUMSOL EQ 204) [P3.SOL = 'Je vais prendre des pauses de travail active et faire des 10 minutes de marche.'];
IF (P3.SUMSOL EQ 205) [P3.SOL = 'Je vais me fixer des moments avec d´autres personnes qui me plaisent pour aller faire de l´activité physique.'];
IF (P3.SUMSOL EQ 301) [P3.SOL = 'Je vais me procurer l´équipement nécessaire pour en faire même quand il pleut, quand il neige ou quand il fait trop chaud.'];
IF (P3.SUMSOL EQ 302) [P3.SOL = 'Je vais faire de l’activité physique chez nous ou à l´intérieur dans un endroit que j´aurai choisi quand il fait moins beau.'];

IF (P3.SUMSOL EQ 303) [P3.SOL = 'Je vais me préparer une activité physique alternative au cas où il serait désagréable d´aller dehors.'];
IF (P3.SUMSOL EQ 304) [P3.SOL = 'Je vais me dresser une liste des activités que je serais prêtE à faire si la température est mauvaise.'];
IF (P3.SUMSOL EQ 401) [P3.SOL = 'Je vais me joindre à un groupe qui pratique l´activité physique que j´aime. Ex : la marche, l´aquaforme ou la natation.'];
IF (P3.SUMSOL EQ 402) [P3.SOL = 'Je vais inviter mes amis, ma famille ou mes collègues à faire de l´activité physique avec moi.'];
IF (P3.SUMSOL EQ 403) [P3.SOL = 'Je vais amener mon chien marcher avec moi.'];
IF (P3.SUMSOL EQ 404) [P3.SOL = 'Je vais participer dans des discussions en ligne sur des pages facebook : Diabète Québec et autres.'];
IF (P3.SUMSOL EQ 501) [P3.SOL = 'Je vais faire de la marche ou du vélo dehors, c´est simple et gratuit.'];
IF (P3.SUMSOL EQ 502) [P3.SOL = 'Je vais faire de l´activité physique dans ma maison comme du yoga, un programme sur DVD, vélo stationnaire, tapis roulant, etc.'];
IF (P3.SUMSOL EQ 503) [P3.SOL = 'Je vais aller voir au centre communautaire de mon quartier pour me donner des options.'];
IF (P3.SUMSOL EQ 504) [P3.SOL = 'Je vais discuter avec mon médecin, avec un ami ou un spécialiste de l´activité physique pour avoir des conseils.'];
IF (P3.SUMSOL EQ 601) [P3.SOL = 'Je vais trouver des activités physiques abordables et simples : marcher dehors, faire du vélo, nager à la piscine communautaire.'];
IF (P3.SUMSOL EQ 602) [P3.SOL = 'Je vais aller voir au centre communautaire de mon quartier pour me donner des options.'];
IF (P3.SUMSOL EQ 603) [P3.SOL = 'Je vais économiser pour m´acheter un DVD d´activité physique ou pour une autre activité physique que j´aimerais faire.'];
IF (P3.SUMSOL EQ 604) [P3.SOL = 'Je vais aller marcher avec ma famille, des amis ou des collègues.'];
IF (P3.SUMSOL EQ 701) [P3.SOL = 'Je vais choisir une activité physique simple comme la marche ou la bicyclette.'];
IF (P3.SUMSOL EQ 702) [P3.SOL = 'Je vais aller faire de l´activité physique avec des gens qui m´acceptent comme je suis, qui évitent de me juger.'];
IF (P3.SUMSOL EQ 703) [P3.SOL = 'Au départ, je vais faire de l´activité physique dans des endroits où je suis seul[LETTRE_E] pour prendre confiance.'];
IF (P3.SUMSOL EQ 704) [P3.SOL = 'Je vais éviter des endroits qui me gêne davantage comme les centres de conditionnement.'];
IF (P3.SUMSOL EQ 801) [P3.SOL = 'Je vais consulter mon médecin pour mettre au clair si je peux faire de l´activité physique.'];
IF (P3.SUMSOL EQ 802) [P3.SOL = 'Je vais consulter un spécialiste de l´activité physique pour qu´il me rassure et me conseille sur ce qui est sécuritaire pour moi.'];
IF (P3.SUMSOL EQ 803) [P3.SOL = 'Je vais commencer à petite dose. 10 à 20 minutes maximum d´activités physiques les jours où je suis actif, active.'];
IF (P3.SUMSOL EQ 804) [P3.SOL = 'Je vais faire de l´activité physique accompagnéE d´un spécialiste de l´activité physique.'];

IF (P3.SUMSOL EQ 805) [P3.SOL = 'Je vais faire de l´activité physique dans un groupe où un spécialiste de l´activité physique est présent.'];
IF (P3.SUMSOL EQ 901) [P3.SOL = 'Je vais signer un contrat papier personnel comme quoi je m´engage à être actif, active.'];
IF (P3.SUMSOL EQ 902) [P3.SOL = 'Je vais signer un contrat papier avec mes proches comme quoi je m´engage à être actif, active.'];
IF (P3.SUMSOL EQ 903) [P3.SOL = 'Je vais essayer de nouvelles activités physiques pour trouver celles qui me plaisent vraiment.'];
IF (P3.SUMSOL EQ 904) [P3.SOL = 'À chaque activité physique que je fais, je prends un temps pour me rappeler ce que cela va m´apporter de mieux dans ma vie.'];
IF (P3.SUMSOL EQ 905) [P3.SOL = 'Je vais faire de l´activité physique avec des gens que j´aime pour me motiver.'];
IF ((P3.SUMSOL EQ 999) AND (P3.P8QSOLOPEN NE 0)) [P3.SOL = P3.P8QSOLOPEN];
IF (P3.SUMSOL EQ 0) [P3.SOL = P3.P8QSOL_10];
PLAN DE LA semaine 4
formules p4
IF ((P4.QACTI12 EQ 112) AND (P4.QAOPEN NE 0)) [P4.QACTIVITE_12 = 112];
[P4.2SUMAP2 = P4.QACTIVITE1];
[P4.3SUMAP2 = P4.QACTIVITE1+P4.QACTIVITE2];
[P4.4SUMAP2 = P4.QACTIVITE1+P4.QACTIVITE2+P4.QACTIVITE3];
[P4.5SUMAP2 = P4.QACTIVITE1+P4.QACTIVITE2+P4.QACTIVITE3+P4.QACTIVITE4];
[P4.6SUMAP2 = P4.QACTIVITE1+P4.QACTIVITE2+P4.QACTIVITE3+P4.QACTIVITE4+P4.QACTIVITE5];
[P4.7SUMAP2 = P4.QACTIVITE1+P4.QACTIVITE2+P4.QACTIVITE3+P4.QACTIVITE4+P4.QACTIVITE5+P4.QACTIVITE6];
[P4.8SUMAP2 = P4.QACTIVITE1+P4.QACTIVITE2+P4.QACTIVITE3+P4.QACTIVITE4+P4.QACTIVITE5+P4.QACTIVITE6+P4.QACTIVITE7];
[P4.9SUMAP2 = P4.QACTIVITE1+P4.QACTIVITE2+P4.QACTIVITE3+P4.QACTIVITE4+P4.QACTIVITE5+P4.QACTIVITE6+P4.QACTIVITE7+P4.QACTIVITE8];
[P4.10SUMAP2 = P4.QACTIVITE1+P4.QACTIVITE2+P4.QACTIVITE3+P4.QACTIVITE4+P4.QACTIVITE5+P4.QACTIVITE6+P4.QACTIVITE7+P4.QACTIVITE8+P4.QACTIVITE9];
[P4.11SUMAP2 = P4.QACTIVITE1+P4.QACTIVITE2+P4.QACTIVITE3+P4.QACTIVITE4+P4.QACTIVITE5+P4.QACTIVITE6+P4.QACTIVITE7+P4.QACTIVITE8+P4.QACTIVITE9+P4.QACTIVITE_10];
[P4.12SUMAP2 = P4.QACTIVITE1+P4.QACTIVITE2+P4.QACTIVITE3+P4.QACTIVITE4+P4.QACTIVITE5+P4.QACTIVITE6+P4.QACTIVITE7+P4.QACTIVITE8+P4.QACTIVITE9+P4.QACTIVITE_10+P4.QACTIVITE_11];
[P4.3SUMAP3 = P4.QACTIVITE1+P4.QACTIVITE2];
[P4.4SUMAP3 = P4.QACTIVITE1+P4.QACTIVITE2+P4.QACTIVITE3];
[P4.5SUMAP3 = P4.QACTIVITE1+P4.QACTIVITE2+P4.QACTIVITE3+P4.QACTIVITE4];

```
[P4.6SUMAP3 = P4.QACTIVITE1+P4.QACTIVITE2+P4.QACTIVITE3+P4.QACTIVITE4+P4.QACTIVITE5];
[P4.7SUMAP3 =
P4.QACTIVITE1+P4.QACTIVITE2+P4.QACTIVITE3+P4.QACTIVITE4+P4.QACTIVITE5+P4.QACTIVITE6];
[P4.8SUMAP3 =
P4.QACTIVITE1+P4.QACTIVITE2+P4.QACTIVITE3+P4.QACTIVITE4+P4.QACTIVITE5+P4.QACTIVITE6+P4.QACTI
VITE7];
[P4.9SUMAP3 =
P4.QACTIVITE1+P4.QACTIVITE2+P4.QACTIVITE3+P4.QACTIVITE4+P4.QACTIVITE5+P4.QACTIVITE6+P4.QACTI
VITE7+P4.QACTIVITE8];
[P4.10SUMAP3 =
P4.QACTIVITE1+P4.QACTIVITE2+P4.QACTIVITE3+P4.QACTIVITE4+P4.QACTIVITE5+P4.QACTIVITE6+P4.QACTI
VITE7+P4.QACTIVITE8+P4.QACTIVITE9];
[P4.11SUMAP3 =
P4.QACTIVITE1+P4.QACTIVITE2+P4.QACTIVITE3+P4.QACTIVITE4+P4.QACTIVITE5+P4.QACTIVITE6+P4.QACTI
VITE7+P4.QACTIVITE8+P4.QACTIVITE9+P4.QACTIVITE_10];
[P4.12SUMAP3 =
P4.QACTIVITE1+P4.QACTIVITE2+P4.QACTIVITE3+P4.QACTIVITE4+P4.QACTIVITE5+P4.QACTIVITE6+P4.QACTI
VITE7+P4.QACTIVITE8+P4.QACTIVITE9+P4.QACTIVITE_10+P4.QACTIVITE_11];
[P4.SUMAP =
P4.QACTIVITE1+P4.QACTIVITE2+P4.QACTIVITE3+P4.QACTIVITE4+P4.QACTIVITE5+P4.QACTIVITE6+P4.QACTI
VITE7+P4.QACTIVITE8+P4.QACTIVITE9+P4.QACTIVITE_10+P4.QACTIVITE_11+P4.QACTIVITE_12];
[P4.SUMSOL =
P4.P8QSOL1+P4.P8QSOL2+P4.P8QSOL3+P4.P8QSOL4+P4.P8QSOL5+P4.P8QSOL6+P4.P8QSOL7+P4.P8QSOL8
+P4.P8QSOL9];
IF (P4.QACTIVITE1 EQ 101) [P4.AP1 = 'marche rapide'];
IF ((P4.QACTIVITE1 NE 101) AND (P4.QACTIVITE2 EQ 102)) [P4.AP1 = 'raquettes à neige'];
IF ((P4.QACTIVITE1 NE 101) AND (P4.QACTIVITE2 NE 102) AND (P4.QACTIVITE3 EQ 103)) [P4.AP1 = 'vélo'];
IF ((P4.QACTIVITE1 NE 101) AND (P4.QACTIVITE2 NE 102) AND (P4.QACTIVITE3 NE 103) AND
(P4.QACTIVITE4 EQ 104)) [P4.AP1 = 'natation'];
IF ((P4.QACTIVITE1 NE 101) AND (P4.QACTIVITE2 NE 102) AND (P4.QACTIVITE3 NE 103) AND
(P4.QACTIVITE4 NE 104) AND (P4.QACTIVITE5 EQ 105)) [P4.AP1 = 'ski de fond'];
IF ((P4.QACTIVITE1 NE 101) AND (P4.QACTIVITE2 NE 102) AND (P4.QACTIVITE3 NE 103) AND
(P4.QACTIVITE4 NE 104) AND (P4.QACTIVITE5 NE 105) AND (P4.QACTIVITE6 EQ 106)) [P4.AP1 = 'tennis'];
IF ((P4.QACTIVITE1 NE 101) AND (P4.QACTIVITE2 NE 102) AND (P4.QACTIVITE3 NE 103) AND
(P4.QACTIVITE4 NE 104) AND (P4.QACTIVITE5 NE 105) AND (P4.QACTIVITE6 NE 106) AND (P4.QACTIVITE7
EQ 107)) [P4.AP1 = 'course à pieds'];
IF ((P4.QACTIVITE1 NE 101) AND (P4.QACTIVITE2 NE 102) AND (P4.QACTIVITE3 NE 103) AND
(P4.QACTIVITE4 NE 104) AND (P4.QACTIVITE5 NE 105) AND (P4.QACTIVITE6 NE 106) AND (P4.QACTIVITE7
NE 107) AND (P4.QACTIVITE8 EQ 108)) [P4.AP1 = 'hockey'];
IF ((P4.QACTIVITE1 NE 101) AND (P4.QACTIVITE2 NE 102) AND (P4.QACTIVITE3 NE 103) AND
(P4.QACTIVITE4 NE 104) AND (P4.QACTIVITE5 NE 105) AND (P4.QACTIVITE6 NE 106) AND (P4.QACTIVITE7
NE 107) AND (P4.QACTIVITE8 NE 108) AND (P4.QACTIVITE9 EQ 109)) [P4.AP1 = 'basketball'];
```

IF ((P4.QACTIVITE1 NE 101) AND (P4.QACTIVITE2 NE 102) AND (P4.QACTIVITE3 NE 103) AND (P4.QACTIVITE4 NE 104) AND (P4.QACTIVITE5 NE 105) AND (P4.QACTIVITE6 NE 106) AND (P4.QACTIVITE7 NE 107) AND (P4.QACTIVITE8 NE 108) AND (P4.QACTIVITE9 NE 109) AND (P4.QACTIVITE_10 EQ 110)) [P4.AP1 = 'soccer'];
IF ((P4.QACTIVITE1 NE 101) AND (P4.QACTIVITE2 NE 102) AND (P4.QACTIVITE3 NE 103) AND (P4.QACTIVITE4 NE 104) AND (P4.QACTIVITE5 NE 105) AND (P4.QACTIVITE6 NE 106) AND (P4.QACTIVITE7 NE 107) AND (P4.QACTIVITE8 NE 108) AND (P4.QACTIVITE9 NE 109) AND (P4.QACTIVITE_10 NE 110) AND (P4.QACTIVITE_11 EQ 111)) [P4.AP1 = 'centre de conditionnement'];
IF ((P4.QACTIVITE1 NE 101) AND (P4.QACTIVITE2 NE 102) AND (P4.QACTIVITE3 NE 103) AND (P4.QACTIVITE4 NE 104) AND (P4.QACTIVITE5 NE 105) AND (P4.QACTIVITE6 NE 106) AND (P4.QACTIVITE7 NE 107) AND (P4.QACTIVITE8 NE 108) AND (P4.QACTIVITE9 NE 109) AND (P4.QACTIVITE_10 NE 110) AND (P4.QACTIVITE_11 NE 111) AND (P4.QACTIVITE_12 EQ 112)) [P4.AP1 = P4.QAOPEN];
IF ((P4.2SUMAP2 GE 100) AND (P4.2SUMAP2 LT 200) AND (P4.QACTIVITE2 EQ 102)) [P4.AP2 = 'raquettes à neige'];
IF ((P4.3SUMAP2 GE 100) AND (P4.3SUMAP2 LT 200) AND (P4.QACTIVITE3 EQ 103)) [P4.AP2 = 'vélo'];
IF ((P4.4SUMAP2 GE 100) AND (P4.4SUMAP2 LT 200) AND (P4.QACTIVITE4 EQ 104)) [P4.AP2 = 'natation'];
IF ((P4.5SUMAP2 GE 100) AND (P4.5SUMAP2 LT 200) AND (P4.QACTIVITE5 EQ 105)) [P4.AP2 = 'ski de fond'];
IF ((P4.6SUMAP2 GE 100) AND (P4.6SUMAP2 LT 200) AND (P4.QACTIVITE6 EQ 106)) [P4.AP2 = 'tennis'];
IF ((P4.7SUMAP2 GE 100) AND (P4.7SUMAP2 LT 200) AND (P4.QACTIVITE7 EQ 107)) [P4.AP2 = 'course à pieds'];
IF ((P4.8SUMAP2 GE 100) AND (P4.8SUMAP2 LT 200) AND (P4.QACTIVITE8 EQ 108)) [P4.AP2 = 'hockey'];
IF ((P4.9SUMAP2 GE 100) AND (P4.9SUMAP2 LT 200) AND (P4.QACTIVITE9 EQ 109)) [P4.AP2 = 'basketball'];
IF ((P4.10SUMAP2 GE 100) AND (P4.10SUMAP2 LT 200) AND (P4.QACTIVITE_10 EQ 110)) [P4.AP2 = 'soccer'];
IF ((P4.11SUMAP2 GE 100) AND (P4.11SUMAP2 LT 200) AND (P4.QACTIVITE_11 EQ 111)) [P4.AP2 = 'centre de conditionnement'];
IF ((P4.12SUMAP2 GE 100) AND (P4.12SUMAP2 LT 200) AND (P4.QACTIVITE_12 EQ 112)) [P4.AP2 = P4.QAOPEN];
IF ((P4.3SUMAP3 GE 200) AND (P4.3SUMAP3 LT 300) AND (P4.QACTIVITE3 EQ 103)) [P4.AP3 = 'vélo'];
IF ((P4.4SUMAP3 GE 200) AND (P4.4SUMAP3 LT 300) AND (P4.QACTIVITE4 EQ 104)) [P4.AP3 = 'natation'];
IF ((P4.5SUMAP3 GE 200) AND (P4.5SUMAP3 LT 300) AND (P4.QACTIVITE5 EQ 105)) [P4.AP3 = 'ski de fond'];
IF ((P4.6SUMAP3 GE 200) AND (P4.6SUMAP3 LT 300) AND (P4.QACTIVITE6 EQ 106)) [P4.AP3 = 'tennis'];
IF ((P4.7SUMAP3 GE 200) AND (P4.7SUMAP3 LT 300) AND (P4.QACTIVITE7 EQ 107)) [P4.AP3 = 'course à pieds'];
IF ((P4.8SUMAP3 GE 200) AND (P4.8SUMAP3 LT 300) AND (P4.QACTIVITE8 EQ 108)) [P4.AP3 = 'hockey'];
IF ((P4.9SUMAP3 GE 200) AND (P4.9SUMAP3 LT 300) AND (P4.QACTIVITE9 EQ 109)) [P4.AP3 = 'basketball'];
IF ((P4.10SUMAP3 GE 200) AND (P4.10SUMAP3 LT 300) AND (P4.QACTIVITE_10 EQ 110)) [P4.AP3 = 'soccer'];
IF ((P4.11SUMAP3 GE 200) AND (P4.11SUMAP3 LT 300) AND (P4.QACTIVITE_11 EQ 111)) [P4.AP3 = 'centre de conditionnement'];
IF ((P4.12SUMAP3 GE 200) AND (P4.12SUMAP3 LT 300) AND (P4.QACTIVITE_12 EQ 112)) [P4.AP3 = P4.QAOPEN];

ACTIVITÉ 1 DANS LE PLAN POUR CHAQUE JOUR DE LA SEMAINE
IF ((P4.QACTIVITE1 EQ 101) AND (P4.AP1JOUR EQ 1)) [P4.LUNDIAP1 = P4.AP1];
IF ((P4.QACTIVITE1 NE 101) AND (P4.QACTIVITE2 EQ 102) AND (P4.AP2JOUR EQ 1)) [P4.LUNDIAP1 = P4.AP1];
IF ((P4.QACTIVITE1 NE 101) AND (P4.QACTIVITE2 NE 102) AND (P4.QACTIVITE3 EQ 103) AND (P4.AP3JOUR EQ 1)) [P4.LUNDIAP1 = P4.AP1];
IF ((P4.QACTIVITE1 NE 101) AND (P4.QACTIVITE2 NE 102) AND (P4.QACTIVITE3 NE 103) AND (P4.QACTIVITE4 EQ 104) AND (P4.AP4JOUR EQ 1)) [P4.LUNDIAP1 = P4.AP1];
IF ((P4.QACTIVITE1 NE 101) AND (P4.QACTIVITE2 NE 102) AND (P4.QACTIVITE3 NE 103) AND (P4.QACTIVITE4 NE 104) AND (P4.QACTIVITE5 EQ 105) AND (P4.AP5JOUR EQ 1)) [P4.LUNDIAP1 = P4.AP1];
IF ((P4.QACTIVITE1 NE 101) AND (P4.QACTIVITE2 NE 102) AND (P4.QACTIVITE3 NE 103) AND (P4.QACTIVITE4 NE 104) AND (P4.QACTIVITE5 NE 105) AND (P4.QACTIVITE6 EQ 106) AND (P4.AP6JOUR EQ 1)) [P4.LUNDIAP1 = P4.AP1];
IF ((P4.QACTIVITE1 NE 101) AND (P4.QACTIVITE2 NE 102) AND (P4.QACTIVITE3 NE 103) AND (P4.QACTIVITE4 NE 104) AND (P4.QACTIVITE5 NE 105) AND (P4.QACTIVITE6 NE 106) AND (P4.QACTIVITE7 EQ 107) AND (P4.AP7JOUR EQ 1)) [P4.LUNDIAP1 = P4.AP1];
IF ((P4.QACTIVITE1 NE 101) AND (P4.QACTIVITE2 NE 102) AND (P4.QACTIVITE3 NE 103) AND (P4.QACTIVITE4 NE 104) AND (P4.QACTIVITE5 NE 105) AND (P4.QACTIVITE6 NE 106) AND (P4.QACTIVITE7 NE 107) AND (P4.QACTIVITE8 EQ 108) AND (P4.AP8JOUR EQ 1)) [P4.LUNDIAP1 = P4.AP1];
IF ((P4.QACTIVITE1 NE 101) AND (P4.QACTIVITE2 NE 102) AND (P4.QACTIVITE3 NE 103) AND (P4.QACTIVITE4 NE 104) AND (P4.QACTIVITE5 NE 105) AND (P4.QACTIVITE6 NE 106) AND (P4.QACTIVITE7 NE 107) AND (P4.QACTIVITE8 NE 108) AND (P4.QACTIVITE9 EQ 109) AND (P4.AP9JOUR EQ 1)) [P4.LUNDIAP1 = P4.AP1];
IF ((P4.QACTIVITE1 NE 101) AND (P4.QACTIVITE2 NE 102) AND (P4.QACTIVITE3 NE 103) AND (P4.QACTIVITE4 NE 104) AND (P4.QACTIVITE5 NE 105) AND (P4.QACTIVITE6 NE 106) AND (P4.QACTIVITE7 NE 107) AND (P4.QACTIVITE8 NE 108) AND (P4.QACTIVITE9 NE 109) AND (P4.QACTIVITE_10 EQ 110) AND (P4.AP10JOUR EQ 1)) [P4.LUNDIAP1 = P4.AP1];
IF ((P4.QACTIVITE1 NE 101) AND (P4.QACTIVITE2 NE 102) AND (P4.QACTIVITE3 NE 103) AND (P4.QACTIVITE4 NE 104) AND (P4.QACTIVITE5 NE 105) AND (P4.QACTIVITE6 NE 106) AND (P4.QACTIVITE7 NE 107) AND (P4.QACTIVITE8 NE 108) AND (P4.QACTIVITE9 NE 109) AND (P4.QACTIVITE_10 NE 110) AND (P4.QACTIVITE_11 EQ 111) AND (P4.AP11JOUR EQ 1)) [P4.LUNDIAP1 = P4.AP1];
IF ((P4.QACTIVITE1 NE 101) AND (P4.QACTIVITE2 NE 102) AND (P4.QACTIVITE3 NE 103) AND (P4.QACTIVITE4 NE 104) AND (P4.QACTIVITE5 NE 105) AND (P4.QACTIVITE6 NE 106) AND (P4.QACTIVITE7 NE 107) AND (P4.QACTIVITE8 NE 108) AND (P4.QACTIVITE9 NE 109) AND (P4.QACTIVITE_10 NE 110) AND (P4.QACTIVITE_11 NE 111) AND (P4.QACTIVITE_12 EQ 112) AND (P4.AP12JOUR EQ 1)) [P4.LUNDIAP1 = P4.AP1];
IF ((P4.QACTIVITE1 EQ 101) AND (P4.AP1JOUR EQ 2)) [P4.MARDIAP1 = P4.AP1];
IF ((P4.QACTIVITE1 NE 101) AND (P4.QACTIVITE2 EQ 102) AND (P4.AP2JOUR EQ 2)) [P4.MARDIAP1 = P4.AP1];
IF ((P4.QACTIVITE1 NE 101) AND (P4.QACTIVITE2 NE 102) AND (P4.QACTIVITE3 EQ 103) AND (P4.AP3JOUR EQ 2)) [P4.MARDIAP1 = P4.AP1];

IF ((P4.QACTIVITE1 NE 101) AND (P4.QACTIVITE2 NE 102) AND (P4.QACTIVITE3 NE 103) AND (P4.QACTIVITE4 EQ 104) AND (P4.AP4JOUR EQ 2)) [P4.MARDIAP1 = P4.AP1];
IF ((P4.QACTIVITE1 NE 101) AND (P4.QACTIVITE2 NE 102) AND (P4.QACTIVITE3 NE 103) AND (P4.QACTIVITE4 NE 104) AND (P4.QACTIVITE5 EQ 105) AND (P4.AP5JOUR EQ 2)) [P4.MARDIAP1 = P4.AP1];
IF ((P4.QACTIVITE1 NE 101) AND (P4.QACTIVITE2 NE 102) AND (P4.QACTIVITE3 NE 103) AND (P4.QACTIVITE4 NE 104) AND (P4.QACTIVITE5 NE 105) AND (P4.QACTIVITE6 EQ 106) AND (P4.AP6JOUR EQ 2)) [P4.MARDIAP1 = P4.AP1];
IF ((P4.QACTIVITE1 NE 101) AND (P4.QACTIVITE2 NE 102) AND (P4.QACTIVITE3 NE 103) AND (P4.QACTIVITE4 NE 104) AND (P4.QACTIVITE5 NE 105) AND (P4.QACTIVITE6 NE 106) AND (P4.QACTIVITE7 EQ 107) AND (P4.AP7JOUR EQ 2)) [P4.MARDIAP1 = P4.AP1];
IF ((P4.QACTIVITE1 NE 101) AND (P4.QACTIVITE2 NE 102) AND (P4.QACTIVITE3 NE 103) AND (P4.QACTIVITE4 NE 104) AND (P4.QACTIVITE5 NE 105) AND (P4.QACTIVITE6 NE 106) AND (P4.QACTIVITE7 NE 107) AND (P4.QACTIVITE8 EQ 108) AND (P4.AP8JOUR EQ 2)) [P4.MARDIAP1 = P4.AP1];
IF ((P4.QACTIVITE1 NE 101) AND (P4.QACTIVITE2 NE 102) AND (P4.QACTIVITE3 NE 103) AND (P4.QACTIVITE4 NE 104) AND (P4.QACTIVITE5 NE 105) AND (P4.QACTIVITE6 NE 106) AND (P4.QACTIVITE7 NE 107) AND (P4.QACTIVITE8 NE 108) AND (P4.QACTIVITE9 EQ 109) AND (P4.AP9JOUR EQ 2)) [P4.MARDIAP1 = P4.AP1];
IF ((P4.QACTIVITE1 NE 101) AND (P4.QACTIVITE2 NE 102) AND (P4.QACTIVITE3 NE 103) AND (P4.QACTIVITE4 NE 104) AND (P4.QACTIVITE5 NE 105) AND (P4.QACTIVITE6 NE 106) AND (P4.QACTIVITE7 NE 107) AND (P4.QACTIVITE8 NE 108) AND (P4.QACTIVITE9 NE 109) AND (P4.QACTIVITE_10 EQ 110) AND (P4.AP10JOUR EQ 2)) [P4.MARDIAP1 = P4.AP1];
IF ((P4.QACTIVITE1 NE 101) AND (P4.QACTIVITE2 NE 102) AND (P4.QACTIVITE3 NE 103) AND (P4.QACTIVITE4 NE 104) AND (P4.QACTIVITE5 NE 105) AND (P4.QACTIVITE6 NE 106) AND (P4.QACTIVITE7 NE 107) AND (P4.QACTIVITE8 NE 108) AND (P4.QACTIVITE9 NE 109) AND (P4.QACTIVITE_10 NE 110) AND (P4.QACTIVITE_11 EQ 111) AND (P4.AP11JOUR EQ 2)) [P4.MARDIAP1 = P4.AP1];
IF ((P4.QACTIVITE1 NE 101) AND (P4.QACTIVITE2 NE 102) AND (P4.QACTIVITE3 NE 103) AND (P4.QACTIVITE4 NE 104) AND (P4.QACTIVITE5 NE 105) AND (P4.QACTIVITE6 NE 106) AND (P4.QACTIVITE7 NE 107) AND (P4.QACTIVITE8 NE 108) AND (P4.QACTIVITE9 NE 109) AND (P4.QACTIVITE_10 NE 110) AND (P4.QACTIVITE_11 NE 111) AND (P4.QACTIVITE_12 EQ 112) AND (P4.AP12JOUR EQ 2)) [P4.MARDIAP1 = P4.AP1];
IF ((P4.QACTIVITE1 EQ 101) AND (P4.AP1JOUR EQ 3)) [P4.MERCREDIAP1 = P4.AP1];
IF ((P4.QACTIVITE1 NE 101) AND (P4.QACTIVITE2 EQ 102) AND (P4.AP2JOUR EQ 3)) [P4.MERCREDIAP1 = P4.AP1];
IF ((P4.QACTIVITE1 NE 101) AND (P4.QACTIVITE2 NE 102) AND (P4.QACTIVITE3 EQ 103) AND (P4.AP3JOUR EQ 3)) [P4.MERCREDIAP1 = P4.AP1];
IF ((P4.QACTIVITE1 NE 101) AND (P4.QACTIVITE2 NE 102) AND (P4.QACTIVITE3 NE 103) AND (P4.QACTIVITE4 EQ 104) AND (P4.AP4JOUR EQ 3)) [P4.MERCREDIAP1 = P4.AP1];
IF ((P4.QACTIVITE1 NE 101) AND (P4.QACTIVITE2 NE 102) AND (P4.QACTIVITE3 NE 103) AND (P4.QACTIVITE4 NE 104) AND (P4.QACTIVITE5 EQ 105) AND (P4.AP5JOUR EQ 3)) [P4.MERCREDIAP1 = P4.AP1];

IF ((P4.QACTIVITE1 NE 101) AND (P4.QACTIVITE2 NE 102) AND (P4.QACTIVITE3 NE 103) AND (P4.QACTIVITE4 NE 104) AND (P4.QACTIVITES5 NE 105) AND (P4.QACTIVITE6 EQ 106) AND (P4.AP6JOUR EQ 3)) [P4.MERCREDIAP1 = P4.AP1];
IF ((P4.QACTIVITE1 NE 101) AND (P4.QACTIVITE2 NE 102) AND (P4.QACTIVITE3 NE 103) AND (P4.QACTIVITE4 NE 104) AND (P4.QACTIVITES5 NE 105) AND (P4.QACTIVITE6 NE 106) AND (P4.QACTIVITE7 EQ 107) AND (P4.AP7JOUR EQ 3)) [P4.MERCREDIAP1 = P4.AP1];
IF ((P4.QACTIVITE1 NE 101) AND (P4.QACTIVITE2 NE 102) AND (P4.QACTIVITE3 NE 103) AND (P4.QACTIVITE4 NE 104) AND (P4.QACTIVITES5 NE 105) AND (P4.QACTIVITE6 NE 106) AND (P4.QACTIVITE7 NE 107) AND (P4.QACTIVITE8 EQ 108) AND (P4.AP8JOUR EQ 3)) [P4.MERCREDIAP1 = P4.AP1];
IF ((P4.QACTIVITE1 NE 101) AND (P4.QACTIVITE2 NE 102) AND (P4.QACTIVITE3 NE 103) AND (P4.QACTIVITE4 NE 104) AND (P4.QACTIVITES5 NE 105) AND (P4.QACTIVITE6 NE 106) AND (P4.QACTIVITE7 NE 107) AND (P4.QACTIVITE8 NE 108) AND (P4.QACTIVITE9 EQ 109) AND (P4.AP9JOUR EQ 3)) [P4.MERCREDIAP1 = P4.AP1];
IF ((P4.QACTIVITE1 NE 101) AND (P4.QACTIVITE2 NE 102) AND (P4.QACTIVITE3 NE 103) AND (P4.QACTIVITE4 NE 104) AND (P4.QACTIVITES5 NE 105) AND (P4.QACTIVITE6 NE 106) AND (P4.QACTIVITE7 NE 107) AND (P4.QACTIVITE8 NE 108) AND (P4.QACTIVITE9 NE 109) AND (P4.QACTIVITE_10 EQ 110) AND (P4.AP10JOUR EQ 3)) [P4.MERCREDIAP1 = P4.AP1];
IF ((P4.QACTIVITE1 NE 101) AND (P4.QACTIVITE2 NE 102) AND (P4.QACTIVITE3 NE 103) AND (P4.QACTIVITE4 NE 104) AND (P4.QACTIVITES5 NE 105) AND (P4.QACTIVITE6 NE 106) AND (P4.QACTIVITE7 NE 107) AND (P4.QACTIVITE8 NE 108) AND (P4.QACTIVITE9 NE 109) AND (P4.QACTIVITE_10 NE 110) AND (P4.QACTIVITE_11 EQ 111) AND (P4.AP11JOUR EQ 3)) [P4.MERCREDIAP1 = P4.AP1];
IF ((P4.QACTIVITE1 NE 101) AND (P4.QACTIVITE2 NE 102) AND (P4.QACTIVITE3 NE 103) AND (P4.QACTIVITE4 NE 104) AND (P4.QACTIVITES5 NE 105) AND (P4.QACTIVITE6 NE 106) AND (P4.QACTIVITE7 NE 107) AND (P4.QACTIVITE8 NE 108) AND (P4.QACTIVITE9 NE 109) AND (P4.QACTIVITE_10 NE 110) AND (P4.QACTIVITE_11 NE 111) AND (P4.QACTIVITE_12 EQ 112) AND (P4.AP12JOUR EQ 3)) [P4.MERCREDIAP1 = P4.AP1];
IF ((P4.QACTIVITE1 EQ 101) AND (P4.AP1JOUR EQ 4)) [P4.JEUDIAP1 = P4.AP1];
IF ((P4.QACTIVITE1 NE 101) AND (P4.QACTIVITE2 EQ 102) AND (P4.AP2JOUR EQ 4)) [P4.JEUDIAP1 = P4.AP1];
IF ((P4.QACTIVITE1 NE 101) AND (P4.QACTIVITE2 NE 102) AND (P4.QACTIVITE3 EQ 103) AND (P4.AP3JOUR EQ 4)) [P4.JEUDIAP1 = P4.AP1];
IF ((P4.QACTIVITE1 NE 101) AND (P4.QACTIVITE2 NE 102) AND (P4.QACTIVITE3 NE 103) AND (P4.QACTIVITE4 EQ 104) AND (P4.AP4JOUR EQ 4)) [P4.JEUDIAP1 = P4.AP1];
IF ((P4.QACTIVITE1 NE 101) AND (P4.QACTIVITE2 NE 102) AND (P4.QACTIVITE3 NE 103) AND (P4.QACTIVITE4 NE 104) AND (P4.QACTIVITES5 EQ 105) AND (P4.AP5JOUR EQ 4)) [P4.JEUDIAP1 = P4.AP1];
IF ((P4.QACTIVITE1 NE 101) AND (P4.QACTIVITE2 NE 102) AND (P4.QACTIVITE3 NE 103) AND (P4.QACTIVITE4 NE 104) AND (P4.QACTIVITES5 NE 105) AND (P4.QACTIVITE6 EQ 106) AND (P4.AP6JOUR EQ 4)) [P4.JEUDIAP1 = P4.AP1];
IF ((P4.QACTIVITE1 NE 101) AND (P4.QACTIVITE2 NE 102) AND (P4.QACTIVITE3 NE 103) AND (P4.QACTIVITE4 NE 104) AND (P4.QACTIVITES5 NE 105) AND (P4.QACTIVITE6 NE 106) AND (P4.QACTIVITE7 EQ 107) AND (P4.AP7JOUR EQ 4)) [P4.JEUDIAP1 = P4.AP1];

IF ((P4.QACTIVITE1 NE 101) AND (P4.QACTIVITE2 NE 102) AND (P4.QACTIVITE3 NE 103) AND (P4.QACTIVITE4 NE 104) AND (P4.QACTIVITE5 NE 105) AND (P4.QACTIVITE6 NE 106) AND (P4.QACTIVITE7 NE 107) AND (P4.QACTIVITE8 EQ 108) AND (P4.AP8JOUR EQ 4)) [P4.JEUDIAP1 = P4.AP1];
IF ((P4.QACTIVITE1 NE 101) AND (P4.QACTIVITE2 NE 102) AND (P4.QACTIVITE3 NE 103) AND (P4.QACTIVITE4 NE 104) AND (P4.QACTIVITE5 NE 105) AND (P4.QACTIVITE6 NE 106) AND (P4.QACTIVITE7 NE 107) AND (P4.QACTIVITE8 NE 108) AND (P4.QACTIVITE9 EQ 109) AND (P4.AP9JOUR EQ 4)) [P4.JEUDIAP1 = P4.AP1];
IF ((P4.QACTIVITE1 NE 101) AND (P4.QACTIVITE2 NE 102) AND (P4.QACTIVITE3 NE 103) AND (P4.QACTIVITE4 NE 104) AND (P4.QACTIVITE5 NE 105) AND (P4.QACTIVITE6 NE 106) AND (P4.QACTIVITE7 NE 107) AND (P4.QACTIVITE8 NE 108) AND (P4.QACTIVITE9 NE 109) AND (P4.QACTIVITE_10 EQ 110) AND (P4.AP10JOUR EQ 4)) [P4.JEUDIAP1 = P4.AP1];
IF ((P4.QACTIVITE1 NE 101) AND (P4.QACTIVITE2 NE 102) AND (P4.QACTIVITE3 NE 103) AND (P4.QACTIVITE4 NE 104) AND (P4.QACTIVITE5 NE 105) AND (P4.QACTIVITE6 NE 106) AND (P4.QACTIVITE7 NE 107) AND (P4.QACTIVITE8 NE 108) AND (P4.QACTIVITE9 NE 109) AND (P4.QACTIVITE_10 NE 110) AND (P4.QACTIVITE_11 EQ 111) AND (P4.AP11JOUR EQ 4)) [P4.JEUDIAP1 = P4.AP1];
IF ((P4.QACTIVITE1 NE 101) AND (P4.QACTIVITE2 NE 102) AND (P4.QACTIVITE3 NE 103) AND (P4.QACTIVITE4 NE 104) AND (P4.QACTIVITE5 NE 105) AND (P4.QACTIVITE6 NE 106) AND (P4.QACTIVITE7 NE 107) AND (P4.QACTIVITE8 NE 108) AND (P4.QACTIVITE9 NE 109) AND (P4.QACTIVITE_10 NE 110) AND (P4.QACTIVITE_11 NE 111) AND (P4.QACTIVITE_12 EQ 112) AND (P4.AP12JOUR EQ 4)) [P4.JEUDIAP1 = P4.AP1];
IF ((P4.QACTIVITE1 EQ 101) AND (P4.AP1JOUR EQ 5)) [P4.VENDREDIAP1 = P4.AP1];
IF ((P4.QACTIVITE1 NE 101) AND (P4.QACTIVITE2 EQ 102) AND (P4.AP2JOUR EQ 5)) [P4.VENDREDIAP1 = P4.AP1];
IF ((P4.QACTIVITE1 NE 101) AND (P4.QACTIVITE2 NE 102) AND (P4.QACTIVITE3 EQ 103) AND (P4.AP3JOUR EQ 5)) [P4.VENDREDIAP1 = P4.AP1];
IF ((P4.QACTIVITE1 NE 101) AND (P4.QACTIVITE2 NE 102) AND (P4.QACTIVITE3 NE 103) AND (P4.QACTIVITE4 EQ 104) AND (P4.AP4JOUR EQ 5)) [P4.VENDREDIAP1 = P4.AP1];
IF ((P4.QACTIVITE1 NE 101) AND (P4.QACTIVITE2 NE 102) AND (P4.QACTIVITE3 NE 103) AND (P4.QACTIVITE4 NE 104) AND (P4.QACTIVITE5 EQ 105) AND (P4.AP5JOUR EQ 5)) [P4.VENDREDIAP1 = P4.AP1];
IF ((P4.QACTIVITE1 NE 101) AND (P4.QACTIVITE2 NE 102) AND (P4.QACTIVITE3 NE 103) AND (P4.QACTIVITE4 NE 104) AND (P4.QACTIVITE5 NE 105) AND (P4.QACTIVITE6 EQ 106) AND (P4.AP6JOUR EQ 5)) [P4.VENDREDIAP1 = P4.AP1];
IF ((P4.QACTIVITE1 NE 101) AND (P4.QACTIVITE2 NE 102) AND (P4.QACTIVITE3 NE 103) AND (P4.QACTIVITE4 NE 104) AND (P4.QACTIVITE5 NE 105) AND (P4.QACTIVITE6 NE 106) AND (P4.QACTIVITE7 EQ 107) AND (P4.AP7JOUR EQ 5)) [P4.VENDREDIAP1 = P4.AP1];
IF ((P4.QACTIVITE1 NE 101) AND (P4.QACTIVITE2 NE 102) AND (P4.QACTIVITE3 NE 103) AND (P4.QACTIVITE4 NE 104) AND (P4.QACTIVITE5 NE 105) AND (P4.QACTIVITE6 NE 106) AND (P4.QACTIVITE7 NE 107) AND (P4.QACTIVITE8 EQ 108) AND (P4.AP8JOUR EQ 5)) [P4.VENDREDIAP1 = P4.AP1];
IF ((P4.QACTIVITE1 NE 101) AND (P4.QACTIVITE2 NE 102) AND (P4.QACTIVITE3 NE 103) AND (P4.QACTIVITE4 NE 104) AND (P4.QACTIVITE5 NE 105) AND (P4.QACTIVITE6 NE 106) AND (P4.QACTIVITE7

NE 107) AND (P4.QACTIVITE8 NE 108) AND (P4.QACTIVITE9 EQ 109) AND (P4.AP9JOUR EQ 5))
[P4.VENDREDIAP1 = P4.AP1];
IF ((P4.QACTIVITE1 NE 101) AND (P4.QACTIVITE2 NE 102) AND (P4.QACTIVITE3 NE 103) AND (P4.QACTIVITE4 NE 104) AND (P4.QACTIVITES5 NE 105) AND (P4.QACTIVITE6 NE 106) AND (P4.QACTIVITE7 NE 107) AND (P4.QACTIVITE8 NE 108) AND (P4.QACTIVITE9 NE 109) AND (P4.QACTIVITE_10 EQ 110) AND (P4.AP10JOUR EQ 5)) [P4.VENDREDIAP1 = P4.AP1];
IF ((P4.QACTIVITE1 NE 101) AND (P4.QACTIVITE2 NE 102) AND (P4.QACTIVITE3 NE 103) AND (P4.QACTIVITE4 NE 104) AND (P4.QACTIVITES5 NE 105) AND (P4.QACTIVITE6 NE 106) AND (P4.QACTIVITE7 NE 107) AND (P4.QACTIVITE8 NE 108) AND (P4.QACTIVITE9 NE 109) AND (P4.QACTIVITE_10 NE 110) AND (P4.QACTIVITE_11 EQ 111) AND (P4.AP11JOUR EQ 5)) [P4.VENDREDIAP1 = P4.AP1];
IF ((P4.QACTIVITE1 NE 101) AND (P4.QACTIVITE2 NE 102) AND (P4.QACTIVITE3 NE 103) AND (P4.QACTIVITE4 NE 104) AND (P4.QACTIVITES5 NE 105) AND (P4.QACTIVITE6 NE 106) AND (P4.QACTIVITE7 NE 107) AND (P4.QACTIVITE8 NE 108) AND (P4.QACTIVITE9 NE 109) AND (P4.QACTIVITE_10 NE 110) AND (P4.QACTIVITE_11 NE 111) AND (P4.QACTIVITE_12 EQ 112) AND (P4.AP12JOUR EQ 5)) [P4.VENDREDIAP1 = P4.AP1];
IF ((P4.QACTIVITE1 EQ 101) AND (P4.AP1JOUR EQ 6)) [P4.SAMEDIAP1 = P4.AP1];
IF ((P4.QACTIVITE1 NE 101) AND (P4.QACTIVITE2 EQ 102) AND (P4.AP2JOUR EQ 6)) [P4.SAMEDIAP1 = P4.AP1];
IF ((P4.QACTIVITE1 NE 101) AND (P4.QACTIVITE2 NE 102) AND (P4.QACTIVITE3 EQ 103) AND (P4.AP3JOUR EQ 6)) [P4.SAMEDIAP1 = P4.AP1];
IF ((P4.QACTIVITE1 NE 101) AND (P4.QACTIVITE2 NE 102) AND (P4.QACTIVITE3 NE 103) AND (P4.QACTIVITE4 EQ 104) AND (P4.AP4JOUR EQ 6)) [P4.SAMEDIAP1 = P4.AP1];
IF ((P4.QACTIVITE1 NE 101) AND (P4.QACTIVITE2 NE 102) AND (P4.QACTIVITE3 NE 103) AND (P4.QACTIVITE4 NE 104) AND (P4.QACTIVITES5 EQ 105) AND (P4.AP5JOUR EQ 6)) [P4.SAMEDIAP1 = P4.AP1];
IF ((P4.QACTIVITE1 NE 101) AND (P4.QACTIVITE2 NE 102) AND (P4.QACTIVITE3 NE 103) AND (P4.QACTIVITE4 NE 104) AND (P4.QACTIVITES5 NE 105) AND (P4.QACTIVITE6 EQ 106) AND (P4.AP6JOUR EQ 6)) [P4.SAMEDIAP1 = P4.AP1];
IF ((P4.QACTIVITE1 NE 101) AND (P4.QACTIVITE2 NE 102) AND (P4.QACTIVITE3 NE 103) AND (P4.QACTIVITE4 NE 104) AND (P4.QACTIVITES5 NE 105) AND (P4.QACTIVITE6 NE 106) AND (P4.QACTIVITE7 EQ 107) AND (P4.AP7JOUR EQ 6)) [P4.SAMEDIAP1 = P4.AP1];
IF ((P4.QACTIVITE1 NE 101) AND (P4.QACTIVITE2 NE 102) AND (P4.QACTIVITE3 NE 103) AND (P4.QACTIVITE4 NE 104) AND (P4.QACTIVITES5 NE 105) AND (P4.QACTIVITE6 NE 106) AND (P4.QACTIVITE7 NE 107) AND (P4.QACTIVITE8 EQ 108) AND (P4.AP8JOUR EQ 6)) [P4.SAMEDIAP1 = P4.AP1];
IF ((P4.QACTIVITE1 NE 101) AND (P4.QACTIVITE2 NE 102) AND (P4.QACTIVITE3 NE 103) AND (P4.QACTIVITE4 NE 104) AND (P4.QACTIVITES5 NE 105) AND (P4.QACTIVITE6 NE 106) AND (P4.QACTIVITE7 NE 107) AND (P4.QACTIVITE8 NE 108) AND (P4.QACTIVITE9 EQ 109) AND (P4.AP9JOUR EQ 6)) [P4.SAMEDIAP1 = P4.AP1];
IF ((P4.QACTIVITE1 NE 101) AND (P4.QACTIVITE2 NE 102) AND (P4.QACTIVITE3 NE 103) AND (P4.QACTIVITE4 NE 104) AND (P4.QACTIVITES5 NE 105) AND (P4.QACTIVITE6 NE 106) AND (P4.QACTIVITE7 NE 107) AND (P4.QACTIVITE8 NE 108) AND (P4.QACTIVITE9 NE 109) AND (P4.QACTIVITE_10 EQ 110) AND (P4.AP10JOUR EQ 6)) [P4.SAMEDIAP1 = P4.AP1];

IF ((P4.QACTIVITE1 NE 101) AND (P4.QACTIVITE2 NE 102) AND (P4.QACTIVITE3 NE 103) AND (P4.QACTIVITE4 NE 104) AND (P4.QACTIVITES5 NE 105) AND (P4.QACTIVITE6 NE 106) AND (P4.QACTIVITE7 NE 107) AND (P4.QACTIVITE8 NE 108) AND (P4.QACTIVITE9 NE 109) AND (P4.QACTIVITE_10 NE 110) AND (P4.QACTIVITE_11 EQ 111) AND (P4.AP11JOUR EQ 6)) [P4.SAMEDIAP1 = P4.AP1];
IF ((P4.QACTIVITE1 NE 101) AND (P4.QACTIVITE2 NE 102) AND (P4.QACTIVITE3 NE 103) AND (P4.QACTIVITE4 NE 104) AND (P4.QACTIVITES5 NE 105) AND (P4.QACTIVITE6 NE 106) AND (P4.QACTIVITE7 NE 107) AND (P4.QACTIVITE8 NE 108) AND (P4.QACTIVITE9 NE 109) AND (P4.QACTIVITE_10 NE 110) AND (P4.QACTIVITE_11 NE 111) AND (P4.QACTIVITE_12 EQ 112) AND (P4.AP12JOUR EQ 6)) [P4.SAMEDIAP1 = P4.AP1];
IF ((P4.QACTIVITE1 EQ 101) AND (P4.AP1JOUR EQ 7)) [P4.DIMANCHEAP1 = P4.AP1];
IF ((P4.QACTIVITE1 NE 101) AND (P4.QACTIVITE2 EQ 102) AND (P4.AP2JOUR EQ 7)) [P4.DIMANCHEAP1 = P4.AP1];
IF ((P4.QACTIVITE1 NE 101) AND (P4.QACTIVITE2 NE 102) AND (P4.QACTIVITE3 EQ 103) AND (P4.AP3JOUR EQ 7)) [P4.DIMANCHEAP1 = P4.AP1];
IF ((P4.QACTIVITE1 NE 101) AND (P4.QACTIVITE2 NE 102) AND (P4.QACTIVITE3 NE 103) AND (P4.QACTIVITE4 EQ 104) AND (P4.AP4JOUR EQ 7)) [P4.DIMANCHEAP1 = P4.AP1];
IF ((P4.QACTIVITE1 NE 101) AND (P4.QACTIVITE2 NE 102) AND (P4.QACTIVITE3 NE 103) AND (P4.QACTIVITE4 NE 104) AND (P4.QACTIVITES5 EQ 105) AND (P4.AP5JOUR EQ 7)) [P4.DIMANCHEAP1 = P4.AP1];
IF ((P4.QACTIVITE1 NE 101) AND (P4.QACTIVITE2 NE 102) AND (P4.QACTIVITE3 NE 103) AND (P4.QACTIVITE4 NE 104) AND (P4.QACTIVITES5 NE 105) AND (P4.QACTIVITE6 EQ 106) AND (P4.AP6JOUR EQ 7)) [P4.DIMANCHEAP1 = P4.AP1];
IF ((P4.QACTIVITE1 NE 101) AND (P4.QACTIVITE2 NE 102) AND (P4.QACTIVITE3 NE 103) AND (P4.QACTIVITE4 NE 104) AND (P4.QACTIVITES5 NE 105) AND (P4.QACTIVITE6 NE 106) AND (P4.QACTIVITE7 EQ 107) AND (P4.AP7JOUR EQ 7)) [P4.DIMANCHEAP1 = P4.AP1];
IF ((P4.QACTIVITE1 NE 101) AND (P4.QACTIVITE2 NE 102) AND (P4.QACTIVITE3 NE 103) AND (P4.QACTIVITE4 NE 104) AND (P4.QACTIVITES5 NE 105) AND (P4.QACTIVITE6 NE 106) AND (P4.QACTIVITE7 NE 107) AND (P4.QACTIVITE8 EQ 108) AND (P4.AP8JOUR EQ 7)) [P4.DIMANCHEAP1 = P4.AP1];
IF ((P4.QACTIVITE1 NE 101) AND (P4.QACTIVITE2 NE 102) AND (P4.QACTIVITE3 NE 103) AND (P4.QACTIVITE4 NE 104) AND (P4.QACTIVITES5 NE 105) AND (P4.QACTIVITE6 NE 106) AND (P4.QACTIVITE7 NE 107) AND (P4.QACTIVITE8 NE 108) AND (P4.QACTIVITE9 EQ 109) AND (P4.AP9JOUR EQ 7)) [P4.DIMANCHEAP1 = P4.AP1];
IF ((P4.QACTIVITE1 NE 101) AND (P4.QACTIVITE2 NE 102) AND (P4.QACTIVITE3 NE 103) AND (P4.QACTIVITE4 NE 104) AND (P4.QACTIVITES5 NE 105) AND (P4.QACTIVITE6 NE 106) AND (P4.QACTIVITE7 NE 107) AND (P4.QACTIVITE8 NE 108) AND (P4.QACTIVITE9 NE 109) AND (P4.QACTIVITE_10 EQ 110) AND (P4.AP10JOUR EQ 7)) [P4.DIMANCHEAP1 = P4.AP1];
IF ((P4.QACTIVITE1 NE 101) AND (P4.QACTIVITE2 NE 102) AND (P4.QACTIVITE3 NE 103) AND (P4.QACTIVITE4 NE 104) AND (P4.QACTIVITES5 NE 105) AND (P4.QACTIVITE6 NE 106) AND (P4.QACTIVITE7 NE 107) AND (P4.QACTIVITE8 NE 108) AND (P4.QACTIVITE9 NE 109) AND (P4.QACTIVITE_10 NE 110) AND (P4.QACTIVITE_11 EQ 111) AND (P4.AP11JOUR EQ 7)) [P4.DIMANCHEAP1 = P4.AP1];
IF ((P4.QACTIVITE1 NE 101) AND (P4.QACTIVITE2 NE 102) AND (P4.QACTIVITE3 NE 103) AND (P4.QACTIVITE4 NE 104) AND (P4.QACTIVITES5 NE 105) AND (P4.QACTIVITE6 NE 106) AND (P4.QACTIVITE7

NE 107) AND (P4.QACTIVITE8 NE 108) AND (P4.QACTIVITE9 NE 109) AND (P4.QACTIVITE_10 NE 110) AND (P4.QACTIVITE_11 NE 111) AND (P4.QACTIVITE_12 EQ 112) AND (P4.AP12JOUR EQ 7)) [P4.DIMANCHEAP1 = P4.AP1];
ACTIVITÉ 2 DANS LE PLAN POUR CHAQUE JOUR DE LA SEMAINE
IF ((P4.2SUMAP2 GE 100) AND (P4.2SUMAP2 LT 200) AND (P4.QACTIVITE2 EQ 102) AND (P4.AP2JOUR EQ 1)) [P4.LUNDIAP2 = P4.AP2];
IF ((P4.3SUMAP2 GE 100) AND (P4.3SUMAP2 LT 200) AND (P4.QACTIVITE3 EQ 103) AND (P4.AP3JOUR EQ 1)) [P4.LUNDIAP2 = P4.AP2];
IF ((P4.4SUMAP2 GE 100) AND (P4.4SUMAP2 LT 200) AND (P4.QACTIVITE4 EQ 104) AND (P4.AP4JOUR EQ 1)) [P4.LUNDIAP2 = P4.AP2];
IF ((P4.5SUMAP2 GE 100) AND (P4.5SUMAP2 LT 200) AND (P4.QACTIVITE5 EQ 105) AND (P4.AP5JOUR EQ 1)) [P4.LUNDIAP2 = P4.AP2];
IF ((P4.6SUMAP2 GE 100) AND (P4.6SUMAP2 LT 200) AND (P4.QACTIVITE6 EQ 106) AND (P4.AP6JOUR EQ 1)) [P4.LUNDIAP2 = P4.AP2];
IF ((P4.7SUMAP2 GE 100) AND (P4.7SUMAP2 LT 200) AND (P4.QACTIVITE7 EQ 107) AND (P4.AP7JOUR EQ 1)) [P4.LUNDIAP2 = P4.AP2];
IF ((P4.8SUMAP2 GE 100) AND (P4.8SUMAP2 LT 200) AND (P4.QACTIVITE8 EQ 108) AND (P4.AP8JOUR EQ 1)) [P4.LUNDIAP2 = P4.AP2];
IF ((P4.9SUMAP2 GE 100) AND (P4.9SUMAP2 LT 200) AND (P4.QACTIVITE9 EQ 109) AND (P4.AP9JOUR EQ 1)) [P4.LUNDIAP2 = P4.AP2];
IF ((P4.10SUMAP2 GE 100) AND (P4.10SUMAP2 LT 200) AND (P4.QACTIVITE_10 EQ 110) AND (P4.AP10JOUR EQ 1)) [P4.LUNDIAP2 = P4.AP2];
IF ((P4.11SUMAP2 GE 100) AND (P4.11SUMAP2 LT 200) AND (P4.QACTIVITE_11 EQ 111) AND (P4.AP11JOUR EQ 1)) [P4.LUNDIAP2 = P4.AP2];
IF ((P4.12SUMAP2 GE 100) AND (P4.12SUMAP2 LT 200) AND (P4.QACTIVITE_12 EQ 112) AND (P4.AP12JOUR EQ 1)) [P4.LUNDIAP2 = P4.AP2];
IF ((P4.2SUMAP2 GE 100) AND (P4.2SUMAP2 LT 200) AND (P4.QACTIVITE2 EQ 102) AND (P4.AP2JOUR EQ 2)) [P4.MARDIAP2 = P4.AP2];
IF ((P4.3SUMAP2 GE 100) AND (P4.3SUMAP2 LT 200) AND (P4.QACTIVITE3 EQ 103) AND (P4.AP3JOUR EQ 2)) [P4.MARDIAP2 = P4.AP2];
IF ((P4.4SUMAP2 GE 100) AND (P4.4SUMAP2 LT 200) AND (P4.QACTIVITE4 EQ 104) AND (P4.AP4JOUR EQ 2)) [P4.MARDIAP2 = P4.AP2];
IF ((P4.5SUMAP2 GE 100) AND (P4.5SUMAP2 LT 200) AND (P4.QACTIVITE5 EQ 105) AND (P4.AP5JOUR EQ 2)) [P4.MARDIAP2 = P4.AP2];
IF ((P4.6SUMAP2 GE 100) AND (P4.6SUMAP2 LT 200) AND (P4.QACTIVITE6 EQ 106) AND (P4.AP6JOUR EQ 2)) [P4.MARDIAP2 = P4.AP2];
IF ((P4.7SUMAP2 GE 100) AND (P4.7SUMAP2 LT 200) AND (P4.QACTIVITE7 EQ 107) AND (P4.AP7JOUR EQ 2)) [P4.MARDIAP2 = P4.AP2];
IF ((P4.8SUMAP2 GE 100) AND (P4.8SUMAP2 LT 200) AND (P4.QACTIVITE8 EQ 108) AND (P4.AP8JOUR EQ 2)) [P4.MARDIAP2 = P4.AP2];
IF ((P4.9SUMAP2 GE 100) AND (P4.9SUMAP2 LT 200) AND (P4.QACTIVITE9 EQ 109) AND (P4.AP9JOUR EQ 2)) [P4.MARDIAP2 = P4.AP2];

IF ((P4.10SUMAP2 GE 100) AND (P4.10SUMAP2 LT 200) AND (P4.QACTIVITE_10 EQ 110) AND (P4.AP10JOUR EQ 2)) [P4.MARDIAP2 = P4.AP2];
IF ((P4.11SUMAP2 GE 100) AND (P4.11SUMAP2 LT 200) AND (P4.QACTIVITE_11 EQ 111) AND (P4.AP11JOUR EQ 2)) [P4.MARDIAP2 = P4.AP2];
IF ((P4.12SUMAP2 GE 100) AND (P4.12SUMAP2 LT 200) AND (P4.QACTIVITE_12 EQ 112) AND (P4.AP12JOUR EQ 2)) [P4.MARDIAP2 = P4.AP2];
IF ((P4.2SUMAP2 GE 100) AND (P4.2SUMAP2 LT 200) AND (P4.QACTIVITE2 EQ 102) AND (P4.AP2JOUR EQ 3)) [P4.MERCREDIAP2 = P4.AP2];
IF ((P4.3SUMAP2 GE 100) AND (P4.3SUMAP2 LT 200) AND (P4.QACTIVITE3 EQ 103) AND (P4.AP3JOUR EQ 3)) [P4.MERCREDIAP2 = P4.AP2];
IF ((P4.4SUMAP2 GE 100) AND (P4.4SUMAP2 LT 200) AND (P4.QACTIVITE4 EQ 104) AND (P4.AP4JOUR EQ 3)) [P4.MERCREDIAP2 = P4.AP2];
IF ((P4.5SUMAP2 GE 100) AND (P4.5SUMAP2 LT 200) AND (P4.QACTIVITE5 EQ 105) AND (P4.AP5JOUR EQ 3)) [P4.MERCREDIAP2 = P4.AP2];
IF ((P4.6SUMAP2 GE 100) AND (P4.6SUMAP2 LT 200) AND (P4.QACTIVITE6 EQ 106) AND (P4.AP6JOUR EQ 3)) [P4.MERCREDIAP2 = P4.AP2];
IF ((P4.7SUMAP2 GE 100) AND (P4.7SUMAP2 LT 200) AND (P4.QACTIVITE7 EQ 107) AND (P4.AP7JOUR EQ 3)) [P4.MERCREDIAP2 = P4.AP2];
IF ((P4.8SUMAP2 GE 100) AND (P4.8SUMAP2 LT 200) AND (P4.QACTIVITE8 EQ 108) AND (P4.AP8JOUR EQ 3)) [P4.MERCREDIAP2 = P4.AP2];
IF ((P4.9SUMAP2 GE 100) AND (P4.9SUMAP2 LT 200) AND (P4.QACTIVITE9 EQ 109) AND (P4.AP9JOUR EQ 3)) [P4.MERCREDIAP2 = P4.AP2];
IF ((P4.10SUMAP2 GE 100) AND (P4.10SUMAP2 LT 200) AND (P4.QACTIVITE_10 EQ 110) AND (P4.AP10JOUR EQ 3)) [P4.MERCREDIAP2 = P4.AP2];
IF ((P4.11SUMAP2 GE 100) AND (P4.11SUMAP2 LT 200) AND (P4.QACTIVITE_11 EQ 111) AND (P4.AP11JOUR EQ 3)) [P4.MERCREDIAP2 = P4.AP2];
IF ((P4.12SUMAP2 GE 100) AND (P4.12SUMAP2 LT 200) AND (P4.QACTIVITE_12 EQ 112) AND (P4.AP12JOUR EQ 3)) [P4.MERCREDIAP2 = P4.AP2];
IF ((P4.2SUMAP2 GE 100) AND (P4.2SUMAP2 LT 200) AND (P4.QACTIVITE2 EQ 102) AND (P4.AP2JOUR EQ 4)) [P4.JEUDIAP2 = P4.AP2];
IF ((P4.3SUMAP2 GE 100) AND (P4.3SUMAP2 LT 200) AND (P4.QACTIVITE3 EQ 103) AND (P4.AP3JOUR EQ 4)) [P4.JEUDIAP2 = P4.AP2];
IF ((P4.4SUMAP2 GE 100) AND (P4.4SUMAP2 LT 200) AND (P4.QACTIVITE4 EQ 104) AND (P4.AP4JOUR EQ 4)) [P4.JEUDIAP2 = P4.AP2];
IF ((P4.5SUMAP2 GE 100) AND (P4.5SUMAP2 LT 200) AND (P4.QACTIVITE5 EQ 105) AND (P4.AP5JOUR EQ 4)) [P4.JEUDIAP2 = P4.AP2];
IF ((P4.6SUMAP2 GE 100) AND (P4.6SUMAP2 LT 200) AND (P4.QACTIVITE6 EQ 106) AND (P4.AP6JOUR EQ 4)) [P4.JEUDIAP2 = P4.AP2];
IF ((P4.7SUMAP2 GE 100) AND (P4.7SUMAP2 LT 200) AND (P4.QACTIVITE7 EQ 107) AND (P4.AP7JOUR EQ 4)) [P4.JEUDIAP2 = P4.AP2];
IF ((P4.8SUMAP2 GE 100) AND (P4.8SUMAP2 LT 200) AND (P4.QACTIVITE8 EQ 108) AND (P4.AP8JOUR EQ 4)) [P4.JEUDIAP2 = P4.AP2];

IF ((P4.9SUMAP2 GE 100) AND (P4.9SUMAP2 LT 200) AND (P4.QACTIVITE9 EQ 109) AND (P4.AP9JOUR EQ 4)) [P4.JEUDIAP2 = P4.AP2];
IF ((P4.10SUMAP2 GE 100) AND (P4.10SUMAP2 LT 200) AND (P4.QACTIVITE_10 EQ 110) AND (P4.AP10JOUR EQ 4)) [P4.JEUDIAP2 = P4.AP2];
IF ((P4.11SUMAP2 GE 100) AND (P4.11SUMAP2 LT 200) AND (P4.QACTIVITE_11 EQ 111) AND (P4.AP11JOUR EQ 4)) [P4.JEUDIAP2 = P4.AP2];
IF ((P4.12SUMAP2 GE 100) AND (P4.12SUMAP2 LT 200) AND (P4.QACTIVITE_12 EQ 112) AND (P4.AP12JOUR EQ 4)) [P4.JEUDIAP2 = P4.AP2];
IF ((P4.2SUMAP2 GE 100) AND (P4.2SUMAP2 LT 200) AND (P4.QACTIVITE2 EQ 102) AND (P4.AP2JOUR EQ 5)) [P4.VENDREDIAP2 = P4.AP2];
IF ((P4.3SUMAP2 GE 100) AND (P4.3SUMAP2 LT 200) AND (P4.QACTIVITE3 EQ 103) AND (P4.AP3JOUR EQ 5)) [P4.VENDREDIAP2 = P4.AP2];
IF ((P4.4SUMAP2 GE 100) AND (P4.4SUMAP2 LT 200) AND (P4.QACTIVITE4 EQ 104) AND (P4.AP4JOUR EQ 5)) [P4.VENDREDIAP2 = P4.AP2];
IF ((P4.5SUMAP2 GE 100) AND (P4.5SUMAP2 LT 200) AND (P4.QACTIVITE5 EQ 105) AND (P4.AP5JOUR EQ 5)) [P4.VENDREDIAP2 = P4.AP2];
IF ((P4.6SUMAP2 GE 100) AND (P4.6SUMAP2 LT 200) AND (P4.QACTIVITE6 EQ 106) AND (P4.AP6JOUR EQ 5)) [P4.VENDREDIAP2 = P4.AP2];
IF ((P4.7SUMAP2 GE 100) AND (P4.7SUMAP2 LT 200) AND (P4.QACTIVITE7 EQ 107) AND (P4.AP7JOUR EQ 5)) [P4.VENDREDIAP2 = P4.AP2];
IF ((P4.8SUMAP2 GE 100) AND (P4.8SUMAP2 LT 200) AND (P4.QACTIVITE8 EQ 108) AND (P4.AP8JOUR EQ 5)) [P4.VENDREDIAP2 = P4.AP2];
IF ((P4.9SUMAP2 GE 100) AND (P4.9SUMAP2 LT 200) AND (P4.QACTIVITE9 EQ 109) AND (P4.AP9JOUR EQ 5)) [P4.VENDREDIAP2 = P4.AP2];
IF ((P4.10SUMAP2 GE 100) AND (P4.10SUMAP2 LT 200) AND (P4.QACTIVITE_10 EQ 110) AND (P4.AP10JOUR EQ 5)) [P4.VENDREDIAP2 = P4.AP2];
IF ((P4.11SUMAP2 GE 100) AND (P4.11SUMAP2 LT 200) AND (P4.QACTIVITE_11 EQ 111) AND (P4.AP11JOUR EQ 5)) [P4.VENDREDIAP2 = P4.AP2];
IF ((P4.12SUMAP2 GE 100) AND (P4.12SUMAP2 LT 200) AND (P4.QACTIVITE_12 EQ 112) AND (P4.AP12JOUR EQ 5)) [P4.VENDREDIAP2 = P4.AP2];
IF ((P4.2SUMAP2 GE 100) AND (P4.2SUMAP2 LT 200) AND (P4.QACTIVITE2 EQ 102) AND (P4.AP2JOUR EQ 6)) [P4.SAMEDIAP2 = P4.AP2];
IF ((P4.3SUMAP2 GE 100) AND (P4.3SUMAP2 LT 200) AND (P4.QACTIVITE3 EQ 103) AND (P4.AP3JOUR EQ 6)) [P4.SAMEDIAP2 = P4.AP2];
IF ((P4.4SUMAP2 GE 100) AND (P4.4SUMAP2 LT 200) AND (P4.QACTIVITE4 EQ 104) AND (P4.AP4JOUR EQ 6)) [P4.SAMEDIAP2 = P4.AP2];
IF ((P4.5SUMAP2 GE 100) AND (P4.5SUMAP2 LT 200) AND (P4.QACTIVITE5 EQ 105) AND (P4.AP5JOUR EQ 6)) [P4.SAMEDIAP2 = P4.AP2];
IF ((P4.6SUMAP2 GE 100) AND (P4.6SUMAP2 LT 200) AND (P4.QACTIVITE6 EQ 106) AND (P4.AP6JOUR EQ 6)) [P4.SAMEDIAP2 = P4.AP2];
IF ((P4.7SUMAP2 GE 100) AND (P4.7SUMAP2 LT 200) AND (P4.QACTIVITE7 EQ 107) AND (P4.AP7JOUR EQ 6)) [P4.SAMEDIAP2 = P4.AP2];

IF ((P4.8SUMAP2 GE 100) AND (P4.8SUMAP2 LT 200) AND (P4.QACTIVITE8 EQ 108) AND (P4.AP8JOUR EQ 6)) [P4.SAMEDIAP2 = P4.AP2];
IF ((P4.9SUMAP2 GE 100) AND (P4.9SUMAP2 LT 200) AND (P4.QACTIVITE9 EQ 109) AND (P4.AP9JOUR EQ 6)) [P4.SAMEDIAP2 = P4.AP2];
IF ((P4.10SUMAP2 GE 100) AND (P4.10SUMAP2 LT 200) AND (P4.QACTIVITE_10 EQ 110) AND (P4.AP10JOUR EQ 6)) [P4.SAMEDIAP2 = P4.AP2];
IF ((P4.11SUMAP2 GE 100) AND (P4.11SUMAP2 LT 200) AND (P4.QACTIVITE_11 EQ 111) AND (P4.AP11JOUR EQ 6)) [P4.SAMEDIAP2 = P4.AP2];
IF ((P4.12SUMAP2 GE 100) AND (P4.12SUMAP2 LT 200) AND (P4.QACTIVITE_12 EQ 112) AND (P4.AP12JOUR EQ 6)) [P4.SAMEDIAP2 = P4.AP2];
IF ((P4.2SUMAP2 GE 100) AND (P4.2SUMAP2 LT 200) AND (P4.QACTIVITE2 EQ 102) AND (P4.AP2JOUR EQ 7)) [P4.DIMANCHEAP2 = P4.AP2];
IF ((P4.3SUMAP2 GE 100) AND (P4.3SUMAP2 LT 200) AND (P4.QACTIVITE3 EQ 103) AND (P4.AP3JOUR EQ 7)) [P4.DIMANCHEAP2 = P4.AP2];
IF ((P4.4SUMAP2 GE 100) AND (P4.4SUMAP2 LT 200) AND (P4.QACTIVITE4 EQ 104) AND (P4.AP4JOUR EQ 7)) [P4.DIMANCHEAP2 = P4.AP2];
IF ((P4.5SUMAP2 GE 100) AND (P4.5SUMAP2 LT 200) AND (P4.QACTIVITE5 EQ 105) AND (P4.AP5JOUR EQ 7)) [P4.DIMANCHEAP2 = P4.AP2];
IF ((P4.6SUMAP2 GE 100) AND (P4.6SUMAP2 LT 200) AND (P4.QACTIVITE6 EQ 106) AND (P4.AP6JOUR EQ 7)) [P4.DIMANCHEAP2 = P4.AP2];
IF ((P4.7SUMAP2 GE 100) AND (P4.7SUMAP2 LT 200) AND (P4.QACTIVITE7 EQ 107) AND (P4.AP7JOUR EQ 7)) [P4.DIMANCHEAP2 = P4.AP2];
IF ((P4.8SUMAP2 GE 100) AND (P4.8SUMAP2 LT 200) AND (P4.QACTIVITE8 EQ 108) AND (P4.AP8JOUR EQ 7)) [P4.DIMANCHEAP2 = P4.AP2];
IF ((P4.9SUMAP2 GE 100) AND (P4.9SUMAP2 LT 200) AND (P4.QACTIVITE9 EQ 109) AND (P4.AP9JOUR EQ 7)) [P4.DIMANCHEAP2 = P4.AP2];
IF ((P4.10SUMAP2 GE 100) AND (P4.10SUMAP2 LT 200) AND (P4.QACTIVITE_10 EQ 110) AND (P4.AP10JOUR EQ 7)) [P4.DIMANCHEAP2 = P4.AP2];
IF ((P4.11SUMAP2 GE 100) AND (P4.11SUMAP2 LT 200) AND (P4.QACTIVITE_11 EQ 111) AND (P4.AP11JOUR EQ 7)) [P4.DIMANCHEAP2 = P4.AP2];
IF ((P4.12SUMAP2 GE 100) AND (P4.12SUMAP2 LT 200) AND (P4.QACTIVITE_12 EQ 112) AND (P4.AP12JOUR EQ 7)) [P4.DIMANCHEAP2 = P4.AP2];
ACTIVITÉ 3 DANS LE PLAN POUR CHAQUE JOUR DE LA SEMAINE
IF ((P4.3SUMAP3 GE 200) AND (P4.3SUMAP3 LT 300) AND (P4.QACTIVITE3 EQ 103) AND (P4.AP3JOUR EQ 1)) [P4.LUNDIAP3 = P4.AP3];
IF ((P4.4SUMAP3 GE 200) AND (P4.4SUMAP3 LT 300) AND (P4.QACTIVITE4 EQ 104) AND (P4.AP4JOUR EQ 1)) [P4.LUNDIAP3 = P4.AP3];
IF ((P4.5SUMAP3 GE 200) AND (P4.5SUMAP3 LT 300) AND (P4.QACTIVITE5 EQ 105) AND (P4.AP5JOUR EQ 1)) [P4.LUNDIAP3 = P4.AP3];
IF ((P4.6SUMAP3 GE 200) AND (P4.6SUMAP3 LT 300) AND (P4.QACTIVITE6 EQ 106) AND (P4.AP6JOUR EQ 1)) [P4.LUNDIAP3 = P4.AP3];

IF ((P4.7SUMAP3 GE 200) AND (P4.7SUMAP3 LT 300) AND (P4.QACTIVITE7 EQ 107) AND (P4.AP7JOUR EQ 1)) [P4.LUNDIAP3 = P4.AP3];
IF ((P4.8SUMAP3 GE 200) AND (P4.8SUMAP3 LT 300) AND (P4.QACTIVITE8 EQ 108) AND (P4.AP8JOUR EQ 1)) [P4.LUNDIAP3 = P4.AP3];
IF ((P4.9SUMAP3 GE 200) AND (P4.9SUMAP3 LT 300) AND (P4.QACTIVITE9 EQ 109) AND (P4.AP9JOUR EQ 1)) [P4.LUNDIAP3 = P4.AP3];
IF ((P4.10SUMAP3 GE 200) AND (P4.10SUMAP3 LT 300) AND (P4.QACTIVITE_10 EQ 110) AND (P4.AP10JOUR EQ 1)) [P4.LUNDIAP3 = P4.AP3];
IF ((P4.11SUMAP3 GE 200) AND (P4.11SUMAP3 LT 300) AND (P4.QACTIVITE_11 EQ 111) AND (P4.AP11JOUR EQ 1)) [P4.LUNDIAP3 = P4.AP3];
IF ((P4.12SUMAP3 GE 200) AND (P4.12SUMAP3 LT 300) AND (P4.QACTIVITE_12 EQ 112) AND (P4.AP12JOUR EQ 1)) [P4.LUNDIAP3 = P4.AP3];
IF ((P4.3SUMAP3 GE 200) AND (P4.3SUMAP3 LT 300) AND (P4.QACTIVITE3 EQ 103) AND (P4.AP3JOUR EQ 2)) [P4.MARDIAP3 = P4.AP3];
IF ((P4.4SUMAP3 GE 200) AND (P4.4SUMAP3 LT 300) AND (P4.QACTIVITE4 EQ 104) AND (P4.AP4JOUR EQ 2)) [P4.MARDIAP3 = P4.AP3];
IF ((P4.5SUMAP3 GE 200) AND (P4.5SUMAP3 LT 300) AND (P4.QACTIVITE5 EQ 105) AND (P4.AP5JOUR EQ 2)) [P4.MARDIAP3 = P4.AP3];
IF ((P4.6SUMAP3 GE 200) AND (P4.6SUMAP3 LT 300) AND (P4.QACTIVITE6 EQ 106) AND (P4.AP6JOUR EQ 2)) [P4.MARDIAP3 = P4.AP3];
IF ((P4.7SUMAP3 GE 200) AND (P4.7SUMAP3 LT 300) AND (P4.QACTIVITE7 EQ 107) AND (P4.AP7JOUR EQ 2)) [P4.MARDIAP3 = P4.AP3];
IF ((P4.8SUMAP3 GE 200) AND (P4.8SUMAP3 LT 300) AND (P4.QACTIVITE8 EQ 108) AND (P4.AP8JOUR EQ 2)) [P4.MARDIAP3 = P4.AP3];
IF ((P4.9SUMAP3 GE 200) AND (P4.9SUMAP3 LT 300) AND (P4.QACTIVITE9 EQ 109) AND (P4.AP9JOUR EQ 2)) [P4.MARDIAP3 = P4.AP3];
IF ((P4.10SUMAP3 GE 200) AND (P4.10SUMAP3 LT 300) AND (P4.QACTIVITE_10 EQ 110) AND (P4.AP10JOUR EQ 2)) [P4.MARDIAP3 = P4.AP3];
IF ((P4.11SUMAP3 GE 200) AND (P4.11SUMAP3 LT 300) AND (P4.QACTIVITE_11 EQ 111) AND (P4.AP11JOUR EQ 2)) [P4.MARDIAP3 = P4.AP3];
IF ((P4.12SUMAP3 GE 200) AND (P4.12SUMAP3 LT 300) AND (P4.QACTIVITE_12 EQ 112) AND (P4.AP12JOUR EQ 2)) [P4.MARDIAP3 = P4.AP3];
IF ((P4.3SUMAP3 GE 200) AND (P4.3SUMAP3 LT 300) AND (P4.QACTIVITE3 EQ 103) AND (P4.AP3JOUR EQ 3)) [P4.MERCREDIAP3 = P4.AP3];
IF ((P4.4SUMAP3 GE 200) AND (P4.4SUMAP3 LT 300) AND (P4.QACTIVITE4 EQ 104) AND (P4.AP4JOUR EQ 3)) [P4.MERCREDIAP3 = P4.AP3];
IF ((P4.5SUMAP3 GE 200) AND (P4.5SUMAP3 LT 300) AND (P4.QACTIVITE5 EQ 105) AND (P4.AP5JOUR EQ 3)) [P4.MERCREDIAP3 = P4.AP3];
IF ((P4.6SUMAP3 GE 200) AND (P4.6SUMAP3 LT 300) AND (P4.QACTIVITE6 EQ 106) AND (P4.AP6JOUR EQ 3)) [P4.MERCREDIAP3 = P4.AP3];
IF ((P4.7SUMAP3 GE 200) AND (P4.7SUMAP3 LT 300) AND (P4.QACTIVITE7 EQ 107) AND (P4.AP7JOUR EQ 3)) [P4.MERCREDIAP3 = P4.AP3];

IF ((P4.8SUMAP3 GE 200) AND (P4.8SUMAP3 LT 300) AND (P4.QACTIVITE8 EQ 108) AND (P4.AP8JOUR EQ 3)) [P4.MERCREDIAP3 = P4.AP3];
IF ((P4.9SUMAP3 GE 200) AND (P4.9SUMAP3 LT 300) AND (P4.QACTIVITE9 EQ 109) AND (P4.AP9JOUR EQ 3)) [P4.MERCREDIAP3 = P4.AP3];
IF ((P4.10SUMAP3 GE 200) AND (P4.10SUMAP3 LT 300) AND (P4.QACTIVITE_10 EQ 110) AND (P4.AP10JOUR EQ 3)) [P4.MERCREDIAP3 = P4.AP3];
IF ((P4.11SUMAP3 GE 200) AND (P4.11SUMAP3 LT 300) AND (P4.QACTIVITE_11 EQ 111) AND (P4.AP11JOUR EQ 3)) [P4.MERCREDIAP3 = P4.AP3];
IF ((P4.12SUMAP3 GE 200) AND (P4.12SUMAP3 LT 300) AND (P4.QACTIVITE_12 EQ 112) AND (P4.AP12JOUR EQ 3)) [P4.MERCREDIAP3 = P4.AP3];
IF ((P4.3SUMAP3 GE 200) AND (P4.3SUMAP3 LT 300) AND (P4.QACTIVITE3 EQ 103) AND (P4.AP3JOUR EQ 4)) [P4.JEUDIAP3 = P4.AP3];
IF ((P4.4SUMAP3 GE 200) AND (P4.4SUMAP3 LT 300) AND (P4.QACTIVITE4 EQ 104) AND (P4.AP4JOUR EQ 4)) [P4.JEUDIAP3 = P4.AP3];
IF ((P4.5SUMAP3 GE 200) AND (P4.5SUMAP3 LT 300) AND (P4.QACTIVITE5 EQ 105) AND (P4.AP5JOUR EQ 4)) [P4.JEUDIAP3 = P4.AP3];
IF ((P4.6SUMAP3 GE 200) AND (P4.6SUMAP3 LT 300) AND (P4.QACTIVITE6 EQ 106) AND (P4.AP6JOUR EQ 4)) [P4.JEUDIAP3 = P4.AP3];
IF ((P4.7SUMAP3 GE 200) AND (P4.7SUMAP3 LT 300) AND (P4.QACTIVITE7 EQ 107) AND (P4.AP7JOUR EQ 4)) [P4.JEUDIAP3 = P4.AP3];
IF ((P4.8SUMAP3 GE 200) AND (P4.8SUMAP3 LT 300) AND (P4.QACTIVITE8 EQ 108) AND (P4.AP8JOUR EQ 4)) [P4.JEUDIAP3 = P4.AP3];
IF ((P4.9SUMAP3 GE 200) AND (P4.9SUMAP3 LT 300) AND (P4.QACTIVITE9 EQ 109) AND (P4.AP9JOUR EQ 4)) [P4.JEUDIAP3 = P4.AP3];
IF ((P4.10SUMAP3 GE 200) AND (P4.10SUMAP3 LT 300) AND (P4.QACTIVITE_10 EQ 110) AND (P4.AP10JOUR EQ 4)) [P4.JEUDIAP3 = P4.AP3];
IF ((P4.11SUMAP3 GE 200) AND (P4.11SUMAP3 LT 300) AND (P4.QACTIVITE_11 EQ 111) AND (P4.AP11JOUR EQ 4)) [P4.JEUDIAP3 = P4.AP3];
IF ((P4.12SUMAP3 GE 200) AND (P4.12SUMAP3 LT 300) AND (P4.QACTIVITE_12 EQ 112) AND (P4.AP12JOUR EQ 4)) [P4.JEUDIAP3 = P4.AP3];
IF ((P4.3SUMAP3 GE 200) AND (P4.3SUMAP3 LT 300) AND (P4.QACTIVITE3 EQ 103) AND (P4.AP3JOUR EQ 5)) [P4.VENDREDIAP3 = P4.AP3];
IF ((P4.4SUMAP3 GE 200) AND (P4.4SUMAP3 LT 300) AND (P4.QACTIVITE4 EQ 104) AND (P4.AP4JOUR EQ 5)) [P4.VENDREDIAP3 = P4.AP3];
IF ((P4.5SUMAP3 GE 200) AND (P4.5SUMAP3 LT 300) AND (P4.QACTIVITE5 EQ 105) AND (P4.AP5JOUR EQ 5)) [P4.VENDREDIAP3 = P4.AP3];
IF ((P4.6SUMAP3 GE 200) AND (P4.6SUMAP3 LT 300) AND (P4.QACTIVITE6 EQ 106) AND (P4.AP6JOUR EQ 5)) [P4.VENDREDIAP3 = P4.AP3];
IF ((P4.7SUMAP3 GE 200) AND (P4.7SUMAP3 LT 300) AND (P4.QACTIVITE7 EQ 107) AND (P4.AP7JOUR EQ 5)) [P4.VENDREDIAP3 = P4.AP3];
IF ((P4.8SUMAP3 GE 200) AND (P4.8SUMAP3 LT 300) AND (P4.QACTIVITE8 EQ 108) AND (P4.AP8JOUR EQ 5)) [P4.VENDREDIAP3 = P4.AP3];

IF ((P4.9SUMAP3 GE 200) AND (P4.9SUMAP3 LT 300) AND (P4.QACTIVITE9 EQ 109) AND (P4.AP9JOUR EQ 5)) [P4.VENDREDIAP3 = P4.AP3];
IF ((P4.10SUMAP3 GE 200) AND (P4.10SUMAP3 LT 300) AND (P4.QACTIVITE_10 EQ 110) AND (P4.AP10JOUR EQ 5)) [P4.VENDREDIAP3 = P4.AP3];
IF ((P4.11SUMAP3 GE 200) AND (P4.11SUMAP3 LT 300) AND (P4.QACTIVITE_11 EQ 111) AND (P4.AP11JOUR EQ 5)) [P4.VENDREDIAP3 = P4.AP3];
IF ((P4.12SUMAP3 GE 200) AND (P4.12SUMAP3 LT 300) AND (P4.QACTIVITE_12 EQ 112) AND (P4.AP12JOUR EQ 5)) [P4.VENDREDIAP3 = P4.AP3];
IF ((P4.3SUMAP3 GE 200) AND (P4.3SUMAP3 LT 300) AND (P4.QACTIVITE3 EQ 103) AND (P4.AP3JOUR EQ 6)) [P4.SAMEDIAP3 = P4.AP3];
IF ((P4.4SUMAP3 GE 200) AND (P4.4SUMAP3 LT 300) AND (P4.QACTIVITE4 EQ 104) AND (P4.AP4JOUR EQ 6)) [P4.SAMEDIAP3 = P4.AP3];
IF ((P4.5SUMAP3 GE 200) AND (P4.5SUMAP3 LT 300) AND (P4.QACTIVITE5 EQ 105) AND (P4.AP5JOUR EQ 6)) [P4.SAMEDIAP3 = P4.AP3];
IF ((P4.6SUMAP3 GE 200) AND (P4.6SUMAP3 LT 300) AND (P4.QACTIVITE6 EQ 106) AND (P4.AP6JOUR EQ 6)) [P4.SAMEDIAP3 = P4.AP3];
IF ((P4.7SUMAP3 GE 200) AND (P4.7SUMAP3 LT 300) AND (P4.QACTIVITE7 EQ 107) AND (P4.AP7JOUR EQ 6)) [P4.SAMEDIAP3 = P4.AP3];
IF ((P4.8SUMAP3 GE 200) AND (P4.8SUMAP3 LT 300) AND (P4.QACTIVITE8 EQ 108) AND (P4.AP8JOUR EQ 6)) [P4.SAMEDIAP3 = P4.AP3];
IF ((P4.9SUMAP3 GE 200) AND (P4.9SUMAP3 LT 300) AND (P4.QACTIVITE9 EQ 109) AND (P4.AP9JOUR EQ 6)) [P4.SAMEDIAP3 = P4.AP3];
IF ((P4.10SUMAP3 GE 200) AND (P4.10SUMAP3 LT 300) AND (P4.QACTIVITE_10 EQ 110) AND (P4.AP10JOUR EQ 6)) [P4.SAMEDIAP3 = P4.AP3];
IF ((P4.11SUMAP3 GE 200) AND (P4.11SUMAP3 LT 300) AND (P4.QACTIVITE_11 EQ 111) AND (P4.AP11JOUR EQ 6)) [P4.SAMEDIAP3 = P4.AP3];
IF ((P4.12SUMAP3 GE 200) AND (P4.12SUMAP3 LT 300) AND (P4.QACTIVITE_12 EQ 112) AND (P4.AP12JOUR EQ 6)) [P4.SAMEDIAP3 = P4.AP3];
IF ((P4.3SUMAP3 GE 200) AND (P4.3SUMAP3 LT 300) AND (P4.QACTIVITE3 EQ 103) AND (P4.AP3JOUR EQ 7)) [P4.DIMANCHEAP3 = P4.AP3];
IF ((P4.4SUMAP3 GE 200) AND (P4.4SUMAP3 LT 300) AND (P4.QACTIVITE4 EQ 104) AND (P4.AP4JOUR EQ 7)) [P4.DIMANCHEAP3 = P4.AP3];
IF ((P4.5SUMAP3 GE 200) AND (P4.5SUMAP3 LT 300) AND (P4.QACTIVITE5 EQ 105) AND (P4.AP5JOUR EQ 7)) [P4.DIMANCHEAP3 = P4.AP3];
IF ((P4.6SUMAP3 GE 200) AND (P4.6SUMAP3 LT 300) AND (P4.QACTIVITE6 EQ 106) AND (P4.AP6JOUR EQ 7)) [P4.DIMANCHEAP3 = P4.AP3];
IF ((P4.7SUMAP3 GE 200) AND (P4.7SUMAP3 LT 300) AND (P4.QACTIVITE7 EQ 107) AND (P4.AP7JOUR EQ 7)) [P4.DIMANCHEAP3 = P4.AP3];
IF ((P4.8SUMAP3 GE 200) AND (P4.8SUMAP3 LT 300) AND (P4.QACTIVITE8 EQ 108) AND (P4.AP8JOUR EQ 7)) [P4.DIMANCHEAP3 = P4.AP3];
IF ((P4.9SUMAP3 GE 200) AND (P4.9SUMAP3 LT 300) AND (P4.QACTIVITE9 EQ 109) AND (P4.AP9JOUR EQ 7)) [P4.DIMANCHEAP3 = P4.AP3];

IF ((P4.10SUMAP3 GE 200) AND (P4.10SUMAP3 LT 300) AND (P4.QACTIVITE_10 EQ 110) AND (P4.AP10JOUR EQ 7)) [P4.DIMANCHEAP3 = P4.AP3];
IF ((P4.11SUMAP3 GE 200) AND (P4.11SUMAP3 LT 300) AND (P4.QACTIVITE_11 EQ 111) AND (P4.AP11JOUR EQ 7)) [P4.DIMANCHEAP3 = P4.AP3];
IF ((P4.12SUMAP3 GE 200) AND (P4.12SUMAP3 LT 300) AND (P4.QACTIVITE_12 EQ 112) AND (P4.AP12JOUR EQ 7)) [P4.DIMANCHEAP3 = P4.AP3];
POURSUITE DU CODE
IF (P4.P7BARCHOICE EQ 101) [P4.BAR = 'Être trop fatigué ou fatiguée pour faire de l´activité physique'];
IF (P4.P7BARCHOICE EQ 102) [P4.BAR = 'Avoir beaucoup de choses à faire mis à part l´activité physique'];
IF (P4.P7BARCHOICE EQ 103) [P4.BAR = 'température sera trop mauvaise pour faire de l´activité physique'];
IF (P4.P7BARCHOICE EQ 104) [P4.BAR = 'Ne pas avoir personne avec qui faire de l´activité physique'];
IF (P4.P7BARCHOICE EQ 105) [P4.BAR = 'Ne pas avoir accès à des emplacements pour faire de l´activité physique'];
IF (P4.P7BARCHOICE EQ 106) [P4.BAR = 'Ne pas avoir d´argent pour faire de l´activité physique'];
IF (P4.P7BARCHOICE EQ 107) [P4.BAR = 'Être trop gêné ou gênée, manquer de confiance pour aller faire de l´activité physique'];
IF (P4.P7BARCHOICE EQ 108) [P4.BAR = 'Avoir peur d´être insuffisamment en santé pour faire de l´activité physique'];
IF (P4.P7BARCHOICE EQ 109) [P4.BAR = 'Manquer de motivation'];
IF ((P4.P7BARCHOICE EQ 110) AND (P4.Q_BARANSWER NE 0)) [P4.BAR = P4.Q_BARANSWER];
IF (P4.SUMSOL EQ 101) [P4.SOL = 'Je vais faire de l´activité tôt dans la journée, en matinée ou à l´heure du midi.'];
IF (P4.SUMSOL EQ 102) [P4.SOL = 'J´essaie de garder en tête que si je fais de l´activité physique, j´aurai de plus en plus d´énergie dans la vie.'];
IF (P4.SUMSOL EQ 103) [P4.SOL = 'Je vais planifier des activités avec d´autres personnes qui me motiveront.'];
IF (P4.SUMSOL EQ 104) [P4.SOL = 'Je prends une collation pour me donner de l´énergie et j´y vais quand même.'];
IF (P4.SUMSOL EQ 201) [P4.SOL = 'Faire de l´activité physique par bloc de 10 minutes les journées où je n´ai pas de temps.'];
IF (P4.SUMSOL EQ 202) [P4.SOL = 'Je vais remplacer des activités où je suis assis ou assise, télé, ordinateur ou autres, par des activités physiques.'];
IF (P4.SUMSOL EQ 203) [P4.SOL = 'Je vais me procurer un agenda et inscrire dans mon horaire les moments où je vais faire de l´activité physique.'];
IF (P4.SUMSOL EQ 204) [P4.SOL = 'Je vais prendre des pauses de travail active et faire des 10 minutes de marche.'];
IF (P4.SUMSOL EQ 205) [P4.SOL = 'Je vais me fixer des moments avec d´autres personnes qui me plaisent pour aller faire de l´activité physique.'];
IF (P4.SUMSOL EQ 301) [P4.SOL = 'Je vais me procurer l´équipement nécessaire pour en faire même quand il pleut, quand il neige ou quand il fait trop chaud.'];
IF (P4.SUMSOL EQ 302) [P4.SOL = 'Je vais faire de l’activité physique chez nous ou à l´intérieur dans un endroit que j´aurai choisi quand il fait moins beau.'];

IF (P4.SUMSOL EQ 303) [P4.SOL = 'Je vais me préparer une activité physique alternative au cas où il serait désagréable d´aller dehors.'];
IF (P4.SUMSOL EQ 304) [P4.SOL = 'Je vais me dresser une liste des activités que je serais prêtE à faire si la température est mauvaise.'];
IF (P4.SUMSOL EQ 401) [P4.SOL = 'Je vais me joindre à un groupe qui pratique l´activité physique que j´aime. Ex : la marche, l´aquaforme ou la natation.'];
IF (P4.SUMSOL EQ 402) [P4.SOL = 'Je vais inviter mes amis, ma famille ou mes collègues à faire de l´activité physique avec moi.'];
IF (P4.SUMSOL EQ 403) [P4.SOL = 'Je vais amener mon chien marcher avec moi.'];
IF (P4.SUMSOL EQ 404) [P4.SOL = 'Je vais participer dans des discussions en ligne sur des pages facebook : Diabète Québec et autres.'];
IF (P4.SUMSOL EQ 501) [P4.SOL = 'Je vais faire de la marche ou du vélo dehors, c´est simple et gratuit.'];
IF (P4.SUMSOL EQ 502) [P4.SOL = 'Je vais faire de l´activité physique dans ma maison comme du yoga, un programme sur DVD, vélo stationnaire, tapis roulant, etc.'];
IF (P4.SUMSOL EQ 503) [P4.SOL = 'Je vais aller voir au centre communautaire de mon quartier pour me donner des options.'];
IF (P4.SUMSOL EQ 504) [P4.SOL = 'Je vais discuter avec mon médecin, avec un ami ou un spécialiste de l´activité physique pour avoir des conseils.'];
IF (P4.SUMSOL EQ 601) [P4.SOL = 'Je vais trouver des activités physiques abordables et simples : marcher dehors, faire du vélo, nager à la piscine communautaire.'];
IF (P4.SUMSOL EQ 602) [P4.SOL = 'Je vais aller voir au centre communautaire de mon quartier pour me donner des options.'];
IF (P4.SUMSOL EQ 603) [P4.SOL = 'Je vais économiser pour m´acheter un DVD d´activité physique ou pour une autre activité physique que j´aimerais faire.'];
IF (P4.SUMSOL EQ 604) [P4.SOL = 'Je vais aller marcher avec ma famille, des amis ou des collègues.'];
IF (P4.SUMSOL EQ 701) [P4.SOL = 'Je vais choisir une activité physique simple comme la marche ou la bicyclette.'];
IF (P4.SUMSOL EQ 702) [P4.SOL = 'Je vais aller faire de l´activité physique avec des gens qui m´acceptent comme je suis, qui évitent de me juger.'];
IF (P4.SUMSOL EQ 703) [P4.SOL = 'Au départ, je vais faire de l´activité physique dans des endroits où je suis seul[LETTRE_E] pour prendre confiance.'];
IF (P4.SUMSOL EQ 704) [P4.SOL = 'Je vais éviter des endroits qui me gêne davantage comme les centres de conditionnement.'];
IF (P4.SUMSOL EQ 801) [P4.SOL = 'Je vais consulter mon médecin pour mettre au clair si je peux faire de l´activité physique.'];
IF (P4.SUMSOL EQ 802) [P4.SOL = 'Je vais consulter un spécialiste de l´activité physique pour qu´il me rassure et me conseille sur ce qui est sécuritaire pour moi.'];
IF (P4.SUMSOL EQ 803) [P4.SOL = 'Je vais commencer à petite dose. 10 à 20 minutes maximum d´activités physiques les jours où je suis actif, active.'];
IF (P4.SUMSOL EQ 804) [P4.SOL = 'Je vais faire de l´activité physique accompagnéE d´un spécialiste de l´activité physique.'];

IF (P4.SUMSOL EQ 805) [P4.SOL = 'Je vais faire de l´activité physique dans un groupe où un spécialiste de l´activité physique est présent.'];
IF (P4.SUMSOL EQ 901) [P4.SOL = 'Je vais signer un contrat papier personnel comme quoi je m´engage à être actif, active.'];
IF (P4.SUMSOL EQ 902) [P4.SOL = 'Je vais signer un contrat papier avec mes proches comme quoi je m´engage à être actif, active.'];
IF (P4.SUMSOL EQ 903) [P4.SOL = 'Je vais essayer de nouvelles activités physiques pour trouver celles qui me plaisent vraiment.'];
IF (P4.SUMSOL EQ 904) [P4.SOL = 'À chaque activité physique que je fais, je prends un temps pour me rappeler ce que cela va m´apporter de mieux dans ma vie.'];
IF (P4.SUMSOL EQ 905) [P4.SOL = 'Je vais faire de l´activité physique avec des gens que j´aime pour me motiver.'];
IF ((P4.SUMSOL EQ 999) AND (P4.P8QSOLOPEN NE 0)) [P4.SOL = P4.P8QSOLOPEN];
IF (P4.SUMSOL EQ 0) [P4.SOL = P4.P8QSOL_10];
PLAN DE LA semaine 5
formules p5
IF ((P5.QACTI12 EQ 112) AND (P5.QAOPEN NE 0)) [P5.QACTIVITE_12 = 112];
[P5.2SUMAP2 = P5.QACTIVITE1];
[P5.3SUMAP2 = P5.QACTIVITE1+P5.QACTIVITE2];
[P5.4SUMAP2 = P5.QACTIVITE1+P5.QACTIVITE2+P5.QACTIVITE3];
[P5.5SUMAP2 = P5.QACTIVITE1+P5.QACTIVITE2+P5.QACTIVITE3+P5.QACTIVITE4];
[P5.6SUMAP2 = P5.QACTIVITE1+P5.QACTIVITE2+P5.QACTIVITE3+P5.QACTIVITE4+P5.QACTIVITE5];
[P5.7SUMAP2 =
P5.QACTIVITE1+P5.QACTIVITE2+P5.QACTIVITE3+P5.QACTIVITE4+P5.QACTIVITE5+P5.QACTIVITE6];
[P5.8SUMAP2 =
P5.QACTIVITE1+P5.QACTIVITE2+P5.QACTIVITE3+P5.QACTIVITE4+P5.QACTIVITE5+P5.QACTIVITE6+P5.QACTIVITE7];
[P5.9SUMAP2 =
P5.QACTIVITE1+P5.QACTIVITE2+P5.QACTIVITE3+P5.QACTIVITE4+P5.QACTIVITE5+P5.QACTIVITE6+P5.QACTIVITE7+P5.QACTIVITE8];
[P5.10SUMAP2 =
P5.QACTIVITE1+P5.QACTIVITE2+P5.QACTIVITE3+P5.QACTIVITE4+P5.QACTIVITE5+P5.QACTIVITE6+P5.QACTIVITE7+P5.QACTIVITE8+P5.QACTIVITE9];
[P5.11SUMAP2 =
P5.QACTIVITE1+P5.QACTIVITE2+P5.QACTIVITE3+P5.QACTIVITE4+P5.QACTIVITE5+P5.QACTIVITE6+P5.QACTIVITE7+P5.QACTIVITE8+P5.QACTIVITE9+P5.QACTIVITE_10];
[P5.12SUMAP2 =
P5.QACTIVITE1+P5.QACTIVITE2+P5.QACTIVITE3+P5.QACTIVITE4+P5.QACTIVITE5+P5.QACTIVITE6+P5.QACTIVITE7+P5.QACTIVITE8+P5.QACTIVITE9+P5.QACTIVITE_10+P5.QACTIVITE_11];
[P5.3SUMAP3 = P5.QACTIVITE1+P5.QACTIVITE2];
[P5.4SUMAP3 = P5.QACTIVITE1+P5.QACTIVITE2+P5.QACTIVITE3];
[P5.5SUMAP3 = P5.QACTIVITE1+P5.QACTIVITE2+P5.QACTIVITE3+P5.QACTIVITE4];

[P5.6SUMAP3 = P5.QACTIVITE1+P5.QACTIVITE2+P5.QACTIVITE3+P5.QACTIVITE4+P5.QACTIVITE5];
[P5.7SUMAP3 =
P5.QACTIVITE1+P5.QACTIVITE2+P5.QACTIVITE3+P5.QACTIVITE4+P5.QACTIVITE5+P5.QACTIVITE6];
[P5.8SUMAP3 =
P5.QACTIVITE1+P5.QACTIVITE2+P5.QACTIVITE3+P5.QACTIVITE4+P5.QACTIVITE5+P5.QACTIVITE6+P5.QACTIVITE7];
[P5.9SUMAP3 =
P5.QACTIVITE1+P5.QACTIVITE2+P5.QACTIVITE3+P5.QACTIVITE4+P5.QACTIVITE5+P5.QACTIVITE6+P5.QACTIVITE7+P5.QACTIVITE8];
[P5.10SUMAP3 =
P5.QACTIVITE1+P5.QACTIVITE2+P5.QACTIVITE3+P5.QACTIVITE4+P5.QACTIVITE5+P5.QACTIVITE6+P5.QACTIVITE7+P5.QACTIVITE8+P5.QACTIVITE9];
[P5.11SUMAP3 =
P5.QACTIVITE1+P5.QACTIVITE2+P5.QACTIVITE3+P5.QACTIVITE4+P5.QACTIVITE5+P5.QACTIVITE6+P5.QACTIVITE7+P5.QACTIVITE8+P5.QACTIVITE9+P5.QACTIVITE_10];
[P5.12SUMAP3 =
P5.QACTIVITE1+P5.QACTIVITE2+P5.QACTIVITE3+P5.QACTIVITE4+P5.QACTIVITE5+P5.QACTIVITE6+P5.QACTIVITE7+P5.QACTIVITE8+P5.QACTIVITE9+P5.QACTIVITE_10+P5.QACTIVITE_11];
[P5.SUMAP =
P5.QACTIVITE1+P5.QACTIVITE2+P5.QACTIVITE3+P5.QACTIVITE4+P5.QACTIVITE5+P5.QACTIVITE6+P5.QACTIVITE7+P5.QACTIVITE8+P5.QACTIVITE9+P5.QACTIVITE_10+P5.QACTIVITE_11+P5.QACTIVITE_12];
[P5.SUMSOL =
P5.P8QSOL1+P5.P8QSOL2+P5.P8QSOL3+P5.P8QSOL4+P5.P8QSOL5+P5.P8QSOL6+P5.P8QSOL7+P5.P8QSOL8+P5.P8QSOL9];
IF (P5.QACTIVITE1 EQ 101) [P5.AP1 = 'marche rapide'];
IF ((P5.QACTIVITE1 NE 101) AND (P5.QACTIVITE2 EQ 102)) [P5.AP1 = 'raquettes à neige'];
IF ((P5.QACTIVITE1 NE 101) AND (P5.QACTIVITE2 NE 102) AND (P5.QACTIVITE3 EQ 103)) [P5.AP1 = 'vélo'];
IF ((P5.QACTIVITE1 NE 101) AND (P5.QACTIVITE2 NE 102) AND (P5.QACTIVITE3 NE 103) AND (P5.QACTIVITE4 EQ 104)) [P5.AP1 = 'natation'];
IF ((P5.QACTIVITE1 NE 101) AND (P5.QACTIVITE2 NE 102) AND (P5.QACTIVITE3 NE 103) AND (P5.QACTIVITE4 NE 104) AND (P5.QACTIVITE5 EQ 105)) [P5.AP1 = 'ski de fond'];
IF ((P5.QACTIVITE1 NE 101) AND (P5.QACTIVITE2 NE 102) AND (P5.QACTIVITE3 NE 103) AND (P5.QACTIVITE4 NE 104) AND (P5.QACTIVITE5 NE 105) AND (P5.QACTIVITE6 EQ 106)) [P5.AP1 = 'tennis'];
IF ((P5.QACTIVITE1 NE 101) AND (P5.QACTIVITE2 NE 102) AND (P5.QACTIVITE3 NE 103) AND (P5.QACTIVITE4 NE 104) AND (P5.QACTIVITE5 NE 105) AND (P5.QACTIVITE6 NE 106) AND (P5.QACTIVITE7 EQ 107)) [P5.AP1 = 'course à pieds'];
IF ((P5.QACTIVITE1 NE 101) AND (P5.QACTIVITE2 NE 102) AND (P5.QACTIVITE3 NE 103) AND (P5.QACTIVITE4 NE 104) AND (P5.QACTIVITE5 NE 105) AND (P5.QACTIVITE6 NE 106) AND (P5.QACTIVITE7 NE 107) AND (P5.QACTIVITE8 EQ 108)) [P5.AP1 = 'hockey'];
IF ((P5.QACTIVITE1 NE 101) AND (P5.QACTIVITE2 NE 102) AND (P5.QACTIVITE3 NE 103) AND (P5.QACTIVITE4 NE 104) AND (P5.QACTIVITE5 NE 105) AND (P5.QACTIVITE6 NE 106) AND (P5.QACTIVITE7 NE 107) AND (P5.QACTIVITE8 NE 108) AND (P5.QACTIVITE9 EQ 109)) [P5.AP1 = 'basketball'];

IF ((P5.QACTIVITE1 NE 101) AND (P5.QACTIVITE2 NE 102) AND (P5.QACTIVITE3 NE 103) AND (P5.QACTIVITE4 NE 104) AND (P5.QACTIVITE5 NE 105) AND (P5.QACTIVITE6 NE 106) AND (P5.QACTIVITE7 NE 107) AND (P5.QACTIVITE8 NE 108) AND (P5.QACTIVITE9 NE 109) AND (P5.QACTIVITE_10 EQ 110)) [P5.AP1 = 'soccer'];
IF ((P5.QACTIVITE1 NE 101) AND (P5.QACTIVITE2 NE 102) AND (P5.QACTIVITE3 NE 103) AND (P5.QACTIVITE4 NE 104) AND (P5.QACTIVITE5 NE 105) AND (P5.QACTIVITE6 NE 106) AND (P5.QACTIVITE7 NE 107) AND (P5.QACTIVITE8 NE 108) AND (P5.QACTIVITE9 NE 109) AND (P5.QACTIVITE_10 NE 110) AND (P5.QACTIVITE_11 EQ 111)) [P5.AP1 = 'centre de conditionnement'];
IF ((P5.QACTIVITE1 NE 101) AND (P5.QACTIVITE2 NE 102) AND (P5.QACTIVITE3 NE 103) AND (P5.QACTIVITE4 NE 104) AND (P5.QACTIVITE5 NE 105) AND (P5.QACTIVITE6 NE 106) AND (P5.QACTIVITE7 NE 107) AND (P5.QACTIVITE8 NE 108) AND (P5.QACTIVITE9 NE 109) AND (P5.QACTIVITE_10 NE 110) AND (P5.QACTIVITE_11 NE 111) AND (P5.QACTIVITE_12 EQ 112)) [P5.AP1 = P5.QAOPEN];
IF ((P5.2SUMAP2 GE 100) AND (P5.2SUMAP2 LT 200) AND (P5.QACTIVITE2 EQ 102)) [P5.AP2 = 'raquettes à neige'];
IF ((P5.3SUMAP2 GE 100) AND (P5.3SUMAP2 LT 200) AND (P5.QACTIVITE3 EQ 103)) [P5.AP2 = 'vélo'];
IF ((P5.4SUMAP2 GE 100) AND (P5.4SUMAP2 LT 200) AND (P5.QACTIVITE4 EQ 104)) [P5.AP2 = 'natation'];
IF ((P5.5SUMAP2 GE 100) AND (P5.5SUMAP2 LT 200) AND (P5.QACTIVITE5 EQ 105)) [P5.AP2 = 'ski de fond'];
IF ((P5.6SUMAP2 GE 100) AND (P5.6SUMAP2 LT 200) AND (P5.QACTIVITE6 EQ 106)) [P5.AP2 = 'tennis'];
IF ((P5.7SUMAP2 GE 100) AND (P5.7SUMAP2 LT 200) AND (P5.QACTIVITE7 EQ 107)) [P5.AP2 = 'course à pieds'];
IF ((P5.8SUMAP2 GE 100) AND (P5.8SUMAP2 LT 200) AND (P5.QACTIVITE8 EQ 108)) [P5.AP2 = 'hockey'];
IF ((P5.9SUMAP2 GE 100) AND (P5.9SUMAP2 LT 200) AND (P5.QACTIVITE9 EQ 109)) [P5.AP2 = 'basketball'];
IF ((P5.10SUMAP2 GE 100) AND (P5.10SUMAP2 LT 200) AND (P5.QACTIVITE_10 EQ 110)) [P5.AP2 = 'soccer'];
IF ((P5.11SUMAP2 GE 100) AND (P5.11SUMAP2 LT 200) AND (P5.QACTIVITE_11 EQ 111)) [P5.AP2 = 'centre de conditionnement'];
IF ((P5.12SUMAP2 GE 100) AND (P5.12SUMAP2 LT 200) AND (P5.QACTIVITE_12 EQ 112)) [P5.AP2 = P5.QAOPEN];
IF ((P5.3SUMAP3 GE 200) AND (P5.3SUMAP3 LT 300) AND (P5.QACTIVITE3 EQ 103)) [P5.AP3 = 'vélo'];
IF ((P5.4SUMAP3 GE 200) AND (P5.4SUMAP3 LT 300) AND (P5.QACTIVITE4 EQ 104)) [P5.AP3 = 'natation'];
IF ((P5.5SUMAP3 GE 200) AND (P5.5SUMAP3 LT 300) AND (P5.QACTIVITE5 EQ 105)) [P5.AP3 = 'ski de fond'];
IF ((P5.6SUMAP3 GE 200) AND (P5.6SUMAP3 LT 300) AND (P5.QACTIVITE6 EQ 106)) [P5.AP3 = 'tennis'];
IF ((P5.7SUMAP3 GE 200) AND (P5.7SUMAP3 LT 300) AND (P5.QACTIVITE7 EQ 107)) [P5.AP3 = 'course à pieds'];
IF ((P5.8SUMAP3 GE 200) AND (P5.8SUMAP3 LT 300) AND (P5.QACTIVITE8 EQ 108)) [P5.AP3 = 'hockey'];
IF ((P5.9SUMAP3 GE 200) AND (P5.9SUMAP3 LT 300) AND (P5.QACTIVITE9 EQ 109)) [P5.AP3 = 'basketball'];
IF ((P5.10SUMAP3 GE 200) AND (P5.10SUMAP3 LT 300) AND (P5.QACTIVITE_10 EQ 110)) [P5.AP3 = 'soccer'];
IF ((P5.11SUMAP3 GE 200) AND (P5.11SUMAP3 LT 300) AND (P5.QACTIVITE_11 EQ 111)) [P5.AP3 = 'centre de conditionnement'];
IF ((P5.12SUMAP3 GE 200) AND (P5.12SUMAP3 LT 300) AND (P5.QACTIVITE_12 EQ 112)) [P5.AP3 = P5.QAOPEN];

ACTIVITÉ 1 DANS LE PLAN POUR CHAQUE JOUR DE LA SEMAINE
IF ((P5.QACTIVITE1 EQ 101) AND (P5.AP1JOUR EQ 1)) [P5.LUNDIAP1 = P5.AP1];
IF ((P5.QACTIVITE1 NE 101) AND (P5.QACTIVITE2 EQ 102) AND (P5.AP2JOUR EQ 1)) [P5.LUNDIAP1 = P5.AP1];
IF ((P5.QACTIVITE1 NE 101) AND (P5.QACTIVITE2 NE 102) AND (P5.QACTIVITE3 EQ 103) AND (P5.AP3JOUR EQ 1)) [P5.LUNDIAP1 = P5.AP1];
IF ((P5.QACTIVITE1 NE 101) AND (P5.QACTIVITE2 NE 102) AND (P5.QACTIVITE3 NE 103) AND (P5.QACTIVITE4 EQ 104) AND (P5.AP4JOUR EQ 1)) [P5.LUNDIAP1 = P5.AP1];
IF ((P5.QACTIVITE1 NE 101) AND (P5.QACTIVITE2 NE 102) AND (P5.QACTIVITE3 NE 103) AND (P5.QACTIVITE4 NE 104) AND (P5.QACTIVITE5 EQ 105) AND (P5.AP5JOUR EQ 1)) [P5.LUNDIAP1 = P5.AP1];
IF ((P5.QACTIVITE1 NE 101) AND (P5.QACTIVITE2 NE 102) AND (P5.QACTIVITE3 NE 103) AND (P5.QACTIVITE4 NE 104) AND (P5.QACTIVITE5 NE 105) AND (P5.QACTIVITE6 EQ 106) AND (P5.AP6JOUR EQ 1)) [P5.LUNDIAP1 = P5.AP1];
IF ((P5.QACTIVITE1 NE 101) AND (P5.QACTIVITE2 NE 102) AND (P5.QACTIVITE3 NE 103) AND (P5.QACTIVITE4 NE 104) AND (P5.QACTIVITE5 NE 105) AND (P5.QACTIVITE6 NE 106) AND (P5.QACTIVITE7 EQ 107) AND (P5.AP7JOUR EQ 1)) [P5.LUNDIAP1 = P5.AP1];
IF ((P5.QACTIVITE1 NE 101) AND (P5.QACTIVITE2 NE 102) AND (P5.QACTIVITE3 NE 103) AND (P5.QACTIVITE4 NE 104) AND (P5.QACTIVITE5 NE 105) AND (P5.QACTIVITE6 NE 106) AND (P5.QACTIVITE7 NE 107) AND (P5.QACTIVITE8 EQ 108) AND (P5.AP8JOUR EQ 1)) [P5.LUNDIAP1 = P5.AP1];
IF ((P5.QACTIVITE1 NE 101) AND (P5.QACTIVITE2 NE 102) AND (P5.QACTIVITE3 NE 103) AND (P5.QACTIVITE4 NE 104) AND (P5.QACTIVITE5 NE 105) AND (P5.QACTIVITE6 NE 106) AND (P5.QACTIVITE7 NE 107) AND (P5.QACTIVITE8 NE 108) AND (P5.QACTIVITE9 EQ 109) AND (P5.AP9JOUR EQ 1)) [P5.LUNDIAP1 = P5.AP1];
IF ((P5.QACTIVITE1 NE 101) AND (P5.QACTIVITE2 NE 102) AND (P5.QACTIVITE3 NE 103) AND (P5.QACTIVITE4 NE 104) AND (P5.QACTIVITE5 NE 105) AND (P5.QACTIVITE6 NE 106) AND (P5.QACTIVITE7 NE 107) AND (P5.QACTIVITE8 NE 108) AND (P5.QACTIVITE9 NE 109) AND (P5.QACTIVITE_10 EQ 110) AND (P5.AP10JOUR EQ 1)) [P5.LUNDIAP1 = P5.AP1];
IF ((P5.QACTIVITE1 NE 101) AND (P5.QACTIVITE2 NE 102) AND (P5.QACTIVITE3 NE 103) AND (P5.QACTIVITE4 NE 104) AND (P5.QACTIVITE5 NE 105) AND (P5.QACTIVITE6 NE 106) AND (P5.QACTIVITE7 NE 107) AND (P5.QACTIVITE8 NE 108) AND (P5.QACTIVITE9 NE 109) AND (P5.QACTIVITE_10 NE 110) AND (P5.QACTIVITE_11 EQ 111) AND (P5.AP11JOUR EQ 1)) [P5.LUNDIAP1 = P5.AP1];
IF ((P5.QACTIVITE1 NE 101) AND (P5.QACTIVITE2 NE 102) AND (P5.QACTIVITE3 NE 103) AND (P5.QACTIVITE4 NE 104) AND (P5.QACTIVITE5 NE 105) AND (P5.QACTIVITE6 NE 106) AND (P5.QACTIVITE7 NE 107) AND (P5.QACTIVITE8 NE 108) AND (P5.QACTIVITE9 NE 109) AND (P5.QACTIVITE_10 NE 110) AND (P5.QACTIVITE_11 NE 111) AND (P5.QACTIVITE_12 EQ 112) AND (P5.AP12JOUR EQ 1)) [P5.LUNDIAP1 = P5.AP1];
IF ((P5.QACTIVITE1 EQ 101) AND (P5.AP1JOUR EQ 2)) [P5.MARDIAP1 = P5.AP1];
IF ((P5.QACTIVITE1 NE 101) AND (P5.QACTIVITE2 EQ 102) AND (P5.AP2JOUR EQ 2)) [P5.MARDIAP1 = P5.AP1];
IF ((P5.QACTIVITE1 NE 101) AND (P5.QACTIVITE2 NE 102) AND (P5.QACTIVITE3 EQ 103) AND (P5.AP3JOUR EQ 2)) [P5.MARDIAP1 = P5.AP1];

IF ((P5.QACTIVITE1 NE 101) AND (P5.QACTIVITE2 NE 102) AND (P5.QACTIVITE3 NE 103) AND (P5.QACTIVITE4 EQ 104) AND (P5.AP4JOUR EQ 2)) [P5.MARDIAP1 = P5.AP1];
IF ((P5.QACTIVITE1 NE 101) AND (P5.QACTIVITE2 NE 102) AND (P5.QACTIVITE3 NE 103) AND (P5.QACTIVITE4 NE 104) AND (P5.QACTIVITE5 EQ 105) AND (P5.AP5JOUR EQ 2)) [P5.MARDIAP1 = P5.AP1];
IF ((P5.QACTIVITE1 NE 101) AND (P5.QACTIVITE2 NE 102) AND (P5.QACTIVITE3 NE 103) AND (P5.QACTIVITE4 NE 104) AND (P5.QACTIVITE5 NE 105) AND (P5.QACTIVITE6 EQ 106) AND (P5.AP6JOUR EQ 2)) [P5.MARDIAP1 = P5.AP1];
IF ((P5.QACTIVITE1 NE 101) AND (P5.QACTIVITE2 NE 102) AND (P5.QACTIVITE3 NE 103) AND (P5.QACTIVITE4 NE 104) AND (P5.QACTIVITE5 NE 105) AND (P5.QACTIVITE6 NE 106) AND (P5.QACTIVITE7 EQ 107) AND (P5.AP7JOUR EQ 2)) [P5.MARDIAP1 = P5.AP1];
IF ((P5.QACTIVITE1 NE 101) AND (P5.QACTIVITE2 NE 102) AND (P5.QACTIVITE3 NE 103) AND (P5.QACTIVITE4 NE 104) AND (P5.QACTIVITE5 NE 105) AND (P5.QACTIVITE6 NE 106) AND (P5.QACTIVITE7 NE 107) AND (P5.QACTIVITE8 EQ 108) AND (P5.AP8JOUR EQ 2)) [P5.MARDIAP1 = P5.AP1];
IF ((P5.QACTIVITE1 NE 101) AND (P5.QACTIVITE2 NE 102) AND (P5.QACTIVITE3 NE 103) AND (P5.QACTIVITE4 NE 104) AND (P5.QACTIVITE5 NE 105) AND (P5.QACTIVITE6 NE 106) AND (P5.QACTIVITE7 NE 107) AND (P5.QACTIVITE8 NE 108) AND (P5.QACTIVITE9 EQ 109) AND (P5.AP9JOUR EQ 2)) [P5.MARDIAP1 = P5.AP1];
IF ((P5.QACTIVITE1 NE 101) AND (P5.QACTIVITE2 NE 102) AND (P5.QACTIVITE3 NE 103) AND (P5.QACTIVITE4 NE 104) AND (P5.QACTIVITE5 NE 105) AND (P5.QACTIVITE6 NE 106) AND (P5.QACTIVITE7 NE 107) AND (P5.QACTIVITE8 NE 108) AND (P5.QACTIVITE9 NE 109) AND (P5.QACTIVITE_10 EQ 110) AND (P5.AP10JOUR EQ 2)) [P5.MARDIAP1 = P5.AP1];
IF ((P5.QACTIVITE1 NE 101) AND (P5.QACTIVITE2 NE 102) AND (P5.QACTIVITE3 NE 103) AND (P5.QACTIVITE4 NE 104) AND (P5.QACTIVITE5 NE 105) AND (P5.QACTIVITE6 NE 106) AND (P5.QACTIVITE7 NE 107) AND (P5.QACTIVITE8 NE 108) AND (P5.QACTIVITE9 NE 109) AND (P5.QACTIVITE_10 NE 110) AND (P5.QACTIVITE_11 EQ 111) AND (P5.AP11JOUR EQ 2)) [P5.MARDIAP1 = P5.AP1];
IF ((P5.QACTIVITE1 NE 101) AND (P5.QACTIVITE2 NE 102) AND (P5.QACTIVITE3 NE 103) AND (P5.QACTIVITE4 NE 104) AND (P5.QACTIVITE5 NE 105) AND (P5.QACTIVITE6 NE 106) AND (P5.QACTIVITE7 NE 107) AND (P5.QACTIVITE8 NE 108) AND (P5.QACTIVITE9 NE 109) AND (P5.QACTIVITE_10 NE 110) AND (P5.QACTIVITE_11 NE 111) AND (P5.QACTIVITE_12 EQ 112) AND (P5.AP12JOUR EQ 2)) [P5.MARDIAP1 = P5.AP1];
IF ((P5.QACTIVITE1 EQ 101) AND (P5.AP1JOUR EQ 3)) [P5.MERCREDIAP1 = P5.AP1];
IF ((P5.QACTIVITE1 NE 101) AND (P5.QACTIVITE2 EQ 102) AND (P5.AP2JOUR EQ 3)) [P5.MERCREDIAP1 = P5.AP1];
IF ((P5.QACTIVITE1 NE 101) AND (P5.QACTIVITE2 NE 102) AND (P5.QACTIVITE3 EQ 103) AND (P5.AP3JOUR EQ 3)) [P5.MERCREDIAP1 = P5.AP1];
IF ((P5.QACTIVITE1 NE 101) AND (P5.QACTIVITE2 NE 102) AND (P5.QACTIVITE3 NE 103) AND (P5.QACTIVITE4 EQ 104) AND (P5.AP4JOUR EQ 3)) [P5.MERCREDIAP1 = P5.AP1];
IF ((P5.QACTIVITE1 NE 101) AND (P5.QACTIVITE2 NE 102) AND (P5.QACTIVITE3 NE 103) AND (P5.QACTIVITE4 NE 104) AND (P5.QACTIVITE5 EQ 105) AND (P5.AP5JOUR EQ 3)) [P5.MERCREDIAP1 = P5.AP1];

IF ((P5.QACTIVITE1 NE 101) AND (P5.QACTIVITE2 NE 102) AND (P5.QACTIVITE3 NE 103) AND (P5.QACTIVITE4 NE 104) AND (P5.QACTIVITES5 NE 105) AND (P5.QACTIVITE6 EQ 106) AND (P5.AP6JOUR EQ 3)) [P5.MERCREDIAP1 = P5.AP1];
IF ((P5.QACTIVITE1 NE 101) AND (P5.QACTIVITE2 NE 102) AND (P5.QACTIVITE3 NE 103) AND (P5.QACTIVITE4 NE 104) AND (P5.QACTIVITES5 NE 105) AND (P5.QACTIVITE6 NE 106) AND (P5.QACTIVITE7 EQ 107) AND (P5.AP7JOUR EQ 3)) [P5.MERCREDIAP1 = P5.AP1];
IF ((P5.QACTIVITE1 NE 101) AND (P5.QACTIVITE2 NE 102) AND (P5.QACTIVITE3 NE 103) AND (P5.QACTIVITE4 NE 104) AND (P5.QACTIVITES5 NE 105) AND (P5.QACTIVITE6 NE 106) AND (P5.QACTIVITE7 NE 107) AND (P5.QACTIVITE8 EQ 108) AND (P5.AP8JOUR EQ 3)) [P5.MERCREDIAP1 = P5.AP1];
IF ((P5.QACTIVITE1 NE 101) AND (P5.QACTIVITE2 NE 102) AND (P5.QACTIVITE3 NE 103) AND (P5.QACTIVITE4 NE 104) AND (P5.QACTIVITES5 NE 105) AND (P5.QACTIVITE6 NE 106) AND (P5.QACTIVITE7 NE 107) AND (P5.QACTIVITE8 NE 108) AND (P5.QACTIVITE9 EQ 109) AND (P5.AP9JOUR EQ 3)) [P5.MERCREDIAP1 = P5.AP1];
IF ((P5.QACTIVITE1 NE 101) AND (P5.QACTIVITE2 NE 102) AND (P5.QACTIVITE3 NE 103) AND (P5.QACTIVITE4 NE 104) AND (P5.QACTIVITES5 NE 105) AND (P5.QACTIVITE6 NE 106) AND (P5.QACTIVITE7 NE 107) AND (P5.QACTIVITE8 NE 108) AND (P5.QACTIVITE9 NE 109) AND (P5.QACTIVITE_10 EQ 110) AND (P5.AP10JOUR EQ 3)) [P5.MERCREDIAP1 = P5.AP1];
IF ((P5.QACTIVITE1 NE 101) AND (P5.QACTIVITE2 NE 102) AND (P5.QACTIVITE3 NE 103) AND (P5.QACTIVITE4 NE 104) AND (P5.QACTIVITES5 NE 105) AND (P5.QACTIVITE6 NE 106) AND (P5.QACTIVITE7 NE 107) AND (P5.QACTIVITE8 NE 108) AND (P5.QACTIVITE9 NE 109) AND (P5.QACTIVITE_10 NE 110) AND (P5.QACTIVITE_11 EQ 111) AND (P5.AP11JOUR EQ 3)) [P5.MERCREDIAP1 = P5.AP1];
IF ((P5.QACTIVITE1 NE 101) AND (P5.QACTIVITE2 NE 102) AND (P5.QACTIVITE3 NE 103) AND (P5.QACTIVITE4 NE 104) AND (P5.QACTIVITES5 NE 105) AND (P5.QACTIVITE6 NE 106) AND (P5.QACTIVITE7 NE 107) AND (P5.QACTIVITE8 NE 108) AND (P5.QACTIVITE9 NE 109) AND (P5.QACTIVITE_10 NE 110) AND (P5.QACTIVITE_11 NE 111) AND (P5.QACTIVITE_12 EQ 112) AND (P5.AP12JOUR EQ 3)) [P5.MERCREDIAP1 = P5.AP1];
IF ((P5.QACTIVITE1 EQ 101) AND (P5.AP1JOUR EQ 4)) [P5.JEUDIAP1 = P5.AP1];
IF ((P5.QACTIVITE1 NE 101) AND (P5.QACTIVITE2 EQ 102) AND (P5.AP2JOUR EQ 4)) [P5.JEUDIAP1 = P5.AP1];
IF ((P5.QACTIVITE1 NE 101) AND (P5.QACTIVITE2 NE 102) AND (P5.QACTIVITE3 EQ 103) AND (P5.AP3JOUR EQ 4)) [P5.JEUDIAP1 = P5.AP1];
IF ((P5.QACTIVITE1 NE 101) AND (P5.QACTIVITE2 NE 102) AND (P5.QACTIVITE3 NE 103) AND (P5.QACTIVITE4 EQ 104) AND (P5.AP4JOUR EQ 4)) [P5.JEUDIAP1 = P5.AP1];
IF ((P5.QACTIVITE1 NE 101) AND (P5.QACTIVITE2 NE 102) AND (P5.QACTIVITE3 NE 103) AND (P5.QACTIVITE4 NE 104) AND (P5.QACTIVITES5 EQ 105) AND (P5.AP5JOUR EQ 4)) [P5.JEUDIAP1 = P5.AP1];
IF ((P5.QACTIVITE1 NE 101) AND (P5.QACTIVITE2 NE 102) AND (P5.QACTIVITE3 NE 103) AND (P5.QACTIVITE4 NE 104) AND (P5.QACTIVITES5 NE 105) AND (P5.QACTIVITE6 EQ 106) AND (P5.AP6JOUR EQ 4)) [P5.JEUDIAP1 = P5.AP1];
IF ((P5.QACTIVITE1 NE 101) AND (P5.QACTIVITE2 NE 102) AND (P5.QACTIVITE3 NE 103) AND (P5.QACTIVITE4 NE 104) AND (P5.QACTIVITES5 NE 105) AND (P5.QACTIVITE6 NE 106) AND (P5.QACTIVITE7 EQ 107) AND (P5.AP7JOUR EQ 4)) [P5.JEUDIAP1 = P5.AP1];

IF ((P5.QACTIVITE1 NE 101) AND (P5.QACTIVITE2 NE 102) AND (P5.QACTIVITE3 NE 103) AND (P5.QACTIVITE4 NE 104) AND (P5.QACTIVITES5 NE 105) AND (P5.QACTIVITE6 NE 106) AND (P5.QACTIVITE7 NE 107) AND (P5.QACTIVITE8 EQ 108) AND (P5.AP8JOUR EQ 4)) [P5.JEUDIAP1 = P5.AP1];
IF ((P5.QACTIVITE1 NE 101) AND (P5.QACTIVITE2 NE 102) AND (P5.QACTIVITE3 NE 103) AND (P5.QACTIVITE4 NE 104) AND (P5.QACTIVITES5 NE 105) AND (P5.QACTIVITE6 NE 106) AND (P5.QACTIVITE7 NE 107) AND (P5.QACTIVITE8 NE 108) AND (P5.QACTIVITE9 EQ 109) AND (P5.AP9JOUR EQ 4)) [P5.JEUDIAP1 = P5.AP1];
IF ((P5.QACTIVITE1 NE 101) AND (P5.QACTIVITE2 NE 102) AND (P5.QACTIVITE3 NE 103) AND (P5.QACTIVITE4 NE 104) AND (P5.QACTIVITES5 NE 105) AND (P5.QACTIVITE6 NE 106) AND (P5.QACTIVITE7 NE 107) AND (P5.QACTIVITE8 NE 108) AND (P5.QACTIVITE9 NE 109) AND (P5.QACTIVITE_10 EQ 110) AND (P5.AP10JOUR EQ 4)) [P5.JEUDIAP1 = P5.AP1];
IF ((P5.QACTIVITE1 NE 101) AND (P5.QACTIVITE2 NE 102) AND (P5.QACTIVITE3 NE 103) AND (P5.QACTIVITE4 NE 104) AND (P5.QACTIVITES5 NE 105) AND (P5.QACTIVITE6 NE 106) AND (P5.QACTIVITE7 NE 107) AND (P5.QACTIVITE8 NE 108) AND (P5.QACTIVITE9 NE 109) AND (P5.QACTIVITE_10 NE 110) AND (P5.QACTIVITE_11 EQ 111) AND (P5.AP11JOUR EQ 4)) [P5.JEUDIAP1 = P5.AP1];
IF ((P5.QACTIVITE1 NE 101) AND (P5.QACTIVITE2 NE 102) AND (P5.QACTIVITE3 NE 103) AND (P5.QACTIVITE4 NE 104) AND (P5.QACTIVITES5 NE 105) AND (P5.QACTIVITE6 NE 106) AND (P5.QACTIVITE7 NE 107) AND (P5.QACTIVITE8 NE 108) AND (P5.QACTIVITE9 NE 109) AND (P5.QACTIVITE_10 NE 110) AND (P5.QACTIVITE_11 NE 111) AND (P5.QACTIVITE_12 EQ 112) AND (P5.AP12JOUR EQ 4)) [P5.JEUDIAP1 = P5.AP1];
IF ((P5.QACTIVITE1 EQ 101) AND (P5.AP1JOUR EQ 5)) [P5.VENDREDIAP1 = P5.AP1];
IF ((P5.QACTIVITE1 NE 101) AND (P5.QACTIVITE2 EQ 102) AND (P5.AP2JOUR EQ 5)) [P5.VENDREDIAP1 = P5.AP1];
IF ((P5.QACTIVITE1 NE 101) AND (P5.QACTIVITE2 NE 102) AND (P5.QACTIVITE3 EQ 103) AND (P5.AP3JOUR EQ 5)) [P5.VENDREDIAP1 = P5.AP1];
IF ((P5.QACTIVITE1 NE 101) AND (P5.QACTIVITE2 NE 102) AND (P5.QACTIVITE3 NE 103) AND (P5.QACTIVITE4 EQ 104) AND (P5.AP4JOUR EQ 5)) [P5.VENDREDIAP1 = P5.AP1];
IF ((P5.QACTIVITE1 NE 101) AND (P5.QACTIVITE2 NE 102) AND (P5.QACTIVITE3 NE 103) AND (P5.QACTIVITE4 NE 104) AND (P5.QACTIVITES5 EQ 105) AND (P5.AP5JOUR EQ 5)) [P5.VENDREDIAP1 = P5.AP1];
IF ((P5.QACTIVITE1 NE 101) AND (P5.QACTIVITE2 NE 102) AND (P5.QACTIVITE3 NE 103) AND (P5.QACTIVITE4 NE 104) AND (P5.QACTIVITES5 NE 105) AND (P5.QACTIVITE6 EQ 106) AND (P5.AP6JOUR EQ 5)) [P5.VENDREDIAP1 = P5.AP1];
IF ((P5.QACTIVITE1 NE 101) AND (P5.QACTIVITE2 NE 102) AND (P5.QACTIVITE3 NE 103) AND (P5.QACTIVITE4 NE 104) AND (P5.QACTIVITES5 NE 105) AND (P5.QACTIVITE6 NE 106) AND (P5.QACTIVITE7 EQ 107) AND (P5.AP7JOUR EQ 5)) [P5.VENDREDIAP1 = P5.AP1];
IF ((P5.QACTIVITE1 NE 101) AND (P5.QACTIVITE2 NE 102) AND (P5.QACTIVITE3 NE 103) AND (P5.QACTIVITE4 NE 104) AND (P5.QACTIVITES5 NE 105) AND (P5.QACTIVITE6 NE 106) AND (P5.QACTIVITE7 NE 107) AND (P5.QACTIVITE8 EQ 108) AND (P5.AP8JOUR EQ 5)) [P5.VENDREDIAP1 = P5.AP1];
IF ((P5.QACTIVITE1 NE 101) AND (P5.QACTIVITE2 NE 102) AND (P5.QACTIVITE3 NE 103) AND (P5.QACTIVITE4 NE 104) AND (P5.QACTIVITES5 NE 105) AND (P5.QACTIVITE6 NE 106) AND (P5.QACTIVITE7

NE 107) AND (P5.QACTIVITE8 NE 108) AND (P5.QACTIVITE9 EQ 109) AND (P5.AP9JOUR EQ 5))
[P5.VENDREDIAP1 = P5.AP1];
IF ((P5.QACTIVITE1 NE 101) AND (P5.QACTIVITE2 NE 102) AND (P5.QACTIVITE3 NE 103) AND
(P5.QACTIVITE4 NE 104) AND (P5.QACTIVITE5 NE 105) AND (P5.QACTIVITE6 NE 106) AND (P5.QACTIVITE7
NE 107) AND (P5.QACTIVITE8 NE 108) AND (P5.QACTIVITE9 NE 109) AND (P5.QACTIVITE_10 EQ 110) AND
(P5.AP10JOUR EQ 5)) [P5.VENDREDIAP1 = P5.AP1];
IF ((P5.QACTIVITE1 NE 101) AND (P5.QACTIVITE2 NE 102) AND (P5.QACTIVITE3 NE 103) AND
(P5.QACTIVITE4 NE 104) AND (P5.QACTIVITE5 NE 105) AND (P5.QACTIVITE6 NE 106) AND (P5.QACTIVITE7
NE 107) AND (P5.QACTIVITE8 NE 108) AND (P5.QACTIVITE9 NE 109) AND (P5.QACTIVITE_10 NE 110) AND
(P5.QACTIVITE_11 EQ 111) AND (P5.AP11JOUR EQ 5)) [P5.VENDREDIAP1 = P5.AP1];
IF ((P5.QACTIVITE1 NE 101) AND (P5.QACTIVITE2 NE 102) AND (P5.QACTIVITE3 NE 103) AND
(P5.QACTIVITE4 NE 104) AND (P5.QACTIVITE5 NE 105) AND (P5.QACTIVITE6 NE 106) AND (P5.QACTIVITE7
NE 107) AND (P5.QACTIVITE8 NE 108) AND (P5.QACTIVITE9 NE 109) AND (P5.QACTIVITE_10 NE 110) AND
(P5.QACTIVITE_11 NE 111) AND (P5.QACTIVITE_12 EQ 112) AND (P5.AP12JOUR EQ 5)) [P5.VENDREDIAP1 =
P5.AP1];
IF ((P5.QACTIVITE1 EQ 101) AND (P5.AP1JOUR EQ 6)) [P5.SAMEDIAP1 = P5.AP1];
IF ((P5.QACTIVITE1 NE 101) AND (P5.QACTIVITE2 EQ 102) AND (P5.AP2JOUR EQ 6)) [P5.SAMEDIAP1 =
P5.AP1];
IF ((P5.QACTIVITE1 NE 101) AND (P5.QACTIVITE2 NE 102) AND (P5.QACTIVITE3 EQ 103) AND (P5.AP3JOUR
EQ 6)) [P5.SAMEDIAP1 = P5.AP1];
IF ((P5.QACTIVITE1 NE 101) AND (P5.QACTIVITE2 NE 102) AND (P5.QACTIVITE3 NE 103) AND
(P5.QACTIVITE4 EQ 104) AND (P5.AP4JOUR EQ 6)) [P5.SAMEDIAP1 = P5.AP1];
IF ((P5.QACTIVITE1 NE 101) AND (P5.QACTIVITE2 NE 102) AND (P5.QACTIVITE3 NE 103) AND
(P5.QACTIVITE4 NE 104) AND (P5.QACTIVITE5 EQ 105) AND (P5.AP5JOUR EQ 6)) [P5.SAMEDIAP1 = P5.AP1];
IF ((P5.QACTIVITE1 NE 101) AND (P5.QACTIVITE2 NE 102) AND (P5.QACTIVITE3 NE 103) AND
(P5.QACTIVITE4 NE 104) AND (P5.QACTIVITE5 NE 105) AND (P5.QACTIVITE6 EQ 106) AND (P5.AP6JOUR EQ
6)) [P5.SAMEDIAP1 = P5.AP1];
IF ((P5.QACTIVITE1 NE 101) AND (P5.QACTIVITE2 NE 102) AND (P5.QACTIVITE3 NE 103) AND
(P5.QACTIVITE4 NE 104) AND (P5.QACTIVITE5 NE 105) AND (P5.QACTIVITE6 NE 106) AND (P5.QACTIVITE7
EQ 107) AND (P5.AP7JOUR EQ 6)) [P5.SAMEDIAP1 = P5.AP1];
IF ((P5.QACTIVITE1 NE 101) AND (P5.QACTIVITE2 NE 102) AND (P5.QACTIVITE3 NE 103) AND
(P5.QACTIVITE4 NE 104) AND (P5.QACTIVITE5 NE 105) AND (P5.QACTIVITE6 NE 106) AND (P5.QACTIVITE7
NE 107) AND (P5.QACTIVITE8 EQ 108) AND (P5.AP8JOUR EQ 6)) [P5.SAMEDIAP1 = P5.AP1];
IF ((P5.QACTIVITE1 NE 101) AND (P5.QACTIVITE2 NE 102) AND (P5.QACTIVITE3 NE 103) AND
(P5.QACTIVITE4 NE 104) AND (P5.QACTIVITE5 NE 105) AND (P5.QACTIVITE6 NE 106) AND (P5.QACTIVITE7
NE 107) AND (P5.QACTIVITE8 NE 108) AND (P5.QACTIVITE9 EQ 109) AND (P5.AP9JOUR EQ 6))
[P5.SAMEDIAP1 = P5.AP1];
IF ((P5.QACTIVITE1 NE 101) AND (P5.QACTIVITE2 NE 102) AND (P5.QACTIVITE3 NE 103) AND
(P5.QACTIVITE4 NE 104) AND (P5.QACTIVITE5 NE 105) AND (P5.QACTIVITE6 NE 106) AND (P5.QACTIVITE7
NE 107) AND (P5.QACTIVITE8 NE 108) AND (P5.QACTIVITE9 NE 109) AND (P5.QACTIVITE_10 EQ 110) AND
(P5.AP10JOUR EQ 6)) [P5.SAMEDIAP1 = P5.AP1];

IF ((P5.QACTIVITE1 NE 101) AND (P5.QACTIVITE2 NE 102) AND (P5.QACTIVITE3 NE 103) AND (P5.QACTIVITE4 NE 104) AND (P5.QACTIVITES5 NE 105) AND (P5.QACTIVITE6 NE 106) AND (P5.QACTIVITE7 NE 107) AND (P5.QACTIVITE8 NE 108) AND (P5.QACTIVITE9 NE 109) AND (P5.QACTIVITE_10 NE 110) AND (P5.QACTIVITE_11 EQ 111) AND (P5.AP11JOUR EQ 6)) [P5.SAMEDIAP1 = P5.AP1];
IF ((P5.QACTIVITE1 NE 101) AND (P5.QACTIVITE2 NE 102) AND (P5.QACTIVITE3 NE 103) AND (P5.QACTIVITE4 NE 104) AND (P5.QACTIVITES5 NE 105) AND (P5.QACTIVITE6 NE 106) AND (P5.QACTIVITE7 NE 107) AND (P5.QACTIVITE8 NE 108) AND (P5.QACTIVITE9 NE 109) AND (P5.QACTIVITE_10 NE 110) AND (P5.QACTIVITE_11 NE 111) AND (P5.QACTIVITE_12 EQ 112) AND (P5.AP12JOUR EQ 6)) [P5.SAMEDIAP1 = P5.AP1];
IF ((P5.QACTIVITE1 EQ 101) AND (P5.AP1JOUR EQ 7)) [P5.DIMANCHEAP1 = P5.AP1];
IF ((P5.QACTIVITE1 NE 101) AND (P5.QACTIVITE2 EQ 102) AND (P5.AP2JOUR EQ 7)) [P5.DIMANCHEAP1 = P5.AP1];
IF ((P5.QACTIVITE1 NE 101) AND (P5.QACTIVITE2 NE 102) AND (P5.QACTIVITE3 EQ 103) AND (P5.AP3JOUR EQ 7)) [P5.DIMANCHEAP1 = P5.AP1];
IF ((P5.QACTIVITE1 NE 101) AND (P5.QACTIVITE2 NE 102) AND (P5.QACTIVITE3 NE 103) AND (P5.QACTIVITE4 EQ 104) AND (P5.AP4JOUR EQ 7)) [P5.DIMANCHEAP1 = P5.AP1];
IF ((P5.QACTIVITE1 NE 101) AND (P5.QACTIVITE2 NE 102) AND (P5.QACTIVITE3 NE 103) AND (P5.QACTIVITE4 NE 104) AND (P5.QACTIVITES5 EQ 105) AND (P5.AP5JOUR EQ 7)) [P5.DIMANCHEAP1 = P5.AP1];
IF ((P5.QACTIVITE1 NE 101) AND (P5.QACTIVITE2 NE 102) AND (P5.QACTIVITE3 NE 103) AND (P5.QACTIVITE4 NE 104) AND (P5.QACTIVITES5 NE 105) AND (P5.QACTIVITE6 EQ 106) AND (P5.AP6JOUR EQ 7)) [P5.DIMANCHEAP1 = P5.AP1];
IF ((P5.QACTIVITE1 NE 101) AND (P5.QACTIVITE2 NE 102) AND (P5.QACTIVITE3 NE 103) AND (P5.QACTIVITE4 NE 104) AND (P5.QACTIVITES5 NE 105) AND (P5.QACTIVITE6 NE 106) AND (P5.QACTIVITE7 EQ 107) AND (P5.AP7JOUR EQ 7)) [P5.DIMANCHEAP1 = P5.AP1];
IF ((P5.QACTIVITE1 NE 101) AND (P5.QACTIVITE2 NE 102) AND (P5.QACTIVITE3 NE 103) AND (P5.QACTIVITE4 NE 104) AND (P5.QACTIVITES5 NE 105) AND (P5.QACTIVITE6 NE 106) AND (P5.QACTIVITE7 NE 107) AND (P5.QACTIVITE8 EQ 108) AND (P5.AP8JOUR EQ 7)) [P5.DIMANCHEAP1 = P5.AP1];
IF ((P5.QACTIVITE1 NE 101) AND (P5.QACTIVITE2 NE 102) AND (P5.QACTIVITE3 NE 103) AND (P5.QACTIVITE4 NE 104) AND (P5.QACTIVITES5 NE 105) AND (P5.QACTIVITE6 NE 106) AND (P5.QACTIVITE7 NE 107) AND (P5.QACTIVITE8 NE 108) AND (P5.QACTIVITE9 EQ 109) AND (P5.AP9JOUR EQ 7)) [P5.DIMANCHEAP1 = P5.AP1];
IF ((P5.QACTIVITE1 NE 101) AND (P5.QACTIVITE2 NE 102) AND (P5.QACTIVITE3 NE 103) AND (P5.QACTIVITE4 NE 104) AND (P5.QACTIVITES5 NE 105) AND (P5.QACTIVITE6 NE 106) AND (P5.QACTIVITE7 NE 107) AND (P5.QACTIVITE8 NE 108) AND (P5.QACTIVITE9 NE 109) AND (P5.QACTIVITE_10 EQ 110) AND (P5.AP10JOUR EQ 7)) [P5.DIMANCHEAP1 = P5.AP1];
IF ((P5.QACTIVITE1 NE 101) AND (P5.QACTIVITE2 NE 102) AND (P5.QACTIVITE3 NE 103) AND (P5.QACTIVITE4 NE 104) AND (P5.QACTIVITES5 NE 105) AND (P5.QACTIVITE6 NE 106) AND (P5.QACTIVITE7 NE 107) AND (P5.QACTIVITE8 NE 108) AND (P5.QACTIVITE9 NE 109) AND (P5.QACTIVITE_10 NE 110) AND (P5.QACTIVITE_11 EQ 111) AND (P5.AP11JOUR EQ 7)) [P5.DIMANCHEAP1 = P5.AP1];
IF ((P5.QACTIVITE1 NE 101) AND (P5.QACTIVITE2 NE 102) AND (P5.QACTIVITE3 NE 103) AND (P5.QACTIVITE4 NE 104) AND (P5.QACTIVITES5 NE 105) AND (P5.QACTIVITE6 NE 106) AND (P5.QACTIVITE7

NE 107) AND (P5.QACTIVITE8 NE 108) AND (P5.QACTIVITE9 NE 109) AND (P5.QACTIVITE_10 NE 110) AND (P5.QACTIVITE_11 NE 111) AND (P5.QACTIVITE_12 EQ 112) AND (P5.AP12JOUR EQ 7)) [P5.DIMANCHEAP1 = P5.AP1];
ACTIVITÉ 2 DANS LE PLAN POUR CHAQUE JOUR DE LA SEMAINE
IF ((P5.2SUMAP2 GE 100) AND (P5.2SUMAP2 LT 200) AND (P5.QACTIVITE2 EQ 102) AND (P5.AP2JOUR EQ 1)) [P5.LUNDIAP2 = P5.AP2];
IF ((P5.3SUMAP2 GE 100) AND (P5.3SUMAP2 LT 200) AND (P5.QACTIVITE3 EQ 103) AND (P5.AP3JOUR EQ 1)) [P5.LUNDIAP2 = P5.AP2];
IF ((P5.4SUMAP2 GE 100) AND (P5.4SUMAP2 LT 200) AND (P5.QACTIVITE4 EQ 104) AND (P5.AP4JOUR EQ 1)) [P5.LUNDIAP2 = P5.AP2];
IF ((P5.5SUMAP2 GE 100) AND (P5.5SUMAP2 LT 200) AND (P5.QACTIVITE5 EQ 105) AND (P5.AP5JOUR EQ 1)) [P5.LUNDIAP2 = P5.AP2];
IF ((P5.6SUMAP2 GE 100) AND (P5.6SUMAP2 LT 200) AND (P5.QACTIVITE6 EQ 106) AND (P5.AP6JOUR EQ 1)) [P5.LUNDIAP2 = P5.AP2];
IF ((P5.7SUMAP2 GE 100) AND (P5.7SUMAP2 LT 200) AND (P5.QACTIVITE7 EQ 107) AND (P5.AP7JOUR EQ 1)) [P5.LUNDIAP2 = P5.AP2];
IF ((P5.8SUMAP2 GE 100) AND (P5.8SUMAP2 LT 200) AND (P5.QACTIVITE8 EQ 108) AND (P5.AP8JOUR EQ 1)) [P5.LUNDIAP2 = P5.AP2];
IF ((P5.9SUMAP2 GE 100) AND (P5.9SUMAP2 LT 200) AND (P5.QACTIVITE9 EQ 109) AND (P5.AP9JOUR EQ 1)) [P5.LUNDIAP2 = P5.AP2];
IF ((P5.10SUMAP2 GE 100) AND (P5.10SUMAP2 LT 200) AND (P5.QACTIVITE_10 EQ 110) AND (P5.AP10JOUR EQ 1)) [P5.LUNDIAP2 = P5.AP2];
IF ((P5.11SUMAP2 GE 100) AND (P5.11SUMAP2 LT 200) AND (P5.QACTIVITE_11 EQ 111) AND (P5.AP11JOUR EQ 1)) [P5.LUNDIAP2 = P5.AP2];
IF ((P5.12SUMAP2 GE 100) AND (P5.12SUMAP2 LT 200) AND (P5.QACTIVITE_12 EQ 112) AND (P5.AP12JOUR EQ 1)) [P5.LUNDIAP2 = P5.AP2];
IF ((P5.2SUMAP2 GE 100) AND (P5.2SUMAP2 LT 200) AND (P5.QACTIVITE2 EQ 102) AND (P5.AP2JOUR EQ 2)) [P5.MARDIAP2 = P5.AP2];
IF ((P5.3SUMAP2 GE 100) AND (P5.3SUMAP2 LT 200) AND (P5.QACTIVITE3 EQ 103) AND (P5.AP3JOUR EQ 2)) [P5.MARDIAP2 = P5.AP2];
IF ((P5.4SUMAP2 GE 100) AND (P5.4SUMAP2 LT 200) AND (P5.QACTIVITE4 EQ 104) AND (P5.AP4JOUR EQ 2)) [P5.MARDIAP2 = P5.AP2];
IF ((P5.5SUMAP2 GE 100) AND (P5.5SUMAP2 LT 200) AND (P5.QACTIVITE5 EQ 105) AND (P5.AP5JOUR EQ 2)) [P5.MARDIAP2 = P5.AP2];
IF ((P5.6SUMAP2 GE 100) AND (P5.6SUMAP2 LT 200) AND (P5.QACTIVITE6 EQ 106) AND (P5.AP6JOUR EQ 2)) [P5.MARDIAP2 = P5.AP2];
IF ((P5.7SUMAP2 GE 100) AND (P5.7SUMAP2 LT 200) AND (P5.QACTIVITE7 EQ 107) AND (P5.AP7JOUR EQ 2)) [P5.MARDIAP2 = P5.AP2];
IF ((P5.8SUMAP2 GE 100) AND (P5.8SUMAP2 LT 200) AND (P5.QACTIVITE8 EQ 108) AND (P5.AP8JOUR EQ 2)) [P5.MARDIAP2 = P5.AP2];
IF ((P5.9SUMAP2 GE 100) AND (P5.9SUMAP2 LT 200) AND (P5.QACTIVITE9 EQ 109) AND (P5.AP9JOUR EQ 2)) [P5.MARDIAP2 = P5.AP2];

IF ((P5.10SUMAP2 GE 100) AND (P5.10SUMAP2 LT 200) AND (P5.QACTIVITE_10 EQ 110) AND (P5.AP10JOUR EQ 2)) [P5.MARDIAP2 = P5.AP2];
IF ((P5.11SUMAP2 GE 100) AND (P5.11SUMAP2 LT 200) AND (P5.QACTIVITE_11 EQ 111) AND (P5.AP11JOUR EQ 2)) [P5.MARDIAP2 = P5.AP2];
IF ((P5.12SUMAP2 GE 100) AND (P5.12SUMAP2 LT 200) AND (P5.QACTIVITE_12 EQ 112) AND (P5.AP12JOUR EQ 2)) [P5.MARDIAP2 = P5.AP2];
IF ((P5.2SUMAP2 GE 100) AND (P5.2SUMAP2 LT 200) AND (P5.QACTIVITE2 EQ 102) AND (P5.AP2JOUR EQ 3)) [P5.MERCREDIAP2 = P5.AP2];
IF ((P5.3SUMAP2 GE 100) AND (P5.3SUMAP2 LT 200) AND (P5.QACTIVITE3 EQ 103) AND (P5.AP3JOUR EQ 3)) [P5.MERCREDIAP2 = P5.AP2];
IF ((P5.4SUMAP2 GE 100) AND (P5.4SUMAP2 LT 200) AND (P5.QACTIVITE4 EQ 104) AND (P5.AP4JOUR EQ 3)) [P5.MERCREDIAP2 = P5.AP2];
IF ((P5.5SUMAP2 GE 100) AND (P5.5SUMAP2 LT 200) AND (P5.QACTIVITE5 EQ 105) AND (P5.AP5JOUR EQ 3)) [P5.MERCREDIAP2 = P5.AP2];
IF ((P5.6SUMAP2 GE 100) AND (P5.6SUMAP2 LT 200) AND (P5.QACTIVITE6 EQ 106) AND (P5.AP6JOUR EQ 3)) [P5.MERCREDIAP2 = P5.AP2];
IF ((P5.7SUMAP2 GE 100) AND (P5.7SUMAP2 LT 200) AND (P5.QACTIVITE7 EQ 107) AND (P5.AP7JOUR EQ 3)) [P5.MERCREDIAP2 = P5.AP2];
IF ((P5.8SUMAP2 GE 100) AND (P5.8SUMAP2 LT 200) AND (P5.QACTIVITE8 EQ 108) AND (P5.AP8JOUR EQ 3)) [P5.MERCREDIAP2 = P5.AP2];
IF ((P5.9SUMAP2 GE 100) AND (P5.9SUMAP2 LT 200) AND (P5.QACTIVITE9 EQ 109) AND (P5.AP9JOUR EQ 3)) [P5.MERCREDIAP2 = P5.AP2];
IF ((P5.10SUMAP2 GE 100) AND (P5.10SUMAP2 LT 200) AND (P5.QACTIVITE_10 EQ 110) AND (P5.AP10JOUR EQ 3)) [P5.MERCREDIAP2 = P5.AP2];
IF ((P5.11SUMAP2 GE 100) AND (P5.11SUMAP2 LT 200) AND (P5.QACTIVITE_11 EQ 111) AND (P5.AP11JOUR EQ 3)) [P5.MERCREDIAP2 = P5.AP2];
IF ((P5.12SUMAP2 GE 100) AND (P5.12SUMAP2 LT 200) AND (P5.QACTIVITE_12 EQ 112) AND (P5.AP12JOUR EQ 3)) [P5.MERCREDIAP2 = P5.AP2];
IF ((P5.2SUMAP2 GE 100) AND (P5.2SUMAP2 LT 200) AND (P5.QACTIVITE2 EQ 102) AND (P5.AP2JOUR EQ 4)) [P5.JEUDIAP2 = P5.AP2];
IF ((P5.3SUMAP2 GE 100) AND (P5.3SUMAP2 LT 200) AND (P5.QACTIVITE3 EQ 103) AND (P5.AP3JOUR EQ 4)) [P5.JEUDIAP2 = P5.AP2];
IF ((P5.4SUMAP2 GE 100) AND (P5.4SUMAP2 LT 200) AND (P5.QACTIVITE4 EQ 104) AND (P5.AP4JOUR EQ 4)) [P5.JEUDIAP2 = P5.AP2];
IF ((P5.5SUMAP2 GE 100) AND (P5.5SUMAP2 LT 200) AND (P5.QACTIVITE5 EQ 105) AND (P5.AP5JOUR EQ 4)) [P5.JEUDIAP2 = P5.AP2];
IF ((P5.6SUMAP2 GE 100) AND (P5.6SUMAP2 LT 200) AND (P5.QACTIVITE6 EQ 106) AND (P5.AP6JOUR EQ 4)) [P5.JEUDIAP2 = P5.AP2];
IF ((P5.7SUMAP2 GE 100) AND (P5.7SUMAP2 LT 200) AND (P5.QACTIVITE7 EQ 107) AND (P5.AP7JOUR EQ 4)) [P5.JEUDIAP2 = P5.AP2];
IF ((P5.8SUMAP2 GE 100) AND (P5.8SUMAP2 LT 200) AND (P5.QACTIVITE8 EQ 108) AND (P5.AP8JOUR EQ 4)) [P5.JEUDIAP2 = P5.AP2];

```
IF ((P5.9SUMAP2 GE 100) AND (P5.9SUMAP2 LT 200) AND (P5.QACTIVITE9 EQ 109) AND (P5.AP9JOUR EQ
4)) [P5.JEUDIAP2 = P5.AP2];
IF ((P5.10SUMAP2 GE 100) AND (P5.10SUMAP2 LT 200) AND (P5.QACTIVITE_10 EQ 110) AND (P5.AP10JOUR
EQ 4)) [P5.JEUDIAP2 = P5.AP2];
IF ((P5.11SUMAP2 GE 100) AND (P5.11SUMAP2 LT 200) AND (P5.QACTIVITE_11 EQ 111) AND (P5.AP11JOUR
EQ 4)) [P5.JEUDIAP2 = P5.AP2];
IF ((P5.12SUMAP2 GE 100) AND (P5.12SUMAP2 LT 200) AND (P5.QACTIVITE_12 EQ 112) AND (P5.AP12JOUR
EQ 4)) [P5.JEUDIAP2 = P5.AP2];
IF ((P5.2SUMAP2 GE 100) AND (P5.2SUMAP2 LT 200) AND (P5.QACTIVITE2 EQ 102) AND (P5.AP2JOUR EQ
5)) [P5.VENDREDIAP2 = P5.AP2];
IF ((P5.3SUMAP2 GE 100) AND (P5.3SUMAP2 LT 200) AND (P5.QACTIVITE3 EQ 103) AND (P5.AP3JOUR EQ
5)) [P5.VENDREDIAP2 = P5.AP2];
IF ((P5.4SUMAP2 GE 100) AND (P5.4SUMAP2 LT 200) AND (P5.QACTIVITE4 EQ 104) AND (P5.AP4JOUR EQ
5)) [P5.VENDREDIAP2 = P5.AP2];
IF ((P5.5SUMAP2 GE 100) AND (P5.5SUMAP2 LT 200) AND (P5.QACTIVITE5 EQ 105) AND (P5.AP5JOUR EQ
5)) [P5.VENDREDIAP2 = P5.AP2];
IF ((P5.6SUMAP2 GE 100) AND (P5.6SUMAP2 LT 200) AND (P5.QACTIVITE6 EQ 106) AND (P5.AP6JOUR EQ
5)) [P5.VENDREDIAP2 = P5.AP2];
IF ((P5.7SUMAP2 GE 100) AND (P5.7SUMAP2 LT 200) AND (P5.QACTIVITE7 EQ 107) AND (P5.AP7JOUR EQ
5)) [P5.VENDREDIAP2 = P5.AP2];
IF ((P5.8SUMAP2 GE 100) AND (P5.8SUMAP2 LT 200) AND (P5.QACTIVITE8 EQ 108) AND (P5.AP8JOUR EQ
5)) [P5.VENDREDIAP2 = P5.AP2];
IF ((P5.9SUMAP2 GE 100) AND (P5.9SUMAP2 LT 200) AND (P5.QACTIVITE9 EQ 109) AND (P5.AP9JOUR EQ
5)) [P5.VENDREDIAP2 = P5.AP2];
IF ((P5.10SUMAP2 GE 100) AND (P5.10SUMAP2 LT 200) AND (P5.QACTIVITE_10 EQ 110) AND (P5.AP10JOUR
EQ 5)) [P5.VENDREDIAP2 = P5.AP2];
IF ((P5.11SUMAP2 GE 100) AND (P5.11SUMAP2 LT 200) AND (P5.QACTIVITE_11 EQ 111) AND (P5.AP11JOUR
EQ 5)) [P5.VENDREDIAP2 = P5.AP2];
IF ((P5.12SUMAP2 GE 100) AND (P5.12SUMAP2 LT 200) AND (P5.QACTIVITE_12 EQ 112) AND (P5.AP12JOUR
EQ 5)) [P5.VENDREDIAP2 = P5.AP2];
IF ((P5.2SUMAP2 GE 100) AND (P5.2SUMAP2 LT 200) AND (P5.QACTIVITE2 EQ 102) AND (P5.AP2JOUR EQ
6)) [P5.SAMEDIAP2 = P5.AP2];
IF ((P5.3SUMAP2 GE 100) AND (P5.3SUMAP2 LT 200) AND (P5.QACTIVITE3 EQ 103) AND (P5.AP3JOUR EQ
6)) [P5.SAMEDIAP2 = P5.AP2];
IF ((P5.4SUMAP2 GE 100) AND (P5.4SUMAP2 LT 200) AND (P5.QACTIVITE4 EQ 104) AND (P5.AP4JOUR EQ
6)) [P5.SAMEDIAP2 = P5.AP2];
IF ((P5.5SUMAP2 GE 100) AND (P5.5SUMAP2 LT 200) AND (P5.QACTIVITE5 EQ 105) AND (P5.AP5JOUR EQ
6)) [P5.SAMEDIAP2 = P5.AP2];
IF ((P5.6SUMAP2 GE 100) AND (P5.6SUMAP2 LT 200) AND (P5.QACTIVITE6 EQ 106) AND (P5.AP6JOUR EQ
6)) [P5.SAMEDIAP2 = P5.AP2];
IF ((P5.7SUMAP2 GE 100) AND (P5.7SUMAP2 LT 200) AND (P5.QACTIVITE7 EQ 107) AND (P5.AP7JOUR EQ
6)) [P5.SAMEDIAP2 = P5.AP2];
```

IF ((P5.8SUMAP2 GE 100) AND (P5.8SUMAP2 LT 200) AND (P5.QACTIVITE8 EQ 108) AND (P5.AP8JOUR EQ 6)) [P5.SAMEDIAP2 = P5.AP2];
IF ((P5.9SUMAP2 GE 100) AND (P5.9SUMAP2 LT 200) AND (P5.QACTIVITE9 EQ 109) AND (P5.AP9JOUR EQ 6)) [P5.SAMEDIAP2 = P5.AP2];
IF ((P5.10SUMAP2 GE 100) AND (P5.10SUMAP2 LT 200) AND (P5.QACTIVITE_10 EQ 110) AND (P5.AP10JOUR EQ 6)) [P5.SAMEDIAP2 = P5.AP2];
IF ((P5.11SUMAP2 GE 100) AND (P5.11SUMAP2 LT 200) AND (P5.QACTIVITE_11 EQ 111) AND (P5.AP11JOUR EQ 6)) [P5.SAMEDIAP2 = P5.AP2];
IF ((P5.12SUMAP2 GE 100) AND (P5.12SUMAP2 LT 200) AND (P5.QACTIVITE_12 EQ 112) AND (P5.AP12JOUR EQ 6)) [P5.SAMEDIAP2 = P5.AP2];
IF ((P5.2SUMAP2 GE 100) AND (P5.2SUMAP2 LT 200) AND (P5.QACTIVITE2 EQ 102) AND (P5.AP2JOUR EQ 7)) [P5.DIMANCHEAP2 = P5.AP2];
IF ((P5.3SUMAP2 GE 100) AND (P5.3SUMAP2 LT 200) AND (P5.QACTIVITE3 EQ 103) AND (P5.AP3JOUR EQ 7)) [P5.DIMANCHEAP2 = P5.AP2];
IF ((P5.4SUMAP2 GE 100) AND (P5.4SUMAP2 LT 200) AND (P5.QACTIVITE4 EQ 104) AND (P5.AP4JOUR EQ 7)) [P5.DIMANCHEAP2 = P5.AP2];
IF ((P5.5SUMAP2 GE 100) AND (P5.5SUMAP2 LT 200) AND (P5.QACTIVITE5 EQ 105) AND (P5.AP5JOUR EQ 7)) [P5.DIMANCHEAP2 = P5.AP2];
IF ((P5.6SUMAP2 GE 100) AND (P5.6SUMAP2 LT 200) AND (P5.QACTIVITE6 EQ 106) AND (P5.AP6JOUR EQ 7)) [P5.DIMANCHEAP2 = P5.AP2];
IF ((P5.7SUMAP2 GE 100) AND (P5.7SUMAP2 LT 200) AND (P5.QACTIVITE7 EQ 107) AND (P5.AP7JOUR EQ 7)) [P5.DIMANCHEAP2 = P5.AP2];
IF ((P5.8SUMAP2 GE 100) AND (P5.8SUMAP2 LT 200) AND (P5.QACTIVITE8 EQ 108) AND (P5.AP8JOUR EQ 7)) [P5.DIMANCHEAP2 = P5.AP2];
IF ((P5.9SUMAP2 GE 100) AND (P5.9SUMAP2 LT 200) AND (P5.QACTIVITE9 EQ 109) AND (P5.AP9JOUR EQ 7)) [P5.DIMANCHEAP2 = P5.AP2];
IF ((P5.10SUMAP2 GE 100) AND (P5.10SUMAP2 LT 200) AND (P5.QACTIVITE_10 EQ 110) AND (P5.AP10JOUR EQ 7)) [P5.DIMANCHEAP2 = P5.AP2];
IF ((P5.11SUMAP2 GE 100) AND (P5.11SUMAP2 LT 200) AND (P5.QACTIVITE_11 EQ 111) AND (P5.AP11JOUR EQ 7)) [P5.DIMANCHEAP2 = P5.AP2];
IF ((P5.12SUMAP2 GE 100) AND (P5.12SUMAP2 LT 200) AND (P5.QACTIVITE_12 EQ 112) AND (P5.AP12JOUR EQ 7)) [P5.DIMANCHEAP2 = P5.AP2];
ACTIVITÉ 3 DANS LE PLAN POUR CHAQUE JOUR DE LA SEMAINE
IF ((P5.3SUMAP3 GE 200) AND (P5.3SUMAP3 LT 300) AND (P5.QACTIVITE3 EQ 103) AND (P5.AP3JOUR EQ 1)) [P5.LUNDIAP3 = P5.AP3];
IF ((P5.4SUMAP3 GE 200) AND (P5.4SUMAP3 LT 300) AND (P5.QACTIVITE4 EQ 104) AND (P5.AP4JOUR EQ 1)) [P5.LUNDIAP3 = P5.AP3];
IF ((P5.5SUMAP3 GE 200) AND (P5.5SUMAP3 LT 300) AND (P5.QACTIVITE5 EQ 105) AND (P5.AP5JOUR EQ 1)) [P5.LUNDIAP3 = P5.AP3];
IF ((P5.6SUMAP3 GE 200) AND (P5.6SUMAP3 LT 300) AND (P5.QACTIVITE6 EQ 106) AND (P5.AP6JOUR EQ 1)) [P5.LUNDIAP3 = P5.AP3];

IF ((P5.7SUMAP3 GE 200) AND (P5.7SUMAP3 LT 300) AND (P5.QACTIVITE7 EQ 107) AND (P5.AP7JOUR EQ 1)) [P5.LUNDIAP3 = P5.AP3];
IF ((P5.8SUMAP3 GE 200) AND (P5.8SUMAP3 LT 300) AND (P5.QACTIVITE8 EQ 108) AND (P5.AP8JOUR EQ 1)) [P5.LUNDIAP3 = P5.AP3];
IF ((P5.9SUMAP3 GE 200) AND (P5.9SUMAP3 LT 300) AND (P5.QACTIVITE9 EQ 109) AND (P5.AP9JOUR EQ 1)) [P5.LUNDIAP3 = P5.AP3];
IF ((P5.10SUMAP3 GE 200) AND (P5.10SUMAP3 LT 300) AND (P5.QACTIVITE_10 EQ 110) AND (P5.AP10JOUR EQ 1)) [P5.LUNDIAP3 = P5.AP3];
IF ((P5.11SUMAP3 GE 200) AND (P5.11SUMAP3 LT 300) AND (P5.QACTIVITE_11 EQ 111) AND (P5.AP11JOUR EQ 1)) [P5.LUNDIAP3 = P5.AP3];
IF ((P5.12SUMAP3 GE 200) AND (P5.12SUMAP3 LT 300) AND (P5.QACTIVITE_12 EQ 112) AND (P5.AP12JOUR EQ 1)) [P5.LUNDIAP3 = P5.AP3];
IF ((P5.3SUMAP3 GE 200) AND (P5.3SUMAP3 LT 300) AND (P5.QACTIVITE3 EQ 103) AND (P5.AP3JOUR EQ 2)) [P5.MARDIAP3 = P5.AP3];
IF ((P5.4SUMAP3 GE 200) AND (P5.4SUMAP3 LT 300) AND (P5.QACTIVITE4 EQ 104) AND (P5.AP4JOUR EQ 2)) [P5.MARDIAP3 = P5.AP3];
IF ((P5.5SUMAP3 GE 200) AND (P5.5SUMAP3 LT 300) AND (P5.QACTIVITE5 EQ 105) AND (P5.AP5JOUR EQ 2)) [P5.MARDIAP3 = P5.AP3];
IF ((P5.6SUMAP3 GE 200) AND (P5.6SUMAP3 LT 300) AND (P5.QACTIVITE6 EQ 106) AND (P5.AP6JOUR EQ 2)) [P5.MARDIAP3 = P5.AP3];
IF ((P5.7SUMAP3 GE 200) AND (P5.7SUMAP3 LT 300) AND (P5.QACTIVITE7 EQ 107) AND (P5.AP7JOUR EQ 2)) [P5.MARDIAP3 = P5.AP3];
IF ((P5.8SUMAP3 GE 200) AND (P5.8SUMAP3 LT 300) AND (P5.QACTIVITE8 EQ 108) AND (P5.AP8JOUR EQ 2)) [P5.MARDIAP3 = P5.AP3];
IF ((P5.9SUMAP3 GE 200) AND (P5.9SUMAP3 LT 300) AND (P5.QACTIVITE9 EQ 109) AND (P5.AP9JOUR EQ 2)) [P5.MARDIAP3 = P5.AP3];
IF ((P5.10SUMAP3 GE 200) AND (P5.10SUMAP3 LT 300) AND (P5.QACTIVITE_10 EQ 110) AND (P5.AP10JOUR EQ 2)) [P5.MARDIAP3 = P5.AP3];
IF ((P5.11SUMAP3 GE 200) AND (P5.11SUMAP3 LT 300) AND (P5.QACTIVITE_11 EQ 111) AND (P5.AP11JOUR EQ 2)) [P5.MARDIAP3 = P5.AP3];
IF ((P5.12SUMAP3 GE 200) AND (P5.12SUMAP3 LT 300) AND (P5.QACTIVITE_12 EQ 112) AND (P5.AP12JOUR EQ 2)) [P5.MARDIAP3 = P5.AP3];
IF ((P5.3SUMAP3 GE 200) AND (P5.3SUMAP3 LT 300) AND (P5.QACTIVITE3 EQ 103) AND (P5.AP3JOUR EQ 3)) [P5.MERCREDIAP3 = P5.AP3];
IF ((P5.4SUMAP3 GE 200) AND (P5.4SUMAP3 LT 300) AND (P5.QACTIVITE4 EQ 104) AND (P5.AP4JOUR EQ 3)) [P5.MERCREDIAP3 = P5.AP3];
IF ((P5.5SUMAP3 GE 200) AND (P5.5SUMAP3 LT 300) AND (P5.QACTIVITE5 EQ 105) AND (P5.AP5JOUR EQ 3)) [P5.MERCREDIAP3 = P5.AP3];
IF ((P5.6SUMAP3 GE 200) AND (P5.6SUMAP3 LT 300) AND (P5.QACTIVITE6 EQ 106) AND (P5.AP6JOUR EQ 3)) [P5.MERCREDIAP3 = P5.AP3];
IF ((P5.7SUMAP3 GE 200) AND (P5.7SUMAP3 LT 300) AND (P5.QACTIVITE7 EQ 107) AND (P5.AP7JOUR EQ 3)) [P5.MERCREDIAP3 = P5.AP3];

IF ((P5.8SUMAP3 GE 200) AND (P5.8SUMAP3 LT 300) AND (P5.QACTIVITE8 EQ 108) AND (P5.AP8JOUR EQ 3)) [P5.MERCREDIAP3 = P5.AP3];
IF ((P5.9SUMAP3 GE 200) AND (P5.9SUMAP3 LT 300) AND (P5.QACTIVITE9 EQ 109) AND (P5.AP9JOUR EQ 3)) [P5.MERCREDIAP3 = P5.AP3];
IF ((P5.10SUMAP3 GE 200) AND (P5.10SUMAP3 LT 300) AND (P5.QACTIVITE_10 EQ 110) AND (P5.AP10JOUR EQ 3)) [P5.MERCREDIAP3 = P5.AP3];
IF ((P5.11SUMAP3 GE 200) AND (P5.11SUMAP3 LT 300) AND (P5.QACTIVITE_11 EQ 111) AND (P5.AP11JOUR EQ 3)) [P5.MERCREDIAP3 = P5.AP3];
IF ((P5.12SUMAP3 GE 200) AND (P5.12SUMAP3 LT 300) AND (P5.QACTIVITE_12 EQ 112) AND (P5.AP12JOUR EQ 3)) [P5.MERCREDIAP3 = P5.AP3];
IF ((P5.3SUMAP3 GE 200) AND (P5.3SUMAP3 LT 300) AND (P5.QACTIVITE3 EQ 103) AND (P5.AP3JOUR EQ 4)) [P5.JEUDIAP3 = P5.AP3];
IF ((P5.4SUMAP3 GE 200) AND (P5.4SUMAP3 LT 300) AND (P5.QACTIVITE4 EQ 104) AND (P5.AP4JOUR EQ 4)) [P5.JEUDIAP3 = P5.AP3];
IF ((P5.5SUMAP3 GE 200) AND (P5.5SUMAP3 LT 300) AND (P5.QACTIVITE5 EQ 105) AND (P5.AP5JOUR EQ 4)) [P5.JEUDIAP3 = P5.AP3];
IF ((P5.6SUMAP3 GE 200) AND (P5.6SUMAP3 LT 300) AND (P5.QACTIVITE6 EQ 106) AND (P5.AP6JOUR EQ 4)) [P5.JEUDIAP3 = P5.AP3];
IF ((P5.7SUMAP3 GE 200) AND (P5.7SUMAP3 LT 300) AND (P5.QACTIVITE7 EQ 107) AND (P5.AP7JOUR EQ 4)) [P5.JEUDIAP3 = P5.AP3];
IF ((P5.8SUMAP3 GE 200) AND (P5.8SUMAP3 LT 300) AND (P5.QACTIVITE8 EQ 108) AND (P5.AP8JOUR EQ 4)) [P5.JEUDIAP3 = P5.AP3];
IF ((P5.9SUMAP3 GE 200) AND (P5.9SUMAP3 LT 300) AND (P5.QACTIVITE9 EQ 109) AND (P5.AP9JOUR EQ 4)) [P5.JEUDIAP3 = P5.AP3];
IF ((P5.10SUMAP3 GE 200) AND (P5.10SUMAP3 LT 300) AND (P5.QACTIVITE_10 EQ 110) AND (P5.AP10JOUR EQ 4)) [P5.JEUDIAP3 = P5.AP3];
IF ((P5.11SUMAP3 GE 200) AND (P5.11SUMAP3 LT 300) AND (P5.QACTIVITE_11 EQ 111) AND (P5.AP11JOUR EQ 4)) [P5.JEUDIAP3 = P5.AP3];
IF ((P5.12SUMAP3 GE 200) AND (P5.12SUMAP3 LT 300) AND (P5.QACTIVITE_12 EQ 112) AND (P5.AP12JOUR EQ 4)) [P5.JEUDIAP3 = P5.AP3];
IF ((P5.3SUMAP3 GE 200) AND (P5.3SUMAP3 LT 300) AND (P5.QACTIVITE3 EQ 103) AND (P5.AP3JOUR EQ 5)) [P5.VENDREDIAP3 = P5.AP3];
IF ((P5.4SUMAP3 GE 200) AND (P5.4SUMAP3 LT 300) AND (P5.QACTIVITE4 EQ 104) AND (P5.AP4JOUR EQ 5)) [P5.VENDREDIAP3 = P5.AP3];
IF ((P5.5SUMAP3 GE 200) AND (P5.5SUMAP3 LT 300) AND (P5.QACTIVITE5 EQ 105) AND (P5.AP5JOUR EQ 5)) [P5.VENDREDIAP3 = P5.AP3];
IF ((P5.6SUMAP3 GE 200) AND (P5.6SUMAP3 LT 300) AND (P5.QACTIVITE6 EQ 106) AND (P5.AP6JOUR EQ 5)) [P5.VENDREDIAP3 = P5.AP3];
IF ((P5.7SUMAP3 GE 200) AND (P5.7SUMAP3 LT 300) AND (P5.QACTIVITE7 EQ 107) AND (P5.AP7JOUR EQ 5)) [P5.VENDREDIAP3 = P5.AP3];
IF ((P5.8SUMAP3 GE 200) AND (P5.8SUMAP3 LT 300) AND (P5.QACTIVITE8 EQ 108) AND (P5.AP8JOUR EQ 5)) [P5.VENDREDIAP3 = P5.AP3];

IF ((P5.9SUMAP3 GE 200) AND (P5.9SUMAP3 LT 300) AND (P5.QACTIVITE9 EQ 109) AND (P5.AP9JOUR EQ 5)) [P5.VENDREDIAP3 = P5.AP3];
IF ((P5.10SUMAP3 GE 200) AND (P5.10SUMAP3 LT 300) AND (P5.QACTIVITE_10 EQ 110) AND (P5.AP10JOUR EQ 5)) [P5.VENDREDIAP3 = P5.AP3];
IF ((P5.11SUMAP3 GE 200) AND (P5.11SUMAP3 LT 300) AND (P5.QACTIVITE_11 EQ 111) AND (P5.AP11JOUR EQ 5)) [P5.VENDREDIAP3 = P5.AP3];
IF ((P5.12SUMAP3 GE 200) AND (P5.12SUMAP3 LT 300) AND (P5.QACTIVITE_12 EQ 112) AND (P5.AP12JOUR EQ 5)) [P5.VENDREDIAP3 = P5.AP3];
IF ((P5.3SUMAP3 GE 200) AND (P5.3SUMAP3 LT 300) AND (P5.QACTIVITE3 EQ 103) AND (P5.AP3JOUR EQ 6)) [P5.SAMEDIAP3 = P5.AP3];
IF ((P5.4SUMAP3 GE 200) AND (P5.4SUMAP3 LT 300) AND (P5.QACTIVITE4 EQ 104) AND (P5.AP4JOUR EQ 6)) [P5.SAMEDIAP3 = P5.AP3];
IF ((P5.5SUMAP3 GE 200) AND (P5.5SUMAP3 LT 300) AND (P5.QACTIVITE5 EQ 105) AND (P5.AP5JOUR EQ 6)) [P5.SAMEDIAP3 = P5.AP3];
IF ((P5.6SUMAP3 GE 200) AND (P5.6SUMAP3 LT 300) AND (P5.QACTIVITE6 EQ 106) AND (P5.AP6JOUR EQ 6)) [P5.SAMEDIAP3 = P5.AP3];
IF ((P5.7SUMAP3 GE 200) AND (P5.7SUMAP3 LT 300) AND (P5.QACTIVITE7 EQ 107) AND (P5.AP7JOUR EQ 6)) [P5.SAMEDIAP3 = P5.AP3];
IF ((P5.8SUMAP3 GE 200) AND (P5.8SUMAP3 LT 300) AND (P5.QACTIVITE8 EQ 108) AND (P5.AP8JOUR EQ 6)) [P5.SAMEDIAP3 = P5.AP3];
IF ((P5.9SUMAP3 GE 200) AND (P5.9SUMAP3 LT 300) AND (P5.QACTIVITE9 EQ 109) AND (P5.AP9JOUR EQ 6)) [P5.SAMEDIAP3 = P5.AP3];
IF ((P5.10SUMAP3 GE 200) AND (P5.10SUMAP3 LT 300) AND (P5.QACTIVITE_10 EQ 110) AND (P5.AP10JOUR EQ 6)) [P5.SAMEDIAP3 = P5.AP3];
IF ((P5.11SUMAP3 GE 200) AND (P5.11SUMAP3 LT 300) AND (P5.QACTIVITE_11 EQ 111) AND (P5.AP11JOUR EQ 6)) [P5.SAMEDIAP3 = P5.AP3];
IF ((P5.12SUMAP3 GE 200) AND (P5.12SUMAP3 LT 300) AND (P5.QACTIVITE_12 EQ 112) AND (P5.AP12JOUR EQ 6)) [P5.SAMEDIAP3 = P5.AP3];
IF ((P5.3SUMAP3 GE 200) AND (P5.3SUMAP3 LT 300) AND (P5.QACTIVITE3 EQ 103) AND (P5.AP3JOUR EQ 7)) [P5.DIMANCHEAP3 = P5.AP3];
IF ((P5.4SUMAP3 GE 200) AND (P5.4SUMAP3 LT 300) AND (P5.QACTIVITE4 EQ 104) AND (P5.AP4JOUR EQ 7)) [P5.DIMANCHEAP3 = P5.AP3];
IF ((P5.5SUMAP3 GE 200) AND (P5.5SUMAP3 LT 300) AND (P5.QACTIVITE5 EQ 105) AND (P5.AP5JOUR EQ 7)) [P5.DIMANCHEAP3 = P5.AP3];
IF ((P5.6SUMAP3 GE 200) AND (P5.6SUMAP3 LT 300) AND (P5.QACTIVITE6 EQ 106) AND (P5.AP6JOUR EQ 7)) [P5.DIMANCHEAP3 = P5.AP3];
IF ((P5.7SUMAP3 GE 200) AND (P5.7SUMAP3 LT 300) AND (P5.QACTIVITE7 EQ 107) AND (P5.AP7JOUR EQ 7)) [P5.DIMANCHEAP3 = P5.AP3];
IF ((P5.8SUMAP3 GE 200) AND (P5.8SUMAP3 LT 300) AND (P5.QACTIVITE8 EQ 108) AND (P5.AP8JOUR EQ 7)) [P5.DIMANCHEAP3 = P5.AP3];
IF ((P5.9SUMAP3 GE 200) AND (P5.9SUMAP3 LT 300) AND (P5.QACTIVITE9 EQ 109) AND (P5.AP9JOUR EQ 7)) [P5.DIMANCHEAP3 = P5.AP3];

IF ((P5.10SUMAP3 GE 200) AND (P5.10SUMAP3 LT 300) AND (P5.QACTIVITE_10 EQ 110) AND (P5.AP10JOUR EQ 7)) [P5.DIMANCHEAP3 = P5.AP3];
IF ((P5.11SUMAP3 GE 200) AND (P5.11SUMAP3 LT 300) AND (P5.QACTIVITE_11 EQ 111) AND (P5.AP11JOUR EQ 7)) [P5.DIMANCHEAP3 = P5.AP3];
IF ((P5.12SUMAP3 GE 200) AND (P5.12SUMAP3 LT 300) AND (P5.QACTIVITE_12 EQ 112) AND (P5.AP12JOUR EQ 7)) [P5.DIMANCHEAP3 = P5.AP3];
POURSUITE DU CODE
IF (P5.P7BARCHOICE EQ 101) [P5.BAR = 'Être trop fatigué ou fatiguée pour faire de l´activité physique'];
IF (P5.P7BARCHOICE EQ 102) [P5.BAR = 'Avoir beaucoup de choses à faire mis à part l´activité physique'];
IF (P5.P7BARCHOICE EQ 103) [P5.BAR = 'température sera trop mauvaise pour faire de l´activité physique'];
IF (P5.P7BARCHOICE EQ 104) [P5.BAR = 'Ne pas avoir personne avec qui faire de l´activité physique'];
IF (P5.P7BARCHOICE EQ 105) [P5.BAR = 'Ne pas avoir accès à des emplacements pour faire de l´activité physique'];
IF (P5.P7BARCHOICE EQ 106) [P5.BAR = 'Ne pas avoir d´argent pour faire de l´activité physique'];
IF (P5.P7BARCHOICE EQ 107) [P5.BAR = 'Être trop gêné ou gênée, manquer de confiance pour aller faire de l´activité physique'];
IF (P5.P7BARCHOICE EQ 108) [P5.BAR = 'Avoir peur d´être insuffisamment en santé pour faire de l´activité physique'];
IF (P5.P7BARCHOICE EQ 109) [P5.BAR = 'Manquer de motivation'];
IF ((P5.P7BARCHOICE EQ 110) AND (P5.Q_BARANSWER NE 0)) [P5.BAR = P5.Q_BARANSWER];
IF (P5.SUMSOL EQ 101) [P5.SOL = 'Je vais faire de l´activité tôt dans la journée, en matinée ou à l´heure du midi.'];
IF (P5.SUMSOL EQ 102) [P5.SOL = 'J´essaie de garder en tête que si je fais de l´activité physique, j´aurai de plus en plus d´énergie dans la vie.'];
IF (P5.SUMSOL EQ 103) [P5.SOL = 'Je vais planifier des activités avec d´autres personnes qui me motiveront.'];
IF (P5.SUMSOL EQ 104) [P5.SOL = 'Je prends une collation pour me donner de l´énergie et j´y vais quand même.'];
IF (P5.SUMSOL EQ 201) [P5.SOL = 'Faire de l´activité physique par bloc de 10 minutes les journées où je n´ai pas de temps.'];
IF (P5.SUMSOL EQ 202) [P5.SOL = 'Je vais remplacer des activités où je suis assis ou assise, télé, ordinateur ou autres, par des activités physiques.'];
IF (P5.SUMSOL EQ 203) [P5.SOL = 'Je vais me procurer un agenda et inscrire dans mon horaire les moments où je vais faire de l´activité physique.'];
IF (P5.SUMSOL EQ 204) [P5.SOL = 'Je vais prendre des pauses de travail active et faire des 10 minutes de marche.'];
IF (P5.SUMSOL EQ 205) [P5.SOL = 'Je vais me fixer des moments avec d´autres personnes qui me plaisent pour aller faire de l´activité physique.'];
IF (P5.SUMSOL EQ 301) [P5.SOL = 'Je vais me procurer l´équipement nécessaire pour en faire même quand il pleut, quand il neige ou quand il fait trop chaud.'];
IF (P5.SUMSOL EQ 302) [P5.SOL = 'Je vais faire de l’activité physique chez nous ou à l´intérieur dans un endroit que j´aurai choisi quand il fait moins beau.'];

IF (P5.SUMSOL EQ 303) [P5.SOL = 'Je vais me préparer une activité physique alternative au cas où il serait désagréable d´aller dehors.'];
IF (P5.SUMSOL EQ 304) [P5.SOL = 'Je vais me dresser une liste des activités que je serais prêtE à faire si la température est mauvaise.'];
IF (P5.SUMSOL EQ 401) [P5.SOL = 'Je vais me joindre à un groupe qui pratique l´activité physique que j´aime. Ex : la marche, l´aquaforme ou la natation.'];
IF (P5.SUMSOL EQ 402) [P5.SOL = 'Je vais inviter mes amis, ma famille ou mes collègues à faire de l´activité physique avec moi.'];
IF (P5.SUMSOL EQ 403) [P5.SOL = 'Je vais amener mon chien marcher avec moi.'];
IF (P5.SUMSOL EQ 404) [P5.SOL = 'Je vais participer dans des discussions en ligne sur des pages facebook : Diabète Québec et autres.'];
IF (P5.SUMSOL EQ 501) [P5.SOL = 'Je vais faire de la marche ou du vélo dehors, c´est simple et gratuit.'];
IF (P5.SUMSOL EQ 502) [P5.SOL = 'Je vais faire de l´activité physique dans ma maison comme du yoga, un programme sur DVD, vélo stationnaire, tapis roulant, etc.'];
IF (P5.SUMSOL EQ 503) [P5.SOL = 'Je vais aller voir au centre communautaire de mon quartier pour me donner des options.'];
IF (P5.SUMSOL EQ 504) [P5.SOL = 'Je vais discuter avec mon médecin, avec un ami ou un spécialiste de l´activité physique pour avoir des conseils.'];
IF (P5.SUMSOL EQ 601) [P5.SOL = 'Je vais trouver des activités physiques abordables et simples : marcher dehors, faire du vélo, nager à la piscine communautaire.'];
IF (P5.SUMSOL EQ 602) [P5.SOL = 'Je vais aller voir au centre communautaire de mon quartier pour me donner des options.'];
IF (P5.SUMSOL EQ 603) [P5.SOL = 'Je vais économiser pour m´acheter un DVD d´activité physique ou pour une autre activité physique que j´aimerais faire.'];
IF (P5.SUMSOL EQ 604) [P5.SOL = 'Je vais aller marcher avec ma famille, des amis ou des collègues.'];
IF (P5.SUMSOL EQ 701) [P5.SOL = 'Je vais choisir une activité physique simple comme la marche ou la bicyclette.'];
IF (P5.SUMSOL EQ 702) [P5.SOL = 'Je vais aller faire de l´activité physique avec des gens qui m´acceptent comme je suis, qui évitent de me juger.'];
IF (P5.SUMSOL EQ 703) [P5.SOL = 'Au départ, je vais faire de l´activité physique dans des endroits où je suis seul[LETTRE_E] pour prendre confiance.'];
IF (P5.SUMSOL EQ 704) [P5.SOL = 'Je vais éviter des endroits qui me gêne davantage comme les centres de conditionnement.'];
IF (P5.SUMSOL EQ 801) [P5.SOL = 'Je vais consulter mon médecin pour mettre au clair si je peux faire de l´activité physique.'];
IF (P5.SUMSOL EQ 802) [P5.SOL = 'Je vais consulter un spécialiste de l´activité physique pour qu´il me rassure et me conseille sur ce qui est sécuritaire pour moi.'];
IF (P5.SUMSOL EQ 803) [P5.SOL = 'Je vais commencer à petite dose. 10 à 20 minutes maximum d´activités physiques les jours où je suis actif, active.'];
IF (P5.SUMSOL EQ 804) [P5.SOL = 'Je vais faire de l´activité physique accompagnéE d´un spécialiste de l´activité physique.'];

IF (P5.SUMSOL EQ 805) [P5.SOL = 'Je vais faire de l´activité physique dans un groupe où un spécialiste de l´activité physique est présent.'];
IF (P5.SUMSOL EQ 901) [P5.SOL = 'Je vais signer un contrat papier personnel comme quoi je m´engage à être actif, active.'];
IF (P5.SUMSOL EQ 902) [P5.SOL = 'Je vais signer un contrat papier avec mes proches comme quoi je m´engage à être actif, active.'];
IF (P5.SUMSOL EQ 903) [P5.SOL = 'Je vais essayer de nouvelles activités physiques pour trouver celles qui me plaisent vraiment.'];
IF (P5.SUMSOL EQ 904) [P5.SOL = 'À chaque activité physique que je fais, je prends un temps pour me rappeler ce que cela va m´apporter de mieux dans ma vie.'];
IF (P5.SUMSOL EQ 905) [P5.SOL = 'Je vais faire de l´activité physique avec des gens que j´aime pour me motiver.'];
IF ((P5.SUMSOL EQ 999) AND (P5.P8QSOLOPEN NE 0)) [P5.SOL = P5.P8QSOLOPEN];
IF (P5.SUMSOL EQ 0) [P5.SOL = P5.P8QSOL_10];
PLAN DE LA semaine 6
formules p6
IF ((P6.QACTI12 EQ 112) AND (P6.QAOPEN NE 0)) [P6.QACTIVITE_12 = 112];
[P6.2SUMAP2 = P6.QACTIVITE1];
[P6.3SUMAP2 = P6.QACTIVITE1+P6.QACTIVITE2];
[P6.4SUMAP2 = P6.QACTIVITE1+P6.QACTIVITE2+P6.QACTIVITE3];
[P6.5SUMAP2 = P6.QACTIVITE1+P6.QACTIVITE2+P6.QACTIVITE3+P6.QACTIVITE4];
[P6.6SUMAP2 = P6.QACTIVITE1+P6.QACTIVITE2+P6.QACTIVITE3+P6.QACTIVITE4+P6.QACTIVITE5];
[P6.7SUMAP2 =
P6.QACTIVITE1+P6.QACTIVITE2+P6.QACTIVITE3+P6.QACTIVITE4+P6.QACTIVITE5+P6.QACTIVITE6];
[P6.8SUMAP2 =
P6.QACTIVITE1+P6.QACTIVITE2+P6.QACTIVITE3+P6.QACTIVITE4+P6.QACTIVITE5+P6.QACTIVITE6+P6.QACTIVITE7];
[P6.9SUMAP2 =
P6.QACTIVITE1+P6.QACTIVITE2+P6.QACTIVITE3+P6.QACTIVITE4+P6.QACTIVITE5+P6.QACTIVITE6+P6.QACTIVITE7+P6.QACTIVITE8];
[P6.10SUMAP2 =
P6.QACTIVITE1+P6.QACTIVITE2+P6.QACTIVITE3+P6.QACTIVITE4+P6.QACTIVITE5+P6.QACTIVITE6+P6.QACTIVITE7+P6.QACTIVITE8+P6.QACTIVITE9];
[P6.11SUMAP2 =
P6.QACTIVITE1+P6.QACTIVITE2+P6.QACTIVITE3+P6.QACTIVITE4+P6.QACTIVITE5+P6.QACTIVITE6+P6.QACTIVITE7+P6.QACTIVITE8+P6.QACTIVITE9+P6.QACTIVITE_10];
[P6.12SUMAP2 =
P6.QACTIVITE1+P6.QACTIVITE2+P6.QACTIVITE3+P6.QACTIVITE4+P6.QACTIVITE5+P6.QACTIVITE6+P6.QACTIVITE7+P6.QACTIVITE8+P6.QACTIVITE9+P6.QACTIVITE_10+P6.QACTIVITE_11];
[P6.3SUMAP3 = P6.QACTIVITE1+P6.QACTIVITE2];
[P6.4SUMAP3 = P6.QACTIVITE1+P6.QACTIVITE2+P6.QACTIVITE3];
[P6.5SUMAP3 = P6.QACTIVITE1+P6.QACTIVITE2+P6.QACTIVITE3+P6.QACTIVITE4];

[P6.6SUMAP3 = P6.QACTIVITE1+P6.QACTIVITE2+P6.QACTIVITE3+P6.QACTIVITE4+P6.QACTIVITE5];
[P6.7SUMAP3 =
P6.QACTIVITE1+P6.QACTIVITE2+P6.QACTIVITE3+P6.QACTIVITE4+P6.QACTIVITE5+P6.QACTIVITE6];
[P6.8SUMAP3 =
P6.QACTIVITE1+P6.QACTIVITE2+P6.QACTIVITE3+P6.QACTIVITE4+P6.QACTIVITE5+P6.QACTIVITE6+P6.QACTI
VITE7];
[P6.9SUMAP3 =
P6.QACTIVITE1+P6.QACTIVITE2+P6.QACTIVITE3+P6.QACTIVITE4+P6.QACTIVITE5+P6.QACTIVITE6+P6.QACTI
VITE7+P6.QACTIVITE8];
[P6.10SUMAP3 =
P6.QACTIVITE1+P6.QACTIVITE2+P6.QACTIVITE3+P6.QACTIVITE4+P6.QACTIVITE5+P6.QACTIVITE6+P6.QACTI
VITE7+P6.QACTIVITE8+P6.QACTIVITE9];
[P6.11SUMAP3 =
P6.QACTIVITE1+P6.QACTIVITE2+P6.QACTIVITE3+P6.QACTIVITE4+P6.QACTIVITE5+P6.QACTIVITE6+P6.QACTI
VITE7+P6.QACTIVITE8+P6.QACTIVITE9+P6.QACTIVITE_10];
[P6.12SUMAP3 =
P6.QACTIVITE1+P6.QACTIVITE2+P6.QACTIVITE3+P6.QACTIVITE4+P6.QACTIVITE5+P6.QACTIVITE6+P6.QACTI
VITE7+P6.QACTIVITE8+P6.QACTIVITE9+P6.QACTIVITE_10+P6.QACTIVITE_11];
[P6.SUMAP =
P6.QACTIVITE1+P6.QACTIVITE2+P6.QACTIVITE3+P6.QACTIVITE4+P6.QACTIVITE5+P6.QACTIVITE6+P6.QACTI
VITE7+P6.QACTIVITE8+P6.QACTIVITE9+P6.QACTIVITE_10+P6.QACTIVITE_11+P6.QACTIVITE_12];
[P6.SUMSOL =
P6.P8QSOL1+P6.P8QSOL2+P6.P8QSOL3+P6.P8QSOL4+P6.P8QSOL5+P6.P8QSOL6+P6.P8QSOL7+P6.P8QSOL8
+P6.P8QSOL9];
IF (P6.QACTIVITE1 EQ 101) [P6.AP1 = 'marche rapide'];
IF ((P6.QACTIVITE1 NE 101) AND (P6.QACTIVITE2 EQ 102)) [P6.AP1 = 'raquettes à neige'];
IF ((P6.QACTIVITE1 NE 101) AND (P6.QACTIVITE2 NE 102) AND (P6.QACTIVITE3 EQ 103)) [P6.AP1 = 'vélo'];
IF ((P6.QACTIVITE1 NE 101) AND (P6.QACTIVITE2 NE 102) AND (P6.QACTIVITE3 NE 103) AND
(P6.QACTIVITE4 EQ 104)) [P6.AP1 = 'natation'];
IF ((P6.QACTIVITE1 NE 101) AND (P6.QACTIVITE2 NE 102) AND (P6.QACTIVITE3 NE 103) AND
(P6.QACTIVITE4 NE 104) AND (P6.QACTIVITE5 EQ 105)) [P6.AP1 = 'ski de fond'];
IF ((P6.QACTIVITE1 NE 101) AND (P6.QACTIVITE2 NE 102) AND (P6.QACTIVITE3 NE 103) AND
(P6.QACTIVITE4 NE 104) AND (P6.QACTIVITE5 NE 105) AND (P6.QACTIVITE6 EQ 106)) [P6.AP1 = 'tennis'];
IF ((P6.QACTIVITE1 NE 101) AND (P6.QACTIVITE2 NE 102) AND (P6.QACTIVITE3 NE 103) AND
(P6.QACTIVITE4 NE 104) AND (P6.QACTIVITE5 NE 105) AND (P6.QACTIVITE6 NE 106) AND (P6.QACTIVITE7
EQ 107)) [P6.AP1 = 'course à pieds'];
IF ((P6.QACTIVITE1 NE 101) AND (P6.QACTIVITE2 NE 102) AND (P6.QACTIVITE3 NE 103) AND
(P6.QACTIVITE4 NE 104) AND (P6.QACTIVITE5 NE 105) AND (P6.QACTIVITE6 NE 106) AND (P6.QACTIVITE7
NE 107) AND (P6.QACTIVITE8 EQ 108)) [P6.AP1 = 'hockey'];
IF ((P6.QACTIVITE1 NE 101) AND (P6.QACTIVITE2 NE 102) AND (P6.QACTIVITE3 NE 103) AND
(P6.QACTIVITE4 NE 104) AND (P6.QACTIVITE5 NE 105) AND (P6.QACTIVITE6 NE 106) AND (P6.QACTIVITE7
NE 107) AND (P6.QACTIVITE8 NE 108) AND (P6.QACTIVITE9 EQ 109)) [P6.AP1 = 'basketball'];

IF ((P6.QACTIVITE1 NE 101) AND (P6.QACTIVITE2 NE 102) AND (P6.QACTIVITE3 NE 103) AND (P6.QACTIVITE4 NE 104) AND (P6.QACTIVITE5 NE 105) AND (P6.QACTIVITE6 NE 106) AND (P6.QACTIVITE7 NE 107) AND (P6.QACTIVITE8 NE 108) AND (P6.QACTIVITE9 NE 109) AND (P6.QACTIVITE_10 EQ 110)) [P6.AP1 = 'soccer'];
IF ((P6.QACTIVITE1 NE 101) AND (P6.QACTIVITE2 NE 102) AND (P6.QACTIVITE3 NE 103) AND (P6.QACTIVITE4 NE 104) AND (P6.QACTIVITE5 NE 105) AND (P6.QACTIVITE6 NE 106) AND (P6.QACTIVITE7 NE 107) AND (P6.QACTIVITE8 NE 108) AND (P6.QACTIVITE9 NE 109) AND (P6.QACTIVITE_10 NE 110) AND (P6.QACTIVITE_11 EQ 111)) [P6.AP1 = 'centre de conditionnement'];
IF ((P6.QACTIVITE1 NE 101) AND (P6.QACTIVITE2 NE 102) AND (P6.QACTIVITE3 NE 103) AND (P6.QACTIVITE4 NE 104) AND (P6.QACTIVITE5 NE 105) AND (P6.QACTIVITE6 NE 106) AND (P6.QACTIVITE7 NE 107) AND (P6.QACTIVITE8 NE 108) AND (P6.QACTIVITE9 NE 109) AND (P6.QACTIVITE_10 NE 110) AND (P6.QACTIVITE_11 NE 111) AND (P6.QACTIVITE_12 EQ 112)) [P6.AP1 = P6.QAOPEN];
IF ((P6.2SUMAP2 GE 100) AND (P6.2SUMAP2 LT 200) AND (P6.QACTIVITE2 EQ 102)) [P6.AP2 = 'raquettes à neige'];
IF ((P6.3SUMAP2 GE 100) AND (P6.3SUMAP2 LT 200) AND (P6.QACTIVITE3 EQ 103)) [P6.AP2 = 'vélo'];
IF ((P6.4SUMAP2 GE 100) AND (P6.4SUMAP2 LT 200) AND (P6.QACTIVITE4 EQ 104)) [P6.AP2 = 'natation'];
IF ((P6.5SUMAP2 GE 100) AND (P6.5SUMAP2 LT 200) AND (P6.QACTIVITE5 EQ 105)) [P6.AP2 = 'ski de fond'];
IF ((P6.6SUMAP2 GE 100) AND (P6.6SUMAP2 LT 200) AND (P6.QACTIVITE6 EQ 106)) [P6.AP2 = 'tennis'];
IF ((P6.7SUMAP2 GE 100) AND (P6.7SUMAP2 LT 200) AND (P6.QACTIVITE7 EQ 107)) [P6.AP2 = 'course à pieds'];
IF ((P6.8SUMAP2 GE 100) AND (P6.8SUMAP2 LT 200) AND (P6.QACTIVITE8 EQ 108)) [P6.AP2 = 'hockey'];
IF ((P6.9SUMAP2 GE 100) AND (P6.9SUMAP2 LT 200) AND (P6.QACTIVITE9 EQ 109)) [P6.AP2 = 'basketball'];
IF ((P6.10SUMAP2 GE 100) AND (P6.10SUMAP2 LT 200) AND (P6.QACTIVITE_10 EQ 110)) [P6.AP2 = 'soccer'];
IF ((P6.11SUMAP2 GE 100) AND (P6.11SUMAP2 LT 200) AND (P6.QACTIVITE_11 EQ 111)) [P6.AP2 = 'centre de conditionnement'];
IF ((P6.12SUMAP2 GE 100) AND (P6.12SUMAP2 LT 200) AND (P6.QACTIVITE_12 EQ 112)) [P6.AP2 = P6.QAOPEN];
IF ((P6.3SUMAP3 GE 200) AND (P6.3SUMAP3 LT 300) AND (P6.QACTIVITE3 EQ 103)) [P6.AP3 = 'vélo'];
IF ((P6.4SUMAP3 GE 200) AND (P6.4SUMAP3 LT 300) AND (P6.QACTIVITE4 EQ 104)) [P6.AP3 = 'natation'];
IF ((P6.5SUMAP3 GE 200) AND (P6.5SUMAP3 LT 300) AND (P6.QACTIVITE5 EQ 105)) [P6.AP3 = 'ski de fond'];
IF ((P6.6SUMAP3 GE 200) AND (P6.6SUMAP3 LT 300) AND (P6.QACTIVITE6 EQ 106)) [P6.AP3 = 'tennis'];
IF ((P6.7SUMAP3 GE 200) AND (P6.7SUMAP3 LT 300) AND (P6.QACTIVITE7 EQ 107)) [P6.AP3 = 'course à pieds'];
IF ((P6.8SUMAP3 GE 200) AND (P6.8SUMAP3 LT 300) AND (P6.QACTIVITE8 EQ 108)) [P6.AP3 = 'hockey'];
IF ((P6.9SUMAP3 GE 200) AND (P6.9SUMAP3 LT 300) AND (P6.QACTIVITE9 EQ 109)) [P6.AP3 = 'basketball'];
IF ((P6.10SUMAP3 GE 200) AND (P6.10SUMAP3 LT 300) AND (P6.QACTIVITE_10 EQ 110)) [P6.AP3 = 'soccer'];
IF ((P6.11SUMAP3 GE 200) AND (P6.11SUMAP3 LT 300) AND (P6.QACTIVITE_11 EQ 111)) [P6.AP3 = 'centre de conditionnement'];
IF ((P6.12SUMAP3 GE 200) AND (P6.12SUMAP3 LT 300) AND (P6.QACTIVITE_12 EQ 112)) [P6.AP3 = P6.QAOPEN];

ACTIVITÉ 1 DANS LE PLAN POUR CHAQUE JOUR DE LA SEMAINE
IF ((P6.QACTIVITE1 EQ 101) AND (P6.AP1JOUR EQ 1)) [P6.LUNDIAP1 = P6.AP1];
IF ((P6.QACTIVITE1 NE 101) AND (P6.QACTIVITE2 EQ 102) AND (P6.AP2JOUR EQ 1)) [P6.LUNDIAP1 = P6.AP1];
IF ((P6.QACTIVITE1 NE 101) AND (P6.QACTIVITE2 NE 102) AND (P6.QACTIVITE3 EQ 103) AND (P6.AP3JOUR EQ 1)) [P6.LUNDIAP1 = P6.AP1];
IF ((P6.QACTIVITE1 NE 101) AND (P6.QACTIVITE2 NE 102) AND (P6.QACTIVITE3 NE 103) AND (P6.QACTIVITE4 EQ 104) AND (P6.AP4JOUR EQ 1)) [P6.LUNDIAP1 = P6.AP1];
IF ((P6.QACTIVITE1 NE 101) AND (P6.QACTIVITE2 NE 102) AND (P6.QACTIVITE3 NE 103) AND (P6.QACTIVITE4 NE 104) AND (P6.QACTIVITE5 EQ 105) AND (P6.AP5JOUR EQ 1)) [P6.LUNDIAP1 = P6.AP1];
IF ((P6.QACTIVITE1 NE 101) AND (P6.QACTIVITE2 NE 102) AND (P6.QACTIVITE3 NE 103) AND (P6.QACTIVITE4 NE 104) AND (P6.QACTIVITE5 NE 105) AND (P6.QACTIVITE6 EQ 106) AND (P6.AP6JOUR EQ 1)) [P6.LUNDIAP1 = P6.AP1];
IF ((P6.QACTIVITE1 NE 101) AND (P6.QACTIVITE2 NE 102) AND (P6.QACTIVITE3 NE 103) AND (P6.QACTIVITE4 NE 104) AND (P6.QACTIVITE5 NE 105) AND (P6.QACTIVITE6 NE 106) AND (P6.QACTIVITE7 EQ 107) AND (P6.AP7JOUR EQ 1)) [P6.LUNDIAP1 = P6.AP1];
IF ((P6.QACTIVITE1 NE 101) AND (P6.QACTIVITE2 NE 102) AND (P6.QACTIVITE3 NE 103) AND (P6.QACTIVITE4 NE 104) AND (P6.QACTIVITE5 NE 105) AND (P6.QACTIVITE6 NE 106) AND (P6.QACTIVITE7 NE 107) AND (P6.QACTIVITE8 EQ 108) AND (P6.AP8JOUR EQ 1)) [P6.LUNDIAP1 = P6.AP1];
IF ((P6.QACTIVITE1 NE 101) AND (P6.QACTIVITE2 NE 102) AND (P6.QACTIVITE3 NE 103) AND (P6.QACTIVITE4 NE 104) AND (P6.QACTIVITE5 NE 105) AND (P6.QACTIVITE6 NE 106) AND (P6.QACTIVITE7 NE 107) AND (P6.QACTIVITE8 NE 108) AND (P6.QACTIVITE9 EQ 109) AND (P6.AP9JOUR EQ 1)) [P6.LUNDIAP1 = P6.AP1];
IF ((P6.QACTIVITE1 NE 101) AND (P6.QACTIVITE2 NE 102) AND (P6.QACTIVITE3 NE 103) AND (P6.QACTIVITE4 NE 104) AND (P6.QACTIVITE5 NE 105) AND (P6.QACTIVITE6 NE 106) AND (P6.QACTIVITE7 NE 107) AND (P6.QACTIVITE8 NE 108) AND (P6.QACTIVITE9 NE 109) AND (P6.QACTIVITE_10 EQ 110) AND (P6.AP10JOUR EQ 1)) [P6.LUNDIAP1 = P6.AP1];
IF ((P6.QACTIVITE1 NE 101) AND (P6.QACTIVITE2 NE 102) AND (P6.QACTIVITE3 NE 103) AND (P6.QACTIVITE4 NE 104) AND (P6.QACTIVITE5 NE 105) AND (P6.QACTIVITE6 NE 106) AND (P6.QACTIVITE7 NE 107) AND (P6.QACTIVITE8 NE 108) AND (P6.QACTIVITE9 NE 109) AND (P6.QACTIVITE_10 NE 110) AND (P6.QACTIVITE_11 EQ 111) AND (P6.AP11JOUR EQ 1)) [P6.LUNDIAP1 = P6.AP1];
IF ((P6.QACTIVITE1 NE 101) AND (P6.QACTIVITE2 NE 102) AND (P6.QACTIVITE3 NE 103) AND (P6.QACTIVITE4 NE 104) AND (P6.QACTIVITE5 NE 105) AND (P6.QACTIVITE6 NE 106) AND (P6.QACTIVITE7 NE 107) AND (P6.QACTIVITE8 NE 108) AND (P6.QACTIVITE9 NE 109) AND (P6.QACTIVITE_10 NE 110) AND (P6.QACTIVITE_11 NE 111) AND (P6.QACTIVITE_12 EQ 112) AND (P6.AP12JOUR EQ 1)) [P6.LUNDIAP1 = P6.AP1];
IF ((P6.QACTIVITE1 EQ 101) AND (P6.AP1JOUR EQ 2)) [P6.MARDIAP1 = P6.AP1];
IF ((P6.QACTIVITE1 NE 101) AND (P6.QACTIVITE2 EQ 102) AND (P6.AP2JOUR EQ 2)) [P6.MARDIAP1 = P6.AP1];
IF ((P6.QACTIVITE1 NE 101) AND (P6.QACTIVITE2 NE 102) AND (P6.QACTIVITE3 EQ 103) AND (P6.AP3JOUR EQ 2)) [P6.MARDIAP1 = P6.AP1];

IF ((P6.QACTIVITE1 NE 101) AND (P6.QACTIVITE2 NE 102) AND (P6.QACTIVITE3 NE 103) AND (P6.QACTIVITE4 EQ 104) AND (P6.AP4JOUR EQ 2)) [P6.MARDIAP1 = P6.AP1];
IF ((P6.QACTIVITE1 NE 101) AND (P6.QACTIVITE2 NE 102) AND (P6.QACTIVITE3 NE 103) AND (P6.QACTIVITE4 NE 104) AND (P6.QACTIVITE5 EQ 105) AND (P6.AP5JOUR EQ 2)) [P6.MARDIAP1 = P6.AP1];
IF ((P6.QACTIVITE1 NE 101) AND (P6.QACTIVITE2 NE 102) AND (P6.QACTIVITE3 NE 103) AND (P6.QACTIVITE4 NE 104) AND (P6.QACTIVITE5 NE 105) AND (P6.QACTIVITE6 EQ 106) AND (P6.AP6JOUR EQ 2)) [P6.MARDIAP1 = P6.AP1];
IF ((P6.QACTIVITE1 NE 101) AND (P6.QACTIVITE2 NE 102) AND (P6.QACTIVITE3 NE 103) AND (P6.QACTIVITE4 NE 104) AND (P6.QACTIVITE5 NE 105) AND (P6.QACTIVITE6 NE 106) AND (P6.QACTIVITE7 EQ 107) AND (P6.AP7JOUR EQ 2)) [P6.MARDIAP1 = P6.AP1];
IF ((P6.QACTIVITE1 NE 101) AND (P6.QACTIVITE2 NE 102) AND (P6.QACTIVITE3 NE 103) AND (P6.QACTIVITE4 NE 104) AND (P6.QACTIVITE5 NE 105) AND (P6.QACTIVITE6 NE 106) AND (P6.QACTIVITE7 NE 107) AND (P6.QACTIVITE8 EQ 108) AND (P6.AP8JOUR EQ 2)) [P6.MARDIAP1 = P6.AP1];
IF ((P6.QACTIVITE1 NE 101) AND (P6.QACTIVITE2 NE 102) AND (P6.QACTIVITE3 NE 103) AND (P6.QACTIVITE4 NE 104) AND (P6.QACTIVITE5 NE 105) AND (P6.QACTIVITE6 NE 106) AND (P6.QACTIVITE7 NE 107) AND (P6.QACTIVITE8 NE 108) AND (P6.QACTIVITE9 EQ 109) AND (P6.AP9JOUR EQ 2)) [P6.MARDIAP1 = P6.AP1];
IF ((P6.QACTIVITE1 NE 101) AND (P6.QACTIVITE2 NE 102) AND (P6.QACTIVITE3 NE 103) AND (P6.QACTIVITE4 NE 104) AND (P6.QACTIVITE5 NE 105) AND (P6.QACTIVITE6 NE 106) AND (P6.QACTIVITE7 NE 107) AND (P6.QACTIVITE8 NE 108) AND (P6.QACTIVITE9 NE 109) AND (P6.QACTIVITE_10 EQ 110) AND (P6.AP10JOUR EQ 2)) [P6.MARDIAP1 = P6.AP1];
IF ((P6.QACTIVITE1 NE 101) AND (P6.QACTIVITE2 NE 102) AND (P6.QACTIVITE3 NE 103) AND (P6.QACTIVITE4 NE 104) AND (P6.QACTIVITE5 NE 105) AND (P6.QACTIVITE6 NE 106) AND (P6.QACTIVITE7 NE 107) AND (P6.QACTIVITE8 NE 108) AND (P6.QACTIVITE9 NE 109) AND (P6.QACTIVITE_10 NE 110) AND (P6.QACTIVITE_11 EQ 111) AND (P6.AP11JOUR EQ 2)) [P6.MARDIAP1 = P6.AP1];
IF ((P6.QACTIVITE1 NE 101) AND (P6.QACTIVITE2 NE 102) AND (P6.QACTIVITE3 NE 103) AND (P6.QACTIVITE4 NE 104) AND (P6.QACTIVITE5 NE 105) AND (P6.QACTIVITE6 NE 106) AND (P6.QACTIVITE7 NE 107) AND (P6.QACTIVITE8 NE 108) AND (P6.QACTIVITE9 NE 109) AND (P6.QACTIVITE_10 NE 110) AND (P6.QACTIVITE_11 NE 111) AND (P6.QACTIVITE_12 EQ 112) AND (P6.AP12JOUR EQ 2)) [P6.MARDIAP1 = P6.AP1];
IF ((P6.QACTIVITE1 EQ 101) AND (P6.AP1JOUR EQ 3)) [P6.MERCREDIAP1 = P6.AP1];
IF ((P6.QACTIVITE1 NE 101) AND (P6.QACTIVITE2 EQ 102) AND (P6.AP2JOUR EQ 3)) [P6.MERCREDIAP1 = P6.AP1];
IF ((P6.QACTIVITE1 NE 101) AND (P6.QACTIVITE2 NE 102) AND (P6.QACTIVITE3 EQ 103) AND (P6.AP3JOUR EQ 3)) [P6.MERCREDIAP1 = P6.AP1];
IF ((P6.QACTIVITE1 NE 101) AND (P6.QACTIVITE2 NE 102) AND (P6.QACTIVITE3 NE 103) AND (P6.QACTIVITE4 EQ 104) AND (P6.AP4JOUR EQ 3)) [P6.MERCREDIAP1 = P6.AP1];
IF ((P6.QACTIVITE1 NE 101) AND (P6.QACTIVITE2 NE 102) AND (P6.QACTIVITE3 NE 103) AND (P6.QACTIVITE4 NE 104) AND (P6.QACTIVITE5 EQ 105) AND (P6.AP5JOUR EQ 3)) [P6.MERCREDIAP1 = P6.AP1];

IF ((P6.QACTIVITE1 NE 101) AND (P6.QACTIVITE2 NE 102) AND (P6.QACTIVITE3 NE 103) AND (P6.QACTIVITE4 NE 104) AND (P6.QACTIVITES5 NE 105) AND (P6.QACTIVITE6 EQ 106) AND (P6.AP6JOUR EQ 3)) [P6.MERCREDIAP1 = P6.AP1];
IF ((P6.QACTIVITE1 NE 101) AND (P6.QACTIVITE2 NE 102) AND (P6.QACTIVITE3 NE 103) AND (P6.QACTIVITE4 NE 104) AND (P6.QACTIVITES5 NE 105) AND (P6.QACTIVITE6 NE 106) AND (P6.QACTIVITE7 EQ 107) AND (P6.AP7JOUR EQ 3)) [P6.MERCREDIAP1 = P6.AP1];
IF ((P6.QACTIVITE1 NE 101) AND (P6.QACTIVITE2 NE 102) AND (P6.QACTIVITE3 NE 103) AND (P6.QACTIVITE4 NE 104) AND (P6.QACTIVITES5 NE 105) AND (P6.QACTIVITE6 NE 106) AND (P6.QACTIVITE7 NE 107) AND (P6.QACTIVITE8 EQ 108) AND (P6.AP8JOUR EQ 3)) [P6.MERCREDIAP1 = P6.AP1];
IF ((P6.QACTIVITE1 NE 101) AND (P6.QACTIVITE2 NE 102) AND (P6.QACTIVITE3 NE 103) AND (P6.QACTIVITE4 NE 104) AND (P6.QACTIVITES5 NE 105) AND (P6.QACTIVITE6 NE 106) AND (P6.QACTIVITE7 NE 107) AND (P6.QACTIVITE8 NE 108) AND (P6.QACTIVITE9 EQ 109) AND (P6.AP9JOUR EQ 3)) [P6.MERCREDIAP1 = P6.AP1];
IF ((P6.QACTIVITE1 NE 101) AND (P6.QACTIVITE2 NE 102) AND (P6.QACTIVITE3 NE 103) AND (P6.QACTIVITE4 NE 104) AND (P6.QACTIVITES5 NE 105) AND (P6.QACTIVITE6 NE 106) AND (P6.QACTIVITE7 NE 107) AND (P6.QACTIVITE8 NE 108) AND (P6.QACTIVITE9 NE 109) AND (P6.QACTIVITE_10 EQ 110) AND (P6.AP10JOUR EQ 3)) [P6.MERCREDIAP1 = P6.AP1];
IF ((P6.QACTIVITE1 NE 101) AND (P6.QACTIVITE2 NE 102) AND (P6.QACTIVITE3 NE 103) AND (P6.QACTIVITE4 NE 104) AND (P6.QACTIVITES5 NE 105) AND (P6.QACTIVITE6 NE 106) AND (P6.QACTIVITE7 NE 107) AND (P6.QACTIVITE8 NE 108) AND (P6.QACTIVITE9 NE 109) AND (P6.QACTIVITE_10 NE 110) AND (P6.QACTIVITE_11 EQ 111) AND (P6.AP11JOUR EQ 3)) [P6.MERCREDIAP1 = P6.AP1];
IF ((P6.QACTIVITE1 NE 101) AND (P6.QACTIVITE2 NE 102) AND (P6.QACTIVITE3 NE 103) AND (P6.QACTIVITE4 NE 104) AND (P6.QACTIVITES5 NE 105) AND (P6.QACTIVITE6 NE 106) AND (P6.QACTIVITE7 NE 107) AND (P6.QACTIVITE8 NE 108) AND (P6.QACTIVITE9 NE 109) AND (P6.QACTIVITE_10 NE 110) AND (P6.QACTIVITE_11 NE 111) AND (P6.QACTIVITE_12 EQ 112) AND (P6.AP12JOUR EQ 3)) [P6.MERCREDIAP1 = P6.AP1];
IF ((P6.QACTIVITE1 EQ 101) AND (P6.AP1JOUR EQ 4)) [P6.JEUDIAP1 = P6.AP1];
IF ((P6.QACTIVITE1 NE 101) AND (P6.QACTIVITE2 EQ 102) AND (P6.AP2JOUR EQ 4)) [P6.JEUDIAP1 = P6.AP1];
IF ((P6.QACTIVITE1 NE 101) AND (P6.QACTIVITE2 NE 102) AND (P6.QACTIVITE3 EQ 103) AND (P6.AP3JOUR EQ 4)) [P6.JEUDIAP1 = P6.AP1];
IF ((P6.QACTIVITE1 NE 101) AND (P6.QACTIVITE2 NE 102) AND (P6.QACTIVITE3 NE 103) AND (P6.QACTIVITE4 EQ 104) AND (P6.AP4JOUR EQ 4)) [P6.JEUDIAP1 = P6.AP1];
IF ((P6.QACTIVITE1 NE 101) AND (P6.QACTIVITE2 NE 102) AND (P6.QACTIVITE3 NE 103) AND (P6.QACTIVITE4 NE 104) AND (P6.QACTIVITES5 EQ 105) AND (P6.AP5JOUR EQ 4)) [P6.JEUDIAP1 = P6.AP1];
IF ((P6.QACTIVITE1 NE 101) AND (P6.QACTIVITE2 NE 102) AND (P6.QACTIVITE3 NE 103) AND (P6.QACTIVITE4 NE 104) AND (P6.QACTIVITES5 NE 105) AND (P6.QACTIVITE6 EQ 106) AND (P6.AP6JOUR EQ 4)) [P6.JEUDIAP1 = P6.AP1];
IF ((P6.QACTIVITE1 NE 101) AND (P6.QACTIVITE2 NE 102) AND (P6.QACTIVITE3 NE 103) AND (P6.QACTIVITE4 NE 104) AND (P6.QACTIVITES5 NE 105) AND (P6.QACTIVITE6 NE 106) AND (P6.QACTIVITE7 EQ 107) AND (P6.AP7JOUR EQ 4)) [P6.JEUDIAP1 = P6.AP1];

IF ((P6.QACTIVITE1 NE 101) AND (P6.QACTIVITE2 NE 102) AND (P6.QACTIVITE3 NE 103) AND (P6.QACTIVITE4 NE 104) AND (P6.QACTIVITES5 NE 105) AND (P6.QACTIVITE6 NE 106) AND (P6.QACTIVITE7 NE 107) AND (P6.QACTIVITE8 EQ 108) AND (P6.AP8JOUR EQ 4)) [P6.JEUDIAP1 = P6.AP1];
IF ((P6.QACTIVITE1 NE 101) AND (P6.QACTIVITE2 NE 102) AND (P6.QACTIVITE3 NE 103) AND (P6.QACTIVITE4 NE 104) AND (P6.QACTIVITES5 NE 105) AND (P6.QACTIVITE6 NE 106) AND (P6.QACTIVITE7 NE 107) AND (P6.QACTIVITE8 NE 108) AND (P6.QACTIVITE9 EQ 109) AND (P6.AP9JOUR EQ 4)) [P6.JEUDIAP1 = P6.AP1];
IF ((P6.QACTIVITE1 NE 101) AND (P6.QACTIVITE2 NE 102) AND (P6.QACTIVITE3 NE 103) AND (P6.QACTIVITE4 NE 104) AND (P6.QACTIVITES5 NE 105) AND (P6.QACTIVITE6 NE 106) AND (P6.QACTIVITE7 NE 107) AND (P6.QACTIVITE8 NE 108) AND (P6.QACTIVITE9 NE 109) AND (P6.QACTIVITE_10 EQ 110) AND (P6.AP10JOUR EQ 4)) [P6.JEUDIAP1 = P6.AP1];
IF ((P6.QACTIVITE1 NE 101) AND (P6.QACTIVITE2 NE 102) AND (P6.QACTIVITE3 NE 103) AND (P6.QACTIVITE4 NE 104) AND (P6.QACTIVITES5 NE 105) AND (P6.QACTIVITE6 NE 106) AND (P6.QACTIVITE7 NE 107) AND (P6.QACTIVITE8 NE 108) AND (P6.QACTIVITE9 NE 109) AND (P6.QACTIVITE_10 NE 110) AND (P6.QACTIVITE_11 EQ 111) AND (P6.AP11JOUR EQ 4)) [P6.JEUDIAP1 = P6.AP1];
IF ((P6.QACTIVITE1 NE 101) AND (P6.QACTIVITE2 NE 102) AND (P6.QACTIVITE3 NE 103) AND (P6.QACTIVITE4 NE 104) AND (P6.QACTIVITES5 NE 105) AND (P6.QACTIVITE6 NE 106) AND (P6.QACTIVITE7 NE 107) AND (P6.QACTIVITE8 NE 108) AND (P6.QACTIVITE9 NE 109) AND (P6.QACTIVITE_10 NE 110) AND (P6.QACTIVITE_11 NE 111) AND (P6.QACTIVITE_12 EQ 112) AND (P6.AP12JOUR EQ 4)) [P6.JEUDIAP1 = P6.AP1];
IF ((P6.QACTIVITE1 EQ 101) AND (P6.AP1JOUR EQ 5)) [P6.VENDREDIAP1 = P6.AP1];
IF ((P6.QACTIVITE1 NE 101) AND (P6.QACTIVITE2 EQ 102) AND (P6.AP2JOUR EQ 5)) [P6.VENDREDIAP1 = P6.AP1];
IF ((P6.QACTIVITE1 NE 101) AND (P6.QACTIVITE2 NE 102) AND (P6.QACTIVITE3 EQ 103) AND (P6.AP3JOUR EQ 5)) [P6.VENDREDIAP1 = P6.AP1];
IF ((P6.QACTIVITE1 NE 101) AND (P6.QACTIVITE2 NE 102) AND (P6.QACTIVITE3 NE 103) AND (P6.QACTIVITE4 EQ 104) AND (P6.AP4JOUR EQ 5)) [P6.VENDREDIAP1 = P6.AP1];
IF ((P6.QACTIVITE1 NE 101) AND (P6.QACTIVITE2 NE 102) AND (P6.QACTIVITE3 NE 103) AND (P6.QACTIVITE4 NE 104) AND (P6.QACTIVITES5 EQ 105) AND (P6.AP5JOUR EQ 5)) [P6.VENDREDIAP1 = P6.AP1];
IF ((P6.QACTIVITE1 NE 101) AND (P6.QACTIVITE2 NE 102) AND (P6.QACTIVITE3 NE 103) AND (P6.QACTIVITE4 NE 104) AND (P6.QACTIVITES5 NE 105) AND (P6.QACTIVITE6 EQ 106) AND (P6.AP6JOUR EQ 5)) [P6.VENDREDIAP1 = P6.AP1];
IF ((P6.QACTIVITE1 NE 101) AND (P6.QACTIVITE2 NE 102) AND (P6.QACTIVITE3 NE 103) AND (P6.QACTIVITE4 NE 104) AND (P6.QACTIVITES5 NE 105) AND (P6.QACTIVITE6 NE 106) AND (P6.QACTIVITE7 EQ 107) AND (P6.AP7JOUR EQ 5)) [P6.VENDREDIAP1 = P6.AP1];
IF ((P6.QACTIVITE1 NE 101) AND (P6.QACTIVITE2 NE 102) AND (P6.QACTIVITE3 NE 103) AND (P6.QACTIVITE4 NE 104) AND (P6.QACTIVITES5 NE 105) AND (P6.QACTIVITE6 NE 106) AND (P6.QACTIVITE7 NE 107) AND (P6.QACTIVITE8 EQ 108) AND (P6.AP8JOUR EQ 5)) [P6.VENDREDIAP1 = P6.AP1];
IF ((P6.QACTIVITE1 NE 101) AND (P6.QACTIVITE2 NE 102) AND (P6.QACTIVITE3 NE 103) AND (P6.QACTIVITE4 NE 104) AND (P6.QACTIVITES5 NE 105) AND (P6.QACTIVITE6 NE 106) AND (P6.QACTIVITE7

NE 107) AND (P6.QACTIVITE8 NE 108) AND (P6.QACTIVITE9 EQ 109) AND (P6.AP9JOUR EQ 5))
[P6.VENDREDIAP1 = P6.AP1];
IF ((P6.QACTIVITE1 NE 101) AND (P6.QACTIVITE2 NE 102) AND (P6.QACTIVITE3 NE 103) AND
(P6.QACTIVITE4 NE 104) AND (P6.QACTIVITES5 NE 105) AND (P6.QACTIVITE6 NE 106) AND (P6.QACTIVITE7
NE 107) AND (P6.QACTIVITE8 NE 108) AND (P6.QACTIVITE9 NE 109) AND (P6.QACTIVITE_10 EQ 110) AND
(P6.AP10JOUR EQ 5)) [P6.VENDREDIAP1 = P6.AP1];
IF ((P6.QACTIVITE1 NE 101) AND (P6.QACTIVITE2 NE 102) AND (P6.QACTIVITE3 NE 103) AND
(P6.QACTIVITE4 NE 104) AND (P6.QACTIVITES5 NE 105) AND (P6.QACTIVITE6 NE 106) AND (P6.QACTIVITE7
NE 107) AND (P6.QACTIVITE8 NE 108) AND (P6.QACTIVITE9 NE 109) AND (P6.QACTIVITE_10 NE 110) AND
(P6.QACTIVITE_11 EQ 111) AND (P6.AP11JOUR EQ 5)) [P6.VENDREDIAP1 = P6.AP1];
IF ((P6.QACTIVITE1 NE 101) AND (P6.QACTIVITE2 NE 102) AND (P6.QACTIVITE3 NE 103) AND
(P6.QACTIVITE4 NE 104) AND (P6.QACTIVITES5 NE 105) AND (P6.QACTIVITE6 NE 106) AND (P6.QACTIVITE7
NE 107) AND (P6.QACTIVITE8 NE 108) AND (P6.QACTIVITE9 NE 109) AND (P6.QACTIVITE_10 NE 110) AND
(P6.QACTIVITE_11 NE 111) AND (P6.QACTIVITE_12 EQ 112) AND (P6.AP12JOUR EQ 5)) [P6.VENDREDIAP1 =
P6.AP1];
IF ((P6.QACTIVITE1 EQ 101) AND (P6.AP1JOUR EQ 6)) [P6.SAMEDIAP1 = P6.AP1];
IF ((P6.QACTIVITE1 NE 101) AND (P6.QACTIVITE2 EQ 102) AND (P6.AP2JOUR EQ 6)) [P6.SAMEDIAP1 =
P6.AP1];
IF ((P6.QACTIVITE1 NE 101) AND (P6.QACTIVITE2 NE 102) AND (P6.QACTIVITE3 EQ 103) AND (P6.AP3JOUR
EQ 6)) [P6.SAMEDIAP1 = P6.AP1];
IF ((P6.QACTIVITE1 NE 101) AND (P6.QACTIVITE2 NE 102) AND (P6.QACTIVITE3 NE 103) AND
(P6.QACTIVITE4 EQ 104) AND (P6.AP4JOUR EQ 6)) [P6.SAMEDIAP1 = P6.AP1];
IF ((P6.QACTIVITE1 NE 101) AND (P6.QACTIVITE2 NE 102) AND (P6.QACTIVITE3 NE 103) AND
(P6.QACTIVITE4 NE 104) AND (P6.QACTIVITES5 EQ 105) AND (P6.AP5JOUR EQ 6)) [P6.SAMEDIAP1 = P6.AP1];
IF ((P6.QACTIVITE1 NE 101) AND (P6.QACTIVITE2 NE 102) AND (P6.QACTIVITE3 NE 103) AND
(P6.QACTIVITE4 NE 104) AND (P6.QACTIVITES5 NE 105) AND (P6.QACTIVITE6 EQ 106) AND (P6.AP6JOUR EQ
6)) [P6.SAMEDIAP1 = P6.AP1];
IF ((P6.QACTIVITE1 NE 101) AND (P6.QACTIVITE2 NE 102) AND (P6.QACTIVITE3 NE 103) AND
(P6.QACTIVITE4 NE 104) AND (P6.QACTIVITES5 NE 105) AND (P6.QACTIVITE6 NE 106) AND (P6.QACTIVITE7
EQ 107) AND (P6.AP7JOUR EQ 6)) [P6.SAMEDIAP1 = P6.AP1];
IF ((P6.QACTIVITE1 NE 101) AND (P6.QACTIVITE2 NE 102) AND (P6.QACTIVITE3 NE 103) AND
(P6.QACTIVITE4 NE 104) AND (P6.QACTIVITES5 NE 105) AND (P6.QACTIVITE6 NE 106) AND (P6.QACTIVITE7
NE 107) AND (P6.QACTIVITE8 EQ 108) AND (P6.AP8JOUR EQ 6)) [P6.SAMEDIAP1 = P6.AP1];
IF ((P6.QACTIVITE1 NE 101) AND (P6.QACTIVITE2 NE 102) AND (P6.QACTIVITE3 NE 103) AND
(P6.QACTIVITE4 NE 104) AND (P6.QACTIVITES5 NE 105) AND (P6.QACTIVITE6 NE 106) AND (P6.QACTIVITE7
NE 107) AND (P6.QACTIVITE8 NE 108) AND (P6.QACTIVITE9 EQ 109) AND (P6.AP9JOUR EQ 6))
[P6.SAMEDIAP1 = P6.AP1];
IF ((P6.QACTIVITE1 NE 101) AND (P6.QACTIVITE2 NE 102) AND (P6.QACTIVITE3 NE 103) AND
(P6.QACTIVITE4 NE 104) AND (P6.QACTIVITES5 NE 105) AND (P6.QACTIVITE6 NE 106) AND (P6.QACTIVITE7
NE 107) AND (P6.QACTIVITE8 NE 108) AND (P6.QACTIVITE9 NE 109) AND (P6.QACTIVITE_10 EQ 110) AND
(P6.AP10JOUR EQ 6)) [P6.SAMEDIAP1 = P6.AP1];

IF ((P6.QACTIVITE1 NE 101) AND (P6.QACTIVITE2 NE 102) AND (P6.QACTIVITE3 NE 103) AND (P6.QACTIVITE4 NE 104) AND (P6.QACTIVITE5 NE 105) AND (P6.QACTIVITE6 NE 106) AND (P6.QACTIVITE7 NE 107) AND (P6.QACTIVITE8 NE 108) AND (P6.QACTIVITE9 NE 109) AND (P6.QACTIVITE_10 NE 110) AND (P6.QACTIVITE_11 EQ 111) AND (P6.AP11JOUR EQ 6)) [P6.SAMEDIAP1 = P6.AP1];
IF ((P6.QACTIVITE1 NE 101) AND (P6.QACTIVITE2 NE 102) AND (P6.QACTIVITE3 NE 103) AND (P6.QACTIVITE4 NE 104) AND (P6.QACTIVITE5 NE 105) AND (P6.QACTIVITE6 NE 106) AND (P6.QACTIVITE7 NE 107) AND (P6.QACTIVITE8 NE 108) AND (P6.QACTIVITE9 NE 109) AND (P6.QACTIVITE_10 NE 110) AND (P6.QACTIVITE_11 NE 111) AND (P6.QACTIVITE_12 EQ 112) AND (P6.AP12JOUR EQ 6)) [P6.SAMEDIAP1 = P6.AP1];
IF ((P6.QACTIVITE1 EQ 101) AND (P6.AP1JOUR EQ 7)) [P6.DIMANCHEAP1 = P6.AP1];
IF ((P6.QACTIVITE1 NE 101) AND (P6.QACTIVITE2 EQ 102) AND (P6.AP2JOUR EQ 7)) [P6.DIMANCHEAP1 = P6.AP1];
IF ((P6.QACTIVITE1 NE 101) AND (P6.QACTIVITE2 NE 102) AND (P6.QACTIVITE3 EQ 103) AND (P6.AP3JOUR EQ 7)) [P6.DIMANCHEAP1 = P6.AP1];
IF ((P6.QACTIVITE1 NE 101) AND (P6.QACTIVITE2 NE 102) AND (P6.QACTIVITE3 NE 103) AND (P6.QACTIVITE4 EQ 104) AND (P6.AP4JOUR EQ 7)) [P6.DIMANCHEAP1 = P6.AP1];
IF ((P6.QACTIVITE1 NE 101) AND (P6.QACTIVITE2 NE 102) AND (P6.QACTIVITE3 NE 103) AND (P6.QACTIVITE4 NE 104) AND (P6.QACTIVITE5 EQ 105) AND (P6.AP5JOUR EQ 7)) [P6.DIMANCHEAP1 = P6.AP1];
IF ((P6.QACTIVITE1 NE 101) AND (P6.QACTIVITE2 NE 102) AND (P6.QACTIVITE3 NE 103) AND (P6.QACTIVITE4 NE 104) AND (P6.QACTIVITE5 NE 105) AND (P6.QACTIVITE6 EQ 106) AND (P6.AP6JOUR EQ 7)) [P6.DIMANCHEAP1 = P6.AP1];
IF ((P6.QACTIVITE1 NE 101) AND (P6.QACTIVITE2 NE 102) AND (P6.QACTIVITE3 NE 103) AND (P6.QACTIVITE4 NE 104) AND (P6.QACTIVITE5 NE 105) AND (P6.QACTIVITE6 NE 106) AND (P6.QACTIVITE7 EQ 107) AND (P6.AP7JOUR EQ 7)) [P6.DIMANCHEAP1 = P6.AP1];
IF ((P6.QACTIVITE1 NE 101) AND (P6.QACTIVITE2 NE 102) AND (P6.QACTIVITE3 NE 103) AND (P6.QACTIVITE4 NE 104) AND (P6.QACTIVITE5 NE 105) AND (P6.QACTIVITE6 NE 106) AND (P6.QACTIVITE7 NE 107) AND (P6.QACTIVITE8 EQ 108) AND (P6.AP8JOUR EQ 7)) [P6.DIMANCHEAP1 = P6.AP1];
IF ((P6.QACTIVITE1 NE 101) AND (P6.QACTIVITE2 NE 102) AND (P6.QACTIVITE3 NE 103) AND (P6.QACTIVITE4 NE 104) AND (P6.QACTIVITE5 NE 105) AND (P6.QACTIVITE6 NE 106) AND (P6.QACTIVITE7 NE 107) AND (P6.QACTIVITE8 NE 108) AND (P6.QACTIVITE9 EQ 109) AND (P6.AP9JOUR EQ 7)) [P6.DIMANCHEAP1 = P6.AP1];
IF ((P6.QACTIVITE1 NE 101) AND (P6.QACTIVITE2 NE 102) AND (P6.QACTIVITE3 NE 103) AND (P6.QACTIVITE4 NE 104) AND (P6.QACTIVITE5 NE 105) AND (P6.QACTIVITE6 NE 106) AND (P6.QACTIVITE7 NE 107) AND (P6.QACTIVITE8 NE 108) AND (P6.QACTIVITE9 NE 109) AND (P6.QACTIVITE_10 EQ 110) AND (P6.AP10JOUR EQ 7)) [P6.DIMANCHEAP1 = P6.AP1];
IF ((P6.QACTIVITE1 NE 101) AND (P6.QACTIVITE2 NE 102) AND (P6.QACTIVITE3 NE 103) AND (P6.QACTIVITE4 NE 104) AND (P6.QACTIVITE5 NE 105) AND (P6.QACTIVITE6 NE 106) AND (P6.QACTIVITE7 NE 107) AND (P6.QACTIVITE8 NE 108) AND (P6.QACTIVITE9 NE 109) AND (P6.QACTIVITE_10 NE 110) AND (P6.QACTIVITE_11 EQ 111) AND (P6.AP11JOUR EQ 7)) [P6.DIMANCHEAP1 = P6.AP1];
IF ((P6.QACTIVITE1 NE 101) AND (P6.QACTIVITE2 NE 102) AND (P6.QACTIVITE3 NE 103) AND (P6.QACTIVITE4 NE 104) AND (P6.QACTIVITE5 NE 105) AND (P6.QACTIVITE6 NE 106) AND (P6.QACTIVITE7

NE 107) AND (P6.QACTIVITE8 NE 108) AND (P6.QACTIVITE9 NE 109) AND (P6.QACTIVITE_10 NE 110) AND (P6.QACTIVITE_11 NE 111) AND (P6.QACTIVITE_12 EQ 112) AND (P6.AP12JOUR EQ 7)) [P6.DIMANCHEAP1 = P6.AP1];
ACTIVITÉ 2 DANS LE PLAN POUR CHAQUE JOUR DE LA SEMAINE
IF ((P6.2SUMAP2 GE 100) AND (P6.2SUMAP2 LT 200) AND (P6.QACTIVITE2 EQ 102) AND (P6.AP2JOUR EQ 1)) [P6.LUNDIAP2 = P6.AP2];
IF ((P6.3SUMAP2 GE 100) AND (P6.3SUMAP2 LT 200) AND (P6.QACTIVITE3 EQ 103) AND (P6.AP3JOUR EQ 1)) [P6.LUNDIAP2 = P6.AP2];
IF ((P6.4SUMAP2 GE 100) AND (P6.4SUMAP2 LT 200) AND (P6.QACTIVITE4 EQ 104) AND (P6.AP4JOUR EQ 1)) [P6.LUNDIAP2 = P6.AP2];
IF ((P6.5SUMAP2 GE 100) AND (P6.5SUMAP2 LT 200) AND (P6.QACTIVITE5 EQ 105) AND (P6.AP5JOUR EQ 1)) [P6.LUNDIAP2 = P6.AP2];
IF ((P6.6SUMAP2 GE 100) AND (P6.6SUMAP2 LT 200) AND (P6.QACTIVITE6 EQ 106) AND (P6.AP6JOUR EQ 1)) [P6.LUNDIAP2 = P6.AP2];
IF ((P6.7SUMAP2 GE 100) AND (P6.7SUMAP2 LT 200) AND (P6.QACTIVITE7 EQ 107) AND (P6.AP7JOUR EQ 1)) [P6.LUNDIAP2 = P6.AP2];
IF ((P6.8SUMAP2 GE 100) AND (P6.8SUMAP2 LT 200) AND (P6.QACTIVITE8 EQ 108) AND (P6.AP8JOUR EQ 1)) [P6.LUNDIAP2 = P6.AP2];
IF ((P6.9SUMAP2 GE 100) AND (P6.9SUMAP2 LT 200) AND (P6.QACTIVITE9 EQ 109) AND (P6.AP9JOUR EQ 1)) [P6.LUNDIAP2 = P6.AP2];
IF ((P6.10SUMAP2 GE 100) AND (P6.10SUMAP2 LT 200) AND (P6.QACTIVITE_10 EQ 110) AND (P6.AP10JOUR EQ 1)) [P6.LUNDIAP2 = P6.AP2];
IF ((P6.11SUMAP2 GE 100) AND (P6.11SUMAP2 LT 200) AND (P6.QACTIVITE_11 EQ 111) AND (P6.AP11JOUR EQ 1)) [P6.LUNDIAP2 = P6.AP2];
IF ((P6.12SUMAP2 GE 100) AND (P6.12SUMAP2 LT 200) AND (P6.QACTIVITE_12 EQ 112) AND (P6.AP12JOUR EQ 1)) [P6.LUNDIAP2 = P6.AP2];
IF ((P6.2SUMAP2 GE 100) AND (P6.2SUMAP2 LT 200) AND (P6.QACTIVITE2 EQ 102) AND (P6.AP2JOUR EQ 2)) [P6.MARDIAP2 = P6.AP2];
IF ((P6.3SUMAP2 GE 100) AND (P6.3SUMAP2 LT 200) AND (P6.QACTIVITE3 EQ 103) AND (P6.AP3JOUR EQ 2)) [P6.MARDIAP2 = P6.AP2];
IF ((P6.4SUMAP2 GE 100) AND (P6.4SUMAP2 LT 200) AND (P6.QACTIVITE4 EQ 104) AND (P6.AP4JOUR EQ 2)) [P6.MARDIAP2 = P6.AP2];
IF ((P6.5SUMAP2 GE 100) AND (P6.5SUMAP2 LT 200) AND (P6.QACTIVITE5 EQ 105) AND (P6.AP5JOUR EQ 2)) [P6.MARDIAP2 = P6.AP2];
IF ((P6.6SUMAP2 GE 100) AND (P6.6SUMAP2 LT 200) AND (P6.QACTIVITE6 EQ 106) AND (P6.AP6JOUR EQ 2)) [P6.MARDIAP2 = P6.AP2];
IF ((P6.7SUMAP2 GE 100) AND (P6.7SUMAP2 LT 200) AND (P6.QACTIVITE7 EQ 107) AND (P6.AP7JOUR EQ 2)) [P6.MARDIAP2 = P6.AP2];
IF ((P6.8SUMAP2 GE 100) AND (P6.8SUMAP2 LT 200) AND (P6.QACTIVITE8 EQ 108) AND (P6.AP8JOUR EQ 2)) [P6.MARDIAP2 = P6.AP2];
IF ((P6.9SUMAP2 GE 100) AND (P6.9SUMAP2 LT 200) AND (P6.QACTIVITE9 EQ 109) AND (P6.AP9JOUR EQ 2)) [P6.MARDIAP2 = P6.AP2];

IF ((P6.10SUMAP2 GE 100) AND (P6.10SUMAP2 LT 200) AND (P6.QACTIVITE_10 EQ 110) AND (P6.AP10JOUR EQ 2)) [P6.MARDIAP2 = P6.AP2];
IF ((P6.11SUMAP2 GE 100) AND (P6.11SUMAP2 LT 200) AND (P6.QACTIVITE_11 EQ 111) AND (P6.AP11JOUR EQ 2)) [P6.MARDIAP2 = P6.AP2];
IF ((P6.12SUMAP2 GE 100) AND (P6.12SUMAP2 LT 200) AND (P6.QACTIVITE_12 EQ 112) AND (P6.AP12JOUR EQ 2)) [P6.MARDIAP2 = P6.AP2];
IF ((P6.2SUMAP2 GE 100) AND (P6.2SUMAP2 LT 200) AND (P6.QACTIVITE2 EQ 102) AND (P6.AP2JOUR EQ 3)) [P6.MERCREDIAP2 = P6.AP2];
IF ((P6.3SUMAP2 GE 100) AND (P6.3SUMAP2 LT 200) AND (P6.QACTIVITE3 EQ 103) AND (P6.AP3JOUR EQ 3)) [P6.MERCREDIAP2 = P6.AP2];
IF ((P6.4SUMAP2 GE 100) AND (P6.4SUMAP2 LT 200) AND (P6.QACTIVITE4 EQ 104) AND (P6.AP4JOUR EQ 3)) [P6.MERCREDIAP2 = P6.AP2];
IF ((P6.5SUMAP2 GE 100) AND (P6.5SUMAP2 LT 200) AND (P6.QACTIVITE5 EQ 105) AND (P6.AP5JOUR EQ 3)) [P6.MERCREDIAP2 = P6.AP2];
IF ((P6.6SUMAP2 GE 100) AND (P6.6SUMAP2 LT 200) AND (P6.QACTIVITE6 EQ 106) AND (P6.AP6JOUR EQ 3)) [P6.MERCREDIAP2 = P6.AP2];
IF ((P6.7SUMAP2 GE 100) AND (P6.7SUMAP2 LT 200) AND (P6.QACTIVITE7 EQ 107) AND (P6.AP7JOUR EQ 3)) [P6.MERCREDIAP2 = P6.AP2];
IF ((P6.8SUMAP2 GE 100) AND (P6.8SUMAP2 LT 200) AND (P6.QACTIVITE8 EQ 108) AND (P6.AP8JOUR EQ 3)) [P6.MERCREDIAP2 = P6.AP2];
IF ((P6.9SUMAP2 GE 100) AND (P6.9SUMAP2 LT 200) AND (P6.QACTIVITE9 EQ 109) AND (P6.AP9JOUR EQ 3)) [P6.MERCREDIAP2 = P6.AP2];
IF ((P6.10SUMAP2 GE 100) AND (P6.10SUMAP2 LT 200) AND (P6.QACTIVITE_10 EQ 110) AND (P6.AP10JOUR EQ 3)) [P6.MERCREDIAP2 = P6.AP2];
IF ((P6.11SUMAP2 GE 100) AND (P6.11SUMAP2 LT 200) AND (P6.QACTIVITE_11 EQ 111) AND (P6.AP11JOUR EQ 3)) [P6.MERCREDIAP2 = P6.AP2];
IF ((P6.12SUMAP2 GE 100) AND (P6.12SUMAP2 LT 200) AND (P6.QACTIVITE_12 EQ 112) AND (P6.AP12JOUR EQ 3)) [P6.MERCREDIAP2 = P6.AP2];
IF ((P6.2SUMAP2 GE 100) AND (P6.2SUMAP2 LT 200) AND (P6.QACTIVITE2 EQ 102) AND (P6.AP2JOUR EQ 4)) [P6.JEUDIAP2 = P6.AP2];
IF ((P6.3SUMAP2 GE 100) AND (P6.3SUMAP2 LT 200) AND (P6.QACTIVITE3 EQ 103) AND (P6.AP3JOUR EQ 4)) [P6.JEUDIAP2 = P6.AP2];
IF ((P6.4SUMAP2 GE 100) AND (P6.4SUMAP2 LT 200) AND (P6.QACTIVITE4 EQ 104) AND (P6.AP4JOUR EQ 4)) [P6.JEUDIAP2 = P6.AP2];
IF ((P6.5SUMAP2 GE 100) AND (P6.5SUMAP2 LT 200) AND (P6.QACTIVITE5 EQ 105) AND (P6.AP5JOUR EQ 4)) [P6.JEUDIAP2 = P6.AP2];
IF ((P6.6SUMAP2 GE 100) AND (P6.6SUMAP2 LT 200) AND (P6.QACTIVITE6 EQ 106) AND (P6.AP6JOUR EQ 4)) [P6.JEUDIAP2 = P6.AP2];
IF ((P6.7SUMAP2 GE 100) AND (P6.7SUMAP2 LT 200) AND (P6.QACTIVITE7 EQ 107) AND (P6.AP7JOUR EQ 4)) [P6.JEUDIAP2 = P6.AP2];
IF ((P6.8SUMAP2 GE 100) AND (P6.8SUMAP2 LT 200) AND (P6.QACTIVITE8 EQ 108) AND (P6.AP8JOUR EQ 4)) [P6.JEUDIAP2 = P6.AP2];

IF ((P6.9SUMAP2 GE 100) AND (P6.9SUMAP2 LT 200) AND (P6.QACTIVITE9 EQ 109) AND (P6.AP9JOUR EQ 4)) [P6.JEUDIAP2 = P6.AP2];
IF ((P6.10SUMAP2 GE 100) AND (P6.10SUMAP2 LT 200) AND (P6.QACTIVITE_10 EQ 110) AND (P6.AP10JOUR EQ 4)) [P6.JEUDIAP2 = P6.AP2];
IF ((P6.11SUMAP2 GE 100) AND (P6.11SUMAP2 LT 200) AND (P6.QACTIVITE_11 EQ 111) AND (P6.AP11JOUR EQ 4)) [P6.JEUDIAP2 = P6.AP2];
IF ((P6.12SUMAP2 GE 100) AND (P6.12SUMAP2 LT 200) AND (P6.QACTIVITE_12 EQ 112) AND (P6.AP12JOUR EQ 4)) [P6.JEUDIAP2 = P6.AP2];
IF ((P6.2SUMAP2 GE 100) AND (P6.2SUMAP2 LT 200) AND (P6.QACTIVITE2 EQ 102) AND (P6.AP2JOUR EQ 5)) [P6.VENDREDIAP2 = P6.AP2];
IF ((P6.3SUMAP2 GE 100) AND (P6.3SUMAP2 LT 200) AND (P6.QACTIVITE3 EQ 103) AND (P6.AP3JOUR EQ 5)) [P6.VENDREDIAP2 = P6.AP2];
IF ((P6.4SUMAP2 GE 100) AND (P6.4SUMAP2 LT 200) AND (P6.QACTIVITE4 EQ 104) AND (P6.AP4JOUR EQ 5)) [P6.VENDREDIAP2 = P6.AP2];
IF ((P6.5SUMAP2 GE 100) AND (P6.5SUMAP2 LT 200) AND (P6.QACTIVITE5 EQ 105) AND (P6.AP5JOUR EQ 5)) [P6.VENDREDIAP2 = P6.AP2];
IF ((P6.6SUMAP2 GE 100) AND (P6.6SUMAP2 LT 200) AND (P6.QACTIVITE6 EQ 106) AND (P6.AP6JOUR EQ 5)) [P6.VENDREDIAP2 = P6.AP2];
IF ((P6.7SUMAP2 GE 100) AND (P6.7SUMAP2 LT 200) AND (P6.QACTIVITE7 EQ 107) AND (P6.AP7JOUR EQ 5)) [P6.VENDREDIAP2 = P6.AP2];
IF ((P6.8SUMAP2 GE 100) AND (P6.8SUMAP2 LT 200) AND (P6.QACTIVITE8 EQ 108) AND (P6.AP8JOUR EQ 5)) [P6.VENDREDIAP2 = P6.AP2];
IF ((P6.9SUMAP2 GE 100) AND (P6.9SUMAP2 LT 200) AND (P6.QACTIVITE9 EQ 109) AND (P6.AP9JOUR EQ 5)) [P6.VENDREDIAP2 = P6.AP2];
IF ((P6.10SUMAP2 GE 100) AND (P6.10SUMAP2 LT 200) AND (P6.QACTIVITE_10 EQ 110) AND (P6.AP10JOUR EQ 5)) [P6.VENDREDIAP2 = P6.AP2];
IF ((P6.11SUMAP2 GE 100) AND (P6.11SUMAP2 LT 200) AND (P6.QACTIVITE_11 EQ 111) AND (P6.AP11JOUR EQ 5)) [P6.VENDREDIAP2 = P6.AP2];
IF ((P6.12SUMAP2 GE 100) AND (P6.12SUMAP2 LT 200) AND (P6.QACTIVITE_12 EQ 112) AND (P6.AP12JOUR EQ 5)) [P6.VENDREDIAP2 = P6.AP2];
IF ((P6.2SUMAP2 GE 100) AND (P6.2SUMAP2 LT 200) AND (P6.QACTIVITE2 EQ 102) AND (P6.AP2JOUR EQ 6)) [P6.SAMEDIAP2 = P6.AP2];
IF ((P6.3SUMAP2 GE 100) AND (P6.3SUMAP2 LT 200) AND (P6.QACTIVITE3 EQ 103) AND (P6.AP3JOUR EQ 6)) [P6.SAMEDIAP2 = P6.AP2];
IF ((P6.4SUMAP2 GE 100) AND (P6.4SUMAP2 LT 200) AND (P6.QACTIVITE4 EQ 104) AND (P6.AP4JOUR EQ 6)) [P6.SAMEDIAP2 = P6.AP2];
IF ((P6.5SUMAP2 GE 100) AND (P6.5SUMAP2 LT 200) AND (P6.QACTIVITE5 EQ 105) AND (P6.AP5JOUR EQ 6)) [P6.SAMEDIAP2 = P6.AP2];
IF ((P6.6SUMAP2 GE 100) AND (P6.6SUMAP2 LT 200) AND (P6.QACTIVITE6 EQ 106) AND (P6.AP6JOUR EQ 6)) [P6.SAMEDIAP2 = P6.AP2];
IF ((P6.7SUMAP2 GE 100) AND (P6.7SUMAP2 LT 200) AND (P6.QACTIVITE7 EQ 107) AND (P6.AP7JOUR EQ 6)) [P6.SAMEDIAP2 = P6.AP2];

IF ((P6.8SUMAP2 GE 100) AND (P6.8SUMAP2 LT 200) AND (P6.QACTIVITE8 EQ 108) AND (P6.AP8JOUR EQ 6)) [P6.SAMEDIAP2 = P6.AP2];
IF ((P6.9SUMAP2 GE 100) AND (P6.9SUMAP2 LT 200) AND (P6.QACTIVITE9 EQ 109) AND (P6.AP9JOUR EQ 6)) [P6.SAMEDIAP2 = P6.AP2];
IF ((P6.10SUMAP2 GE 100) AND (P6.10SUMAP2 LT 200) AND (P6.QACTIVITE_10 EQ 110) AND (P6.AP10JOUR EQ 6)) [P6.SAMEDIAP2 = P6.AP2];
IF ((P6.11SUMAP2 GE 100) AND (P6.11SUMAP2 LT 200) AND (P6.QACTIVITE_11 EQ 111) AND (P6.AP11JOUR EQ 6)) [P6.SAMEDIAP2 = P6.AP2];
IF ((P6.12SUMAP2 GE 100) AND (P6.12SUMAP2 LT 200) AND (P6.QACTIVITE_12 EQ 112) AND (P6.AP12JOUR EQ 6)) [P6.SAMEDIAP2 = P6.AP2];
IF ((P6.2SUMAP2 GE 100) AND (P6.2SUMAP2 LT 200) AND (P6.QACTIVITE2 EQ 102) AND (P6.AP2JOUR EQ 7)) [P6.DIMANCHEAP2 = P6.AP2];
IF ((P6.3SUMAP2 GE 100) AND (P6.3SUMAP2 LT 200) AND (P6.QACTIVITE3 EQ 103) AND (P6.AP3JOUR EQ 7)) [P6.DIMANCHEAP2 = P6.AP2];
IF ((P6.4SUMAP2 GE 100) AND (P6.4SUMAP2 LT 200) AND (P6.QACTIVITE4 EQ 104) AND (P6.AP4JOUR EQ 7)) [P6.DIMANCHEAP2 = P6.AP2];
IF ((P6.5SUMAP2 GE 100) AND (P6.5SUMAP2 LT 200) AND (P6.QACTIVITE5 EQ 105) AND (P6.AP5JOUR EQ 7)) [P6.DIMANCHEAP2 = P6.AP2];
IF ((P6.6SUMAP2 GE 100) AND (P6.6SUMAP2 LT 200) AND (P6.QACTIVITE6 EQ 106) AND (P6.AP6JOUR EQ 7)) [P6.DIMANCHEAP2 = P6.AP2];
IF ((P6.7SUMAP2 GE 100) AND (P6.7SUMAP2 LT 200) AND (P6.QACTIVITE7 EQ 107) AND (P6.AP7JOUR EQ 7)) [P6.DIMANCHEAP2 = P6.AP2];
IF ((P6.8SUMAP2 GE 100) AND (P6.8SUMAP2 LT 200) AND (P6.QACTIVITE8 EQ 108) AND (P6.AP8JOUR EQ 7)) [P6.DIMANCHEAP2 = P6.AP2];
IF ((P6.9SUMAP2 GE 100) AND (P6.9SUMAP2 LT 200) AND (P6.QACTIVITE9 EQ 109) AND (P6.AP9JOUR EQ 7)) [P6.DIMANCHEAP2 = P6.AP2];
IF ((P6.10SUMAP2 GE 100) AND (P6.10SUMAP2 LT 200) AND (P6.QACTIVITE_10 EQ 110) AND (P6.AP10JOUR EQ 7)) [P6.DIMANCHEAP2 = P6.AP2];
IF ((P6.11SUMAP2 GE 100) AND (P6.11SUMAP2 LT 200) AND (P6.QACTIVITE_11 EQ 111) AND (P6.AP11JOUR EQ 7)) [P6.DIMANCHEAP2 = P6.AP2];
IF ((P6.12SUMAP2 GE 100) AND (P6.12SUMAP2 LT 200) AND (P6.QACTIVITE_12 EQ 112) AND (P6.AP12JOUR EQ 7)) [P6.DIMANCHEAP2 = P6.AP2];
ACTIVITÉ 3 DANS LE PLAN POUR CHAQUE JOUR DE LA SEMAINE
IF ((P6.3SUMAP3 GE 200) AND (P6.3SUMAP3 LT 300) AND (P6.QACTIVITE3 EQ 103) AND (P6.AP3JOUR EQ 1)) [P6.LUNDIAP3 = P6.AP3];
IF ((P6.4SUMAP3 GE 200) AND (P6.4SUMAP3 LT 300) AND (P6.QACTIVITE4 EQ 104) AND (P6.AP4JOUR EQ 1)) [P6.LUNDIAP3 = P6.AP3];
IF ((P6.5SUMAP3 GE 200) AND (P6.5SUMAP3 LT 300) AND (P6.QACTIVITE5 EQ 105) AND (P6.AP5JOUR EQ 1)) [P6.LUNDIAP3 = P6.AP3];
IF ((P6.6SUMAP3 GE 200) AND (P6.6SUMAP3 LT 300) AND (P6.QACTIVITE6 EQ 106) AND (P6.AP6JOUR EQ 1)) [P6.LUNDIAP3 = P6.AP3];

IF ((P6.7SUMAP3 GE 200) AND (P6.7SUMAP3 LT 300) AND (P6.QACTIVITE7 EQ 107) AND (P6.AP7JOUR EQ 1)) [P6.LUNDIAP3 = P6.AP3];
IF ((P6.8SUMAP3 GE 200) AND (P6.8SUMAP3 LT 300) AND (P6.QACTIVITE8 EQ 108) AND (P6.AP8JOUR EQ 1)) [P6.LUNDIAP3 = P6.AP3];
IF ((P6.9SUMAP3 GE 200) AND (P6.9SUMAP3 LT 300) AND (P6.QACTIVITE9 EQ 109) AND (P6.AP9JOUR EQ 1)) [P6.LUNDIAP3 = P6.AP3];
IF ((P6.10SUMAP3 GE 200) AND (P6.10SUMAP3 LT 300) AND (P6.QACTIVITE_10 EQ 110) AND (P6.AP10JOUR EQ 1)) [P6.LUNDIAP3 = P6.AP3];
IF ((P6.11SUMAP3 GE 200) AND (P6.11SUMAP3 LT 300) AND (P6.QACTIVITE_11 EQ 111) AND (P6.AP11JOUR EQ 1)) [P6.LUNDIAP3 = P6.AP3];
IF ((P6.12SUMAP3 GE 200) AND (P6.12SUMAP3 LT 300) AND (P6.QACTIVITE_12 EQ 112) AND (P6.AP12JOUR EQ 1)) [P6.LUNDIAP3 = P6.AP3];
IF ((P6.3SUMAP3 GE 200) AND (P6.3SUMAP3 LT 300) AND (P6.QACTIVITE3 EQ 103) AND (P6.AP3JOUR EQ 2)) [P6.MARDIAP3 = P6.AP3];
IF ((P6.4SUMAP3 GE 200) AND (P6.4SUMAP3 LT 300) AND (P6.QACTIVITE4 EQ 104) AND (P6.AP4JOUR EQ 2)) [P6.MARDIAP3 = P6.AP3];
IF ((P6.5SUMAP3 GE 200) AND (P6.5SUMAP3 LT 300) AND (P6.QACTIVITE5 EQ 105) AND (P6.AP5JOUR EQ 2)) [P6.MARDIAP3 = P6.AP3];
IF ((P6.6SUMAP3 GE 200) AND (P6.6SUMAP3 LT 300) AND (P6.QACTIVITE6 EQ 106) AND (P6.AP6JOUR EQ 2)) [P6.MARDIAP3 = P6.AP3];
IF ((P6.7SUMAP3 GE 200) AND (P6.7SUMAP3 LT 300) AND (P6.QACTIVITE7 EQ 107) AND (P6.AP7JOUR EQ 2)) [P6.MARDIAP3 = P6.AP3];
IF ((P6.8SUMAP3 GE 200) AND (P6.8SUMAP3 LT 300) AND (P6.QACTIVITE8 EQ 108) AND (P6.AP8JOUR EQ 2)) [P6.MARDIAP3 = P6.AP3];
IF ((P6.9SUMAP3 GE 200) AND (P6.9SUMAP3 LT 300) AND (P6.QACTIVITE9 EQ 109) AND (P6.AP9JOUR EQ 2)) [P6.MARDIAP3 = P6.AP3];
IF ((P6.10SUMAP3 GE 200) AND (P6.10SUMAP3 LT 300) AND (P6.QACTIVITE_10 EQ 110) AND (P6.AP10JOUR EQ 2)) [P6.MARDIAP3 = P6.AP3];
IF ((P6.11SUMAP3 GE 200) AND (P6.11SUMAP3 LT 300) AND (P6.QACTIVITE_11 EQ 111) AND (P6.AP11JOUR EQ 2)) [P6.MARDIAP3 = P6.AP3];
IF ((P6.12SUMAP3 GE 200) AND (P6.12SUMAP3 LT 300) AND (P6.QACTIVITE_12 EQ 112) AND (P6.AP12JOUR EQ 2)) [P6.MARDIAP3 = P6.AP3];
IF ((P6.3SUMAP3 GE 200) AND (P6.3SUMAP3 LT 300) AND (P6.QACTIVITE3 EQ 103) AND (P6.AP3JOUR EQ 3)) [P6.MERCREDIAP3 = P6.AP3];
IF ((P6.4SUMAP3 GE 200) AND (P6.4SUMAP3 LT 300) AND (P6.QACTIVITE4 EQ 104) AND (P6.AP4JOUR EQ 3)) [P6.MERCREDIAP3 = P6.AP3];
IF ((P6.5SUMAP3 GE 200) AND (P6.5SUMAP3 LT 300) AND (P6.QACTIVITE5 EQ 105) AND (P6.AP5JOUR EQ 3)) [P6.MERCREDIAP3 = P6.AP3];
IF ((P6.6SUMAP3 GE 200) AND (P6.6SUMAP3 LT 300) AND (P6.QACTIVITE6 EQ 106) AND (P6.AP6JOUR EQ 3)) [P6.MERCREDIAP3 = P6.AP3];
IF ((P6.7SUMAP3 GE 200) AND (P6.7SUMAP3 LT 300) AND (P6.QACTIVITE7 EQ 107) AND (P6.AP7JOUR EQ 3)) [P6.MERCREDIAP3 = P6.AP3];

IF ((P6.8SUMAP3 GE 200) AND (P6.8SUMAP3 LT 300) AND (P6.QACTIVITE8 EQ 108) AND (P6.AP8JOUR EQ 3)) [P6.MERCREDIAP3 = P6.AP3];
IF ((P6.9SUMAP3 GE 200) AND (P6.9SUMAP3 LT 300) AND (P6.QACTIVITE9 EQ 109) AND (P6.AP9JOUR EQ 3)) [P6.MERCREDIAP3 = P6.AP3];
IF ((P6.10SUMAP3 GE 200) AND (P6.10SUMAP3 LT 300) AND (P6.QACTIVITE_10 EQ 110) AND (P6.AP10JOUR EQ 3)) [P6.MERCREDIAP3 = P6.AP3];
IF ((P6.11SUMAP3 GE 200) AND (P6.11SUMAP3 LT 300) AND (P6.QACTIVITE_11 EQ 111) AND (P6.AP11JOUR EQ 3)) [P6.MERCREDIAP3 = P6.AP3];
IF ((P6.12SUMAP3 GE 200) AND (P6.12SUMAP3 LT 300) AND (P6.QACTIVITE_12 EQ 112) AND (P6.AP12JOUR EQ 3)) [P6.MERCREDIAP3 = P6.AP3];
IF ((P6.3SUMAP3 GE 200) AND (P6.3SUMAP3 LT 300) AND (P6.QACTIVITE3 EQ 103) AND (P6.AP3JOUR EQ 4)) [P6.JEUDIAP3 = P6.AP3];
IF ((P6.4SUMAP3 GE 200) AND (P6.4SUMAP3 LT 300) AND (P6.QACTIVITE4 EQ 104) AND (P6.AP4JOUR EQ 4)) [P6.JEUDIAP3 = P6.AP3];
IF ((P6.5SUMAP3 GE 200) AND (P6.5SUMAP3 LT 300) AND (P6.QACTIVITE5 EQ 105) AND (P6.AP5JOUR EQ 4)) [P6.JEUDIAP3 = P6.AP3];
IF ((P6.6SUMAP3 GE 200) AND (P6.6SUMAP3 LT 300) AND (P6.QACTIVITE6 EQ 106) AND (P6.AP6JOUR EQ 4)) [P6.JEUDIAP3 = P6.AP3];
IF ((P6.7SUMAP3 GE 200) AND (P6.7SUMAP3 LT 300) AND (P6.QACTIVITE7 EQ 107) AND (P6.AP7JOUR EQ 4)) [P6.JEUDIAP3 = P6.AP3];
IF ((P6.8SUMAP3 GE 200) AND (P6.8SUMAP3 LT 300) AND (P6.QACTIVITE8 EQ 108) AND (P6.AP8JOUR EQ 4)) [P6.JEUDIAP3 = P6.AP3];
IF ((P6.9SUMAP3 GE 200) AND (P6.9SUMAP3 LT 300) AND (P6.QACTIVITE9 EQ 109) AND (P6.AP9JOUR EQ 4)) [P6.JEUDIAP3 = P6.AP3];
IF ((P6.10SUMAP3 GE 200) AND (P6.10SUMAP3 LT 300) AND (P6.QACTIVITE_10 EQ 110) AND (P6.AP10JOUR EQ 4)) [P6.JEUDIAP3 = P6.AP3];
IF ((P6.11SUMAP3 GE 200) AND (P6.11SUMAP3 LT 300) AND (P6.QACTIVITE_11 EQ 111) AND (P6.AP11JOUR EQ 4)) [P6.JEUDIAP3 = P6.AP3];
IF ((P6.12SUMAP3 GE 200) AND (P6.12SUMAP3 LT 300) AND (P6.QACTIVITE_12 EQ 112) AND (P6.AP12JOUR EQ 4)) [P6.JEUDIAP3 = P6.AP3];
IF ((P6.3SUMAP3 GE 200) AND (P6.3SUMAP3 LT 300) AND (P6.QACTIVITE3 EQ 103) AND (P6.AP3JOUR EQ 5)) [P6.VENDREDIAP3 = P6.AP3];
IF ((P6.4SUMAP3 GE 200) AND (P6.4SUMAP3 LT 300) AND (P6.QACTIVITE4 EQ 104) AND (P6.AP4JOUR EQ 5)) [P6.VENDREDIAP3 = P6.AP3];
IF ((P6.5SUMAP3 GE 200) AND (P6.5SUMAP3 LT 300) AND (P6.QACTIVITE5 EQ 105) AND (P6.AP5JOUR EQ 5)) [P6.VENDREDIAP3 = P6.AP3];
IF ((P6.6SUMAP3 GE 200) AND (P6.6SUMAP3 LT 300) AND (P6.QACTIVITE6 EQ 106) AND (P6.AP6JOUR EQ 5)) [P6.VENDREDIAP3 = P6.AP3];
IF ((P6.7SUMAP3 GE 200) AND (P6.7SUMAP3 LT 300) AND (P6.QACTIVITE7 EQ 107) AND (P6.AP7JOUR EQ 5)) [P6.VENDREDIAP3 = P6.AP3];
IF ((P6.8SUMAP3 GE 200) AND (P6.8SUMAP3 LT 300) AND (P6.QACTIVITE8 EQ 108) AND (P6.AP8JOUR EQ 5)) [P6.VENDREDIAP3 = P6.AP3];

IF ((P6.9SUMAP3 GE 200) AND (P6.9SUMAP3 LT 300) AND (P6.QACTIVITE9 EQ 109) AND (P6.AP9JOUR EQ 5)) [P6.VENDREDIAP3 = P6.AP3];
IF ((P6.10SUMAP3 GE 200) AND (P6.10SUMAP3 LT 300) AND (P6.QACTIVITE_10 EQ 110) AND (P6.AP10JOUR EQ 5)) [P6.VENDREDIAP3 = P6.AP3];
IF ((P6.11SUMAP3 GE 200) AND (P6.11SUMAP3 LT 300) AND (P6.QACTIVITE_11 EQ 111) AND (P6.AP11JOUR EQ 5)) [P6.VENDREDIAP3 = P6.AP3];
IF ((P6.12SUMAP3 GE 200) AND (P6.12SUMAP3 LT 300) AND (P6.QACTIVITE_12 EQ 112) AND (P6.AP12JOUR EQ 5)) [P6.VENDREDIAP3 = P6.AP3];
IF ((P6.3SUMAP3 GE 200) AND (P6.3SUMAP3 LT 300) AND (P6.QACTIVITE3 EQ 103) AND (P6.AP3JOUR EQ 6)) [P6.SAMEDIAP3 = P6.AP3];
IF ((P6.4SUMAP3 GE 200) AND (P6.4SUMAP3 LT 300) AND (P6.QACTIVITE4 EQ 104) AND (P6.AP4JOUR EQ 6)) [P6.SAMEDIAP3 = P6.AP3];
IF ((P6.5SUMAP3 GE 200) AND (P6.5SUMAP3 LT 300) AND (P6.QACTIVITE5 EQ 105) AND (P6.AP5JOUR EQ 6)) [P6.SAMEDIAP3 = P6.AP3];
IF ((P6.6SUMAP3 GE 200) AND (P6.6SUMAP3 LT 300) AND (P6.QACTIVITE6 EQ 106) AND (P6.AP6JOUR EQ 6)) [P6.SAMEDIAP3 = P6.AP3];
IF ((P6.7SUMAP3 GE 200) AND (P6.7SUMAP3 LT 300) AND (P6.QACTIVITE7 EQ 107) AND (P6.AP7JOUR EQ 6)) [P6.SAMEDIAP3 = P6.AP3];
IF ((P6.8SUMAP3 GE 200) AND (P6.8SUMAP3 LT 300) AND (P6.QACTIVITE8 EQ 108) AND (P6.AP8JOUR EQ 6)) [P6.SAMEDIAP3 = P6.AP3];
IF ((P6.9SUMAP3 GE 200) AND (P6.9SUMAP3 LT 300) AND (P6.QACTIVITE9 EQ 109) AND (P6.AP9JOUR EQ 6)) [P6.SAMEDIAP3 = P6.AP3];
IF ((P6.10SUMAP3 GE 200) AND (P6.10SUMAP3 LT 300) AND (P6.QACTIVITE_10 EQ 110) AND (P6.AP10JOUR EQ 6)) [P6.SAMEDIAP3 = P6.AP3];
IF ((P6.11SUMAP3 GE 200) AND (P6.11SUMAP3 LT 300) AND (P6.QACTIVITE_11 EQ 111) AND (P6.AP11JOUR EQ 6)) [P6.SAMEDIAP3 = P6.AP3];
IF ((P6.12SUMAP3 GE 200) AND (P6.12SUMAP3 LT 300) AND (P6.QACTIVITE_12 EQ 112) AND (P6.AP12JOUR EQ 6)) [P6.SAMEDIAP3 = P6.AP3];
IF ((P6.3SUMAP3 GE 200) AND (P6.3SUMAP3 LT 300) AND (P6.QACTIVITE3 EQ 103) AND (P6.AP3JOUR EQ 7)) [P6.DIMANCHEAP3 = P6.AP3];
IF ((P6.4SUMAP3 GE 200) AND (P6.4SUMAP3 LT 300) AND (P6.QACTIVITE4 EQ 104) AND (P6.AP4JOUR EQ 7)) [P6.DIMANCHEAP3 = P6.AP3];
IF ((P6.5SUMAP3 GE 200) AND (P6.5SUMAP3 LT 300) AND (P6.QACTIVITE5 EQ 105) AND (P6.AP5JOUR EQ 7)) [P6.DIMANCHEAP3 = P6.AP3];
IF ((P6.6SUMAP3 GE 200) AND (P6.6SUMAP3 LT 300) AND (P6.QACTIVITE6 EQ 106) AND (P6.AP6JOUR EQ 7)) [P6.DIMANCHEAP3 = P6.AP3];
IF ((P6.7SUMAP3 GE 200) AND (P6.7SUMAP3 LT 300) AND (P6.QACTIVITE7 EQ 107) AND (P6.AP7JOUR EQ 7)) [P6.DIMANCHEAP3 = P6.AP3];
IF ((P6.8SUMAP3 GE 200) AND (P6.8SUMAP3 LT 300) AND (P6.QACTIVITE8 EQ 108) AND (P6.AP8JOUR EQ 7)) [P6.DIMANCHEAP3 = P6.AP3];
IF ((P6.9SUMAP3 GE 200) AND (P6.9SUMAP3 LT 300) AND (P6.QACTIVITE9 EQ 109) AND (P6.AP9JOUR EQ 7)) [P6.DIMANCHEAP3 = P6.AP3];

IF ((P6.10SUMAP3 GE 200) AND (P6.10SUMAP3 LT 300) AND (P6.QACTIVITE_10 EQ 110) AND (P6.AP10JOUR EQ 7)) [P6.DIMANCHEAP3 = P6.AP3];
IF ((P6.11SUMAP3 GE 200) AND (P6.11SUMAP3 LT 300) AND (P6.QACTIVITE_11 EQ 111) AND (P6.AP11JOUR EQ 7)) [P6.DIMANCHEAP3 = P6.AP3];
IF ((P6.12SUMAP3 GE 200) AND (P6.12SUMAP3 LT 300) AND (P6.QACTIVITE_12 EQ 112) AND (P6.AP12JOUR EQ 7)) [P6.DIMANCHEAP3 = P6.AP3];
POURSUITE DU CODE
IF (P6.P7BARCHOICE EQ 101) [P6.BAR = 'Être trop fatigué ou fatiguée pour faire de l´activité physique'];
IF (P6.P7BARCHOICE EQ 102) [P6.BAR = 'Avoir beaucoup de choses à faire mis à part l´activité physique'];
IF (P6.P7BARCHOICE EQ 103) [P6.BAR = 'température sera trop mauvaise pour faire de l´activité physique'];
IF (P6.P7BARCHOICE EQ 104) [P6.BAR = 'Ne pas avoir personne avec qui faire de l´activité physique'];
IF (P6.P7BARCHOICE EQ 105) [P6.BAR = 'Ne pas avoir accès à des emplacements pour faire de l´activité physique'];
IF (P6.P7BARCHOICE EQ 106) [P6.BAR = 'Ne pas avoir d´argent pour faire de l´activité physique'];
IF (P6.P7BARCHOICE EQ 107) [P6.BAR = 'Être trop gêné ou gênée, manquer de confiance pour aller faire de l´activité physique'];
IF (P6.P7BARCHOICE EQ 108) [P6.BAR = 'Avoir peur d´être insuffisamment en santé pour faire de l´activité physique'];
IF (P6.P7BARCHOICE EQ 109) [P6.BAR = 'Manquer de motivation'];
IF ((P6.P7BARCHOICE EQ 110) AND (P6.Q_BARANSWER NE 0)) [P6.BAR = P6.Q_BARANSWER];
IF (P6.SUMSOL EQ 101) [P6.SOL = 'Je vais faire de l´activité tôt dans la journée, en matinée ou à l´heure du midi.'];
IF (P6.SUMSOL EQ 102) [P6.SOL = 'J´essaie de garder en tête que si je fais de l´activité physique, j´aurai de plus en plus d´énergie dans la vie.'];
IF (P6.SUMSOL EQ 103) [P6.SOL = 'Je vais planifier des activités avec d´autres personnes qui me motiveront.'];
IF (P6.SUMSOL EQ 104) [P6.SOL = 'Je prends une collation pour me donner de l´énergie et j´y vais quand même.'];
IF (P6.SUMSOL EQ 201) [P6.SOL = 'Faire de l´activité physique par bloc de 10 minutes les journées où je n´ai pas de temps.'];
IF (P6.SUMSOL EQ 202) [P6.SOL = 'Je vais remplacer des activités où je suis assis ou assise, télé, ordinateur ou autres, par des activités physiques.'];
IF (P6.SUMSOL EQ 203) [P6.SOL = 'Je vais me procurer un agenda et inscrire dans mon horaire les moments où je vais faire de l´activité physique.'];
IF (P6.SUMSOL EQ 204) [P6.SOL = 'Je vais prendre des pauses de travail active et faire des 10 minutes de marche.'];
IF (P6.SUMSOL EQ 205) [P6.SOL = 'Je vais me fixer des moments avec d´autres personnes qui me plaisent pour aller faire de l´activité physique.'];
IF (P6.SUMSOL EQ 301) [P6.SOL = 'Je vais me procurer l´équipement nécessaire pour en faire même quand il pleut, quand il neige ou quand il fait trop chaud.'];
IF (P6.SUMSOL EQ 302) [P6.SOL = 'Je vais faire de l’activité physique chez nous ou à l´intérieur dans un endroit que j´aurai choisi quand il fait moins beau.'];

IF (P6.SUMSOL EQ 303) [P6.SOL = 'Je vais me préparer une activité physique alternative au cas où il serait désagréable d´aller dehors.'];
IF (P6.SUMSOL EQ 304) [P6.SOL = 'Je vais me dresser une liste des activités que je serais prêtE à faire si la température est mauvaise.'];
IF (P6.SUMSOL EQ 401) [P6.SOL = 'Je vais me joindre à un groupe qui pratique l´activité physique que j´aime. Ex : la marche, l´aquaforme ou la natation.'];
IF (P6.SUMSOL EQ 402) [P6.SOL = 'Je vais inviter mes amis, ma famille ou mes collègues à faire de l´activité physique avec moi.'];
IF (P6.SUMSOL EQ 403) [P6.SOL = 'Je vais amener mon chien marcher avec moi.'];
IF (P6.SUMSOL EQ 404) [P6.SOL = 'Je vais participer dans des discussions en ligne sur des pages facebook : Diabète Québec et autres.'];
IF (P6.SUMSOL EQ 501) [P6.SOL = 'Je vais faire de la marche ou du vélo dehors, c´est simple et gratuit.'];
IF (P6.SUMSOL EQ 502) [P6.SOL = 'Je vais faire de l´activité physique dans ma maison comme du yoga, un programme sur DVD, vélo stationnaire, tapis roulant, etc.'];
IF (P6.SUMSOL EQ 503) [P6.SOL = 'Je vais aller voir au centre communautaire de mon quartier pour me donner des options.'];
IF (P6.SUMSOL EQ 504) [P6.SOL = 'Je vais discuter avec mon médecin, avec un ami ou un spécialiste de l´activité physique pour avoir des conseils.'];
IF (P6.SUMSOL EQ 601) [P6.SOL = 'Je vais trouver des activités physiques abordables et simples : marcher dehors, faire du vélo, nager à la piscine communautaire.'];
IF (P6.SUMSOL EQ 602) [P6.SOL = 'Je vais aller voir au centre communautaire de mon quartier pour me donner des options.'];
IF (P6.SUMSOL EQ 603) [P6.SOL = 'Je vais économiser pour m´acheter un DVD d´activité physique ou pour une autre activité physique que j´aimerais faire.'];
IF (P6.SUMSOL EQ 604) [P6.SOL = 'Je vais aller marcher avec ma famille, des amis ou des collègues.'];
IF (P6.SUMSOL EQ 701) [P6.SOL = 'Je vais choisir une activité physique simple comme la marche ou la bicyclette.'];
IF (P6.SUMSOL EQ 702) [P6.SOL = 'Je vais aller faire de l´activité physique avec des gens qui m´acceptent comme je suis, qui évitent de me juger.'];
IF (P6.SUMSOL EQ 703) [P6.SOL = 'Au départ, je vais faire de l´activité physique dans des endroits où je suis seul[LETTRE_E] pour prendre confiance.'];
IF (P6.SUMSOL EQ 704) [P6.SOL = 'Je vais éviter des endroits qui me gêne davantage comme les centres de conditionnement.'];
IF (P6.SUMSOL EQ 801) [P6.SOL = 'Je vais consulter mon médecin pour mettre au clair si je peux faire de l´activité physique.'];
IF (P6.SUMSOL EQ 802) [P6.SOL = 'Je vais consulter un spécialiste de l´activité physique pour qu´il me rassure et me conseille sur ce qui est sécuritaire pour moi.'];
IF (P6.SUMSOL EQ 803) [P6.SOL = 'Je vais commencer à petite dose. 10 à 20 minutes maximum d´activités physiques les jours où je suis actif, active.'];
IF (P6.SUMSOL EQ 804) [P6.SOL = 'Je vais faire de l´activité physique accompagnéE d´un spécialiste de l´activité physique.'];

IF (P6.SUMSOL EQ 805) [P6.SOL = 'Je vais faire de l´activité physique dans un groupe où un spécialiste de l´activité physique est présent.'];
IF (P6.SUMSOL EQ 901) [P6.SOL = 'Je vais signer un contrat papier personnel comme quoi je m´engage à être actif, active.'];
IF (P6.SUMSOL EQ 902) [P6.SOL = 'Je vais signer un contrat papier avec mes proches comme quoi je m´engage à être actif, active.'];
IF (P6.SUMSOL EQ 903) [P6.SOL = 'Je vais essayer de nouvelles activités physiques pour trouver celles qui me plaisent vraiment.'];
IF (P6.SUMSOL EQ 904) [P6.SOL = 'À chaque activité physique que je fais, je prends un temps pour me rappeler ce que cela va m´apporter de mieux dans ma vie.'];
IF (P6.SUMSOL EQ 905) [P6.SOL = 'Je vais faire de l´activité physique avec des gens que j´aime pour me motiver.'];
IF ((P6.SUMSOL EQ 999) AND (P6.P8QSOLOPEN NE 0)) [P6.SOL = P6.P8QSOLOPEN];
IF (P6.SUMSOL EQ 0) [P6.SOL = P6.P8QSOL_10];
PLAN DE LA semaine 7
formules p7
IF ((P7.QACTI12 EQ 112) AND (P7.QAOPEN NE 0)) [P7.QACTIVITE_12 = 112];
[P7.2SUMAP2 = P7.QACTIVITE1];
[P7.3SUMAP2 = P7.QACTIVITE1+P7.QACTIVITE2];
[P7.4SUMAP2 = P7.QACTIVITE1+P7.QACTIVITE2+P7.QACTIVITE3];
[P7.5SUMAP2 = P7.QACTIVITE1+P7.QACTIVITE2+P7.QACTIVITE3+P7.QACTIVITE4];
[P7.6SUMAP2 = P7.QACTIVITE1+P7.QACTIVITE2+P7.QACTIVITE3+P7.QACTIVITE4+P7.QACTIVITE5];
[P7.7SUMAP2 =
P7.QACTIVITE1+P7.QACTIVITE2+P7.QACTIVITE3+P7.QACTIVITE4+P7.QACTIVITE5+P7.QACTIVITE6];
[P7.8SUMAP2 =
P7.QACTIVITE1+P7.QACTIVITE2+P7.QACTIVITE3+P7.QACTIVITE4+P7.QACTIVITE5+P7.QACTIVITE6+P7.QACTIVITE7];
[P7.9SUMAP2 =
P7.QACTIVITE1+P7.QACTIVITE2+P7.QACTIVITE3+P7.QACTIVITE4+P7.QACTIVITE5+P7.QACTIVITE6+P7.QACTIVITE7+P7.QACTIVITE8];
[P7.10SUMAP2 =
P7.QACTIVITE1+P7.QACTIVITE2+P7.QACTIVITE3+P7.QACTIVITE4+P7.QACTIVITE5+P7.QACTIVITE6+P7.QACTIVITE7+P7.QACTIVITE8+P7.QACTIVITE9];
[P7.11SUMAP2 =
P7.QACTIVITE1+P7.QACTIVITE2+P7.QACTIVITE3+P7.QACTIVITE4+P7.QACTIVITE5+P7.QACTIVITE6+P7.QACTIVITE7+P7.QACTIVITE8+P7.QACTIVITE9+P7.QACTIVITE_10];
[P7.12SUMAP2 =
P7.QACTIVITE1+P7.QACTIVITE2+P7.QACTIVITE3+P7.QACTIVITE4+P7.QACTIVITE5+P7.QACTIVITE6+P7.QACTIVITE7+P7.QACTIVITE8+P7.QACTIVITE9+P7.QACTIVITE_10+P7.QACTIVITE_11];
[P7.3SUMAP3 = P7.QACTIVITE1+P7.QACTIVITE2];
[P7.4SUMAP3 = P7.QACTIVITE1+P7.QACTIVITE2+P7.QACTIVITE3];
[P7.5SUMAP3 = P7.QACTIVITE1+P7.QACTIVITE2+P7.QACTIVITE3+P7.QACTIVITE4];

```
[P7.6SUMAP3 = P7.QACTIVITE1+P7.QACTIVITE2+P7.QACTIVITE3+P7.QACTIVITE4+P7.QACTIVITE5];
[P7.7SUMAP3 =
P7.QACTIVITE1+P7.QACTIVITE2+P7.QACTIVITE3+P7.QACTIVITE4+P7.QACTIVITE5+P7.QACTIVITE6];
[P7.8SUMAP3 =
P7.QACTIVITE1+P7.QACTIVITE2+P7.QACTIVITE3+P7.QACTIVITE4+P7.QACTIVITE5+P7.QACTIVITE6+P7.QACTI
VITE7];
[P7.9SUMAP3 =
P7.QACTIVITE1+P7.QACTIVITE2+P7.QACTIVITE3+P7.QACTIVITE4+P7.QACTIVITE5+P7.QACTIVITE6+P7.QACTI
VITE7+P7.QACTIVITE8];
[P7.10SUMAP3 =
P7.QACTIVITE1+P7.QACTIVITE2+P7.QACTIVITE3+P7.QACTIVITE4+P7.QACTIVITE5+P7.QACTIVITE6+P7.QACTI
VITE7+P7.QACTIVITE8+P7.QACTIVITE9];
[P7.11SUMAP3 =
P7.QACTIVITE1+P7.QACTIVITE2+P7.QACTIVITE3+P7.QACTIVITE4+P7.QACTIVITE5+P7.QACTIVITE6+P7.QACTI
VITE7+P7.QACTIVITE8+P7.QACTIVITE9+P7.QACTIVITE_10];
[P7.12SUMAP3 =
P7.QACTIVITE1+P7.QACTIVITE2+P7.QACTIVITE3+P7.QACTIVITE4+P7.QACTIVITE5+P7.QACTIVITE6+P7.QACTI
VITE7+P7.QACTIVITE8+P7.QACTIVITE9+P7.QACTIVITE_10+P7.QACTIVITE_11];
[P7.SUMAP =
P7.QACTIVITE1+P7.QACTIVITE2+P7.QACTIVITE3+P7.QACTIVITE4+P7.QACTIVITE5+P7.QACTIVITE6+P7.QACTI
VITE7+P7.QACTIVITE8+P7.QACTIVITE9+P7.QACTIVITE_10+P7.QACTIVITE_11+P7.QACTIVITE_12];
[P7.SUMSOL =
P7.P8QSOL1+P7.P8QSOL2+P7.P8QSOL3+P7.P8QSOL4+P7.P8QSOL5+P7.P8QSOL6+P7.P8QSOL7+P7.P8QSOL8
+P7.P8QSOL9];
IF (P7.QACTIVITE1 EQ 101) [P7.AP1 = 'marche rapide'];
IF ((P7.QACTIVITE1 NE 101) AND (P7.QACTIVITE2 EQ 102)) [P7.AP1 = 'raquettes à neige'];
IF ((P7.QACTIVITE1 NE 101) AND (P7.QACTIVITE2 NE 102) AND (P7.QACTIVITE3 EQ 103)) [P7.AP1 = 'vélo'];
IF ((P7.QACTIVITE1 NE 101) AND (P7.QACTIVITE2 NE 102) AND (P7.QACTIVITE3 NE 103) AND
(P7.QACTIVITE4 EQ 104)) [P7.AP1 = 'natation'];
IF ((P7.QACTIVITE1 NE 101) AND (P7.QACTIVITE2 NE 102) AND (P7.QACTIVITE3 NE 103) AND
(P7.QACTIVITE4 NE 104) AND (P7.QACTIVITE5 EQ 105)) [P7.AP1 = 'ski de fond'];
IF ((P7.QACTIVITE1 NE 101) AND (P7.QACTIVITE2 NE 102) AND (P7.QACTIVITE3 NE 103) AND
(P7.QACTIVITE4 NE 104) AND (P7.QACTIVITE5 NE 105) AND (P7.QACTIVITE6 EQ 106)) [P7.AP1 = 'tennis'];
IF ((P7.QACTIVITE1 NE 101) AND (P7.QACTIVITE2 NE 102) AND (P7.QACTIVITE3 NE 103) AND
(P7.QACTIVITE4 NE 104) AND (P7.QACTIVITE5 NE 105) AND (P7.QACTIVITE6 NE 106) AND (P7.QACTIVITE7
EQ 107)) [P7.AP1 = 'course à pieds'];
IF ((P7.QACTIVITE1 NE 101) AND (P7.QACTIVITE2 NE 102) AND (P7.QACTIVITE3 NE 103) AND
(P7.QACTIVITE4 NE 104) AND (P7.QACTIVITE5 NE 105) AND (P7.QACTIVITE6 NE 106) AND (P7.QACTIVITE7
NE 107) AND (P7.QACTIVITE8 EQ 108)) [P7.AP1 = 'hockey'];
IF ((P7.QACTIVITE1 NE 101) AND (P7.QACTIVITE2 NE 102) AND (P7.QACTIVITE3 NE 103) AND
(P7.QACTIVITE4 NE 104) AND (P7.QACTIVITE5 NE 105) AND (P7.QACTIVITE6 NE 106) AND (P7.QACTIVITE7
NE 107) AND (P7.QACTIVITE8 NE 108) AND (P7.QACTIVITE9 EQ 109)) [P7.AP1 = 'basketball'];
```

IF ((P7.QACTIVITE1 NE 101) AND (P7.QACTIVITE2 NE 102) AND (P7.QACTIVITE3 NE 103) AND (P7.QACTIVITE4 NE 104) AND (P7.QACTIVITE5 NE 105) AND (P7.QACTIVITE6 NE 106) AND (P7.QACTIVITE7 NE 107) AND (P7.QACTIVITE8 NE 108) AND (P7.QACTIVITE9 NE 109) AND (P7.QACTIVITE_10 EQ 110)) [P7.AP1 = 'soccer'];
IF ((P7.QACTIVITE1 NE 101) AND (P7.QACTIVITE2 NE 102) AND (P7.QACTIVITE3 NE 103) AND (P7.QACTIVITE4 NE 104) AND (P7.QACTIVITE5 NE 105) AND (P7.QACTIVITE6 NE 106) AND (P7.QACTIVITE7 NE 107) AND (P7.QACTIVITE8 NE 108) AND (P7.QACTIVITE9 NE 109) AND (P7.QACTIVITE_10 NE 110) AND (P7.QACTIVITE_11 EQ 111)) [P7.AP1 = 'centre de conditionnement'];
IF ((P7.QACTIVITE1 NE 101) AND (P7.QACTIVITE2 NE 102) AND (P7.QACTIVITE3 NE 103) AND (P7.QACTIVITE4 NE 104) AND (P7.QACTIVITE5 NE 105) AND (P7.QACTIVITE6 NE 106) AND (P7.QACTIVITE7 NE 107) AND (P7.QACTIVITE8 NE 108) AND (P7.QACTIVITE9 NE 109) AND (P7.QACTIVITE_10 NE 110) AND (P7.QACTIVITE_11 NE 111) AND (P7.QACTIVITE_12 EQ 112)) [P7.AP1 = P7.QAOPEN];
IF ((P7.2SUMAP2 GE 100) AND (P7.2SUMAP2 LT 200) AND (P7.QACTIVITE2 EQ 102)) [P7.AP2 = 'raquettes à neige'];
IF ((P7.3SUMAP2 GE 100) AND (P7.3SUMAP2 LT 200) AND (P7.QACTIVITE3 EQ 103)) [P7.AP2 = 'vélo'];
IF ((P7.4SUMAP2 GE 100) AND (P7.4SUMAP2 LT 200) AND (P7.QACTIVITE4 EQ 104)) [P7.AP2 = 'natation'];
IF ((P7.5SUMAP2 GE 100) AND (P7.5SUMAP2 LT 200) AND (P7.QACTIVITE5 EQ 105)) [P7.AP2 = 'ski de fond'];
IF ((P7.6SUMAP2 GE 100) AND (P7.6SUMAP2 LT 200) AND (P7.QACTIVITE6 EQ 106)) [P7.AP2 = 'tennis'];
IF ((P7.7SUMAP2 GE 100) AND (P7.7SUMAP2 LT 200) AND (P7.QACTIVITE7 EQ 107)) [P7.AP2 = 'course à pieds'];
IF ((P7.8SUMAP2 GE 100) AND (P7.8SUMAP2 LT 200) AND (P7.QACTIVITE8 EQ 108)) [P7.AP2 = 'hockey'];
IF ((P7.9SUMAP2 GE 100) AND (P7.9SUMAP2 LT 200) AND (P7.QACTIVITE9 EQ 109)) [P7.AP2 = 'basketball'];
IF ((P7.10SUMAP2 GE 100) AND (P7.10SUMAP2 LT 200) AND (P7.QACTIVITE_10 EQ 110)) [P7.AP2 = 'soccer'];
IF ((P7.11SUMAP2 GE 100) AND (P7.11SUMAP2 LT 200) AND (P7.QACTIVITE_11 EQ 111)) [P7.AP2 = 'centre de conditionnement'];
IF ((P7.12SUMAP2 GE 100) AND (P7.12SUMAP2 LT 200) AND (P7.QACTIVITE_12 EQ 112)) [P7.AP2 = P7.QAOPEN];
IF ((P7.3SUMAP3 GE 200) AND (P7.3SUMAP3 LT 300) AND (P7.QACTIVITE3 EQ 103)) [P7.AP3 = 'vélo'];
IF ((P7.4SUMAP3 GE 200) AND (P7.4SUMAP3 LT 300) AND (P7.QACTIVITE4 EQ 104)) [P7.AP3 = 'natation'];
IF ((P7.5SUMAP3 GE 200) AND (P7.5SUMAP3 LT 300) AND (P7.QACTIVITE5 EQ 105)) [P7.AP3 = 'ski de fond'];
IF ((P7.6SUMAP3 GE 200) AND (P7.6SUMAP3 LT 300) AND (P7.QACTIVITE6 EQ 106)) [P7.AP3 = 'tennis'];
IF ((P7.7SUMAP3 GE 200) AND (P7.7SUMAP3 LT 300) AND (P7.QACTIVITE7 EQ 107)) [P7.AP3 = 'course à pieds'];
IF ((P7.8SUMAP3 GE 200) AND (P7.8SUMAP3 LT 300) AND (P7.QACTIVITE8 EQ 108)) [P7.AP3 = 'hockey'];
IF ((P7.9SUMAP3 GE 200) AND (P7.9SUMAP3 LT 300) AND (P7.QACTIVITE9 EQ 109)) [P7.AP3 = 'basketball'];
IF ((P7.10SUMAP3 GE 200) AND (P7.10SUMAP3 LT 300) AND (P7.QACTIVITE_10 EQ 110)) [P7.AP3 = 'soccer'];
IF ((P7.11SUMAP3 GE 200) AND (P7.11SUMAP3 LT 300) AND (P7.QACTIVITE_11 EQ 111)) [P7.AP3 = 'centre de conditionnement'];
IF ((P7.12SUMAP3 GE 200) AND (P7.12SUMAP3 LT 300) AND (P7.QACTIVITE_12 EQ 112)) [P7.AP3 = P7.QAOPEN];

ACTIVITÉ 1 DANS LE PLAN POUR CHAQUE JOUR DE LA SEMAINE
IF ((P7.QACTIVITE1 EQ 101) AND (P7.AP1JOUR EQ 1)) [P7.LUNDIAP1 = P7.AP1];
IF ((P7.QACTIVITE1 NE 101) AND (P7.QACTIVITE2 EQ 102) AND (P7.AP2JOUR EQ 1)) [P7.LUNDIAP1 = P7.AP1];
IF ((P7.QACTIVITE1 NE 101) AND (P7.QACTIVITE2 NE 102) AND (P7.QACTIVITE3 EQ 103) AND (P7.AP3JOUR EQ 1)) [P7.LUNDIAP1 = P7.AP1];
IF ((P7.QACTIVITE1 NE 101) AND (P7.QACTIVITE2 NE 102) AND (P7.QACTIVITE3 NE 103) AND (P7.QACTIVITE4 EQ 104) AND (P7.AP4JOUR EQ 1)) [P7.LUNDIAP1 = P7.AP1];
IF ((P7.QACTIVITE1 NE 101) AND (P7.QACTIVITE2 NE 102) AND (P7.QACTIVITE3 NE 103) AND (P7.QACTIVITE4 NE 104) AND (P7.QACTIVITE5 EQ 105) AND (P7.AP5JOUR EQ 1)) [P7.LUNDIAP1 = P7.AP1];
IF ((P7.QACTIVITE1 NE 101) AND (P7.QACTIVITE2 NE 102) AND (P7.QACTIVITE3 NE 103) AND (P7.QACTIVITE4 NE 104) AND (P7.QACTIVITE5 NE 105) AND (P7.QACTIVITE6 EQ 106) AND (P7.AP6JOUR EQ 1)) [P7.LUNDIAP1 = P7.AP1];
IF ((P7.QACTIVITE1 NE 101) AND (P7.QACTIVITE2 NE 102) AND (P7.QACTIVITE3 NE 103) AND (P7.QACTIVITE4 NE 104) AND (P7.QACTIVITE5 NE 105) AND (P7.QACTIVITE6 NE 106) AND (P7.QACTIVITE7 EQ 107) AND (P7.AP7JOUR EQ 1)) [P7.LUNDIAP1 = P7.AP1];
IF ((P7.QACTIVITE1 NE 101) AND (P7.QACTIVITE2 NE 102) AND (P7.QACTIVITE3 NE 103) AND (P7.QACTIVITE4 NE 104) AND (P7.QACTIVITE5 NE 105) AND (P7.QACTIVITE6 NE 106) AND (P7.QACTIVITE7 NE 107) AND (P7.QACTIVITE8 EQ 108) AND (P7.AP8JOUR EQ 1)) [P7.LUNDIAP1 = P7.AP1];
IF ((P7.QACTIVITE1 NE 101) AND (P7.QACTIVITE2 NE 102) AND (P7.QACTIVITE3 NE 103) AND (P7.QACTIVITE4 NE 104) AND (P7.QACTIVITE5 NE 105) AND (P7.QACTIVITE6 NE 106) AND (P7.QACTIVITE7 NE 107) AND (P7.QACTIVITE8 NE 108) AND (P7.QACTIVITE9 EQ 109) AND (P7.AP9JOUR EQ 1)) [P7.LUNDIAP1 = P7.AP1];
IF ((P7.QACTIVITE1 NE 101) AND (P7.QACTIVITE2 NE 102) AND (P7.QACTIVITE3 NE 103) AND (P7.QACTIVITE4 NE 104) AND (P7.QACTIVITE5 NE 105) AND (P7.QACTIVITE6 NE 106) AND (P7.QACTIVITE7 NE 107) AND (P7.QACTIVITE8 NE 108) AND (P7.QACTIVITE9 NE 109) AND (P7.QACTIVITE_10 EQ 110) AND (P7.AP10JOUR EQ 1)) [P7.LUNDIAP1 = P7.AP1];
IF ((P7.QACTIVITE1 NE 101) AND (P7.QACTIVITE2 NE 102) AND (P7.QACTIVITE3 NE 103) AND (P7.QACTIVITE4 NE 104) AND (P7.QACTIVITE5 NE 105) AND (P7.QACTIVITE6 NE 106) AND (P7.QACTIVITE7 NE 107) AND (P7.QACTIVITE8 NE 108) AND (P7.QACTIVITE9 NE 109) AND (P7.QACTIVITE_10 NE 110) AND (P7.QACTIVITE_11 EQ 111) AND (P7.AP11JOUR EQ 1)) [P7.LUNDIAP1 = P7.AP1];
IF ((P7.QACTIVITE1 NE 101) AND (P7.QACTIVITE2 NE 102) AND (P7.QACTIVITE3 NE 103) AND (P7.QACTIVITE4 NE 104) AND (P7.QACTIVITE5 NE 105) AND (P7.QACTIVITE6 NE 106) AND (P7.QACTIVITE7 NE 107) AND (P7.QACTIVITE8 NE 108) AND (P7.QACTIVITE9 NE 109) AND (P7.QACTIVITE_10 NE 110) AND (P7.QACTIVITE_11 NE 111) AND (P7.QACTIVITE_12 EQ 112) AND (P7.AP12JOUR EQ 1)) [P7.LUNDIAP1 = P7.AP1];
IF ((P7.QACTIVITE1 EQ 101) AND (P7.AP1JOUR EQ 2)) [P7.MARDIAP1 = P7.AP1];
IF ((P7.QACTIVITE1 NE 101) AND (P7.QACTIVITE2 EQ 102) AND (P7.AP2JOUR EQ 2)) [P7.MARDIAP1 = P7.AP1];
IF ((P7.QACTIVITE1 NE 101) AND (P7.QACTIVITE2 NE 102) AND (P7.QACTIVITE3 EQ 103) AND (P7.AP3JOUR EQ 2)) [P7.MARDIAP1 = P7.AP1];

IF ((P7.QACTIVITE1 NE 101) AND (P7.QACTIVITE2 NE 102) AND (P7.QACTIVITE3 NE 103) AND (P7.QACTIVITE4 EQ 104) AND (P7.AP4JOUR EQ 2)) [P7.MARDIAP1 = P7.AP1];
IF ((P7.QACTIVITE1 NE 101) AND (P7.QACTIVITE2 NE 102) AND (P7.QACTIVITE3 NE 103) AND (P7.QACTIVITE4 NE 104) AND (P7.QACTIVITE5 EQ 105) AND (P7.AP5JOUR EQ 2)) [P7.MARDIAP1 = P7.AP1];
IF ((P7.QACTIVITE1 NE 101) AND (P7.QACTIVITE2 NE 102) AND (P7.QACTIVITE3 NE 103) AND (P7.QACTIVITE4 NE 104) AND (P7.QACTIVITE5 NE 105) AND (P7.QACTIVITE6 EQ 106) AND (P7.AP6JOUR EQ 2)) [P7.MARDIAP1 = P7.AP1];
IF ((P7.QACTIVITE1 NE 101) AND (P7.QACTIVITE2 NE 102) AND (P7.QACTIVITE3 NE 103) AND (P7.QACTIVITE4 NE 104) AND (P7.QACTIVITE5 NE 105) AND (P7.QACTIVITE6 NE 106) AND (P7.QACTIVITE7 EQ 107) AND (P7.AP7JOUR EQ 2)) [P7.MARDIAP1 = P7.AP1];
IF ((P7.QACTIVITE1 NE 101) AND (P7.QACTIVITE2 NE 102) AND (P7.QACTIVITE3 NE 103) AND (P7.QACTIVITE4 NE 104) AND (P7.QACTIVITE5 NE 105) AND (P7.QACTIVITE6 NE 106) AND (P7.QACTIVITE7 NE 107) AND (P7.QACTIVITE8 EQ 108) AND (P7.AP8JOUR EQ 2)) [P7.MARDIAP1 = P7.AP1];
IF ((P7.QACTIVITE1 NE 101) AND (P7.QACTIVITE2 NE 102) AND (P7.QACTIVITE3 NE 103) AND (P7.QACTIVITE4 NE 104) AND (P7.QACTIVITE5 NE 105) AND (P7.QACTIVITE6 NE 106) AND (P7.QACTIVITE7 NE 107) AND (P7.QACTIVITE8 NE 108) AND (P7.QACTIVITE9 EQ 109) AND (P7.AP9JOUR EQ 2)) [P7.MARDIAP1 = P7.AP1];
IF ((P7.QACTIVITE1 NE 101) AND (P7.QACTIVITE2 NE 102) AND (P7.QACTIVITE3 NE 103) AND (P7.QACTIVITE4 NE 104) AND (P7.QACTIVITE5 NE 105) AND (P7.QACTIVITE6 NE 106) AND (P7.QACTIVITE7 NE 107) AND (P7.QACTIVITE8 NE 108) AND (P7.QACTIVITE9 NE 109) AND (P7.QACTIVITE_10 EQ 110) AND (P7.AP10JOUR EQ 2)) [P7.MARDIAP1 = P7.AP1];
IF ((P7.QACTIVITE1 NE 101) AND (P7.QACTIVITE2 NE 102) AND (P7.QACTIVITE3 NE 103) AND (P7.QACTIVITE4 NE 104) AND (P7.QACTIVITE5 NE 105) AND (P7.QACTIVITE6 NE 106) AND (P7.QACTIVITE7 NE 107) AND (P7.QACTIVITE8 NE 108) AND (P7.QACTIVITE9 NE 109) AND (P7.QACTIVITE_10 NE 110) AND (P7.QACTIVITE_11 EQ 111) AND (P7.AP11JOUR EQ 2)) [P7.MARDIAP1 = P7.AP1];
IF ((P7.QACTIVITE1 NE 101) AND (P7.QACTIVITE2 NE 102) AND (P7.QACTIVITE3 NE 103) AND (P7.QACTIVITE4 NE 104) AND (P7.QACTIVITE5 NE 105) AND (P7.QACTIVITE6 NE 106) AND (P7.QACTIVITE7 NE 107) AND (P7.QACTIVITE8 NE 108) AND (P7.QACTIVITE9 NE 109) AND (P7.QACTIVITE_10 NE 110) AND (P7.QACTIVITE_11 NE 111) AND (P7.QACTIVITE_12 EQ 112) AND (P7.AP12JOUR EQ 2)) [P7.MARDIAP1 = P7.AP1];
IF ((P7.QACTIVITE1 EQ 101) AND (P7.AP1JOUR EQ 3)) [P7.MERCREDIAP1 = P7.AP1];
IF ((P7.QACTIVITE1 NE 101) AND (P7.QACTIVITE2 EQ 102) AND (P7.AP2JOUR EQ 3)) [P7.MERCREDIAP1 = P7.AP1];
IF ((P7.QACTIVITE1 NE 101) AND (P7.QACTIVITE2 NE 102) AND (P7.QACTIVITE3 EQ 103) AND (P7.AP3JOUR EQ 3)) [P7.MERCREDIAP1 = P7.AP1];
IF ((P7.QACTIVITE1 NE 101) AND (P7.QACTIVITE2 NE 102) AND (P7.QACTIVITE3 NE 103) AND (P7.QACTIVITE4 EQ 104) AND (P7.AP4JOUR EQ 3)) [P7.MERCREDIAP1 = P7.AP1];
IF ((P7.QACTIVITE1 NE 101) AND (P7.QACTIVITE2 NE 102) AND (P7.QACTIVITE3 NE 103) AND (P7.QACTIVITE4 NE 104) AND (P7.QACTIVITE5 EQ 105) AND (P7.AP5JOUR EQ 3)) [P7.MERCREDIAP1 = P7.AP1];

IF ((P7.QACTIVITE1 NE 101) AND (P7.QACTIVITE2 NE 102) AND (P7.QACTIVITE3 NE 103) AND (P7.QACTIVITE4 NE 104) AND (P7.QACTIVITES5 NE 105) AND (P7.QACTIVITE6 EQ 106) AND (P7.AP6JOUR EQ 3)) [P7.MERCREDIAP1 = P7.AP1];
IF ((P7.QACTIVITE1 NE 101) AND (P7.QACTIVITE2 NE 102) AND (P7.QACTIVITE3 NE 103) AND (P7.QACTIVITE4 NE 104) AND (P7.QACTIVITES5 NE 105) AND (P7.QACTIVITE6 NE 106) AND (P7.QACTIVITE7 EQ 107) AND (P7.AP7JOUR EQ 3)) [P7.MERCREDIAP1 = P7.AP1];
IF ((P7.QACTIVITE1 NE 101) AND (P7.QACTIVITE2 NE 102) AND (P7.QACTIVITE3 NE 103) AND (P7.QACTIVITE4 NE 104) AND (P7.QACTIVITES5 NE 105) AND (P7.QACTIVITE6 NE 106) AND (P7.QACTIVITE7 NE 107) AND (P7.QACTIVITE8 EQ 108) AND (P7.AP8JOUR EQ 3)) [P7.MERCREDIAP1 = P7.AP1];
IF ((P7.QACTIVITE1 NE 101) AND (P7.QACTIVITE2 NE 102) AND (P7.QACTIVITE3 NE 103) AND (P7.QACTIVITE4 NE 104) AND (P7.QACTIVITES5 NE 105) AND (P7.QACTIVITE6 NE 106) AND (P7.QACTIVITE7 NE 107) AND (P7.QACTIVITE8 NE 108) AND (P7.QACTIVITE9 EQ 109) AND (P7.AP9JOUR EQ 3)) [P7.MERCREDIAP1 = P7.AP1];
IF ((P7.QACTIVITE1 NE 101) AND (P7.QACTIVITE2 NE 102) AND (P7.QACTIVITE3 NE 103) AND (P7.QACTIVITE4 NE 104) AND (P7.QACTIVITES5 NE 105) AND (P7.QACTIVITE6 NE 106) AND (P7.QACTIVITE7 NE 107) AND (P7.QACTIVITE8 NE 108) AND (P7.QACTIVITE9 NE 109) AND (P7.QACTIVITE_10 EQ 110) AND (P7.AP10JOUR EQ 3)) [P7.MERCREDIAP1 = P7.AP1];
IF ((P7.QACTIVITE1 NE 101) AND (P7.QACTIVITE2 NE 102) AND (P7.QACTIVITE3 NE 103) AND (P7.QACTIVITE4 NE 104) AND (P7.QACTIVITES5 NE 105) AND (P7.QACTIVITE6 NE 106) AND (P7.QACTIVITE7 NE 107) AND (P7.QACTIVITE8 NE 108) AND (P7.QACTIVITE9 NE 109) AND (P7.QACTIVITE_10 NE 110) AND (P7.QACTIVITE_11 EQ 111) AND (P7.AP11JOUR EQ 3)) [P7.MERCREDIAP1 = P7.AP1];
IF ((P7.QACTIVITE1 NE 101) AND (P7.QACTIVITE2 NE 102) AND (P7.QACTIVITE3 NE 103) AND (P7.QACTIVITE4 NE 104) AND (P7.QACTIVITES5 NE 105) AND (P7.QACTIVITE6 NE 106) AND (P7.QACTIVITE7 NE 107) AND (P7.QACTIVITE8 NE 108) AND (P7.QACTIVITE9 NE 109) AND (P7.QACTIVITE_10 NE 110) AND (P7.QACTIVITE_11 NE 111) AND (P7.QACTIVITE_12 EQ 112) AND (P7.AP12JOUR EQ 3)) [P7.MERCREDIAP1 = P7.AP1];
IF ((P7.QACTIVITE1 EQ 101) AND (P7.AP1JOUR EQ 4)) [P7.JEUDIAP1 = P7.AP1];
IF ((P7.QACTIVITE1 NE 101) AND (P7.QACTIVITE2 EQ 102) AND (P7.AP2JOUR EQ 4)) [P7.JEUDIAP1 = P7.AP1];
IF ((P7.QACTIVITE1 NE 101) AND (P7.QACTIVITE2 NE 102) AND (P7.QACTIVITE3 EQ 103) AND (P7.AP3JOUR EQ 4)) [P7.JEUDIAP1 = P7.AP1];
IF ((P7.QACTIVITE1 NE 101) AND (P7.QACTIVITE2 NE 102) AND (P7.QACTIVITE3 NE 103) AND (P7.QACTIVITE4 EQ 104) AND (P7.AP4JOUR EQ 4)) [P7.JEUDIAP1 = P7.AP1];
IF ((P7.QACTIVITE1 NE 101) AND (P7.QACTIVITE2 NE 102) AND (P7.QACTIVITE3 NE 103) AND (P7.QACTIVITE4 NE 104) AND (P7.QACTIVITES5 EQ 105) AND (P7.AP5JOUR EQ 4)) [P7.JEUDIAP1 = P7.AP1];
IF ((P7.QACTIVITE1 NE 101) AND (P7.QACTIVITE2 NE 102) AND (P7.QACTIVITE3 NE 103) AND (P7.QACTIVITE4 NE 104) AND (P7.QACTIVITES5 NE 105) AND (P7.QACTIVITE6 EQ 106) AND (P7.AP6JOUR EQ 4)) [P7.JEUDIAP1 = P7.AP1];
IF ((P7.QACTIVITE1 NE 101) AND (P7.QACTIVITE2 NE 102) AND (P7.QACTIVITE3 NE 103) AND (P7.QACTIVITE4 NE 104) AND (P7.QACTIVITES5 NE 105) AND (P7.QACTIVITE6 NE 106) AND (P7.QACTIVITE7 EQ 107) AND (P7.AP7JOUR EQ 4)) [P7.JEUDIAP1 = P7.AP1];

IF ((P7.QACTIVITE1 NE 101) AND (P7.QACTIVITE2 NE 102) AND (P7.QACTIVITE3 NE 103) AND (P7.QACTIVITE4 NE 104) AND (P7.QACTIVITE5 NE 105) AND (P7.QACTIVITE6 NE 106) AND (P7.QACTIVITE7 NE 107) AND (P7.QACTIVITE8 EQ 108) AND (P7.AP8JOUR EQ 4)) [P7.JEUDIAP1 = P7.AP1];
IF ((P7.QACTIVITE1 NE 101) AND (P7.QACTIVITE2 NE 102) AND (P7.QACTIVITE3 NE 103) AND (P7.QACTIVITE4 NE 104) AND (P7.QACTIVITE5 NE 105) AND (P7.QACTIVITE6 NE 106) AND (P7.QACTIVITE7 NE 107) AND (P7.QACTIVITE8 NE 108) AND (P7.QACTIVITE9 EQ 109) AND (P7.AP9JOUR EQ 4)) [P7.JEUDIAP1 = P7.AP1];
IF ((P7.QACTIVITE1 NE 101) AND (P7.QACTIVITE2 NE 102) AND (P7.QACTIVITE3 NE 103) AND (P7.QACTIVITE4 NE 104) AND (P7.QACTIVITE5 NE 105) AND (P7.QACTIVITE6 NE 106) AND (P7.QACTIVITE7 NE 107) AND (P7.QACTIVITE8 NE 108) AND (P7.QACTIVITE9 NE 109) AND (P7.QACTIVITE_10 EQ 110) AND (P7.AP10JOUR EQ 4)) [P7.JEUDIAP1 = P7.AP1];
IF ((P7.QACTIVITE1 NE 101) AND (P7.QACTIVITE2 NE 102) AND (P7.QACTIVITE3 NE 103) AND (P7.QACTIVITE4 NE 104) AND (P7.QACTIVITE5 NE 105) AND (P7.QACTIVITE6 NE 106) AND (P7.QACTIVITE7 NE 107) AND (P7.QACTIVITE8 NE 108) AND (P7.QACTIVITE9 NE 109) AND (P7.QACTIVITE_10 NE 110) AND (P7.QACTIVITE_11 EQ 111) AND (P7.AP11JOUR EQ 4)) [P7.JEUDIAP1 = P7.AP1];
IF ((P7.QACTIVITE1 NE 101) AND (P7.QACTIVITE2 NE 102) AND (P7.QACTIVITE3 NE 103) AND (P7.QACTIVITE4 NE 104) AND (P7.QACTIVITE5 NE 105) AND (P7.QACTIVITE6 NE 106) AND (P7.QACTIVITE7 NE 107) AND (P7.QACTIVITE8 NE 108) AND (P7.QACTIVITE9 NE 109) AND (P7.QACTIVITE_10 NE 110) AND (P7.QACTIVITE_11 NE 111) AND (P7.QACTIVITE_12 EQ 112) AND (P7.AP12JOUR EQ 4)) [P7.JEUDIAP1 = P7.AP1];
IF ((P7.QACTIVITE1 EQ 101) AND (P7.AP1JOUR EQ 5)) [P7.VENDREDIAP1 = P7.AP1];
IF ((P7.QACTIVITE1 NE 101) AND (P7.QACTIVITE2 EQ 102) AND (P7.AP2JOUR EQ 5)) [P7.VENDREDIAP1 = P7.AP1];
IF ((P7.QACTIVITE1 NE 101) AND (P7.QACTIVITE2 NE 102) AND (P7.QACTIVITE3 EQ 103) AND (P7.AP3JOUR EQ 5)) [P7.VENDREDIAP1 = P7.AP1];
IF ((P7.QACTIVITE1 NE 101) AND (P7.QACTIVITE2 NE 102) AND (P7.QACTIVITE3 NE 103) AND (P7.QACTIVITE4 EQ 104) AND (P7.AP4JOUR EQ 5)) [P7.VENDREDIAP1 = P7.AP1];
IF ((P7.QACTIVITE1 NE 101) AND (P7.QACTIVITE2 NE 102) AND (P7.QACTIVITE3 NE 103) AND (P7.QACTIVITE4 NE 104) AND (P7.QACTIVITE5 EQ 105) AND (P7.AP5JOUR EQ 5)) [P7.VENDREDIAP1 = P7.AP1];
IF ((P7.QACTIVITE1 NE 101) AND (P7.QACTIVITE2 NE 102) AND (P7.QACTIVITE3 NE 103) AND (P7.QACTIVITE4 NE 104) AND (P7.QACTIVITE5 NE 105) AND (P7.QACTIVITE6 EQ 106) AND (P7.AP6JOUR EQ 5)) [P7.VENDREDIAP1 = P7.AP1];
IF ((P7.QACTIVITE1 NE 101) AND (P7.QACTIVITE2 NE 102) AND (P7.QACTIVITE3 NE 103) AND (P7.QACTIVITE4 NE 104) AND (P7.QACTIVITE5 NE 105) AND (P7.QACTIVITE6 NE 106) AND (P7.QACTIVITE7 EQ 107) AND (P7.AP7JOUR EQ 5)) [P7.VENDREDIAP1 = P7.AP1];
IF ((P7.QACTIVITE1 NE 101) AND (P7.QACTIVITE2 NE 102) AND (P7.QACTIVITE3 NE 103) AND (P7.QACTIVITE4 NE 104) AND (P7.QACTIVITE5 NE 105) AND (P7.QACTIVITE6 NE 106) AND (P7.QACTIVITE7 NE 107) AND (P7.QACTIVITE8 EQ 108) AND (P7.AP8JOUR EQ 5)) [P7.VENDREDIAP1 = P7.AP1];
IF ((P7.QACTIVITE1 NE 101) AND (P7.QACTIVITE2 NE 102) AND (P7.QACTIVITE3 NE 103) AND (P7.QACTIVITE4 NE 104) AND (P7.QACTIVITE5 NE 105) AND (P7.QACTIVITE6 NE 106) AND (P7.QACTIVITE7

NE 107) AND (P7.QACTIVITE8 NE 108) AND (P7.QACTIVITE9 EQ 109) AND (P7.AP9JOUR EQ 5)) [P7.VENDREDIAP1 = P7.AP1];
IF ((P7.QACTIVITE1 NE 101) AND (P7.QACTIVITE2 NE 102) AND (P7.QACTIVITE3 NE 103) AND (P7.QACTIVITE4 NE 104) AND (P7.QACTIVITE5 NE 105) AND (P7.QACTIVITE6 NE 106) AND (P7.QACTIVITE7 NE 107) AND (P7.QACTIVITE8 NE 108) AND (P7.QACTIVITE9 NE 109) AND (P7.QACTIVITE_10 EQ 110) AND (P7.AP10JOUR EQ 5)) [P7.VENDREDIAP1 = P7.AP1];
IF ((P7.QACTIVITE1 NE 101) AND (P7.QACTIVITE2 NE 102) AND (P7.QACTIVITE3 NE 103) AND (P7.QACTIVITE4 NE 104) AND (P7.QACTIVITE5 NE 105) AND (P7.QACTIVITE6 NE 106) AND (P7.QACTIVITE7 NE 107) AND (P7.QACTIVITE8 NE 108) AND (P7.QACTIVITE9 NE 109) AND (P7.QACTIVITE_10 NE 110) AND (P7.QACTIVITE_11 EQ 111) AND (P7.AP11JOUR EQ 5)) [P7.VENDREDIAP1 = P7.AP1];
IF ((P7.QACTIVITE1 NE 101) AND (P7.QACTIVITE2 NE 102) AND (P7.QACTIVITE3 NE 103) AND (P7.QACTIVITE4 NE 104) AND (P7.QACTIVITE5 NE 105) AND (P7.QACTIVITE6 NE 106) AND (P7.QACTIVITE7 NE 107) AND (P7.QACTIVITE8 NE 108) AND (P7.QACTIVITE9 NE 109) AND (P7.QACTIVITE_10 NE 110) AND (P7.QACTIVITE_11 NE 111) AND (P7.QACTIVITE_12 EQ 112) AND (P7.AP12JOUR EQ 5)) [P7.VENDREDIAP1 = P7.AP1];
IF ((P7.QACTIVITE1 EQ 101) AND (P7.AP1JOUR EQ 6)) [P7.SAMEDIAP1 = P7.AP1];
IF ((P7.QACTIVITE1 NE 101) AND (P7.QACTIVITE2 EQ 102) AND (P7.AP2JOUR EQ 6)) [P7.SAMEDIAP1 = P7.AP1];
IF ((P7.QACTIVITE1 NE 101) AND (P7.QACTIVITE2 NE 102) AND (P7.QACTIVITE3 EQ 103) AND (P7.AP3JOUR EQ 6)) [P7.SAMEDIAP1 = P7.AP1];
IF ((P7.QACTIVITE1 NE 101) AND (P7.QACTIVITE2 NE 102) AND (P7.QACTIVITE3 NE 103) AND (P7.QACTIVITE4 EQ 104) AND (P7.AP4JOUR EQ 6)) [P7.SAMEDIAP1 = P7.AP1];
IF ((P7.QACTIVITE1 NE 101) AND (P7.QACTIVITE2 NE 102) AND (P7.QACTIVITE3 NE 103) AND (P7.QACTIVITE4 NE 104) AND (P7.QACTIVITE5 EQ 105) AND (P7.AP5JOUR EQ 6)) [P7.SAMEDIAP1 = P7.AP1];
IF ((P7.QACTIVITE1 NE 101) AND (P7.QACTIVITE2 NE 102) AND (P7.QACTIVITE3 NE 103) AND (P7.QACTIVITE4 NE 104) AND (P7.QACTIVITE5 NE 105) AND (P7.QACTIVITE6 EQ 106) AND (P7.AP6JOUR EQ 6)) [P7.SAMEDIAP1 = P7.AP1];
IF ((P7.QACTIVITE1 NE 101) AND (P7.QACTIVITE2 NE 102) AND (P7.QACTIVITE3 NE 103) AND (P7.QACTIVITE4 NE 104) AND (P7.QACTIVITE5 NE 105) AND (P7.QACTIVITE6 NE 106) AND (P7.QACTIVITE7 EQ 107) AND (P7.AP7JOUR EQ 6)) [P7.SAMEDIAP1 = P7.AP1];
IF ((P7.QACTIVITE1 NE 101) AND (P7.QACTIVITE2 NE 102) AND (P7.QACTIVITE3 NE 103) AND (P7.QACTIVITE4 NE 104) AND (P7.QACTIVITE5 NE 105) AND (P7.QACTIVITE6 NE 106) AND (P7.QACTIVITE7 NE 107) AND (P7.QACTIVITE8 EQ 108) AND (P7.AP8JOUR EQ 6)) [P7.SAMEDIAP1 = P7.AP1];
IF ((P7.QACTIVITE1 NE 101) AND (P7.QACTIVITE2 NE 102) AND (P7.QACTIVITE3 NE 103) AND (P7.QACTIVITE4 NE 104) AND (P7.QACTIVITE5 NE 105) AND (P7.QACTIVITE6 NE 106) AND (P7.QACTIVITE7 NE 107) AND (P7.QACTIVITE8 NE 108) AND (P7.QACTIVITE9 EQ 109) AND (P7.AP9JOUR EQ 6)) [P7.SAMEDIAP1 = P7.AP1];
IF ((P7.QACTIVITE1 NE 101) AND (P7.QACTIVITE2 NE 102) AND (P7.QACTIVITE3 NE 103) AND (P7.QACTIVITE4 NE 104) AND (P7.QACTIVITE5 NE 105) AND (P7.QACTIVITE6 NE 106) AND (P7.QACTIVITE7 NE 107) AND (P7.QACTIVITE8 NE 108) AND (P7.QACTIVITE9 NE 109) AND (P7.QACTIVITE_10 EQ 110) AND (P7.AP10JOUR EQ 6)) [P7.SAMEDIAP1 = P7.AP1];

IF ((P7.QACTIVITE1 NE 101) AND (P7.QACTIVITE2 NE 102) AND (P7.QACTIVITE3 NE 103) AND (P7.QACTIVITE4 NE 104) AND (P7.QACTIVITE5 NE 105) AND (P7.QACTIVITE6 NE 106) AND (P7.QACTIVITE7 NE 107) AND (P7.QACTIVITE8 NE 108) AND (P7.QACTIVITE9 NE 109) AND (P7.QACTIVITE_10 NE 110) AND (P7.QACTIVITE_11 EQ 111) AND (P7.AP11JOUR EQ 6)) [P7.SAMEDIAP1 = P7.AP1];
IF ((P7.QACTIVITE1 NE 101) AND (P7.QACTIVITE2 NE 102) AND (P7.QACTIVITE3 NE 103) AND (P7.QACTIVITE4 NE 104) AND (P7.QACTIVITE5 NE 105) AND (P7.QACTIVITE6 NE 106) AND (P7.QACTIVITE7 NE 107) AND (P7.QACTIVITE8 NE 108) AND (P7.QACTIVITE9 NE 109) AND (P7.QACTIVITE_10 NE 110) AND (P7.QACTIVITE_11 NE 111) AND (P7.QACTIVITE_12 EQ 112) AND (P7.AP12JOUR EQ 6)) [P7.SAMEDIAP1 = P7.AP1];
IF ((P7.QACTIVITE1 EQ 101) AND (P7.AP1JOUR EQ 7)) [P7.DIMANCHEAP1 = P7.AP1];
IF ((P7.QACTIVITE1 NE 101) AND (P7.QACTIVITE2 EQ 102) AND (P7.AP2JOUR EQ 7)) [P7.DIMANCHEAP1 = P7.AP1];
IF ((P7.QACTIVITE1 NE 101) AND (P7.QACTIVITE2 NE 102) AND (P7.QACTIVITE3 EQ 103) AND (P7.AP3JOUR EQ 7)) [P7.DIMANCHEAP1 = P7.AP1];
IF ((P7.QACTIVITE1 NE 101) AND (P7.QACTIVITE2 NE 102) AND (P7.QACTIVITE3 NE 103) AND (P7.QACTIVITE4 EQ 104) AND (P7.AP4JOUR EQ 7)) [P7.DIMANCHEAP1 = P7.AP1];
IF ((P7.QACTIVITE1 NE 101) AND (P7.QACTIVITE2 NE 102) AND (P7.QACTIVITE3 NE 103) AND (P7.QACTIVITE4 NE 104) AND (P7.QACTIVITE5 EQ 105) AND (P7.AP5JOUR EQ 7)) [P7.DIMANCHEAP1 = P7.AP1];
IF ((P7.QACTIVITE1 NE 101) AND (P7.QACTIVITE2 NE 102) AND (P7.QACTIVITE3 NE 103) AND (P7.QACTIVITE4 NE 104) AND (P7.QACTIVITE5 NE 105) AND (P7.QACTIVITE6 EQ 106) AND (P7.AP6JOUR EQ 7)) [P7.DIMANCHEAP1 = P7.AP1];
IF ((P7.QACTIVITE1 NE 101) AND (P7.QACTIVITE2 NE 102) AND (P7.QACTIVITE3 NE 103) AND (P7.QACTIVITE4 NE 104) AND (P7.QACTIVITE5 NE 105) AND (P7.QACTIVITE6 NE 106) AND (P7.QACTIVITE7 EQ 107) AND (P7.AP7JOUR EQ 7)) [P7.DIMANCHEAP1 = P7.AP1];
IF ((P7.QACTIVITE1 NE 101) AND (P7.QACTIVITE2 NE 102) AND (P7.QACTIVITE3 NE 103) AND (P7.QACTIVITE4 NE 104) AND (P7.QACTIVITE5 NE 105) AND (P7.QACTIVITE6 NE 106) AND (P7.QACTIVITE7 NE 107) AND (P7.QACTIVITE8 EQ 108) AND (P7.AP8JOUR EQ 7)) [P7.DIMANCHEAP1 = P7.AP1];
IF ((P7.QACTIVITE1 NE 101) AND (P7.QACTIVITE2 NE 102) AND (P7.QACTIVITE3 NE 103) AND (P7.QACTIVITE4 NE 104) AND (P7.QACTIVITE5 NE 105) AND (P7.QACTIVITE6 NE 106) AND (P7.QACTIVITE7 NE 107) AND (P7.QACTIVITE8 NE 108) AND (P7.QACTIVITE9 EQ 109) AND (P7.AP9JOUR EQ 7)) [P7.DIMANCHEAP1 = P7.AP1];
IF ((P7.QACTIVITE1 NE 101) AND (P7.QACTIVITE2 NE 102) AND (P7.QACTIVITE3 NE 103) AND (P7.QACTIVITE4 NE 104) AND (P7.QACTIVITE5 NE 105) AND (P7.QACTIVITE6 NE 106) AND (P7.QACTIVITE7 NE 107) AND (P7.QACTIVITE8 NE 108) AND (P7.QACTIVITE9 NE 109) AND (P7.QACTIVITE_10 EQ 110) AND (P7.AP10JOUR EQ 7)) [P7.DIMANCHEAP1 = P7.AP1];
IF ((P7.QACTIVITE1 NE 101) AND (P7.QACTIVITE2 NE 102) AND (P7.QACTIVITE3 NE 103) AND (P7.QACTIVITE4 NE 104) AND (P7.QACTIVITE5 NE 105) AND (P7.QACTIVITE6 NE 106) AND (P7.QACTIVITE7 NE 107) AND (P7.QACTIVITE8 NE 108) AND (P7.QACTIVITE9 NE 109) AND (P7.QACTIVITE_10 NE 110) AND (P7.QACTIVITE_11 EQ 111) AND (P7.AP11JOUR EQ 7)) [P7.DIMANCHEAP1 = P7.AP1];
IF ((P7.QACTIVITE1 NE 101) AND (P7.QACTIVITE2 NE 102) AND (P7.QACTIVITE3 NE 103) AND (P7.QACTIVITE4 NE 104) AND (P7.QACTIVITE5 NE 105) AND (P7.QACTIVITE6 NE 106) AND (P7.QACTIVITE7

NE 107) AND (P7.QACTIVITE8 NE 108) AND (P7.QACTIVITE9 NE 109) AND (P7.QACTIVITE_10 NE 110) AND (P7.QACTIVITE_11 NE 111) AND (P7.QACTIVITE_12 EQ 112) AND (P7.AP12JOUR EQ 7)) [P7.DIMANCHEAP1 = P7.AP1];
ACTIVITÉ 2 DANS LE PLAN POUR CHAQUE JOUR DE LA SEMAINE
IF ((P7.2SUMAP2 GE 100) AND (P7.2SUMAP2 LT 200) AND (P7.QACTIVITE2 EQ 102) AND (P7.AP2JOUR EQ 1)) [P7.LUNDIAP2 = P7.AP2];
IF ((P7.3SUMAP2 GE 100) AND (P7.3SUMAP2 LT 200) AND (P7.QACTIVITE3 EQ 103) AND (P7.AP3JOUR EQ 1)) [P7.LUNDIAP2 = P7.AP2];
IF ((P7.4SUMAP2 GE 100) AND (P7.4SUMAP2 LT 200) AND (P7.QACTIVITE4 EQ 104) AND (P7.AP4JOUR EQ 1)) [P7.LUNDIAP2 = P7.AP2];
IF ((P7.5SUMAP2 GE 100) AND (P7.5SUMAP2 LT 200) AND (P7.QACTIVITE5 EQ 105) AND (P7.AP5JOUR EQ 1)) [P7.LUNDIAP2 = P7.AP2];
IF ((P7.6SUMAP2 GE 100) AND (P7.6SUMAP2 LT 200) AND (P7.QACTIVITE6 EQ 106) AND (P7.AP6JOUR EQ 1)) [P7.LUNDIAP2 = P7.AP2];
IF ((P7.7SUMAP2 GE 100) AND (P7.7SUMAP2 LT 200) AND (P7.QACTIVITE7 EQ 107) AND (P7.AP7JOUR EQ 1)) [P7.LUNDIAP2 = P7.AP2];
IF ((P7.8SUMAP2 GE 100) AND (P7.8SUMAP2 LT 200) AND (P7.QACTIVITE8 EQ 108) AND (P7.AP8JOUR EQ 1)) [P7.LUNDIAP2 = P7.AP2];
IF ((P7.9SUMAP2 GE 100) AND (P7.9SUMAP2 LT 200) AND (P7.QACTIVITE9 EQ 109) AND (P7.AP9JOUR EQ 1)) [P7.LUNDIAP2 = P7.AP2];
IF ((P7.10SUMAP2 GE 100) AND (P7.10SUMAP2 LT 200) AND (P7.QACTIVITE_10 EQ 110) AND (P7.AP10JOUR EQ 1)) [P7.LUNDIAP2 = P7.AP2];
IF ((P7.11SUMAP2 GE 100) AND (P7.11SUMAP2 LT 200) AND (P7.QACTIVITE_11 EQ 111) AND (P7.AP11JOUR EQ 1)) [P7.LUNDIAP2 = P7.AP2];
IF ((P7.12SUMAP2 GE 100) AND (P7.12SUMAP2 LT 200) AND (P7.QACTIVITE_12 EQ 112) AND (P7.AP12JOUR EQ 1)) [P7.LUNDIAP2 = P7.AP2];
IF ((P7.2SUMAP2 GE 100) AND (P7.2SUMAP2 LT 200) AND (P7.QACTIVITE2 EQ 102) AND (P7.AP2JOUR EQ 2)) [P7.MARDIAP2 = P7.AP2];
IF ((P7.3SUMAP2 GE 100) AND (P7.3SUMAP2 LT 200) AND (P7.QACTIVITE3 EQ 103) AND (P7.AP3JOUR EQ 2)) [P7.MARDIAP2 = P7.AP2];
IF ((P7.4SUMAP2 GE 100) AND (P7.4SUMAP2 LT 200) AND (P7.QACTIVITE4 EQ 104) AND (P7.AP4JOUR EQ 2)) [P7.MARDIAP2 = P7.AP2];
IF ((P7.5SUMAP2 GE 100) AND (P7.5SUMAP2 LT 200) AND (P7.QACTIVITE5 EQ 105) AND (P7.AP5JOUR EQ 2)) [P7.MARDIAP2 = P7.AP2];
IF ((P7.6SUMAP2 GE 100) AND (P7.6SUMAP2 LT 200) AND (P7.QACTIVITE6 EQ 106) AND (P7.AP6JOUR EQ 2)) [P7.MARDIAP2 = P7.AP2];
IF ((P7.7SUMAP2 GE 100) AND (P7.7SUMAP2 LT 200) AND (P7.QACTIVITE7 EQ 107) AND (P7.AP7JOUR EQ 2)) [P7.MARDIAP2 = P7.AP2];
IF ((P7.8SUMAP2 GE 100) AND (P7.8SUMAP2 LT 200) AND (P7.QACTIVITE8 EQ 108) AND (P7.AP8JOUR EQ 2)) [P7.MARDIAP2 = P7.AP2];
IF ((P7.9SUMAP2 GE 100) AND (P7.9SUMAP2 LT 200) AND (P7.QACTIVITE9 EQ 109) AND (P7.AP9JOUR EQ 2)) [P7.MARDIAP2 = P7.AP2];

IF ((P7.10SUMAP2 GE 100) AND (P7.10SUMAP2 LT 200) AND (P7.QACTIVITE_10 EQ 110) AND (P7.AP10JOUR EQ 2)) [P7.MARDIAP2 = P7.AP2];
IF ((P7.11SUMAP2 GE 100) AND (P7.11SUMAP2 LT 200) AND (P7.QACTIVITE_11 EQ 111) AND (P7.AP11JOUR EQ 2)) [P7.MARDIAP2 = P7.AP2];
IF ((P7.12SUMAP2 GE 100) AND (P7.12SUMAP2 LT 200) AND (P7.QACTIVITE_12 EQ 112) AND (P7.AP12JOUR EQ 2)) [P7.MARDIAP2 = P7.AP2];
IF ((P7.2SUMAP2 GE 100) AND (P7.2SUMAP2 LT 200) AND (P7.QACTIVITE2 EQ 102) AND (P7.AP2JOUR EQ 3)) [P7.MERCREDIAP2 = P7.AP2];
IF ((P7.3SUMAP2 GE 100) AND (P7.3SUMAP2 LT 200) AND (P7.QACTIVITE3 EQ 103) AND (P7.AP3JOUR EQ 3)) [P7.MERCREDIAP2 = P7.AP2];
IF ((P7.4SUMAP2 GE 100) AND (P7.4SUMAP2 LT 200) AND (P7.QACTIVITE4 EQ 104) AND (P7.AP4JOUR EQ 3)) [P7.MERCREDIAP2 = P7.AP2];
IF ((P7.5SUMAP2 GE 100) AND (P7.5SUMAP2 LT 200) AND (P7.QACTIVITE5 EQ 105) AND (P7.AP5JOUR EQ 3)) [P7.MERCREDIAP2 = P7.AP2];
IF ((P7.6SUMAP2 GE 100) AND (P7.6SUMAP2 LT 200) AND (P7.QACTIVITE6 EQ 106) AND (P7.AP6JOUR EQ 3)) [P7.MERCREDIAP2 = P7.AP2];
IF ((P7.7SUMAP2 GE 100) AND (P7.7SUMAP2 LT 200) AND (P7.QACTIVITE7 EQ 107) AND (P7.AP7JOUR EQ 3)) [P7.MERCREDIAP2 = P7.AP2];
IF ((P7.8SUMAP2 GE 100) AND (P7.8SUMAP2 LT 200) AND (P7.QACTIVITE8 EQ 108) AND (P7.AP8JOUR EQ 3)) [P7.MERCREDIAP2 = P7.AP2];
IF ((P7.9SUMAP2 GE 100) AND (P7.9SUMAP2 LT 200) AND (P7.QACTIVITE9 EQ 109) AND (P7.AP9JOUR EQ 3)) [P7.MERCREDIAP2 = P7.AP2];
IF ((P7.10SUMAP2 GE 100) AND (P7.10SUMAP2 LT 200) AND (P7.QACTIVITE_10 EQ 110) AND (P7.AP10JOUR EQ 3)) [P7.MERCREDIAP2 = P7.AP2];
IF ((P7.11SUMAP2 GE 100) AND (P7.11SUMAP2 LT 200) AND (P7.QACTIVITE_11 EQ 111) AND (P7.AP11JOUR EQ 3)) [P7.MERCREDIAP2 = P7.AP2];
IF ((P7.12SUMAP2 GE 100) AND (P7.12SUMAP2 LT 200) AND (P7.QACTIVITE_12 EQ 112) AND (P7.AP12JOUR EQ 3)) [P7.MERCREDIAP2 = P7.AP2];
IF ((P7.2SUMAP2 GE 100) AND (P7.2SUMAP2 LT 200) AND (P7.QACTIVITE2 EQ 102) AND (P7.AP2JOUR EQ 4)) [P7.JEUDIAP2 = P7.AP2];
IF ((P7.3SUMAP2 GE 100) AND (P7.3SUMAP2 LT 200) AND (P7.QACTIVITE3 EQ 103) AND (P7.AP3JOUR EQ 4)) [P7.JEUDIAP2 = P7.AP2];
IF ((P7.4SUMAP2 GE 100) AND (P7.4SUMAP2 LT 200) AND (P7.QACTIVITE4 EQ 104) AND (P7.AP4JOUR EQ 4)) [P7.JEUDIAP2 = P7.AP2];
IF ((P7.5SUMAP2 GE 100) AND (P7.5SUMAP2 LT 200) AND (P7.QACTIVITE5 EQ 105) AND (P7.AP5JOUR EQ 4)) [P7.JEUDIAP2 = P7.AP2];
IF ((P7.6SUMAP2 GE 100) AND (P7.6SUMAP2 LT 200) AND (P7.QACTIVITE6 EQ 106) AND (P7.AP6JOUR EQ 4)) [P7.JEUDIAP2 = P7.AP2];
IF ((P7.7SUMAP2 GE 100) AND (P7.7SUMAP2 LT 200) AND (P7.QACTIVITE7 EQ 107) AND (P7.AP7JOUR EQ 4)) [P7.JEUDIAP2 = P7.AP2];
IF ((P7.8SUMAP2 GE 100) AND (P7.8SUMAP2 LT 200) AND (P7.QACTIVITE8 EQ 108) AND (P7.AP8JOUR EQ 4)) [P7.JEUDIAP2 = P7.AP2];

IF ((P7.9SUMAP2 GE 100) AND (P7.9SUMAP2 LT 200) AND (P7.QACTIVITE9 EQ 109) AND (P7.AP9JOUR EQ 4)) [P7.JEUDIAP2 = P7.AP2];
IF ((P7.10SUMAP2 GE 100) AND (P7.10SUMAP2 LT 200) AND (P7.QACTIVITE_10 EQ 110) AND (P7.AP10JOUR EQ 4)) [P7.JEUDIAP2 = P7.AP2];
IF ((P7.11SUMAP2 GE 100) AND (P7.11SUMAP2 LT 200) AND (P7.QACTIVITE_11 EQ 111) AND (P7.AP11JOUR EQ 4)) [P7.JEUDIAP2 = P7.AP2];
IF ((P7.12SUMAP2 GE 100) AND (P7.12SUMAP2 LT 200) AND (P7.QACTIVITE_12 EQ 112) AND (P7.AP12JOUR EQ 4)) [P7.JEUDIAP2 = P7.AP2];
IF ((P7.2SUMAP2 GE 100) AND (P7.2SUMAP2 LT 200) AND (P7.QACTIVITE2 EQ 102) AND (P7.AP2JOUR EQ 5)) [P7.VENDREDIAP2 = P7.AP2];
IF ((P7.3SUMAP2 GE 100) AND (P7.3SUMAP2 LT 200) AND (P7.QACTIVITE3 EQ 103) AND (P7.AP3JOUR EQ 5)) [P7.VENDREDIAP2 = P7.AP2];
IF ((P7.4SUMAP2 GE 100) AND (P7.4SUMAP2 LT 200) AND (P7.QACTIVITE4 EQ 104) AND (P7.AP4JOUR EQ 5)) [P7.VENDREDIAP2 = P7.AP2];
IF ((P7.5SUMAP2 GE 100) AND (P7.5SUMAP2 LT 200) AND (P7.QACTIVITE5 EQ 105) AND (P7.AP5JOUR EQ 5)) [P7.VENDREDIAP2 = P7.AP2];
IF ((P7.6SUMAP2 GE 100) AND (P7.6SUMAP2 LT 200) AND (P7.QACTIVITE6 EQ 106) AND (P7.AP6JOUR EQ 5)) [P7.VENDREDIAP2 = P7.AP2];
IF ((P7.7SUMAP2 GE 100) AND (P7.7SUMAP2 LT 200) AND (P7.QACTIVITE7 EQ 107) AND (P7.AP7JOUR EQ 5)) [P7.VENDREDIAP2 = P7.AP2];
IF ((P7.8SUMAP2 GE 100) AND (P7.8SUMAP2 LT 200) AND (P7.QACTIVITE8 EQ 108) AND (P7.AP8JOUR EQ 5)) [P7.VENDREDIAP2 = P7.AP2];
IF ((P7.9SUMAP2 GE 100) AND (P7.9SUMAP2 LT 200) AND (P7.QACTIVITE9 EQ 109) AND (P7.AP9JOUR EQ 5)) [P7.VENDREDIAP2 = P7.AP2];
IF ((P7.10SUMAP2 GE 100) AND (P7.10SUMAP2 LT 200) AND (P7.QACTIVITE_10 EQ 110) AND (P7.AP10JOUR EQ 5)) [P7.VENDREDIAP2 = P7.AP2];
IF ((P7.11SUMAP2 GE 100) AND (P7.11SUMAP2 LT 200) AND (P7.QACTIVITE_11 EQ 111) AND (P7.AP11JOUR EQ 5)) [P7.VENDREDIAP2 = P7.AP2];
IF ((P7.12SUMAP2 GE 100) AND (P7.12SUMAP2 LT 200) AND (P7.QACTIVITE_12 EQ 112) AND (P7.AP12JOUR EQ 5)) [P7.VENDREDIAP2 = P7.AP2];
IF ((P7.2SUMAP2 GE 100) AND (P7.2SUMAP2 LT 200) AND (P7.QACTIVITE2 EQ 102) AND (P7.AP2JOUR EQ 6)) [P7.SAMEDIAP2 = P7.AP2];
IF ((P7.3SUMAP2 GE 100) AND (P7.3SUMAP2 LT 200) AND (P7.QACTIVITE3 EQ 103) AND (P7.AP3JOUR EQ 6)) [P7.SAMEDIAP2 = P7.AP2];
IF ((P7.4SUMAP2 GE 100) AND (P7.4SUMAP2 LT 200) AND (P7.QACTIVITE4 EQ 104) AND (P7.AP4JOUR EQ 6)) [P7.SAMEDIAP2 = P7.AP2];
IF ((P7.5SUMAP2 GE 100) AND (P7.5SUMAP2 LT 200) AND (P7.QACTIVITE5 EQ 105) AND (P7.AP5JOUR EQ 6)) [P7.SAMEDIAP2 = P7.AP2];
IF ((P7.6SUMAP2 GE 100) AND (P7.6SUMAP2 LT 200) AND (P7.QACTIVITE6 EQ 106) AND (P7.AP6JOUR EQ 6)) [P7.SAMEDIAP2 = P7.AP2];
IF ((P7.7SUMAP2 GE 100) AND (P7.7SUMAP2 LT 200) AND (P7.QACTIVITE7 EQ 107) AND (P7.AP7JOUR EQ 6)) [P7.SAMEDIAP2 = P7.AP2];

IF ((P7.8SUMAP2 GE 100) AND (P7.8SUMAP2 LT 200) AND (P7.QACTIVITE8 EQ 108) AND (P7.AP8JOUR EQ 6)) [P7.SAMEDIAP2 = P7.AP2];
IF ((P7.9SUMAP2 GE 100) AND (P7.9SUMAP2 LT 200) AND (P7.QACTIVITE9 EQ 109) AND (P7.AP9JOUR EQ 6)) [P7.SAMEDIAP2 = P7.AP2];
IF ((P7.10SUMAP2 GE 100) AND (P7.10SUMAP2 LT 200) AND (P7.QACTIVITE_10 EQ 110) AND (P7.AP10JOUR EQ 6)) [P7.SAMEDIAP2 = P7.AP2];
IF ((P7.11SUMAP2 GE 100) AND (P7.11SUMAP2 LT 200) AND (P7.QACTIVITE_11 EQ 111) AND (P7.AP11JOUR EQ 6)) [P7.SAMEDIAP2 = P7.AP2];
IF ((P7.12SUMAP2 GE 100) AND (P7.12SUMAP2 LT 200) AND (P7.QACTIVITE_12 EQ 112) AND (P7.AP12JOUR EQ 6)) [P7.SAMEDIAP2 = P7.AP2];
IF ((P7.2SUMAP2 GE 100) AND (P7.2SUMAP2 LT 200) AND (P7.QACTIVITE2 EQ 102) AND (P7.AP2JOUR EQ 7)) [P7.DIMANCHEAP2 = P7.AP2];
IF ((P7.3SUMAP2 GE 100) AND (P7.3SUMAP2 LT 200) AND (P7.QACTIVITE3 EQ 103) AND (P7.AP3JOUR EQ 7)) [P7.DIMANCHEAP2 = P7.AP2];
IF ((P7.4SUMAP2 GE 100) AND (P7.4SUMAP2 LT 200) AND (P7.QACTIVITE4 EQ 104) AND (P7.AP4JOUR EQ 7)) [P7.DIMANCHEAP2 = P7.AP2];
IF ((P7.5SUMAP2 GE 100) AND (P7.5SUMAP2 LT 200) AND (P7.QACTIVITE5 EQ 105) AND (P7.AP5JOUR EQ 7)) [P7.DIMANCHEAP2 = P7.AP2];
IF ((P7.6SUMAP2 GE 100) AND (P7.6SUMAP2 LT 200) AND (P7.QACTIVITE6 EQ 106) AND (P7.AP6JOUR EQ 7)) [P7.DIMANCHEAP2 = P7.AP2];
IF ((P7.7SUMAP2 GE 100) AND (P7.7SUMAP2 LT 200) AND (P7.QACTIVITE7 EQ 107) AND (P7.AP7JOUR EQ 7)) [P7.DIMANCHEAP2 = P7.AP2];
IF ((P7.8SUMAP2 GE 100) AND (P7.8SUMAP2 LT 200) AND (P7.QACTIVITE8 EQ 108) AND (P7.AP8JOUR EQ 7)) [P7.DIMANCHEAP2 = P7.AP2];
IF ((P7.9SUMAP2 GE 100) AND (P7.9SUMAP2 LT 200) AND (P7.QACTIVITE9 EQ 109) AND (P7.AP9JOUR EQ 7)) [P7.DIMANCHEAP2 = P7.AP2];
IF ((P7.10SUMAP2 GE 100) AND (P7.10SUMAP2 LT 200) AND (P7.QACTIVITE_10 EQ 110) AND (P7.AP10JOUR EQ 7)) [P7.DIMANCHEAP2 = P7.AP2];
IF ((P7.11SUMAP2 GE 100) AND (P7.11SUMAP2 LT 200) AND (P7.QACTIVITE_11 EQ 111) AND (P7.AP11JOUR EQ 7)) [P7.DIMANCHEAP2 = P7.AP2];
IF ((P7.12SUMAP2 GE 100) AND (P7.12SUMAP2 LT 200) AND (P7.QACTIVITE_12 EQ 112) AND (P7.AP12JOUR EQ 7)) [P7.DIMANCHEAP2 = P7.AP2];
ACTIVITÉ 3 DANS LE PLAN POUR CHAQUE JOUR DE LA SEMAINE
IF ((P7.3SUMAP3 GE 200) AND (P7.3SUMAP3 LT 300) AND (P7.QACTIVITE3 EQ 103) AND (P7.AP3JOUR EQ 1)) [P7.LUNDIAP3 = P7.AP3];
IF ((P7.4SUMAP3 GE 200) AND (P7.4SUMAP3 LT 300) AND (P7.QACTIVITE4 EQ 104) AND (P7.AP4JOUR EQ 1)) [P7.LUNDIAP3 = P7.AP3];
IF ((P7.5SUMAP3 GE 200) AND (P7.5SUMAP3 LT 300) AND (P7.QACTIVITE5 EQ 105) AND (P7.AP5JOUR EQ 1)) [P7.LUNDIAP3 = P7.AP3];
IF ((P7.6SUMAP3 GE 200) AND (P7.6SUMAP3 LT 300) AND (P7.QACTIVITE6 EQ 106) AND (P7.AP6JOUR EQ 1)) [P7.LUNDIAP3 = P7.AP3];

IF ((P7.7SUMAP3 GE 200) AND (P7.7SUMAP3 LT 300) AND (P7.QACTIVITE7 EQ 107) AND (P7.AP7JOUR EQ 1)) [P7.LUNDIAP3 = P7.AP3];
IF ((P7.8SUMAP3 GE 200) AND (P7.8SUMAP3 LT 300) AND (P7.QACTIVITE8 EQ 108) AND (P7.AP8JOUR EQ 1)) [P7.LUNDIAP3 = P7.AP3];
IF ((P7.9SUMAP3 GE 200) AND (P7.9SUMAP3 LT 300) AND (P7.QACTIVITE9 EQ 109) AND (P7.AP9JOUR EQ 1)) [P7.LUNDIAP3 = P7.AP3];
IF ((P7.10SUMAP3 GE 200) AND (P7.10SUMAP3 LT 300) AND (P7.QACTIVITE_10 EQ 110) AND (P7.AP10JOUR EQ 1)) [P7.LUNDIAP3 = P7.AP3];
IF ((P7.11SUMAP3 GE 200) AND (P7.11SUMAP3 LT 300) AND (P7.QACTIVITE_11 EQ 111) AND (P7.AP11JOUR EQ 1)) [P7.LUNDIAP3 = P7.AP3];
IF ((P7.12SUMAP3 GE 200) AND (P7.12SUMAP3 LT 300) AND (P7.QACTIVITE_12 EQ 112) AND (P7.AP12JOUR EQ 1)) [P7.LUNDIAP3 = P7.AP3];
IF ((P7.3SUMAP3 GE 200) AND (P7.3SUMAP3 LT 300) AND (P7.QACTIVITE3 EQ 103) AND (P7.AP3JOUR EQ 2)) [P7.MARDIAP3 = P7.AP3];
IF ((P7.4SUMAP3 GE 200) AND (P7.4SUMAP3 LT 300) AND (P7.QACTIVITE4 EQ 104) AND (P7.AP4JOUR EQ 2)) [P7.MARDIAP3 = P7.AP3];
IF ((P7.5SUMAP3 GE 200) AND (P7.5SUMAP3 LT 300) AND (P7.QACTIVITE5 EQ 105) AND (P7.AP5JOUR EQ 2)) [P7.MARDIAP3 = P7.AP3];
IF ((P7.6SUMAP3 GE 200) AND (P7.6SUMAP3 LT 300) AND (P7.QACTIVITE6 EQ 106) AND (P7.AP6JOUR EQ 2)) [P7.MARDIAP3 = P7.AP3];
IF ((P7.7SUMAP3 GE 200) AND (P7.7SUMAP3 LT 300) AND (P7.QACTIVITE7 EQ 107) AND (P7.AP7JOUR EQ 2)) [P7.MARDIAP3 = P7.AP3];
IF ((P7.8SUMAP3 GE 200) AND (P7.8SUMAP3 LT 300) AND (P7.QACTIVITE8 EQ 108) AND (P7.AP8JOUR EQ 2)) [P7.MARDIAP3 = P7.AP3];
IF ((P7.9SUMAP3 GE 200) AND (P7.9SUMAP3 LT 300) AND (P7.QACTIVITE9 EQ 109) AND (P7.AP9JOUR EQ 2)) [P7.MARDIAP3 = P7.AP3];
IF ((P7.10SUMAP3 GE 200) AND (P7.10SUMAP3 LT 300) AND (P7.QACTIVITE_10 EQ 110) AND (P7.AP10JOUR EQ 2)) [P7.MARDIAP3 = P7.AP3];
IF ((P7.11SUMAP3 GE 200) AND (P7.11SUMAP3 LT 300) AND (P7.QACTIVITE_11 EQ 111) AND (P7.AP11JOUR EQ 2)) [P7.MARDIAP3 = P7.AP3];
IF ((P7.12SUMAP3 GE 200) AND (P7.12SUMAP3 LT 300) AND (P7.QACTIVITE_12 EQ 112) AND (P7.AP12JOUR EQ 2)) [P7.MARDIAP3 = P7.AP3];
IF ((P7.3SUMAP3 GE 200) AND (P7.3SUMAP3 LT 300) AND (P7.QACTIVITE3 EQ 103) AND (P7.AP3JOUR EQ 3)) [P7.MERCREDIAP3 = P7.AP3];
IF ((P7.4SUMAP3 GE 200) AND (P7.4SUMAP3 LT 300) AND (P7.QACTIVITE4 EQ 104) AND (P7.AP4JOUR EQ 3)) [P7.MERCREDIAP3 = P7.AP3];
IF ((P7.5SUMAP3 GE 200) AND (P7.5SUMAP3 LT 300) AND (P7.QACTIVITE5 EQ 105) AND (P7.AP5JOUR EQ 3)) [P7.MERCREDIAP3 = P7.AP3];
IF ((P7.6SUMAP3 GE 200) AND (P7.6SUMAP3 LT 300) AND (P7.QACTIVITE6 EQ 106) AND (P7.AP6JOUR EQ 3)) [P7.MERCREDIAP3 = P7.AP3];
IF ((P7.7SUMAP3 GE 200) AND (P7.7SUMAP3 LT 300) AND (P7.QACTIVITE7 EQ 107) AND (P7.AP7JOUR EQ 3)) [P7.MERCREDIAP3 = P7.AP3];

IF ((P7.8SUMAP3 GE 200) AND (P7.8SUMAP3 LT 300) AND (P7.QACTIVITE8 EQ 108) AND (P7.AP8JOUR EQ 3)) [P7.MERCREDIAP3 = P7.AP3];
IF ((P7.9SUMAP3 GE 200) AND (P7.9SUMAP3 LT 300) AND (P7.QACTIVITE9 EQ 109) AND (P7.AP9JOUR EQ 3)) [P7.MERCREDIAP3 = P7.AP3];
IF ((P7.10SUMAP3 GE 200) AND (P7.10SUMAP3 LT 300) AND (P7.QACTIVITE_10 EQ 110) AND (P7.AP10JOUR EQ 3)) [P7.MERCREDIAP3 = P7.AP3];
IF ((P7.11SUMAP3 GE 200) AND (P7.11SUMAP3 LT 300) AND (P7.QACTIVITE_11 EQ 111) AND (P7.AP11JOUR EQ 3)) [P7.MERCREDIAP3 = P7.AP3];
IF ((P7.12SUMAP3 GE 200) AND (P7.12SUMAP3 LT 300) AND (P7.QACTIVITE_12 EQ 112) AND (P7.AP12JOUR EQ 3)) [P7.MERCREDIAP3 = P7.AP3];
IF ((P7.3SUMAP3 GE 200) AND (P7.3SUMAP3 LT 300) AND (P7.QACTIVITE3 EQ 103) AND (P7.AP3JOUR EQ 4)) [P7.JEUDIAP3 = P7.AP3];
IF ((P7.4SUMAP3 GE 200) AND (P7.4SUMAP3 LT 300) AND (P7.QACTIVITE4 EQ 104) AND (P7.AP4JOUR EQ 4)) [P7.JEUDIAP3 = P7.AP3];
IF ((P7.5SUMAP3 GE 200) AND (P7.5SUMAP3 LT 300) AND (P7.QACTIVITE5 EQ 105) AND (P7.AP5JOUR EQ 4)) [P7.JEUDIAP3 = P7.AP3];
IF ((P7.6SUMAP3 GE 200) AND (P7.6SUMAP3 LT 300) AND (P7.QACTIVITE6 EQ 106) AND (P7.AP6JOUR EQ 4)) [P7.JEUDIAP3 = P7.AP3];
IF ((P7.7SUMAP3 GE 200) AND (P7.7SUMAP3 LT 300) AND (P7.QACTIVITE7 EQ 107) AND (P7.AP7JOUR EQ 4)) [P7.JEUDIAP3 = P7.AP3];
IF ((P7.8SUMAP3 GE 200) AND (P7.8SUMAP3 LT 300) AND (P7.QACTIVITE8 EQ 108) AND (P7.AP8JOUR EQ 4)) [P7.JEUDIAP3 = P7.AP3];
IF ((P7.9SUMAP3 GE 200) AND (P7.9SUMAP3 LT 300) AND (P7.QACTIVITE9 EQ 109) AND (P7.AP9JOUR EQ 4)) [P7.JEUDIAP3 = P7.AP3];
IF ((P7.10SUMAP3 GE 200) AND (P7.10SUMAP3 LT 300) AND (P7.QACTIVITE_10 EQ 110) AND (P7.AP10JOUR EQ 4)) [P7.JEUDIAP3 = P7.AP3];
IF ((P7.11SUMAP3 GE 200) AND (P7.11SUMAP3 LT 300) AND (P7.QACTIVITE_11 EQ 111) AND (P7.AP11JOUR EQ 4)) [P7.JEUDIAP3 = P7.AP3];
IF ((P7.12SUMAP3 GE 200) AND (P7.12SUMAP3 LT 300) AND (P7.QACTIVITE_12 EQ 112) AND (P7.AP12JOUR EQ 4)) [P7.JEUDIAP3 = P7.AP3];
IF ((P7.3SUMAP3 GE 200) AND (P7.3SUMAP3 LT 300) AND (P7.QACTIVITE3 EQ 103) AND (P7.AP3JOUR EQ 5)) [P7.VENDREDIAP3 = P7.AP3];
IF ((P7.4SUMAP3 GE 200) AND (P7.4SUMAP3 LT 300) AND (P7.QACTIVITE4 EQ 104) AND (P7.AP4JOUR EQ 5)) [P7.VENDREDIAP3 = P7.AP3];
IF ((P7.5SUMAP3 GE 200) AND (P7.5SUMAP3 LT 300) AND (P7.QACTIVITE5 EQ 105) AND (P7.AP5JOUR EQ 5)) [P7.VENDREDIAP3 = P7.AP3];
IF ((P7.6SUMAP3 GE 200) AND (P7.6SUMAP3 LT 300) AND (P7.QACTIVITE6 EQ 106) AND (P7.AP6JOUR EQ 5)) [P7.VENDREDIAP3 = P7.AP3];
IF ((P7.7SUMAP3 GE 200) AND (P7.7SUMAP3 LT 300) AND (P7.QACTIVITE7 EQ 107) AND (P7.AP7JOUR EQ 5)) [P7.VENDREDIAP3 = P7.AP3];
IF ((P7.8SUMAP3 GE 200) AND (P7.8SUMAP3 LT 300) AND (P7.QACTIVITE8 EQ 108) AND (P7.AP8JOUR EQ 5)) [P7.VENDREDIAP3 = P7.AP3];

IF ((P7.9SUMAP3 GE 200) AND (P7.9SUMAP3 LT 300) AND (P7.QACTIVITE9 EQ 109) AND (P7.AP9JOUR EQ 5)) [P7.VENDREDIAP3 = P7.AP3];
IF ((P7.10SUMAP3 GE 200) AND (P7.10SUMAP3 LT 300) AND (P7.QACTIVITE_10 EQ 110) AND (P7.AP10JOUR EQ 5)) [P7.VENDREDIAP3 = P7.AP3];
IF ((P7.11SUMAP3 GE 200) AND (P7.11SUMAP3 LT 300) AND (P7.QACTIVITE_11 EQ 111) AND (P7.AP11JOUR EQ 5)) [P7.VENDREDIAP3 = P7.AP3];
IF ((P7.12SUMAP3 GE 200) AND (P7.12SUMAP3 LT 300) AND (P7.QACTIVITE_12 EQ 112) AND (P7.AP12JOUR EQ 5)) [P7.VENDREDIAP3 = P7.AP3];
IF ((P7.3SUMAP3 GE 200) AND (P7.3SUMAP3 LT 300) AND (P7.QACTIVITE3 EQ 103) AND (P7.AP3JOUR EQ 6)) [P7.SAMEDIAP3 = P7.AP3];
IF ((P7.4SUMAP3 GE 200) AND (P7.4SUMAP3 LT 300) AND (P7.QACTIVITE4 EQ 104) AND (P7.AP4JOUR EQ 6)) [P7.SAMEDIAP3 = P7.AP3];
IF ((P7.5SUMAP3 GE 200) AND (P7.5SUMAP3 LT 300) AND (P7.QACTIVITE5 EQ 105) AND (P7.AP5JOUR EQ 6)) [P7.SAMEDIAP3 = P7.AP3];
IF ((P7.6SUMAP3 GE 200) AND (P7.6SUMAP3 LT 300) AND (P7.QACTIVITE6 EQ 106) AND (P7.AP6JOUR EQ 6)) [P7.SAMEDIAP3 = P7.AP3];
IF ((P7.7SUMAP3 GE 200) AND (P7.7SUMAP3 LT 300) AND (P7.QACTIVITE7 EQ 107) AND (P7.AP7JOUR EQ 6)) [P7.SAMEDIAP3 = P7.AP3];
IF ((P7.8SUMAP3 GE 200) AND (P7.8SUMAP3 LT 300) AND (P7.QACTIVITE8 EQ 108) AND (P7.AP8JOUR EQ 6)) [P7.SAMEDIAP3 = P7.AP3];
IF ((P7.9SUMAP3 GE 200) AND (P7.9SUMAP3 LT 300) AND (P7.QACTIVITE9 EQ 109) AND (P7.AP9JOUR EQ 6)) [P7.SAMEDIAP3 = P7.AP3];
IF ((P7.10SUMAP3 GE 200) AND (P7.10SUMAP3 LT 300) AND (P7.QACTIVITE_10 EQ 110) AND (P7.AP10JOUR EQ 6)) [P7.SAMEDIAP3 = P7.AP3];
IF ((P7.11SUMAP3 GE 200) AND (P7.11SUMAP3 LT 300) AND (P7.QACTIVITE_11 EQ 111) AND (P7.AP11JOUR EQ 6)) [P7.SAMEDIAP3 = P7.AP3];
IF ((P7.12SUMAP3 GE 200) AND (P7.12SUMAP3 LT 300) AND (P7.QACTIVITE_12 EQ 112) AND (P7.AP12JOUR EQ 6)) [P7.SAMEDIAP3 = P7.AP3];
IF ((P7.3SUMAP3 GE 200) AND (P7.3SUMAP3 LT 300) AND (P7.QACTIVITE3 EQ 103) AND (P7.AP3JOUR EQ 7)) [P7.DIMANCHEAP3 = P7.AP3];
IF ((P7.4SUMAP3 GE 200) AND (P7.4SUMAP3 LT 300) AND (P7.QACTIVITE4 EQ 104) AND (P7.AP4JOUR EQ 7)) [P7.DIMANCHEAP3 = P7.AP3];
IF ((P7.5SUMAP3 GE 200) AND (P7.5SUMAP3 LT 300) AND (P7.QACTIVITE5 EQ 105) AND (P7.AP5JOUR EQ 7)) [P7.DIMANCHEAP3 = P7.AP3];
IF ((P7.6SUMAP3 GE 200) AND (P7.6SUMAP3 LT 300) AND (P7.QACTIVITE6 EQ 106) AND (P7.AP6JOUR EQ 7)) [P7.DIMANCHEAP3 = P7.AP3];
IF ((P7.7SUMAP3 GE 200) AND (P7.7SUMAP3 LT 300) AND (P7.QACTIVITE7 EQ 107) AND (P7.AP7JOUR EQ 7)) [P7.DIMANCHEAP3 = P7.AP3];
IF ((P7.8SUMAP3 GE 200) AND (P7.8SUMAP3 LT 300) AND (P7.QACTIVITE8 EQ 108) AND (P7.AP8JOUR EQ 7)) [P7.DIMANCHEAP3 = P7.AP3];
IF ((P7.9SUMAP3 GE 200) AND (P7.9SUMAP3 LT 300) AND (P7.QACTIVITE9 EQ 109) AND (P7.AP9JOUR EQ 7)) [P7.DIMANCHEAP3 = P7.AP3];

IF ((P7.10SUMAP3 GE 200) AND (P7.10SUMAP3 LT 300) AND (P7.QACTIVITE_10 EQ 110) AND (P7.AP10JOUR EQ 7)) [P7.DIMANCHEAP3 = P7.AP3];
IF ((P7.11SUMAP3 GE 200) AND (P7.11SUMAP3 LT 300) AND (P7.QACTIVITE_11 EQ 111) AND (P7.AP11JOUR EQ 7)) [P7.DIMANCHEAP3 = P7.AP3];
IF ((P7.12SUMAP3 GE 200) AND (P7.12SUMAP3 LT 300) AND (P7.QACTIVITE_12 EQ 112) AND (P7.AP12JOUR EQ 7)) [P7.DIMANCHEAP3 = P7.AP3];
POURSUITE DU CODE
IF (P7.P7BARCHOICE EQ 101) [P7.BAR = 'Être trop fatigué ou fatiguée pour faire de l´activité physique'];
IF (P7.P7BARCHOICE EQ 102) [P7.BAR = 'Avoir beaucoup de choses à faire mis à part l´activité physique'];
IF (P7.P7BARCHOICE EQ 103) [P7.BAR = 'température sera trop mauvaise pour faire de l´activité physique'];
IF (P7.P7BARCHOICE EQ 104) [P7.BAR = 'Ne pas avoir personne avec qui faire de l´activité physique'];
IF (P7.P7BARCHOICE EQ 105) [P7.BAR = 'Ne pas avoir accès à des emplacements pour faire de l´activité physique'];
IF (P7.P7BARCHOICE EQ 106) [P7.BAR = 'Ne pas avoir d´argent pour faire de l´activité physique'];
IF (P7.P7BARCHOICE EQ 107) [P7.BAR = 'Être trop gêné ou gênée, manquer de confiance pour aller faire de l´activité physique'];
IF (P7.P7BARCHOICE EQ 108) [P7.BAR = 'Avoir peur d´être insuffisamment en santé pour faire de l´activité physique'];
IF (P7.P7BARCHOICE EQ 109) [P7.BAR = 'Manquer de motivation'];
IF ((P7.P7BARCHOICE EQ 110) AND (P7.Q_BARANSWER NE 0)) [P7.BAR = P7.Q_BARANSWER];
IF (P7.SUMSOL EQ 101) [P7.SOL = 'Je vais faire de l´activité tôt dans la journée, en matinée ou à l´heure du midi.'];
IF (P7.SUMSOL EQ 102) [P7.SOL = 'J´essaie de garder en tête que si je fais de l´activité physique, j´aurai de plus en plus d´énergie dans la vie.'];
IF (P7.SUMSOL EQ 103) [P7.SOL = 'Je vais planifier des activités avec d´autres personnes qui me motiveront.'];
IF (P7.SUMSOL EQ 104) [P7.SOL = 'Je prends une collation pour me donner de l´énergie et j´y vais quand même.'];
IF (P7.SUMSOL EQ 201) [P7.SOL = 'Faire de l´activité physique par bloc de 10 minutes les journées où je n´ai pas de temps.'];
IF (P7.SUMSOL EQ 202) [P7.SOL = 'Je vais remplacer des activités où je suis assis ou assise, télé, ordinateur ou autres, par des activités physiques.'];
IF (P7.SUMSOL EQ 203) [P7.SOL = 'Je vais me procurer un agenda et inscrire dans mon horaire les moments où je vais faire de l´activité physique.'];
IF (P7.SUMSOL EQ 204) [P7.SOL = 'Je vais prendre des pauses de travail active et faire des 10 minutes de marche.'];
IF (P7.SUMSOL EQ 205) [P7.SOL = 'Je vais me fixer des moments avec d´autres personnes qui me plaisent pour aller faire de l´activité physique.'];
IF (P7.SUMSOL EQ 301) [P7.SOL = 'Je vais me procurer l´équipement nécessaire pour en faire même quand il pleut, quand il neige ou quand il fait trop chaud.'];
IF (P7.SUMSOL EQ 302) [P7.SOL = 'Je vais faire de l’activité physique chez nous ou à l´intérieur dans un endroit que j´aurai choisi quand il fait moins beau.'];

IF (P7.SUMSOL EQ 303) [P7.SOL = 'Je vais me préparer une activité physique alternative au cas où il serait désagréable d´aller dehors.'];
IF (P7.SUMSOL EQ 304) [P7.SOL = 'Je vais me dresser une liste des activités que je serais prêtE à faire si la température est mauvaise.'];
IF (P7.SUMSOL EQ 401) [P7.SOL = 'Je vais me joindre à un groupe qui pratique l´activité physique que j´aime. Ex : la marche, l´aquaforme ou la natation.'];
IF (P7.SUMSOL EQ 402) [P7.SOL = 'Je vais inviter mes amis, ma famille ou mes collègues à faire de l´activité physique avec moi.'];
IF (P7.SUMSOL EQ 403) [P7.SOL = 'Je vais amener mon chien marcher avec moi.'];
IF (P7.SUMSOL EQ 404) [P7.SOL = 'Je vais participer dans des discussions en ligne sur des pages facebook : Diabète Québec et autres.'];
IF (P7.SUMSOL EQ 501) [P7.SOL = 'Je vais faire de la marche ou du vélo dehors, c´est simple et gratuit.'];
IF (P7.SUMSOL EQ 502) [P7.SOL = 'Je vais faire de l´activité physique dans ma maison comme du yoga, un programme sur DVD, vélo stationnaire, tapis roulant, etc.'];
IF (P7.SUMSOL EQ 503) [P7.SOL = 'Je vais aller voir au centre communautaire de mon quartier pour me donner des options.'];
IF (P7.SUMSOL EQ 504) [P7.SOL = 'Je vais discuter avec mon médecin, avec un ami ou un spécialiste de l´activité physique pour avoir des conseils.'];
IF (P7.SUMSOL EQ 601) [P7.SOL = 'Je vais trouver des activités physiques abordables et simples : marcher dehors, faire du vélo, nager à la piscine communautaire.'];
IF (P7.SUMSOL EQ 602) [P7.SOL = 'Je vais aller voir au centre communautaire de mon quartier pour me donner des options.'];
IF (P7.SUMSOL EQ 603) [P7.SOL = 'Je vais économiser pour m´acheter un DVD d´activité physique ou pour une autre activité physique que j´aimerais faire.'];
IF (P7.SUMSOL EQ 604) [P7.SOL = 'Je vais aller marcher avec ma famille, des amis ou des collègues.'];
IF (P7.SUMSOL EQ 701) [P7.SOL = 'Je vais choisir une activité physique simple comme la marche ou la bicyclette.'];
IF (P7.SUMSOL EQ 702) [P7.SOL = 'Je vais aller faire de l´activité physique avec des gens qui m´acceptent comme je suis, qui évitent de me juger.'];
IF (P7.SUMSOL EQ 703) [P7.SOL = 'Au départ, je vais faire de l´activité physique dans des endroits où je suis seul[LETTRE_E] pour prendre confiance.'];
IF (P7.SUMSOL EQ 704) [P7.SOL = 'Je vais éviter des endroits qui me gêne davantage comme les centres de conditionnement.'];
IF (P7.SUMSOL EQ 801) [P7.SOL = 'Je vais consulter mon médecin pour mettre au clair si je peux faire de l´activité physique.'];
IF (P7.SUMSOL EQ 802) [P7.SOL = 'Je vais consulter un spécialiste de l´activité physique pour qu´il me rassure et me conseille sur ce qui est sécuritaire pour moi.'];
IF (P7.SUMSOL EQ 803) [P7.SOL = 'Je vais commencer à petite dose. 10 à 20 minutes maximum d´activités physiques les jours où je suis actif, active.'];
IF (P7.SUMSOL EQ 804) [P7.SOL = 'Je vais faire de l´activité physique accompagnéE d´un spécialiste de l´activité physique.'];

IF (P7.SUMSOL EQ 805) [P7.SOL = 'Je vais faire de l´activité physique dans un groupe où un spécialiste de l´activité physique est présent.'];
IF (P7.SUMSOL EQ 901) [P7.SOL = 'Je vais signer un contrat papier personnel comme quoi je m´engage à être actif, active.'];
IF (P7.SUMSOL EQ 902) [P7.SOL = 'Je vais signer un contrat papier avec mes proches comme quoi je m´engage à être actif, active.'];
IF (P7.SUMSOL EQ 903) [P7.SOL = 'Je vais essayer de nouvelles activités physiques pour trouver celles qui me plaisent vraiment.'];
IF (P7.SUMSOL EQ 904) [P7.SOL = 'À chaque activité physique que je fais, je prends un temps pour me rappeler ce que cela va m´apporter de mieux dans ma vie.'];
IF (P7.SUMSOL EQ 905) [P7.SOL = 'Je vais faire de l´activité physique avec des gens que j´aime pour me motiver.'];
IF ((P7.SUMSOL EQ 999) AND (P7.P8QSOLOPEN NE 0)) [P7.SOL = P7.P8QSOLOPEN];
IF (P7.SUMSOL EQ 0) [P7.SOL = P7.P8QSOL_10];
PLAN DE LA semaine 8
formules p8
IF ((P8.QACTI12 EQ 112) AND (P8.QAOPEN NE 0)) [P8.QACTIVITE_12 = 112];
[P8.2SUMAP2 = P8.QACTIVITE1];
[P8.3SUMAP2 = P8.QACTIVITE1+P8.QACTIVITE2];
[P8.4SUMAP2 = P8.QACTIVITE1+P8.QACTIVITE2+P8.QACTIVITE3];
[P8.5SUMAP2 = P8.QACTIVITE1+P8.QACTIVITE2+P8.QACTIVITE3+P8.QACTIVITE4];
[P8.6SUMAP2 = P8.QACTIVITE1+P8.QACTIVITE2+P8.QACTIVITE3+P8.QACTIVITE4+P8.QACTIVITE5];
[P8.7SUMAP2 =
P8.QACTIVITE1+P8.QACTIVITE2+P8.QACTIVITE3+P8.QACTIVITE4+P8.QACTIVITE5+P8.QACTIVITE6];
[P8.8SUMAP2 =
P8.QACTIVITE1+P8.QACTIVITE2+P8.QACTIVITE3+P8.QACTIVITE4+P8.QACTIVITE5+P8.QACTIVITE6+P8.QACTIVITE7];
[P8.9SUMAP2 =
P8.QACTIVITE1+P8.QACTIVITE2+P8.QACTIVITE3+P8.QACTIVITE4+P8.QACTIVITE5+P8.QACTIVITE6+P8.QACTIVITE7+P8.QACTIVITE8];
[P8.10SUMAP2 =
P8.QACTIVITE1+P8.QACTIVITE2+P8.QACTIVITE3+P8.QACTIVITE4+P8.QACTIVITE5+P8.QACTIVITE6+P8.QACTIVITE7+P8.QACTIVITE8+P8.QACTIVITE9];
[P8.11SUMAP2 =
P8.QACTIVITE1+P8.QACTIVITE2+P8.QACTIVITE3+P8.QACTIVITE4+P8.QACTIVITE5+P8.QACTIVITE6+P8.QACTIVITE7+P8.QACTIVITE8+P8.QACTIVITE9+P8.QACTIVITE_10];
[P8.12SUMAP2 =
P8.QACTIVITE1+P8.QACTIVITE2+P8.QACTIVITE3+P8.QACTIVITE4+P8.QACTIVITE5+P8.QACTIVITE6+P8.QACTIVITE7+P8.QACTIVITE8+P8.QACTIVITE9+P8.QACTIVITE_10+P8.QACTIVITE_11];
[P8.3SUMAP3 = P8.QACTIVITE1+P8.QACTIVITE2];
[P8.4SUMAP3 = P8.QACTIVITE1+P8.QACTIVITE2+P8.QACTIVITE3];
[P8.5SUMAP3 = P8.QACTIVITE1+P8.QACTIVITE2+P8.QACTIVITE3+P8.QACTIVITE4];

[P8.6SUMAP3 = P8.QACTIVITE1+P8.QACTIVITE2+P8.QACTIVITE3+P8.QACTIVITE4+P8.QACTIVITE5];
[P8.7SUMAP3 =
P8.QACTIVITE1+P8.QACTIVITE2+P8.QACTIVITE3+P8.QACTIVITE4+P8.QACTIVITE5+P8.QACTIVITE6];
[P8.8SUMAP3 =
P8.QACTIVITE1+P8.QACTIVITE2+P8.QACTIVITE3+P8.QACTIVITE4+P8.QACTIVITE5+P8.QACTIVITE6+P8.QACTI
VITE7];
[P8.9SUMAP3 =
P8.QACTIVITE1+P8.QACTIVITE2+P8.QACTIVITE3+P8.QACTIVITE4+P8.QACTIVITE5+P8.QACTIVITE6+P8.QACTI
VITE7+P8.QACTIVITE8];
[P8.10SUMAP3 =
P8.QACTIVITE1+P8.QACTIVITE2+P8.QACTIVITE3+P8.QACTIVITE4+P8.QACTIVITE5+P8.QACTIVITE6+P8.QACTI
VITE7+P8.QACTIVITE8+P8.QACTIVITE9];
[P8.11SUMAP3 =
P8.QACTIVITE1+P8.QACTIVITE2+P8.QACTIVITE3+P8.QACTIVITE4+P8.QACTIVITE5+P8.QACTIVITE6+P8.QACTI
VITE7+P8.QACTIVITE8+P8.QACTIVITE9+P8.QACTIVITE_10];
[P8.12SUMAP3 =
P8.QACTIVITE1+P8.QACTIVITE2+P8.QACTIVITE3+P8.QACTIVITE4+P8.QACTIVITE5+P8.QACTIVITE6+P8.QACTI
VITE7+P8.QACTIVITE8+P8.QACTIVITE9+P8.QACTIVITE_10+P8.QACTIVITE_11];
[P8.SUMAP =
P8.QACTIVITE1+P8.QACTIVITE2+P8.QACTIVITE3+P8.QACTIVITE4+P8.QACTIVITE5+P8.QACTIVITE6+P8.QACTI
VITE7+P8.QACTIVITE8+P8.QACTIVITE9+P8.QACTIVITE_10+P8.QACTIVITE_11+P8.QACTIVITE_12];
[P8.SUMSOL =
P8.P8QSOL1+P8.P8QSOL2+P8.P8QSOL3+P8.P8QSOL4+P8.P8QSOL5+P8.P8QSOL6+P8.P8QSOL7+P8.P8QSOL8
+P8.P8QSOL9];
IF (P8.QACTIVITE1 EQ 101) [P8.AP1 = 'marche rapide'];
IF ((P8.QACTIVITE1 NE 101) AND (P8.QACTIVITE2 EQ 102)) [P8.AP1 = 'raquettes à neige'];
IF ((P8.QACTIVITE1 NE 101) AND (P8.QACTIVITE2 NE 102) AND (P8.QACTIVITE3 EQ 103)) [P8.AP1 = 'vélo'];
IF ((P8.QACTIVITE1 NE 101) AND (P8.QACTIVITE2 NE 102) AND (P8.QACTIVITE3 NE 103) AND
(P8.QACTIVITE4 EQ 104)) [P8.AP1 = 'natation'];
IF ((P8.QACTIVITE1 NE 101) AND (P8.QACTIVITE2 NE 102) AND (P8.QACTIVITE3 NE 103) AND
(P8.QACTIVITE4 NE 104) AND (P8.QACTIVITE5 EQ 105)) [P8.AP1 = 'ski de fond'];
IF ((P8.QACTIVITE1 NE 101) AND (P8.QACTIVITE2 NE 102) AND (P8.QACTIVITE3 NE 103) AND
(P8.QACTIVITE4 NE 104) AND (P8.QACTIVITE5 NE 105) AND (P8.QACTIVITE6 EQ 106)) [P8.AP1 = 'tennis'];
IF ((P8.QACTIVITE1 NE 101) AND (P8.QACTIVITE2 NE 102) AND (P8.QACTIVITE3 NE 103) AND
(P8.QACTIVITE4 NE 104) AND (P8.QACTIVITE5 NE 105) AND (P8.QACTIVITE6 NE 106) AND (P8.QACTIVITE7
EQ 107)) [P8.AP1 = 'course à pieds'];
IF ((P8.QACTIVITE1 NE 101) AND (P8.QACTIVITE2 NE 102) AND (P8.QACTIVITE3 NE 103) AND
(P8.QACTIVITE4 NE 104) AND (P8.QACTIVITE5 NE 105) AND (P8.QACTIVITE6 NE 106) AND (P8.QACTIVITE7
NE 107) AND (P8.QACTIVITE8 EQ 108)) [P8.AP1 = 'hockey'];
IF ((P8.QACTIVITE1 NE 101) AND (P8.QACTIVITE2 NE 102) AND (P8.QACTIVITE3 NE 103) AND
(P8.QACTIVITE4 NE 104) AND (P8.QACTIVITE5 NE 105) AND (P8.QACTIVITE6 NE 106) AND (P8.QACTIVITE7
NE 107) AND (P8.QACTIVITE8 NE 108) AND (P8.QACTIVITE9 EQ 109)) [P8.AP1 = 'basketball'];

IF ((P8.QACTIVITE1 NE 101) AND (P8.QACTIVITE2 NE 102) AND (P8.QACTIVITE3 NE 103) AND (P8.QACTIVITE4 NE 104) AND (P8.QACTIVITE5 NE 105) AND (P8.QACTIVITE6 NE 106) AND (P8.QACTIVITE7 NE 107) AND (P8.QACTIVITE8 NE 108) AND (P8.QACTIVITE9 NE 109) AND (P8.QACTIVITE_10 EQ 110)) [P8.AP1 = 'soccer'];
IF ((P8.QACTIVITE1 NE 101) AND (P8.QACTIVITE2 NE 102) AND (P8.QACTIVITE3 NE 103) AND (P8.QACTIVITE4 NE 104) AND (P8.QACTIVITE5 NE 105) AND (P8.QACTIVITE6 NE 106) AND (P8.QACTIVITE7 NE 107) AND (P8.QACTIVITE8 NE 108) AND (P8.QACTIVITE9 NE 109) AND (P8.QACTIVITE_10 NE 110) AND (P8.QACTIVITE_11 EQ 111)) [P8.AP1 = 'centre de conditionnement'];
IF ((P8.QACTIVITE1 NE 101) AND (P8.QACTIVITE2 NE 102) AND (P8.QACTIVITE3 NE 103) AND (P8.QACTIVITE4 NE 104) AND (P8.QACTIVITE5 NE 105) AND (P8.QACTIVITE6 NE 106) AND (P8.QACTIVITE7 NE 107) AND (P8.QACTIVITE8 NE 108) AND (P8.QACTIVITE9 NE 109) AND (P8.QACTIVITE_10 NE 110) AND (P8.QACTIVITE_11 NE 111) AND (P8.QACTIVITE_12 EQ 112)) [P8.AP1 = P8.QAOPEN];
IF ((P8.2SUMAP2 GE 100) AND (P8.2SUMAP2 LT 200) AND (P8.QACTIVITE2 EQ 102)) [P8.AP2 = 'raquettes à neige'];
IF ((P8.3SUMAP2 GE 100) AND (P8.3SUMAP2 LT 200) AND (P8.QACTIVITE3 EQ 103)) [P8.AP2 = 'vélo'];
IF ((P8.4SUMAP2 GE 100) AND (P8.4SUMAP2 LT 200) AND (P8.QACTIVITE4 EQ 104)) [P8.AP2 = 'natation'];
IF ((P8.5SUMAP2 GE 100) AND (P8.5SUMAP2 LT 200) AND (P8.QACTIVITE5 EQ 105)) [P8.AP2 = 'ski de fond'];
IF ((P8.6SUMAP2 GE 100) AND (P8.6SUMAP2 LT 200) AND (P8.QACTIVITE6 EQ 106)) [P8.AP2 = 'tennis'];
IF ((P8.7SUMAP2 GE 100) AND (P8.7SUMAP2 LT 200) AND (P8.QACTIVITE7 EQ 107)) [P8.AP2 = 'course à pieds'];
IF ((P8.8SUMAP2 GE 100) AND (P8.8SUMAP2 LT 200) AND (P8.QACTIVITE8 EQ 108)) [P8.AP2 = 'hockey'];
IF ((P8.9SUMAP2 GE 100) AND (P8.9SUMAP2 LT 200) AND (P8.QACTIVITE9 EQ 109)) [P8.AP2 = 'basketball'];
IF ((P8.10SUMAP2 GE 100) AND (P8.10SUMAP2 LT 200) AND (P8.QACTIVITE_10 EQ 110)) [P8.AP2 = 'soccer'];
IF ((P8.11SUMAP2 GE 100) AND (P8.11SUMAP2 LT 200) AND (P8.QACTIVITE_11 EQ 111)) [P8.AP2 = 'centre de conditionnement'];
IF ((P8.12SUMAP2 GE 100) AND (P8.12SUMAP2 LT 200) AND (P8.QACTIVITE_12 EQ 112)) [P8.AP2 = P8.QAOPEN];
IF ((P8.3SUMAP3 GE 200) AND (P8.3SUMAP3 LT 300) AND (P8.QACTIVITE3 EQ 103)) [P8.AP3 = 'vélo'];
IF ((P8.4SUMAP3 GE 200) AND (P8.4SUMAP3 LT 300) AND (P8.QACTIVITE4 EQ 104)) [P8.AP3 = 'natation'];
IF ((P8.5SUMAP3 GE 200) AND (P8.5SUMAP3 LT 300) AND (P8.QACTIVITE5 EQ 105)) [P8.AP3 = 'ski de fond'];
IF ((P8.6SUMAP3 GE 200) AND (P8.6SUMAP3 LT 300) AND (P8.QACTIVITE6 EQ 106)) [P8.AP3 = 'tennis'];
IF ((P8.7SUMAP3 GE 200) AND (P8.7SUMAP3 LT 300) AND (P8.QACTIVITE7 EQ 107)) [P8.AP3 = 'course à pieds'];
IF ((P8.8SUMAP3 GE 200) AND (P8.8SUMAP3 LT 300) AND (P8.QACTIVITE8 EQ 108)) [P8.AP3 = 'hockey'];
IF ((P8.9SUMAP3 GE 200) AND (P8.9SUMAP3 LT 300) AND (P8.QACTIVITE9 EQ 109)) [P8.AP3 = 'basketball'];
IF ((P8.10SUMAP3 GE 200) AND (P8.10SUMAP3 LT 300) AND (P8.QACTIVITE_10 EQ 110)) [P8.AP3 = 'soccer'];
IF ((P8.11SUMAP3 GE 200) AND (P8.11SUMAP3 LT 300) AND (P8.QACTIVITE_11 EQ 111)) [P8.AP3 = 'centre de conditionnement'];
IF ((P8.12SUMAP3 GE 200) AND (P8.12SUMAP3 LT 300) AND (P8.QACTIVITE_12 EQ 112)) [P8.AP3 = P8.QAOPEN];

ACTIVITÉ 1 DANS LE PLAN POUR CHAQUE JOUR DE LA SEMAINE
IF ((P8.QACTIVITE1 EQ 101) AND (P8.AP1JOUR EQ 1)) [P8.LUNDIAP1 = P8.AP1];
IF ((P8.QACTIVITE1 NE 101) AND (P8.QACTIVITE2 EQ 102) AND (P8.AP2JOUR EQ 1)) [P8.LUNDIAP1 = P8.AP1];
IF ((P8.QACTIVITE1 NE 101) AND (P8.QACTIVITE2 NE 102) AND (P8.QACTIVITE3 EQ 103) AND (P8.AP3JOUR EQ 1)) [P8.LUNDIAP1 = P8.AP1];
IF ((P8.QACTIVITE1 NE 101) AND (P8.QACTIVITE2 NE 102) AND (P8.QACTIVITE3 NE 103) AND (P8.QACTIVITE4 EQ 104) AND (P8.AP4JOUR EQ 1)) [P8.LUNDIAP1 = P8.AP1];
IF ((P8.QACTIVITE1 NE 101) AND (P8.QACTIVITE2 NE 102) AND (P8.QACTIVITE3 NE 103) AND (P8.QACTIVITE4 NE 104) AND (P8.QACTIVITE5 EQ 105) AND (P8.AP5JOUR EQ 1)) [P8.LUNDIAP1 = P8.AP1];
IF ((P8.QACTIVITE1 NE 101) AND (P8.QACTIVITE2 NE 102) AND (P8.QACTIVITE3 NE 103) AND (P8.QACTIVITE4 NE 104) AND (P8.QACTIVITE5 NE 105) AND (P8.QACTIVITE6 EQ 106) AND (P8.AP6JOUR EQ 1)) [P8.LUNDIAP1 = P8.AP1];
IF ((P8.QACTIVITE1 NE 101) AND (P8.QACTIVITE2 NE 102) AND (P8.QACTIVITE3 NE 103) AND (P8.QACTIVITE4 NE 104) AND (P8.QACTIVITE5 NE 105) AND (P8.QACTIVITE6 NE 106) AND (P8.QACTIVITE7 EQ 107) AND (P8.AP7JOUR EQ 1)) [P8.LUNDIAP1 = P8.AP1];
IF ((P8.QACTIVITE1 NE 101) AND (P8.QACTIVITE2 NE 102) AND (P8.QACTIVITE3 NE 103) AND (P8.QACTIVITE4 NE 104) AND (P8.QACTIVITE5 NE 105) AND (P8.QACTIVITE6 NE 106) AND (P8.QACTIVITE7 NE 107) AND (P8.QACTIVITE8 EQ 108) AND (P8.AP8JOUR EQ 1)) [P8.LUNDIAP1 = P8.AP1];
IF ((P8.QACTIVITE1 NE 101) AND (P8.QACTIVITE2 NE 102) AND (P8.QACTIVITE3 NE 103) AND (P8.QACTIVITE4 NE 104) AND (P8.QACTIVITE5 NE 105) AND (P8.QACTIVITE6 NE 106) AND (P8.QACTIVITE7 NE 107) AND (P8.QACTIVITE8 NE 108) AND (P8.QACTIVITE9 EQ 109) AND (P8.AP9JOUR EQ 1)) [P8.LUNDIAP1 = P8.AP1];
IF ((P8.QACTIVITE1 NE 101) AND (P8.QACTIVITE2 NE 102) AND (P8.QACTIVITE3 NE 103) AND (P8.QACTIVITE4 NE 104) AND (P8.QACTIVITE5 NE 105) AND (P8.QACTIVITE6 NE 106) AND (P8.QACTIVITE7 NE 107) AND (P8.QACTIVITE8 NE 108) AND (P8.QACTIVITE9 NE 109) AND (P8.QACTIVITE_10 EQ 110) AND (P8.AP10JOUR EQ 1)) [P8.LUNDIAP1 = P8.AP1];
IF ((P8.QACTIVITE1 NE 101) AND (P8.QACTIVITE2 NE 102) AND (P8.QACTIVITE3 NE 103) AND (P8.QACTIVITE4 NE 104) AND (P8.QACTIVITE5 NE 105) AND (P8.QACTIVITE6 NE 106) AND (P8.QACTIVITE7 NE 107) AND (P8.QACTIVITE8 NE 108) AND (P8.QACTIVITE9 NE 109) AND (P8.QACTIVITE_10 NE 110) AND (P8.QACTIVITE_11 EQ 111) AND (P8.AP11JOUR EQ 1)) [P8.LUNDIAP1 = P8.AP1];
IF ((P8.QACTIVITE1 NE 101) AND (P8.QACTIVITE2 NE 102) AND (P8.QACTIVITE3 NE 103) AND (P8.QACTIVITE4 NE 104) AND (P8.QACTIVITE5 NE 105) AND (P8.QACTIVITE6 NE 106) AND (P8.QACTIVITE7 NE 107) AND (P8.QACTIVITE8 NE 108) AND (P8.QACTIVITE9 NE 109) AND (P8.QACTIVITE_10 NE 110) AND (P8.QACTIVITE_11 NE 111) AND (P8.QACTIVITE_12 EQ 112) AND (P8.AP12JOUR EQ 1)) [P8.LUNDIAP1 = P8.AP1];
IF ((P8.QACTIVITE1 EQ 101) AND (P8.AP1JOUR EQ 2)) [P8.MARDIAP1 = P8.AP1];
IF ((P8.QACTIVITE1 NE 101) AND (P8.QACTIVITE2 EQ 102) AND (P8.AP2JOUR EQ 2)) [P8.MARDIAP1 = P8.AP1];
IF ((P8.QACTIVITE1 NE 101) AND (P8.QACTIVITE2 NE 102) AND (P8.QACTIVITE3 EQ 103) AND (P8.AP3JOUR EQ 2)) [P8.MARDIAP1 = P8.AP1];

IF ((P8.QACTIVITE1 NE 101) AND (P8.QACTIVITE2 NE 102) AND (P8.QACTIVITE3 NE 103) AND (P8.QACTIVITE4 EQ 104) AND (P8.AP4JOUR EQ 2)) [P8.MARDIAP1 = P8.AP1];
IF ((P8.QACTIVITE1 NE 101) AND (P8.QACTIVITE2 NE 102) AND (P8.QACTIVITE3 NE 103) AND (P8.QACTIVITE4 NE 104) AND (P8.QACTIVITE5 EQ 105) AND (P8.AP5JOUR EQ 2)) [P8.MARDIAP1 = P8.AP1];
IF ((P8.QACTIVITE1 NE 101) AND (P8.QACTIVITE2 NE 102) AND (P8.QACTIVITE3 NE 103) AND (P8.QACTIVITE4 NE 104) AND (P8.QACTIVITE5 NE 105) AND (P8.QACTIVITE6 EQ 106) AND (P8.AP6JOUR EQ 2)) [P8.MARDIAP1 = P8.AP1];
IF ((P8.QACTIVITE1 NE 101) AND (P8.QACTIVITE2 NE 102) AND (P8.QACTIVITE3 NE 103) AND (P8.QACTIVITE4 NE 104) AND (P8.QACTIVITE5 NE 105) AND (P8.QACTIVITE6 NE 106) AND (P8.QACTIVITE7 EQ 107) AND (P8.AP7JOUR EQ 2)) [P8.MARDIAP1 = P8.AP1];
IF ((P8.QACTIVITE1 NE 101) AND (P8.QACTIVITE2 NE 102) AND (P8.QACTIVITE3 NE 103) AND (P8.QACTIVITE4 NE 104) AND (P8.QACTIVITE5 NE 105) AND (P8.QACTIVITE6 NE 106) AND (P8.QACTIVITE7 NE 107) AND (P8.QACTIVITE8 EQ 108) AND (P8.AP8JOUR EQ 2)) [P8.MARDIAP1 = P8.AP1];
IF ((P8.QACTIVITE1 NE 101) AND (P8.QACTIVITE2 NE 102) AND (P8.QACTIVITE3 NE 103) AND (P8.QACTIVITE4 NE 104) AND (P8.QACTIVITE5 NE 105) AND (P8.QACTIVITE6 NE 106) AND (P8.QACTIVITE7 NE 107) AND (P8.QACTIVITE8 NE 108) AND (P8.QACTIVITE9 EQ 109) AND (P8.AP9JOUR EQ 2)) [P8.MARDIAP1 = P8.AP1];
IF ((P8.QACTIVITE1 NE 101) AND (P8.QACTIVITE2 NE 102) AND (P8.QACTIVITE3 NE 103) AND (P8.QACTIVITE4 NE 104) AND (P8.QACTIVITE5 NE 105) AND (P8.QACTIVITE6 NE 106) AND (P8.QACTIVITE7 NE 107) AND (P8.QACTIVITE8 NE 108) AND (P8.QACTIVITE9 NE 109) AND (P8.QACTIVITE_10 EQ 110) AND (P8.AP10JOUR EQ 2)) [P8.MARDIAP1 = P8.AP1];
IF ((P8.QACTIVITE1 NE 101) AND (P8.QACTIVITE2 NE 102) AND (P8.QACTIVITE3 NE 103) AND (P8.QACTIVITE4 NE 104) AND (P8.QACTIVITE5 NE 105) AND (P8.QACTIVITE6 NE 106) AND (P8.QACTIVITE7 NE 107) AND (P8.QACTIVITE8 NE 108) AND (P8.QACTIVITE9 NE 109) AND (P8.QACTIVITE_10 NE 110) AND (P8.QACTIVITE_11 EQ 111) AND (P8.AP11JOUR EQ 2)) [P8.MARDIAP1 = P8.AP1];
IF ((P8.QACTIVITE1 NE 101) AND (P8.QACTIVITE2 NE 102) AND (P8.QACTIVITE3 NE 103) AND (P8.QACTIVITE4 NE 104) AND (P8.QACTIVITE5 NE 105) AND (P8.QACTIVITE6 NE 106) AND (P8.QACTIVITE7 NE 107) AND (P8.QACTIVITE8 NE 108) AND (P8.QACTIVITE9 NE 109) AND (P8.QACTIVITE_10 NE 110) AND (P8.QACTIVITE_11 NE 111) AND (P8.QACTIVITE_12 EQ 112) AND (P8.AP12JOUR EQ 2)) [P8.MARDIAP1 = P8.AP1];
IF ((P8.QACTIVITE1 EQ 101) AND (P8.AP1JOUR EQ 3)) [P8.MERCREDIAP1 = P8.AP1];
IF ((P8.QACTIVITE1 NE 101) AND (P8.QACTIVITE2 EQ 102) AND (P8.AP2JOUR EQ 3)) [P8.MERCREDIAP1 = P8.AP1];
IF ((P8.QACTIVITE1 NE 101) AND (P8.QACTIVITE2 NE 102) AND (P8.QACTIVITE3 EQ 103) AND (P8.AP3JOUR EQ 3)) [P8.MERCREDIAP1 = P8.AP1];
IF ((P8.QACTIVITE1 NE 101) AND (P8.QACTIVITE2 NE 102) AND (P8.QACTIVITE3 NE 103) AND (P8.QACTIVITE4 EQ 104) AND (P8.AP4JOUR EQ 3)) [P8.MERCREDIAP1 = P8.AP1];
IF ((P8.QACTIVITE1 NE 101) AND (P8.QACTIVITE2 NE 102) AND (P8.QACTIVITE3 NE 103) AND (P8.QACTIVITE4 NE 104) AND (P8.QACTIVITE5 EQ 105) AND (P8.AP5JOUR EQ 3)) [P8.MERCREDIAP1 = P8.AP1];

IF ((P8.QACTIVITE1 NE 101) AND (P8.QACTIVITE2 NE 102) AND (P8.QACTIVITE3 NE 103) AND (P8.QACTIVITE4 NE 104) AND (P8.QACTIVITES5 NE 105) AND (P8.QACTIVITE6 EQ 106) AND (P8.AP6JOUR EQ 3)) [P8.MERCREDIAP1 = P8.AP1];
IF ((P8.QACTIVITE1 NE 101) AND (P8.QACTIVITE2 NE 102) AND (P8.QACTIVITE3 NE 103) AND (P8.QACTIVITE4 NE 104) AND (P8.QACTIVITES5 NE 105) AND (P8.QACTIVITE6 NE 106) AND (P8.QACTIVITE7 EQ 107) AND (P8.AP7JOUR EQ 3)) [P8.MERCREDIAP1 = P8.AP1];
IF ((P8.QACTIVITE1 NE 101) AND (P8.QACTIVITE2 NE 102) AND (P8.QACTIVITE3 NE 103) AND (P8.QACTIVITE4 NE 104) AND (P8.QACTIVITES5 NE 105) AND (P8.QACTIVITE6 NE 106) AND (P8.QACTIVITE7 NE 107) AND (P8.QACTIVITE8 EQ 108) AND (P8.AP8JOUR EQ 3)) [P8.MERCREDIAP1 = P8.AP1];
IF ((P8.QACTIVITE1 NE 101) AND (P8.QACTIVITE2 NE 102) AND (P8.QACTIVITE3 NE 103) AND (P8.QACTIVITE4 NE 104) AND (P8.QACTIVITES5 NE 105) AND (P8.QACTIVITE6 NE 106) AND (P8.QACTIVITE7 NE 107) AND (P8.QACTIVITE8 NE 108) AND (P8.QACTIVITE9 EQ 109) AND (P8.AP9JOUR EQ 3)) [P8.MERCREDIAP1 = P8.AP1];
IF ((P8.QACTIVITE1 NE 101) AND (P8.QACTIVITE2 NE 102) AND (P8.QACTIVITE3 NE 103) AND (P8.QACTIVITE4 NE 104) AND (P8.QACTIVITES5 NE 105) AND (P8.QACTIVITE6 NE 106) AND (P8.QACTIVITE7 NE 107) AND (P8.QACTIVITE8 NE 108) AND (P8.QACTIVITE9 NE 109) AND (P8.QACTIVITE_10 EQ 110) AND (P8.AP10JOUR EQ 3)) [P8.MERCREDIAP1 = P8.AP1];
IF ((P8.QACTIVITE1 NE 101) AND (P8.QACTIVITE2 NE 102) AND (P8.QACTIVITE3 NE 103) AND (P8.QACTIVITE4 NE 104) AND (P8.QACTIVITES5 NE 105) AND (P8.QACTIVITE6 NE 106) AND (P8.QACTIVITE7 NE 107) AND (P8.QACTIVITE8 NE 108) AND (P8.QACTIVITE9 NE 109) AND (P8.QACTIVITE_10 NE 110) AND (P8.QACTIVITE_11 EQ 111) AND (P8.AP11JOUR EQ 3)) [P8.MERCREDIAP1 = P8.AP1];
IF ((P8.QACTIVITE1 NE 101) AND (P8.QACTIVITE2 NE 102) AND (P8.QACTIVITE3 NE 103) AND (P8.QACTIVITE4 NE 104) AND (P8.QACTIVITES5 NE 105) AND (P8.QACTIVITE6 NE 106) AND (P8.QACTIVITE7 NE 107) AND (P8.QACTIVITE8 NE 108) AND (P8.QACTIVITE9 NE 109) AND (P8.QACTIVITE_10 NE 110) AND (P8.QACTIVITE_11 NE 111) AND (P8.QACTIVITE_12 EQ 112) AND (P8.AP12JOUR EQ 3)) [P8.MERCREDIAP1 = P8.AP1];
IF ((P8.QACTIVITE1 EQ 101) AND (P8.AP1JOUR EQ 4)) [P8.JEUDIAP1 = P8.AP1];
IF ((P8.QACTIVITE1 NE 101) AND (P8.QACTIVITE2 EQ 102) AND (P8.AP2JOUR EQ 4)) [P8.JEUDIAP1 = P8.AP1];
IF ((P8.QACTIVITE1 NE 101) AND (P8.QACTIVITE2 NE 102) AND (P8.QACTIVITE3 EQ 103) AND (P8.AP3JOUR EQ 4)) [P8.JEUDIAP1 = P8.AP1];
IF ((P8.QACTIVITE1 NE 101) AND (P8.QACTIVITE2 NE 102) AND (P8.QACTIVITE3 NE 103) AND (P8.QACTIVITE4 EQ 104) AND (P8.AP4JOUR EQ 4)) [P8.JEUDIAP1 = P8.AP1];
IF ((P8.QACTIVITE1 NE 101) AND (P8.QACTIVITE2 NE 102) AND (P8.QACTIVITE3 NE 103) AND (P8.QACTIVITE4 NE 104) AND (P8.QACTIVITES5 EQ 105) AND (P8.AP5JOUR EQ 4)) [P8.JEUDIAP1 = P8.AP1];
IF ((P8.QACTIVITE1 NE 101) AND (P8.QACTIVITE2 NE 102) AND (P8.QACTIVITE3 NE 103) AND (P8.QACTIVITE4 NE 104) AND (P8.QACTIVITES5 NE 105) AND (P8.QACTIVITE6 EQ 106) AND (P8.AP6JOUR EQ 4)) [P8.JEUDIAP1 = P8.AP1];
IF ((P8.QACTIVITE1 NE 101) AND (P8.QACTIVITE2 NE 102) AND (P8.QACTIVITE3 NE 103) AND (P8.QACTIVITE4 NE 104) AND (P8.QACTIVITES5 NE 105) AND (P8.QACTIVITE6 NE 106) AND (P8.QACTIVITE7 EQ 107) AND (P8.AP7JOUR EQ 4)) [P8.JEUDIAP1 = P8.AP1];

IF ((P8.QACTIVITE1 NE 101) AND (P8.QACTIVITE2 NE 102) AND (P8.QACTIVITE3 NE 103) AND (P8.QACTIVITE4 NE 104) AND (P8.QACTIVITES5 NE 105) AND (P8.QACTIVITE6 NE 106) AND (P8.QACTIVITE7 NE 107) AND (P8.QACTIVITE8 EQ 108) AND (P8.AP8JOUR EQ 4)) [P8.JEUDIAP1 = P8.AP1];
IF ((P8.QACTIVITE1 NE 101) AND (P8.QACTIVITE2 NE 102) AND (P8.QACTIVITE3 NE 103) AND (P8.QACTIVITE4 NE 104) AND (P8.QACTIVITES5 NE 105) AND (P8.QACTIVITE6 NE 106) AND (P8.QACTIVITE7 NE 107) AND (P8.QACTIVITE8 NE 108) AND (P8.QACTIVITE9 EQ 109) AND (P8.AP9JOUR EQ 4)) [P8.JEUDIAP1 = P8.AP1];
IF ((P8.QACTIVITE1 NE 101) AND (P8.QACTIVITE2 NE 102) AND (P8.QACTIVITE3 NE 103) AND (P8.QACTIVITE4 NE 104) AND (P8.QACTIVITES5 NE 105) AND (P8.QACTIVITE6 NE 106) AND (P8.QACTIVITE7 NE 107) AND (P8.QACTIVITE8 NE 108) AND (P8.QACTIVITE9 NE 109) AND (P8.QACTIVITE_10 EQ 110) AND (P8.AP10JOUR EQ 4)) [P8.JEUDIAP1 = P8.AP1];
IF ((P8.QACTIVITE1 NE 101) AND (P8.QACTIVITE2 NE 102) AND (P8.QACTIVITE3 NE 103) AND (P8.QACTIVITE4 NE 104) AND (P8.QACTIVITES5 NE 105) AND (P8.QACTIVITE6 NE 106) AND (P8.QACTIVITE7 NE 107) AND (P8.QACTIVITE8 NE 108) AND (P8.QACTIVITE9 NE 109) AND (P8.QACTIVITE_10 NE 110) AND (P8.QACTIVITE_11 EQ 111) AND (P8.AP11JOUR EQ 4)) [P8.JEUDIAP1 = P8.AP1];
IF ((P8.QACTIVITE1 NE 101) AND (P8.QACTIVITE2 NE 102) AND (P8.QACTIVITE3 NE 103) AND (P8.QACTIVITE4 NE 104) AND (P8.QACTIVITES5 NE 105) AND (P8.QACTIVITE6 NE 106) AND (P8.QACTIVITE7 NE 107) AND (P8.QACTIVITE8 NE 108) AND (P8.QACTIVITE9 NE 109) AND (P8.QACTIVITE_10 NE 110) AND (P8.QACTIVITE_11 NE 111) AND (P8.QACTIVITE_12 EQ 112) AND (P8.AP12JOUR EQ 4)) [P8.JEUDIAP1 = P8.AP1];
IF ((P8.QACTIVITE1 EQ 101) AND (P8.AP1JOUR EQ 5)) [P8.VENDREDIAP1 = P8.AP1];
IF ((P8.QACTIVITE1 NE 101) AND (P8.QACTIVITE2 EQ 102) AND (P8.AP2JOUR EQ 5)) [P8.VENDREDIAP1 = P8.AP1];
IF ((P8.QACTIVITE1 NE 101) AND (P8.QACTIVITE2 NE 102) AND (P8.QACTIVITE3 EQ 103) AND (P8.AP3JOUR EQ 5)) [P8.VENDREDIAP1 = P8.AP1];
IF ((P8.QACTIVITE1 NE 101) AND (P8.QACTIVITE2 NE 102) AND (P8.QACTIVITE3 NE 103) AND (P8.QACTIVITE4 EQ 104) AND (P8.AP4JOUR EQ 5)) [P8.VENDREDIAP1 = P8.AP1];
IF ((P8.QACTIVITE1 NE 101) AND (P8.QACTIVITE2 NE 102) AND (P8.QACTIVITE3 NE 103) AND (P8.QACTIVITE4 NE 104) AND (P8.QACTIVITES5 EQ 105) AND (P8.AP5JOUR EQ 5)) [P8.VENDREDIAP1 = P8.AP1];
IF ((P8.QACTIVITE1 NE 101) AND (P8.QACTIVITE2 NE 102) AND (P8.QACTIVITE3 NE 103) AND (P8.QACTIVITE4 NE 104) AND (P8.QACTIVITES5 NE 105) AND (P8.QACTIVITE6 EQ 106) AND (P8.AP6JOUR EQ 5)) [P8.VENDREDIAP1 = P8.AP1];
IF ((P8.QACTIVITE1 NE 101) AND (P8.QACTIVITE2 NE 102) AND (P8.QACTIVITE3 NE 103) AND (P8.QACTIVITE4 NE 104) AND (P8.QACTIVITES5 NE 105) AND (P8.QACTIVITE6 NE 106) AND (P8.QACTIVITE7 EQ 107) AND (P8.AP7JOUR EQ 5)) [P8.VENDREDIAP1 = P8.AP1];
IF ((P8.QACTIVITE1 NE 101) AND (P8.QACTIVITE2 NE 102) AND (P8.QACTIVITE3 NE 103) AND (P8.QACTIVITE4 NE 104) AND (P8.QACTIVITES5 NE 105) AND (P8.QACTIVITE6 NE 106) AND (P8.QACTIVITE7 NE 107) AND (P8.QACTIVITE8 EQ 108) AND (P8.AP8JOUR EQ 5)) [P8.VENDREDIAP1 = P8.AP1];
IF ((P8.QACTIVITE1 NE 101) AND (P8.QACTIVITE2 NE 102) AND (P8.QACTIVITE3 NE 103) AND (P8.QACTIVITE4 NE 104) AND (P8.QACTIVITES5 NE 105) AND (P8.QACTIVITE6 NE 106) AND (P8.QACTIVITE7

```
NE 107) AND (P8.QACTIVITE8 NE 108) AND (P8.QACTIVITE9 EQ 109) AND (P8.AP9JOUR EQ 5))
[P8.VENDREDIAP1 = P8.AP1];
IF ((P8.QACTIVITE1 NE 101) AND (P8.QACTIVITE2 NE 102) AND (P8.QACTIVITE3 NE 103) AND
(P8.QACTIVITE4 NE 104) AND (P8.QACTIVITE5 NE 105) AND (P8.QACTIVITE6 NE 106) AND (P8.QACTIVITE7
NE 107) AND (P8.QACTIVITE8 NE 108) AND (P8.QACTIVITE9 NE 109) AND (P8.QACTIVITE_10 EQ 110) AND
(P8.AP10JOUR EQ 5)) [P8.VENDREDIAP1 = P8.AP1];
IF ((P8.QACTIVITE1 NE 101) AND (P8.QACTIVITE2 NE 102) AND (P8.QACTIVITE3 NE 103) AND
(P8.QACTIVITE4 NE 104) AND (P8.QACTIVITE5 NE 105) AND (P8.QACTIVITE6 NE 106) AND (P8.QACTIVITE7
NE 107) AND (P8.QACTIVITE8 NE 108) AND (P8.QACTIVITE9 NE 109) AND (P8.QACTIVITE_10 NE 110) AND
(P8.QACTIVITE_11 EQ 111) AND (P8.AP11JOUR EQ 5)) [P8.VENDREDIAP1 = P8.AP1];
IF ((P8.QACTIVITE1 NE 101) AND (P8.QACTIVITE2 NE 102) AND (P8.QACTIVITE3 NE 103) AND
(P8.QACTIVITE4 NE 104) AND (P8.QACTIVITE5 NE 105) AND (P8.QACTIVITE6 NE 106) AND (P8.QACTIVITE7
NE 107) AND (P8.QACTIVITE8 NE 108) AND (P8.QACTIVITE9 NE 109) AND (P8.QACTIVITE_10 NE 110) AND
(P8.QACTIVITE_11 NE 111) AND (P8.QACTIVITE_12 EQ 112) AND (P8.AP12JOUR EQ 5)) [P8.VENDREDIAP1 =
P8.AP1];
IF ((P8.QACTIVITE1 EQ 101) AND (P8.AP1JOUR EQ 6)) [P8.SAMEDIAP1 = P8.AP1];
IF ((P8.QACTIVITE1 NE 101) AND (P8.QACTIVITE2 EQ 102) AND (P8.AP2JOUR EQ 6)) [P8.SAMEDIAP1 =
P8.AP1];
IF ((P8.QACTIVITE1 NE 101) AND (P8.QACTIVITE2 NE 102) AND (P8.QACTIVITE3 EQ 103) AND (P8.AP3JOUR
EQ 6)) [P8.SAMEDIAP1 = P8.AP1];
IF ((P8.QACTIVITE1 NE 101) AND (P8.QACTIVITE2 NE 102) AND (P8.QACTIVITE3 NE 103) AND
(P8.QACTIVITE4 EQ 104) AND (P8.AP4JOUR EQ 6)) [P8.SAMEDIAP1 = P8.AP1];
IF ((P8.QACTIVITE1 NE 101) AND (P8.QACTIVITE2 NE 102) AND (P8.QACTIVITE3 NE 103) AND
(P8.QACTIVITE4 NE 104) AND (P8.QACTIVITE5 EQ 105) AND (P8.AP5JOUR EQ 6)) [P8.SAMEDIAP1 = P8.AP1];
IF ((P8.QACTIVITE1 NE 101) AND (P8.QACTIVITE2 NE 102) AND (P8.QACTIVITE3 NE 103) AND
(P8.QACTIVITE4 NE 104) AND (P8.QACTIVITE5 NE 105) AND (P8.QACTIVITE6 EQ 106) AND (P8.AP6JOUR EQ
6)) [P8.SAMEDIAP1 = P8.AP1];
IF ((P8.QACTIVITE1 NE 101) AND (P8.QACTIVITE2 NE 102) AND (P8.QACTIVITE3 NE 103) AND
(P8.QACTIVITE4 NE 104) AND (P8.QACTIVITE5 NE 105) AND (P8.QACTIVITE6 NE 106) AND (P8.QACTIVITE7
EQ 107) AND (P8.AP7JOUR EQ 6)) [P8.SAMEDIAP1 = P8.AP1];
IF ((P8.QACTIVITE1 NE 101) AND (P8.QACTIVITE2 NE 102) AND (P8.QACTIVITE3 NE 103) AND
(P8.QACTIVITE4 NE 104) AND (P8.QACTIVITE5 NE 105) AND (P8.QACTIVITE6 NE 106) AND (P8.QACTIVITE7
NE 107) AND (P8.QACTIVITE8 EQ 108) AND (P8.AP8JOUR EQ 6)) [P8.SAMEDIAP1 = P8.AP1];
IF ((P8.QACTIVITE1 NE 101) AND (P8.QACTIVITE2 NE 102) AND (P8.QACTIVITE3 NE 103) AND
(P8.QACTIVITE4 NE 104) AND (P8.QACTIVITE5 NE 105) AND (P8.QACTIVITE6 NE 106) AND (P8.QACTIVITE7
NE 107) AND (P8.QACTIVITE8 NE 108) AND (P8.QACTIVITE9 EQ 109) AND (P8.AP9JOUR EQ 6))
[P8.SAMEDIAP1 = P8.AP1];
IF ((P8.QACTIVITE1 NE 101) AND (P8.QACTIVITE2 NE 102) AND (P8.QACTIVITE3 NE 103) AND
(P8.QACTIVITE4 NE 104) AND (P8.QACTIVITE5 NE 105) AND (P8.QACTIVITE6 NE 106) AND (P8.QACTIVITE7
NE 107) AND (P8.QACTIVITE8 NE 108) AND (P8.QACTIVITE9 NE 109) AND (P8.QACTIVITE_10 EQ 110) AND
(P8.AP10JOUR EQ 6)) [P8.SAMEDIAP1 = P8.AP1];
```

IF ((P8.QACTIVITE1 NE 101) AND (P8.QACTIVITE2 NE 102) AND (P8.QACTIVITE3 NE 103) AND (P8.QACTIVITE4 NE 104) AND (P8.QACTIVITES5 NE 105) AND (P8.QACTIVITE6 NE 106) AND (P8.QACTIVITE7 NE 107) AND (P8.QACTIVITE8 NE 108) AND (P8.QACTIVITE9 NE 109) AND (P8.QACTIVITE_10 NE 110) AND (P8.QACTIVITE_11 EQ 111) AND (P8.AP11JOUR EQ 6)) [P8.SAMEDIAP1 = P8.AP1];
IF ((P8.QACTIVITE1 NE 101) AND (P8.QACTIVITE2 NE 102) AND (P8.QACTIVITE3 NE 103) AND (P8.QACTIVITE4 NE 104) AND (P8.QACTIVITES5 NE 105) AND (P8.QACTIVITE6 NE 106) AND (P8.QACTIVITE7 NE 107) AND (P8.QACTIVITE8 NE 108) AND (P8.QACTIVITE9 NE 109) AND (P8.QACTIVITE_10 NE 110) AND (P8.QACTIVITE_11 NE 111) AND (P8.QACTIVITE_12 EQ 112) AND (P8.AP12JOUR EQ 6)) [P8.SAMEDIAP1 = P8.AP1];
IF ((P8.QACTIVITE1 EQ 101) AND (P8.AP1JOUR EQ 7)) [P8.DIMANCHEAP1 = P8.AP1];
IF ((P8.QACTIVITE1 NE 101) AND (P8.QACTIVITE2 EQ 102) AND (P8.AP2JOUR EQ 7)) [P8.DIMANCHEAP1 = P8.AP1];
IF ((P8.QACTIVITE1 NE 101) AND (P8.QACTIVITE2 NE 102) AND (P8.QACTIVITE3 EQ 103) AND (P8.AP3JOUR EQ 7)) [P8.DIMANCHEAP1 = P8.AP1];
IF ((P8.QACTIVITE1 NE 101) AND (P8.QACTIVITE2 NE 102) AND (P8.QACTIVITE3 NE 103) AND (P8.QACTIVITE4 EQ 104) AND (P8.AP4JOUR EQ 7)) [P8.DIMANCHEAP1 = P8.AP1];
IF ((P8.QACTIVITE1 NE 101) AND (P8.QACTIVITE2 NE 102) AND (P8.QACTIVITE3 NE 103) AND (P8.QACTIVITE4 NE 104) AND (P8.QACTIVITES5 EQ 105) AND (P8.AP5JOUR EQ 7)) [P8.DIMANCHEAP1 = P8.AP1];
IF ((P8.QACTIVITE1 NE 101) AND (P8.QACTIVITE2 NE 102) AND (P8.QACTIVITE3 NE 103) AND (P8.QACTIVITE4 NE 104) AND (P8.QACTIVITES5 NE 105) AND (P8.QACTIVITE6 EQ 106) AND (P8.AP6JOUR EQ 7)) [P8.DIMANCHEAP1 = P8.AP1];
IF ((P8.QACTIVITE1 NE 101) AND (P8.QACTIVITE2 NE 102) AND (P8.QACTIVITE3 NE 103) AND (P8.QACTIVITE4 NE 104) AND (P8.QACTIVITES5 NE 105) AND (P8.QACTIVITE6 NE 106) AND (P8.QACTIVITE7 EQ 107) AND (P8.AP7JOUR EQ 7)) [P8.DIMANCHEAP1 = P8.AP1];
IF ((P8.QACTIVITE1 NE 101) AND (P8.QACTIVITE2 NE 102) AND (P8.QACTIVITE3 NE 103) AND (P8.QACTIVITE4 NE 104) AND (P8.QACTIVITES5 NE 105) AND (P8.QACTIVITE6 NE 106) AND (P8.QACTIVITE7 NE 107) AND (P8.QACTIVITE8 EQ 108) AND (P8.AP8JOUR EQ 7)) [P8.DIMANCHEAP1 = P8.AP1];
IF ((P8.QACTIVITE1 NE 101) AND (P8.QACTIVITE2 NE 102) AND (P8.QACTIVITE3 NE 103) AND (P8.QACTIVITE4 NE 104) AND (P8.QACTIVITES5 NE 105) AND (P8.QACTIVITE6 NE 106) AND (P8.QACTIVITE7 NE 107) AND (P8.QACTIVITE8 NE 108) AND (P8.QACTIVITE9 EQ 109) AND (P8.AP9JOUR EQ 7)) [P8.DIMANCHEAP1 = P8.AP1];
IF ((P8.QACTIVITE1 NE 101) AND (P8.QACTIVITE2 NE 102) AND (P8.QACTIVITE3 NE 103) AND (P8.QACTIVITE4 NE 104) AND (P8.QACTIVITES5 NE 105) AND (P8.QACTIVITE6 NE 106) AND (P8.QACTIVITE7 NE 107) AND (P8.QACTIVITE8 NE 108) AND (P8.QACTIVITE9 NE 109) AND (P8.QACTIVITE_10 EQ 110) AND (P8.AP10JOUR EQ 7)) [P8.DIMANCHEAP1 = P8.AP1];
IF ((P8.QACTIVITE1 NE 101) AND (P8.QACTIVITE2 NE 102) AND (P8.QACTIVITE3 NE 103) AND (P8.QACTIVITE4 NE 104) AND (P8.QACTIVITES5 NE 105) AND (P8.QACTIVITE6 NE 106) AND (P8.QACTIVITE7 NE 107) AND (P8.QACTIVITE8 NE 108) AND (P8.QACTIVITE9 NE 109) AND (P8.QACTIVITE_10 NE 110) AND (P8.QACTIVITE_11 EQ 111) AND (P8.AP11JOUR EQ 7)) [P8.DIMANCHEAP1 = P8.AP1];
IF ((P8.QACTIVITE1 NE 101) AND (P8.QACTIVITE2 NE 102) AND (P8.QACTIVITE3 NE 103) AND (P8.QACTIVITE4 NE 104) AND (P8.QACTIVITES5 NE 105) AND (P8.QACTIVITE6 NE 106) AND (P8.QACTIVITE7

NE 107) AND (P8.QACTIVITE8 NE 108) AND (P8.QACTIVITE9 NE 109) AND (P8.QACTIVITE_10 NE 110) AND (P8.QACTIVITE_11 NE 111) AND (P8.QACTIVITE_12 EQ 112) AND (P8.AP12JOUR EQ 7)) [P8.DIMANCHEAP1 = P8.AP1];
ACTIVITÉ 2 DANS LE PLAN POUR CHAQUE JOUR DE LA SEMAINE
IF ((P8.2SUMAP2 GE 100) AND (P8.2SUMAP2 LT 200) AND (P8.QACTIVITE2 EQ 102) AND (P8.AP2JOUR EQ 1)) [P8.LUNDIAP2 = P8.AP2];
IF ((P8.3SUMAP2 GE 100) AND (P8.3SUMAP2 LT 200) AND (P8.QACTIVITE3 EQ 103) AND (P8.AP3JOUR EQ 1)) [P8.LUNDIAP2 = P8.AP2];
IF ((P8.4SUMAP2 GE 100) AND (P8.4SUMAP2 LT 200) AND (P8.QACTIVITE4 EQ 104) AND (P8.AP4JOUR EQ 1)) [P8.LUNDIAP2 = P8.AP2];
IF ((P8.5SUMAP2 GE 100) AND (P8.5SUMAP2 LT 200) AND (P8.QACTIVITE5 EQ 105) AND (P8.AP5JOUR EQ 1)) [P8.LUNDIAP2 = P8.AP2];
IF ((P8.6SUMAP2 GE 100) AND (P8.6SUMAP2 LT 200) AND (P8.QACTIVITE6 EQ 106) AND (P8.AP6JOUR EQ 1)) [P8.LUNDIAP2 = P8.AP2];
IF ((P8.7SUMAP2 GE 100) AND (P8.7SUMAP2 LT 200) AND (P8.QACTIVITE7 EQ 107) AND (P8.AP7JOUR EQ 1)) [P8.LUNDIAP2 = P8.AP2];
IF ((P8.8SUMAP2 GE 100) AND (P8.8SUMAP2 LT 200) AND (P8.QACTIVITE8 EQ 108) AND (P8.AP8JOUR EQ 1)) [P8.LUNDIAP2 = P8.AP2];
IF ((P8.9SUMAP2 GE 100) AND (P8.9SUMAP2 LT 200) AND (P8.QACTIVITE9 EQ 109) AND (P8.AP9JOUR EQ 1)) [P8.LUNDIAP2 = P8.AP2];
IF ((P8.10SUMAP2 GE 100) AND (P8.10SUMAP2 LT 200) AND (P8.QACTIVITE_10 EQ 110) AND (P8.AP10JOUR EQ 1)) [P8.LUNDIAP2 = P8.AP2];
IF ((P8.11SUMAP2 GE 100) AND (P8.11SUMAP2 LT 200) AND (P8.QACTIVITE_11 EQ 111) AND (P8.AP11JOUR EQ 1)) [P8.LUNDIAP2 = P8.AP2];
IF ((P8.12SUMAP2 GE 100) AND (P8.12SUMAP2 LT 200) AND (P8.QACTIVITE_12 EQ 112) AND (P8.AP12JOUR EQ 1)) [P8.LUNDIAP2 = P8.AP2];
IF ((P8.2SUMAP2 GE 100) AND (P8.2SUMAP2 LT 200) AND (P8.QACTIVITE2 EQ 102) AND (P8.AP2JOUR EQ 2)) [P8.MARDIAP2 = P8.AP2];
IF ((P8.3SUMAP2 GE 100) AND (P8.3SUMAP2 LT 200) AND (P8.QACTIVITE3 EQ 103) AND (P8.AP3JOUR EQ 2)) [P8.MARDIAP2 = P8.AP2];
IF ((P8.4SUMAP2 GE 100) AND (P8.4SUMAP2 LT 200) AND (P8.QACTIVITE4 EQ 104) AND (P8.AP4JOUR EQ 2)) [P8.MARDIAP2 = P8.AP2];
IF ((P8.5SUMAP2 GE 100) AND (P8.5SUMAP2 LT 200) AND (P8.QACTIVITE5 EQ 105) AND (P8.AP5JOUR EQ 2)) [P8.MARDIAP2 = P8.AP2];
IF ((P8.6SUMAP2 GE 100) AND (P8.6SUMAP2 LT 200) AND (P8.QACTIVITE6 EQ 106) AND (P8.AP6JOUR EQ 2)) [P8.MARDIAP2 = P8.AP2];
IF ((P8.7SUMAP2 GE 100) AND (P8.7SUMAP2 LT 200) AND (P8.QACTIVITE7 EQ 107) AND (P8.AP7JOUR EQ 2)) [P8.MARDIAP2 = P8.AP2];
IF ((P8.8SUMAP2 GE 100) AND (P8.8SUMAP2 LT 200) AND (P8.QACTIVITE8 EQ 108) AND (P8.AP8JOUR EQ 2)) [P8.MARDIAP2 = P8.AP2];
IF ((P8.9SUMAP2 GE 100) AND (P8.9SUMAP2 LT 200) AND (P8.QACTIVITE9 EQ 109) AND (P8.AP9JOUR EQ 2)) [P8.MARDIAP2 = P8.AP2];

IF ((P8.10SUMAP2 GE 100) AND (P8.10SUMAP2 LT 200) AND (P8.QACTIVITE_10 EQ 110) AND (P8.AP10JOUR EQ 2)) [P8.MARDIAP2 = P8.AP2];
IF ((P8.11SUMAP2 GE 100) AND (P8.11SUMAP2 LT 200) AND (P8.QACTIVITE_11 EQ 111) AND (P8.AP11JOUR EQ 2)) [P8.MARDIAP2 = P8.AP2];
IF ((P8.12SUMAP2 GE 100) AND (P8.12SUMAP2 LT 200) AND (P8.QACTIVITE_12 EQ 112) AND (P8.AP12JOUR EQ 2)) [P8.MARDIAP2 = P8.AP2];
IF ((P8.2SUMAP2 GE 100) AND (P8.2SUMAP2 LT 200) AND (P8.QACTIVITE2 EQ 102) AND (P8.AP2JOUR EQ 3)) [P8.MERCREDIAP2 = P8.AP2];
IF ((P8.3SUMAP2 GE 100) AND (P8.3SUMAP2 LT 200) AND (P8.QACTIVITE3 EQ 103) AND (P8.AP3JOUR EQ 3)) [P8.MERCREDIAP2 = P8.AP2];
IF ((P8.4SUMAP2 GE 100) AND (P8.4SUMAP2 LT 200) AND (P8.QACTIVITE4 EQ 104) AND (P8.AP4JOUR EQ 3)) [P8.MERCREDIAP2 = P8.AP2];
IF ((P8.5SUMAP2 GE 100) AND (P8.5SUMAP2 LT 200) AND (P8.QACTIVITE5 EQ 105) AND (P8.AP5JOUR EQ 3)) [P8.MERCREDIAP2 = P8.AP2];
IF ((P8.6SUMAP2 GE 100) AND (P8.6SUMAP2 LT 200) AND (P8.QACTIVITE6 EQ 106) AND (P8.AP6JOUR EQ 3)) [P8.MERCREDIAP2 = P8.AP2];
IF ((P8.7SUMAP2 GE 100) AND (P8.7SUMAP2 LT 200) AND (P8.QACTIVITE7 EQ 107) AND (P8.AP7JOUR EQ 3)) [P8.MERCREDIAP2 = P8.AP2];
IF ((P8.8SUMAP2 GE 100) AND (P8.8SUMAP2 LT 200) AND (P8.QACTIVITE8 EQ 108) AND (P8.AP8JOUR EQ 3)) [P8.MERCREDIAP2 = P8.AP2];
IF ((P8.9SUMAP2 GE 100) AND (P8.9SUMAP2 LT 200) AND (P8.QACTIVITE9 EQ 109) AND (P8.AP9JOUR EQ 3)) [P8.MERCREDIAP2 = P8.AP2];
IF ((P8.10SUMAP2 GE 100) AND (P8.10SUMAP2 LT 200) AND (P8.QACTIVITE_10 EQ 110) AND (P8.AP10JOUR EQ 3)) [P8.MERCREDIAP2 = P8.AP2];
IF ((P8.11SUMAP2 GE 100) AND (P8.11SUMAP2 LT 200) AND (P8.QACTIVITE_11 EQ 111) AND (P8.AP11JOUR EQ 3)) [P8.MERCREDIAP2 = P8.AP2];
IF ((P8.12SUMAP2 GE 100) AND (P8.12SUMAP2 LT 200) AND (P8.QACTIVITE_12 EQ 112) AND (P8.AP12JOUR EQ 3)) [P8.MERCREDIAP2 = P8.AP2];
IF ((P8.2SUMAP2 GE 100) AND (P8.2SUMAP2 LT 200) AND (P8.QACTIVITE2 EQ 102) AND (P8.AP2JOUR EQ 4)) [P8.JEUDIAP2 = P8.AP2];
IF ((P8.3SUMAP2 GE 100) AND (P8.3SUMAP2 LT 200) AND (P8.QACTIVITE3 EQ 103) AND (P8.AP3JOUR EQ 4)) [P8.JEUDIAP2 = P8.AP2];
IF ((P8.4SUMAP2 GE 100) AND (P8.4SUMAP2 LT 200) AND (P8.QACTIVITE4 EQ 104) AND (P8.AP4JOUR EQ 4)) [P8.JEUDIAP2 = P8.AP2];
IF ((P8.5SUMAP2 GE 100) AND (P8.5SUMAP2 LT 200) AND (P8.QACTIVITE5 EQ 105) AND (P8.AP5JOUR EQ 4)) [P8.JEUDIAP2 = P8.AP2];
IF ((P8.6SUMAP2 GE 100) AND (P8.6SUMAP2 LT 200) AND (P8.QACTIVITE6 EQ 106) AND (P8.AP6JOUR EQ 4)) [P8.JEUDIAP2 = P8.AP2];
IF ((P8.7SUMAP2 GE 100) AND (P8.7SUMAP2 LT 200) AND (P8.QACTIVITE7 EQ 107) AND (P8.AP7JOUR EQ 4)) [P8.JEUDIAP2 = P8.AP2];
IF ((P8.8SUMAP2 GE 100) AND (P8.8SUMAP2 LT 200) AND (P8.QACTIVITE8 EQ 108) AND (P8.AP8JOUR EQ 4)) [P8.JEUDIAP2 = P8.AP2];

IF ((P8.9SUMAP2 GE 100) AND (P8.9SUMAP2 LT 200) AND (P8.QACTIVITE9 EQ 109) AND (P8.AP9JOUR EQ 4)) [P8.JEUDIAP2 = P8.AP2];
IF ((P8.10SUMAP2 GE 100) AND (P8.10SUMAP2 LT 200) AND (P8.QACTIVITE_10 EQ 110) AND (P8.AP10JOUR EQ 4)) [P8.JEUDIAP2 = P8.AP2];
IF ((P8.11SUMAP2 GE 100) AND (P8.11SUMAP2 LT 200) AND (P8.QACTIVITE_11 EQ 111) AND (P8.AP11JOUR EQ 4)) [P8.JEUDIAP2 = P8.AP2];
IF ((P8.12SUMAP2 GE 100) AND (P8.12SUMAP2 LT 200) AND (P8.QACTIVITE_12 EQ 112) AND (P8.AP12JOUR EQ 4)) [P8.JEUDIAP2 = P8.AP2];
IF ((P8.2SUMAP2 GE 100) AND (P8.2SUMAP2 LT 200) AND (P8.QACTIVITE2 EQ 102) AND (P8.AP2JOUR EQ 5)) [P8.VENDREDIAP2 = P8.AP2];
IF ((P8.3SUMAP2 GE 100) AND (P8.3SUMAP2 LT 200) AND (P8.QACTIVITE3 EQ 103) AND (P8.AP3JOUR EQ 5)) [P8.VENDREDIAP2 = P8.AP2];
IF ((P8.4SUMAP2 GE 100) AND (P8.4SUMAP2 LT 200) AND (P8.QACTIVITE4 EQ 104) AND (P8.AP4JOUR EQ 5)) [P8.VENDREDIAP2 = P8.AP2];
IF ((P8.5SUMAP2 GE 100) AND (P8.5SUMAP2 LT 200) AND (P8.QACTIVITE5 EQ 105) AND (P8.AP5JOUR EQ 5)) [P8.VENDREDIAP2 = P8.AP2];
IF ((P8.6SUMAP2 GE 100) AND (P8.6SUMAP2 LT 200) AND (P8.QACTIVITE6 EQ 106) AND (P8.AP6JOUR EQ 5)) [P8.VENDREDIAP2 = P8.AP2];
IF ((P8.7SUMAP2 GE 100) AND (P8.7SUMAP2 LT 200) AND (P8.QACTIVITE7 EQ 107) AND (P8.AP7JOUR EQ 5)) [P8.VENDREDIAP2 = P8.AP2];
IF ((P8.8SUMAP2 GE 100) AND (P8.8SUMAP2 LT 200) AND (P8.QACTIVITE8 EQ 108) AND (P8.AP8JOUR EQ 5)) [P8.VENDREDIAP2 = P8.AP2];
IF ((P8.9SUMAP2 GE 100) AND (P8.9SUMAP2 LT 200) AND (P8.QACTIVITE9 EQ 109) AND (P8.AP9JOUR EQ 5)) [P8.VENDREDIAP2 = P8.AP2];
IF ((P8.10SUMAP2 GE 100) AND (P8.10SUMAP2 LT 200) AND (P8.QACTIVITE_10 EQ 110) AND (P8.AP10JOUR EQ 5)) [P8.VENDREDIAP2 = P8.AP2];
IF ((P8.11SUMAP2 GE 100) AND (P8.11SUMAP2 LT 200) AND (P8.QACTIVITE_11 EQ 111) AND (P8.AP11JOUR EQ 5)) [P8.VENDREDIAP2 = P8.AP2];
IF ((P8.12SUMAP2 GE 100) AND (P8.12SUMAP2 LT 200) AND (P8.QACTIVITE_12 EQ 112) AND (P8.AP12JOUR EQ 5)) [P8.VENDREDIAP2 = P8.AP2];
IF ((P8.2SUMAP2 GE 100) AND (P8.2SUMAP2 LT 200) AND (P8.QACTIVITE2 EQ 102) AND (P8.AP2JOUR EQ 6)) [P8.SAMEDIAP2 = P8.AP2];
IF ((P8.3SUMAP2 GE 100) AND (P8.3SUMAP2 LT 200) AND (P8.QACTIVITE3 EQ 103) AND (P8.AP3JOUR EQ 6)) [P8.SAMEDIAP2 = P8.AP2];
IF ((P8.4SUMAP2 GE 100) AND (P8.4SUMAP2 LT 200) AND (P8.QACTIVITE4 EQ 104) AND (P8.AP4JOUR EQ 6)) [P8.SAMEDIAP2 = P8.AP2];
IF ((P8.5SUMAP2 GE 100) AND (P8.5SUMAP2 LT 200) AND (P8.QACTIVITE5 EQ 105) AND (P8.AP5JOUR EQ 6)) [P8.SAMEDIAP2 = P8.AP2];
IF ((P8.6SUMAP2 GE 100) AND (P8.6SUMAP2 LT 200) AND (P8.QACTIVITE6 EQ 106) AND (P8.AP6JOUR EQ 6)) [P8.SAMEDIAP2 = P8.AP2];
IF ((P8.7SUMAP2 GE 100) AND (P8.7SUMAP2 LT 200) AND (P8.QACTIVITE7 EQ 107) AND (P8.AP7JOUR EQ 6)) [P8.SAMEDIAP2 = P8.AP2];

IF ((P8.8SUMAP2 GE 100) AND (P8.8SUMAP2 LT 200) AND (P8.QACTIVITE8 EQ 108) AND (P8.AP8JOUR EQ 6)) [P8.SAMEDIAP2 = P8.AP2];
IF ((P8.9SUMAP2 GE 100) AND (P8.9SUMAP2 LT 200) AND (P8.QACTIVITE9 EQ 109) AND (P8.AP9JOUR EQ 6)) [P8.SAMEDIAP2 = P8.AP2];
IF ((P8.10SUMAP2 GE 100) AND (P8.10SUMAP2 LT 200) AND (P8.QACTIVITE_10 EQ 110) AND (P8.AP10JOUR EQ 6)) [P8.SAMEDIAP2 = P8.AP2];
IF ((P8.11SUMAP2 GE 100) AND (P8.11SUMAP2 LT 200) AND (P8.QACTIVITE_11 EQ 111) AND (P8.AP11JOUR EQ 6)) [P8.SAMEDIAP2 = P8.AP2];
IF ((P8.12SUMAP2 GE 100) AND (P8.12SUMAP2 LT 200) AND (P8.QACTIVITE_12 EQ 112) AND (P8.AP12JOUR EQ 6)) [P8.SAMEDIAP2 = P8.AP2];
IF ((P8.2SUMAP2 GE 100) AND (P8.2SUMAP2 LT 200) AND (P8.QACTIVITE2 EQ 102) AND (P8.AP2JOUR EQ 7)) [P8.DIMANCHEAP2 = P8.AP2];
IF ((P8.3SUMAP2 GE 100) AND (P8.3SUMAP2 LT 200) AND (P8.QACTIVITE3 EQ 103) AND (P8.AP3JOUR EQ 7)) [P8.DIMANCHEAP2 = P8.AP2];
IF ((P8.4SUMAP2 GE 100) AND (P8.4SUMAP2 LT 200) AND (P8.QACTIVITE4 EQ 104) AND (P8.AP4JOUR EQ 7)) [P8.DIMANCHEAP2 = P8.AP2];
IF ((P8.5SUMAP2 GE 100) AND (P8.5SUMAP2 LT 200) AND (P8.QACTIVITE5 EQ 105) AND (P8.AP5JOUR EQ 7)) [P8.DIMANCHEAP2 = P8.AP2];
IF ((P8.6SUMAP2 GE 100) AND (P8.6SUMAP2 LT 200) AND (P8.QACTIVITE6 EQ 106) AND (P8.AP6JOUR EQ 7)) [P8.DIMANCHEAP2 = P8.AP2];
IF ((P8.7SUMAP2 GE 100) AND (P8.7SUMAP2 LT 200) AND (P8.QACTIVITE7 EQ 107) AND (P8.AP7JOUR EQ 7)) [P8.DIMANCHEAP2 = P8.AP2];
IF ((P8.8SUMAP2 GE 100) AND (P8.8SUMAP2 LT 200) AND (P8.QACTIVITE8 EQ 108) AND (P8.AP8JOUR EQ 7)) [P8.DIMANCHEAP2 = P8.AP2];
IF ((P8.9SUMAP2 GE 100) AND (P8.9SUMAP2 LT 200) AND (P8.QACTIVITE9 EQ 109) AND (P8.AP9JOUR EQ 7)) [P8.DIMANCHEAP2 = P8.AP2];
IF ((P8.10SUMAP2 GE 100) AND (P8.10SUMAP2 LT 200) AND (P8.QACTIVITE_10 EQ 110) AND (P8.AP10JOUR EQ 7)) [P8.DIMANCHEAP2 = P8.AP2];
IF ((P8.11SUMAP2 GE 100) AND (P8.11SUMAP2 LT 200) AND (P8.QACTIVITE_11 EQ 111) AND (P8.AP11JOUR EQ 7)) [P8.DIMANCHEAP2 = P8.AP2];
IF ((P8.12SUMAP2 GE 100) AND (P8.12SUMAP2 LT 200) AND (P8.QACTIVITE_12 EQ 112) AND (P8.AP12JOUR EQ 7)) [P8.DIMANCHEAP2 = P8.AP2];
ACTIVITÉ 3 DANS LE PLAN POUR CHAQUE JOUR DE LA SEMAINE
IF ((P8.3SUMAP3 GE 200) AND (P8.3SUMAP3 LT 300) AND (P8.QACTIVITE3 EQ 103) AND (P8.AP3JOUR EQ 1)) [P8.LUNDIAP3 = P8.AP3];
IF ((P8.4SUMAP3 GE 200) AND (P8.4SUMAP3 LT 300) AND (P8.QACTIVITE4 EQ 104) AND (P8.AP4JOUR EQ 1)) [P8.LUNDIAP3 = P8.AP3];
IF ((P8.5SUMAP3 GE 200) AND (P8.5SUMAP3 LT 300) AND (P8.QACTIVITE5 EQ 105) AND (P8.AP5JOUR EQ 1)) [P8.LUNDIAP3 = P8.AP3];
IF ((P8.6SUMAP3 GE 200) AND (P8.6SUMAP3 LT 300) AND (P8.QACTIVITE6 EQ 106) AND (P8.AP6JOUR EQ 1)) [P8.LUNDIAP3 = P8.AP3];

```
IF ((P8.7SUMAP3 GE 200) AND (P8.7SUMAP3 LT 300) AND (P8.QACTIVITE7 EQ 107) AND (P8.AP7JOUR EQ
1)) [P8.LUNDIAP3 = P8.AP3];
IF ((P8.8SUMAP3 GE 200) AND (P8.8SUMAP3 LT 300) AND (P8.QACTIVITE8 EQ 108) AND (P8.AP8JOUR EQ
1)) [P8.LUNDIAP3 = P8.AP3];
IF ((P8.9SUMAP3 GE 200) AND (P8.9SUMAP3 LT 300) AND (P8.QACTIVITE9 EQ 109) AND (P8.AP9JOUR EQ
1)) [P8.LUNDIAP3 = P8.AP3];
IF ((P8.10SUMAP3 GE 200) AND (P8.10SUMAP3 LT 300) AND (P8.QACTIVITE_10 EQ 110) AND (P8.AP10JOUR
EQ 1)) [P8.LUNDIAP3 = P8.AP3];
IF ((P8.11SUMAP3 GE 200) AND (P8.11SUMAP3 LT 300) AND (P8.QACTIVITE_11 EQ 111) AND (P8.AP11JOUR
EQ 1)) [P8.LUNDIAP3 = P8.AP3];
IF ((P8.12SUMAP3 GE 200) AND (P8.12SUMAP3 LT 300) AND (P8.QACTIVITE_12 EQ 112) AND (P8.AP12JOUR
EQ 1)) [P8.LUNDIAP3 = P8.AP3];
IF ((P8.3SUMAP3 GE 200) AND (P8.3SUMAP3 LT 300) AND (P8.QACTIVITE3 EQ 103) AND (P8.AP3JOUR EQ
2)) [P8.MARDIAP3 = P8.AP3];
IF ((P8.4SUMAP3 GE 200) AND (P8.4SUMAP3 LT 300) AND (P8.QACTIVITE4 EQ 104) AND (P8.AP4JOUR EQ
2)) [P8.MARDIAP3 = P8.AP3];
IF ((P8.5SUMAP3 GE 200) AND (P8.5SUMAP3 LT 300) AND (P8.QACTIVITE5 EQ 105) AND (P8.AP5JOUR EQ
2)) [P8.MARDIAP3 = P8.AP3];
IF ((P8.6SUMAP3 GE 200) AND (P8.6SUMAP3 LT 300) AND (P8.QACTIVITE6 EQ 106) AND (P8.AP6JOUR EQ
2)) [P8.MARDIAP3 = P8.AP3];
IF ((P8.7SUMAP3 GE 200) AND (P8.7SUMAP3 LT 300) AND (P8.QACTIVITE7 EQ 107) AND (P8.AP7JOUR EQ
2)) [P8.MARDIAP3 = P8.AP3];
IF ((P8.8SUMAP3 GE 200) AND (P8.8SUMAP3 LT 300) AND (P8.QACTIVITE8 EQ 108) AND (P8.AP8JOUR EQ
2)) [P8.MARDIAP3 = P8.AP3];
IF ((P8.9SUMAP3 GE 200) AND (P8.9SUMAP3 LT 300) AND (P8.QACTIVITE9 EQ 109) AND (P8.AP9JOUR EQ
2)) [P8.MARDIAP3 = P8.AP3];
IF ((P8.10SUMAP3 GE 200) AND (P8.10SUMAP3 LT 300) AND (P8.QACTIVITE_10 EQ 110) AND (P8.AP10JOUR
EQ 2)) [P8.MARDIAP3 = P8.AP3];
IF ((P8.11SUMAP3 GE 200) AND (P8.11SUMAP3 LT 300) AND (P8.QACTIVITE_11 EQ 111) AND (P8.AP11JOUR
EQ 2)) [P8.MARDIAP3 = P8.AP3];
IF ((P8.12SUMAP3 GE 200) AND (P8.12SUMAP3 LT 300) AND (P8.QACTIVITE_12 EQ 112) AND (P8.AP12JOUR
EQ 2)) [P8.MARDIAP3 = P8.AP3];
IF ((P8.3SUMAP3 GE 200) AND (P8.3SUMAP3 LT 300) AND (P8.QACTIVITE3 EQ 103) AND (P8.AP3JOUR EQ
3)) [P8.MERCREDIAP3 = P8.AP3];
IF ((P8.4SUMAP3 GE 200) AND (P8.4SUMAP3 LT 300) AND (P8.QACTIVITE4 EQ 104) AND (P8.AP4JOUR EQ
3)) [P8.MERCREDIAP3 = P8.AP3];
IF ((P8.5SUMAP3 GE 200) AND (P8.5SUMAP3 LT 300) AND (P8.QACTIVITE5 EQ 105) AND (P8.AP5JOUR EQ
3)) [P8.MERCREDIAP3 = P8.AP3];
IF ((P8.6SUMAP3 GE 200) AND (P8.6SUMAP3 LT 300) AND (P8.QACTIVITE6 EQ 106) AND (P8.AP6JOUR EQ
3)) [P8.MERCREDIAP3 = P8.AP3];
IF ((P8.7SUMAP3 GE 200) AND (P8.7SUMAP3 LT 300) AND (P8.QACTIVITE7 EQ 107) AND (P8.AP7JOUR EQ
3)) [P8.MERCREDIAP3 = P8.AP3];
```

IF ((P8.8SUMAP3 GE 200) AND (P8.8SUMAP3 LT 300) AND (P8.QACTIVITE8 EQ 108) AND (P8.AP8JOUR EQ 3)) [P8.MERCREDIAP3 = P8.AP3];
IF ((P8.9SUMAP3 GE 200) AND (P8.9SUMAP3 LT 300) AND (P8.QACTIVITE9 EQ 109) AND (P8.AP9JOUR EQ 3)) [P8.MERCREDIAP3 = P8.AP3];
IF ((P8.10SUMAP3 GE 200) AND (P8.10SUMAP3 LT 300) AND (P8.QACTIVITE_10 EQ 110) AND (P8.AP10JOUR EQ 3)) [P8.MERCREDIAP3 = P8.AP3];
IF ((P8.11SUMAP3 GE 200) AND (P8.11SUMAP3 LT 300) AND (P8.QACTIVITE_11 EQ 111) AND (P8.AP11JOUR EQ 3)) [P8.MERCREDIAP3 = P8.AP3];
IF ((P8.12SUMAP3 GE 200) AND (P8.12SUMAP3 LT 300) AND (P8.QACTIVITE_12 EQ 112) AND (P8.AP12JOUR EQ 3)) [P8.MERCREDIAP3 = P8.AP3];
IF ((P8.3SUMAP3 GE 200) AND (P8.3SUMAP3 LT 300) AND (P8.QACTIVITE3 EQ 103) AND (P8.AP3JOUR EQ 4)) [P8.JEUDIAP3 = P8.AP3];
IF ((P8.4SUMAP3 GE 200) AND (P8.4SUMAP3 LT 300) AND (P8.QACTIVITE4 EQ 104) AND (P8.AP4JOUR EQ 4)) [P8.JEUDIAP3 = P8.AP3];
IF ((P8.5SUMAP3 GE 200) AND (P8.5SUMAP3 LT 300) AND (P8.QACTIVITE5 EQ 105) AND (P8.AP5JOUR EQ 4)) [P8.JEUDIAP3 = P8.AP3];
IF ((P8.6SUMAP3 GE 200) AND (P8.6SUMAP3 LT 300) AND (P8.QACTIVITE6 EQ 106) AND (P8.AP6JOUR EQ 4)) [P8.JEUDIAP3 = P8.AP3];
IF ((P8.7SUMAP3 GE 200) AND (P8.7SUMAP3 LT 300) AND (P8.QACTIVITE7 EQ 107) AND (P8.AP7JOUR EQ 4)) [P8.JEUDIAP3 = P8.AP3];
IF ((P8.8SUMAP3 GE 200) AND (P8.8SUMAP3 LT 300) AND (P8.QACTIVITE8 EQ 108) AND (P8.AP8JOUR EQ 4)) [P8.JEUDIAP3 = P8.AP3];
IF ((P8.9SUMAP3 GE 200) AND (P8.9SUMAP3 LT 300) AND (P8.QACTIVITE9 EQ 109) AND (P8.AP9JOUR EQ 4)) [P8.JEUDIAP3 = P8.AP3];
IF ((P8.10SUMAP3 GE 200) AND (P8.10SUMAP3 LT 300) AND (P8.QACTIVITE_10 EQ 110) AND (P8.AP10JOUR EQ 4)) [P8.JEUDIAP3 = P8.AP3];
IF ((P8.11SUMAP3 GE 200) AND (P8.11SUMAP3 LT 300) AND (P8.QACTIVITE_11 EQ 111) AND (P8.AP11JOUR EQ 4)) [P8.JEUDIAP3 = P8.AP3];
IF ((P8.12SUMAP3 GE 200) AND (P8.12SUMAP3 LT 300) AND (P8.QACTIVITE_12 EQ 112) AND (P8.AP12JOUR EQ 4)) [P8.JEUDIAP3 = P8.AP3];
IF ((P8.3SUMAP3 GE 200) AND (P8.3SUMAP3 LT 300) AND (P8.QACTIVITE3 EQ 103) AND (P8.AP3JOUR EQ 5)) [P8.VENDREDIAP3 = P8.AP3];
IF ((P8.4SUMAP3 GE 200) AND (P8.4SUMAP3 LT 300) AND (P8.QACTIVITE4 EQ 104) AND (P8.AP4JOUR EQ 5)) [P8.VENDREDIAP3 = P8.AP3];
IF ((P8.5SUMAP3 GE 200) AND (P8.5SUMAP3 LT 300) AND (P8.QACTIVITE5 EQ 105) AND (P8.AP5JOUR EQ 5)) [P8.VENDREDIAP3 = P8.AP3];
IF ((P8.6SUMAP3 GE 200) AND (P8.6SUMAP3 LT 300) AND (P8.QACTIVITE6 EQ 106) AND (P8.AP6JOUR EQ 5)) [P8.VENDREDIAP3 = P8.AP3];
IF ((P8.7SUMAP3 GE 200) AND (P8.7SUMAP3 LT 300) AND (P8.QACTIVITE7 EQ 107) AND (P8.AP7JOUR EQ 5)) [P8.VENDREDIAP3 = P8.AP3];
IF ((P8.8SUMAP3 GE 200) AND (P8.8SUMAP3 LT 300) AND (P8.QACTIVITE8 EQ 108) AND (P8.AP8JOUR EQ 5)) [P8.VENDREDIAP3 = P8.AP3];

IF ((P8.9SUMAP3 GE 200) AND (P8.9SUMAP3 LT 300) AND (P8.QACTIVITE9 EQ 109) AND (P8.AP9JOUR EQ 5)) [P8.VENDREDIAP3 = P8.AP3];
IF ((P8.10SUMAP3 GE 200) AND (P8.10SUMAP3 LT 300) AND (P8.QACTIVITE_10 EQ 110) AND (P8.AP10JOUR EQ 5)) [P8.VENDREDIAP3 = P8.AP3];
IF ((P8.11SUMAP3 GE 200) AND (P8.11SUMAP3 LT 300) AND (P8.QACTIVITE_11 EQ 111) AND (P8.AP11JOUR EQ 5)) [P8.VENDREDIAP3 = P8.AP3];
IF ((P8.12SUMAP3 GE 200) AND (P8.12SUMAP3 LT 300) AND (P8.QACTIVITE_12 EQ 112) AND (P8.AP12JOUR EQ 5)) [P8.VENDREDIAP3 = P8.AP3];
IF ((P8.3SUMAP3 GE 200) AND (P8.3SUMAP3 LT 300) AND (P8.QACTIVITE3 EQ 103) AND (P8.AP3JOUR EQ 6)) [P8.SAMEDIAP3 = P8.AP3];
IF ((P8.4SUMAP3 GE 200) AND (P8.4SUMAP3 LT 300) AND (P8.QACTIVITE4 EQ 104) AND (P8.AP4JOUR EQ 6)) [P8.SAMEDIAP3 = P8.AP3];
IF ((P8.5SUMAP3 GE 200) AND (P8.5SUMAP3 LT 300) AND (P8.QACTIVITE5 EQ 105) AND (P8.AP5JOUR EQ 6)) [P8.SAMEDIAP3 = P8.AP3];
IF ((P8.6SUMAP3 GE 200) AND (P8.6SUMAP3 LT 300) AND (P8.QACTIVITE6 EQ 106) AND (P8.AP6JOUR EQ 6)) [P8.SAMEDIAP3 = P8.AP3];
IF ((P8.7SUMAP3 GE 200) AND (P8.7SUMAP3 LT 300) AND (P8.QACTIVITE7 EQ 107) AND (P8.AP7JOUR EQ 6)) [P8.SAMEDIAP3 = P8.AP3];
IF ((P8.8SUMAP3 GE 200) AND (P8.8SUMAP3 LT 300) AND (P8.QACTIVITE8 EQ 108) AND (P8.AP8JOUR EQ 6)) [P8.SAMEDIAP3 = P8.AP3];
IF ((P8.9SUMAP3 GE 200) AND (P8.9SUMAP3 LT 300) AND (P8.QACTIVITE9 EQ 109) AND (P8.AP9JOUR EQ 6)) [P8.SAMEDIAP3 = P8.AP3];
IF ((P8.10SUMAP3 GE 200) AND (P8.10SUMAP3 LT 300) AND (P8.QACTIVITE_10 EQ 110) AND (P8.AP10JOUR EQ 6)) [P8.SAMEDIAP3 = P8.AP3];
IF ((P8.11SUMAP3 GE 200) AND (P8.11SUMAP3 LT 300) AND (P8.QACTIVITE_11 EQ 111) AND (P8.AP11JOUR EQ 6)) [P8.SAMEDIAP3 = P8.AP3];
IF ((P8.12SUMAP3 GE 200) AND (P8.12SUMAP3 LT 300) AND (P8.QACTIVITE_12 EQ 112) AND (P8.AP12JOUR EQ 6)) [P8.SAMEDIAP3 = P8.AP3];
IF ((P8.3SUMAP3 GE 200) AND (P8.3SUMAP3 LT 300) AND (P8.QACTIVITE3 EQ 103) AND (P8.AP3JOUR EQ 7)) [P8.DIMANCHEAP3 = P8.AP3];
IF ((P8.4SUMAP3 GE 200) AND (P8.4SUMAP3 LT 300) AND (P8.QACTIVITE4 EQ 104) AND (P8.AP4JOUR EQ 7)) [P8.DIMANCHEAP3 = P8.AP3];
IF ((P8.5SUMAP3 GE 200) AND (P8.5SUMAP3 LT 300) AND (P8.QACTIVITE5 EQ 105) AND (P8.AP5JOUR EQ 7)) [P8.DIMANCHEAP3 = P8.AP3];
IF ((P8.6SUMAP3 GE 200) AND (P8.6SUMAP3 LT 300) AND (P8.QACTIVITE6 EQ 106) AND (P8.AP6JOUR EQ 7)) [P8.DIMANCHEAP3 = P8.AP3];
IF ((P8.7SUMAP3 GE 200) AND (P8.7SUMAP3 LT 300) AND (P8.QACTIVITE7 EQ 107) AND (P8.AP7JOUR EQ 7)) [P8.DIMANCHEAP3 = P8.AP3];
IF ((P8.8SUMAP3 GE 200) AND (P8.8SUMAP3 LT 300) AND (P8.QACTIVITE8 EQ 108) AND (P8.AP8JOUR EQ 7)) [P8.DIMANCHEAP3 = P8.AP3];
IF ((P8.9SUMAP3 GE 200) AND (P8.9SUMAP3 LT 300) AND (P8.QACTIVITE9 EQ 109) AND (P8.AP9JOUR EQ 7)) [P8.DIMANCHEAP3 = P8.AP3];

IF ((P8.10SUMAP3 GE 200) AND (P8.10SUMAP3 LT 300) AND (P8.QACTIVITE_10 EQ 110) AND (P8.AP10JOUR EQ 7)) [P8.DIMANCHEAP3 = P8.AP3];
IF ((P8.11SUMAP3 GE 200) AND (P8.11SUMAP3 LT 300) AND (P8.QACTIVITE_11 EQ 111) AND (P8.AP11JOUR EQ 7)) [P8.DIMANCHEAP3 = P8.AP3];
IF ((P8.12SUMAP3 GE 200) AND (P8.12SUMAP3 LT 300) AND (P8.QACTIVITE_12 EQ 112) AND (P8.AP12JOUR EQ 7)) [P8.DIMANCHEAP3 = P8.AP3];
POURSUITE DU CODE
IF (P8.P7BARCHOICE EQ 101) [P8.BAR = 'Être trop fatigué ou fatiguée pour faire de l´activité physique'];
IF (P8.P7BARCHOICE EQ 102) [P8.BAR = 'Avoir beaucoup de choses à faire mis à part l´activité physique'];
IF (P8.P7BARCHOICE EQ 103) [P8.BAR = 'température sera trop mauvaise pour faire de l´activité physique'];
IF (P8.P7BARCHOICE EQ 104) [P8.BAR = 'Ne pas avoir personne avec qui faire de l´activité physique'];
IF (P8.P7BARCHOICE EQ 105) [P8.BAR = 'Ne pas avoir accès à des emplacements pour faire de l´activité physique'];
IF (P8.P7BARCHOICE EQ 106) [P8.BAR = 'Ne pas avoir d´argent pour faire de l´activité physique'];
IF (P8.P7BARCHOICE EQ 107) [P8.BAR = 'Être trop gêné ou gênée, manquer de confiance pour aller faire de l´activité physique'];
IF (P8.P7BARCHOICE EQ 108) [P8.BAR = 'Avoir peur d´être insuffisamment en santé pour faire de l´activité physique'];
IF (P8.P7BARCHOICE EQ 109) [P8.BAR = 'Manquer de motivation'];
IF ((P8.P7BARCHOICE EQ 110) AND (P8.Q_BARANSWER NE 0)) [P8.BAR = P8.Q_BARANSWER];
IF (P8.SUMSOL EQ 101) [P8.SOL = 'Je vais faire de l´activité tôt dans la journée, en matinée ou à l´heure du midi.'];
IF (P8.SUMSOL EQ 102) [P8.SOL = 'J´essaie de garder en tête que si je fais de l´activité physique, j´aurai de plus en plus d´énergie dans la vie.'];
IF (P8.SUMSOL EQ 103) [P8.SOL = 'Je vais planifier des activités avec d´autres personnes qui me motiveront.'];
IF (P8.SUMSOL EQ 104) [P8.SOL = 'Je prends une collation pour me donner de l´énergie et j´y vais quand même.'];
IF (P8.SUMSOL EQ 201) [P8.SOL = 'Faire de l´activité physique par bloc de 10 minutes les journées où je n´ai pas de temps.'];
IF (P8.SUMSOL EQ 202) [P8.SOL = 'Je vais remplacer des activités où je suis assis ou assise, télé, ordinateur ou autres, par des activités physiques.'];
IF (P8.SUMSOL EQ 203) [P8.SOL = 'Je vais me procurer un agenda et inscrire dans mon horaire les moments où je vais faire de l´activité physique.'];
IF (P8.SUMSOL EQ 204) [P8.SOL = 'Je vais prendre des pauses de travail active et faire des 10 minutes de marche.'];
IF (P8.SUMSOL EQ 205) [P8.SOL = 'Je vais me fixer des moments avec d´autres personnes qui me plaisent pour aller faire de l´activité physique.'];
IF (P8.SUMSOL EQ 301) [P8.SOL = 'Je vais me procurer l´équipement nécessaire pour en faire même quand il pleut, quand il neige ou quand il fait trop chaud.'];
IF (P8.SUMSOL EQ 302) [P8.SOL = 'Je vais faire de l’activité physique chez nous ou à l´intérieur dans un endroit que j´aurai choisi quand il fait moins beau.'];

IF (P8.SUMSOL EQ 303) [P8.SOL = 'Je vais me préparer une activité physique alternative au cas où il serait désagréable d´aller dehors.'];
IF (P8.SUMSOL EQ 304) [P8.SOL = 'Je vais me dresser une liste des activités que je serais prêtE à faire si la température est mauvaise.'];
IF (P8.SUMSOL EQ 401) [P8.SOL = 'Je vais me joindre à un groupe qui pratique l´activité physique que j´aime. Ex : la marche, l´aquaforme ou la natation.'];
IF (P8.SUMSOL EQ 402) [P8.SOL = 'Je vais inviter mes amis, ma famille ou mes collègues à faire de l´activité physique avec moi.'];
IF (P8.SUMSOL EQ 403) [P8.SOL = 'Je vais amener mon chien marcher avec moi.'];
IF (P8.SUMSOL EQ 404) [P8.SOL = 'Je vais participer dans des discussions en ligne sur des pages facebook : Diabète Québec et autres.'];
IF (P8.SUMSOL EQ 501) [P8.SOL = 'Je vais faire de la marche ou du vélo dehors, c´est simple et gratuit.'];
IF (P8.SUMSOL EQ 502) [P8.SOL = 'Je vais faire de l´activité physique dans ma maison comme du yoga, un programme sur DVD, vélo stationnaire, tapis roulant, etc.'];
IF (P8.SUMSOL EQ 503) [P8.SOL = 'Je vais aller voir au centre communautaire de mon quartier pour me donner des options.'];
IF (P8.SUMSOL EQ 504) [P8.SOL = 'Je vais discuter avec mon médecin, avec un ami ou un spécialiste de l´activité physique pour avoir des conseils.'];
IF (P8.SUMSOL EQ 601) [P8.SOL = 'Je vais trouver des activités physiques abordables et simples : marcher dehors, faire du vélo, nager à la piscine communautaire.'];
IF (P8.SUMSOL EQ 602) [P8.SOL = 'Je vais aller voir au centre communautaire de mon quartier pour me donner des options.'];
IF (P8.SUMSOL EQ 603) [P8.SOL = 'Je vais économiser pour m´acheter un DVD d´activité physique ou pour une autre activité physique que j´aimerais faire.'];
IF (P8.SUMSOL EQ 604) [P8.SOL = 'Je vais aller marcher avec ma famille, des amis ou des collègues.'];
IF (P8.SUMSOL EQ 701) [P8.SOL = 'Je vais choisir une activité physique simple comme la marche ou la bicyclette.'];
IF (P8.SUMSOL EQ 702) [P8.SOL = 'Je vais aller faire de l´activité physique avec des gens qui m´acceptent comme je suis, qui évitent de me juger.'];
IF (P8.SUMSOL EQ 703) [P8.SOL = 'Au départ, je vais faire de l´activité physique dans des endroits où je suis seul[LETTRE_E] pour prendre confiance.'];
IF (P8.SUMSOL EQ 704) [P8.SOL = 'Je vais éviter des endroits qui me gêne davantage comme les centres de conditionnement.'];
IF (P8.SUMSOL EQ 801) [P8.SOL = 'Je vais consulter mon médecin pour mettre au clair si je peux faire de l´activité physique.'];
IF (P8.SUMSOL EQ 802) [P8.SOL = 'Je vais consulter un spécialiste de l´activité physique pour qu´il me rassure et me conseille sur ce qui est sécuritaire pour moi.'];
IF (P8.SUMSOL EQ 803) [P8.SOL = 'Je vais commencer à petite dose. 10 à 20 minutes maximum d´activités physiques les jours où je suis actif, active.'];
IF (P8.SUMSOL EQ 804) [P8.SOL = 'Je vais faire de l´activité physique accompagnéE d´un spécialiste de l´activité physique.'];

IF (P8.SUMSOL EQ 805) [P8.SOL = 'Je vais faire de l´activité physique dans un groupe où un spécialiste de l´activité physique est présent.'];
IF (P8.SUMSOL EQ 901) [P8.SOL = 'Je vais signer un contrat papier personnel comme quoi je m´engage à être actif, active.'];
IF (P8.SUMSOL EQ 902) [P8.SOL = 'Je vais signer un contrat papier avec mes proches comme quoi je m´engage à être actif, active.'];
IF (P8.SUMSOL EQ 903) [P8.SOL = 'Je vais essayer de nouvelles activités physiques pour trouver celles qui me plaisent vraiment.'];
IF (P8.SUMSOL EQ 904) [P8.SOL = 'À chaque activité physique que je fais, je prends un temps pour me rappeler ce que cela va m´apporter de mieux dans ma vie.'];
IF (P8.SUMSOL EQ 905) [P8.SOL = 'Je vais faire de l´activité physique avec des gens que j´aime pour me motiver.'];
IF ((P8.SUMSOL EQ 999) AND (P8.P8QSOLOPEN NE 0)) [P8.SOL = P8.P8QSOLOPEN];
IF (P8.SUMSOL EQ 0) [P8.SOL = P8.P8QSOL_10];
Séance 7
IF (F.3PRISK EQ 0) [RR.TRAIT = '• '];
IF (S1_RISKOPENTEXT NE 0) [R.OPTRAIT = '• '];
IF (((F.RISK GE 200) AND (F.RISK LT 300)) OR (F.3PRISK GE 200)) [S7.SUPRAELA2 = F1.SUPRAELA2];
IF (((BENSUM GE 200) AND (BENSUM LT 300)) OR (SUM.3PBEN GE 200)) [B.TRAIT = '• '];
IF (((BENSUM GE 200) AND (BENSUM LT 300)) OR (SUM.3PBEN GE 200)) [S7.SUPRABEN2 = S2.ELABTXT2];
IF (Q.BENOPEN NE 0) [B.OPTRAIT = '• '];
IF (S5.QVAL4 EQ 104) [S7.STR4 = 'capable, '];
IF ((S5.QVAL5 EQ 105) AND (SEXE EQ 1)) [S7.STR5 = 'compétent, '];
IF ((S5.QVAL5 EQ 105) AND (SEXE EQ 2)) [S7.STR5 = 'compétente, '];
IF ((S5.QVAL6 EQ 106) AND (SEXE EQ 1)) [S7.STR6 = 'confiant, '];
IF ((S5.QVAL6 EQ 106) AND (SEXE EQ 2)) [S7.STR6 = 'confiante, '];
IF ((S5.QVAL7 EQ 107) AND (SEXE EQ 1)) [S7.STR7 = 'décidé, '];
IF ((S5.QVAL7 EQ 107) AND (SEXE EQ 2)) [S7.STR7 = 'décidée, '];
IF ((S5.QVAL8 EQ 108) AND (SEXE EQ 1)) [S7.STR8 = 'déterminé, '];
IF ((S5.QVAL8 EQ 108) AND (SEXE EQ 2)) [S7.STR8 = 'déterminée, '];
IF ((S5.QVAL9 EQ 109) AND (SEXE EQ 1)) [S7.STR9 = 'dévoué, '];
IF ((S5.QVAL9 EQ 109) AND (SEXE EQ 2)) [S7.STR9 = 'dévouée, '];
IF (S5.QVAL_10 EQ 101.1) [S7.STR_10 = 'efficace, '];
IF ((S5.QVAL_13 EQ 101.4) AND (SEXE EQ 1)) [S7.STR_13 = 'gagnant, '];
IF ((S5.QVAL_13 EQ 101.4) AND (SEXE EQ 2)) [S7.STR_13 = 'gagnante, '];
IF (S5.QVAL_14 EQ 101.5) [S7.STR_14 = 'mature, '];
IF ((S5.QVAL_17 EQ 101.6) AND (SEXE EQ 1)) [S7.STR_17 = 'persévérant, '];
IF ((S5.QVAL_17 EQ 101.6) AND (SEXE EQ 2)) [S7.STR_17 = 'persévérante, '];
IF ((S5.QVAL_18 EQ 101.7) AND (SEXE EQ 1)) [S7.STR_18 = 'prévoyant, '];
IF ((S5.QVAL_18 EQ 101.7) AND (SEXE EQ 2)) [S7.STR_18 = 'prévoyante, '];
IF ((S5.QVAL_21 EQ 102.2) AND (SEXE EQ 1)) [S7.STR_21 = 'rempli de ressources, '];
IF ((S5.QVAL_21 EQ 102.2) AND (SEXE EQ 2)) [S7.STR_21 = 'remplie de ressources, '];

```
IF ((S5.QVAL_23 EQ 102.4) AND (SEXE EQ 1)) [S7.STR_23 = 'vaillant, '];
IF ((S5.QVAL_23 EQ 102.4) AND (SEXE EQ 2)) [S7.STR_23 = 'vaillante, '];
IF (S5.QVAL_24 EQ 102.5) [S7.STR_24 = 'visionnaire, '];
IF ((S5.QVAL_25 EQ 102.6) AND (SEXE EQ 1)) [S7.STR_25 = 'vrai, '];
IF ((S5.QVAL_25 EQ 102.6) AND (SEXE EQ 2)) [S7.STR_25 = 'vraie, '];
IF (S5.QVAL_OPEN NE 0) [STR.TRAIT = '• '];
*Additional equation for S4*
[S4.QATT_MI = S4.CQATT_MI-1];
*Additional equation for S8*
[S8.QEFF_MI = S8.CQEFF_MI-1];
*Additional equation for satisfaction*
[PAP.VRAI_NPS = PAP.NPS-1];
*variable pour accueil plan d'action*
IF (P1.ING EQ 1) [P1.TEXT_COMP = 'COMPLÉTÉ'];
IF (P2.ING EQ 1) [P2.TEXT_COMP = 'COMPLÉTÉ'];
IF (P3.ING EQ 1) [P3.TEXT_COMP = 'COMPLÉTÉ'];
IF (P4.ING EQ 1) [P4.TEXT_COMP = 'COMPLÉTÉ'];
IF (P5.ING EQ 1) [P5.TEXT_COMP = 'COMPLÉTÉ'];
IF (P6.ING EQ 1) [P6.TEXT_COMP = 'COMPLÉTÉ'];
IF (P7.ING EQ 1) [P7.TEXT_COMP = 'COMPLÉTÉ'];
IF (P8.ING EQ 1) [P8.TEXT_COMP = 'COMPLÉTÉ'];
*menu séances perso'
IF (S4.ING EQ 1) [SP_ACC_TEXT = 'COMPLÉTÉE'];
IF (S4.ING EQ 0) [SP_ACC_TEXT = 'TEMPS ÉCOULÉ'];
IF (S1.ING EQ 1) [SP_ACCS1TXT = 'COMPLÉTÉE'];
IF (S2.ING EQ 1) [SP_ACCS2TXT = 'COMPLÉTÉE'];
IF (S3.ING EQ 1) [SP_ACCS3TXT = 'COMPLÉTÉE'];
IF (S4.ING EQ 1) [SP_ACCS4TXT = 'COMPLÉTÉE'];
IF (S5.ING EQ 1) [SP_ACCS5TXT = 'COMPLÉTÉE'];
IF (S6.ING EQ 1) [SP_ACCS6TXT = 'COMPLÉTÉE'];
IF (S7.ING EQ 1) [SP_ACCS7TXT = 'COMPLÉTÉE'];
IF (S8.ING EQ 1) [SP_ACCS8TXT = 'COMPLÉTÉE'];
```

MAIN MENU – ADVICE FORMULAS

pre-program

IF ((SI.ING EQ 1) AND (CONSENT EQ 1) AND (TU.ING EQ 0) AND (((DATE_OFF EQ 18) AND (TBVAR_TIMENOW GE 600)) OR ((DATE_OFF GE 19) AND (DATE_OFF LE 24)) OR ((DATE_OFF EQ 25) AND (TBVAR_TIMENOW LT 600)))) [SA.AA_TUTORIEL];

IF (((DATE_OFF LE 17) OR ((TBVAR_TIMENOW LT 600) AND (DATE_OFF EQ 18))) AND (SICONSENT.ING EQ 0) AND (SIQAP.ING EQ 0) AND (((INCLUSION_AGE EQ 0) OR (INCLUSION_DIABETE EQ 0) OR (INCLUSION_LANGUE EQ 0) OR (INCLUSION_CONTRE EQ 0)) OR ((INCLUSION_AGE EQ 1) AND (INCLUSION_DIABETE EQ 1) AND (INCLUSION_LANGUE EQ 1) AND (INCLUSION_CONTRE EQ 2) AND (F.APTOT LT 150)))) [SA.A_LINK];

IF (((DATE_OFF LE 17) OR ((TBVAR_TIMENOW LT 600) AND (DATE_OFF EQ 18))) AND ((SICONSENT.ING EQ 1) OR (SIQAP.ING EQ 1)) AND (INCLUSION_AGE EQ 1) AND (INCLUSION_DIABETE EQ 1) AND (INCLUSION_LANGUE EQ 1) AND (INCLUSION_CONTRE EQ 2) AND (F.APTOT LT 150) AND (SI.ING EQ 0) AND ((CONSENT EQ 0) OR (CONSENT EQ 1))) [SA.A_poursuivre];

IF ((INCLUSION_AGE NE 0) AND (INCLUSION_DIABETE NE 0) AND (INCLUSION_LANGUE NE 0) AND (INCLUSION_CONTRE NE 0) AND (F.APTOT GE 150)) [SA.EX_TOOACTIVE];

IF (((DATE_OFF LE 17) OR ((TBVAR_TIMENOW LT 600) AND (DATE_OFF EQ 18))) AND (INCLUSION_AGE EQ 1) AND (INCLUSION_DIABETE EQ 1) AND (INCLUSION_LANGUE EQ 1) AND (INCLUSION_CONTRE EQ 2) AND (F.APTOT LT 150) AND (SI.ING EQ 1)) [SA.COMPLETED_LINK];

IF (CONSENT EQ 2) [SA.CONSENT_REFUSAL];

IF (((INCLUSION_AGE EQ 2) OR (INCLUSION_DIABETE EQ 2) OR (INCLUSION_LANGUE EQ 2) OR (INCLUSION_CONTRE EQ 1)) AND (F.APTOT LT 150)) [SA.EX_CRITERIA];

IF ((SI.ING EQ 0) AND (((DATE_OFF EQ 18) AND (TBVAR_TIMENOW GE 600)) OR (DATE_OFF GE 19))) [SA.INOTCOMPLETED];

semaine 1

IF ((CONSENT EQ 1) AND (SI.ING EQ 1) AND (TU.ING EQ 1) AND (S1.ING EQ 0) AND (((DATE_OFF EQ 18) AND (TBVAR_TIMENOW GE 600)) OR ((DATE_OFF GE 19) AND (DATE_OFF LE 24)) OR ((DATE_OFF EQ 25) AND (TBVAR_TIMENOW LT 600)))) [SA.LINK3];

IF ((CONSENT EQ 1) AND (SI.ING EQ 1) AND (TU.ING EQ 1) AND (S1.ING EQ 1) AND (((DATE_OFF EQ 18) AND (TBVAR_TIMENOW GE 600)) OR ((DATE_OFF GE 19) AND (DATE_OFF LE 24)) OR ((DATE_OFF EQ 25) AND (TBVAR_TIMENOW LT 600)))) [SA.LINK4];

semaine 2

IF ((CONSENT EQ 1) AND (SI.ING EQ 1) AND (S1.ING EQ 1) AND (S2.ING EQ 0) AND (((DATE_OFF EQ 25) AND (TBVAR_TIMENOW GE 600)) OR ((DATE_OFF GE 26) AND (DATE_OFF LE 31)) OR ((DATE_OFF EQ 32) AND (TBVAR_TIMENOW LT 600)))) [SA.LINK9];

IF ((CONSENT EQ 1) AND (SI.ING EQ 1) AND (S1.ING EQ 0) AND (S2.ING EQ 0) AND (((DATE_OFF EQ 25) AND (TBVAR_TIMENOW GE 600)) OR ((DATE_OFF GE 26) AND (DATE_OFF LE 31)) OR ((DATE_OFF EQ 32) AND (TBVAR_TIMENOW LT 600)))) [SA.LINK_13];

IF ((CONSENT EQ 1) AND (SI.ING EQ 1) AND (S1.ING EQ 1) AND (S2.ING EQ 1) AND (((DATE_OFF EQ 25) AND (TBVAR_TIMENOW GE 600)) OR ((DATE_OFF GE 26) AND (DATE_OFF LE 31)) OR ((DATE_OFF EQ 32) AND (TBVAR_TIMENOW LT 600)))) [SA.LINK_17];

semaine 3

IF ((CONSENT EQ 1) AND (SI.ING EQ 1) AND (S2.ING EQ 1) AND (S3.ING EQ 0) AND (((DATE_OFF EQ 32) AND (TBVAR_TIMENOW GE 600)) OR ((DATE_OFF GE 33) AND (DATE_OFF LE 38)) OR ((DATE_OFF EQ 39) AND (TBVAR_TIMENOW LT 600)))) [SA.LINK_21];

IF ((CONSENT EQ 1) AND (SI.ING EQ 1) AND (S2.ING EQ 0) AND (S3.ING EQ 0) AND (((DATE_OFF EQ 32) AND (TBVAR_TIMENOW GE 600)) OR ((DATE_OFF GE 33) AND (DATE_OFF LE 38)) OR ((DATE_OFF EQ 39) AND (TBVAR_TIMENOW LT 600)))) [SA.LINK_25];

IF ((CONSENT EQ 1) AND (SI.ING EQ 1) AND (S2.ING EQ 1) AND (S3.ING EQ 1) AND (((DATE_OFF EQ 32) AND (TBVAR_TIMENOW GE 600)) OR ((DATE_OFF GE 33) AND (DATE_OFF LE 38)) OR ((DATE_OFF EQ 39) AND (TBVAR_TIMENOW LT 600)))) [SA.LINK_29];

semaine 4

IF ((CONSENT EQ 1) AND (SI.ING EQ 1) AND (S4.ING EQ 0) AND (((DATE_OFF EQ 39) AND (TBVAR_TIMENOW GE 600)) OR ((DATE_OFF GE 40) AND (DATE_OFF LE 45)) OR ((DATE_OFF EQ 46) AND (TBVAR_TIMENOW LT 600)))) [SA.LINK_33];

IF ((CONSENT EQ 1) AND (SI.ING EQ 1) AND (S4.ING EQ 1) AND (((DATE_OFF EQ 39) AND (TBVAR_TIMENOW GE 600)) OR ((DATE_OFF GE 40) AND (DATE_OFF LE 45)) OR ((DATE_OFF EQ 46) AND (TBVAR_TIMENOW LT 600)))) [SA.LINK_37];

semaine 5

IF ((CONSENT EQ 1) AND (SI.ING EQ 1) AND (S3.ING EQ 1) AND (S5.ING EQ 0) AND (((DATE_OFF EQ 46) AND (TBVAR_TIMENOW GE 600)) OR ((DATE_OFF GE 47) AND (DATE_OFF LE 52)) OR ((DATE_OFF EQ 53) AND (TBVAR_TIMENOW LT 600)))) [SA.LINK_41];

IF ((CONSENT EQ 1) AND (SI.ING EQ 1) AND (S3.ING EQ 0) AND (S5.ING EQ 0) AND (((DATE_OFF EQ 46) AND (TBVAR_TIMENOW GE 600)) OR ((DATE_OFF GE 47) AND (DATE_OFF LE 52)) OR ((DATE_OFF EQ 53) AND (TBVAR_TIMENOW LT 600)))) [SA.LINK_45];

IF ((CONSENT EQ 1) AND (SI.ING EQ 1) AND (S3.ING EQ 1) AND (S5.ING EQ 1) AND (((DATE_OFF EQ 46) AND (TBVAR_TIMENOW GE 600)) OR ((DATE_OFF GE 47) AND (DATE_OFF LE 52)) OR ((DATE_OFF EQ 53) AND (TBVAR_TIMENOW LT 600)))) [SA.LINK_49];

semaine 6

IF ((CONSENT EQ 1) AND (SI.ING EQ 1) AND (S5.ING EQ 1) AND (S6.ING EQ 0) AND (((DATE_OFF EQ 53) AND (TBVAR_TIMENOW GE 600)) OR ((DATE_OFF GE 54) AND (DATE_OFF LE 59)) OR ((DATE_OFF EQ 60) AND (TBVAR_TIMENOW LT 600)))) [SA.LINK_53];

IF ((CONSENT EQ 1) AND (SI.ING EQ 1) AND (S5.ING EQ 0) AND (S6.ING EQ 0) AND (((DATE_OFF EQ 53) AND (TBVAR_TIMENOW GE 600)) OR ((DATE_OFF GE 54) AND (DATE_OFF LE 59)) OR ((DATE_OFF EQ 60) AND (TBVAR_TIMENOW LT 600)))) [SA.LINK_57];

IF ((CONSENT EQ 1) AND (SI.ING EQ 1) AND (S5.ING EQ 1) AND (S6.ING EQ 1) AND (((DATE_OFF EQ 53) AND (TBVAR_TIMENOW GE 600)) OR ((DATE_OFF GE 54) AND (DATE_OFF LE 59)) OR ((DATE_OFF EQ 60) AND (TBVAR_TIMENOW LT 600)))) [SA.LINK_61];

semaine 7

IF ((CONSENT EQ 1) AND (SI.ING EQ 1) AND (S6.ING EQ 1) AND (S7.ING EQ 0) AND (((DATE_OFF EQ 60) AND (TBVAR_TIMENOW GE 600)) OR ((DATE_OFF GE 61) AND (DATE_OFF LE 66)) OR ((DATE_OFF EQ 67) AND (TBVAR_TIMENOW LT 600)))) [SA.LINK_65];

IF ((CONSENT EQ 1) AND (SI.ING EQ 1) AND (S6.ING EQ 0) AND (S7.ING EQ 0) AND (((DATE_OFF EQ 60) AND (TBVAR_TIMENOW GE 600)) OR ((DATE_OFF GE 61) AND (DATE_OFF LE 66)) OR ((DATE_OFF EQ 67) AND (TBVAR_TIMENOW LT 600)))) [SA.LINK_69];

IF ((CONSENT EQ 1) AND (SI.ING EQ 1) AND (S6.ING EQ 1) AND (S7.ING EQ 1) AND (((DATE_OFF EQ 60) AND (TBVAR_TIMENOW GE 600)) OR ((DATE_OFF GE 61) AND (DATE_OFF LE 66)) OR ((DATE_OFF EQ 67) AND (TBVAR_TIMENOW LT 600)))) [SA.LINK_73];

semaine 8

IF ((CONSENT EQ 1) AND (SI.ING EQ 1) AND (S8.ING EQ 0) AND (((DATE_OFF EQ 67) AND (TBVAR_TIMENOW GE 600)) OR ((DATE_OFF GE 68) AND (DATE_OFF LE 73)) OR ((DATE_OFF EQ 74) AND (TBVAR_TIMENOW LE 600)))) [SA.LINK_77];

IF ((CONSENT EQ 1) AND (SI.ING EQ 1) AND (S8.ING EQ 1) AND (((DATE_OFF EQ 67) AND (TBVAR_TIMENOW GE 600)) OR ((DATE_OFF GE 68) AND (DATE_OFF LE 73)) OR ((DATE_OFF EQ 74) AND (TBVAR_TIMENOW LE 600)))) [SA.LINK_81];

post-program

IF ((CONSENT EQ 1) AND (SI.ING EQ 1) AND (TU.ING EQ 1) AND (((DATE_OFF EQ 74) AND (TBVAR_TIMENOW GE 600)) OR ((DATE_OFF GE 75) AND (DATE_OFF LE 88)) OR ((DATE_OFF EQ 89) AND (TBVAR_TIMENOW LE 600)))) [SA.PERIODELAT1];

IF ((CONSENT EQ 1) AND (SI.ING EQ 1) AND (((DATE_OFF EQ 102) AND (TBVAR_TIMENOW GE 600)) OR ((DATE_OFF GE 103) AND (DATE_OFF LE 235)) OR ((DATE_OFF EQ 236) AND (TBVAR_TIMENOW LE 600)))) [SA.PERIODELAT2];

IF ((CONSENT EQ 1) AND (SI.ING EQ 1) AND (TU.ING EQ 0) AND (((DATE_OFF EQ 74) AND (TBVAR_TIMENOW GE 600)) OR ((DATE_OFF GE 75) AND (DATE_OFF LE 88)) OR ((DATE_OFF EQ 89) AND (TBVAR_TIMENOW LE 600)))) [SA.PERIODELATSAT1];

IF ((CONSENT EQ 1) AND (SI.ING EQ 1) AND ((TU.ING EQ 1) OR (TU.ING EQ 0)) AND (PT1.ING EQ 1) AND (((DATE_OFF EQ 89) AND (TBVAR_TIMENOW GE 600)) OR ((DATE_OFF GE 90) AND (DATE_OFF LE 101)) OR ((DATE_OFF EQ 102) AND (TBVAR_TIMENOW LE 600)))) [SA.POSTQ1];

IF ((CONSENT EQ 1) AND (SI.ING EQ 1) AND (PT2.ING EQ 0) AND (((DATE_OFF EQ 236) AND (TBVAR_TIMENOW GE 600)) OR ((DATE_OFF GE 237) AND (DATE_OFF LE 259)) OR ((DATE_OFF EQ 260) AND (TBVAR_TIMENOW LE 600)))) [SA.POSTQ2];

IF ((CONSENT EQ 1) AND (SI.ING EQ 1) AND (TU.ING EQ 1) AND (PT1.ING EQ 1) AND (((DATE_OFF EQ 89) AND (TBVAR_TIMENOW GE 600)) OR ((DATE_OFF GE 90) AND (DATE_OFF LE 101)) OR ((DATE_OFF EQ 102) AND (TBVAR_TIMENOW LE 600)))) [SA.POSTTESTCOMP1];

IF ((CONSENT EQ 1) AND (SI.ING EQ 1) AND (PT2.ING EQ 1) AND (((DATE_OFF EQ 236) AND (TBVAR_TIMENOW GE 600)) OR ((DATE_OFF GE 237) AND (DATE_OFF LE 259)) OR ((DATE_OFF EQ 260) AND (TBVAR_TIMENOW LE 600)))) [SA.POSTTESTCOMP2];

IF ((CONSENT EQ 1) AND (SI.ING EQ 1) AND (TU.ING EQ 0) AND (PT1.ING EQ 1) AND (((DATE_OFF EQ 89) AND (TBVAR_TIMENOW GE 600)) OR ((DATE_OFF GE 90) AND (DATE_OFF LE 101)) OR ((DATE_OFF EQ 102) AND (TBVAR_TIMENOW LE 600)))) [SA.POSTTESTCOMPSAT1];

MENU OF THE MOTIVATIONAL SESSIONS

semaine 1

IF ((CONSENT EQ 1) AND (SI.ING EQ 1) AND ((DATE_OFF LE 24) OR ((DATE_OFF EQ 25) AND (TBVAR_TIMENOW LT 600)))) [SP_ACCW1_A];

semaine 2

IF ((CONSENT EQ 1) AND (SI.ING EQ 1) AND (S1.ING EQ 1) AND (((DATE_OFF EQ 25) AND (TBVAR_TIMENOW GE 600)) OR ((DATE_OFF GE 26) AND (DATE_OFF LE 31)) OR ((DATE_OFF EQ 32) AND (TBVAR_TIMENOW LT 600)))) [SP_ACCW2_AD];

IF ((CONSENT EQ 1) AND (SI.ING EQ 1) AND (S1.ING EQ 0) AND (((DATE_OFF EQ 25) AND (TBVAR_TIMENOW GE 600)) OR ((DATE_OFF GE 26) AND (DATE_OFF LE 31)) OR ((DATE_OFF EQ 32) AND (TBVAR_TIMENOW LT 600)))) [SP_ACCW2_FA];

semaine 3

IF ((CONSENT EQ 1) AND (SI.ING EQ 1) AND (S1.ING EQ 0) AND (S2.ING EQ 0) AND (((DATE_OFF EQ 32) AND (TBVAR_TIMENOW GE 600)) OR ((DATE_OFF GE 33) AND (DATE_OFF LE 38)) OR ((DATE_OFF EQ 39) AND (TBVAR_TIMENOW LT 600)))) [SP_ACCW3_ADD];

IF ((CONSENT EQ 1) AND (SI.ING EQ 1) AND (S1.ING EQ 1) AND (S2.ING EQ 0) AND (((DATE_OFF EQ 32) AND (TBVAR_TIMENOW GE 600)) OR ((DATE_OFF GE 33) AND (DATE_OFF LE 38)) OR ((DATE_OFF EQ 39) AND (TBVAR_TIMENOW LT 600)))) [SP_ACCW3_FAD];

IF ((CONSENT EQ 1) AND (SI.ING EQ 1) AND (S1.ING EQ 1) AND (S2.ING EQ 1) AND (((DATE_OFF EQ 32) AND (TBVAR_TIMENOW GE 600)) OR ((DATE_OFF GE 33) AND (DATE_OFF LE 38)) OR ((DATE_OFF EQ 39) AND (TBVAR_TIMENOW LT 600)))) [SP_ACCW3_FFA];

semaine 4

IF ((CONSENT EQ 1) AND (SI.ING EQ 1) AND (S1.ING EQ 0) AND (S2.ING EQ 0) AND (((DATE_OFF EQ 39) AND (TBVAR_TIMENOW GE 600)) OR ((DATE_OFF GE 40) AND (DATE_OFF LE 45)) OR ((DATE_OFF EQ 46) AND (TBVAR_TIMENOW LT 600)))) [SP_ACCW4_ADD];

IF ((CONSENT EQ 1) AND (SI.ING EQ 1) AND (S1.ING EQ 1) AND (S2.ING EQ 0) AND (((DATE_OFF EQ 39) AND (TBVAR_TIMENOW GE 600)) OR ((DATE_OFF GE 40) AND (DATE_OFF LE 45)) OR ((DATE_OFF EQ 46) AND (TBVAR_TIMENOW LT 600)))) [SP_ACCW4_FAD];

IF ((CONSENT EQ 1) AND (SI.ING EQ 1) AND (S1.ING EQ 1) AND (S2.ING EQ 1) AND (((DATE_OFF EQ 39) AND (TBVAR_TIMENOW GE 600)) OR ((DATE_OFF GE 40) AND (DATE_OFF LE 45)) OR ((DATE_OFF EQ 46) AND (TBVAR_TIMENOW LT 600)))) [SP_ACCW4_FFA];

semaine 5

IF ((CONSENT EQ 1) AND (SI.ING EQ 1) AND (S1.ING EQ 0) AND (S2.ING EQ 0) AND (S3.ING EQ 0) AND (((DATE_OFF EQ 46) AND (TBVAR_TIMENOW GE 600)) OR ((DATE_OFF GE 47) AND (DATE_OFF LE 52)) OR ((DATE_OFF EQ 53) AND (TBVAR_TIMENOW LT 600)))) [SP_ACCW5_ADDD];

IF ((CONSENT EQ 1) AND (SI.ING EQ 1) AND (S1.ING EQ 1) AND (S2.ING EQ 0) AND (S3.ING EQ 0) AND (((DATE_OFF EQ 46) AND (TBVAR_TIMENOW GE 600)) OR ((DATE_OFF GE 47) AND (DATE_OFF LE 52)) OR ((DATE_OFF EQ 53) AND (TBVAR_TIMENOW LT 600)))) [SP_ACCW5_FADD];

IF ((CONSENT EQ 1) AND (SI.ING EQ 1) AND (S1.ING EQ 1) AND (S2.ING EQ 1) AND (S3.ING EQ 0) AND (((DATE_OFF EQ 46) AND (TBVAR_TIMENOW GE 600)) OR ((DATE_OFF GE 47) AND (DATE_OFF LE 52)) OR ((DATE_OFF EQ 53) AND (TBVAR_TIMENOW LT 600)))) [SP_ACCW5_FFAD];

IF ((CONSENT EQ 1) AND (SI.ING EQ 1) AND (S1.ING EQ 1) AND (S2.ING EQ 1) AND (S3.ING EQ 1) AND (((DATE_OFF EQ 46) AND (TBVAR_TIMENOW GE 600)) OR ((DATE_OFF GE 47) AND (DATE_OFF LE 52)) OR ((DATE_OFF EQ 53) AND (TBVAR_TIMENOW LT 600)))) [SP_ACCW5_FFFA];

semaine 6

IF ((CONSENT EQ 1) AND (SI.ING EQ 1) AND (S1.ING EQ 0) AND (S2.ING EQ 0) AND (S3.ING EQ 0) AND (S5.ING EQ 0) AND (((DATE_OFF EQ 53) AND (TBVAR_TIMENOW GE 600)) OR ((DATE_OFF GE 54) AND (DATE_OFF LE 59)) OR ((DATE_OFF EQ 60) AND (TBVAR_TIMENOW LT 600)))) [SP_ACCW6_ADDDD];

IF ((CONSENT EQ 1) AND (SI.ING EQ 1) AND (S1.ING EQ 1) AND (S2.ING EQ 0) AND (S3.ING EQ 0) AND (S5.ING EQ 0) AND (((DATE_OFF EQ 53) AND (TBVAR_TIMENOW GE 600)) OR ((DATE_OFF GE 54) AND (DATE_OFF LE 59)) OR ((DATE_OFF EQ 60) AND (TBVAR_TIMENOW LT 600)))) [SP_ACCW6_FADDD];

IF ((CONSENT EQ 1) AND (SI.ING EQ 1) AND (S1.ING EQ 1) AND (S2.ING EQ 1) AND (S3.ING EQ 0) AND (S5.ING EQ 0) AND (((DATE_OFF EQ 53) AND (TBVAR_TIMENOW GE 600)) OR ((DATE_OFF GE 54) AND (DATE_OFF LE 59)) OR ((DATE_OFF EQ 60) AND (TBVAR_TIMENOW LT 600)))) [SP_ACCW6_FFADD];

IF ((CONSENT EQ 1) AND (SI.ING EQ 1) AND (S1.ING EQ 1) AND (S2.ING EQ 1) AND (S3.ING EQ 1) AND (S5.ING EQ 0) AND (((DATE_OFF EQ 53) AND (TBVAR_TIMENOW GE 600)) OR ((DATE_OFF GE 54) AND (DATE_OFF LE 59)) OR ((DATE_OFF EQ 60) AND (TBVAR_TIMENOW LT 600)))) [SP_ACCW6_FFFAD];

IF ((CONSENT EQ 1) AND (SI.ING EQ 1) AND (S1.ING EQ 1) AND (S2.ING EQ 1) AND (S3.ING EQ 1) AND (S5.ING EQ 1) AND (((DATE_OFF EQ 53) AND (TBVAR_TIMENOW GE 600)) OR ((DATE_OFF GE 54) AND (DATE_OFF LE 59)) OR ((DATE_OFF EQ 60) AND (TBVAR_TIMENOW LT 600)))) [SP_ACCW6_FFFFA];

semaine 7

IF ((CONSENT EQ 1) AND (SI.ING EQ 1) AND (S1.ING EQ 0) AND (S2.ING EQ 0) AND (S3.ING EQ 0) AND (S5.ING EQ 0) AND (S6.ING EQ 0) AND (((DATE_OFF EQ 60) AND (TBVAR_TIMENOW GE 600)) OR ((DATE_OFF GE 61) AND (DATE_OFF LE 66)) OR ((DATE_OFF EQ 67) AND (TBVAR_TIMENOW LT 600)))) [SP_ACCW7_ADDDDD];

IF ((CONSENT EQ 1) AND (SI.ING EQ 1) AND (S1.ING EQ 1) AND (S2.ING EQ 0) AND (S3.ING EQ 0) AND (S5.ING EQ 0) AND (S6.ING EQ 0) AND (((DATE_OFF EQ 60) AND (TBVAR_TIMENOW GE 600)) OR ((DATE_OFF GE 61) AND (DATE_OFF LE 66)) OR ((DATE_OFF EQ 67) AND (TBVAR_TIMENOW LT 600)))) [SP_ACCW7_FADDDD];

IF ((CONSENT EQ 1) AND (SI.ING EQ 1) AND (S1.ING EQ 1) AND (S2.ING EQ 1) AND (S3.ING EQ 0) AND (S5.ING EQ 0) AND (S6.ING EQ 0) AND (((DATE_OFF EQ 60) AND (TBVAR_TIMENOW GE 600)) OR ((DATE_OFF GE 61) AND (DATE_OFF LE 66)) OR ((DATE_OFF EQ 67) AND (TBVAR_TIMENOW LT 600)))) [SP_ACCW7_FFADDD];

IF ((CONSENT EQ 1) AND (SI.ING EQ 1) AND (S1.ING EQ 1) AND (S2.ING EQ 1) AND (S3.ING EQ 1) AND (S5.ING EQ 0) AND (S6.ING EQ 0) AND (((DATE_OFF EQ 60) AND (TBVAR_TIMENOW GE 600)) OR ((DATE_OFF GE 61) AND (DATE_OFF LE 66)) OR ((DATE_OFF EQ 67) AND (TBVAR_TIMENOW LT 600)))) [SP_ACCW7_FFFADD];

IF ((CONSENT EQ 1) AND (SI.ING EQ 1) AND (S1.ING EQ 1) AND (S2.ING EQ 1) AND (S3.ING EQ 1) AND (S5.ING EQ 1) AND (S6.ING EQ 0) AND (((DATE_OFF EQ 60) AND (TBVAR_TIMENOW GE 600)) OR ((DATE_OFF GE 61) AND (DATE_OFF LE 66)) OR ((DATE_OFF EQ 67) AND (TBVAR_TIMENOW LT 600)))) [SP_ACCW7_FFFFAD];

IF ((CONSENT EQ 1) AND (SI.ING EQ 1) AND (S1.ING EQ 1) AND (S2.ING EQ 1) AND (S3.ING EQ 1) AND (S5.ING EQ 1) AND (S6.ING EQ 1) AND (((DATE_OFF EQ 60) AND (TBVAR_TIMENOW GE 600)) OR ((DATE_OFF GE 61) AND (DATE_OFF LE 66)) OR ((DATE_OFF EQ 67) AND (TBVAR_TIMENOW LT 600)))) [SP_ACCW7_FFFFFA];

semaine 8

IF ((CONSENT EQ 1) AND (SI.ING EQ 1) AND (S1.ING EQ 0) AND (S2.ING EQ 0) AND (S3.ING EQ 0) AND (S5.ING EQ 0) AND (S6.ING EQ 0) AND (((DATE_OFF EQ 67) AND (TBVAR_TIMENOW GE 600)) OR ((DATE_OFF GE 68) AND (DATE_OFF LE 73)) OR ((DATE_OFF EQ 74) AND (TBVAR_TIMENOW LE 600)))) [SP_ACCW8_ADDDDD];

IF ((CONSENT EQ 1) AND (SI.ING EQ 1) AND (S1.ING EQ 1) AND (S2.ING EQ 0) AND (S3.ING EQ 0) AND (S5.ING EQ 0) AND (S6.ING EQ 0) AND (((DATE_OFF EQ 67) AND (TBVAR_TIMENOW GE 600)) OR ((DATE_OFF GE 68) AND (DATE_OFF LE 73)) OR ((DATE_OFF EQ 74) AND (TBVAR_TIMENOW LE 600)))) [SP_ACCW8_FADDDD];

IF ((CONSENT EQ 1) AND (SI.ING EQ 1) AND (S1.ING EQ 1) AND (S2.ING EQ 1) AND (S3.ING EQ 0) AND (S5.ING EQ 0) AND (S6.ING EQ 0) AND (((DATE_OFF EQ 67) AND (TBVAR_TIMENOW GE 600)) OR ((DATE_OFF GE 68) AND (DATE_OFF LE 73)) OR ((DATE_OFF EQ 74) AND (TBVAR_TIMENOW LE 600)))) [SP_ACCW8_FFADDD];

IF ((CONSENT EQ 1) AND (SI.ING EQ 1) AND (S1.ING EQ 1) AND (S2.ING EQ 1) AND (S3.ING EQ 1) AND (S5.ING EQ 0) AND (S6.ING EQ 0) AND (((DATE_OFF EQ 67) AND (TBVAR_TIMENOW GE 600)) OR ((DATE_OFF GE 68) AND (DATE_OFF LE 73)) OR ((DATE_OFF EQ 74) AND (TBVAR_TIMENOW LE 600)))) [SP_ACCW8_FFFADD];

IF ((CONSENT EQ 1) AND (SI.ING EQ 1) AND (S1.ING EQ 1) AND (S2.ING EQ 1) AND (S3.ING EQ 1) AND (S5.ING EQ 1) AND (S6.ING EQ 0) AND (((DATE_OFF EQ 67) AND (TBVAR_TIMENOW GE 600)) OR ((DATE_OFF GE 68) AND (DATE_OFF LE 73)) OR ((DATE_OFF EQ 74) AND (TBVAR_TIMENOW LE 600)))) [SP_ACCW8_FFFFAD];

IF ((CONSENT EQ 1) AND (SI.ING EQ 1) AND (S1.ING EQ 1) AND (S2.ING EQ 1) AND (S3.ING EQ 1) AND (S5.ING EQ 1) AND (S6.ING EQ 1) AND (((DATE_OFF EQ 67) AND (TBVAR_TIMENOW GE 600)) OR ((DATE_OFF GE 68) AND (DATE_OFF LE 73)) OR ((DATE_OFF EQ 74) AND (TBVAR_TIMENOW LE 600)))) [SP_ACCW8_FFFFFA];

MENU OF THE ACTION PLAN TOOL

IF ((CONSENT EQ 1) AND (SI.ING EQ 1) AND ((DATE_OFF LE 24) OR ((DATE_OFF EQ 25) AND (TBVAR_TIMENOW LT 600)))) [P.LINK3];

IF ((CONSENT EQ 1) AND (SI.ING EQ 1) AND (((DATE_OFF EQ 25) AND (TBVAR_TIMENOW GE 600)) OR ((DATE_OFF GE 26) AND (DATE_OFF LE 31)) OR ((DATE_OFF EQ 32) AND (TBVAR_TIMENOW LT 600)))) [P.LINK9];

IF ((CONSENT EQ 1) AND (SI.ING EQ 1) AND (((DATE_OFF EQ 32) AND (TBVAR_TIMENOW GE 600)) OR ((DATE_OFF GE 33) AND (DATE_OFF LE 38)) OR ((DATE_OFF EQ 39) AND (TBVAR_TIMENOW LT 600)))) [P.LINK_21];

IF ((CONSENT EQ 1) AND (SI.ING EQ 1) AND (((DATE_OFF EQ 39) AND (TBVAR_TIMENOW GE 600)) OR ((DATE_OFF GE 40) AND (DATE_OFF LE 45)) OR ((DATE_OFF EQ 46) AND (TBVAR_TIMENOW LT 600)))) [P.LINK_33];

IF ((CONSENT EQ 1) AND (SI.ING EQ 1) AND (((DATE_OFF EQ 46) AND (TBVAR_TIMENOW GE 600)) OR ((DATE_OFF GE 47) AND (DATE_OFF LE 52)) OR ((DATE_OFF EQ 53) AND (TBVAR_TIMENOW LT 600)))) [P.LINK_41];

IF ((CONSENT EQ 1) AND (SI.ING EQ 1) AND (((DATE_OFF EQ 53) AND (TBVAR_TIMENOW GE 600)) OR ((DATE_OFF GE 54) AND (DATE_OFF LE 59)) OR ((DATE_OFF EQ 60) AND (TBVAR_TIMENOW LT 600)))) [P.LINK_53];

IF ((CONSENT EQ 1) AND (SI.ING EQ 1) AND (((DATE_OFF EQ 60) AND (TBVAR_TIMENOW GE 600)) OR ((DATE_OFF GE 61) AND (DATE_OFF LE 66)) OR ((DATE_OFF EQ 67) AND (TBVAR_TIMENOW LT 600)))) [P.LINK_65];

IF ((CONSENT EQ 1) AND (SI.ING EQ 1) AND (((DATE_OFF EQ 67) AND (TBVAR_TIMENOW GE 600)) OR ((DATE_OFF GE 68) AND (DATE_OFF LE 73)) OR ((DATE_OFF EQ 74) AND (TBVAR_TIMENOW LE 600)))) [P.LINK_77];

REGISTRATION SESSION – END FORMULAS

```
IF (BESOINC EQ 0)
    {
    [BESOIN_C = 4];
    }
    ELSE
        {
        IF (BESOINC EQ 1)
            {
            [BESOIN_C = 3];
            }
            ELSE
                {
                IF (BESOINC EQ 2)
                {
                [BESOIN_C = 2];
                }
                ELSE
                    {
                    IF (BESOINC EQ 3)
                    {
                    [BESOIN_C = 1];
                    }
                    ELSE
                        {
```

```
IF (BESOINC
EQ 4)
{
[BESOIN_C =
0];
}
ELSE
{
IF (BESOINC EQ 888)
{
[BESOIN_C = 888];
}
}
}
}
}
}
IF (BESOINJ EQ 0)
{
[BESOIN_J = 4];
}
ELSE
{
```

```
IF (BESOINJ EQ 1)
    {
    [BESOIN_J = 3];
    }
    ELSE
        {
        IF (BESOINJ EQ 2)
        {
        [BESOIN_J = 2];
        }
        ELSE
            {
            IF (BESOINJ EQ 3)
            {
            [BESOIN_J = 1];
            }
            ELSE
                {
                IF (BESOINJ
EQ 4)
                {
                [BESOIN_J =
0];
                }
                ELSE
    {
```

```
IF (BESOINJ EQ 888)
{
[BESOIN_J = 888];
}
}
                                        }
                              }
                    }
          }
IF (BESOINO EQ 0)
     {
     [BESOIN_O = 4];
     }
     ELSE
          {
          IF (BESOINO EQ 1)
               {
               [BESOIN_O = 3];
               }
               ELSE
                         {
                         IF (BESOINO EQ 2)
```

```
{
[BESOIN_O = 2];
}
ELSE
{
IF (BESOINO EQ 3)
{
[BESOIN_O = 1];
}
ELSE
{
IF (BESOINO
EQ 4)
{
[BESOIN_O
= 0];
}
ELSE
{
IF (BESOINO EQ 888)
{
[BESOIN_O = 888];
}
```

```
}
}
}
}
}
[TESTDATE = 15-04-2014];
[AMOTITOT = MOTIVATIONF+MOTIVATIONK+MOTIVATIONN+MOTIVATIONV];
[PREF..AMOTIV = AMOTITOT/4];
[F.AMOTIV = PREF..AMOTIV - 1];
[MOTIEXTTOT = MOTIVATIONB+MOTIVATIONG+MOTIVATIONM+MOTIVATIONS];
[PREF..MOTEXT = MOTIEXTTOT/4];
[F.MOTEXT = PREF..MOTEXT - 1];
[MOTIINTROTOT = MOTIVATIONC+MOTIVATIONH+MOTIVATIONP];
[PREF..MOTINTRO = MOTIINTROTOT/3];
[F.MOTINTRO = PREF..MOTINTRO - 1];
[MOTIIDENTOT = MOTIVATIONI+MOTIVATIONT+MOTIVATIONA+MOTIVATIONQ];
[PREF..MOTIDEN = MOTIIDENTOT/4];
[F.MOTIDEN = PREF..MOTIDEN - 1];
[MOTIINTEGRTOT = MOTIVATIOND+MOTIVATIONJ+MOTIVATIONO+MOTIVATIONW];
[PREF..MOTINTEG = MOTIINTEGRTOT/4];
[F.MOTINTEG = PREF..MOTINTEG - 1];
[MOTIINTRINTOT = MOTIVATIONE+MOTIVATIONL+MOTIVATIONR+MOTIVATIONU];
[PREF..MOTINTRIN = MOTIINTRINTOT/4];
[TONOMIETOT = BESOINA+BESOIND+BESOING+BESOIN_J+BESOINM];
[F.AUTO = TONOMIETOT/5];
```

[COMPETTOT = BESOIN_C+BESOINF+BESOINI+BESOIN_O+BESOINL];

[F.COMP = COMPETTOT/5];

[PROXIMTOT = BESOINB+BESOINE+BESOINH+BESOINK+BESOINN];

[F.PROX = PROXIMTOT/5];

[TOTINTENT = INTENTION1+INTENTION2+INTENTION3];

[F.INTEN = TOTINTENT/3];

[TOTATT = ATTITUDE1+ATTITUDE2+ATTITUDE3+ATTITUDE4+ATTITUDE5+ATTITUDE6];

[F.ATT = TOTATT/6];

[TOTEFF = EFFICA1+EFFICA2+EFFICA3+EFFICA4];

[GRANDEURMETRE = GRANDEUR/100];

[GRANDEURCARRE = GRANDEURMETRE*GRANDEURMETRE];

[IMC = POIDS/GRANDEURCARRE];

[RECOM = 150];

[I.APVIG = APINTMIN*APINTX*2];

[I.AP2VIG = APINTMIN*APINTX];

[I.APMOD = APMODMIN*APMODX];

[F.APLEG = APLEGMIN*APLEGX];

[I.METVIG = APINTMIN*APINTX*8];

[I.METMOD = I.APMOD*4];

[I.METLEG = F.APLEG*3.3];

[I.METTOT = I.METVIG+I.METMOD+I.METLEG];

[F.APTOT = I.APVIG+I.APMOD];

[SI_PART1MOTTOT =
MOTIVATIONA+MOTIVATIONB+MOTIVATIONC+MOTIVATIOND+MOTIVATIONE+MOTIVATIONF+MOTIVATIONG+MOTIVATIONH+MOTIVATIONI+MOTIVATIONJ+MOTIVATIONK+MOTIVATIONL];

[SI_PART2MOTTOT = MOTIVATIONM+MOTIVATIONN+MOTIVATIONO+MOTIVATIONP+MOTIVATIONQ+MOTIVATIONR+MOTIVATIONS+MOTIVATIONT+MOTIVATIONU+MOTIVATIONV+MOTIVATIONW];

IF (SEXE EQ 2) [LETTREE = 'e'];

[SI_MI_ATTITUDE = SI_0MI_ATTITUDE - 1];

[SI_MI_EFFIC = SI_0MI_EFFIC - 1];

REGISTRATION SESSION– ADVICE AND ROUTING FORMULAS

```
*ROUTING 1*

IF ((SIQAP.ING EQ 0) AND (SICONSENT.ING EQ 0)) [SI.INTRO_CRITERE];

IF ((SIQAP.ING EQ 0) AND (SICONSENT.ING EQ 1)) [CONSENT];

IF ((SIQAP.ING EQ 1) AND (SICONSENT.ING EQ 1)) [QuestionnaireIntro];

*Routing critères*

IF (INCLUSION_AGE EQ 2)
        {
        [CONCLU];
        }
        ELSE
                {
                IF (INCLUSION_DIABETE EQ 2)
                        {
                        [CONCLU];
                        }
                        ELSE
                                {
                                IF (INCLUSION_LANGUE EQ 2)
                                {
                                [CONCLU];
                                }
                                ELSE
                                        {
                                        IF (INCLUSION_CONTRE EQ 1)
                                        {
```

```
[CONCLU];
}
ELSE
{
IF (F.APTOT
GE 150)
{
[CONCLU];
}
}
}
}}
```

Routings consentement et erreurs

IF (CONSENT EQ 2) [CONCLU];

IF (PRENOM NE 0) [SI.INTRO_TYPEMOT];

IF (PRENOM EQ 0) [Intro_2.Questionnaire];

IF ((MOTIVATIONA NE 0) AND (MOTIVATIONB NE 0) AND (MOTIVATIONC NE 0) AND (MOTIVATIOND NE 0) AND (MOTIVATIONE NE 0) AND (MOTIVATIONF NE 0) AND (MOTIVATIONG NE 0) AND (MOTIVATIONH NE 0) AND (MOTIVATIONI NE 0) AND (MOTIVATIONJ NE 0) AND (MOTIVATIONK NE 0) AND (MOTIVATIONL NE 0)) [SI.2INTROBREQ];

IF ((MOTIVATIONA EQ 0) OR (MOTIVATIONB EQ 0) OR (MOTIVATIONC EQ 0) OR (MOTIVATIOND EQ 0) OR (MOTIVATIONE EQ 0) OR (MOTIVATIONF EQ 0) OR (MOTIVATIONG EQ 0) OR (MOTIVATIONH EQ 0) OR (MOTIVATIONI EQ 0) OR (MOTIVATIONJ EQ 0) OR (MOTIVATIONK EQ 0) OR (MOTIVATIONL EQ 0)) [SI_BREQERROR];

IF ((MOTIVATIONM NE 0) AND (MOTIVATIONN NE 0) AND (MOTIVATIONO NE 0) AND (MOTIVATIONP NE 0) AND (MOTIVATIONQ NE 0) AND (MOTIVATIONR NE 0) AND (MOTIVATIONS NE 0) AND (MOTIVATIONT NE 0) AND (MOTIVATIONU NE 0) AND (MOTIVATIONV NE 0) AND (MOTIVATIONW NE 0)) [SI.INTRO_ICHANGE];

IF ((MOTIVATIONM EQ 0) OR (MOTIVATIONN EQ 0) OR (MOTIVATIONO EQ 0) OR (MOTIVATIONP EQ 0) OR (MOTIVATIONQ EQ 0) OR (MOTIVATIONR EQ 0) OR (MOTIVATIONS EQ 0) OR (MOTIVATIONT EQ 0) OR (MOTIVATIONU EQ 0) OR (MOTIVATIONV EQ 0) OR (MOTIVATIONW EQ 0)) [SI_2BREQERROR];

IF ((SI_0MI_ATTITUDE NE 0) AND (SI_0MI_EFFIC NE 0)) [SI.INTRO_ICHANGE2];

IF ((SI_0MI_ATTITUDE EQ 0) OR (SI_0MI_EFFIC EQ 0)) [SI.2INTRO_ICHANGE];

IF ((INTENTION1 NE 500) AND (INTENTION2 NE 500) AND (INTENTION3 NE 500) AND (EFFICA1 NE 500) AND (ATTITUDE1 NE 500) AND (ATTITUDE3 NE 500) AND (ATTITUDE4 NE 500) AND (ATTITUDES5 NE 500)) [SI.INTRO_ICHANGE3];

IF ((INTENTION1 EQ 500) OR (INTENTION2 EQ 500) OR (INTENTION3 EQ 500) OR (EFFICA1 EQ 500) OR (ATTITUDE1 EQ 500) OR (ATTITUDE3 EQ 500) OR (ATTITUDE4 EQ 500) OR (ATTITUDES5 EQ 500)) [SI.22INTRO_ICHANGE2];

IF ((EFFICA2 NE 500) AND (EFFICA3 NE 500) AND (SOCIALSUP1 NE 500) AND (SOCIALNORM1 NE 500) AND (SOCIALNORM3 NE 500)) [SI.INTROSOCIODEMO];

IF ((EFFICA2 EQ 500) OR (EFFICA3 EQ 500) OR (SOCIALSUP1 EQ 500) OR (SOCIALNORM1 EQ 500) OR (SOCIALNORM3 EQ 500)) [SI.222INTRO_ICHANGE3];

IF (((GRANDEUR_PIEDS NE 0) OR (GRANDEUR_CM NE 0)) AND ((POIDS_LIVRES NE 0) OR (POIDS_KG NE 0)) AND (AGE GE 18) AND (AGE LE 65) AND (SEXE NE 500) AND (STATCIVIL NE 500) AND (ORIGINES NE 500) AND (SCOLARITE NE 500) AND (OCCUPATION NE 500)) [s1.script1];

IF (((GRANDEUR_PIEDS EQ 0) AND (GRANDEUR_CM EQ 0)) OR ((POIDS_LIVRES EQ 0) AND (POIDS_KG EQ 0)) OR (AGE LT 18) OR (AGE GT 65) OR (SEXE EQ 500) OR (STATCIVIL EQ 500) OR (ORIGINES EQ 500) OR (SCOLARITE EQ 500) OR (OCCUPATION EQ 500)) [SI.22INTROSOCIODEMO];

ADVICE CONCLUSION

IF ((CONSENT EQ 1) AND (INCLUSION_AGE EQ 1) AND (INCLUSION_DIABETE EQ 1) AND (INCLUSION_LANGUE EQ 1) AND (INCLUSION_CONTRE EQ 2) AND (F.APTOT LT 150)) [SI_FINAL_PREPROG];

IF (CONSENT EQ 2) [SI_FB_NOCONSENT];

```
IF (F.APTOT GE 150)
        {
        [SI_FB_TOOACTIVE];
        }
        ELSE
                {
```

```
IF (INCLUSION_AGE EQ 2)
    {
    [SI_FB_EXCLUDED];
    }
    ELSE
        {
        IF (INCLUSION_DIABETE EQ 2)
        {
        [SI_FB_EXCLUDED];
        }
        ELSE
            {
            IF (INCLUSION_LANGUE EQ 2)
            {
            [SI_FB_EXCLUDED];
            }
            ELSE
                {
                IF
(INCLUSION_CONTRE EQ 1)
                {

    [SI_FB_EXCLUDED];

                }
            }
        }
```

}}

TAILORED MOTIVATIONAL SESSION 1 – ADVICE FORMULAS

feedback AP

[S1_TITREP3];

IF ((F.APTOT EQ 0) AND (SEXE EQ 1))[SEDRECH];

IF ((F.APTOT EQ 0) AND (SEXE EQ 2))[SEDRECF];

IF ((F.APTOT GE 10) AND (F.APTOT LE 50) AND (SEXE EQ 1))[LOWRECH];

IF ((F.APTOT GE 10) AND (F.APTOT LE 50) AND (SEXE EQ 2))[LOWRECF];

IF ((F.APTOT GT 50) AND (F.APTOT LT 100) AND (SEXE EQ 1))[MIDRECH];

IF ((F.APTOT GT 50) AND (F.APTOT LT 100) AND (SEXE EQ 2))[MIDRECF];

IF ((F.APTOT GE 100) AND (SEXE EQ 1))[HIGHRECH];

IF ((F.APTOT GE 100) AND (SEXE EQ 2))[HIGHRECF];

feedback consequences

IF ((F.RISK GE 300) AND (F.3PRISK GE 300)) [S1_REFERROR1];

IF ((F.RISK EQ 0) AND (SEXE EQ 1)) [REFRISK0H];

IF ((F.RISK EQ 0) AND (SEXE EQ 2)) [REFRISK0F];

IF (((F.RISK EQ 100) AND (SEXE EQ 1)) OR ((F.RISK EQ 101) AND (SEXE EQ 1)) OR ((F.RISK EQ 102) AND (SEXE EQ 1)) OR ((F.RISK EQ 103) AND (SEXE EQ 1)) OR ((F.RISK EQ 104) AND (SEXE EQ 1)) OR ((F.RISK EQ 105) AND (SEXE EQ 1)) OR ((F.RISK EQ 106) AND (SEXE EQ 1)) OR ((F.RISK EQ 107) AND (SEXE EQ 1))) [REFRISK1H];

IF (((F.RISK EQ 100) AND (SEXE EQ 2)) OR ((F.RISK EQ 101) AND (SEXE EQ 2)) OR ((F.RISK EQ 102) AND (SEXE EQ 2)) OR ((F.RISK EQ 103) AND (SEXE EQ 2)) OR ((F.RISK EQ 104) AND (SEXE EQ 2)) OR ((F.RISK EQ 105) AND (SEXE EQ 2)) OR ((F.RISK EQ 106) AND (SEXE EQ 2)) OR ((F.RISK EQ 107) AND (SEXE EQ 2))) [REFRISK1F];

IF ((F.RISK GE 201) AND (F.RISK LT 300) AND (SEXE EQ 1)) [REFRISK2H];

IF ((F.RISK GE 201) AND (F.RISK LT 300) AND (SEXE EQ 2)) [REFRISK2F];

IF ((F.RISK GT 300) AND (SEXE EQ 1)) [REFRISK3H];

IF ((F.RISK GT 300) AND (SEXE EQ 2)) [REFRISK3F];

FEEDBACK INFO

IF (((Q.INFO0 EQ 1) OR (Q.INFO0 EQ 1)) AND (F.RISK EQ 0)) [NINFO0];

IF (((Q.INFO1 EQ 1) OR (Q.INFO2 EQ 1)) AND (F.RISK GE 100)) [NINFOGEN];

IF (((Q.INFO1 EQ 1) OR (Q.INFO2 EQ 1)) AND ((F.RISK GE 100) AND (F.RISK LT 300) AND (RISQUES EQ 100)) OR (3PRISK1 EQ 100)) [NINFO1];

IF (((Q.INFO1 EQ 1) OR (Q.INFO2 EQ 1)) AND ((F.RISK GE 100) AND (F.RISK LT 300) AND (RISK2 EQ 101) AND (RISQUES EQ 0)) OR ((3PRISK1 EQ 0) AND (3PRISK2 EQ 101))) [NINFO2];

IF (((Q.INFO1 EQ 1) OR (Q.INFO2 EQ 1)) AND ((F.RISK GE 100) AND (F.RISK LT 300) AND (RISK3 EQ 102) AND (RISK2 EQ 0) AND (RISQUES EQ 0)) OR ((3PRISK1 EQ 0) AND (3PRISK2 EQ 0) AND (3PRISK3 EQ 102))) [NINFO3];

IF (((Q.INFO1 EQ 1) OR (Q.INFO2 EQ 1)) AND ((F.RISK GE 100) AND (F.RISK LT 300) AND (RISK4 EQ 103) AND (RISK3 EQ 0) AND (RISK2 EQ 0) AND (RISQUES EQ 0)) OR ((3PRISK1 EQ 0) AND (3PRISK2 EQ 0) AND (3PRISK3 EQ 0) AND (3PRISK4 EQ 103))) [NINFO4];

IF (((Q.INFO1 EQ 1) OR (Q.INFO2 EQ 1)) AND ((F.RISK GE 100) AND (F.RISK LT 300) AND (RISK5 EQ 104) AND (RISK4 EQ 0) AND (RISK3 EQ 0) AND (RISK2 EQ 0) AND (RISQUES EQ 0)) OR ((3PRISK1 EQ 0) AND (3PRISK2 EQ 0) AND (3PRISK3 EQ 0) AND (3PRISK4 EQ 0) AND (3PRISK5 EQ 104))) [NINFO5];

IF (((Q.INFO1 EQ 1) OR (Q.INFO2 EQ 1)) AND ((F.RISK GE 100) AND (F.RISK LT 300) AND (RISK6 EQ 105) AND (RISK5 EQ 0) AND (RISK4 EQ 0) AND (RISK3 EQ 0) AND (RISK2 EQ 0) AND (RISQUES EQ 0)) OR ((3PRISK1 EQ 0) AND (3PRISK2 EQ 0) AND (3PRISK3 EQ 0) AND (3PRISK4 EQ 0) AND (3PRISK5 EQ 0) AND (3PRISK6 EQ 105))) [NINFO6];

IF (((Q.INFO1 EQ 1) OR (Q.INFO2 EQ 1)) AND ((F.RISK GE 100) AND (F.RISK LT 300) AND (RISK7 EQ 106) AND (RISK6 EQ 0) AND (RISK5 EQ 0) AND (RISK4 EQ 0) AND (RISK3 EQ 0) AND (RISK2 EQ 0) AND (RISQUES EQ 0)) OR ((3PRISK1 EQ 0) AND (3PRISK2 EQ 0) AND (3PRISK3 EQ 0) AND (3PRISK4 EQ 0) AND (3PRISK5 EQ 0) AND (3PRISK6 EQ 0) AND (3PRISK7 EQ 106))) [NINFO7];

IF (((Q.INFO1 EQ 1) OR (Q.INFO2 EQ 1)) AND ((F.RISK GE 100) AND (F.RISK LT 300) AND (RISK8 EQ 107) AND (RISK7 EQ 0) AND (RISK6 EQ 0) AND (RISK5 EQ 0) AND (RISK4 EQ 0) AND (RISK3 EQ 0) AND (RISK2 EQ 0) AND (RISQUES EQ 0)) OR ((3PRISK1 EQ 0) AND (3PRISK2 EQ 0) AND (3PRISK3 EQ 0) AND (3PRISK4 EQ 0) AND (3PRISK5 EQ 0) AND (3PRISK6 EQ 0) AND (3PRISK7 EQ 0) AND (3PRISK8 EQ 107))) [NINFO8];

IF (((Q.INFO1 EQ 1) OR (Q.INFO2 EQ 1)) AND ((F.RISK GE 100) AND (F.RISK LT 300) AND (RISK2 EQ 101) AND (RISQUES EQ 100)) OR ((3PRISK1 EQ 100) AND (3PRISK2 EQ 101))) [2NINFO2];

IF (((Q.INFO1 EQ 1) OR (Q.INFO2 EQ 1)) AND ((F.RISK GE 100) AND (F.RISK LT 300) AND (RISK3 EQ 102) AND ((RISK2 EQ 101) OR (RISQUES EQ 100))) OR (((3PRISK1 EQ 100) OR (3PRISK2 EQ 101)) AND (3PRISK3 EQ 102))) [2NINFO3];

IF (((Q.INFO1 EQ 1) OR (Q.INFO2 EQ 1)) AND ((F.RISK GE 100) AND (F.RISK LT 300) AND (RISK4 EQ 103) AND ((RISK3 EQ 102) OR (RISK2 EQ 101) OR (RISQUES EQ 100))) OR (((3PRISK1 EQ 100) OR (3PRISK2 EQ 101) OR (3PRISK3 EQ 102)) AND (3PRISK4 EQ 103))) [2NINFO4];

IF (((Q.INFO1 EQ 1) OR (Q.INFO2 EQ 1)) AND ((F.RISK GE 100) AND (F.RISK LT 300) AND (RISK5 EQ 104) AND ((RISK4 EQ 103) OR (RISK3 EQ 102) OR (RISK2 EQ 101) OR (RISQUES EQ 100))) OR (((3PRISK1 EQ 100) OR (3PRISK2 EQ 101) OR (3PRISK3 EQ 102) OR (3PRISK4 EQ 103)) AND (3PRISK5 EQ 104))) [2NINFO5];

IF (((Q.INFO1 EQ 1) OR (Q.INFO2 EQ 1)) AND ((F.RISK GE 100) AND (F.RISK LT 300) AND (RISK6 EQ 105) AND ((RISK5 EQ 104) OR (RISK4 EQ 103) OR (RISK3 EQ 102) OR (RISK2 EQ 101) OR (RISQUES EQ 100))) OR (((3PRISK1 EQ 100) OR (3PRISK2 EQ 101) OR (3PRISK3 EQ 102) OR (3PRISK4 EQ 103) OR (3PRISK5 EQ 104)) AND (3PRISK6 EQ 105))) [2NINFO6];

IF (((Q.INFO1 EQ 1) OR (Q.INFO2 EQ 1)) AND ((F.RISK GE 100) AND (F.RISK LT 300) AND (RISK7 EQ 106) AND ((RISK6 EQ 105) OR (RISK5 EQ 104) OR (RISK4 EQ 103) OR (RISK3 EQ 102) OR (RISK2 EQ 101) OR (RISQUES EQ 100))) OR (((3PRISK1 EQ 100) OR (3PRISK2 EQ 101) OR (3PRISK3 EQ 102) OR (3PRISK4 EQ 103) OR (3PRISK5 EQ 104) OR (3PRISK6 EQ 105)) AND (3PRISK7 EQ 106))) [2NINFO7];

IF (((Q.INFO1 EQ 1) OR (Q.INFO2 EQ 1)) AND ((F.RISK GE 100) AND (F.RISK LT 300) AND (RISK8 EQ 107) AND ((RISK7 EQ 106) OR (RISK6 EQ 105) OR (RISK5 EQ 104) OR (RISK4 EQ 103) OR (RISK3 EQ 102) OR (RISK2 EQ 101) OR (RISQUES EQ 100))) OR (((3PRISK1 EQ 100) OR (3PRISK2 EQ 101) OR (3PRISK3 EQ 102) OR (3PRISK4 EQ 103) OR (3PRISK5 EQ 104) OR (3PRISK6 EQ 105) OR (3PRISK7 EQ 106)) AND (3PRISK8 EQ 107))) [2NINFO8];

IF ((Q.INFO1 EQ 1) OR (Q.INFO2 EQ 1)) [NINFOCLOSE];

IF ((Q.INFO1 EQ 1) OR (Q.INFO2 EQ 1)) [NINFOCON];

FEEDBACK RÉSUMÉ

IF ((F.RISK EQ 0) AND (S1_RISKOPENTEXT EQ 0) AND (Q.INFO0 EQ 2)) [F1.RES000];

IF ((F.RISK EQ 0) AND (S1_RISKOPENTEXT EQ 0) AND (Q.INFO0 EQ 1)) [F1.RES001];

IF ((F.RISK EQ 0) AND (S1_RISKOPENTEXT NE 0) AND (Q.INFO0 EQ 2)) [F1.RES010];

IF ((F.RISK EQ 0) AND (S1_RISKOPENTEXT NE 0) AND (Q.INFO0 EQ 1)) [F1.RES011];

IF ((F.RISK GE 100) AND (F.RISK LT 200) AND (Q.ELAB1 EQ 0) AND (S1_RISKOPENTEXT EQ 0) AND (Q.INFO1 EQ 2)) [F1.RES1000];

IF ((F.RISK GE 100) AND (F.RISK LT 200) AND (Q.ELAB1 EQ 0) AND (S1_RISKOPENTEXT EQ 0) AND (Q.INFO1 EQ 1)) [F1.RES1001];

IF ((F.RISK GE 100) AND (F.RISK LT 200) AND (Q.ELAB1 EQ 0) AND (S1_RISKOPENTEXT NE 0) AND (Q.INFO1 EQ 1)) [F1.RES1011];

IF ((F.RISK GE 100) AND (F.RISK LT 200) AND (Q.ELAB1 NE 0) AND (S1_RISKOPENTEXT NE 0) AND (Q.INFO1 EQ 1)) [F1.RES1111];

IF ((F.RISK GE 100) AND (F.RISK LT 200) AND (Q.ELAB1 EQ 0) AND (S1_RISKOPENTEXT NE 0) AND (Q.INFO1 EQ 2)) [F1.RES1010];

IF ((F.RISK GE 100) AND (F.RISK LT 200) AND (Q.ELAB1 NE 0) AND (S1_RISKOPENTEXT EQ 0) AND (Q.INFO1 EQ 2)) [F1.RES1100];

IF ((F.RISK GE 100) AND (F.RISK LT 200) AND (Q.ELAB1 NE 0) AND (S1_RISKOPENTEXT NE 0) AND (Q.INFO1 EQ 2)) [F1.RES1110];

IF ((F.RISK GE 100) AND (F.RISK LT 200) AND (Q.ELAB1 NE 0) AND (S1_RISKOPENTEXT EQ 0) AND (Q.INFO1 EQ 1)) [F1.RES1101];

IF ((F.RISK GE 300) AND (F.3PRISK LT 200) AND (F.3PRISK GE 100) AND (Q.ELAB3P1 EQ 0) AND (S1_RISKOPENTEXT EQ 0) AND (Q.INFO1 EQ 2)) [F1.REF1.3_000];

IF ((F.RISK GE 300) AND (F.3PRISK LT 200) AND (F.3PRISK GE 100) AND (Q.ELAB3P1 EQ 0) AND (S1_RISKOPENTEXT EQ 0) AND (Q.INFO1 EQ 1)) [F1.REF1.3_001];

IF ((F.RISK GE 300) AND (F.3PRISK LT 200) AND (F.3PRISK GE 100) AND (Q.ELAB3P1 EQ 0) AND (S1_RISKOPENTEXT NE 0) AND (Q.INFO1 EQ 1)) [F1.REF1.3_011];

IF ((F.RISK GE 300) AND (F.3PRISK LT 200) AND (F.3PRISK GE 100) AND (Q.ELAB3P1 NE 0) AND (S1_RISKOPENTEXT NE 0) AND (Q.INFO1 EQ 1)) [F1.REF1.3_111];

IF ((F.RISK GE 300) AND (F.3PRISK LT 200) AND (F.3PRISK GE 100) AND (Q.ELAB3P1 EQ 0) AND (S1_RISKOPENTEXT NE 0) AND (Q.INFO1 EQ 2)) [F1.REF1.3_010];

IF ((F.RISK GE 300) AND (F.3PRISK LT 200) AND (F.3PRISK GE 100) AND (Q.ELAB3P1 NE 0) AND (S1_RISKOPENTEXT EQ 0) AND (Q.INFO1 EQ 2)) [F1.REF1.3_100];

IF ((F.RISK GE 300) AND (F.3PRISK LT 200) AND (F.3PRISK GE 100) AND (Q.ELAB3P1 NE 0) AND (S1_RISKOPENTEXT NE 0) AND (Q.INFO1 EQ 2)) [F1.REF1.3_110];

IF ((F.RISK GE 300) AND (F.3PRISK LT 200) AND (F.3PRISK GE 100) AND (Q.ELAB3P1 NE 0) AND (S1_RISKOPENTEXT EQ 0) AND (Q.INFO1 EQ 1)) [F1.REF1.3_101];

IF ((F.RISK GE 200) AND (F.RISK LT 300) AND (Q.ELAB1 EQ 0) AND (Q.ELAB2 EQ 0) AND (S1_RISKOPENTEXT EQ 0) AND (Q.INFO2 EQ 2)) [F1.RES2.00.00];

IF ((F.RISK GE 200) AND (F.RISK LT 300) AND (Q.ELAB1 EQ 0) AND (Q.ELAB2 EQ 0) AND (S1_RISKOPENTEXT EQ 0) AND (Q.INFO2 EQ 1)) [F1.RES2.00.01];

IF ((F.RISK GE 200) AND (F.RISK LT 300) AND (Q.ELAB1 EQ 0) AND (Q.ELAB2 EQ 0) AND (S1_RISKOPENTEXT NE 0) AND (Q.INFO2 EQ 1)) [F1.RES2.00.11];

IF ((F.RISK GE 200) AND (F.RISK LT 300) AND (Q.ELAB1 NE 0) AND (Q.ELAB2 EQ 0) AND (S1_RISKOPENTEXT NE 0) AND (Q.INFO2 EQ 1)) [F1.RES2.10.11];

IF ((F.RISK GE 200) AND (F.RISK LT 300) AND (Q.ELAB1 EQ 0) AND (Q.ELAB2 EQ 0) AND (S1_RISKOPENTEXT NE 0) AND (Q.INFO2 EQ 2)) [F1.RES2.00.10];

IF ((F.RISK GE 200) AND (F.RISK LT 300) AND (Q.ELAB1 NE 0) AND (Q.ELAB2 EQ 0) AND (S1_RISKOPENTEXT EQ 0) AND (Q.INFO2 EQ 2)) [F1.RES2.10.00];

IF ((F.RISK GE 200) AND (F.RISK LT 300) AND (Q.ELAB1 NE 0) AND (Q.ELAB2 EQ 0) AND (S1_RISKOPENTEXT NE 0) AND (Q.INFO2 EQ 2)) [F1.RES2.10.10];

IF ((F.RISK GE 200) AND (F.RISK LT 300) AND (Q.ELAB1 NE 0) AND (Q.ELAB2 EQ 0) AND (S1_RISKOPENTEXT EQ 0) AND (Q.INFO2 EQ 1)) [F1.RES2.10.01];

IF ((F.RISK GE 200) AND (F.RISK LT 300) AND (Q.ELAB1 EQ 0) AND (Q.ELAB2 NE 0) AND (S1_RISKOPENTEXT EQ 0) AND (Q.INFO2 EQ 2)) [F1.RES2.01.00];

IF ((F.RISK GE 200) AND (F.RISK LT 300) AND (Q.ELAB1 EQ 0) AND (Q.ELAB2 NE 0) AND (S1_RISKOPENTEXT EQ 0) AND (Q.INFO2 EQ 1)) [F1.RES2.01.01];

IF ((F.RISK GE 200) AND (F.RISK LT 300) AND (Q.ELAB1 EQ 0) AND (Q.ELAB2 NE 0) AND (S1_RISKOPENTEXT NE 0) AND (Q.INFO2 EQ 1)) [F1.RES2.01.11];

IF ((F.RISK GE 200) AND (F.RISK LT 300) AND (Q.ELAB1 NE 0) AND (Q.ELAB2 NE 0) AND (S1_RISKOPENTEXT NE 0) AND (Q.INFO2 EQ 1)) [F1.RES2.11.11];

IF ((F.RISK GE 200) AND (F.RISK LT 300) AND (Q.ELAB1 EQ 0) AND (Q.ELAB2 NE 0) AND (S1_RISKOPENTEXT NE 0) AND (Q.INFO2 EQ 2)) [F1.RES2.01.10];

IF ((F.RISK GE 200) AND (F.RISK LT 300) AND (Q.ELAB1 NE 0) AND (Q.ELAB2 NE 0) AND (S1_RISKOPENTEXT EQ 0) AND (Q.INFO2 EQ 2)) [F1.RES2.11.00];

IF ((F.RISK GE 200) AND (F.RISK LT 300) AND (Q.ELAB1 NE 0) AND (Q.ELAB2 NE 0) AND (S1_RISKOPENTEXT NE 0) AND (Q.INFO2 EQ 2)) [F1.RES2.11.10];

IF ((F.RISK GE 200) AND (F.RISK LT 300) AND (Q.ELAB1 NE 0) AND (Q.ELAB2 NE 0) AND (S1_RISKOPENTEXT EQ 0) AND (Q.INFO2 EQ 1)) [F1.RES2.11.01];

IF ((F.RISK GE 300) AND (F.3PRISK LT 300) AND (F.3PRISK GE 200) AND (Q.ELAB3P1 EQ 0) AND (Q.ELAB3P2 EQ 0) AND (S1_RISKOPENTEXT EQ 0) AND (Q.INFO2 EQ 2)) [F1.RES2.3_.00.00];

IF ((F.RISK GE 300) AND (F.3PRISK LT 300) AND (F.3PRISK GE 200) AND (Q.ELAB3P1 EQ 0) AND (Q.ELAB3P2 EQ 0) AND (S1_RISKOPENTEXT EQ 0) AND (Q.INFO2 EQ 1)) [F1.RES2.3_.00.01];

IF ((F.RISK GE 300) AND (F.3PRISK LT 300) AND (F.3PRISK GE 200) AND (Q.ELAB3P1 EQ 0) AND (Q.ELAB3P2 EQ 0) AND (S1_RISKOPENTEXT NE 0) AND (Q.INFO2 EQ 1)) [F1.RES2.3_.00.11];

IF ((F.RISK GE 300) AND (F.3PRISK LT 300) AND (F.3PRISK GE 200) AND (Q.ELAB3P1 NE 0) AND (Q.ELAB3P2 EQ 0) AND (S1_RISKOPENTEXT NE 0) AND (Q.INFO2 EQ 1)) [F1.RES2.3_.10.11];

IF ((F.RISK GE 300) AND (F.3PRISK LT 300) AND (F.3PRISK GE 200) AND (Q.ELAB3P1 EQ 0) AND (Q.ELAB3P2 EQ 0) AND (S1_RISKOPENTEXT NE 0) AND (Q.INFO2 EQ 2)) [F1.RES2.3_.00.10];

IF ((F.RISK GE 300) AND (F.3PRISK LT 300) AND (F.3PRISK GE 200) AND (Q.ELAB3P1 NE 0) AND (Q.ELAB3P2 EQ 0) AND (S1_RISKOPENTEXT EQ 0) AND (Q.INFO2 EQ 2)) [F1.RES2.3_.10.00];

IF ((F.RISK GE 300) AND (F.3PRISK LT 300) AND (F.3PRISK GE 200) AND (Q.ELAB3P1 NE 0) AND (Q.ELAB3P2 EQ 0) AND (S1_RISKOPENTEXT NE 0) AND (Q.INFO2 EQ 2)) [F1.RES2.3_.10.10];

IF ((F.RISK GE 300) AND (F.3PRISK LT 300) AND (F.3PRISK GE 200) AND (Q.ELAB3P1 NE 0) AND (Q.ELAB3P2 EQ 0) AND (S1_RISKOPENTEXT EQ 0) AND (Q.INFO2 EQ 1)) [F1.RES2.3_.10.01];

IF ((F.RISK GE 300) AND (F.3PRISK LT 300) AND (F.3PRISK GE 200) AND (Q.ELAB3P1 EQ 0) AND (Q.ELAB3P2 NE 0) AND (S1_RISKOPENTEXT EQ 0) AND (Q.INFO2 EQ 2)) [F1.RES2.3_.01.00];

IF ((F.RISK GE 300) AND (F.3PRISK LT 300) AND (F.3PRISK GE 200) AND (Q.ELAB3P1 EQ 0) AND (Q.ELAB3P2 NE 0) AND (S1_RISKOPENTEXT EQ 0) AND (Q.INFO2 EQ 1)) [F1.RES2.3_.01.01];

IF ((F.RISK GE 300) AND (F.3PRISK LT 300) AND (F.3PRISK GE 200) AND (Q.ELAB3P1 EQ 0) AND (Q.ELAB3P2 NE 0) AND (S1_RISKOPENTEXT NE 0) AND (Q.INFO2 EQ 1)) [F1.RES2.3_.01.11];

IF ((F.RISK GE 300) AND (F.3PRISK LT 300) AND (F.3PRISK GE 200) AND (Q.ELAB3P1 NE 0) AND (Q.ELAB3P2 NE 0) AND (S1_RISKOPENTEXT NE 0) AND (Q.INFO2 EQ 1)) [F1.RES2.3_.11.11];

IF ((F.RISK GE 300) AND (F.3PRISK LT 300) AND (F.3PRISK GE 200) AND (Q.ELAB3P1 EQ 0) AND (Q.ELAB3P2 NE 0) AND (S1_RISKOPENTEXT NE 0) AND (Q.INFO2 EQ 2)) [F1.RES2.3_.01.10];

IF ((F.RISK GE 300) AND (F.3PRISK LT 300) AND (F.3PRISK GE 200) AND (Q.ELAB3P1 NE 0) AND (Q.ELAB3P2 NE 0) AND (S1_RISKOPENTEXT EQ 0) AND (Q.INFO2 EQ 2)) [F1.RES2.3_.11.00];

IF ((F.RISK GE 300) AND (F.3PRISK LT 300) AND (F.3PRISK GE 200) AND (Q.ELAB3P1 NE 0) AND (Q.ELAB3P2 NE 0) AND (S1_RISKOPENTEXT NE 0) AND (Q.INFO2 EQ 2)) [F1.RES2.3_.11.10];

IF ((F.RISK GE 300) AND (F.3PRISK LT 300) AND (F.3PRISK GE 200) AND (Q.ELAB3P1 NE 0) AND (Q.ELAB3P2 NE 0) AND (S1_RISKOPENTEXT EQ 0) AND (Q.INFO2 EQ 1)) [F1.RES2.3_.11.01];

feedback lastpage

IF ((S1_ACTIONPLAN EQ 1) AND (CONSENT EQ 1) AND (SI.ING EQ 1) AND ((DATE_OFF LE 24) OR ((DATE_OFF EQ 25) AND (TBVAR_TIMENOW LT 600)))) [S1_A.PLAN1];

IF ((S1_ACTIONPLAN EQ 1) AND (CONSENT EQ 1) AND (SI.ING EQ 1) AND (((DATE_OFF EQ 25) AND (TBVAR_TIMENOW GE 600)) OR ((DATE_OFF GE 26) AND (DATE_OFF LE 31)) OR ((DATE_OFF EQ 32) AND (TBVAR_TIMENOW LT 600)))) [S1_A.PLAN2];

IF ((S1_ACTIONPLAN EQ 1) AND (CONSENT EQ 1) AND (SI.ING EQ 1) AND (((DATE_OFF EQ 32) AND (TBVAR_TIMENOW GE 600)) OR ((DATE_OFF GE 33) AND (DATE_OFF LE 38)) OR ((DATE_OFF EQ 39) AND (TBVAR_TIMENOW LT 600)))) [S1_A.PLAN3];

IF ((S1_ACTIONPLAN EQ 1) AND (CONSENT EQ 1) AND (SI.ING EQ 1) AND (((DATE_OFF EQ 39) AND (TBVAR_TIMENOW GE 600)) OR ((DATE_OFF GE 40) AND (DATE_OFF LE 45)) OR ((DATE_OFF EQ 46) AND (TBVAR_TIMENOW LT 600)))) [S1_A.PLAN4];

IF ((S1_ACTIONPLAN EQ 1) AND (CONSENT EQ 1) AND (SI.ING EQ 1) AND (((DATE_OFF EQ 46) AND (TBVAR_TIMENOW GE 600)) OR ((DATE_OFF GE 47) AND (DATE_OFF LE 52)) OR ((DATE_OFF EQ 53) AND (TBVAR_TIMENOW LT 600)))) [S1_A.PLAN5];

IF ((S1_ACTIONPLAN EQ 1) AND (CONSENT EQ 1) AND (SI.ING EQ 1) AND (((DATE_OFF EQ 53) AND (TBVAR_TIMENOW GE 600)) OR ((DATE_OFF GE 54) AND (DATE_OFF LE 59)) OR ((DATE_OFF EQ 60) AND (TBVAR_TIMENOW LT 600)))) [S1_A.PLAN6];

IF ((S1_ACTIONPLAN EQ 1) AND (CONSENT EQ 1) AND (SI.ING EQ 1) AND (((DATE_OFF EQ 60) AND (TBVAR_TIMENOW GE 600)) OR ((DATE_OFF GE 61) AND (DATE_OFF LE 66)) OR ((DATE_OFF EQ 67) AND (TBVAR_TIMENOW LT 600)))) [S1_A.PLAN7];

IF ((S1_ACTIONPLAN EQ 1) AND (CONSENT EQ 1) AND (SI.ING EQ 1) AND (((DATE_OFF EQ 67) AND (TBVAR_TIMENOW GE 600)) OR ((DATE_OFF GE 68) AND (DATE_OFF LE 73)) OR ((DATE_OFF EQ 74) AND (TBVAR_TIMENOW LE 600)))) [S1_A.PLAN8];

IF (S1_ACTIONPLAN EQ 3) [S1_RETOURACCUEIL];

TAILORED MOTIVATIONAL SESSION 2– ADVICE FORMULAS

attitude

IF (SI_MI_ATTITUDE EQ 0) [S2.ATTI0];

IF ((SI_MI_ATTITUDE GE 1) AND (SI_MI_ATTITUDE LE 5)) [S2.ATTILOW];

IF ((SI_MI_ATTITUDE GE 6) AND (SI_MI_ATTITUDE LE 8)) [S2.ATTIMID];

IF ((SI_MI_ATTITUDE GE 9) AND (SI_MI_ATTITUDE LE 10)) [S2.ATTIHIGH];

reflet benefice 1

IF (SUM.3PBEN GE 300) [S2.P5ERROR];

IF ((BENSUM GE 101) AND (BENSUM LT 200)) [S2.P5REFBEN1];

IF ((BENSUM GE 200) AND (BENSUM LT 300)) [S2.P5REFBEN2];

IF (BENSUM GE 300) [S2.P5REFBEN3];

reflet benefices 2

IF ((BENSUM GE 100) AND (BENSUM LT 200)) [S2.P6INTROBENLEAB1];

IF ((BENSUM GE 200) AND (BENSUM LT 300)) [S2.P6INTROBENLEAB2];

IF ((BENSUM GE 300) AND (SUM.3PBEN GE 100) AND (SUM.3PBEN LT 200)) [S2.P6INTRO3PBENLEAB1];

IF ((BENSUM GE 300) AND (SUM.3PBEN GE 200) AND (SUM.3PBEN LT 300)) [S2.P6INTRO3PBENLEAB2];

reflet info

[S2.P8INTROINFO];

IF (BENSUM LT 100) [S2.P8INFOBEN0];

IF (((BENSUM GE 100) AND (BENSUM LT 300) AND (QBEN1 EQ 101)) OR ((BENSUM GE 300) AND (Q.3PBEN1 EQ 101))) [S2.P8INFOBEN1];

IF (((BENSUM GE 100) AND (BENSUM LT 300) AND (QBEN1 EQ 0) AND (QBEN2 EQ 102)) OR ((BENSUM GE 300) AND (Q.3PBEN1 EQ 0) AND (Q.3PBEN2 EQ 102))) [S2.P8INFOBEN2];

IF (((BENSUM GE 100) AND (BENSUM LT 300) AND (QBEN1 EQ 0) AND (QBEN2 EQ 0) AND (QBEN3 EQ 103)) OR ((BENSUM GE 300) AND (Q.3PBEN1 EQ 0) AND (Q.3PBEN2 EQ 0) AND (Q.3PBEN3 EQ 103))) [S2.P8INFOBEN3];

IF (((BENSUM GE 100) AND (BENSUM LT 300) AND (QBEN1 EQ 0) AND (QBEN2 EQ 0) AND (QBEN3 EQ 0) AND (QBEN4 EQ 104)) OR ((BENSUM GE 300) AND (Q.3PBEN1 EQ 0) AND (Q.3PBEN2 EQ 0) AND (Q.3PBEN3 EQ 0) AND (Q.3PBEN4 EQ 104))) [S2.P8INFOBEN4];

IF (((BENSUM GE 100) AND (BENSUM LT 300) AND (QBEN1 EQ 0) AND (QBEN2 EQ 0) AND (QBEN3 EQ 0) AND (QBEN4 EQ 0) AND (QBEN5 EQ 105)) OR ((BENSUM GE 300) AND (Q.3PBEN1 EQ 0) AND (Q.3PBEN2 EQ 0) AND (Q.3PBEN3 EQ 0) AND (Q.3PBEN4 EQ 0) AND (Q.3PBEN5 EQ 105))) [S2.P8INFOBEN5];

IF (((BENSUM GE 100) AND (BENSUM LT 300) AND (QBEN1 EQ 0) AND (QBEN2 EQ 0) AND (QBEN3 EQ 0) AND (QBEN4 EQ 0) AND (QBEN5 EQ 0) AND (QBEN6 EQ 106)) OR ((BENSUM GE 300) AND (Q.3PBEN1 EQ 0) AND (Q.3PBEN2 EQ 0) AND (Q.3PBEN3 EQ 0) AND (Q.3PBEN4 EQ 0) AND (Q.3PBEN5 EQ 0) AND (Q.3PBEN6 EQ 106))) [S2.P8INFOBEN6];

IF (((BENSUM GE 100) AND (BENSUM LT 300) AND (QBEN1 EQ 0) AND (QBEN2 EQ 0) AND (QBEN3 EQ 0) AND (QBEN4 EQ 0) AND (QBEN5 EQ 0) AND (QBEN6 EQ 0) AND (QBEN7 EQ 107)) OR ((BENSUM GE 300) AND (Q.3PBEN1 EQ 0) AND (Q.3PBEN2 EQ 0) AND (Q.3PBEN3 EQ 0) AND (Q.3PBEN4 EQ 0) AND (Q.3PBEN5 EQ 0) AND (Q.3PBEN6 EQ 0) AND (Q.3PBEN7 EQ 107))) [S2.P8INFOBEN7];

IF (((BENSUM GE 100) AND (BENSUM LT 300) AND (QBEN1 EQ 0) AND (QBEN2 EQ 0) AND (QBEN3 EQ 0) AND (QBEN4 EQ 0) AND (QBEN5 EQ 0) AND (QBEN6 EQ 0) AND (QBEN7 EQ 0) AND (QBEN8 EQ 108)) OR ((BENSUM GE 300) AND (Q.3PBEN1 EQ 0) AND (Q.3PBEN2 EQ 0) AND (Q.3PBEN3 EQ 0) AND (Q.3PBEN4 EQ

0) AND (Q.3PBEN5 EQ 0) AND (Q.3PBEN6 EQ 0) AND (Q.3PBEN7 EQ 0) AND (Q.3PBEN8 EQ 108))) [S2.P8INFOBEN8];

IF (((BENSUM GE 100) AND (BENSUM LT 300) AND (QBEN1 EQ 101) AND (QBEN2 EQ 102)) OR ((BENSUM GE 300) AND (Q.3PBEN1 EQ 101) AND (Q.3PBEN2 EQ 102))) [S2.P8INFOBEN2.2];

IF (((BENSUM GE 100) AND (BENSUM LT 300) AND ((QBEN1 EQ 101) OR (QBEN2 EQ 102)) AND (QBEN3 EQ 103)) OR ((BENSUM GE 300) AND ((Q.3PBEN1 EQ 101) OR (Q.3PBEN2 EQ 102)) AND (Q.3PBEN3 EQ 103))) [S2.P8INFOBEN3.2];

IF (((BENSUM GE 100) AND (BENSUM LT 300) AND ((QBEN1 EQ 101) OR (QBEN2 EQ 102) OR (QBEN3 EQ 103)) AND (QBEN4 EQ 104)) OR ((BENSUM GE 300) AND ((Q.3PBEN1 EQ 101) OR (Q.3PBEN2 EQ 102) OR (Q.3PBEN3 EQ 103)) AND (Q.3PBEN4 EQ 104))) [S2.P8INFOBEN4.2];

IF (((BENSUM GE 100) AND (BENSUM LT 300) AND ((QBEN1 EQ 101) OR (QBEN2 EQ 102) OR (QBEN3 EQ 103) OR (QBEN4 EQ 104)) AND (QBEN5 EQ 105)) OR ((BENSUM GE 300) AND ((Q.3PBEN1 EQ 101) OR (Q.3PBEN2 EQ 102) OR (Q.3PBEN3 EQ 103) OR (Q.3PBEN4 EQ 104)) AND (Q.3PBEN5 EQ 105))) [S2.P8INFOBEN5.2];

IF (((BENSUM GE 100) AND (BENSUM LT 300) AND ((QBEN1 EQ 101) OR (QBEN2 EQ 102) OR (QBEN3 EQ 103) OR (QBEN4 EQ 104) OR (QBEN5 EQ 105)) AND (QBEN6 EQ 106)) OR ((BENSUM GE 300) AND ((Q.3PBEN1 EQ 101) OR (Q.3PBEN2 EQ 102) OR (Q.3PBEN3 EQ 103) OR (Q.3PBEN4 EQ 104) OR (Q.3PBEN5 EQ 105)) AND (Q.3PBEN6 EQ 106))) [S2.P8INFOBEN6.2];

IF (((BENSUM GE 100) AND (BENSUM LT 300) AND ((QBEN1 EQ 101) OR (QBEN2 EQ 102) OR (QBEN3 EQ 103) OR (QBEN4 EQ 104) OR (QBEN5 EQ 105) OR (QBEN6 EQ 106)) AND (QBEN7 EQ 107)) OR ((BENSUM GE 300) AND ((Q.3PBEN1 EQ 101) OR (Q.3PBEN2 EQ 102) OR (Q.3PBEN3 EQ 103) OR (Q.3PBEN4 EQ 104) OR (Q.3PBEN5 EQ 105) OR (Q.3PBEN6 EQ 106)) AND (Q.3PBEN7 EQ 107))) [S2.P8INFOBEN7.2];

IF (((BENSUM GE 100) AND (BENSUM LT 300) AND ((QBEN1 EQ 101) OR (QBEN2 EQ 102) OR (QBEN3 EQ 103) OR (QBEN4 EQ 104) OR (QBEN5 EQ 105) OR (QBEN6 EQ 106) OR (QBEN7 EQ 107)) AND (QBEN8 EQ 108)) OR ((BENSUM GE 300) AND ((Q.3PBEN1 EQ 101) AND (Q.3PBEN2 EQ 102) AND (Q.3PBEN3 EQ 103) AND (Q.3PBEN4 EQ 104) AND (Q.3PBEN5 EQ 105) AND (Q.3PBEN6 EQ 106) AND (Q.3PBEN7 EQ 107)) AND (Q.3PBEN8 EQ 108))) [S2.P8INFOBEN8.2];

[S2.P8INFOGEN];

feedback résumé

IF ((((BENSUM GE 100) AND (BENSUM LT 200)) OR ((BENSUM GE 300) AND (SUM.3PBEN LT 200) AND (SUM.3PBEN GE 100))) AND (Q.BENOPEN NE 0)) [S2.AARESUME1OPEN];

IF ((((BENSUM GE 100) AND (BENSUM LT 200)) OR ((BENSUM GE 300) AND (SUM.3PBEN LT 200) AND (SUM.3PBEN GE 100))) AND (Q.BENOPEN EQ 0)) [S2.AARESUME1NOOP];

IF ((((BENSUM GE 200) AND (BENSUM LT 300)) OR ((BENSUM GE 300) AND (SUM.3PBEN LT 300) AND (SUM.3PBEN GE 200))) AND (Q.BENOPEN EQ 0)) [S2.AARESUME2NOOP];

IF ((((BENSUM GE 200) AND (BENSUM LT 300)) OR ((BENSUM GE 300) AND (SUM.3PBEN LT 300) AND (SUM.3PBEN GE 200))) AND (Q.BENOPEN NE 0)) [S2.AARESUME2OPEN];

feedback retour

IF ((S2.P10QFINALE EQ 1) AND (CONSENT EQ 1) AND (SI.ING EQ 1) AND (((DATE_OFF EQ 25) AND (TBVAR_TIMENOW GE 600)) OR ((DATE_OFF GE 26) AND (DATE_OFF LE 31)) OR ((DATE_OFF EQ 32) AND (TBVAR_TIMENOW LT 600)))) [S2.P11PLAN2];

IF ((S2.P10QFINALE EQ 1) AND (CONSENT EQ 1) AND (SI.ING EQ 1) AND (((DATE_OFF EQ 32) AND (TBVAR_TIMENOW GE 600)) OR ((DATE_OFF GE 33) AND (DATE_OFF LE 38)) OR ((DATE_OFF EQ 39) AND (TBVAR_TIMENOW LT 600)))) [S2.P11PLAN3];

IF ((S2.P10QFINALE EQ 1) AND (CONSENT EQ 1) AND (SI.ING EQ 1) AND (((DATE_OFF EQ 39) AND (TBVAR_TIMENOW GE 600)) OR ((DATE_OFF GE 40) AND (DATE_OFF LE 45)) OR ((DATE_OFF EQ 46) AND (TBVAR_TIMENOW LT 600)))) [S2.P11PLAN4];

IF ((S2.P10QFINALE EQ 1) AND (CONSENT EQ 1) AND (SI.ING EQ 1) AND (((DATE_OFF EQ 46) AND (TBVAR_TIMENOW GE 600)) OR ((DATE_OFF GE 47) AND (DATE_OFF LE 52)) OR ((DATE_OFF EQ 53) AND (TBVAR_TIMENOW LT 600)))) [S2.P11PLAN5];

IF ((S2.P10QFINALE EQ 1) AND (CONSENT EQ 1) AND (SI.ING EQ 1) AND (((DATE_OFF EQ 53) AND (TBVAR_TIMENOW GE 600)) OR ((DATE_OFF GE 54) AND (DATE_OFF LE 59)) OR ((DATE_OFF EQ 60) AND (TBVAR_TIMENOW LT 600)))) [S2.P11PLAN6];

IF ((S2.P10QFINALE EQ 1) AND (CONSENT EQ 1) AND (SI.ING EQ 1) AND (((DATE_OFF EQ 60) AND (TBVAR_TIMENOW GE 600)) OR ((DATE_OFF GE 61) AND (DATE_OFF LE 66)) OR ((DATE_OFF EQ 67) AND (TBVAR_TIMENOW LT 600)))) [S2.P11PLAN7];

IF ((S2.P10QFINALE EQ 1) AND (CONSENT EQ 1) AND (SI.ING EQ 1) AND (((DATE_OFF EQ 67) AND (TBVAR_TIMENOW GE 600)) OR ((DATE_OFF GE 68) AND (DATE_OFF LE 73)) OR ((DATE_OFF EQ 74) AND (TBVAR_TIMENOW LE 600)))) [S2.P11PLAN8];

IF (S2.P10QFINALE EQ 3) [S2.P11retour];

TAILORED MOTIVATIONAL SESSION 3– ADVICE FORMULAS

feedback type motivation

IF ((F.AMOTIV GE 2.5) AND (F.MOTEXT LT 2.5) AND (F.MOTINTRO LT 2.5) AND (F.MOTIDEN LT 2.5) AND (F.MOTINTEG LT 2.5)) [S3.P3TYPMOTAMOT];

IF ((F.MOTEXT GE 2.5) AND (F.MOTINTRO LT 2.5) AND (F.MOTIDEN LT 2.5) AND (F.MOTINTEG LT 2.5)) [S3.P3TYPMOTEXT];

IF ((F.MOTINTRO GE 2.5) AND (F.MOTIDEN LT 2.5) AND (F.MOTINTEG LT 2.5)) [S3.P3TYPMOTINTRO];

IF ((F.MOTIDEN GE 2.5) AND (F.MOTINTEG LT 2.5)) [S3.P3TYPMOTIDEN];

IF (F.MOTINTEG GE 2.5) [S3.P3TYPMOTINTEG];

IF ((F.AMOTIV LT 2.5) AND (F.MOTEXT LT 2.5) AND (F.MOTINTRO LT 2.5) AND (F.MOTIDEN LT 2.5) AND (F.MOTINTEG LT 2.5)) [S3.P3TYPMOTUNKNOWN];

feedback valeurs

IF (S3.2PSUMVAL GE 300) [S3.P6ERROR];

IF ((S3.SUMVAL GE 300) AND (S3.SUMVAL LT 1100) AND (SEXE EQ 1)) [S3.P6REFVAL310M];

IF ((S3.SUMVAL GE 300) AND (S3.SUMVAL LT 1100) AND (SEXE EQ 2)) [S3.P6REFVAL310F];

IF ((S3.SUMVAL GE 200) AND (S3.SUMVAL LT 300)) [S3.P6REFVAL2];

feedback résumé

[S3_P12AAASUPRA];

feedback retour

IF ((S3_P13_ACTION EQ 1) AND (CONSENT EQ 1) AND (SI.ING EQ 1) AND (((DATE_OFF EQ 32) AND (TBVAR_TIMENOW GE 600)) OR ((DATE_OFF GE 33) AND (DATE_OFF LE 38)) OR ((DATE_OFF EQ 39) AND (TBVAR_TIMENOW LT 600)))) [s3_a.plan3];

IF ((S3_P13_ACTION EQ 1) AND (CONSENT EQ 1) AND (SI.ING EQ 1) AND (((DATE_OFF EQ 39) AND (TBVAR_TIMENOW GE 600)) OR ((DATE_OFF GE 40) AND (DATE_OFF LE 45)) OR ((DATE_OFF EQ 46) AND (TBVAR_TIMENOW LT 600)))) [s3_a.plan4];

IF ((S3_P13_ACTION EQ 1) AND (CONSENT EQ 1) AND (SI.ING EQ 1) AND (((DATE_OFF EQ 46) AND (TBVAR_TIMENOW GE 600)) OR ((DATE_OFF GE 47) AND (DATE_OFF LE 52)) OR ((DATE_OFF EQ 53) AND (TBVAR_TIMENOW LT 600)))) [s3_a.plan5];

IF ((S3_P13_ACTION EQ 1) AND (CONSENT EQ 1) AND (SI.ING EQ 1) AND (((DATE_OFF EQ 53) AND (TBVAR_TIMENOW GE 600)) OR ((DATE_OFF GE 54) AND (DATE_OFF LE 59)) OR ((DATE_OFF EQ 60) AND (TBVAR_TIMENOW LT 600)))) [s3_a.plan6];

IF ((S3_P13_ACTION EQ 1) AND (CONSENT EQ 1) AND (SI.ING EQ 1) AND (((DATE_OFF EQ 60) AND (TBVAR_TIMENOW GE 600)) OR ((DATE_OFF GE 61) AND (DATE_OFF LE 66)) OR ((DATE_OFF EQ 67) AND (TBVAR_TIMENOW LT 600)))) [s3_a.plan7];

IF ((S3_P13_ACTION EQ 1) AND (CONSENT EQ 1) AND (SI.ING EQ 1) AND (((DATE_OFF EQ 67) AND (TBVAR_TIMENOW GE 600)) OR ((DATE_OFF GE 68) AND (DATE_OFF LE 73)) OR ((DATE_OFF EQ 74) AND (TBVAR_TIMENOW LE 600)))) [s3_a.plan8];

IF (S3_P13_ACTION EQ 3) [s3_retouracc];

TAILORED MOTIVATIONAL SESSION 4– ADVICE FORMULAS

feedback attitude

IF ((S4.QATT_MI GT SI_MI_ATTITUDE) AND (S4.QATT_MI GE 6)) [S4_P4SUPHIGH];

IF ((S4.QATT_MI GT SI_MI_ATTITUDE) AND (S4.QATT_MI LT 6)) [S4_P4SUPLOW];

IF ((S4.QATT_MI LT SI_MI_ATTITUDE) AND (S4.QATT_MI GE 6)) [S4_P4INFHIGH];

IF ((S4.QATT_MI LT SI_MI_ATTITUDE) AND (S4.QATT_MI LT 6) AND (S4.QATT_MI NE 0)) [S4_P4INFLOW];

IF ((S4.QATT_MI LT SI_MI_ATTITUDE) AND (S4.QATT_MI EQ 0)) [S4_P4INFZERO];

IF ((S4.QATT_MI EQ SI_MI_ATTITUDE) AND (S4.QATT_MI GE 6)) [S4_P4EQHIGH];

IF ((S4.QATT_MI EQ SI_MI_ATTITUDE) AND (S4.QATT_MI LT 6)) [S4_P4EQLOW];

feedback intention

feedback intention

IF ((S4.INTEN GT F.INTEN) AND (S4.INTEN GT 0)) [S4.INTSUPHIGH];

IF ((S4.INTEN GT F.INTEN) AND (S4.INTEN LE 0)) [S4.INTSUPLOW];

IF ((S4.INTEN LT F.INTEN) AND (S4.INTEN GT 0)) [S4.INTINFHIGH];

IF ((S4.INTEN LT F.INTEN) AND (S4.INTEN LE 0)) [S4.INTINFLOW];

IF ((S4.INTEN EQ F.INTEN) AND (S4.INTEN GT 0)) [S4.INTEQHIGH];

IF ((S4.INTEN EQ F.INTEN) AND (S4.INTEN LE 0)) [S4.INTEQLOW];

feedback ap

IF ((S4.APTOT GT F.APTOT) AND (S4.APTOT LT 150)) [S4_P8PASUP];

IF ((S4.APTOT GT F.APTOT) AND (S4.APTOT GE 150) AND (SEXE EQ 1)) [S4_P8AP150H];

IF ((S4.APTOT GT F.APTOT) AND (S4.APTOT GE 150) AND (SEXE EQ 2)) [S4_P8AP150F];

IF (S4.APTOT LT F.APTOT) [S4_P8APINF];

IF (S4.APTOT EQ F.APTOT) [S4_P8EGAL];

[S4_P8CONCLU];

retour

IF ((S4_P8QFINAL EQ 1) AND (CONSENT EQ 1) AND (SI.ING EQ 1) AND (((DATE_OFF EQ 39) AND (TBVAR_TIMENOW GE 600)) OR ((DATE_OFF GE 40) AND (DATE_OFF LE 45)) OR ((DATE_OFF EQ 46) AND (TBVAR_TIMENOW LT 600)))) [S4_P9plan4];

IF ((S4_P8QFINAL EQ 1) AND (CONSENT EQ 1) AND (SI.ING EQ 1) AND (((DATE_OFF EQ 46) AND (TBVAR_TIMENOW GE 600)) OR ((DATE_OFF GE 47) AND (DATE_OFF LE 52)) OR ((DATE_OFF EQ 53) AND (TBVAR_TIMENOW LT 600)))) [S4_P9plan5];

IF ((S4_P8QFINAL EQ 1) AND (CONSENT EQ 1) AND (SI.ING EQ 1) AND (((DATE_OFF EQ 53) AND (TBVAR_TIMENOW GE 600)) OR ((DATE_OFF GE 54) AND (DATE_OFF LE 59)) OR ((DATE_OFF EQ 60) AND (TBVAR_TIMENOW LT 600)))) [S4_P9plan6];

IF ((S4_P8QFINAL EQ 1) AND (CONSENT EQ 1) AND (SI.ING EQ 1) AND (((DATE_OFF EQ 60) AND (TBVAR_TIMENOW GE 600)) OR ((DATE_OFF GE 61) AND (DATE_OFF LE 66)) OR ((DATE_OFF EQ 67) AND (TBVAR_TIMENOW LT 600)))) [S4_P9plan7];

IF ((S4_P8QFINAL EQ 1) AND (CONSENT EQ 1) AND (SI.ING EQ 1) AND (((DATE_OFF EQ 67) AND (TBVAR_TIMENOW GE 600)) OR ((DATE_OFF GE 68) AND (DATE_OFF LE 73)) OR ((DATE_OFF EQ 74) AND (TBVAR_TIMENOW LE 600)))) [S4_P9plan8];

IF (S4_P8QFINAL EQ 2) [S4_P9ACCUEIL];

TAILORED MOTIVATIONAL SESSION 5– ADVICE FORMULAS

feedback confiance

IF (SI_MI_EFFIC LE 2) [S5.EFF_LOW];

IF ((SI_MI_EFFIC GT 2) AND (SI_MI_EFFIC LE 5)) [S5.EFF_MIDLOW];

IF ((SI_MI_EFFIC GT 5) AND (SI_MI_EFFIC LE 7)) [S5.EFF_MIDHIGH];

IF (SI_MI_EFFIC GE 8) [S5.EFF_HIGH];

feedback forces

IF (S5.2PSUMVAL GE 300) [S5_P6ERROR];

IF ((S5.SUMVAL GE 200) AND (S5.SUMVAL LT 300)) [S5_P6REFSTR2];

IF ((S5.SUMVAL GE 200) AND (S5.SUMVAL LT 300) AND (S5.QVAL_OPEN NE 0)) [S5_P6REFVALOP];

IF ((S5.SUMVAL GE 300) AND (S5.SUMVAL LT 900)) [S5_P6REFSTR3P];

feedback résumé

IF (S5.QVAL_OPEN EQ 0) [S5_P10AAAASUPRANOOP];

IF (S5.QVAL_OPEN NE 0) [S5_P10AAAASUPRAOPEN];

retour

IF ((S5_P11QACTION EQ 1) AND (CONSENT EQ 1) AND (SI.ING EQ 1) AND (((DATE_OFF EQ 46) AND (TBVAR_TIMENOW GE 600)) OR ((DATE_OFF GE 47) AND (DATE_OFF LE 52)) OR ((DATE_OFF EQ 53) AND (TBVAR_TIMENOW LT 600)))) [s5_a.plan5];

IF ((S5_P11QACTION EQ 1) AND (CONSENT EQ 1) AND (SI.ING EQ 1) AND (((DATE_OFF EQ 53) AND (TBVAR_TIMENOW GE 600)) OR ((DATE_OFF GE 54) AND (DATE_OFF LE 59)) OR ((DATE_OFF EQ 60) AND (TBVAR_TIMENOW LT 600)))) [s5_a.plan6];

IF ((S5_P11QACTION EQ 1) AND (CONSENT EQ 1) AND (SI.ING EQ 1) AND (((DATE_OFF EQ 60) AND (TBVAR_TIMENOW GE 600)) OR ((DATE_OFF GE 61) AND (DATE_OFF LE 66)) OR ((DATE_OFF EQ 67) AND (TBVAR_TIMENOW LT 600)))) [s5_a.plan7];

IF ((S5_P11QACTION EQ 1) AND (CONSENT EQ 1) AND (SI.ING EQ 1) AND (((DATE_OFF EQ 67) AND (TBVAR_TIMENOW GE 600)) OR ((DATE_OFF GE 68) AND (DATE_OFF LE 73)) OR ((DATE_OFF EQ 74) AND (TBVAR_TIMENOW LE 600)))) [s5_a.plan8];

IF (S5_P11QACTION EQ 3) [s5_retouracc];

TAILORED MOTIVATIONAL SESSION 6–ADVICE FORMULAS

raisons

IF (S6.QRAISONS EQ 0) [S6.P7REFNO];

IF (S6.QRAISONS NE 0) [S6.P7REFYES];

solutions

IF (((S6.P9QFEELF EQ 104) OR (S6.P9QFEELH EQ 104)) AND (S6.P9QFEELOPEN EQ 0)) [S6.ERRORP9];

IF ((SEXE EQ 2) AND ((S6.QBAR EQ 2) OR ((S6.QBAR EQ 1) AND (S6.P7BARCHOICE LE 100)) OR ((S6.QBAR EQ 1) AND (S6.P7BARCHOICE GE 100) AND (S6.P7BARCHOICE LE 200) AND (S6.Q_BARANSWER EQ 0) AND (S6.P7BARCHOICE EQ 110)))) [S6.NOBARP9F];

IF ((SEXE EQ 1) AND ((S6.QBAR EQ 2) OR ((S6.QBAR EQ 1) AND (S6.P7BARCHOICE LE 100)) OR ((S6.QBAR EQ 1) AND (S6.P7BARCHOICE GE 100) AND (S6.P7BARCHOICE LE 200) AND (S6.Q_BARANSWER EQ 0) AND (S6.P7BARCHOICE EQ 110)))) [S6.NOBARP9H];

IF (((S6.QBAR EQ 1) AND (S6.P7BARCHOICE GE 100) AND (S6.P7BARCHOICE LE 109)) OR ((S6.QBAR EQ 1) AND (S6.P7BARCHOICE EQ 110) AND (S6.Q_BARANSWER NE 0))) [S6.P9];

résumé

IF (((S6.QBAR EQ 1) AND (((S6.P7BARCHOICE GE 101) AND (S6.P7BARCHOICE LE 109)) OR ((S6.P7BARCHOICE EQ 110) AND (S6.Q_BARANSWER NE 0)))) AND (((S6.P9QFEELF GE 101) AND (S6.P9QFEELF LE 103)) OR ((S6.P9QFEELH GE 101) AND (S6.P9QFEELH LE 103)))) [S6.P10];

IF (((S6.QBAR EQ 1) AND (((S6.P7BARCHOICE GE 101) AND (S6.P7BARCHOICE LE 109)) OR ((S6.P7BARCHOICE EQ 110) AND (S6.Q_BARANSWER NE 0)))) AND (((S6.P9QFEELF EQ 104) AND (S6.P9QFEELOPEN NE 0)) OR ((S6.P9QFEELH EQ 104) AND (S6.P9QFEELOPEN NE 0)))) [S6.2P10];

IF (((S6.QBAR EQ 2) OR ((S6.QBAR EQ 1) AND ((S6.P7BARCHOICE EQ 0) OR (((S6.P7BARCHOICE EQ 110) AND (S6.Q_BARANSWER EQ 0)))))) AND (((S6.P9QFEELF GE 101) AND (S6.P9QFEELF LE 103)) OR ((S6.P9QFEELH GE 101) AND (S6.P9QFEELH LE 103)))) [S6.NOBAR10];

IF (((S6.QBAR EQ 2) OR ((S6.QBAR EQ 1) AND ((S6.P7BARCHOICE EQ 0) OR (((S6.P7BARCHOICE EQ 110) AND (S6.Q_BARANSWER EQ 0)))))) AND (((S6.P9QFEELF EQ 104) AND (S6.P9QFEELOPEN NE 0)) OR ((S6.P9QFEELH EQ 104) AND (S6.P9QFEELOPEN NE 0)))) [S6.2PNOBAR10];

retour

IF ((S6.P11QACTION EQ 1) AND (CONSENT EQ 1) AND (SI.ING EQ 1) AND (((DATE_OFF EQ 53) AND (TBVAR_TIMENOW GE 600)) OR ((DATE_OFF GE 54) AND (DATE_OFF LE 59)) OR ((DATE_OFF EQ 60) AND (TBVAR_TIMENOW LT 600)))) [S6.a.plan6];

IF ((S6.P11QACTION EQ 1) AND (CONSENT EQ 1) AND (SI.ING EQ 1) AND (((DATE_OFF EQ 60) AND (TBVAR_TIMENOW GE 600)) OR ((DATE_OFF GE 61) AND (DATE_OFF LE 66)) OR ((DATE_OFF EQ 67) AND (TBVAR_TIMENOW LT 600)))) [S6.a.plan7];

IF ((S6.P11QACTION EQ 1) AND (CONSENT EQ 1) AND (SI.ING EQ 1) AND (((DATE_OFF EQ 67) AND (TBVAR_TIMENOW GE 600)) OR ((DATE_OFF GE 68) AND (DATE_OFF LE 73)) OR ((DATE_OFF EQ 74) AND (TBVAR_TIMENOW LE 600)))) [S6.a.plan8];

IF (S6.P11QACTION EQ 2) [S6.RETOURACC];

TAILORED MOTIVATIONAL SESSION 7– ADVICE FORMULAS

retour ap

IF (S4.ING EQ 0) [S7_P21NO];

IF (S4.ING EQ 1) [S7_P21YES];

IF (S4.ING EQ 0) [S7_P2NOTABLE];

IF (S4.ING EQ 1) [S7_P2TABLE];

[S7_P22YES];

retour importante changement

IF ((F.RISK GE 100) AND (F.RISK LT 300) AND (S3.P9QELAB1 NE 0) AND (S3.P9QELAB2 NE 0)) [S7_P32];

IF ((F.RISK GE 100) AND (F.RISK LT 300) AND (S3.P9QELAB1 NE 0) AND (S3.P9QELAB2 EQ 0)) [S7_P311];

IF ((F.RISK GE 100) AND (F.RISK LT 300) AND (S3.P9QELAB1 EQ 0) AND (S3.P9QELAB2 NE 0)) [S7_P312];

IF ((F.RISK GE 100) AND (F.RISK LT 300) AND (S3.P9QELAB1 EQ 0) AND (S3.P9QELAB2 EQ 0)) [S7_P30];

IF ((F.RISK GE 300) AND (F.3PRISK GE 100) AND (S3.P9QELAB1 NE 0) AND (S3.P9QELAB2 NE 0)) [S7_3PP32];

IF ((F.RISK GE 300) AND (F.3PRISK GE 100) AND (S3.P9QELAB1 NE 0) AND (S3.P9QELAB2 EQ 0)) [S7_3PP311];

IF ((F.RISK GE 300) AND (F.3PRISK GE 100) AND (S3.P9QELAB1 EQ 0) AND (S3.P9QELAB2 NE 0)) [S7_3PP312];

IF ((F.RISK GE 300) AND (F.3PRISK GE 100) AND (S3.P9QELAB1 EQ 0) AND (S3.P9QELAB2 EQ 0)) [S7_3PP30];

retour confiance

IF ((S6.P9QFEELH EQ 104) OR (S6.P9QFEELF EQ 104)) [S7_P4OPEN];

IF ((S6.P9QFEELH NE 104) AND (S6.P9QFEELF NE 104)) [S7_P4NO];

retour

IF ((S7.P6 EQ 1) AND (CONSENT EQ 1) AND (SI.ING EQ 1) AND (((DATE_OFF EQ 60) AND (TBVAR_TIMENOW GE 600)) OR ((DATE_OFF GE 61) AND (DATE_OFF LE 66)) OR ((DATE_OFF EQ 67) AND (TBVAR_TIMENOW LT 600)))) [S7_P8PLAN7];

IF ((S7.P6 EQ 1) AND (CONSENT EQ 1) AND (SI.ING EQ 1) AND (((DATE_OFF EQ 67) AND (TBVAR_TIMENOW GE 600)) OR ((DATE_OFF GE 68) AND (DATE_OFF LE 73)) OR ((DATE_OFF EQ 74) AND (TBVAR_TIMENOW LE 600)))) [S7_P8PLAN8];

IF (S7.P6 EQ 3) [S7_P8ACCUEIL];

TAILORED MOTIVATIONAL SESSION 8– ADVICE FORMULAS

feedback confiance

IF ((S8.QEFF_MI GT SI_MI_EFFIC) AND (S8.QEFF_MI GE 6)) [S8_P4SUPHIGH];

IF ((S8.QEFF_MI GT SI_MI_EFFIC) AND (S8.QEFF_MI LT 6)) [S8_P4SUPLOW];

IF ((S8.QEFF_MI LT SI_MI_EFFIC) AND (S8.QEFF_MI GE 6)) [S8_P4INFHIGH];

IF ((S8.QEFF_MI LT SI_MI_EFFIC) AND (S8.QEFF_MI LT 6) AND (S8.QEFF_MI NE 0)) [S8_P4INFLOW];

IF ((S8.QEFF_MI LT SI_MI_EFFIC) AND (S8.QEFF_MI EQ 0)) [S8_P4INFZERO];

IF ((S8.QEFF_MI EQ SI_MI_EFFIC) AND (S8.QEFF_MI GE 6) AND (S8.QEFF_MI NE 0)) [S8_P4EQHIGH];

IF ((S8.QEFF_MI EQ SI_MI_EFFIC) AND (S8.QEFF_MI LT 6) AND (S8.QEFF_MI NE 0)) [S8_P4EQLOW];

IF ((S8.QEFF_MI EQ SI_MI_EFFIC) AND (S8.QEFF_MI EQ 0)) [S8_P4EQZERO];

feedback intention

feedback intention

IF ((S8.1INTEN GT S4.INTEN) AND (S8.1INTEN GT 0) AND (S4.ING EQ 1)) [S8.1INTSUPHIGH];

IF ((S8.INTEN GT S4.INTEN) AND (S8.1INTEN LE 0) AND (S4.ING EQ 1)) [S8.1INTSUPLOW];

IF ((S8.1INTEN LT S4.INTEN) AND (S8.1INTEN GT 0) AND (S4.ING EQ 1)) [S8.1INTINFHIGH];

IF ((S8.INTEN LT S4.INTEN) AND (S8.1INTEN LE 0) AND (S4.ING EQ 1)) [S8.1INTINFLOW];

IF ((S8.1INTEN EQ S4.INTEN) AND (S8.1INTEN GT 0) AND (S4.ING EQ 1)) [S8.1INTEQHIGH];

IF ((S8.1INTEN EQ S4.INTEN) AND (S8.1INTEN LE 0) AND (S4.ING EQ 1)) [S8.1INTEQLOW];

IF ((S8.INTEN GT F.INTEN) AND (S8.INTEN GT 0) AND (S4.ING EQ 0)) [S8.INTSUPHIGH];

```
IF ((S8.INTEN GT F.INTEN) AND (S8.INTEN LE 0) AND (S4.ING EQ 0)) [S8.INTSUPLOW];

IF ((S8.INTEN LT F.INTEN) AND (S8.INTEN GT 0) AND (S4.ING EQ 0)) [S8.INTINFHIGH];

IF ((S8.INTEN LT F.INTEN) AND (S8.INTEN LE 0) AND (S4.ING EQ 0)) [S8.INTINFLOW];

IF ((S8.INTEN EQ F.INTEN) AND (S8.INTEN GT 0) AND (S4.ING EQ 0)) [S8.INTEQHIGH];

IF ((S8.INTEN EQ F.INTEN) AND (S8.INTEN LE 0) AND (S4.ING EQ 0)) [S8.INTEQLOW];

*feedback ap*

IF ((S8.APTOT GT F.APTOT) AND (S8.APTOT LT 150) AND (S4.ING EQ 0)) [S8_P8PASUP];

IF ((S8.APTOT GT F.APTOT) AND (S8.APTOT GE 150) AND (SEXE EQ 1) AND (S4.ING EQ 0)) [S8_P8AP150H];

IF ((S8.APTOT GT F.APTOT) AND (S8.APTOT GE 150) AND (SEXE EQ 2) AND (S4.ING EQ 0)) [S8_P8AP150F];

IF ((S8.APTOT LT F.APTOT) AND (S4.ING EQ 0)) [S8_P8APINF];

IF ((S8.APTOT EQ F.APTOT) AND (S4.ING EQ 0)) [S8_P8EGAL];

IF ((S8.APTOT GT S4.APTOT) AND (S8.APTOT LT 150) AND (S4.ING EQ 1)) [S8_1P8PASUP];

IF ((S8.APTOT GE 150) AND (SEXE EQ 1) AND (S4.ING EQ 1)) [S8_1P8AP150H];

IF ((S8.APTOT GE 150) AND (SEXE EQ 2) AND (S4.ING EQ 1)) [S8_1P8AP150F];

IF ((S8.APTOT LT S4.APTOT) AND (S8.APTOT LT 150) AND (S4.ING EQ 1)) [S8_1P8APINF];

IF ((S8.APTOT EQ S4.APTOT) AND (S8.APTOT LT 150) AND (S4.ING EQ 1)) [S8_1P8EGAL];

[S8_P8CONCLU];
```

ACTION PLAN WEEK 1 – ADVICE FORMULAS

routing si aucune barriers versus barriers

IF ((P1_P2 EQ 2) OR ((P1_P2 EQ 1) AND (P1.P7BARCHOICE EQ 110) AND (P1.Q_BARANSWER EQ 0)))
[P1__P4];

skip solution question

IF (P1.P7BARCHOICE NE 101) [SKIP];

IF (P1.P7BARCHOICE NE 102) [SKIP];

IF (P1.P7BARCHOICE NE 103) [SKIP];

IF (P1.P7BARCHOICE NE 104) [SKIP];

IF (P1.P7BARCHOICE NE 105) [SKIP];

IF (P1.P7BARCHOICE NE 106) [SKIP];

IF (P1.P7BARCHOICE NE 107) [SKIP];

IF (P1.P7BARCHOICE NE 108) [SKIP];

IF (P1.P7BARCHOICE NE 109) [SKIP];

IF (P1.P7BARCHOICE NE 110) [SKIP];

feedback solution

IF (((P1_P2 EQ 1) AND (P1.P7BARCHOICE GE 101) AND (P1.P7BARCHOICE LE 109)) OR ((P1_P2 EQ 1) AND (P1.P7BARCHOICE EQ 110) AND (P1.Q_BARANSWER NE 0))) [P1.0P4];

IF ((P1_P2 EQ 2) OR ((P1_P2 EQ 1) AND (P1.P7BARCHOICE EQ 110) AND (P1.Q_BARANSWER EQ 0))) [P1.P4NOOBSTACLE];

question raison

IF (P1_P4OBJECTIF GT 0) [P1_P7];

IF (P1_P4OBJECTIF LE 0) [P1_0P7];

résumé plan

IF (P1.SUMAP EQ 0) [P1_P80];

IF ((P1.SUMAP GE 100) AND (P1.SUMAP LT 200) AND ((P1_P2 EQ 2) OR ((P1_P2 EQ 1) AND (P1.P7BARCHOICE EQ 110) AND (P1.Q_BARANSWER EQ 0)))) [P1_NOOBP81];

IF ((P1.SUMAP GE 200) AND (P1.SUMAP LT 300) AND ((P1_P2 EQ 2) OR ((P1_P2 EQ 1) AND (P1.P7BARCHOICE EQ 110) AND (P1.Q_BARANSWER EQ 0)))) [P1_NOOBP82];

IF ((P1.SUMAP GE 300) AND (P1.SUMAP LT 400) AND ((P1_P2 EQ 2) OR ((P1_P2 EQ 1) AND (P1.P7BARCHOICE EQ 110) AND (P1.Q_BARANSWER EQ 0)))) [P1_NOOBP83];

IF ((P1.SUMAP GE 100) AND (P1.SUMAP LT 200) AND (((P1_P2 EQ 1) AND (P1.P7BARCHOICE GE 101) AND (P1.P7BARCHOICE LE 109)) OR ((P1_P2 EQ 1) AND (P1.P7BARCHOICE EQ 110) AND (P1.Q_BARANSWER NE 0)))) [P1_P81];

IF ((P1.SUMAP GE 200) AND (P1.SUMAP LT 300) AND (((P1_P2 EQ 1) AND (P1.P7BARCHOICE GE 101) AND (P1.P7BARCHOICE LE 109)) OR ((P1_P2 EQ 1) AND (P1.P7BARCHOICE EQ 110) AND (P1.Q_BARANSWER NE 0)))) [P1_P82];

IF ((P1.SUMAP GE 300) AND (P1.SUMAP LT 400) AND (((P1_P2 EQ 1) AND (P1.P7BARCHOICE GE 101) AND (P1.P7BARCHOICE LE 109)) OR ((P1_P2 EQ 1) AND (P1.P7BARCHOICE EQ 110) AND (P1.Q_BARANSWER NE 0)))) [P1_P83];

ACTION PLAN WEEK 2–ADVICE FORMULAS

routing si aucune barriers versus barriers

IF ((P2_P2 EQ 2) OR ((P2_P2 EQ 1) AND (P2.P7BARCHOICE EQ 110) AND (P2.Q_BARANSWER EQ 0))) [P2__P4];

skip solution question

IF (P2.P7BARCHOICE NE 101) [SKIP];

IF (P2.P7BARCHOICE NE 102) [SKIP];

IF (P2.P7BARCHOICE NE 103) [SKIP];

IF (P2.P7BARCHOICE NE 104) [SKIP];

IF (P2.P7BARCHOICE NE 105) [SKIP];

IF (P2.P7BARCHOICE NE 106) [SKIP];

IF (P2.P7BARCHOICE NE 107) [SKIP];

IF (P2.P7BARCHOICE NE 108) [SKIP];

IF (P2.P7BARCHOICE NE 109) [SKIP];

IF (P2.P7BARCHOICE NE 110) [SKIP];

feedback solution

IF (((P2_P2 EQ 1) AND (P2.P7BARCHOICE GE 101) AND (P2.P7BARCHOICE LE 109)) OR ((P2_P2 EQ 1) AND (P2.P7BARCHOICE EQ 110) AND (P2.Q_BARANSWER NE 0))) [P2.0P4];

IF ((P2_P2 EQ 2) OR ((P2_P2 EQ 1) AND (P2.P7BARCHOICE EQ 110) AND (P2.Q_BARANSWER EQ 0))) [P2.P4NOOBSTACLE];

question raison

IF (P2_P4OBJECTIF GT 0) [P2_P7];

IF (P2_P4OBJECTIF LE 0) [P2_0P7];

résumé plan

IF (P2.SUMAP EQ 0) [P2_P80];

IF ((P2.SUMAP GE 100) AND (P2.SUMAP LT 200) AND ((P2_P2 EQ 2) OR ((P2_P2 EQ 1) AND (P2.P7BARCHOICE EQ 110) AND (P2.Q_BARANSWER EQ 0)))) [P2_NOOBP81];

IF ((P2.SUMAP GE 200) AND (P2.SUMAP LT 300) AND ((P2_P2 EQ 2) OR ((P2_P2 EQ 1) AND (P2.P7BARCHOICE EQ 110) AND (P2.Q_BARANSWER EQ 0)))) [P2_NOOBP82];

IF ((P2.SUMAP GE 300) AND (P2.SUMAP LT 400) AND ((P2_P2 EQ 2) OR ((P2_P2 EQ 1) AND (P2.P7BARCHOICE EQ 110) AND (P2.Q_BARANSWER EQ 0)))) [P2_NOOBP83];

IF ((P2.SUMAP GE 100) AND (P2.SUMAP LT 200) AND (((P2_P2 EQ 1) AND (P2.P7BARCHOICE GE 101) AND (P2.P7BARCHOICE LE 109)) OR ((P2_P2 EQ 1) AND (P2.P7BARCHOICE EQ 110) AND (P2.Q_BARANSWER NE 0)))) [P2_P81];

IF ((P2.SUMAP GE 200) AND (P2.SUMAP LT 300) AND (((P2_P2 EQ 1) AND (P2.P7BARCHOICE GE 101) AND (P2.P7BARCHOICE LE 109)) OR ((P2_P2 EQ 1) AND (P2.P7BARCHOICE EQ 110) AND (P2.Q_BARANSWER NE 0)))) [P2_P82];

IF ((P2.SUMAP GE 300) AND (P2.SUMAP LT 400) AND (((P2_P2 EQ 1) AND (P2.P7BARCHOICE GE 101) AND (P2.P7BARCHOICE LE 109)) OR ((P2_P2 EQ 1) AND (P2.P7BARCHOICE EQ 110) AND (P2.Q_BARANSWER NE 0)))) [P2_P83];

ACTION PLAN WEEK 3–ADVICE FORMULAS

routing si aucune barriers versus barriers

IF ((P3_P2 EQ 2) OR ((P3_P2 EQ 1) AND (P3.P7BARCHOICE EQ 110) AND (P3.Q_BARANSWER EQ 0))) [P3__P4];

skip solution question

IF (P3.P7BARCHOICE NE 101) [SKIP];

IF (P3.P7BARCHOICE NE 102) [SKIP];

IF (P3.P7BARCHOICE NE 103) [SKIP];

IF (P3.P7BARCHOICE NE 104) [SKIP];

IF (P3.P7BARCHOICE NE 105) [SKIP];

IF (P3.P7BARCHOICE NE 106) [SKIP];

IF (P3.P7BARCHOICE NE 107) [SKIP];

IF (P3.P7BARCHOICE NE 108) [SKIP];

IF (P3.P7BARCHOICE NE 109) [SKIP];

IF (P3.P7BARCHOICE NE 110) [SKIP];

feedback solution

IF (((P3_P2 EQ 1) AND (P3.P7BARCHOICE GE 101) AND (P3.P7BARCHOICE LE 109)) OR ((P3_P2 EQ 1) AND (P3.P7BARCHOICE EQ 110) AND (P3.Q_BARANSWER NE 0))) [P3.0P4];

IF ((P3_P2 EQ 2) OR ((P3_P2 EQ 1) AND (P3.P7BARCHOICE EQ 110) AND (P3.Q_BARANSWER EQ 0))) [P3.P4NOOBSTACLE];

question raison

IF (P3_P4OBJECTIF GT 0) [P3_P7];

IF (P3_P4OBJECTIF LE 0) [P3_0P7];

résumé plan

IF (P3.SUMAP EQ 0) [P3_P80];

IF ((P3.SUMAP GE 100) AND (P3.SUMAP LT 200) AND ((P3_P2 EQ 2) OR ((P3_P2 EQ 1) AND (P3.P7BARCHOICE EQ 110) AND (P3.Q_BARANSWER EQ 0)))) [P3_NOOBP81];

IF ((P3.SUMAP GE 200) AND (P3.SUMAP LT 300) AND ((P3_P2 EQ 2) OR ((P3_P2 EQ 1) AND (P3.P7BARCHOICE EQ 110) AND (P3.Q_BARANSWER EQ 0)))) [P3_NOOBP82];

IF ((P3.SUMAP GE 300) AND (P3.SUMAP LT 400) AND ((P3_P2 EQ 2) OR ((P3_P2 EQ 1) AND (P3.P7BARCHOICE EQ 110) AND (P3.Q_BARANSWER EQ 0)))) [P3_NOOBP83];

IF ((P3.SUMAP GE 100) AND (P3.SUMAP LT 200) AND (((P3_P2 EQ 1) AND (P3.P7BARCHOICE GE 101) AND (P3.P7BARCHOICE LE 109)) OR ((P3_P2 EQ 1) AND (P3.P7BARCHOICE EQ 110) AND (P3.Q_BARANSWER NE 0)))) [P3_P81];

IF ((P3.SUMAP GE 200) AND (P3.SUMAP LT 300) AND (((P3_P2 EQ 1) AND (P3.P7BARCHOICE GE 101) AND (P3.P7BARCHOICE LE 109)) OR ((P3_P2 EQ 1) AND (P3.P7BARCHOICE EQ 110) AND (P3.Q_BARANSWER NE 0)))) [P3_P82];

IF ((P3.SUMAP GE 300) AND (P3.SUMAP LT 400) AND (((P3_P2 EQ 1) AND (P3.P7BARCHOICE GE 101) AND (P3.P7BARCHOICE LE 109)) OR ((P3_P2 EQ 1) AND (P3.P7BARCHOICE EQ 110) AND (P3.Q_BARANSWER NE 0)))) [P3_P83];

ACTION PLAN WEEK 4–ADVICE FORMULAS

routing si aucune barriers versus barriers

IF ((P4_P2 EQ 2) OR ((P4_P2 EQ 1) AND (P4.P7BARCHOICE EQ 110) AND (P4.Q_BARANSWER EQ 0))) [P4__P4];

skip solution question

IF (P4.P7BARCHOICE NE 101) [SKIP];

IF (P4.P7BARCHOICE NE 102) [SKIP];

IF (P4.P7BARCHOICE NE 103) [SKIP];

IF (P4.P7BARCHOICE NE 104) [SKIP];

IF (P4.P7BARCHOICE NE 105) [SKIP];

IF (P4.P7BARCHOICE NE 106) [SKIP];

IF (P4.P7BARCHOICE NE 107) [SKIP];

IF (P4.P7BARCHOICE NE 108) [SKIP];

IF (P4.P7BARCHOICE NE 109) [SKIP];

IF (P4.P7BARCHOICE NE 110) [SKIP];

feedback solution

IF (((P4_P2 EQ 1) AND (P4.P7BARCHOICE GE 101) AND (P4.P7BARCHOICE LE 109)) OR ((P4_P2 EQ 1) AND (P4.P7BARCHOICE EQ 110) AND (P4.Q_BARANSWER NE 0))) [P4.0P4];

IF ((P4_P2 EQ 2) OR ((P4_P2 EQ 1) AND (P4.P7BARCHOICE EQ 110) AND (P4.Q_BARANSWER EQ 0))) [P4.P4NOOBSTACLE];

question raison

IF (P4_P4OBJECTIF GT 0) [P4_P7];

IF (P4_P4OBJECTIF LE 0) [P4_0P7];

résumé plan

IF (P4.SUMAP EQ 0) [P4_P80];

IF ((P4.SUMAP GE 100) AND (P4.SUMAP LT 200) AND ((P4_P2 EQ 2) OR ((P4_P2 EQ 1) AND (P4.P7BARCHOICE EQ 110) AND (P4.Q_BARANSWER EQ 0)))) [P4_NOOBP81];

IF ((P4.SUMAP GE 200) AND (P4.SUMAP LT 300) AND ((P4_P2 EQ 2) OR ((P4_P2 EQ 1) AND (P4.P7BARCHOICE EQ 110) AND (P4.Q_BARANSWER EQ 0)))) [P4_NOOBP82];

IF ((P4.SUMAP GE 300) AND (P4.SUMAP LT 400) AND ((P4_P2 EQ 2) OR ((P4_P2 EQ 1) AND (P4.P7BARCHOICE EQ 110) AND (P4.Q_BARANSWER EQ 0)))) [P4_NOOBP83];

IF ((P4.SUMAP GE 100) AND (P4.SUMAP LT 200) AND (((P4_P2 EQ 1) AND (P4.P7BARCHOICE GE 101) AND (P4.P7BARCHOICE LE 109)) OR ((P4_P2 EQ 1) AND (P4.P7BARCHOICE EQ 110) AND (P4.Q_BARANSWER NE 0)))) [P4_P81];

IF ((P4.SUMAP GE 200) AND (P4.SUMAP LT 300) AND (((P4_P2 EQ 1) AND (P4.P7BARCHOICE GE 101) AND (P4.P7BARCHOICE LE 109)) OR ((P4_P2 EQ 1) AND (P4.P7BARCHOICE EQ 110) AND (P4.Q_BARANSWER NE 0)))) [P4_P82];

IF ((P4.SUMAP GE 300) AND (P4.SUMAP LT 400) AND (((P4_P2 EQ 1) AND (P4.P7BARCHOICE GE 101) AND (P4.P7BARCHOICE LE 109)) OR ((P4_P2 EQ 1) AND (P4.P7BARCHOICE EQ 110) AND (P4.Q_BARANSWER NE 0)))) [P4_P83];

ACTION PLAN WEEK 5–ADVICE FORMULAS

routing si aucune barriers versus barriers

IF ((P5_P2 EQ 2) OR ((P5_P2 EQ 1) AND (P5.P7BARCHOICE EQ 110) AND (P5.Q_BARANSWER EQ 0))) [P5__P4];

skip solution question

IF (P5.P7BARCHOICE NE 101) [SKIP];

IF (P5.P7BARCHOICE NE 102) [SKIP];

IF (P5.P7BARCHOICE NE 103) [SKIP];

IF (P5.P7BARCHOICE NE 104) [SKIP];

IF (P5.P7BARCHOICE NE 105) [SKIP];

IF (P5.P7BARCHOICE NE 106) [SKIP];

IF (P5.P7BARCHOICE NE 107) [SKIP];

IF (P5.P7BARCHOICE NE 108) [SKIP];

IF (P5.P7BARCHOICE NE 109) [SKIP];

IF (P5.P7BARCHOICE NE 110) [SKIP];

feedback solution

IF (((P5_P2 EQ 1) AND (P5.P7BARCHOICE GE 101) AND (P5.P7BARCHOICE LE 109)) OR ((P5_P2 EQ 1) AND (P5.P7BARCHOICE EQ 110) AND (P5.Q_BARANSWER NE 0))) [P5.0P4];

IF ((P5_P2 EQ 2) OR ((P5_P2 EQ 1) AND (P5.P7BARCHOICE EQ 110) AND (P5.Q_BARANSWER EQ 0))) [P5.P4NOOBSTACLE];

question raison

IF (P5_P4OBJECTIF GT 0) [P5_P7];

IF (P5_P4OBJECTIF LE 0) [P5_0P7];

résumé plan

IF (P5.SUMAP EQ 0) [P5_P80];

IF ((P5.SUMAP GE 100) AND (P5.SUMAP LT 200) AND ((P5_P2 EQ 2) OR ((P5_P2 EQ 1) AND (P5.P7BARCHOICE EQ 110) AND (P5.Q_BARANSWER EQ 0)))) [P5_NOOBP81];

IF ((P5.SUMAP GE 200) AND (P5.SUMAP LT 300) AND ((P5_P2 EQ 2) OR ((P5_P2 EQ 1) AND (P5.P7BARCHOICE EQ 110) AND (P5.Q_BARANSWER EQ 0)))) [P5_NOOBP82];

IF ((P5.SUMAP GE 300) AND (P5.SUMAP LT 400) AND ((P5_P2 EQ 2) OR ((P5_P2 EQ 1) AND (P5.P7BARCHOICE EQ 110) AND (P5.Q_BARANSWER EQ 0)))) [P5_NOOBP83];

IF ((P5.SUMAP GE 100) AND (P5.SUMAP LT 200) AND (((P5_P2 EQ 1) AND (P5.P7BARCHOICE GE 101) AND (P5.P7BARCHOICE LE 109)) OR ((P5_P2 EQ 1) AND (P5.P7BARCHOICE EQ 110) AND (P5.Q_BARANSWER NE 0)))) [P5_P81];

IF ((P5.SUMAP GE 200) AND (P5.SUMAP LT 300) AND (((P5_P2 EQ 1) AND (P5.P7BARCHOICE GE 101) AND (P5.P7BARCHOICE LE 109)) OR ((P5_P2 EQ 1) AND (P5.P7BARCHOICE EQ 110) AND (P5.Q_BARANSWER NE 0)))) [P5_P82];

IF ((P5.SUMAP GE 300) AND (P5.SUMAP LT 400) AND (((P5_P2 EQ 1) AND (P5.P7BARCHOICE GE 101) AND (P5.P7BARCHOICE LE 109)) OR ((P5_P2 EQ 1) AND (P5.P7BARCHOICE EQ 110) AND (P5.Q_BARANSWER NE 0)))) [P5_P83];

ACTION PLAN WEEK 6–ADVICE FORMULAS

routing si aucune barriers versus barriers

IF ((P6_P2 EQ 2) OR ((P6_P2 EQ 1) AND (P6.P7BARCHOICE EQ 110) AND (P6.Q_BARANSWER EQ 0))) [P6__P4];

skip solution question

IF (P6.P7BARCHOICE NE 101) [SKIP];

IF (P6.P7BARCHOICE NE 102) [SKIP];

IF (P6.P7BARCHOICE NE 103) [SKIP];

IF (P6.P7BARCHOICE NE 104) [SKIP];

IF (P6.P7BARCHOICE NE 105) [SKIP];

IF (P6.P7BARCHOICE NE 106) [SKIP];

IF (P6.P7BARCHOICE NE 107) [SKIP];

IF (P6.P7BARCHOICE NE 108) [SKIP];

IF (P6.P7BARCHOICE NE 109) [SKIP];

IF (P6.P7BARCHOICE NE 110) [SKIP];

feedback solution

IF (((P6_P2 EQ 1) AND (P6.P7BARCHOICE GE 101) AND (P6.P7BARCHOICE LE 109)) OR ((P6_P2 EQ 1) AND (P6.P7BARCHOICE EQ 110) AND (P6.Q_BARANSWER NE 0))) [P6.0P4];

IF ((P6_P2 EQ 2) OR ((P6_P2 EQ 1) AND (P6.P7BARCHOICE EQ 110) AND (P6.Q_BARANSWER EQ 0))) [P6.P4NOOBSTACLE];

question raison

IF (P6_P4OBJECTIF GT 0) [P6_P7];

IF (P6_P4OBJECTIF LE 0) [P6_0P7];

résumé plan

IF (P6.SUMAP EQ 0) [P6_P80];

IF ((P6.SUMAP GE 100) AND (P6.SUMAP LT 200) AND ((P6_P2 EQ 2) OR ((P6_P2 EQ 1) AND (P6.P7BARCHOICE EQ 110) AND (P6.Q_BARANSWER EQ 0)))) [P6_NOOBP81];

IF ((P6.SUMAP GE 200) AND (P6.SUMAP LT 300) AND ((P6_P2 EQ 2) OR ((P6_P2 EQ 1) AND (P6.P7BARCHOICE EQ 110) AND (P6.Q_BARANSWER EQ 0)))) [P6_NOOBP82];

IF ((P6.SUMAP GE 300) AND (P6.SUMAP LT 400) AND ((P6_P2 EQ 2) OR ((P6_P2 EQ 1) AND (P6.P7BARCHOICE EQ 110) AND (P6.Q_BARANSWER EQ 0)))) [P6_NOOBP83];

IF ((P6.SUMAP GE 100) AND (P6.SUMAP LT 200) AND (((P6_P2 EQ 1) AND (P6.P7BARCHOICE GE 101) AND (P6.P7BARCHOICE LE 109)) OR ((P6_P2 EQ 1) AND (P6.P7BARCHOICE EQ 110) AND (P6.Q_BARANSWER NE 0)))) [P6_P81];

IF ((P6.SUMAP GE 200) AND (P6.SUMAP LT 300) AND (((P6_P2 EQ 1) AND (P6.P7BARCHOICE GE 101) AND (P6.P7BARCHOICE LE 109)) OR ((P6_P2 EQ 1) AND (P6.P7BARCHOICE EQ 110) AND (P6.Q_BARANSWER NE 0)))) [P6_P82];

IF ((P6.SUMAP GE 300) AND (P6.SUMAP LT 400) AND (((P6_P2 EQ 1) AND (P6.P7BARCHOICE GE 101) AND (P6.P7BARCHOICE LE 109)) OR ((P6_P2 EQ 1) AND (P6.P7BARCHOICE EQ 110) AND (P6.Q_BARANSWER NE 0)))) [P6_P83];

ACTION PLAN WEEK 7–ADVICE FORMULAS

routing si aucune barriers versus barriers

IF ((P7_P2 EQ 2) OR ((P7_P2 EQ 1) AND (P7.P7BARCHOICE EQ 110) AND (P7.Q_BARANSWER EQ 0))) [P7__P4];

skip solution question

IF (P7.P7BARCHOICE NE 101) [SKIP];

IF (P7.P7BARCHOICE NE 102) [SKIP];

IF (P7.P7BARCHOICE NE 103) [SKIP];

IF (P7.P7BARCHOICE NE 104) [SKIP];

IF (P7.P7BARCHOICE NE 105) [SKIP];

IF (P7.P7BARCHOICE NE 106) [SKIP];

IF (P7.P7BARCHOICE NE 107) [SKIP];

IF (P7.P7BARCHOICE NE 108) [SKIP];

IF (P7.P7BARCHOICE NE 109) [SKIP];

IF (P7.P7BARCHOICE NE 110) [SKIP];

feedback solution

IF (((P7_P2 EQ 1) AND (P7.P7BARCHOICE GE 101) AND (P7.P7BARCHOICE LE 109)) OR ((P7_P2 EQ 1) AND (P7.P7BARCHOICE EQ 110) AND (P7.Q_BARANSWER NE 0))) [P7.0P4];

IF ((P7_P2 EQ 2) OR ((P7_P2 EQ 1) AND (P7.P7BARCHOICE EQ 110) AND (P7.Q_BARANSWER EQ 0))) [P7.P4NOOBSTACLE];

question raison

IF (P7_P4OBJECTIF GT 0) [P7_P7];

IF (P7_P4OBJECTIF LE 0) [P7_0P7];

résumé plan

IF (P7.SUMAP EQ 0) [P7_P80];

IF ((P7.SUMAP GE 100) AND (P7.SUMAP LT 200) AND ((P7_P2 EQ 2) OR ((P7_P2 EQ 1) AND (P7.P7BARCHOICE EQ 110) AND (P7.Q_BARANSWER EQ 0)))) [P7_NOOBP81];

IF ((P7.SUMAP GE 200) AND (P7.SUMAP LT 300) AND ((P7_P2 EQ 2) OR ((P7_P2 EQ 1) AND (P7.P7BARCHOICE EQ 110) AND (P7.Q_BARANSWER EQ 0)))) [P7_NOOBP82];

IF ((P7.SUMAP GE 300) AND (P7.SUMAP LT 400) AND ((P7_P2 EQ 2) OR ((P7_P2 EQ 1) AND (P7.P7BARCHOICE EQ 110) AND (P7.Q_BARANSWER EQ 0)))) [P7_NOOBP83];

IF ((P7.SUMAP GE 100) AND (P7.SUMAP LT 200) AND (((P7_P2 EQ 1) AND (P7.P7BARCHOICE GE 101) AND (P7.P7BARCHOICE LE 109)) OR ((P7_P2 EQ 1) AND (P7.P7BARCHOICE EQ 110) AND (P7.Q_BARANSWER NE 0)))) [P7_P81];

IF ((P7.SUMAP GE 200) AND (P7.SUMAP LT 300) AND (((P7_P2 EQ 1) AND (P7.P7BARCHOICE GE 101) AND (P7.P7BARCHOICE LE 109)) OR ((P7_P2 EQ 1) AND (P7.P7BARCHOICE EQ 110) AND (P7.Q_BARANSWER NE 0)))) [P7_P82];

IF ((P7.SUMAP GE 300) AND (P7.SUMAP LT 400) AND (((P7_P2 EQ 1) AND (P7.P7BARCHOICE GE 101) AND (P7.P7BARCHOICE LE 109)) OR ((P7_P2 EQ 1) AND (P7.P7BARCHOICE EQ 110) AND (P7.Q_BARANSWER NE 0)))) [P7_P83];

ACTION PLAN WEEK 8–ADVICE FORMULAS

routing si aucune barriers versus barriers

IF ((P8_P2 EQ 2) OR ((P8_P2 EQ 1) AND (P8.P7BARCHOICE EQ 110) AND (P8.Q_BARANSWER EQ 0))) [P8__P4];

skip solution question

IF (P8.P7BARCHOICE NE 101) [SKIP];

IF (P8.P7BARCHOICE NE 102) [SKIP];

IF (P8.P7BARCHOICE NE 103) [SKIP];

IF (P8.P7BARCHOICE NE 104) [SKIP];

IF (P8.P7BARCHOICE NE 105) [SKIP];

IF (P8.P7BARCHOICE NE 106) [SKIP];

IF (P8.P7BARCHOICE NE 107) [SKIP];

IF (P8.P7BARCHOICE NE 108) [SKIP];

IF (P8.P7BARCHOICE NE 109) [SKIP];

IF (P8.P7BARCHOICE NE 110) [SKIP];

feedback solution

IF (((P8_P2 EQ 1) AND (P8.P7BARCHOICE GE 101) AND (P8.P7BARCHOICE LE 109)) OR ((P8_P2 EQ 1) AND (P8.P7BARCHOICE EQ 110) AND (P8.Q_BARANSWER NE 0))) [P8.0P4];

IF ((P8_P2 EQ 2) OR ((P8_P2 EQ 1) AND (P8.P7BARCHOICE EQ 110) AND (P8.Q_BARANSWER EQ 0))) [P8.P4NOOBSTACLE];

question raison

IF (P8_P4OBJECTIF GT 0) [P8_P7];

IF (P8_P4OBJECTIF LE 0) [P8_0P7];

résumé plan

IF (P8.SUMAP EQ 0) [P8_P80];

IF ((P8.SUMAP GE 100) AND (P8.SUMAP LT 200) AND ((P8_P2 EQ 2) OR ((P8_P2 EQ 1) AND (P8.P7BARCHOICE EQ 110) AND (P8.Q_BARANSWER EQ 0)))) [P8_NOOBP81];

IF ((P8.SUMAP GE 200) AND (P8.SUMAP LT 300) AND ((P8_P2 EQ 2) OR ((P8_P2 EQ 1) AND (P8.P7BARCHOICE EQ 110) AND (P8.Q_BARANSWER EQ 0)))) [P8_NOOBP82];

IF ((P8.SUMAP GE 300) AND (P8.SUMAP LT 400) AND ((P8_P2 EQ 2) OR ((P8_P2 EQ 1) AND (P8.P7BARCHOICE EQ 110) AND (P8.Q_BARANSWER EQ 0)))) [P8_NOOBP83];

IF ((P8.SUMAP GE 100) AND (P8.SUMAP LT 200) AND (((P8_P2 EQ 1) AND (P8.P7BARCHOICE GE 101) AND (P8.P7BARCHOICE LE 109)) OR ((P8_P2 EQ 1) AND (P8.P7BARCHOICE EQ 110) AND (P8.Q_BARANSWER NE 0)))) [P8_P81];

IF ((P8.SUMAP GE 200) AND (P8.SUMAP LT 300) AND (((P8_P2 EQ 1) AND (P8.P7BARCHOICE GE 101) AND (P8.P7BARCHOICE LE 109)) OR ((P8_P2 EQ 1) AND (P8.P7BARCHOICE EQ 110) AND (P8.Q_BARANSWER NE 0)))) [P8_P82];

IF ((P8.SUMAP GE 300) AND (P8.SUMAP LT 400) AND (((P8_P2 EQ 1) AND (P8.P7BARCHOICE GE 101) AND (P8.P7BARCHOICE LE 109)) OR ((P8_P2 EQ 1) AND (P8.P7BARCHOICE EQ 110) AND (P8.Q_BARANSWER NE 0)))) [P8_P83];

STYLESHEET - MENU OF THE ACTION PLAN TOOL

```
div {
    color:#00cc00;
    font-weight:bold;
    font-size:28px;
    text-align:center;
    }

a {
    -moz-box-shadow:inset 0px 1px 0px 0px #bbdaf7;
    -webkit-box-shadow:inset 0px 1px 0px 0px #bbdaf7;
    box-shadow:inset 0px 1px 0px 0px #bbdaf7;
    background:-webkit-gradient( linear, left top, left bottom, color-stop(0.05, #79bbff), color-stop(1,
#378de5) );
    background:-moz-linear-gradient( center top, #79bbff 5%, #378de5 100% );
    filter:progid:DXImageTransform.Microsoft.gradient(startColorstr='#79bbff', endColorstr='#378de5');
    background-color:#79bbff;
    -webkit-border-top-left-radius:10px;
    -moz-border-radius-topleft:10px;
    border-top-left-radius:10px;
    -webkit-border-top-right-radius:10px;
    -moz-border-radius-topright:10px;
    border-top-right-radius:10px;
    -webkit-border-bottom-right-radius:10px;
    -moz-border-radius-bottomright:10px;
    border-bottom-right-radius:10px;
```

```
   -webkit-border-bottom-left-radius:10px;
   -moz-border-radius-bottomleft:10px;
   border-bottom-left-radius:10px;
   text-indent:0;
   border:1px solid #84bbf3;
   display:inline-block;
   color:#ffffff;
   font-family:Arial;
   font-size:16px;
   font-weight:bold;
   font-style:normal;
   height:35px;
   line-height:35px;
   width:136px;
   text-decoration:none;
   text-align:center;
   text-shadow:1px 1px 0px #528ecc;
}
a:hover {
   background:-webkit-gradient( linear, left top, left bottom, color-stop(0.05, #378de5), color-stop(1,
#79bbff) );
   background:-moz-linear-gradient( center top, #378de5 5%, #79bbff 100% );
   filter:progid:DXImageTransform.Microsoft.gradient(startColorstr='#378de5', endColorstr='#79bbff');
   background-color:#378de5;
}
a:active {
```

```
   position:relative;
   top:1px;
}

/*classes pour sp_accueil*/

.reduce {
   font-size:18px;
   color:black;
   text-align:center;
   }

.green {
   font-size:18px;
   color:#00cc00;
   font-weight:bold;
   text-align:center;
   }
.grey {
   font-size:18px;
   color:#b3b3b3;
   font-weight:700;
   text-align:center;

   }
```

```
.blue {
   font-size:16px;
   color:#4285f4;
   font-weight:700;
   text-align:center;

   }

.bigblue {
   font-size:18px;
   color:#4285f4;
   font-weight:700;
   text-align:center;

   }
      /*class pour hr*/
.hrblue {
    color:#4285f4;
    background-color:#4285f4;
    height:10px;
    border:none;
    width: auto;

}
/*for home page*/
```

```
.welcome {
   margin:auto;
   border:10px outset EDEDF3;
   background-color:4285f4;
   text-align:center;
   padding:3 white;
   }


.white {
   color:white;
   font-weight:bold;
   font-size:24px;
   text-align:center;

}
.smallwhite {
   color:white;
   font-weight:bold;
   font-size:20px;

}
.black {
   color:black;
   font-weight:bold;
```

```
   font-size:24px;
}

table tbody tr strong a {
   color:yellow;
   font-weight:bold;
   text-decoration: none;
   font-size: 22px;
   }

table tbody tr strong a:hover {
   color:yellow;
   font-weight:bold;
   text-decoration:underline;
   font-size:22px;
   }

img.trans {
   opacity: 0.3;
   filter: alpha(opacity=40); /* For IE8 and earlier */
}
```

STYLE SHEET - MOTIVATIONAL SESSIONS, TUTORIAL and APPRECIATION

```
table.box {

    border-right: 5px solid #4285f4;

    border-left: 5px solid #4285f4;

    border-bottom: 5px solid #4285f4;

    border-top: 5px solid #4285f4;

}

body {

 font-family:Arial, Helvetica, Sans-serif;

}

.plan {

    -moz-box-shadow:inset 0px 1px 0px 0px #fafafa;

    -webkit-box-shadow:inset 0px 1px 0px 0px #fafafa;

    box-shadow:inset 0px 1px 0px 0px #fafafa;

    background:-webkit-gradient( linear, left top, left bottom, color-stop(0.05, #9fed9f), color-stop(1,
#00cc00) );

    background:-moz-linear-gradient( center top, #9fed9f 5%, #00cc00 100% );

    filter:progid:DXImageTransform.Microsoft.gradient(startColorstr='#9fed9f', endColorstr='#00cc00');

    background-color:#9fed9f;

    -webkit-border-top-left-radius:20px;

    -moz-border-radius-topleft:20px;

    border-top-left-radius:20px;

    -webkit-border-top-right-radius:20px;

    -moz-border-radius-topright:20px;

    border-top-right-radius:20px;

    -webkit-border-bottom-right-radius:20px;
```

```
    -moz-border-radius-bottomright:20px;
    border-bottom-right-radius:20px;
    -webkit-border-bottom-left-radius:20px;
    -moz-border-radius-bottomleft:20px;
    border-bottom-left-radius:20px;
    text-indent:0;
    border:1px solid #4286f4;
    display:inline-block;
    color:#ffffff;
    font-family:Arial, Helvetica, Sans-serif;
    font-size:15px;
    font-weight:bold;
    font-style:normal;
    height:75px;
    line-height:75px;
    width:200px;
    text-decoration:none;
    text-align:center;
    text-shadow:1px 1px 0px #00cc00;
}
.plan:hover {
    background:-webkit-gradient( linear, left top, left bottom, color-stop(0.05, #00cc00), color-stop(1,
#9fed9f) );
    background:-moz-linear-gradient( center top, #00cc00 5%, #9fed9f 100% );
    filter:progid:DXImageTransform.Microsoft.gradient(startColorstr='#00cc00', endColorstr='#9fed9f');
    background-color:#00cc00;
```

```
}
.plan:active {
    position:relative;
    top:1px;
}
.patype {
    margin:0px;padding:0px;
    width:100%;
    box-shadow: 10px 10px 5px #888888;
    border:1px solid #4285f4;

    -moz-border-radius-bottomleft:9px;
    -webkit-border-bottom-left-radius:9px;
    border-bottom-left-radius:9px;

    -moz-border-radius-bottomright:9px;
    -webkit-border-bottom-right-radius:9px;
    border-bottom-right-radius:9px;

    -moz-border-radius-topright:9px;
    -webkit-border-top-right-radius:9px;
    border-top-right-radius:9px;

    -moz-border-radius-topleft:9px;
    -webkit-border-top-left-radius:9px;
```

```
    border-top-left-radius:9px;
}
.patype table {
    border-collapse: collapse;
        border-spacing: 0;
    width:100%;
    height:100%;
    margin:0px;padding:0px;
}
.patype tr:last-child td:last-child {
    -moz-border-radius-bottomright:9px;
    -webkit-border-bottom-right-radius:9px;
    border-bottom-right-radius:9px;
}
.patype table tr:first-child td:first-child {
    -moz-border-radius-topleft:9px;
    -webkit-border-top-left-radius:9px;
    border-top-left-radius:9px;
}
.patype table tr:first-child td:last-child {
    -moz-border-radius-topright:9px;
    -webkit-border-top-right-radius:9px;
    border-top-right-radius:9px;
}
.patype tr:last-child td:first-child {
```

```
    -moz-border-radius-bottomleft:9px;

    -webkit-border-bottom-left-radius:9px;

    border-bottom-left-radius:9px;

}

.patype tr:hover td {

    background-color:#ffffff;


}

.patype td {

    vertical-align:middle;

        background:-o-linear-gradient(bottom, #e5f9e5 5%, #ffffff 100%);    background:-webkit-gradient( linear, left top, left bottom, color-stop(0.05, #e5f9e5), color-stop(1, #ffffff) );

    background:-moz-linear-gradient( center top, #e5f9e5 5%, #ffffff 100% );

    filter:progid:DXImageTransform.Microsoft.gradient(startColorstr="#e5f9e5", endColorstr="#ffffff");    background: -o-linear-gradient(top,#e5f9e5,ffffff);

    background-color:#e5f9e5;

    border:1px solid #4285f4;

    border-width:0px 1px 1px 0px;

    text-align:center;

    padding:11px;

    font-size:10px;

    font-family:Arial, Helvetica, Sans-serif;

    font-weight:normal;

    color:#000000;

}

.patype tr:last-child td {
```

```
    border-width:0px 1px 0px 0px;
}
.patype tr td:last-child {
    border-width:0px 0px 1px 0px;
}
.patype tr:last-child td:last-child {
    border-width:0px 0px 0px 0px;
}
.patype tr:first-child td {
        background:-o-linear-gradient(bottom, #4285f4 5%, #aeccfc 100%);    background:-webkit-gradient(
linear, left top, left bottom, color-stop(0.05, #4285f4), color-stop(1, #aeccfc) );
    background:-moz-linear-gradient( center top, #4285f4 5%, #aeccfc 100% );
    filter:progid:DXImageTransform.Microsoft.gradient(startColorstr="#4285f4", endColorstr="#aeccfc");
background: -o-linear-gradient(top,#4285f4,aeccfc);
    background-color:#4285f4;
    border:0px solid #4285f4;
    text-align:center;
    border-width:0px 0px 1px 1px;
    font-size:14px;
    font-family:Arial, Helvetica, Sans-serif;
    font-weight:bold;
    color:#ffffff;
}
.patype tr:first-child:hover td {
    background:-o-linear-gradient(bottom, #4285f4 5%, #aeccfc 100%);    background:-webkit-gradient(
linear, left top, left bottom, color-stop(0.05, #4285f4), color-stop(1, #aeccfc) );
    background:-moz-linear-gradient( center top, #4285f4 5%, #aeccfc 100% );
```

```
    filter:progid:DXImageTransform.Microsoft.gradient(startColorstr="#4285f4", endColorstr="#aeccfc");
background: -o-linear-gradient(top,#4285f4,aeccfc);
    background-color:#4285f4;
}
.patype tr:first-child td:first-child {
    border-width:0px 0px 1px 0px;
}
.patype tr:first-child td:last-child {
    border-width:0px 0px 1px 1px;
}
.paben {
    margin:0px;padding:0px;
    width:100%;
    box-shadow: 10px 10px 5px #888888;
    border:1px solid #4285f4;

    -moz-border-radius-bottomleft:9px;
    -webkit-border-bottom-left-radius:9px;
    border-bottom-left-radius:9px;

    -moz-border-radius-bottomright:9px;
    -webkit-border-bottom-right-radius:9px;
    border-bottom-right-radius:9px;

    -moz-border-radius-topright:9px;
    -webkit-border-top-right-radius:9px;
```

```
    border-top-right-radius:9px;


    -moz-border-radius-topleft:9px;
    -webkit-border-top-left-radius:9px;
    border-top-left-radius:9px;
}.paben table{
    border-collapse: collapse;
        border-spacing: 0;
    width:100%;
    height:100%;
    margin:0px;padding:0px;
}
.paben tr:last-child td:last-child {
    -moz-border-radius-bottomright:9px;
    -webkit-border-bottom-right-radius:9px;
    border-bottom-right-radius:9px;
}
.paben table tr:first-child td:first-child {
    -moz-border-radius-topleft:9px;
    -webkit-border-top-left-radius:9px;
    border-top-left-radius:9px;
}
.paben table tr:first-child td:last-child {
    -moz-border-radius-topright:9px;
    -webkit-border-top-right-radius:9px;
```

```
    border-top-right-radius:9px;
}
.paben tr:last-child td:first-child {
    -moz-border-radius-bottomleft:9px;
    -webkit-border-bottom-left-radius:9px;
    border-bottom-left-radius:9px;
}
.paben tr:hover td {
    background-color:#ffffff;


}
.paben td {
    vertical-align:middle;
        background:-o-linear-gradient(bottom, #e5f9e5 5%, #ffffff 100%);    background:-webkit-gradient( linear, left top, left bottom, color-stop(0.05, #e5f9e5), color-stop(1, #ffffff) );
    background:-moz-linear-gradient( center top, #e5f9e5 5%, #ffffff 100% );
    filter:progid:DXImageTransform.Microsoft.gradient(startColorstr="#e5f9e5", endColorstr="#ffffff"); background: -o-linear-gradient(top,#e5f9e5,ffffff);
    background-color:#e5f9e5;
    border:1px solid #4285f4;
    border-width:0px 1px 1px 0px;
    text-align:left;
    padding:11px;
    font-size:10px;
    font-family:Arial, Helvetica, Sans-serif;
    font-weight:normal;
```

```
   color:#000000;
}
.paben tr:last-child td {
   border-width:0px 1px 0px 0px;
}
.paben tr td:last-child {
   border-width:0px 0px 1px 0px;
}
.paben tr:last-child td:last-child {
   border-width:0px 0px 0px 0px;
}
.paben tr:first-child td {
      background:-o-linear-gradient(bottom, #4285f4 5%, #aeccfc 100%);    background:-webkit-gradient( linear, left top, left bottom, color-stop(0.05, #4285f4), color-stop(1, #aeccfc) );
   background:-moz-linear-gradient( center top, #4285f4 5%, #aeccfc 100% );
   filter:progid:DXImageTransform.Microsoft.gradient(startColorstr="#4285f4", endColorstr="#aeccfc"); background: -o-linear-gradient(top,#4285f4,aeccfc);
   background-color:#4285f4;
   border:0px solid #4285f4;
   text-align:center;
   border-width:0px 0px 1px 1px;
   font-size:14px;
   font-family:Arial, Helvetica, Sans-serif;
   font-weight:bold;
   color:#ffffff;
}
```

```
.paben tr:first-child:hover td {
   background:-o-linear-gradient(bottom, #4285f4 5%, #aeccfc 100%);    background:-webkit-gradient(
linear, left top, left bottom, color-stop(0.05, #4285f4), color-stop(1, #aeccfc) );
   background:-moz-linear-gradient( center top, #4285f4 5%, #aeccfc 100% );
   filter:progid:DXImageTransform.Microsoft.gradient(startColorstr="#4285f4", endColorstr="#aeccfc");
background: -o-linear-gradient(top,#4285f4,aeccfc);
   background-color:#4285f4;
}
.paben tr:first-child td:first-child {
   border-width:0px 0px 1px 0px;
}
.paben tr:first-child td:last-child {
   border-width:0px 0px 1px 1px;
}
#grad {
  background: -webkit-linear-gradient(4285f4, aeccfc); /* For Safari 5.1 to 6.0 */
  background: -o-linear-gradient(4285f4, aeccfc); /* For Opera 11.1 to 12.0 */
  background: -moz-linear-gradient(4285f4, aeccfc); /* For Firefox 3.6 to 15 */
  background: linear-gradient(4285f4, aeccfc); /* Standard syntax */
}
.seances {
   margin:0px;padding:0px;
   width:100%;
   box-shadow: 10px 10px 5px #888888;
   border:1px solid #ffffff;
```

```
    -moz-border-radius-bottomleft:10px;
    -webkit-border-bottom-left-radius:10px;
    border-bottom-left-radius:10px;

    -moz-border-radius-bottomright:10px;
    -webkit-border-bottom-right-radius:10px;
    border-bottom-right-radius:10px;

    -moz-border-radius-topright:10px;
    -webkit-border-top-right-radius:10px;
    border-top-right-radius:10px;

    -moz-border-radius-topleft:10px;
    -webkit-border-top-left-radius:10px;
    border-top-left-radius:10px;
}
.seances table {
    border-collapse: collapse;
        border-spacing: 0;
    width:100%;
    height:100%;
    margin:0px;padding:0px;
}
.seances tr:last-child td:last-child {
    -moz-border-radius-bottomright:10px;
```

```
   -webkit-border-bottom-right-radius:10px;
   border-bottom-right-radius:10px;
}
.seances table tr:first-child td:first-child {
   -moz-border-radius-topleft:10px;
   -webkit-border-top-left-radius:10px;
   border-top-left-radius:10px;
}
.seances table tr:first-child td:last-child {
   -moz-border-radius-topright:10px;
   -webkit-border-top-right-radius:10px;
   border-top-right-radius:10px;
}
.seances tr:last-child td:first-child {
   -moz-border-radius-bottomleft:10px;
   -webkit-border-bottom-left-radius:10px;
   border-bottom-left-radius:10px;
}
.seances tr:hover td {
   background-color:#ffffff;


}
.seances td {
   vertical-align:middle;
      background:-o-linear-gradient(bottom, #6e9fef 5%, #ffffff 100%);    background:-webkit-gradient(
linear, left top, left bottom, color-stop(0.05, #6e9fef), color-stop(1, #ffffff) );
```

```
    background:-moz-linear-gradient( center top, #6e9fef 5%, #ffffff 100% );
    filter:progid:DXImageTransform.Microsoft.gradient(startColorstr="#6e9fef", endColorstr="#ffffff");
background: -o-linear-gradient(top,#6e9fef,ffffff);
    background-color:#6e9fef;
    border:1px solid #ffffff;
    border-width:0px 1px 1px 0px;
    text-align:center;
    padding:7px;
    font-size:10px;
    font-family:Arial, Helvetica, Sans-serif;
    font-weight:normal;
    color:#000000;
}
.seances tr:last-child td {
    border-width:0px 1px 0px 0px;
}
.seances tr td:last-child {
    border-width:0px 0px 1px 0px;
}
.seances tr:last-child td:last-child {
    border-width:0px 0px 0px 0px;
}
.seances tr:first-child td {
        background:-o-linear-gradient(bottom, #00cc00 5%, #a3f7a3 100%);    background:-webkit-gradient(
linear, left top, left bottom, color-stop(0.05, #00cc00), color-stop(1, #a3f7a3) );
    background:-moz-linear-gradient( center top, #00cc00 5%, #a3f7a3 100% );
```

```
    filter:progid:DXImageTransform.Microsoft.gradient(startColorstr="#00cc00", endColorstr="#a3f7a3");
background: -o-linear-gradient(top,#00cc00,a3f7a3);

    background-color:#00cc00;

    border:0px solid #ffffff;

    text-align:center;

    border-width:0px 0px 1px 1px;

    font-size:14px;

    font-family:Arial, Helvetica, Sans-serif;

    font-weight:bold;

    color:#ffffff;

}

.seances tr:first-child:hover td {

    background:-o-linear-gradient(bottom, #00cc00 5%, #a3f7a3 100%);    background:-webkit-gradient(
linear, left top, left bottom, color-stop(0.05, #00cc00), color-stop(1, #a3f7a3) );

    background:-moz-linear-gradient( center top, #00cc00 5%, #a3f7a3 100% );

    filter:progid:DXImageTransform.Microsoft.gradient(startColorstr="#00cc00", endColorstr="#a3f7a3");
background: -o-linear-gradient(top,#00cc00,a3f7a3);

    background-color:#00cc00;

}

.seances tr:first-child td:first-child {

    border-width:0px 0px 1px 0px;

}

.seances tr:first-child td:last-child {

    border-width:0px 0px 1px 1px;

}

.intro {

    margin:0px;padding:0px;
```

```
    width:100%;
    border:1px solid #ffffff;

    -moz-border-radius-bottomleft:16px;
    -webkit-border-bottom-left-radius:16px;
    border-bottom-left-radius:16px;

    -moz-border-radius-bottomright:16px;
    -webkit-border-bottom-right-radius:16px;
    border-bottom-right-radius:16px;

    -moz-border-radius-topright:16px;
    -webkit-border-top-right-radius:16px;
    border-top-right-radius:16px;

    -moz-border-radius-topleft:16px;
    -webkit-border-top-left-radius:16px;
    border-top-left-radius:16px;
}.intro table{
    border-collapse: collapse;
        border-spacing: 0;
    width:100%;
    height:100%;
    margin:0px;padding:0px;
}.intro tr:last-child td:last-child {
```

```
    -moz-border-radius-bottomright:16px;
    -webkit-border-bottom-right-radius:16px;
    border-bottom-right-radius:16px;
}
.intro table tr:first-child td:first-child {
    -moz-border-radius-topleft:16px;
    -webkit-border-top-left-radius:16px;
    border-top-left-radius:16px;
}
.intro table tr:first-child td:last-child {
    -moz-border-radius-topright:16px;
    -webkit-border-top-right-radius:16px;
    border-top-right-radius:16px;
}.intro tr:last-child td:first-child{
    -moz-border-radius-bottomleft:16px;
    -webkit-border-bottom-left-radius:16px;
    border-bottom-left-radius:16px;
}.intro tr:hover td{
    background-color:#ffffff;


}
.intro td{
    vertical-align:middle;
        background:-o-linear-gradient(bottom, #f7f7f7 5%, #ffffff 100%);    background:-webkit-gradient(
linear, left top, left bottom, color-stop(0.05, #f7f7f7), color-stop(1, #ffffff) );
    background:-moz-linear-gradient( center top, #f7f7f7 5%, #ffffff 100% );
```

```
    filter:progid:DXImageTransform.Microsoft.gradient(startColorstr="#f7f7f7", endColorstr="#ffffff");
background: -o-linear-gradient(top,#f7f7f7,ffffff);

    background-color:#f7f7f7;

    border:1px solid #ffffff;

    border-width:0px 1px 1px 0px;

    text-align:left;

    padding:10px;

    font-size:10px;

    font-family:Arial, Helvetica, Sans-serif;

    font-weight:normal;

    color:#000000;

}.intro tr:last-child td{

    border-width:0px 1px 0px 0px;

}.intro tr td:last-child{

    border-width:0px 0px 1px 0px;

}.intro tr:last-child td:last-child{

    border-width:0px 0px 0px 0px;

}

.intro tr:first-child td{

        background:-o-linear-gradient(bottom, #4285f4 5%, #82b1ff 100%);    background:-webkit-gradient(
linear, left top, left bottom, color-stop(0.05, #4285f4), color-stop(1, #82b1ff) );

    background:-moz-linear-gradient( center top, #4285f4 5%, #82b1ff 100% );

    filter:progid:DXImageTransform.Microsoft.gradient(startColorstr="#4285f4", endColorstr="#82b1ff");
background: -o-linear-gradient(top,#4285f4,82b1ff);

    background-color:#4285f4;

    border:0px solid #ffffff;

    text-align:center;
```

```
    border-width:0px 0px 1px 1px;

    font-size:14px;

    font-family:Arial, Helvetica, Sans-serif;

    font-weight:bold;

    color:#ffffff;

}

.intro tr:first-child:hover td{

    background:-o-linear-gradient(bottom, #4285f4 5%, #82b1ff 100%);    background:-webkit-gradient( linear, left top, left bottom, color-stop(0.05, #4285f4), color-stop(1, #82b1ff) );

    background:-moz-linear-gradient( center top, #4285f4 5%, #82b1ff 100% );

    filter:progid:DXImageTransform.Microsoft.gradient(startColorstr="#4285f4", endColorstr="#82b1ff");   background: -o-linear-gradient(top,#4285f4,82b1ff);

    background-color:#4285f4;

}

.intro tr:first-child td:first-child{

    border-width:0px 0px 1px 0px;

}

.intro tr:first-child td:last-child{

    border-width:0px 0px 1px 1px;

}

.resume {

    margin:0px;padding:0px;

    width:100%;

    box-shadow: 10px 10px 5px #888888;

    border:1px solid #394bdb;
```

```
    -moz-border-radius-bottomleft:5px;
    -webkit-border-bottom-left-radius:5px;
    border-bottom-left-radius:5px;

    -moz-border-radius-bottomright:5px;
    -webkit-border-bottom-right-radius:5px;
    border-bottom-right-radius:5px;

    -moz-border-radius-topright:5px;
    -webkit-border-top-right-radius:5px;
    border-top-right-radius:5px;

    -moz-border-radius-topleft:5px;
    -webkit-border-top-left-radius:5px;
    border-top-left-radius:5px;
}.resume table{
    border-collapse: collapse;
        border-spacing: 0;
    width:100%;
    height:100%;
    margin:0px;padding:0px;
}.resume tr:last-child td:last-child {
    -moz-border-radius-bottomright:5px;
    -webkit-border-bottom-right-radius:5px;
    border-bottom-right-radius:5px;
```

```
}
.resume table tr:first-child td:first-child {
   -moz-border-radius-topleft:5px;
   -webkit-border-top-left-radius:5px;
   border-top-left-radius:5px;
}
.resume table tr:first-child td:last-child {
   -moz-border-radius-topright:5px;
   -webkit-border-top-right-radius:5px;
   border-top-right-radius:5px;
}.resume tr:last-child td:first-child{
   -moz-border-radius-bottomleft:5px;
   -webkit-border-bottom-left-radius:5px;
   border-bottom-left-radius:5px;
}.resume tr:hover td{
   background-color:#ffffff;


}
.resume td{
   vertical-align:middle;


   background-color:#ffffff;
   border:1px solid #394bdb;
   border-width:0px 1px 1px 0px;
   text-align:left;
```

```
    padding:5px;

    font-size:16px;

    font-family:Arial;

    font-weight:normal;

    color:#000000;

}.resume tr:last-child td{

    border-width:0px 1px 0px 0px;

}.resume tr td:last-child{

    border-width:0px 0px 1px 0px;

}.resume tr:last-child td:last-child{

    border-width:0px 0px 0px 0px;

}

.resume tr:first-child td{

        background:-o-linear-gradient(bottom, #4285f4 5%, #394bdb 100%);    background:-webkit-gradient(
linear, left top, left bottom, color-stop(0.05, #4285f4), color-stop(1, #394bdb) );

    background:-moz-linear-gradient( center top, #4285f4 5%, #394bdb 100% );

    filter:progid:DXImageTransform.Microsoft.gradient(startColorstr="#4285f4", endColorstr="#394bdb");
background: -o-linear-gradient(top,#4285f4,394bdb);

    background-color:#4285f4;

    border:0px solid #394bdb;

    text-align:center;

    border-width:0px 0px 1px 1px;

    font-size:18px;

    font-family:Arial;

    font-weight:bold;

    color:#ffffff;
```

```
}

.resume tr:first-child:hover td{

   background:-o-linear-gradient(bottom, #4285f4 5%, #394bdb 100%);    background:-webkit-gradient( linear, left top, left bottom, color-stop(0.05, #4285f4), color-stop(1, #394bdb) );

   background:-moz-linear-gradient( center top, #4285f4 5%, #394bdb 100% );

   filter:progid:DXImageTransform.Microsoft.gradient(startColorstr="#4285f4", endColorstr="#394bdb"); background: -o-linear-gradient(top,#4285f4,394bdb);

   background-color:#4285f4;

}

.resume tr:first-child td:first-child{

   border-width:0px 0px 1px 0px;

}

.resume tr:first-child td:last-child{

   border-width:0px 0px 1px 1px;

}

div.bigbig {

   color:#4285f4;

   font-weight:bold;

   font-size:36px;

   text-align:center;

   }

div.begin {

   text-align:left;

   }

table tbody tr td table tbody tr td table tbody tr td div a

{
```

```
-moz-box-shadow:inset 0px 1px 0px 0px #bbdaf7;

-webkit-box-shadow:inset 0px 1px 0px 0px #bbdaf7;

box-shadow:inset 0px 1px 0px 0px #bbdaf7;

background:-webkit-gradient( linear, left top, left bottom, color-stop(0.05, #79bbff), color-stop(1, #4286f4) );

background:-moz-linear-gradient( center top, #79bbff 5%, #4286f4 100% );

filter:progid:DXImageTransform.Microsoft.gradient(startColorstr='#79bbff', endColorstr='#4286f4');

background-color:#79bbff;

-webkit-border-top-left-radius:31px;

-moz-border-radius-topleft:31px;

border-top-left-radius:31px;

-webkit-border-top-right-radius:31px;

-moz-border-radius-topright:31px;

border-top-right-radius:31px;

-webkit-border-bottom-right-radius:31px;

-moz-border-radius-bottomright:31px;

border-bottom-right-radius:31px;

-webkit-border-bottom-left-radius:31px;

-moz-border-radius-bottomleft:31px;

border-bottom-left-radius:31px;

text-indent:0px;

border:1px solid #84bbf3;

display:inline-block;

color:#ffffff;

font-family:Arial;

font-size:24px;
```

```
    font-weight:bold;
    font-style:normal;
    height:88px;
    line-height:88px;
    width:300px;
    text-decoration:none;
    text-align:center;
    text-shadow:1px 1px 0px #528ecc;
}
table tbody tr td table tbody tr td table tbody tr td div a:hover {
    background:-webkit-gradient( linear, left top, left bottom, color-stop(0.05, #4286f4), color-stop(1,
#79bbff) );
    background:-moz-linear-gradient( center top, #4286f4 5%, #79bbff 100% );
    filter:progid:DXImageTransform.Microsoft.gradient(startColorstr='#4286f4', endColorstr='#79bbff');
    background-color:#4286f4;
}
table tbody tr td table tbody tr td table tbody tr td div a:active {
    position:relative;
    top:1px;
}
table tbody tr table tbody tr a {
    -moz-box-shadow:inset 0px 1px 0px 0px #cae3fc;
    -webkit-box-shadow:inset 0px 1px 0px 0px #cae3fc;
    box-shadow:inset 0px 1px 0px 0px #cae3fc;
    background:-webkit-gradient( linear, left top, left bottom, color-stop(0.05, #79bbff), color-stop(1,
#4197ee) );
```

```
background:-moz-linear-gradient( center top, #79bbff 5%, #4197ee 100% );

filter:progid:DXImageTransform.Microsoft.gradient(startColorstr='#79bbff', endColorstr='#4197ee');

background-color:#79bbff;

-webkit-border-top-left-radius:10px;

-moz-border-radius-topleft:10px;

border-top-left-radius:10px;

-webkit-border-top-right-radius:10px;

-moz-border-radius-topright:10px;

border-top-right-radius:10px;

-webkit-border-bottom-right-radius:10px;

-moz-border-radius-bottomright:10px;

border-bottom-right-radius:10px;

-webkit-border-bottom-left-radius:10px;

-moz-border-radius-bottomleft:10px;

border-bottom-left-radius:10px;

text-indent:0;

border:1px solid #469df5;

display:inline-block;

color:#ffffff;

font-family:Arial;

font-size:15px;

font-weight:bold;

font-style:normal;

height:40px;

line-height:40px;
```

```
    width:130px;

    text-decoration:none;

    text-align:center;

    text-shadow:1px 1px 0px #287ace;

    }

table tbody tr table tbody tr a:hover {

    background:-webkit-gradient( linear, left top, left bottom, color-stop(0.05, #4197ee), color-stop(1, 
#79bbff) );

    background:-moz-linear-gradient( center top, #4197ee 5%, #79bbff 100% );

    filter:progid:DXImageTransform.Microsoft.gradient(startColorstr='#4197ee', endColorstr='#79bbff');

    background-color:#4197ee;

}

table tbody tr table tbody tr a:active {

    position:relative;

    top:1px;

    }


/*classes pour sp_accueil*/


.reduce {

    font-size:16px;

    color:black;

    text-align:center;

    }


.green {
```

```
   font-size:22px;
   color:#00cc00;
   font-weight:bold;
   text-align:center;
   }
.grey {
   font-size:22px;
   color:#b3b3b3;
   font-weight:700;
   text-align:center;

   }
.blue {
   font-size:16px;
   color:#4285f4;
   font-weight:700;
   text-align:center;

   }

.bigblue {
   font-size:22px;
   color:#4285f4;
   font-weight:700;
   text-align:center;
```

```
   }
       /*class pour hr*/
.hrblue {
    color:#4285f4;
    background-color:#4285f4;
    height:10px;
    border:none;
    width: auto;

}
/*for home page*/
.welcome {
   margin:auto;
   border:10px outset EDEDF3;
   background-color:4285f4;
   text-align:center;
   padding:3 white;
   }


.white {
   color:white;
   font-weight:normal;
   font-size:20px;
```

```
    text-align:center;

}
.smallwhite {
    color:white;
    font-weight:normal;
    font-size:20px;

}
.black {
    color:black;
    font-weight:bold;
    font-size:20px;

}


table tbody tr strong a {
    -moz-box-shadow:inset 0px 1px 0px 0px #00cc00;
    -webkit-box-shadow:inset 0px 1px 0px 0px #00cc00;
    box-shadow:inset 0px 1px 0px 0px #00cc00;
    background:-webkit-gradient( linear, left top, left bottom, color-stop(0.05, #63f08d), color-stop(1,
#00cc00) );
    background:-moz-linear-gradient( center top, #63f08d 5%, #00cc00 100% );
    filter:progid:DXImageTransform.Microsoft.gradient(startColorstr='#63f08d', endColorstr='#00cc00');
    background-color:#63f08d;
```

```
-webkit-border-top-left-radius:10px;
-moz-border-radius-topleft:10px;
border-top-left-radius:10px;
-webkit-border-top-right-radius:10px;
-moz-border-radius-topright:10px;
border-top-right-radius:10px;
-webkit-border-bottom-right-radius:10px;
-moz-border-radius-bottomright:10px;
border-bottom-right-radius:10px;
-webkit-border-bottom-left-radius:10px;
-moz-border-radius-bottomleft:10px;
border-bottom-left-radius:10px;
text-indent:0;
border:1px solid #00cc00;
display:inline-block;
color:#ffffff;
font-family:Arial;
font-size:18px;
font-weight:bold;
font-style:normal;
height:29px;
line-height:29px;
width:150px;
text-decoration:none;
text-align:center;
```

```
    text-shadow:1px 1px 0px #00cc00;
}
table tbody tr strong a:hover {
    background:-webkit-gradient( linear, left top, left bottom, color-stop(0.05, #00cc00), color-stop(1,
#63f08d) );
    background:-moz-linear-gradient( center top, #00cc00 5%, #63f08d 100% );
    filter:progid:DXImageTransform.Microsoft.gradient(startColorstr='#00cc00', endColorstr='#63f08d');
    background-color:#00cc00;
}
table tbody tr strong a:active {
    position:relative;
    top:1px;
}
div.titleplan {
    color:#00cc00;
    font-weight:bold;
    font-size:28px;
    text-align:center;
    }


    .patype {
    margin:0px;padding:0px;
    width:100%;
    box-shadow: 10px 10px 5px #888888;
    border:1px solid #4285f4;
```

```
    -moz-border-radius-bottomleft:9px;
    -webkit-border-bottom-left-radius:9px;
    border-bottom-left-radius:9px;

    -moz-border-radius-bottomright:9px;
    -webkit-border-bottom-right-radius:9px;
    border-bottom-right-radius:9px;

    -moz-border-radius-topright:9px;
    -webkit-border-top-right-radius:9px;
    border-top-right-radius:9px;

    -moz-border-radius-topleft:9px;
    -webkit-border-top-left-radius:9px;
    border-top-left-radius:9px;
}
.patype table {
    border-collapse: collapse;
        border-spacing: 0;
    width:100%;
    height:100%;
    margin:0px;padding:0px;
}
.patype tr:last-child td:last-child {
```

```
   -moz-border-radius-bottomright:9px;
   -webkit-border-bottom-right-radius:9px;
   border-bottom-right-radius:9px;
}
.patype table tr:first-child td:first-child {
   -moz-border-radius-topleft:9px;
   -webkit-border-top-left-radius:9px;
   border-top-left-radius:9px;
}
.patype table tr:first-child td:last-child {
   -moz-border-radius-topright:9px;
   -webkit-border-top-right-radius:9px;
   border-top-right-radius:9px;
}
.patype tr:last-child td:first-child {
   -moz-border-radius-bottomleft:9px;
   -webkit-border-bottom-left-radius:9px;
   border-bottom-left-radius:9px;
}
.patype tr:hover td {
   background-color:#ffffff;

}
.patype td {
   vertical-align:middle;
```

```
        background:-o-linear-gradient(bottom, #e5f9e5 5%, #ffffff 100%);    background:-webkit-gradient( linear, left top, left bottom, color-stop(0.05, #e5f9e5), color-stop(1, #ffffff) );

    background:-moz-linear-gradient( center top, #e5f9e5 5%, #ffffff 100% );

    filter:progid:DXImageTransform.Microsoft.gradient(startColorstr="#e5f9e5", endColorstr="#ffffff"); background: -o-linear-gradient(top,#e5f9e5,ffffff);

    background-color:#e5f9e5;

    border:1px solid #4285f4;

    border-width:0px 1px 1px 0px;

    text-align:center;

    padding:11px;

    font-size:10px;

    font-family:Arial, Helvetica, Sans-serif;

    font-weight:normal;

    color:#000000;

}

.patype tr:last-child td {

    border-width:0px 1px 0px 0px;

}

.patype tr td:last-child {

    border-width:0px 0px 1px 0px;

}

.patype tr:last-child td:last-child {

    border-width:0px 0px 0px 0px;

}

.patype tr:first-child td {

        background:-o-linear-gradient(bottom, #4285f4 5%, #aeccfc 100%);    background:-webkit-gradient( linear, left top, left bottom, color-stop(0.05, #4285f4), color-stop(1, #aeccfc) );
```

```
    background:-moz-linear-gradient( center top, #4285f4 5%, #aeccfc 100% );
    filter:progid:DXImageTransform.Microsoft.gradient(startColorstr="#4285f4", endColorstr="#aeccfc");
background: -o-linear-gradient(top,#4285f4,aeccfc);
    background-color:#4285f4;
    border:0px solid #4285f4;
    text-align:center;
    border-width:0px 0px 1px 1px;
    font-size:14px;
    font-family:Arial, Helvetica, Sans-serif;
    font-weight:bold;
    color:#ffffff;
}
.patype tr:first-child:hover td {
    background:-o-linear-gradient(bottom, #4285f4 5%, #aeccfc 100%);    background:-webkit-gradient(
linear, left top, left bottom, color-stop(0.05, #4285f4), color-stop(1, #aeccfc) );
    background:-moz-linear-gradient( center top, #4285f4 5%, #aeccfc 100% );
    filter:progid:DXImageTransform.Microsoft.gradient(startColorstr="#4285f4", endColorstr="#aeccfc");
background: -o-linear-gradient(top,#4285f4,aeccfc);
    background-color:#4285f4;
}
.patype tr:first-child td:first-child {
    border-width:0px 0px 1px 0px;
}
.patype tr:first-child td:last-child {
    border-width:0px 0px 1px 1px;
}
a.final {
```

```
-moz-box-shadow:inset 0px 1px 0px 0px #bbdaf7;
-webkit-box-shadow:inset 0px 1px 0px 0px #bbdaf7;
box-shadow:inset 0px 1px 0px 0px #bbdaf7;
background:-webkit-gradient( linear, left top, left bottom, color-stop(0.05, #79bbff), color-stop(1,
#4286f4) );
background:-moz-linear-gradient( center top, #79bbff 5%, #4286f4 100% );
filter:progid:DXImageTransform.Microsoft.gradient(startColorstr='#79bbff', endColorstr='#4286f4');
background-color:#79bbff;
-webkit-border-top-left-radius:31px;
-moz-border-radius-topleft:31px;
border-top-left-radius:31px;
-webkit-border-top-right-radius:31px;
-moz-border-radius-topright:31px;
border-top-right-radius:31px;
-webkit-border-bottom-right-radius:31px;
-moz-border-radius-bottomright:31px;
border-bottom-right-radius:31px;
-webkit-border-bottom-left-radius:31px;
-moz-border-radius-bottomleft:31px;
border-bottom-left-radius:31px;
text-indent:0px;
border:1px solid #84bbf3;
display:inline-block;
color:#ffffff;
font-family:Arial;
font-size:24px;
```

```
    font-weight:bold;
    font-style:normal;
    height:88px;
    line-height:88px;
    width:300px;
    text-decoration:none;
    text-align:center;
    text-shadow:1px 1px 0px #528ecc;
}
a.final:hover {
    background:-webkit-gradient( linear, left top, left bottom, color-stop(0.05, #4286f4), color-stop(1,
#79bbff) );
    background:-moz-linear-gradient( center top, #4286f4 5%, #79bbff 100% );
    filter:progid:DXImageTransform.Microsoft.gradient(startColorstr='#4286f4', endColorstr='#79bbff');
    background-color:#4286f4;
}
a.final:active {
    position:relative;
    top:1px;
}
a.start {
    -moz-box-shadow:inset 0px 1px 0px 0px #cae3fc;
    -webkit-box-shadow:inset 0px 1px 0px 0px #cae3fc;
    box-shadow:inset 0px 1px 0px 0px #cae3fc;
    background:-webkit-gradient( linear, left top, left bottom, color-stop(0.05, #79bbff), color-stop(1,
#4197ee) );
```

```
background:-moz-linear-gradient( center top, #79bbff 5%, #4197ee 100% );
filter:progid:DXImageTransform.Microsoft.gradient(startColorstr='#79bbff', endColorstr='#4197ee');
background-color:#79bbff;
-webkit-border-top-left-radius:10px;
-moz-border-radius-topleft:10px;
border-top-left-radius:10px;
-webkit-border-top-right-radius:10px;
-moz-border-radius-topright:10px;
border-top-right-radius:10px;
-webkit-border-bottom-right-radius:10px;
-moz-border-radius-bottomright:10px;
border-bottom-right-radius:10px;
-webkit-border-bottom-left-radius:10px;
-moz-border-radius-bottomleft:10px;
border-bottom-left-radius:10px;
text-indent:0;
border:1px solid #469df5;
display:inline-block;
color:#ffffff;
font-family:Arial;
font-size:15px;
font-weight:bold;
font-style:normal;
height:40px;
line-height:40px;
```

```
   width:130px;
   text-decoration:none;
   text-align:center;
   text-shadow:1px 1px 0px #287ace;
   }
a.start:hover {
   background:-webkit-gradient( linear, left top, left bottom, color-stop(0.05, #4197ee), color-stop(1,
#79bbff) );
   background:-moz-linear-gradient( center top, #4197ee 5%, #79bbff 100% );
   filter:progid:DXImageTransform.Microsoft.gradient(startColorstr='#4197ee', endColorstr='#79bbff');
   background-color:#4197ee;
}
a.start:active {
   position:relative;
   top:1px;
   }



a.tutoriel {
   -moz-box-shadow:inset 0px 1px 0px 0px #cae3fc;
   -webkit-box-shadow:inset 0px 1px 0px 0px #cae3fc;
   box-shadow:inset 0px 1px 0px 0px #cae3fc;
   background:-webkit-gradient( linear, left top, left bottom, color-stop(0.05, #79bbff), color-stop(1,
#4197ee) );
   background:-moz-linear-gradient( center top, #79bbff 5%, #4197ee 100% );
```

```
filter:progid:DXImageTransform.Microsoft.gradient(startColorstr='#79bbff', endColorstr='#4197ee');

background-color:#79bbff;

-webkit-border-top-left-radius:10px;

-moz-border-radius-topleft:10px;

border-top-left-radius:10px;

-webkit-border-top-right-radius:10px;

-moz-border-radius-topright:10px;

border-top-right-radius:10px;

-webkit-border-bottom-right-radius:10px;

-moz-border-radius-bottomright:10px;

border-bottom-right-radius:10px;

-webkit-border-bottom-left-radius:10px;

-moz-border-radius-bottomleft:10px;

border-bottom-left-radius:10px;

text-indent:0;

border:1px solid #469df5;

display:inline-block;

color:#ffffff;

font-family:Arial;

font-size:15px;

font-weight:bold;

font-style:normal;

height:40px;

line-height:40px;

width:240px;
```

```
    text-decoration:none;

    text-align:center;

    text-shadow:1px 1px 0px #287ace;

    }

a.tutoriel:hover {

    background:-webkit-gradient( linear, left top, left bottom, color-stop(0.05, #4197ee), color-stop(1,
#79bbff) );

    background:-moz-linear-gradient( center top, #4197ee 5%, #79bbff 100% );

    filter:progid:DXImageTransform.Microsoft.gradient(startColorstr='#4197ee', endColorstr='#79bbff');

    background-color:#4197ee;

}

a.tutoriel:active {

    position:relative;

    top:1px;

    }
```

STYLESHEET - ACTION PLANS AND REGISTRATION SESSION

```
table.box {


    border-right: 5px solid #4285f4;

    border-left: 5px solid #4285f4;

    border-bottom: 5px solid #4285f4;

    border-top: 5px solid #4285f4;


}


body {


 font-family:Arial, Helvetica, Sans-serif;


}


.plan {

    -moz-box-shadow:inset 0px 1px 0px 0px #fafafa;

    -webkit-box-shadow:inset 0px 1px 0px 0px #fafafa;

    box-shadow:inset 0px 1px 0px 0px #fafafa;

    background:-webkit-gradient( linear, left top, left bottom, color-stop(0.05, #9fed9f), color-stop(1,
#00cc00) );

    background:-moz-linear-gradient( center top, #9fed9f 5%, #00cc00 100% );

    filter:progid:DXImageTransform.Microsoft.gradient(startColorstr='#9fed9f', endColorstr='#00cc00');

    background-color:#9fed9f;

    -webkit-border-top-left-radius:20px;
```

```
-moz-border-radius-topleft:20px;
border-top-left-radius:20px;
-webkit-border-top-right-radius:20px;
-moz-border-radius-topright:20px;
border-top-right-radius:20px;
-webkit-border-bottom-right-radius:20px;
-moz-border-radius-bottomright:20px;
border-bottom-right-radius:20px;
-webkit-border-bottom-left-radius:20px;
-moz-border-radius-bottomleft:20px;
border-bottom-left-radius:20px;
text-indent:0;
border:1px solid #4286f4;
display:inline-block;
color:#ffffff;
font-family:Arial, Helvetica, Sans-serif;
font-size:15px;
font-weight:bold;
font-style:normal;
height:75px;
line-height:75px;
width:200px;
text-decoration:none;
text-align:center;
text-shadow:1px 1px 0px #00cc00;
```

```
}
.plan:hover {
   background:-webkit-gradient( linear, left top, left bottom, color-stop(0.05, #00cc00), color-stop(1,
#9fed9f) );
   background:-moz-linear-gradient( center top, #00cc00 5%, #9fed9f 100% );
   filter:progid:DXImageTransform.Microsoft.gradient(startColorstr='#00cc00', endColorstr='#9fed9f');
   background-color:#00cc00;
}
.plan:active {
   position:relative;
   top:1px;
}

.patype {
   margin:0px;padding:0px;
   width:100%;
   box-shadow: 10px 10px 5px #888888;
   border:1px solid #4285f4;

   -moz-border-radius-bottomleft:9px;
   -webkit-border-bottom-left-radius:9px;
   border-bottom-left-radius:9px;

   -moz-border-radius-bottomright:9px;
   -webkit-border-bottom-right-radius:9px;
   border-bottom-right-radius:9px;
```

```
    -moz-border-radius-topright:9px;
    -webkit-border-top-right-radius:9px;
    border-top-right-radius:9px;

    -moz-border-radius-topleft:9px;
    -webkit-border-top-left-radius:9px;
    border-top-left-radius:9px;
}

.patype table {
    border-collapse: collapse;
        border-spacing: 0;
    width:100%;
    height:100%;
    margin:0px;padding:0px;
}

.patype tr:last-child td:last-child {
    -moz-border-radius-bottomright:9px;
    -webkit-border-bottom-right-radius:9px;
    border-bottom-right-radius:9px;
}

.patype table tr:first-child td:first-child {
```

```
    -moz-border-radius-topleft:9px;
    -webkit-border-top-left-radius:9px;
    border-top-left-radius:9px;
}

.patype table tr:first-child td:last-child {
    -moz-border-radius-topright:9px;
    -webkit-border-top-right-radius:9px;
    border-top-right-radius:9px;
}

.patype tr:last-child td:first-child {
    -moz-border-radius-bottomleft:9px;
    -webkit-border-bottom-left-radius:9px;
    border-bottom-left-radius:9px;
}

.patype tr:hover td {
    background-color:#ffffff;


}

.patype td {
    vertical-align:middle;
```

```
      background:-o-linear-gradient(bottom, #e5f9e5 5%, #ffffff 100%);    background:-webkit-gradient( linear, left top, left bottom, color-stop(0.05, #e5f9e5), color-stop(1, #ffffff) );
    background:-moz-linear-gradient( center top, #e5f9e5 5%, #ffffff 100% );
    filter:progid:DXImageTransform.Microsoft.gradient(startColorstr="#e5f9e5", endColorstr="#ffffff"); background: -o-linear-gradient(top,#e5f9e5,ffffff);

    background-color:#e5f9e5;

    border:1px solid #4285f4;
    border-width:0px 1px 1px 0px;
    text-align:center;
    padding:11px;
    font-size:10px;
    font-family:Arial, Helvetica, Sans-serif;
    font-weight:normal;
    color:#000000;
}

.patype tr:last-child td {
    border-width:0px 1px 0px 0px;
}

.patype tr td:last-child {
    border-width:0px 0px 1px 0px;
}
```

```
.patype tr:last-child td:last-child {
   border-width:0px 0px 0px 0px;
}

.patype tr:first-child td {
      background:-o-linear-gradient(bottom, #4285f4 5%, #aeccfc 100%);   background:-webkit-gradient(
linear, left top, left bottom, color-stop(0.05, #4285f4), color-stop(1, #aeccfc) );
   background:-moz-linear-gradient( center top, #4285f4 5%, #aeccfc 100% );
   filter:progid:DXImageTransform.Microsoft.gradient(startColorstr="#4285f4", endColorstr="#aeccfc");
background: -o-linear-gradient(top,#4285f4,aeccfc);

   background-color:#4285f4;
   border:0px solid #4285f4;
   text-align:center;
   border-width:0px 0px 1px 1px;
   font-size:14px;
   font-family:Arial, Helvetica, Sans-serif;
   font-weight:bold;
   color:#ffffff;
}
.patype tr:first-child:hover td {
   background:-o-linear-gradient(bottom, #4285f4 5%, #aeccfc 100%);   background:-webkit-gradient(
linear, left top, left bottom, color-stop(0.05, #4285f4), color-stop(1, #aeccfc) );
   background:-moz-linear-gradient( center top, #4285f4 5%, #aeccfc 100% );
   filter:progid:DXImageTransform.Microsoft.gradient(startColorstr="#4285f4", endColorstr="#aeccfc");
background: -o-linear-gradient(top,#4285f4,aeccfc);
```

```
    background-color:#4285f4;
}

.patype tr:first-child td:first-child {
    border-width:0px 0px 1px 0px;
}

.patype tr:first-child td:last-child {
    border-width:0px 0px 1px 1px;
}

.paben {
    margin:0px;padding:0px;
    width:100%;
    box-shadow: 10px 10px 5px #888888;
    border:1px solid #4285f4;

    -moz-border-radius-bottomleft:9px;
    -webkit-border-bottom-left-radius:9px;
    border-bottom-left-radius:9px;

    -moz-border-radius-bottomright:9px;
    -webkit-border-bottom-right-radius:9px;
    border-bottom-right-radius:9px;
```

```
    -moz-border-radius-topright:9px;
    -webkit-border-top-right-radius:9px;
    border-top-right-radius:9px;

    -moz-border-radius-topleft:9px;
    -webkit-border-top-left-radius:9px;
    border-top-left-radius:9px;
}.paben table{
    border-collapse: collapse;
        border-spacing: 0;
    width:100%;
    height:100%;
    margin:0px;padding:0px;
}

.paben tr:last-child td:last-child {
    -moz-border-radius-bottomright:9px;
    -webkit-border-bottom-right-radius:9px;
    border-bottom-right-radius:9px;
}

.paben table tr:first-child td:first-child {
    -moz-border-radius-topleft:9px;
    -webkit-border-top-left-radius:9px;
    border-top-left-radius:9px;
```

```
}

.paben table tr:first-child td:last-child {
   -moz-border-radius-topright:9px;
   -webkit-border-top-right-radius:9px;
   border-top-right-radius:9px;
}

.paben tr:last-child td:first-child {
   -moz-border-radius-bottomleft:9px;
   -webkit-border-bottom-left-radius:9px;
   border-bottom-left-radius:9px;
}

.paben tr:hover td {
   background-color:#ffffff;


}

.paben td {
   vertical-align:middle;
      background:-o-linear-gradient(bottom, #e5f9e5 5%, #ffffff 100%);    background:-webkit-gradient(
linear, left top, left bottom, color-stop(0.05, #e5f9e5), color-stop(1, #ffffff) );
   background:-moz-linear-gradient( center top, #e5f9e5 5%, #ffffff 100% );
```

```
    filter:progid:DXImageTransform.Microsoft.gradient(startColorstr="#e5f9e5", endColorstr="#ffffff");
background: -o-linear-gradient(top,#e5f9e5,ffffff);

    background-color:#e5f9e5;

    border:1px solid #4285f4;
    border-width:0px 1px 1px 0px;
    text-align:left;
    padding:11px;
    font-size:10px;
    font-family:Arial, Helvetica, Sans-serif;
    font-weight:normal;
    color:#000000;
}

.paben tr:last-child td {
    border-width:0px 1px 0px 0px;
}

.paben tr td:last-child {
    border-width:0px 0px 1px 0px;
}

.paben tr:last-child td:last-child {
    border-width:0px 0px 0px 0px;
}
```

```
.paben tr:first-child td {

       background:-o-linear-gradient(bottom, #4285f4 5%, #aeccfc 100%);    background:-webkit-gradient(
linear, left top, left bottom, color-stop(0.05, #4285f4), color-stop(1, #aeccfc) );

    background:-moz-linear-gradient( center top, #4285f4 5%, #aeccfc 100% );

    filter:progid:DXImageTransform.Microsoft.gradient(startColorstr="#4285f4", endColorstr="#aeccfc");
background: -o-linear-gradient(top,#4285f4,aeccfc);


    background-color:#4285f4;

    border:0px solid #4285f4;

    text-align:center;

    border-width:0px 0px 1px 1px;

    font-size:14px;

    font-family:Arial, Helvetica, Sans-serif;

    font-weight:bold;

    color:#ffffff;

}


.paben tr:first-child:hover td {

    background:-o-linear-gradient(bottom, #4285f4 5%, #aeccfc 100%);    background:-webkit-gradient(
linear, left top, left bottom, color-stop(0.05, #4285f4), color-stop(1, #aeccfc) );

    background:-moz-linear-gradient( center top, #4285f4 5%, #aeccfc 100% );

    filter:progid:DXImageTransform.Microsoft.gradient(startColorstr="#4285f4", endColorstr="#aeccfc");
background: -o-linear-gradient(top,#4285f4,aeccfc);


    background-color:#4285f4;

}
```

```
.paben tr:first-child td:first-child {
    border-width:0px 0px 1px 0px;
}

.paben tr:first-child td:last-child {
    border-width:0px 0px 1px 1px;
}

#grad {
  background: -webkit-linear-gradient(4285f4, aeccfc); /* For Safari 5.1 to 6.0 */
  background: -o-linear-gradient(4285f4, aeccfc); /* For Opera 11.1 to 12.0 */
  background: -moz-linear-gradient(4285f4, aeccfc); /* For Firefox 3.6 to 15 */
  background: linear-gradient(4285f4, aeccfc); /* Standard syntax */
}

.seances {
    margin:0px;padding:0px;
    width:100%;
    box-shadow: 10px 10px 5px #888888;
    border:1px solid #ffffff;

    -moz-border-radius-bottomleft:10px;
    -webkit-border-bottom-left-radius:10px;
```

```
    border-bottom-left-radius:10px;

    -moz-border-radius-bottomright:10px;
    -webkit-border-bottom-right-radius:10px;
    border-bottom-right-radius:10px;

    -moz-border-radius-topright:10px;
    -webkit-border-top-right-radius:10px;
    border-top-right-radius:10px;

    -moz-border-radius-topleft:10px;
    -webkit-border-top-left-radius:10px;
    border-top-left-radius:10px;
}

.seances table {
    border-collapse: collapse;
        border-spacing: 0;
    width:100%;
    height:100%;
    margin:0px;padding:0px;
}

.seances tr:last-child td:last-child {
    -moz-border-radius-bottomright:10px;
```

```
    -webkit-border-bottom-right-radius:10px;
    border-bottom-right-radius:10px;
}

.seances table tr:first-child td:first-child {
    -moz-border-radius-topleft:10px;
    -webkit-border-top-left-radius:10px;
    border-top-left-radius:10px;
}

.seances table tr:first-child td:last-child {
    -moz-border-radius-topright:10px;
    -webkit-border-top-right-radius:10px;
    border-top-right-radius:10px;
}

.seances tr:last-child td:first-child {
    -moz-border-radius-bottomleft:10px;
    -webkit-border-bottom-left-radius:10px;
    border-bottom-left-radius:10px;
}

.seances tr:hover td {
    background-color:#ffffff;
```

```
}

.seances td {
   vertical-align:middle;
      background:-o-linear-gradient(bottom, #6e9fef 5%, #ffffff 100%);    background:-webkit-gradient( linear, left top, left bottom, color-stop(0.05, #6e9fef), color-stop(1, #ffffff) );
   background:-moz-linear-gradient( center top, #6e9fef 5%, #ffffff 100% );
   filter:progid:DXImageTransform.Microsoft.gradient(startColorstr="#6e9fef", endColorstr="#ffffff"); background: -o-linear-gradient(top,#6e9fef,ffffff);

   background-color:#6e9fef;

   border:1px solid #ffffff;
   border-width:0px 1px 1px 0px;
   text-align:center;
   padding:7px;
   font-size:10px;
   font-family:Arial;
   font-weight:normal;
   color:#000000;
}

.seances tr:last-child td {
   border-width:0px 1px 0px 0px;
```

```
}

.seances tr td:last-child {
   border-width:0px 0px 1px 0px;
}

.seances tr:last-child td:last-child {
   border-width:0px 0px 0px 0px;
}

.seances tr:first-child td {
      background:-o-linear-gradient(bottom, #00cc00 5%, #a3f7a3 100%);    background:-webkit-gradient(
linear, left top, left bottom, color-stop(0.05, #00cc00), color-stop(1, #a3f7a3) );
   background:-moz-linear-gradient( center top, #00cc00 5%, #a3f7a3 100% );
   filter:progid:DXImageTransform.Microsoft.gradient(startColorstr="#00cc00", endColorstr="#a3f7a3");
background: -o-linear-gradient(top,#00cc00,a3f7a3);

   background-color:#00cc00;
   border:0px solid #ffffff;
   text-align:center;
   border-width:0px 0px 1px 1px;
   font-size:14px;
   font-family:Arial;
   font-weight:bold;
   color:#ffffff;
}
```

```
.seances tr:first-child:hover td {
    background:-o-linear-gradient(bottom, #00cc00 5%, #a3f7a3 100%);    background:-webkit-gradient(
linear, left top, left bottom, color-stop(0.05, #00cc00), color-stop(1, #a3f7a3) );
    background:-moz-linear-gradient( center top, #00cc00 5%, #a3f7a3 100% );
    filter:progid:DXImageTransform.Microsoft.gradient(startColorstr="#00cc00", endColorstr="#a3f7a3");
background: -o-linear-gradient(top,#00cc00,a3f7a3);

    background-color:#00cc00;
}

.seances tr:first-child td:first-child {
    border-width:0px 0px 1px 0px;
}

.seances tr:first-child td:last-child {
    border-width:0px 0px 1px 1px;
}

table tbody tr td table tbody tr td table tbody tr td div a {
    -moz-box-shadow:inset 0px 1px 0px 0px #bbdaf7;
    -webkit-box-shadow:inset 0px 1px 0px 0px #bbdaf7;
    box-shadow:inset 0px 1px 0px 0px #bbdaf7;
    background:-webkit-gradient( linear, left top, left bottom, color-stop(0.05, #79bbff), color-stop(1,
#4286f4) );
    background:-moz-linear-gradient( center top, #79bbff 5%, #4286f4 100% );
    filter:progid:DXImageTransform.Microsoft.gradient(startColorstr='#79bbff', endColorstr='#4286f4');
    background-color:#79bbff;
```

```
-webkit-border-top-left-radius:31px;
-moz-border-radius-topleft:31px;
border-top-left-radius:31px;
-webkit-border-top-right-radius:31px;
-moz-border-radius-topright:31px;
border-top-right-radius:31px;
-webkit-border-bottom-right-radius:31px;
-moz-border-radius-bottomright:31px;
border-bottom-right-radius:31px;
-webkit-border-bottom-left-radius:31px;
-moz-border-radius-bottomleft:31px;
border-bottom-left-radius:31px;
text-indent:0px;
border:1px solid #84bbf3;
display:inline-block;
color:#ffffff;
font-family:Arial;
font-size:24px;
font-weight:bold;
font-style:normal;
height:88px;
line-height:88px;
width:300px;
text-decoration:none;
text-align:center;
```

```
    text-shadow:1px 1px 0px #528ecc;
}
table tbody tr td table tbody tr td table tbody tr td div a:hover {
    background:-webkit-gradient( linear, left top, left bottom, color-stop(0.05, #4286f4), color-stop(1,
#79bbff) );
    background:-moz-linear-gradient( center top, #4286f4 5%, #79bbff 100% );
    filter:progid:DXImageTransform.Microsoft.gradient(startColorstr='#4286f4', endColorstr='#79bbff');
    background-color:#4286f4;
}
table tbody tr td table tbody tr td table tbody tr td div a:active {
    position:relative;
    top:1px;
}

a.final {
    -moz-box-shadow:inset 0px 1px 0px 0px #bbdaf7;
    -webkit-box-shadow:inset 0px 1px 0px 0px #bbdaf7;
    box-shadow:inset 0px 1px 0px 0px #bbdaf7;
    background:-webkit-gradient( linear, left top, left bottom, color-stop(0.05, #79bbff), color-stop(1,
#4286f4) );
    background:-moz-linear-gradient( center top, #79bbff 5%, #4286f4 100% );
    filter:progid:DXImageTransform.Microsoft.gradient(startColorstr='#79bbff', endColorstr='#4286f4');
    background-color:#79bbff;
    -webkit-border-top-left-radius:31px;
    -moz-border-radius-topleft:31px;
    border-top-left-radius:31px;
```

```
    -webkit-border-top-right-radius:31px;
    -moz-border-radius-topright:31px;
    border-top-right-radius:31px;
    -webkit-border-bottom-right-radius:31px;
    -moz-border-radius-bottomright:31px;
    border-bottom-right-radius:31px;
    -webkit-border-bottom-left-radius:31px;
    -moz-border-radius-bottomleft:31px;
    border-bottom-left-radius:31px;
    text-indent:0px;
    border:1px solid #84bbf3;
    display:inline-block;
    color:#ffffff;
    font-family:Arial;
    font-size:24px;
    font-weight:bold;
    font-style:normal;
    height:88px;
    line-height:88px;
    width:300px;
    text-decoration:none;
    text-align:center;
    text-shadow:1px 1px 0px #528ecc;
}
```

```
a.final:hover {
    background:-webkit-gradient( linear, left top, left bottom, color-stop(0.05, #4286f4), color-stop(1, #79bbff) );
    background:-moz-linear-gradient( center top, #4286f4 5%, #79bbff 100% );
    filter:progid:DXImageTransform.Microsoft.gradient(startColorstr='#4286f4', endColorstr='#79bbff');
    background-color:#4286f4;
}

a.final:active {
    position:relative;
    top:1px;
}



a.start {
    -moz-box-shadow:inset 0px 1px 0px 0px #cae3fc;
    -webkit-box-shadow:inset 0px 1px 0px 0px #cae3fc;
    box-shadow:inset 0px 1px 0px 0px #cae3fc;
    background:-webkit-gradient( linear, left top, left bottom, color-stop(0.05, #79bbff), color-stop(1, #4197ee) );
    background:-moz-linear-gradient( center top, #79bbff 5%, #4197ee 100% );
    filter:progid:DXImageTransform.Microsoft.gradient(startColorstr='#79bbff', endColorstr='#4197ee');
    background-color:#79bbff;
    -webkit-border-top-left-radius:10px;
    -moz-border-radius-topleft:10px;
```

```
border-top-left-radius:10px;
-webkit-border-top-right-radius:10px;
-moz-border-radius-topright:10px;
border-top-right-radius:10px;
-webkit-border-bottom-right-radius:10px;
-moz-border-radius-bottomright:10px;
border-bottom-right-radius:10px;
-webkit-border-bottom-left-radius:10px;
-moz-border-radius-bottomleft:10px;
border-bottom-left-radius:10px;
text-indent:0;
border:1px solid #469df5;
display:inline-block;
color:#ffffff;
font-family:Arial;
font-size:15px;
font-weight:bold;
font-style:normal;
height:40px;
line-height:40px;
width:130px;
text-decoration:none;
text-align:center;
text-shadow:1px 1px 0px #287ace;
}
```

```
a.start:hover {
   background:-webkit-gradient( linear, left top, left bottom, color-stop(0.05, #4197ee), color-stop(1,
#79bbff) );
   background:-moz-linear-gradient( center top, #4197ee 5%, #79bbff 100% );
   filter:progid:DXImageTransform.Microsoft.gradient(startColorstr='#4197ee', endColorstr='#79bbff');
   background-color:#4197ee;
}
a.start:active {
   position:relative;
   top:1px;
   }


.resume {
   margin:0px;padding:0px;
   width:100%;
   box-shadow: 10px 10px 5px #888888;
   border:1px solid #394bdb;

   -moz-border-radius-bottomleft:5px;
   -webkit-border-bottom-left-radius:5px;
   border-bottom-left-radius:5px;

   -moz-border-radius-bottomright:5px;
   -webkit-border-bottom-right-radius:5px;
   border-bottom-right-radius:5px;
```

```
    -moz-border-radius-topright:5px;
    -webkit-border-top-right-radius:5px;
    border-top-right-radius:5px;

    -moz-border-radius-topleft:5px;
    -webkit-border-top-left-radius:5px;
    border-top-left-radius:5px;
}.resume table{
    border-collapse: collapse;
        border-spacing: 0;
    width:100%;
    height:100%;
    margin:0px;padding:0px;
}.resume tr:last-child td:last-child {
    -moz-border-radius-bottomright:5px;
    -webkit-border-bottom-right-radius:5px;
    border-bottom-right-radius:5px;
}
.resume table tr:first-child td:first-child {
    -moz-border-radius-topleft:5px;
    -webkit-border-top-left-radius:5px;
    border-top-left-radius:5px;
}
.resume table tr:first-child td:last-child {
```

```
    -moz-border-radius-topright:5px;
    -webkit-border-top-right-radius:5px;
    border-top-right-radius:5px;
}.resume tr:last-child td:first-child{
    -moz-border-radius-bottomleft:5px;
    -webkit-border-bottom-left-radius:5px;
    border-bottom-left-radius:5px;
}.resume tr:hover td{
    background-color:#ffffff;


}
.resume td{
    vertical-align:middle;

    background-color:#ffffff;

    border:1px solid #394bdb;
    border-width:0px 1px 1px 0px;
    text-align:left;
    padding:5px;
    font-size:16px;
    font-family:Arial;
    font-weight:normal;
    color:#000000;
```

```
}.resume tr:last-child td{
   border-width:0px 1px 0px 0px;
}.resume tr td:last-child{
   border-width:0px 0px 1px 0px;
}.resume tr:last-child td:last-child{
   border-width:0px 0px 0px 0px;
}
.resume tr:first-child td{
      background:-o-linear-gradient(bottom, #4285f4 5%, #394bdb 100%);    background:-webkit-gradient( linear, left top, left bottom, color-stop(0.05, #4285f4), color-stop(1, #394bdb) );
   background:-moz-linear-gradient( center top, #4285f4 5%, #394bdb 100% );
   filter:progid:DXImageTransform.Microsoft.gradient(startColorstr="#4285f4", endColorstr="#394bdb");  background: -o-linear-gradient(top,#4285f4,394bdb);


   background-color:#4285f4;
   border:0px solid #394bdb;
   text-align:center;
   border-width:0px 0px 1px 1px;
   font-size:18px;
   font-family:Arial;
   font-weight:bold;
   color:#ffffff;
}
.resume tr:first-child:hover td{
   background:-o-linear-gradient(bottom, #4285f4 5%, #394bdb 100%);    background:-webkit-gradient( linear, left top, left bottom, color-stop(0.05, #4285f4), color-stop(1, #394bdb) );
   background:-moz-linear-gradient( center top, #4285f4 5%, #394bdb 100% );
```

```
    filter:progid:DXImageTransform.Microsoft.gradient(startColorstr="#4285f4", endColorstr="#394bdb");
background: -o-linear-gradient(top,#4285f4,394bdb);


    background-color:#4285f4;

}

.resume tr:first-child td:first-child{

    border-width:0px 0px 1px 0px;

}

.resume tr:first-child td:last-child{

    border-width:0px 0px 1px 1px;

}



.actionplan {

    margin:0px;padding:0px;

    width:100%;

    border:1px solid #4285f4;


    -moz-border-radius-bottomleft:9px;

    -webkit-border-bottom-left-radius:9px;

    border-bottom-left-radius:9px;


    -moz-border-radius-bottomright:9px;

    -webkit-border-bottom-right-radius:9px;

    border-bottom-right-radius:9px;
```

```
    -moz-border-radius-topright:9px;
    -webkit-border-top-right-radius:9px;
    border-top-right-radius:9px;

    -moz-border-radius-topleft:9px;
    -webkit-border-top-left-radius:9px;
    border-top-left-radius:9px;
}.actionplan table{
    border-collapse: collapse;
        border-spacing: 0;
    width:100%;
    height:100%;
    margin:0px;padding:0px;
}

.actionplan tr:last-child td:last-child {
    -moz-border-radius-bottomright:9px;
    -webkit-border-bottom-right-radius:9px;
    border-bottom-right-radius:9px;
}

.actionplan table tr:first-child td:first-child {
    -moz-border-radius-topleft:9px;
    -webkit-border-top-left-radius:9px;
    border-top-left-radius:9px;
```

```
}

.actionplan table tr:first-child td:last-child {
    -moz-border-radius-topright:9px;
    -webkit-border-top-right-radius:9px;
    border-top-right-radius:9px;
}

.actionplan tr:last-child td:first-child {
    -moz-border-radius-bottomleft:9px;
    -webkit-border-bottom-left-radius:9px;
    border-bottom-left-radius:9px;
}

.actionplan tr:hover td {
    background-color:#ffffff;



}

.actionplan td {
    vertical-align:middle;
        background:-o-linear-gradient(bottom, #e5f9e5 5%, #ffffff 100%);    background:-webkit-gradient(
linear, left top, left bottom, color-stop(0.05, #e5f9e5), color-stop(1, #ffffff) );
    background:-moz-linear-gradient( center top, #e5f9e5 5%, #ffffff 100% );
```

```
    filter:progid:DXImageTransform.Microsoft.gradient(startColorstr="#e5f9e5", endColorstr="#ffffff");
background: -o-linear-gradient(top,#e5f9e5,ffffff);

    background-color:#e5f9e5;

    border:1px solid #4285f4;
    border-width:0px 1px 1px 0px;
    text-align:left;
    padding:11px;
    font-size:10px;
    font-family:Arial, Helvetica, Sans-serif;
    font-weight:normal;
    color:#000000;
}

.actionplan tr:last-child td {
    border-width:0px 1px 0px 0px;
}

.actionplan tr td:last-child {
    border-width:0px 0px 1px 0px;
}

.actionplan tr:last-child td:last-child {
    border-width:0px 0px 0px 0px;
}
```

```
.actionplan tr:first-child td {
      background:-o-linear-gradient(bottom, #4285f4 5%, #aeccfc 100%);    background:-webkit-gradient( linear, left top, left bottom, color-stop(0.05, #4285f4), color-stop(1, #aeccfc) );
   background:-moz-linear-gradient( center top, #4285f4 5%, #aeccfc 100% );
   filter:progid:DXImageTransform.Microsoft.gradient(startColorstr="#4285f4", endColorstr="#aeccfc"); background: -o-linear-gradient(top,#4285f4,aeccfc);

   background-color:#4285f4;
   border:0px solid #4285f4;
   text-align:center;
   border-width:0px 0px 1px 1px;
   font-size:14px;
   font-family:Arial, Helvetica, Sans-serif;
   font-weight:bold;
   color:#ffffff;
}

.actionplan tr:first-child:hover td {
   background:-o-linear-gradient(bottom, #4285f4 5%, #aeccfc 100%);    background:-webkit-gradient( linear, left top, left bottom, color-stop(0.05, #4285f4), color-stop(1, #aeccfc) );
   background:-moz-linear-gradient( center top, #4285f4 5%, #aeccfc 100% );
   filter:progid:DXImageTransform.Microsoft.gradient(startColorstr="#4285f4", endColorstr="#aeccfc"); background: -o-linear-gradient(top,#4285f4,aeccfc);

   background-color:#4285f4;
}
```

```
.actionplan tr:first-child td:first-child {
   border-width:0px 0px 1px 0px;
}

.actionplan tr:first-child td:last-child {
   border-width:0px 0px 1px 1px;
}
```

STYLESHEET - MOTIVATIONAL SESSIONS MENU

```
div {
    color:#4285f4;
    font-weight:bold;
    font-size:28px;
    text-align:center;
    }

table tbody tr td table tbody tr td table tbody tr td div a {
    text-decoration: none;
    color: white;
    margin-top:40%;
    font-family: Arial, helvetica, sans-serif;
}
table tbody tr td table tbody tr td table tbody tr td div a:hover {
    color:#ffee00;
    }

a {
    -moz-box-shadow:inset 0px 1px 0px 0px #ffffff;
    -webkit-box-shadow:inset 0px 1px 0px 0px #ffffff;
    box-shadow:inset 0px 1px 0px 0px #ffffff;
    background:-webkit-gradient( linear, left top, left bottom, color-stop(0.05, #63f08d), color-stop(1, #00cc00) );
    background:-moz-linear-gradient( center top, #63f08d 5%, #00cc00 100% );
    filter:progid:DXImageTransform.Microsoft.gradient(startColorstr='#63f08d', endColorstr='#00cc00');
```

```
background-color:#63f08d;
-webkit-border-top-left-radius:10px;
-moz-border-radius-topleft:10px;
border-top-left-radius:10px;
-webkit-border-top-right-radius:10px;
-moz-border-radius-topright:10px;
border-top-right-radius:10px;
-webkit-border-bottom-right-radius:10px;
-moz-border-radius-bottomright:10px;
border-bottom-right-radius:10px;
-webkit-border-bottom-left-radius:10px;
-moz-border-radius-bottomleft:10px;
border-bottom-left-radius:10px;
text-indent:0;
border:1px solid #00cc00;
display:inline-block;
color:#ffffff;
font-family:Arial;
font-size:16px;
font-weight:bold;
font-style:normal;
height:35px;
line-height:35px;
width:136px;
text-decoration:none;
```

```
    text-align:center;
    text-shadow:1px 1px 0px #00cc00;
}
a:hover {
    background:-webkit-gradient( linear, left top, left bottom, color-stop(0.05, #00cc00), color-stop(1,
#63f08d) );
    background:-moz-linear-gradient( center top, #00cc00 5%, #63f08d 100% );
    filter:progid:DXImageTransform.Microsoft.gradient(startColorstr='#00cc00', endColorstr='#63f08d');
    background-color:#00cc00;
}
a:active {
    position:relative;
    top:1px;
}

/*classes pour sp_accueil*/

.reduce {
    font-size:18px;
    color:black;
    text-align:center;
    }

.green {
    font-size:14px;
    color:#00cc00;
```

```
    font-weight:bold;
    text-align:center;
    }
.grey {
    font-size:18px;
    color:#b3b3b3;
    font-weight:700;
    text-align:center;

    }
.blue {
    font-size:16px;
    color:#4285f4;
    font-weight:700;
    text-align:center;

    }

.bigblue {
    font-size:18px;
    color:#4285f4;
    font-weight:700;
    text-align:center;

    }
```

```
    /*class pour hr*/
.hrblue {
   color:#4285f4;
   background-color:#4285f4;
   height:10px;
   border:none;
   width: auto;


}
/*for home page*/
.welcome {
  margin:auto;
  border:10px outset EDEDF3;
  background-color:4285f4;
  text-align:center;
  padding:3 white;
  }



.white {
  color:white;
  font-weight:bold;
  font-size:24px;
  text-align:center;
```

```
}
.smallwhite {
    color:white;
    font-weight:bold;
    font-size:20px;


}
.black {
    color:black;
    font-weight:bold;
    font-size:24px;
}


table tbody tr strong a {
    color:yellow;
    font-weight:bold;
    text-decoration: none;
    font-size: 22px;
    }


table tbody tr strong a:hover {
    color:yellow;
    font-weight:bold;
    text-decoration:underline;
    font-size:22px;
```

```
    }

img.trans {
    opacity: 0.3;
    filter: alpha(opacity=40); /* For IE8 and earlier */
}

.transblue {
    font-size:16px;
    color:#4285f4;
    font-weight:700;
    text-align:center;
    opacity: 0.5;
    filter: alpha(opacity=40); /* For IE8 and earlier */

    }

    .transblack {
    font-size:16px;
    color:black;
    font-weight:700;
    text-align:center;
    opacity: 0.5;
    filter: alpha(opacity=40); /* For IE8 and earlier */}
```

STYLESHEET - MAIN MENU

```
body {
 font-family:Arial, Helvetica, Sans-serif;
 }
div.nothing {
    color:black;
    font-weight:bold;
    font-size:22px;
    }
div {
    color:#4285f4;
    font-weight:bold;
    font-size:36px;
    text-align:center;
    }
div.begin {
    text-align:left;
    }
table tbody tr td table tbody tr td table tbody tr td div a {
    -moz-box-shadow:inset 0px 1px 0px 0px #bbdaf7;
    -webkit-box-shadow:inset 0px 1px 0px 0px #bbdaf7;
    box-shadow:inset 0px 1px 0px 0px #bbdaf7;
    background:-webkit-gradient( linear, left top, left bottom, color-stop(0.05, #79bbff), color-stop(1,
#4286f4) );
    background:-moz-linear-gradient( center top, #79bbff 5%, #4286f4 100% );
    filter:progid:DXImageTransform.Microsoft.gradient(startColorstr='#79bbff', endColorstr='#4286f4');
```

```
background-color:#79bbff;
-webkit-border-top-left-radius:31px;
-moz-border-radius-topleft:31px;
border-top-left-radius:31px;
-webkit-border-top-right-radius:31px;
-moz-border-radius-topright:31px;
border-top-right-radius:31px;
-webkit-border-bottom-right-radius:31px;
-moz-border-radius-bottomright:31px;
border-bottom-right-radius:31px;
-webkit-border-bottom-left-radius:31px;
-moz-border-radius-bottomleft:31px;
border-bottom-left-radius:31px;
text-indent:0px;
border:1px solid #84bbf3;
display:inline-block;
color:#ffffff;
font-family:Arial;
font-size:24px;
font-weight:bold;
font-style:normal;
height:88px;
line-height:88px;
width:300px;
text-decoration:none;
```

```
    text-align:center;
    text-shadow:1px 1px 0px #528ecc;
}
table tbody tr td table tbody tr td table tbody tr td div a:hover {
    background:-webkit-gradient( linear, left top, left bottom, color-stop(0.05, #4286f4), color-stop(1, #79bbff) );
    background:-moz-linear-gradient( center top, #4286f4 5%, #79bbff 100% );
    filter:progid:DXImageTransform.Microsoft.gradient(startColorstr='#4286f4', endColorstr='#79bbff');
    background-color:#4286f4;
}
table tbody tr td table tbody tr td table tbody tr td div a:active {
    position:relative;
    top:1px;
}
a {
    -moz-box-shadow:inset 0px 1px 0px 0px #cae3fc;
    -webkit-box-shadow:inset 0px 1px 0px 0px #cae3fc;
    box-shadow:inset 0px 1px 0px 0px #cae3fc;
    background:-webkit-gradient( linear, left top, left bottom, color-stop(0.05, #79bbff), color-stop(1, #4197ee) );
    background:-moz-linear-gradient( center top, #79bbff 5%, #4197ee 100% );
    filter:progid:DXImageTransform.Microsoft.gradient(startColorstr='#79bbff', endColorstr='#4197ee');
    background-color:#79bbff;
    -webkit-border-top-left-radius:10px;
    -moz-border-radius-topleft:10px;
    border-top-left-radius:10px;
```

```
    -webkit-border-top-right-radius:10px;
    -moz-border-radius-topright:10px;
    border-top-right-radius:10px;
    -webkit-border-bottom-right-radius:10px;
    -moz-border-radius-bottomright:10px;
    border-bottom-right-radius:10px;
    -webkit-border-bottom-left-radius:10px;
    -moz-border-radius-bottomleft:10px;
    border-bottom-left-radius:10px;
    text-indent:0;
    border:1px solid #469df5;
    display:inline-block;
    color:#ffffff;
    font-family:Arial;
    font-size:15px;
    font-weight:bold;
    font-style:normal;
    height:40px;
    line-height:40px;
    width:130px;
    text-decoration:none;
    text-align:center;
    text-shadow:1px 1px 0px #287ace;
    }
a:hover {
```

```
    background:-webkit-gradient( linear, left top, left bottom, color-stop(0.05, #4197ee), color-stop(1, #79bbff) );

    background:-moz-linear-gradient( center top, #4197ee 5%, #79bbff 100% );

    filter:progid:DXImageTransform.Microsoft.gradient(startColorstr='#4197ee', endColorstr='#79bbff');

    background-color:#4197ee;

}

a:active {

    position:relative;

    top:1px;

    }


/*classes pour sp_accueil*/


.reduce {

    font-size:16px;

    color:black;

    text-align:center;

    }


.green {

    font-size:22px;

    color:#00cc00;

    font-weight:bold;

    text-align:center;

    }

.grey {
```

```
   font-size:22px;
   color:#b3b3b3;
   font-weight:700;
   text-align:center;

   }
.blue {
   font-size:16px;
   color:#4285f4;
   font-weight:700;
   text-align:center;

   }

.bigblue {
   font-size:22px;
   color:#4285f4;
   font-weight:700;
   text-align:center;

   }
     /*class pour hr*/
.hrblue {
    color:#4285f4;
    background-color:#4285f4;
```

```
    height:10px;
    border:none;
    width: auto;

}
/*for home page*/
.welcome {
   margin:auto;
   border:10px outset EDEDF3;
   background-color:4285f4;
   text-align:center;
   padding:3 white;
   }


.white {
   color:white;
   font-weight:normal;
   font-size:18px;
   text-align:center;

}
.smallwhite {
   color:white;
   font-weight:normal;
```

```
    font-size:18px;


}
.black {
    color:black;
    font-weight:bold;
    font-size:20px;


}



table tbody tr strong a {
    -moz-box-shadow:inset 0px 1px 0px 0px #00cc00;
    -webkit-box-shadow:inset 0px 1px 0px 0px #00cc00;
    box-shadow:inset 0px 1px 0px 0px #00cc00;
    background:-webkit-gradient( linear, left top, left bottom, color-stop(0.05, #63f08d), color-stop(1,
#00cc00) );
    background:-moz-linear-gradient( center top, #63f08d 5%, #00cc00 100% );
    filter:progid:DXImageTransform.Microsoft.gradient(startColorstr='#63f08d', endColorstr='#00cc00');
    background-color:#63f08d;
    -webkit-border-top-left-radius:10px;
    -moz-border-radius-topleft:10px;
    border-top-left-radius:10px;
    -webkit-border-top-right-radius:10px;
    -moz-border-radius-topright:10px;
    border-top-right-radius:10px;
```

```
    -webkit-border-bottom-right-radius:10px;
    -moz-border-radius-bottomright:10px;
    border-bottom-right-radius:10px;
    -webkit-border-bottom-left-radius:10px;
    -moz-border-radius-bottomleft:10px;
    border-bottom-left-radius:10px;
    text-indent:0;
    border:1px solid #00cc00;
    display:inline-block;
    color:#ffffff;
    font-family:Arial;
    font-size:18px;
    font-weight:bold;
    font-style:normal;
    height:29px;
    line-height:29px;
    width:109px;
    text-decoration:none;
    text-align:center;
    text-shadow:1px 1px 0px #00cc00;
}
table tbody tr strong a:hover {
    background:-webkit-gradient( linear, left top, left bottom, color-stop(0.05, #00cc00), color-stop(1,
#63f08d) );
    background:-moz-linear-gradient( center top, #00cc00 5%, #63f08d 100% );
    filter:progid:DXImageTransform.Microsoft.gradient(startColorstr='#00cc00', endColorstr='#63f08d');
```

```
    background-color:#00cc00;
}
table tbody tr strong a:active {
    position:relative;
    top:1px;
}

a.final {
    -moz-box-shadow:inset 0px 1px 0px 0px #d9fbbe;
    -webkit-box-shadow:inset 0px 1px 0px 0px #d9fbbe;
    box-shadow:inset 0px 1px 0px 0px #d9fbbe;
    background:-webkit-gradient( linear, left top, left bottom, color-stop(0.05, #00cc00), color-stop(1,
#c4ffc4) );
    background:-moz-linear-gradient( center top, #00cc00 5%, #c4ffc4 100% );
    filter:progid:DXImageTransform.Microsoft.gradient(startColorstr='#00cc00', endColorstr='#c4ffc4');
    background-color:#00cc00;
    -webkit-border-top-left-radius:22px;
    -moz-border-radius-topleft:22px;
    border-top-left-radius:22px;
    -webkit-border-top-right-radius:22px;
    -moz-border-radius-topright:22px;
    border-top-right-radius:22px;
    -webkit-border-bottom-right-radius:22px;
    -moz-border-radius-bottomright:22px;
    border-bottom-right-radius:22px;
    -webkit-border-bottom-left-radius:22px;
```

```
   -moz-border-radius-bottomleft:22px;
   border-bottom-left-radius:22px;
   text-indent:0px;
   border:1px solid #83c41a;
   display:inline-block;
   color:#ffffff;
   font-family:Arial;
   font-size:24px;
   font-weight:bold;
   font-style:normal;
   height:72px;
   line-height:76px;
   width:250px;
   text-decoration:none;
   text-align:center;
   text-shadow:1px 1px 0px #86ae47;
}
a.final {
   background:-webkit-gradient( linear, left top, left bottom, color-stop(0.05, #c4ffc4), color-stop(1,
#00cc00) );
   background:-moz-linear-gradient( center top, #c4ffc4 5%, #00cc00 100% );
   filter:progid:DXImageTransform.Microsoft.gradient(startColorstr='#c4ffc4', endColorstr='#00cc00');
   background-color:#c4ffc4;
}a.final {
   position:relative;
   top:1px;
```

```
}

a.poursuivre {

    -moz-box-shadow:inset 0px 1px 0px 0px #bbdaf7;

    -webkit-box-shadow:inset 0px 1px 0px 0px #bbdaf7;

    box-shadow:inset 0px 1px 0px 0px #bbdaf7;

    background:-webkit-gradient( linear, left top, left bottom, color-stop(0.05, #79bbff), color-stop(1,
#4286f4) );

    background:-moz-linear-gradient( center top, #79bbff 5%, #4286f4 100% );

    filter:progid:DXImageTransform.Microsoft.gradient(startColorstr='#79bbff', endColorstr='#4286f4');

    background-color:#79bbff;

    -webkit-border-top-left-radius:31px;

    -moz-border-radius-topleft:31px;

    border-top-left-radius:31px;

    -webkit-border-top-right-radius:31px;

    -moz-border-radius-topright:31px;

    border-top-right-radius:31px;

    -webkit-border-bottom-right-radius:31px;

    -moz-border-radius-bottomright:31px;

    border-bottom-right-radius:31px;

    -webkit-border-bottom-left-radius:31px;

    -moz-border-radius-bottomleft:31px;

    border-bottom-left-radius:31px;

    text-indent:0px;

    border:1px solid #84bbf3;

    display:inline-block;

    color:#ffffff;
```

```
    font-family:Arial;
    font-size:24px;
    font-weight:bold;
    font-style:normal;
    height:88px;
    line-height:88px;
    width:300px;
    text-decoration:none;
    text-align:center;
    text-shadow:1px 1px 0px #528ecc;
}
a.poursuivre:hover {
    background:-webkit-gradient( linear, left top, left bottom, color-stop(0.05, #4286f4), color-stop(1,
#79bbff) );
    background:-moz-linear-gradient( center top, #4286f4 5%, #79bbff 100% );
    filter:progid:DXImageTransform.Microsoft.gradient(startColorstr='#4286f4', endColorstr='#79bbff');
    background-color:#4286f4;
}
a.poursuivre:active {
    position:relative;
    top:1px;
}
a.courriel{
    -moz-box-shadow:inset 0px 1px 0px 0px #cae3fc;
    -webkit-box-shadow:inset 0px 1px 0px 0px #cae3fc;
    box-shadow:inset 0px 1px 0px 0px #cae3fc;
```

```
background:-webkit-gradient( linear, left top, left bottom, color-stop(0.05, #79bbff), color-stop(1, #4197ee) );
background:-moz-linear-gradient( center top, #79bbff 5%, #4197ee 100% );
filter:progid:DXImageTransform.Microsoft.gradient(startColorstr='#79bbff', endColorstr='#4197ee');
background-color:#79bbff;
-webkit-border-top-left-radius:10px;
-moz-border-radius-topleft:10px;
border-top-left-radius:10px;
-webkit-border-top-right-radius:10px;
-moz-border-radius-topright:10px;
border-top-right-radius:10px;
-webkit-border-bottom-right-radius:10px;
-moz-border-radius-bottomright:10px;
border-bottom-right-radius:10px;
-webkit-border-bottom-left-radius:10px;
-moz-border-radius-bottomleft:10px;
border-bottom-left-radius:10px;
text-indent:0;
border:1px solid #469df5;
display:inline-block;
color:#ffffff;
font-family:Arial;
font-size:15px;
font-weight:bold;
font-style:normal;
height:40px;
```

```
   line-height:40px;

   width:250px;

   text-decoration:none;

   text-align:center;

   text-shadow:1px 1px 0px #287ace;

   }

a.courriel:hover {

   background:-webkit-gradient( linear, left top, left bottom, color-stop(0.05, #4197ee), color-stop(1, #79bbff) );

   background:-moz-linear-gradient( center top, #4197ee 5%, #79bbff 100% );

   filter:progid:DXImageTransform.Microsoft.gradient(startColorstr='#4197ee', endColorstr='#79bbff');

   background-color:#4197ee;

}

a.courriel:active {

   position:relative;

   top:1px;

   }
```
