# Supplementary material for: Development of a Fully Automated, Web-Based, Tailored Intervention Promoting Regular Physical Activity Among Insufficiently Active Adults With Type 2 Diabetes: Integrating the I-Change Model, Self-Determination Theory, and Motivational Interviewing Components
Source: JMIR Res Protoc. 2015 Feb 17;4(1):e25. doi: 10.2196/resprot.4099 (PMC4376153; doi:10.2196/resprot.4099)
Supplement: Supplementary file 6 [file resprot_v4i1e25_app6.pdf]

CLINK THE LINK BELOW TO ACCESS THE DEF TAILORED INTERVENTION VIDEOS:

https://vimeo.com/album/3140264

USE THE PASSWORD BELOW TO UNLOCK THE ALBUM:

DEF_2014
