# Supplementary material for: Development of a Fully Automated, Web-Based, Tailored Intervention Promoting Regular Physical Activity Among Insufficiently Active Adults With Type 2 Diabetes: Integrating the I-Change Model, Self-Determination Theory, and Motivational Interviewing Components
Source: JMIR Res Protoc. 2015 Feb 17;4(1):e25. doi: 10.2196/resprot.4099 (PMC4376153; doi:10.2196/resprot.4099)
Supplement: Supplementary file 7 [file resprot_v4i1e25_app7.pdf]

CLINK THE LINK BELOW TO ACCESS THE DEF TAILORED INTERVENTION DEMO WEBSITE:

http://www.diabeteenforme-demo.ca/

USE THE EMAIL AND PASSWORD BELOW TO ACCESS THE INTERVENTION:

Email: test_def@uqtr.ca
Password : def101
