# Supplementary material for: Development of a Fully Automated, Web-Based, Tailored Intervention Promoting Regular Physical Activity Among Insufficiently Active Adults With Type 2 Diabetes: Integrating the I-Change Model, Self-Determination Theory, and Motivational Interviewing Components
Source: JMIR Res Protoc. 2015 Feb 17;4(1):e25. doi: 10.2196/resprot.4099 (PMC4376153; doi:10.2196/resprot.4099)

- ***Use "Ctrl" and "+" to zoom in and "Ctrl" and "-" to zoom out***
- ***This document has not been translated in English except for the title of each mock-up template***

/*Home-page - Registration Period

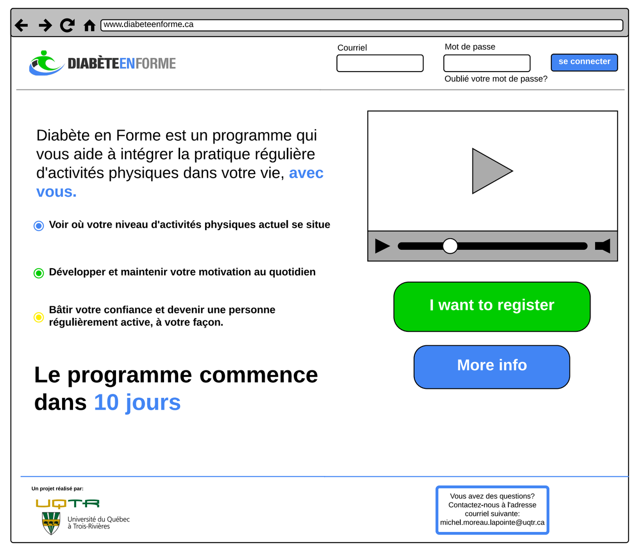

"More info tab"

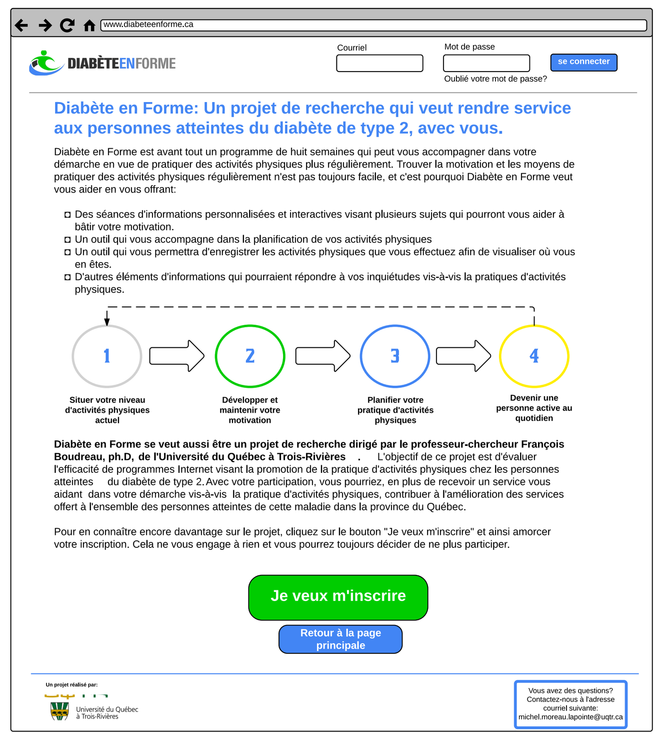

/*Home-page - registration period is over -first week of the program

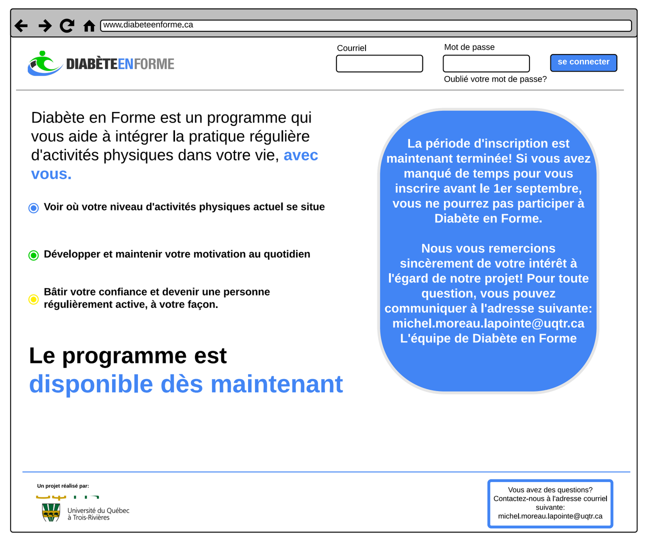

/*Home-page - registration period is over -second week and rest of the program

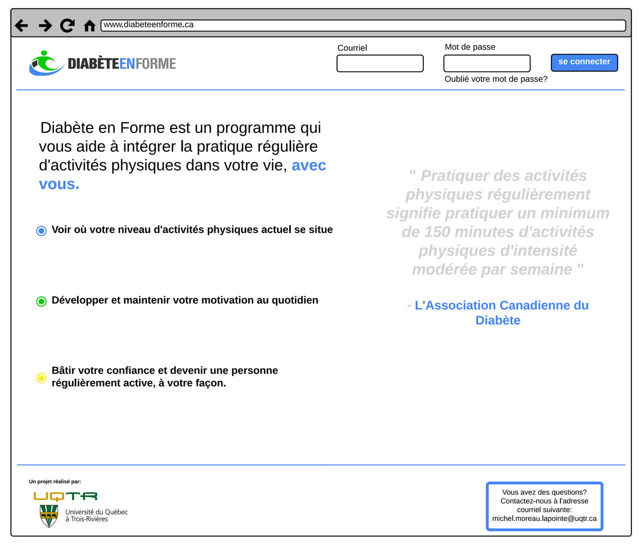

/*Password forgotten

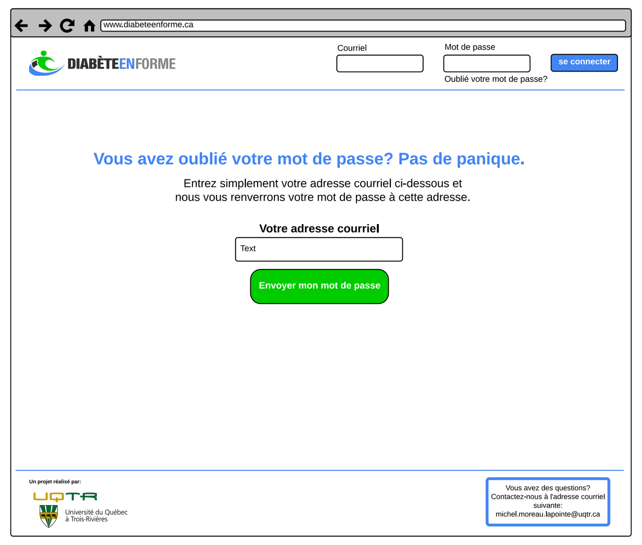

/*Account creation

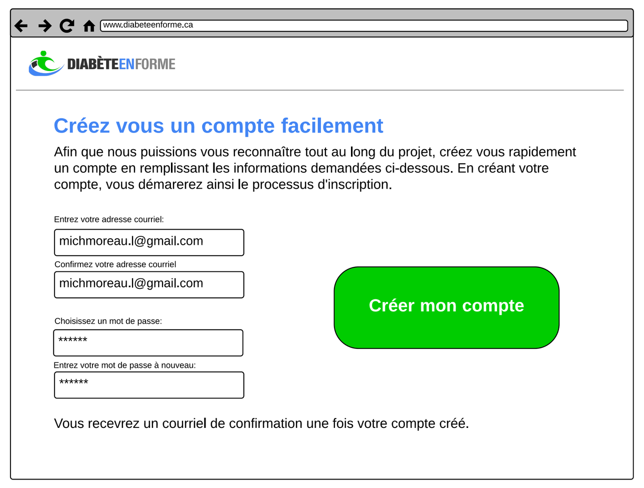

/*Main menu when participants are logged in

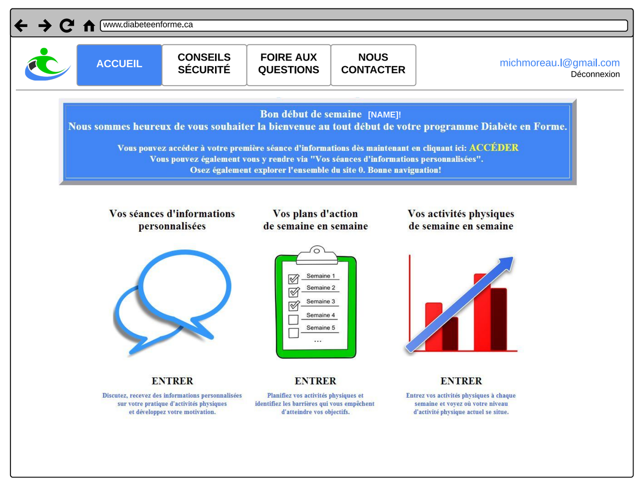

/*Log out page

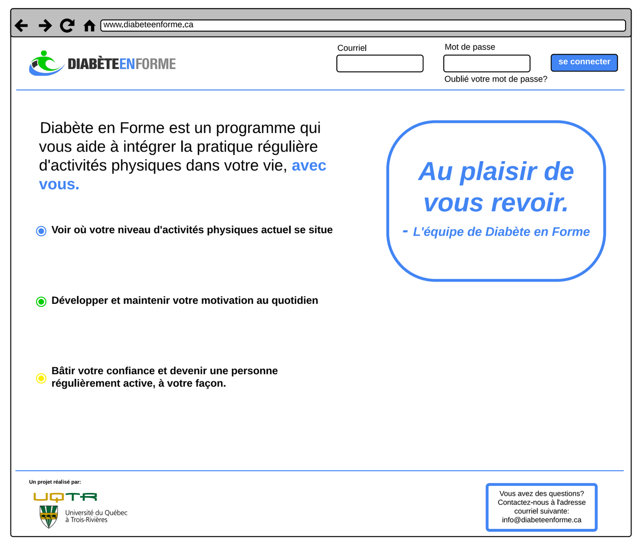

/*Self-monitoring tool

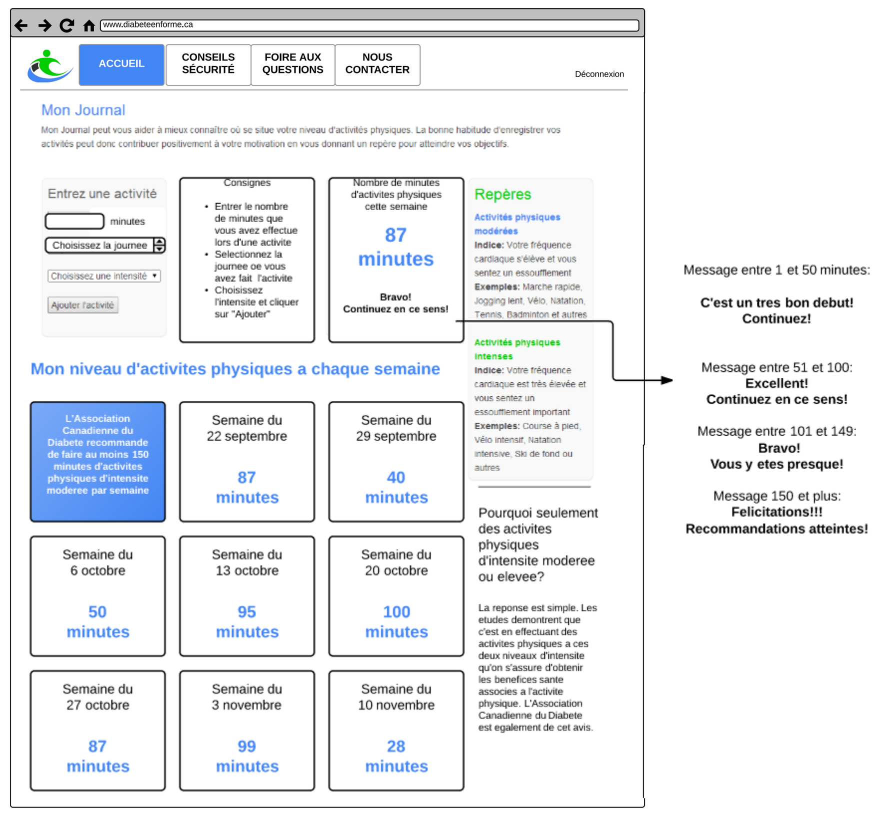

/*Safety tips tab

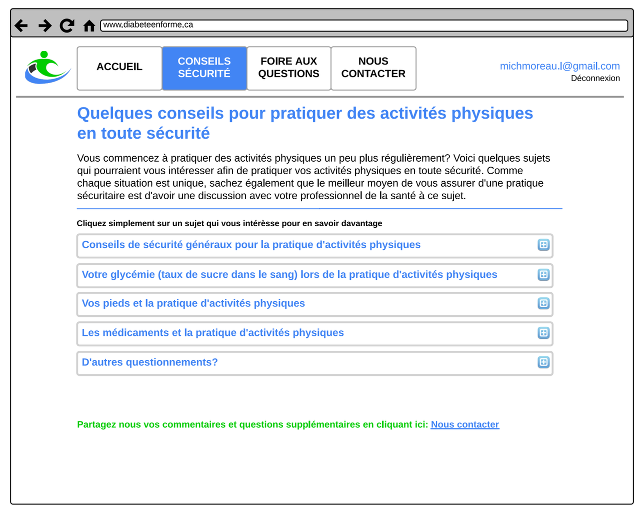

/*FAQ Tab

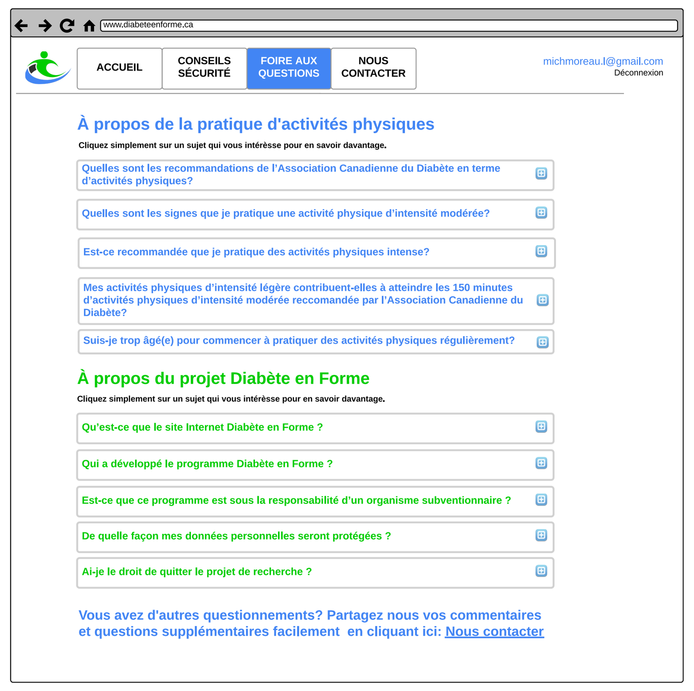

/*Contact tab

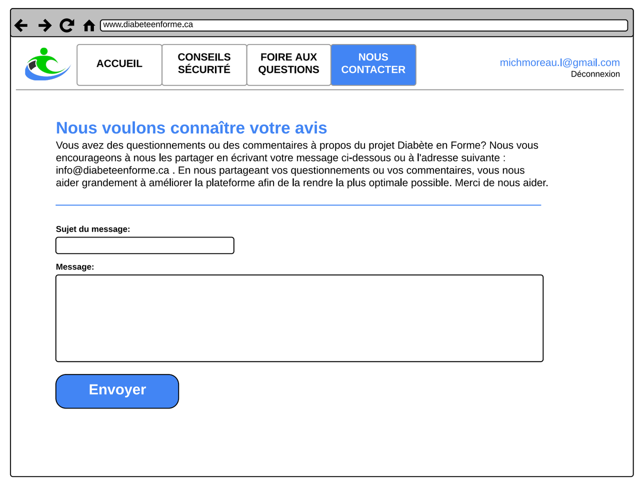

/*Message sent

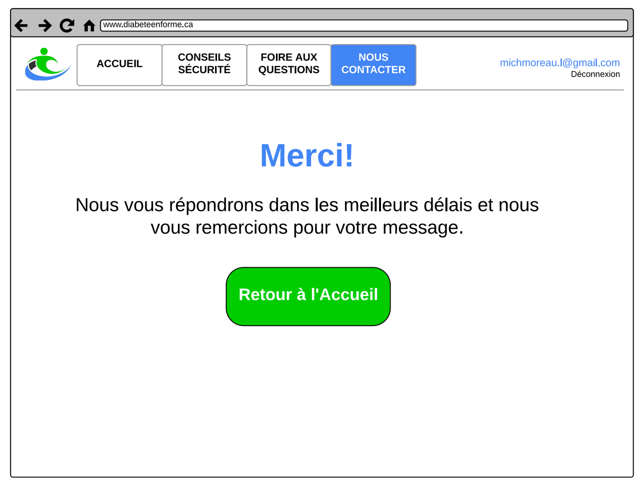

Supplement: Supplementary file 4 [file resprot_v4i1e25_app4.pdf]
